# Seasonal effects on miRNA and transcriptomic profile of oocytes and follicular cells in buffalo (*Bubalus bubalis*)

**DOI:** 10.1038/s41598-020-70546-5

**Published:** 2020-08-11

**Authors:** Emanuele Capra, Barbara Lazzari, Marco Russo, Michal Andrzej Kosior, Giovanni Della Valle, Valentina Longobardi, Alessandra Stella, Anna Lange Consiglio, Bianca Gasparrini

**Affiliations:** 1 Istituto di Biologia e Biotecnologia Agraria, Consiglio Nazionale Delle Ricerche IBBA CNR, Via Einstein 1, 26900 Lodi, Italy; 2 Dipartimento di Medicina Veterinaria e Produzioni Animali (DMVPA), Università Degli Studi di Napoli Federico II, Via F. Delpino 1, 80137 Naples, Italy; 3 Dipartimento di Medicina Veterinaria (DIMEVET), Università Degli Studi di Milano, Via Celoria, 10, 20133 Milan, Italy; 4 Centro Clinico-Veterinario e Zootecnico-Sperimentale di Ateneo, Università Degli Studi di Milano, Via dell’Università, 6, 26900 Lodi, Italy

**Keywords:** Biological techniques, Biotechnology, Cell biology, Developmental biology, Genetics, Molecular biology

## Abstract

Season clearly influences oocyte competence in buffalo (*Bubalus bubalis*); however, changes in the oocyte molecular status in relation to season are poorly understood. This study characterizes the microRNA (miRNA) and transcriptomic profiles of oocytes (OOs) and corresponding follicular cells (FCs) from buffalo ovaries collected in the breeding (BS) and non-breeding (NBS) seasons. In the BS, cleavage and blastocyst rates are significantly higher compared to NBS. Thirteen miRNAs and two mRNAs showed differential expression (DE) in FCs between BS and NBS. DE-miRNAs target gene analysis uncovered pathways associated with transforming growth factor β (TGFβ) and circadian clock photoperiod. Oocytes cluster in function of season for their miRNA content, showing 13 DE-miRNAs between BS and NBS. Between the two seasons, 22 differentially expressed genes were also observed. Gene Ontology (GO) analysis of miRNA target genes and differentially expressed genes (DEGs) in OOs highlights pathways related to triglyceride and sterol biosynthesis and storage. Co-expression analysis of miRNAs and mRNAs revealed a positive correlation between miR-296-3p and genes related to metabolism and hormone regulation. In conclusion, season significantly affects female fertility in buffalo and impacts on oocyte transcriptomic of genes related to folliculogenesis and acquisition of oocyte competence.

## Introduction

Water buffalo (*Bubalus bubalis*) is an important livestock resource for both developing and developed countries. The major factor affecting buffalo farming profitability is reproductive seasonality, resulting in cycles of calving and milk production. Buffalo is a short-day breeder, with increased fertility in response to decreasing day length^1,2^. This photoperiod-dependent seasonality pattern is more pronounced as distance from the equator, together with variations in the light/dark ratio, increases. In Italy, in order to satisfy market demand, out of breeding mating strategy (OBMS), consisting in interrupting sexual promiscuity or the use of artificial insemination (AI) during the breeding season (BS), is commonly utilized^2^. The OBMS improves the distribution of calving throughout the year, but it reduces fertility^3^. Longer post-partum anoestrus periods as well as higher incidence of embryonic mortality are observed in months with increasing daylight length and particularly in mid-winter, which coincides with the transition to seasonal anoestrus at Italian latitudes^1,4^. The embryonic mortality is due to inadequate luteal growth and function, resulting in reduced progesterone secretion^5^. This has a negative impact on embryo growth, associated with alterations in transcriptomic and proteomic profiles of the embryos and chorioamnios/caruncles^6,7^, which ultimately impair embryo attachment to the uterine endometrium.

An additional factor determining reproductive failure in the non-breeding season (NBS) is the oocyte developmental competence. Indian authors reported decreased efficiency of ovum pick-up (OPU) during the NBS, mainly due to the reduced follicular population^8^. A seasonal effect on the number of follicles and oocytes, as well as on oocyte competence, has also been reported in Egyptian buffaloes^9^. In Italian Mediterranean buffaloes, season clearly influences oocyte competence, as indicated by improved blastocyst yields recorded during months with decreasing daylight^10,11^. In Murrah buffalo heifers, the decreased oocyte quality recorded during long day months was associated to reduced concentration of oestradiol both in plasma and follicular fluid, as well as of intrafollicular IGF-1^12^. Despite the evidence of a seasonal influence in buffalo, the molecular mechanisms affecting oocyte competence in the NBS are poorly understood.

A fine-tuned spatio-temporal expression of multiple genes is known to be essential for follicular development and oocyte maturation, and requires a strict interaction between mRNAs and regulatory miRNAs^13^. In addition, in many tissues a time-controlled gene expression is mediated by miRNAs, which regulate core clock genes coordinating daily rhythms in physiology and behaviour^14^. A relationship between the variation of mRNAs abundance for specific genes related to folliculogenesis in ovaries and changes in photoperiods was previously reported in other non-ruminant species such as the Siberian hamster^15^. Changes in transcriptome and miRNA expression in relation to season were further investigated in sheep, another short-day breeder, where oocyte competence was observed to decline during the NBS, as indicated by impaired in vitro embryo development^16^. Furthermore, transcriptome variations potentially associated with off-season reproduction were reported in sheep ovaries^17^. Again, differences in miRNA profiling in ovaries of Tan sheep and Small Tail Han (STH) sheep were related to ovine anoestrus and BS^18^. Seasonal differences in the expression of miRNAs involved in hormone regulation, follicular growth and angiogenesis were also observed in Kazakh sheep ovaries during oestrus^19^. Recently, an integrated analysis of mRNA and miRNA expression in European mouflon (*Ovis musimon*) and sheep (*Ovis aries*) depicted a miRNA-mRNA regulatory network associated with reproductive traits in *Ovis* species^20^.

MicroRNAs play a significant role during follicle development in bovine^21,22^. In buffalo, heat stress was observed to alter the blood miRNA and mRNA content^23^. The role of miRNAs is also demonstrated in the regulation of lactating physiology in the buffalo mammary gland^24^, and miRNA expression changes were observed in buffalo corpus luteum during pregnancy^25^.

Therefore, in this study we investigated, for the first time, if reproductive failure in the NBS is associated to changes in gene expression affecting oocyte developmental competence in buffalo. To evaluate seasonal effects on oocyte competence, the miRNA and transcriptomic profiles of oocytes and corresponding follicular cells were characterized from abattoir-derived ovaries collected in the BS and NBS.

## Results

### Cleavage and blastocyst rates

With regard to oocyte competence, cleavage rate was higher in the BS compared to the NBS (69.4%; vs. 60.7; *P* < 0.05). In addition, an improvement in blastocyst yields was recorded in the BS, both in relation to total COCs (26.5 vs. 16.3%; *P* < 0.01) and cleaved oocytes (38.7 vs. 27.4%; *P* < 0.05).

### miRNAs

Twenty samples, i.e. 10 pools of OOs (sample OO1-OO5 from NBS and OO6-OO10 from BS) and 10 pools of FCs (sample FC1-FC5 from NBS and FC6-FC10 from BS), were characterized for their miRNA content.

About 10 million reads were sequenced for both OOs and FCs (see Supplementary File S1 for statistics). About 1% and 20% of them were assigned to miRNAs in OOs and FCs, respectively.

In total, 769 miRNAs were identified in at least three samples in all conditions (468 *Bos taurus* bta-miRNAs, 279 novels, and 22 novels homologous to related species). Among them, 467 were detected in at least three OO samples and 635 in at least three FC samples. Principal Component Analysis (PCA) clearly separates OOs and FCs according to their miRNA content, with 44% of the variance explained by component 1 (Supplementary file S2).

**Figure 1:**
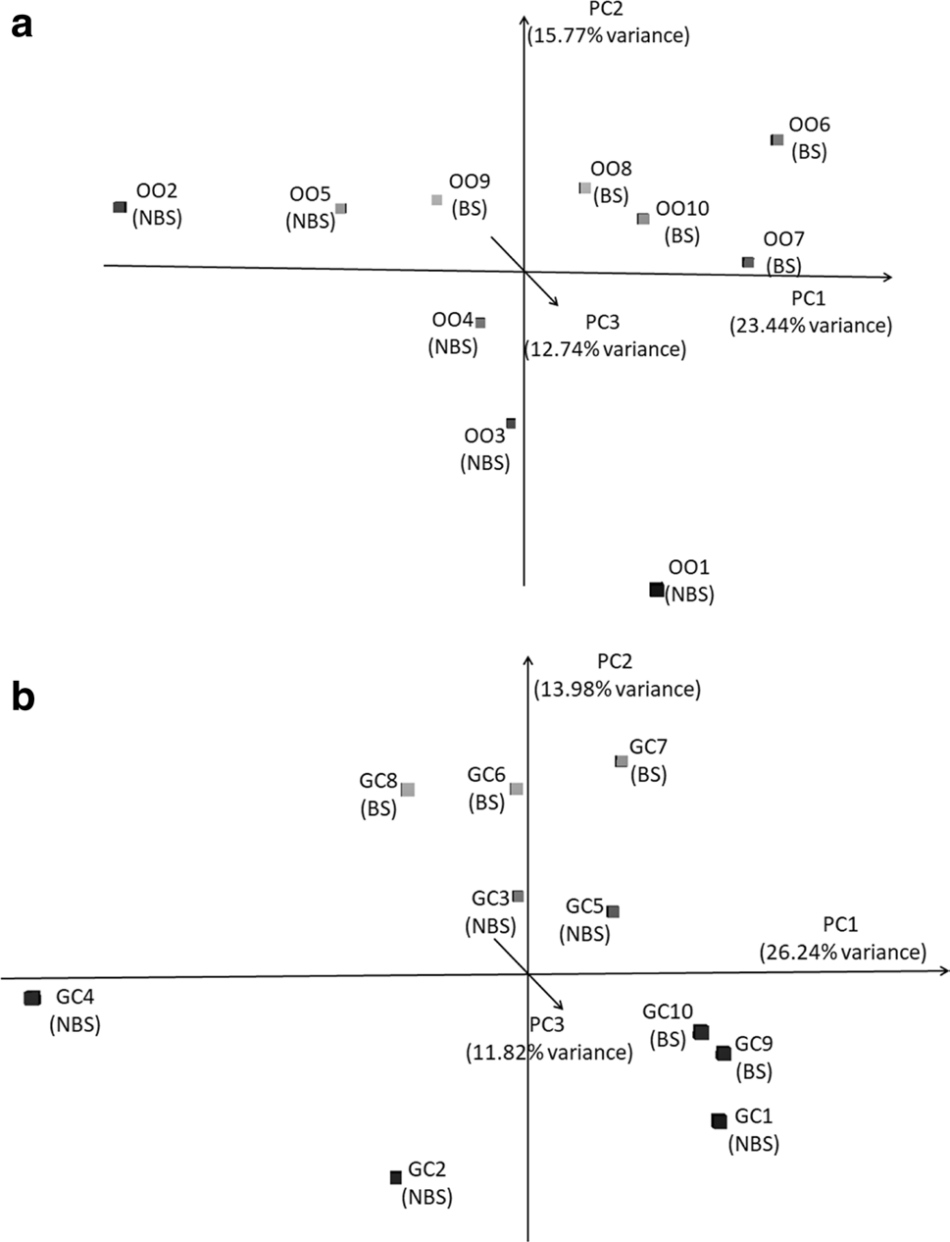
Principal component analysis considering (**a**) the 467 miRNAs expressed at least in triplicate in the oocytes (OOs), (**b**) the 635 miRNAs expressed at least in triplicate in the Follicular cells (FCs). Samples 1–5 from non-breeding season (NBS), samples 6–10 from breeding season (BS).

Few of the most expressed miRNAs showed a similar relative abundance in OOs and FCs (bta-miR-10b, bta-miR-148a and bta-miR-26a); on the contrary, expression rate of most miRNAs differed in the two cellular types (bta-miR-21-5p was highly expressed in FCs, whereas bta-miR-423-3p in OOs), (Supplementary file 3). In fact, there was a statistically significant difference in the expression of a high proportion of the miRNAs (n = 413) (False Discovery Rate FDR < 0.05) between OOs and FCs (Supplementary file 4). When the two seasons were considered, the PCA produced a good distinction between oocytes collected in BS and NBS, whereas FCs from BS and NBS could not be clearly distinguished (Fig. 1).

**Figure 2:**
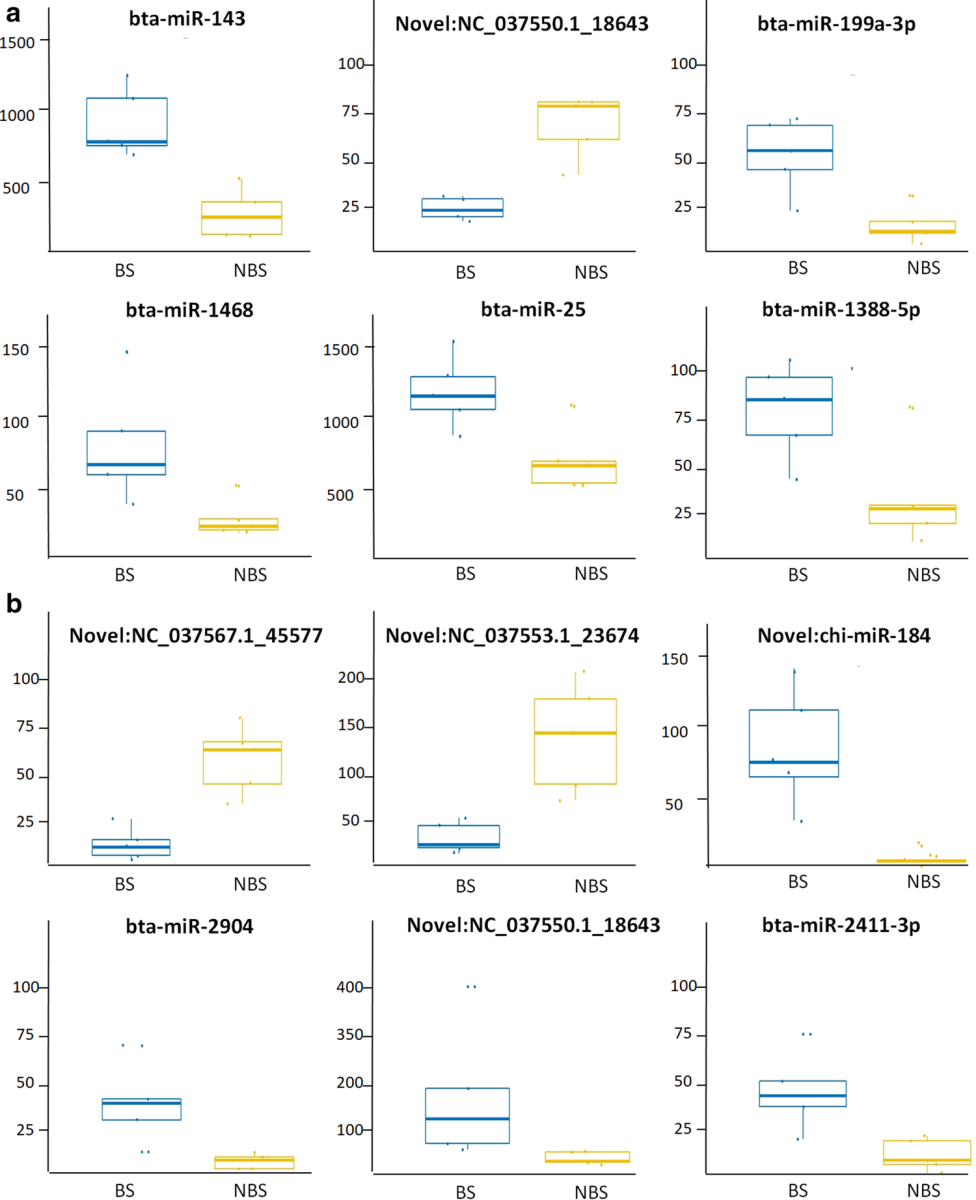
Box Plot of the most significant (top six sorted by FDR value) differentially expressed miRNAs (DE-miRNAs) in (**a**) oocyte and (**b**) follicular cells from animals between breeding season (BS) and non breeding season (NBS).

The number of differentially expressed miRNAs (DE-miRNAs, FDR < 0.05) between the two seasons was 13 for both OOs and FCs (Supplementary file 5 and Table 1). A view of the normalized expression of the most representative DE-miRNAs is shown in Fig. 2. Target prediction using human miRNAs homologous to buffalo DE-miRNAs led to the identification of 6,712 and 4,847 genes potentially regulated in OOs and FCs, respectively (*P* < 0.05). GO analysis using a subset of more significant target genes (n = 136 with *P* < 0.0005 for OOs, n = 139 with *P* < 0.001 for FCs) identified pathways related to triglyceride and cholesterol metabolism and transport, and mesoderm and epithelial cell morphogenesis differentiation for OOs, and related to photoperiodism, circadian clock regulation, and transforming growth factor beta signalling for FCs (Table2).

**Table 1:** Differentially expressed miRNAs DE-miRNAs (false discovery rate (FDR) < 0.05) between the two seasons (NBS = non breeding season, BS = breeding season) for oocytes (OOs) and follicular cells (FCs).

OOs (NBS vs BS)			FCs (NBS vs BS)		
miRNAs	logFC	FDR	miRNAs	logFC	FDR
bta-miR-143	− 2.07	5.42E−05	Novel:NC_037567.1_45577	2.65	1.69E−05
Novel:NC_037550.1_18643	1.58	7.24E−04	Novel:NC_037553.1_23674	2.09	3.78E−05
bta-miR-199a-3p	− 2.10	7.24E−04	Novel:chi-miR-184	− 4.24	1.54E−04
bta-miR-1468	− 1.83	3.69E−03	bta-miR-2904	− 2.90	2.50E−04
bta-miR-25	− 0.91	1.44E−02	Novel:NC_037550.1_18643	− 2.37	4.25E−04
bta-miR-1388-5p	− 4.55	3.55E−02	bta-miR-2411-3p	− 2.01	1.54E−03
bta-miR-296-3p	− 1.41	3.91E−02	bta-miR-2440	− 1.86	2.98E−03
Novel:NC_037557.1_30140	− 1.36	3.91E−02	bta-miR-2332	− 1.47	4.48E−03
Novel:NC_037569.1_47305	− 1.36	3.91E−02	bta-miR-141	− 3.73	4.48E−03
Novel:NC_037564.1_42998	− 1.36	3.91E−02	bta-miR-2478	1.68	4.97E−03
bta-miR-331-5p	− 4.19	4.12E−02	bta-miR-34b	− 4.11	8.16E−03
bta-miR-199a-5p	− 2.09	4.47E−02	bta-miR-34c	− 4.02	8.16E−03
bta-miR-222	− 1.35	4.95E−02	bta-miR-486	0.96	3.65E−02

**Table 2:** GO terms identified for the target genes of differentially expressed miRNAs between the two seasons for oocytes (OOs) and Follicular Cells (FCs).

	GOID	Associated genes found	GO term	*P* value*
**OOs**	0010866	[DGAT2, FITM2, NR1H3]	Regulation of triglyceride biosynthetic process	0.004
	0060742	[NOTCH1, SFTPA1, SFTPA2]	Epithelial cell differentiation involved in prostate gland development	0.005
	0015918	[ABCG4, APOB, NR1H3, OSBPL6, PNLIP]	Sterol transport	0.009
	0030850	[NOTCH1, SFTPA1, SFTPA2, WNT5A]	Prostate gland development	0.012
	0048332	[AXIN1, GNPDA1, WNT5A]	Mesoderm morphogenesis	0.014
	0019915	[APOB, DGAT2, FITM2, NR1H3]	Lipid storage	0.026
	0001707	[AXIN1, GNPDA1, WNT5A]	Mesoderm formation	0.027
	0006536	[ATAT1, GGT1, GLUD2]	Glutamate metabolic process	0.028
	0006641	[APOB, DGAT2, FITM2, INSIG1, NR1H3]	Triglyceride metabolic process	0.028
	0042116	[IL13, NR1H3, WNT5A]	Macrophage activation	0.028
	0090207	[DGAT2, FITM2, NR1H3]	Regulation of triglyceride metabolic process	0.029
	0008206	[ACAA1, CYP27A1, OSBPL6]	Bile acid metabolic process	0.030
	0002637	[EXOSC3, GNPDA1, IL13]	Regulation of immunoglobulin production	0.033
	0070527	[FERMT3, MYH9, PRKCQ]	Platelet aggregation	0.033
	0002067	[IL13, NOTCH1, WNT5A]	Glandular epithelial cell differentiation	0.033
	0019217	[DGAT2, INSIG1, NR1H3, PDHB]	Regulation of fatty acid metabolic process	0.034
	0097006	[APOB, DGAT2, PLAGL2]	Regulation of plasma lipoprotein particle levels	0.035
	0055090	[DGAT2, FITM2, NR1H3]	Acylglycerol homeostasis	0.035
	0070328	[DGAT2, FITM2, NR1H3]	Triglyceride homeostasis	0.035
	0030301	[ABCG4, APOB, NR1H3, PNLIP]	Cholesterol transport	0.037
	0050830	[DROSHA, HIST1H2BK, PGLYRP1, PGLYRP3]	Defence response to Gram-positive bacterium	0.037
	0033344	[ABCG4, APOB, NR1H3]	Cholesterol efflux	0.038
	0002702	[EXOSC3, GNPDA1, IL13, WNT5A]	Positive regulation of production of molecular mediator of immune response	0.038
	0030514	[CHRDL1, NOTCH1, WNT5A]	Negative regulation of BMP signaling pathway	0.038
	0010883	[APOB, FITM2, NR1H3]	Regulation of lipid storage	0.039
	0019432	[DGAT2, FITM2, NR1H3]	Triglyceride biosynthetic process	0.039
	0043030	[IL13, NR1H3, WNT5A]	Regulation of macrophage activation	0.039
	0045599	[AXIN1, INSIG1, WNT5A]	Negative regulation of fat cell differentiation	0.040
	0002639	[EXOSC3, GNPDA1, IL13]	Positive regulation of immunoglobulin production	0.040
	0046460	[DGAT2, FITM2, NR1H3]	Neutral lipid biosynthetic process	0.040
	0046463	[DGAT2, FITM2, NR1H3]	Acylglycerol biosynthetic process	0.040
**FCs**	0043153	[BHLHE40, PPP1CB, PPP1CC]	Entrainment of circadian clock by photoperiod	0.004
	1903844	[ING3, ONECUT2, SKI, STRAP, XBP1]	Regulation of cellular response to transforming growth factor beta stimulus	0.008
	0017015	[ING3, ONECUT2, SKI, STRAP, XBP1]	Regulation of transforming growth factor beta receptor signaling pathway	0.008
	0009648	[BHLHE40, PPP1CB, PPP1CC]	Photoperiodism	0.008
	0009649	[BHLHE40, PPP1CB, PPP1CC]	Entrainment of circadian clock	0.008
	1903845	[ONECUT2, SKI, STRAP, XBP1]	Negative regulation of cellular response to transforming growth factor beta stimulus	0.012
	0030512	[ONECUT2, SKI, STRAP, XBP1]	Negative regulation of transforming growth factor beta receptor signaling pathway	0.012
	0010923	[FKBP1B, PPP1R1B, TMEM225]	Negative regulation of phosphatase activity	0.014
	0032755	[IL1RL2, TLR6, XBP1]	Positive regulation of interleukin-6 production	0.025
	0045582	[IL1RL2, ITPKB, XBP1]	Positive regulation of T cell differentiation	0.025
	0035304	[FKBP1B, PPP1R1B, SMPD1]	Regulation of protein dephosphorylation	0.035
	0071230	[CASTOR1, PDGFC, XBP1]	Cellular response to amino acid stimulus	0.035
	1903036	[FKBP1B, SCARF1, XBP1]	Positive regulation of response to wounding	0.040
	0010257	[NDUFAF6, NDUFC1, NDUFS7]	NADH dehydrogenase complex assembly	0.042
	0032981	[NDUFAF6, NDUFC1, NDUFS7]	Mitochondrial respiratory chain complex I assembly	0.042
	0097031	[NDUFAF6, NDUFC1, NDUFS7]	Mitochondrial respiratory chain complex I biogenesis	0.042
	0032922	[BHLHE40, PPP1CB, PPP1CC]	Circadian regulation of gene expression	0.047

Indicated are gene ontology IDs (GO-ID), gene ontology terms (GO-term), associated genes found and corrected *P* values as determined by ClueGO (https://apps.cytoscape.org/apps/cluego). *Term *P* value corrected with Bonferroni step down.

### RNASeq

**Figure 3:**
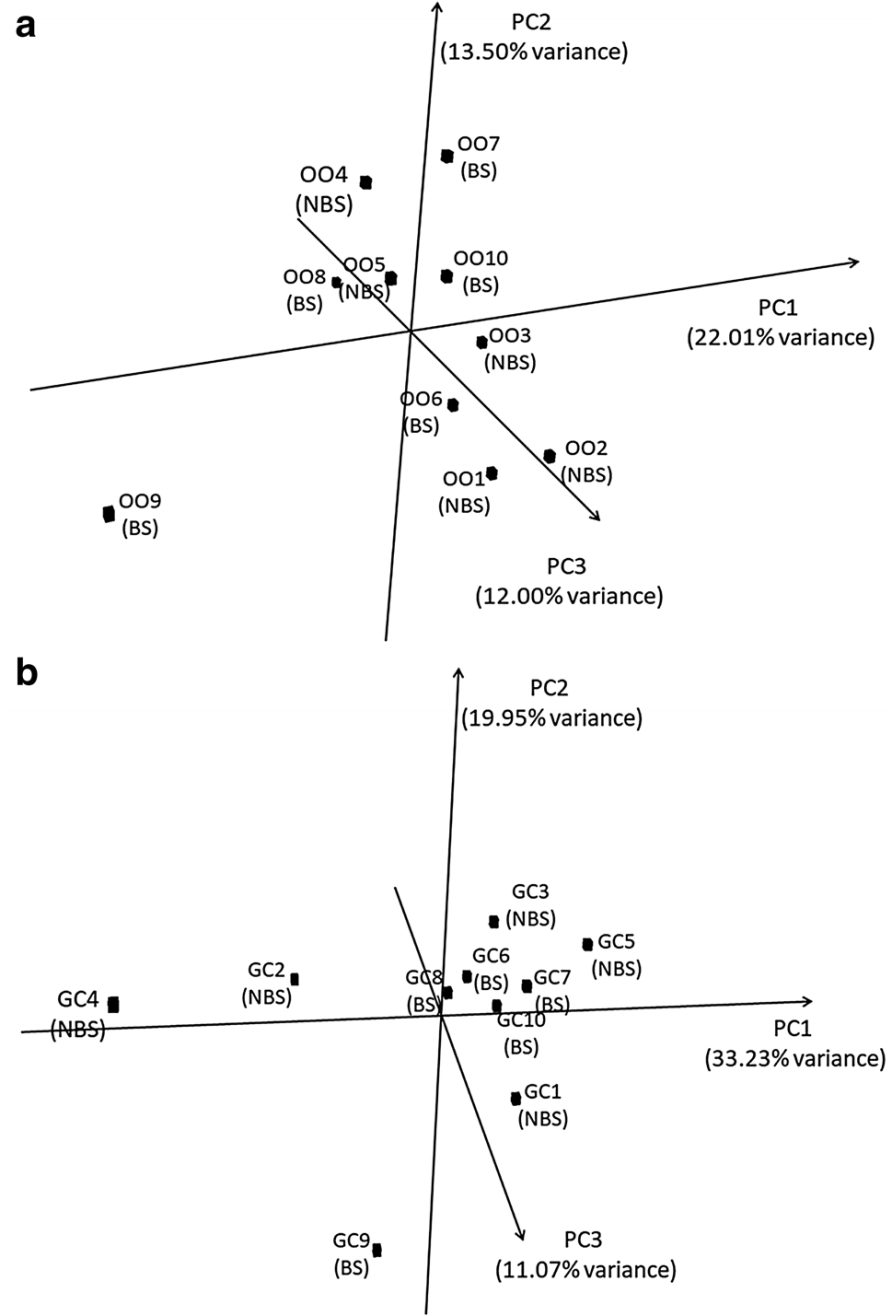
Principal component analysis considering (**a**) the 19,240 mRNAs expressed at least in triplicate in the oocytes (OOs), (**b**) the 21,277 mRNAs expressed at least in triplicate in the Follicular cells (FCs). Samples 1–5 from non-breeding season (NBS), samples 6–10 from breeding season (BS).

RNA-seq analysis was performed on the same samples used for miRNA profiling to evaluate the gene expression variation between the two cellular types and seasons. Approximately 23.5 ± 4.4 and 54.5 ± 10.5 millions of reads were obtained for OOs and FCs samples with a mapping rate of 93.5% and 92.4%, respectively (Supplementary file 6). A total of 22,013 unique genes present in at least three samples from both cellular types were identified (19,240 counted in at least three OOs and 21,277 counted in at least three FCs samples). PCA considering the relative expression of these genes showed a clear separation between the two cellular types (Supplementary file 7). There seems to be no seasonal effect in the overall transcript abundance for both OOs and FCs (Fig. 3).

The relative expression of mRNAs in the two cellular types was very different, with 14,680 (66.7%) DEGs between OOs and FCs. When DEGs were calculated between the two seasons, 22 mRNAs were found to differ between NBS and BS for OOs, whereas only two DEGs were present in FCs (Supplementary file 8 and Table 3).

**Table 3:** Differentially expressed gene DEGs (false discovery rate (FDR) < 0.05) calculated between the two seasons (non breeding season NBS, breeding season) for oocytes (OOs) and follicular cells (FCs).

OOs (NBS vs BS)				FCs (NBS vs BS)			
GENE ID	Human and cattle ortholog	logFC	FDR	GENE ID	Human and cattle ortholog	logFC	FDR
APOE	APOE	− 4.7	8.6E−07	LOC102409538	RNF213	− 4.7	5.6E−04
LOC102397479	LOC102397479	− 5.2	1.3E−05	COL26A1	COL26A1	2.1	2.3E−02
PLXNA4	PLXNA4	− 2.3	2.6E−05				
IGF2	IGF2	− 4.7	5.2E−04				
FOLR2	FOLR2	− 7.5	8.0E−04				
CD14	CD14	− 8.7	8.7E−04				
SPP1	SPP1	− 3.2	2.5E−03				
LOC102409999	CD163	− 4.7	2.8E−03				
CTSS	CTSS	− 5.3	2.8E−03				
LOC102413141	GTF2IRD2	− 0.7	3.9E−03				
LOC102392787	IL1B	− 5.0	6.1E−03				
LOC112581169	LOC112581169	− 0.9	7.6E−03				
CCL1	CCL1	− 1.2	9.7E−03				
CTSK	CTSK	− 3.8	9.7E−03				
LOC102415727	regakine 1	− 5.3	1.1E−02				
MSR1	MSR1	− 3.7	1.2E−02				
RUNX2	RUNX2	− 1.3	1.9E−02				
LOC102404545	LOC102404545	2.3	2.1E−02				
LOC102400151	CYP11A1	− 2.6	2.3E−02				
LOC102409533	HSPA1A	− 0.8	3.6E−02				
NMB	NMB	− 1.9	4.7E−02				
LOC112582161	LOC112582161	0.9	4.8E−02				

Although a limited number of DEGs was found to differ in OOs between the two seasons, GO analysis revealed that some of them were related to lipid storage and localization and regulation of interleukin-8 (IL8) production (Table 4).

**Table 4:** GO terms identified for the differentially expressed gene (DEGs) between the two seasons for oocytes (OOs).

GOID	Associated genes found	GO term	*P* value*
GO:19915	[APOE, IL1B, MSR1]	Lipid storage	7.17E−05
GO:32370	[APOE, IL1B, SPP1]	Positive regulation of lipid transport	8.94E−05
GO:32677	[CD14, HSPA1A, IL1B]	Regulation of interleukin-8 production	4.09E−05
GO:32757	[CD14, HSPA1A, IL1B]	Positive regulation of interleukin-8 production	6.29E−05
GO:1905954	[APOE, IL1B, MSR1, SPP1]	Positive regulation of lipid localization	5.76E−06

Indicated are gene ontology IDs (GO-ID), gene ontology terms (GO-term), associated genes found and corrected *P* values as determined by ClueGO (https://apps.cytoscape.org/apps/cluego).

* Term *P* value corrected with Bonferroni step down.

### miRNAs and mRNA interaction

**Figure 4:**
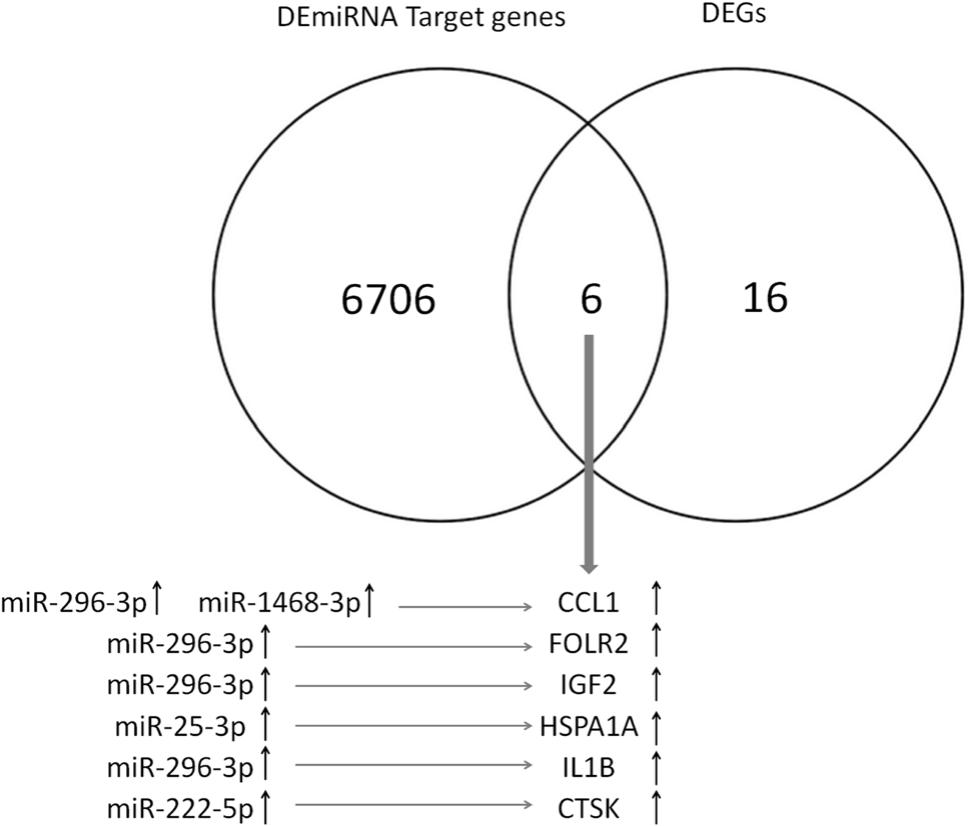
Venn diagram (https://bioinfogp.cnb.csic.es/tools/venny/ version 2.1) representing the intersection (n = 6) between differentially expressed genes (DEGs, n = 22) and target genes of the differentially expressed miRNAs (DE-miRNAs, n = 6,712) in oocytes collected in the NBS and BS. Shared genes with their relative target miRNAs are reported. ^↑^indicates overexpression in breeding season (both for genes and miRNAs).

In order to evaluate whether miRNAs could potentially regulate the expression of specific genes, the list of genes differentially expressed in oocytes between the two seasons was intersected with the list of DE-miRNA target genes observed in the same experimental condition. Six genes (*CCL1*, *FOLR2*, *IGF2*, *HSPA1A*, *IL1B*, *CTSK*) were found to be potentially regulated by specific DE-miRNAs. Interestingly, among the 8 DE-miRNAs (miR-143, miR-1468, miR-199a-3p, miR-199a-5p, miR-222, miR-25, miR-296-3p, miR-331-5p) targeting 6,712 genes, miR-296-3p targets 4 out of the 6 shared DEGs. In addition, all the miRNAs show a positive correlation with gene expression (Fig. 4).

## Discussion

The present study aims to investigate the causes of the decreased oocyte competence during the NBS in buffalo. Many biological processes are required for developmental competence, with the exchange of information between oocyte and follicular environment promoting oocyte maturation^26^. Therefore, the focus of this study was to evaluate differences in the miRNA and transcriptomic profiles of OOs and corresponding FCs from buffalo ovaries collected in the BS and NBS. To our knowledge, this is the first study reporting together the miRNA and mRNA profiling from pools of low numbers of oocytes and corresponding FCs collected from abattoir-derived ovaries in livestock, as well as the first time that the seasonal effects on miRNA and mRNA profiling of oocytes and FCs are investigated in buffalo. Unfortunately, the amount of RNA obtained from such a limited number of oocytes was not sufficient to perform further experiments to validate our results.

In accordance with previous findings^10,11^, in the present study we observed a reduced oocyte developmental competence in the NBS, as indicated by decreased cleavage and blastocyst rates after in vitro fertilization (IVF), and this was associated to changes in miRNA and transcriptomic profiles both in OOs and FCs. Being miRNAs only one of the small RNA components, as expected those identified in buffalo represent only a fraction of the total small non-coding RNA present in both OOs and FCs^27,28^. The overall miRNA expression pattern was different between OOs obtained in the two seasons. However, FCs from the two seasons cluster together. Considering that different phenotypes of FCs can be observed within the follicle, it is likely that miRNA expression in FCs is mainly driven by cell position and function, thus masking seasonal effects^29^. Recently, Zhang et al.^30^ reviewed miRNA profile studies related to ovarian development and function in mammals. Although many studies reported miRNA ovary profiling, only a few investigated miRNA variations in granulosa cells and only one in OOs^30^.

Interestingly, many of the differentially expressed miRNAs in the two seasons for both OOs and FCs are involved in follicular maturation and development regulation. In our study seasonal changes modified the expression of miR-143, miR-25, miR-222 and miR-199a in buffalo OOs. In the mouse ovary, miR-143 is highly expressed and related to oestradiol production and steroidogenesis gene expression^31^. MiR-143 and miR-25 were also shown to promote progesterone release in human ovarian granulosa cells^32^. Furthermore, cyclic variations in the expression of miR-222 and miR-199a were reported in cattle during follicle maturation, with expression increasing until the mid-luteal phase, and decreasing in the late follicular phase in the bovine dominant follicle^13^. Some of the differentially expressed miRNAs identified between the NBS and BS in buffalo FCs (miR-184, miR-2411 and miR-34c) were also reported to exhibit expression modulation during the cycle in cattle. In particular, temporal miRNA expression dynamics were observed for miR-184 in FCs between days 3 and 7 of the bovine oestrous cycle and for miR-2411 and miR-34c between subordinate and dominant follicles during the early luteal phase^33^. MiR-34c was shown to exert anti-proliferative and pro-apoptotic effects in porcine granulosa cells by targeting Forkhead box O3a (FoxO3a)^34^.

In addition, the expression of some of the DE-miRNAs detected in our study differs in several ovarian disorders. It was reported that mir-141 and miR-199a are respectively up and down regulated in human ovarian cancer^35^, miR-184 is a potential predictor of recurrence in human ovarian granulosa cell tumours^36^, and miR-486-5p is downregulated in cumulus cells collected from women affected by polycystic ovary syndrome^37^.

Interestingly, GO analysis of the predicted target genes for DE-microRNAs uncovered pathways associated with OOs and FCs physiology. Oocytes collected from the BS and NBS showed DE-miRNAs able to regulate genes for triglyceride and sterol biosynthesis essential for lipid metabolism, which provides a potent source of energy during oocyte maturation^38^. In FCs, the DE-miRNA target genes were related to pathways involved in transformation of growth factor β (TGFβ) and circadian clock photoperiod. TGFβ promotes granulosa cell proliferation regulating the expression of luteinizing hormone receptor (LH-R)^39–41^. Altered photoperiod can affect mRNA expression in ovaries^15^, in fact, transcriptome changes occurred between BS and NBS samples.

Considering DEGs between seasons, it is interesting to note that although only two DEGs were found in FCs, many of the DEGs in the OOs are known to be related to oocyte competence. In the NBS, decreased oocyte competence in buffalo was associated to change in the expression of secreted phosphoprotein 1 (*SPP1*), RUNX family transcription factor 2 (*RUNX2*) and Cathepsin K (*CTSK*) in OOs. Both *SPP1* and *RUNX2* expression was observed to change in oocyte and-granulosa cell complexes at various stages of follicle development in pigs^42^. In addition, variations in the expression of *SPP1* were recorded in cumulus cells derived from cumulus-oocyte-complexes (COCs) collected from cows undergoing FSH priming, as a model of high oocyte competence^43^, and *RUNX2* expression was associated with controlled ovarian stimulation outcome in assisted reproductive technology treatment in women^44^. Furthermore, *CTSK* in cumulus cells was suggested as a predictive marker for oocyte competence in bovine COC^45^.

In buffalo OOs during the NBS, a decreased expression of heat shock protein family A (Hsp70) member 1A (*HSPA1A*), known to be related to oocyte survival and apoptosis, was also observed. The *HSPA1A* plays a critical role through its protective action against apoptosis and its expression is reduced in poor, as compared to competent, ovine COCs^46^.

Another transcript down-regulated in the NBS OOs is interleukin-1 beta (*IL-1β*). Although *IL-1β* deficiency in mice prolongs ovarian lifespan^47^), IL-1β stimulates the growth and sustains maturation in mare^48^ and bovine oocytes^49^. In addition, *IL-1β* was postulated to be involved in different ovulation-associated events such as prostaglandin production and steroidogenesis^50^. Modulation of expression levels was observed in this study also for other genes related to gonadotropic hormone synthesis and metabolism, showing a reduced expression in buffalo OOs during NBS. Apolipoprotein E (*APOE*) is expressed in cultured ovarian granulosa cells, and is present in human follicular fluid where its relative levels are correlated with serum estrogen concentration^51^. In rats, APOE exerts a role in directing cholesterol during steroidogenesis and regulating follicular estrogenic production^52^. Insulin like growth factor 2 (*IGF2*) was observed to be expressed in bovine oocytes^53^. The expression of *IGF2* is modulated by growth hormone (GH) in in vitro matured *Rhesus macaque* oocytes^54^. Furthermore, it is known that IGF2, in combination with follicle stimulating hormone (FSH), acts directly on oocyte competence in caprine follicles^55^. Finally, the folate receptor beta (*FOLR2*), also down-regulated in OOs during the NBS, is a key gene linked to methionine/folate cycles in bovine oocyte^56^, also involved in folate transport in mice oocytes during follicular development^57^.

In our study, a positive correlation between DEGs and DE-miRNA target genes was observed. MiRNAs usually mediate repression of their target mRNAs by inhibiting their translation, therefore reducing the abundance of their products^58^. However, several studies reported a positive miRNA-mRNA regulation with a feed-forward mechanism probably mediated by transcription factors^59^. Notably, among all DE-miRNAs, miR-296-3p is the most correlated with transcripts changing in OOs between BS and NBS. MicroR-296-3p was previously reported to be expressed in ovaries in mice^60^ and to repress cell plasticity in different tumour lines^61^, promoting apoptosis in liver^62^ and in mammalian pancreatic α cells^63^. Recently, altered expression of bta-miR-296-3p was detected in muscle, kidney, and liver, in bovine foetuses with large offspring syndrome (LOS)^64^. In addition, miR-296 was also observed to be epigenetically regulated as a part of the imprinted Gnas/GNAS clusters^65^.

## Conclusion

In conclusion, the reduced oocyte developmental competence recorded during the NBS in buffalo is associated with changes in miRNA and mRNA content in OOs and corresponding FCs. The GO analysis showed over-representation of key genes related to lipid and sterol biosynthesis and hormone regulation, crucial for folliculogenesis and acquisition of oocyte competence. These observations might help to explain the seasonal difference in the potential of buffalo oocytes, thus providing the basis for the development of strategies to improve oocyte competence in the NBS. Nevertheless, further efforts are still needed to validate expression modulation of miRNAs and key genes identified in our study and deeply investigate their role in seasonal reproduction in buffalo.

## Materials and methods

### Collection of oocytes and granulosa cells

The study was carried out in Southern Italy (latitude 40.5°–41.5° N and longitude 13.5–15.5) in October, i.e. autumn (BS) and January, i.e. mid-winter (NBS). Buffalo ovaries were collected at a local slaughterhouse (Real Beef s.r.l., Flumeri (AV), Italy under national food hygiene regulations, and transported to the laboratory in physiological saline supplemented with 150 mg/L kanamycin at 30–35 °C within 4 h after slaughter. In order to reduce variability, the ovaries were collected from a homogeneous population of buffaloes, i.e. 134 cyclic multiparous Italian Mediterranean Buffalo cows with a mean weight and age of 552.6. ± 12.1 kg and 5.3 ± 0.4 years, over a total of 10 replicates (5/season). Cyclic ovarian activity was assessed by two clinical examinations carried out 12 days apart before slaughter, to detect the presence of a follicle greater than 1 cm and/or corpus luteum on the ovary.

For each day of collection (n = 10), 2–8 mm follicles were aspirated under controlled pressure to collect both OOs and FCs for molecular analyses, while a group of cumulus oocyte complexes (COCs) were in vitro matured, fertilized and cultured up to the blastocyst stage (n = 238 and 234, respectively in the BS and NBS).

Follicular fluid was aspirated using an 18 G needle under vacuum (40–50 mm Hg) in Falcon tubes and poured into a petri dish for COC recovery. The COCs were evaluated according to morphology and classified according to Di Francesco et al.^10^. Grade A and B COCs, considered suitable for in vitro embryo production (IVEP), were quickly selected from the dish and washed thoroughly in medium H199.

For each replicate, COCs were denuded of their cumulus cells by gentle pipetting and denuded oocytes were washed in phosphate buffer solution (PBS) + 0.1% polyvinyl alcohol (PVA), pooled (20/pool), snap frozen in liquid nitrogen and stored at − 80 °C until RNA isolation.

The follicular fluid was centrifuged at 300×*g* for 10 min at 4 °C to separate the follicular fluid and the FCs. After centrifugation, the supernatant was centrifuged again at 2000*g* for 10 min and the pellet containing FCs was snap frozen in liquid nitrogen and stored at − 80 °C until RNA isolation.

### In vitro embryo production

Unless otherwise stated, reagents were purchased from Sigma Chemical Company (Milano, Italy). The methods for in vitro maturation (IVM) described below have been reproduced in part from Gasparrini et al.^66^. For each replicate, Grade A and B COCs recovered by follicular aspiration were rinsed in HEPES-buffered TCM199 supplemented with 10% fetal calf serum (FCS) and in vitro matured, fertilized and cultured to the blastocyst stage. Briefly, COCs were allocated to 50 µL drops (10 per drop) of IVM medium, i.e. in TCM199 buffered with 25 mM sodium bicarbonate and supplemented with 10% FCS, 0.2 mM sodium pyruvate, 0.5 µg/mL FSH, 5 µg/mL LH, 1 µg/mL 17 β-estradiol and 50 µg/mL kanamycin, and incubated at 38.5° C for 21 h in a controlled gas atmosphere of 5% CO_2_ in humidified air^66^.

The methods for in vitro fertilization (IVF) and culture (IVC) described below have been reproduced from Di Francesco et al. 2012^11^. Frozen straw from a bull previously tested for IVF were thawed at 37 °C for 40 s and sperm was selected by centrifugation (25 min at 300*g*) on a Percoll discontinuous gradient (45% and 80%). The sperm pellet was re-suspended to a final concentration of 2 × 10^6^ mL^−1^ in the IVF medium, consisting of Tyrode albumin lactate pyruvate^67^ supplemented with 0.2 mM penicillamine, 0.1 mM hypotaurine and 0.01 mM heparin. Insemination was performed in 50 µL drops of IVF medium under mineral oil (5 oocytes per drop) at 38.5° C under humidified 5% CO_2_ in air. Twenty hours after IVF, putative zygotes were removed from the IVF medium, stripped of cumulus cells by gentle pipetting and allocated to 20 µ@@L drops of IVC medium, i.e. synthetic oviduct fluid (SOF) including essential and non-essential amino acids and 8 mg/mL bovine serum albumin^68^. Culture was carried out under humidified air with 5% CO_2_, 7% O_2_ and 88% N_2_ at 38.5 °C. On day 5 post-insemination (pi) the cleavage rate was assessed and the embryos transferred into fresh medium for further 2 days of IVC, when blastocyst rates were recorded.

### RNA isolation

Samples for RNA isolation were obtained from pools (n = 20) of OOs and FCs for both conditions (BS and NBS). The methods described below have been reproduced in part from Lange-Consiglio et al.^69^. Total RNA was isolated by NucleoSpin miRNA kit (Macherey–Nagel, Germany), following the protocol in combination with TRIzol (Invitrogen, Carlsbad, CA, USA) lysis with small and large RNA in one fraction (total RNA). Concentration and quality of RNA were determined by Agilent 2,100 Bioanalyzer (RIN ≥ 6.5 and 7.5 for OOs and FCs, respectively) (Santa Clara, CA, USA). The isolated RNAs were stored at − 80 °C until use.

### Library preparation and sequencing

In total, 20 libraries of small RNA and 20 libraries of RNA-Seq were obtained from five animals per group (n = 5) of two cellular types (OOs and FCs) in both seasons (BS and NBS). Small RNA libraries were prepared using TruSeq Small RNA Library Preparation kit, according to manufacturer’s instructions (Illumina). Small RNA (sRNAs) libraries were pooled together and purified with Agencourt AMPure XP (Beckman, Coulter, Brea, CA) (1 Vol. sample: 1.8 Vol. beads) twice^69^. The methods described below have been reproduced in part from Frattini et al. 2017^70^. RNA-Seq libraries were generated using the Illumina TruSeq RNA Sample Preparation v2 Kit but with one-half of the recommended reagent volumes. Concentration and profile of libraries were determined by Agilent 2100 Bioanalyzer before library sequencing on a single lane of Illumina Novaseq 6000 (San Diego, CA, USA), (Supplementary file 9).

### Data analysis

#### miRNA analysis

Illumina raw sequences were quality checked with FastQC (https://www.bioinformatics.babraham.ac.uk/projects/fastqc/) and trimmed with Trimmomatic (version 0.32)^71^, then miRDeep2 (miRDeep2 (version 2.0.0.5)^72^ was used for miRNA detection and discovery. Known miRNAs available at MirBase (https://www.mirbase.org/) were used to support miRNA identification. In particular, Bos taurus miRNAs were input to support known miRNA detection and miRNAs from related species (sheep, goat and human) were input to support novel miRNA identification. All the identified miRNAs were quantified using the miRDeep2 quantifier module. The Bioconductor edgeR package (version 2.4) was used to identify statistically significant differential expression between groups of samples (false discovery rate [FDR] < 0.05)^73^. Predicted miRNA gene targeting of differentially expressed *Bos taurus* miRNAs (DEmiRNAs) was performed with miRWalk2.0^74^, using homologous human miRNAs as input identifiers.

Target genes were submitted to GO analysis. GO classification of the DEGs was performed according to canonical GO categories, using the Cytoscape (version.3.2.1) plug-in ClueGO (version 2.3.5) which integrates GO and enhances biological interpretation of large lists of genes^75^. MicroRNA cluster analysis was performed with Genesis (version1.8.1)^76^.

#### RNA-seq analysis

RNA-Seq raw data were trimmed using Trimmomatic (version 0.32)^71^. Sequences were aligned to the buffalo reference genome version UOA_WB_1 (GCF_003121395.1) using STAR_2.3.0^77^. Subsequently, HTSeq-count (version 0.6.1p1)^78^ was used to count sequences aligned to each gene. The software package EdgeR of Bioconductor (version 3.6) was used to estimate differential expression between groups of samples^73^. RNAseq cluster analysis was performed with Genesis (version1.8.1)^76^. Differentially expressed genes DEGs were submitted to GO analysis, using the Cytoscape (version.3.2.1) plug-in ClueGO (version 2.3.5)^75^. Venn diagrams for intersection between DEGs and miRNAs target genes, using the Venn Diagrams software (https://bioinfogp.cnb.csic.es/tools/venny/ version 2.1).

#### In vitro embryo production

Differences in cleavage and blastocyst rates between seasons were analyzed by Chi square test. The level of significance was set at *P* < 0.05.

## Supplementary information

Supplementary file S1–S9.

Supplementary file S10.

Supplementary file S11.

## Data Availability

RNA-Seq data are available in the Sequence Reads Archive (SRA), BioProject accession number, PRJNA599337. Novel miRNA precursors and novel miRNA mature sequences are reported in Supplementary files S10 and S11.
