# Supplementary material for: Seasonal effects on miRNA and transcriptomic profile of oocytes and follicular cells in buffalo (Bubalus bubalis)
Source: Sci Rep. 2020 Aug 11;10:13557. doi: 10.1038/s41598-020-70546-5 (PMC7419291; doi:10.1038/s41598-020-70546-5)
Supplement: Supplementary file 1 — Supplementary file S1–S9. [file 41598_2020_70546_MOESM1_ESM.pdf]

Seasonal effects on miRNA and transcriptomic profile of oocytes and follicular cells in buffalo (Bubalus bubalis)

Emanuele Capra[1†], Barbara Lazzari[1†], Marco Russo[2], Michal Andrzej Kosior[2], Giovanni Della Valle[2], Valentina Longobardi[2], Alessandra Stella[1], Anna Lange Consiglio[3,4]*, Bianca Gasparrini[2]

[1]Istituto di Biologia e Biotecnologia Agraria, Consiglio Nazionale delle Ricerche IBBA CNR, Lodi;
[2]Department of Veterinary Medicine and Animal Production, Federico II University, Via F. Delpino 1, 80137 Naples, Italy;
[3]Università degli Studi di Milano, Dipartimento di Medicina Veterinaria (DIMEVET);
[4]Centro Clinico-Veterinario e Zootecnico-Sperimentale di Ateneo, Università degli Studi di Milano.

	NBS	NBS	NBS	NBS	NBS	BS	BS	BS	BS	BS	
Oocytes	OO1	OO2	OO3	OO4	OO5	OO6	OO7	OO8	OO9	OO10	Average
total reads count after trimming	1.0E+07	9.9E+06	5.2E+06	9.8E+06	1.3E+07	7.7E+06	1.2E+07	1.2E+07	9.2E+06	6.4E+06	9.5E+06
n. of reads assigned to miRNAs	9.7E+04	6.2E+04	5.3E+04	9.4E+04	1.6E+05	1.2E+05	2.2E+05	1.8E+05	1.1E+05	9.1E+04	1.2E+05
n. of reads assigned to miRNAs (%)	0.95	0.62	1.02	0.96	1.27	1.59	1.87	1.47	1.22	1.42	1.25
Follicular Cells	FC1	FC2	FC3	FC4	FC5	FC6	FC7	FC8	FC9	FC10	Average
total reads count after trimming	1.1E+07	7.0E+06	7.3E+06	6.3E+06	9.0E+06	1.4E+07	8.6E+06	1.4E+07	9.5E+06	9.6E+06	9.6E+06
n. of reads assigned to miRNAs	2.2E+06	1.6E+06	1.5E+06	8.9E+05	2.3E+06	1.9E+06	1.9E+06	1.9E+06	2.4E+06	1.6E+06	1.8E+06
n. of reads assigned to miRNAs (%)	20.39	23.42	20.46	14.05	25.90	12.87	22.47	14.22	25.53	17.21	19.21

Supplementary file S1. Statistics of miRNA sequencing experiments for oocytes (OO1-OO10) and follicular cells (FC1-FC10) in the breeding (BS) and non-breeding (NBS) season.

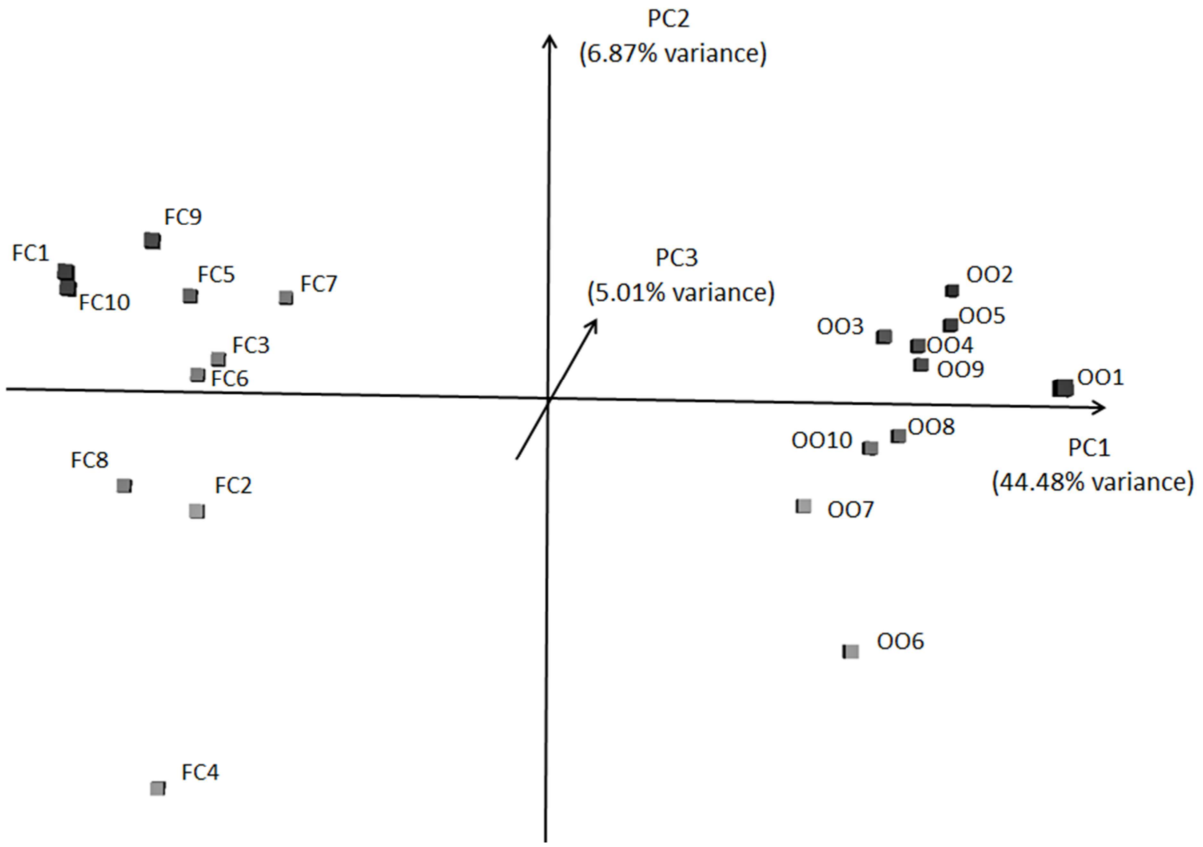

Supplementary file S2. Principal component analysis considering the 769 miRNAs expressed at least in triplicate in oocytes (OO) or follicular cell (FCs) samples.

	% OO abundance	% FC abundance	Fold OO/FC
bta-miR-10b	34.03785	30.66203	1.11010
bta-miR-148a	5.42230	4.59631	1.17971
bta-miR-92a	5.13207	1.96445	2.61247
bta-miR-30d	4.92369	1.69054	2.91250
bta-miR-22-3p	3.92559	2.15180	1.82433
bta-miR-26a	3.71096	3.97212	0.93425
bta-miR-30a-5p	1.60467	4.06962	0.39430
bta-miR-423-5p	1.37182	0.09851	13.92643
bta-let-7f	1.32808	2.31191	0.57445
bta-miR-30e-5p	1.18876	4.79180	0.24808
bta-miR-191	1.12845	2.66746	0.42304
bta-miR-16b	0.85828	0.99769	0.86026
bta-let-7a-5p	0.73408	2.91948	0.25144
bta-miR-21-5p	0.65680	11.03253	0.05953
bta-miR-423-3p	0.65529	0.25088	2.61197
bta-miR-186	0.62359	2.11354	0.29505
bta-miR-27b	0.53922	0.61845	0.87189
bta-miR-30c	0.46863	0.43714	1.07202
bta-miR-31	0.45871	1.20450	0.38083
bta-let-7i	0.38377	1.75338	0.21887
bta-miR-25	0.37702	1.40101	0.26910
bta-miR-92b	0.32584	0.14228	2.29014
bta-miR-181a	0.30948	0.64858	0.47717
bta-miR-30b-5p	0.30932	0.23696	1.30541
bta-miR-125a	0.27857	0.45654	0.61018
bta-miR-182	0.24195	0.00133	181.60813
bta-miR-143	0.22538	0.60919	0.36997
bta-miR-660	0.22192	0.39444	0.56263
bta-miR-16a	0.21692	0.44771	0.48452
bta-miR-9-5p	0.21210	0.00047	447.72485
bta-let-7b	0.19667	0.56032	0.35099
bta-miR-204	0.15701	0.14042	1.11811
bta-miR-767	0.13949	0.00018	769.45061
bta-miR-6119-5p	0.13077	0.51315	0.25484
bta-miR-34c	0.12966	0.02313	5.60487
bta-let-7g	0.12656	0.60219	0.21016
bta-miR-155	0.12166	0.23570	0.51614
bta-miR-19b	0.12127	0.70003	0.17323
bta-let-7e	0.11651	0.21584	0.53979
bta-miR-151-3p	0.11642	0.31832	0.36572
bta-miR-101	0.11490	0.35791	0.32103
bta-miR-146b	0.11358	0.02781	4.08479
bta-miR-301a	0.10497	0.17995	0.58333
bta-let-7c	0.10303	0.20201	0.51002

bta-miR-26b	0.10092	0.42875	0.23539
bta-miR-29a	0.09178	0.18540	0.49503
bta-miR-2284x	0.09114	0.13298	0.68535
bta-miR-320a	0.08893	0.15816	0.56227
bta-miR-1247-5p	0.08791	0.00024	365.37759
bta-miR-151-5p	0.08746	0.15661	0.55848
bta-miR-17-5p	0.08314	0.22837	0.36407
bta-miR-93	0.07639	0.53810	0.14196
bta-miR-375	0.07510	0.00062	121.15423
bta-miR-1246	0.07227	0.03272	2.20892
bta-miR-138	0.07067	0.00044	160.41646
bta-miR-10a	0.07025	0.45398	0.15473
bta-miR-103	0.06933	0.32205	0.21528
bta-miR-342	0.06669	0.11808	0.56479
bta-miR-130a	0.06540	0.38037	0.17192
bta-miR-140	0.06337	0.38690	0.16380
bta-miR-202	0.06000	0.62267	0.09635
bta-miR-425-5p	0.05828	0.08659	0.67303
bta-miR-192	0.05587	0.05487	1.01809
bta-miR-125b	0.05420	0.06167	0.87880
bta-miR-195	0.05361	0.47051	0.11394
bta-miR-532	0.05332	0.18610	0.28650
bta-miR-222	0.05050	0.09286	0.54377
bta-miR-378	0.04954	0.34968	0.14166
bta-miR-99b	0.04876	0.41759	0.11678
bta-miR-146a	0.04814	0.00684	7.03267
bta-miR-497	0.04733	0.22060	0.21453
bta-miR-141	0.04666	0.00054	86.64133
bta-miR-210	0.04326	0.09832	0.44003
bta-miR-339a	0.04187	0.05036	0.83149
bta-let-7d	0.04160	0.06596	0.63071
bta-miR-27a-3p	0.04156	0.04104	1.01278
bta-miR-20a	0.04127	0.23073	0.17888
bta-miR-181b	0.04106	0.07381	0.55633
bta-miR-105a	0.04032	0.00002	1.775.87505
bta-miR-148b	0.03664	0.29120	0.12583
bta-miR-200c	0.03584	0.00039	92.36705
bta-miR-335	0.03446	0.01391	2.47757
bta-miR-449a	0.03310	0.01699	1.94869
bta-miR-10174-3p	0.03222	0.54534	0.05908
bta-miR-19a	0.02889	0.17966	0.16079
bta-miR-128	0.02698	0.01365	1.97687
bta-miR-6123	0.02454	0.00861	2.85061
bta-miR-2483-5p	0.02436	0.00813	2.99500
bta-miR-421	0.02305	0.04152	0.55503

bta-miR-296-3p	0.02226	0.02758	0.80700
bta-miR-1468	0.02083	0.02160	0.96448
bta-miR-105b	0.02073	-	#DIV/0!
bta-miR-98	0.02060	0.45826	0.04495
bta-miR-450b	0.02010	0.09897	0.20313
bta-miR-15b	0.01962	0.07138	0.27480
bta-miR-181c	0.01948	0.02833	0.68773
bta-miR-338	0.01766	0.00207	8.53794
bta-miR-486	0.01741	0.15659	0.11116
bta-miR-107	0.01654	0.04195	0.39437
bta-miR-194	0.01644	0.00776	2.11887
bta-miR-1839	0.01606	0.02299	0.69840
bta-miR-361	0.01525	0.04550	0.33513
bta-miR-484	0.01523	0.02649	0.57498
bta-miR-99a-5p	0.01501	0.10516	0.14274
bta-miR-15a	0.01465	0.04055	0.36134
bta-miR-2435	0.01428	0.01262	1.13148
bta-miR-221	0.01368	0.11332	0.12074
bta-miR-199a-3p	0.01365	0.01912	0.71391
bta-miR-744	0.01315	0.01869	0.70355
bta-miR-2478	0.01308	0.00341	3.83075
bta-miR-328	0.01242	0.00351	3.53680
bta-miR-30f	0.01229	0.09178	0.13391
bta-miR-769	0.01216	0.05577	0.21805
bta-miR-133a	0.01216	0.00112	10.81602
bta-miR-877	0.01065	0.00106	10.01770
bta-miR-18a	0.01044	0.05455	0.19134
bta-miR-2484	0.00988	0.00403	2.44991
bta-miR-1307	0.00970	0.02296	0.42251
bta-miR-7857-5p	0.00921	0.00008	117.57951
bta-miR-24-3p	0.00918	0.08302	0.11057
bta-miR-193a-3p	0.00908	0.03575	0.25401
bta-miR-28	0.00899	0.02515	0.35734
bta-miR-2440	0.00893	0.03190	0.27980
bta-miR-106b	0.00857	0.04587	0.18675
bta-miR-331-3p	0.00844	0.02629	0.32115
bta-miR-23b-3p	0.00841	0.14202	0.05924
bta-miR-34b	0.00807	0.00207	3.89137
bta-miR-127	0.00807	0.02887	0.27957
bta-miR-2483-3p	0.00748	0.00132	5.64658
bta-miR-1343-3p	0.00746	0.00832	0.89653
bta-miR-216b	0.00740	-	#DIV/0!
bta-miR-2285aj-5p	0.00735	0.01365	0.53822
bta-miR-383	0.00644	0.00047	13.65999
bta-miR-132	0.00611	0.01185	0.51534

bta-miR-424-5p	0.00608	0.02182	0.27859
bta-let-7a-3p	0.00607	0.02073	0.29280
bta-miR-505	0.00599	0.01355	0.44193
bta-miR-149-5p	0.00590	0.01301	0.45338
bta-miR-205	0.00562	0.00003	204.38735
bta-miR-677	0.00550	0.04171	0.13184
bta-miR-363	0.00494	0.00010	51.13653
bta-miR-652	0.00493	0.04253	0.11603
bta-miR-193b	0.00486	0.06798	0.07155
bta-miR-374a	0.00474	0.05490	0.08634
bta-miR-301b	0.00472	0.00796	0.59237
bta-miR-183	0.00467	0.00013	35.26569
bta-miR-374b	0.00446	0.03670	0.12157
bta-miR-451	0.00435	0.09792	0.04441
bta-miR-17-3p	0.00428	0.00889	0.48192
bta-miR-365-3p	0.00428	0.01476	0.28984
bta-miR-574	0.00422	0.05606	0.07522
bta-miR-449c	0.00419	0.00531	0.78976
bta-miR-362-5p	0.00419	0.00845	0.49519
bta-miR-7857-3p	0.00415	0.00040	10.44392
bta-miR-499	0.00415	0.00126	3.28711
bta-miR-181d	0.00409	0.00526	0.77795
bta-miR-3660	0.00399	0.00001	354.20167
bta-miR-106a	0.00393	0.00123	3.20070
bta-miR-100	0.00390	0.01800	0.21670
bta-miR-20b	0.00370	0.00086	4.32566
bta-miR-9-3p	0.00366	-	#DIV/0!
bta-miR-126-3p	0.00356	0.04405	0.08078
bta-miR-152	0.00354	0.00650	0.54430
bta-miR-199b	0.00346	0.00782	0.44192
bta-miR-199a-5p	0.00330	0.01023	0.32245
bta-miR-145	0.00326	0.02638	0.12371
bta-miR-760-3p	0.00324	0.00328	0.98860
bta-miR-29c	0.00321	0.01156	0.27787
bta-miR-2285bz	0.00320	0.00627	0.51102
bta-miR-99a-3p	0.00315	0.00882	0.35704
bta-miR-197	0.00307	0.02917	0.10540
bta-miR-12006	0.00304	0.00015	19.64503
bta-miR-142-5p	0.00293	0.01495	0.19627
bta-miR-153	0.00292	0.02609	0.11205
bta-miR-450a	0.00290	0.01033	0.28067
bta-miR-1296	0.00281	0.01431	0.19616
bta-miR-411a	0.00280	0.00440	0.63543
bta-miR-454	0.00275	0.01721	0.16008
bta-miR-218	0.00275	0.00043	6.40416

bta-miR-21-3p	0.00274	0.04610	0.05952
bta-miR-6518	0.00271	0.00180	1.50390
bta-miR-2904	0.00271	0.00080	3.38043
bta-miR-126-5p	0.00265	0.01341	0.19753
bta-miR-500	0.00251	0.00580	0.43265
bta-miR-23a	0.00249	0.01258	0.19757
bta-miR-7	0.00248	0.00998	0.24829
bta-miR-1224	0.00236	0.00055	4.30993
bta-miR-2285cj	0.00233	0.01034	0.22496
bta-miR-874	0.00221	0.00603	0.36735
bta-miR-502b	0.00219	0.00531	0.41288
bta-miR-424-3p	0.00215	0.00080	2.70201
bta-miR-29b	0.00215	0.01958	0.10970
bta-miR-122	0.00208	-	#DIV/0!
bta-miR-215	0.00206	0.00689	0.29895
bta-miR-188	0.00192	0.00607	0.31585
bta-miR-96	0.00189	0.00002	99.68565
bta-miR-1271	0.00187	0.02228	0.08393
bta-miR-2285bl	0.00185	0.01109	0.16670
bta-miR-184	0.00172	0.00012	14.05760
bta-miR-340	0.00169	0.00146	1.15559
bta-miR-6517	0.00168	0.00222	0.75587
bta-miR-455-5p	0.00159	0.00744	0.21379
bta-miR-214	0.00158	0.00233	0.67898
bta-miR-381	0.00153	0.00276	0.55532
bta-miR-200a	0.00150	0.00195	0.76798
bta-miR-187	0.00149	0.00002	69.78911
bta-miR-504	0.00146	0.00493	0.29561
bta-miR-150	0.00139	0.00091	1.53434
bta-miR-4449	0.00137	0.00217	0.63272
bta-miR-503-5p	0.00134	0.00067	2.00862
bta-miR-190b	0.00132	0.02033	0.06474
bta-miR-1260b	0.00128	0.00159	0.80684
bta-miR-628	0.00128	0.00224	0.56997
bta-miR-502a	0.00124	0.00021	5.82776
bta-miR-32	0.00123	0.00774	0.15844
bta-miR-154c	0.00121	0.00079	1.52615
bta-miR-223	0.00114	0.00135	0.84249
bta-miR-2285bn	0.00113	0.00811	0.13915
bta-miR-551b	0.00112	0.00920	0.12197
bta-miR-2285e	0.00112	0.00368	0.30443
bta-miR-1388-5p	0.00111	0.01265	0.08761
bta-miR-2424	0.00108	0.00110	0.98026
bta-miR-18b	0.00107	0.00018	5.96806
bta-miR-6529a	0.00104	0.00098	1.06989

bta-miR-425-3p	0.00103	0.00394	0.26179
bta-miR-339b	0.00102	0.00106	0.96538
bta-miR-190a	0.00100	0.00315	0.31661
bta-miR-2411-5p	0.00099	0.00018	5.64657
bta-miR-449b	0.00099	0.00292	0.33841
bta-miR-345-3p	0.00095	0.00913	0.10403
bta-miR-708	0.00094	0.00787	0.11963
bta-miR-11986b	0.00091	0.00179	0.50733
bta-miR-1249	0.00088	0.00141	0.62556
bta-miR-2285cm	0.00088	0.00303	0.29026
bta-miR-331-5p	0.00088	0.00321	0.27342
bta-miR-34a	0.00086	0.00309	0.27926
bta-miR-7180	0.00084	0.00053	1.57507
bta-miR-224	0.00083	0.00084	0.98555
bta-miR-2284ab	0.00082	0.00363	0.22650
bta-miR-1291	0.00081	0.00023	3.50109
bta-miR-491	0.00076	0.00369	0.20609
bta-miR-1388-3p	0.00074	0.00217	0.34218
bta-miR-2285ce	0.00073	0.00313	0.23250
bta-miR-1306	0.00070	0.00262	0.26684
bta-miR-2285dh	0.00069	0.00259	0.26746
bta-miR-2318	0.00068	0.00500	0.13491
bta-miR-2411-3p	0.00067	0.00119	0.56808
bta-miR-2285f	0.00060	0.00321	0.18668
bta-miR-208b	0.00060	0.00062	0.95954
bta-miR-665	0.00058	0.00068	0.84934
bta-miR-3431	0.00058	0.00179	0.32174
bta-miR-2285q	0.00054	0.00309	0.17338
bta-miR-483	0.00053	0.00150	0.35529
bta-miR-216a	0.00051	0.00002	30.95036
bta-miR-142-3p	0.00050	0.00553	0.09126
bta-miR-193a-5p	0.00048	0.00062	0.77960
bta-miR-2285p	0.00048	0.00044	1.10058
bta-miR-10164-3p	0.00048	0.00141	0.33918
bta-miR-671	0.00048	0.00216	0.22060
bta-miR-2285s	0.00047	0.00049	0.96156
bta-miR-455-3p	0.00046	0.00196	0.23444
bta-miR-6520	0.00045	0.00060	0.74672
bta-miR-135a	0.00044	0.00303	0.14526
bta-miR-2284y	0.00044	0.00554	0.07908
bta-miR-2285co	0.00043	0.00059	0.73279
bta-miR-2887	0.00043	0.00014	3.11773
bta-miR-2299-3p	0.00042	0.00003	12.68612
bta-miR-362-3p	0.00041	0.00071	0.58265
bta-miR-2284z	0.00038	0.00055	0.67868

bta-miR-296-5p	0.00037	0.00022	1.68374
bta-miR-592	0.00035	0.00023	1.52761
bta-miR-324	0.00035	0.00412	0.08515
bta-miR-6119-3p	0.00033	0.00571	0.05861
bta-miR-2284aa	0.00032	0.00014	2.32694
bta-miR-130b	0.00032	0.00005	6.67716
bta-miR-2285k	0.00031	0.00693	0.04548
bta-miR-27a-5p	0.00031	0.00451	0.06957
bta-miR-487b	0.00030	0.00017	1.73913
bta-miR-212	0.00027	0.00088	0.31216
bta-miR-582	0.00027	0.00068	0.38894
bta-miR-380-3p	0.00027	0.00006	4.22549
bta-miR-432	0.00025	0.00072	0.35044
bta-miR-503-3p	0.00025	0.00067	0.38046
bta-miR-542-5p	0.00025	0.00191	0.13286
bta-miR-3613a	0.00025	0.00209	0.12025
bta-miR-545-5p	0.00024	0.00390	0.06253
bta-miR-2285ai-5p	0.00023	0.00003	7.93536
bta-miR-24	0.00023	0.00119	0.19398
bta-miR-33a	0.00022	0.00022	1.02783
bta-miR-760-5p	0.00022	0.00007	3.28184
bta-miR-135b	0.00021	0.00001	14.66346
bta-miR-196a	0.00021	0.00010	2.14151
bta-miR-1298	0.00021	-	#DIV/0!
bta-miR-2419-5p	0.00021	0.00004	4.70690
bta-miR-6524	0.00021	0.00016	1.27826
bta-miR-12034	0.00020	0.00017	1.17689
bta-miR-2299-5p	0.00020	0.00131	0.15293
bta-miR-664b	0.00019	0.00023	0.83101
bta-miR-139	0.00017	0.00040	0.41598
bta-miR-378b	0.00016	0.00226	0.07293
bta-miR-2285ag-3p	0.00014	0.00020	0.72809
bta-miR-2284p	0.00014	0.00008	1.75175
bta-miR-2285da	0.00014	0.00022	0.62635
bta-miR-147	0.00014	0.00090	0.15210
bta-miR-493	0.00014	0.00036	0.37755
bta-miR-10182-5p	0.00013	0.00008	1.70838
bta-miR-485	0.00013	0.00004	3.14110
bta-miR-2284b	0.00013	0.00031	0.40762
bta-miR-543	0.00013	0.00007	1.71201
bta-miR-208a	0.00012	0.00028	0.44446
bta-miR-7859	0.00012	0.00005	2.47006
bta-miR-2285c	0.00012	0.00021	0.54906
bta-miR-196b	0.00011	0.00005	2.43162
bta-miR-185	0.00011	0.00103	0.10315

bta-miR-374c	0.00011	0.00052	0.20563
bta-miR-10182-3p	0.00010	0.00010	1.04815
bta-miR-137	0.00010	0.00022	0.46779
bta-miR-379	0.00010	0.00006	1.60135
bta-miR-299	0.00009	0.00002	4.07354
bta-miR-95	0.00009	0.00024	0.37762
bta-miR-129-3p	0.00009	0.00028	0.32105
bta-miR-2284k	0.00009	0.00036	0.24747
bta-miR-488	0.00009	0.00013	0.70235
bta-miR-191b	0.00008	0.00016	0.51920
bta-miR-200b	0.00008	0.00037	0.22016
bta-miR-378c	0.00008	0.00146	0.05578
bta-miR-410	0.00008	0.00019	0.42283
bta-miR-655	0.00008	0.00007	1.09498
bta-miR-873	0.00008	0.00026	0.30823
bta-miR-3432a	0.00006	0.00007	0.93865
bta-miR-452	0.00006	0.00011	0.56718
bta-miR-433	0.00006	0.00006	0.87529
bta-miR-494	0.00006	0.00060	0.09285
bta-miR-6516	0.00006	0.00002	2.58925
bta-miR-219-5p	0.00005	0.00007	0.61448
bta-miR-2285ab	0.00005	0.00010	0.44296
bta-miR-654	0.00005	0.00005	0.95178
bta-miR-10172-3p	-	0.00004	-
bta-miR-11972	-	0.00010	-
bta-miR-11985	-	0.00011	-
bta-miR-11986c	-	0.00060	-
bta-miR-11987	-	0.00009	-
bta-miR-11991	-	0.00007	-
bta-miR-11992	-	0.00030	-
bta-miR-11996	-	0.00002	-
bta-miR-12004	-	0.00002	-
bta-miR-12017	-	0.00017	-
bta-miR-12023	-	0.00005	-
bta-miR-12030	-	0.00011	-
bta-miR-12031	-	0.00001	-
bta-miR-12038	-	0.00002	-
bta-miR-12041	-	0.00006	-
bta-miR-124a	-	0.00007	-
bta-miR-124b	-	0.00003	-
bta-miR-1277	-	0.00007	-
bta-miR-1343-5p	-	0.00012	-
bta-miR-136	-	0.00012	-
bta-miR-1434-3p	-	0.00002	-
bta-miR-1434-5p	-	0.00027	-

bta-miR-144	-	0.00016	-
bta-miR-154b	-	0.00004	-
bta-miR-1949	-	0.00002	-
bta-miR-194b	-	0.00032	-
bta-miR-194b-3p	-	0.00032	-
bta-miR-199c	-	0.00043	-
bta-miR-211	-	0.00015	-
bta-miR-219	-	0.00018	-
bta-miR-219b-3p	-	0.00010	-
bta-miR-22-5p	-	0.00213	-
bta-miR-2284a	-	0.00057	-
bta-miR-2284c	-	0.00030	-
bta-miR-2284f	-	0.00012	-
bta-miR-2284o	-	0.00009	-
bta-miR-2284v	-	0.00011	-
bta-miR-2284w	-	0.00005	-
bta-miR-2285aa	-	0.00037	-
bta-miR-2285ad	-	0.00025	-
bta-miR-2285ak-5p	-	0.00006	-
bta-miR-2285al-5p	-	0.00003	-
bta-miR-2285am-5p	-	0.00005	-
bta-miR-2285ao	-	0.00003	-
bta-miR-2285ar	-	0.00014	-
bta-miR-2285au	-	0.00039	-
bta-miR-2285aw	-	0.00063	-
bta-miR-2285b	-	0.00090	-
bta-miR-2285be	-	0.00003	-
bta-miR-2285bf	-	0.00014	-
bta-miR-2285bo	-	0.00009	-
bta-miR-2285bp	-	0.00007	-
bta-miR-2285br	-	0.00048	-
bta-miR-2285bs	-	0.00004	-
bta-miR-2285bu	-	0.00024	-
bta-miR-2285bw	-	0.00031	-
bta-miR-2285ch	-	0.00008	-
bta-miR-2285cw	-	0.00006	-
bta-miR-2285dc	-	0.00005	-
bta-miR-2285dd	-	0.00010	-
bta-miR-2285dl-3p	-	0.00020	-
bta-miR-2285g	-	0.00009	-
bta-miR-2285l	-	0.00002	-
bta-miR-2285m	-	0.00002	-
bta-miR-2285o	-	0.00022	-
bta-miR-2285u	-	0.00003	-
bta-miR-2311	-	0.00003	-

bta-miR-2312	-	0.00014	-
bta-miR-2314	-	0.00006	-
bta-miR-2320-3p	-	0.00002	-
bta-miR-2331-3p	-	0.00039	-
bta-miR-2331-5p	-	0.00006	-
bta-miR-2332	-	0.00104	-
bta-miR-2334	-	0.00004	-
bta-miR-2335	-	0.00003	-
bta-miR-2355-3p	-	0.00009	-
bta-miR-2366	-	0.00002	-
bta-miR-2367-5p	-	0.00010	-
bta-miR-2370-3p	-	0.00040	-
bta-miR-2373-5p	-	0.00002	-
bta-miR-2382-3p	-	0.00006	-
bta-miR-2387	-	0.00027	-
bta-miR-2389	-	0.00002	-
bta-miR-2397-3p	-	0.00008	-
bta-miR-2403	-	0.00015	-
bta-miR-2416	-	0.00039	-
bta-miR-2422	-	0.00003	-
bta-miR-2427	-	0.00008	-
bta-miR-2431-3p	-	0.00002	-
bta-miR-2431-5p	-	0.00003	-
bta-miR-2432	-	0.00002	-
bta-miR-2446	-	0.00030	-
bta-miR-2447	-	0.00002	-
bta-miR-2461-3p	-	0.00006	-
bta-miR-2468	-	0.00048	-
bta-miR-2475	-	0.00011	-
bta-miR-2477	-	0.00009	-
bta-miR-2903	-	0.00005	-
bta-miR-29d-5p	-	0.00100	-
bta-miR-29e	-	0.00005	-
bta-miR-30b-3p	-	0.00049	-
bta-miR-326	-	0.00014	-
bta-miR-330	-	0.00023	-
bta-miR-33b	-	0.00013	-
bta-miR-345-5p	-	0.00046	-
bta-miR-365-5p	-	0.00029	-
bta-miR-369-3p	-	0.00006	-
bta-miR-376b	-	0.00002	-
bta-miR-376e	-	0.00005	-
bta-miR-378d	-	0.00008	-
bta-miR-382	-	0.00011	-
bta-miR-409a	-	0.00002	-

bta-miR-411c-3p	-	0.00076	-
bta-miR-411c-5p	-	0.00031	-
bta-miR-4286	-	0.00005	-
bta-miR-429	-	0.00026	-
bta-miR-487a	-	0.00006	-
bta-miR-490	-	0.00006	-
bta-miR-495	-	0.00031	-
bta-miR-541	-	0.00013	-
bta-miR-545-3p	-	0.00196	-
bta-miR-6121-3p	-	0.00013	-
bta-miR-615	-	0.00004	-
bta-miR-6522	-	0.00007	-
bta-miR-6535	-	0.00003	-
bta-miR-6536	-	0.00006	-
bta-miR-758	-	0.00001	-
bta-miR-7858	-	0.00012	-
bta-miR-7862	-	0.00002	-
bta-miR-885	-	0.00002	-
bta-miR-935	-	0.00005	-

Supplementary file S3. Percentage of *Bos taurus* bta-miRNA abundance found in oocytes (OOs) and follicular cells (FCs) and their relative ratio.

	logFC (OOs_vs_FCs)	FDR
Novel:chi-miR-30a-3p	14.96	0.00E+00
Novel:NC_037562.1_40333	13.83	0.00E+00
Novel:NC_037561.1_38157	13.33	0.00E+00
Novel:NC_037561.1_38294	13.36	2.50E-282
Novel:NC_037549.1_13176	11.09	1.96E-250
Novel:NC_037559.1_33577	14.03	5.88E-226
Novel:NC_037550.1_15803	12.79	4.15E-216
bta-miR-9-5p	9.35	1.23E-208
Novel:NC_037552.1_22735	13.24	2.59E-204
Novel:NC_037566.1_44909	14.23	3.74E-204
Novel:NC_037556.1_29003	12.43	2.03E-197
Novel:NC_037548.1_11587	11.22	1.25E-185
bta-miR-767	10.10	6.03E-184
Novel:NC_037555.1_25337	13.59	3.54E-179
bta-miR-182	8.06	1.71E-177
bta-miR-105a	11.00	1.90E-175
Novel:NC_037550.1_16127	13.09	4.38E-173
Novel:NC_037557.1_30243	13.70	1.76E-162
Novel:chi-miR-2404	12.41	5.04E-155
Novel:NC_037563.1_41848	11.56	1.27E-150
bta-miR-105b	11.19	5.67E-150

Novel:NC_037569.1_48104	8.42	1.29E-148
Novel:NC_037569.1_48108	8.42	3.05E-147
bta-miR-138	7.89	6.51E-139
Novel:NC_037547.1_8475	11.25	4.58E-133
Novel:NC_037546.1_6523	13.39	6.60E-132
Novel:NC_037547.1_8434	13.25	1.97E-131
Novel:NC_037558.1_31385	11.19	4.16E-131
Novel:NC_037546.1_6477	11.16	5.09E-127
Novel:chi-miR-345-5p	14.30	1.04E-126
Novel:NC_037554.1_24869	11.00	2.23E-118
Novel:NC_037559.1_34044	10.89	1.15E-114
Novel:NC_037553.1_24445	13.39	1.97E-113
Novel:NC_037557.1_29725	10.48	2.00E-113
Novel:NC_037548.1_12261	10.50	2.40E-113
Novel:NC_037549.1_15527	10.50	9.60E-113
Novel:NC_037560.1_36420	10.48	2.96E-112
Novel:NC_037563.1_40601	10.70	3.69E-112
Novel:NC_037550.1_18562	10.48	7.07E-111
Novel:NC_037545.1_835	12.18	1.53E-108
Novel:NC_037547.1_9642	9.70	2.52E-105
Novel:NC_037561.1_39262	12.20	7.71E-104
bta-miR-1247-5p	9.24	2.51E-102
Novel:NC_037546.1_4671	14.30	2.04E-100
Novel:NC_037563.1_42211	13.33	4.51E-95
Novel:NC_037569.1_46617	10.58	3.16E-94
Novel:NC_037550.1_16338	9.52	6.30E-91
Novel:NC_037566.1_43986	12.59	2.48E-86
Novel:NC_037550.1_16194	13.59	1.18E-85
Novel:NC_037566.1_44115	12.62	3.08E-85
Novel:NC_037549.1_13991	9.69	2.40E-82
Novel:NC_037562.1_40133	10.49	3.61E-81
Novel:NC_037569.1_48096	8.44	3.78E-81
Novel:NC_037547.1_10745	9.51	4.06E-81
Novel:NC_037569.1_48098	8.44	1.21E-80
bta-miR-30d	2.15	5.70E-79
Novel:NC_037555.1_26692	9.77	1.26E-77
Novel:NC_037552.1_22845	5.05	6.34E-76
Novel:NC_037565.1_43691	9.86	2.76E-75
Novel:NC_037558.1_31562	10.01	1.36E-72
Novel:chi-miR-665	9.22	2.33E-71
bta-miR-92a	1.99	3.62E-71
bta-miR-216b	9.71	4.26E-71
Novel:NC_037555.1_27099	10.83	2.70E-70
Novel:NC_037547.1_10452	9.74	4.83E-70
Novel:chi-miR-421-3p	9.85	2.68E-68
Novel:NC_037547.1_10766	10.59	2.65E-65
bta-miR-7857-5p	7.20	1.19E-64

bta-miR-423-5p	4.43	2.41E-63
Novel:NC_037548.1_12574	9.67	4.44E-62
Novel:NC_037556.1_28867	9.67	1.10E-61
Novel:chi-miR-1271-5p	10.11	5.65E-60
bta-miR-338	3.72	3.79E-58
Novel:NC_037547.1_10260	9.71	1.71E-56
Novel:NC_037546.1_5892	9.50	2.00E-54
Novel:NC_037551.1_21150	8.98	2.54E-54
bta-miR-3660	8.30	3.87E-52
Novel:NC_037558.1_30609	8.89	2.58E-51
Novel:NC_037550.1_17836	9.56	1.43E-49
Novel:NC_037547.1_10767	9.35	2.69E-49
Novel:chi-miR-324-5p	9.38	5.05E-49
Novel:NC_037563.1_40470	8.74	7.18E-49
Novel:NC_037549.1_14113	8.85	5.47E-48
Novel:NC_037546.1_2163	8.84	2.31E-47
Novel:chi-miR-877-3p	9.82	3.80E-47
Novel:NC_037569.1_48102	5.55	2.38E-46
Novel:NC_037569.1_48106	5.55	3.23E-46
Novel:NC_037550.1_18175	9.64	5.06E-45
Novel:NC_037549.1_14727	9.51	1.22E-44
Novel:NC_037549.1_14793	9.04	4.91E-43
Novel:NC_037561.1_37655	9.50	1.63E-42
bta-miR-183	5.53	2.18E-42
Novel:NC_037563.1_40476	8.86	3.46E-42
Novel:NC_037558.1_32432	9.70	7.11E-41
Novel:NC_037567.1_45367	8.72	1.02E-40
Novel:NC_037546.1_6365	8.63	3.79E-40
Novel:NC_037558.1_32599	9.30	5.51E-40
Novel:NC_037561.1_36870	8.66	2.22E-39
bta-miR-21-5p	-3.52	9.33E-38
Novel:NC_037555.1_25442	8.21	1.19E-37
Novel:NC_037548.1_11964	8.56	2.78E-37
Novel:NC_037558.1_32568	9.04	1.54E-36
Novel:NC_037545.1_1469	3.85	2.97E-36
bta-miR-10174-3p	-3.60	3.80E-36
bta-miR-375	7.47	4.74E-36
Novel:NC_037548.1_11355	8.40	5.44E-36
Novel:NC_037556.1_28703	8.61	2.39E-34
bta-miR-363	6.19	9.10E-34
bta-miR-205	7.59	1.44E-33
Novel:NC_037545.1_51	7.99	2.72E-33
bta-miR-877	3.96	5.47E-33
bta-miR-98	-3.99	2.66E-32
Novel:NC_037546.1_5316	8.75	3.92E-32
Novel:NC_037565.1_43646	9.10	4.05E-31
bta-miR-93	-2.26	5.86E-31

Novel:NC_037551.1_21843	8.46	8.33E-31
bta-miR-23b-3p	-3.64	8.33E-31
Novel:NC_037567.1_45572	7.80	1.57E-29
Novel:chi-miR-202-5p	8.09	2.62E-29
bta-miR-328	2.42	7.56E-29
bta-miR-451	-4.12	6.73E-28
Novel:NC_037568.1_46154	8.46	1.02E-27
bta-miR-9-3p	8.76	4.89E-27
bta-miR-383	4.35	6.66E-27
Novel:NC_037553.1_23614	7.70	1.59E-26
Novel:NC_037545.1_897	8.21	2.84E-26
Novel:NC_037567.1_45497	8.46	3.59E-26
bta-miR-12006	4.96	8.14E-26
Novel:NC_037550.1_16151	7.54	1.45E-25
Novel:NC_037569.1_47739	8.28	2.39E-25
bta-miR-2483-3p	3.22	2.68E-25
Novel:NC_037560.1_35672	7.57	2.92E-25
Novel:NC_037550.1_18316	7.77	6.58E-25
bta-miR-193b	-3.25	7.04E-25
bta-miR-200c	7.07	3.31E-24
bta-miR-7857-3p	3.93	3.35E-24
Novel:NC_037552.1_23013	10.97	5.69E-24
Novel:NC_037564.1_42469	7.54	1.97E-23
bta-miR-21-3p	-3.56	2.64E-23
bta-miR-423-3p	1.99	3.05E-23
Novel:NC_037547.1_8284	8.01	5.72E-23
bta-miR-195	-2.61	1.06E-22
Novel:NC_037555.1_25446	8.30	2.69E-22
bta-miR-148b	-2.42	2.91E-22
Novel:NC_037565.1_43880	7.35	4.37E-22
Novel:NC_037563.1_40828	7.67	2.83E-21
bta-miR-19b	-2.00	5.30E-21
bta-miR-574	-3.31	7.43E-21
bta-miR-190b	-3.62	7.83E-21
Novel:NC_037550.1_18749	7.27	8.52E-21
bta-miR-141	6.98	8.79E-21
bta-miR-99b	-2.58	1.13E-20
Novel:NC_037555.1_25386	7.58	3.29E-20
Novel:chi-miR-130a-3p	2.62	3.34E-20
Novel:NC_037562.1_40324	7.27	3.38E-20
bta-miR-96	6.34	8.75E-20
bta-miR-99a-5p	-2.22	2.64E-19
bta-miR-146a	3.44	1.23E-18
bta-miR-92b	1.81	2.14E-18
bta-miR-374b	-2.48	2.81E-18
bta-miR-146b	2.53	3.82E-18
bta-miR-335	1.91	4.05E-18

Novel:NC_037561.1_36993	7.88	5.65E-18
Novel:NC_037555.1_26084	7.25	6.17E-18
Novel:NC_037550.1_18739	7.86	9.02E-18
bta-miR-19a	-2.09	9.11E-18
Novel:NC_037569.1_46616	7.77	1.15E-17
bta-miR-20a	-1.94	2.22E-17
bta-let-7i	-1.62	2.95E-17
bta-miR-103	-1.65	3.70E-17
bta-miR-30e-5p	-1.44	4.03E-17
bta-miR-26b	-1.52	7.08E-17
bta-miR-374a	-2.90	8.04E-17
bta-miR-202	-2.85	9.87E-17
Novel:NC_037562.1_40173	7.51	1.27E-16
bta-miR-6123	2.04	1.67E-16
bta-let-7g	-1.69	1.87E-16
bta-miR-24-3p	-2.74	2.23E-16
bta-let-7a-5p	-1.43	2.40E-16
Novel:NC_037546.1_6452	7.44	2.92E-16
bta-miR-652	-2.43	5.18E-16
Novel:NC_037566.1_44593	6.88	6.26E-16
bta-miR-194	1.68	6.66E-16
bta-miR-133a	4.10	1.44E-15
Novel:NC_037545.1_1119	6.86	1.81E-15
bta-miR-197	-2.72	2.37E-15
bta-miR-122	7.70	2.42E-15
Novel:NC_037569.1_48099	6.23	6.45E-15
Novel:NC_037547.1_8508	6.80	9.76E-15
Novel:NC_037547.1_8269	7.20	1.06E-14
Novel:oar-miR-3956-3p	6.83	1.84E-14
Novel:NC_037558.1_32918	7.21	1.92E-14
bta-miR-140	-2.06	2.18E-14
Novel:NC_037555.1_27066	8.31	2.18E-14
bta-miR-187	5.85	2.48E-14
Novel:NC_037563.1_40505	7.02	6.68E-14
bta-miR-1271	-2.95	1.12E-13
bta-miR-499	2.32	1.48E-13
Novel:NC_037563.1_41120	6.95	1.54E-13
Novel:NC_037566.1_44408	6.93	2.09E-13
bta-miR-153	-2.46	2.32E-13
Novel:NC_037546.1_2390	6.96	3.64E-13
bta-miR-128	1.63	4.07E-13
Novel:oar-miR-1197-5p	6.74	4.25E-13
bta-miR-677	-2.31	5.93E-13
bta-miR-106a	2.39	1.06E-12
bta-miR-486	-2.63	1.63E-12
Novel:NC_037569.1_47342	6.67	1.64E-12
bta-miR-2285k	-4.17	1.81E-12

Novel:NC_037566.1_44712	6.59	1.99E-12
Novel:NC_037545.1_1537	7.35	2.28E-12
bta-miR-29b	-2.65	2.33E-12
bta-miR-2483-5p	2.06	2.72E-12
bta-miR-30f	-2.38	3.18E-12
Novel:NC_037567.1_45420	2.01	3.99E-12
bta-miR-20b	2.59	4.60E-12
bta-miR-454	-2.27	4.71E-12
Novel:NC_037558.1_32922	-2.58	4.79E-12
Novel:NC_037564.1_42624	6.50	5.23E-12
Novel:NC_037546.1_2418	6.51	6.63E-12
Novel:NC_037550.1_18777	6.82	6.91E-12
Novel:NC_037548.1_11571	6.89	9.24E-12
Novel:NC_037550.1_16312	6.53	9.97E-12
bta-miR-6119-5p	-1.38	1.19E-11
bta-miR-221	-2.47	2.91E-11
bta-miR-218	3.20	3.02E-11
bta-miR-22-3p	1.55	3.61E-11
bta-miR-2478	2.55	4.67E-11
Novel:NC_037549.1_14734	6.86	4.78E-11
Novel:NC_037563.1_40790	6.78	5.14E-11
bta-miR-769	-1.59	5.54E-11
bta-miR-148a	0.81	8.63E-11
Novel:NC_037552.1_22884	6.54	9.27E-11
bta-miR-378	-2.25	9.27E-11
Novel:chi-miR-543-5p	7.16	9.57E-11
bta-miR-25	-1.33	9.76E-11
Novel:NC_037550.1_18621	6.85	1.15E-10
bta-miR-130a	-1.98	1.76E-10
Novel:NC_037561.1_37274	-3.45	2.79E-10
bta-miR-145	-2.38	4.32E-10
bta-miR-10a	-2.20	8.65E-10
Novel:NC_037555.1_27401	7.37	1.18E-09
bta-miR-106b	-1.84	1.19E-09
bta-miR-2285bl	-2.08	1.56E-09
bta-miR-126-3p	-3.25	1.67E-09
bta-miR-1388-5p	-2.67	1.84E-09
bta-miR-497	-1.61	2.03E-09
Novel:NC_037569.1_48401	7.14	2.71E-09
bta-miR-6119-3p	-3.50	2.96E-09
bta-miR-184	4.06	3.10E-09
Novel:NC_037552.1_22658	5.92	4.25E-09
bta-miR-30a-5p	-0.79	4.98E-09
Novel:NC_037547.1_9249	6.45	8.30E-09
bta-miR-345-3p	-2.14	1.25E-08
Novel:NC_037552.1_22451	6.63	1.29E-08
bta-miR-2284y	-2.46	1.90E-08

bta-miR-186	-1.18	2.02E-08
bta-miR-532	-1.24	4.68E-08
bta-miR-2285bn	-2.01	4.75E-08
bta-miR-545-5p	-3.33	6.05E-08
bta-miR-449a	1.57	9.28E-08
Novel:chi-miR-188-3p	6.65	1.28E-07
bta-miR-2484	1.76	1.84E-07
bta-miR-101	-1.10	1.92E-07
bta-miR-18a	-1.92	2.30E-07
bta-miR-1296	-1.59	2.43E-07
Novel:NC_037550.1_18643	2.74	2.71E-07
bta-miR-32	-2.23	2.83E-07
bta-miR-22-5p	-7.38	3.18E-07
Novel:NC_037553.1_24305	7.05	3.34E-07
bta-miR-15b	-1.36	3.45E-07
bta-miR-545-3p	-7.26	4.87E-07
bta-miR-551b	-2.38	7.35E-07
bta-miR-1246	1.78	7.41E-07
bta-miR-26a	0.51	1.26E-06
bta-miR-708	-2.90	1.47E-06
bta-miR-324	-2.71	1.58E-06
bta-miR-17-5p	-0.89	1.69E-06
bta-miR-450b	-1.65	2.02E-06
Novel:NC_037569.1_48011	-3.38	2.02E-06
bta-miR-193a-3p	-1.40	2.37E-06
bta-miR-1224	2.60	2.45E-06
bta-miR-30b-5p	0.95	4.60E-06
bta-miR-2285cj	-1.60	4.67E-06
bta-miR-100	-1.42	5.37E-06
bta-miR-191	-0.67	5.50E-06
bta-miR-2318	-2.30	7.36E-06
bta-miR-23a	-1.52	1.07E-05
bta-miR-151-3p	-0.85	1.20E-05
bta-miR-502a	2.83	1.59E-05
bta-miR-18b	2.97	1.96E-05
bta-let-7a-3p	-1.09	2.57E-05
Novel:NC_037565.1_43614	-3.10	4.66E-05
bta-miR-1298	4.87	4.72E-05
bta-miR-365-3p	-1.27	5.49E-05
bta-miR-142-3p	-2.80	5.95E-05
bta-miR-126-5p	-1.79	6.01E-05
bta-miR-378b	-3.08	7.73E-05
bta-miR-216a	4.48	8.43E-05
bta-miR-361	-0.94	8.56E-05
bta-miR-455-5p	-1.73	9.59E-05
bta-miR-34c	3.17	9.75E-05
bta-let-7b	-0.96	1.21E-04

bta-miR-424-3p	1.97	1.64E-04
bta-miR-331-3p	-1.13	1.69E-04
bta-miR-2435	0.78	1.77E-04
bta-miR-31	-0.82	1.90E-04
bta-miR-15a	-0.90	2.12E-04
bta-miR-10b	0.68	2.33E-04
bta-miR-29d-5p	-6.32	4.39E-04
bta-miR-3613a	-2.44	4.55E-04
bta-miR-378c	-3.41	4.62E-04
bta-miR-491	-1.88	4.82E-04
bta-miR-2332	-6.36	5.55E-04
bta-miR-135a	-2.01	6.45E-04
bta-miR-2285f	-1.68	7.09E-04
bta-miR-2285b	-6.13	7.81E-04
Novel:NC_037567.1_45279	-1.65	8.49E-04
Novel:NC_037561.1_37749	-2.28	9.87E-04
bta-miR-27a-5p	-2.64	1.01E-03
bta-miR-29c	-1.03	1.01E-03
Novel:NC_037551.1_21705	-1.52	1.32E-03
bta-miR-7	-1.54	1.47E-03
bta-miR-127	-1.25	1.63E-03
bta-miR-28	-0.84	1.68E-03
bta-miR-30c	0.67	1.89E-03
bta-miR-542-5p	-2.31	1.90E-03
bta-miR-215	-1.16	1.96E-03
Novel:NC_037547.1_9210	2.03	2.02E-03
bta-miR-188	-1.16	2.32E-03
bta-miR-424-5p	-1.24	2.42E-03
Novel:NC_037568.1_46052	0.80	2.49E-03
bta-miR-34b	2.63	2.55E-03
bta-miR-99a-3p	-0.93	2.59E-03
bta-miR-2411-5p	2.59	2.59E-03
bta-miR-204	0.73	2.99E-03
Novel:NC_037546.1_4570	-1.34	3.23E-03
bta-miR-6518	1.22	3.28E-03
bta-miR-2284ab	-1.42	3.33E-03
bta-miR-192	0.61	3.36E-03
bta-miR-671	-1.76	3.59E-03
Novel:NC_037564.1_42815	-2.99	3.87E-03
bta-miR-2285aw	-5.64	3.96E-03
bta-miR-1306	-1.75	4.31E-03
bta-miR-425-3p	-1.18	4.84E-03
bta-miR-411c-3p	-6.04	5.41E-03
bta-miR-2440	-1.32	5.48E-03
bta-miR-2285ce	-1.29	6.10E-03
bta-miR-107	-0.72	6.17E-03
bta-miR-2284a	-5.48	6.27E-03

bta-miR-185	-2.92	6.37E-03
bta-miR-2285e	-1.16	6.40E-03
bta-miR-11986c	-5.55	6.44E-03
Novel:NC_037545.1_493	-5.47	7.53E-03
Novel:NC_037564.1_42483	1.31	7.53E-03
bta-miR-27b	0.42	7.54E-03
Novel:NC_037547.1_10467	-1.96	8.08E-03
Novel:NC_037556.1_28335	-1.96	8.10E-03
bta-miR-2285q	-1.41	9.46E-03
bta-miR-16b	0.39	9.46E-03
bta-miR-450a	-1.10	1.08E-02
bta-miR-2299-3p	3.50	1.11E-02
bta-miR-2419-5p	2.93	1.23E-02
bta-miR-2285br	-5.28	1.24E-02
bta-miR-30b-3p	-5.28	1.28E-02
Novel:NC_037546.1_4836	-5.24	1.40E-02
bta-miR-142-5p	-1.63	1.41E-02
bta-miR-16a	-0.51	1.46E-02
Novel:NC_037547.1_9221	-1.02	1.50E-02
bta-miR-2468	-5.24	1.51E-02
Novel:NC_037553.1_24530	2.31	1.54E-02
bta-miR-1291	1.98	1.55E-02
bta-miR-2887	2.28	1.55E-02
Novel:NC_037547.1_7888	2.02	1.55E-02
bta-miR-345-5p	-5.23	1.55E-02
bta-miR-874	-0.96	1.59E-02
bta-miR-2299-5p	-1.79	1.73E-02
bta-miR-130b	2.81	1.74E-02
Novel:NC_037550.1_17661	-5.17	1.75E-02
Novel:NC_037547.1_8177	-5.12	1.77E-02
bta-miR-504	-1.04	1.80E-02
Novel:NC_037553.1_24464	-2.06	1.81E-02
bta-miR-27a-3p	0.58	1.85E-02
Novel:NC_037569.1_48575	1.49	1.99E-02
bta-miR-199a-5p	-0.96	2.17E-02
bta-miR-1307	-0.58	2.22E-02
bta-miR-2285dh	-1.19	2.24E-02
Novel:NC_037556.1_29271	-2.51	2.58E-02
Novel:NC_037564.1_42998	-0.61	2.88E-02
Novel:NC_037557.1_30140	-0.61	2.88E-02
Novel:NC_037569.1_47305	-0.61	2.88E-02
bta-miR-2285aa	-4.95	2.88E-02
bta-miR-181a	-0.46	2.90E-02
bta-miR-2331-3p	-4.97	2.93E-02
Novel:NC_037551.1_21229	-0.73	2.94E-02
bta-miR-2285cm	-0.98	2.98E-02
bta-miR-2416	-4.99	3.07E-02

bta-miR-199c	-4.95	3.24E-02
Novel:NC_037557.1_29957	-1.60	3.29E-02
bta-miR-449b	-1.21	3.29E-02
bta-miR-2370-3p	-4.99	3.31E-02
bta-miR-500	-0.89	3.61E-02
bta-miR-210	-0.61	3.64E-02
bta-miR-331-5p	-0.95	3.68E-02
bta-miR-190a	-1.10	4.00E-02
bta-miR-154c	1.37	4.11E-02
bta-miR-2904	2.32	4.17E-02
bta-miR-455-3p	-1.36	4.17E-02
Novel:NC_037567.1_45577	-2.23	4.24E-02
Novel:NC_037548.1_10786	-1.08	4.79E-02
Novel:NC_037546.1_7398	1.36	4.93E-02
Novel:NC_037547.1_9181	-4.67	4.93E-02
Novel:NC_037561.1_36952	-1.53	5.11E-02
bta-miR-2285bw	-4.62	5.16E-02
bta-miR-495	-4.68	5.18E-02
bta-miR-194b	-4.63	5.31E-02
bta-miR-194b-3p	-4.63	5.31E-02
bta-miR-2285ai-5p	2.73	5.35E-02
bta-miR-2446	-4.63	5.37E-02
bta-miR-150	1.24	5.37E-02
bta-miR-34a	-1.00	5.49E-02
bta-miR-2284c	-4.57	5.71E-02
Novel:NC_037556.1_29441	1.11	5.98E-02
bta-miR-125b	0.47	5.99E-02
Novel:NC_037554.1_24708	2.22	6.20E-02
bta-miR-11992	-4.57	6.32E-02
bta-miR-503-5p	1.44	6.89E-02
bta-miR-147	-1.73	6.89E-02
bta-miR-365-5p	-4.44	6.89E-02
bta-miR-340	0.81	7.03E-02
bta-miR-2387	-4.53	7.20E-02
bta-miR-411c-5p	-4.58	7.35E-02
Novel:NC_037553.1_24430	-4.46	7.40E-02
Novel:chi-miR-3432-3p	1.65	7.55E-02
bta-miR-143	-0.82	7.67E-02
bta-miR-760-3p	0.66	7.67E-02
Novel:NC_037548.1_11804	2.03	7.91E-02
bta-miR-2285bu	-4.34	8.04E-02
bta-miR-1434-5p	-4.40	8.24E-02
Novel:NC_037557.1_30322	-4.49	8.35E-02
Novel:NC_037547.1_7592	-4.39	8.42E-02
bta-miR-429	-4.34	8.46E-02
Novel:NC_037547.1_8617	-0.64	8.59E-02
bta-miR-3431	-1.00	8.61E-02

bta-miR-2285ad	-4.29	8.61E-02
bta-miR-494	-2.18	8.74E-02
bta-miR-1343-3p	0.49	9.10E-02
bta-miR-485	2.35	9.13E-02
bta-miR-2284p	2.03	9.30E-02
Novel:NC_037547.1_7809	-4.29	9.38E-02
Novel:chi-miR-2284d	-4.18	9.75E-02
bta-miR-502b	-0.63	9.92E-02
bta-miR-2284aa	1.95	1.01E-01
bta-miR-330	-4.19	1.01E-01
bta-miR-224	0.93	1.01E-01
bta-miR-2285o	-4.14	1.02E-01
Novel:NC_037563.1_41002	-4.09	1.12E-01
bta-miR-24	-1.24	1.17E-01
bta-miR-2285dl-3p	-4.04	1.18E-01
bta-miR-374c	-1.93	1.18E-01
bta-let-7c	-0.38	1.19E-01
bta-miR-2285au	-4.89	1.21E-01
Novel:NC_037553.1_23674	-1.03	1.22E-01
Novel:NC_037545.1_9	-4.07	1.26E-01
Novel:NC_037558.1_32904	-4.04	1.28E-01
Novel:NC_037568.1_45736	-4.03	1.31E-01
Novel:NC_037558.1_31773	1.04	1.33E-01
Novel:NC_037555.1_26130	-3.97	1.33E-01
bta-miR-1468	0.69	1.34E-01
bta-miR-362-5p	-0.74	1.34E-01
Novel:NC_037547.1_9085	-3.95	1.36E-01
bta-miR-380-3p	2.12	1.40E-01
Novel:NC_037548.1_12085	-3.88	1.44E-01
Novel:NC_037565.1_43873	-3.87	1.46E-01
Novel:NC_037553.1_24503	-3.84	1.49E-01
bta-miR-219	-3.84	1.51E-01
bta-miR-17-3p	-0.42	1.54E-01
bta-miR-12017	-3.85	1.56E-01
Novel:NC_037562.1_40274	-3.83	1.57E-01
Novel:NC_037548.1_11132	-3.79	1.57E-01
Novel:NC_037557.1_30374	-3.85	1.58E-01
Novel:NC_037562.1_40225	-0.84	1.64E-01
Novel:NC_037548.1_10972	-3.75	1.70E-01
bta-miR-199b	-0.51	1.70E-01
Novel:NC_037551.1_21750	-3.74	1.71E-01
Novel:chi-miR-184	-3.95	1.71E-01
bta-miR-7859	1.99	1.74E-01
bta-miR-152	-0.49	1.75E-01
Novel:NC_037548.1_11027	1.83	1.75E-01
bta-miR-149-5p	-0.40	1.78E-01
bta-miR-505	-0.52	1.80E-01

Novel:NC_037561.1_37393	-0.78	1.80E-01
bta-miR-29a	-0.35	1.85E-01
bta-miR-144	-3.66	1.88E-01
Novel:NC_037564.1_42846	-3.61	1.89E-01
bta-miR-211	-3.67	1.89E-01
bta-miR-660	-0.28	1.92E-01
Novel:NC_037550.1_17518	-3.62	1.94E-01
bta-miR-155	-0.40	1.94E-01
bta-miR-2403	-3.58	1.94E-01
bta-miR-326	-3.59	1.94E-01
Novel:chi-miR-1388-3p	-0.63	1.99E-01
bta-miR-543	1.73	1.99E-01
bta-miR-2285ar	-3.59	2.00E-01
Novel:NC_037567.1_45438	-3.56	2.02E-01
bta-miR-2312	-3.51	2.02E-01
bta-miR-212	-1.14	2.04E-01
Novel:NC_037553.1_23348	-3.57	2.04E-01
Novel:NC_037568.1_45743	-0.66	2.09E-01
bta-miR-541	-3.52	2.09E-01
Novel:NC_037552.1_22200	-3.57	2.09E-01
bta-miR-6121-3p	-3.50	2.11E-01
Novel:NC_037568.1_46078	-3.51	2.11E-01
bta-miR-2285bf	-3.57	2.11E-01
bta-miR-339a	0.38	2.12E-01
Novel:NC_037561.1_36933	-3.49	2.15E-01
bta-let-7e	-0.34	2.17E-01
Novel:NC_037561.1_37297	1.71	2.19E-01
bta-miR-2284f	-3.40	2.24E-01
Novel:NC_037546.1_6902	-3.40	2.30E-01
Novel:NC_037567.1_45690	-0.49	2.30E-01
bta-miR-33b	-3.36	2.31E-01
Novel:NC_037548.1_11807	1.29	2.36E-01
bta-let-7f	-0.24	2.36E-01
bta-miR-1343-5p	-3.34	2.36E-01
bta-miR-7858	-3.33	2.37E-01
bta-miR-6529a	0.67	2.39E-01
bta-miR-136	-3.37	2.40E-01
bta-miR-483	-0.78	2.45E-01
bta-miR-2285s	0.82	2.52E-01
bta-miR-11985	-3.34	2.52E-01
Novel:NC_037559.1_33436	-3.28	2.53E-01
bta-miR-10182-5p	1.49	2.54E-01
bta-miR-2475	-3.25	2.54E-01
bta-miR-12030	-3.23	2.55E-01
bta-miR-2367-5p	-3.20	2.59E-01
bta-miR-382	-3.18	2.67E-01
Novel:NC_037567.1_45399	-3.16	2.67E-01

Novel:NC_037556.1_28871	-3.16	2.67E-01
bta-miR-11972	-3.18	2.67E-01
bta-miR-2285bz	-0.41	2.71E-01
Novel:NC_037545.1_973	-1.45	2.71E-01
bta-miR-296-5p	1.08	2.71E-01
bta-miR-219b-3p	-3.17	2.72E-01
bta-miR-2477	-3.13	2.74E-01
bta-miR-342	-0.26	2.76E-01
bta-miR-6517	0.43	2.79E-01
Novel:NC_037560.1_36165	-3.10	2.81E-01
Novel:NC_037548.1_10849	-1.44	2.83E-01
Novel:NC_037558.1_31721	-3.08	2.84E-01
bta-miR-2355-3p	-3.08	2.84E-01
bta-miR-487b	1.15	2.84E-01
bta-miR-2284o	-3.02	2.92E-01
Novel:NC_037560.1_36156	-3.02	2.92E-01
bta-miR-10164-3p	-0.66	2.96E-01
bta-miR-2285dd	-3.09	2.96E-01
Novel:NC_037549.1_13802	-1.39	2.96E-01
Novel:NC_037548.1_11519	-3.02	2.96E-01
bta-miR-2284v	-3.02	3.01E-01
Novel:NC_037546.1_4545	-3.15	3.08E-01
bta-miR-12034	1.09	3.11E-01
bta-miR-1388-3p	-0.53	3.12E-01
bta-miR-2284k	-1.35	3.12E-01
bta-miR-11987	-2.99	3.12E-01
bta-miR-181b	-0.24	3.13E-01
Novel:NC_037548.1_11872	-2.95	3.13E-01
bta-miR-2285g	-2.99	3.15E-01
bta-miR-151-5p	-0.23	3.15E-01
bta-miR-200b	-1.42	3.15E-01
bta-miR-582	-0.88	3.20E-01
bta-miR-2427	-2.87	3.21E-01
bta-miR-196a	1.40	3.25E-01
bta-miR-2285bo	-3.00	3.25E-01
Novel:NC_037554.1_25238	-2.82	3.29E-01
bta-miR-2397-3p	-2.80	3.36E-01
Novel:NC_037565.1_43588	-2.78	3.36E-01
Novel:NC_037553.1_24501	-2.77	3.38E-01
bta-miR-1277	-2.76	3.39E-01
bta-miR-296-3p	0.31	3.39E-01
Novel:NC_037568.1_45778	1.33	3.41E-01
bta-miR-2285bp	-2.74	3.45E-01
Novel:NC_037553.1_23805	-2.74	3.51E-01
bta-miR-2285aj-5p	-0.30	3.51E-01
bta-miR-378d	-2.69	3.52E-01
bta-miR-132	-0.35	3.52E-01

bta-miR-320a	-0.22	3.52E-01
bta-miR-301a	-0.18	3.56E-01
Novel:NC_037552.1_22118	-2.67	3.60E-01
bta-miR-11986b	-0.50	3.71E-01
bta-miR-6522	-2.59	3.71E-01
bta-miR-11991	-2.60	3.71E-01
Novel:NC_037545.1_808	-2.58	3.72E-01
bta-miR-2331-5p	-2.58	3.72E-01
Novel:NC_037556.1_28823	1.17	3.85E-01
Novel:NC_037557.1_29612	-2.55	3.88E-01
bta-miR-665	0.68	3.89E-01
bta-miR-124a	-2.60	3.91E-01
bta-miR-12041	-2.49	3.92E-01
Novel:chi-miR-378-3p	-2.52	3.94E-01
bta-miR-7180	0.75	3.94E-01
Novel:NC_037565.1_43904	1.50	3.97E-01
bta-miR-2285cw	-2.48	4.04E-01
bta-miR-592	0.90	4.22E-01
bta-miR-6536	-2.47	4.23E-01
bta-miR-129-3p	-1.09	4.23E-01
Novel:NC_037546.1_6434	1.02	4.27E-01
bta-miR-2461-3p	-2.33	4.30E-01
bta-miR-2382-3p	-2.37	4.31E-01
bta-miR-2284w	-2.25	4.41E-01
bta-miR-196b	1.32	4.48E-01
bta-miR-654	1.27	4.51E-01
bta-miR-208b	0.52	4.61E-01
bta-miR-2285da	0.78	4.62E-01
Novel:NC_037560.1_36199	0.65	4.65E-01
Novel:chi-miR-2284a	-0.40	4.70E-01
bta-miR-4286	-2.15	4.70E-01
Novel:NC_037556.1_28884	-2.16	4.70E-01
bta-miR-301b	-0.27	4.72E-01
bta-miR-339b	0.39	4.72E-01
Novel:NC_037568.1_46470	-0.63	4.74E-01
bta-miR-873	-1.01	4.83E-01
bta-miR-33a	0.71	4.86E-01
bta-miR-432	-0.58	5.20E-01
bta-miR-421	-0.19	5.20E-01
Novel:NC_037564.1_42315	1.05	5.28E-01
Novel:NC_037545.1_938	0.46	5.43E-01
bta-miR-484	-0.17	5.51E-01
bta-miR-181d	0.20	5.53E-01
bta-miR-381	-0.32	5.67E-01
bta-miR-449c	0.30	5.67E-01
bta-miR-2311	-1.64	5.73E-01
bta-miR-2424	0.34	5.84E-01

bta-miR-222	-0.22	5.85E-01
bta-miR-139	-0.63	5.95E-01
bta-miR-137	-0.78	6.08E-01
bta-miR-6524	0.60	6.29E-01
Novel:NC_037550.1_16100	-0.74	6.31E-01
bta-miR-433	0.83	6.41E-01
Novel:NC_037566.1_44459	0.79	6.42E-01
bta-miR-3432a	0.74	6.60E-01
bta-miR-503-3p	-0.37	6.68E-01
Novel:NC_037565.1_43585	0.74	6.72E-01
bta-miR-1249	-0.29	6.80E-01
bta-miR-4449	-0.26	6.82E-01
bta-miR-362-3p	0.29	6.84E-01
bta-miR-628	-0.20	6.87E-01
bta-miR-199a-3p	0.21	6.87E-01
bta-miR-125a	-0.10	6.90E-01
Novel:NC_037560.1_36384	-0.61	6.91E-01
bta-miR-1839	0.10	6.94E-01
bta-miR-379	0.69	7.00E-01
Novel:NC_037560.1_35793	0.64	7.00E-01
bta-miR-744	0.12	7.05E-01
bta-miR-493	-0.42	7.12E-01
Novel:NC_037549.1_14019	-0.53	7.24E-01
bta-miR-655	0.60	7.31E-01
bta-miR-219-5p	0.56	7.36E-01
Novel:NC_037558.1_31832	-0.38	7.41E-01
bta-let-7d	-0.10	7.47E-01
Novel:NC_037558.1_30589	-0.47	7.56E-01
bta-miR-223	0.28	7.58E-01
Novel:NC_037564.1_42641	-0.35	7.61E-01
Novel:chi-miR-3432-5p	0.12	7.69E-01
bta-miR-2284x	0.06	7.76E-01
Novel:NC_037565.1_43872	0.45	7.80E-01
bta-miR-2411-3p	-0.21	7.96E-01
bta-miR-2285ag-3p	0.31	7.96E-01
bta-miR-410	-0.40	7.99E-01
bta-miR-10182-3p	0.43	7.99E-01
Novel:NC_037559.1_33382	0.41	8.04E-01
bta-miR-411a	-0.13	8.13E-01
bta-miR-2285p	0.20	8.27E-01
Novel:NC_037553.1_24324	0.21	8.36E-01
Novel:NC_037549.1_14821	0.25	8.38E-01
bta-miR-2285ab	0.33	8.39E-01
Novel:NC_037551.1_19028	-0.29	8.48E-01
bta-miR-6520	0.15	8.51E-01
bta-miR-2284b	-0.20	8.66E-01
bta-miR-181c	0.05	8.80E-01

Novel:NC_037556.1_29588	-0.15	8.84E-01
bta-miR-664b	0.17	8.94E-01
bta-miR-191b	-0.21	8.94E-01
bta-miR-452	0.23	8.94E-01
bta-miR-2285c	0.15	9.03E-01
bta-miR-200a	0.07	9.16E-01
bta-miR-208a	-0.12	9.16E-01
bta-miR-2285co	0.08	9.20E-01
bta-miR-425-5p	0.02	9.31E-01
bta-miR-488	-0.12	9.37E-01
bta-miR-1260b	0.04	9.48E-01
bta-miR-95	0.06	9.59E-01
bta-miR-214	0.03	9.63E-01
Novel:NC_037551.1_21257	-0.04	9.72E-01
bta-miR-2284z	0.03	9.73E-01
Novel:NC_037557.1_30145	-0.04	9.83E-01
bta-miR-193a-5p	-0.01	9.95E-01
Novel:NC_037546.1_5728	-0.01	1.00E+00

Supplementary file S4. List of differentially expressed miRNAs (DE-miRNAs) between oocytes (OOs) and follicular cells (FCs). For each DE-miRNA. the Log Fold Change (LogFC) and the False Discovery Rate (FDR) were reported.

OO-NBS_vs_OO-BS				FC-NBS_vs_FC-BS			
	logFC	PValue	FDR		logFC	PValue	FDR
bta-miR-143	-2.07	1.2E-07	5.4E-05	Novel:NC_037567.1_45577	2.65	3.1E-08	1.7E-05
Novel:NC_037550.1_18643	1.58	3.4E-06	7.2E-04	Novel:NC_037553.1_23674	2.09	1.4E-07	3.8E-05
bta-miR-199a-3p	-2.10	4.7E-06	7.2E-04	Novel:chi-miR-184	-4.24	8.5E-07	1.5E-04
bta-miR-1468	-1.83	3.2E-05	3.7E-03	bta-miR-2904	-2.90	1.9E-06	2.5E-04
bta-miR-25	-0.91	1.5E-04	1.4E-02	Novel:NC_037550.1_18643	-2.37	3.9E-06	4.2E-04
bta-miR-1388-5p	-4.55	4.6E-04	3.5E-02	bta-miR-2411-3p	-2.01	1.7E-05	1.5E-03
bta-miR-296-3p	-1.41	7.4E-04	3.9E-02	bta-miR-2440	-1.86	3.9E-05	3.0E-03
Novel:NC_037557.1_30140	-1.36	8.2E-04	3.9E-02	bta-miR-2332	-1.47	7.3E-05	4.5E-03
Novel:NC_037569.1_47305	-1.36	8.3E-04	3.9E-02	bta-miR-141	-3.73	7.5E-05	4.5E-03
Novel:NC_037564.1_42998	-1.36	8.4E-04	3.9E-02	bta-miR-2478	1.68	9.2E-05	5.0E-03
bta-miR-331-5p	-4.19	9.7E-04	4.1E-02	bta-miR-34b	-4.11	1.7E-04	8.2E-03
bta-miR-199a-5p	-2.09	1.1E-03	4.5E-02	bta-miR-34c	-4.02	1.8E-04	8.2E-03
bta-miR-222	-1.35	1.4E-03	5.0E-02	bta-miR-486	0.96	8.8E-04	3.6E-02
bta-miR-153	-2.14	1.7E-03	5.4E-02	bta-miR-200c	-2.82	1.8E-03	7.1E-02
bta-miR-1246	-1.07	1.7E-03	5.4E-02	bta-miR-6536	-4.13	2.1E-03	7.4E-02
Novel:NC_037552.1_23013	2.29	1.9E-03	5.5E-02	Novel:NC_037557.1_30322	2.11	2.3E-03	7.6E-02
Novel:NC_037548.1_11587	0.69	2.2E-03	5.9E-02	Novel:NC_037546.1_4545	-3.53	3.0E-03	9.7E-02
Novel:chi-miR-345-5p	0.93	2.4E-03	5.9E-02	bta-miR-375	-2.13	4.2E-03	1.2E-01
Novel:NC_037557.1_29957	4.00	2.4E-03	5.9E-02	bta-miR-11985	-2.38	4.7E-03	1.3E-01

bta-miR-130a	-1.21	2.7E-03	5.9E-02	bta-miR-200a	-1.26	5.2E-03	1.4E-01
bta-miR-423-5p	-0.76	2.8E-03	5.9E-02	bta-miR-1434-5p	1.50	5.7E-03	1.5E-01
Novel:NC_037545.1_835	0.99	2.9E-03	5.9E-02	bta-miR-452	-2.02	9.9E-03	2.4E-01
bta-miR-186	-0.88	3.0E-03	5.9E-02	Novel:chi-miR-378-3p	-2.55	1.1E-02	2.5E-01
Novel:NC_037559.1_33577	0.84	3.1E-03	5.9E-02	bta-miR-4449	-1.10	1.1E-02	2.5E-01
Novel:NC_037550.1_16194	1.23	3.1E-03	5.9E-02	Novel:NC_037568.1_45736	-1.54	1.2E-02	2.6E-01
Novel:NC_037547.1_9642	0.95	3.9E-03	6.9E-02	bta-miR-1224	1.33	1.3E-02	2.6E-01
bta-miR-140	-1.03	4.2E-03	7.1E-02	bta-miR-222	0.93	1.4E-02	2.8E-01
bta-miR-146b	0.78	4.3E-03	7.1E-02	bta-miR-2284f	-1.53	1.5E-02	2.8E-01
bta-miR-29c	-2.05	4.6E-03	7.4E-02	bta-miR-2370-3p	1.37	1.6E-02	2.9E-01
bta-miR-450b	-1.19	4.9E-03	7.4E-02	Novel:NC_037552.1_22845	0.95	1.6E-02	2.9E-01
bta-miR-1388-3p	-4.01	5.0E-03	7.4E-02	bta-miR-449c	1.09	2.2E-02	3.8E-01
Novel:NC_037569.1_48401	3.44	5.1E-03	7.4E-02	bta-miR-132	0.73	2.6E-02	4.3E-01
bta-miR-29a	-0.76	5.3E-03	7.5E-02	Novel:chi-miR-1388-3p	0.99	2.7E-02	4.3E-01
bta-miR-31	-0.83	5.4E-03	7.5E-02	Novel:NC_037553.1_24430	1.20	2.7E-02	4.3E-01
bta-miR-454	2.10	6.0E-03	8.1E-02	bta-miR-2285bf	-1.64	2.8E-02	4.3E-01
bta-miR-127	-1.19	6.5E-03	8.4E-02	bta-miR-92a	0.45	2.9E-02	4.3E-01
bta-miR-34a	-4.13	6.6E-03	8.4E-02	Novel:NC_037569.1_48575	1.00	2.9E-02	4.3E-01
bta-miR-362-5p	2.12	6.9E-03	8.5E-02	bta-miR-12017	-1.36	3.4E-02	4.7E-01
bta-miR-1296	-2.11	8.7E-03	1.0E-01	bta-miR-2284v	1.79	3.4E-02	4.7E-01
bta-miR-133a	-1.39	9.4E-03	1.1E-01	bta-miR-92b	0.53	3.5E-02	4.7E-01
Novel:NC_037567.1_45420	-0.85	9.5E-03	1.1E-01	Novel:NC_037548.1_10786	0.81	3.6E-02	4.8E-01
bta-miR-665	-3.67	9.5E-03	1.1E-01	bta-miR-451	0.60	3.9E-02	4.9E-01
bta-miR-100	-1.67	1.0E-02	1.1E-01	bta-miR-11986c	-0.84	4.0E-02	4.9E-01
bta-miR-30e-5p	-0.69	1.1E-02	1.1E-01	bta-miR-125a	0.53	4.1E-02	4.9E-01
Novel:NC_037550.1_18175	1.34	1.1E-02	1.2E-01	Novel:NC_037556.1_29588	1.00	4.1E-02	4.9E-01
bta-miR-503-5p	2.57	1.2E-02	1.2E-01	bta-miR-2427	-1.61	4.3E-02	5.0E-01
bta-miR-744	-1.14	1.3E-02	1.3E-01	bta-miR-126-3p	-1.04	4.5E-02	5.0E-01
bta-miR-151-3p	-0.65	1.4E-02	1.3E-01	bta-miR-1468	1.02	4.5E-02	5.0E-01
Novel:NC_037555.1_27099	1.06	1.4E-02	1.3E-01	bta-miR-1247-5p	1.44	4.9E-02	5.3E-01
bta-miR-500	1.76	1.4E-02	1.3E-01	bta-miR-125b	0.55	5.1E-02	5.5E-01
Novel:NC_037550.1_18777	-3.04	1.4E-02	1.3E-01	bta-miR-543	1.62	5.3E-02	5.6E-01
bta-miR-339a	-0.96	1.5E-02	1.3E-01	bta-miR-11992	1.10	5.5E-02	5.7E-01
bta-miR-378	-1.17	1.5E-02	1.3E-01	bta-miR-2887	-1.35	5.6E-02	5.7E-01
bta-miR-532	-0.70	1.6E-02	1.4E-01	bta-miR-2446	-0.94	5.8E-02	5.8E-01
bta-miR-122	2.31	1.6E-02	1.4E-01	bta-miR-29a	0.59	6.3E-02	6.1E-01
bta-miR-216a	3.27	1.7E-02	1.4E-01	Novel:NC_037559.1_33436	1.34	6.4E-02	6.1E-01
bta-miR-484	-1.00	1.9E-02	1.6E-01	bta-miR-449b	0.71	6.4E-02	6.1E-01
bta-miR-23a	-1.92	1.9E-02	1.6E-01	Novel:NC_037548.1_11587	-1.06	6.6E-02	6.2E-01
Novel:NC_037553.1_23674	2.06	2.0E-02	1.6E-01	bta-miR-10182-3p	1.39	7.3E-02	6.6E-01
Novel:NC_037568.1_46052	-1.04	2.0E-02	1.6E-01	Novel:NC_037545.1_1469	0.68	7.4E-02	6.6E-01
bta-miR-345-3p	-2.79	2.1E-02	1.6E-01	Novel:chi-miR-3432-3p	1.24	8.0E-02	7.0E-01
bta-miR-22-3p	-0.51	2.2E-02	1.6E-01	bta-miR-26a	0.32	8.0E-02	7.0E-01
bta-miR-142-5p	-2.29	2.2E-02	1.6E-01	bta-miR-494	-0.91	8.1E-02	7.0E-01
bta-miR-1307	-0.91	2.5E-02	1.8E-01	bta-miR-2285cw	-1.74	8.9E-02	7.5E-01
bta-miR-2284y	-3.38	2.6E-02	1.8E-01	bta-miR-2285q	-0.58	9.0E-02	7.5E-01
Novel:chi-miR-188-3p	2.98	2.7E-02	1.9E-01	Novel:NC_037546.1_5728	1.39	9.5E-02	7.7E-01

bta-miR-423-3p	-0.60	2.7E-02	1.9E-01	bta-miR-411a	-0.80	9.6E-02	7.7E-01
bta-miR-125b	-0.70	2.8E-02	1.9E-01	bta-miR-7180	0.98	1.0E-01	7.9E-01
Novel:NC_037555.1_27066	2.16	2.8E-02	1.9E-01	bta-miR-219	0.91	1.0E-01	7.9E-01
bta-miR-3431	-3.51	2.9E-02	1.9E-01	Novel:NC_037546.1_6902	1.02	1.0E-01	7.9E-01
bta-miR-361	-0.83	2.9E-02	1.9E-01	Novel:NC_037559.1_33577	-1.06	1.0E-01	7.9E-01
bta-let-7b	-0.74	3.0E-02	1.9E-01	bta-miR-12006	1.16	1.1E-01	7.9E-01
bta-miR-2887	-3.21	3.3E-02	2.1E-01	Novel:chi-miR-30a-3p	-0.83	1.1E-01	7.9E-01
bta-miR-320a	-0.62	3.6E-02	2.3E-01	bta-miR-144	1.04	1.1E-01	7.9E-01
Novel:NC_037547.1_7888	-3.17	3.6E-02	2.3E-01	bta-miR-155	0.38	1.1E-01	7.9E-01
bta-miR-592	2.82	3.7E-02	2.3E-01	bta-miR-424-5p	-0.71	1.2E-01	7.9E-01
bta-miR-2484	1.01	3.8E-02	2.3E-01	bta-miR-12034	-0.94	1.2E-01	7.9E-01
bta-miR-660	-0.50	3.8E-02	2.3E-01	bta-miR-2483-3p	0.65	1.2E-01	7.9E-01
bta-let-7c	-0.67	3.9E-02	2.3E-01	bta-miR-2285p	-0.68	1.2E-01	7.9E-01
Novel:NC_037545.1_938	-3.30	4.0E-02	2.3E-01	bta-miR-126-5p	-0.68	1.2E-01	7.9E-01
Novel:NC_037561.1_36993	1.83	4.3E-02	2.5E-01	bta-miR-411c-3p	-1.09	1.2E-01	7.9E-01
Novel:NC_037565.1_43880	1.61	4.4E-02	2.5E-01	bta-miR-24	-0.60	1.2E-01	7.9E-01
Novel:NC_037563.1_40505	2.07	4.5E-02	2.5E-01	bta-miR-129-3p	0.78	1.2E-01	7.9E-01
bta-miR-24-3p	0.91	4.7E-02	2.6E-01	bta-miR-2285dd	1.40	1.2E-01	7.9E-01
Novel:NC_037552.1_22735	0.47	4.7E-02	2.6E-01	bta-miR-6520	0.68	1.3E-01	7.9E-01
Novel:chi-miR-1271-5p	0.93	4.8E-02	2.6E-01	bta-let-7i	0.29	1.3E-01	8.1E-01
Novel:NC_037567.1_45367	-1.23	5.1E-02	2.7E-01	bta-let-7c	0.31	1.3E-01	8.1E-01
bta-miR-324	-2.96	5.2E-02	2.7E-01	bta-miR-21-5p	0.46	1.3E-01	8.1E-01
bta-miR-6123	0.64	5.3E-02	2.7E-01	bta-miR-4286	1.54	1.4E-01	8.1E-01
bta-miR-10a	-0.67	5.3E-02	2.7E-01	bta-miR-339a	0.45	1.4E-01	8.1E-01
bta-miR-421	-0.80	5.4E-02	2.7E-01	Novel:NC_037562.1_40274	0.89	1.4E-01	8.1E-01
Novel:NC_037558.1_32568	1.18	5.7E-02	2.9E-01	bta-miR-767	-0.91	1.4E-01	8.1E-01
bta-miR-184	1.87	5.8E-02	2.9E-01	bta-miR-483	0.61	1.4E-01	8.1E-01
bta-miR-769	-0.68	5.8E-02	2.9E-01	Novel:NC_037567.1_45420	0.61	1.4E-01	8.1E-01
bta-miR-504	-1.83	6.1E-02	2.9E-01	bta-miR-128	0.48	1.4E-01	8.1E-01
Novel:NC_037566.1_44115	-0.86	6.1E-02	2.9E-01	bta-miR-98	0.40	1.5E-01	8.4E-01
Novel:NC_037562.1_40133	0.75	6.1E-02	2.9E-01	bta-miR-383	0.76	1.5E-01	8.4E-01
bta-miR-1247-5p	0.58	6.2E-02	2.9E-01	Novel:NC_037556.1_28884	1.48	1.5E-01	8.4E-01
Novel:chi-miR-421-3p	0.82	6.3E-02	3.0E-01	bta-miR-11987	-1.28	1.5E-01	8.4E-01
bta-let-7i	-0.54	6.5E-02	3.0E-01	bta-miR-301b	-0.45	1.6E-01	8.4E-01
bta-miR-6517	-1.67	7.4E-02	3.4E-01	Novel:NC_037547.1_9181	0.71	1.6E-01	8.4E-01
Novel:NC_037551.1_21229	-1.31	7.6E-02	3.4E-01	bta-miR-574	-0.41	1.6E-01	8.4E-01
bta-miR-145	-1.35	7.6E-02	3.4E-01	bta-miR-223	-1.22	1.6E-01	8.4E-01
bta-miR-652	-0.97	7.8E-02	3.5E-01	bta-miR-760-3p	0.46	1.6E-01	8.5E-01
bta-miR-150	-1.88	8.5E-02	3.7E-01	Novel:NC_037550.1_16100	0.91	1.7E-01	8.5E-01
Novel:NC_037548.1_12574	0.82	8.8E-02	3.7E-01	bta-miR-139	-0.86	1.7E-01	8.5E-01
bta-miR-221	-1.01	8.8E-02	3.7E-01	bta-miR-449a	0.57	1.7E-01	8.5E-01
bta-miR-505	-1.02	8.9E-02	3.7E-01	Novel:NC_037563.1_41002	-0.75	1.7E-01	8.5E-01
bta-miR-2478	0.68	8.9E-02	3.7E-01	bta-miR-503-5p	-0.91	1.7E-01	8.5E-01
bta-miR-202	-0.72	9.1E-02	3.7E-01	bta-miR-27b	0.32	1.7E-01	8.5E-01
Novel:NC_037568.1_45743	1.63	9.1E-02	3.7E-01	bta-miR-10a	-0.56	1.7E-01	8.5E-01
bta-miR-424-5p	0.85	9.1E-02	3.7E-01	bta-miR-181b	0.32	1.8E-01	8.5E-01
Novel:NC_037550.1_16312	-2.11	9.1E-02	3.7E-01	bta-miR-221	0.39	1.8E-01	8.5E-01

bta-miR-200c	0.62	9.1E-02	3.7E-01	bta-miR-664b	-0.89	1.9E-01	8.6E-01
Novel:NC_037547.1_10260	0.84	9.2E-02	3.7E-01	bta-miR-150	0.75	1.9E-01	8.6E-01
Novel:NC_037561.1_36952	-2.49	9.5E-02	3.7E-01	bta-miR-7857-5p	1.10	1.9E-01	8.6E-01
Novel:NC_037561.1_37749	-2.49	9.5E-02	3.7E-01	Novel:NC_037547.1_7888	-0.73	1.9E-01	8.6E-01
bta-miR-19a	-0.55	9.5E-02	3.7E-01	Novel:NC_037561.1_37274	-0.42	2.0E-01	8.6E-01
Novel:NC_037566.1_43986	-0.77	9.5E-02	3.7E-01	Novel:NC_037553.1_24501	1.01	2.0E-01	8.6E-01
bta-miR-1839	-0.61	9.9E-02	3.8E-01	Novel:NC_037562.1_40225	0.41	2.0E-01	8.6E-01
bta-miR-2483-5p	0.53	9.9E-02	3.8E-01	Novel:NC_037548.1_11807	-0.77	2.0E-01	8.6E-01
Novel:NC_037558.1_32432	-0.98	1.0E-01	4.0E-01	Novel:NC_037564.1_42998	0.36	2.0E-01	8.6E-01
Novel:chi-miR-3432-5p	1.09	1.1E-01	4.1E-01	Novel:NC_037569.1_47305	0.36	2.0E-01	8.6E-01
Novel:NC_037551.1_21150	0.83	1.1E-01	4.1E-01	bta-miR-2285cj	-0.37	2.1E-01	8.6E-01
Novel:NC_037550.1_18316	1.18	1.1E-01	4.1E-01	Novel:NC_037557.1_30140	0.36	2.1E-01	8.6E-01
bta-miR-1298	-2.31	1.1E-01	4.1E-01	Novel:NC_037548.1_11519	-0.94	2.1E-01	8.6E-01
Novel:NC_037551.1_21843	1.16	1.1E-01	4.2E-01	bta-miR-499	0.42	2.1E-01	8.6E-01
bta-miR-210	-0.67	1.2E-01	4.2E-01	bta-miR-363	-1.15	2.1E-01	8.6E-01
bta-miR-132	-0.99	1.2E-01	4.3E-01	bta-miR-22-3p	0.50	2.1E-01	8.6E-01
bta-miR-149-5p	-0.87	1.2E-01	4.3E-01	bta-miR-2285ab	0.81	2.1E-01	8.6E-01
bta-miR-19b	-0.38	1.2E-01	4.3E-01	Novel:NC_037568.1_45778	-1.14	2.1E-01	8.6E-01
Novel:NC_037546.1_5892	0.81	1.2E-01	4.3E-01	bta-miR-154c	0.75	2.1E-01	8.6E-01
bta-miR-497	-0.68	1.2E-01	4.3E-01	Novel:NC_037546.1_7398	0.60	2.2E-01	8.6E-01
bta-miR-2285q	-1.98	1.2E-01	4.3E-01	Novel:NC_037551.1_21750	-0.77	2.2E-01	8.6E-01
Novel:NC_037556.1_29003	0.39	1.2E-01	4.3E-01	Novel:NC_037551.1_21257	0.58	2.2E-01	8.6E-01
Novel:NC_037549.1_14793	-0.93	1.3E-01	4.4E-01	Novel:NC_037553.1_23348	-0.84	2.2E-01	8.6E-01
bta-miR-181b	-0.54	1.3E-01	4.4E-01	bta-miR-379	-1.27	2.2E-01	8.6E-01
bta-miR-30d	0.32	1.3E-01	4.4E-01	bta-miR-345-3p	0.37	2.3E-01	8.6E-01
bta-miR-451	1.10	1.3E-01	4.4E-01	bta-miR-202	-0.40	2.3E-01	8.6E-01
bta-miR-375	0.47	1.4E-01	4.6E-01	bta-miR-208a	0.61	2.3E-01	8.6E-01
Novel:NC_037547.1_9249	-2.01	1.4E-01	4.7E-01	bta-miR-151-3p	0.25	2.3E-01	8.6E-01
bta-miR-34b	-0.63	1.5E-01	4.9E-01	bta-miR-135a	-0.44	2.3E-01	8.6E-01
Novel:chi-miR-30a-3p	0.46	1.5E-01	4.9E-01	bta-miR-200b	-0.80	2.3E-01	8.6E-01
bta-miR-450a	-0.96	1.6E-01	5.1E-01	bta-miR-197	0.37	2.3E-01	8.6E-01
Novel:NC_037561.1_37393	-1.70	1.6E-01	5.1E-01	bta-miR-29d-5p	-0.47	2.3E-01	8.6E-01
bta-miR-425-5p	-0.41	1.6E-01	5.1E-01	Novel:NC_037547.1_9085	-0.72	2.4E-01	8.6E-01
bta-miR-874	1.27	1.6E-01	5.2E-01	bta-miR-133a	0.68	2.4E-01	8.6E-01
bta-miR-339b	-1.50	1.7E-01	5.2E-01	bta-miR-212	0.55	2.4E-01	8.6E-01
Novel:NC_037555.1_25337	-0.51	1.7E-01	5.2E-01	bta-miR-484	0.34	2.4E-01	8.6E-01
Novel:NC_037547.1_8475	-0.47	1.7E-01	5.2E-01	bta-miR-345-5p	-0.53	2.4E-01	8.6E-01
Novel:NC_037563.1_42211	-0.62	1.7E-01	5.2E-01	bta-miR-423-3p	0.28	2.4E-01	8.6E-01
bta-miR-1224	1.06	1.7E-01	5.2E-01	bta-miR-541	0.72	2.4E-01	8.6E-01
Novel:NC_037567.1_45279	-1.45	1.7E-01	5.2E-01	Novel:NC_037568.1_46052	0.37	2.4E-01	8.6E-01
bta-miR-30f	-0.61	1.9E-01	5.6E-01	bta-miR-143	0.50	2.5E-01	8.7E-01
bta-miR-1343-3p	-0.70	1.9E-01	5.6E-01	bta-miR-211	-0.80	2.5E-01	8.7E-01
bta-miR-10174-3p	0.57	1.9E-01	5.7E-01	bta-miR-3432a	-0.87	2.6E-01	8.8E-01
Novel:NC_037549.1_13176	0.32	2.0E-01	5.7E-01	bta-miR-12030	0.77	2.6E-01	8.8E-01
bta-miR-216b	0.60	2.0E-01	5.7E-01	bta-miR-199a-3p	0.63	2.6E-01	8.8E-01
Novel:NC_037563.1_40476	-0.86	2.0E-01	5.7E-01	bta-miR-493	0.58	2.6E-01	8.8E-01
bta-miR-2285p	1.75	2.0E-01	5.7E-01	bta-miR-2475	0.75	2.6E-01	8.8E-01

Novel:NC_037569.1_48108	0.39	2.0E-01	5.7E-01	bta-miR-551b	-0.43	2.7E-01	8.8E-01
Novel:NC_037569.1_48104	0.39	2.0E-01	5.7E-01	Novel:NC_037558.1_32904	-0.68	2.7E-01	8.8E-01
Novel:NC_037561.1_37274	1.71	2.0E-01	5.7E-01	bta-miR-2284ab	-0.34	2.7E-01	8.8E-01
Novel:NC_037563.1_40790	-1.71	2.0E-01	5.7E-01	bta-miR-30d	0.22	2.7E-01	8.8E-01
bta-let-7e	0.50	2.0E-01	5.7E-01	bta-miR-1388-5p	-0.39	2.8E-01	8.8E-01
bta-miR-101	-0.31	2.1E-01	5.8E-01	Novel:NC_037567.1_45438	-0.70	2.8E-01	8.8E-01
Novel:NC_037550.1_16127	0.38	2.1E-01	5.8E-01	bta-miR-450a	0.51	2.8E-01	8.8E-01
Novel:NC_037548.1_11355	-0.90	2.1E-01	5.8E-01	Novel:NC_037545.1_9	-0.70	2.8E-01	8.8E-01
bta-miR-151-5p	-0.41	2.1E-01	5.8E-01	bta-miR-12041	0.93	2.8E-01	8.8E-01
Novel:NC_037547.1_9221	0.76	2.1E-01	5.8E-01	Novel:NC_037548.1_11027	0.92	2.8E-01	8.8E-01
bta-miR-128	-0.44	2.1E-01	5.8E-01	Novel:chi-miR-2284d	0.54	2.9E-01	8.8E-01
bta-miR-551b	-1.20	2.2E-01	5.8E-01	Novel:NC_037565.1_43614	-0.35	2.9E-01	8.8E-01
Novel:NC_037553.1_24445	0.48	2.2E-01	5.9E-01	bta-miR-194b-3p	0.55	2.9E-01	8.8E-01
bta-miR-7180	1.77	2.2E-01	5.9E-01	bta-miR-194b	0.55	2.9E-01	8.8E-01
bta-miR-146a	0.38	2.2E-01	5.9E-01	Novel:NC_037560.1_36165	0.78	2.9E-01	8.8E-01
Novel:NC_037562.1_40225	-1.90	2.2E-01	5.9E-01	bta-miR-296-3p	0.31	2.9E-01	8.9E-01
bta-miR-2285bz	-0.95	2.2E-01	5.9E-01	bta-miR-10b	-0.27	2.9E-01	8.9E-01
bta-miR-2285aj-5p	-0.68	2.3E-01	5.9E-01	bta-miR-328	0.32	3.0E-01	8.9E-01
Novel:NC_037545.1_1537	1.37	2.3E-01	5.9E-01	bta-miR-153	-0.32	3.0E-01	8.9E-01
Novel:NC_037558.1_30609	0.66	2.3E-01	5.9E-01	bta-miR-28	0.32	3.0E-01	8.9E-01
bta-miR-99a-5p	-0.48	2.3E-01	5.9E-01	bta-miR-31	0.20	3.0E-01	8.9E-01
bta-miR-30a-5p	-0.27	2.3E-01	5.9E-01	bta-miR-21-3p	0.36	3.0E-01	8.9E-01
bta-miR-105a	0.35	2.3E-01	5.9E-01	Novel:NC_037550.1_15803	-0.87	3.0E-01	8.9E-01
bta-miR-449a	0.41	2.3E-01	5.9E-01	Novel:NC_037556.1_28823	-0.88	3.0E-01	8.9E-01
Novel:NC_037555.1_27401	-1.66	2.4E-01	6.0E-01	bta-miR-30c	0.29	3.1E-01	9.0E-01
bta-miR-301b	0.80	2.4E-01	6.0E-01	bta-miR-2285aa	0.48	3.1E-01	9.0E-01
bta-miR-15b	0.44	2.4E-01	6.0E-01	bta-miR-23b-3p	0.27	3.1E-01	9.0E-01
bta-miR-142-3p	-1.43	2.4E-01	6.0E-01	bta-miR-130a	-0.22	3.1E-01	9.0E-01
bta-miR-193a-3p	-0.51	2.4E-01	6.0E-01	bta-miR-421	0.32	3.2E-01	9.1E-01
bta-miR-197	-0.83	2.4E-01	6.0E-01	bta-miR-2387	-0.64	3.3E-01	9.3E-01
bta-miR-2285s	-1.72	2.5E-01	6.0E-01	bta-miR-324	0.31	3.4E-01	9.4E-01
bta-miR-23b-3p	0.54	2.6E-01	6.2E-01	Novel:NC_037568.1_46470	-0.38	3.4E-01	9.4E-01
bta-miR-483	-1.43	2.6E-01	6.2E-01	Novel:NC_037564.1_42483	0.39	3.4E-01	9.4E-01
bta-miR-125a	-0.36	2.6E-01	6.2E-01	bta-miR-1839	0.27	3.4E-01	9.4E-01
bta-miR-2285ce	-1.28	2.6E-01	6.2E-01	Novel:NC_037548.1_11804	-0.74	3.4E-01	9.4E-01
Novel:NC_037546.1_2390	-1.29	2.6E-01	6.2E-01	bta-miR-196a	-0.87	3.4E-01	9.4E-01
bta-let-7d	-0.51	2.7E-01	6.3E-01	bta-miR-2411-5p	0.67	3.5E-01	9.4E-01
bta-miR-191	-0.27	2.7E-01	6.3E-01	Novel:chi-miR-2284a	0.30	3.5E-01	9.4E-01
bta-miR-338	-0.37	2.7E-01	6.3E-01	bta-miR-204	-0.27	3.5E-01	9.4E-01
Novel:NC_037563.1_40828	0.98	2.7E-01	6.3E-01	Novel:NC_037561.1_38157	-0.83	3.5E-01	9.4E-01
bta-miR-363	0.61	2.7E-01	6.3E-01	bta-miR-495	-0.51	3.6E-01	9.4E-01
Novel:NC_037568.1_46154	0.86	2.8E-01	6.4E-01	bta-miR-505	0.33	3.6E-01	9.4E-01
Novel:NC_037562.1_40173	-1.13	2.8E-01	6.5E-01	bta-miR-502b	-0.27	3.6E-01	9.4E-01
Novel:NC_037564.1_42483	-0.91	2.8E-01	6.5E-01	bta-miR-2285da	0.60	3.6E-01	9.4E-01
bta-miR-18a	0.43	2.9E-01	6.5E-01	bta-miR-1343-5p	-0.57	3.7E-01	9.5E-01
Novel:chi-miR-2404	0.34	2.9E-01	6.5E-01	Novel:NC_037555.1_26130	0.52	3.7E-01	9.5E-01
Novel:NC_037561.1_38294	-0.27	2.9E-01	6.5E-01	bta-miR-592	0.67	3.7E-01	9.5E-01

bta-miR-26b	-0.29	2.9E-01	6.5E-01	bta-miR-485	-0.92	3.7E-01	9.5E-01
bta-miR-7	0.78	2.9E-01	6.5E-01	Novel:NC_037564.1_42641	0.47	3.8E-01	9.5E-01
bta-miR-411a	-0.89	2.9E-01	6.5E-01	bta-miR-2285bw	-0.42	3.8E-01	9.5E-01
bta-miR-205	0.71	2.9E-01	6.5E-01	bta-miR-148a	-0.15	3.8E-01	9.5E-01
bta-miR-2285dh	-1.30	3.0E-01	6.5E-01	Novel:NC_037564.1_42815	0.32	3.8E-01	9.5E-01
Novel:NC_037569.1_47342	1.08	3.0E-01	6.5E-01	bta-miR-3613a	0.30	3.8E-01	9.5E-01
bta-miR-1306	1.21	3.0E-01	6.5E-01	bta-miR-6518	0.34	3.8E-01	9.5E-01
Novel:NC_037549.1_14727	0.61	3.0E-01	6.5E-01	bta-miR-2285k	-0.28	3.8E-01	9.5E-01
bta-miR-486	0.55	3.0E-01	6.5E-01	bta-miR-181a	0.23	3.8E-01	9.5E-01
bta-miR-16a	0.26	3.0E-01	6.5E-01	bta-miR-1343-3p	0.25	3.9E-01	9.5E-01
bta-miR-195	-0.41	3.1E-01	6.5E-01	Novel:NC_037564.1_42846	0.55	3.9E-01	9.5E-01
bta-miR-106b	-0.48	3.1E-01	6.5E-01	Novel:chi-miR-130a-3p	-0.29	3.9E-01	9.5E-01
bta-miR-767	0.26	3.1E-01	6.5E-01	bta-miR-665	0.57	3.9E-01	9.5E-01
Novel:NC_037546.1_4570	-1.04	3.1E-01	6.5E-01	bta-miR-30a-5p	-0.16	3.9E-01	9.5E-01
Novel:NC_037547.1_10452	-0.48	3.1E-01	6.5E-01	bta-miR-1307	0.24	3.9E-01	9.5E-01
Novel:NC_037560.1_35672	-0.82	3.1E-01	6.5E-01	bta-let-7b	0.17	3.9E-01	9.5E-01
Novel:NC_037569.1_48575	1.26	3.2E-01	6.5E-01	bta-miR-532	-0.17	4.0E-01	9.5E-01
bta-miR-200a	0.92	3.2E-01	6.5E-01	bta-miR-340	0.30	4.0E-01	9.5E-01
Novel:NC_037561.1_36870	-0.68	3.2E-01	6.5E-01	bta-miR-361	0.21	4.0E-01	9.6E-01
Novel:chi-miR-202-5p	-0.79	3.2E-01	6.5E-01	Novel:NC_037558.1_30589	0.47	4.0E-01	9.6E-01
bta-miR-126-5p	-0.70	3.2E-01	6.5E-01	bta-miR-11986b	-0.27	4.0E-01	9.6E-01
Novel:NC_037555.1_26084	0.95	3.2E-01	6.5E-01	Novel:NC_037557.1_29612	-0.82	4.1E-01	9.6E-01
Novel:chi-miR-1388-3p	-0.82	3.2E-01	6.5E-01	Novel:chi-miR-3432-5p	-0.26	4.1E-01	9.6E-01
Novel:NC_037552.1_22451	1.31	3.2E-01	6.5E-01	bta-miR-2416	-0.46	4.1E-01	9.6E-01
Novel:NC_037563.1_40470	0.57	3.3E-01	6.6E-01	bta-miR-2285cm	0.24	4.1E-01	9.7E-01
Novel:NC_037556.1_29441	-0.89	3.3E-01	6.6E-01	bta-miR-2424	-0.32	4.2E-01	9.7E-01
bta-miR-2440	0.40	3.3E-01	6.6E-01	bta-miR-335	-0.25	4.2E-01	9.7E-01
Novel:NC_037547.1_8434	0.36	3.3E-01	6.6E-01	bta-miR-2285bu	-0.41	4.2E-01	9.8E-01
Novel:chi-miR-2284a	-1.05	3.4E-01	6.7E-01	bta-miR-1260b	0.32	4.3E-01	9.8E-01
Novel:NC_037561.1_39262	0.39	3.4E-01	6.7E-01	bta-miR-2285aw	0.30	4.4E-01	9.8E-01
bta-miR-99a-3p	-0.76	3.4E-01	6.7E-01	bta-miR-210	0.17	4.4E-01	9.8E-01
Novel:NC_037547.1_10767	-0.55	3.5E-01	6.8E-01	bta-miR-365-3p	-0.24	4.4E-01	9.8E-01
Novel:NC_037546.1_6365	0.61	3.5E-01	6.8E-01	bta-miR-2285ar	-0.55	4.4E-01	9.8E-01
Novel:NC_037548.1_11804	1.33	3.5E-01	6.8E-01	Novel:NC_037567.1_45279	-0.26	4.4E-01	9.8E-01
bta-miR-574	0.58	3.5E-01	6.8E-01	Novel:NC_037548.1_10849	0.37	4.4E-01	9.8E-01
Novel:NC_037550.1_18621	1.11	3.5E-01	6.8E-01	bta-miR-382	-0.53	4.4E-01	9.8E-01
bta-miR-92a	0.19	3.7E-01	7.0E-01	bta-miR-3431	-0.22	4.5E-01	9.8E-01
Novel:NC_037549.1_14734	1.05	3.7E-01	7.0E-01	Novel:NC_037545.1_973	-0.35	4.5E-01	9.8E-01
Novel:NC_037550.1_16338	0.35	3.7E-01	7.1E-01	bta-miR-224	0.33	4.5E-01	9.8E-01
bta-miR-199b	-0.47	3.8E-01	7.1E-01	bta-miR-2285bp	-0.60	4.6E-01	9.8E-01
bta-miR-1271	-0.63	3.8E-01	7.1E-01	bta-miR-2285g	-0.71	4.6E-01	9.8E-01
Novel:NC_037550.1_17836	0.49	3.8E-01	7.1E-01	bta-miR-874	-0.23	4.6E-01	9.8E-01
bta-miR-135a	1.14	3.8E-01	7.1E-01	bta-miR-2483-5p	-0.28	4.7E-01	9.8E-01
Novel:NC_037567.1_45497	0.71	3.9E-01	7.2E-01	bta-miR-2468	0.34	4.7E-01	9.8E-01
Novel:NC_037564.1_42624	-0.97	3.9E-01	7.2E-01	bta-let-7f	0.14	4.7E-01	9.8E-01
bta-miR-218	-0.65	3.9E-01	7.2E-01	bta-miR-2403	-0.43	4.7E-01	9.8E-01
bta-miR-424-3p	0.69	3.9E-01	7.2E-01	Novel:NC_037550.1_17661	0.37	4.7E-01	9.8E-01

bta-miR-105b	0.27	3.9E-01	7.2E-01	bta-miR-378b	0.27	4.7E-01	9.8E-01
bta-miR-374a	-0.66	3.9E-01	7.2E-01	Novel:NC_037560.1_35793	0.55	4.7E-01	9.8E-01
Novel:NC_037558.1_31773	-1.06	4.0E-01	7.3E-01	Novel:NC_037561.1_37749	-0.22	4.7E-01	9.8E-01
bta-miR-193a-5p	0.99	4.0E-01	7.3E-01	bta-miR-95	0.38	4.7E-01	9.8E-01
bta-let-7f	-0.27	4.1E-01	7.3E-01	Novel:NC_037567.1_45690	0.22	4.8E-01	9.8E-01
bta-miR-215	-0.59	4.1E-01	7.4E-01	bta-miR-331-3p	-0.23	4.8E-01	9.8E-01
Novel:chi-miR-665	0.37	4.2E-01	7.6E-01	bta-miR-2484	-0.23	4.8E-01	9.8E-01
Novel:NC_037550.1_15803	0.23	4.2E-01	7.6E-01	bta-miR-1306	0.26	4.8E-01	9.8E-01
bta-miR-2285bn	-0.85	4.3E-01	7.7E-01	bta-miR-23a	0.23	4.8E-01	9.8E-01
bta-miR-26a	-0.17	4.4E-01	7.7E-01	bta-miR-2367-5p	0.48	4.9E-01	9.8E-01
bta-miR-17-3p	-0.42	4.4E-01	7.8E-01	Novel:NC_037561.1_36933	0.47	4.9E-01	9.8E-01
bta-miR-93	-0.22	4.4E-01	7.8E-01	bta-miR-137	0.47	4.9E-01	9.8E-01
bta-miR-2284x	-0.18	4.5E-01	7.8E-01	bta-miR-146a	-0.38	4.9E-01	9.8E-01
Novel:NC_037558.1_31385	0.27	4.5E-01	7.8E-01	bta-miR-142-3p	-0.40	4.9E-01	9.8E-01
bta-miR-4449	0.77	4.5E-01	7.8E-01	Novel:NC_037558.1_31721	0.46	5.0E-01	9.8E-01
bta-miR-3660	0.43	4.5E-01	7.8E-01	bta-miR-500	-0.22	5.0E-01	9.8E-01
Novel:NC_037561.1_37655	0.45	4.5E-01	7.8E-01	bta-miR-1246	-0.31	5.0E-01	9.8E-01
bta-miR-449b	-1.02	4.6E-01	7.9E-01	bta-miR-99a-3p	0.20	5.0E-01	9.8E-01
Novel:NC_037545.1_897	0.61	4.6E-01	7.9E-01	bta-miR-2285br	-0.29	5.0E-01	9.8E-01
bta-miR-6119-5p	-0.24	4.6E-01	7.9E-01	bta-miR-652	-0.19	5.0E-01	9.8E-01
bta-miR-148a	-0.16	4.7E-01	7.9E-01	Novel:NC_037557.1_30374	0.44	5.1E-01	9.8E-01
Novel:NC_037546.1_5316	0.52	4.9E-01	8.2E-01	bta-miR-2397-3p	-0.52	5.1E-01	9.8E-01
Novel:NC_037548.1_11571	0.82	4.9E-01	8.2E-01	bta-miR-16a	-0.14	5.1E-01	9.8E-01
bta-miR-28	-0.35	4.9E-01	8.2E-01	bta-miR-24-3p	-0.19	5.1E-01	9.8E-01
bta-miR-6520	1.00	4.9E-01	8.2E-01	bta-miR-193a-3p	-0.21	5.1E-01	9.8E-01
Novel:NC_037550.1_18749	-0.57	4.9E-01	8.2E-01	bta-miR-15b	-0.17	5.2E-01	9.8E-01
Novel:NC_037553.1_23614	-0.53	5.0E-01	8.2E-01	bta-miR-7	-0.30	5.2E-01	9.8E-01
bta-miR-7857-5p	0.29	5.0E-01	8.2E-01	Novel:NC_037553.1_23805	-0.58	5.2E-01	9.8E-01
Novel:NC_037558.1_32918	0.74	5.0E-01	8.2E-01	bta-miR-208b	-0.27	5.3E-01	9.8E-01
Novel:NC_037545.1_51	-0.45	5.1E-01	8.2E-01	Novel:NC_037549.1_13176	-0.29	5.3E-01	9.8E-01
bta-let-7g	-0.22	5.1E-01	8.2E-01	bta-miR-545-5p	0.19	5.3E-01	9.8E-01
Novel:NC_037554.1_24869	0.25	5.1E-01	8.2E-01	Novel:NC_037557.1_29957	-0.35	5.3E-01	9.8E-01
bta-miR-365-3p	-0.41	5.1E-01	8.2E-01	bta-miR-2311	0.75	5.3E-01	9.8E-01
Novel:NC_037546.1_2418	0.69	5.1E-01	8.2E-01	Novel:NC_037553.1_24503	-0.34	5.3E-01	9.8E-01
Novel:NC_037547.1_8269	0.69	5.2E-01	8.4E-01	bta-miR-365-5p	0.31	5.3E-01	9.8E-01
Novel:NC_037551.1_21705	-0.66	5.2E-01	8.4E-01	bta-miR-2284p	0.52	5.3E-01	9.8E-01
Novel:NC_037558.1_31562	0.30	5.3E-01	8.4E-01	Novel:NC_037547.1_9210	-0.31	5.4E-01	9.8E-01
bta-miR-193b	-0.41	5.3E-01	8.4E-01	Novel:NC_037547.1_8617	0.19	5.4E-01	9.8E-01
bta-miR-187	0.63	5.3E-01	8.4E-01	bta-miR-185	-0.25	5.4E-01	9.8E-01
bta-miR-99b	-0.23	5.3E-01	8.4E-01	bta-miR-26b	-0.12	5.4E-01	9.8E-01
Novel:NC_037569.1_48099	-0.65	5.3E-01	8.4E-01	bta-miR-19b	-0.14	5.4E-01	9.8E-01
bta-miR-381	-0.54	5.3E-01	8.4E-01	bta-miR-183	-0.35	5.4E-01	9.8E-01
bta-miR-2285f	-0.73	5.4E-01	8.4E-01	bta-miR-215	-0.21	5.4E-01	9.8E-01
bta-miR-192	-0.18	5.4E-01	8.4E-01	Novel:NC_037549.1_14019	0.36	5.4E-01	9.8E-01
Novel:NC_037569.1_46617	0.23	5.5E-01	8.5E-01	Novel:NC_037562.1_40333	0.53	5.4E-01	9.8E-01
Novel:chi-miR-543-5p	-0.79	5.6E-01	8.6E-01	bta-miR-330	0.36	5.4E-01	9.8E-01
Novel:NC_037565.1_43646	-0.43	5.6E-01	8.6E-01	Novel:NC_037547.1_7809	-0.38	5.5E-01	9.8E-01

Novel:NC_037546.1_6452	0.60	5.6E-01	8.6E-01	Novel:NC_037560.1_36384	0.40	5.6E-01	9.8E-01
bta-miR-10164-3p	-0.85	5.6E-01	8.6E-01	Novel:NC_037553.1_24324	0.27	5.6E-01	9.8E-01
Novel:NC_037557.1_30243	0.21	5.7E-01	8.7E-01	bta-miR-342	0.14	5.6E-01	9.8E-01
bta-miR-15a	-0.20	5.7E-01	8.7E-01	bta-miR-199c	0.34	5.6E-01	9.8E-01
Novel:NC_037567.1_45572	-0.40	5.7E-01	8.7E-01	bta-miR-127	-0.27	5.6E-01	9.8E-01
Novel:chi-miR-877-3p	0.34	5.7E-01	8.7E-01	bta-miR-2355-3p	-0.43	5.6E-01	9.8E-01
bta-miR-34c	0.14	5.8E-01	8.7E-01	bta-miR-186	0.10	5.7E-01	9.8E-01
bta-miR-16b	-0.13	5.8E-01	8.8E-01	bta-miR-362-5p	-0.22	5.7E-01	9.8E-01
bta-miR-214	-0.57	5.8E-01	8.8E-01	Novel:NC_037556.1_29271	-0.23	5.8E-01	9.8E-01
bta-miR-491	0.71	6.0E-01	9.0E-01	Novel:NC_037555.1_25337	0.41	5.8E-01	9.8E-01
bta-miR-6518	-0.42	6.0E-01	9.0E-01	Novel:NC_037548.1_11872	0.47	5.8E-01	9.8E-01
Novel:chi-miR-324-5p	0.31	6.1E-01	9.0E-01	bta-miR-192	0.14	5.8E-01	9.8E-01
Novel:NC_037563.1_40601	-0.19	6.2E-01	9.2E-01	bta-miR-145	0.22	5.8E-01	9.8E-01
bta-miR-154c	-0.62	6.2E-01	9.2E-01	bta-miR-2285ag-3p	0.28	5.8E-01	9.8E-01
Novel:chi-miR-130a-3p	-0.20	6.2E-01	9.2E-01	bta-miR-11972	0.38	5.8E-01	9.8E-01
Novel:NC_037545.1_1119	0.50	6.3E-01	9.2E-01	bta-miR-2285s	-0.24	5.8E-01	9.8E-01
Novel:NC_037569.1_48102	-0.26	6.3E-01	9.2E-01	bta-miR-677	-0.16	5.8E-01	9.8E-01
Novel:NC_037561.1_38157	0.11	6.3E-01	9.2E-01	bta-miR-362-3p	-0.23	5.9E-01	9.8E-01
Novel:NC_037569.1_48106	-0.26	6.3E-01	9.2E-01	bta-miR-374b	-0.14	5.9E-01	9.8E-01
bta-miR-181d	0.29	6.4E-01	9.3E-01	Novel:NC_037551.1_19028	-0.31	5.9E-01	9.8E-01
bta-miR-106a	0.30	6.4E-01	9.3E-01	Novel:NC_037558.1_31832	-0.27	5.9E-01	9.8E-01
bta-miR-2411-5p	0.63	6.4E-01	9.3E-01	bta-miR-2285dh	-0.16	5.9E-01	9.8E-01
bta-miR-340	-0.37	6.5E-01	9.4E-01	bta-miR-2285bl	0.16	5.9E-01	9.8E-01
bta-miR-503-3p	-0.57	6.6E-01	9.5E-01	bta-miR-193a-5p	-0.22	5.9E-01	9.8E-01
bta-miR-628	0.49	6.7E-01	9.5E-01	bta-miR-34a	-0.18	5.9E-01	9.8E-01
bta-miR-141	-0.13	6.7E-01	9.5E-01	bta-miR-146b	-0.19	5.9E-01	9.8E-01
bta-miR-335	-0.13	6.7E-01	9.5E-01	bta-miR-378d	-0.41	6.0E-01	9.8E-01
bta-miR-152	0.30	6.7E-01	9.5E-01	bta-miR-190a	-0.20	6.0E-01	9.8E-01
Novel:oar-miR-1197-5p	-0.50	6.8E-01	9.5E-01	bta-miR-10182-5p	0.37	6.0E-01	9.8E-01
bta-let-7a-3p	-0.24	6.8E-01	9.5E-01	bta-miR-2285au	0.78	6.0E-01	9.8E-01
bta-miR-92b	-0.11	6.8E-01	9.5E-01	bta-miR-106a	0.18	6.0E-01	9.8E-01
Novel:NC_037547.1_10766	0.19	6.8E-01	9.5E-01	bta-miR-2461-3p	0.47	6.0E-01	9.8E-01
bta-miR-6119-3p	-0.52	6.8E-01	9.5E-01	Novel:NC_037561.1_36952	-0.17	6.1E-01	9.8E-01
Novel:NC_037549.1_13991	-0.18	6.9E-01	9.5E-01	bta-miR-2284aa	-0.41	6.1E-01	9.8E-01
Novel:NC_037552.1_22658	0.43	6.9E-01	9.5E-01	Novel:NC_037548.1_11132	0.29	6.2E-01	9.8E-01
bta-miR-183	-0.22	6.9E-01	9.5E-01	bta-miR-182	-0.18	6.2E-01	9.8E-01
bta-miR-449c	0.32	7.0E-01	9.5E-01	bta-miR-9-5p	0.21	6.2E-01	9.8E-01
Novel:NC_037547.1_8284	-0.35	7.0E-01	9.5E-01	bta-miR-151-5p	0.11	6.2E-01	9.8E-01
Novel:NC_037555.1_25446	0.36	7.0E-01	9.5E-01	bta-miR-425-5p	0.11	6.2E-01	9.8E-01
Novel:NC_037558.1_32599	0.25	7.0E-01	9.6E-01	Novel:NC_037548.1_12085	0.27	6.2E-01	9.8E-01
bta-miR-190a	-0.38	7.1E-01	9.7E-01	Novel:NC_037565.1_43873	0.28	6.3E-01	9.8E-01
Novel:NC_037546.1_6477	0.13	7.1E-01	9.7E-01	Novel:NC_037547.1_9221	0.19	6.3E-01	9.8E-01
Novel:NC_037556.1_28703	0.26	7.2E-01	9.8E-01	bta-miR-15a	-0.13	6.3E-01	9.8E-01
bta-miR-296-5p	0.47	7.2E-01	9.8E-01	Novel:NC_037553.1_24464	0.16	6.4E-01	9.8E-01
bta-miR-2424	-0.40	7.3E-01	9.8E-01	bta-miR-219-5p	-0.35	6.4E-01	9.8E-01
Novel:NC_037548.1_11964	0.25	7.3E-01	9.8E-01	bta-miR-6517	0.14	6.4E-01	9.8E-01
bta-miR-32	0.29	7.4E-01	9.8E-01	bta-miR-582	-0.19	6.4E-01	9.8E-01

bta-miR-2284ab	-0.44	7.4E-01	9.8E-01	bta-miR-381	-0.22	6.4E-01	9.8E-01
bta-miR-181a	0.10	7.4E-01	9.8E-01	bta-miR-10174-3p	0.11	6.5E-01	9.8E-01
bta-miR-2285bl	0.34	7.4E-01	9.8E-01	bta-miR-378c	-0.16	6.5E-01	9.8E-01
bta-miR-194	0.11	7.4E-01	9.8E-01	bta-miR-7858	-0.30	6.5E-01	9.8E-01
Novel:NC_037566.1_44909	0.11	7.4E-01	9.8E-01	bta-miR-488	-0.27	6.6E-01	9.8E-01
Novel:NC_037549.1_14113	0.20	7.4E-01	9.8E-01	bta-miR-455-5p	-0.18	6.6E-01	9.8E-01
Novel:NC_037560.1_36199	0.43	7.5E-01	9.8E-01	bta-miR-27a-3p	-0.13	6.6E-01	9.8E-01
Novel:NC_037566.1_44408	-0.38	7.5E-01	9.8E-01	bta-miR-320a	0.11	6.6E-01	9.8E-01
bta-miR-677	-0.21	7.5E-01	9.8E-01	bta-miR-101	-0.10	6.7E-01	9.8E-01
Novel:NC_037547.1_9210	0.34	7.6E-01	9.8E-01	bta-miR-2285f	-0.13	6.7E-01	9.8E-01
bta-miR-425-3p	0.31	7.6E-01	9.8E-01	Novel:NC_037549.1_13802	-0.21	6.7E-01	9.8E-01
Novel:NC_037569.1_48096	0.13	7.6E-01	9.8E-01	bta-miR-1296	0.12	6.7E-01	9.8E-01
bta-miR-212	-0.46	7.6E-01	9.8E-01	bta-miR-2285co	-0.17	6.7E-01	9.8E-01
Novel:NC_037569.1_48098	0.13	7.6E-01	9.8E-01	Novel:NC_037551.1_21705	-0.14	6.7E-01	9.8E-01
Novel:NC_037552.1_22845	0.10	7.6E-01	9.8E-01	bta-miR-29b	0.15	6.7E-01	9.8E-01
bta-miR-499	-0.16	7.7E-01	9.8E-01	bta-miR-184	0.28	6.7E-01	9.8E-01
bta-miR-98	0.14	7.7E-01	9.8E-01	bta-miR-107	-0.11	6.7E-01	9.8E-01
bta-miR-182	0.07	7.7E-01	9.8E-01	bta-miR-2285c	-0.22	6.8E-01	9.8E-01
bta-miR-30b-5p	-0.06	7.7E-01	9.8E-01	bta-miR-2285bz	-0.13	6.8E-01	9.8E-01
bta-miR-188	-0.25	7.7E-01	9.8E-01	bta-miR-326	-0.25	6.8E-01	9.8E-01
bta-miR-27b	-0.06	7.8E-01	9.8E-01	bta-miR-671	0.13	6.8E-01	9.8E-01
Novel:NC_037547.1_10745	0.12	7.8E-01	9.8E-01	bta-miR-195	-0.09	6.8E-01	9.8E-01
bta-miR-760-3p	-0.20	7.8E-01	9.8E-01	bta-miR-30b-3p	-0.17	6.8E-01	9.8E-01
bta-miR-6529a	-0.33	7.8E-01	9.8E-01	bta-miR-2285b	-0.17	6.8E-01	9.8E-01
bta-miR-2435	0.10	7.9E-01	9.8E-01	bta-miR-199b	0.16	6.8E-01	9.8E-01
bta-miR-1291	0.40	7.9E-01	9.8E-01	bta-miR-99b	0.12	6.9E-01	9.8E-01
bta-miR-20b	-0.14	8.0E-01	9.8E-01	bta-miR-2285ce	-0.12	6.9E-01	9.8E-01
bta-miR-208b	0.41	8.0E-01	9.8E-01	bta-miR-181c	-0.11	6.9E-01	9.8E-01
bta-miR-328	0.08	8.0E-01	9.8E-01	bta-miR-2477	-0.28	6.9E-01	9.8E-01
Novel:NC_037563.1_41848	0.08	8.0E-01	9.8E-01	Novel:NC_037561.1_37297	-0.35	6.9E-01	9.8E-01
Novel:NC_037551.1_21257	-0.37	8.0E-01	9.8E-01	bta-miR-708	0.18	7.0E-01	9.8E-01
bta-miR-2411-3p	-0.28	8.0E-01	9.8E-01	bta-miR-374c	-0.16	7.0E-01	9.8E-01
Novel:NC_037564.1_42469	0.22	8.0E-01	9.8E-01	bta-miR-148b	-0.08	7.0E-01	9.8E-01
Novel:NC_037566.1_44593	0.25	8.1E-01	9.8E-01	bta-miR-877	-0.16	7.0E-01	9.8E-01
Novel:NC_037567.1_45690	-0.22	8.1E-01	9.8E-01	bta-miR-7857-3p	0.19	7.0E-01	9.8E-01
bta-miR-9-3p	-0.19	8.1E-01	9.8E-01	bta-miR-20a	-0.09	7.0E-01	9.8E-01
bta-miR-502b	-0.17	8.1E-01	9.8E-01	bta-miR-214	0.17	7.0E-01	9.8E-01
bta-miR-18b	0.26	8.1E-01	9.8E-01	bta-miR-769	-0.09	7.0E-01	9.8E-01
Novel:NC_037563.1_41120	0.26	8.2E-01	9.8E-01	Novel:NC_037561.1_37393	0.12	7.1E-01	9.8E-01
Novel:NC_037555.1_25442	0.16	8.2E-01	9.8E-01	bta-miR-628	0.12	7.1E-01	9.8E-01
bta-miR-12006	-0.14	8.2E-01	9.8E-01	bta-miR-188	-0.12	7.1E-01	9.8E-01
bta-miR-204	0.06	8.2E-01	9.8E-01	Novel:NC_037547.1_8177	-0.16	7.1E-01	9.8E-01
bta-miR-11986b	0.26	8.2E-01	9.8E-01	bta-miR-2284c	0.19	7.2E-01	9.9E-01
Novel:NC_037555.1_26692	0.10	8.2E-01	9.8E-01	Novel:NC_037556.1_28871	-0.23	7.2E-01	9.9E-01
Novel:NC_037552.1_22884	-0.25	8.3E-01	9.8E-01	bta-miR-2284a	-0.14	7.2E-01	9.9E-01
Novel:NC_037546.1_6523	0.08	8.3E-01	9.9E-01	bta-miR-2285e	0.10	7.2E-01	9.9E-01
bta-miR-362-3p	0.29	8.4E-01	9.9E-01	bta-miR-6529a	0.14	7.3E-01	9.9E-01

Novel:NC_037565.1_43691	-0.09	8.4E-01	9.9E-01	Novel:NC_037569.1_48108	0.21	7.3E-01	9.9E-01
bta-miR-2285cm	0.20	8.4E-01	9.9E-01	Novel:NC_037569.1_48104	0.21	7.3E-01	9.9E-01
Novel:oar-miR-3956-3p	0.20	8.5E-01	9.9E-01	Novel:NC_037561.1_38294	-0.18	7.3E-01	9.9E-01
bta-miR-21-3p	0.15	8.5E-01	9.9E-01	bta-miR-2331-5p	-0.28	7.3E-01	9.9E-01
bta-miR-12034	-0.25	8.5E-01	9.9E-01	bta-miR-378	0.09	7.4E-01	9.9E-01
Novel:NC_037546.1_4671	-0.08	8.6E-01	9.9E-01	bta-miR-2435	0.10	7.4E-01	9.9E-01
Novel:NC_037547.1_8508	0.17	8.6E-01	9.9E-01	bta-miR-219b-3p	0.26	7.4E-01	9.9E-01
bta-miR-877	0.08	8.6E-01	9.9E-01	bta-miR-432	-0.18	7.4E-01	9.9E-01
bta-miR-301a	0.04	8.7E-01	9.9E-01	Novel:NC_037545.1_808	-0.26	7.5E-01	9.9E-01
bta-miR-1260b	0.16	8.7E-01	9.9E-01	bta-miR-2285aj-5p	0.11	7.5E-01	9.9E-01
bta-miR-107	-0.06	8.7E-01	9.9E-01	bta-let-7a-5p	0.06	7.5E-01	9.9E-01
bta-miR-17-5p	-0.04	8.8E-01	9.9E-01	Novel:NC_037566.1_44459	-0.28	7.5E-01	9.9E-01
bta-miR-155	-0.07	8.8E-01	9.9E-01	bta-miR-149-5p	0.10	7.5E-01	9.9E-01
Novel:NC_037550.1_16151	0.12	8.8E-01	9.9E-01	Novel:NC_037546.1_4570	-0.11	7.5E-01	9.9E-01
bta-miR-487b	-0.19	8.8E-01	9.9E-01	bta-miR-2331-3p	0.16	7.5E-01	9.9E-01
bta-miR-96	-0.13	8.8E-01	9.9E-01	Novel:NC_037568.1_45743	-0.09	7.6E-01	9.9E-01
Novel:NC_037562.1_40324	-0.13	8.8E-01	9.9E-01	bta-miR-487b	-0.19	7.6E-01	9.9E-01
bta-miR-1249	0.21	8.8E-01	9.9E-01	bta-miR-99a-5p	0.07	7.6E-01	9.9E-01
bta-miR-2419-5p	-0.18	8.9E-01	9.9E-01	bta-miR-331-5p	0.09	7.7E-01	9.9E-01
Novel:NC_037546.1_2163	0.08	8.9E-01	9.9E-01	bta-miR-30e-5p	-0.05	7.7E-01	9.9E-01
Novel:NC_037562.1_40333	-0.03	9.0E-01	9.9E-01	bta-miR-190b	-0.09	7.7E-01	9.9E-01
Novel:NC_037547.1_8617	-0.11	9.0E-01	9.9E-01	bta-miR-504	-0.10	7.7E-01	9.9E-01
bta-miR-2285e	0.15	9.0E-01	9.9E-01	bta-miR-1388-3p	0.10	7.7E-01	9.9E-01
Novel:NC_037559.1_34044	0.05	9.0E-01	9.9E-01	Novel:NC_037550.1_17518	-0.20	7.7E-01	9.9E-01
bta-miR-138	0.04	9.0E-01	9.9E-01	bta-miR-199a-5p	-0.11	7.8E-01	9.9E-01
bta-miR-29b	0.10	9.1E-01	9.9E-01	bta-miR-450b	0.11	7.8E-01	9.9E-01
bta-miR-2318	0.14	9.1E-01	9.9E-01	bta-miR-30b-5p	-0.07	7.8E-01	9.9E-01
bta-miR-126-3p	-0.09	9.1E-01	9.9E-01	bta-miR-11991	-0.22	7.8E-01	9.9E-01
bta-miR-2904	0.18	9.1E-01	9.9E-01	bta-miR-106b	-0.08	7.9E-01	9.9E-01
bta-miR-2285k	-0.11	9.1E-01	9.9E-01	bta-miR-296-5p	-0.16	7.9E-01	9.9E-01
bta-miR-148b	0.05	9.1E-01	9.9E-01	bta-miR-2285dl-3p	0.14	7.9E-01	9.9E-01
Novel:NC_037546.1_7398	0.15	9.1E-01	9.9E-01	bta-miR-2285ad	0.15	7.9E-01	9.9E-01
bta-let-7a-5p	0.03	9.2E-01	9.9E-01	bta-miR-491	-0.09	7.9E-01	9.9E-01
Novel:NC_037548.1_10786	0.11	9.2E-01	9.9E-01	bta-miR-455-3p	-0.10	7.9E-01	9.9E-01
Novel:NC_037556.1_28867	-0.05	9.2E-01	9.9E-01	Novel:NC_037558.1_31773	0.12	8.0E-01	9.9E-01
Novel:NC_037545.1_1469	-0.03	9.3E-01	9.9E-01	bta-miR-454	0.08	8.0E-01	9.9E-01
bta-miR-2285cj	-0.07	9.3E-01	9.9E-01	bta-miR-6121-3p	-0.16	8.0E-01	9.9E-01
Novel:NC_037569.1_47739	-0.08	9.3E-01	9.9E-01	bta-let-7g	0.05	8.0E-01	9.9E-01
Novel:NC_037553.1_24305	0.12	9.3E-01	9.9E-01	bta-miR-6522	0.20	8.0E-01	9.9E-01
Novel:NC_037555.1_25386	-0.08	9.4E-01	9.9E-01	Novel:NC_037565.1_43588	-0.19	8.0E-01	9.9E-01
bta-miR-181c	-0.03	9.4E-01	9.9E-01	bta-miR-17-5p	0.05	8.0E-01	9.9E-01
bta-miR-30c	0.02	9.4E-01	9.9E-01	Novel:NC_037554.1_24708	-0.21	8.0E-01	9.9E-01
Novel:NC_037550.1_18739	0.06	9.4E-01	9.9E-01	Novel:NC_037556.1_29441	-0.14	8.0E-01	9.9E-01
bta-miR-9-5p	0.02	9.5E-01	9.9E-01	bta-miR-424-3p	0.14	8.0E-01	9.9E-01
bta-miR-455-5p	-0.04	9.5E-01	9.9E-01	bta-miR-873	0.15	8.1E-01	9.9E-01
bta-miR-708	0.09	9.6E-01	9.9E-01	bta-miR-1277	0.18	8.1E-01	9.9E-01
bta-miR-27a-3p	0.01	9.6E-01	9.9E-01	bta-miR-1291	0.14	8.2E-01	9.9E-01

bta-miR-502a	0.05	9.6E-01	9.9E-01
bta-miR-21-5p	0.02	9.7E-01	9.9E-01
Novel:NC_037548.1_12261	-0.02	9.7E-01	9.9E-01
Novel:NC_037549.1_15527	-0.02	9.7E-01	9.9E-01
bta-miR-342	0.01	9.7E-01	9.9E-01
bta-miR-190b	-0.02	9.7E-01	9.9E-01
bta-miR-103	0.01	9.8E-01	9.9E-01
Novel:NC_037569.1_46616	-0.03	9.8E-01	9.9E-01
bta-miR-223	0.04	9.8E-01	9.9E-01
bta-miR-374b	-0.02	9.8E-01	9.9E-01
bta-miR-671	0.04	9.8E-01	9.9E-01
bta-miR-7857-3p	-0.01	9.8E-01	9.9E-01
bta-miR-224	0.02	9.8E-01	9.9E-01
bta-miR-10b	0.01	9.8E-01	9.9E-01
bta-miR-2483-3p	0.01	9.9E-01	9.9E-01
bta-miR-383	0.01	9.9E-01	9.9E-01
Novel:chi-miR-3432-3p	0.00	9.9E-01	9.9E-01
Novel:NC_037550.1_18562	0.00	9.9E-01	9.9E-01
Novel:NC_037557.1_29725	0.00	9.9E-01	9.9E-01
Novel:NC_037560.1_36420	0.00	9.9E-01	9.9E-01
Novel:NC_037558.1_32922	-0.01	9.9E-01	9.9E-01
bta-miR-20a	0.00	9.9E-01	9.9E-01
Novel:NC_037566.1_44712	0.01	9.9E-01	9.9E-01
bta-miR-331-3p	0.00	9.9E-01	9.9E-01
bta-miR-2318	0.08	8.2E-01	9.9E-01
bta-miR-20b	0.09	8.2E-01	9.9E-01
bta-miR-29c	0.07	8.2E-01	9.9E-01
bta-miR-218	-0.12	8.2E-01	9.9E-01
bta-miR-497	0.05	8.2E-01	9.9E-01
bta-miR-193b	-0.05	8.2E-01	9.9E-01
bta-miR-25	-0.04	8.3E-01	9.9E-01
bta-miR-2312	-0.12	8.3E-01	9.9E-01
Novel:NC_037551.1_21229	-0.07	8.3E-01	9.9E-01
bta-miR-103	-0.04	8.3E-01	9.9E-01
bta-miR-2285bo	-0.25	8.3E-01	9.9E-01
bta-miR-2284x	0.05	8.3E-01	9.9E-01
bta-miR-6119-5p	-0.04	8.4E-01	9.9E-01
bta-miR-140	-0.04	8.4E-01	9.9E-01
bta-miR-32	-0.07	8.4E-01	9.9E-01
bta-miR-27a-5p	0.14	8.4E-01	9.9E-01
bta-miR-2299-5p	-0.06	8.4E-01	9.9E-01
bta-miR-429	0.11	8.4E-01	9.9E-01
bta-miR-194	0.06	8.5E-01	9.9E-01
bta-miR-655	-0.18	8.5E-01	9.9E-01
bta-miR-7859	0.19	8.5E-01	9.9E-01
Novel:NC_037569.1_48011	-0.06	8.5E-01	9.9E-01
Novel:NC_037553.1_24530	-0.13	8.6E-01	9.9E-01
bta-miR-147	-0.08	8.6E-01	9.9E-01
bta-miR-10164-3p	0.06	8.6E-01	9.9E-01
bta-miR-33a	-0.09	8.6E-01	9.9E-01
bta-miR-542-5p	-0.07	8.6E-01	9.9E-01
bta-let-7d	-0.04	8.7E-01	9.9E-01
Novel:NC_037545.1_938	0.08	8.7E-01	9.9E-01
bta-miR-16b	0.03	8.7E-01	9.9E-01
Novel:NC_037565.1_43872	-0.11	8.7E-01	9.9E-01
bta-miR-30f	-0.05	8.7E-01	9.9E-01
Novel:NC_037558.1_32922	-0.05	8.7E-01	9.9E-01
Novel:NC_037557.1_30145	-0.11	8.8E-01	9.9E-01
bta-miR-660	-0.03	8.8E-01	9.9E-01
Novel:NC_037568.1_46078	-0.10	8.8E-01	9.9E-01
bta-miR-136	-0.12	8.8E-01	9.9E-01
bta-miR-2285o	-0.08	8.8E-01	9.9E-01
bta-miR-503-3p	-0.06	8.9E-01	9.9E-01
bta-miR-2382-3p	0.15	9.0E-01	9.9E-01
bta-miR-545-3p	0.04	9.0E-01	9.9E-01
bta-miR-93	-0.03	9.0E-01	9.9E-01
bta-let-7a-3p	0.04	9.0E-01	9.9E-01
bta-miR-374a	-0.03	9.0E-01	9.9E-01
bta-miR-18a	0.05	9.0E-01	9.9E-01
bta-miR-1271	0.05	9.0E-01	9.9E-01
Novel:NC_037545.1_493	0.05	9.0E-01	9.9E-01

bta-miR-17-3p	0.04	9.0E-01	9.9E-01
bta-let-7e	0.02	9.0E-01	9.9E-01
bta-miR-2284w	-0.11	9.1E-01	9.9E-01
Novel:NC_037556.1_28335	-0.04	9.1E-01	9.9E-01
Novel:NC_037547.1_10467	-0.04	9.1E-01	9.9E-01
Novel:NC_037549.1_14821	-0.07	9.1E-01	9.9E-01
bta-miR-138	-0.05	9.1E-01	9.9E-01
bta-miR-410	0.08	9.2E-01	9.9E-01
bta-miR-124a	0.13	9.2E-01	9.9E-01
bta-miR-425-3p	0.03	9.2E-01	9.9E-01
bta-miR-1249	0.04	9.2E-01	9.9E-01
Novel:NC_037546.1_4836	-0.04	9.2E-01	9.9E-01
bta-miR-744	-0.03	9.2E-01	9.9E-01
bta-miR-2285bn	-0.03	9.2E-01	9.9E-01
bta-miR-338	0.03	9.3E-01	9.9E-01
bta-miR-2284y	-0.03	9.3E-01	9.9E-01
bta-miR-152	-0.03	9.3E-01	9.9E-01
bta-miR-2284z	-0.05	9.3E-01	9.9E-01
bta-miR-100	0.03	9.3E-01	9.9E-01
Novel:NC_037567.1_45399	0.05	9.3E-01	9.9E-01
bta-miR-22-5p	-0.03	9.4E-01	9.9E-01
bta-miR-181d	-0.02	9.4E-01	9.9E-01
bta-miR-142-5p	0.05	9.4E-01	1.0E+00
Novel:NC_037559.1_33382	0.04	9.5E-01	1.0E+00
Novel:NC_037547.1_7592	0.04	9.5E-01	1.0E+00
bta-miR-6119-3p	-0.02	9.5E-01	1.0E+00
bta-miR-2284o	0.04	9.5E-01	1.0E+00
bta-miR-2284k	-0.03	9.6E-01	1.0E+00
Novel:NC_037557.1_30243	-0.04	9.6E-01	1.0E+00
bta-miR-33b	-0.03	9.6E-01	1.0E+00
Novel:NC_037552.1_22200	-0.04	9.7E-01	1.0E+00
bta-miR-423-5p	-0.01	9.7E-01	1.0E+00
bta-miR-18b	0.02	9.7E-01	1.0E+00
Novel:NC_037560.1_36156	0.02	9.7E-01	1.0E+00
Novel:NC_037546.1_6434	0.02	9.8E-01	1.0E+00
bta-miR-502a	0.02	9.8E-01	1.0E+00
Novel:NC_037548.1_10972	-0.02	9.8E-01	1.0E+00
bta-miR-6524	0.01	9.8E-01	1.0E+00
Novel:NC_037560.1_36199	0.01	9.8E-01	1.0E+00
bta-miR-191	0.00	9.9E-01	1.0E+00
bta-miR-6123	0.00	9.9E-01	1.0E+00
Novel:NC_037569.1_48102	-0.01	9.9E-01	1.0E+00
Novel:NC_037569.1_48106	-0.01	9.9E-01	1.0E+00
bta-miR-191b	0.00	9.9E-01	1.0E+00
bta-miR-2284b	0.01	9.9E-01	1.0E+00
bta-miR-19a	0.00	9.9E-01	1.0E+00
bta-miR-339b	0.00	1.0E+00	1.0E+00

bta-miR-301a	0.00	1.0E+00	1.0E+00
Novel:NC_037552.1_22118	0.00	1.0E+00	1.0E+00
Novel:NC_037554.1_25238	0.00	1.0E+00	1.0E+00
bta-miR-411c-5p	0.00	1.0E+00	1.0E+00

Supplementary file S5. List of differentially expressed miRNAs (DE-miRNAs) in oocytes (OOs) and follicular cells (FCs) between breeding (BS) and non-breeding (NBS) season. For each DE-miRNA. the Log Fold Change (LogFC) and the False Discovery Rate (FDR) were reported.

	**NBS**	**NBS**	**NBS**	**NBS**	**NBS**	**BS**	**BS**	**BS**	**BS**	**BS**	
Oocytes	OO1	OO2	OO3	OO4	OO5	OO6	OO7	OO8	OO9	OO1	Av
MM of input reads	22.2	26.6	25.4	27.6	32.4	23.9	20.5	18.8	16.8	20.6	23.5
MM of Unique mapped reads	20.9	25.2	23.3	25.7	30.2	22.1	19.5	17.7	15.5	19.3	22.0
Uniquely mapped reads %	94.1%	95.1%	91.5%	93.2%	93.2%	92.5%	95.1%	94.0%	92.6%	93.7%	93.5%
Follicular Cells	FC1	FC2	FC3	FC4	FC5	FC6	FC7	FC8	FC9	FC10	Av
MM of input reads	48.6	56.2	61.2	32.7	66.1	46.0	52.8	50.6	58.2	72.1	54.5
MM of Unique mapped reads	45.2	52.7	56.4	30.0	60.9	42.3	48.2	46.8	54.2	67.0	50.4
Uniquely mapped reads %	93.0%	93.8%	92.2%	91.5%	92.0%	92.0%	91.2%	92.6%	93.2%	92.9%	92.4%

Supplementary file S6. RNA-Seq statistics for oocytes (OOs) and follicular cells (FCs) in the Non-Breeding-Season (NBS) and Breeding-Season (BS). For each sample the millions of input reads. the millions of unique mapped reads and their percentage were reported.

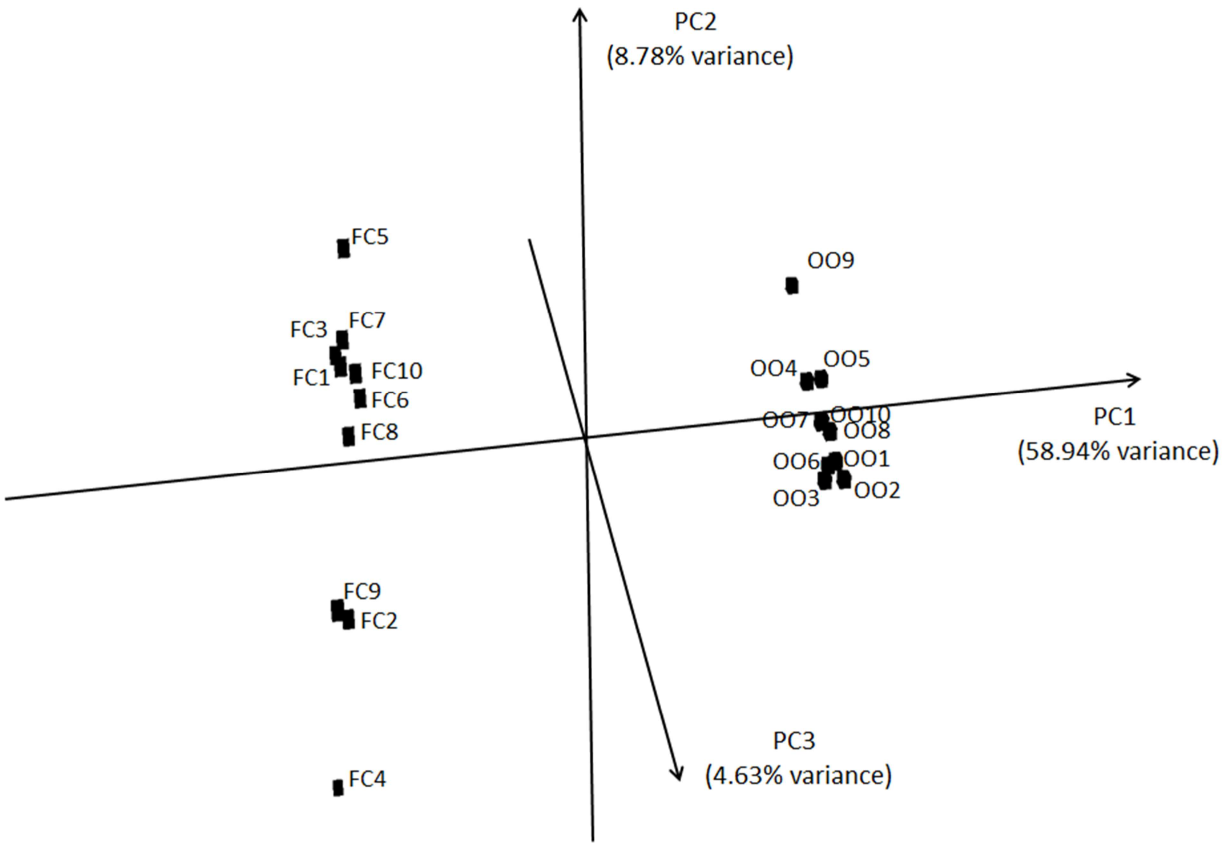

Supplementary file S7. Principal component analysis considering normalized read counts of the 22013 mRNAs expressed at least in triplicate in oocytes (OOs) or follicular cells (FCs) samples.

OO_vs_FC			OO-NBS_vs-OO_BS			FC-NBS_vs_FC-BS		
GENE_ID	logFC	FDR	GENE_ID	logFC	FDR	GENE_ID	logFC	FDR
LOC112577729	1.27E+01	0.00E+00	APOE	-4.68E+00	8.58E-07	LOC102409538	-4.70E+00	5.59E-04
LOC102390720	1.24E+01	0.00E+00	LOC102397479	-5.24E+00	1.31E-05	COL26A1	2.07E+00	2.28E-02
LOC112577758	1.19E+01	0.00E+00	PLXNA4	-2.35E+00	2.55E-05	LOC112583639	2.68E+00	1.27E-01
UNCX	1.18E+01	0.00E+00	IGF2	-4.66E+00	5.24E-04	LOC112585132	4.00E+00	5.15E-01
LOC112577650	1.16E+01	0.00E+00	FOLR2	-7.53E+00	8.01E-04	LOC102397482	1.36E+00	5.15E-01
EIF4E1B	1.15E+01	0.00E+00	CD14	-8.65E+00	8.75E-04	LOC112586997	-8.76E-01	5.15E-01
TCL1B	1.14E+01	0.00E+00	SPP1	-3.24E+00	2.45E-03	PCED1B	6.99E-01	5.15E-01
CRH	1.13E+01	0.00E+00	LOC102409999	-4.71E+00	2.76E-03	CENPA	-6.69E-01	5.15E-01
LOC102390495	1.13E+01	0.00E+00	CTSS	-5.27E+00	2.76E-03	DNAJB1	-1.35E+00	5.15E-01
LOC112581804	1.13E+01	0.00E+00	LOC102413141	-7.32E-01	3.88E-03	LRRN1	1.10E+00	5.15E-01
SPDYC	1.13E+01	0.00E+00	LOC102392787	-5.03E+00	6.10E-03	HMGCR	-9.10E-01	5.59E-01
IZUMO1R	1.12E+01	0.00E+00	LOC112581169	-9.17E-	7.64E-03	LPL	-8.37E-	5.96E-01

				01			01	
NKX2-8	1.12E+01	0.00E+00	CCL1	-1.20E+00	9.75E-03	GSN	7.98E-01	6.61E-01
ESRP1	1.12E+01	0.00E+00	CTSK	-3.84E+00	9.75E-03	GPIHBP1	-1.46E+00	8.24E-01
MOS	1.11E+01	0.00E+00	LOC102415727	-5.29E+00	1.07E-02	NAB2	5.77E-01	8.24E-01
NOBOX	1.11E+01	0.00E+00	MSR1	-3.69E+00	1.23E-02	LDLR	-6.41E-01	8.24E-01
ILDR1	1.11E+01	0.00E+00	RUNX2	-1.32E+00	1.93E-02	ICAM1	-1.99E+00	8.24E-01
LOC102402778	1.11E+01	0.00E+00	LOC102404545	2.25E+00	2.09E-02	LOC102414371	-6.81E-01	8.24E-01
LOC112580137	1.10E+01	0.00E+00	LOC102400151	-2.60E+00	2.31E-02	DSCAM	2.69E+00	8.24E-01
GDF3	1.10E+01	0.00E+00	LOC102409533	-7.78E-01	3.60E-02	LOC112578881	2.18E+00	8.33E-01
LOC102403646	1.10E+01	0.00E+00	NMB	-1.89E+00	4.72E-02	LRRTM4	2.64E+00	8.33E-01
NOTO	1.10E+01	0.00E+00	LOC112582161	8.74E-01	4.77E-02	GALNT15	1.14E+00	8.33E-01
TFAP2B	1.09E+01	0.00E+00	CEBPD	-1.93E+00	6.14E-02	ADCY8	2.01E+00	8.33E-01
LOC112581229	1.09E+01	0.00E+00	LAPTM5	-4.40E+00	6.94E-02	LMOD2	-2.17E+00	8.63E-01
LOC102392766	1.09E+01	0.00E+00	LOC102408966	-1.30E+00	7.60E-02	GDF10	1.16E+00	8.80E-01
CRB3	1.09E+01	0.00E+00	LOC112580184	-1.93E+00	7.60E-02	LOC102409347	5.76E-01	8.80E-01
LAD1	1.09E+01	0.00E+00	GIF	-2.31E+00	9.20E-02	SCARA3	6.65E-01	8.80E-01
SPATA22	1.09E+01	0.00E+00	SOX4	-1.01E+00	9.44E-02	CTNND2	2.48E+00	8.80E-01
LHX8	1.09E+01	0.00E+00	SHE	8.80E-01	1.20E-01	GRID2	2.31E+00	8.80E-01
PRSS2	1.08E+01	0.00E+00	LOC112581627	-1.39E+00	1.21E-01	SFRP5	-2.25E+00	8.80E-01
LOC112579927	1.08E+01	0.00E+00	KRT8	-1.73E+00	1.25E-01	GMPPB	-6.77E-01	8.80E-01
BSX	1.07E+01	0.00E+00	LOC112584971	-2.15E+00	1.50E-01	BCL2L11	7.87E-01	8.80E-01
LOC102390275	1.07E+01	0.00E+00	ERH	-5.07E-01	1.63E-01	SCN5A	8.12E-01	8.80E-01
BARHL2	1.07E+01	0.00E+00	SCD5	-1.36E+00	1.99E-01	ZFAND2A	-1.16E+00	8.80E-01
GTSF1	1.07E+01	0.00E+00	STAR	3.80E-01	2.09E-01	LOC102411450	-1.62E+00	8.80E-01
LOC102416591	1.07E+01	0.00E+00	OIT3	1.12E+00	2.15E-01	NSDHL	-5.11E-01	8.80E-01
DPPA5	1.06E+01	0.00E+00	ARHGDIB	-2.29E+00	2.24E-01	NTS	6.05E-01	8.80E-01
ALX1	1.05E+01	0.00E+00	CCDC186	4.14E-01	2.45E-01	LOC102409533	-1.36E+00	8.80E-01
LOC112582930	1.05E+01	0.00E+00	HDGF	-9.26E-01	2.45E-01	CRELD2	-7.28E-01	8.94E-01
LOC112582130	1.05E+01	0.00E+00	TBL1XR1	4.68E-01	3.30E-01	HPSE2	2.37E+00	9.33E-01
TCL1A	1.05E+01	0.00E+00	GRN	-1.71E+00	3.30E-01	GEM	8.64E-01	9.33E-01
DIO3	1.04E+01	0.00E+00	SPX	1.10E+00	3.36E-01	KHDRBS2	1.87E+00	9.33E-01
CKMT1A	1.04E+01	0.00E+00	CD68	-1.99E+00	3.36E-01	LOC102411446	-1.51E+00	9.33E-01
LOC112587336	1.04E+01	0.00E+00	CLDN1	-1.53E+00	3.36E-01	PDK4	8.21E-01	9.33E-01
PAX9	1.04E+01	0.00E+00	SH3BP4	-1.86E+00	3.76E-01	HERPUD1	-1.22E+00	9.45E-01

LRRIQ4	1.04E+01	0.00E+00	ZBTB7C	-2.04E+00	3.76E-01	RND1	-8.12E-01	9.67E-01
C2H2orf66	1.04E+01	0.00E+00	SAT1	-4.37E-01	3.76E-01	LOC102414504	-8.57E-01	9.67E-01
BTG4	1.03E+01	0.00E+00	ATXN1	3.73E-01	3.76E-01	KCNQ5	1.82E+00	9.72E-01
CDX2	1.03E+01	0.00E+00	SLC4A4	5.14E-01	3.76E-01	NDP	6.11E-01	9.72E-01
FOXN1	1.03E+01	0.00E+00	NKAIN1	6.33E-01	3.76E-01	CARD10	1.06E+00	9.72E-01
SALL4	1.03E+01	0.00E+00	SLC24A2	4.34E-01	3.86E-01	SLC39A8	1.02E+00	1.00E+00
POU5F1	1.03E+01	0.00E+00	CERS4	9.72E-01	4.90E-01	SMAP2	6.92E-01	1.00E+00
LOC102408379	1.03E+01	0.00E+00	TRAK2	4.93E-01	4.98E-01	SGCZ	2.30E+00	1.00E+00
SEZ6	1.02E+01	0.00E+00	CD9	-1.58E+00	4.98E-01	LOC102394332	7.88E-01	1.00E+00
LOC112582129	1.02E+01	0.00E+00	LOC112578659	5.08E-01	5.03E-01	DPP10	2.02E+00	1.00E+00
SPESP1	1.01E+01	0.00E+00	LOC102413993	-4.98E-01	5.12E-01	CAMK4	1.27E+00	1.00E+00
GATA2	1.01E+01	0.00E+00	ZC2HC1A	3.64E-01	5.22E-01	NELL1	1.73E+00	1.00E+00
NKX3-1	1.00E+01	0.00E+00	TRMT1	6.21E-01	5.39E-01	STC1	1.05E+00	1.00E+00
LOC112577799	1.00E+01	0.00E+00	COL18A1	-2.17E+00	5.82E-01	PCDH15	2.16E+00	1.00E+00
CD164L2	9.95E+00	0.00E+00	LOC112582261	6.13E-01	6.14E-01	GPRC5B	7.66E-01	1.00E+00
CRYGN	9.91E+00	0.00E+00	CDH23	3.20E-01	6.20E-01	ANO3	1.07E+00	1.00E+00
SLC7A3	9.86E+00	0.00E+00	LOC112581150	-7.09E-01	6.94E-01	LRP1B	1.91E+00	1.00E+00
LOC112580128	9.83E+00	0.00E+00	MMP19	-1.60E+00	6.94E-01	CKB	-9.94E-01	1.00E+00
DISP3	9.78E+00	0.00E+00	SP5	-4.66E-01	6.94E-01	LOC102402713	5.87E-01	1.00E+00
LOC102397898	9.77E+00	0.00E+00	LOC102393607	-2.17E+00	6.94E-01	SLC16A12	1.13E+00	1.00E+00
LOC102398169	9.76E+00	0.00E+00	SMIM14	3.93E-01	6.94E-01	FAM43A	7.86E-01	1.00E+00
PABPN1L	9.76E+00	0.00E+00	GRM5	-8.71E-01	7.12E-01	NRCAM	1.28E+00	1.00E+00
NLRP5	9.75E+00	0.00E+00	LOC112581595	-8.54E-01	7.12E-01	DHCR24	-6.57E-01	1.00E+00
LOC112579157	9.70E+00	0.00E+00	LPP	-1.50E+00	7.12E-01	THRB	-6.99E-01	1.00E+00
LOC112579487	9.64E+00	0.00E+00	COL12A1	-2.80E+00	7.12E-01	SORCS3	1.96E+00	1.00E+00
ACTL8	9.60E+00	0.00E+00	CCDC42	-4.30E-01	7.12E-01	GABRG3	1.83E+00	1.00E+00
MLN	9.57E+00	0.00E+00	HHAT	3.47E-01	7.12E-01	LOC112584434	-7.12E-01	1.00E+00
ANKRD40CL	9.56E+00	0.00E+00	GRHL3	-4.55E-01	7.30E-01	SLC30A2	1.04E+00	1.00E+00
ZGLP1	9.55E+00	0.00E+00	PIN4	-4.60E-01	7.30E-01	IGDCC4	-6.14E-01	1.00E+00
NKX6-2	9.54E+00	0.00E+00	ARV1	-1.01E+00	7.30E-01	LOC102410360	1.19E+00	1.00E+00
BCL2L10	9.54E+00	0.00E+00	INTS13	-4.50E-01	7.30E-01	NPTX2	1.11E+00	1.00E+00
GTF2A1L	9.52E+00	0.00E+00	LOC112580684	-9.62E-01	7.36E-01	ANKRD42	-5.17E-01	1.00E+00
MAEL	9.46E+00	0.00E+00	PLEKHA2	1.40E+00	7.41E-01	LOC112584668	-1.93E+00	1.00E+00
MGAT5B	9.41E+00	0.00E+00	LOC102399411	-1.00E+00	7.54E-01	CXCL12	8.99E-01	1.00E+00
ELF3	9.36E+00	0.00E+00	IL20RB	-1.63E+00	7.54E-01	ACOXL	1.51E+00	1.00E+00
LMO3	9.34E+00	0.00E+00	CXCR4	7.46E-01	7.55E-01	LOC102403881	8.86E-01	1.00E+00

CLEC4G	9.28E+00	0.00E+00	GOLGA5	-5.38E-01	7.66E-01	IGFBP5	8.28E-01	1.00E+00
LOC102412805	9.27E+00	0.00E+00	LOC112581830	-2.18E+00	7.66E-01	NKAIN2	1.98E+00	1.00E+00
ROS1	9.18E+00	0.00E+00	SULF1	-1.92E+00	8.01E-01	GADD45B	-5.38E-01	1.00E+00
CACNA2D2	9.15E+00	0.00E+00	IGFBPL1	-5.39E-01	8.08E-01	VDR	7.83E-01	1.00E+00
LOC112583470	9.10E+00	0.00E+00	RPS15A	-6.68E-01	8.40E-01	KCTD1	-5.06E-01	1.00E+00
SEC16B	9.09E+00	0.00E+00	HSPA6	-1.16E+00	8.81E-01	LHFPL3	1.76E+00	1.00E+00
LHX4	9.08E+00	0.00E+00	LOC112583966	-1.62E+00	8.99E-01	CSMD2	-5.09E-01	1.00E+00
ELFN1	9.08E+00	0.00E+00	SKP1	-4.50E-01	8.99E-01	IFITM10	7.59E-01	1.00E+00
LOC102413975	9.06E+00	0.00E+00	LOC112578122	-1.48E+00	9.15E-01	AGBL1	2.01E+00	1.00E+00
IGF2BP3	9.05E+00	0.00E+00	HENMT1	-3.39E-01	9.15E-01	TUBB2A	5.64E-01	1.00E+00
NRIP3	9.04E+00	0.00E+00	TXNIP	-8.33E-01	9.15E-01	LINGO2	1.63E+00	1.00E+00
IRF6	8.95E+00	0.00E+00	BAG4	-6.04E-01	9.15E-01	LOC102390348	9.47E-01	1.00E+00
PLEKHG7	8.92E+00	0.00E+00	SOCS3	-1.17E+00	9.15E-01	LOC102399426	8.09E-01	1.00E+00
SLC12A5	8.90E+00	0.00E+00	CCDC149	-3.84E-01	9.15E-01	CLCN2	-5.28E-01	1.00E+00
LOC102393852	8.81E+00	0.00E+00	FADS3	4.55E-01	9.15E-01	ADIPOR2	-4.81E-01	1.00E+00
HAL	8.79E+00	0.00E+00	GOLT1B	2.56E-01	9.23E-01	BMF	6.53E-01	1.00E+00
KHDC1	8.77E+00	0.00E+00	LOC102398329	-6.56E-01	9.23E-01	LOC112585150	1.89E+00	1.00E+00
LOC102394237	8.75E+00	0.00E+00	MXRA8	-1.41E+00	9.23E-01	PAK5	1.60E+00	1.00E+00
CNBD2	8.72E+00	0.00E+00	LOC112587102	1.33E+00	9.23E-01	PPIF	-4.98E-01	1.00E+00
LOC102414934	8.68E+00	0.00E+00	LOC112586799	-6.22E-01	9.23E-01	KLF9	7.99E-01	1.00E+00
LOC102391272	8.60E+00	0.00E+00	LEAP2	-7.69E-01	9.23E-01	CDH18	1.26E+00	1.00E+00
SHD	8.59E+00	0.00E+00	CITED2	3.41E-01	9.23E-01	FSTL4	1.23E+00	1.00E+00
LOC102394362	8.54E+00	0.00E+00	DUSP14	4.81E-01	9.23E-01	GRM5	2.03E+00	1.00E+00
ARHGEF5	8.53E+00	0.00E+00	CNN2	-1.42E+00	9.23E-01	NRXN1	1.65E+00	1.00E+00
LOC102407026	8.52E+00	0.00E+00	LOC112580303	-1.00E+00	9.43E-01	RBFOX1	1.76E+00	1.00E+00
LOC102390572	8.51E+00	0.00E+00	FAM126B	4.02E-01	9.43E-01	VWA1	-5.80E-01	1.00E+00
DUSP13	8.50E+00	0.00E+00	DOK4	-2.89E-01	9.43E-01	SYBU	-1.18E+00	1.00E+00
LMX1B	8.46E+00	0.00E+00	CTSF	5.23E-01	9.43E-01	NKAIN3	1.26E+00	1.00E+00
TKTL1	8.43E+00	0.00E+00	OLFML1	7.13E-01	9.70E-01	HYOU1	-5.36E-01	1.00E+00
HMX2	8.43E+00	0.00E+00	KCNS3	1.43E+00	9.70E-01	MGAT5	7.01E-01	1.00E+00
SLA2	8.41E+00	0.00E+00	DBF4B	3.04E-01	9.93E-01	CEMIP2	6.94E-01	1.00E+00
SLC5A1	8.40E+00	0.00E+00	CACNA2D3	-9.75E-01	9.93E-01	ADAMTSL3	1.52E+00	1.00E+00
GOLGA7B	8.36E+00	0.00E+00	ASPHD1	-6.74E-01	9.93E-01	HIC1	8.77E-01	1.00E+00
SLC6A4	8.34E+00	0.00E+00	TNFRSF1B	-9.20E-01	9.93E-01	ZNF385B	6.05E-01	1.00E+00

BCL6B	8.26E+00	0.00E+00	STK38L	-9.22E-01	9.93E-01	CNTN5	1.66E+00	1.00E+00
AP1M2	8.20E+00	0.00E+00	LOC112582978	-5.68E-01	9.93E-01	TROAP	-5.11E-01	1.00E+00
C9H19orf67	8.19E+00	0.00E+00	SNAI3	-5.47E-01	9.93E-01	ANXA13	1.16E+00	1.00E+00
TMEM92	8.14E+00	0.00E+00	AHNAK	-1.29E+00	9.93E-01	CTNNA3	1.40E+00	1.00E+00
MOGAT1	8.07E+00	0.00E+00	RNF2	3.08E-01	9.93E-01	CFAP299	1.85E+00	1.00E+00
TDRD5	8.05E+00	0.00E+00	CTSH	-1.25E+00	9.93E-01	ITIH5	7.73E-01	1.00E+00
PDE6C	8.04E+00	0.00E+00	LOC102402708	-4.27E-01	9.93E-01	LOC112587361	1.16E+00	1.00E+00
LMOD3	7.99E+00	0.00E+00	DNAJA4	-3.80E-01	9.93E-01	HSPA5	-1.04E+00	1.00E+00
COCH	7.99E+00	0.00E+00	LOC112585883	-7.93E-01	9.93E-01	LOC102404666	-1.14E+00	1.00E+00
LMO1	7.92E+00	0.00E+00	PPFIA3	8.29E-01	9.93E-01	NTM	1.69E+00	1.00E+00
RASD2	7.91E+00	0.00E+00	LOC112580305	-8.20E-01	9.93E-01	LUZP2	1.84E+00	1.00E+00
EBF3	7.82E+00	0.00E+00	DCLK1	-1.45E+00	9.93E-01	CSMD3	1.39E+00	1.00E+00
CARMIL2	7.78E+00	0.00E+00	GAS1	-1.30E+00	9.93E-01	NCS1	7.18E-01	1.00E+00
SBK2	7.72E+00	0.00E+00	FBXO33	4.07E-01	9.93E-01	LOC112579418	1.19E+00	1.00E+00
CCNO	7.68E+00	0.00E+00	GAL3ST1	6.40E-01	1.00E+00	GUCY1A2	1.67E+00	1.00E+00
BSPRY	7.57E+00	0.00E+00	COTL1	-7.60E-01	1.00E+00	MERTK	6.85E-01	1.00E+00
AARD	7.52E+00	0.00E+00	UBTD2	4.22E-01	1.00E+00	SLC7A5	-5.19E-01	1.00E+00
SPNS3	7.51E+00	0.00E+00	LLGL1	4.29E-01	1.00E+00	THSD7B	1.64E+00	1.00E+00
DEPDC7	7.47E+00	0.00E+00	CACNA1E	2.72E-01	1.00E+00	LOC102406481	-5.92E-01	1.00E+00
RHEBL1	7.45E+00	0.00E+00	KCNK10	-1.23E+00	1.00E+00	KCNK3	8.75E-01	1.00E+00
HEY1	7.40E+00	0.00E+00	LOC112582053	-7.54E-01	1.00E+00	FSTL5	2.02E+00	1.00E+00
PRR7	7.38E+00	0.00E+00	LOC112579143	-5.44E-01	1.00E+00	FAM19A2	1.70E+00	1.00E+00
LOC102394468	7.29E+00	0.00E+00	LOC112586864	-1.57E+00	1.00E+00	HOPX	5.32E-01	1.00E+00
USP44	7.27E+00	0.00E+00	RASL12	-1.00E+00	1.00E+00	FILIP1	5.79E-01	1.00E+00
PPL	7.15E+00	0.00E+00	ZFP36	-7.13E-01	1.00E+00	KCNG4	-9.58E-01	1.00E+00
EPHA10	7.12E+00	0.00E+00	IFIT5	-1.21E+00	1.00E+00	NAV3	1.61E+00	1.00E+00
C18H16orf74	7.10E+00	0.00E+00	ZFP36L1	4.95E-01	1.00E+00	VWA5B2	1.19E+00	1.00E+00
SP6	7.03E+00	0.00E+00	LOC102400616	3.88E-01	1.00E+00	DOK7	-8.27E-01	1.00E+00
LOC102404032	6.86E+00	0.00E+00	NUDT13	4.45E-01	1.00E+00	AGBL4	1.63E+00	1.00E+00
SULT4A1	6.81E+00	0.00E+00	COCH	2.73E-01	1.00E+00	STPG2	1.91E+00	1.00E+00
SKOR1	6.79E+00	0.00E+00	WDR73	-6.94E-01	1.00E+00	LOC102403707	-5.59E-01	1.00E+00
MYOZ3	6.77E+00	0.00E+00	LOC102401424	1.75E+00	1.00E+00	ADPRHL2	5.46E-01	1.00E+00
ICA1L	6.67E+00	0.00E+00	EYA4	-1.02E+00	1.00E+00	LOC102398382	5.88E-01	1.00E+00
GRAMD2A	6.63E+00	0.00E+00	RRAD	-1.84E+00	1.00E+00	DLL4	-1.13E+00	1.00E+00
GLS2	6.60E+00	0.00E+00	CST6	-3.94E-01	1.00E+00	HDAC9	1.29E+00	1.00E+00

SEMA3G	6.60E+00	0.00E+00	ADAMTS2	-6.55E-01	1.00E+00	USP40	-4.32E-01	1.00E+00
RASGEF1A	6.56E+00	0.00E+00	ACVR1B	-7.46E-01	1.00E+00	GPAT3	-5.58E-01	1.00E+00
CCDC181	6.43E+00	0.00E+00	MFSD4B	4.27E-01	1.00E+00	LOC102392650	1.31E+00	1.00E+00
STRIP2	6.35E+00	0.00E+00	MPIG6B	2.60E-01	1.00E+00	EPHA6	1.38E+00	1.00E+00
MRVI1	6.07E+00	0.00E+00	LOC102403423	-7.13E-01	1.00E+00	LAMA5	6.67E-01	1.00E+00
DOP1B	6.06E+00	0.00E+00	LACC1	3.75E-01	1.00E+00	GRM7	1.44E+00	1.00E+00
EMP2	-5.91E+00	0.00E+00	LOC102413915	-4.00E-01	1.00E+00	LOC102410999	-1.39E+00	1.00E+00
XKRX	5.80E+00	0.00E+00	LOC102402769	5.33E-01	1.00E+00	GAL	8.48E-01	1.00E+00
RPH3AL	5.75E+00	0.00E+00	HAT1	-2.84E-01	1.00E+00	DES	9.10E-01	1.00E+00
KLHL25	5.72E+00	0.00E+00	PLEKHG3	3.72E-01	1.00E+00	RNF123	-3.64E-01	1.00E+00
AP3M2	5.52E+00	0.00E+00	LGALS3	-5.45E-01	1.00E+00	PMEPA1	5.99E-01	1.00E+00
MYO5B	5.49E+00	0.00E+00	MX1	-2.07E+00	1.00E+00	SEMA6B	7.54E-01	1.00E+00
PPP1R13B	5.18E+00	0.00E+00	KIF12	-1.27E+00	1.00E+00	PITX1	6.56E-01	1.00E+00
BPGM	5.17E+00	0.00E+00	FAM78A	-9.01E-01	1.00E+00	GAS6	5.95E-01	1.00E+00
RAB15	5.01E+00	0.00E+00	LOC112581246	-6.82E-01	1.00E+00	FAT3	1.82E+00	1.00E+00
MAPRE2	4.95E+00	0.00E+00	LOC112586457	-4.69E-01	1.00E+00	PFKFB4	-4.11E-01	1.00E+00
TMEM150A	4.71E+00	0.00E+00	COG4	3.75E-01	1.00E+00	THBS2	8.18E-01	1.00E+00
GCC1	4.67E+00	0.00E+00	ADGRD1	-8.12E-01	1.00E+00	PODNL1	-6.10E-01	1.00E+00
FAM117A	4.66E+00	0.00E+00	P2RX3	1.10E+00	1.00E+00	ZNF385D	1.40E+00	1.00E+00
LSM11	4.60E+00	0.00E+00	LOC102403866	-4.10E-01	1.00E+00	SGCD	9.22E-01	1.00E+00
TMEM120B	4.58E+00	0.00E+00	MAF	-2.04E+00	1.00E+00	RIMS1	1.59E+00	1.00E+00
TTC9C	4.15E+00	0.00E+00	SMYD4	4.48E-01	1.00E+00	RAB27B	5.08E-01	1.00E+00
STK39	4.04E+00	0.00E+00	LOC102391229	4.62E-01	1.00E+00	WNT5A	7.53E-01	1.00E+00
SLC45A4	3.99E+00	0.00E+00	ERICH6B	1.30E+00	1.00E+00	HSPH1	-6.66E-01	1.00E+00
LOC102405452	1.15E+01	4.59481050632359e-322	ARHGEF17	-7.38E-01	1.00E+00	NUPL2	-4.96E-01	1.00E+00
GAD1	1.01E+01	4.59481050632359e-322	LOC112580215	-6.07E-01	1.00E+00	HS3ST1	-5.54E-01	1.00E+00
LOC102414190	1.07E+01	1.36362118252184e-321	XIAP	3.45E-01	1.00E+00	CACNA1B	7.05E-01	1.00E+00
LOC112580349	5.96E+00	1.26530211899943e-320	WDTC1	3.92E-01	1.00E+00	S100A9	-1.88E+00	1.00E+00
GPAT2	7.35E+00	3.14621003271706e-320	PUS10	6.35E-01	1.00E+00	ADGRL3	1.35E+00	1.00E+00
CIART	5.48E+00	4.91644724176624e-320	NSMCE4A	-2.53E-01	1.00E+00	CDKN2B	1.04E+00	1.00E+00
CCDC87	5.85E+00	2.74236077380642e-319	ITGB2	-1.23E+00	1.00E+00	DOK6	1.55E+00	1.00E+00
TRPV6	8.54E+00	3.9459639749532e-318	LOC102389065	1.43E+00	1.00E+00	LOC102414025	1.37E+00	1.00E+00
LOC112581281	1.00E+01	1.49867600307513e-317	METTL22	-2.86E-01	1.00E+00	FAM118A	-4.81E-01	1.00E+00
GDAP2	3.62E+00	1.13306366531304e-315	SYNPO	7.89E-01	1.00E+00	MAGI2	1.24E+00	1.00E+00
DDX25	5.84E+00	2.18894849617762e-315	RFPL4A	-8.94E-01	1.00E+00	SLC22A18	-9.88E-01	1.00E+00

ZNF541	1.02E+01	6.70011226179871e-314	GXYLT1	3.93E-01	1.00E+00	TMEM37	8.01E-01	1.00E+00
SEPT3	6.63E+00	1.28679653469348e-313	ACP4	-5.45E-01	1.00E+00	RAB3C	1.39E+00	1.00E+00
TDRD10	8.24E+00	2.20702717707276e-313	SMAD3	3.07E-01	1.00E+00	TMEM26	9.20E-01	1.00E+00
MTUS1	4.65E+00	5.35820393181773e-313	GLUL	3.40E-01	1.00E+00	EDEM1	-4.45E-01	1.00E+00
LOC112580240	1.07E+01	2.44385843661027e-312	LOC112578527	-1.91E+00	1.00E+00	GALNT12	-5.70E-01	1.00E+00
ATP6V0A4	5.68E+00	1.00097397726986e-311	PIF1	-1.47E+00	1.00E+00	LOC112578878	-4.90E-01	1.00E+00
ARHGEF16	6.12E+00	1.00309768770132e-311	CAMSAP1	3.44E-01	1.00E+00	DAB1	8.47E-01	1.00E+00
LOC102400872	1.02E+01	0.00E+00	LINGO2	-2.98E-01	1.00E+00	FHL3	6.23E-01	1.00E+00
SENP8	5.29E+00	0.00E+00	LAS1L	3.14E-01	1.00E+00	TTC29	1.14E+00	1.00E+00
C2H6orf52	1.08E+01	2.56E-308	TGFBI	-5.35E-01	1.00E+00	GAS1	5.80E-01	1.00E+00
RGS8	9.19E+00	1.35E-306	KIAA1671	-7.91E-01	1.00E+00	COL12A1	6.98E-01	1.00E+00
MPZL3	4.43E+00	2.44E-306	TOP2B	3.64E-01	1.00E+00	DPYSL5	8.93E-01	1.00E+00
BNC1	8.56E+00	3.19E-306	BCHE	-1.14E+00	1.00E+00	GFOD1	1.09E+00	1.00E+00
SALL3	1.04E+01	2.48E-305	PADI1	-1.05E+00	1.00E+00	LFNG	5.78E-01	1.00E+00
KIAA1147	6.71E+00	1.37E-304	SLA2	-3.26E-01	1.00E+00	LOC102415074	5.97E-01	1.00E+00
CASTOR1	5.76E+00	6.76E-304	ZNF112	-7.54E-01	1.00E+00	PLPP7	4.53E-01	1.00E+00
LAMB4	4.53E+00	7.27E-304	HES1	-6.32E-01	1.00E+00	HMCN1	-5.96E-01	1.00E+00
ADORA2B	6.65E+00	7.75E-303	CALD1	-4.94E-01	1.00E+00	DMKN	7.56E-01	1.00E+00
RDH12	8.03E+00	1.81E-301	NRAS	2.89E-01	1.00E+00	LOC102399763	1.19E+00	1.00E+00
NIF3L1	3.93E+00	7.93E-301	FPGT	-9.03E-01	1.00E+00	IL1RAPL2	1.52E+00	1.00E+00
SMURF1	4.96E+00	2.21E-300	MANSC1	7.99E-01	1.00E+00	LOC102409414	-4.42E-01	1.00E+00
LOC102395770	7.00E+00	3.02E-300	TESC	-3.13E-01	1.00E+00	TMEM86A	6.32E-01	1.00E+00
LIN7A	7.13E+00	2.89E-299	ALDH6A1	-1.11E+00	1.00E+00	ABAT	-6.22E-01	1.00E+00
LOC112580810	8.64E+00	3.62E-297	HSD17B11	-1.12E+00	1.00E+00	INSIG1	-7.07E-01	1.00E+00
GPLD1	6.84E+00	5.26E-296	GMNC	1.59E+00	1.00E+00	ALPL	-7.24E-01	1.00E+00
NCAN	9.39E+00	9.87E-296	MBOAT2	-3.90E-01	1.00E+00	LOC112585091	1.09E+00	1.00E+00
DOK4	5.49E+00	1.32E-295	LOC102396801	4.74E-01	1.00E+00	NDRG2	-3.43E-01	1.00E+00
PNOC	8.35E+00	2.59E-295	LOC102404534	-6.70E-01	1.00E+00	LOC112582101	7.86E-01	1.00E+00
LOC112580807	7.36E+00	3.27E-295	ATXN2L	4.35E-01	1.00E+00	DISC1	1.40E+00	1.00E+00
TUBG2	5.19E+00	3.33E-295	UEVLD	4.60E-01	1.00E+00	WNT7B	1.06E+00	1.00E+00
LOC102400552	7.18E+00	3.46E-295	HSPD1	-2.51E-01	1.00E+00	TTC7A	-5.82E-01	1.00E+00
LOC102409898	7.88E+00	4.92E-295	MARCKS	-1.63E+00	1.00E+00	TNFRSF12A	6.94E-01	1.00E+00
DRC7	7.22E+00	1.04E-294	SAP25	-7.01E-01	1.00E+00	PTPN5	6.57E-01	1.00E+00
PNLDC1	6.86E+00	1.48E-294	CPSF2	-3.06E-01	1.00E+00	INSL3	-6.79E-01	1.00E+00

ARL14EPL	8.08E+00	2.00E-294	STARD13	-9.61E-01	1.00E+00	DMD	6.44E-01	1.00E+00
CLP1	5.32E+00	2.31E-294	MIEF1	2.60E-01	1.00E+00	SDS	-1.70E+00	1.00E+00
SLC1A4	6.17E+00	1.54E-293	CLDN12	3.75E-01	1.00E+00	HSPA6	-1.16E+00	1.00E+00
CDYL	4.46E+00	9.76E-293	LOC102407804	-1.54E+00	1.00E+00	LOC102397920	-1.90E+00	1.00E+00
ODF4	7.38E+00	3.74E-292	HERC3	3.30E-01	1.00E+00	ARHGAP36	-9.17E-01	1.00E+00
LOC102395312	1.10E+01	1.69E-291	HRG	-7.31E-01	1.00E+00	CA10	1.45E+00	1.00E+00
CABLES2	3.51E+00	1.89E-290	U2SURP	-3.36E-01	1.00E+00	LOC102399486	-4.84E-01	1.00E+00
TRIM50	5.58E+00	9.06E-290	MARCH5	-3.24E-01	1.00E+00	HOMER3	4.74E-01	1.00E+00
CBLL1	4.79E+00	1.75E-289	IAH1	-3.53E-01	1.00E+00	LOC102399418	-1.08E+00	1.00E+00
LOC112586867	1.20E+01	2.25E-289	LOC112579592	1.07E+00	1.00E+00	RASD1	-7.41E-01	1.00E+00
MYO5C	5.93E+00	4.07E-289	LOC102397551	-1.62E+00	1.00E+00	CXCL8	-2.33E+00	1.00E+00
LIN28A	1.06E+01	9.48E-289	GRPEL2	2.46E-01	1.00E+00	COL21A1	5.72E-01	1.00E+00
LOC102412587	6.21E+00	4.98E-288	LOC102398516	-5.20E-01	1.00E+00	CCSER1	1.27E+00	1.00E+00
HSD11B2	9.39E+00	6.44E-287	ZNF598	2.35E-01	1.00E+00	C4H12orf75	7.35E-01	1.00E+00
ATMIN	3.32E+00	2.30E-286	LOC102395590	6.92E-01	1.00E+00	NOX4	7.92E-01	1.00E+00
TACSTD2	6.99E+00	6.44E-286	LOC112582139	2.73E-01	1.00E+00	GRM8	1.60E+00	1.00E+00
LOC102395384	5.84E+00	4.28E-285	USP31	3.08E-01	1.00E+00	FAM19A4	-6.06E-01	1.00E+00
PRR19	5.69E+00	5.75E-285	TNS2	3.77E-01	1.00E+00	ESRRG	1.64E+00	1.00E+00
LOC112586834	9.64E+00	1.40E-283	JUNB	-1.46E+00	1.00E+00	CAPS	-9.35E-01	1.00E+00
SLC6A20	6.28E+00	3.62E-283	C15H8orf33	-5.13E-01	1.00E+00	MICALL2	6.21E-01	1.00E+00
INA	8.94E+00	9.74E-281	ANKRD16	-4.96E-01	1.00E+00	S100A14	6.75E-01	1.00E+00
C16H11orf16	5.53E+00	1.54E-279	ATG2A	-4.32E-01	1.00E+00	ACSL3	-3.67E-01	1.00E+00
LOC102389932	5.42E+00	2.92E-279	ATF3	-8.07E-01	1.00E+00	ARHGAP33	-5.11E-01	1.00E+00
DBX1	9.09E+00	1.77E-278	ALYREF	-3.26E-01	1.00E+00	WHRN	-7.31E-01	1.00E+00
TUBD1	4.14E+00	5.15E-278	FHOD3	2.68E-01	1.00E+00	CKMT2	-7.57E-01	1.00E+00
LOC112582989	1.07E+01	1.44E-277	ANKS4B	6.32E-01	1.00E+00	ALDH1L1	-9.21E-01	1.00E+00
ARMC2	5.66E+00	2.17E-277	HOXC10	-1.32E+00	1.00E+00	TINAG	1.83E+00	1.00E+00
LRRC8E	4.13E+00	2.59E-277	HSF2	5.36E-01	1.00E+00	PRKN	1.10E+00	1.00E+00
LOC102414381	9.23E+00	2.95E-277	FAM160B1	4.72E-01	1.00E+00	CTBP1	4.07E-01	1.00E+00
SOX30	8.42E+00	1.43E-276	LOC102414988	-5.10E-01	1.00E+00	ATP8A2	-5.26E-01	1.00E+00
STK31	3.69E+00	4.12E-276	CPNE5	-1.06E+00	1.00E+00	FSTL3	6.98E-01	1.00E+00
MAPK8IP2	5.60E+00	4.25E-276	GOPC	3.72E-01	1.00E+00	SRSF2	-4.18E-01	1.00E+00
DIRAS2	9.49E+00	1.46E-275	SLC25A43	-1.49E+00	1.00E+00	EFEMP2	4.46E-01	1.00E+00
RIIAD1	5.36E+00	2.03E-275	LOC112580369	6.13E-01	1.00E+00	KCNQ1	-6.78E-01	1.00E+00
WDR6	-	7.74E-275	CFAP100	-	1.00E+00	MTUS2	1.35E+00	1.00E+00

	4.68E+00			1.04E+00				
RAB3IP	3.86E+00	8.68E-275	ACTG2	-1.38E+00	1.00E+00	OPCML	1.71E+00	1.00E+00
LOC112586636	1.02E+01	1.27E-274	ADGRV1	3.16E-01	1.00E+00	SEMA7A	-5.69E-01	1.00E+00
LOC102413407	7.07E+00	1.33E-274	SCAF4	3.81E-01	1.00E+00	DCC	1.42E+00	1.00E+00
NEIL3	5.48E+00	3.49E-274	XKRX	2.43E-01	1.00E+00	MAP2	7.40E-01	1.00E+00
BRDT	8.31E+00	4.04E-274	CDH11	-1.30E+00	1.00E+00	SGK1	7.58E-01	1.00E+00
SHROOM2	4.50E+00	1.01E-273	ANKLE2	3.31E-01	1.00E+00	BANK1	1.15E+00	1.00E+00
PPA1	4.10E+00	3.42E-273	PHOSPHO1	-5.49E-01	1.00E+00	NF2	3.95E-01	1.00E+00
PALM3	7.94E+00	4.13E-273	APBB1IP	-8.33E-01	1.00E+00	LOC102403234	-6.12E-01	1.00E+00
LOC102400432	6.65E+00	8.42E-273	TBX19	3.98E-01	1.00E+00	VILL	-5.96E-01	1.00E+00
CCDC65	5.54E+00	1.58E-272	LOC112582163	-1.51E+00	1.00E+00	CTXN1	5.85E-01	1.00E+00
CROT	3.92E+00	3.86E-271	ANKRD1	-1.50E+00	1.00E+00	MAOB	6.51E-01	1.00E+00
NEIL2	4.23E+00	3.91E-271	OGDHL	3.77E-01	1.00E+00	ACSS1	-4.84E-01	1.00E+00
LOC102409332	3.42E+00	7.59E-271	VWA1	-1.88E+00	1.00E+00	LOC102411322	-1.07E+00	1.00E+00
LOC112577713	6.17E+00	6.41E-270	LOC102412899	-7.39E-01	1.00E+00	PHLDB2	8.20E-01	1.00E+00
LOC102398488	4.43E+00	6.97E-270	CAVIN1	-7.93E-01	1.00E+00	DPF3	-4.53E-01	1.00E+00
ARHGEF33	5.29E+00	1.61E-269	ABRA	-9.27E-01	1.00E+00	PDE10A	1.29E+00	1.00E+00
EPOP	5.64E+00	3.26E-269	C9H19orf71	9.87E-01	1.00E+00	ITIH2	8.71E-01	1.00E+00
PRDM6	5.87E+00	4.80E-269	LOC112586243	-3.10E-01	1.00E+00	ACACB	-5.23E-01	1.00E+00
SOHLH1	9.80E+00	5.05E-269	ASCC3	-3.50E-01	1.00E+00	LOC102414136	-4.20E-01	1.00E+00
TMEM51	4.99E+00	1.16E-268	FHAD1	-5.29E-01	1.00E+00	SKP1	-5.44E-01	1.00E+00
WIF1	6.81E+00	1.41E-268	NCBP2-AS2	6.57E-01	1.00E+00	LOC102415899	-1.46E+00	1.00E+00
APH1B	4.77E+00	3.68E-268	NDC1	2.42E-01	1.00E+00	SELP	7.55E-01	1.00E+00
ASIC1	6.87E+00	4.98E-268	HOOK1	4.12E-01	1.00E+00	LOC102396820	-7.09E-01	1.00E+00
LOC112585225	9.30E+00	9.99E-268	PHLDA2	-9.88E-01	1.00E+00	SLC27A3	-7.00E-01	1.00E+00
TRPV1	9.35E+00	1.75E-267	SKAP1	-6.06E-01	1.00E+00	B3GNT2	-3.89E-01	1.00E+00
FKBP6	7.01E+00	2.10E-267	MOV10	-4.46E-01	1.00E+00	MDGA2	1.45E+00	1.00E+00
NLRP2	1.08E+01	2.26E-267	GAR1	-2.26E-01	1.00E+00	IRF1	-1.37E+00	1.00E+00
FGF16	9.21E+00	2.09E-265	PRDX3	-2.73E-01	1.00E+00	MFSD2A	-6.16E-01	1.00E+00
LOC112586819	1.01E+01	6.71E-265	AATK	4.28E-01	1.00E+00	PDP2	-3.95E-01	1.00E+00
LOC102393230	6.49E+00	1.12E-264	LOC102393741	4.99E-01	1.00E+00	OXTR	8.97E-01	1.00E+00
TINF2	3.46E+00	2.69E-264	LOC112579141	-4.60E-01	1.00E+00	ASS1	-6.14E-01	1.00E+00
MAJIN	8.16E+00	3.79E-264	SPA17	-6.26E-01	1.00E+00	TMEM117	6.77E-01	1.00E+00
SH2B3	4.19E+00	3.88E-264	LOC102390908	8.21E-01	1.00E+00	KCNH1	1.12E+00	1.00E+00
PRKCH	5.97E+00	1.17E-263	KIF21B	-1.19E+00	1.00E+00	LOC112585187	4.16E-01	1.00E+00

NSG1	5.13E+00	3.48E-263	LOC112581758	-9.09E-01	1.00E+00	LRG1	1.10E+00	1.00E+00
PLD6	8.42E+00	8.65E-262	NMI	-1.36E+00	1.00E+00	EXTL1	8.04E-01	1.00E+00
KCNN3	9.87E+00	1.04E-261	C4H12orf54	-1.33E+00	1.00E+00	NUP210	-3.92E-01	1.00E+00
DGAT2	7.10E+00	2.57E-261	LOC102392935	-1.26E+00	1.00E+00	ENPEP	-8.14E-01	1.00E+00
RAD17	3.38E+00	4.40E-261	NLRP1	2.56E-01	1.00E+00	FBN2	9.13E-01	1.00E+00
DND1	4.55E+00	6.37E-261	ANXA2	-5.90E-01	1.00E+00	WISP1	1.06E+00	1.00E+00
PLA2G4D	9.64E+00	7.64E-261	PIGY	-2.71E-01	1.00E+00	ABCC1	-3.47E-01	1.00E+00
OGDHL	9.14E+00	3.00E-260	NLK	2.36E-01	1.00E+00	FRMPD4	1.32E+00	1.00E+00
RSPO2	8.74E+00	9.40E-260	FMO2	-5.45E-01	1.00E+00	CDH8	6.96E-01	1.00E+00
SLC8B1	4.56E+00	2.79E-259	DR1	4.65E-01	1.00E+00	LOC102410939	-3.56E-01	1.00E+00
LOC102403713	6.97E+00	3.37E-259	SMG7	3.13E-01	1.00E+00	ICOSLG	6.27E-01	1.00E+00
LLGL2	5.42E+00	9.01E-259	NDST1	2.53E-01	1.00E+00	PCP4	1.16E+00	1.00E+00
DRD1	7.57E+00	4.78E-257	LOC112583734	4.28E-01	1.00E+00	CHRM5	6.69E-01	1.00E+00
ZNF783	3.94E+00	8.55E-257	NUDT15	2.35E-01	1.00E+00	WNT6	-8.83E-01	1.00E+00
KIAA0895L	4.31E+00	1.43E-255	GAREM1	-4.77E-01	1.00E+00	CCDC192	1.14E+00	1.00E+00
OSBPL10	4.33E+00	2.49E-255	LOC102408905	-5.63E-01	1.00E+00	LOC102415247	-1.94E+00	1.00E+00
LOC112583539	1.04E+01	2.01E-254	LOC102391413	-1.04E+00	1.00E+00	PRPF38B	-3.79E-01	1.00E+00
TEK	6.80E+00	2.13E-254	SPERT	-6.87E-01	1.00E+00	KIF14	-6.07E-01	1.00E+00
RWDD2A	4.30E+00	1.97E-253	SORT1	2.67E-01	1.00E+00	SERPINA1	6.61E-01	1.00E+00
LOC102398506	5.17E+00	2.62E-253	SV2B	1.65E+00	1.00E+00	STK10	5.92E-01	1.00E+00
MBD4	3.18E+00	1.23E-252	LAMB1	-4.61E-01	1.00E+00	LRRC4C	1.36E+00	1.00E+00
HNF1B	6.60E+00	2.45E-252	ARRDC3	-5.17E-01	1.00E+00	LYVE1	1.36E+00	1.00E+00
APOB	9.66E+00	4.65E-252	MAPT	-4.49E-01	1.00E+00	PLCD3	4.57E-01	1.00E+00
APOA2	9.07E+00	1.17E-251	PDE3A	2.93E-01	1.00E+00	SCML1	-5.41E-01	1.00E+00
FZD4	4.75E+00	1.39E-250	EFNA4	-7.79E-01	1.00E+00	HTR1B	-9.51E-01	1.00E+00
SLC6A5	6.73E+00	2.07E-250	CMIP	2.92E-01	1.00E+00	LRP11	-4.91E-01	1.00E+00
NPM2	1.10E+01	3.69E-250	MFAP2	1.36E+00	1.00E+00	ZNF624	-4.50E-01	1.00E+00
LOC102415842	1.11E+01	4.60E-250	TM9SF4	3.05E-01	1.00E+00	KRT18	7.88E-01	1.00E+00
PEX12	3.37E+00	8.14E-250	SCN4A	3.46E-01	1.00E+00	RND3	7.16E-01	1.00E+00
NUP35	3.63E+00	8.66E-249	PTBP3	3.93E-01	1.00E+00	CCDC178	1.11E+00	1.00E+00
TBC1D7	3.95E+00	1.08E-248	SPATA24	-4.05E-01	1.00E+00	CPEB1	-3.43E-01	1.00E+00
STAP2	6.23E+00	1.52E-248	GNS	2.43E-01	1.00E+00	SCARA5	5.82E-01	1.00E+00
FBXL4	3.30E+00	2.12E-248	KIF18B	3.28E-01	1.00E+00	SLC9A9	1.05E+00	1.00E+00
NLRP14	1.13E+01	1.09E-247	NDRG4	2.08E-01	1.00E+00	LMCD1	6.85E-01	1.00E+00
S100Z	9.81E+00	2.97E-247	RPL18A	-2.78E-01	1.00E+00	NUAK1	8.12E-01	1.00E+00
TMEM52	7.40E+00	5.02E-247	NSL1	3.69E-01	1.00E+00	LIMCH1	5.65E-01	1.00E+00
RELT	4.24E+00	5.96E-247	LOC102408708	-	1.00E+00	GPC5	7.43E-01	1.00E+00

				1.28E+00				
AZIN2	6.81E+00	2.82E-246	LOC102413789	-7.78E-01	1.00E+00	MAP7D1	3.91E-01	1.00E+00
TTC24	8.90E+00	6.71E-246	RDX	2.78E-01	1.00E+00	LZTS1	3.93E-01	1.00E+00
TENT5B	9.30E+00	7.50E-246	LOC112587339	-1.21E+00	1.00E+00	IER5L	6.23E-01	1.00E+00
FIGLA	1.06E+01	8.49E-245	LOC102398407	-1.02E+00	1.00E+00	ORAI2	5.46E-01	1.00E+00
DTX2	3.84E+00	9.90E-245	PLXDC2	-3.73E-01	1.00E+00	LPCAT3	-4.04E-01	1.00E+00
CYFIP2	4.10E+00	5.47E-244	KIAA2012	-7.06E-01	1.00E+00	LOC112580319	-9.38E-01	1.00E+00
AMIGO1	4.28E+00	9.86E-244	FH	2.16E-01	1.00E+00	FICD	-5.64E-01	1.00E+00
RGS16	1.01E+01	2.33E-243	LOC102403333	2.54E-01	1.00E+00	LOC102398455	6.11E-01	1.00E+00
PLOD3	-4.32E+00	3.55E-243	DDX25	-2.17E-01	1.00E+00	HIVEP2	7.45E-01	1.00E+00
ADAM20	7.60E+00	1.39E-242	SH2D2A	-2.31E-01	1.00E+00	LOC112580806	8.31E-01	1.00E+00
AUNIP	3.70E+00	3.28E-242	USP13	3.66E-01	1.00E+00	CCL24	-1.61E+00	1.00E+00
INPP5F	4.52E+00	2.74E-241	SAMD8	2.99E-01	1.00E+00	GNRHR	-7.07E-01	1.00E+00
CNTD2	5.84E+00	4.78E-241	SUPT7L	2.67E-01	1.00E+00	CDC42EP4	3.98E-01	1.00E+00
PLEKHM3	5.70E+00	1.03E-240	LOC112581828	1.04E+00	1.00E+00	ABRACL	4.78E-01	1.00E+00
ARHGEF26	5.92E+00	1.49E-240	SLC22A4	-8.04E-01	1.00E+00	ACAT2	-4.35E-01	1.00E+00
UBASH3A	7.55E+00	4.24E-240	TMEM132D	1.79E+00	1.00E+00	IGFBP3	6.65E-01	1.00E+00
LOC112578659	1.01E+01	1.25E-239	TCERG1	3.66E-01	1.00E+00	DHCR7	-3.79E-01	1.00E+00
MTA2	-5.76E+00	1.69E-239	C3H17orf75	-3.69E-01	1.00E+00	TGFB2	8.96E-01	1.00E+00
GDF9	1.06E+01	1.95E-239	LOC102402191	-7.06E-01	1.00E+00	ROBO4	-8.37E-01	1.00E+00
LRRC31	8.77E+00	5.83E-239	LOC102399593	-1.24E+00	1.00E+00	LOC102406860	-6.18E-01	1.00E+00
POLE2	3.73E+00	8.07E-239	ANKEF1	-1.14E+00	1.00E+00	LOC112585749	-6.40E-01	1.00E+00
SAMD12	4.37E+00	1.40E-238	NTF4	-1.22E+00	1.00E+00	FLNB	4.79E-01	1.00E+00
ZCWPW1	4.32E+00	4.71E-238	BFSP2	-8.43E-01	1.00E+00	CRABP2	5.88E-01	1.00E+00
ABCC2	9.00E+00	5.07E-238	RGS10	-3.70E-01	1.00E+00	CDH11	7.29E-01	1.00E+00
LOC102389548	5.35E+00	5.31E-238	CCT7	-2.81E-01	1.00E+00	LOC112585072	7.57E-01	1.00E+00
CCNB3	6.39E+00	1.99E-237	LOC112578447	-2.41E+00	1.00E+00	CH25H	-7.86E-01	1.00E+00
SPIC	8.41E+00	3.33E-237	RNASEH2C	-3.48E-01	1.00E+00	MGP	6.66E-01	1.00E+00
NEURL3	8.52E+00	7.17E-236	PIGS	3.20E-01	1.00E+00	HECW2	1.13E+00	1.00E+00
SKA1	5.28E+00	9.86E-236	SPRR4	4.89E-01	1.00E+00	MEIS1	6.01E-01	1.00E+00
YPEL4	4.58E+00	2.89E-235	LRRC2	-1.61E+00	1.00E+00	LRP8	-1.02E+00	1.00E+00
RAB27A	4.62E+00	3.06E-235	PNP	-8.85E-01	1.00E+00	CIT	-4.47E-01	1.00E+00
LOC102401975	7.89E+00	4.29E-235	INSR	-1.31E+00	1.00E+00	FBXO32	5.17E-01	1.00E+00
CPA5	7.44E+00	1.19E-234	TPGS2	-8.34E-01	1.00E+00	CACNA1D	7.49E-01	1.00E+00
SH2D2A	8.03E+00	8.50E-234	MRO	-8.16E-01	1.00E+00	PIK3R2	-4.88E-01	1.00E+00

PAQR5	5.27E+00	1.40E-233	TIAM1	4.83E-01	1.00E+00	INPP4A	-4.56E-01	1.00E+00
SKOR2	8.67E+00	1.82E-233	NAPRT	-8.05E-01	1.00E+00	ABHD2	5.06E-01	1.00E+00
DCLK2	4.78E+00	2.19E-233	ZIC1	3.76E-01	1.00E+00	ADCYAP1	-7.93E-01	1.00E+00
SLC25A3	-5.42E+00	6.13E-233	TAF4	4.82E-01	1.00E+00	MARCKSL1	4.14E-01	1.00E+00
FBXO15	4.24E+00	7.01E-233	ZNF622	-2.23E-01	1.00E+00	IGSF9B	8.03E-01	1.00E+00
AK4	4.19E+00	3.15E-232	BROX	3.89E-01	1.00E+00	PRKAG2	4.91E-01	1.00E+00
NRG2	5.58E+00	8.12E-232	GRIA4	-1.21E+00	1.00E+00	ADGRG6	6.42E-01	1.00E+00
SNX25	3.39E+00	2.83E-231	FBXL18	2.54E-01	1.00E+00	SORL1	-3.61E-01	1.00E+00
RIMS3	5.97E+00	2.90E-231	IPPK	3.55E-01	1.00E+00	METRNL	6.51E-01	1.00E+00
GBX2	9.85E+00	2.93E-231	LOC112583829	9.77E-01	1.00E+00	TRIM63	1.02E+00	1.00E+00
UTP18	2.94E+00	7.89E-231	MED12	3.54E-01	1.00E+00	LOC112585698	6.33E-01	1.00E+00
MAD2L2	5.15E+00	1.17E-230	DCK	2.48E-01	1.00E+00	LOC102397853	-9.78E-01	1.00E+00
LOC102390255	-4.40E+00	1.45E-230	RASSF8	2.73E-01	1.00E+00	GPRC5C	8.40E-01	1.00E+00
LHX6	7.88E+00	2.17E-230	LOC112585789	-6.97E-01	1.00E+00	GRHL1	-4.05E-01	1.00E+00
CDK5R2	1.04E+01	2.80E-230	APIP	-3.62E-01	1.00E+00	CNTNAP2	4.83E-01	1.00E+00
P3H1	-4.22E+00	2.80E-230	WTIP	-2.92E-01	1.00E+00	CCDC71L	5.85E-01	1.00E+00
TPBGL	5.51E+00	2.62E-229	HCFC1	5.33E-01	1.00E+00	EFR3B	5.77E-01	1.00E+00
KIF24	3.83E+00	4.98E-229	LOC112587300	1.18E+00	1.00E+00	KIF23	-5.57E-01	1.00E+00
PLXNB2	-4.88E+00	6.03E-229	LOC112583846	-4.15E-01	1.00E+00	MAPK4	9.00E-01	1.00E+00
C17H4orf33	4.27E+00	9.26E-229	C5H11orf68	-6.01E-01	1.00E+00	HAUS5	-3.45E-01	1.00E+00
CTNS	3.97E+00	9.30E-229	LOC102406115	2.96E-01	1.00E+00	MYO16	1.11E+00	1.00E+00
MAD2L1BP	3.40E+00	2.85E-228	TP53BP2	2.62E-01	1.00E+00	SRP14	4.06E-01	1.00E+00
AGK	3.35E+00	3.64E-227	MINPP1	5.78E-01	1.00E+00	MAF	5.99E-01	1.00E+00
LRP10	-4.29E+00	6.48E-227	LOC102409521	2.45E-01	1.00E+00	IL1R1	5.78E-01	1.00E+00
LOC102411292	1.06E+01	1.42E-226	NUP58	2.88E-01	1.00E+00	DDO	-6.04E-01	1.00E+00
GJB4	8.41E+00	1.91E-226	LOC102389123	-9.20E-01	1.00E+00	BBS1	-3.34E-01	1.00E+00
PRRT1B	6.53E+00	3.00E-226	LOC102401975	-3.22E-01	1.00E+00	JAG1	7.68E-01	1.00E+00
DLX6	8.04E+00	3.93E-226	GPX3	2.73E-01	1.00E+00	LOC112577988	9.62E-01	1.00E+00
TBX15	9.49E+00	4.36E-226	TMEM206	4.26E-01	1.00E+00	TMEM132B	9.94E-01	1.00E+00
HOXC13	7.80E+00	6.83E-226	DPP9	2.52E-01	1.00E+00	OIT3	7.40E-01	1.00E+00
KLHL18	3.56E+00	8.14E-226	PHF12	2.76E-01	1.00E+00	PPP1R15A	-8.51E-01	1.00E+00
BTBD10	3.62E+00	2.12E-225	TRIM11	2.65E-01	1.00E+00	CADM2	1.01E+00	1.00E+00
MASTL	4.41E+00	7.05E-225	XDH	-7.21E-01	1.00E+00	PLXDC2	4.66E-01	1.00E+00
NANOS1	4.29E+00	2.55E-224	SLC30A6	4.58E-01	1.00E+00	SH3PXD2B	5.24E-01	1.00E+00
STK35	3.47E+00	3.13E-224	KATNBL1	3.58E-01	1.00E+00	SQLE	-3.16E-01	1.00E+00
THAP9	3.93E+00	8.26E-224	SIRT1	-5.03E-01	1.00E+00	DAPK1	4.56E-01	1.00E+00

GDPD5	5.47E+00	1.32E-223	CPEB4	-9.18E-01	1.00E+00	SH3BP2	5.09E-01	1.00E+00
EWSR1	-3.94E+00	1.60E-223	MYL7	1.37E+00	1.00E+00	SBK1	5.40E-01	1.00E+00
LOC102401932	4.22E+00	2.56E-223	STK10	4.74E-01	1.00E+00	LOC102409300	7.60E-01	1.00E+00
BSND	1.01E+01	8.90E-223	CTGF	-1.53E+00	1.00E+00	S100A16	4.98E-01	1.00E+00
RPGRIP1	3.70E+00	1.03E-222	FOXN2	8.97E-01	1.00E+00	MN1	1.12E+00	1.00E+00
SGMS2	4.25E+00	1.95E-222	TIGD5	-4.82E-01	1.00E+00	PRDM16	8.87E-01	1.00E+00
DHX35	4.20E+00	2.46E-222	PLPP3	-7.72E-01	1.00E+00	LOC102389067	6.57E-01	1.00E+00
RAB25	5.78E+00	2.86E-222	PANK3	2.66E-01	1.00E+00	LOC102392019	-1.24E+00	1.00E+00
DCTN2	-4.26E+00	2.31E-221	SPNS2	2.85E-01	1.00E+00	UACA	3.65E-01	1.00E+00
WEE2	1.12E+01	4.92E-221	GRAMD2B	-4.12E-01	1.00E+00	PCBP3	8.12E-01	1.00E+00
STOX1	5.42E+00	6.41E-221	LOC102396564	-6.29E-01	1.00E+00	CSRP3	7.37E-01	1.00E+00
APLF	3.23E+00	7.87E-221	ZP2	3.10E-01	1.00E+00	TUBB6	5.65E-01	1.00E+00
THEG	9.20E+00	8.53E-221	PPP1R12A	7.10E-01	1.00E+00	LPIN3	-3.89E-01	1.00E+00
GTF2B	2.71E+00	1.01E-220	LOC102390054	2.15E-01	1.00E+00	PDE4D	7.84E-01	1.00E+00
CCNQ	4.23E+00	2.72E-220	UNC119B	2.73E-01	1.00E+00	UPP1	-1.49E+00	1.00E+00
RAE1	2.83E+00	2.71E-219	LHX5	1.46E+00	1.00E+00	ISYNA1	-5.90E-01	1.00E+00
FAM149A	5.36E+00	3.69E-219	HTR1D	-1.06E+00	1.00E+00	KCNK1	-4.21E-01	1.00E+00
CWC25	3.76E+00	5.61E-219	RNF112	-8.29E-01	1.00E+00	MASP1	-1.34E+00	1.00E+00
MNS1	3.94E+00	1.72E-218	DCN	-1.68E+00	1.00E+00	FAM129B	4.38E-01	1.00E+00
SLAIN1	3.59E+00	2.29E-218	SLC39A1	-3.38E-01	1.00E+00	AGER	8.42E-01	1.00E+00
DBF4B	4.85E+00	4.76E-218	INTS3	2.46E-01	1.00E+00	AXL	7.00E-01	1.00E+00
ARFIP1	3.09E+00	4.88E-218	TRPC6	5.33E-01	1.00E+00	IFI6	5.51E-01	1.00E+00
CHRNA3	7.40E+00	8.82E-218	ALG6	3.01E-01	1.00E+00	IFRD1	-5.98E-01	1.00E+00
AQP3	6.67E+00	4.27E-217	C9H19orf57	2.40E-01	1.00E+00	CADM4	3.81E-01	1.00E+00
SLC25A44	3.00E+00	1.65E-216	CAPN6	1.92E+00	1.00E+00	LOC112585535	-3.76E-01	1.00E+00
HSH2D	7.62E+00	1.76E-216	DNAJB2	-4.36E-01	1.00E+00	FAM129C	-1.03E+00	1.00E+00
PALB2	2.92E+00	4.23E-216	LOC102411924	-7.51E-01	1.00E+00	ADAMTS14	6.20E-01	1.00E+00
PITHD1	3.67E+00	4.95E-216	RCN3	-3.99E-01	1.00E+00	MYO1C	4.09E-01	1.00E+00
PNRC2	-3.51E+00	3.10E-215	NR5A2	-1.28E+00	1.00E+00	ANGPT1	6.95E-01	1.00E+00
CCNI2	6.45E+00	5.04E-215	VPS45	2.70E-01	1.00E+00	MEX3A	4.89E-01	1.00E+00
SNX11	3.00E+00	5.87E-215	LIN28B	-4.06E-01	1.00E+00	TPCN2	-3.52E-01	1.00E+00
NFKBIE	4.19E+00	8.32E-215	TOB1	3.93E-01	1.00E+00	KIT	7.40E-01	1.00E+00
SH3BP5	5.11E+00	2.85E-214	ZNF404	5.24E-01	1.00E+00	LSS	-3.48E-01	1.00E+00
ZIC1	1.01E+01	3.53E-213	RGS6	-2.39E-01	1.00E+00	EPB41L4A	-3.54E-01	1.00E+00
DMRT2	7.51E+00	8.45E-213	LRP4	6.87E-01	1.00E+00	LY6E	6.09E-01	1.00E+00
LRIF1	4.04E+00	9.73E-213	SLC2A4	-7.74E-	1.00E+00	LOC102412752	-	1.00E+00

				01			1.14E+00	
SLC38A8	5.70E+00	1.35E-212	ACY1	9.57E-01	1.00E+00	IL22RA1	6.50E-01	1.00E+00
CUL7	-3.98E+00	2.49E-212	LOC112580241	-6.83E-01	1.00E+00	ITGB8	6.62E-01	1.00E+00
FAM222B	3.65E+00	2.97E-212	LSM14A	2.31E-01	1.00E+00	LOC102404341	-7.53E-01	1.00E+00
EIF4ENIF1	4.64E+00	4.15E-212	LOC112582955	-6.55E-01	1.00E+00	LOC102393384	-3.90E-01	1.00E+00
LOC102402736	4.32E+00	8.16E-212	SLC25A36	2.75E-01	1.00E+00	ITGA2	9.89E-01	1.00E+00
NDUFAF4	4.01E+00	1.23E-211	OXR1	-3.88E-01	1.00E+00	HIVEP3	7.89E-01	1.00E+00
CDS1	6.31E+00	3.00E-211	FAM19A2	2.18E-01	1.00E+00	LOC102399263	3.37E-01	1.00E+00
FAM107B	3.28E+00	1.36E-210	TGM1	-5.52E-01	1.00E+00	ITGA8	7.16E-01	1.00E+00
ITGB4	6.31E+00	2.90E-210	S100A11	-1.52E+00	1.00E+00	ARL4C	6.40E-01	1.00E+00
ATOH7	7.46E+00	3.10E-210	PAK2	2.65E-01	1.00E+00	TGM2	7.11E-01	1.00E+00
HIC2	4.45E+00	1.96E-209	C4H1orf198	-3.36E-01	1.00E+00	DNAJA1	-4.29E-01	1.00E+00
COPS7A	3.56E+00	3.44E-209	SFRP1	-4.08E-01	1.00E+00	AMH	-6.47E-01	1.00E+00
NSF	3.45E+00	4.26E-209	SIKE1	-3.47E-01	1.00E+00	DNER	1.18E+00	1.00E+00
AMOT	4.89E+00	5.94E-209	IFIT2	-8.12E-01	1.00E+00	LOC112587417	8.38E-01	1.00E+00
POLD3	3.63E+00	8.78E-209	ZNF500	6.12E-01	1.00E+00	C20H15orf39	-5.30E-01	1.00E+00
OLIG1	6.63E+00	1.49E-208	SUN2	2.88E-01	1.00E+00	TOX	-4.72E-01	1.00E+00
IMPAD1	-3.52E+00	6.85E-208	LOC102398127	8.66E-01	1.00E+00	ATP6AP2	3.54E-01	1.00E+00
MID1IP1	-5.67E+00	1.39E-207	RIN3	-9.46E-01	1.00E+00	FTH1	-7.56E-01	1.00E+00
NAPEPLD	3.73E+00	2.12E-207	FGFR1OP2	5.77E-01	1.00E+00	COL3A1	8.66E-01	1.00E+00
SPC25	4.50E+00	3.10E-207	HDAC11	-2.90E-01	1.00E+00	LOC112577670	-7.03E-01	1.00E+00
SPAG17	6.07E+00	3.30E-207	CFAP97	3.21E-01	1.00E+00	MALRD1	6.72E-01	1.00E+00
LOC112580607	8.68E+00	3.71E-207	TFPT	-4.06E-01	1.00E+00	PAQR9	7.21E-01	1.00E+00
LOC102408544	5.21E+00	4.24E-207	MYO16	-3.79E-01	1.00E+00	KIFC3	5.79E-01	1.00E+00
MBTPS1	-3.94E+00	1.22E-206	B4GAT1	4.94E-01	1.00E+00	MMP2	-9.25E-01	1.00E+00
LOC102397654	7.33E+00	2.09E-206	LOC112587527	7.85E-01	1.00E+00	PPP1R18	3.74E-01	1.00E+00
RSPO1	8.68E+00	4.35E-206	SACS	-5.41E-01	1.00E+00	RUNX2	5.50E-01	1.00E+00
MTMR1	2.89E+00	1.00E-205	LRCH3	2.50E-01	1.00E+00	LOC112577759	1.07E+00	1.00E+00
KIF3C	3.82E+00	7.96E-205	LOC112586514	-1.25E+00	1.00E+00	NSUN3	-3.85E-01	1.00E+00
SRSF6	-4.32E+00	1.23E-204	LMAN1	3.02E-01	1.00E+00	NDEL1	-4.16E-01	1.00E+00
LOC102413071	7.32E+00	5.53E-204	FBLN1	-6.86E-01	1.00E+00	MSX1	7.55E-01	1.00E+00
RNFT2	3.30E+00	5.94E-204	TCOF1	3.20E-01	1.00E+00	RDH11	-3.00E-01	1.00E+00
SPNS2	5.52E+00	7.04E-204	LOC112579575	-4.17E-01	1.00E+00	ANKRD1	5.32E-01	1.00E+00
LMF1	-5.21E+00	7.71E-204	MAP2	3.38E-01	1.00E+00	ZNF469	5.26E-01	1.00E+00
MEGF9	4.77E+00	1.29E-203	SNX22	-5.65E-01	1.00E+00	EFNA4	5.50E-01	1.00E+00

HLF	3.33E+00	1.56E-203	THEM4	-4.57E-01	1.00E+00	MYZAP	-3.44E-01	1.00E+00
MPIG6B	6.07E+00	2.36E-203	DUSP10	2.38E-01	1.00E+00	TSPAN18	5.90E-01	1.00E+00
POU2F1	3.88E+00	3.33E-203	FBXO40	-8.32E-01	1.00E+00	SELENOP	6.54E-01	1.00E+00
BICDL1	3.48E+00	5.34E-203	NAALADL1	-4.52E-01	1.00E+00	FOXO6	-6.40E-01	1.00E+00
CHL1	8.35E+00	7.86E-203	DCTPP1	-5.15E-01	1.00E+00	KIAA0040	7.35E-01	1.00E+00
CLIP2	-4.18E+00	1.11E-202	LOC102410916	-1.16E+00	1.00E+00	LOC102408976	5.91E-01	1.00E+00
GIPR	7.99E+00	5.78E-202	GALE	7.06E-01	1.00E+00	FGF13	9.28E-01	1.00E+00
SPINK5	7.06E+00	1.17E-201	HAPLN1	-8.48E-01	1.00E+00	IL21R	9.29E-01	1.00E+00
SYT9	2.72E+00	2.33E-201	CREG1	2.50E-01	1.00E+00	RAB5IF	-4.04E-01	1.00E+00
LOC102404976	4.46E+00	3.65E-201	DGKI	-1.96E-01	1.00E+00	LOC102406311	8.35E-01	1.00E+00
OCLN	3.98E+00	2.17E-200	LRRC72	6.89E-01	1.00E+00	LOC102412347	8.86E-01	1.00E+00
TPCN1	3.71E+00	3.78E-200	BTLA	7.34E-01	1.00E+00	STS	9.76E-01	1.00E+00
DOK5	3.07E+00	4.10E-200	VAMP8	-5.20E-01	1.00E+00	ZMYM3	-3.63E-01	1.00E+00
LOC112580611	9.21E+00	7.15E-200	STXBP1	2.29E-01	1.00E+00	ARSE	-1.06E+00	1.00E+00
CDH3	4.23E+00	7.85E-200	LY75	-9.82E-01	1.00E+00	SC5D	-5.39E-01	1.00E+00
FRZB	5.71E+00	1.05E-199	DPP10	-5.68E-01	1.00E+00	MAP3K9	6.63E-01	1.00E+00
PTK7	-4.21E+00	2.84E-199	SLC6A5	2.75E-01	1.00E+00	TMED1	4.50E-01	1.00E+00
PLD3	-5.32E+00	4.08E-199	LOC102414399	-1.36E+00	1.00E+00	CKAP2	-3.58E-01	1.00E+00
LOC102394123	9.82E+00	9.78E-199	RGL1	1.90E-01	1.00E+00	NKAIN1	6.96E-01	1.00E+00
PROCA1	3.74E+00	1.27E-198	ADAM8	-1.22E+00	1.00E+00	PVR	4.94E-01	1.00E+00
STAP1	6.40E+00	2.42E-198	PPT2	4.08E-01	1.00E+00	MCAM	-3.44E-01	1.00E+00
NONO	-3.30E+00	2.80E-198	INPP5K	3.57E-01	1.00E+00	NLGN4X	5.02E-01	1.00E+00
MAZ	-4.64E+00	3.30E-198	ZNF879	-2.71E-01	1.00E+00	TMEM44	4.86E-01	1.00E+00
IQCB1	2.86E+00	8.00E-198	MYL6B	4.66E-01	1.00E+00	LPAR3	9.52E-01	1.00E+00
CDKL2	6.78E+00	1.71E-197	LOC102391395	4.40E-01	1.00E+00	NUDCD2	4.27E-01	1.00E+00
SELENON	-3.15E+00	1.87E-197	CNOT3	2.02E-01	1.00E+00	ARNTL2	8.54E-01	1.00E+00
CPS1	7.71E+00	3.09E-197	PPP2R2C	-3.39E-01	1.00E+00	LOC102416225	-1.55E+00	1.00E+00
LOC102389575	6.51E+00	3.31E-197	PKP2	-4.54E-01	1.00E+00	DLG2	8.68E-01	1.00E+00
ISL1	9.22E+00	5.57E-197	HCN2	2.47E-01	1.00E+00	MIA	-5.68E-01	1.00E+00
LOC102408956	-5.05E+00	1.33E-196	PLPP2	-6.98E-01	1.00E+00	VGF	5.89E-01	1.00E+00
SLC7A9	7.29E+00	1.64E-196	MYOF	-2.82E-01	1.00E+00	PNN	-3.30E-01	1.00E+00
SLC25A53	2.88E+00	3.47E-196	SELENOS	-6.17E-01	1.00E+00	CREBZF	-3.05E-01	1.00E+00
LOC102402934	1.13E+01	3.74E-196	ZNF169	5.79E-01	1.00E+00	SLC29A2	-3.73E-01	1.00E+00
PAPOLG	3.98E+00	4.33E-196	CHRNA3	2.68E-01	1.00E+00	TMTC4	4.22E-01	1.00E+00
SLC25A13	3.03E+00	5.20E-196	RPH3A	1.13E+00	1.00E+00	PLEKHA2	4.59E-01	1.00E+00

CRTAP	-4.94E+00	1.51E-195	ZNF554	-9.09E-01	1.00E+00	PPM1L	6.46E-01	1.00E+00
MELK	3.82E+00	1.82E-195	CXADR	4.55E-01	1.00E+00	GPC4	-3.61E-01	1.00E+00
YBX2	7.31E+00	3.79E-195	RIN2	-1.21E+00	1.00E+00	CEP85	-3.44E-01	1.00E+00
ZNF772	3.29E+00	1.70E-194	LOC102406308	3.03E-01	1.00E+00	AOAH	1.30E+00	1.00E+00
LOC102413993	7.71E+00	1.92E-194	SEMA6A	9.10E-01	1.00E+00	PRKCG	-4.16E-01	1.00E+00
TAT	7.52E+00	3.03E-194	STON1	2.73E-01	1.00E+00	SREBF1	-3.65E-01	1.00E+00
CILP	4.69E+00	3.16E-194	CCM2	2.92E-01	1.00E+00	DAO	-6.77E-01	1.00E+00
NCK1	2.92E+00	4.25E-194	PLD1	-8.77E-01	1.00E+00	LOC102412044	4.43E-01	1.00E+00
MST1R	7.61E+00	5.67E-194	CCT8L2	7.14E-01	1.00E+00	UNC5D	1.22E+00	1.00E+00
LOC112578525	9.50E+00	6.56E-194	TTC14	3.37E-01	1.00E+00	LOC112578381	3.96E-01	1.00E+00
CPA1	8.03E+00	1.11E-193	JUND	-4.82E-01	1.00E+00	AKAP6	1.05E+00	1.00E+00
EPSTI1	3.58E+00	1.40E-193	AGL	-7.13E-01	1.00E+00	STAR	-1.21E+00	1.00E+00
TADA2A	2.90E+00	2.17E-193	PPP2R5D	1.99E-01	1.00E+00	PTK7	3.31E-01	1.00E+00
TMEM14A	-4.91E+00	2.78E-193	ATP8A2	-3.68E-01	1.00E+00	LOC112582058	9.78E-01	1.00E+00
LGALS4	4.69E+00	3.28E-193	PML	-6.66E-01	1.00E+00	ITGB3	6.28E-01	1.00E+00
MNX1	9.22E+00	3.58E-193	SCAMP1	2.84E-01	1.00E+00	LOC102410358	-9.85E-01	1.00E+00
ARID3B	3.46E+00	1.06E-192	PRDM2	3.85E-01	1.00E+00	BICC1	1.15E+00	1.00E+00
WDR93	3.68E+00	1.90E-192	IGF2BP3	2.08E-01	1.00E+00	TMCC3	5.29E-01	1.00E+00
RASGRP2	7.63E+00	2.34E-192	CD164	2.34E-01	1.00E+00	ETV6	3.45E-01	1.00E+00
PCOLCE2	-5.78E+00	3.46E-192	LIMK1	3.88E-01	1.00E+00	LOXL4	6.39E-01	1.00E+00
MARK1	-3.38E+00	6.93E-192	PIM2	-4.16E-01	1.00E+00	STX3	-3.17E-01	1.00E+00
FGFR1	-3.34E+00	7.64E-192	MAML2	-9.99E-01	1.00E+00	ABTB2	6.30E-01	1.00E+00
PPP2R3B	3.73E+00	1.53E-191	GALNT14	-4.76E-01	1.00E+00	LOC112579531	5.37E-01	1.00E+00
TBC1D10A	3.69E+00	2.29E-191	SEPT9	-4.71E-01	1.00E+00	BRK1	3.42E-01	1.00E+00
GNL3L	-5.50E+00	7.91E-191	RWDD1	-3.55E-01	1.00E+00	MBOAT1	5.57E-01	1.00E+00
PTPN3	3.19E+00	1.10E-190	CDC20B	4.76E-01	1.00E+00	KAZN	7.51E-01	1.00E+00
POLR3G	3.48E+00	1.51E-190	DOCK6	4.33E-01	1.00E+00	CFAP70	-6.76E-01	1.00E+00
MIB2	-4.19E+00	2.57E-190	LOC112587820	-4.79E-01	1.00E+00	CYR61	-5.91E-01	1.00E+00
CPEB1	4.73E+00	2.85E-190	BAHCC1	-4.78E-01	1.00E+00	NCAPD3	-3.40E-01	1.00E+00
CDK7	3.06E+00	2.89E-190	MBNL1	-9.00E-01	1.00E+00	S100A2	6.10E-01	1.00E+00
HNRNPL	-4.27E+00	4.86E-190	C4H22orf23	9.49E-01	1.00E+00	LOC102390513	8.15E-01	1.00E+00
NUDCD1	2.93E+00	7.85E-190	GOLGB1	2.63E-01	1.00E+00	DACH2	1.15E+00	1.00E+00
ASPHD2	5.20E+00	1.55E-189	NCOA7	7.12E-01	1.00E+00	CCBE1	4.94E-01	1.00E+00
TCP11L1	2.94E+00	2.44E-189	NACAD	5.00E-01	1.00E+00	TBX2	1.00E+00	1.00E+00
LOC112585634	9.73E+00	2.72E-189	RANBP1	-2.11E-01	1.00E+00	ACSS2	-3.42E-01	1.00E+00
CDKAL1	3.19E+00	3.47E-189	MROH8	-7.41E-01	1.00E+00	IGFBP1	8.04E-01	1.00E+00

ZAR1L	5.73E+00	5.17E-189	PDPR	3.03E-01	1.00E+00	DLG4	-3.37E-01	1.00E+00
PSD2	6.45E+00	8.12E-189	LOC112580611	-3.61E-01	1.00E+00	KCTD17	-5.02E-01	1.00E+00
NMD3	3.34E+00	1.41E-188	DCAKD	2.58E-01	1.00E+00	MCFD2	-3.01E-01	1.00E+00
TXNDC5	-3.65E+00	3.25E-188	LOC102413847	-3.33E-01	1.00E+00	TAX1BP3	3.69E-01	1.00E+00
C8H7orf25	2.90E+00	4.34E-188	R3HDM1	2.44E-01	1.00E+00	TOB1	-4.97E-01	1.00E+00
PARP12	3.87E+00	6.18E-188	PPP1R35	-2.82E-01	1.00E+00	FANCA	-4.09E-01	1.00E+00
KDM5B	-3.84E+00	1.28E-187	XG	-1.43E+00	1.00E+00	NR3C2	9.61E-01	1.00E+00
ZFYVE1	3.40E+00	9.48E-187	WASHC4	2.69E-01	1.00E+00	LSAMP	1.28E+00	1.00E+00
CDX1	8.94E+00	1.73E-186	CEBPB	-8.52E-01	1.00E+00	AHNAK	6.01E-01	1.00E+00
TMEM267	3.65E+00	6.49E-186	SEMA3A	6.03E-01	1.00E+00	COL22A1	8.51E-01	1.00E+00
LOC102402624	3.55E+00	7.66E-186	LOC102411053	-1.09E+00	1.00E+00	TMEM106C	4.16E-01	1.00E+00
TERT	6.66E+00	7.75E-186	PTBP2	2.30E-01	1.00E+00	PLXNA2	-3.23E-01	1.00E+00
LOC102403333	3.65E+00	1.09E-185	LOC102402894	-8.25E-01	1.00E+00	CDC42EP2	8.04E-01	1.00E+00
SARAF	-3.11E+00	1.32E-185	CD63	-2.49E-01	1.00E+00	WWC1	4.28E-01	1.00E+00
MZF1	-3.54E+00	5.43E-185	GABRB2	6.02E-01	1.00E+00	SPOCD1	-8.28E-01	1.00E+00
MGAT4A	4.49E+00	2.36E-184	LOC112583968	-6.91E-01	1.00E+00	TENM3	8.05E-01	1.00E+00
OMA1	2.93E+00	2.70E-184	PCLO	3.34E-01	1.00E+00	EWSR1	-3.25E-01	1.00E+00
TFB1M	2.73E+00	3.29E-184	SEC16B	-2.12E-01	1.00E+00	ADGRG1	-3.41E-01	1.00E+00
NLRP9	1.16E+01	3.95E-184	RELL1	3.57E-01	1.00E+00	SH3D19	-3.34E-01	1.00E+00
PABPC1L	4.47E+00	4.34E-184	ITPKC	-4.75E-01	1.00E+00	SNTG2	1.13E+00	1.00E+00
CNNM2	4.44E+00	5.77E-183	XYLT2	5.41E-01	1.00E+00	CCDC194	-7.41E-01	1.00E+00
RNF34	2.89E+00	1.06E-182	MAGIX	-2.43E-01	1.00E+00	STAC2	6.58E-01	1.00E+00
ABHD14B	-4.98E+00	2.44E-182	RGS2	-2.09E-01	1.00E+00	ABCA4	4.47E-01	1.00E+00
DHX58	3.11E+00	2.59E-182	FAM81B	-2.76E-01	1.00E+00	FRMD4B	7.55E-01	1.00E+00
RUNDC3B	7.31E+00	3.36E-182	PRKCI	-4.53E-01	1.00E+00	RETN	-1.09E+00	1.00E+00
HENMT1	4.39E+00	4.48E-182	CDK5R2	2.49E-01	1.00E+00	NRG3	6.74E-01	1.00E+00
VRTN	9.62E+00	5.66E-182	SFRP2	6.62E-01	1.00E+00	TENT5A	6.80E-01	1.00E+00
FANCF	4.51E+00	7.50E-182	ARHGEF39	-4.36E-01	1.00E+00	LOC102403482	-3.11E-01	1.00E+00
LOC102405065	8.95E+00	8.62E-182	ABCC2	2.29E-01	1.00E+00	CCNL1	-5.90E-01	1.00E+00
PDLIM2	4.94E+00	1.41E-181	LOC112581264	-2.72E-01	1.00E+00	TENT5C	-5.38E-01	1.00E+00
PLS1	6.23E+00	1.61E-181	LYPD6B	-4.48E-01	1.00E+00	CCDC149	4.04E-01	1.00E+00
TBPL2	9.42E+00	1.96E-181	NIM1K	1.00E+00	1.00E+00	ZBTB46	8.26E-01	1.00E+00
ARMC1	2.73E+00	2.45E-181	TMED7	2.39E-01	1.00E+00	FMNL3	5.18E-01	1.00E+00
RIPOR1	-2.92E+00	4.14E-181	LOC102413340	-7.70E-01	1.00E+00	GDNF	8.65E-01	1.00E+00

SLC35D1	4.95E+00	4.22E-181	PRR22	1.10E+00	1.00E+00	SLC43A1	-5.86E-01	1.00E+00
RALB	3.32E+00	4.24E-181	UNK	2.55E-01	1.00E+00	CNTN4	8.03E-01	1.00E+00
SMO	-3.73E+00	1.03E-180	SYNJ2BP	2.53E-01	1.00E+00	CAPRIN2	-4.54E-01	1.00E+00
MPHOSPH9	3.69E+00	1.40E-180	ZMYND8	2.88E-01	1.00E+00	PTPRE	6.94E-01	1.00E+00
GPBAR1	6.14E+00	1.61E-180	MAP3K20	-5.60E-01	1.00E+00	LOC102396919	-3.93E-01	1.00E+00
CLK1	-3.86E+00	2.00E-180	MEI4	-6.34E-01	1.00E+00	B3GNT8	5.93E-01	1.00E+00
DMRTC2	1.15E+01	2.04E-180	F8	-1.24E+00	1.00E+00	TM4SF1	5.11E-01	1.00E+00
RBMXL2	9.20E+00	2.73E-180	NADK2	2.35E-01	1.00E+00	PLCE1	8.64E-01	1.00E+00
LOC102395887	1.13E+01	3.51E-180	SLC8A2	-4.98E-01	1.00E+00	PPFIA2	1.01E+00	1.00E+00
PPFIBP2	3.14E+00	4.32E-180	MBOAT1	2.32E-01	1.00E+00	CLDN1	4.84E-01	1.00E+00
ZBTB5	2.82E+00	4.36E-180	EGR2	-1.48E+00	1.00E+00	ATL1	8.65E-01	1.00E+00
CRLF3	2.73E+00	4.39E-180	TUT4	3.47E-01	1.00E+00	INPP1	5.07E-01	1.00E+00
ARHGEF37	5.55E+00	6.27E-180	HCFC2	2.66E-01	1.00E+00	TTC39B	-3.39E-01	1.00E+00
VWA2	6.22E+00	8.60E-180	MYL10	8.78E-01	1.00E+00	LOC112585122	4.84E-01	1.00E+00
ETV5	6.33E+00	1.36E-179	SESTD1	2.15E-01	1.00E+00	PRSS35	-5.74E-01	1.00E+00
TMED2	-3.20E+00	1.47E-179	LOC112577706	5.21E-01	1.00E+00	CDSN	7.79E-01	1.00E+00
TBC1D2B	3.73E+00	1.69E-179	KIAA1191	1.82E-01	1.00E+00	MACROD2	3.33E-01	1.00E+00
ZCCHC7	2.82E+00	2.70E-179	ARHGAP32	3.95E-01	1.00E+00	DDAH1	4.90E-01	1.00E+00
MBNL3	4.48E+00	3.74E-179	GADD45G	-1.51E+00	1.00E+00	TMEM126B	5.34E-01	1.00E+00
RIOX2	3.42E+00	5.10E-179	HOXC13	2.80E-01	1.00E+00	KIF18B	-3.58E-01	1.00E+00
DGKI	4.24E+00	5.73E-179	CEP70	1.98E-01	1.00E+00	LOC102407427	9.37E-01	1.00E+00
CFAP20	3.20E+00	6.08E-179	NLGN2	9.94E-01	1.00E+00	TGFA	8.67E-01	1.00E+00
RGL3	6.40E+00	6.43E-179	TIMP2	-4.30E-01	1.00E+00	LOC102413604	7.29E-01	1.00E+00
TRA2B	-3.99E+00	1.08E-178	PJA2	2.54E-01	1.00E+00	LIFR	-4.06E-01	1.00E+00
SLC12A8	6.84E+00	2.93E-178	NOXO1	-9.60E-01	1.00E+00	LOC112578889	-5.81E-01	1.00E+00
ELL	3.84E+00	3.53E-178	LOC102410846	-4.11E-01	1.00E+00	LOC102404841	8.75E-01	1.00E+00
SIRT2	-3.20E+00	3.77E-178	CTDSP2	2.26E-01	1.00E+00	MMP11	5.30E-01	1.00E+00
LOC102392446	-3.67E+00	4.31E-178	C7	-3.73E-01	1.00E+00	LOC112581264	-4.92E-01	1.00E+00
RAD51	3.15E+00	4.51E-178	TTLL1	-6.73E-01	1.00E+00	WFDC1	-4.57E-01	1.00E+00
UBTF	-3.49E+00	4.71E-178	XIRP2	9.99E-01	1.00E+00	ABCD1	-4.78E-01	1.00E+00
LOC112585040	7.61E+00	9.55E-178	LOC112586887	-6.56E-01	1.00E+00	DLGAP2	1.03E+00	1.00E+00
SLC15A1	4.05E+00	1.15E-177	EIF4EBP2	2.54E-01	1.00E+00	NOCT	-3.98E-01	1.00E+00
CSDC2	-5.49E+00	1.32E-177	LOC112580906	-6.33E-01	1.00E+00	FOXJ1	-1.00E+00	1.00E+00
FAM133B	-3.52E+00	1.33E-177	ZNF146	-4.39E-01	1.00E+00	SPAG16	7.73E-01	1.00E+00
CCDC42	7.23E+00	1.80E-177	ZSCAN31	5.33E-01	1.00E+00	DOCK10	6.33E-01	1.00E+00
ZNF200	4.11E+00	2.05E-177	MYZAP	-8.10E-01	1.00E+00	CGN	-4.32E-01	1.00E+00

KLHDC9	5.39E+00	2.05E-177	C9H19orf25	-5.77E-01	1.00E+00	PIK3C2G	8.99E-01	1.00E+00
GJB5	8.59E+00	2.43E-177	GOT2	-2.01E-01	1.00E+00	ZBTB7C	5.51E-01	1.00E+00
MAOA	-6.53E+00	3.75E-177	LOC102407744	-8.80E-01	1.00E+00	CELF2	1.14E+00	1.00E+00
KCTD9	3.33E+00	8.21E-177	QSOX2	2.71E-01	1.00E+00	ITGA7	5.54E-01	1.00E+00
LOC112579558	4.25E+00	1.13E-176	PAK5	3.73E-01	1.00E+00	ASB9	-4.64E-01	1.00E+00
BBS2	3.07E+00	1.19E-176	BHLHE40	-7.79E-01	1.00E+00	ZFAND5	-5.37E-01	1.00E+00
FAM3C	-4.53E+00	1.61E-176	LOC112581215	-5.27E-01	1.00E+00	LOC102408542	1.09E+00	1.00E+00
CBX3	-5.21E+00	1.71E-176	LOC102397724	-6.09E-01	1.00E+00	KCNH2	-3.18E-01	1.00E+00
MYCL	5.35E+00	2.41E-176	PSPN	-3.91E-01	1.00E+00	SLC4A3	-3.74E-01	1.00E+00
MKRN2	3.14E+00	4.92E-176	SUCLG2	-1.91E-01	1.00E+00	PPP1R12B	-3.19E-01	1.00E+00
SCN8A	3.58E+00	1.20E-175	BSN	4.35E-01	1.00E+00	ACADSB	-3.18E-01	1.00E+00
SLC4A1AP	2.83E+00	3.94E-175	PXDN	8.20E-01	1.00E+00	SIGLEC1	1.45E+00	1.00E+00
LOC112582139	4.81E+00	3.96E-175	POLR2A	3.26E-01	1.00E+00	PALMD	7.02E-01	1.00E+00
ITM2B	-3.84E+00	4.20E-175	RPIA	-2.20E-01	1.00E+00	RIN1	6.15E-01	1.00E+00
CUX1	-3.74E+00	4.22E-175	ELAVL3	2.76E-01	1.00E+00	GRB10	5.00E-01	1.00E+00
ABHD17C	3.06E+00	4.53E-175	CCDC180	-6.61E-01	1.00E+00	PRKCQ	8.46E-01	1.00E+00
TDRP	2.86E+00	5.48E-175	LOC112585546	-7.88E-01	1.00E+00	TPT1	-4.93E-01	1.00E+00
FGFRL1	-4.27E+00	7.63E-175	C20H15orf39	3.93E-01	1.00E+00	CIDEC	5.81E-01	1.00E+00
RAB34	-5.52E+00	8.49E-175	LOC102411786	-1.13E+00	1.00E+00	HDX	7.78E-01	1.00E+00
LOC112585626	9.16E+00	3.76E-174	TMEM119	-7.54E-01	1.00E+00	ZWINT	-3.62E-01	1.00E+00
LOC102398834	9.26E+00	3.93E-174	LOC112586240	-3.00E-01	1.00E+00	THBS1	5.76E-01	1.00E+00
SLC1A5	3.69E+00	4.18E-174	LOC102392766	-2.73E-01	1.00E+00	CD59	3.54E-01	1.00E+00
FBXW7	3.00E+00	4.37E-174	IDH2	-8.88E-01	1.00E+00	SLC41A2	3.75E-01	1.00E+00
KLHL32	3.38E+00	4.55E-174	VSTM2B	-1.02E+00	1.00E+00	EFNA5	3.68E-01	1.00E+00
HOOK2	3.80E+00	4.67E-174	EVC	-7.62E-01	1.00E+00	LOC102411988	-5.95E-01	1.00E+00
MYH15	5.63E+00	4.88E-174	MRPL47	-4.45E-01	1.00E+00	GPR176	7.43E-01	1.00E+00
CPSF7	-3.54E+00	5.63E-174	HTRA2	-4.74E-01	1.00E+00	HDAC6	-2.94E-01	1.00E+00
BMP1	-5.60E+00	8.70E-174	LOC112587918	-4.12E-01	1.00E+00	INAVA	6.02E-01	1.00E+00
RAP2A	-4.26E+00	2.62E-173	LOC112583779	6.08E-01	1.00E+00	LOC112584592	7.63E-01	1.00E+00
RAB35	3.13E+00	3.50E-173	LOC102408728	8.20E-01	1.00E+00	CDON	-3.66E-01	1.00E+00
FADS2	-5.87E+00	4.14E-173	SOS1	4.44E-01	1.00E+00	AFAP1	5.93E-01	1.00E+00
PDK3	3.53E+00	4.50E-173	WDR66	-8.82E-01	1.00E+00	ITPK1	3.50E-01	1.00E+00
PPEF2	6.67E+00	5.21E-173	NELFB	2.04E-01	1.00E+00	LOC102390270	8.46E-01	1.00E+00
LOC102391171	7.95E+00	5.53E-173	LOC112580283	4.09E-01	1.00E+00	STK17B	6.11E-01	1.00E+00

SAFB	-3.04E+00	1.21E-172	LOC112580602	-4.57E-01	1.00E+00	CEBPB	-5.59E-01	1.00E+00
LOC102413670	8.37E+00	1.22E-172	FSTL5	-1.16E+00	1.00E+00	LOC102414965	-4.82E-01	1.00E+00
ARL6IP5	-3.88E+00	1.35E-172	LOC112586804	-8.54E-01	1.00E+00	ERG	1.16E+00	1.00E+00
HCN2	3.70E+00	1.36E-172	LRRC75A	1.01E+00	1.00E+00	AP1S1	3.47E-01	1.00E+00
ACP6	3.32E+00	1.41E-172	IKBKG	2.72E-01	1.00E+00	DTNA	5.41E-01	1.00E+00
SPIN1	-3.23E+00	1.64E-172	ENPP4	8.45E-01	1.00E+00	KIF26B	1.08E+00	1.00E+00
GPR19	4.91E+00	2.59E-172	LOC102405093	4.78E-01	1.00E+00	RAVER2	-3.30E-01	1.00E+00
PLB1	5.45E+00	6.07E-172	LOC112580196	-3.14E-01	1.00E+00	POSTN	1.00E+00	1.00E+00
SPTB	4.16E+00	7.34E-172	SNX14	2.40E-01	1.00E+00	KCNMA1	9.52E-01	1.00E+00
RADIL	4.94E+00	1.15E-171	POLD3	2.23E-01	1.00E+00	CREB3L1	5.61E-01	1.00E+00
DELE1	-3.12E+00	1.34E-171	CORO2B	-7.97E-01	1.00E+00	CLDN11	5.31E-01	1.00E+00
PRKRIP1	3.17E+00	1.65E-171	LOC112587836	-1.04E+00	1.00E+00	PTCH2	-6.48E-01	1.00E+00
THBS4	4.41E+00	1.80E-171	HELZ	3.78E-01	1.00E+00	AKR1E2	-4.49E-01	1.00E+00
TRIM27	-4.23E+00	2.94E-171	RPS12	-3.36E-01	1.00E+00	GLIS3	1.11E+00	1.00E+00
VSX2	8.99E+00	3.27E-171	ZCCHC8	3.05E-01	1.00E+00	DNMBP	-3.98E-01	1.00E+00
LOC112580461	1.05E+01	3.61E-171	CIB3	-5.98E-01	1.00E+00	PRDX3	-3.28E-01	1.00E+00
YPEL5	3.90E+00	3.84E-171	PYCR3	-5.54E-01	1.00E+00	LOC112585337	-7.78E-01	1.00E+00
GNAO1	5.86E+00	4.84E-171	LOC102412910	5.69E-01	1.00E+00	AFF3	5.74E-01	1.00E+00
TMEM30B	8.76E+00	6.50E-171	IVNS1ABP	-4.38E-01	1.00E+00	PBLD	-7.42E-01	1.00E+00
CTSA	-3.56E+00	6.64E-171	CANX	2.00E-01	1.00E+00	ZFPM1	-4.02E-01	1.00E+00
PAIP1	5.30E+00	7.18E-171	NAPG	3.77E-01	1.00E+00	CDH12	7.73E-01	1.00E+00
TBPL1	3.16E+00	7.42E-171	TMEM60	-2.48E-01	1.00E+00	PDLIM4	3.79E-01	1.00E+00
TSPAN7	4.33E+00	8.99E-171	LOC102389131	-1.01E+00	1.00E+00	LOC102395906	9.04E-01	1.00E+00
CDK4	-3.99E+00	3.12E-170	GAB2	3.09E-01	1.00E+00	PRKG1	8.78E-01	1.00E+00
IKBIP	-4.39E+00	3.69E-170	QPCT	-4.50E-01	1.00E+00	CLSPN	-3.89E-01	1.00E+00
GSE1	4.24E+00	3.71E-170	DOCK9	3.54E-01	1.00E+00	FOSL2	4.38E-01	1.00E+00
GMPR	3.48E+00	6.68E-170	PPP1R3C	-2.54E-01	1.00E+00	SVOP	5.08E-01	1.00E+00
SLC9A6	2.98E+00	1.05E-169	LOC112586898	4.72E-01	1.00E+00	HS3ST5	3.75E-01	1.00E+00
TESC	5.01E+00	1.54E-169	COX1	-5.49E-01	1.00E+00	CKS2	-3.59E-01	1.00E+00
FAM169A	2.96E+00	1.92E-169	BBOF1	6.61E-01	1.00E+00	TRIL	6.96E-01	1.00E+00
SYCP3	5.13E+00	2.08E-169	POMGNT1	2.85E-01	1.00E+00	HDAC7	3.81E-01	1.00E+00
GJA4	5.89E+00	2.38E-169	LOC102395053	-5.74E-01	1.00E+00	ZC3H12B	6.22E-01	1.00E+00
PRKCA	2.79E+00	3.10E-169	TXLNB	-4.30E-01	1.00E+00	TLL2	-3.91E-01	1.00E+00
LOC102408435	-4.06E+00	3.14E-169	PPP1R1A	-3.70E-01	1.00E+00	WASF3	-5.09E-01	1.00E+00
MBOAT1	4.61E+00	3.20E-169	EED	-3.76E-01	1.00E+00	HS1BP3	3.46E-01	1.00E+00
MMS22L	3.29E+00	3.24E-169	LOC102389932	-1.79E-	1.00E+00	EML1	3.87E-01	1.00E+00

				01				
HOXD1	6.70E+00	4.23E-169	FAM3D	-1.21E+00	1.00E+00	HPDL	-8.71E-01	1.00E+00
SGF29	3.63E+00	7.42E-169	CAGE1	-6.12E-01	1.00E+00	PRKCB	1.13E+00	1.00E+00
TSPO	-5.11E+00	8.16E-169	CD4	5.61E-01	1.00E+00	CCND3	3.48E-01	1.00E+00
NOD1	5.90E+00	1.89E-168	KIAA1147	1.91E-01	1.00E+00	LOC112578957	-4.37E-01	1.00E+00
ZFP64	3.21E+00	2.81E-168	BARX2	5.20E-01	1.00E+00	CDC14A	-3.51E-01	1.00E+00
NUDT6	2.43E+00	4.59E-168	ARL4C	-6.50E-01	1.00E+00	KRT79	7.42E-01	1.00E+00
KIAA0895	2.95E+00	6.98E-168	MAPK7	1.95E-01	1.00E+00	WNT5B	6.69E-01	1.00E+00
C15H8orf76	2.84E+00	7.75E-168	MYO15A	3.00E-01	1.00E+00	GRK5	-7.23E-01	1.00E+00
LOC102393305	8.63E+00	1.19E-167	TSPAN15	-1.04E+00	1.00E+00	ANKFN1	9.44E-01	1.00E+00
LOC102404426	9.25E+00	2.08E-167	LOC112579125	-6.50E-01	1.00E+00	DECR1	-3.16E-01	1.00E+00
CHAF1A	4.02E+00	2.37E-167	RPUSD3	2.61E-01	1.00E+00	BMPER	6.48E-01	1.00E+00
MYH11	-5.23E+00	3.77E-167	LOC102409898	-2.57E-01	1.00E+00	MID2	6.71E-01	1.00E+00
FAM78A	-6.81E+00	6.12E-167	SKI	2.37E-01	1.00E+00	RAB11FIP4	7.82E-01	1.00E+00
CBX8	3.29E+00	6.73E-167	SMAP2	3.49E-01	1.00E+00	BMP6	5.97E-01	1.00E+00
LOC102411025	9.52E+00	7.18E-167	TECTB	-4.80E-01	1.00E+00	CNRIP1	-3.00E-01	1.00E+00
EPB42	6.87E+00	7.26E-167	ago-02	2.58E-01	1.00E+00	CTSO	-2.99E-01	1.00E+00
LOC102400824	1.07E+01	7.96E-167	LOC102413305	3.14E-01	1.00E+00	PPP1R42	6.49E-01	1.00E+00
SH3BGRL2	4.02E+00	9.04E-167	SP1	2.23E-01	1.00E+00	CA13	7.78E-01	1.00E+00
GP1BA	7.68E+00	2.24E-166	FGD6	-4.53E-01	1.00E+00	F3	3.84E-01	1.00E+00
RNF31	2.61E+00	2.98E-166	LOC112578844	-4.64E-01	1.00E+00	NCAPH	-3.40E-01	1.00E+00
TUB	4.29E+00	4.16E-166	CRTAP	-4.61E-01	1.00E+00	MAFF	-8.62E-01	1.00E+00
UNC5B	6.38E+00	4.42E-166	ENC1	-7.27E-01	1.00E+00	SMIM1	-4.98E-01	1.00E+00
TMEM229B	-3.40E+00	5.75E-166	TUT1	-3.84E-01	1.00E+00	MCTP2	1.03E+00	1.00E+00
MRPS36	3.23E+00	8.51E-166	RALGDS	1.85E-01	1.00E+00	LOC112585316	-6.09E-01	1.00E+00
KPNA4	-3.52E+00	1.25E-165	LOC112577795	-5.07E-01	1.00E+00	CD9	3.97E-01	1.00E+00
LMBRD1	2.48E+00	2.83E-165	TSPO	-4.38E-01	1.00E+00	L3MBTL1	-5.40E-01	1.00E+00
LRP2	8.93E+00	2.85E-165	POLG	3.47E-01	1.00E+00	CCNI2	-7.22E-01	1.00E+00
TDRD7	2.64E+00	3.03E-165	ATXN2	3.11E-01	1.00E+00	TMEM71	8.54E-01	1.00E+00
ORC2	3.42E+00	3.46E-165	LOC102402015	-7.20E-01	1.00E+00	EPB41L4B	4.08E-01	1.00E+00
HDAC5	-3.49E+00	3.94E-165	LOC102406344	3.55E-01	1.00E+00	LOC112580458	-7.25E-01	1.00E+00
ATP6V1B1	8.10E+00	4.85E-165	ZNF317	3.46E-01	1.00E+00	GLI3	5.40E-01	1.00E+00
LOC102403482	-3.70E+00	6.26E-165	TCF20	2.83E-01	1.00E+00	TMEM41B	-4.61E-01	1.00E+00
SRPX	4.34E+00	1.88E-164	CRHR2	-5.23E-01	1.00E+00	GAS2	7.30E-01	1.00E+00
CCDC93	2.31E+00	2.05E-164	ARHGEF16	2.41E-01	1.00E+00	TRIM6	-4.87E-01	1.00E+00

LOC102400686	-6.50E+00	2.43E-164	KYNU	-1.35E+00	1.00E+00	TSPO	3.93E-01	1.00E+00
PAX3	9.26E+00	2.46E-164	RASSF7	-6.68E-01	1.00E+00	VAV3	7.98E-01	1.00E+00
RBM3	-3.97E+00	2.58E-164	DNAJC8	-1.82E-01	1.00E+00	MEGF6	-4.34E-01	1.00E+00
LOC112579575	9.54E+00	2.98E-164	LOC102411496	-6.69E-01	1.00E+00	CEP55	-3.11E-01	1.00E+00
PLAA	3.11E+00	3.15E-164	RNF115	1.80E-01	1.00E+00	TRAIP	-4.54E-01	1.00E+00
DMRT3	8.90E+00	3.84E-164	CTNNBIP1	2.72E-01	1.00E+00	ISG20	7.79E-01	1.00E+00
MCM10	3.15E+00	3.89E-164	LOC112586208	-4.85E-01	1.00E+00	FAM114A1	-4.44E-01	1.00E+00
MSX2	6.24E+00	4.16E-164	LOC102391941	-6.77E-01	1.00E+00	GPR153	4.61E-01	1.00E+00
LOC102400925	9.93E+00	6.82E-164	LOC102391491	-5.20E-01	1.00E+00	PARP8	4.61E-01	1.00E+00
MXRA5	5.43E+00	7.44E-164	PPTC7	2.42E-01	1.00E+00	PLK4	-3.34E-01	1.00E+00
RGL2	-5.38E+00	7.47E-164	RPS3	-2.64E-01	1.00E+00	AACS	-2.85E-01	1.00E+00
ANO10	3.34E+00	9.19E-164	SKAP2	2.35E-01	1.00E+00	ZNF608	4.76E-01	1.00E+00
SHCBP1	3.20E+00	1.07E-163	UBAP1	1.82E-01	1.00E+00	MYOCD	5.36E-01	1.00E+00
RPIA	3.07E+00	2.01E-163	ZNF660	-7.28E-01	1.00E+00	PRKAG3	-4.46E-01	1.00E+00
ELAVL2	7.38E+00	3.97E-163	POLR2I	-2.49E-01	1.00E+00	AKAP1	-2.91E-01	1.00E+00
SHOC2	2.99E+00	6.97E-163	POLR2H	-2.99E-01	1.00E+00	NACC2	5.68E-01	1.00E+00
SDK1	4.57E+00	7.79E-163	ALG8	2.57E-01	1.00E+00	LOC112582343	-6.99E-01	1.00E+00
COG5	2.26E+00	1.25E-162	SERPINE1	1.98E-01	1.00E+00	LOC102400858	3.87E-01	1.00E+00
EPS8L1	6.56E+00	1.26E-162	COG3	2.27E-01	1.00E+00	GALNT3	6.49E-01	1.00E+00
PLCG2	5.82E+00	1.33E-162	SASS6	3.10E-01	1.00E+00	PHC2	3.18E-01	1.00E+00
SELL	-4.30E+00	1.44E-162	LOC102399267	-8.98E-01	1.00E+00	CRYBG1	5.04E-01	1.00E+00
PSMA1	-3.65E+00	1.78E-162	ABCG4	6.59E-01	1.00E+00	NCAPD2	-2.89E-01	1.00E+00
KMT5B	3.08E+00	2.11E-162	ZFP36L2	-8.38E-01	1.00E+00	PRC1	-3.60E-01	1.00E+00
NKIRAS1	3.14E+00	4.07E-162	LOC102402672	3.71E-01	1.00E+00	NCAM1	5.91E-01	1.00E+00
MIS12	3.08E+00	4.91E-162	DUSP23	-4.26E-01	1.00E+00	MAPKAPK3	4.06E-01	1.00E+00
ETV3L	8.75E+00	5.79E-162	KMT2C	3.14E-01	1.00E+00	KYNU	3.04E-01	1.00E+00
LMF2	-3.43E+00	6.20E-162	ARHGAP35	2.82E-01	1.00E+00	SFMBT2	5.87E-01	1.00E+00
PLCH1	5.24E+00	6.22E-162	MAP3K8	-1.77E+00	1.00E+00	PHLDB1	-4.30E-01	1.00E+00
LOC112580364	7.65E+00	7.29E-162	TTC27	-1.76E-01	1.00E+00	ARHGAP20	3.72E-01	1.00E+00
XPO1	-2.75E+00	7.75E-162	METTL26	-3.35E-01	1.00E+00	EFNB2	4.35E-01	1.00E+00
EGLN1	-2.65E+00	1.18E-161	PAM	-8.12E-01	1.00E+00	STEAP1	4.74E-01	1.00E+00
RRAS2	3.08E+00	1.26E-161	LOC102395800	-5.46E-01	1.00E+00	CUL7	-3.01E-01	1.00E+00
ASZ1	6.06E+00	1.46E-161	RERE	2.99E-01	1.00E+00	CHGA	-3.76E-01	1.00E+00
SOCS7	4.54E+00	1.49E-161	TTBK1	-5.98E-01	1.00E+00	TEAD4	5.34E-01	1.00E+00
LYPD1	6.75E+00	1.92E-161	OTUB2	-4.88E-01	1.00E+00	MDK	3.65E-01	1.00E+00

PAQR7	5.23E+00	2.46E-161	CEP250	3.91E-01	1.00E+00	VMP1	3.06E-01	1.00E+00
NAT10	3.88E+00	2.76E-161	BZW2	-3.76E-01	1.00E+00	ZNF367	-3.78E-01	1.00E+00
RNF115	2.54E+00	3.33E-161	LRRC7	-1.18E+00	1.00E+00	GRAMD1B	-3.38E-01	1.00E+00
NR6A1	4.56E+00	7.64E-161	BAD	-3.48E-01	1.00E+00	ZC3H7B	-3.03E-01	1.00E+00
TAF5L	2.46E+00	1.64E-160	AHR	3.80E-01	1.00E+00	PTGS2	5.82E-01	1.00E+00
CORO6	3.67E+00	1.75E-160	LPIN1	-5.23E-01	1.00E+00	PPM1K	-3.40E-01	1.00E+00
FAM193B	-3.60E+00	2.31E-160	TENT5D	3.46E-01	1.00E+00	ECT2	-3.67E-01	1.00E+00
TTC7A	4.53E+00	3.34E-160	DYNLRB1	-2.98E-01	1.00E+00	PYGL	3.16E-01	1.00E+00
ADORA2A	7.66E+00	3.55E-160	OSTC	-2.83E-01	1.00E+00	PLCB1	5.64E-01	1.00E+00
FOXR1	1.10E+01	4.36E-160	ID2	-1.03E+00	1.00E+00	CNTRL	-3.33E-01	1.00E+00
POFUT2	-4.38E+00	5.63E-160	ZNF79	3.60E-01	1.00E+00	EPHA5	-3.21E-01	1.00E+00
PSMA2	-4.72E+00	5.73E-160	HS6ST3	-7.46E-01	1.00E+00	LRRC61	3.71E-01	1.00E+00
AURKA	5.73E+00	8.35E-160	SSNA1	-2.58E-01	1.00E+00	HS6ST2	-3.04E-01	1.00E+00
RAB22A	2.43E+00	1.03E-159	FAM13C	2.56E-01	1.00E+00	ARSI	5.44E-01	1.00E+00
ACCSL	1.12E+01	1.73E-159	ADGRG6	-1.04E+00	1.00E+00	LOC112587328	-7.31E-01	1.00E+00
SCN4A	8.78E+00	2.02E-159	CHMP2A	-2.47E-01	1.00E+00	HEYL	4.76E-01	1.00E+00
TDRKH	4.54E+00	2.29E-159	CCDC122	-1.04E+00	1.00E+00	ARC	4.06E-01	1.00E+00
PEX11A	3.66E+00	2.40E-159	RCCD1	-3.81E-01	1.00E+00	CHAC1	-4.16E-01	1.00E+00
NUP133	2.46E+00	2.56E-159	FBN2	7.92E-01	1.00E+00	SCD	-4.55E-01	1.00E+00
LOC112582261	9.68E+00	3.53E-159	RORB	-5.42E-01	1.00E+00	LOC102390054	-3.86E-01	1.00E+00
C18H16orf46	3.60E+00	6.06E-159	CCDC136	-3.46E-01	1.00E+00	BAG3	-4.06E-01	1.00E+00
LOC112587841	8.47E+00	9.57E-159	LOC102398503	-6.36E-01	1.00E+00	ADAMTS7	3.27E-01	1.00E+00
RWDD4	-3.39E+00	1.20E-158	LOC112587815	-9.90E-01	1.00E+00	PCDH9	1.01E+00	1.00E+00
ZNF304	2.64E+00	2.25E-158	CSTF1	2.75E-01	1.00E+00	PDLIM3	6.57E-01	1.00E+00
IARS2	-2.61E+00	2.51E-158	TCTN2	-4.62E-01	1.00E+00	SRD5A1	3.28E-01	1.00E+00
KDF1	5.17E+00	2.93E-158	SH3BGRL	-1.30E+00	1.00E+00	PDLIM1	4.69E-01	1.00E+00
SLBP	3.22E+00	3.10E-158	CCDC124	-2.46E-01	1.00E+00	LOC102411782	5.57E-01	1.00E+00
SRCIN1	4.81E+00	3.77E-158	FAM110C	1.39E+00	1.00E+00	PPP1R9B	2.89E-01	1.00E+00
CENPQ	3.30E+00	3.92E-158	RBM7	-5.67E-01	1.00E+00	CACNA2D1	7.23E-01	1.00E+00
BLM	2.91E+00	5.50E-158	FHL2	-1.08E+00	1.00E+00	SLC12A7	-3.58E-01	1.00E+00
FLT1	5.30E+00	7.79E-158	TMEM229B	-3.56E-01	1.00E+00	ZNF512B	-3.69E-01	1.00E+00
ABRAXAS2	2.30E+00	2.53E-157	LPAR2	-4.09E-01	1.00E+00	LARGE2	-5.26E-01	1.00E+00
GIPC1	-4.32E+00	3.52E-157	EEF1B2	-2.36E-01	1.00E+00	CDK14	4.48E-01	1.00E+00
TMEM163	4.58E+00	3.91E-157	CFAP47	-5.91E-01	1.00E+00	LRRC7	4.55E-01	1.00E+00

TOMM34	3.51E+00	4.09E-157	PFDN6	-2.57E-01	1.00E+00	ARL2	3.65E-01	1.00E+00
OSBPL3	7.07E+00	6.33E-157	ATP10B	3.41E-01	1.00E+00	SNTG1	1.10E+00	1.00E+00
VDAC2	-3.26E+00	7.11E-157	EMID1	-3.55E-01	1.00E+00	CAPG	6.51E-01	1.00E+00
DPP7	-6.09E+00	1.47E-156	LOC112587363	8.38E-01	1.00E+00	COL5A2	6.59E-01	1.00E+00
LOC112579901	7.60E+00	1.66E-156	TSPAN12	8.23E-01	1.00E+00	TRAF4	3.57E-01	1.00E+00
MYOM1	5.02E+00	1.88E-156	DTNA	-4.92E-01	1.00E+00	EFHD1	-4.82E-01	1.00E+00
ALDH16A1	-5.13E+00	2.40E-156	KIFC2	4.10E-01	1.00E+00	SPRY2	5.48E-01	1.00E+00
SLC25A15	3.15E+00	4.43E-156	LOC102402714	-5.96E-01	1.00E+00	S100A8	-1.42E+00	1.00E+00
PDCL3	-5.09E+00	6.07E-156	GADD45B	-2.97E-01	1.00E+00	HDHD2	-2.89E-01	1.00E+00
CENPU	5.00E+00	6.26E-156	GPC5	2.23E-01	1.00E+00	LOC112584709	1.05E+00	1.00E+00
LOC112583593	9.68E+00	6.70E-156	GSDME	-4.61E-01	1.00E+00	TMEM169	-7.24E-01	1.00E+00
OSGEP	-3.75E+00	7.73E-156	ARHGAP33	6.20E-01	1.00E+00	LOC102396444	-4.31E-01	1.00E+00
CMTM4	3.44E+00	7.99E-156	SPIRE2	2.24E-01	1.00E+00	SEPT5	2.66E-01	1.00E+00
ZNF691	2.91E+00	9.94E-156	CA10	-8.13E-01	1.00E+00	NCKAP5	8.44E-01	1.00E+00
FBXO25	2.28E+00	1.20E-155	TRIM8	2.56E-01	1.00E+00	UBASH3B	5.67E-01	1.00E+00
KIF18A	3.57E+00	1.33E-155	RPL10A	-3.70E-01	1.00E+00	SCRN1	3.34E-01	1.00E+00
SCPEP1	-3.00E+00	1.36E-155	CORO2A	-7.79E-01	1.00E+00	SLC35F1	8.29E-01	1.00E+00
TRIM26	3.24E+00	1.41E-155	YTHDC2	4.70E-01	1.00E+00	LOC102396042	-4.81E-01	1.00E+00
CCDC196	9.14E+00	2.15E-155	FAM114A1	-1.30E+00	1.00E+00	CALR	-5.55E-01	1.00E+00
CNOT4	2.78E+00	2.21E-155	ZBTB7B	-7.86E-01	1.00E+00	MSL2	-2.77E-01	1.00E+00
RNF212	5.03E+00	2.46E-155	ADM	-5.15E-01	1.00E+00	STARD9	-4.62E-01	1.00E+00
RNF114	4.23E+00	4.32E-155	GRK5	-2.78E-01	1.00E+00	CUEDC1	3.13E-01	1.00E+00
MPP7	3.28E+00	4.45E-155	DGUOK	-2.46E-01	1.00E+00	KATNBL1	3.46E-01	1.00E+00
MAPK4	6.39E+00	5.53E-155	PPP4R2	2.36E-01	1.00E+00	LDB3	-7.05E-01	1.00E+00
EFCAB14	2.61E+00	7.54E-155	GATB	2.27E-01	1.00E+00	GADD45G	-4.29E-01	1.00E+00
LOC112588004	8.85E+00	8.79E-155	FRMPD4	-5.89E-01	1.00E+00	SWAP70	4.15E-01	1.00E+00
HNRNPAB	-3.74E+00	9.58E-155	SH3BP5L	1.70E-01	1.00E+00	CGNL1	-5.14E-01	1.00E+00
SPIRE2	3.07E+00	2.27E-154	TEX15	7.86E-01	1.00E+00	PDE5A	6.41E-01	1.00E+00
PACSIN2	3.01E+00	3.96E-154	LOC102413816	9.43E-01	1.00E+00	KLF8	5.03E-01	1.00E+00
PCYOX1	-3.87E+00	4.06E-154	LOC112578234	-6.09E-01	1.00E+00	SMYD2	3.44E-01	1.00E+00
RABGGTA	-5.05E+00	5.83E-154	RPA2	-2.11E-01	1.00E+00	FUK	-2.90E-01	1.00E+00
LOC112581883	8.19E+00	5.98E-154	MRPS33	-2.54E-01	1.00E+00	LBP	9.13E-01	1.00E+00
LOC102388962	9.89E+00	5.99E-154	HSPH1	-1.96E-01	1.00E+00	PDIA4	-4.28E-01	1.00E+00
LOC112586200	7.77E+00	7.41E-154	MAT2B	1.77E-01	1.00E+00	DSP	-9.06E-01	1.00E+00
LOC102402398	6.90E+00	8.47E-154	UGGT2	1.81E-01	1.00E+00	NRG1	1.15E+00	1.00E+00

CDO1	3.07E+00	8.74E-154	TRANK1	-1.09E+00	1.00E+00	LOC112587762	-5.46E-01	1.00E+00
EMC1	-3.81E+00	9.80E-154	TRMT44	-3.77E-01	1.00E+00	UNC13A	-6.53E-01	1.00E+00
GSG1	6.51E+00	1.03E-153	MID2	-8.73E-01	1.00E+00	REEP1	8.00E-01	1.00E+00
NUP88	2.33E+00	1.26E-153	FOXP1	-2.35E-01	1.00E+00	EMILIN3	-7.01E-01	1.00E+00
STIM2	2.64E+00	1.34E-153	DHRS3	-1.05E+00	1.00E+00	WDR90	-2.90E-01	1.00E+00
MARVELD2	3.85E+00	1.42E-153	LOC112578855	-1.50E+00	1.00E+00	RAB31	3.71E-01	1.00E+00
IDH2	-6.48E+00	1.84E-153	GSPT2	-7.12E-01	1.00E+00	F13A1	1.28E+00	1.00E+00
ANXA2	-5.91E+00	2.36E-153	FGF14	-7.72E-01	1.00E+00	C2H6orf47	3.08E-01	1.00E+00
MED18	3.35E+00	2.39E-153	ILDR2	-3.00E-01	1.00E+00	ITGA5	7.14E-01	1.00E+00
LOC112581246	-4.53E+00	2.42E-153	CAPN2	-8.38E-01	1.00E+00	LRP5	-2.79E-01	1.00E+00
MEI1	6.82E+00	2.42E-153	RAB26	6.46E-01	1.00E+00	LOC112581162	6.62E-01	1.00E+00
TMEM100	7.09E+00	3.44E-153	CXCL12	-4.83E-01	1.00E+00	ADM	4.45E-01	1.00E+00
ARMC7	2.78E+00	6.91E-153	SF3B6	-2.19E-01	1.00E+00	DCAF15	-2.86E-01	1.00E+00
TP53	-3.37E+00	7.23E-153	RNF150	-6.54E-01	1.00E+00	FNDC10	3.30E-01	1.00E+00
LOC102405390	1.06E+01	8.46E-153	LOC102415544	6.12E-01	1.00E+00	WIPF3	-2.84E-01	1.00E+00
SYNJ2	5.31E+00	8.61E-153	NCR3LG1	4.50E-01	1.00E+00	PKIG	3.70E-01	1.00E+00
DPH1	-5.80E+00	9.37E-153	C5H11orf24	2.02E-01	1.00E+00	ZHX2	4.69E-01	1.00E+00
ARFGEF3	4.81E+00	1.06E-152	RPL37A	-3.05E-01	1.00E+00	IRGQ	3.36E-01	1.00E+00
BCL2L14	6.84E+00	1.10E-152	ZNF557	-6.37E-01	1.00E+00	GPX3	-3.41E-01	1.00E+00
PLA2G12A	2.79E+00	1.23E-152	LOC102409132	4.20E-01	1.00E+00	RAB43	2.80E-01	1.00E+00
DIP2C	-3.38E+00	1.28E-152	ANO1	8.30E-01	1.00E+00	GPSM1	2.83E-01	1.00E+00
CHRNA1	8.70E+00	1.37E-152	PURA	7.02E-01	1.00E+00	ZP2	-9.56E-01	1.00E+00
LHX2	3.98E+00	5.34E-152	NPAS1	-4.00E-01	1.00E+00	LOC102395276	-3.17E-01	1.00E+00
C1D	-3.36E+00	5.96E-152	DESI2	5.77E-01	1.00E+00	CAMK2G	-3.13E-01	1.00E+00
TMEM87A	2.96E+00	6.21E-152	LOC112583596	-5.53E-01	1.00E+00	BOK	4.76E-01	1.00E+00
LOC102400938	8.88E+00	1.18E-151	TNFAIP8	-2.46E-01	1.00E+00	TTC39A	7.02E-01	1.00E+00
TYRO3	-6.35E+00	2.01E-151	NCKAP1	2.23E-01	1.00E+00	EP400	-3.08E-01	1.00E+00
ZNF346	3.07E+00	2.47E-151	LOC112579228	-1.45E+00	1.00E+00	PDE4B	1.18E+00	1.00E+00
BCL10	3.01E+00	3.52E-151	LOC112585592	-4.72E-01	1.00E+00	LOC112585239	6.76E-01	1.00E+00
ADAMTSL5	6.65E+00	4.80E-151	ZNF639	-2.31E-01	1.00E+00	SKI	-3.20E-01	1.00E+00
GDAP1	5.24E+00	4.94E-151	MINDY2	3.41E-01	1.00E+00	WBP1L	2.69E-01	1.00E+00
OTX1	9.45E+00	5.35E-151	CTNNA3	2.71E-01	1.00E+00	FCHO1	-4.75E-01	1.00E+00
CAD	-5.54E+00	5.81E-151	RBM20	-4.23E-01	1.00E+00	PLEKHG5	3.70E-01	1.00E+00
CASR	8.67E+00	6.53E-151	NEK6	-6.09E-	1.00E+00	JMY	-2.78E-	1.00E+00

				01			01	
TULP3	2.88E+00	6.73E-151	HSP90B1	1.98E-01	1.00E+00	RNF182	3.87E-01	1.00E+00
LOC112578903	7.15E+00	7.42E-151	CLDN10	-2.43E-01	1.00E+00	CSPG4	-5.08E-01	1.00E+00
UTP23	3.03E+00	7.60E-151	MED15	1.96E-01	1.00E+00	GPT	-4.60E-01	1.00E+00
SHROOM3	3.79E+00	7.85E-151	RBMS1	1.85E-01	1.00E+00	CCDC136	4.57E-01	1.00E+00
INTS9	2.58E+00	8.61E-151	IFNLR1	-5.01E-01	1.00E+00	PRR30	5.69E-01	1.00E+00
CDC37L1	2.71E+00	9.07E-151	KNG1	6.69E-01	1.00E+00	SUSD3	-6.70E-01	1.00E+00
LOC112580918	1.06E+01	1.73E-150	LPAR3	5.33E-01	1.00E+00	LOC102395002	8.50E-01	1.00E+00
BTBD18	7.03E+00	1.81E-150	LOC102414126	-7.08E-01	1.00E+00	NATD1	3.26E-01	1.00E+00
SPRR4	1.15E+01	2.47E-150	HSPB1	-7.58E-01	1.00E+00	QSOX1	4.54E-01	1.00E+00
LOC112577761	5.07E+00	9.17E-150	PGM5	-3.71E-01	1.00E+00	BMP4	6.43E-01	1.00E+00
LOC102402231	8.28E+00	9.30E-150	ACYP2	2.13E-01	1.00E+00	FAM234B	-2.53E-01	1.00E+00
ALG11	2.60E+00	1.35E-149	PSMB8	-4.86E-01	1.00E+00	TTYH3	3.29E-01	1.00E+00
B4GALT7	-4.95E+00	2.06E-149	JOSD1	-2.38E-01	1.00E+00	MTMR4	-3.21E-01	1.00E+00
GRPEL2	2.77E+00	3.14E-149	LOC102391278	-6.49E-01	1.00E+00	RIPOR3	6.99E-01	1.00E+00
HHAT	4.77E+00	3.24E-149	EPSTI1	-1.85E-01	1.00E+00	LOC112578089	6.99E-01	1.00E+00
TADA1	2.69E+00	4.36E-149	ZBTB2	-2.48E-01	1.00E+00	PTPRN2	-3.41E-01	1.00E+00
FUNDC1	-4.18E+00	8.22E-149	LOC102407273	9.39E-01	1.00E+00	KLHL40	4.83E-01	1.00E+00
TTLL4	2.64E+00	2.03E-148	BDP1	6.05E-01	1.00E+00	ARHGAP15	6.77E-01	1.00E+00
TLN1	-3.03E+00	2.20E-148	FAM149A	1.90E-01	1.00E+00	CD109	6.62E-01	1.00E+00
CRYBG3	4.37E+00	3.59E-148	FAM20C	-1.13E+00	1.00E+00	MVB12B	3.24E-01	1.00E+00
SVIL	4.48E+00	3.75E-148	DZIP1	2.97E-01	1.00E+00	MMP16	3.17E-01	1.00E+00
PATL2	9.35E+00	7.90E-148	LOC112581369	-9.53E-01	1.00E+00	PRKAR1B	-3.70E-01	1.00E+00
SLC19A2	2.83E+00	1.07E-147	CNOT4	1.80E-01	1.00E+00	FTSJ1	-4.59E-01	1.00E+00
SLC25A22	3.31E+00	1.48E-147	WEE1	-8.19E-01	1.00E+00	KMT5C	-3.71E-01	1.00E+00
POLG2	2.71E+00	3.00E-147	L3MBTL3	3.22E-01	1.00E+00	COBLL1	6.43E-01	1.00E+00
SNAI3	9.45E+00	4.12E-147	ZSCAN23	-2.74E-01	1.00E+00	FJX1	5.10E-01	1.00E+00
GGNBP2	2.57E+00	4.85E-147	TMCO5A	9.35E-01	1.00E+00	TMEM254	3.10E-01	1.00E+00
B3GNT4	5.17E+00	5.29E-147	DMAP1	-2.41E-01	1.00E+00	LOC102409122	-6.62E-01	1.00E+00
TMEM170A	-3.89E+00	6.11E-147	NPHP3	9.95E-01	1.00E+00	PEX5L	7.51E-01	1.00E+00
ORAI3	-5.45E+00	7.27E-147	HYKK	-3.26E-01	1.00E+00	LOC102392387	6.11E-01	1.00E+00
PPP3CC	-2.99E+00	1.04E-146	LHFPL4	2.74E-01	1.00E+00	LOC102414911	8.88E-01	1.00E+00
MTMR7	3.69E+00	1.45E-146	MOXD1	8.63E-01	1.00E+00	RPL22L1	-2.93E-01	1.00E+00
LOC102409521	9.75E+00	1.47E-146	WDR49	-2.78E-01	1.00E+00	BMPR1B	3.77E-01	1.00E+00
PTGES3L	3.79E+00	3.87E-146	GGN	8.64E-01	1.00E+00	PTPDC1	4.59E-01	1.00E+00

RAB3B	4.38E+00	7.86E-146	HEY2	2.39E-01	1.00E+00	LOC102401017	-3.83E-01	1.00E+00
DIMT1	2.54E+00	9.27E-146	ANKH	-4.20E-01	1.00E+00	VAT1	3.98E-01	1.00E+00
SHOX2	7.98E+00	1.36E-145	LOC112581281	-3.17E-01	1.00E+00	ACADL	-3.01E-01	1.00E+00
TTC39C	2.90E+00	1.49E-145	TPRG1	7.52E-01	1.00E+00	BORA	-3.43E-01	1.00E+00
REC114	3.64E+00	1.53E-145	LOC102390926	-7.39E-01	1.00E+00	LOC102404636	3.76E-01	1.00E+00
LOC102408597	-4.93E+00	2.24E-145	OLFM2	-6.29E-01	1.00E+00	MARCH3	6.22E-01	1.00E+00
LOC102391647	1.12E+01	2.48E-145	LOC102408787	-7.82E-01	1.00E+00	TGFB1	4.70E-01	1.00E+00
GRHL3	7.63E+00	2.49E-145	LYRM4	-3.12E-01	1.00E+00	COL24A1	7.72E-01	1.00E+00
BRIP1	2.81E+00	2.74E-145	ELF1	2.30E-01	1.00E+00	SREBF2	-2.79E-01	1.00E+00
TRAF6	2.74E+00	4.08E-145	LOC112585811	-8.84E-01	1.00E+00	GATA4	-2.73E-01	1.00E+00
ORC5	2.56E+00	4.51E-145	ARRDC5	-1.09E+00	1.00E+00	LOC102396257	-2.84E-01	1.00E+00
EPAS1	3.61E+00	4.75E-145	CHADL	-3.00E-01	1.00E+00	PLSCR3	2.65E-01	1.00E+00
RCBTB1	2.16E+00	6.31E-145	MYC	-1.21E+00	1.00E+00	HMCN2	8.11E-01	1.00E+00
LOC102400376	4.84E+00	7.03E-145	SVIP	3.41E-01	1.00E+00	ALG1	-3.49E-01	1.00E+00
C4H1orf198	-3.38E+00	8.23E-145	TCHH	4.00E-01	1.00E+00	CAVIN3	4.74E-01	1.00E+00
CEP57L1	3.22E+00	1.21E-144	BDH1	-3.18E-01	1.00E+00	KANSL2	-2.41E-01	1.00E+00
LOC102391395	7.55E+00	1.24E-144	RBM27	2.70E-01	1.00E+00	RPL9	-4.12E-01	1.00E+00
DLX4	6.53E+00	1.29E-144	RAB31	-5.45E-01	1.00E+00	TRUB1	-3.44E-01	1.00E+00
SUV39H2	2.66E+00	1.70E-144	TMOD2	2.52E-01	1.00E+00	ZNF667	-3.50E-01	1.00E+00
NCAPD2	-4.11E+00	1.95E-144	BCORL1	3.92E-01	1.00E+00	ND6	4.09E-01	1.00E+00
HNRNPA0	-2.76E+00	2.50E-144	IMPACT	3.15E-01	1.00E+00	GDA	8.35E-01	1.00E+00
LZTR1	-3.40E+00	3.14E-144	VLDLR	2.64E-01	1.00E+00	FAM171A2	4.84E-01	1.00E+00
TIMP2	-4.97E+00	3.84E-144	ZDHHC15	-8.09E-01	1.00E+00	GRIA4	8.54E-01	1.00E+00
IFNGR2	-4.59E+00	3.87E-144	LOC102393133	-3.75E-01	1.00E+00	WDR62	-3.08E-01	1.00E+00
EXD1	4.70E+00	7.11E-144	APP	-2.75E-01	1.00E+00	SYDE1	2.43E-01	1.00E+00
FAM81A	4.11E+00	9.15E-144	TRPM4	5.66E-01	1.00E+00	CCDC184	7.16E-01	1.00E+00
KARS	-2.97E+00	1.01E-143	PIK3AP1	-4.00E-01	1.00E+00	SOD3	3.22E-01	1.00E+00
SLC12A4	-4.71E+00	1.37E-143	MAGOH	-2.25E-01	1.00E+00	ESR2	-4.09E-01	1.00E+00
UHRF2	4.27E+00	1.44E-143	ATP11C	1.76E-01	1.00E+00	SHISA6	7.37E-01	1.00E+00
PCBD1	4.70E+00	1.49E-143	PLK2	2.04E-01	1.00E+00	RPAP3	-2.70E-01	1.00E+00
SNRK	3.93E+00	1.64E-143	GIT1	1.70E-01	1.00E+00	SLC12A5	-6.29E-01	1.00E+00
MB21D2	2.84E+00	2.03E-143	ADAD1	-2.15E-01	1.00E+00	MAP1LC3C	6.85E-01	1.00E+00
LMO7	6.48E+00	2.11E-143	INPP5D	-1.02E+00	1.00E+00	PGPEP1	3.06E-01	1.00E+00

FNDC7	7.28E+00	2.28E-143	SEPT8	-2.59E-01	1.00E+00	KLK4	-4.30E-01	1.00E+00
ST6GALNAC4	-4.18E+00	5.09E-143	RNF214	2.21E-01	1.00E+00	PRLR	-5.33E-01	1.00E+00
CRYBB2	6.74E+00	5.87E-143	PADI2	-4.58E-01	1.00E+00	TRPC3	5.20E-01	1.00E+00
KLHL8	2.75E+00	1.06E-142	C18H19orf48	-5.83E-01	1.00E+00	ARHGAP28	4.42E-01	1.00E+00
LRRC8D	-3.97E+00	1.69E-142	ST8SIA3	-6.85E-01	1.00E+00	MLLT11	3.14E-01	1.00E+00
ANKRD49	2.47E+00	2.02E-142	SLC25A12	2.36E-01	1.00E+00	FAM110B	3.47E-01	1.00E+00
LOC112582161	1.21E+01	2.49E-142	PPIL1	-3.06E-01	1.00E+00	KLF6	-6.39E-01	1.00E+00
MPZL1	-3.05E+00	3.90E-142	LIN7B	4.42E-01	1.00E+00	NOX5	-7.19E-01	1.00E+00
TEAD2	-4.01E+00	3.93E-142	LOC102407934	-1.38E+00	1.00E+00	PRKCE	-3.30E-01	1.00E+00
KPNA7	1.10E+01	4.01E-142	RICTOR	2.79E-01	1.00E+00	SH3BP4	-2.57E-01	1.00E+00
C6H1orf146	5.74E+00	4.76E-142	SERPINE2	-3.14E-01	1.00E+00	ALKBH4	2.68E-01	1.00E+00
SHLD2	3.01E+00	5.27E-142	TCL1B	2.49E-01	1.00E+00	LOC112582978	-4.01E-01	1.00E+00
SYP	5.79E+00	5.45E-142	GRAMD2A	2.24E-01	1.00E+00	LOC102398781	4.73E-01	1.00E+00
COL2A1	7.96E+00	6.63E-142	USP45	-5.89E-01	1.00E+00	GSTM3	-5.07E-01	1.00E+00
SRSF1	-2.71E+00	7.52E-142	HERPUD1	-3.09E-01	1.00E+00	CHEK1	-3.55E-01	1.00E+00
TMEM196	7.05E+00	7.91E-142	HES7	-6.48E-01	1.00E+00	JOSD1	2.86E-01	1.00E+00
ITSN2	3.54E+00	8.20E-142	CTDSPL2	2.97E-01	1.00E+00	STK32B	6.26E-01	1.00E+00
LOC112582281	-5.71E+00	9.13E-142	SLC39A9	2.88E-01	1.00E+00	LOC112584382	6.52E-01	1.00E+00
LOC102405198	6.69E+00	1.18E-141	MRTFA	1.94E-01	1.00E+00	TAGLN	5.17E-01	1.00E+00
AKAP10	3.12E+00	1.21E-141	NCALD	-9.89E-01	1.00E+00	NAALADL2	4.37E-01	1.00E+00
CBFB	-3.52E+00	1.37E-141	ATP6V1G2	-5.00E-01	1.00E+00	PRIMPOL	-2.96E-01	1.00E+00
SEC22A	2.50E+00	1.48E-141	FARS2	-2.60E-01	1.00E+00	CABP7	4.54E-01	1.00E+00
KCNA5	5.30E+00	1.71E-141	RASL10B	2.78E-01	1.00E+00	CLASP1	-3.01E-01	1.00E+00
WDCP	3.19E+00	1.71E-141	ARNTL	2.84E-01	1.00E+00	LOC102408136	-8.75E-01	1.00E+00
RNF19B	3.91E+00	2.97E-141	ETFB	-2.70E-01	1.00E+00	LOC102415072	-8.02E-01	1.00E+00
RCOR2	4.03E+00	5.03E-141	LOC112581557	4.11E-01	1.00E+00	HGFAC	-4.44E-01	1.00E+00
SLF1	3.74E+00	5.80E-141	MALSU1	-3.01E-01	1.00E+00	SH3TC2	-4.81E-01	1.00E+00
CDC25C	3.83E+00	8.00E-141	IP6K1	1.91E-01	1.00E+00	LOC112578790	5.81E-01	1.00E+00
IGFBP7	-5.47E+00	8.34E-141	MLLT1	1.77E-01	1.00E+00	SCN3A	8.35E-01	1.00E+00
MEIOC	7.46E+00	8.79E-141	C12H2orf49	-2.52E-01	1.00E+00	ITGA6	-3.42E-01	1.00E+00
LOC112578845	6.56E+00	9.99E-141	CTSC	1.71E-01	1.00E+00	SLC27A1	-4.18E-01	1.00E+00
NCOA4	2.82E+00	1.06E-140	ACAP3	3.66E-01	1.00E+00	XRCC5	-2.50E-01	1.00E+00
PURB	-3.98E+00	1.08E-140	LOC102415074	-5.84E-01	1.00E+00	SLC20A2	5.14E-01	1.00E+00
LOC112580789	7.88E+00	1.12E-140	LOC102395337	1.53E+00	1.00E+00	LIMK2	3.41E-01	1.00E+00

CELF6	5.60E+00	1.32E-140	C1QL1	4.87E-01	1.00E+00	FNBP4	-2.91E-01	1.00E+00
F11R	-3.17E+00	1.40E-140	LOC112581365	9.13E-01	1.00E+00	FBXO16	6.02E-01	1.00E+00
VWF	-4.84E+00	1.85E-140	TBCEL	2.16E-01	1.00E+00	PRKDC	-3.57E-01	1.00E+00
RNF180	3.41E+00	1.96E-140	EHMT1	2.25E-01	1.00E+00	DNMT3B	-4.28E-01	1.00E+00
CDC6	3.60E+00	2.10E-140	UHMK1	2.79E-01	1.00E+00	AMZ1	6.10E-01	1.00E+00
C4H22orf46	8.10E+00	2.20E-140	LOC102393883	-1.20E+00	1.00E+00	CISD3	4.74E-01	1.00E+00
COMT	-4.62E+00	2.52E-140	DACH1	-1.03E+00	1.00E+00	TNNT3	5.05E-01	1.00E+00
DDX20	2.10E+00	2.63E-140	STKLD1	6.24E-01	1.00E+00	COL27A1	4.55E-01	1.00E+00
LOC102416634	5.88E+00	2.68E-140	ARL5A	3.54E-01	1.00E+00	ATP8B3	-4.94E-01	1.00E+00
DNMBP	4.09E+00	2.91E-140	KCNK13	-4.39E-01	1.00E+00	ADAMTSL1	5.62E-01	1.00E+00
LOC102395706	-4.15E+00	3.38E-140	LOC112580887	-1.17E+00	1.00E+00	GLCE	3.44E-01	1.00E+00
LARS2	-3.56E+00	4.03E-140	MAEL	1.77E-01	1.00E+00	ATF4	-3.99E-01	1.00E+00
BAG1	-3.13E+00	5.31E-140	TDRD1	2.04E-01	1.00E+00	AFF1	-2.94E-01	1.00E+00
EPHB2	-3.30E+00	5.75E-140	TRMT5	-4.24E-01	1.00E+00	LOC102404057	-7.11E-01	1.00E+00
SAT2	-8.28E+00	5.95E-140	CACNB4	-7.02E-01	1.00E+00	CCDC150	-3.88E-01	1.00E+00
SLFNL1	3.85E+00	1.09E-139	MRPL10	-1.87E-01	1.00E+00	STIL	-4.05E-01	1.00E+00
SLC51B	8.70E+00	1.10E-139	ANAPC16	-1.23E+00	1.00E+00	DAAM2	3.53E-01	1.00E+00
TSPAN33	4.29E+00	1.28E-139	VWA5B2	7.38E-01	1.00E+00	PLAC9	4.16E-01	1.00E+00
LOC112585053	4.38E+00	1.47E-139	LOC112581586	5.61E-01	1.00E+00	ZNF706	2.86E-01	1.00E+00
BICDL2	7.31E+00	1.69E-139	CDR2	-3.07E-01	1.00E+00	SLC20A1	-3.95E-01	1.00E+00
PLEKHF2	2.90E+00	1.91E-139	IPO13	2.13E-01	1.00E+00	NEDD4	5.51E-01	1.00E+00
LOC102408966	-5.61E+00	2.07E-139	TMPO	3.20E-01	1.00E+00	SASH1	3.31E-01	1.00E+00
MEGF8	-4.89E+00	2.07E-139	LOC112585027	-4.97E-01	1.00E+00	LOC102410987	-8.83E-01	1.00E+00
LOC112580626	9.23E+00	2.11E-139	AMPD3	3.27E-01	1.00E+00	RCSD1	5.80E-01	1.00E+00
IL17RB	6.75E+00	2.68E-139	HHEX	2.62E-01	1.00E+00	FRMD3	-2.55E-01	1.00E+00
DLL3	7.03E+00	4.43E-139	PSMB6	-3.08E-01	1.00E+00	TAF6L	-2.85E-01	1.00E+00
TNKS1BP1	-4.33E+00	4.48E-139	LOC102390251	6.01E-01	1.00E+00	RAB23	2.69E-01	1.00E+00
DLK2	6.26E+00	5.04E-139	LOC102416651	-3.56E-01	1.00E+00	TTK	-3.31E-01	1.00E+00
UAP1	-3.47E+00	5.16E-139	EMC6	-5.40E-01	1.00E+00	CHTF18	-3.61E-01	1.00E+00
CENPJ	2.60E+00	6.35E-139	ZNF319	3.36E-01	1.00E+00	ARHGEF3	4.34E-01	1.00E+00
CREBZF	-3.75E+00	6.39E-139	ADAM17	-3.03E-01	1.00E+00	STXBP5L	4.66E-01	1.00E+00
C3H17orf53	4.27E+00	6.79E-139	COMMD7	-2.51E-01	1.00E+00	DCN	4.19E-01	1.00E+00
GPC1	-4.84E+00	9.73E-139	LOC102406736	5.87E-01	1.00E+00	SOCS1	-6.28E-01	1.00E+00
LOC112587902	5.50E+00	1.21E-138	LOC102416013	2.29E-01	1.00E+00	SPEG	-3.54E-01	1.00E+00
MARVELD3	4.04E+00	1.22E-138	THOC1	-1.67E-	1.00E+00	CHRNB1	5.11E-01	1.00E+00

				01				
RASL10B	4.98E+00	1.38E-138	NRP1	-6.04E-01	1.00E+00	KLC3	-6.77E-01	1.00E+00
RING1	-4.74E+00	1.68E-138	FBXO11	-6.30E-01	1.00E+00	CD34	6.23E-01	1.00E+00
LOC112581824	9.86E+00	1.74E-138	MUTYH	-2.71E-01	1.00E+00	GCLM	-3.10E-01	1.00E+00
USP2	3.46E+00	2.08E-138	TMEM30A	2.14E-01	1.00E+00	ZNF574	-3.11E-01	1.00E+00
TTLL6	7.63E+00	2.27E-138	SFXN3	-3.37E-01	1.00E+00	GALNT16	3.93E-01	1.00E+00
SP5	5.86E+00	2.66E-138	NOVA1	3.74E-01	1.00E+00	ASF1B	-3.48E-01	1.00E+00
TOP1MT	5.75E+00	2.75E-138	FAM8A1	-2.20E-01	1.00E+00	PLEKHA4	2.53E-01	1.00E+00
IBSP	8.32E+00	3.72E-138	SYTL1	-7.10E-01	1.00E+00	ABCC8	-4.93E-01	1.00E+00
ARFGAP3	-3.57E+00	3.85E-138	S100A10	-3.98E-01	1.00E+00	ERRFI1	5.00E-01	1.00E+00
MVP	5.57E+00	4.32E-138	LOC112578702	2.67E-01	1.00E+00	NID2	5.62E-01	1.00E+00
LOC102391313	4.17E+00	5.03E-138	SLC5A5	-8.02E-01	1.00E+00	SHISA4	4.91E-01	1.00E+00
LTBR	-6.08E+00	5.16E-138	NAB1	3.17E-01	1.00E+00	ARHGAP11A	-3.38E-01	1.00E+00
LOC112577775	-7.27E+00	5.81E-138	LOC102390160	4.48E-01	1.00E+00	PRICKLE1	3.08E-01	1.00E+00
HSF5	4.49E+00	6.25E-138	HYOU1	1.94E-01	1.00E+00	SDC4	4.32E-01	1.00E+00
NAA35	2.30E+00	6.87E-138	FCN3	-7.40E-01	1.00E+00	BRICD5	-6.44E-01	1.00E+00
RAPGEFL1	3.60E+00	7.98E-138	ABCA5	-2.63E-01	1.00E+00	MYLK	-4.13E-01	1.00E+00
NEDD4L	4.43E+00	9.55E-138	ZNF536	-3.29E-01	1.00E+00	ATRNL1	-3.25E-01	1.00E+00
ZNF792	3.10E+00	1.18E-137	PRKAG3	-1.30E+00	1.00E+00	EGLN3	5.98E-01	1.00E+00
ESYT3	5.98E+00	1.28E-137	TNIP2	7.58E-01	1.00E+00	CD200	5.12E-01	1.00E+00
C9H5orf15	-5.47E+00	1.52E-137	ZNF329	2.26E-01	1.00E+00	ATF3	-7.62E-01	1.00E+00
RRAGC	2.59E+00	1.74E-137	PLA2G15	1.96E-01	1.00E+00	HES4	5.57E-01	1.00E+00
MFSD9	2.64E+00	1.93E-137	ZNF84	-6.79E-01	1.00E+00	HJURP	-3.29E-01	1.00E+00
UBE2Q1	3.09E+00	2.19E-137	LOC102414331	2.83E-01	1.00E+00	BUB1B	-3.29E-01	1.00E+00
DEK	-3.28E+00	2.51E-137	LOC102405376	-9.71E-01	1.00E+00	TENM2	8.25E-01	1.00E+00
ZAR1	1.12E+01	3.22E-137	CDH15	-4.82E-01	1.00E+00	MEFV	-7.65E-01	1.00E+00
HRH1	8.68E+00	4.04E-137	MAP4	2.28E-01	1.00E+00	LOC112586103	5.93E-01	1.00E+00
LOC112579167	8.40E+00	4.57E-137	TP53BP1	2.05E-01	1.00E+00	C6H1orf52	-2.88E-01	1.00E+00
LOC112586132	9.81E+00	4.57E-137	LOC112581850	-6.09E-01	1.00E+00	IL34	6.95E-01	1.00E+00
ASF1B	4.17E+00	5.31E-137	KPNA5	7.13E-01	1.00E+00	GRID1	7.60E-01	1.00E+00
C17H12orf49	3.10E+00	1.11E-136	RHOB	-4.08E-01	1.00E+00	ASPHD2	6.00E-01	1.00E+00
RFX5	3.58E+00	1.15E-136	CBFB	-3.76E-01	1.00E+00	GLRX	4.77E-01	1.00E+00
SULF2	4.43E+00	1.95E-136	LOC102407686	-1.62E-01	1.00E+00	LURAP1L	5.17E-01	1.00E+00
EQTN	5.80E+00	2.01E-136	SBNO1	1.99E-01	1.00E+00	LOC112580196	6.83E-01	1.00E+00
PLBD2	-3.36E+00	2.08E-136	FAM91A1	-3.20E-01	1.00E+00	DCLK1	5.66E-01	1.00E+00

LOC102404490	2.39E+00	2.38E-136	FNDC3B	1.69E-01	1.00E+00	LOC102404020	6.13E-01	1.00E+00
PPEF1	2.90E+00	2.38E-136	ZNF597	-1.06E+00	1.00E+00	MEST	-3.56E-01	1.00E+00
LOC102406866	5.50E+00	2.53E-136	NDUFC1	-2.35E-01	1.00E+00	PTPN7	-5.45E-01	1.00E+00
PHF20L1	-4.16E+00	2.60E-136	CEP295	-2.86E-01	1.00E+00	LOC112584562	7.02E-01	1.00E+00
ALDOB	5.79E+00	3.35E-136	CEP131	-2.90E-01	1.00E+00	LOC102389143	7.42E-01	1.00E+00
GCA	4.79E+00	4.07E-136	ZNF574	1.78E-01	1.00E+00	ARFGAP3	-3.10E-01	1.00E+00
ASMTL	-5.01E+00	4.55E-136	SMIM11A	-2.85E-01	1.00E+00	CASZ1	4.01E-01	1.00E+00
LOC112586491	8.55E+00	4.64E-136	LOC102416591	2.05E-01	1.00E+00	AKIP1	4.56E-01	1.00E+00
MTF1	3.45E+00	4.72E-136	FGA	-5.16E-01	1.00E+00	IQSEC1	-3.18E-01	1.00E+00
ITM2C	-4.08E+00	4.78E-136	CTSZ	-2.10E-01	1.00E+00	SORBS1	-4.12E-01	1.00E+00
C6H1orf56	6.73E+00	5.07E-136	SEC14L1	2.30E-01	1.00E+00	LOC102415281	-3.78E-01	1.00E+00
RND1	4.65E+00	5.66E-136	ALG14	2.27E-01	1.00E+00	VEPH1	-4.76E-01	1.00E+00
UCKL1	-3.40E+00	6.12E-136	CXCR5	-9.53E-01	1.00E+00	CHCHD6	-5.20E-01	1.00E+00
TAF5	2.49E+00	1.04E-135	PDP2	2.02E-01	1.00E+00	IL6R	-4.47E-01	1.00E+00
ACIN1	-3.12E+00	1.08E-135	CD3EAP	-3.20E-01	1.00E+00	TLE3	3.15E-01	1.00E+00
SLC39A7	-3.46E+00	1.27E-135	FRMPD1	6.29E-01	1.00E+00	TRABD2B	7.17E-01	1.00E+00
POLM	3.12E+00	1.45E-135	MDFIC	2.72E-01	1.00E+00	ALDH1A1	-7.14E-01	1.00E+00
NUDT21	2.26E+00	1.76E-135	FAM171A1	-7.12E-01	1.00E+00	WDR73	-3.11E-01	1.00E+00
LOC102402689	2.39E+00	2.36E-135	LOC102408579	4.30E-01	1.00E+00	LOC102394903	-3.76E-01	1.00E+00
INTS6L	-4.87E+00	3.00E-135	DNAJC19	-3.14E-01	1.00E+00	CDAN1	-2.97E-01	1.00E+00
LOC102413798	-3.75E+00	4.30E-135	MOB1A	5.31E-01	1.00E+00	MX2	7.80E-01	1.00E+00
PEG10	-3.73E+00	5.69E-135	LOC102403745	-9.36E-01	1.00E+00	LOC102410605	5.70E-01	1.00E+00
IRS1	-3.87E+00	5.77E-135	LOC112581565	-1.18E+00	1.00E+00	LOC112582327	3.82E-01	1.00E+00
CIRBP	-3.41E+00	5.90E-135	RPRD2	2.57E-01	1.00E+00	GLDN	-1.04E+00	1.00E+00
WISP3	6.02E+00	7.15E-135	SLC12A7	3.10E-01	1.00E+00	FADS2	-2.95E-01	1.00E+00
TPM4	-4.82E+00	8.00E-135	LOC102412702	5.92E-01	1.00E+00	LOC102391549	5.69E-01	1.00E+00
LOC102413367	4.68E+00	9.06E-135	CREBL2	2.23E-01	1.00E+00	LOC102416352	3.36E-01	1.00E+00
MAN1A1	-4.19E+00	1.12E-134	LOC112583725	-8.56E-01	1.00E+00	LOC112587874	4.87E-01	1.00E+00
SCP2	-2.78E+00	1.48E-134	DMGDH	5.62E-01	1.00E+00	SSUH2	6.81E-01	1.00E+00
ZFP69	2.74E+00	1.57E-134	NTS	-9.90E-01	1.00E+00	LOC112585333	-5.53E-01	1.00E+00
SPDL1	2.85E+00	1.65E-134	LOC102401256	-9.76E-01	1.00E+00	PSPH	-3.98E-01	1.00E+00
DHX9	-3.18E+00	2.79E-134	BLVRA	-7.52E-01	1.00E+00	SEZ6L	8.01E-01	1.00E+00
LOC102400631	6.89E+00	3.65E-134	C6H1orf146	-3.34E-01	1.00E+00	TBC1D30	4.98E-01	1.00E+00

CDCP1	-2.92E+00	4.02E-134	CHCHD1	-4.04E-01	1.00E+00	SLC37A1	4.33E-01	1.00E+00
STK17A	4.02E+00	4.14E-134	GPR12	-5.11E-01	1.00E+00	NLRP12	-5.73E-01	1.00E+00
SEPT1	3.55E+00	6.38E-134	SLF1	2.64E-01	1.00E+00	VWA5A	5.03E-01	1.00E+00
ABCC9	-2.44E+00	1.10E-133	PHACTR4	2.65E-01	1.00E+00	EOGT	-3.23E-01	1.00E+00
EPB41L4B	3.22E+00	1.57E-133	RPS8	-2.47E-01	1.00E+00	LOC112579873	4.85E-01	1.00E+00
AKTIP	3.55E+00	2.41E-133	RRAS2	1.69E-01	1.00E+00	ARHGAP31	3.02E-01	1.00E+00
RAB11FIP4	5.47E+00	2.65E-133	DPY30	-2.73E-01	1.00E+00	DERL3	-6.42E-01	1.00E+00
WIPF2	3.26E+00	3.35E-133	POMC	-3.62E-01	1.00E+00	SREK1	-2.70E-01	1.00E+00
CAMK2G	2.53E+00	3.61E-133	AVIL	-3.74E-01	1.00E+00	PLEC	3.58E-01	1.00E+00
EIF3A	-2.75E+00	3.94E-133	BRAP	-1.87E-01	1.00E+00	LOC102391948	-6.57E-01	1.00E+00
TERB1	4.49E+00	3.97E-133	LOC112578741	1.15E+00	1.00E+00	HNRNPA2B1	-2.98E-01	1.00E+00
DENND1B	2.37E+00	4.45E-133	CALR	1.71E-01	1.00E+00	PLD3	3.03E-01	1.00E+00
TMEM123	-5.04E+00	6.32E-133	SMIM8	-2.68E-01	1.00E+00	LOC112587817	5.64E-01	1.00E+00
TMEM98	-5.81E+00	7.31E-133	IFNGR1	-2.61E-01	1.00E+00	TMEM39A	-2.46E-01	1.00E+00
RND2	4.04E+00	7.37E-133	ZDHHC24	-2.71E-01	1.00E+00	TMEM164	-3.09E-01	1.00E+00
SREBF1	-4.10E+00	8.21E-133	LAMC2	-6.77E-01	1.00E+00	HSP90B1	-4.78E-01	1.00E+00
LMAN1	-2.85E+00	8.81E-133	PRRC2C	3.62E-01	1.00E+00	LOC112583584	-6.00E-01	1.00E+00
CT83	8.65E+00	9.49E-133	FAM168B	1.79E-01	1.00E+00	RIN2	4.16E-01	1.00E+00
CCDC7	5.83E+00	1.01E-132	MEI1	3.16E-01	1.00E+00	PROKR1	7.52E-01	1.00E+00
RIPK4	7.32E+00	1.03E-132	SCAP	1.85E-01	1.00E+00	LOC112584008	4.86E-01	1.00E+00
TAPBP	-3.27E+00	1.18E-132	FBP1	-8.66E-01	1.00E+00	SPTBN4	-3.39E-01	1.00E+00
RASA1	3.38E+00	1.69E-132	TMEM126A	-2.96E-01	1.00E+00	PRPSAP2	-2.57E-01	1.00E+00
SLC39A1	-2.70E+00	1.99E-132	ARHGEF18	2.70E-01	1.00E+00	CAMKK2	-4.44E-01	1.00E+00
CTXN3	7.20E+00	2.16E-132	ATG16L1	2.81E-01	1.00E+00	IQGAP3	-3.52E-01	1.00E+00
RRNAD1	-4.72E+00	2.69E-132	RBMS3	-2.66E-01	1.00E+00	SPARCL1	7.00E-01	1.00E+00
RBFA	-3.27E+00	4.56E-132	TRIR	-2.60E-01	1.00E+00	KLHL17	-3.03E-01	1.00E+00
LOC112583679	6.67E+00	6.79E-132	SKP2	-5.03E-01	1.00E+00	PXDN	3.96E-01	1.00E+00
SPATA5	2.64E+00	6.81E-132	CCDC196	-3.38E-01	1.00E+00	CELSR2	-2.81E-01	1.00E+00
WASF3	3.86E+00	7.01E-132	LOC102396947	-1.05E+00	1.00E+00	PTPRC	-4.16E-01	1.00E+00
PLPP7	-4.59E+00	7.22E-132	WDR34	-2.78E-01	1.00E+00	CAMK2B	5.44E-01	1.00E+00
HSPA4L	2.73E+00	7.70E-132	CAMKMT	-3.25E-01	1.00E+00	F5	5.61E-01	1.00E+00
LOC102401139	1.11E+01	9.16E-132	CACFD1	2.72E-01	1.00E+00	ARHGEF2	3.74E-01	1.00E+00
ADGRG2	-3.58E+00	1.19E-131	HMGB3	2.35E-01	1.00E+00	FANCG	-2.68E-01	1.00E+00
LOC102405658	-4.28E+00	1.56E-131	ATP2B2	-6.12E-01	1.00E+00	PBK	-3.30E-01	1.00E+00
SNRNP40	-	1.57E-131	SSBP2	3.14E-01	1.00E+00	MAP2K6	-4.10E-	1.00E+00

	3.75E+00						01	
SERPINF2	-7.46E+00	1.80E-131	AEBP2	2.68E-01	1.00E+00	SLC22A3	-2.89E-01	1.00E+00
WBP2	3.37E+00	1.86E-131	PIMREG	-4.47E-01	1.00E+00	EPHB4	2.41E-01	1.00E+00
PRRG4	6.96E+00	1.99E-131	LOC112586543	-3.89E-01	1.00E+00	IRF9	-3.03E-01	1.00E+00
SLC25A31	8.96E+00	2.14E-131	CD99	-8.10E-01	1.00E+00	PLXDC1	6.85E-01	1.00E+00
CRYBG1	5.05E+00	2.21E-131	HOMER2	3.24E-01	1.00E+00	PRPS2	-3.14E-01	1.00E+00
KLHL7	3.47E+00	2.24E-131	IPMK	4.06E-01	1.00E+00	GPT2	-3.08E-01	1.00E+00
CCDC117	2.17E+00	3.02E-131	TRIM14	-9.26E-01	1.00E+00	KCNJ2	7.14E-01	1.00E+00
LOC102414988	8.80E+00	3.34E-131	ZBTB40	2.43E-01	1.00E+00	NES	3.87E-01	1.00E+00
CCDC146	5.99E+00	4.35E-131	LOC102401100	-9.80E-01	1.00E+00	PLTP	-6.58E-01	1.00E+00
ZFPM2	-4.85E+00	4.35E-131	LOC102394536	-4.54E-01	1.00E+00	LAMA3	7.50E-01	1.00E+00
PLEKHG1	3.18E+00	5.40E-131	JAML	2.99E-01	1.00E+00	APAF1	-3.38E-01	1.00E+00
USB1	2.57E+00	6.05E-131	FCER1G	-5.41E-01	1.00E+00	ATP8B1	4.07E-01	1.00E+00
LOC112582097	7.56E+00	7.37E-131	MAP1S	2.10E-01	1.00E+00	LOC112585309	-5.27E-01	1.00E+00
TAL1	6.70E+00	8.70E-131	SLCO3A1	-1.86E-01	1.00E+00	ETV4	5.31E-01	1.00E+00
KRTCAP3	4.77E+00	9.00E-131	HYAL3	6.10E-01	1.00E+00	IFI44L	6.96E-01	1.00E+00
TRAPPC2	2.80E+00	1.14E-130	BCAT1	-2.32E-01	1.00E+00	LOC102395004	5.76E-01	1.00E+00
KBTBD8	4.30E+00	1.29E-130	NUDT16	-3.06E-01	1.00E+00	GPR156	5.47E-01	1.00E+00
LIMA1	5.64E+00	1.48E-130	MDGA2	-1.06E+00	1.00E+00	ACTN2	-3.43E-01	1.00E+00
LOC102408340	3.09E+00	1.70E-130	C5H1orf112	1.92E-01	1.00E+00	ANXA3	5.54E-01	1.00E+00
MAP4K1	4.04E+00	2.00E-130	PAX6	5.33E-01	1.00E+00	CDH1	6.60E-01	1.00E+00
SLC35A1	2.30E+00	2.13E-130	SFPQ	-2.63E-01	1.00E+00	LACTB	-2.54E-01	1.00E+00
CFB	5.74E+00	2.63E-130	ERICH1	3.29E-01	1.00E+00	PFKP	2.80E-01	1.00E+00
SSH3	3.04E+00	3.07E-130	SLC30A3	4.11E-01	1.00E+00	FAM92B	-6.70E-01	1.00E+00
LOC102415609	3.02E+00	3.37E-130	TP53	-3.52E-01	1.00E+00	PHACTR1	9.30E-01	1.00E+00
ILK	-2.97E+00	4.54E-130	ZER1	3.90E-01	1.00E+00	LOC102410916	4.44E-01	1.00E+00
UBLCP1	2.56E+00	5.54E-130	SOCS4	2.55E-01	1.00E+00	LOC112581246	2.47E-01	1.00E+00
ZBTB33	3.16E+00	6.16E-130	WIZ	2.14E-01	1.00E+00	TAF1C	-3.23E-01	1.00E+00
ANGPT4	-7.02E+00	9.21E-130	SLC23A2	-2.61E-01	1.00E+00	ANGPT4	-3.71E-01	1.00E+00
LOC102406337	-5.04E+00	2.26E-129	CIC	1.94E-01	1.00E+00	FKBP5	3.16E-01	1.00E+00
ZGPAT	-4.77E+00	2.55E-129	FOXN4	-3.99E-01	1.00E+00	NEMP2	-2.81E-01	1.00E+00
LOC102389936	8.42E+00	2.72E-129	CASQ1	-4.12E-01	1.00E+00	RNPEP	-2.59E-01	1.00E+00
ERICH3	-4.94E+00	3.66E-129	ALPK2	-5.30E-01	1.00E+00	SPHK1	4.21E-01	1.00E+00
ZNF322	-4.90E+00	4.04E-129	GPR107	2.76E-01	1.00E+00	SLTM	-2.65E-01	1.00E+00
GLB1	-	5.14E-129	RMND1	-2.24E-	1.00E+00	LOC102398747	5.76E-01	1.00E+00

	5.11E+00			01				
KHDRBS3	3.77E+00	5.37E-129	LOC102403941	7.54E-01	1.00E+00	METTL16	-3.37E-01	1.00E+00
TDP1	2.71E+00	5.61E-129	ARL3	-3.96E-01	1.00E+00	LOC102405926	1.18E+00	1.00E+00
RAPGEF4	4.56E+00	2.07E-128	KSR2	-3.57E-01	1.00E+00	ID3	-4.64E-01	1.00E+00
LOC102392550	2.62E+00	2.09E-128	TPT1	-3.05E-01	1.00E+00	NCBP3	-2.30E-01	1.00E+00
LOC112579996	1.04E+01	2.88E-128	SP2	2.08E-01	1.00E+00	DCLK2	4.01E-01	1.00E+00
LOC102406036	7.24E+00	4.38E-128	APOB	-3.03E-01	1.00E+00	SLC39A14	-3.30E-01	1.00E+00
TFF2	-8.47E+00	5.10E-128	FYTTD1	2.10E-01	1.00E+00	MMS19	-2.22E-01	1.00E+00
SFN	4.87E+00	6.55E-128	CAMK1D	-4.49E-01	1.00E+00	TNFRSF6B	4.22E-01	1.00E+00
LOC112580286	4.78E+00	6.67E-128	RFT1	2.30E-01	1.00E+00	LOC112587357	7.77E-01	1.00E+00
LOC102392521	-6.05E+00	8.10E-128	SVOP	-7.46E-01	1.00E+00	CCDC85B	4.94E-01	1.00E+00
SLC6A1	7.04E+00	8.99E-128	UMPS	-2.67E-01	1.00E+00	RTEL1	-2.50E-01	1.00E+00
UBQLN4	-2.97E+00	1.45E-127	PTGFRN	2.46E-01	1.00E+00	CENPU	-3.94E-01	1.00E+00
GTF2F2	2.10E+00	1.54E-127	PSMG1	-2.09E-01	1.00E+00	THNSL2	-5.27E-01	1.00E+00
SDF4	-3.74E+00	1.55E-127	TRAF5	-6.66E-01	1.00E+00	MGST1	-3.17E-01	1.00E+00
SEPHS1	-3.05E+00	1.69E-127	LOC112578656	7.27E-01	1.00E+00	KRT8	5.40E-01	1.00E+00
EIF4EBP1	-4.04E+00	2.17E-127	RPS18	-2.32E-01	1.00E+00	LOC102400860	5.50E-01	1.00E+00
LOC112580880	9.66E+00	3.03E-127	BAZ2A	2.47E-01	1.00E+00	PLPP3	-5.26E-01	1.00E+00
LOC112579910	8.04E+00	3.33E-127	KDELC1	-3.14E-01	1.00E+00	LOC102412899	-3.10E-01	1.00E+00
RASSF3	3.82E+00	3.58E-127	LOC102402668	2.14E-01	1.00E+00	MMP1	9.55E-01	1.00E+00
LCMT2	3.56E+00	3.74E-127	DUSP1	-2.07E-01	1.00E+00	RBM5	-2.55E-01	1.00E+00
KCTD5	2.38E+00	3.86E-127	RHOBTB1	-3.44E-01	1.00E+00	ROGDI	-3.70E-01	1.00E+00
MMD	-3.71E+00	4.03E-127	RPP30	-2.21E-01	1.00E+00	NCAPG	-2.59E-01	1.00E+00
LTF	-6.04E+00	4.09E-127	GUCY1A2	-6.00E-01	1.00E+00	ARNT2	3.80E-01	1.00E+00
PDE6D	2.73E+00	4.80E-127	HPSE2	-9.25E-01	1.00E+00	IER2	-6.06E-01	1.00E+00
PIAS4	2.19E+00	5.74E-127	ACSL1	-6.85E-01	1.00E+00	SDC2	2.76E-01	1.00E+00
BMP15	1.15E+01	6.10E-127	SYDE1	-7.75E-01	1.00E+00	LOC102416020	8.14E-01	1.00E+00
NCK2	2.41E+00	6.16E-127	LRRK2	-4.49E-01	1.00E+00	LOC102412798	5.11E-01	1.00E+00
CNIH3	-4.31E+00	6.96E-127	WWC2	2.14E-01	1.00E+00	XPO4	-2.38E-01	1.00E+00
COMMD8	2.86E+00	7.16E-127	PFAS	-7.03E-01	1.00E+00	SUCLG2	-2.43E-01	1.00E+00
EHD2	-6.43E+00	7.20E-127	SLC10A2	-8.22E-01	1.00E+00	C24H7orf61	5.22E-01	1.00E+00
SAT1	-4.36E+00	8.16E-127	RNF38	2.00E-01	1.00E+00	NR5A1	-2.89E-01	1.00E+00
ATN1	-2.65E+00	8.45E-127	ARPP21	-3.23E-01	1.00E+00	NUCB2	-3.68E-01	1.00E+00
MOGS	-	1.31E-126	NXPH4	-6.92E-	1.00E+00	PHACTR2	5.31E-01	1.00E+00

	3.74E+00			01				
CALCR	8.19E+00	1.37E-126	CORO1A	-8.76E-01	1.00E+00	IGFBP6	3.21E-01	1.00E+00
CORO1B	-5.66E+00	1.37E-126	FBXO30	2.50E-01	1.00E+00	TTC21A	-3.61E-01	1.00E+00
LOC102396257	-3.87E+00	1.62E-126	CUL4B	1.74E-01	1.00E+00	LAMB1	4.41E-01	1.00E+00
OCIAD1	-2.78E+00	1.66E-126	TPM4	-3.53E-01	1.00E+00	LOC102398085	-6.09E-01	1.00E+00
BBS1	-4.49E+00	2.21E-126	PKP3	9.24E-01	1.00E+00	PDGFRA	4.47E-01	1.00E+00
FBXO34	3.10E+00	2.92E-126	CPEB2	-5.31E-01	1.00E+00	KLF2	-8.44E-01	1.00E+00
LINGO3	6.26E+00	3.07E-126	FAM193A	1.87E-01	1.00E+00	SLITRK2	3.97E-01	1.00E+00
ZNF398	3.18E+00	3.47E-126	RPL13A	-2.62E-01	1.00E+00	LSMEM1	9.63E-01	1.00E+00
MADCAM1	7.28E+00	4.47E-126	KIF5B	2.45E-01	1.00E+00	RHOB	-5.78E-01	1.00E+00
LOC112579090	1.03E+01	5.64E-126	SPEN	2.52E-01	1.00E+00	EPOR	-3.41E-01	1.00E+00
TTC39A	5.18E+00	6.69E-126	MAN1A1	-3.45E-01	1.00E+00	DNAH3	6.81E-01	1.00E+00
CDHR1	5.04E+00	7.18E-126	LOC102409513	1.52E+00	1.00E+00	CLCF1	5.74E-01	1.00E+00
LOC102410605	4.80E+00	7.63E-126	GNAL	-2.25E-01	1.00E+00	SYVN1	-2.31E-01	1.00E+00
RBM38	3.05E+00	7.97E-126	LOC102410310	-9.06E-01	1.00E+00	SLC8A1	4.03E-01	1.00E+00
TMEM254	-3.69E+00	8.43E-126	MAP3K14	2.01E-01	1.00E+00	TPPP3	-4.60E-01	1.00E+00
RRBP1	-3.37E+00	1.23E-125	PRDX5	-2.27E-01	1.00E+00	ZMYND8	-3.16E-01	1.00E+00
CCT6A	-4.34E+00	1.28E-125	CRYZ	2.26E-01	1.00E+00	RAB3D	2.56E-01	1.00E+00
ARHGEF17	-4.26E+00	1.41E-125	NIP7	2.57E-01	1.00E+00	VPS33B	-2.51E-01	1.00E+00
LOC102403866	7.45E+00	1.85E-125	BLOC1S2	-5.46E-01	1.00E+00	TRPC6	4.68E-01	1.00E+00
CELF4	6.22E+00	1.98E-125	CAPN15	3.84E-01	1.00E+00	G0S2	-5.30E-01	1.00E+00
ILF3	-2.65E+00	2.06E-125	WASF1	2.63E-01	1.00E+00	SF1	-2.76E-01	1.00E+00
SRSF11	-2.74E+00	2.58E-125	ACTB	-1.90E-01	1.00E+00	MEX3D	3.84E-01	1.00E+00
SLC29A1	-3.87E+00	2.63E-125	BLK	-4.99E-01	1.00E+00	gen-01	-3.24E-01	1.00E+00
PRKCSH	-4.14E+00	2.70E-125	QSOX1	2.00E-01	1.00E+00	RIPK2	2.50E-01	1.00E+00
MAP7D1	-3.57E+00	3.11E-125	DISP2	-7.49E-01	1.00E+00	DENND1A	-2.33E-01	1.00E+00
BCAT1	7.76E+00	3.12E-125	SEMA4C	-9.60E-01	1.00E+00	LOC112578394	6.59E-01	1.00E+00
MAPK8IP3	2.79E+00	3.50E-125	TRERF1	-5.00E-01	1.00E+00	LOC102393680	3.83E-01	1.00E+00
PPARG	-3.18E+00	5.15E-125	FNBP1L	2.11E-01	1.00E+00	PLEKHG3	-2.60E-01	1.00E+00
RFC3	3.14E+00	5.25E-125	N6AMT1	-4.54E-01	1.00E+00	ODF2	-2.56E-01	1.00E+00
SCG3	4.95E+00	5.28E-125	LOC102415914	6.27E-01	1.00E+00	SVOPL	3.95E-01	1.00E+00
NECTIN1	-5.82E+00	5.80E-125	PACS1	2.35E-01	1.00E+00	LHFPL6	6.74E-01	1.00E+00
LOC102395812	-3.46E+00	8.18E-125	LOC102390560	-5.92E-01	1.00E+00	PDIA2	-5.58E-01	1.00E+00
SMIM19	-	8.26E-125	BAP1	1.55E-01	1.00E+00	LOC102396947	3.60E-01	1.00E+00

	3.93E+00							
ZC3H7B	-3.61E+00	8.97E-125	LOC112587832	-1.08E+00	1.00E+00	ACCSL	-7.39E-01	1.00E+00
WDR20	2.66E+00	1.14E-124	ADAR	2.51E-01	1.00E+00	LOC102398333	-6.57E-01	1.00E+00
FAM192A	3.15E+00	1.21E-124	LOC102397766	-3.81E-01	1.00E+00	ADCK5	-2.97E-01	1.00E+00
LGALSL	-5.01E+00	1.28E-124	MYBBP1A	2.06E-01	1.00E+00	RNF138	-3.03E-01	1.00E+00
RBCK1	-4.51E+00	1.66E-124	TMEFF1	2.87E-01	1.00E+00	LOC102408478	-6.92E-01	1.00E+00
EPOR	4.07E+00	2.00E-124	TNR	-7.96E-01	1.00E+00	GFRA1	6.60E-01	1.00E+00
CCSAP	2.80E+00	3.14E-124	LOC102406311	-3.67E-01	1.00E+00	ARHGAP26	-3.80E-01	1.00E+00
BZW2	-3.27E+00	5.71E-124	LOC102401443	3.48E-01	1.00E+00	EGFL7	-8.95E-01	1.00E+00
CLCN4	3.24E+00	5.88E-124	MBIP	5.48E-01	1.00E+00	SSH1	-2.69E-01	1.00E+00
IL1R2	7.16E+00	6.37E-124	ITGB1BP2	-3.15E-01	1.00E+00	KLF13	2.83E-01	1.00E+00
ZNF436	2.86E+00	8.48E-124	LOC102410458	-8.10E-01	1.00E+00	LOC102394141	-4.37E-01	1.00E+00
YBX3	-4.20E+00	8.53E-124	EFCAB7	-3.89E-01	1.00E+00	NAGLU	-4.73E-01	1.00E+00
SMOX	-5.99E+00	1.24E-123	GPR63	2.62E-01	1.00E+00	TUFT1	-3.48E-01	1.00E+00
PPA2	-4.32E+00	1.24E-123	RFC4	-1.75E-01	1.00E+00	DUSP4	7.43E-01	1.00E+00
RNF8	3.07E+00	1.40E-123	ENG	-5.68E-01	1.00E+00	PARD3B	-2.53E-01	1.00E+00
MARCKSL1	-3.42E+00	2.34E-123	NOP2	1.53E-01	1.00E+00	TEX15	-3.82E-01	1.00E+00
LOC112583592	8.11E+00	2.47E-123	LOC102410915	-5.61E-01	1.00E+00	LOC112585053	-3.57E-01	1.00E+00
GPR179	5.33E+00	2.88E-123	SLC16A2	5.02E-01	1.00E+00	RDH5	5.90E-01	1.00E+00
STAG3	5.46E+00	3.66E-123	HNRNPA0	-2.47E-01	1.00E+00	FADS3	4.47E-01	1.00E+00
NUP210	-5.11E+00	4.78E-123	ZFHX4	7.02E-01	1.00E+00	ADAM23	5.58E-01	1.00E+00
PSPN	6.49E+00	5.57E-123	PPP1R26	5.15E-01	1.00E+00	HIPK4	5.02E-01	1.00E+00
DDIAS	2.53E+00	6.02E-123	JAKMIP1	-7.17E-01	1.00E+00	MANF	-4.07E-01	1.00E+00
BTBD9	2.31E+00	6.05E-123	ANO6	-2.22E-01	1.00E+00	WFIKKN2	-6.41E-01	1.00E+00
FHDC1	5.64E+00	7.35E-123	DYNLT1	-2.09E-01	1.00E+00	PPARD	-2.19E-01	1.00E+00
CTDSP1	-2.72E+00	7.58E-123	SIMC1	2.34E-01	1.00E+00	SIDT2	3.17E-01	1.00E+00
BLOC1S5	2.29E+00	1.13E-122	SOCS1	-3.53E-01	1.00E+00	GLIS2	2.42E-01	1.00E+00
LOC112583717	6.15E+00	1.26E-122	LHB	-5.57E-01	1.00E+00	HMMR	-3.29E-01	1.00E+00
DCTD	2.86E+00	1.34E-122	COX3	-3.91E-01	1.00E+00	BCAR1	2.54E-01	1.00E+00
GABARAPL1	-3.51E+00	1.44E-122	HIP1	2.18E-01	1.00E+00	CDCA7	-3.09E-01	1.00E+00
SMURF2	2.28E+00	1.81E-122	SOCS6	2.64E-01	1.00E+00	TGFB3	5.12E-01	1.00E+00
BACE1	-3.11E+00	2.62E-122	LOC112587900	-8.08E-01	1.00E+00	RNF128	6.96E-01	1.00E+00
DESI1	2.67E+00	2.95E-122	LOC102409148	-7.01E-01	1.00E+00	USP50	4.88E-01	1.00E+00
CAP2	-	4.18E-122	ARID1A	3.25E-01	1.00E+00	CNKSR3	3.76E-01	1.00E+00

	5.07E+00							
SLC35B4	2.34E+00	4.28E-122	RPL22	-3.02E-01	1.00E+00	SAMD4A	5.05E-01	1.00E+00
CA9	3.97E+00	7.43E-122	PPM1L	-2.83E-01	1.00E+00	PLCL1	4.59E-01	1.00E+00
ZNF219	-3.62E+00	7.49E-122	TSNARE1	2.56E-01	1.00E+00	TECR	-3.32E-01	1.00E+00
FBXL18	2.53E+00	8.13E-122	LOC112581826	5.44E-01	1.00E+00	ZDHHC13	-2.33E-01	1.00E+00
ORC6	3.10E+00	1.01E-121	PPIP5K2	2.29E-01	1.00E+00	LOC102396303	-3.46E-01	1.00E+00
SFPQ	-3.80E+00	1.16E-121	ARL8A	-7.31E-01	1.00E+00	SRSF5	-2.77E-01	1.00E+00
A4GALT	-7.34E+00	1.26E-121	UNC5A	7.62E-01	1.00E+00	EFNA1	3.01E-01	1.00E+00
ZHX2	3.34E+00	1.44E-121	C2H6orf89	1.56E-01	1.00E+00	NPY1R	6.13E-01	1.00E+00
SHBG	-8.03E+00	1.52E-121	IVD	2.78E-01	1.00E+00	NEXN	4.38E-01	1.00E+00
SMTN	-5.58E+00	2.07E-121	ZMYM3	2.35E-01	1.00E+00	LOC112581361	5.95E-01	1.00E+00
GMPS	2.86E+00	2.37E-121	RTKN	-4.90E-01	1.00E+00	ANK2	3.77E-01	1.00E+00
OLA1	-3.20E+00	2.41E-121	NOP14	-1.90E-01	1.00E+00	SKP2	-2.75E-01	1.00E+00
HDGFL1	9.70E+00	3.03E-121	ATRAID	-2.73E-01	1.00E+00	DUOX1	5.06E-01	1.00E+00
INPPL1	-2.60E+00	3.25E-121	EXOSC10	-1.73E-01	1.00E+00	LOC102388984	5.83E-01	1.00E+00
NUFIP1	2.21E+00	3.34E-121	SYMPK	1.87E-01	1.00E+00	ESRRA	2.60E-01	1.00E+00
CLIC1	-4.26E+00	3.68E-121	OTUD5	1.59E-01	1.00E+00	ESPL1	-3.21E-01	1.00E+00
COL4A4	-4.96E+00	4.10E-121	LOC112583714	-7.06E-01	1.00E+00	MTHFD2	-3.47E-01	1.00E+00
GTF2E1	2.40E+00	7.62E-121	C12H2orf73	-8.53E-01	1.00E+00	MARCH4	4.28E-01	1.00E+00
MAT2B	2.56E+00	8.06E-121	LOC112582974	-4.17E-01	1.00E+00	CHN2	-2.58E-01	1.00E+00
RBM39	-2.47E+00	8.29E-121	LOC112583766	3.43E-01	1.00E+00	LOC102402411	-7.44E-01	1.00E+00
MNT	3.16E+00	8.92E-121	LOC102403526	1.41E+00	1.00E+00	NCKIPSD	3.63E-01	1.00E+00
TRIM2	-4.02E+00	9.23E-121	NPEPPS	2.04E-01	1.00E+00	LOC102407618	3.42E-01	1.00E+00
LOC112579707	3.35E+00	1.45E-120	GPR137	2.02E-01	1.00E+00	ARSJ	4.05E-01	1.00E+00
WDR47	3.88E+00	2.38E-120	MTG1	-2.41E-01	1.00E+00	LOC112579072	-5.52E-01	1.00E+00
TMEM145	3.43E+00	2.47E-120	OSBPL6	2.59E-01	1.00E+00	JUND	-5.16E-01	1.00E+00
CCDC88C	3.00E+00	2.91E-120	HUNK	4.53E-01	1.00E+00	PDZD2	-2.66E-01	1.00E+00
IGFBP2	-4.58E+00	2.96E-120	CASC1	4.67E-01	1.00E+00	PCSK4	-4.01E-01	1.00E+00
RGCC	-4.17E+00	2.99E-120	ASPM	1.71E-01	1.00E+00	PRSS53	-3.01E-01	1.00E+00
SGO1	3.15E+00	3.19E-120	LOC102398949	4.37E-01	1.00E+00	MAT2A	2.48E-01	1.00E+00
FBXO28	-3.58E+00	3.43E-120	UNC13C	1.91E-01	1.00E+00	MMP15	3.26E-01	1.00E+00
PTPRK	-5.30E+00	3.68E-120	RNASEH2B	-3.05E-01	1.00E+00	CHPF2	2.38E-01	1.00E+00
PPM1H	2.58E+00	3.80E-120	SUCO	2.27E-01	1.00E+00	GLIPR2	4.24E-01	1.00E+00
ATP6V1G1	-2.37E+00	4.20E-120	PHC1	2.26E-01	1.00E+00	ZNF580	-5.26E-01	1.00E+00
LOC102394862	-	4.66E-120	LOC112587335	8.15E-01	1.00E+00	MID1IP1	-2.49E-	1.00E+00

	4.40E+00						01	
PTTG1	5.36E+00	4.69E-120	AKT3	1.89E-01	1.00E+00	LOC112582095	-1.11E+00	1.00E+00
GNG12	2.61E+00	4.77E-120	BRPF3	2.03E-01	1.00E+00	PAXIP1	-2.52E-01	1.00E+00
PRR14	2.82E+00	4.86E-120	CNN3	2.23E-01	1.00E+00	GRK4	3.29E-01	1.00E+00
PBK	2.89E+00	5.19E-120	IL17RE	-5.50E-01	1.00E+00	LOC102413952	-8.07E-01	1.00E+00
PCP4L1	6.33E+00	5.44E-120	BNC2	1.75E-01	1.00E+00	GINS1	-3.65E-01	1.00E+00
MYH7B	3.34E+00	5.64E-120	TNFAIP8L1	-5.01E-01	1.00E+00	RPS6	-3.78E-01	1.00E+00
LOC102395641	-6.66E+00	5.67E-120	ASTL	2.13E-01	1.00E+00	SLC44A3	2.52E-01	1.00E+00
GLO1	3.21E+00	5.71E-120	PHLDA3	-6.05E-01	1.00E+00	EPPK1	-6.00E-01	1.00E+00
QARS	-2.64E+00	7.26E-120	COL2A1	2.25E-01	1.00E+00	TFF1	4.88E-01	1.00E+00
PWP1	2.16E+00	1.26E-119	SLC33A1	3.27E-01	1.00E+00	FEZ1	-2.74E-01	1.00E+00
LOC102406873	3.76E+00	1.71E-119	SMARCA1	-6.80E-01	1.00E+00	ANKRD6	3.07E-01	1.00E+00
LRRC20	5.50E+00	1.76E-119	FEZ1	-3.98E-01	1.00E+00	GRIK2	5.44E-01	1.00E+00
LUC7L	-3.36E+00	2.41E-119	CAND2	2.06E-01	1.00E+00	LRRC6	-3.53E-01	1.00E+00
LOC112581169	9.18E+00	2.50E-119	PHF20	1.91E-01	1.00E+00	ADAD2	-6.32E-01	1.00E+00
HNRNPA1	-3.84E+00	2.54E-119	TNFAIP8L3	-2.72E-01	1.00E+00	NADK2	-2.75E-01	1.00E+00
LIFR	-4.19E+00	2.66E-119	CUEDC1	1.57E-01	1.00E+00	DDAH2	2.81E-01	1.00E+00
PVR	-7.41E+00	2.69E-119	LOC112579661	-8.28E-01	1.00E+00	UBB	-5.01E-01	1.00E+00
STK19	-3.80E+00	2.74E-119	LOC112581735	-4.75E-01	1.00E+00	RC3H1	-2.44E-01	1.00E+00
LOC112585227	3.42E+00	2.92E-119	ATP6	-3.91E-01	1.00E+00	KLF4	-8.86E-01	1.00E+00
RXYLT1	-4.78E+00	3.80E-119	CHST10	1.73E-01	1.00E+00	ABCB4	4.95E-01	1.00E+00
FOXD2	9.13E+00	6.23E-119	TMEM178B	-1.98E-01	1.00E+00	KIAA0930	-2.66E-01	1.00E+00
ATP6V1C1	2.23E+00	8.59E-119	INVS	-3.10E-01	1.00E+00	ATP2B3	5.37E-01	1.00E+00
EVL	-2.75E+00	8.97E-119	CRB2	-4.91E-01	1.00E+00	LOC112587824	-6.14E-01	1.00E+00
LOC102402131	-8.24E+00	1.34E-118	LOC102397215	-3.37E-01	1.00E+00	LOC112579939	5.30E-01	1.00E+00
AP4E1	-3.33E+00	1.40E-118	TAC1	1.31E+00	1.00E+00	ADAM19	5.04E-01	1.00E+00
TIAM2	2.50E+00	1.47E-118	NAE1	-1.68E-01	1.00E+00	ACLY	-2.56E-01	1.00E+00
DDHD2	2.69E+00	1.54E-118	LMO2	-9.62E-01	1.00E+00	UBAP2	-2.16E-01	1.00E+00
KIN	2.28E+00	1.67E-118	PHLPP2	2.76E-01	1.00E+00	BDH1	3.23E-01	1.00E+00
LOC102405093	1.07E+01	1.94E-118	CDC42EP3	-1.17E+00	1.00E+00	FLT1	5.02E-01	1.00E+00
TPGS1	-4.25E+00	2.15E-118	EXD1	1.85E-01	1.00E+00	TIPARP	-4.64E-01	1.00E+00
TEP1	-5.54E+00	2.35E-118	FAM118B	-1.52E-01	1.00E+00	INPP5A	2.46E-01	1.00E+00
POMK	-2.91E+00	2.87E-118	USP32	2.50E-01	1.00E+00	RPRD1A	-2.22E-01	1.00E+00

TPP1	-3.90E+00	4.88E-118	DCDC1	-4.64E-01	1.00E+00	TCF7	2.69E-01	1.00E+00
ZC3H7A	-3.04E+00	4.89E-118	LOC102413975	-1.65E-01	1.00E+00	PITPNC1	4.42E-01	1.00E+00
APEH	-3.51E+00	4.94E-118	LOC102390255	-3.36E-01	1.00E+00	OLIG1	4.85E-01	1.00E+00
HNRNPR	-2.17E+00	5.61E-118	GSTCD	5.15E-01	1.00E+00	CDC42EP3	4.30E-01	1.00E+00
CCDC6	2.39E+00	5.79E-118	GMFG	-5.03E-01	1.00E+00	SZT2	-2.67E-01	1.00E+00
SSUH2	5.41E+00	1.04E-117	DHCR24	-2.69E-01	1.00E+00	WDR37	-2.34E-01	1.00E+00
P3H4	-4.58E+00	1.10E-117	GARNL3	-2.45E-01	1.00E+00	GUCY2C	-2.95E-01	1.00E+00
DCAF11	-3.25E+00	1.32E-117	LOC102411292	-2.69E-01	1.00E+00	XKR5	-3.06E-01	1.00E+00
LOC112586188	8.93E+00	2.16E-117	LOC102412752	-7.06E-01	1.00E+00	DPP3	2.33E-01	1.00E+00
SUPT4H1	-4.98E+00	2.19E-117	KRT5	4.90E-01	1.00E+00	PPIC	-2.53E-01	1.00E+00
STRIP1	2.39E+00	2.35E-117	PDP1	1.97E-01	1.00E+00	ELMO2	2.52E-01	1.00E+00
CNPY4	-3.87E+00	2.38E-117	RPS14	-2.82E-01	1.00E+00	HSPA12A	6.10E-01	1.00E+00
E4F1	-4.17E+00	2.97E-117	ERCC6L	-2.25E-01	1.00E+00	AMPD3	7.28E-01	1.00E+00
DAGLA	3.37E+00	3.41E-117	LOC102404894	-1.32E+00	1.00E+00	WDR76	-3.09E-01	1.00E+00
NR2F6	-3.85E+00	3.50E-117	RFWD3	2.82E-01	1.00E+00	BIN1	-2.49E-01	1.00E+00
NR3C2	7.77E+00	3.58E-117	USP35	2.74E-01	1.00E+00	SERPINF2	2.86E-01	1.00E+00
PPP2R5A	-3.91E+00	5.94E-117	TTC39C	-2.01E-01	1.00E+00	SAP30	3.01E-01	1.00E+00
GJA1	-4.55E+00	6.43E-117	MTMR6	1.75E-01	1.00E+00	MSI2	-3.03E-01	1.00E+00
LAMA3	6.47E+00	7.13E-117	GLIS2	-9.54E-01	1.00E+00	TCF4	3.57E-01	1.00E+00
TSPAN6	-4.71E+00	9.35E-117	MTMR9	2.80E-01	1.00E+00	BCL7A	2.61E-01	1.00E+00
PCGF2	-2.88E+00	9.71E-117	SLC29A2	4.37E-01	1.00E+00	MYB	5.07E-01	1.00E+00
SMC1B	9.35E+00	9.93E-117	LMTK3	3.71E-01	1.00E+00	MUC13	3.90E-01	1.00E+00
MLLT6	-3.44E+00	1.10E-116	NT5DC1	-2.12E-01	1.00E+00	MCU	3.50E-01	1.00E+00
LSG1	2.10E+00	1.45E-116	DSG2	3.88E-01	1.00E+00	GFPT2	4.79E-01	1.00E+00
LOC112582239	6.41E+00	1.71E-116	RCBTB2	2.93E-01	1.00E+00	FLRT2	2.35E-01	1.00E+00
LOC102396801	5.96E+00	1.75E-116	ITGAM	-1.24E+00	1.00E+00	RBMS3	3.86E-01	1.00E+00
PRIMPOL	2.50E+00	1.76E-116	AATF	-2.43E-01	1.00E+00	LOC102410337	-1.20E+00	1.00E+00
SEPT9	-3.48E+00	2.24E-116	ADAT1	3.41E-01	1.00E+00	FGF22	5.48E-01	1.00E+00
SRSF5	-3.26E+00	2.28E-116	VIP	-8.70E-01	1.00E+00	LOC112580274	5.51E-01	1.00E+00
C2H6orf47	-5.07E+00	2.36E-116	MOB3C	-6.36E-01	1.00E+00	KRT10	4.15E-01	1.00E+00
P4HA1	-2.35E+00	2.36E-116	TOR2A	1.57E-01	1.00E+00	LOC102399312	6.00E-01	1.00E+00
FAM13C	4.94E+00	2.68E-116	LOC112587105	1.00E+00	1.00E+00	BMP8B	6.34E-01	1.00E+00
TRIM6	4.36E+00	2.78E-116	ADCY3	-2.71E-01	1.00E+00	ENPP1	3.81E-01	1.00E+00
COPS5	-2.79E+00	3.70E-116	LOC112579183	-7.86E-01	1.00E+00	TMTC1	-3.23E-01	1.00E+00

RBM10	-2.93E+00	3.96E-116	NEMP2	1.64E-01	1.00E+00	FYB2	-4.64E-01	1.00E+00
LOC102408561	3.56E+00	4.61E-116	LOC102397198	5.38E-01	1.00E+00	BUB1	-2.58E-01	1.00E+00
MRPS31	2.42E+00	5.04E-116	KIAA0319L	1.81E-01	1.00E+00	SEMA4G	-3.40E-01	1.00E+00
NUDT15	2.61E+00	5.30E-116	SHPK	-9.76E-01	1.00E+00	UPP2	-5.78E-01	1.00E+00
DERL2	2.71E+00	5.30E-116	UST	-7.57E-01	1.00E+00	CLIC2	5.45E-01	1.00E+00
LOC112578041	7.78E+00	5.86E-116	RPL37	-2.92E-01	1.00E+00	CCDC158	2.75E-01	1.00E+00
LOC112585122	-8.67E+00	7.01E-116	PDK4	-1.10E+00	1.00E+00	ADAMTS20	7.22E-01	1.00E+00
SEC11A	-3.43E+00	7.32E-116	LOC102400385	4.08E-01	1.00E+00	RAD54L	-2.69E-01	1.00E+00
PEPD	-2.56E+00	9.88E-116	KLC4	-2.19E-01	1.00E+00	NCAPG2	-2.61E-01	1.00E+00
C19H5orf47	7.01E+00	1.28E-115	RAB34	-5.21E-01	1.00E+00	IGSF1	-5.91E-01	1.00E+00
IPO8	-4.02E+00	1.28E-115	DND1	-1.77E-01	1.00E+00	SNAP91	-3.12E-01	1.00E+00
MAGEH1	-5.54E+00	1.30E-115	LOC102395814	-3.17E-01	1.00E+00	CACNA1A	6.00E-01	1.00E+00
EPB41	3.06E+00	1.60E-115	GLRX	-3.18E-01	1.00E+00	HNRNPDL	-2.99E-01	1.00E+00
RNF223	6.13E+00	1.94E-115	LOC102402202	-1.04E+00	1.00E+00	MEGF9	3.59E-01	1.00E+00
LOC102406086	6.35E+00	1.99E-115	LOC102402227	-2.61E-01	1.00E+00	TERF1	-2.62E-01	1.00E+00
FSHR	-3.12E+00	2.00E-115	LOC112583896	7.22E-01	1.00E+00	GRM1	5.47E-01	1.00E+00
HDAC2	-2.32E+00	2.15E-115	HNRNPAB	-2.72E-01	1.00E+00	SDHB	2.67E-01	1.00E+00
DYM	2.18E+00	2.17E-115	LOC102398572	-7.52E-01	1.00E+00	C18H19orf54	-2.85E-01	1.00E+00
MLKL	7.21E+00	2.26E-115	XPOT	1.66E-01	1.00E+00	NTN1	-2.78E-01	1.00E+00
BCL2L1	2.07E+00	3.02E-115	FZD7	1.14E+00	1.00E+00	GGT5	-9.10E-01	1.00E+00
PRKCQ	5.68E+00	3.21E-115	LOC112586444	-3.73E-01	1.00E+00	LOC112577987	5.15E-01	1.00E+00
VARS	-2.60E+00	3.44E-115	NFXL1	2.07E-01	1.00E+00	SHCBP1	-2.80E-01	1.00E+00
FAM114A2	2.20E+00	4.59E-115	LOC102404792	-5.15E-01	1.00E+00	FLNC	5.32E-01	1.00E+00
DLGAP5	3.53E+00	5.30E-115	MAJIN	-2.25E-01	1.00E+00	LOC112577753	4.64E-01	1.00E+00
ATG5	2.02E+00	5.64E-115	RPL22L1	-3.27E-01	1.00E+00	TIAM1	7.82E-01	1.00E+00
AUP1	-5.40E+00	5.64E-115	MANF	-4.30E-01	1.00E+00	RHBG	-4.34E-01	1.00E+00
HSPA13	-4.86E+00	6.13E-115	HSD17B10	-2.13E-01	1.00E+00	LOC112585299	-4.08E-01	1.00E+00
NSUN6	2.52E+00	7.18E-115	LOC112586540	6.33E-01	1.00E+00	ELOA	-2.88E-01	1.00E+00
INO80E	2.14E+00	7.31E-115	SYN2	-2.50E-01	1.00E+00	AGPAT4	5.62E-01	1.00E+00
MLH1	2.66E+00	7.52E-115	MEIS1	-9.70E-01	1.00E+00	PHLDA3	2.98E-01	1.00E+00
TNRC18	-2.89E+00	1.02E-114	ELMOD2	2.61E-01	1.00E+00	SHROOM3	3.72E-01	1.00E+00
NUSAP1	3.51E+00	1.10E-114	CDIPT	2.89E-01	1.00E+00	EFNB3	5.38E-01	1.00E+00
MBNL2	-	1.21E-114	AMOT	2.22E-01	1.00E+00	CCNB3	-3.42E-	1.00E+00

	3.81E+00						01	
HPS4	-2.91E+00	1.25E-114	LOC102398353	3.92E-01	1.00E+00	LOC102412912	5.51E-01	1.00E+00
HIPK3	-3.73E+00	1.36E-114	GFPT2	-5.54E-01	1.00E+00	PPRC1	-2.23E-01	1.00E+00
SELENOS	-4.22E+00	1.47E-114	PRKAB2	2.88E-01	1.00E+00	REL	6.08E-01	1.00E+00
RABGEF1	2.99E+00	1.82E-114	SLC2A13	-6.83E-01	1.00E+00	LOC102405064	-5.19E-01	1.00E+00
CDCA8	3.18E+00	1.93E-114	EML4	2.04E-01	1.00E+00	SLC22A7	-5.77E-01	1.00E+00
NEMP2	2.32E+00	1.94E-114	PEPD	-2.66E-01	1.00E+00	VASH1	3.72E-01	1.00E+00
LOC102399272	-4.25E+00	2.04E-114	AIDA	1.63E-01	1.00E+00	RNF43	6.02E-01	1.00E+00
RBSN	-3.49E+00	2.05E-114	RBM44	-5.25E-01	1.00E+00	CPSF4	-2.25E-01	1.00E+00
NR1I3	6.96E+00	2.19E-114	GIGYF2	1.80E-01	1.00E+00	LOC102395010	3.31E-01	1.00E+00
CHST2	-7.83E+00	2.34E-114	NKPD1	6.42E-01	1.00E+00	RGCC	2.48E-01	1.00E+00
GPR39	6.49E+00	2.34E-114	ZNF318	2.79E-01	1.00E+00	TWIST1	6.65E-01	1.00E+00
ST3GAL2	-3.49E+00	2.37E-114	ID4	-7.02E-01	1.00E+00	CDK5	-3.17E-01	1.00E+00
ADK	-2.81E+00	2.39E-114	ZNF526	1.83E-01	1.00E+00	FAM20C	2.42E-01	1.00E+00
SGPP2	8.98E+00	2.91E-114	MAPK8IP3	1.75E-01	1.00E+00	ZNF536	-2.90E-01	1.00E+00
IER5	-5.18E+00	4.35E-114	TANK	-2.16E-01	1.00E+00	LPAR1	4.48E-01	1.00E+00
HOXA7	6.21E+00	4.63E-114	ZBTB8A	-5.84E-01	1.00E+00	SH3KBP1	3.45E-01	1.00E+00
DDX39B	-2.61E+00	5.18E-114	SENP2	2.11E-01	1.00E+00	ARHGEF19	-4.09E-01	1.00E+00
ANXA6	-5.62E+00	5.69E-114	IPCEF1	3.42E-01	1.00E+00	LIF	5.49E-01	1.00E+00
BEX3	-4.46E+00	7.17E-114	RTN4R	-3.80E-01	1.00E+00	IRAK2	4.42E-01	1.00E+00
FAM210B	-2.73E+00	8.06E-114	R3HDM2	2.76E-01	1.00E+00	DYNC1I1	6.03E-01	1.00E+00
SMPD4	-2.57E+00	1.30E-113	RLIM	2.42E-01	1.00E+00	LRCH4	-2.32E-01	1.00E+00
GRIK2	5.17E+00	1.47E-113	ADD3	2.69E-01	1.00E+00	DHH	-4.58E-01	1.00E+00
FAM107A	-4.36E+00	1.72E-113	ZNF366	4.10E-01	1.00E+00	ZNF365	-3.09E-01	1.00E+00
LOC102399864	2.26E+00	1.99E-113	CAB39	1.95E-01	1.00E+00	LOC102393150	-4.71E-01	1.00E+00
LOC102412967	5.13E+00	2.04E-113	CALN1	-3.72E-01	1.00E+00	SLC7A6	-3.43E-01	1.00E+00
LYPLA1	-3.23E+00	2.15E-113	SNAPC4	3.10E-01	1.00E+00	ARSB	-2.66E-01	1.00E+00
NINJ1	-3.04E+00	2.27E-113	TTLL4	1.74E-01	1.00E+00	CYTH3	-4.23E-01	1.00E+00
NR1H4	4.95E+00	2.50E-113	MFSD1	1.72E-01	1.00E+00	BACH1	4.53E-01	1.00E+00
HMGN1	-2.79E+00	2.66E-113	EEF2K	1.91E-01	1.00E+00	PNISR	-2.90E-01	1.00E+00
FBXO10	3.08E+00	3.09E-113	KLF17	-9.44E-01	1.00E+00	LOC102397734	7.54E-01	1.00E+00
DSN1	2.29E+00	3.23E-113	APOA1	-1.20E+00	1.00E+00	EFCAB6	6.68E-01	1.00E+00
RNF187	-5.96E+00	3.75E-113	NGEF	1.20E+00	1.00E+00	CATSPERB	5.60E-01	1.00E+00
LINGO1	5.95E+00	6.41E-113	FBXO5	2.06E-01	1.00E+00	KANK2	3.00E-01	1.00E+00

EVPL	4.88E+00	8.37E-113	MAOA	-8.52E-01	1.00E+00	EXOC4	-2.20E-01	1.00E+00
AZI2	-2.36E+00	8.44E-113	IMPG1	-8.62E-01	1.00E+00	LOC102398822	4.12E-01	1.00E+00
RBBP6	-3.30E+00	1.05E-112	GID4	1.73E-01	1.00E+00	DAPL1	-6.30E-01	1.00E+00
TMEM169	5.43E+00	1.26E-112	TMEM33	2.30E-01	1.00E+00	ARHGAP19	-3.51E-01	1.00E+00
ZNF282	-4.04E+00	1.28E-112	CGRRF1	-5.88E-01	1.00E+00	ARGLU1	-2.70E-01	1.00E+00
SOCS2	4.85E+00	1.44E-112	FAM208B	2.90E-01	1.00E+00	PNPLA2	-2.56E-01	1.00E+00
LAPTM4B	-4.96E+00	1.47E-112	MGAT3	-7.98E-01	1.00E+00	SLC22A15	6.64E-01	1.00E+00
DHX15	-2.14E+00	2.04E-112	BIRC3	-4.55E-01	1.00E+00	UBE4B	-2.30E-01	1.00E+00
DEPDC1B	2.78E+00	2.41E-112	SNTB1	-1.68E-01	1.00E+00	INTS6L	-2.76E-01	1.00E+00
CD99	-6.08E+00	2.57E-112	PHF2	-5.81E-01	1.00E+00	PWWP2B	5.25E-01	1.00E+00
GIT2	2.85E+00	3.10E-112	CSMD2	-2.44E-01	1.00E+00	PEMT	-2.83E-01	1.00E+00
HMOX1	-3.84E+00	3.92E-112	PIK3C2G	-3.57E-01	1.00E+00	IFT52	-2.21E-01	1.00E+00
TUBB	-3.86E+00	3.99E-112	MGAT5	1.66E-01	1.00E+00	IFNGR2	2.22E-01	1.00E+00
RPAP1	2.66E+00	4.57E-112	SRGAP3	3.84E-01	1.00E+00	TMEM107	-2.35E-01	1.00E+00
KLF12	2.52E+00	4.69E-112	UBE2D2	-1.71E-01	1.00E+00	LOC102406753	-3.83E-01	1.00E+00
GRAMD4	-4.27E+00	5.66E-112	GM2A	-4.91E-01	1.00E+00	IFNAR1	3.67E-01	1.00E+00
LOC112585609	8.37E+00	6.92E-112	SLC37A3	1.91E-01	1.00E+00	ADAMTS17	4.06E-01	1.00E+00
LOC112586104	7.23E+00	6.98E-112	LOC112586836	-4.78E-01	1.00E+00	ANKRD39	-2.95E-01	1.00E+00
TFB2M	-3.51E+00	9.72E-112	CALHM2	1.04E+00	1.00E+00	LPIN1	-2.83E-01	1.00E+00
LOC112579141	7.49E+00	1.05E-111	NSG1	-2.11E-01	1.00E+00	ZNF217	3.98E-01	1.00E+00
NUP155	2.10E+00	1.10E-111	SF1	1.71E-01	1.00E+00	SMC2	-2.77E-01	1.00E+00
KDELC1	-2.96E+00	1.26E-111	BTBD10	1.72E-01	1.00E+00	LOC102412377	7.34E-01	1.00E+00
HDAC7	4.14E+00	1.92E-111	TSPAN19	-1.06E+00	1.00E+00	AQP10	-4.57E-01	1.00E+00
IER3IP1	-2.77E+00	2.02E-111	HMGXB3	1.76E-01	1.00E+00	MGARP	4.23E-01	1.00E+00
LOC102407694	-5.32E+00	2.19E-111	LOC112586916	-8.56E-01	1.00E+00	FNBP1	4.13E-01	1.00E+00
PCBP2	-3.02E+00	2.19E-111	BMF	3.07E-01	1.00E+00	LIAS	-2.69E-01	1.00E+00
SYCP1	6.28E+00	2.33E-111	RGL3	2.24E-01	1.00E+00	WSB2	2.15E-01	1.00E+00
SNX17	-2.79E+00	2.41E-111	TSPAN14	1.53E-01	1.00E+00	SEMA5A	5.39E-01	1.00E+00
COLGALT1	-5.48E+00	2.41E-111	LOC102404604	1.13E+00	1.00E+00	CLCN6	-2.73E-01	1.00E+00
EDRF1	2.65E+00	2.65E-111	ZNF629	2.41E-01	1.00E+00	LDLRAD4	4.40E-01	1.00E+00
NSD3	-2.96E+00	2.78E-111	SDK2	-3.46E-01	1.00E+00	TUB	-2.87E-01	1.00E+00
CDK8	2.09E+00	2.80E-111	GRIN2C	9.12E-01	1.00E+00	FAM160A1	4.83E-01	1.00E+00
EIF3D	-3.20E+00	3.22E-111	TRAFD1	1.45E-01	1.00E+00	MRTFA	2.89E-01	1.00E+00
HSF2BP	2.64E+00	3.25E-111	LOC112580409	-3.96E-	1.00E+00	LOC102402159	7.10E-01	1.00E+00

				01				
HSD17B7	-4.98E+00	3.45E-111	GMEB1	3.35E-01	1.00E+00	PPARGC1B	4.04E-01	1.00E+00
NQO1	3.13E+00	3.60E-111	PRKDC	2.86E-01	1.00E+00	GAS7	8.55E-01	1.00E+00
PLA2G4F	5.87E+00	3.66E-111	LOC112578832	-5.68E-01	1.00E+00	SHF	3.15E-01	1.00E+00
LOC102394590	7.49E+00	4.61E-111	LOC102403829	-2.47E-01	1.00E+00	GTPBP1	-2.13E-01	1.00E+00
CCDC60	4.48E+00	4.89E-111	HIC1	-3.87E-01	1.00E+00	ANO6	3.68E-01	1.00E+00
AIF1L	-5.59E+00	5.62E-111	RAPGEF6	2.30E-01	1.00E+00	NFATC2IP	-2.83E-01	1.00E+00
GAL3ST1	8.85E+00	6.06E-111	ATP2C1	1.53E-01	1.00E+00	ASB16	-6.28E-01	1.00E+00
DCAF1	2.90E+00	8.58E-111	BSND	2.70E-01	1.00E+00	DHRS1	-2.60E-01	1.00E+00
LOC102398198	7.06E+00	1.18E-110	ASIP	-5.08E-01	1.00E+00	ADAMTS15	-4.69E-01	1.00E+00
TAPBPL	-4.84E+00	1.31E-110	ACBD7	-2.77E-01	1.00E+00	SATB2	5.43E-01	1.00E+00
RNF146	2.02E+00	1.59E-110	IFT52	-2.46E-01	1.00E+00	ENO2	5.38E-01	1.00E+00
ZC3H4	-3.23E+00	2.02E-110	PCMTD2	2.67E-01	1.00E+00	RHBDF2	3.72E-01	1.00E+00
POC5	1.99E+00	2.08E-110	NONO	-2.04E-01	1.00E+00	LTBP1	-3.02E-01	1.00E+00
KRCC1	-6.44E+00	2.08E-110	CABIN1	2.25E-01	1.00E+00	B3GNT3	-3.10E-01	1.00E+00
C20H14orf132	-6.25E+00	2.20E-110	ATOH7	-2.71E-01	1.00E+00	OTUD7A	6.77E-01	1.00E+00
PAPLN	7.06E+00	2.59E-110	BAMBI	-5.50E-01	1.00E+00	MYO19	-2.55E-01	1.00E+00
RIPK2	-2.62E+00	2.77E-110	MOV10L1	2.44E-01	1.00E+00	ANKRD9	-3.04E-01	1.00E+00
TMIGD1	5.76E+00	3.29E-110	LOC112587017	-7.25E-01	1.00E+00	LOC102415619	3.15E-01	1.00E+00
SSH2	3.50E+00	4.40E-110	GABARAP	-2.52E-01	1.00E+00	SNN	2.17E-01	1.00E+00
ATXN7L3	-6.15E+00	5.21E-110	VGF	-7.93E-01	1.00E+00	LOC102410286	4.35E-01	1.00E+00
PNPLA3	1.01E+01	6.09E-110	RCVRN	8.21E-01	1.00E+00	SCAF4	-2.32E-01	1.00E+00
STAT6	-4.56E+00	6.98E-110	PAG1	3.55E-01	1.00E+00	TRMT61A	3.31E-01	1.00E+00
MPP2	3.78E+00	8.10E-110	KLK10	1.08E+00	1.00E+00	CKM	-4.92E-01	1.00E+00
MFSD8	2.39E+00	8.17E-110	MYO1E	-3.26E-01	1.00E+00	LOC112580425	-3.73E-01	1.00E+00
SLC25A29	-3.95E+00	8.38E-110	WFDC2	5.78E-01	1.00E+00	MTMR11	3.03E-01	1.00E+00
MTM1	2.91E+00	9.51E-110	LHFPL2	2.82E-01	1.00E+00	TRAK1	-2.44E-01	1.00E+00
LOC112578702	7.03E+00	1.01E-109	DNMT3A	1.81E-01	1.00E+00	PGF	-3.09E-01	1.00E+00
ALDOA	-4.39E+00	1.24E-109	STX3	1.87E-01	1.00E+00	DLC1	5.93E-01	1.00E+00
RTP1	9.45E+00	1.30E-109	SUSD5	-4.15E-01	1.00E+00	ADAMTS6	4.48E-01	1.00E+00
CNPY3	-3.93E+00	1.53E-109	SYNPR	-1.21E+00	1.00E+00	LOC112585035	6.95E-01	1.00E+00
PHF5A	-3.79E+00	1.60E-109	FOSL2	-1.58E-01	1.00E+00	NFATC3	-2.93E-01	1.00E+00
LOC112584624	7.26E+00	1.81E-109	RPL27	-2.12E-01	1.00E+00	RDH10	-2.51E-01	1.00E+00

LOC102412417	1.95E+00	2.64E-109	NMT1	-1.95E-01	1.00E+00	CFAP47	5.59E-01	1.00E+00
PIGT	-2.61E+00	2.95E-109	LOC102411988	-5.51E-01	1.00E+00	TP53INP1	3.00E-01	1.00E+00
CHST14	-3.79E+00	3.05E-109	NUDT9	-2.08E-01	1.00E+00	LOC102399127	7.54E-01	1.00E+00
NUDT4	3.03E+00	3.40E-109	LOC102413367	-2.64E-01	1.00E+00	KBTBD7	-3.64E-01	1.00E+00
BLVRA	-5.92E+00	5.84E-109	SUN1	1.96E-01	1.00E+00	PSMB8	3.23E-01	1.00E+00
VRK1	2.73E+00	6.25E-109	AHSA1	-1.49E-01	1.00E+00	PPP2CB	2.27E-01	1.00E+00
ALKBH5	3.19E+00	6.25E-109	ND3	-3.13E-01	1.00E+00	SMC6	-2.29E-01	1.00E+00
ZNF358	-4.22E+00	6.49E-109	CYTH4	4.20E-01	1.00E+00	TPMT	3.58E-01	1.00E+00
PIP5K1B	4.09E+00	9.33E-109	PPP1R7	-2.75E-01	1.00E+00	BUD13	-2.14E-01	1.00E+00
RBMX	-2.30E+00	1.48E-108	ABTB1	-6.49E-01	1.00E+00	CCNK	-2.16E-01	1.00E+00
TIMM9	2.29E+00	1.59E-108	SRRD	5.22E-01	1.00E+00	UBC	-3.28E-01	1.00E+00
FBLN1	-6.04E+00	1.90E-108	DMXL1	3.97E-01	1.00E+00	ADAM8	2.31E-01	1.00E+00
ETF1	-2.50E+00	2.15E-108	DLG3	-1.90E-01	1.00E+00	SERPINE1	4.69E-01	1.00E+00
SERBP1	-2.65E+00	2.49E-108	RPS3A	-2.55E-01	1.00E+00	MAP3K6	4.24E-01	1.00E+00
DPF1	3.62E+00	2.58E-108	SHC2	-3.33E-01	1.00E+00	SPRY4	6.91E-01	1.00E+00
MFGE8	-5.07E+00	2.58E-108	INHBB	-1.07E+00	1.00E+00	NHS	4.44E-01	1.00E+00
EPHB4	-5.30E+00	2.73E-108	MRPL30	-3.02E-01	1.00E+00	TSPYL2	-2.56E-01	1.00E+00
LOC112582299	7.56E+00	3.18E-108	ERLIN2	2.13E-01	1.00E+00	TMEM250	2.18E-01	1.00E+00
NKTR	-2.71E+00	3.66E-108	LOC102407917	4.50E-01	1.00E+00	USHBP1	-3.89E-01	1.00E+00
RASL10A	5.72E+00	3.67E-108	LOC102416515	-8.11E-01	1.00E+00	PIGS	-2.74E-01	1.00E+00
LOC102393257	6.35E+00	3.85E-108	FRMD7	-4.24E-01	1.00E+00	IQGAP2	-2.61E-01	1.00E+00
ALG2	2.51E+00	4.04E-108	LYPLA1	-3.98E-01	1.00E+00	IL2RB	-6.40E-01	1.00E+00
NTS	-7.37E+00	4.59E-108	EIF2S1	-1.78E-01	1.00E+00	CD209	8.57E-01	1.00E+00
SMIM10L1	-3.15E+00	5.59E-108	MORF4L1	-2.14E-01	1.00E+00	CSPP1	-2.45E-01	1.00E+00
ZNF143	2.34E+00	7.67E-108	CHDH	-3.62E-01	1.00E+00	TNC	6.51E-01	1.00E+00
FADS1	-3.47E+00	9.22E-108	DHFR	-2.70E-01	1.00E+00	TEC	2.31E-01	1.00E+00
AATK	4.69E+00	1.36E-107	LOC112584689	5.60E-01	1.00E+00	TNK1	4.45E-01	1.00E+00
RABGAP1	2.46E+00	1.52E-107	PTPN11	2.34E-01	1.00E+00	TRAF3IP3	5.11E-01	1.00E+00
RUSC1	-4.35E+00	1.77E-107	TMEM176B	-1.09E+00	1.00E+00	NAA30	2.39E-01	1.00E+00
LOC102416114	9.30E+00	1.87E-107	VSIG10	2.35E-01	1.00E+00	S1PR3	6.12E-01	1.00E+00
LOC102389719	-2.71E+00	1.98E-107	TRPC5	-3.58E-01	1.00E+00	RCAN1	2.72E-01	1.00E+00
SLC8A1	3.73E+00	2.00E-107	LHFPL5	-4.72E-01	1.00E+00	TMEM108	6.72E-01	1.00E+00
RAB11FIP2	-3.66E+00	2.29E-107	BASP1	-2.55E-01	1.00E+00	EGR3	2.31E-01	1.00E+00
SHC2	5.77E+00	2.69E-107	SNRNP48	-2.84E-	1.00E+00	GXYLT2	3.52E-01	1.00E+00

				01				
FBXO43	5.10E+00	2.70E-107	LOC112580428	-5.85E-01	1.00E+00	TNRC6C	-2.10E-01	1.00E+00
KIAA1211	2.89E+00	3.18E-107	COG6	2.99E-01	1.00E+00	LOC112582199	4.21E-01	1.00E+00
CLDN10	7.29E+00	3.65E-107	USP5	1.57E-01	1.00E+00	PLIN2	3.29E-01	1.00E+00
LOC102402580	2.71E+00	4.40E-107	PRR16	-3.20E-01	1.00E+00	C9H19orf57	-3.48E-01	1.00E+00
TMUB2	-4.44E+00	4.48E-107	MYBPC2	-4.73E-01	1.00E+00	PPP1R11	2.37E-01	1.00E+00
TUBGCP6	-2.80E+00	4.77E-107	LOC102398514	6.57E-01	1.00E+00	ARHGAP32	2.98E-01	1.00E+00
LUZP1	-3.39E+00	5.26E-107	ACTR10	-2.14E-01	1.00E+00	FBXO17	-2.91E-01	1.00E+00
ACYP2	2.69E+00	5.40E-107	ADAT2	-6.25E-01	1.00E+00	GATA6	-2.85E-01	1.00E+00
PPP4R3A	-1.88E+00	5.47E-107	DLGAP2	-3.94E-01	1.00E+00	RAB11FIP3	2.10E-01	1.00E+00
DERA	-4.50E+00	6.36E-107	UBQLN4	-3.14E-01	1.00E+00	ST3GAL4	-3.31E-01	1.00E+00
APBA1	4.91E+00	6.45E-107	MEIKIN	-2.92E-01	1.00E+00	ZDHHC23	3.66E-01	1.00E+00
POLR3F	-4.66E+00	6.78E-107	TXNDC15	-3.77E-01	1.00E+00	RAPGEF4	5.27E-01	1.00E+00
LOC112586515	9.14E+00	8.32E-107	CACNG1	-5.83E-01	1.00E+00	MYH11	-2.57E-01	1.00E+00
DECR1	-4.92E+00	8.67E-107	TRAM1L1	3.23E-01	1.00E+00	SLC40A1	4.85E-01	1.00E+00
AES	-4.58E+00	9.80E-107	TREH	-3.05E-01	1.00E+00	RMND1	-2.75E-01	1.00E+00
FSD1	3.26E+00	1.39E-106	LOC102409830	-1.01E+00	1.00E+00	PTPN14	3.00E-01	1.00E+00
WDSUB1	1.90E+00	1.59E-106	SFXN5	-3.72E-01	1.00E+00	SH2D5	3.38E-01	1.00E+00
NOP53	-3.35E+00	1.73E-106	NUDC	-1.67E-01	1.00E+00	VASH2	4.70E-01	1.00E+00
POU3F2	7.03E+00	1.76E-106	MTRR	3.28E-01	1.00E+00	THBS4	3.87E-01	1.00E+00
STIMATE	2.16E+00	1.81E-106	ACAT1	-1.76E-01	1.00E+00	CLDN15	5.26E-01	1.00E+00
PCYT2	-4.08E+00	1.86E-106	CAPN14	-6.47E-01	1.00E+00	RGS12	-2.22E-01	1.00E+00
ZFP91	2.24E+00	2.00E-106	PQLC1	-4.99E-01	1.00E+00	NFKB2	2.64E-01	1.00E+00
TEC	-3.88E+00	2.18E-106	TGIF2	1.77E-01	1.00E+00	ARHGEF39	-3.00E-01	1.00E+00
KIAA1324L	3.98E+00	2.89E-106	SPTB	2.09E-01	1.00E+00	RNF216	2.18E-01	1.00E+00
KHNYN	-5.94E+00	4.65E-106	TBX3	7.27E-01	1.00E+00	DENND2A	2.40E-01	1.00E+00
PLEKHA4	-4.88E+00	4.80E-106	ATP5MD	-2.25E-01	1.00E+00	SUPT3H	2.48E-01	1.00E+00
PRPS1	-2.63E+00	4.85E-106	LOC112581447	-2.46E-01	1.00E+00	ENC1	2.97E-01	1.00E+00
HAUS6	2.05E+00	5.70E-106	PITPNB	-2.67E-01	1.00E+00	LOC102392630	-5.54E-01	1.00E+00
HEXA	-2.54E+00	5.70E-106	PDCD4	1.99E-01	1.00E+00	TMEM176A	-2.77E-01	1.00E+00
GFI1	6.88E+00	5.97E-106	NET1	1.96E-01	1.00E+00	ADCY10	5.99E-01	1.00E+00
AMER1	2.90E+00	7.03E-106	PRKACB	2.60E-01	1.00E+00	RFLNB	5.80E-01	1.00E+00
HMGN3	-3.92E+00	7.20E-106	KDM6B	3.04E-01	1.00E+00	LOC112580779	4.09E-01	1.00E+00
PDZK1	3.16E+00	7.65E-106	NFATC2IP	2.92E-01	1.00E+00	GATM	3.01E-01	1.00E+00
CBFA2T2	2.83E+00	7.86E-106	LOC112585014	-4.99E-01	1.00E+00	CCDC170	3.92E-01	1.00E+00

APP	-3.21E+00	7.92E-106	LOC112585226	-6.13E-01	1.00E+00	PGLS	-2.88E-01	1.00E+00
ZNF215	4.37E+00	8.56E-106	CRYGN	1.88E-01	1.00E+00	TP53INP2	-5.38E-01	1.00E+00
IL18BP	-3.84E+00	9.98E-106	LOC102410713	-6.12E-01	1.00E+00	HUS1	-2.34E-01	1.00E+00
ATP5F1B	-3.16E+00	1.05E-105	LSAMP	-7.47E-01	1.00E+00	TANC2	2.50E-01	1.00E+00
ECPAS	-1.96E+00	1.14E-105	NEK10	-5.27E-01	1.00E+00	PLAT	4.15E-01	1.00E+00
ICAM4	8.97E+00	1.63E-105	BNC1	2.09E-01	1.00E+00	LOC102404254	6.59E-01	1.00E+00
IGFALS	5.13E+00	1.81E-105	CLOCK	2.54E-01	1.00E+00	LOC112580183	-5.97E-01	1.00E+00
TSC22D1	-4.03E+00	1.86E-105	ACSL5	-2.49E-01	1.00E+00	TRIM16	7.08E-01	1.00E+00
NBDY	-4.59E+00	2.05E-105	PSMA7	-1.68E-01	1.00E+00	LOC102398414	3.43E-01	1.00E+00
PGM1	-2.57E+00	2.23E-105	ZWINT	3.29E-01	1.00E+00	CORO6	-2.95E-01	1.00E+00
GALNS	-4.75E+00	3.45E-105	POMP	-1.98E-01	1.00E+00	LTB4R	4.59E-01	1.00E+00
HNRNPLL	-4.24E+00	4.20E-105	PPIL3	-3.63E-01	1.00E+00	RGS9	4.42E-01	1.00E+00
ZNF235	2.87E+00	4.96E-105	CBL	2.91E-01	1.00E+00	CENPV	-4.31E-01	1.00E+00
SLC25A46	-2.86E+00	5.03E-105	MKNK2	1.96E-01	1.00E+00	LOC112587459	6.78E-01	1.00E+00
RSRP1	-3.90E+00	5.20E-105	ATXN1L	2.64E-01	1.00E+00	TTYH2	4.08E-01	1.00E+00
PIAS1	2.20E+00	5.34E-105	HSD11B2	2.34E-01	1.00E+00	DFFA	2.04E-01	1.00E+00
PHYHIPL	9.61E+00	5.43E-105	FHOD1	3.43E-01	1.00E+00	AKAP8	-2.10E-01	1.00E+00
LTV1	2.12E+00	6.30E-105	PTPN14	2.32E-01	1.00E+00	FAM83D	-2.93E-01	1.00E+00
ARHGAP44	3.02E+00	8.20E-105	PRDX6	-2.92E-01	1.00E+00	RBM25	-2.49E-01	1.00E+00
PSMC5	-3.23E+00	9.56E-105	GRK6	1.82E-01	1.00E+00	SLC16A3	3.24E-01	1.00E+00
FAM20C	-6.72E+00	1.08E-104	KMT2A	3.87E-01	1.00E+00	TXN	2.95E-01	1.00E+00
HNRNPK	-2.13E+00	1.10E-104	C8H7orf57	-6.16E-01	1.00E+00	LOC102415015	7.78E-01	1.00E+00
AASS	-6.51E+00	1.19E-104	RB1CC1	1.92E-01	1.00E+00	MRO	3.00E-01	1.00E+00
CAPN2	-6.44E+00	1.41E-104	AMDHD2	-5.18E-01	1.00E+00	NPR1	-3.51E-01	1.00E+00
HPCAL1	-2.32E+00	1.52E-104	VANGL1	1.66E-01	1.00E+00	KCNA5	5.29E-01	1.00E+00
TLE6	4.08E+00	1.75E-104	ARHGAP1	2.03E-01	1.00E+00	MFAP2	3.69E-01	1.00E+00
NDUFS2	-3.35E+00	1.81E-104	DAP	1.76E-01	1.00E+00	SORBS3	-2.15E-01	1.00E+00
TSC22D3	-3.94E+00	2.65E-104	YJU2	2.10E-01	1.00E+00	TMEM237	-3.35E-01	1.00E+00
LOC102403870	3.87E+00	2.75E-104	FSD1	-1.77E-01	1.00E+00	FBXO40	-5.50E-01	1.00E+00
TACC2	-4.63E+00	2.89E-104	LOC102390493	4.65E-01	1.00E+00	ZFP57	-4.46E-01	1.00E+00
LOC102412874	-4.95E+00	2.99E-104	KLF15	-3.95E-01	1.00E+00	MDFIC	-3.45E-01	1.00E+00
LOC112580264	8.72E+00	3.44E-104	LOC102406990	8.85E-01	1.00E+00	TRPM6	5.57E-01	1.00E+00
PKDCC	-3.64E+00	4.36E-104	GOLGA7B	1.66E-01	1.00E+00	SRF	-2.82E-01	1.00E+00
SPTBN4	-	4.54E-104	SMIM30	-2.77E-	1.00E+00	ZNF710	2.85E-01	1.00E+00

	4.42E+00			01				
NAA11	8.41E+00	4.67E-104	ETFA	-1.70E-01	1.00E+00	CNIH3	-2.82E-01	1.00E+00
SIAH3	5.32E+00	6.29E-104	DXO	2.73E-01	1.00E+00	ROBO2	-3.25E-01	1.00E+00
SYNRG	2.09E+00	6.33E-104	CLDN15	-7.78E-01	1.00E+00	CCL25	-5.23E-01	1.00E+00
UFSP2	-2.67E+00	6.45E-104	LOC112579549	8.76E-01	1.00E+00	PLK1	-2.63E-01	1.00E+00
MYH14	5.89E+00	6.90E-104	SYCE1L	-4.41E-01	1.00E+00	BPHL	-2.75E-01	1.00E+00
SLC25A28	-3.23E+00	9.71E-104	MBOAT7	-1.90E-01	1.00E+00	IL3RA	-4.19E-01	1.00E+00
MED14	3.14E+00	9.86E-104	MMP14	-8.87E-01	1.00E+00	EMP3	2.90E-01	1.00E+00
RAB12	-3.51E+00	1.20E-103	C2H6orf62	2.07E-01	1.00E+00	LOC112586816	6.40E-01	1.00E+00
ELAVL3	3.81E+00	1.32E-103	EIF2AK2	-3.11E-01	1.00E+00	CBLB	3.04E-01	1.00E+00
KIAA1671	-4.60E+00	1.32E-103	TNFRSF12A	-8.93E-01	1.00E+00	STK11IP	-2.32E-01	1.00E+00
LOC112588008	5.31E+00	1.34E-103	TTC4	-1.76E-01	1.00E+00	CR2	8.58E-01	1.00E+00
LOC102410517	-1.86E+00	1.38E-103	RIMS2	2.43E-01	1.00E+00	TULP3	2.63E-01	1.00E+00
ATP5MC2	-3.62E+00	1.41E-103	SLC25A13	1.43E-01	1.00E+00	SLC9A3R1	-2.53E-01	1.00E+00
TEKT1	5.91E+00	1.47E-103	ACSS1	-6.29E-01	1.00E+00	HS6ST3	5.59E-01	1.00E+00
ACOT9	2.56E+00	1.66E-103	ZBTB37	2.55E-01	1.00E+00	GDF9	-5.61E-01	1.00E+00
TBC1D15	2.47E+00	1.66E-103	H6PD	4.58E-01	1.00E+00	JUN	-5.80E-01	1.00E+00
PPP2R5C	-1.96E+00	1.83E-103	C5H11orf86	5.62E-01	1.00E+00	TBATA	5.48E-01	1.00E+00
OXCT1	-2.64E+00	1.92E-103	COL28A1	3.95E-01	1.00E+00	PCNX2	7.12E-01	1.00E+00
LOC112581539	7.56E+00	1.98E-103	THAP7	-2.50E-01	1.00E+00	SPAG5	-2.67E-01	1.00E+00
RAMAC	2.28E+00	2.87E-103	LOC102402338	-7.12E-01	1.00E+00	CDH17	5.05E-01	1.00E+00
LOC102409148	-6.27E+00	2.89E-103	FBLIM1	-1.95E-01	1.00E+00	NDUFAF8	3.79E-01	1.00E+00
MLF1	2.77E+00	3.05E-103	ATPAF2	-1.93E-01	1.00E+00	RPS27A	-5.19E-01	1.00E+00
HSD11B1L	-3.61E+00	3.49E-103	LOC112586233	4.38E-01	1.00E+00	ZSWIM4	3.99E-01	1.00E+00
ONECUT2	5.50E+00	3.61E-103	BCAM	-1.12E+00	1.00E+00	LOC112587187	-3.82E-01	1.00E+00
ICK	-4.70E+00	3.76E-103	LOC102398418	-6.09E-01	1.00E+00	PPFIBP1	4.02E-01	1.00E+00
HNRNPA2B1	-2.81E+00	3.80E-103	LOC102411979	2.94E-01	1.00E+00	ALS2CL	-2.69E-01	1.00E+00
TES	3.64E+00	4.49E-103	SLCO4A1	-1.14E+00	1.00E+00	EML6	4.87E-01	1.00E+00
PHKG2	-3.20E+00	4.58E-103	LOC102390572	-1.85E-01	1.00E+00	HACD4	2.93E-01	1.00E+00
TBCCD1	-4.37E+00	5.06E-103	ST5	2.19E-01	1.00E+00	F11R	-2.25E-01	1.00E+00
EGFL8	5.96E+00	5.67E-103	CDYL2	2.56E-01	1.00E+00	LOC102408803	-6.60E-01	1.00E+00
KCNMB4	2.59E+00	5.95E-103	SCTR	1.02E+00	1.00E+00	AMPD2	-1.98E-01	1.00E+00
LOC112578955	8.76E+00	5.97E-103	RPS27A	-4.67E-	1.00E+00	SLC27A4	2.71E-01	1.00E+00

				01				
ZDHHC2	-3.25E+00	6.58E-103	PARP10	-5.09E-01	1.00E+00	ARHGAP12	-2.49E-01	1.00E+00
LOC112584648	7.63E+00	7.97E-103	LOC102402718	-3.61E-01	1.00E+00	SRRM3	5.21E-01	1.00E+00
SNX7	-3.80E+00	8.24E-103	TMTC2	1.63E-01	1.00E+00	EPB41L3	2.56E-01	1.00E+00
ARNT	-3.97E+00	1.10E-102	MTMR12	1.55E-01	1.00E+00	LDLRAP1	-2.03E-01	1.00E+00
CLNS1A	-3.17E+00	1.21E-102	SERPINI1	4.97E-01	1.00E+00	CDADC1	2.36E-01	1.00E+00
IDH3B	-2.96E+00	1.21E-102	ARHGAP6	-7.45E-01	1.00E+00	LOC112584007	-3.06E-01	1.00E+00
ZNF316	-3.91E+00	1.57E-102	L2HGDH	-6.18E-01	1.00E+00	NBN	-2.01E-01	1.00E+00
ZNF274	2.68E+00	1.62E-102	MRPS5	-2.17E-01	1.00E+00	FTSJ3	2.16E-01	1.00E+00
CTSV	-5.77E+00	1.62E-102	NEK2	2.78E-01	1.00E+00	PCBD1	-4.04E-01	1.00E+00
CDK13	-2.75E+00	1.69E-102	SLC51B	-3.66E-01	1.00E+00	C2H2orf88	4.37E-01	1.00E+00
TMEM178B	2.37E+00	2.02E-102	GALNT11	-1.83E-01	1.00E+00	CNKSR1	-2.69E-01	1.00E+00
KHDC3L	1.14E+01	2.03E-102	CCDC106	-1.91E-01	1.00E+00	ADGRB2	-6.41E-01	1.00E+00
BOC	4.85E+00	2.10E-102	LOC112577660	-4.77E-01	1.00E+00	CEP170B	3.89E-01	1.00E+00
WDR73	-4.29E+00	2.15E-102	ZNF131	-3.76E-01	1.00E+00	TSPAN15	3.90E-01	1.00E+00
UBE2G1	1.99E+00	2.28E-102	USH2A	2.67E-01	1.00E+00	KRBA1	-2.50E-01	1.00E+00
AIG1	-2.98E+00	2.54E-102	ARHGAP18	-2.22E-01	1.00E+00	TRIB2	-3.59E-01	1.00E+00
FGFR1OP	2.00E+00	2.55E-102	SYCP2L	-2.76E-01	1.00E+00	MCF2L2	3.20E-01	1.00E+00
CCIN	7.31E+00	2.71E-102	NEMP1	1.83E-01	1.00E+00	SIRT3	-3.36E-01	1.00E+00
FBN1	-3.94E+00	2.90E-102	AKAP13	3.11E-01	1.00E+00	TTC14	-2.60E-01	1.00E+00
UNC13A	4.88E+00	3.08E-102	TECTA	-4.37E-01	1.00E+00	SRSF12	-2.88E-01	1.00E+00
USP48	2.12E+00	3.54E-102	LOC102400552	1.94E-01	1.00E+00	ELL	-2.46E-01	1.00E+00
STX16	-3.03E+00	3.58E-102	NRN1	-9.67E-01	1.00E+00	ARHGEF40	2.68E-01	1.00E+00
TGIF1	-4.15E+00	3.71E-102	B3GALT4	-2.65E-01	1.00E+00	NUDT9	2.15E-01	1.00E+00
MYZAP	-4.71E+00	3.77E-102	LONRF1	2.10E-01	1.00E+00	RPS15A	-5.83E-01	1.00E+00
RAD54B	2.31E+00	4.72E-102	ZNF341	3.20E-01	1.00E+00	PIWIL2	-4.63E-01	1.00E+00
LOC112586105	8.63E+00	5.28E-102	E2F5	4.05E-01	1.00E+00	PLEKHF1	5.41E-01	1.00E+00
ZNF366	8.39E+00	5.72E-102	DENND4A	1.75E-01	1.00E+00	AKAP7	-3.48E-01	1.00E+00
KIF3B	2.50E+00	6.62E-102	ARL10	1.57E-01	1.00E+00	LOC112582100	5.49E-01	1.00E+00
ZC2HC1A	2.98E+00	6.70E-102	CDSN	7.40E-01	1.00E+00	SEMA6C	-3.54E-01	1.00E+00
PEX3	2.54E+00	7.11E-102	OAT	1.82E-01	1.00E+00	AK6	2.43E-01	1.00E+00
ZNF326	-3.69E+00	7.22E-102	COBLL1	2.69E-01	1.00E+00	FOXL2	-3.18E-01	1.00E+00
PIGS	-3.27E+00	7.84E-102	LOC112578003	-7.52E-01	1.00E+00	PRRX2	5.01E-01	1.00E+00
AHDC1	-	8.07E-102	GABRA3	2.41E-01	1.00E+00	SLC6A17	5.64E-01	1.00E+00

	3.77E+00							
VEZT	-2.41E+00	8.12E-102	FAM189A1	-5.49E-01	1.00E+00	ADAP2	-3.65E-01	1.00E+00
TMED8	2.46E+00	1.36E-101	LOC112584711	4.57E-01	1.00E+00	CNN1	4.19E-01	1.00E+00
LOC102407725	7.96E+00	1.36E-101	ANKFY1	2.23E-01	1.00E+00	KIF9	-4.01E-01	1.00E+00
ZNF827	-3.79E+00	1.40E-101	PIK3R5	4.41E-01	1.00E+00	BTG1	-2.68E-01	1.00E+00
CREBL2	3.00E+00	1.44E-101	PID1	-3.09E-01	1.00E+00	FAM20A	5.25E-01	1.00E+00
NLRP8	1.12E+01	1.55E-101	RPA1	1.47E-01	1.00E+00	SLC44A5	5.01E-01	1.00E+00
TUBB6	-4.87E+00	1.62E-101	MON1B	2.03E-01	1.00E+00	FRMD4A	3.18E-01	1.00E+00
LRP1	-5.39E+00	1.70E-101	IER2	-8.47E-01	1.00E+00	WSB1	-3.05E-01	1.00E+00
ASTN2	4.67E+00	1.81E-101	PSAP	1.55E-01	1.00E+00	APOA1	-3.76E-01	1.00E+00
AJUBA	-3.84E+00	1.82E-101	KMT2B	2.37E-01	1.00E+00	TBC1D22B	-2.16E-01	1.00E+00
CFAP44	8.18E+00	1.90E-101	CRABP2	-7.83E-01	1.00E+00	OGFR	2.61E-01	1.00E+00
NCBP1	2.12E+00	2.35E-101	LOC112583626	-6.73E-01	1.00E+00	RYR3	-4.05E-01	1.00E+00
GPC6	-3.11E+00	2.38E-101	PPP1R14B	-2.57E-01	1.00E+00	WDR12	2.31E-01	1.00E+00
TMEM88B	8.09E+00	2.47E-101	CKS2	-2.19E-01	1.00E+00	GJB3	4.26E-01	1.00E+00
INIP	2.09E+00	2.69E-101	AHCY	1.47E-01	1.00E+00	RIMS2	3.68E-01	1.00E+00
SNX2	-2.24E+00	2.84E-101	CLCN7	2.84E-01	1.00E+00	HCFC1	-2.26E-01	1.00E+00
TXNDC15	-3.19E+00	3.34E-101	IMPDH2	-2.32E-01	1.00E+00	ATF6B	-2.52E-01	1.00E+00
USP36	2.15E+00	3.36E-101	RPL29	-2.14E-01	1.00E+00	FECH	-2.39E-01	1.00E+00
SAMD1	-3.60E+00	3.58E-101	C11H14orf119	-1.66E-01	1.00E+00	GALNT18	-3.46E-01	1.00E+00
GOLT1B	2.21E+00	3.86E-101	MAFF	-4.68E-01	1.00E+00	LOC112585578	-4.07E-01	1.00E+00
TCHH	7.15E+00	4.93E-101	SIDT2	1.90E-01	1.00E+00	NLRP8	-7.39E-01	1.00E+00
ARL8A	-4.88E+00	5.47E-101	POU4F3	6.63E-01	1.00E+00	LOC102395838	-4.49E-01	1.00E+00
LOC112581171	5.97E+00	5.75E-101	PSMD2	1.42E-01	1.00E+00	FREM2	-4.06E-01	1.00E+00
LITAF	-5.15E+00	5.85E-101	MAP4K4	1.70E-01	1.00E+00	RARB	6.72E-01	1.00E+00
NPR2	-2.95E+00	5.97E-101	SLC25A26	1.78E-01	1.00E+00	SPECC1	2.51E-01	1.00E+00
MAGIX	3.55E+00	7.25E-101	SRC	-3.70E-01	1.00E+00	CAMK2A	6.11E-01	1.00E+00
ROR2	-5.36E+00	7.55E-101	ATG12	1.53E-01	1.00E+00	RABAC1	2.93E-01	1.00E+00
ARRDC4	-3.77E+00	1.03E-100	LOC102412637	-1.08E+00	1.00E+00	LOC102415609	3.29E-01	1.00E+00
DCK	2.63E+00	1.03E-100	SF3B4	1.35E-01	1.00E+00	LRRC72	3.60E-01	1.00E+00
TKT	-4.13E+00	1.08E-100	SIRPB2	-5.91E-01	1.00E+00	DBN1	2.43E-01	1.00E+00
LOC102402877	-6.29E+00	1.09E-100	CTTNBP2	2.30E-01	1.00E+00	GPR137C	4.88E-01	1.00E+00
RAPGEF2	-2.40E+00	1.30E-100	ARHGEF38	-4.45E-01	1.00E+00	GPC1	2.52E-01	1.00E+00
EMID1	-4.63E+00	1.35E-100	RD3	-3.65E-01	1.00E+00	PRR11	-3.68E-01	1.00E+00

LOC112582010	4.59E+00	1.61E-100	LOC102390320	-6.13E-01	1.00E+00	LOC102397479	3.90E-01	1.00E+00
MAP3K14	2.57E+00	1.64E-100	WDR26	1.61E-01	1.00E+00	REPIN1	2.60E-01	1.00E+00
PHOX2A	5.26E+00	1.66E-100	HTD2	3.42E-01	1.00E+00	CDC42EP1	3.56E-01	1.00E+00
FANK1	3.58E+00	1.73E-100	TSC22D4	2.03E-01	1.00E+00	TAPBP	-2.51E-01	1.00E+00
SAXO1	7.99E+00	2.11E-100	ESPL1	2.23E-01	1.00E+00	LHX2	-4.11E-01	1.00E+00
LPCAT4	-2.84E+00	2.38E-100	LOC112582964	-7.14E-01	1.00E+00	ZWILCH	-2.41E-01	1.00E+00
SAP30BP	1.87E+00	2.81E-100	PLEKHG4	2.11E-01	1.00E+00	PSKH1	2.37E-01	1.00E+00
LOC102412878	2.34E+00	2.82E-100	PPP6C	2.27E-01	1.00E+00	ACTA2	4.23E-01	1.00E+00
SIPA1L3	2.11E+00	2.84E-100	LOC112578124	3.38E-01	1.00E+00	FAAP20	2.80E-01	1.00E+00
CAVIN1	-6.46E+00	2.84E-100	PUS7L	2.08E-01	1.00E+00	TBRG1	2.03E-01	1.00E+00
RGL1	2.95E+00	2.93E-100	PGAP3	-1.85E-01	1.00E+00	KCNC3	4.41E-01	1.00E+00
ABHD13	-3.53E+00	2.96E-100	CREBBP	2.63E-01	1.00E+00	LACC1	3.73E-01	1.00E+00
C8H7orf31	5.95E+00	3.29E-100	INPP4B	-3.91E-01	1.00E+00	DVL2	-2.00E-01	1.00E+00
HDAC11	3.81E+00	3.30E-100	TRRAP	3.21E-01	1.00E+00	LOC102409467	-9.87E-01	1.00E+00
PRPF38B	-2.68E+00	3.30E-100	LRRC42	-1.53E-01	1.00E+00	GALNTL6	3.26E-01	1.00E+00
ZNF813	7.28E+00	3.77E-100	SNX9	1.53E-01	1.00E+00	CHD2	-2.08E-01	1.00E+00
CAPN5	-4.58E+00	3.80E-100	PEBP1	-1.52E-01	1.00E+00	DAPK3	2.91E-01	1.00E+00
PIGO	-2.71E+00	3.86E-100	SECISBP2L	2.01E-01	1.00E+00	KLHL32	3.04E-01	1.00E+00
LOC112580365	1.03E+01	4.11E-100	ANAPC10	-2.19E-01	1.00E+00	MMP17	3.86E-01	1.00E+00
UCHL3	2.66E+00	4.59E-100	ZC3HC1	-1.71E-01	1.00E+00	RAPGEF5	5.86E-01	1.00E+00
PGK1	-3.58E+00	5.21E-100	KBTBD7	2.12E-01	1.00E+00	SIAE	-2.12E-01	1.00E+00
GFI1B	7.97E+00	5.41E-100	LOC112578241	-9.32E-01	1.00E+00	NEK11	3.48E-01	1.00E+00
MLLT11	2.43E+00	5.71E-100	LOC102416423	-5.68E-01	1.00E+00	RGS7	2.80E-01	1.00E+00
PANK4	-2.73E+00	7.71E-100	PGF	9.76E-01	1.00E+00	WDR72	5.69E-01	1.00E+00
DAPK1	-4.50E+00	8.20E-100	LOC112585287	-3.57E-01	1.00E+00	SH2D6	4.66E-01	1.00E+00
CIITA	6.41E+00	9.01E-100	LOC102396266	-6.96E-01	1.00E+00	LRRC47	-2.28E-01	1.00E+00
IL6ST	-3.43E+00	1.15E-99	SYS1	1.84E-01	1.00E+00	ST6GALNAC6	-2.61E-01	1.00E+00
YBX1	-4.02E+00	1.16E-99	MPG	-2.01E-01	1.00E+00	FADS1	-2.74E-01	1.00E+00
PADI6	1.09E+01	1.19E-99	PDZD2	1.34E-01	1.00E+00	IGSF10	5.58E-01	1.00E+00
WDPCP	2.85E+00	1.27E-99	TMEM237	-3.40E-01	1.00E+00	LOC102412769	-4.49E-01	1.00E+00
ANXA5	-2.80E+00	1.28E-99	LOC102395002	1.89E-01	1.00E+00	BAIAP2	2.81E-01	1.00E+00
NNT	-2.28E+00	1.36E-99	WWOX	1.71E-01	1.00E+00	RIOK3	-2.97E-01	1.00E+00
RPGRIP1L	2.52E+00	1.37E-99	IL34	5.93E-01	1.00E+00	ID1	-5.58E-01	1.00E+00
PEX5	1.94E+00	1.46E-99	MED7	-2.01E-01	1.00E+00	NALCN	-2.47E-01	1.00E+00

KIAA1217	2.30E+00	1.57E-99	ZRANB2	1.69E-01	1.00E+00	PNPLA7	-2.18E-01	1.00E+00
CROCC	-5.22E+00	2.24E-99	LOC112579184	6.27E-01	1.00E+00	LOC112587462	5.09E-01	1.00E+00
LEUTX	8.88E+00	2.66E-99	NDUFA4L2	1.07E+00	1.00E+00	MSANTD1	-4.39E-01	1.00E+00
FOXN4	7.09E+00	2.71E-99	FANCA	3.00E-01	1.00E+00	MYO1G	-1.03E+00	1.00E+00
APRT	-4.31E+00	2.80E-99	LOC102397317	4.15E-01	1.00E+00	ADGRG2	2.64E-01	1.00E+00
NUCB1	-2.71E+00	3.23E-99	SGIP1	4.69E-01	1.00E+00	TP53I11	3.97E-01	1.00E+00
SCN4B	4.52E+00	3.52E-99	CETN3	-1.71E-01	1.00E+00	TEP1	-2.03E-01	1.00E+00
LOC112578777	7.95E+00	3.74E-99	RNF144B	1.53E-01	1.00E+00	CCDC86	2.67E-01	1.00E+00
METTL17	-5.25E+00	4.14E-99	NAB2	-3.57E-01	1.00E+00	TUBE1	-2.85E-01	1.00E+00
EPB41L3	-3.04E+00	5.54E-99	PPP2R1B	1.54E-01	1.00E+00	C1QTNF6	-3.46E-01	1.00E+00
KIF5A	4.19E+00	5.97E-99	RAC3	-2.31E-01	1.00E+00	LOC112586495	-4.89E-01	1.00E+00
BCAS3	2.03E+00	7.19E-99	IDH1	-2.61E-01	1.00E+00	BHLHE22	2.20E-01	1.00E+00
SMDT1	-4.83E+00	7.42E-99	WDFY4	-9.35E-01	1.00E+00	LNP1	-2.86E-01	1.00E+00
THRAP3	-2.11E+00	7.70E-99	PIGP	-1.98E-01	1.00E+00	ABHD17C	2.44E-01	1.00E+00
CNKSR1	2.54E+00	8.28E-99	LOC102396103	-1.61E-01	1.00E+00	TPX2	-2.40E-01	1.00E+00
ZDHHC5	2.37E+00	8.57E-99	SCFD2	2.43E-01	1.00E+00	CHAF1A	-2.20E-01	1.00E+00
PTN	-7.26E+00	9.54E-99	LOC102409611	6.05E-01	1.00E+00	DEPDC1	-3.17E-01	1.00E+00
TMPRSS3	6.88E+00	9.56E-99	TULP4	2.77E-01	1.00E+00	SRPK3	4.29E-01	1.00E+00
ANKRD13A	2.20E+00	1.23E-98	RNF182	6.23E-01	1.00E+00	NR4A1	-5.36E-01	1.00E+00
GOLGA7	1.89E+00	1.28E-98	HACD4	-1.75E-01	1.00E+00	PERP	4.79E-01	1.00E+00
PARL	2.31E+00	1.31E-98	LOC102397361	-5.05E-01	1.00E+00	KCNIP4	-3.38E-01	1.00E+00
CHN1	2.48E+00	1.33E-98	VBP1	-1.99E-01	1.00E+00	GNA14	-3.92E-01	1.00E+00
RCN1	-3.30E+00	1.42E-98	LOC112580915	-6.99E-01	1.00E+00	LOC102398019	4.60E-01	1.00E+00
PHF20	2.40E+00	1.44E-98	HIPK3	-3.95E-01	1.00E+00	JAK3	-3.43E-01	1.00E+00
ELOA	-3.45E+00	1.44E-98	SPSB4	-8.58E-01	1.00E+00	PSTPIP2	4.54E-01	1.00E+00
LIPT2	4.43E+00	1.49E-98	TP53I13	2.69E-01	1.00E+00	DOHH	2.54E-01	1.00E+00
LOC102405836	4.89E+00	1.57E-98	C21H3orf49	-3.28E-01	1.00E+00	LOC112583913	-5.31E-01	1.00E+00
MKNK1	2.35E+00	1.66E-98	DSCAM	-5.92E-01	1.00E+00	BMP15	-6.59E-01	1.00E+00
FUS	-2.87E+00	1.87E-98	ANGPTL4	-5.59E-01	1.00E+00	KCNN2	-3.95E-01	1.00E+00
LOC102411389	-7.41E+00	1.88E-98	WDR18	1.98E-01	1.00E+00	MCUB	2.70E-01	1.00E+00
CRNKL1	1.81E+00	2.35E-98	LOC112581276	-7.21E-01	1.00E+00	WFS1	-2.46E-01	1.00E+00
GABARAP	-3.38E+00	2.39E-98	PSKH1	2.03E-01	1.00E+00	INCENP	-2.43E-01	1.00E+00
LOC112585135	8.00E+00	2.55E-98	IHH	1.35E+00	1.00E+00	PRPF40B	-2.14E-01	1.00E+00

JPT2	2.48E+00	2.58E-98	THAP12	-8.04E-01	1.00E+00	ACSL1	-2.86E-01	1.00E+00
SORBS2	-3.91E+00	2.61E-98	PLEKHG5	4.78E-01	1.00E+00	NAALAD2	-3.58E-01	1.00E+00
LRR1	3.21E+00	2.62E-98	SLC1A4	2.39E-01	1.00E+00	NICN1	-2.00E-01	1.00E+00
SEC63	-2.06E+00	2.64E-98	DUSP11	-2.84E-01	1.00E+00	PIGO	-2.05E-01	1.00E+00
PITPNB	-2.19E+00	2.69E-98	RFFL	1.50E-01	1.00E+00	TUSC1	3.04E-01	1.00E+00
ILDR2	4.34E+00	2.69E-98	FAM181B	-6.55E-01	1.00E+00	ATG16L2	3.05E-01	1.00E+00
PDE7B	-3.66E+00	3.00E-98	LOC102398210	4.27E-01	1.00E+00	NMI	2.19E-01	1.00E+00
KAT7	2.09E+00	3.23E-98	LOC112584008	4.70E-01	1.00E+00	BRSK1	2.52E-01	1.00E+00
RALGDS	2.18E+00	3.30E-98	DHX57	2.00E-01	1.00E+00	PACSIN3	2.99E-01	1.00E+00
STARD7	-3.35E+00	3.51E-98	L1CAM	2.93E-01	1.00E+00	MYEF2	-3.66E-01	1.00E+00
CHGB	6.84E+00	4.06E-98	RUNX1T1	1.46E-01	1.00E+00	LOC102390387	5.83E-01	1.00E+00
STK24	2.04E+00	4.12E-98	CCL22	5.40E-01	1.00E+00	SLC25A34	-5.29E-01	1.00E+00
PEMT	-4.16E+00	4.32E-98	LOC102395434	-2.60E-01	1.00E+00	ZC3H7A	-2.28E-01	1.00E+00
TTK	2.71E+00	4.48E-98	CAMSAP3	2.52E-01	1.00E+00	MFSD10	2.19E-01	1.00E+00
FAM227B	2.91E+00	4.50E-98	ING5	1.73E-01	1.00E+00	ITGAV	3.88E-01	1.00E+00
SLC38A2	-5.09E+00	4.66E-98	GLRA4	-6.14E-01	1.00E+00	LOC112580450	-6.59E-01	1.00E+00
CSTF2	2.11E+00	4.75E-98	PDZK1	-2.14E-01	1.00E+00	TIGIT	-4.81E-01	1.00E+00
PLEKHH3	-4.47E+00	4.90E-98	LOC102394903	-3.21E-01	1.00E+00	PDGFB	6.69E-01	1.00E+00
KIAA1211L	5.01E+00	5.10E-98	ZNF3	-2.66E-01	1.00E+00	ELK3	6.26E-01	1.00E+00
NLRC5	4.96E+00	5.23E-98	GGCT	-2.08E-01	1.00E+00	U2SURP	-1.97E-01	1.00E+00
MSANTD2	-3.34E+00	5.83E-98	MTFR1	1.48E-01	1.00E+00	BMI1	2.86E-01	1.00E+00
SLC25A10	-2.76E+00	6.39E-98	TRPC3	1.08E+00	1.00E+00	MRPL33	2.40E-01	1.00E+00
BAIAP2	2.80E+00	6.59E-98	CLEC4F	4.09E-01	1.00E+00	TICRR	-2.65E-01	1.00E+00
PNPLA6	-2.73E+00	6.59E-98	OCRL	1.56E-01	1.00E+00	E2F8	-2.82E-01	1.00E+00
KLF3	-3.76E+00	6.79E-98	GTF3C2	1.50E-01	1.00E+00	TRIM68	-2.47E-01	1.00E+00
DGCR2	-3.26E+00	7.21E-98	LTBP4	-4.08E-01	1.00E+00	APTX	-2.41E-01	1.00E+00
LOC102396919	-3.64E+00	1.11E-97	SHISA4	3.97E-01	1.00E+00	PTPRT	-6.00E-01	1.00E+00
PLCD1	-6.15E+00	1.15E-97	TRIM54	-6.89E-01	1.00E+00	DYSF	-2.66E-01	1.00E+00
ERO1A	-3.04E+00	1.19E-97	LOC102398588	-3.38E-01	1.00E+00	TONSL	-2.66E-01	1.00E+00
TCIM	-9.82E+00	1.46E-97	RAB11FIP4	-1.72E-01	1.00E+00	MOSPD1	3.16E-01	1.00E+00
BGN	-8.13E+00	1.50E-97	KIF26B	-2.62E-01	1.00E+00	CDH2	2.82E-01	1.00E+00
LOC112581847	4.76E+00	1.54E-97	PRR3	2.04E-01	1.00E+00	IFIT3	4.97E-01	1.00E+00
SMAD4	-2.95E+00	1.57E-97	NR2C2	1.43E-01	1.00E+00	LONRF1	-3.07E-01	1.00E+00
PPID	-2.80E+00	1.70E-97	PSD4	2.06E-01	1.00E+00	TAF4	-2.48E-01	1.00E+00

LOC102410561	9.80E+00	1.75E-97	MDM1	2.09E-01	1.00E+00	UBE2QL1	-4.32E-01	1.00E+00
FBRSL1	2.55E+00	1.84E-97	NUP153	2.27E-01	1.00E+00	ZP4	-7.88E-01	1.00E+00
XYLT2	-3.82E+00	1.84E-97	HIPK1	2.76E-01	1.00E+00	GNAL	2.09E-01	1.00E+00
GNG10	-2.41E+00	1.86E-97	PGS1	1.56E-01	1.00E+00	LOC112583857	-2.29E-01	1.00E+00
RHBDD2	-2.88E+00	1.94E-97	TMEM234	3.81E-01	1.00E+00	KIFC2	-2.18E-01	1.00E+00
MOV10L1	6.98E+00	1.98E-97	ENPP5	2.60E-01	1.00E+00	KCNE5	5.02E-01	1.00E+00
ZNF174	2.54E+00	2.17E-97	PTGDR2	-4.55E-01	1.00E+00	PRRG3	-3.00E-01	1.00E+00
OBSL1	-4.78E+00	2.54E-97	CMC2	-2.09E-01	1.00E+00	FAM213A	-2.48E-01	1.00E+00
NENF	-3.95E+00	2.64E-97	MGRN1	2.07E-01	1.00E+00	SRSF11	-2.36E-01	1.00E+00
CCDC77	-3.89E+00	3.09E-97	LOC112587372	-9.31E-01	1.00E+00	PAPPA	-3.61E-01	1.00E+00
CDH6	-4.20E+00	3.44E-97	ABCA1	-3.57E-01	1.00E+00	TCF7L1	5.20E-01	1.00E+00
ANKRD10	-2.46E+00	3.49E-97	MTURN	2.13E-01	1.00E+00	ASAH1	2.29E-01	1.00E+00
MROH1	-3.95E+00	3.57E-97	LOC112587338	-8.41E-01	1.00E+00	LOC102413340	4.96E-01	1.00E+00
HMBS	-3.86E+00	3.58E-97	SMG5	1.52E-01	1.00E+00	ADGRL4	5.17E-01	1.00E+00
NAGA	-2.40E+00	3.93E-97	TRAM2	1.89E-01	1.00E+00	LOC102403829	-2.27E-01	1.00E+00
C18H19orf48	-3.93E+00	4.24E-97	SLU7	-2.28E-01	1.00E+00	H6PD	-2.28E-01	1.00E+00
INPP5E	-4.03E+00	4.38E-97	PRR14	1.65E-01	1.00E+00	ZNF346	-2.69E-01	1.00E+00
DDX17	-3.48E+00	4.44E-97	MGAT4B	1.45E-01	1.00E+00	DSEL	2.69E-01	1.00E+00
KIAA1614	-3.28E+00	4.45E-97	LDB3	2.43E-01	1.00E+00	DBNDD2	4.13E-01	1.00E+00
BOLL	7.02E+00	4.81E-97	TOR1A	-1.62E-01	1.00E+00	HORMAD2	5.73E-01	1.00E+00
SNAPC3	-2.14E+00	5.19E-97	PLPPR1	-2.14E-01	1.00E+00	LENG9	3.93E-01	1.00E+00
NMUR2	6.22E+00	6.83E-97	FUT11	1.14E+00	1.00E+00	ZBTB42	3.50E-01	1.00E+00
FBXW11	2.91E+00	6.85E-97	LOC112578085	-3.55E-01	1.00E+00	LOC102408196	5.75E-01	1.00E+00
RHOBTB3	-4.01E+00	6.98E-97	ACSF2	-7.94E-01	1.00E+00	TTLL3	-2.29E-01	1.00E+00
E2F2	3.42E+00	7.90E-97	FLT1	-1.89E-01	1.00E+00	PTPRD	2.83E-01	1.00E+00
MIP	7.77E+00	8.68E-97	GRB14	-7.98E-01	1.00E+00	LOC112586135	-4.71E-01	1.00E+00
MAP2K4	1.84E+00	9.38E-97	IQCE	-7.62E-01	1.00E+00	PTPN22	4.25E-01	1.00E+00
PLA2G12B	6.61E+00	1.00E-96	NLN	2.27E-01	1.00E+00	LOC102415279	2.33E-01	1.00E+00
TMEM120A	-3.67E+00	1.03E-96	PPME1	1.89E-01	1.00E+00	APH1B	-3.28E-01	1.00E+00
PTCH2	3.97E+00	1.31E-96	DUT	-1.62E-01	1.00E+00	ABLIM3	5.93E-01	1.00E+00
HIP1	2.99E+00	1.36E-96	WRNIP1	1.37E-01	1.00E+00	TRIM25	-2.27E-01	1.00E+00
LOC112587405	8.12E+00	1.41E-96	SCML1	-6.22E-01	1.00E+00	OSGEP	-2.05E-01	1.00E+00
POMGNT1	-2.55E+00	1.52E-96	DIABLO	4.16E-01	1.00E+00	SGO1	-2.67E-01	1.00E+00

MVB12A	-3.71E+00	1.62E-96	GABPB1	1.95E-01	1.00E+00	BRI3BP	-2.40E-01	1.00E+00
FBXO5	2.84E+00	1.71E-96	DNAH9	-5.85E-01	1.00E+00	MMP12	8.80E-01	1.00E+00
TMEM178A	5.16E+00	1.85E-96	LOC102409438	-1.33E+00	1.00E+00	LGALS3BP	-2.65E-01	1.00E+00
MARC2	-3.67E+00	1.85E-96	TATDN2	1.67E-01	1.00E+00	SMC4	-2.28E-01	1.00E+00
LOC112581557	8.09E+00	2.11E-96	SLIT1	-5.62E-01	1.00E+00	CPEB2	4.15E-01	1.00E+00
MYOF	-2.57E+00	2.16E-96	PGM2L1	2.44E-01	1.00E+00	PADI6	-7.62E-01	1.00E+00
LOC102401189	-6.45E+00	2.43E-96	FOXE3	5.73E-01	1.00E+00	CMTM8	3.30E-01	1.00E+00
LOC102392547	1.01E+01	2.53E-96	LOC112585754	-6.50E-01	1.00E+00	CNN2	2.56E-01	1.00E+00
RPS14	-5.03E+00	2.56E-96	RUBCN	2.79E-01	1.00E+00	PTPN4	-2.24E-01	1.00E+00
TENT5C	4.89E+00	2.66E-96	CPAMD8	-6.69E-01	1.00E+00	CENPN	-2.90E-01	1.00E+00
LDB3	4.81E+00	2.78E-96	RPN2	1.55E-01	1.00E+00	EIF2AK3	-2.45E-01	1.00E+00
CTIF	-4.14E+00	3.15E-96	TIGIT	-1.02E+00	1.00E+00	FAM102A	-2.61E-01	1.00E+00
ISL2	5.96E+00	3.43E-96	THAP2	2.81E-01	1.00E+00	ASPM	-2.88E-01	1.00E+00
PRPF4	1.92E+00	3.59E-96	BICRA	2.14E-01	1.00E+00	LOC112581727	5.83E-01	1.00E+00
POFUT1	-2.75E+00	3.95E-96	LOC102407981	5.50E-01	1.00E+00	CCDC90B	-2.08E-01	1.00E+00
RMC1	2.28E+00	4.34E-96	CLIC1	-2.89E-01	1.00E+00	IKZF5	-2.85E-01	1.00E+00
VOPP1	-3.50E+00	4.40E-96	CCDC167	-1.94E-01	1.00E+00	ITPKC	2.12E-01	1.00E+00
THEGL	9.05E+00	4.43E-96	MITD1	4.11E-01	1.00E+00	TCF25	-2.21E-01	1.00E+00
PSPH	2.91E+00	4.49E-96	ADAM20	1.93E-01	1.00E+00	MAGT1	-2.06E-01	1.00E+00
KAT14	-2.31E+00	4.82E-96	METTL8	2.08E-01	1.00E+00	RAB36	-2.95E-01	1.00E+00
LOC112578959	-7.49E+00	5.16E-96	ZSWIM8	2.02E-01	1.00E+00	TUBGCP5	-2.60E-01	1.00E+00
ASB11	2.76E+00	5.37E-96	VASH1	3.80E-01	1.00E+00	TMEM106A	-2.91E-01	1.00E+00
LTBP3	-4.26E+00	5.68E-96	FRMD8	2.30E-01	1.00E+00	ANGEL1	-2.17E-01	1.00E+00
YARS	-2.58E+00	5.73E-96	LANCL3	2.45E-01	1.00E+00	ZNF516	6.04E-01	1.00E+00
LOC112585042	5.73E+00	5.75E-96	RPL30	-2.08E-01	1.00E+00	ELMO1	4.59E-01	1.00E+00
RABGGTB	-2.10E+00	5.85E-96	COL4A6	-4.09E-01	1.00E+00	TMEM232	4.60E-01	1.00E+00
TIFA	4.41E+00	7.42E-96	SORBS3	2.37E-01	1.00E+00	CNTNAP5	5.80E-01	1.00E+00
CNTLN	-2.61E+00	7.94E-96	KLHL5	2.16E-01	1.00E+00	CLK2	-1.87E-01	1.00E+00
ALDH3A2	-3.61E+00	8.33E-96	LOC112580357	-7.44E-01	1.00E+00	SLC16A2	4.93E-01	1.00E+00
TCP11	3.65E+00	8.53E-96	AQR	2.79E-01	1.00E+00	NOS2	-2.67E-01	1.00E+00
NRP1	-5.15E+00	9.29E-96	JARID2	2.47E-01	1.00E+00	LOC102391231	5.08E-01	1.00E+00
CBY1	-3.21E+00	1.12E-95	TTBK2	2.90E-01	1.00E+00	TIAM2	2.11E-01	1.00E+00
TLE3	-4.48E+00	1.15E-95	TSR3	3.35E-01	1.00E+00	RPS6KA2	2.31E-01	1.00E+00

TAP1	-2.75E+00	1.19E-95	SLC2A6	4.76E-01	1.00E+00	POLR2A	-2.25E-01	1.00E+00
CTDSPL	-3.01E+00	1.42E-95	ARF4	1.44E-01	1.00E+00	KIFAP3	2.05E-01	1.00E+00
SNRPB	-2.59E+00	1.48E-95	PTPN4	2.56E-01	1.00E+00	BCL2L12	2.14E-01	1.00E+00
ZNF8	-3.35E+00	1.54E-95	TPBG	-8.55E-01	1.00E+00	SLAIN1	2.24E-01	1.00E+00
ACSS2	-3.56E+00	1.74E-95	SCAF1	1.76E-01	1.00E+00	LOC102400714	2.42E-01	1.00E+00
SEPT8	2.54E+00	1.74E-95	USF3	-2.30E-01	1.00E+00	ENTPD7	-3.42E-01	1.00E+00
PRRC2B	-3.27E+00	2.03E-95	PIAS1	1.66E-01	1.00E+00	BAALC	5.06E-01	1.00E+00
ENC1	-6.14E+00	2.14E-95	LOC112583470	1.64E-01	1.00E+00	BDNF	5.79E-01	1.00E+00
PDXP	2.52E+00	2.69E-95	RPS17	-2.04E-01	1.00E+00	FBXL19	2.25E-01	1.00E+00
BOD1L1	-4.90E+00	2.70E-95	SH3GL3	-9.54E-01	1.00E+00	CLIC1	2.76E-01	1.00E+00
DAZAP1	-2.12E+00	2.76E-95	FBXL17	-3.19E-01	1.00E+00	SYF2	-2.35E-01	1.00E+00
SOX12	-3.15E+00	3.09E-95	AFMID	4.03E-01	1.00E+00	CDC25B	-2.92E-01	1.00E+00
STAG1	-2.29E+00	3.21E-95	LRRC14	6.42E-01	1.00E+00	LOC112581152	-8.05E-01	1.00E+00
SLC44A2	-2.03E+00	4.57E-95	PAFAH2	-4.23E-01	1.00E+00	LOC102408084	5.01E-01	1.00E+00
FCGRT	-2.94E+00	4.62E-95	SUN3	-5.01E-01	1.00E+00	LOC102401601	5.39E-01	1.00E+00
CEP85	2.54E+00	4.83E-95	CALU	1.68E-01	1.00E+00	SNAI2	5.49E-01	1.00E+00
TBL1X	-2.43E+00	6.59E-95	LOC102408654	-7.91E-01	1.00E+00	LOC102389856	-4.46E-01	1.00E+00
NEB	5.69E+00	6.63E-95	GALNT13	1.70E-01	1.00E+00	HK2	-2.94E-01	1.00E+00
CRELD1	-2.73E+00	6.90E-95	LOC112583730	3.31E-01	1.00E+00	SMAD7	3.99E-01	1.00E+00
KCTD13	1.99E+00	6.91E-95	NR1H3	2.34E-01	1.00E+00	PQLC1	2.91E-01	1.00E+00
ZBED8	-3.78E+00	7.56E-95	ABI3	-4.74E-01	1.00E+00	STK38L	3.11E-01	1.00E+00
YAP1	-2.41E+00	7.92E-95	AFF4	-6.40E-01	1.00E+00	LOC112579839	4.65E-01	1.00E+00
LOC102392450	1.02E+01	8.89E-95	SETD1A	2.14E-01	1.00E+00	MXRA7	2.81E-01	1.00E+00
RNF111	2.94E+00	9.25E-95	ZFP57	8.52E-01	1.00E+00	SERPINF1	-4.67E-01	1.00E+00
ASPH	-3.97E+00	9.70E-95	LOC112580904	7.98E-01	1.00E+00	FGF14	-3.37E-01	1.00E+00
RHOBTB1	-2.87E+00	1.03E-94	LOC112581803	4.96E-01	1.00E+00	LOC102402496	-5.52E-01	1.00E+00
LOC112578440	9.59E+00	1.13E-94	GRM3	-3.53E-01	1.00E+00	SFXN2	-2.91E-01	1.00E+00
SETD2	-3.37E+00	1.16E-94	PHLPP1	1.50E-01	1.00E+00	EME1	-2.46E-01	1.00E+00
ADM	-5.67E+00	1.17E-94	CDC25A	1.80E-01	1.00E+00	RAD51AP1	-2.55E-01	1.00E+00
SIAH1	2.63E+00	1.35E-94	LOC112583825	-5.79E-01	1.00E+00	THSD4	6.27E-01	1.00E+00
ASAP1	2.93E+00	1.44E-94	LOC102414934	-1.99E-01	1.00E+00	LBR	-3.95E-01	1.00E+00
THSD1	4.27E+00	1.58E-94	ACSS3	2.96E-01	1.00E+00	LOC102409002	-2.33E-01	1.00E+00
ZUP1	1.83E+00	1.59E-94	MRPL50	-3.46E-01	1.00E+00	MAP3K8	4.24E-01	1.00E+00

ORC3	2.10E+00	1.64E-94	ENPEP	-5.82E-01	1.00E+00	MORF4L1	-1.92E-01	1.00E+00
TRIM25	-4.96E+00	1.99E-94	PRRX1	6.97E-01	1.00E+00	ACTR1B	2.09E-01	1.00E+00
EEPD1	2.82E+00	2.08E-94	TMEM47	1.29E-01	1.00E+00	LOC102415310	-4.99E-01	1.00E+00
LOC112585084	-8.84E+00	2.08E-94	LOC112584596	-4.12E-01	1.00E+00	VPS37B	1.98E-01	1.00E+00
SLC12A7	-3.52E+00	2.09E-94	FAM76A	4.04E-01	1.00E+00	ZACN	3.95E-01	1.00E+00
RNPEP	-3.75E+00	2.10E-94	TRIM71	3.07E-01	1.00E+00	WIPI1	-1.95E-01	1.00E+00
IL10RB	-5.76E+00	2.19E-94	DNAJA2	-1.36E-01	1.00E+00	DUSP14	-2.48E-01	1.00E+00
HNRNPH1	-3.28E+00	2.54E-94	LOC112579902	-3.41E-01	1.00E+00	S100A11	3.23E-01	1.00E+00
RPL30	-4.49E+00	2.59E-94	SNX32	7.87E-01	1.00E+00	GOLGA1	-2.15E-01	1.00E+00
FBXO45	3.29E+00	2.64E-94	LOC102392047	4.04E-01	1.00E+00	FBXO5	-2.62E-01	1.00E+00
ATRNL1	-3.64E+00	2.66E-94	C9H19orf24	3.07E-01	1.00E+00	LRBA	-2.15E-01	1.00E+00
LOC112585675	7.99E+00	2.72E-94	KATNB1	1.54E-01	1.00E+00	ANLN	-2.35E-01	1.00E+00
LOC102410721	3.03E+00	2.82E-94	RPL6	-1.88E-01	1.00E+00	LOC112580279	-6.63E-01	1.00E+00
MCCC2	-3.88E+00	3.49E-94	GLG1	2.14E-01	1.00E+00	ELOVL5	-2.16E-01	1.00E+00
PPM1K	-5.82E+00	3.74E-94	PPP1R2	2.07E-01	1.00E+00	SEMA3C	-3.37E-01	1.00E+00
NREP	-4.11E+00	4.28E-94	RDH11	1.80E-01	1.00E+00	DUSP6	3.00E-01	1.00E+00
PHF10	-2.64E+00	5.11E-94	ERBB2	-9.40E-01	1.00E+00	VASP	2.22E-01	1.00E+00
LOC102401727	4.48E+00	5.11E-94	NMNAT1	-2.03E-01	1.00E+00	MANBA	2.58E-01	1.00E+00
PSPC1	-2.13E+00	5.20E-94	LCA5	3.04E-01	1.00E+00	LOC102409478	-3.61E-01	1.00E+00
DHCR24	-4.14E+00	5.22E-94	MRPL18	-1.43E-01	1.00E+00	ASAP2	-2.02E-01	1.00E+00
PPT1	-2.19E+00	5.33E-94	RTCA	-1.99E-01	1.00E+00	ZBTB14	-2.73E-01	1.00E+00
IDH1	-3.81E+00	5.76E-94	CARMIL1	1.82E-01	1.00E+00	FHOD3	2.14E-01	1.00E+00
ZP2	1.13E+01	6.14E-94	EFCAB10	-4.37E-01	1.00E+00	CLN5	2.38E-01	1.00E+00
UBR7	1.87E+00	6.23E-94	LENG8	2.33E-01	1.00E+00	NECAB3	-3.42E-01	1.00E+00
GLB1L	-5.29E+00	6.64E-94	DDX3X	1.88E-01	1.00E+00	LOC102388954	2.90E-01	1.00E+00
RBM46	8.28E+00	6.75E-94	LOC112585046	8.02E-01	1.00E+00	LOC102399533	4.39E-01	1.00E+00
VPS52	-2.23E+00	7.28E-94	REC8	-3.82E-01	1.00E+00	IP6K2	1.98E-01	1.00E+00
TMEM97	-3.60E+00	7.55E-94	CHCHD6	-2.04E-01	1.00E+00	TTC28	-2.29E-01	1.00E+00
MYO18B	4.79E+00	7.74E-94	TMEM154	6.31E-01	1.00E+00	ANTXR2	2.38E-01	1.00E+00
FAM53C	3.10E+00	9.53E-94	SLC27A5	-3.06E-01	1.00E+00	DGKA	-2.04E-01	1.00E+00
GTPBP2	2.40E+00	1.05E-93	LOC112578703	-4.16E-01	1.00E+00	SCD5	-2.50E-01	1.00E+00
EMC10	-2.50E+00	1.25E-93	LOC102412560	8.17E-01	1.00E+00	TENM1	-4.19E-01	1.00E+00
LOC112583712	-3.81E+00	1.34E-93	GFPT1	1.89E-01	1.00E+00	PSPC1	-1.82E-01	1.00E+00

LOC112578679	8.90E+00	1.51E-93	EVA1B	-5.86E-01	1.00E+00	LOC102416299	4.64E-01	1.00E+00
LOC102394697	8.92E+00	1.51E-93	TBK1	2.18E-01	1.00E+00	COL4A1	3.37E-01	1.00E+00
SMUG1	-3.13E+00	1.60E-93	TNPO1	1.64E-01	1.00E+00	LOC102390493	4.45E-01	1.00E+00
RXRA	-3.57E+00	1.60E-93	NUP188	1.77E-01	1.00E+00	TFPI2	5.67E-01	1.00E+00
AFAP1L1	5.34E+00	1.62E-93	LOC112586189	4.91E-01	1.00E+00	PCDH7	2.56E-01	1.00E+00
SETD4	-3.61E+00	1.78E-93	NRROS	-7.48E-01	1.00E+00	GNAO1	5.33E-01	1.00E+00
ZP4	1.12E+01	1.80E-93	NPC1	3.00E-01	1.00E+00	DGAT1	-2.31E-01	1.00E+00
GPR156	3.62E+00	2.24E-93	LOC102405577	6.66E-01	1.00E+00	ZBTB37	-4.46E-01	1.00E+00
LOC102399179	-3.28E+00	2.43E-93	MTA3	2.58E-01	1.00E+00	LOC112578057	5.76E-01	1.00E+00
LOC112580468	4.96E+00	2.67E-93	EPS15	1.68E-01	1.00E+00	ITPKA	-2.91E-01	1.00E+00
TXNRD2	-3.61E+00	2.84E-93	CHST12	1.64E-01	1.00E+00	SOX6	6.12E-01	1.00E+00
CCDC32	2.20E+00	2.99E-93	IL1RN	-9.09E-01	1.00E+00	SVEP1	5.77E-01	1.00E+00
RNASEK	-2.92E+00	3.03E-93	CDK8	1.47E-01	1.00E+00	AHI1	2.44E-01	1.00E+00
CLCN7	-2.46E+00	3.18E-93	JMJD8	2.66E-01	1.00E+00	ZNF76	-2.16E-01	1.00E+00
IGFBPL1	5.89E+00	3.24E-93	TNFSF13	-5.57E-01	1.00E+00	LOC102396893	4.53E-01	1.00E+00
GSDMC	7.99E+00	3.45E-93	ARHGAP25	1.37E-01	1.00E+00	IGSF5	5.78E-01	1.00E+00
RAB37	6.17E+00	3.52E-93	NOS2	-5.89E-01	1.00E+00	MSRB3	2.42E-01	1.00E+00
PARP4	-3.51E+00	3.52E-93	PAQR7	1.63E-01	1.00E+00	ITGBL1	-3.37E-01	1.00E+00
MDC1	-2.81E+00	3.55E-93	TBC1D19	-4.52E-01	1.00E+00	PFKM	-2.43E-01	1.00E+00
SEPT5	-2.43E+00	3.74E-93	ARMC10	-2.01E-01	1.00E+00	RIMKLA	-2.28E-01	1.00E+00
NR2F1	-3.66E+00	3.87E-93	KBTBD11	2.79E-01	1.00E+00	GRTP1	4.80E-01	1.00E+00
STARD3	-3.03E+00	4.00E-93	ST6GALNAC6	2.76E-01	1.00E+00	TSC22D4	2.03E-01	1.00E+00
FAM25A	7.47E+00	4.72E-93	CLTB	2.20E-01	1.00E+00	CHL1	5.48E-01	1.00E+00
ECH1	-3.37E+00	5.22E-93	GRIPAP1	1.57E-01	1.00E+00	CRY2	-2.16E-01	1.00E+00
SOX15	3.40E+00	5.84E-93	WNT5A	-5.62E-01	1.00E+00	CSRP1	2.39E-01	1.00E+00
FOXC2	8.85E+00	6.57E-93	SNX5	-1.89E-01	1.00E+00	MAST2	1.86E-01	1.00E+00
LOC112583970	6.66E+00	6.77E-93	CHSY3	4.37E-01	1.00E+00	GINS2	-2.55E-01	1.00E+00
ARHGEF11	-3.54E+00	6.84E-93	TUBB2A	1.58E-01	1.00E+00	RNH1	2.37E-01	1.00E+00
S1PR2	-3.71E+00	7.05E-93	EXOC4	1.39E-01	1.00E+00	GBF1	-2.87E-01	1.00E+00
DBN1	-2.30E+00	7.06E-93	FUOM	-5.39E-01	1.00E+00	THAP12	-4.78E-01	1.00E+00
CLSTN1	-2.62E+00	7.27E-93	SCN5A	1.45E-01	1.00E+00	DDX31	-1.94E-01	1.00E+00
PPP1R16A	-3.40E+00	7.60E-93	LOC102415329	-5.72E-01	1.00E+00	BBS9	2.51E-01	1.00E+00
MBIP	-3.65E+00	7.70E-93	CISD1	-2.53E-01	1.00E+00	LOC102399541	5.45E-01	1.00E+00
HOXB7	6.43E+00	9.01E-93	ZNF605	3.59E-01	1.00E+00	MVP	2.15E-01	1.00E+00

PCNX3	-2.46E+00	9.75E-93	FBXO45	2.11E-01	1.00E+00	OCLN	-3.46E-01	1.00E+00
GOLM1	-1.96E+00	9.77E-93	CAMTA2	1.55E-01	1.00E+00	SFPQ	-2.62E-01	1.00E+00
LOC112583730	6.43E+00	9.97E-93	CLK2	1.35E-01	1.00E+00	TENT4B	-2.72E-01	1.00E+00
GSTA4	-5.61E+00	1.05E-92	SHPRH	3.36E-01	1.00E+00	TFRC	-2.20E-01	1.00E+00
NUP210L	-3.64E+00	1.06E-92	KIAA0391	1.73E-01	1.00E+00	ATP11C	-2.07E-01	1.00E+00
GAMT	-5.38E+00	1.06E-92	TGOLN2	1.41E-01	1.00E+00	NYAP1	2.24E-01	1.00E+00
NADK2	2.61E+00	1.30E-92	CCDC171	-2.12E-01	1.00E+00	SFRP4	4.64E-01	1.00E+00
B9D1	-3.98E+00	1.59E-92	ELP6	3.53E-01	1.00E+00	BMPR2	2.24E-01	1.00E+00
LOC112586908	7.66E+00	1.70E-92	NCBP1	1.42E-01	1.00E+00	ANGPTL4	3.38E-01	1.00E+00
LOC102393424	2.30E+00	1.91E-92	LOC102404549	-2.11E-01	1.00E+00	RAPGEFL1	3.62E-01	1.00E+00
SNX33	-5.68E+00	2.38E-92	SRL	1.09E+00	1.00E+00	FIGNL2	3.92E-01	1.00E+00
MDK	-6.59E+00	2.53E-92	DEPTOR	-2.18E-01	1.00E+00	MAPRE1	-2.66E-01	1.00E+00
DPP4	-2.82E+00	2.62E-92	LOC102410270	-3.88E-01	1.00E+00	ADSS	2.61E-01	1.00E+00
FZD1	-4.57E+00	3.35E-92	BMI1	4.99E-01	1.00E+00	SH2B2	4.20E-01	1.00E+00
PPP4R4	3.79E+00	3.57E-92	SLC24A3	-4.55E-01	1.00E+00	HNRNPAB	-2.28E-01	1.00E+00
CNTNAP2	3.36E+00	3.71E-92	SCG3	1.92E-01	1.00E+00	ZNF395	1.96E-01	1.00E+00
DOCK1	-2.51E+00	4.41E-92	SIRT3	-1.76E-01	1.00E+00	TSPOAP1	4.67E-01	1.00E+00
HOXA9	8.42E+00	4.48E-92	EDEM2	1.79E-01	1.00E+00	PLEKHH1	-3.46E-01	1.00E+00
GIPC2	5.32E+00	4.63E-92	TCTN1	1.32E-01	1.00E+00	MKI67	-2.57E-01	1.00E+00
LOC112578820	7.17E+00	4.76E-92	LOC112580688	-6.79E-01	1.00E+00	LOC102405950	-3.90E-01	1.00E+00
NAA30	2.35E+00	4.79E-92	MBLAC2	-5.47E-01	1.00E+00	GTF2IRD1	2.18E-01	1.00E+00
LOC102411804	1.98E+00	4.86E-92	STK17A	-1.60E-01	1.00E+00	BEND4	-2.98E-01	1.00E+00
MATR3	-2.28E+00	4.91E-92	NAGA	2.30E-01	1.00E+00	PDIA3	-3.11E-01	1.00E+00
ZNF330	2.06E+00	5.02E-92	LZIC	-1.44E-01	1.00E+00	UPF3B	-1.91E-01	1.00E+00
RPL22L1	-3.06E+00	5.46E-92	ZP4	2.04E-01	1.00E+00	IRF3	2.16E-01	1.00E+00
LOC102402866	5.65E+00	5.65E-92	CEP63	-1.78E-01	1.00E+00	ITPRIPL2	2.62E-01	1.00E+00
CCT7	-2.88E+00	5.75E-92	EFR3A	1.96E-01	1.00E+00	N6AMT1	4.17E-01	1.00E+00
FAM98B	3.24E+00	5.90E-92	IGFALS	2.52E-01	1.00E+00	ago-03	-3.35E-01	1.00E+00
TRIM71	6.00E+00	6.11E-92	SNRK	1.69E-01	1.00E+00	LOC112583816	4.63E-01	1.00E+00
PPP1R13L	2.41E+00	6.12E-92	PKD2L1	-7.83E-01	1.00E+00	LOC102403810	4.51E-01	1.00E+00
ARHGAP6	-5.54E+00	8.73E-92	TTN	-1.87E-01	1.00E+00	HCN4	4.59E-01	1.00E+00
B3GAT3	-3.56E+00	8.95E-92	LOC102395098	7.32E-01	1.00E+00	NPAS2	-2.59E-01	1.00E+00
ANAPC5	-3.50E+00	9.07E-92	LRRC8A	1.57E-01	1.00E+00	EEF2K	-1.89E-01	1.00E+00

LOC112582229	7.75E+00	9.53E-92	NLRP8	2.04E-01	1.00E+00	FAT4	5.46E-01	1.00E+00
HS6ST1	-4.01E+00	1.02E-91	MAP3K7	2.44E-01	1.00E+00	RNF225	-4.25E-01	1.00E+00
RAD50	2.30E+00	1.14E-91	STMN1	-1.99E-01	1.00E+00	LNX2	-2.34E-01	1.00E+00
PMS1	2.06E+00	1.17E-91	MANEAL	-6.17E-01	1.00E+00	SLC25A25	-2.76E-01	1.00E+00
LOC102411901	-4.10E+00	1.30E-91	TAOK3	2.20E-01	1.00E+00	POU6F1	4.80E-01	1.00E+00
ZNF787	-3.80E+00	1.35E-91	LOC102396855	-5.46E-01	1.00E+00	LOC102406142	-2.98E-01	1.00E+00
XKR8	3.40E+00	1.35E-91	RNF128	-4.28E-01	1.00E+00	LTF	-3.37E-01	1.00E+00
GANAB	-2.93E+00	1.54E-91	LOC112578762	-8.38E-01	1.00E+00	GRAMD2A	4.34E-01	1.00E+00
ARHGAP31	-4.07E+00	1.60E-91	SPRED3	-4.78E-01	1.00E+00	LGALS3	3.47E-01	1.00E+00
MVD	-3.74E+00	1.65E-91	TAX1BP1	1.71E-01	1.00E+00	ALK	6.48E-01	1.00E+00
TPK1	2.12E+00	1.77E-91	COPS7B	1.37E-01	1.00E+00	WDYHV1	-1.89E-01	1.00E+00
LOC102404003	2.10E+00	1.90E-91	NCAM1	-5.69E-01	1.00E+00	ZMIZ1	2.78E-01	1.00E+00
RAB10	2.25E+00	1.97E-91	HGS	1.46E-01	1.00E+00	PLA2G1B	-4.02E-01	1.00E+00
ELOVL3	5.52E+00	2.01E-91	LOC102394666	-3.08E-01	1.00E+00	GNB4	3.87E-01	1.00E+00
CABIN1	-2.48E+00	2.16E-91	SNAPC5	-4.65E-01	1.00E+00	ERH	-2.15E-01	1.00E+00
FGF20	7.16E+00	2.28E-91	LOC112579999	-6.07E-01	1.00E+00	SNAP25	-4.46E-01	1.00E+00
PIK3CD	3.91E+00	2.31E-91	ZBTB20	-3.26E-01	1.00E+00	LOC102414166	6.57E-01	1.00E+00
KIF2C	2.91E+00	2.34E-91	HNRNPUL1	2.58E-01	1.00E+00	ACAD9	-2.44E-01	1.00E+00
DEDD	1.77E+00	3.27E-91	HADH	1.32E-01	1.00E+00	AMACR	-2.33E-01	1.00E+00
TCAF2	-6.96E+00	3.40E-91	RPL35	-1.78E-01	1.00E+00	NLN	2.25E-01	1.00E+00
LOC102414399	-5.79E+00	3.55E-91	LOC102412527	-3.40E-01	1.00E+00	LOC102410341	-5.03E-01	1.00E+00
ZBTB7B	-4.52E+00	4.40E-91	PRSS23	3.72E-01	1.00E+00	GPR39	6.04E-01	1.00E+00
EPHA1	4.38E+00	4.83E-91	FAM122B	1.69E-01	1.00E+00	FAM83H	2.39E-01	1.00E+00
NIPAL3	-3.10E+00	5.44E-91	UBASH3B	1.49E-01	1.00E+00	SERINC4	-2.65E-01	1.00E+00
BLOC1S6	-3.56E+00	5.60E-91	KMT5A	3.86E-01	1.00E+00	MAPK8	-1.96E-01	1.00E+00
NETO2	2.79E+00	5.94E-91	PLLP	-9.46E-01	1.00E+00	ATG9B	-2.91E-01	1.00E+00
STAG2	-3.14E+00	6.08E-91	SLC38A10	2.03E-01	1.00E+00	PDIA5	-2.49E-01	1.00E+00
TENT2	1.93E+00	6.96E-91	SRP19	-1.71E-01	1.00E+00	ITGA1	2.86E-01	1.00E+00
LOC112582017	7.58E+00	6.96E-91	FLRT2	7.30E-01	1.00E+00	FKBP9	-2.72E-01	1.00E+00
WBP1	-4.72E+00	8.25E-91	RPS6KA6	2.49E-01	1.00E+00	PHTF1	2.24E-01	1.00E+00
LOC112581581	7.62E+00	9.80E-91	LOC112579839	2.66E-01	1.00E+00	MYH15	4.94E-01	1.00E+00
ERBB2	-6.89E+00	1.04E-90	MDM2	1.46E-01	1.00E+00	PLIN4	-4.11E-01	1.00E+00
C7	7.86E+00	1.11E-90	LOC112582131	-2.53E-01	1.00E+00	NFIB	6.10E-01	1.00E+00

GIGYF1	-2.65E+00	1.13E-90	GTF3C3	-2.07E-01	1.00E+00	DUSP7	2.50E-01	1.00E+00
RPLP1	-4.12E+00	1.14E-90	LOC112586485	-9.94E-01	1.00E+00	LOC102406115	2.19E-01	1.00E+00
LOC112587746	-3.54E+00	1.30E-90	MFSD12	-5.04E-01	1.00E+00	NUDT15	-2.35E-01	1.00E+00
CCAR2	-2.10E+00	1.31E-90	DPP4	2.14E-01	1.00E+00	ZNF414	-2.32E-01	1.00E+00
RPS12	-3.97E+00	1.32E-90	C4H12orf45	-5.25E-01	1.00E+00	NOX1	4.55E-01	1.00E+00
BRCA1	2.34E+00	1.96E-90	TIFA	3.29E-01	1.00E+00	LOC112585317	-3.42E-01	1.00E+00
PTPRM	-4.13E+00	1.98E-90	DLGAP3	-3.20E-01	1.00E+00	DNAJB11	-2.72E-01	1.00E+00
SP3	-3.48E+00	1.98E-90	TRIM6	1.39E-01	1.00E+00	SH3RF3	5.27E-01	1.00E+00
SLC3A1	-2.99E+00	2.37E-90	LOC102403265	-3.09E-01	1.00E+00	A3GALT2	3.69E-01	1.00E+00
MAP3K13	3.02E+00	2.38E-90	ALOX5AP	2.99E-01	1.00E+00	NAT14	2.49E-01	1.00E+00
ZFAND3	1.82E+00	2.88E-90	RYR2	2.03E-01	1.00E+00	CHCHD2	-2.82E-01	1.00E+00
GLP1R	6.80E+00	3.07E-90	TRIM33	1.58E-01	1.00E+00	PCSK6	3.10E-01	1.00E+00
PDE3B	4.73E+00	3.07E-90	CCNC	2.68E-01	1.00E+00	MMP23B	3.15E-01	1.00E+00
RNFT1	-4.26E+00	3.27E-90	MAD2L1BP	1.38E-01	1.00E+00	ESYT1	2.26E-01	1.00E+00
CGNL1	4.09E+00	3.29E-90	UTP6	1.77E-01	1.00E+00	AKAP17A	-1.97E-01	1.00E+00
BANP	2.52E+00	3.53E-90	DYNC1I2	1.63E-01	1.00E+00	ZDHHC1	-2.84E-01	1.00E+00
RPS3	-4.59E+00	3.98E-90	RECQL4	1.99E-01	1.00E+00	CAMTA2	-1.94E-01	1.00E+00
LRRK1	-2.53E+00	4.03E-90	VANGL2	5.35E-01	1.00E+00	LOC102401766	2.38E-01	1.00E+00
GREB1L	-3.67E+00	4.53E-90	FGD5	1.71E-01	1.00E+00	LENG8	-2.69E-01	1.00E+00
ATXN7L1	2.15E+00	4.63E-90	KIF27	-4.13E-01	1.00E+00	BBS2	-1.87E-01	1.00E+00
ACTB	-4.49E+00	5.03E-90	LOC102413559	-9.78E-01	1.00E+00	TFEC	7.02E-01	1.00E+00
ZNF526	2.51E+00	5.30E-90	ARL14EPL	1.92E-01	1.00E+00	PAQR7	4.45E-01	1.00E+00
AEBP1	-7.30E+00	5.37E-90	TLN1	-1.92E-01	1.00E+00	NUDT7	2.99E-01	1.00E+00
MTMR2	2.31E+00	7.06E-90	LOC102399486	-2.52E-01	1.00E+00	HMGA1	3.49E-01	1.00E+00
KCNA7	7.85E+00	7.48E-90	MDH1B	-9.92E-01	1.00E+00	XKR6	6.09E-01	1.00E+00
TYMS	2.74E+00	9.85E-90	SLC12A6	2.15E-01	1.00E+00	SLC25A15	2.07E-01	1.00E+00
TLCD1	3.71E+00	1.02E-89	PLEKHA7	1.54E-01	1.00E+00	DCAF4	-1.93E-01	1.00E+00
SLC45A2	3.64E+00	1.07E-89	TATDN3	3.91E-01	1.00E+00	LOC112581854	-4.58E-01	1.00E+00
LSS	-2.60E+00	1.09E-89	CCL25	1.92E-01	1.00E+00	KCNC2	4.95E-01	1.00E+00
CACNA1H	-4.27E+00	1.13E-89	MZB1	-4.62E-01	1.00E+00	FST	-3.30E-01	1.00E+00
OGFRL1	-2.36E+00	1.13E-89	GLRX3	-1.72E-01	1.00E+00	CDYL2	2.30E-01	1.00E+00
STK3	-2.04E+00	1.17E-89	SCAMP5	-2.04E-01	1.00E+00	SLC35A4	2.15E-01	1.00E+00
HRG	9.52E+00	1.27E-89	LOC102416336	-5.54E-01	1.00E+00	TRIM65	-2.67E-01	1.00E+00
FAM219A	2.24E+00	1.31E-89	ZFP91	1.46E-01	1.00E+00	S1PR5	-4.48E-01	1.00E+00

SVIP	5.96E+00	1.37E-89	EXOSC7	-2.22E-01	1.00E+00	GLRX5	-3.04E-01	1.00E+00
LOC102412900	5.75E+00	1.48E-89	TRIM38	-3.57E-01	1.00E+00	RNF152	4.49E-01	1.00E+00
UBE2J1	-3.72E+00	1.53E-89	IL17B	3.61E-01	1.00E+00	RNF149	-2.26E-01	1.00E+00
FNBP4	-3.00E+00	1.70E-89	LOC102391441	-1.94E-01	1.00E+00	ADAMTS2	4.30E-01	1.00E+00
AKAP1	2.19E+00	1.94E-89	B4GALT3	1.66E-01	1.00E+00	TMEM25	-2.49E-01	1.00E+00
FAM160B2	-2.20E+00	1.98E-89	DMRTC2	-3.15E-01	1.00E+00	DDX59	-2.11E-01	1.00E+00
MESP1	9.04E+00	2.05E-89	LOC112587869	-5.13E-01	1.00E+00	RASL11A	3.73E-01	1.00E+00
DESI2	-3.42E+00	2.12E-89	GPR160	-2.33E-01	1.00E+00	KLHL15	-2.37E-01	1.00E+00
SOX1	9.91E+00	2.22E-89	GALNT12	-3.44E-01	1.00E+00	LSR	-1.91E-01	1.00E+00
MTFR1	2.44E+00	2.35E-89	TUBGCP3	1.66E-01	1.00E+00	FAS	2.47E-01	1.00E+00
ZBTB7A	-3.17E+00	2.50E-89	FBRSL1	1.70E-01	1.00E+00	AHNAK2	4.31E-01	1.00E+00
ENHO	4.79E+00	2.53E-89	FCHO1	-2.18E-01	1.00E+00	CLASRP	-2.00E-01	1.00E+00
PHACTR3	9.22E+00	2.83E-89	KLC3	-2.77E-01	1.00E+00	IL6ST	2.69E-01	1.00E+00
CCDC85C	-3.13E+00	2.90E-89	MARCH7	1.82E-01	1.00E+00	ARMH1	3.52E-01	1.00E+00
RECK	-3.70E+00	2.92E-89	PARP6	-3.46E-01	1.00E+00	TG	4.89E-01	1.00E+00
RARB	7.02E+00	2.94E-89	INTS4	1.53E-01	1.00E+00	FAM241A	-3.43E-01	1.00E+00
PIP5K1A	-4.20E+00	3.04E-89	PIGH	-6.94E-01	1.00E+00	CCDC60	-4.72E-01	1.00E+00
GNPTAB	-3.55E+00	3.05E-89	C4H12orf60	-3.97E-01	1.00E+00	POMP	2.21E-01	1.00E+00
TRIP4	1.83E+00	3.40E-89	SPRTN	2.30E-01	1.00E+00	GLIPR1	5.78E-01	1.00E+00
LOC112585592	7.70E+00	3.72E-89	PAX9	1.65E-01	1.00E+00	LOC102409669	-5.17E-01	1.00E+00
RFX6	4.98E+00	3.74E-89	GRID1	-3.69E-01	1.00E+00	OAT	-2.08E-01	1.00E+00
CLSPN	2.84E+00	3.78E-89	BOC	2.32E-01	1.00E+00	CASP3	2.08E-01	1.00E+00
SLC47A1	2.36E+00	3.90E-89	FNIP2	2.33E-01	1.00E+00	MTR	-3.35E-01	1.00E+00
NAV1	-3.09E+00	4.02E-89	NOTCH1	6.44E-01	1.00E+00	DIP2A	-2.42E-01	1.00E+00
HMCES	2.14E+00	4.52E-89	MRPL49	1.64E-01	1.00E+00	CARD6	5.93E-01	1.00E+00
EFHC1	2.31E+00	4.65E-89	CENPH	5.14E-01	1.00E+00	ZNF805	-2.31E-01	1.00E+00
IPO7	-2.25E+00	5.67E-89	SP140	-9.47E-01	1.00E+00	RBM17	-1.96E-01	1.00E+00
IQCE	-5.65E+00	6.12E-89	TRMU	-2.00E-01	1.00E+00	AMT	-2.23E-01	1.00E+00
ZMYM5	2.23E+00	6.38E-89	TSPAN6	-4.62E-01	1.00E+00	TRA2B	-2.23E-01	1.00E+00
SMIM3	-3.90E+00	6.65E-89	RASD2	1.70E-01	1.00E+00	PCNA	-2.16E-01	1.00E+00
PGAP2	-2.67E+00	6.68E-89	FAM19A4	-3.62E-01	1.00E+00	KCTD10	2.41E-01	1.00E+00
ZFPM1	-3.62E+00	6.80E-89	TMEM266	2.71E-01	1.00E+00	BRIX1	-2.62E-01	1.00E+00
ANAPC10	2.43E+00	7.23E-89	MEX3C	2.96E-01	1.00E+00	SLC2A9	7.05E-01	1.00E+00
CASC4	-3.51E+00	7.34E-89	ROS1	-1.43E-01	1.00E+00	CENPE	-2.30E-01	1.00E+00

CENPW	2.94E+00	7.52E-89	BRD1	1.92E-01	1.00E+00	ZDHHC4	1.94E-01	1.00E+00
MAP1A	-3.29E+00	7.58E-89	MAP7D2	-2.00E-01	1.00E+00	LOC102391278	2.93E-01	1.00E+00
FBLN5	2.78E+00	7.70E-89	LOC102396276	2.10E-01	1.00E+00	RNF150	3.05E-01	1.00E+00
LOC102388956	-3.77E+00	8.36E-89	AFAP1L1	-3.12E-01	1.00E+00	S100A10	2.92E-01	1.00E+00
ETHE1	-3.40E+00	9.32E-89	KLHL17	9.76E-01	1.00E+00	GATA3	-4.71E-01	1.00E+00
NARF	1.85E+00	9.46E-89	CDH4	-2.53E-01	1.00E+00	HOXD9	4.00E-01	1.00E+00
FAR1	-2.76E+00	9.58E-89	ZNF623	2.68E-01	1.00E+00	EIF5	-2.36E-01	1.00E+00
ADAD1	8.66E+00	9.64E-89	TUBGCP4	1.36E-01	1.00E+00	RCAN2	5.20E-01	1.00E+00
KCTD11	-6.53E+00	1.26E-88	ZCCHC12	-2.72E-01	1.00E+00	ETS2	3.98E-01	1.00E+00
LOC102401342	7.50E+00	1.61E-88	CNTRL	2.41E-01	1.00E+00	FRAT1	3.64E-01	1.00E+00
CEP68	1.69E+00	1.61E-88	SIK2	3.57E-01	1.00E+00	LOC112579981	4.73E-01	1.00E+00
SHTN1	3.03E+00	1.76E-88	GDF3	1.46E-01	1.00E+00	DNAH10	5.54E-01	1.00E+00
LOC102395160	-5.19E+00	1.80E-88	LOC112587794	8.23E-01	1.00E+00	WDHD1	-2.00E-01	1.00E+00
FKBP10	-6.47E+00	1.81E-88	ARL6IP6	-6.57E-01	1.00E+00	GEMIN7	2.56E-01	1.00E+00
CXCL16	-5.31E+00	2.07E-88	LOC102413852	-3.69E-01	1.00E+00	LOC112580673	5.94E-01	1.00E+00
EDARADD	7.54E+00	2.13E-88	SAMD14	-2.65E-01	1.00E+00	PTMA	-3.24E-01	1.00E+00
MFSD6	3.83E+00	2.23E-88	CTXN1	-4.80E-01	1.00E+00	CCNF	-2.41E-01	1.00E+00
TMED1	-4.38E+00	2.32E-88	DEF8	1.84E-01	1.00E+00	MTIF2	-2.21E-01	1.00E+00
ENOX1	3.44E+00	2.37E-88	YWHAG	1.37E-01	1.00E+00	UHRF1	-2.14E-01	1.00E+00
C16H11orf52	7.64E+00	2.39E-88	LOC102409101	1.83E-01	1.00E+00	MRC1	8.43E-01	1.00E+00
MAP1S	2.35E+00	2.42E-88	BCL9L	2.99E-01	1.00E+00	LOC112586123	-2.41E-01	1.00E+00
FBXL17	-2.61E+00	2.43E-88	POLDIP2	1.23E-01	1.00E+00	TNN	3.60E-01	1.00E+00
SFXN4	3.31E+00	2.58E-88	MTMR2	1.33E-01	1.00E+00	MTHFD1L	1.91E-01	1.00E+00
SPTBN1	-3.28E+00	2.66E-88	TGFB1	-2.91E-01	1.00E+00	LCT	3.52E-01	1.00E+00
CEP164	2.25E+00	2.87E-88	EPHA1	2.85E-01	1.00E+00	PLSCR5	2.80E-01	1.00E+00
GYG2	-5.05E+00	3.08E-88	NDUFA6	-1.71E-01	1.00E+00	SFRP1	3.71E-01	1.00E+00
PGS1	2.09E+00	3.28E-88	PROSER3	3.09E-01	1.00E+00	RHOC	2.30E-01	1.00E+00
CPEB3	3.92E+00	3.36E-88	EEF1G	-1.53E-01	1.00E+00	CAPN5	2.36E-01	1.00E+00
JAZF1	2.52E+00	3.58E-88	DPH2	1.70E-01	1.00E+00	SRSF3	-2.39E-01	1.00E+00
IFRD2	-3.78E+00	3.59E-88	MED19	-1.94E-01	1.00E+00	TPPP2	-3.36E-01	1.00E+00
LOC102410939	-2.33E+00	4.12E-88	EXTL3	1.58E-01	1.00E+00	ETNK1	-2.53E-01	1.00E+00
PKD2	-4.68E+00	4.76E-88	CELF2	1.43E-01	1.00E+00	EIF3J	-2.28E-01	1.00E+00
JAML	5.76E+00	5.00E-88	YIF1B	-2.03E-01	1.00E+00	C1QTNF2	5.33E-01	1.00E+00
C5H1orf21	-4.85E+00	5.07E-88	LOC102402689	-1.52E-01	1.00E+00	MYOM1	4.56E-01	1.00E+00
PIGX	2.16E+00	5.47E-88	RPE	1.73E-01	1.00E+00	ANKH	3.07E-01	1.00E+00
ZFP69B	3.48E+00	5.79E-88	SCRN3	-4.62E-01	1.00E+00	SPPL2A	-2.46E-01	1.00E+00

PLCXD1	-6.09E+00	6.05E-88	UBE2Q2	1.32E-01	1.00E+00	SUCNR1	5.96E-01	1.00E+00
MAST4	5.79E+00	6.25E-88	NCOA5	2.47E-01	1.00E+00	LOC102392428	-6.33E-01	1.00E+00
ULK4	2.08E+00	7.29E-88	FAM84B	5.24E-01	1.00E+00	PRIM1	-2.41E-01	1.00E+00
BCL2L15	5.35E+00	7.62E-88	ZIM2	4.24E-01	1.00E+00	L3MBTL3	2.11E-01	1.00E+00
TSPAN4	-4.49E+00	7.70E-88	NR1H2	1.42E-01	1.00E+00	TNFRSF21	3.62E-01	1.00E+00
LOC112578085	6.33E+00	8.79E-88	SNAP23	2.31E-01	1.00E+00	CD101	-2.84E-01	1.00E+00
NPAS1	4.93E+00	8.94E-88	CEP72	1.54E-01	1.00E+00	LOC112578513	5.62E-01	1.00E+00
SACM1L	2.01E+00	9.46E-88	AP3M1	1.58E-01	1.00E+00	PHF21A	-1.88E-01	1.00E+00
POLRMT	-2.31E+00	9.49E-88	TGM7	6.41E-01	1.00E+00	B4GAT1	-2.03E-01	1.00E+00
SRSF3	-2.62E+00	9.95E-88	ATRX	2.76E-01	1.00E+00	GABPB2	-2.58E-01	1.00E+00
ADAM8	-5.78E+00	1.02E-87	SLC4A2	1.76E-01	1.00E+00	PI4KA	-2.16E-01	1.00E+00
MAML3	-3.10E+00	1.21E-87	LOC102400532	-2.10E-01	1.00E+00	MYO5B	2.66E-01	1.00E+00
LOC112582206	6.63E+00	1.30E-87	PLAGL1	3.56E-01	1.00E+00	SLC17A9	6.33E-01	1.00E+00
IPO5	-2.21E+00	1.30E-87	LOC102400809	2.85E-01	1.00E+00	PLA2G7	4.45E-01	1.00E+00
RPL22	-4.45E+00	1.32E-87	KHDC1	-1.75E-01	1.00E+00	HMGCL	2.22E-01	1.00E+00
WDR49	-5.31E+00	1.39E-87	DAB1	-1.58E-01	1.00E+00	STX11	5.12E-01	1.00E+00
HINT3	-4.07E+00	1.45E-87	SOX15	2.36E-01	1.00E+00	CDA	4.42E-01	1.00E+00
FAM3A	-4.12E+00	1.62E-87	LOC102409123	4.74E-01	1.00E+00	SEPT8	-1.86E-01	1.00E+00
TACC3	4.81E+00	1.67E-87	LOC102401726	-9.07E-01	1.00E+00	B4GALT5	1.92E-01	1.00E+00
EMC4	-2.58E+00	1.86E-87	TMEM68	-5.40E-01	1.00E+00	LOC102398353	-1.94E-01	1.00E+00
BTD	-3.01E+00	1.92E-87	RIC8B	2.08E-01	1.00E+00	BCL3	2.62E-01	1.00E+00
SLC38A10	-2.65E+00	2.10E-87	STIM2	1.46E-01	1.00E+00	LOC102398588	5.34E-01	1.00E+00
MRPL37	2.75E+00	2.21E-87	LOC102398348	2.33E-01	1.00E+00	EVL	-2.09E-01	1.00E+00
LOC102403153	2.39E+00	2.43E-87	ASTE1	-3.18E-01	1.00E+00	RAB11FIP1	2.78E-01	1.00E+00
RNF17	6.66E+00	2.90E-87	HSPA1L	-2.93E-01	1.00E+00	DLEU7	-5.18E-01	1.00E+00
NRXN3	2.40E+00	2.93E-87	CCDC120	2.44E-01	1.00E+00	ING2	-1.94E-01	1.00E+00
TCF12	-2.78E+00	3.03E-87	NOTCH2	2.41E-01	1.00E+00	LOC102393752	5.00E-01	1.00E+00
VAMP7	1.91E+00	3.34E-87	CDKAL1	1.31E-01	1.00E+00	LOC102403863	-3.04E-01	1.00E+00
RPL23	-4.65E+00	3.47E-87	TMX3	3.38E-01	1.00E+00	ZFAND1	-2.85E-01	1.00E+00
HEXIM1	-3.94E+00	3.63E-87	PNOC	-1.79E-01	1.00E+00	OVGP1	-4.30E-01	1.00E+00
ELMOD3	-3.12E+00	5.14E-87	LOC102414277	-2.22E-01	1.00E+00	NEK2	-2.80E-01	1.00E+00
RPS27	-3.89E+00	5.30E-87	GK2	-7.53E-01	1.00E+00	LOC102407213	-4.18E-01	1.00E+00
GYG1	2.81E+00	5.34E-87	FANCM	2.48E-01	1.00E+00	CBS	-2.82E-01	1.00E+00

B3GALNT2	2.02E+00	5.56E-87	MLF2	-1.49E-01	1.00E+00	SERTAD2	3.72E-01	1.00E+00
LARP6	-3.89E+00	5.71E-87	EML6	3.27E-01	1.00E+00	CENPK	-2.72E-01	1.00E+00
VTA1	1.88E+00	5.80E-87	KCNN1	3.82E-01	1.00E+00	CEP95	-2.62E-01	1.00E+00
SEBOX	8.97E+00	6.04E-87	SNX31	1.44E+00	1.00E+00	LOC112577787	4.93E-01	1.00E+00
MDM2	2.16E+00	6.16E-87	PHTF1	1.46E-01	1.00E+00	FANCI	-2.10E-01	1.00E+00
RNF38	2.36E+00	6.25E-87	TMEM250	1.34E-01	1.00E+00	SLC38A5	3.01E-01	1.00E+00
LOC102403797	8.96E+00	6.42E-87	LOC102407467	2.09E-01	1.00E+00	RAB13	-2.53E-01	1.00E+00
CCDC149	2.77E+00	6.46E-87	LOC112579106	-4.82E-01	1.00E+00	NELFA	-1.86E-01	1.00E+00
SYCP2L	6.23E+00	7.97E-87	PSTK	-3.85E-01	1.00E+00	SMIM14	2.21E-01	1.00E+00
AIFM2	-6.71E+00	8.58E-87	FIGNL2	-8.66E-01	1.00E+00	LOC112587820	2.40E-01	1.00E+00
LANCL3	4.30E+00	8.62E-87	ATP10D	2.02E-01	1.00E+00	PDZK1	3.49E-01	1.00E+00
LOC102404009	-3.30E+00	8.75E-87	LOC102400054	8.64E-01	1.00E+00	PRKACB	2.08E-01	1.00E+00
SMPD1	-4.81E+00	9.35E-87	RPL38	-2.37E-01	1.00E+00	C18H16orf87	-4.53E-01	1.00E+00
ADIPOR1	2.01E+00	1.01E-86	CCR1	-5.35E-01	1.00E+00	MELTF	-4.36E-01	1.00E+00
GATAD2A	2.23E+00	1.10E-86	KIF5C	-2.63E-01	1.00E+00	JAZF1	-1.98E-01	1.00E+00
GLIS2	-5.95E+00	1.18E-86	FEM1B	2.08E-01	1.00E+00	CTHRC1	5.41E-01	1.00E+00
TIRAP	-6.60E+00	1.25E-86	BCL6B	-1.52E-01	1.00E+00	ROR2	-2.22E-01	1.00E+00
RPAP2	2.08E+00	1.29E-86	FGD1	1.79E-01	1.00E+00	PLEKHO2	2.17E-01	1.00E+00
SMARCC2	-2.40E+00	1.42E-86	RXFP2	-5.17E-01	1.00E+00	SEC61G	-3.02E-01	1.00E+00
LOC102391278	-4.94E+00	1.45E-86	PDZD9	-6.78E-01	1.00E+00	MICAL3	1.84E-01	1.00E+00
MCUR1	1.96E+00	1.52E-86	CCDC85C	3.73E-01	1.00E+00	ABCD4	-2.03E-01	1.00E+00
NANS	-4.87E+00	1.61E-86	COL14A1	-3.94E-01	1.00E+00	MED9	2.10E-01	1.00E+00
MPP6	2.40E+00	1.72E-86	TRMT10C	-2.74E-01	1.00E+00	MKS1	-2.03E-01	1.00E+00
B3GNTL1	3.34E+00	2.02E-86	KIRREL3	-4.71E-01	1.00E+00	LOC102414126	3.92E-01	1.00E+00
TMEM201	2.01E+00	2.21E-86	PCOLCE2	-5.46E-01	1.00E+00	ZNF575	2.41E-01	1.00E+00
PSEN1	1.81E+00	2.25E-86	NEGR1	2.04E-01	1.00E+00	VAT1L	4.13E-01	1.00E+00
WDR76	2.39E+00	2.33E-86	LOC102390492	-3.13E-01	1.00E+00	LOC112587945	5.51E-01	1.00E+00
HCN3	4.35E+00	2.49E-86	TMED3	-1.87E-01	1.00E+00	ATP6V0E2	3.42E-01	1.00E+00
C6H1orf226	3.53E+00	2.53E-86	RHOBTB2	1.82E-01	1.00E+00	LOC102389719	-2.14E-01	1.00E+00
ILVBL	-2.52E+00	3.35E-86	ABHD16A	1.78E-01	1.00E+00	LOC102398431	4.05E-01	1.00E+00
ITGB1BP2	-4.62E+00	3.39E-86	NRXN3	-1.36E-01	1.00E+00	SLC6A9	-4.03E-01	1.00E+00
LOC112581611	6.06E+00	3.74E-86	LTBP3	4.75E-01	1.00E+00	LOC102409782	-3.95E-01	1.00E+00
NRXN2	6.08E+00	3.75E-86	WDR90	-2.52E-01	1.00E+00	MPV17L2	-2.45E-01	1.00E+00
COL1A1	-8.13E+00	3.76E-86	POMT2	1.64E-01	1.00E+00	CACNA1C	4.50E-01	1.00E+00

SLC16A13	3.29E+00		3.82E-86	PHF8	2.06E-01	1.00E+00	ABCA2	-2.24E-01	1.00E+00
SPAG6	5.22E+00		3.91E-86	UNC13A	-2.27E-01	1.00E+00	LRMDA	4.04E-01	1.00E+00
FOXJ3	2.76E+00		4.26E-86	MED30	-1.91E-01	1.00E+00	TGFB1I1	2.20E-01	1.00E+00
RTN4R	5.30E+00		4.45E-86	RNF44	2.12E-01	1.00E+00	KCND1	2.80E-01	1.00E+00
SETD6	-3.03E+00		4.95E-86	AGRN	4.67E-01	1.00E+00	SGMS2	3.27E-01	1.00E+00
ANKRD17	3.19E+00		5.16E-86	TBC1D7	1.42E-01	1.00E+00	LRRC10	4.13E-01	1.00E+00
RACGAP1	2.49E+00		5.39E-86	LOC102409439	-7.78E-01	1.00E+00	SQOR	3.15E-01	1.00E+00
KMT5A	-3.22E+00		5.71E-86	TEP1	-6.90E-01	1.00E+00	LOC112580239	6.57E-01	1.00E+00
DSEL	-3.79E+00		5.92E-86	RIBC1	-1.90E-01	1.00E+00	LOC102391514	-2.14E-01	1.00E+00
SPATS2	1.75E+00		5.98E-86	ARHGAP17	2.09E-01	1.00E+00	INO80D	-2.19E-01	1.00E+00
MCU	2.64E+00		6.02E-86	NCOA1	2.62E-01	1.00E+00	ICA1	1.88E-01	1.00E+00
PAXBP1	-2.97E+00		6.37E-86	WBP1	4.28E-01	1.00E+00	DSCC1	-2.01E-01	1.00E+00
CSNK1A1	-1.88E+00		6.45E-86	SH3GL2	-2.74E-01	1.00E+00	HTRA1	2.20E-01	1.00E+00
CACNA1F	6.20E+00		6.47E-86	ANKRD66	-8.36E-01	1.00E+00	CLIP4	4.55E-01	1.00E+00
ARMCX2	-5.78E+00		7.02E-86	LOC112586179	-7.21E-01	1.00E+00	ABCG8	-4.28E-01	1.00E+00
S100A10	-4.03E+00		7.17E-86	ZCRB1	-2.22E-01	1.00E+00	ACVR2B	-2.30E-01	1.00E+00
TGFBR1	3.23E+00		7.61E-86	LOC102395010	-1.18E+00	1.00E+00	NDUFB1	2.50E-01	1.00E+00
TAF1D	-4.28E+00		7.80E-86	LOC102409673	2.06E-01	1.00E+00	GNG2	4.07E-01	1.00E+00
PRC1	3.91E+00		8.17E-86	MCAM	2.61E-01	1.00E+00	KDELR2	-1.89E-01	1.00E+00
PTPRQ	9.55E+00		8.23E-86	HP1BP3	1.50E-01	1.00E+00	FAXC	3.00E-01	1.00E+00
KIAA1210	6.30E+00		9.13E-86	SYK	-5.79E-01	1.00E+00	FAM129A	2.30E-01	1.00E+00
PITPNM2	2.80E+00		9.98E-86	IFT57	-1.76E-01	1.00E+00	TFEB	3.82E-01	1.00E+00
IFIH1	-7.14E+00		1.14E-85	SGSH	2.21E-01	1.00E+00	FGF23	-4.33E-01	1.00E+00
BRINP2	7.49E+00		1.43E-85	DIXDC1	3.29E-01	1.00E+00	ERLEC1	-1.72E-01	1.00E+00
HCN4	3.89E+00		1.54E-85	LOC102396408	-5.71E-01	1.00E+00	SEPT4	-3.86E-01	1.00E+00
TREH	7.00E+00		1.61E-85	FAR2	7.83E-01	1.00E+00	SOX9	3.84E-01	1.00E+00
PLCB3	-4.65E+00		1.75E-85	RGCC	-4.34E-01	1.00E+00	TIGD5	2.01E-01	1.00E+00
CACNA1E	7.96E+00		2.11E-85	KIAA0355	2.18E-01	1.00E+00	LOC102399397	2.79E-01	1.00E+00
ERGIC3	-2.70E+00		2.18E-85	GABARAPL1	-2.41E-01	1.00E+00	PLXNC1	-3.47E-01	1.00E+00
RCN2	-2.03E+00		2.50E-85	SIPA1L2	2.49E-01	1.00E+00	POU2F2	-3.97E-01	1.00E+00
WDR37	-2.33E+00		2.69E-85	IL7R	-4.12E-01	1.00E+00	PRPF39	-1.92E-01	1.00E+00
ZNF583	1.99E+00		2.71E-85	SERINC5	1.37E-01	1.00E+00	LOC102400283	3.67E-01	1.00E+00
AGRN	-3.97E+00		3.26E-85	FAM222B	1.40E-01	1.00E+00	EXT1	2.16E-01	1.00E+00
TMEM60	2.63E+00		3.59E-85	TM6SF1	-6.26E-01	1.00E+00	LOC102393700	-4.68E-01	1.00E+00
EXO1	2.28E+00		4.29E-85	CCDC103	1.04E+00	1.00E+00	PTBP1	-2.27E-	1.00E+00

							01	
MFSD2A	3.59E+00	4.64E-85	LOC102394431	2.63E-01	1.00E+00	MET	4.54E-01	1.00E+00
BTRC	1.91E+00	4.74E-85	STPG4	-6.40E-01	1.00E+00	TPBG	2.33E-01	1.00E+00
CD58	2.29E+00	4.78E-85	MTF1	1.99E-01	1.00E+00	SLC25A42	-2.86E-01	1.00E+00
ABI2	1.75E+00	4.86E-85	LOC112579690	-3.49E-01	1.00E+00	GYG1	2.47E-01	1.00E+00
TBP	1.75E+00	4.93E-85	DDX24	1.20E-01	1.00E+00	SCNN1A	-4.42E-01	1.00E+00
MANF	-4.84E+00	4.94E-85	SNRPD3	-1.57E-01	1.00E+00	PCCB	-2.54E-01	1.00E+00
SLC25A37	-4.83E+00	5.16E-85	MROH7	4.04E-01	1.00E+00	HMGCS1	-2.20E-01	1.00E+00
NCAPG	2.38E+00	5.36E-85	WIPF2	1.77E-01	1.00E+00	PDLIM7	2.41E-01	1.00E+00
GPR12	9.97E+00	6.21E-85	SPOPL	3.04E-01	1.00E+00	MCCC1	-2.04E-01	1.00E+00
NFATC2	6.66E+00	6.40E-85	MYLIP	1.71E-01	1.00E+00	PASK	-2.09E-01	1.00E+00
GHRL	5.66E+00	6.71E-85	NEURL1	3.23E-01	1.00E+00	FRMD6	5.60E-01	1.00E+00
CAST	-3.41E+00	7.33E-85	ALDH1A2	-4.30E-01	1.00E+00	MTCL1	2.96E-01	1.00E+00
FAM81B	7.62E+00	8.92E-85	NMT2	-2.76E-01	1.00E+00	LVRN	4.44E-01	1.00E+00
LOC112578844	5.96E+00	9.17E-85	KLHL35	-7.35E-01	1.00E+00	RNF135	-1.95E-01	1.00E+00
LOC102407744	-4.78E+00	9.35E-85	LOC102415829	-1.70E-01	1.00E+00	LOC112581828	-4.70E-01	1.00E+00
ILF2	-2.22E+00	9.82E-85	FAM19A1	-2.49E-01	1.00E+00	SVIL	3.00E-01	1.00E+00
LOC112586549	4.55E+00	1.03E-84	CTC1	-1.75E-01	1.00E+00	ZPR1	1.93E-01	1.00E+00
CCDC102A	-5.10E+00	1.04E-84	GRIP1	-5.26E-01	1.00E+00	VPS13C	3.04E-01	1.00E+00
LOC102402897	-3.65E+00	1.25E-84	LOC102403487	3.13E-01	1.00E+00	SERP2	2.74E-01	1.00E+00
EPB41L2	2.04E+00	1.25E-84	ZNF783	1.27E-01	1.00E+00	FADS6	-3.93E-01	1.00E+00
ASPM	3.12E+00	1.28E-84	DMKN	5.28E-01	1.00E+00	MATN2	3.09E-01	1.00E+00
RPL36A	-4.15E+00	1.43E-84	GDAP1	-1.88E-01	1.00E+00	NXF1	-1.70E-01	1.00E+00
FGF12	-3.69E+00	1.46E-84	LOC102389936	-3.07E-01	1.00E+00	SYT11	2.45E-01	1.00E+00
HNRNPU	-3.48E+00	1.67E-84	SLC25A3	-3.34E-01	1.00E+00	LOC102391973	3.97E-01	1.00E+00
MTX2	-2.91E+00	1.84E-84	ORMDL1	4.11E-01	1.00E+00	SLC17A5	4.00E-01	1.00E+00
ABLIM3	4.88E+00	1.85E-84	PCBP2	1.89E-01	1.00E+00	SLC25A37	-1.69E-01	1.00E+00
OPA1	1.81E+00	2.10E-84	MRPS23	-2.19E-01	1.00E+00	THBS3	-2.47E-01	1.00E+00
ANK2	2.97E+00	2.49E-84	SLC44A4	1.15E+00	1.00E+00	HEXIM2	-4.70E-01	1.00E+00
DHX32	-1.96E+00	2.50E-84	GUCD1	1.80E-01	1.00E+00	ENAH	1.93E-01	1.00E+00
ISG20L2	-5.21E+00	2.59E-84	KIAA0100	1.94E-01	1.00E+00	NFKBIA	-2.65E-01	1.00E+00
PRELID3A	2.28E+00	2.73E-84	ACTL7B	-3.49E-01	1.00E+00	DENND3	3.42E-01	1.00E+00
CTSD	-3.34E+00	2.73E-84	SNX17	-2.14E-01	1.00E+00	PLPPR5	5.20E-01	1.00E+00
EIF4B	-2.36E+00	2.80E-84	CCDC121	-3.27E-01	1.00E+00	COL4A2	2.60E-01	1.00E+00

TPT1	-4.14E+00	2.99E-84	CASP8AP2	2.92E-01	1.00E+00	TIPIN	-1.88E-01	1.00E+00
LOC102398622	2.66E+00	3.04E-84	CDK5R1	2.32E-01	1.00E+00	ARHGAP23	-2.40E-01	1.00E+00
EPM2AIP1	-3.33E+00	3.23E-84	LOC102392808	-4.89E-01	1.00E+00	SETD7	2.67E-01	1.00E+00
PRPF39	-2.47E+00	3.88E-84	TMSB4X	-1.81E-01	1.00E+00	BCKDHB	-1.97E-01	1.00E+00
LOC112582279	3.24E+00	3.88E-84	UPRT	1.19E-01	1.00E+00	GORASP2	-1.79E-01	1.00E+00
TLN2	2.85E+00	3.98E-84	MFSD14B	1.48E-01	1.00E+00	GLDC	2.59E-01	1.00E+00
WWC3	-2.44E+00	4.07E-84	TRUB2	3.49E-01	1.00E+00	PARVB	6.03E-01	1.00E+00
HNRNPDL	-2.68E+00	4.48E-84	NFS1	2.50E-01	1.00E+00	KNL1	-3.28E-01	1.00E+00
TTN	3.61E+00	6.59E-84	ANKRD44	-1.76E-01	1.00E+00	ATG10	3.36E-01	1.00E+00
LOC112581449	-7.33E+00	6.74E-84	MAML3	-2.97E-01	1.00E+00	LRRK1	-1.84E-01	1.00E+00
MPP5	2.16E+00	6.74E-84	RASSF3	2.08E-01	1.00E+00	CNTNAP1	-4.30E-01	1.00E+00
LOC112580248	7.67E+00	7.25E-84	LOC112585239	6.01E-01	1.00E+00	TOPORS	-2.74E-01	1.00E+00
PIGQ	-2.43E+00	7.33E-84	LOC112581152	-5.92E-01	1.00E+00	SDR42E1	-2.40E-01	1.00E+00
NMNAT2	-2.78E+00	7.89E-84	MEGF10	2.06E-01	1.00E+00	MAP1B	2.82E-01	1.00E+00
ZYX	-3.41E+00	7.97E-84	CEP95	1.66E-01	1.00E+00	DNMT1	-1.80E-01	1.00E+00
GPSM1	-5.49E+00	8.24E-84	SH2D3C	3.39E-01	1.00E+00	TMPO	-2.12E-01	1.00E+00
DDOST	-2.41E+00	8.82E-84	LRP8	-7.50E-01	1.00E+00	ANXA10	4.99E-01	1.00E+00
IGF2BP2	6.60E+00	9.38E-84	KAT14	-2.55E-01	1.00E+00	CLN6	-1.91E-01	1.00E+00
C11H15orf41	2.02E+00	1.03E-83	COPRS	-1.83E-01	1.00E+00	SPON1	-2.89E-01	1.00E+00
PDPK1	3.00E+00	1.05E-83	DNM1L	1.74E-01	1.00E+00	HIP1R	1.99E-01	1.00E+00
CDC14A	-4.42E+00	1.16E-83	LOC102391841	5.67E-01	1.00E+00	LRCH1	2.59E-01	1.00E+00
C7H4orf3	-2.42E+00	1.38E-83	SCARA3	-1.18E+00	1.00E+00	ACVR1	2.89E-01	1.00E+00
DAXX	-2.20E+00	1.38E-83	ARC	-7.46E-01	1.00E+00	CATSPER4	-4.65E-01	1.00E+00
RPL12	-4.39E+00	1.44E-83	ZNF541	2.11E-01	1.00E+00	NRP2	3.69E-01	1.00E+00
VEGFB	-3.51E+00	1.53E-83	LEPROT	1.88E-01	1.00E+00	NR2F1	1.91E-01	1.00E+00
TRIM52	1.80E+00	1.55E-83	CD247	2.75E-01	1.00E+00	SHTN1	2.94E-01	1.00E+00
SSR2	-2.82E+00	1.59E-83	LOC102407094	-1.31E+00	1.00E+00	NEBL	-2.23E-01	1.00E+00
TRMT61B	2.90E+00	1.77E-83	MOCS1	1.75E-01	1.00E+00	CAMKK1	4.45E-01	1.00E+00
PDZRN3	3.82E+00	1.79E-83	TCF7L2	-3.44E-01	1.00E+00	HSPA8	-3.35E-01	1.00E+00
BAK1	-2.83E+00	2.07E-83	ADAMTSL2	7.36E-01	1.00E+00	DPF1	-3.91E-01	1.00E+00
SCNN1A	3.89E+00	2.44E-83	PALD1	-2.80E-01	1.00E+00	LOC102414187	-1.77E-01	1.00E+00
FHL2	-6.83E+00	2.51E-83	FSD1L	2.03E-01	1.00E+00	EPN2	2.06E-01	1.00E+00
DYRK2	-3.02E+00	2.80E-83	LOC102397289	-5.29E-01	1.00E+00	FGFR1OP2	2.39E-01	1.00E+00
TMEM59	-	2.85E-83	PDE4C	4.20E-01	1.00E+00	POMT2	-1.81E-	1.00E+00

	2.00E+00						01	
LOC102414462	-5.66E+00	2.85E-83	LOC102392458	-4.77E-01	1.00E+00	PRSS12	4.57E-01	1.00E+00
PPM1F	-3.36E+00	3.06E-83	CCNJL	-6.55E-01	1.00E+00	PTGFRN	-2.53E-01	1.00E+00
PHLDB3	-2.67E+00	3.10E-83	AKAP11	2.26E-01	1.00E+00	WLS	2.57E-01	1.00E+00
GNA14	3.21E+00	3.15E-83	MAP2K6	-4.72E-01	1.00E+00	PGBD5	4.40E-01	1.00E+00
SRRM1	-3.99E+00	3.24E-83	PPM1M	4.87E-01	1.00E+00	VGLL3	3.33E-01	1.00E+00
LOC112580806	4.38E+00	3.55E-83	NRG1	-8.17E-01	1.00E+00	USP36	-1.83E-01	1.00E+00
MRPS14	2.67E+00	3.72E-83	CHMP6	3.78E-01	1.00E+00	PITRM1	1.92E-01	1.00E+00
KBTBD7	2.79E+00	4.10E-83	CCDC141	-6.07E-01	1.00E+00	LOC102398313	-4.97E-01	1.00E+00
NRIP1	-4.32E+00	4.74E-83	LOC112587336	-1.71E-01	1.00E+00	MAOA	1.74E-01	1.00E+00
PEF1	-2.44E+00	4.79E-83	RAP1GAP	5.45E-01	1.00E+00	RBM24	4.13E-01	1.00E+00
METTL14	2.14E+00	4.97E-83	ARHGAP12	1.52E-01	1.00E+00	LOC102389548	-3.83E-01	1.00E+00
PPOX	-2.90E+00	5.23E-83	CCDC24	6.83E-01	1.00E+00	LOC102401813	-2.42E-01	1.00E+00
TOMM5	-2.83E+00	5.48E-83	DENND3	-2.08E-01	1.00E+00	SYN1	3.64E-01	1.00E+00
NUP93	2.09E+00	5.50E-83	ERRFI1	2.01E-01	1.00E+00	RAB39B	4.47E-01	1.00E+00
GCLC	-4.30E+00	5.69E-83	SLC7A9	-1.99E-01	1.00E+00	PPP4R1	1.89E-01	1.00E+00
SGCE	-2.39E+00	6.22E-83	LOC112580332	7.87E-01	1.00E+00	EGFLAM	-5.89E-01	1.00E+00
LOC102393420	7.96E+00	6.48E-83	LAT2	-4.30E-01	1.00E+00	NOL4L	-1.83E-01	1.00E+00
NRBP2	-6.40E+00	6.95E-83	GGH	3.76E-01	1.00E+00	IL1RAPL1	4.20E-01	1.00E+00
C9H19orf57	2.97E+00	6.95E-83	LOC112584434	-8.56E-01	1.00E+00	PARM1	3.68E-01	1.00E+00
LHFPL4	4.83E+00	7.08E-83	LOC102395812	-1.79E-01	1.00E+00	RBM19	1.91E-01	1.00E+00
LTO1	2.12E+00	7.09E-83	ZNF664	2.14E-01	1.00E+00	SLC25A51	1.79E-01	1.00E+00
HNRNPM	-2.51E+00	7.46E-83	ATP5F1D	-2.04E-01	1.00E+00	WWC2	-2.81E-01	1.00E+00
EIF1B	2.29E+00	7.89E-83	EIF3K	-1.50E-01	1.00E+00	CNGA2	-4.65E-01	1.00E+00
MSL2	-2.53E+00	8.08E-83	THTPA	1.91E-01	1.00E+00	SRRT	-1.83E-01	1.00E+00
IL27RA	-6.81E+00	8.29E-83	LOC102416080	-8.75E-01	1.00E+00	BCL6	2.50E-01	1.00E+00
RPL13A	-3.85E+00	8.58E-83	POC1A	1.43E-01	1.00E+00	RAB6B	-2.27E-01	1.00E+00
C3H9orf152	6.00E+00	8.61E-83	PLCL2	-2.62E-01	1.00E+00	NT5E	3.89E-01	1.00E+00
CHADL	3.04E+00	9.09E-83	BEND7	2.08E-01	1.00E+00	RUSC2	3.09E-01	1.00E+00
SEMA4C	-5.51E+00	9.15E-83	LOC102413428	-4.52E-01	1.00E+00	ZNF142	-1.84E-01	1.00E+00
LOC112579965	7.72E+00	9.47E-83	YDJC	-2.72E-01	1.00E+00	DUSP18	3.02E-01	1.00E+00
LOC102414277	-4.37E+00	9.81E-83	MFSD14A	-2.32E-01	1.00E+00	LOC102399202	4.93E-01	1.00E+00
SLC12A3	6.58E+00	1.03E-82	MRPS17	-2.14E-01	1.00E+00	RPL7	-2.88E-01	1.00E+00
COL5A1	-6.53E+00	1.15E-82	ZDHHC2	3.15E-01	1.00E+00	CDH3	2.21E-01	1.00E+00

MAP3K20	-3.45E+00	1.28E-82	PRKAB1	1.93E-01	1.00E+00	ESR1	-1.76E-01	1.00E+00
INO80B	-3.59E+00	1.28E-82	WDR53	-1.80E-01	1.00E+00	CHD6	-2.00E-01	1.00E+00
ELF1	3.20E+00	1.30E-82	SOCS2	2.48E-01	1.00E+00	RENBP	-2.46E-01	1.00E+00
BTBD2	-2.47E+00	1.33E-82	TRPV6	1.73E-01	1.00E+00	DCBLD2	4.26E-01	1.00E+00
SYTL2	6.45E+00	1.40E-82	GLOD4	-1.38E-01	1.00E+00	BCL9L	-2.20E-01	1.00E+00
WFS1	2.15E+00	1.45E-82	ZNF529	2.55E-01	1.00E+00	ALKBH6	-2.70E-01	1.00E+00
USF3	-3.83E+00	1.51E-82	KCNN2	1.89E-01	1.00E+00	STAMBPL1	-1.76E-01	1.00E+00
PLA2G15	2.25E+00	1.52E-82	WASF3	1.94E-01	1.00E+00	CMTM7	2.33E-01	1.00E+00
SLC27A3	-5.33E+00	1.56E-82	MRPS30	-2.09E-01	1.00E+00	NFS1	-1.82E-01	1.00E+00
DSG2	-3.01E+00	1.56E-82	COMMD10	-2.77E-01	1.00E+00	LOC102411899	7.13E-01	1.00E+00
WDR97	-6.31E+00	1.56E-82	PARVA	1.70E-01	1.00E+00	NINJ1	-2.00E-01	1.00E+00
WASHC4	2.63E+00	1.63E-82	LOC102408561	1.77E-01	1.00E+00	LOC102407686	-1.90E-01	1.00E+00
UTRN	-4.21E+00	1.71E-82	IER3	-6.96E-01	1.00E+00	PTPRS	-1.95E-01	1.00E+00
ASPHD1	6.81E+00	1.78E-82	TSC22D1	-2.17E-01	1.00E+00	RYR1	-4.34E-01	1.00E+00
NFKBID	-4.08E+00	1.91E-82	SRSF2	1.26E-01	1.00E+00	NYNRIN	-2.21E-01	1.00E+00
SSR4	-3.58E+00	1.95E-82	OTULIN	2.34E-01	1.00E+00	WDR20	-1.98E-01	1.00E+00
GNPTG	-2.75E+00	2.09E-82	STRN	1.80E-01	1.00E+00	LSM10	2.39E-01	1.00E+00
CDKN2C	-4.08E+00	2.39E-82	ENAH	1.31E-01	1.00E+00	LOC112585335	-4.29E-01	1.00E+00
ST3GAL1	-5.30E+00	2.42E-82	MBTD1	3.81E-01	1.00E+00	EIF2B1	2.30E-01	1.00E+00
VGF	-6.87E+00	2.59E-82	PACRG	-6.74E-01	1.00E+00	LOC102399479	4.89E-01	1.00E+00
SLC7A6	2.63E+00	3.20E-82	FSD2	-2.70E-01	1.00E+00	MST1	-2.76E-01	1.00E+00
TICAM2	-4.67E+00	3.21E-82	CHEK2	2.46E-01	1.00E+00	MYBPC3	4.39E-01	1.00E+00
ASH2L	1.87E+00	3.25E-82	TMEM246	-6.33E-01	1.00E+00	LOC102408370	4.15E-01	1.00E+00
SENP5	1.93E+00	3.37E-82	VPS72	-1.28E-01	1.00E+00	APBA1	3.94E-01	1.00E+00
MBNL1	-4.97E+00	3.42E-82	LOC102399617	-4.50E-01	1.00E+00	LOC102407809	-6.28E-01	1.00E+00
PRELID3B	-2.08E+00	3.47E-82	CPNE2	-2.09E-01	1.00E+00	NFKBIE	2.65E-01	1.00E+00
LOC112586944	7.96E+00	3.49E-82	LOC112582263	-7.52E-01	1.00E+00	SOCS2	4.41E-01	1.00E+00
LPCAT2	5.25E+00	3.51E-82	RCE1	1.78E-01	1.00E+00	TUT4	-2.32E-01	1.00E+00
CXHXorf67	5.94E+00	3.80E-82	ICA1	-2.14E-01	1.00E+00	ZNF239	2.43E-01	1.00E+00
TEX12	5.72E+00	3.96E-82	LOC102402161	3.28E-01	1.00E+00	CDCP1	1.66E-01	1.00E+00
REPIN1	-2.84E+00	3.99E-82	LOC112584475	-4.72E-01	1.00E+00	KLC2	-1.85E-01	1.00E+00
PGR	-5.16E+00	4.00E-82	LOC102407764	-1.02E+00	1.00E+00	MSN	2.47E-01	1.00E+00
RASSF1	-4.78E+00	4.01E-82	AFF1	1.92E-01	1.00E+00	PIGZ	-2.74E-01	1.00E+00

HDAC6	-2.91E+00	4.18E-82	ETV3L	2.35E-01	1.00E+00	KCNA4	4.10E-01	1.00E+00
SLC27A1	-3.92E+00	4.30E-82	TMEM56	1.80E-01	1.00E+00	LRRC49	2.20E-01	1.00E+00
C7H4orf17	4.54E+00	4.62E-82	LOC112581628	5.70E-01	1.00E+00	PCBP4	2.22E-01	1.00E+00
COMMD1	-3.32E+00	4.79E-82	LRRFIP1	4.21E-01	1.00E+00	LOC112586645	-4.48E-01	1.00E+00
CHMP2B	2.08E+00	4.99E-82	STAU2	3.58E-01	1.00E+00	PKDCC	2.05E-01	1.00E+00
RAB3A	3.82E+00	5.70E-82	NCKAP5L	1.94E-01	1.00E+00	SNX4	-3.12E-01	1.00E+00
TRPM6	4.42E+00	5.75E-82	CENPL	-1.97E-01	1.00E+00	GOSR2	-1.75E-01	1.00E+00
PSD3	-4.70E+00	5.81E-82	EML2	1.94E-01	1.00E+00	SSTR1	4.64E-01	1.00E+00
LOC112578957	-7.34E+00	5.84E-82	PARD6G	-3.11E-01	1.00E+00	LOC102413428	-5.34E-01	1.00E+00
CIDEB	-4.37E+00	5.91E-82	MAP1B	-3.15E-01	1.00E+00	TAF1D	-1.82E-01	1.00E+00
CYFIP1	-1.87E+00	6.24E-82	YTHDC1	1.55E-01	1.00E+00	TANC1	-2.62E-01	1.00E+00
RNF43	4.47E+00	6.58E-82	NFYC	1.27E-01	1.00E+00	NUP205	-1.84E-01	1.00E+00
ASTL	1.08E+01	7.61E-82	LOC112583712	3.55E-01	1.00E+00	IGSF9	5.23E-01	1.00E+00
CXHXorf58	4.14E+00	7.92E-82	SCNN1B	-5.16E-01	1.00E+00	ZBTB3	-4.21E-01	1.00E+00
UBFD1	2.73E+00	8.45E-82	ZNF451	1.94E-01	1.00E+00	MOCS1	-2.27E-01	1.00E+00
CSPG4	-8.34E+00	8.54E-82	PEX19	1.83E-01	1.00E+00	SRL	3.90E-01	1.00E+00
LOC102413429	-6.58E+00	9.10E-82	LOC102394989	-7.07E-01	1.00E+00	LOC102412583	-3.18E-01	1.00E+00
PMPCB	-2.79E+00	9.48E-82	C4H12orf57	-2.10E-01	1.00E+00	ACVR1B	2.15E-01	1.00E+00
TMEM47	1.80E+00	9.62E-82	MTSS1	1.40E-01	1.00E+00	JOSD2	2.70E-01	1.00E+00
TVP23A	5.08E+00	1.13E-81	LOC112580817	-6.79E-01	1.00E+00	SESTD1	2.04E-01	1.00E+00
LOC102398516	4.46E+00	1.20E-81	C6H1orf226	1.37E-01	1.00E+00	PIDD1	-2.54E-01	1.00E+00
DDX1	-1.84E+00	1.22E-81	LOC102398530	2.83E-01	1.00E+00	LOC102395459	4.62E-01	1.00E+00
C19H5orf34	2.37E+00	1.27E-81	TSC22D2	2.22E-01	1.00E+00	GANAB	-2.39E-01	1.00E+00
ADCK5	-3.20E+00	1.34E-81	GDF10	1.33E+00	1.00E+00	EXOC6	2.07E-01	1.00E+00
CCL1	9.69E+00	1.35E-81	ACSF3	-4.95E-01	1.00E+00	PDGFRB	3.87E-01	1.00E+00
MIDN	-4.84E+00	1.38E-81	NOL9	1.78E-01	1.00E+00	LONRF2	-2.97E-01	1.00E+00
PNP	-6.99E+00	1.46E-81	ACO2	1.27E-01	1.00E+00	DDX11	-2.00E-01	1.00E+00
TNFAIP8L3	-3.11E+00	1.48E-81	MSL1	1.39E-01	1.00E+00	CDR2	-2.17E-01	1.00E+00
LOC102409358	-2.12E+00	1.48E-81	EVPL	2.48E-01	1.00E+00	NTRK1	-6.17E-01	1.00E+00
STBD1	-2.50E+00	1.54E-81	WNK1	2.25E-01	1.00E+00	NFIA	-3.72E-01	1.00E+00
TTC13	-2.25E+00	1.56E-81	GNA13	1.49E-01	1.00E+00	USP42	-2.68E-01	1.00E+00
ACD	2.50E+00	1.57E-81	USP53	-4.91E-01	1.00E+00	PIM2	2.70E-01	1.00E+00
MYO15A	4.14E+00	1.64E-81	MPRIP	1.68E-01	1.00E+00	TMSB4X	3.25E-01	1.00E+00
INTS14	-3.65E+00	1.85E-81	SELENON	-2.25E-01	1.00E+00	SURF6	2.24E-01	1.00E+00

LOC112580606	6.77E+00	1.89E-81	MIB1	2.11E-01	1.00E+00	ZNF423	4.52E-01	1.00E+00
ELL2	-2.46E+00	1.92E-81	FAAP100	-2.47E-01	1.00E+00	CFI	4.72E-01	1.00E+00
INPP5J	3.85E+00	1.95E-81	LDLRAD3	6.68E-01	1.00E+00	LOC112586509	5.59E-01	1.00E+00
JPH1	6.09E+00	1.99E-81	ARHGAP23	5.20E-01	1.00E+00	LOC112583862	4.19E-01	1.00E+00
MAD1L1	2.45E+00	1.99E-81	ZBTB45	4.31E-01	1.00E+00	SH3GL3	-2.13E-01	1.00E+00
CDK6	-2.76E+00	2.07E-81	UBA3	1.83E-01	1.00E+00	PDE1B	4.34E-01	1.00E+00
MFSD13A	2.41E+00	2.07E-81	UGCG	2.78E-01	1.00E+00	LIMD2	2.23E-01	1.00E+00
LOC102413337	7.37E+00	2.07E-81	FBLL1	-4.06E-01	1.00E+00	LOC102415270	2.22E-01	1.00E+00
ARMH3	1.68E+00	2.12E-81	SLC15A1	1.78E-01	1.00E+00	CFAP221	4.79E-01	1.00E+00
FAM83D	2.44E+00	2.29E-81	KCNA2	-4.75E-01	1.00E+00	PDLIM5	3.89E-01	1.00E+00
ST3GAL5	-3.67E+00	2.34E-81	GRK2	3.16E-01	1.00E+00	TSC2	-1.70E-01	1.00E+00
ZBTB49	1.71E+00	2.35E-81	ASB13	-3.81E-01	1.00E+00	TNXB	4.59E-01	1.00E+00
LOC112580127	9.96E+00	2.46E-81	ADCK1	1.87E-01	1.00E+00	LOC112581805	5.94E-01	1.00E+00
TEX14	2.84E+00	2.73E-81	AMMECR1	-3.31E-01	1.00E+00	PLEKHF2	2.39E-01	1.00E+00
RAB5B	2.07E+00	2.73E-81	NR0B1	-5.06E-01	1.00E+00	KIF15	-2.22E-01	1.00E+00
SDF2	-3.23E+00	3.13E-81	COIL	2.02E-01	1.00E+00	MROH1	-1.86E-01	1.00E+00
LOC102399157	-3.93E+00	3.20E-81	CBLL1	1.55E-01	1.00E+00	LOC112580440	2.48E-01	1.00E+00
NHS	3.62E+00	3.98E-81	TDRD5	1.23E-01	1.00E+00	GPR88	-3.57E-01	1.00E+00
CUEDC2	-3.23E+00	4.01E-81	ACTR6	-1.62E-01	1.00E+00	LOC112577760	2.29E-01	1.00E+00
BOK	-8.05E+00	4.05E-81	CARM1	1.76E-01	1.00E+00	LOC102416510	6.60E-01	1.00E+00
NDN	-5.27E+00	4.17E-81	RAD51C	1.80E-01	1.00E+00	PELP1	-2.36E-01	1.00E+00
ZNF74	-4.93E+00	4.24E-81	LOC112584524	-4.86E-01	1.00E+00	IGFBP2	2.78E-01	1.00E+00
CPNE2	4.12E+00	4.89E-81	RSBN1	-4.37E-01	1.00E+00	ADAMTS5	4.87E-01	1.00E+00
LOC102410356	2.06E+00	5.29E-81	LOC112579090	-3.27E-01	1.00E+00	GAS2L3	-2.93E-01	1.00E+00
PDCL	-3.25E+00	5.35E-81	RPL23	-2.25E-01	1.00E+00	RALGDS	1.95E-01	1.00E+00
ACAD10	-3.16E+00	5.66E-81	LOC112580873	3.90E-01	1.00E+00	AMPH	4.01E-01	1.00E+00
MAP3K5	-3.48E+00	6.37E-81	DZIP3	2.20E-01	1.00E+00	UNG	-1.70E-01	1.00E+00
MALRD1	3.93E+00	6.53E-81	C1H4orf47	-3.31E-01	1.00E+00	ANP32B	-2.25E-01	1.00E+00
C12H2orf16	5.41E+00	7.75E-81	GLCCI1	-2.30E-01	1.00E+00	CHEK2	-2.07E-01	1.00E+00
HSD17B4	-2.01E+00	7.77E-81	ANKMY2	4.70E-01	1.00E+00	LOC112586532	4.22E-01	1.00E+00
CDC42BPA	2.34E+00	7.88E-81	MEP1A	-7.90E-01	1.00E+00	NUP88	-1.64E-01	1.00E+00
OTUD3	2.18E+00	7.93E-81	PER1	1.88E-01	1.00E+00	CSF1	-4.49E-01	1.00E+00
H6PD	-3.68E+00	7.95E-81	PDPN	-6.27E-01	1.00E+00	FBXL6	-2.70E-01	1.00E+00
TECTB	6.71E+00	8.28E-81	MECP2	1.85E-01	1.00E+00	DNAJC6	4.48E-01	1.00E+00
GRHPR	3.05E+00	8.53E-81	VEZF1	3.18E-01	1.00E+00	PPARGC1A	4.97E-01	1.00E+00

ZRANB3	1.74E+00	9.06E-81	FAM161A	-1.33E-01	1.00E+00	UBE2D2	1.88E-01	1.00E+00
LOC102391011	-3.19E+00	9.49E-81	FBXO46	2.02E-01	1.00E+00	LY96	3.61E-01	1.00E+00
SELENOO	-2.98E+00	9.93E-81	SLC26A10	-5.42E-01	1.00E+00	C24H16orf58	-2.01E-01	1.00E+00
GALNT18	-6.74E+00	1.15E-80	ZFYVE26	1.53E-01	1.00E+00	MARC2	-2.01E-01	1.00E+00
CHRNA5	4.78E+00	1.23E-80	NPAS2	-3.36E-01	1.00E+00	UCKL1	-1.97E-01	1.00E+00
LOC102395245	-6.29E+00	1.41E-80	TMEM256	6.40E-01	1.00E+00	LRIG3	2.62E-01	1.00E+00
RPS18	-4.28E+00	1.43E-80	MYO19	2.00E-01	1.00E+00	LOC102414721	-2.41E-01	1.00E+00
MAPK7	1.99E+00	1.47E-80	FUT4	-6.34E-01	1.00E+00	PLSCR4	2.77E-01	1.00E+00
EYA2	6.79E+00	1.60E-80	LRIG2	2.56E-01	1.00E+00	XRCC4	2.01E-01	1.00E+00
TSHZ1	-3.08E+00	1.79E-80	TSPYL5	-3.94E-01	1.00E+00	INTS5	2.03E-01	1.00E+00
TSPAN14	1.92E+00	1.88E-80	PDPK1	1.73E-01	1.00E+00	C1QTNF1	-2.46E-01	1.00E+00
KCNMA1	6.50E+00	2.10E-80	CADM2	-4.76E-01	1.00E+00	SLC22A23	2.04E-01	1.00E+00
CHURC1	1.74E+00	2.25E-80	ATAD2B	2.41E-01	1.00E+00	SS18L2	2.16E-01	1.00E+00
SMARCD3	-3.60E+00	2.49E-80	SYCE2	-2.53E-01	1.00E+00	MARCH1	3.53E-01	1.00E+00
PCED1B	-4.63E+00	2.53E-80	PLXDC1	-3.98E-01	1.00E+00	FAM171A1	2.11E-01	1.00E+00
EIF5B	2.48E+00	2.69E-80	SLC7A6	1.30E-01	1.00E+00	LOC102390085	4.19E-01	1.00E+00
GAS2L1	-3.84E+00	2.69E-80	TMEM182	6.33E-01	1.00E+00	CKAP5	-1.95E-01	1.00E+00
LACC1	3.38E+00	2.80E-80	LOC102399631	5.32E-01	1.00E+00	CASKIN1	-3.42E-01	1.00E+00
USP27X	2.23E+00	3.16E-80	ARL16	-2.65E-01	1.00E+00	TMEM187	-3.76E-01	1.00E+00
MSRA	-5.05E+00	3.30E-80	LOC102398520	-5.42E-01	1.00E+00	LOC102410549	3.15E-01	1.00E+00
LTA4H	-4.97E+00	3.47E-80	LOC102402606	1.22E-01	1.00E+00	HMOX1	-2.26E-01	1.00E+00
GRK2	-2.75E+00	3.61E-80	LOC102397907	3.49E-01	1.00E+00	LPCAT4	-1.65E-01	1.00E+00
LOC102391228	4.89E+00	3.67E-80	SIX5	3.00E-01	1.00E+00	PGM2	-3.70E-01	1.00E+00
GALK2	2.03E+00	3.72E-80	MYRF	-2.94E-01	1.00E+00	PAQR4	4.37E-01	1.00E+00
PTPRN2	-2.89E+00	3.75E-80	MXRA5	2.38E-01	1.00E+00	RILPL2	2.10E-01	1.00E+00
LHX1	7.50E+00	3.80E-80	RCAN1	4.91E-01	1.00E+00	TTF2	-1.80E-01	1.00E+00
DOC2B	5.01E+00	4.06E-80	ZNF774	5.07E-01	1.00E+00	FOS	-5.28E-01	1.00E+00
TUBGCP4	1.90E+00	4.28E-80	ZNF521	-1.91E-01	1.00E+00	TCTN2	-1.73E-01	1.00E+00
PROS1	-7.15E+00	4.54E-80	FBXO42	1.72E-01	1.00E+00	PRDM1	3.65E-01	1.00E+00
C3H17orf58	2.41E+00	4.57E-80	RBL2	1.91E-01	1.00E+00	ASPH	-1.92E-01	1.00E+00
FABP3	3.70E+00	4.63E-80	ADPRHL2	-2.99E-01	1.00E+00	VANGL1	-2.43E-01	1.00E+00
CDC42SE2	1.83E+00	5.12E-80	SLC26A4	6.47E-01	1.00E+00	CMBL	-2.18E-01	1.00E+00
CCT4	-2.06E+00	5.24E-80	SLC31A2	-3.55E-01	1.00E+00	ADCY1	-4.29E-01	1.00E+00

AGMAT	5.06E+00	5.40E-80	C5H1orf74	2.78E-01	1.00E+00	MIIP	-2.07E-01	1.00E+00
LOC102403530	5.73E+00	5.57E-80	LOC102407140	4.34E-01	1.00E+00	MMP9	7.23E-01	1.00E+00
LOC112581623	-2.74E+00	5.68E-80	MED16	1.78E-01	1.00E+00	PEX2	2.20E-01	1.00E+00
MCF2	6.58E+00	5.78E-80	CHD4	2.00E-01	1.00E+00	LOC102394061	3.59E-01	1.00E+00
IVNS1ABP	-4.98E+00	5.82E-80	RUSC2	-5.90E-01	1.00E+00	ZFP36L1	4.12E-01	1.00E+00
FNIP2	3.50E+00	7.16E-80	RPL36A	-3.24E-01	1.00E+00	ZNF81	2.75E-01	1.00E+00
AFAP1L2	3.62E+00	7.24E-80	TMEM39A	-1.26E-01	1.00E+00	IL4I1	2.38E-01	1.00E+00
SLC4A2	-1.86E+00	7.33E-80	MMAA	-1.52E-01	1.00E+00	MAD2L1	-2.22E-01	1.00E+00
PTGES2	-3.80E+00	7.42E-80	KCNQ5	-5.38E-01	1.00E+00	SPRN	-3.44E-01	1.00E+00
UBE2U	3.01E+00	7.76E-80	LOC112584667	3.75E-01	1.00E+00	AGAP2	3.48E-01	1.00E+00
TCN2	-5.40E+00	8.12E-80	ST3GAL2	-3.44E-01	1.00E+00	NDE1	-2.00E-01	1.00E+00
KRT18	-9.23E+00	8.64E-80	PLCE1	-2.19E-01	1.00E+00	WNK2	-1.93E-01	1.00E+00
PCP2	5.37E+00	9.32E-80	PAFAH1B2	1.27E-01	1.00E+00	RAPGEF1	1.93E-01	1.00E+00
DDX43	8.05E+00	9.57E-80	NUDT22	-2.03E-01	1.00E+00	PPP1R10	-1.66E-01	1.00E+00
HEXDC	-3.39E+00	9.70E-80	LOC102404203	-4.62E-01	1.00E+00	MED26	-2.21E-01	1.00E+00
MTR	-3.58E+00	9.96E-80	NMRK1	3.37E-01	1.00E+00	ITPRIP	2.89E-01	1.00E+00
FGA	9.02E+00	1.07E-79	TOMM70	1.26E-01	1.00E+00	TGM1	-4.03E-01	1.00E+00
CKS1B	3.55E+00	1.09E-79	RPL9	-4.01E-01	1.00E+00	FOXRED1	-2.01E-01	1.00E+00
FGD5	2.77E+00	1.17E-79	TMC7	-4.43E-01	1.00E+00	LOC102390086	2.32E-01	1.00E+00
SLC16A7	-6.90E+00	1.20E-79	RAVER1	1.73E-01	1.00E+00	IMPAD1	-1.69E-01	1.00E+00
SUZ12	-2.89E+00	1.22E-79	DGKZ	1.34E-01	1.00E+00	IRS1	2.30E-01	1.00E+00
SLC25A25	-3.48E+00	1.25E-79	LOC102404071	-4.53E-01	1.00E+00	TAGLN2	3.57E-01	1.00E+00
LOC102390435	-5.72E+00	1.30E-79	DNMT1	1.69E-01	1.00E+00	RAB26	-2.48E-01	1.00E+00
TPPP2	-8.14E+00	1.34E-79	FADS1	2.07E-01	1.00E+00	PLEKHD1	-2.64E-01	1.00E+00
LOC112580440	-4.46E+00	1.62E-79	JCHAIN	6.24E-01	1.00E+00	GSTA1	-4.09E-01	1.00E+00
DYNLL2	2.58E+00	1.76E-79	TAF3	1.91E-01	1.00E+00	PKP4	-1.70E-01	1.00E+00
LOC102396947	-6.78E+00	1.90E-79	FCHSD2	1.66E-01	1.00E+00	AMHR2	-2.78E-01	1.00E+00
MAGI3	-3.86E+00	2.06E-79	LOC102400376	-1.84E-01	1.00E+00	DNM3	3.34E-01	1.00E+00
ZBTB17	-1.99E+00	2.10E-79	LOC112584536	6.37E-01	1.00E+00	TUBGCP3	-1.93E-01	1.00E+00
RASSF8	2.73E+00	2.13E-79	PHLDB2	-9.43E-01	1.00E+00	HRAS	2.46E-01	1.00E+00
RPS9	-4.49E+00	2.29E-79	APBA1	-1.72E-01	1.00E+00	LOC102407231	-2.04E-01	1.00E+00
GABBR1	-5.14E+00	2.50E-79	GMPR	-1.74E-01	1.00E+00	GPAM	-2.23E-01	1.00E+00
OPLAH	-5.15E+00	2.65E-79	C17H4orf45	-6.13E-01	1.00E+00	TESC	2.60E-01	1.00E+00
ATG14	1.94E+00	2.67E-79	NFE2L3	-2.83E-	1.00E+00	SNX5	-1.65E-	1.00E+00

				01			01	
SPICE1	1.77E+00	2.77E-79	LOC102400622	4.47E-01	1.00E+00	ZNF131	-2.09E-01	1.00E+00
DCLRE1C	2.57E+00	2.81E-79	LOC102400938	2.58E-01	1.00E+00	LOC112585568	4.38E-01	1.00E+00
PIWIL2	3.37E+00	2.85E-79	ME2	-1.21E-01	1.00E+00	C6H1orf210	3.56E-01	1.00E+00
RPS11	-4.32E+00	3.07E-79	SEPT4	4.85E-01	1.00E+00	ZCCHC9	-1.79E-01	1.00E+00
EEF2	-3.40E+00	3.21E-79	LOC102409615	7.78E-01	1.00E+00	HSF2BP	-2.71E-01	1.00E+00
COLEC12	6.15E+00	3.33E-79	TXNL4B	-2.11E-01	1.00E+00	SERPINE2	-3.86E-01	1.00E+00
ATP2C2	2.93E+00	3.57E-79	RBKS	-6.22E-01	1.00E+00	IVD	-2.02E-01	1.00E+00
PLXNA3	-4.69E+00	3.60E-79	USP15	1.53E-01	1.00E+00	CCDC24	-2.96E-01	1.00E+00
ARHGEF10	-3.22E+00	3.72E-79	HTR5A	-6.38E-01	1.00E+00	PLOD1	-2.59E-01	1.00E+00
LOC102395880	-7.00E+00	3.87E-79	LOC102399590	5.59E-01	1.00E+00	CD83	-5.64E-01	1.00E+00
CREB3L2	-2.90E+00	3.95E-79	RUBCNL	-1.75E-01	1.00E+00	LOC112586833	3.75E-01	1.00E+00
FKBP3	-2.78E+00	4.17E-79	CTNS	1.42E-01	1.00E+00	SEMA3A	3.77E-01	1.00E+00
STRN	2.45E+00	4.47E-79	MAZ	-2.46E-01	1.00E+00	CABIN1	-1.84E-01	1.00E+00
PML	-3.59E+00	4.90E-79	LRRC8B	1.79E-01	1.00E+00	LOC112580467	-4.20E-01	1.00E+00
IQGAP3	3.44E+00	5.13E-79	HSP90AA1	-1.75E-01	1.00E+00	PCDH17	5.18E-01	1.00E+00
USP54	2.64E+00	5.22E-79	MYCL	-1.49E-01	1.00E+00	CCL3	-8.64E-01	1.00E+00
ELK1	2.02E+00	5.37E-79	AKAP17A	2.02E-01	1.00E+00	CDH7	3.90E-01	1.00E+00
ZMPSTE24	-3.16E+00	5.46E-79	EMB	-2.50E-01	1.00E+00	ZDHHC14	-1.97E-01	1.00E+00
SLTM	-2.26E+00	5.66E-79	CDO1	1.36E-01	1.00E+00	YIPF2	-1.82E-01	1.00E+00
NECTIN2	-7.49E+00	6.04E-79	WDR38	-6.34E-01	1.00E+00	GREB1L	-2.38E-01	1.00E+00
ADGRG1	-2.76E+00	6.56E-79	LOC102395959	-6.68E-01	1.00E+00	FAM69A	3.31E-01	1.00E+00
SHE	6.98E+00	6.69E-79	MORC3	2.38E-01	1.00E+00	NUSAP1	-2.84E-01	1.00E+00
ACSF3	-4.29E+00	6.69E-79	COL22A1	-2.08E-01	1.00E+00	RETREG1	2.93E-01	1.00E+00
LOC112585719	3.70E+00	6.76E-79	ARHGAP8	-3.49E-01	1.00E+00	COL11A1	5.09E-01	1.00E+00
ZBTB4	-2.56E+00	6.84E-79	ATP5F1B	-1.34E-01	1.00E+00	BZW1	1.94E-01	1.00E+00
ABCC5	-2.42E+00	7.06E-79	LOC102393778	-1.58E-01	1.00E+00	COL11A2	3.52E-01	1.00E+00
LOC112578831	4.66E+00	7.63E-79	NECTIN1	5.24E-01	1.00E+00	INPP5E	-2.02E-01	1.00E+00
IFITM5	-6.31E+00	7.89E-79	TLE3	-5.52E-01	1.00E+00	NIN	2.91E-01	1.00E+00
FSCB	9.28E+00	7.90E-79	TUBA4A	3.98E-01	1.00E+00	RELL2	2.73E-01	1.00E+00
SYDE1	-5.01E+00	8.61E-79	ZFP41	-3.42E-01	1.00E+00	MLST8	-2.40E-01	1.00E+00
OXNAD1	-3.30E+00	8.74E-79	GDF9	1.62E-01	1.00E+00	PKIA	2.89E-01	1.00E+00
KLHL22	-2.95E+00	9.04E-79	GNB4	-4.44E-01	1.00E+00	WTIP	-1.90E-01	1.00E+00
CLPX	2.14E+00	9.30E-79	LOC112579607	-2.41E-	1.00E+00	ZNRF1	1.92E-01	1.00E+00

				01				
AS3MT	3.59E+00	9.38E-79	CDC25B	1.34E-01	1.00E+00	LOC112579234	-3.81E-01	1.00E+00
IFT172	-2.88E+00	9.58E-79	LOC112580427	-2.38E-01	1.00E+00	CANT1	-1.78E-01	1.00E+00
SUGP1	-2.49E+00	1.01E-78	KCNK6	-4.95E-01	1.00E+00	SNX10	-2.18E-01	1.00E+00
STX10	-5.33E+00	1.10E-78	PATJ	-1.55E-01	1.00E+00	ZNF500	-2.03E-01	1.00E+00
FANCL	2.06E+00	1.15E-78	LOC102414092	7.08E-01	1.00E+00	LOC112585002	-3.68E-01	1.00E+00
PARP2	-2.40E+00	1.30E-78	LOC102389957	-4.48E-01	1.00E+00	MORN4	1.80E-01	1.00E+00
TET1	-3.63E+00	1.41E-78	TMED8	1.78E-01	1.00E+00	PIGV	-1.98E-01	1.00E+00
PRSS53	2.47E+00	1.58E-78	CDK16	1.29E-01	1.00E+00	GINM1	-1.69E-01	1.00E+00
TRNAG-GCC-12	9.71E+00	1.61E-78	GPR26	4.40E-01	1.00E+00	TNFRSF25	3.97E-01	1.00E+00
ZEB2	-4.43E+00	1.79E-78	PIGBOS1	-4.55E-01	1.00E+00	HHATL	4.34E-01	1.00E+00
DTNB	2.18E+00	1.86E-78	ZCCHC9	-1.64E-01	1.00E+00	HECA	2.29E-01	1.00E+00
C2H6orf106	1.82E+00	1.94E-78	NADK	1.27E-01	1.00E+00	TRA2A	-1.91E-01	1.00E+00
DBNL	-1.81E+00	1.95E-78	SPAG6	2.46E-01	1.00E+00	GPC3	-2.30E-01	1.00E+00
SLC17A6	7.27E+00	2.01E-78	LOC102398665	7.11E-01	1.00E+00	AMN	4.29E-01	1.00E+00
FOXRED1	-2.63E+00	2.03E-78	GADL1	2.18E-01	1.00E+00	TSPAN4	-2.17E-01	1.00E+00
N4BP2L1	-4.34E+00	2.07E-78	ANAPC13	2.34E-01	1.00E+00	LOC102398515	-4.65E-01	1.00E+00
HELQ	1.93E+00	2.16E-78	LRRIQ4	-1.83E-01	1.00E+00	DAXX	-1.76E-01	1.00E+00
TRADD	-3.64E+00	2.27E-78	GPBP1L1	1.26E-01	1.00E+00	KLHL25	2.48E-01	1.00E+00
ARSD	-5.29E+00	2.46E-78	DCTD	1.48E-01	1.00E+00	GABRB3	4.46E-01	1.00E+00
RALBP1	1.74E+00	2.46E-78	NDUFV1	1.76E-01	1.00E+00	EFNB1	2.07E-01	1.00E+00
PDIA6	-2.68E+00	2.52E-78	LOC102411280	-4.61E-01	1.00E+00	C5H1orf115	-2.33E-01	1.00E+00
BID	-3.26E+00	2.64E-78	ARHGAP28	-8.42E-01	1.00E+00	RIPOR1	-1.63E-01	1.00E+00
PLEKHG4	4.77E+00	2.68E-78	ICA1L	1.37E-01	1.00E+00	LOC102393741	7.65E-01	1.00E+00
DCLRE1A	2.07E+00	2.88E-78	FARP1	1.34E-01	1.00E+00	PRRC1	-3.04E-01	1.00E+00
ZHX3	2.36E+00	3.19E-78	BTBD2	1.49E-01	1.00E+00	SMIM3	2.15E-01	1.00E+00
PIGK	-2.84E+00	3.31E-78	SLC22A31	4.08E-01	1.00E+00	COPS3	-1.61E-01	1.00E+00
ZBTB37	4.06E+00	3.68E-78	ACBD5	3.42E-01	1.00E+00	RNF165	4.22E-01	1.00E+00
ARL15	2.74E+00	3.73E-78	LRRC73	-8.28E-01	1.00E+00	ATXN2L	-1.79E-01	1.00E+00
SWT1	-2.66E+00	4.16E-78	LOC102401543	-5.19E-01	1.00E+00	NAT16	3.73E-01	1.00E+00
CCDC8	-6.23E+00	4.27E-78	TNIP1	-1.90E-01	1.00E+00	PLPPR1	4.08E-01	1.00E+00
OS9	-2.55E+00	4.33E-78	SEC16A	1.75E-01	1.00E+00	MPHOSPH10	-1.88E-01	1.00E+00
PNRC1	-3.87E+00	4.46E-78	SHISA6	1.08E+00	1.00E+00	FAM117A	-2.52E-01	1.00E+00
NXN	2.33E+00	4.47E-78	LSM3	-1.98E-01	1.00E+00	DIDO1	-2.00E-01	1.00E+00

FDFT1	-2.13E+00	4.91E-78	C6H1orf122	2.43E-01	1.00E+00	FAM84B	3.91E-01	1.00E+00
LOC102415074	-4.72E+00	4.95E-78	FIGN	3.90E-01	1.00E+00	DDX46	-1.78E-01	1.00E+00
ITGA4	2.54E+00	4.97E-78	FANK1	-2.20E-01	1.00E+00	NDUFA4L2	3.91E-01	1.00E+00
SLC35F4	6.33E+00	5.03E-78	ORC2	1.35E-01	1.00E+00	MYO1H	3.32E-01	1.00E+00
ZBED4	2.19E+00	5.05E-78	PDCD5	-1.71E-01	1.00E+00	LOC102405260	2.88E-01	1.00E+00
TOMM70	1.96E+00	5.26E-78	QKI	2.28E-01	1.00E+00	LOC102394446	2.31E-01	1.00E+00
PSMB9	-3.65E+00	5.42E-78	LOC112586434	-6.59E-01	1.00E+00	N4BP3	3.34E-01	1.00E+00
ADAMTS10	-5.07E+00	5.84E-78	ADAM10	1.48E-01	1.00E+00	SLC35D1	-2.28E-01	1.00E+00
GPD2	-4.16E+00	6.42E-78	PARD3B	1.28E-01	1.00E+00	OAS1	3.38E-01	1.00E+00
PANK1	2.07E+00	6.64E-78	ZBTB43	2.74E-01	1.00E+00	PLAGL2	2.54E-01	1.00E+00
RANGRF	3.17E+00	6.69E-78	MIIP	-1.89E-01	1.00E+00	NPAS3	3.49E-01	1.00E+00
LOC112578863	5.14E+00	7.08E-78	LOC102401515	-3.11E-01	1.00E+00	DNAJB5	3.28E-01	1.00E+00
NOC2L	-2.59E+00	7.29E-78	FSTL1	1.76E-01	1.00E+00	KCNK13	4.39E-01	1.00E+00
RPL34	-3.88E+00	7.44E-78	INAVA	3.82E-01	1.00E+00	RHOT2	1.79E-01	1.00E+00
PAK1IP1	-2.56E+00	7.49E-78	KRR1	-2.20E-01	1.00E+00	B3GALT1	4.66E-01	1.00E+00
PPHLN1	-1.99E+00	7.76E-78	LOC102401107	7.41E-01	1.00E+00	LONRF3	2.31E-01	1.00E+00
LIMS2	-6.52E+00	8.20E-78	IFT140	-2.02E-01	1.00E+00	NPR2	-1.75E-01	1.00E+00
ZNF879	2.34E+00	8.37E-78	LOC112584648	1.70E-01	1.00E+00	VWF	-1.84E-01	1.00E+00
RUNDC1	2.39E+00	8.57E-78	WDR92	-2.12E-01	1.00E+00	HSPB7	4.03E-01	1.00E+00
KCNB2	-4.69E+00	9.00E-78	USP36	1.36E-01	1.00E+00	ARHGEF4	2.83E-01	1.00E+00
LOC102414448	-3.06E+00	9.51E-78	FKBP15	1.26E-01	1.00E+00	IFT122	-1.71E-01	1.00E+00
LOC112583621	3.53E+00	9.61E-78	LOC112586986	-1.89E-01	1.00E+00	ZBTB17	1.61E-01	1.00E+00
CPT2	-3.00E+00	9.63E-78	IGFBP5	-9.87E-01	1.00E+00	ZNF169	2.16E-01	1.00E+00
MSI2	-3.07E+00	1.04E-77	ERBB4	-2.67E-01	1.00E+00	LOC102403983	-1.62E-01	1.00E+00
EFNB1	2.20E+00	1.08E-77	TSPYL4	-2.10E-01	1.00E+00	LOC102405714	-2.65E-01	1.00E+00
MTIF3	-2.46E+00	1.10E-77	ASMTL	-3.49E-01	1.00E+00	LOC102413645	-5.63E-01	1.00E+00
GTDC1	-1.96E+00	1.14E-77	TC2N	4.58E-01	1.00E+00	GMNN	-1.99E-01	1.00E+00
SAP30L	1.77E+00	1.36E-77	ASMT	-3.09E-01	1.00E+00	TRERF1	-2.03E-01	1.00E+00
TCF7L2	-3.22E+00	1.37E-77	TMEM185B	1.53E-01	1.00E+00	LOC102406308	-2.73E-01	1.00E+00
SLC7A7	3.28E+00	1.39E-77	MEIOC	2.60E-01	1.00E+00	CCDC34	-2.30E-01	1.00E+00
FASTKD2	1.66E+00	1.52E-77	TTC7B	-3.14E-01	1.00E+00	UTRN	-2.19E-01	1.00E+00
GALNT16	-6.87E+00	1.56E-77	DOK1	-4.93E-01	1.00E+00	UBASH3A	4.32E-01	1.00E+00
LOC102410024	-2.85E+00	1.69E-77	NDUFA13	-1.76E-01	1.00E+00	LOC102395151	-4.95E-01	1.00E+00

LOC102410880	6.93E+00	1.73E-77	CKAP2	1.66E-01	1.00E+00	TDRKH	-3.19E-01	1.00E+00
RSPRY1	2.06E+00	1.94E-77	TEX49	-6.43E-01	1.00E+00	LOC102404534	3.58E-01	1.00E+00
UST	-4.95E+00	2.08E-77	ACTN1	1.29E-01	1.00E+00	PRKD1	-1.85E-01	1.00E+00
RHOD	3.41E+00	2.12E-77	ZBTB22	-2.03E-01	1.00E+00	EAF1	2.04E-01	1.00E+00
RIC3	2.83E+00	2.21E-77	LOC102412561	-6.64E-01	1.00E+00	PHGDH	-3.49E-01	1.00E+00
PLCB4	-3.90E+00	2.29E-77	LOC102408597	-2.36E-01	1.00E+00	RABGEF1	-2.17E-01	1.00E+00
GORAB	-5.07E+00	2.61E-77	ISOC2	-4.24E-01	1.00E+00	AGBL5	-1.93E-01	1.00E+00
DAAM1	2.76E+00	2.70E-77	MPP5	1.67E-01	1.00E+00	LOC102401424	-4.02E-01	1.00E+00
IRX5	2.33E+00	2.70E-77	OPA1	1.32E-01	1.00E+00	LOC112582136	3.81E-01	1.00E+00
GSN	-5.06E+00	2.71E-77	ZCCHC2	2.42E-01	1.00E+00	YBX2	-4.00E-01	1.00E+00
OTUD6B	1.68E+00	2.71E-77	RNF145	1.33E-01	1.00E+00	LOC112583714	-4.23E-01	1.00E+00
KCTD3	1.93E+00	2.80E-77	CCK	-3.32E-01	1.00E+00	ASCC2	1.86E-01	1.00E+00
MED22	1.84E+00	2.80E-77	GLS	3.17E-01	1.00E+00	ACBD3	-1.77E-01	1.00E+00
PEX5L	4.73E+00	2.86E-77	LOC112577707	-4.66E-01	1.00E+00	IRF7	-2.39E-01	1.00E+00
RBPMS	2.28E+00	3.00E-77	DPYSL2	-8.33E-01	1.00E+00	F2RL1	-3.77E-01	1.00E+00
PACSIN1	-3.79E+00	3.56E-77	DDX52	-2.24E-01	1.00E+00	NT5C2	1.68E-01	1.00E+00
MESP2	7.16E+00	3.61E-77	SDAD1	-1.80E-01	1.00E+00	LOC102409066	3.60E-01	1.00E+00
CLUAP1	1.92E+00	4.07E-77	LOC102413264	-2.45E-01	1.00E+00	GCDH	-1.75E-01	1.00E+00
STK38L	-4.76E+00	4.31E-77	LOC102403530	-3.20E-01	1.00E+00	LONP2	1.55E-01	1.00E+00
SLC22A16	-3.31E+00	4.74E-77	MAPK9	1.98E-01	1.00E+00	PLCB3	-2.18E-01	1.00E+00
STMN1	-2.77E+00	4.88E-77	INO80	2.33E-01	1.00E+00	LOC112584472	4.81E-01	1.00E+00
ZNF148	-2.53E+00	5.14E-77	CKMT1A	1.28E-01	1.00E+00	PRKD2	1.95E-01	1.00E+00
CHD9	-3.70E+00	5.32E-77	SNX3	-1.34E-01	1.00E+00	SAP18	-1.72E-01	1.00E+00
RPLP2	-4.64E+00	5.41E-77	NUP210L	-3.69E-01	1.00E+00	EREG	-6.59E-01	1.00E+00
ZNF189	-2.92E+00	5.43E-77	C3H17orf97	-4.72E-01	1.00E+00	NOTCH3	3.66E-01	1.00E+00
PTGES3	-2.28E+00	6.23E-77	MED17	1.69E-01	1.00E+00	WNK4	-3.86E-01	1.00E+00
ZDHHC18	2.01E+00	6.34E-77	TOP3A	1.65E-01	1.00E+00	LOC112582255	-4.01E-01	1.00E+00
LOC112580421	2.50E+00	7.10E-77	LOC102399668	-1.69E-01	1.00E+00	TMEM255B	-4.01E-01	1.00E+00
CALD1	-3.38E+00	7.44E-77	PRPSAP2	-1.64E-01	1.00E+00	TMEM97	-2.22E-01	1.00E+00
ART3	6.35E+00	8.29E-77	LOC102394506	-2.10E-01	1.00E+00	ARHGEF17	-1.86E-01	1.00E+00
S100A13	-7.69E+00	8.34E-77	ELFN2	-5.90E-01	1.00E+00	ODC1	-1.97E-01	1.00E+00
TAX1BP1	2.34E+00	8.37E-77	TTC19	-1.30E-01	1.00E+00	LOC102390987	-2.12E-01	1.00E+00
LOC102397907	7.22E+00	9.09E-77	RAD54L2	2.45E-01	1.00E+00	ERLIN2	-1.64E-	1.00E+00

							01	
ZP3	1.08E+01	9.23E-77	LOC102402179	8.10E-01	1.00E+00	CRYBG3	3.66E-01	1.00E+00
HOXD9	3.46E+00	1.00E-76	VWA3B	-4.77E-01	1.00E+00	ELMOD1	3.13E-01	1.00E+00
SRC	-2.89E+00	1.01E-76	LOC112585771	4.43E-01	1.00E+00	SLC25A36	-2.08E-01	1.00E+00
GPBP1	1.97E+00	1.03E-76	FAM49A	4.36E-01	1.00E+00	DAB2IP	-2.36E-01	1.00E+00
RPS24	-5.18E+00	1.04E-76	TMEM94	1.79E-01	1.00E+00	ADPRM	-2.29E-01	1.00E+00
SS18	-2.37E+00	1.06E-76	LOC112581539	-3.01E-01	1.00E+00	FANCL	-2.16E-01	1.00E+00
DNAJB6	1.76E+00	1.24E-76	LIN54	2.72E-01	1.00E+00	LIN54	-2.47E-01	1.00E+00
TMEM266	4.06E+00	1.26E-76	UPP2	-3.46E-01	1.00E+00	PCCA	-1.57E-01	1.00E+00
RPS8	-4.90E+00	1.29E-76	NEUROG2	-4.12E-01	1.00E+00	LITAF	2.52E-01	1.00E+00
ZNF146	-3.00E+00	1.37E-76	MDC1	2.54E-01	1.00E+00	C9H19orf66	3.26E-01	1.00E+00
C1QL1	6.87E+00	1.37E-76	BTBD11	-5.05E-01	1.00E+00	IL10RA	3.25E-01	1.00E+00
LOC102408196	4.59E+00	1.43E-76	SLC39A12	2.05E-01	1.00E+00	HOXA4	4.35E-01	1.00E+00
PRSS50	3.65E+00	1.44E-76	TMEM243	-2.22E-01	1.00E+00	PCYOX1L	-1.97E-01	1.00E+00
MLXIP	-2.38E+00	1.51E-76	ANKRD11	1.84E-01	1.00E+00	ACAP3	1.82E-01	1.00E+00
FAM163A	2.94E+00	1.57E-76	ADAMTS17	-2.32E-01	1.00E+00	CNTLN	-1.64E-01	1.00E+00
MGRN1	-2.09E+00	1.58E-76	RSPRY1	1.28E-01	1.00E+00	DOCK1	-1.94E-01	1.00E+00
LOC102414455	-3.67E+00	1.60E-76	MRPL19	-2.12E-01	1.00E+00	PCSK2	3.44E-01	1.00E+00
DTL	1.97E+00	1.61E-76	AARS	1.37E-01	1.00E+00	FNDC4	3.14E-01	1.00E+00
LOC102403961	6.31E+00	1.64E-76	MAGI2	1.41E-01	1.00E+00	ARG2	-2.65E-01	1.00E+00
LOC112586095	7.92E+00	1.69E-76	BOLL	-1.44E-01	1.00E+00	NAV2	2.85E-01	1.00E+00
CENPB	-4.25E+00	1.77E-76	ZC2HC1B	4.13E-01	1.00E+00	LTV1	-1.64E-01	1.00E+00
AVIL	4.57E+00	2.08E-76	RBBP4	-1.57E-01	1.00E+00	ASTN2	3.96E-01	1.00E+00
BHLHA9	8.63E+00	2.84E-76	ADAMTS6	6.89E-01	1.00E+00	AHR	3.89E-01	1.00E+00
ECE1	-2.83E+00	2.91E-76	EIF1AX	-1.29E-01	1.00E+00	MMD	-2.32E-01	1.00E+00
ANKS4B	7.94E+00	2.98E-76	SERINC1	1.68E-01	1.00E+00	FIBIN	3.38E-01	1.00E+00
OSBPL9	2.06E+00	3.17E-76	TLDC1	1.65E-01	1.00E+00	DENND4C	2.59E-01	1.00E+00
SLC39A2	-5.17E+00	3.18E-76	MLEC	1.45E-01	1.00E+00	IL20RA	-4.54E-01	1.00E+00
LOC102405716	8.94E+00	3.28E-76	HOXD4	-3.21E-01	1.00E+00	ENDOD1	4.39E-01	1.00E+00
GPATCH1	1.62E+00	3.50E-76	LOC112579558	-1.93E-01	1.00E+00	RAVER1	-1.60E-01	1.00E+00
OLAH	8.59E+00	3.56E-76	SNAI1	3.79E-01	1.00E+00	NOD1	4.20E-01	1.00E+00
RNF169	1.91E+00	3.68E-76	KCNK15	4.99E-01	1.00E+00	NT5DC3	4.19E-01	1.00E+00
BET1L	-2.14E+00	3.72E-76	LOC112583591	-7.21E-01	1.00E+00	ALYREF	-2.12E-01	1.00E+00
RAD18	1.71E+00	3.76E-76	KCNK12	-7.15E-01	1.00E+00	PBX3	1.57E-01	1.00E+00
PHETA2	2.15E+00	4.04E-76	NENF	2.80E-01	1.00E+00	PCNT	-1.61E-01	1.00E+00

ATG4A	2.31E+00	4.24E-76	EDC4	1.36E-01	1.00E+00	LOC102400844	-3.50E-01	1.00E+00
RPL5	-3.37E+00	4.31E-76	ZNF821	-2.81E-01	1.00E+00	STAT4	3.74E-01	1.00E+00
PDE8A	1.96E+00	4.34E-76	ZBTB21	3.19E-01	1.00E+00	GPX7	-2.10E-01	1.00E+00
LOC102397708	-3.75E+00	4.48E-76	GTF2E1	1.31E-01	1.00E+00	MUSK	3.00E-01	1.00E+00
SERPINE2	-5.27E+00	4.64E-76	ERN1	2.07E-01	1.00E+00	ARHGAP24	5.62E-01	1.00E+00
IFI6	-6.72E+00	6.11E-76	OIP5	3.41E-01	1.00E+00	LOC112586981	-2.35E-01	1.00E+00
EEF1G	-3.58E+00	6.84E-76	A4GALT	-5.74E-01	1.00E+00	USB1	1.91E-01	1.00E+00
MTSS1L	2.19E+00	7.05E-76	DNAH3	5.72E-01	1.00E+00	PARP12	2.13E-01	1.00E+00
LOC102401999	2.85E+00	7.37E-76	LOC102403347	-3.87E-01	1.00E+00	ARL4A	-1.92E-01	1.00E+00
PPP1R14B	2.58E+00	7.38E-76	LOC102407026	-1.38E-01	1.00E+00	CCNB1	-2.15E-01	1.00E+00
ATP8B2	-2.98E+00	7.68E-76	ICAM1	-2.15E-01	1.00E+00	TSPAN9	2.13E-01	1.00E+00
FKRP	-3.62E+00	7.80E-76	MIER2	-1.51E-01	1.00E+00	LOC102403160	2.67E-01	1.00E+00
CCNT2	-2.22E+00	8.01E-76	SMG1	2.29E-01	1.00E+00	NR2C2	-2.06E-01	1.00E+00
CAND2	2.05E+00	8.96E-76	CREM	-1.81E-01	1.00E+00	E2F1	-2.16E-01	1.00E+00
LOC112587765	9.53E+00	9.06E-76	WDR3	1.48E-01	1.00E+00	ZMIZ2	1.66E-01	1.00E+00
TMEM165	-2.87E+00	9.87E-76	PLPP7	-3.42E-01	1.00E+00	LOC102403104	3.57E-01	1.00E+00
GAS6	-5.25E+00	1.06E-75	CSMD1	-1.65E-01	1.00E+00	MPP2	-3.00E-01	1.00E+00
HMOX2	2.48E+00	1.20E-75	B4GALNT1	5.09E-01	1.00E+00	RBMX	-1.84E-01	1.00E+00
SLC35C2	-2.64E+00	1.26E-75	NME3	-2.97E-01	1.00E+00	IFNLR1	2.86E-01	1.00E+00
SAV1	-3.64E+00	1.26E-75	STPG2	-3.36E-01	1.00E+00	MINK1	-1.70E-01	1.00E+00
CHMP6	-3.41E+00	1.28E-75	CYTB	-2.66E-01	1.00E+00	FAM81A	3.73E-01	1.00E+00
ANTXR2	-4.98E+00	1.30E-75	MISP3	-1.90E-01	1.00E+00	RPRD1B	-1.80E-01	1.00E+00
DMBX1	9.00E+00	1.30E-75	LOC112578877	-5.56E-01	1.00E+00	ABLIM1	-1.97E-01	1.00E+00
LOC112587480	7.35E+00	1.30E-75	DNAJC27	1.70E-01	1.00E+00	TRIP4	-1.65E-01	1.00E+00
PUM1	-2.80E+00	1.42E-75	AP5B1	2.35E-01	1.00E+00	CNTN3	4.98E-01	1.00E+00
LONP1	-2.49E+00	1.51E-75	PYGL	2.65E-01	1.00E+00	MAST4	4.71E-01	1.00E+00
LOC102405577	-5.01E+00	1.51E-75	EAPP	-1.57E-01	1.00E+00	FANK1	-2.81E-01	1.00E+00
GTF3C2	1.79E+00	1.51E-75	TPP1	-3.60E-01	1.00E+00	SLC16A13	-2.99E-01	1.00E+00
ABCG8	3.58E+00	1.59E-75	TOP1MT	-2.07E-01	1.00E+00	LGALS1	3.51E-01	1.00E+00
NABP1	2.85E+00	1.67E-75	CHERP	2.53E-01	1.00E+00	SH3PXD2A	-1.97E-01	1.00E+00
CMTR1	2.18E+00	1.70E-75	LOC102393147	-3.66E-01	1.00E+00	UNC80	5.76E-01	1.00E+00
CCDC54	8.52E+00	1.72E-75	LOC102394622	-2.87E-01	1.00E+00	DPY19L1	1.98E-01	1.00E+00
RNH1	-2.74E+00	1.75E-75	EPS8L1	1.91E-01	1.00E+00	SMNDC1	-1.88E-01	1.00E+00

DNASE1L1	-4.90E+00	1.79E-75	RING1	-3.76E-01	1.00E+00	ANKRD55	3.67E-01	1.00E+00
GNG4	5.40E+00	1.80E-75	PIK3R2	2.69E-01	1.00E+00	GSTA4	-1.93E-01	1.00E+00
SORBS1	3.05E+00	1.92E-75	RCSD1	-6.17E-01	1.00E+00	LOC102389538	3.31E-01	1.00E+00
CPED1	-5.39E+00	2.01E-75	GABRA5	-3.63E-01	1.00E+00	ZNF235	-2.45E-01	1.00E+00
MAOB	-7.95E+00	2.02E-75	LOC112581444	-3.48E-01	1.00E+00	SLC39A3	2.29E-01	1.00E+00
GNA12	1.89E+00	2.19E-75	PIK3C3	-1.50E-01	1.00E+00	LOC112578689	-4.06E-01	1.00E+00
KEAP1	-2.38E+00	2.21E-75	RAB32	-1.60E-01	1.00E+00	GM2A	-1.93E-01	1.00E+00
SDC3	4.08E+00	2.22E-75	NFIL3	-1.20E-01	1.00E+00	BTLA	-1.75E-01	1.00E+00
MRO	-5.21E+00	2.30E-75	PIP4K2A	-2.35E-01	1.00E+00	CCDC7	4.55E-01	1.00E+00
RNF144B	2.55E+00	2.49E-75	KANSL1	1.49E-01	1.00E+00	SPTY2D1	-3.26E-01	1.00E+00
DDX28	2.27E+00	2.52E-75	PSD3	-5.00E-01	1.00E+00	LOC102411164	2.69E-01	1.00E+00
PCDH18	-6.94E+00	2.56E-75	LUC7L	-2.24E-01	1.00E+00	HIPK2	2.38E-01	1.00E+00
MPV17	-3.41E+00	2.87E-75	ZFPM2	-4.72E-01	1.00E+00	CCNL2	-1.68E-01	1.00E+00
UNC45A	-2.29E+00	2.99E-75	ZNF699	2.58E-01	1.00E+00	MIA2	-1.69E-01	1.00E+00
CCNB2	3.42E+00	3.05E-75	SLC7A8	-3.81E-01	1.00E+00	SLC39A11	1.79E-01	1.00E+00
THOC3	2.14E+00	3.36E-75	RPL39	-2.11E-01	1.00E+00	ZNF157	3.36E-01	1.00E+00
ASPDH	5.76E+00	3.91E-75	ZFYVE1	1.26E-01	1.00E+00	SMIM30	1.93E-01	1.00E+00
RIC1	2.72E+00	4.06E-75	HYI	4.92E-01	1.00E+00	C5H1orf159	1.98E-01	1.00E+00
TMEM35B	4.30E+00	4.30E-75	LOC102389658	-3.38E-01	1.00E+00	CLHC1	3.04E-01	1.00E+00
RPS16	-3.67E+00	4.48E-75	ELOC	-2.03E-01	1.00E+00	DAP	-1.83E-01	1.00E+00
EGR4	5.66E+00	4.85E-75	MFHAS1	1.16E-01	1.00E+00	DCHS1	1.99E-01	1.00E+00
PIGP	2.73E+00	4.93E-75	HMX2	1.68E-01	1.00E+00	PIGT	-1.60E-01	1.00E+00
PHLDA3	-4.43E+00	5.14E-75	GAS2L1	3.88E-01	1.00E+00	CAPN6	2.31E-01	1.00E+00
CMYA5	4.17E+00	5.18E-75	CCDC6	1.34E-01	1.00E+00	REV1	-1.76E-01	1.00E+00
ATP5F1D	-2.85E+00	5.24E-75	EAF1	1.34E-01	1.00E+00	PUS7L	-2.27E-01	1.00E+00
LOC102397558	-3.84E+00	5.39E-75	MBD6	2.05E-01	1.00E+00	FAM155A	4.02E-01	1.00E+00
RPS26	-3.95E+00	5.41E-75	FRMD6	2.93E-01	1.00E+00	ZNF214	-3.44E-01	1.00E+00
RMND5B	1.88E+00	5.53E-75	DPP3	-1.75E-01	1.00E+00	LOC102410310	-3.95E-01	1.00E+00
CCDC158	-3.72E+00	5.68E-75	ADORA2A	-2.24E-01	1.00E+00	ZNF783	2.29E-01	1.00E+00
ENO1	-3.86E+00	5.78E-75	TMCO3	1.90E-01	1.00E+00	TAF1	-2.40E-01	1.00E+00
RPL32	-4.73E+00	6.21E-75	DSP	-5.61E-01	1.00E+00	LOC102411401	-4.94E-01	1.00E+00
CHST11	-2.49E+00	6.63E-75	PPHLN1	-2.35E-01	1.00E+00	LOC102405097	-4.45E-01	1.00E+00
LOC112577774	-3.10E+00	6.74E-75	PDE4DIP	1.66E-01	1.00E+00	DIAPH3	-1.57E-01	1.00E+00

CD276	-2.29E+00	6.77E-75	EFEMP2	8.59E-01	1.00E+00	LOC102399929	2.27E-01	1.00E+00
LRP3	-5.32E+00	6.93E-75	TMEM198	2.70E-01	1.00E+00	ID2	-2.19E-01	1.00E+00
RBKS	-4.18E+00	7.48E-75	LOC102391628	-1.80E-01	1.00E+00	ADAMTS10	-1.87E-01	1.00E+00
ITPKB	-4.36E+00	7.52E-75	CCNH	-1.64E-01	1.00E+00	FAM133B	-1.64E-01	1.00E+00
ABTB1	-4.38E+00	7.72E-75	LOC102411316	4.33E-01	1.00E+00	P4HA3	-2.36E-01	1.00E+00
HDAC8	-2.94E+00	8.65E-75	C21H3orf20	2.98E-01	1.00E+00	SMYD3	2.17E-01	1.00E+00
ZNHIT6	1.78E+00	1.00E-74	DAZAP1	-1.70E-01	1.00E+00	LOC102390532	-5.39E-01	1.00E+00
LINS1	1.87E+00	1.03E-74	DYRK1A	-1.65E-01	1.00E+00	CAPN7	-1.65E-01	1.00E+00
FAM8A1	-1.84E+00	1.07E-74	SEC11A	-1.78E-01	1.00E+00	TMOD2	4.69E-01	1.00E+00
PPP1R26	-3.27E+00	1.24E-74	ZNF839	-1.96E-01	1.00E+00	CAPN2	1.78E-01	1.00E+00
CLIP4	3.66E+00	1.36E-74	TRAPPC6B	-1.93E-01	1.00E+00	VPS18	1.88E-01	1.00E+00
SP2	1.95E+00	1.37E-74	PSME4	2.20E-01	1.00E+00	DTX1	3.94E-01	1.00E+00
LOC112577987	-9.49E+00	1.40E-74	LOC102395425	-4.26E-01	1.00E+00	PIKFYVE	-3.42E-01	1.00E+00
TXLNG	2.18E+00	1.40E-74	RAD1	2.96E-01	1.00E+00	LOC102413429	-2.77E-01	1.00E+00
DZIP1	-2.18E+00	1.43E-74	LOC102395815	3.29E-01	1.00E+00	EIF2B4	-1.77E-01	1.00E+00
CARM1	2.11E+00	1.47E-74	MASTL	1.24E-01	1.00E+00	ZYX	2.47E-01	1.00E+00
LIG1	3.64E+00	1.53E-74	MYO3B	-1.69E-01	1.00E+00	LOC102394491	2.49E-01	1.00E+00
INVS	-2.05E+00	1.58E-74	ELOVL3	-2.71E-01	1.00E+00	LOC102410667	-4.79E-01	1.00E+00
ADNP2	2.11E+00	1.72E-74	TBCK	1.90E-01	1.00E+00	CCDC97	2.41E-01	1.00E+00
NCOR2	-3.53E+00	1.76E-74	INTS5	1.51E-01	1.00E+00	ZNF579	-2.17E-01	1.00E+00
HVCN1	4.24E+00	1.98E-74	GLIS1	-6.97E-01	1.00E+00	JAK1	2.13E-01	1.00E+00
SIN3B	1.90E+00	2.45E-74	KLHDC9	-1.91E-01	1.00E+00	GJA5	3.33E-01	1.00E+00
TMEM164	-2.72E+00	2.48E-74	MICALL2	-7.61E-01	1.00E+00	SPA17	-3.10E-01	1.00E+00
CMSS1	-6.08E+00	2.57E-74	DRD1	1.33E-01	1.00E+00	CHST11	-2.18E-01	1.00E+00
MAN2C1	-2.73E+00	2.59E-74	MARK2	1.46E-01	1.00E+00	SLC29A1	-2.30E-01	1.00E+00
RPL37	-4.49E+00	2.67E-74	DHX38	2.58E-01	1.00E+00	CCDC77	-1.93E-01	1.00E+00
TCF15	6.56E+00	2.70E-74	ATP5PO	-2.10E-01	1.00E+00	STRA6	-2.96E-01	1.00E+00
LOC112578116	-3.80E+00	2.82E-74	TRAF6	1.08E-01	1.00E+00	ZNF322	1.87E-01	1.00E+00
CSPG5	2.80E+00	2.87E-74	CASP6	-2.16E-01	1.00E+00	ANKS1A	-1.68E-01	1.00E+00
MBD2	-1.80E+00	3.31E-74	CNST	3.11E-01	1.00E+00	C6H1orf50	1.84E-01	1.00E+00
TTC23	-5.06E+00	3.36E-74	AKAP4	2.71E-01	1.00E+00	TSNARE1	-2.09E-01	1.00E+00
DST	-4.27E+00	3.36E-74	LOC102416091	-4.10E-01	1.00E+00	FUBP3	-2.00E-01	1.00E+00
CLMP	-4.19E+00	3.60E-74	SMARCA2	2.18E-01	1.00E+00	BCL11A	-2.00E-01	1.00E+00

KIF1C	-1.99E+00	3.77E-74	FBXO21	1.34E-01	1.00E+00	KIAA1211	1.98E-01	1.00E+00
LOC102393839	2.14E+00	3.80E-74	IDS	-2.22E-01	1.00E+00	LOC102392605	-2.00E-01	1.00E+00
PSMD7	-2.57E+00	3.87E-74	FAIM2	-3.28E-01	1.00E+00	MFSD8	-2.08E-01	1.00E+00
LOC112582121	6.08E+00	3.99E-74	HAUS8	-1.58E-01	1.00E+00	ILDR2	4.11E-01	1.00E+00
SLC35F5	-2.18E+00	4.18E-74	EBF2	-4.23E-01	1.00E+00	SRRM2	-2.55E-01	1.00E+00
PLK3	2.13E+00	4.54E-74	UNKL	2.41E-01	1.00E+00	G6PD	-2.50E-01	1.00E+00
RPS28	-3.90E+00	4.78E-74	ATP6V1FNB	-2.47E-01	1.00E+00	GIPC3	4.75E-01	1.00E+00
PDCD10	-2.46E+00	4.82E-74	ATP11B	1.61E-01	1.00E+00	PID1	2.27E-01	1.00E+00
LOC112585503	2.76E+00	5.00E-74	FOXB1	-1.05E+00	1.00E+00	MTURN	2.24E-01	1.00E+00
CHPF	-2.93E+00	5.02E-74	SNX2	-2.15E-01	1.00E+00	RARG	3.22E-01	1.00E+00
ABI1	1.79E+00	5.02E-74	TNFRSF1A	-7.69E-01	1.00E+00	ABHD6	-2.05E-01	1.00E+00
CELSR3	1.80E+00	5.17E-74	LOC102389062	2.99E-01	1.00E+00	RPUSD4	-1.68E-01	1.00E+00
STOML1	-4.11E+00	5.22E-74	TEAD3	1.76E-01	1.00E+00	USP54	-2.06E-01	1.00E+00
GLE1	1.67E+00	5.27E-74	LOC102389399	2.99E-01	1.00E+00	ATP2A2	-1.98E-01	1.00E+00
SLC29A4	2.17E+00	5.57E-74	ALDH3B1	-7.50E-01	1.00E+00	NOC4L	-1.76E-01	1.00E+00
COL4A1	-5.86E+00	6.15E-74	SH3TC1	1.03E+00	1.00E+00	SNX9	2.10E-01	1.00E+00
LOC112578061	9.35E+00	6.16E-74	TMEM8A	3.26E-01	1.00E+00	MAU2	-1.86E-01	1.00E+00
ARRDC2	4.83E+00	6.31E-74	EDIL3	-2.81E-01	1.00E+00	CDR2L	2.43E-01	1.00E+00
U2SURP	-1.93E+00	6.46E-74	C18H19orf47	1.76E-01	1.00E+00	TSPAN32	-4.06E-01	1.00E+00
OLFML2B	-5.10E+00	7.65E-74	ATP5S	5.99E-01	1.00E+00	ACOX2	-2.46E-01	1.00E+00
BBS5	2.19E+00	7.83E-74	CA13	-5.82E-01	1.00E+00	ISM1	3.55E-01	1.00E+00
TRIM13	1.58E+00	8.12E-74	LIPT2	-1.82E-01	1.00E+00	KIF20B	-2.01E-01	1.00E+00
LOC112587954	-4.25E+00	8.16E-74	ATP5F1C	-1.24E-01	1.00E+00	KIAA1217	2.10E-01	1.00E+00
WDFY1	-1.58E+00	8.32E-74	KBTBD2	1.73E-01	1.00E+00	TBL1XR1	1.58E-01	1.00E+00
WLS	2.42E+00	8.64E-74	TMEM86A	-1.52E-01	1.00E+00	GREB1	-2.54E-01	1.00E+00
DNAJB4	-3.38E+00	1.08E-73	LOC112583845	-6.02E-01	1.00E+00	RGS6	2.61E-01	1.00E+00
ZIM2	7.74E+00	1.09E-73	RAP1GDS1	1.55E-01	1.00E+00	RTKN2	-2.02E-01	1.00E+00
HERC3	2.71E+00	1.14E-73	SRPX	-1.33E-01	1.00E+00	PLCL2	1.97E-01	1.00E+00
USP46	1.83E+00	1.26E-73	MED21	-1.81E-01	1.00E+00	ADAMTS3	-1.77E-01	1.00E+00
BRMS1L	1.89E+00	1.29E-73	HIPK4	4.42E-01	1.00E+00	PDXDC1	-1.69E-01	1.00E+00
SLC25A35	2.83E+00	1.31E-73	SGF29	1.56E-01	1.00E+00	STARD13	2.98E-01	1.00E+00
FRMD3	1.81E+00	1.35E-73	THOC2	2.20E-01	1.00E+00	HAP1	-4.66E-01	1.00E+00
DUPD1	6.46E+00	1.40E-73	RAB40B	8.29E-01	1.00E+00	MAPK8IP1	-2.21E-	1.00E+00

							01	
NUDCD3	-2.01E+00	1.43E-73	SAG	-5.54E-01	1.00E+00	POLR1C	1.92E-01	1.00E+00
DYNLL1	2.69E+00	1.53E-73	SNX15	-2.08E-01	1.00E+00	LOC112581374	-5.87E-01	1.00E+00
BCAM	-7.60E+00	1.62E-73	MEMO1	1.45E-01	1.00E+00	MYBPC2	3.33E-01	1.00E+00
SHF	-4.43E+00	1.63E-73	KDM7A	-4.58E-01	1.00E+00	GGCT	-2.67E-01	1.00E+00
ZNF746	1.90E+00	1.86E-73	CETN2	-1.35E-01	1.00E+00	KLHL33	-3.77E-01	1.00E+00
LOC102403078	-8.77E+00	1.92E-73	AK3	-1.68E-01	1.00E+00	FNDC11	-3.27E-01	1.00E+00
NT5C3B	-2.69E+00	2.02E-73	TNFRSF13B	-4.08E-01	1.00E+00	KIAA1551	2.52E-01	1.00E+00
HMGXB3	1.94E+00	2.07E-73	LARS2	-3.32E-01	1.00E+00	ASGR1	-3.73E-01	1.00E+00
ZBTB24	-3.01E+00	2.08E-73	SRCAP	2.72E-01	1.00E+00	TMEM54	3.95E-01	1.00E+00
TNNT3	-9.20E+00	2.10E-73	LOC102409972	-4.47E-01	1.00E+00	LOC102416503	4.28E-01	1.00E+00
NFE2L1	-2.47E+00	2.19E-73	GATAD2B	2.02E-01	1.00E+00	CPM	-3.09E-01	1.00E+00
SOX9	-5.85E+00	2.30E-73	RPL12	-2.12E-01	1.00E+00	BGN	2.97E-01	1.00E+00
TSC22D4	-2.11E+00	2.41E-73	BCL11A	-8.12E-01	1.00E+00	SKAP2	2.45E-01	1.00E+00
ADPGK	-2.11E+00	2.51E-73	ISM1	-2.71E-01	1.00E+00	FAM229A	-3.95E-01	1.00E+00
ZCCHC10	-4.88E+00	2.55E-73	OSR2	2.69E-01	1.00E+00	PELI1	2.45E-01	1.00E+00
ADGRA3	-2.56E+00	2.56E-73	HYPK	-1.76E-01	1.00E+00	HDAC4	2.07E-01	1.00E+00
CLEC16A	1.96E+00	2.58E-73	EFNA5	-2.47E-01	1.00E+00	PPP3CA	2.10E-01	1.00E+00
MAP3K7CL	5.39E+00	2.66E-73	STAP1	2.06E-01	1.00E+00	EFS	3.97E-01	1.00E+00
ATP10D	3.01E+00	2.90E-73	CDC14A	-4.38E-01	1.00E+00	DOT1L	1.94E-01	1.00E+00
LOC102409738	5.26E+00	2.97E-73	B3GNT2	-5.14E-01	1.00E+00	LOC112583669	-3.72E-01	1.00E+00
ROM1	2.86E+00	3.02E-73	RADIL	1.19E-01	1.00E+00	SLC39A10	2.55E-01	1.00E+00
GTF2I	-1.79E+00	3.11E-73	LOC102404358	-2.71E-01	1.00E+00	HSPA4	1.65E-01	1.00E+00
SH3GLB1	2.08E+00	3.14E-73	ANKRD9	-2.78E-01	1.00E+00	TRIM35	1.79E-01	1.00E+00
POLR3E	-2.07E+00	3.61E-73	PLD3	-2.86E-01	1.00E+00	CPNE8	2.77E-01	1.00E+00
WAC	2.75E+00	3.89E-73	LOC112579214	-6.75E-01	1.00E+00	GAB3	3.56E-01	1.00E+00
PRAF2	-2.98E+00	3.96E-73	EEF1D	-1.53E-01	1.00E+00	CASP2	-1.67E-01	1.00E+00
CUX2	6.70E+00	3.98E-73	BICD1	3.09E-01	1.00E+00	BOD1	1.74E-01	1.00E+00
PUF60	-2.29E+00	4.07E-73	ST3GAL6	1.53E-01	1.00E+00	LOC102416091	3.84E-01	1.00E+00
PLP2	-2.69E+00	4.13E-73	PHF21A	1.54E-01	1.00E+00	TRIP13	-1.89E-01	1.00E+00
STAU2	-3.16E+00	4.16E-73	LOC102415288	2.42E-01	1.00E+00	GRASP	1.95E-01	1.00E+00
PCDH7	-6.70E+00	4.20E-73	NUS1	1.53E-01	1.00E+00	LOC102389990	-3.23E-01	1.00E+00
FAM118B	1.58E+00	4.40E-73	HECTD4	1.68E-01	1.00E+00	NAGA	1.53E-01	1.00E+00
LOC112581795	5.52E+00	4.41E-73	ADGRG2	-2.16E-01	1.00E+00	KDM1B	3.90E-01	1.00E+00

WDR5	2.09E+00	4.69E-73	LNX2	1.26E-01	1.00E+00	PSMA2	1.72E-01	1.00E+00
SALL1	7.28E+00	4.85E-73	DECR1	-4.41E-01	1.00E+00	POLQ	-2.22E-01	1.00E+00
QRICH2	3.21E+00	4.94E-73	SPATA22	-1.37E-01	1.00E+00	RASAL2	2.27E-01	1.00E+00
FUBP3	-3.03E+00	5.06E-73	CABP2	-5.02E-01	1.00E+00	PARP3	1.99E-01	1.00E+00
PTOV1	-2.66E+00	5.07E-73	NHLRC3	3.72E-01	1.00E+00	RBM15	-1.74E-01	1.00E+00
PTMA	-3.66E+00	5.09E-73	BUD13	-1.36E-01	1.00E+00	EDN1	2.97E-01	1.00E+00
PEX11G	-7.74E+00	5.10E-73	ARID4A	4.32E-01	1.00E+00	LOC112585216	-5.09E-01	1.00E+00
LOC112587851	5.80E+00	5.16E-73	JMJD7	-5.43E-01	1.00E+00	HIP1	2.29E-01	1.00E+00
LOC102404792	8.65E+00	5.28E-73	MIEN1	-4.81E-01	1.00E+00	LOC102392890	1.75E-01	1.00E+00
ABCF2	1.84E+00	5.48E-73	FBXO34	1.29E-01	1.00E+00	LOC102398905	-1.83E-01	1.00E+00
GPX7	-5.85E+00	6.63E-73	CDC27	1.77E-01	1.00E+00	C16H11orf96	-3.68E-01	1.00E+00
ZNF740	-2.85E+00	6.74E-73	DDX51	-2.76E-01	1.00E+00	LOC112583892	4.09E-01	1.00E+00
CARNMT1	2.11E+00	6.76E-73	ST6GALNAC4	-2.94E-01	1.00E+00	POLA1	-1.66E-01	1.00E+00
ZNF212	1.90E+00	6.82E-73	MVB12B	1.33E-01	1.00E+00	CLCN5	2.00E-01	1.00E+00
DIDO1	-2.82E+00	7.03E-73	ORC1	2.00E-01	1.00E+00	HSD17B7	-1.75E-01	1.00E+00
WDR90	-2.42E+00	7.23E-73	FOXP4	1.85E-01	1.00E+00	SYAP1	1.76E-01	1.00E+00
MYO18A	2.19E+00	7.81E-73	LIG3	1.97E-01	1.00E+00	FBXW8	1.65E-01	1.00E+00
TOPAZ1	4.72E+00	8.29E-73	WNK3	2.06E-01	1.00E+00	HIKESHI	1.82E-01	1.00E+00
LOC102400966	2.97E+00	8.40E-73	KLF11	-1.39E-01	1.00E+00	CD63	2.29E-01	1.00E+00
TRIM47	-6.45E+00	8.45E-73	LOC102408209	-3.07E-01	1.00E+00	LOC102410187	4.15E-01	1.00E+00
AFF2	-5.18E+00	8.53E-73	TBCC	1.38E-01	1.00E+00	ABCA10	4.41E-01	1.00E+00
KBTBD12	8.85E+00	8.65E-73	TRADD	-4.16E-01	1.00E+00	PTPRG	2.39E-01	1.00E+00
ZDHHC17	-3.17E+00	8.65E-73	MTMR3	2.15E-01	1.00E+00	EXOC5	2.18E-01	1.00E+00
PLXNB1	-2.89E+00	8.86E-73	SLC25A47	-4.37E-01	1.00E+00	BOP1	2.10E-01	1.00E+00
RYK	-2.03E+00	9.04E-73	SLIT3	-2.07E-01	1.00E+00	MSI1	1.97E-01	1.00E+00
CLK4	-1.92E+00	9.49E-73	ASTN2	-1.17E-01	1.00E+00	LOC102405154	-2.47E-01	1.00E+00
DHDDS	1.58E+00	1.06E-72	HCN4	-1.87E-01	1.00E+00	FGF9	3.71E-01	1.00E+00
LOC102413289	1.88E+00	1.07E-72	SWSAP1	-1.99E-01	1.00E+00	RABGGTA	-1.95E-01	1.00E+00
NRK	5.86E+00	1.08E-72	SRSF11	1.58E-01	1.00E+00	HAUS6	1.72E-01	1.00E+00
DHX36	1.80E+00	1.28E-72	ZNF215	2.01E-01	1.00E+00	UPK3B	3.50E-01	1.00E+00
RAPH1	2.70E+00	1.32E-72	NCAPH2	1.88E-01	1.00E+00	MYO1E	1.88E-01	1.00E+00
ING2	-2.83E+00	1.39E-72	LOC102400287	-1.47E-01	1.00E+00	TRIM62	-4.09E-01	1.00E+00
RBBP7	2.26E+00	1.39E-72	WFDC1	4.65E-01	1.00E+00	CAST	2.20E-01	1.00E+00
DCP1B	-6.28E+00	1.62E-72	FBXL15	-4.54E-01	1.00E+00	BRD8	-1.58E-01	1.00E+00
MAPK14	1.71E+00	1.83E-72	LOC112584709	-3.27E-01	1.00E+00	ADCY3	3.24E-01	1.00E+00

TAP2	-5.34E+00	1.98E-72	CDKN1C	-3.05E-01	1.00E+00	ZBTB34	-3.63E-01	1.00E+00
PHF2	-4.12E+00	2.14E-72	FOXM1	1.60E-01	1.00E+00	SMARCA1	-1.66E-01	1.00E+00
SLC39A12	6.53E+00	2.45E-72	ARMC1	1.13E-01	1.00E+00	SCARB1	-2.29E-01	1.00E+00
LOC102389871	7.85E+00	2.73E-72	URB2	2.59E-01	1.00E+00	THSD7A	-4.07E-01	1.00E+00
RPL27A	-3.93E+00	2.77E-72	MAP3K2	3.69E-01	1.00E+00	DGAT2	-4.35E-01	1.00E+00
RPSA	-3.52E+00	2.78E-72	C15H8orf76	-1.23E-01	1.00E+00	UBE2C	-2.53E-01	1.00E+00
LOC102399966	-3.43E+00	2.90E-72	EXOSC6	-2.75E-01	1.00E+00	ABCC3	2.12E-01	1.00E+00
EDEM3	-2.44E+00	3.05E-72	BRD3	1.52E-01	1.00E+00	LOC112579974	3.66E-01	1.00E+00
VPS54	-2.05E+00	3.06E-72	KDELR3	-1.90E-01	1.00E+00	METTL22	-2.21E-01	1.00E+00
SRSF9	-2.09E+00	3.13E-72	GCC2	2.09E-01	1.00E+00	CDC42BPG	-2.73E-01	1.00E+00
KCNC3	3.29E+00	3.18E-72	ACP6	-1.22E-01	1.00E+00	PRUNE1	-1.74E-01	1.00E+00
TICRR	2.52E+00	3.20E-72	ITSN1	2.25E-01	1.00E+00	PAN2	-1.56E-01	1.00E+00
TPI1	-3.47E+00	3.27E-72	ZNF454	2.14E-01	1.00E+00	DRP2	4.09E-01	1.00E+00
MXRA7	-3.09E+00	3.29E-72	LOC102406721	-7.25E-01	1.00E+00	PPM1E	4.26E-01	1.00E+00
EVC	-4.02E+00	3.53E-72	SDC3	1.33E-01	1.00E+00	SRSF6	-1.90E-01	1.00E+00
AKAP8L	-2.27E+00	3.64E-72	LOC112580306	-6.38E-01	1.00E+00	CHPT1	-1.71E-01	1.00E+00
KIFC2	-2.82E+00	3.68E-72	GCH1	-1.67E-01	1.00E+00	HHAT	3.67E-01	1.00E+00
C16H11orf58	-1.69E+00	3.94E-72	KIF2B	8.02E-01	1.00E+00	NEU1	1.80E-01	1.00E+00
LRRC8C	3.16E+00	4.10E-72	VAV2	-1.77E-01	1.00E+00	TRIM14	2.12E-01	1.00E+00
TMEM161A	-3.38E+00	4.19E-72	CASP2	1.43E-01	1.00E+00	LOC102414928	3.78E-01	1.00E+00
CACUL1	-2.07E+00	5.59E-72	HAUS2	1.47E-01	1.00E+00	PKD2L1	-3.13E-01	1.00E+00
ICOSLG	-6.19E+00	5.86E-72	LOC102406687	-3.16E-01	1.00E+00	NUMBL	1.82E-01	1.00E+00
HTRA2	-3.09E+00	5.96E-72	ILF2	-1.50E-01	1.00E+00	PP2D1	3.13E-01	1.00E+00
LOC112583991	6.67E+00	6.16E-72	LOC112578076	-7.19E-01	1.00E+00	NXPE3	2.10E-01	1.00E+00
LOC102389399	4.65E+00	7.08E-72	LRRC27	2.90E-01	1.00E+00	LOC112577775	-1.91E-01	1.00E+00
FUBP1	-1.68E+00	7.25E-72	TENM4	-1.56E-01	1.00E+00	HCLS1	8.00E-01	1.00E+00
BNIP2	-2.26E+00	7.38E-72	LOC112586931	3.12E-01	1.00E+00	LOC102404166	3.82E-01	1.00E+00
SYNE1	-3.44E+00	7.56E-72	CHMP4B	-1.26E-01	1.00E+00	VEGFC	1.82E-01	1.00E+00
COBLL1	4.51E+00	7.64E-72	CORIN	3.00E-01	1.00E+00	ZNF311	-2.70E-01	1.00E+00
NTN1	-2.19E+00	7.68E-72	SREK1	1.48E-01	1.00E+00	MAPT	3.77E-01	1.00E+00
ZFP36L2	-5.78E+00	8.11E-72	TRIM67	-5.27E-01	1.00E+00	LOC102414399	-1.62E-01	1.00E+00
ATXN1	2.75E+00	8.46E-72	GRIA3	-2.38E-01	1.00E+00	TDRD9	6.07E-01	1.00E+00

TWSG1	-4.72E+00	9.52E-72	CCAR2	1.52E-01	1.00E+00	SNRK	-2.53E-01	1.00E+00
LOC102392890	1.65E+00	1.04E-71	GNA12	1.13E-01	1.00E+00	YY1	-1.50E-01	1.00E+00
ZDHHC23	3.00E+00	1.11E-71	HAX1	-1.65E-01	1.00E+00	ACCS	-2.73E-01	1.00E+00
AKNAD1	5.15E+00	1.16E-71	LOC112587638	6.79E-01	1.00E+00	SLX4IP	1.85E-01	1.00E+00
FOXG1	5.29E+00	1.23E-71	TSEN15	-3.75E-01	1.00E+00	GMFB	1.83E-01	1.00E+00
POLH	-2.98E+00	1.25E-71	ART3	1.51E-01	1.00E+00	ZXDC	-1.62E-01	1.00E+00
RIMS4	6.20E+00	1.26E-71	TENT2	1.35E-01	1.00E+00	ZSCAN30	-3.85E-01	1.00E+00
PRUNE2	2.82E+00	1.27E-71	EPB41	1.84E-01	1.00E+00	TMEM115	1.71E-01	1.00E+00
FAIM	-2.54E+00	1.29E-71	SULT2B1	6.01E-01	1.00E+00	TFDP2	1.51E-01	1.00E+00
ELAC1	-3.22E+00	1.29E-71	DUSP13	1.38E-01	1.00E+00	ZKSCAN1	-2.23E-01	1.00E+00
PKN2	-2.85E+00	1.32E-71	EZR	1.55E-01	1.00E+00	RAD1	-2.61E-01	1.00E+00
PPIC	-4.79E+00	1.59E-71	LOC102389028	-2.97E-01	1.00E+00	RBMS1	-1.86E-01	1.00E+00
KCNQ4	5.99E+00	1.59E-71	SNRPG	-2.43E-01	1.00E+00	LRRC41	-2.74E-01	1.00E+00
ARFRP1	-2.67E+00	1.69E-71	LOC112581808	-6.34E-01	1.00E+00	NTN4	2.32E-01	1.00E+00
XXYLT1	1.85E+00	1.75E-71	PHF13	1.62E-01	1.00E+00	RPS4X	-2.18E-01	1.00E+00
ELF2	-3.63E+00	1.79E-71	ND1	-2.73E-01	1.00E+00	CSGALNACT2	-2.06E-01	1.00E+00
PCLAF	-3.84E+00	2.11E-71	FAM168A	1.76E-01	1.00E+00	CHKA	-1.59E-01	1.00E+00
NOS2	-5.56E+00	2.21E-71	SMARCAL1	1.15E-01	1.00E+00	KCNK12	-4.81E-01	1.00E+00
PARP9	-4.17E+00	2.34E-71	CCDC88A	1.83E-01	1.00E+00	NCSTN	-1.55E-01	1.00E+00
SUSD3	4.41E+00	2.45E-71	JRK	1.92E-01	1.00E+00	LUC7L3	-1.84E-01	1.00E+00
RAB43	-2.60E+00	2.72E-71	MAP2K7	1.35E-01	1.00E+00	NUS1	-1.62E-01	1.00E+00
LOC102397361	-3.29E+00	2.77E-71	SLC25A21	-5.66E-01	1.00E+00	ANO1	3.75E-01	1.00E+00
PPP2R2C	-4.39E+00	2.93E-71	LIMS1	-5.06E-01	1.00E+00	CES5A	2.87E-01	1.00E+00
LOC112581450	-5.62E+00	3.05E-71	TAMM41	-2.09E-01	1.00E+00	GLI1	4.08E-01	1.00E+00
LOC112581586	9.76E+00	3.12E-71	NXPE3	2.33E-01	1.00E+00	WDFY4	-1.87E-01	1.00E+00
LOC102411782	-8.19E+00	3.15E-71	ANKRD52	2.29E-01	1.00E+00	RASSF8	2.24E-01	1.00E+00
PHKA1	-2.10E+00	3.28E-71	POLE4	-2.01E-01	1.00E+00	CD19	-4.79E-01	1.00E+00
ABR	1.57E+00	3.39E-71	SHOC2	1.33E-01	1.00E+00	FOXM1	-2.22E-01	1.00E+00
SLC17A7	4.53E+00	3.53E-71	PPRC1	1.63E-01	1.00E+00	LOC112585051	-3.84E-01	1.00E+00
MFAP3	-3.15E+00	3.60E-71	LOC102392425	-3.17E-01	1.00E+00	SPOCK2	3.89E-01	1.00E+00
ICMT	2.28E+00	4.51E-71	SETDB1	1.47E-01	1.00E+00	WAC	-1.72E-01	1.00E+00
MSX1	4.72E+00	4.55E-71	LOC102407872	-1.47E-01	1.00E+00	LOC102407915	3.70E-01	1.00E+00
MTHFD1	2.28E+00	4.70E-71	RPS23	-1.72E-01	1.00E+00	ABI3BP	5.22E-01	1.00E+00

STAM	-2.60E+00	5.61E-71	DPY19L4	2.73E-01	1.00E+00	ARMC8	-1.65E-01	1.00E+00
TMEM106A	2.48E+00	5.66E-71	SYF2	-1.15E-01	1.00E+00	RPUSD2	2.25E-01	1.00E+00
CBR3	4.27E+00	5.84E-71	RASA1	1.42E-01	1.00E+00	SUMO3	-1.59E-01	1.00E+00
GAS1	-6.66E+00	6.00E-71	KAT6B	2.18E-01	1.00E+00	FBLN7	3.88E-01	1.00E+00
GLMN	1.83E+00	6.30E-71	FBXO47	-6.04E-01	1.00E+00	SLC43A3	2.17E-01	1.00E+00
TUSC3	-1.60E+00	6.50E-71	LOC112587028	6.93E-01	1.00E+00	LOC112587926	4.20E-01	1.00E+00
PHTF1	1.98E+00	7.10E-71	PCYT1A	1.76E-01	1.00E+00	FBXO34	-1.95E-01	1.00E+00
SLC35B2	-2.70E+00	7.53E-71	LYSMD4	-2.20E-01	1.00E+00	RECK	-2.17E-01	1.00E+00
LOC112581592	7.11E+00	8.40E-71	DGKE	3.24E-01	1.00E+00	RYR2	2.30E-01	1.00E+00
NEXMIF	-5.83E+00	9.32E-71	METTL1	-3.82E-01	1.00E+00	SGSM2	2.04E-01	1.00E+00
SPATA2L	3.69E+00	1.03E-70	ZKSCAN2	2.15E-01	1.00E+00	DUSP5	3.27E-01	1.00E+00
PFAS	-4.28E+00	1.05E-70	DCAF5	1.80E-01	1.00E+00	MRPL57	-2.87E-01	1.00E+00
SRMS	-6.18E+00	1.08E-70	SH2B3	1.15E-01	1.00E+00	SLC39A13	1.48E-01	1.00E+00
RPL3	-4.11E+00	1.09E-70	HIVEP1	3.10E-01	1.00E+00	MDC1	-1.77E-01	1.00E+00
SMARCAL1	1.53E+00	1.19E-70	RBPMS	-1.32E-01	1.00E+00	CYGB	-3.62E-01	1.00E+00
BLOC1S2	-3.05E+00	1.39E-70	LOC112588003	-3.91E-01	1.00E+00	USP2	-2.53E-01	1.00E+00
NAB2	-3.12E+00	1.50E-70	ADHFE1	-6.75E-01	1.00E+00	IRAK1	1.54E-01	1.00E+00
C6H1orf194	4.11E+00	1.53E-70	ITGA11	-4.78E-01	1.00E+00	SPDL1	-1.92E-01	1.00E+00
FAM160A1	3.29E+00	1.57E-70	PFDN2	-1.72E-01	1.00E+00	IGIP	4.39E-01	1.00E+00
LOC102400809	6.23E+00	1.64E-70	BTBD18	2.54E-01	1.00E+00	GPRC5A	4.23E-01	1.00E+00
SHC1	2.08E+00	1.75E-70	PCGF2	2.52E-01	1.00E+00	PRKCD	2.31E-01	1.00E+00
CHCHD10	-5.62E+00	1.76E-70	ANLN	1.62E-01	1.00E+00	PARS2	-2.20E-01	1.00E+00
MFN2	1.74E+00	2.01E-70	LRRN1	-5.90E-01	1.00E+00	RABEP2	1.82E-01	1.00E+00
PDHB	-2.18E+00	2.03E-70	CRAT	1.26E-01	1.00E+00	TMEM127	1.62E-01	1.00E+00
MAPK8	-2.14E+00	2.35E-70	UXT	-2.31E-01	1.00E+00	NRBF2	-2.25E-01	1.00E+00
SMC6	-2.09E+00	2.41E-70	DUS1L	-2.31E-01	1.00E+00	CADPS2	-1.63E-01	1.00E+00
ARHGAP23	-4.04E+00	2.50E-70	TRAPPC2	-1.13E-01	1.00E+00	ABCC6	-3.66E-01	1.00E+00
PYCR1	-5.65E+00	2.60E-70	NUP54	1.49E-01	1.00E+00	ADARB1	1.93E-01	1.00E+00
BYSL	1.76E+00	2.67E-70	CXHXorf67	-2.86E-01	1.00E+00	KIF2C	-2.13E-01	1.00E+00
FOXK1	-2.35E+00	2.70E-70	PTN	-6.14E-01	1.00E+00	HSPB2	4.15E-01	1.00E+00
CAPNS1	-2.56E+00	2.72E-70	LOC102400645	-6.41E-01	1.00E+00	SLC49A3	-1.77E-01	1.00E+00
FHIT	2.70E+00	2.85E-70	LOC112577644	-5.70E-01	1.00E+00	CLIP3	1.74E-01	1.00E+00
LOC102395582	1.92E+00	3.14E-70	NDRG2	-8.26E-01	1.00E+00	E2F7	-2.63E-01	1.00E+00
PGLS	-	3.17E-70	SELENBP1	-2.39E-	1.00E+00	CALB2	-3.73E-	1.00E+00

	3.04E+00			01			01	
ARHGEF2	-4.22E+00	3.32E-70	ABL2	2.88E-01	1.00E+00	PTP4A1	-1.91E-01	1.00E+00
RD3	5.37E+00	3.33E-70	LOC102392295	-1.70E-01	1.00E+00	LOC102410588	-4.00E-01	1.00E+00
COL22A1	4.54E+00	3.34E-70	FRYL	1.23E-01	1.00E+00	AIG1	-1.70E-01	1.00E+00
MAP7D2	-1.95E+00	3.45E-70	DIP2A	3.30E-01	1.00E+00	ATAD2	-2.34E-01	1.00E+00
ZNF484	-4.05E+00	3.69E-70	KIAA1549	1.87E-01	1.00E+00	HSD11B1L	-1.79E-01	1.00E+00
PPP1R18	-6.40E+00	3.79E-70	LOC102411142	-2.59E-01	1.00E+00	ERCC2	-1.79E-01	1.00E+00
RANBP10	-2.03E+00	3.99E-70	PPP2R1A	1.19E-01	1.00E+00	CTGF	2.84E-01	1.00E+00
LOC102411198	6.59E+00	4.16E-70	LOC112586217	-4.84E-01	1.00E+00	TPM4	2.32E-01	1.00E+00
KYAT1	-4.83E+00	4.41E-70	FAM78B	-6.19E-01	1.00E+00	LOC102413264	-1.51E-01	1.00E+00
ADRA2C	8.46E+00	4.43E-70	TTLL8	3.96E-01	1.00E+00	NEPRO	-2.05E-01	1.00E+00
BVES	4.41E+00	4.52E-70	APH1A	1.21E-01	1.00E+00	TEX45	-2.71E-01	1.00E+00
FMO2	5.93E+00	4.64E-70	CDK15	-6.15E-01	1.00E+00	SEPT9	1.64E-01	1.00E+00
UTP25	1.63E+00	4.91E-70	NRDE2	-3.59E-01	1.00E+00	DAPP1	4.72E-01	1.00E+00
B3GNT8	-8.01E+00	5.08E-70	FAM170A	-4.57E-01	1.00E+00	LOC102406736	3.68E-01	1.00E+00
TPM3	-1.85E+00	5.20E-70	CHMP5	1.12E-01	1.00E+00	ATP13A2	-1.78E-01	1.00E+00
LOC102396503	-5.02E+00	5.72E-70	LOC102389856	-2.91E-01	1.00E+00	LOC112578016	2.86E-01	1.00E+00
CD47	-2.50E+00	6.15E-70	LOC112582945	-3.30E-01	1.00E+00	RPLP1	-2.44E-01	1.00E+00
KLHL24	-4.21E+00	6.45E-70	CLCN5	2.41E-01	1.00E+00	PELI2	2.17E-01	1.00E+00
HEMK1	-4.06E+00	6.47E-70	MVK	-1.47E-01	1.00E+00	CNIH4	1.68E-01	1.00E+00
POLG	-2.18E+00	6.78E-70	ELMO2	1.22E-01	1.00E+00	ADCY7	6.23E-01	1.00E+00
LOC102397684	-3.62E+00	7.20E-70	STAB1	-3.88E-01	1.00E+00	PQLC3	4.22E-01	1.00E+00
SYF2	1.93E+00	7.21E-70	USO1	1.49E-01	1.00E+00	HMGB3	-2.10E-01	1.00E+00
MMP23B	-5.25E+00	7.29E-70	SPICE1	1.16E-01	1.00E+00	LOC102407968	2.17E-01	1.00E+00
HAUS5	-3.33E+00	7.32E-70	LOC102415186	-1.57E-01	1.00E+00	CHRDL1	-1.94E-01	1.00E+00
NLGN1	-4.52E+00	7.78E-70	APAF1	2.08E-01	1.00E+00	PEX12	2.20E-01	1.00E+00
C1H3orf58	-4.43E+00	8.00E-70	GTF3C5	1.09E-01	1.00E+00	DPYD	2.31E-01	1.00E+00
TCAP	-5.87E+00	8.14E-70	TSTA3	-1.90E-01	1.00E+00	C9H19orf71	2.21E-01	1.00E+00
LEXM	5.60E+00	8.18E-70	MED25	1.21E-01	1.00E+00	MLEC	-1.96E-01	1.00E+00
SESTD1	2.07E+00	8.59E-70	ZNHIT1	-2.11E-01	1.00E+00	DCHS2	2.20E-01	1.00E+00
PTPN12	-3.28E+00	9.04E-70	HHATL	-2.48E-01	1.00E+00	LOC102393170	3.80E-01	1.00E+00
FBLN2	4.15E+00	9.11E-70	LACTBL1	-5.06E-01	1.00E+00	PPP3R1	-1.98E-01	1.00E+00
CPT1C	3.15E+00	9.18E-70	HAPLN3	6.02E-01	1.00E+00	AIF1L	2.04E-01	1.00E+00

FCHSD2	1.98E+00	1.03E-69	FGB	-3.86E-01	1.00E+00	C10H6orf120	-1.94E-01	1.00E+00
UGGT1	-2.73E+00	1.05E-69	LOC112581596	4.41E-01	1.00E+00	KIF13B	-1.63E-01	1.00E+00
RRP1B	1.52E+00	1.07E-69	CDPF1	-3.87E-01	1.00E+00	TP53BP1	-1.68E-01	1.00E+00
NFRKB	2.15E+00	1.07E-69	ZYG11A	2.10E-01	1.00E+00	RNF125	2.99E-01	1.00E+00
BORCS5	2.01E+00	1.15E-69	NFIA	-3.54E-01	1.00E+00	ZBTB33	2.22E-01	1.00E+00
DCAF10	-3.52E+00	1.18E-69	LOC102415514	-1.83E-01	1.00E+00	IRX1	4.57E-01	1.00E+00
UBE2Q2	1.69E+00	1.27E-69	LOC102406631	-3.90E-01	1.00E+00	GIT2	1.88E-01	1.00E+00
LOC102402536	-5.47E+00	1.38E-69	EXO5	2.83E-01	1.00E+00	KIF1BP	-1.67E-01	1.00E+00
WDFY4	-5.42E+00	1.40E-69	LOC102400922	-4.92E-01	1.00E+00	LOC102411053	3.54E-01	1.00E+00
WNT7A	7.82E+00	1.40E-69	LOC112579564	-3.70E-01	1.00E+00	SAMD14	3.75E-01	1.00E+00
CLN6	-3.21E+00	1.52E-69	PAPOLG	1.31E-01	1.00E+00	CAVIN2	-3.87E-01	1.00E+00
RPS3A	-4.70E+00	1.54E-69	LOC102401452	-8.24E-01	1.00E+00	LOC102401727	3.88E-01	1.00E+00
DNAJC14	1.58E+00	1.73E-69	LOC102415974	1.85E-01	1.00E+00	PRKAA2	-2.68E-01	1.00E+00
PSMB10	-4.04E+00	1.82E-69	LOC102406024	1.82E-01	1.00E+00	CDC25C	-2.42E-01	1.00E+00
PPP4R3B	2.27E+00	1.82E-69	MLST8	2.35E-01	1.00E+00	LOC102403870	-3.59E-01	1.00E+00
PPCS	1.91E+00	1.92E-69	TXNDC9	1.42E-01	1.00E+00	LOC102416255	-7.79E-01	1.00E+00
BRD9	-2.35E+00	2.07E-69	ZDHHC20	2.08E-01	1.00E+00	BRCA1	-1.91E-01	1.00E+00
DSE	-2.17E+00	2.23E-69	ZBTB16	1.94E-01	1.00E+00	LOC102402326	5.45E-01	1.00E+00
GRIN3B	4.93E+00	2.40E-69	CNTROB	1.22E-01	1.00E+00	SLC22A16	-2.05E-01	1.00E+00
PGBD1	-3.18E+00	2.44E-69	PDK2	1.57E-01	1.00E+00	LOC102415962	-2.97E-01	1.00E+00
LOC112578173	6.34E+00	2.45E-69	LOC112579984	-9.20E-01	1.00E+00	LUM	3.85E-01	1.00E+00
ST3GAL4	-6.56E+00	2.60E-69	EPN2	1.40E-01	1.00E+00	TRIM56	1.73E-01	1.00E+00
LETM2	2.71E+00	2.61E-69	LYPD4	4.65E-01	1.00E+00	LOC102409453	2.35E-01	1.00E+00
IFT88	1.59E+00	2.70E-69	PSMB7	-1.46E-01	1.00E+00	FAM43B	4.62E-01	1.00E+00
LOC112585537	6.39E+00	3.19E-69	RIPOR2	3.56E-01	1.00E+00	ETV1	4.70E-01	1.00E+00
ITPR2	-3.16E+00	3.39E-69	BOP1	1.62E-01	1.00E+00	NOP16	1.98E-01	1.00E+00
ENGASE	-4.94E+00	3.47E-69	SASH1	8.56E-01	1.00E+00	CIAPIN1	1.54E-01	1.00E+00
KCNMB3	-3.95E+00	3.50E-69	LOC112586254	-2.07E-01	1.00E+00	LOC102403559	-1.85E-01	1.00E+00
RPS17	-4.66E+00	3.53E-69	LIN7C	2.85E-01	1.00E+00	TMC6	1.69E-01	1.00E+00
ELOB	-3.18E+00	3.57E-69	ATXN7	1.16E-01	1.00E+00	TCERG1	-1.74E-01	1.00E+00
E2F1	2.38E+00	3.59E-69	VPS33B	2.42E-01	1.00E+00	USPL1	-2.22E-01	1.00E+00
PROM2	6.40E+00	3.69E-69	XPO1	1.62E-01	1.00E+00	LOC112582330	3.60E-01	1.00E+00
RPL13	-4.03E+00	4.17E-69	TIMMDC1	-2.86E-01	1.00E+00	SRRM1	-1.62E-01	1.00E+00
SLITRK6	5.24E+00	4.40E-69	AMFR	1.98E-01	1.00E+00	NGRN	1.73E-01	1.00E+00

USPL1	-4.03E+00	4.45E-69	SS18L2	-1.65E-01	1.00E+00	HR	3.82E-01	1.00E+00
SNN	-2.00E+00	4.50E-69	SLC35E1	1.51E-01	1.00E+00	LOC102405773	-3.11E-01	1.00E+00
LOC102414676	7.37E+00	4.54E-69	SOSTDC1	-5.24E-01	1.00E+00	ALDH5A1	-2.89E-01	1.00E+00
RUNX1T1	7.11E+00	5.19E-69	RPL4	-1.46E-01	1.00E+00	SLC35B3	-1.77E-01	1.00E+00
FER	2.30E+00	5.28E-69	NUDT4	1.31E-01	1.00E+00	LOC112578959	-2.14E-01	1.00E+00
LIPA	-1.98E+00	5.30E-69	ICAM5	-4.24E-01	1.00E+00	RAB34	1.85E-01	1.00E+00
NAPSA	6.19E+00	5.31E-69	LOC112585042	-2.56E-01	1.00E+00	DYNLL1	1.68E-01	1.00E+00
SCRIB	-1.94E+00	5.69E-69	ATP6AP1	1.26E-01	1.00E+00	SLC4A8	-1.98E-01	1.00E+00
CRHBP	8.05E+00	5.71E-69	MMP15	1.77E-01	1.00E+00	SLC9B2	3.71E-01	1.00E+00
LOC102410118	-3.92E+00	5.71E-69	PACRGL	-2.51E-01	1.00E+00	VGLL4	1.87E-01	1.00E+00
MAFG	2.66E+00	5.75E-69	MICU1	-1.66E-01	1.00E+00	MACROD1	-2.80E-01	1.00E+00
DDAH2	-2.44E+00	5.92E-69	ERCC1	-2.19E-01	1.00E+00	ILK	1.62E-01	1.00E+00
ALS2CL	-3.78E+00	6.89E-69	GPRC5C	2.02E-01	1.00E+00	LOC102414462	2.80E-01	1.00E+00
LOC112578878	-4.19E+00	6.91E-69	PLPPR5	-5.95E-01	1.00E+00	LOC102393576	-4.49E-01	1.00E+00
RACK1	-3.49E+00	7.13E-69	SETD2	-3.03E-01	1.00E+00	NEURL2	2.33E-01	1.00E+00
HSPD1	-2.32E+00	7.15E-69	LOC102402934	1.39E-01	1.00E+00	CCNA1	-3.62E-01	1.00E+00
TGFB1I1	-5.80E+00	7.69E-69	RARRES1	1.45E-01	1.00E+00	LOC112581794	4.09E-01	1.00E+00
FDXR	-5.97E+00	8.00E-69	NUSAP1	1.40E-01	1.00E+00	CTSK	4.20E-01	1.00E+00
ZMIZ1	-5.23E+00	8.19E-69	LOC112579702	-7.75E-01	1.00E+00	RBM39	-1.86E-01	1.00E+00
SUGT1	2.17E+00	8.65E-69	ZNF385D	4.80E-01	1.00E+00	HIF1A	-2.74E-01	1.00E+00
DIAPH1	1.87E+00	8.82E-69	ZNF182	1.83E-01	1.00E+00	CPSF6	-1.71E-01	1.00E+00
G6PC3	-2.88E+00	9.43E-69	PITX2	-2.89E-01	1.00E+00	WDR19	-1.78E-01	1.00E+00
PSME1	-2.88E+00	1.01E-68	ARHGEF25	3.90E-01	1.00E+00	DPP9	1.60E-01	1.00E+00
ANO8	-6.53E+00	1.03E-68	CWC15	-1.26E-01	1.00E+00	FAM200A	-3.64E-01	1.00E+00
A1CF	9.09E+00	1.06E-68	LOC102401106	-2.98E-01	1.00E+00	NDC80	-1.92E-01	1.00E+00
DKKL1	-5.97E+00	1.14E-68	ZNF397	-2.16E-01	1.00E+00	PLEKHM2	1.50E-01	1.00E+00
BZW1	-1.82E+00	1.19E-68	CALML4	-4.56E-01	1.00E+00	ACIN1	-1.91E-01	1.00E+00
NDUFS8	-3.03E+00	1.23E-68	STAU1	1.30E-01	1.00E+00	FAM173B	-1.63E-01	1.00E+00
LOC112579873	-7.57E+00	1.29E-68	ZDHHC4	1.96E-01	1.00E+00	MARVELD3	3.32E-01	1.00E+00
LOC102396420	8.62E+00	1.53E-68	CACNA1D	2.21E-01	1.00E+00	ZNF672	2.34E-01	1.00E+00
WASHC1	2.21E+00	1.54E-68	ACAP1	-6.14E-01	1.00E+00	ARMH4	-1.59E-01	1.00E+00
FXYD3	-7.37E+00	1.56E-68	FBXW2	1.40E-01	1.00E+00	ACTRT3	-2.66E-01	1.00E+00
PTGFRN	-2.76E+00	1.81E-68	EFCAB6	-4.38E-01	1.00E+00	LOC102389840	-4.46E-01	1.00E+00

RPL36	-3.90E+00	1.87E-68	HRH1	1.48E-01	1.00E+00	MICAL1	1.95E-01	1.00E+00
ACAT1	-2.23E+00	1.87E-68	NSMCE1	-1.47E-01	1.00E+00	LOC112578778	3.47E-01	1.00E+00
NFAT5	-3.45E+00	1.97E-68	RIPOR3	-4.44E-01	1.00E+00	IKBIP	-1.47E-01	1.00E+00
GAA	-1.85E+00	2.03E-68	TESK2	-1.84E-01	1.00E+00	GDPGP1	1.86E-01	1.00E+00
MAN2A2	-3.71E+00	2.22E-68	MIGA2	1.33E-01	1.00E+00	RNF26	-1.79E-01	1.00E+00
ADAP1	5.48E+00	2.22E-68	ABHD17A	1.11E-01	1.00E+00	UNC13C	2.09E-01	1.00E+00
PHYKPL	-4.88E+00	2.30E-68	SDK1	-1.10E-01	1.00E+00	ZNF784	2.50E-01	1.00E+00
ARMT1	-3.36E+00	2.30E-68	CTNNBL1	-1.25E-01	1.00E+00	PES1	1.93E-01	1.00E+00
CNN2	-5.44E+00	2.31E-68	COL26A1	5.08E-01	1.00E+00	PRKAR2B	-2.09E-01	1.00E+00
CEP290	-2.94E+00	2.40E-68	MYD88	1.72E-01	1.00E+00	TTC9	3.86E-01	1.00E+00
PPP1R3F	2.48E+00	2.52E-68	FDXACB1	3.44E-01	1.00E+00	MYADM	-2.41E-01	1.00E+00
CHST15	-6.12E+00	2.61E-68	IRS1	-2.36E-01	1.00E+00	KRCC1	-1.79E-01	1.00E+00
PSMC3	-2.92E+00	2.74E-68	STXBP3	1.34E-01	1.00E+00	KCTD11	2.21E-01	1.00E+00
C22H18orf25	1.66E+00	2.81E-68	PXN	1.43E-01	1.00E+00	NGLY1	-1.48E-01	1.00E+00
RBM18	1.76E+00	2.88E-68	CPT1C	-2.32E-01	1.00E+00	PRRG1	2.24E-01	1.00E+00
LOC102416301	-4.17E+00	2.89E-68	ZNF213	-4.88E-01	1.00E+00	ZP3	-6.14E-01	1.00E+00
ZMYM3	-2.24E+00	3.13E-68	CDCA8	1.20E-01	1.00E+00	ITGA4	-2.61E-01	1.00E+00
DPYD	-4.55E+00	3.20E-68	LOC112585078	-2.69E-01	1.00E+00	MXRA5	3.62E-01	1.00E+00
PIN1	-2.84E+00	3.24E-68	TOMM22	1.55E-01	1.00E+00	SMC3	-1.50E-01	1.00E+00
FAM177A1	-3.03E+00	3.27E-68	CD74	-3.94E-01	1.00E+00	LOC102414630	-4.34E-01	1.00E+00
LOC102400625	-6.75E+00	3.39E-68	TMEM218	-2.41E-01	1.00E+00	BOC	3.75E-01	1.00E+00
GTPBP3	-5.86E+00	3.39E-68	RASAL3	-7.37E-01	1.00E+00	ZNF473	-2.83E-01	1.00E+00
RPL18A	-5.18E+00	3.46E-68	OTUD3	1.21E-01	1.00E+00	LOC112585766	-3.31E-01	1.00E+00
LOC102392697	3.77E+00	3.77E-68	LOC102397524	-4.40E-01	1.00E+00	ZDHHC15	2.28E-01	1.00E+00
PKM	-3.05E+00	3.82E-68	LOC112587945	4.93E-01	1.00E+00	RUNX3	5.47E-01	1.00E+00
LOC112579680	7.82E+00	4.22E-68	FAM57A	-3.45E-01	1.00E+00	KCNMB3	1.86E-01	1.00E+00
GORASP1	1.57E+00	4.38E-68	GLT1D1	4.78E-01	1.00E+00	RPS13	-2.15E-01	1.00E+00
LARP4B	1.68E+00	4.49E-68	CCDC51	1.94E-01	1.00E+00	SLC10A7	-1.60E-01	1.00E+00
LOC112579728	6.93E+00	4.57E-68	USP19	1.40E-01	1.00E+00	ANKRD29	3.34E-01	1.00E+00
CPA6	8.71E+00	4.72E-68	LOC112580425	2.08E-01	1.00E+00	LOC102400859	3.95E-01	1.00E+00
RNF149	-3.32E+00	4.73E-68	LOC112580203	-3.33E-01	1.00E+00	FMN1	3.58E-01	1.00E+00
SDHB	-3.44E+00	4.89E-68	GBX1	-2.80E-01	1.00E+00	ALOX5	-4.77E-01	1.00E+00
P3H3	-4.78E+00	5.04E-68	KLF12	1.28E-01	1.00E+00	NIPSNAP1	-1.62E-01	1.00E+00

ACADVL	-2.38E+00	5.67E-68	ANXA5	-1.46E-01	1.00E+00	NKAPD1	-1.60E-01	1.00E+00
MTCH1	-1.65E+00	5.69E-68	SMIM7	-1.65E-01	1.00E+00	CUBN	4.07E-01	1.00E+00
FSTL4	5.10E+00	5.86E-68	LOC102403497	-2.23E-01	1.00E+00	ASTL	-6.54E-01	1.00E+00
PARP10	-3.39E+00	6.03E-68	RB1	4.23E-01	1.00E+00	LLGL1	-1.60E-01	1.00E+00
APMAP	-2.04E+00	6.26E-68	TAF15	-1.91E-01	1.00E+00	RHPN2	3.60E-01	1.00E+00
FLYWCH1	-6.10E+00	6.37E-68	CHRM2	1.13E-01	1.00E+00	CCL27	-2.47E-01	1.00E+00
SUCLG1	-2.39E+00	6.47E-68	SPINT2	1.25E-01	1.00E+00	ASB3	-1.86E-01	1.00E+00
NMI	-5.71E+00	6.68E-68	LOC102390734	-3.00E-01	1.00E+00	KLHL11	1.75E-01	1.00E+00
SERPINA1	-8.03E+00	6.78E-68	CBX5	1.69E-01	1.00E+00	SLC25A17	1.81E-01	1.00E+00
XG	-6.32E+00	6.87E-68	TBL2	1.35E-01	1.00E+00	CXADR	1.93E-01	1.00E+00
SPNS1	-1.81E+00	6.90E-68	LOC102394468	1.38E-01	1.00E+00	TINF2	-1.82E-01	1.00E+00
VCP	-2.04E+00	7.30E-68	SPTLC2	1.49E-01	1.00E+00	FARP1	1.40E-01	1.00E+00
SMPD3	3.40E+00	7.60E-68	PKNOX1	-1.87E-01	1.00E+00	NIPAL3	-1.69E-01	1.00E+00
SLC39A13	-2.03E+00	7.72E-68	GRIN3A	-6.41E-01	1.00E+00	KDELR1	-1.72E-01	1.00E+00
LDLR	-5.60E+00	7.76E-68	SCAPER	1.66E-01	1.00E+00	SEC22B	-1.52E-01	1.00E+00
CNPY1	7.02E+00	7.76E-68	PIAS2	1.14E-01	1.00E+00	RSBN1	2.65E-01	1.00E+00
PCSK7	-1.83E+00	7.87E-68	PI4K2B	2.32E-01	1.00E+00	RAI2	3.67E-01	1.00E+00
RAB31	-3.72E+00	8.17E-68	EGFL8	-2.42E-01	1.00E+00	ASH2L	-1.53E-01	1.00E+00
TMOD2	4.26E+00	8.19E-68	TNK1	2.34E-01	1.00E+00	LIG3	-1.49E-01	1.00E+00
FLYWCH2	-3.92E+00	8.47E-68	LOC112587835	3.65E-01	1.00E+00	DNAAF5	-1.70E-01	1.00E+00
RPL39	-3.27E+00	9.39E-68	SYT6	-5.57E-01	1.00E+00	ARID4A	-2.24E-01	1.00E+00
ADAD2	4.36E+00	9.65E-68	LOC112587841	2.09E-01	1.00E+00	SETDB1	-1.58E-01	1.00E+00
KIAA0586	1.86E+00	9.91E-68	MIP	-3.09E-01	1.00E+00	UST	1.71E-01	1.00E+00
PPIB	-3.23E+00	1.04E-67	RPS26	-1.71E-01	1.00E+00	MGME1	-1.88E-01	1.00E+00
C2H6orf141	-6.07E+00	1.07E-67	ANKRD33B	6.04E-01	1.00E+00	OTUD7B	1.45E-01	1.00E+00
SGSM2	2.00E+00	1.09E-67	AARD	1.49E-01	1.00E+00	C6H1orf216	3.73E-01	1.00E+00
C2H6orf62	-1.81E+00	1.12E-67	WDR43	1.41E-01	1.00E+00	ADAM17	-1.47E-01	1.00E+00
KALRN	2.75E+00	1.13E-67	MTR	-1.55E-01	1.00E+00	ARSA	-2.03E-01	1.00E+00
ASCC2	-2.02E+00	1.15E-67	RAB1B	1.12E-01	1.00E+00	INPP5B	-1.69E-01	1.00E+00
LOC102398226	-6.90E+00	1.26E-67	ARPP19	2.07E-01	1.00E+00	PSD3	-1.81E-01	1.00E+00
THOC1	1.66E+00	1.28E-67	TRIM23	2.33E-01	1.00E+00	CUL2	1.62E-01	1.00E+00
LHX3	5.36E+00	1.32E-67	NTN1	1.31E-01	1.00E+00	CCDC148	4.42E-01	1.00E+00
ANXA4	-5.13E+00	1.38E-67	HSD17B7	-6.12E-01	1.00E+00	HSPA2	-1.62E-01	1.00E+00
LMNB2	-	1.38E-67	NUP107	-1.16E-	1.00E+00	METAP1	-1.70E-	1.00E+00

	2.32E+00			01			01	
ODC1	2.77E+00	1.64E-67	C4H12orf66	2.07E-01	1.00E+00	LMOD1	3.61E-01	1.00E+00
CCDC92	2.03E+00	1.70E-67	LOC112586095	-2.49E-01	1.00E+00	LOC112582099	-3.62E-01	1.00E+00
HSPB1	-6.61E+00	1.90E-67	TLL2	5.35E-01	1.00E+00	GTDC1	-1.55E-01	1.00E+00
SREK1IP1	2.14E+00	1.90E-67	ZNF106	1.92E-01	1.00E+00	SLC31A1	-1.71E-01	1.00E+00
LOC112586812	6.46E+00	1.93E-67	TNKS	1.60E-01	1.00E+00	IRS2	2.51E-01	1.00E+00
MEST	2.32E+00	1.99E-67	NAA30	1.41E-01	1.00E+00	LOC102400533	3.59E-01	1.00E+00
ZNF286A	1.80E+00	2.00E-67	LOC102390371	-4.48E-01	1.00E+00	ZCCHC24	2.35E-01	1.00E+00
EXOG	2.11E+00	2.12E-67	RAB10	1.18E-01	1.00E+00	KLHL29	4.08E-01	1.00E+00
FAM171A1	-3.74E+00	2.29E-67	SRRM4	-1.68E-01	1.00E+00	ARFRP1	1.86E-01	1.00E+00
ARIH2	-1.56E+00	2.30E-67	TRAPPC3	-1.67E-01	1.00E+00	SPATA18	3.91E-01	1.00E+00
C1H3orf38	1.63E+00	2.36E-67	SLC26A5	-3.95E-01	1.00E+00	LRRC73	3.43E-01	1.00E+00
VWA5A	-7.33E+00	2.42E-67	LOC112580240	1.98E-01	1.00E+00	LOC112586500	3.74E-01	1.00E+00
LOC102392001	-4.83E+00	2.54E-67	STRIP2	1.23E-01	1.00E+00	SLCO4A1	4.22E-01	1.00E+00
LRP5	-6.04E+00	2.57E-67	SEPT3	1.48E-01	1.00E+00	ARHGAP29	2.19E-01	1.00E+00
OVCH1	2.06E+00	2.79E-67	VWA7	3.59E-01	1.00E+00	PLXNA1	-1.82E-01	1.00E+00
METTL16	-2.42E+00	2.79E-67	MEF2A	-2.75E-01	1.00E+00	BAIAP3	-3.43E-01	1.00E+00
CCDC91	-1.71E+00	2.83E-67	OGT	-1.36E-01	1.00E+00	LOC112578482	3.49E-01	1.00E+00
REEP3	2.25E+00	2.89E-67	LOC102395838	-4.31E-01	1.00E+00	LOC102405836	3.19E-01	1.00E+00
LOC112580203	6.30E+00	3.06E-67	TRIAP1	-1.33E-01	1.00E+00	CPD	-1.94E-01	1.00E+00
PSMD9	1.78E+00	3.17E-67	LOC102410605	-2.14E-01	1.00E+00	E2F6	1.75E-01	1.00E+00
RPL10	-3.61E+00	3.22E-67	LOC112587762	2.90E-01	1.00E+00	SAMSN1	-6.06E-01	1.00E+00
NDE1	-2.92E+00	3.55E-67	CACNA1H	-3.83E-01	1.00E+00	SLC25A33	-2.20E-01	1.00E+00
TRAF2	2.03E+00	3.59E-67	LOC102403883	-5.80E-01	1.00E+00	LDLRAD3	-1.46E-01	1.00E+00
LOC112578454	-2.38E+00	3.62E-67	TRMT13	3.46E-01	1.00E+00	GJC1	1.68E-01	1.00E+00
LOC112579960	7.59E+00	3.93E-67	MIPOL1	2.25E-01	1.00E+00	FNDC3B	-2.21E-01	1.00E+00
CGAS	2.52E+00	3.93E-67	ATG14	1.43E-01	1.00E+00	TOP2A	-2.09E-01	1.00E+00
SMARCA1	-4.86E+00	3.95E-67	MFN2	1.32E-01	1.00E+00	NETO2	2.30E-01	1.00E+00
BSG	-2.95E+00	3.95E-67	TMEM184C	1.19E-01	1.00E+00	AMOTL1	1.88E-01	1.00E+00
LARGE2	-6.84E+00	3.98E-67	RASGEF1B	-3.57E-01	1.00E+00	FBF1	-1.48E-01	1.00E+00
TNNI3	-6.98E+00	4.08E-67	LOC102416352	-1.26E-01	1.00E+00	FARSB	1.89E-01	1.00E+00
MCL1	-3.22E+00	4.20E-67	AREL1	1.70E-01	1.00E+00	ANKRA2	-1.69E-01	1.00E+00
CDH15	6.04E+00	4.36E-67	PRDM1	2.48E-01	1.00E+00	HEPH	3.90E-01	1.00E+00
GCNT3	4.59E+00	4.56E-67	SLC52A2	1.29E-01	1.00E+00	STRBP	-1.50E-01	1.00E+00

EFR3B	-5.91E+00	4.65E-67	LSM6	-2.00E-01	1.00E+00	DNAJB2	1.64E-01	1.00E+00
SLC5A2	-3.77E+00	4.66E-67	PDXP	-1.49E-01	1.00E+00	LOC112577795	2.04E-01	1.00E+00
RPL11	-3.84E+00	4.68E-67	TOX3	2.57E-01	1.00E+00	MGST2	-3.07E-01	1.00E+00
SGPP1	-3.72E+00	5.04E-67	TIAM2	1.37E-01	1.00E+00	TSSC4	2.12E-01	1.00E+00
LOC102391376	-3.00E+00	5.10E-67	RORC	-4.42E-01	1.00E+00	TJP2	2.24E-01	1.00E+00
HS3ST5	-4.27E+00	5.12E-67	FRAT1	-4.06E-01	1.00E+00	SDHAF1	2.36E-01	1.00E+00
FBF1	-2.38E+00	5.42E-67	LOC102415115	5.22E-01	1.00E+00	DEPDC1B	-2.09E-01	1.00E+00
IGFBP5	-8.31E+00	5.42E-67	AKIP1	2.66E-01	1.00E+00	BNIP3	1.80E-01	1.00E+00
LOC102415603	-5.19E+00	5.74E-67	BLOC1S3	1.54E-01	1.00E+00	SLC26A8	3.03E-01	1.00E+00
CENPA	-3.13E+00	6.05E-67	GOLPH3	1.38E-01	1.00E+00	TM7SF3	-1.62E-01	1.00E+00
RPL10A	-4.14E+00	6.37E-67	LMTK2	2.89E-01	1.00E+00	CDK2	-1.72E-01	1.00E+00
HS6ST2	-5.07E+00	6.57E-67	STX18	-2.09E-01	1.00E+00	LMAN1	-1.62E-01	1.00E+00
LOC112586499	5.14E+00	6.91E-67	CAMK2D	1.48E-01	1.00E+00	STRIP2	-3.32E-01	1.00E+00
LOC102399344	2.08E+00	6.96E-67	PTPN18	-3.21E-01	1.00E+00	SLC25A29	1.71E-01	1.00E+00
TM9SF1	-1.99E+00	7.00E-67	LOC102405595	3.98E-01	1.00E+00	DCTN5	1.51E-01	1.00E+00
EVC2	-3.89E+00	7.17E-67	MAP3K21	3.13E-01	1.00E+00	ATP6V1A	2.17E-01	1.00E+00
EPC2	-1.93E+00	7.61E-67	IGF1R	1.28E-01	1.00E+00	ALDH3A2	-2.19E-01	1.00E+00
FDX1	1.86E+00	9.23E-67	LPGAT1	2.90E-01	1.00E+00	MAP4K2	-1.88E-01	1.00E+00
EMP3	-5.76E+00	9.26E-67	SDR39U1	-3.82E-01	1.00E+00	PPP1R2	1.66E-01	1.00E+00
SPG7	-2.13E+00	9.26E-67	PRKX	2.40E-01	1.00E+00	HSP90AA1	-2.46E-01	1.00E+00
POMT1	-2.34E+00	9.30E-67	KIAA0895	1.36E-01	1.00E+00	ZNRF2	1.88E-01	1.00E+00
LOC102403983	-1.60E+00	9.72E-67	POM121C	1.61E-01	1.00E+00	POLE	-1.75E-01	1.00E+00
DNAJC27	2.14E+00	9.90E-67	TENT5A	-4.83E-01	1.00E+00	ENO4	-2.68E-01	1.00E+00
AHCYL2	2.21E+00	9.96E-67	LOC102416007	-2.41E-01	1.00E+00	LOC102406498	4.10E-01	1.00E+00
MAML2	-4.69E+00	1.05E-66	LOC102409414	2.40E-01	1.00E+00	SLC19A2	-1.86E-01	1.00E+00
WDR3	2.38E+00	1.10E-66	MAP4K5	1.33E-01	1.00E+00	GCLC	-2.53E-01	1.00E+00
PSMD8	-2.47E+00	1.30E-66	KDM4A	1.28E-01	1.00E+00	ZNF827	-1.72E-01	1.00E+00
ESPL1	2.68E+00	1.35E-66	LOC102412762	-5.20E-01	1.00E+00	LRRFIP1	2.14E-01	1.00E+00
TMEM30A	-1.83E+00	1.39E-66	CENPM	1.38E-01	1.00E+00	LIMS2	2.62E-01	1.00E+00
RPL37A	-4.32E+00	1.45E-66	AP1B1	1.20E-01	1.00E+00	ZNF132	-2.10E-01	1.00E+00
TCTN1	1.72E+00	1.58E-66	PLXND1	2.14E-01	1.00E+00	FOSB	-5.91E-01	1.00E+00
ORAI1	2.36E+00	1.62E-66	ZNF45	1.68E-01	1.00E+00	UBXN2A	2.31E-01	1.00E+00
NEU1	-	1.63E-66	LOC112582191	-3.34E-	1.00E+00	TDP1	-1.71E-	1.00E+00

	2.89E+00			01			01	
HIF1A	-3.86E+00	1.64E-66	BSCL2	1.45E-01	1.00E+00	SLC2A12	3.95E-01	1.00E+00
ARAP2	6.01E+00	1.69E-66	SLC38A7	1.92E-01	1.00E+00	TTL	1.80E-01	1.00E+00
LOC112580815	3.36E+00	1.84E-66	LTF	-3.80E-01	1.00E+00	COL6A3	4.03E-01	1.00E+00
SLC37A1	-6.19E+00	1.85E-66	MN1	4.09E-01	1.00E+00	ND1	-3.07E-01	1.00E+00
HSD3B7	-6.51E+00	2.04E-66	ERC1	1.47E-01	1.00E+00	RBPJ	1.73E-01	1.00E+00
LOC112586799	6.15E+00	2.05E-66	NRCAM	-5.56E-01	1.00E+00	SNTB1	3.39E-01	1.00E+00
LOC112581150	5.24E+00	2.10E-66	LOC102390435	5.30E-01	1.00E+00	MICU1	1.40E-01	1.00E+00
FAM213B	-5.67E+00	2.54E-66	LOC112586935	-4.86E-01	1.00E+00	ERG28	-1.82E-01	1.00E+00
DENND6B	-3.03E+00	2.58E-66	HOXA5	-7.55E-01	1.00E+00	TBC1D16	2.08E-01	1.00E+00
LOC112580369	6.33E+00	2.61E-66	RHBDF2	1.89E-01	1.00E+00	CLEC11A	-3.71E-01	1.00E+00
TPBG	-6.86E+00	2.63E-66	HOXC6	-5.78E-01	1.00E+00	AKAIN1	3.28E-01	1.00E+00
IFNAR1	-4.03E+00	2.73E-66	LOC102405452	1.32E-01	1.00E+00	KYAT1	-2.29E-01	1.00E+00
TTC4	1.84E+00	2.87E-66	SERGEF	-4.25E-01	1.00E+00	SCOC	1.65E-01	1.00E+00
ADCY6	-4.28E+00	2.90E-66	MYO7B	-7.13E-01	1.00E+00	LOC112581723	-3.50E-01	1.00E+00
DIP2A	-3.09E+00	2.92E-66	EXTL1	-4.07E-01	1.00E+00	TMEM119	3.82E-01	1.00E+00
PXN	1.87E+00	3.00E-66	MAX	2.09E-01	1.00E+00	LOC102392178	3.26E-01	1.00E+00
USF1	-2.77E+00	3.38E-66	CCNB1	1.23E-01	1.00E+00	CCDC3	-2.49E-01	1.00E+00
LOC102398329	4.93E+00	3.40E-66	KHDRBS2	-3.65E-01	1.00E+00	UCHL1	1.99E-01	1.00E+00
GSS	-3.08E+00	3.42E-66	TCF3	1.22E-01	1.00E+00	LOC112581357	3.75E-01	1.00E+00
LIMS1	-4.15E+00	4.04E-66	MAST4	1.58E-01	1.00E+00	GORAB	-1.89E-01	1.00E+00
LOC112586244	6.90E+00	4.05E-66	PREB	1.53E-01	1.00E+00	PEG10	-1.85E-01	1.00E+00
KIF21B	-6.21E+00	4.07E-66	FAM3A	-3.79E-01	1.00E+00	ADGRL1	1.90E-01	1.00E+00
BLOC1S3	2.09E+00	4.33E-66	NEIL1	-2.70E-01	1.00E+00	RPS29	-2.32E-01	1.00E+00
HAPLN4	5.46E+00	4.46E-66	COPG1	1.31E-01	1.00E+00	OSBPL10	1.82E-01	1.00E+00
KLHL20	1.97E+00	4.76E-66	C15H8orf59	-2.13E-01	1.00E+00	CC2D2B	-3.53E-01	1.00E+00
RPS23	-3.78E+00	4.89E-66	ZNF567	5.65E-01	1.00E+00	FAM13C	-3.89E-01	1.00E+00
TDG	-4.73E+00	4.97E-66	CHUK	1.41E-01	1.00E+00	SLC38A4	4.30E-01	1.00E+00
PCK2	-4.63E+00	5.09E-66	PPIA	-1.55E-01	1.00E+00	PTGES	2.82E-01	1.00E+00
STAT3	2.70E+00	5.67E-66	ARG2	-1.74E-01	1.00E+00	IL4R	2.56E-01	1.00E+00
IGFBP6	-4.98E+00	6.27E-66	BMP1	-3.43E-01	1.00E+00	IARS	-1.50E-01	1.00E+00
LOC112579839	3.38E+00	6.51E-66	HNRNPC	-1.08E-01	1.00E+00	TSPAN5	1.70E-01	1.00E+00
NKX1-1	7.00E+00	6.59E-66	CCNT1	2.01E-01	1.00E+00	LEF1	3.08E-01	1.00E+00
LOC112585027	5.90E+00	6.81E-66	AAK1	1.96E-01	1.00E+00	ZCCHC12	2.83E-01	1.00E+00
ST3GAL3	-	6.83E-66	LOC102407124	-1.07E-	1.00E+00	PMPCB	-1.39E-	1.00E+00

	2.03E+00			01			01	
PTPRF	-4.50E+00	7.18E-66	LOC112585150	-2.02E-01	1.00E+00	KIAA1257	-2.09E-01	1.00E+00
ABT1	1.56E+00	7.49E-66	INPP5F	1.39E-01	1.00E+00	SPIN4	-3.50E-01	1.00E+00
MTHFD2L	-5.54E+00	7.49E-66	RC3H1	2.40E-01	1.00E+00	AP2A1	-1.45E-01	1.00E+00
ACSS1	-4.55E+00	7.78E-66	SLC4A9	-7.58E-01	1.00E+00	PSTPIP1	5.57E-01	1.00E+00
RPL29	-4.19E+00	7.86E-66	PER2	2.50E-01	1.00E+00	CCDC15	-1.74E-01	1.00E+00
CCK	5.55E+00	8.12E-66	GOSR2	-1.44E-01	1.00E+00	UNK	-1.63E-01	1.00E+00
PLEKHA7	1.93E+00	8.20E-66	UNC93B1	-8.30E-01	1.00E+00	AGBL3	-3.16E-01	1.00E+00
SYCP2	6.66E+00	8.55E-66	RGR	-4.93E-01	1.00E+00	CLU	2.70E-01	1.00E+00
PYCR3	-3.51E+00	8.60E-66	GJB1	5.13E-01	1.00E+00	LOC102389794	-3.23E-01	1.00E+00
TSEN54	-2.42E+00	8.76E-66	SHROOM1	3.84E-01	1.00E+00	NUDT4	1.77E-01	1.00E+00
B4GALNT3	6.08E+00	8.81E-66	LOC102408258	-2.41E-01	1.00E+00	LAS1L	-1.59E-01	1.00E+00
LOC112578381	-5.48E+00	8.89E-66	EMC9	-3.45E-01	1.00E+00	RUNX1	2.11E-01	1.00E+00
FMN1	3.41E+00	9.07E-66	FCRLA	-7.54E-01	1.00E+00	ABCG2	-2.05E-01	1.00E+00
DES	-6.65E+00	9.30E-66	THEG	2.01E-01	1.00E+00	RACGAP1	-1.61E-01	1.00E+00
SDSL	4.84E+00	1.06E-65	DVL1	1.53E-01	1.00E+00	SHANK2	3.93E-01	1.00E+00
KAT2A	1.88E+00	1.08E-65	SAV1	-4.26E-01	1.00E+00	RILPL1	1.41E-01	1.00E+00
LRPPRC	-1.65E+00	1.10E-65	XRCC1	1.39E-01	1.00E+00	MYRIP	3.67E-01	1.00E+00
CLEC3B	-8.16E+00	1.15E-65	LIN7A	1.45E-01	1.00E+00	ZFHX3	-2.34E-01	1.00E+00
INTS8	-1.58E+00	1.16E-65	SHF	-4.27E-01	1.00E+00	CARHSP1	-2.09E-01	1.00E+00
PJA1	-2.31E+00	1.21E-65	NOCT	1.72E-01	1.00E+00	TEPSIN	-1.51E-01	1.00E+00
BTLA	-3.55E+00	1.21E-65	SCIN	-3.19E-01	1.00E+00	WDR59	-1.74E-01	1.00E+00
TRAPPC8	2.17E+00	1.22E-65	LOC112578841	8.21E-01	1.00E+00	GNA12	1.45E-01	1.00E+00
LDLRAD3	-5.29E+00	1.23E-65	LOC112579065	-1.68E-01	1.00E+00	IFT20	-1.66E-01	1.00E+00
FSD2	3.25E+00	1.28E-65	LOC102398703	3.83E-01	1.00E+00	JAKMIP1	-4.07E-01	1.00E+00
C3H9orf64	2.03E+00	1.31E-65	ARL6IP5	-3.04E-01	1.00E+00	BCL2L13	1.39E-01	1.00E+00
TEPSIN	-1.88E+00	1.35E-65	LOC112583973	-4.89E-01	1.00E+00	LOC102403941	-3.02E-01	1.00E+00
LOC102411662	-3.32E+00	1.39E-65	VWCE	3.77E-01	1.00E+00	GLG1	-1.79E-01	1.00E+00
LOC112580153	-5.51E+00	1.39E-65	ABCG5	2.92E-01	1.00E+00	FLOT2	-1.46E-01	1.00E+00
NDRG2	-6.10E+00	1.39E-65	TST	4.01E-01	1.00E+00	SYPL1	1.76E-01	1.00E+00
FIZ1	-3.06E+00	1.40E-65	NFIC	1.57E-01	1.00E+00	PGAM5	-1.70E-01	1.00E+00
LRRN4	6.23E+00	1.46E-65	RPS11	-1.62E-01	1.00E+00	JRKL	-2.48E-01	1.00E+00
RHOQ	-5.43E+00	1.52E-65	SLC8B1	1.21E-01	1.00E+00	SH3BP5L	-1.46E-01	1.00E+00

AMDHD2	-3.84E+00	1.60E-65	KIF19	7.11E-01	1.00E+00	ZSWIM5	3.57E-01	1.00E+00
GINS3	2.24E+00	1.67E-65	TMEM87A	1.03E-01	1.00E+00	LOC112582191	-3.55E-01	1.00E+00
SH3BGRL3	-2.71E+00	1.69E-65	DRD5	-4.43E-01	1.00E+00	LOC102390255	1.60E-01	1.00E+00
SMIM1	-6.33E+00	1.76E-65	BSDC1	1.01E-01	1.00E+00	YEATS2	-2.05E-01	1.00E+00
THBS1	-4.64E+00	1.78E-65	MINDY3	1.19E-01	1.00E+00	RNF130	1.54E-01	1.00E+00
TFAM	-4.88E+00	1.79E-65	TOR1AIP1	3.32E-01	1.00E+00	CREBRF	-2.09E-01	1.00E+00
PRSS45	7.54E+00	1.98E-65	EBF4	2.13E-01	1.00E+00	SEPT10	-2.06E-01	1.00E+00
ERCC3	1.46E+00	2.07E-65	PROSER2	3.11E-01	1.00E+00	CLEC16A	1.73E-01	1.00E+00
PCBD2	-3.05E+00	2.18E-65	EP400	2.61E-01	1.00E+00	NDNF	-2.66E-01	1.00E+00
MIER1	-3.00E+00	2.20E-65	CCDC114	4.43E-01	1.00E+00	PGAP2	-1.49E-01	1.00E+00
FEZ1	-3.07E+00	2.25E-65	FZD1	4.56E-01	1.00E+00	NECAP2	1.45E-01	1.00E+00
LRRC47	-2.27E+00	2.31E-65	HOXD1	-1.82E-01	1.00E+00	OSTF1	2.44E-01	1.00E+00
LOC112579865	4.39E+00	2.80E-65	MZT1	-7.57E-01	1.00E+00	TAF7	-1.47E-01	1.00E+00
PKN1	-1.94E+00	3.10E-65	CNOT10	1.73E-01	1.00E+00	PAWR	-1.58E-01	1.00E+00
CD302	4.50E+00	3.14E-65	HID1	2.17E-01	1.00E+00	AIFM1	-1.54E-01	1.00E+00
ADRA2A	6.16E+00	3.20E-65	ZNF652	1.66E-01	1.00E+00	APBB2	1.45E-01	1.00E+00
TCTN2	-2.60E+00	3.26E-65	ZNF484	-5.59E-01	1.00E+00	TST	-2.82E-01	1.00E+00
DENR	-2.78E+00	3.33E-65	USP20	2.47E-01	1.00E+00	UCN	-2.86E-01	1.00E+00
LOC112584672	8.05E+00	3.39E-65	LAMC1	1.89E-01	1.00E+00	BAHD1	1.84E-01	1.00E+00
TOM1L1	-2.84E+00	3.44E-65	CDCA3	1.14E-01	1.00E+00	DBF4B	-2.60E-01	1.00E+00
ARF5	2.57E+00	3.77E-65	SBK1	-2.00E-01	1.00E+00	HCFC1R1	2.13E-01	1.00E+00
PARP3	-5.72E+00	3.78E-65	LOC112581592	3.08E-01	1.00E+00	SDC3	3.18E-01	1.00E+00
HDDC2	-2.69E+00	3.84E-65	LOC112581131	-4.87E-01	1.00E+00	PRADC1	2.54E-01	1.00E+00
TMEM70	-3.14E+00	3.89E-65	GMPPB	-2.68E-01	1.00E+00	LOC102401890	2.58E-01	1.00E+00
LOC102407804	-7.30E+00	3.99E-65	OLFM1	5.64E-01	1.00E+00	GNS	2.22E-01	1.00E+00
KCTD20	-1.95E+00	4.11E-65	TSSK2	6.37E-01	1.00E+00	CABYR	2.45E-01	1.00E+00
PLXNA2	-2.34E+00	4.18E-65	LOC112585675	3.04E-01	1.00E+00	IGLON5	3.94E-01	1.00E+00
NPHP4	-3.80E+00	4.20E-65	INTS1	1.67E-01	1.00E+00	TSPAN11	-3.71E-01	1.00E+00
LOC112582363	-2.46E+00	4.27E-65	CEP41	-1.74E-01	1.00E+00	RAB10	1.49E-01	1.00E+00
ZNF131	-2.69E+00	4.40E-65	SCNN1G	4.00E-01	1.00E+00	CAT	-1.37E-01	1.00E+00
MAN1C1	3.71E+00	4.63E-65	KLHDC4	-1.69E-01	1.00E+00	ATPAF1	-1.52E-01	1.00E+00
EIF2B5	-2.22E+00	4.68E-65	RASA3	-5.68E-01	1.00E+00	LOC102395271	-1.48E-01	1.00E+00
STX12	1.57E+00	4.72E-65	SLFNL1	-1.60E-01	1.00E+00	FAM122B	-1.88E-01	1.00E+00

FAM110A	2.86E+00	4.94E-65	TMEM245	2.24E-01	1.00E+00	DUSP11	1.56E-01	1.00E+00
RAB11A	2.13E+00	5.02E-65	LOC102390275	1.38E-01	1.00E+00	CPT1A	2.67E-01	1.00E+00
LOC102391411	-2.21E+00	5.42E-65	BBS10	4.12E-01	1.00E+00	DTL	-1.73E-01	1.00E+00
CAMLG	1.83E+00	5.59E-65	LOC102390802	-3.22E-01	1.00E+00	LOC112579217	-3.08E-01	1.00E+00
NDC1	1.79E+00	6.06E-65	C12H9orf16	-4.48E-01	1.00E+00	OTUD1	-2.65E-01	1.00E+00
BCLAF1	-2.93E+00	6.14E-65	LOC112583111	-3.77E-01	1.00E+00	DNAJB9	1.70E-01	1.00E+00
NDUFS6	-3.20E+00	6.31E-65	LOC112584551	2.94E-01	1.00E+00	TPK1	1.74E-01	1.00E+00
RAB23	-2.32E+00	6.36E-65	MS4A1	4.55E-01	1.00E+00	UBE2I	-1.47E-01	1.00E+00
ACTL7B	5.37E+00	6.63E-65	PDXDC1	1.27E-01	1.00E+00	CNOT3	-1.53E-01	1.00E+00
LOC102402027	-4.87E+00	6.64E-65	TGFBR1	-1.59E-01	1.00E+00	CPLANE2	1.83E-01	1.00E+00
DCAF4	-2.45E+00	6.87E-65	CRMP1	-1.57E-01	1.00E+00	RPP14	-2.98E-01	1.00E+00
TTLL1	-3.27E+00	7.54E-65	CYS1	-8.17E-01	1.00E+00	TMEM94	-1.45E-01	1.00E+00
LIN28B	-3.44E+00	8.48E-65	PSMA3	-1.27E-01	1.00E+00	PADI4	-3.77E-01	1.00E+00
TMEM175	-2.37E+00	9.57E-65	AP5Z1	3.08E-01	1.00E+00	DMTN	2.90E-01	1.00E+00
SLC44A1	-2.61E+00	1.01E-64	SLIRP	-1.79E-01	1.00E+00	RUNDC3A	-3.64E-01	1.00E+00
LZIC	1.67E+00	1.06E-64	VPS28	-1.60E-01	1.00E+00	SLC25A28	-1.74E-01	1.00E+00
ADAMTS7	-2.54E+00	1.22E-64	SLC9A2	-6.83E-01	1.00E+00	ANKS1B	2.04E-01	1.00E+00
GALE	-3.61E+00	1.22E-64	VAT1L	-1.80E-01	1.00E+00	WDR46	1.85E-01	1.00E+00
ZNF205	-2.76E+00	1.35E-64	TOMM20L	2.33E-01	1.00E+00	MAGEF1	-1.85E-01	1.00E+00
OSBPL5	-2.51E+00	1.36E-64	RAB20	-4.71E-01	1.00E+00	CYTL1	4.09E-01	1.00E+00
TMEM231	1.79E+00	1.39E-64	LOC112582377	6.73E-01	1.00E+00	LOC102404258	-1.67E-01	1.00E+00
ALPL	-5.54E+00	1.42E-64	PFN4	4.80E-01	1.00E+00	LOC112587007	-3.98E-01	1.00E+00
IL17RA	-4.07E+00	1.50E-64	ATG13	1.44E-01	1.00E+00	UBLCP1	-1.80E-01	1.00E+00
PTPRG	2.41E+00	1.57E-64	C12H9orf78	-1.55E-01	1.00E+00	SPATA9	2.98E-01	1.00E+00
VPS37A	1.97E+00	1.61E-64	CEP85	1.19E-01	1.00E+00	CDHR3	-3.39E-01	1.00E+00
SALL2	-2.78E+00	1.72E-64	SIPA1L1	1.02E-01	1.00E+00	CCER2	-3.26E-01	1.00E+00
TOX2	-6.08E+00	1.74E-64	CACUL1	1.95E-01	1.00E+00	LOC112581146	-3.70E-01	1.00E+00
MRPL12	-3.22E+00	1.77E-64	LOC102399551	2.14E-01	1.00E+00	PDE8B	-1.82E-01	1.00E+00
SRSF10	2.02E+00	1.82E-64	PAH	-3.99E-01	1.00E+00	CYSRT1	-3.35E-01	1.00E+00
ECI1	-3.07E+00	1.89E-64	PELI1	1.29E-01	1.00E+00	PLEK	-4.44E-01	1.00E+00
NLRP1	7.89E+00	2.12E-64	LOC112585131	5.32E-01	1.00E+00	PARP10	1.74E-01	1.00E+00
LOC112581132	6.27E+00	2.39E-64	TMEM123	-3.73E-01	1.00E+00	ZMAT5	2.08E-01	1.00E+00
APBB3	-3.26E+00	2.42E-64	CXHXorf65	-8.04E-01	1.00E+00	LOC112585765	3.30E-01	1.00E+00

RAD51AP1	1.97E+00	2.53E-64	NAV2	1.06E-01	1.00E+00	BRD2	-2.05E-01	1.00E+00
ATP5MG	-2.89E+00	2.56E-64	WFS1	1.31E-01	1.00E+00	MNT	-1.93E-01	1.00E+00
GTF3A	-2.37E+00	2.86E-64	NELL1	1.15E-01	1.00E+00	FBXO9	-1.35E-01	1.00E+00
KIF13A	-2.57E+00	2.87E-64	PUM2	1.41E-01	1.00E+00	CRACR2A	1.88E-01	1.00E+00
SLC8A2	6.77E+00	2.93E-64	BICDL1	1.12E-01	1.00E+00	VIPR2	4.49E-01	1.00E+00
MRPL1	1.88E+00	2.97E-64	LOC102409782	-2.31E-01	1.00E+00	LOC112583811	3.59E-01	1.00E+00
POU4F1	5.29E+00	2.99E-64	PRDM13	4.09E-01	1.00E+00	LOC112581623	-1.63E-01	1.00E+00
CDCA2	2.71E+00	3.00E-64	RPF2	-1.87E-01	1.00E+00	LOC112581768	3.82E-01	1.00E+00
TBC1D22B	1.68E+00	3.02E-64	POLR2C	1.14E-01	1.00E+00	PDE8A	-1.67E-01	1.00E+00
RAVER2	-2.46E+00	3.04E-64	ADGRL1	2.01E-01	1.00E+00	CCNA2	-1.77E-01	1.00E+00
ARSG	-4.20E+00	3.05E-64	RNF219	1.46E-01	1.00E+00	ULK3	-1.49E-01	1.00E+00
COL4A3BP	1.71E+00	3.10E-64	CD6	-4.82E-01	1.00E+00	CDC45	-1.78E-01	1.00E+00
CDK5RAP1	1.59E+00	3.10E-64	THBS3	4.41E-01	1.00E+00	HBP1	1.65E-01	1.00E+00
SH3BP1	2.53E+00	3.11E-64	GRIK4	2.62E-01	1.00E+00	LOC112585310	-3.10E-01	1.00E+00
ELOVL4	3.33E+00	3.25E-64	TPD52	-1.06E-01	1.00E+00	SLC30A3	2.94E-01	1.00E+00
HNRNPD	-1.91E+00	3.28E-64	DHX32	1.55E-01	1.00E+00	LOC102402580	1.54E-01	1.00E+00
CD40	4.47E+00	3.37E-64	GUCY2C	-6.43E-01	1.00E+00	CAPN3	-5.15E-01	1.00E+00
DDIT4	-2.94E+00	3.45E-64	DYRK4	-4.54E-01	1.00E+00	ATP8	-2.55E-01	1.00E+00
ARIH1	1.57E+00	3.55E-64	SLC25A51	1.28E-01	1.00E+00	TCEAL9	2.26E-01	1.00E+00
COMMD5	-2.73E+00	3.61E-64	KLHL8	1.26E-01	1.00E+00	RADIL	-2.83E-01	1.00E+00
RPL8	-4.33E+00	3.62E-64	USP40	-1.19E-01	1.00E+00	USP31	-1.53E-01	1.00E+00
LOC112586986	-3.67E+00	3.90E-64	HDHD3	-4.36E-01	1.00E+00	MYC	-1.73E-01	1.00E+00
MGST2	2.94E+00	4.07E-64	OPRD1	-5.41E-01	1.00E+00	FLCN	1.74E-01	1.00E+00
PCYOX1L	-2.67E+00	4.07E-64	PNRC1	2.65E-01	1.00E+00	LST1	-4.13E-01	1.00E+00
FBXO11	-3.85E+00	4.12E-64	SMIM27	-5.00E-01	1.00E+00	LOC102402091	2.53E-01	1.00E+00
S100A1	-7.40E+00	4.27E-64	RFXAP	-1.85E-01	1.00E+00	ASNSD1	1.68E-01	1.00E+00
CUEDC1	1.99E+00	4.37E-64	LOC102406087	-5.21E-01	1.00E+00	LOC102389262	2.39E-01	1.00E+00
CNOT11	2.67E+00	4.63E-64	EP300	2.57E-01	1.00E+00	PARN	-1.38E-01	1.00E+00
SEMA3B	-3.65E+00	4.72E-64	HELQ	-1.08E-01	1.00E+00	MKRN1	-1.90E-01	1.00E+00
UBE2D3	2.34E+00	4.82E-64	ANKRD10	-1.73E-01	1.00E+00	GPC6	1.70E-01	1.00E+00
LOC102389096	-4.57E+00	4.86E-64	LOC112578041	-2.21E-01	1.00E+00	P3H2	2.45E-01	1.00E+00
DENND1C	7.11E+00	5.36E-64	POLR1C	-2.22E-01	1.00E+00	SELENOH	-2.69E-01	1.00E+00
LOC102398050	-2.51E+00	5.37E-64	RTN4RL1	5.12E-01	1.00E+00	BCAS4	3.12E-01	1.00E+00

NEUROG2	6.93E+00	5.52E-64	SSX2IP	-1.62E-01	1.00E+00	AZIN1	-1.76E-01	1.00E+00
LY96	-7.98E+00	6.41E-64	TRAPPC13	1.54E-01	1.00E+00	PROX1	3.71E-01	1.00E+00
LOC102397633	-6.90E+00	6.53E-64	ZP3	1.57E-01	1.00E+00	ERP29	-2.18E-01	1.00E+00
CCDC66	1.78E+00	7.39E-64	LOC102406612	-7.71E-01	1.00E+00	LOC112586534	-3.65E-01	1.00E+00
ITGBL1	-6.62E+00	7.77E-64	SCEL	1.97E-01	1.00E+00	C19H5orf58	3.27E-01	1.00E+00
GDA	5.16E+00	7.78E-64	TLE4	1.04E-01	1.00E+00	SOX12	-1.90E-01	1.00E+00
SPACA6	-4.31E+00	8.32E-64	MCFD2	-2.65E-01	1.00E+00	GSTO2	4.28E-01	1.00E+00
PIDD1	-4.53E+00	8.73E-64	PADI6	1.42E-01	1.00E+00	MYO7B	3.53E-01	1.00E+00
GPT	-6.56E+00	9.24E-64	LOC112586230	4.16E-01	1.00E+00	LOC102406687	1.83E-01	1.00E+00
PRDX6	-2.72E+00	9.43E-64	LOC112578199	2.80E-01	1.00E+00	FGGY	2.03E-01	1.00E+00
SGSM3	1.86E+00	9.48E-64	TNKS1BP1	-3.13E-01	1.00E+00	SCAMP4	1.55E-01	1.00E+00
RPL26	-2.93E+00	9.86E-64	TTC9C	1.12E-01	1.00E+00	GABARAPL1	1.73E-01	1.00E+00
TYK2	-4.19E+00	9.97E-64	ASXL3	2.49E-01	1.00E+00	SEMA3F	3.33E-01	1.00E+00
BIN1	-5.65E+00	1.01E-63	ADK	-2.42E-01	1.00E+00	EVA1B	3.32E-01	1.00E+00
FUK	1.98E+00	1.19E-63	SH3PXD2B	-3.22E-01	1.00E+00	GDF11	2.73E-01	1.00E+00
MEAF6	-1.87E+00	1.20E-63	TMEM19	3.73E-01	1.00E+00	PAF1	-1.66E-01	1.00E+00
OSBP2	6.97E+00	1.20E-63	NCSTN	1.14E-01	1.00E+00	SCIN	-3.38E-01	1.00E+00
NDUFS1	-1.75E+00	1.32E-63	RAB43	-2.74E-01	1.00E+00	MXD1	-3.30E-01	1.00E+00
SLC25A6	-3.51E+00	1.36E-63	DOLPP1	-3.42E-01	1.00E+00	NEDD4L	3.33E-01	1.00E+00
DYNC2H1	-2.34E+00	1.37E-63	LOC102416404	-2.12E-01	1.00E+00	MFSD14A	-1.69E-01	1.00E+00
RHBDD3	-2.75E+00	1.43E-63	PACSIN2	1.25E-01	1.00E+00	SNRPA1	-1.56E-01	1.00E+00
LOC102415974	2.22E+00	1.49E-63	LOC112578799	-6.02E-01	1.00E+00	LOC102413479	-2.95E-01	1.00E+00
NEURL1B	-3.21E+00	1.55E-63	FBXO17	-5.92E-01	1.00E+00	NIPA1	-2.21E-01	1.00E+00
SNAPC4	-2.20E+00	1.59E-63	KPNA6	1.44E-01	1.00E+00	CENPT	-2.26E-01	1.00E+00
RABAC1	-2.93E+00	1.59E-63	SPAG1	4.36E-01	1.00E+00	ACAN	3.58E-01	1.00E+00
WIPF3	-4.35E+00	1.68E-63	MCTP1	-2.10E-01	1.00E+00	TRIM59	-2.30E-01	1.00E+00
EXTL2	-2.41E+00	1.78E-63	PGBD1	-3.81E-01	1.00E+00	NAPB	-1.80E-01	1.00E+00
LOC102407177	-7.18E+00	1.82E-63	EXOC6B	1.72E-01	1.00E+00	KLHL7	-2.36E-01	1.00E+00
PRG4	3.77E+00	2.00E-63	KLHDC10	1.93E-01	1.00E+00	ORC1	-2.37E-01	1.00E+00
ARGLU1	-2.16E+00	2.52E-63	LOC102394123	-2.00E-01	1.00E+00	ECE1	-2.00E-01	1.00E+00
LOC102410234	2.22E+00	2.68E-63	NIT1	-2.01E-01	1.00E+00	MPND	1.76E-01	1.00E+00
IER3	-6.46E+00	2.76E-63	MUSK	-2.78E-01	1.00E+00	FAM163A	2.27E-01	1.00E+00

TELO2	-2.57E+00	3.05E-63	HDDC3	-2.37E-01	1.00E+00	PARVA	1.75E-01	1.00E+00
CDC25A	1.96E+00	3.08E-63	SLC26A2	-2.53E-01	1.00E+00	SHC1	-1.50E-01	1.00E+00
LOC102410978	7.42E+00	3.34E-63	ARPC3	-1.31E-01	1.00E+00	TMCO3	1.64E-01	1.00E+00
COL4A2	-6.28E+00	3.50E-63	PFN2	-1.03E-01	1.00E+00	LOC102414362	3.57E-01	1.00E+00
AR	-5.62E+00	3.57E-63	XPO4	-1.60E-01	1.00E+00	CD244	6.54E-01	1.00E+00
MPST	-3.52E+00	4.03E-63	C1GALT1	2.03E-01	1.00E+00	LOC102406855	-3.62E-01	1.00E+00
LRRC57	-2.60E+00	4.06E-63	TWNK	-2.78E-01	1.00E+00	SLC25A40	1.58E-01	1.00E+00
KRBA1	-5.35E+00	4.27E-63	GTF2B	-1.00E-01	1.00E+00	RARS	1.51E-01	1.00E+00
RNF141	2.53E+00	4.27E-63	ENKUR	-3.13E-01	1.00E+00	NXPH3	3.64E-01	1.00E+00
SEMA4A	5.18E+00	4.37E-63	LOC112578002	-2.69E-01	1.00E+00	FAM149A	-2.66E-01	1.00E+00
FAM49A	-4.08E+00	4.57E-63	GON7	-1.58E-01	1.00E+00	ZDHHC7	1.39E-01	1.00E+00
GPR63	2.66E+00	4.67E-63	PPP1R37	1.42E-01	1.00E+00	SPATA5	-1.65E-01	1.00E+00
KLC1	-1.58E+00	4.92E-63	PTPRG	1.18E-01	1.00E+00	PLA2G6	-1.64E-01	1.00E+00
DENND6A	-3.30E+00	5.42E-63	DDX43	-1.96E-01	1.00E+00	RAB14	-4.46E-01	1.00E+00
KLHL40	-5.32E+00	5.43E-63	FAAP24	-1.78E-01	1.00E+00	NUDT21	-1.58E-01	1.00E+00
TMEM129	-2.47E+00	5.66E-63	RNF24	1.87E-01	1.00E+00	LOC102403725	2.31E-01	1.00E+00
LOC102404930	-2.10E+00	5.72E-63	BRWD1	1.57E-01	1.00E+00	DKK3	-2.39E-01	1.00E+00
VEZF1	-2.80E+00	5.79E-63	B3GALNT2	1.12E-01	1.00E+00	HOMER1	2.20E-01	1.00E+00
CTH	2.12E+00	5.85E-63	WDR17	-1.87E-01	1.00E+00	GINS4	1.57E-01	1.00E+00
TDRD1	8.61E+00	5.92E-63	PARP2	1.61E-01	1.00E+00	DUSP1	-3.59E-01	1.00E+00
ASB8	1.62E+00	5.99E-63	LOC102393493	-5.91E-01	1.00E+00	C2H6orf136	-1.77E-01	1.00E+00
LOC112588003	6.88E+00	6.03E-63	ENPP2	-2.77E-01	1.00E+00	FKBPL	3.05E-01	1.00E+00
FAM234B	-1.75E+00	6.13E-63	RNF130	-1.47E-01	1.00E+00	EXOC1	1.57E-01	1.00E+00
PCSK1N	-5.32E+00	6.19E-63	LOC102393384	1.05E-01	1.00E+00	ALPK1	2.71E-01	1.00E+00
LOC112585257	2.47E+00	6.26E-63	PHF1	1.44E-01	1.00E+00	ATF7IP2	-1.82E-01	1.00E+00
GLTP	1.74E+00	6.53E-63	PLEKHG2	2.40E-01	1.00E+00	CPQ	1.39E-01	1.00E+00
TM6SF1	-4.22E+00	7.50E-63	PHACTR2	-4.31E-01	1.00E+00	ISLR2	3.71E-01	1.00E+00
TNPO2	-1.63E+00	7.81E-63	LOC112582099	-5.27E-01	1.00E+00	SRPX	2.71E-01	1.00E+00
FBXO31	1.73E+00	7.90E-63	SLC2A9	-3.97E-01	1.00E+00	CMKLR1	6.63E-01	1.00E+00
G0S2	-9.32E+00	7.94E-63	CENPBD1	-2.20E-01	1.00E+00	RALGPS2	-1.82E-01	1.00E+00
B3GNT2	-4.30E+00	8.19E-63	USP42	1.44E-01	1.00E+00	SUV39H1	-1.89E-01	1.00E+00
IRF9	-4.05E+00	8.37E-63	GBF1	1.76E-01	1.00E+00	TBC1D32	2.64E-01	1.00E+00
B4GALT4	-	8.62E-63	NLRP9	1.29E-01	1.00E+00	ARMC12	-3.58E-	1.00E+00

	1.75E+00						01	
SBNO1	2.15E+00	8.69E-63	LOC102411025	-2.47E-01	1.00E+00	ZNF652	-2.16E-01	1.00E+00
FAM129A	-2.10E+00	8.78E-63	RFESD	3.30E-01	1.00E+00	LOC102394267	-2.65E-01	1.00E+00
SRSF7	-1.89E+00	9.20E-63	SLC25A5	-1.06E-01	1.00E+00	FHL2	1.69E-01	1.00E+00
FMC1	-3.39E+00	9.35E-63	USP6NL	1.28E-01	1.00E+00	HOXA1	3.64E-01	1.00E+00
TMEM246	-4.55E+00	9.39E-63	ACLY	1.45E-01	1.00E+00	MLF1	-1.98E-01	1.00E+00
NUPR1	-6.29E+00	9.48E-63	NREP	-3.23E-01	1.00E+00	CLOCK	1.78E-01	1.00E+00
MEF2A	-2.58E+00	9.79E-63	LOC112577759	6.00E-01	1.00E+00	ADCY6	-1.44E-01	1.00E+00
NCAPH	2.15E+00	1.03E-62	SNX16	1.72E-01	1.00E+00	PTGIS	4.72E-01	1.00E+00
DTX1	3.68E+00	1.05E-62	CEP128	1.65E-01	1.00E+00	PARPBP	-2.47E-01	1.00E+00
PCM1	-2.12E+00	1.12E-62	UHRF1	1.50E-01	1.00E+00	TSEN15	-1.64E-01	1.00E+00
FEM1C	1.91E+00	1.31E-62	SIGLECL1	-4.49E-01	1.00E+00	HDAC10	-2.08E-01	1.00E+00
SYNE2	-2.41E+00	1.36E-62	GLCE	2.10E-01	1.00E+00	DLEC1	-1.50E-01	1.00E+00
PNO1	1.61E+00	1.42E-62	KDF1	1.80E-01	1.00E+00	LRRC24	1.67E-01	1.00E+00
MIS18A	2.53E+00	1.59E-62	ITGA2B	-5.39E-01	1.00E+00	WASHC3	2.19E-01	1.00E+00
OSBPL7	-2.41E+00	1.66E-62	DEGS2	-6.56E-01	1.00E+00	LOC102413176	-2.17E-01	1.00E+00
RERG	-6.15E+00	1.73E-62	BCAS4	-3.33E-01	1.00E+00	NRBP2	-1.65E-01	1.00E+00
TM9SF4	-1.72E+00	1.77E-62	CHIC2	-1.70E-01	1.00E+00	NPEPL1	-1.68E-01	1.00E+00
CACNG5	5.55E+00	1.85E-62	LOC102388962	-2.39E-01	1.00E+00	KPNA2	-1.62E-01	1.00E+00
SP110	-5.34E+00	1.86E-62	ZBTB39	1.91E-01	1.00E+00	PRR12	-1.50E-01	1.00E+00
CLN3	-2.24E+00	1.96E-62	DCLRE1A	1.08E-01	1.00E+00	DNAJC24	-2.22E-01	1.00E+00
NCOA5	-1.97E+00	2.10E-62	CLPX	1.25E-01	1.00E+00	ZDHHC8	-1.52E-01	1.00E+00
EIF2AK3	-2.96E+00	2.11E-62	UBAP2	1.28E-01	1.00E+00	TAF2	-1.81E-01	1.00E+00
SCFD1	1.67E+00	2.16E-62	LOC102396068	-1.19E+00	1.00E+00	LRRC8C	3.11E-01	1.00E+00
CTNND1	1.98E+00	2.29E-62	LOC112582106	4.45E-01	1.00E+00	CKAP2L	-2.04E-01	1.00E+00
LOC112582101	-7.93E+00	2.38E-62	RTN1	2.24E-01	1.00E+00	NR4A3	-5.80E-01	1.00E+00
PPTC7	1.99E+00	2.40E-62	ANKRD54	1.60E-01	1.00E+00	EHMT2	-1.45E-01	1.00E+00
RPL31	-4.10E+00	2.41E-62	LOC102392881	7.96E-01	1.00E+00	GALNS	-1.55E-01	1.00E+00
NDRG1	-5.15E+00	2.89E-62	HEG1	2.39E-01	1.00E+00	CATSPERE	2.26E-01	1.00E+00
CCNB1	2.53E+00	3.02E-62	LOC102394594	1.68E-01	1.00E+00	KIAA2013	1.69E-01	1.00E+00
LTC4S	-7.05E+00	3.03E-62	CRH	-1.27E-01	1.00E+00	ARPC4	1.65E-01	1.00E+00
LOC102401890	-3.64E+00	3.41E-62	STARD4	2.92E-01	1.00E+00	LOC102408090	-1.89E-01	1.00E+00
FEM1A	-1.81E+00	3.57E-62	CASC3	1.23E-01	1.00E+00	ITPRID2	2.66E-01	1.00E+00
ERAP2	-	3.76E-62	ZBTB46	3.72E-01	1.00E+00	MDM2	-1.61E-	1.00E+00

	5.97E+00						01	
LOC112586962	3.69E+00	3.78E-62	SVBP	-2.49E-01	1.00E+00	SH3GL1	1.46E-01	1.00E+00
EMC6	-3.38E+00	3.82E-62	ACP5	5.95E-01	1.00E+00	LOC102412844	-2.40E-01	1.00E+00
LOC112583109	-8.47E+00	4.46E-62	SPIDR	1.11E-01	1.00E+00	XXYLT1	-1.56E-01	1.00E+00
LYRM9	-2.56E+00	4.68E-62	WAC	1.41E-01	1.00E+00	ITGA9	-2.04E-01	1.00E+00
TTYH3	-6.29E+00	4.69E-62	B4GALNT3	1.79E-01	1.00E+00	NEIL1	-2.17E-01	1.00E+00
LOC102392464	-1.72E+00	4.82E-62	HPSE	8.62E-01	1.00E+00	JPT1	1.76E-01	1.00E+00
GALM	-4.96E+00	5.14E-62	SEC31A	1.44E-01	1.00E+00	CCDC146	4.20E-01	1.00E+00
LOC112582189	-2.83E+00	5.30E-62	RNPEP	3.69E-01	1.00E+00	SLC6A15	3.26E-01	1.00E+00
DAB2	-6.12E+00	5.37E-62	CSGALNACT1	-3.48E-01	1.00E+00	LOC102414482	-3.01E-01	1.00E+00
RPL14	-3.99E+00	6.08E-62	NUP93	1.00E-01	1.00E+00	FAT1	1.93E-01	1.00E+00
MCAT	-2.12E+00	6.09E-62	CNOT6L	1.88E-01	1.00E+00	ZNF274	2.08E-01	1.00E+00
ABCG2	2.03E+00	6.20E-62	SLC35D1	1.49E-01	1.00E+00	CLMP	-1.76E-01	1.00E+00
CATSPERG	-3.99E+00	6.30E-62	KNTC1	1.79E-01	1.00E+00	C3	-3.20E-01	1.00E+00
SYNPO	-3.89E+00	6.66E-62	NUDT18	-3.65E-01	1.00E+00	CD80	-3.18E-01	1.00E+00
TRIM3	1.66E+00	6.97E-62	DNAJC12	-6.85E-01	1.00E+00	CCDC50	1.34E-01	1.00E+00
GZF1	-2.42E+00	7.30E-62	DTNB	1.23E-01	1.00E+00	TMEM198	-3.05E-01	1.00E+00
LGALS3BP	-7.04E+00	7.46E-62	IGSF11	1.25E-01	1.00E+00	IKBKB	1.43E-01	1.00E+00
FXYD1	-6.90E+00	8.16E-62	LOC102400440	-1.72E-01	1.00E+00	CHD3	-2.85E-01	1.00E+00
LOC102409495	5.06E+00	8.62E-62	LOC102407985	3.72E-01	1.00E+00	CCDC142	-2.80E-01	1.00E+00
CDH2	-5.47E+00	8.69E-62	C1H3orf70	1.99E-01	1.00E+00	ALKBH1	-1.42E-01	1.00E+00
ZC2HC1C	2.68E+00	9.34E-62	CENPA	-2.61E-01	1.00E+00	LOC102401116	2.15E-01	1.00E+00
PSMG2	-2.56E+00	1.01E-61	NQO2	-2.45E-01	1.00E+00	LOC102399668	1.81E-01	1.00E+00
ISLR	-5.62E+00	1.06E-61	FAM219A	1.29E-01	1.00E+00	BAZ1A	2.05E-01	1.00E+00
TAOK1	-2.11E+00	1.06E-61	EFNB3	3.29E-01	1.00E+00	UBE2S	-2.29E-01	1.00E+00
PARD6A	2.59E+00	1.08E-61	SLAIN2	1.40E-01	1.00E+00	GNPNAT1	1.53E-01	1.00E+00
CPN1	4.74E+00	1.11E-61	UBE2N	1.35E-01	1.00E+00	SHBG	-2.05E-01	1.00E+00
ZIC3	5.07E+00	1.21E-61	FTSJ1	1.57E-01	1.00E+00	C9H19orf25	1.96E-01	1.00E+00
CPNE3	-4.92E+00	1.33E-61	CHCHD2	-2.94E-01	1.00E+00	CDCA7L	-1.85E-01	1.00E+00
ATOX1	-3.48E+00	1.36E-61	LOC112580806	-1.80E-01	1.00E+00	CTCF	-1.42E-01	1.00E+00
TEX30	2.17E+00	1.37E-61	ESS2	1.96E-01	1.00E+00	MARCH9	1.64E-01	1.00E+00
LOC112582992	-5.56E+00	1.44E-61	SREBF2	2.34E-01	1.00E+00	LOC102393424	1.86E-01	1.00E+00
TIAL1	-1.42E+00	1.47E-61	ZNF462	2.12E-01	1.00E+00	RCHY1	1.50E-01	1.00E+00
CADM3	5.85E+00	1.47E-61	S100PBP	1.74E-01	1.00E+00	IL18	4.95E-01	1.00E+00

ACTR1B	-1.77E+00	1.59E-61	DGCR2	2.22E-01	1.00E+00	PAK3	1.91E-01	1.00E+00
AP2A1	-1.47E+00	1.68E-61	GATC	-1.55E-01	1.00E+00	RHBDD1	1.67E-01	1.00E+00
MYL6B	-3.32E+00	1.75E-61	RBM25	1.34E-01	1.00E+00	HELLS	-1.57E-01	1.00E+00
GLS	-3.00E+00	1.85E-61	C18H19orf54	1.71E-01	1.00E+00	LOC102394407	-1.40E-01	1.00E+00
ANKFY1	2.25E+00	1.89E-61	CLK1	-2.15E-01	1.00E+00	KIAA1324L	3.33E-01	1.00E+00
TEX9	2.16E+00	1.89E-61	LOC112587797	-5.32E-01	1.00E+00	HDAC8	1.49E-01	1.00E+00
CASP7	-5.72E+00	2.03E-61	STARD7	2.75E-01	1.00E+00	LOC112579065	2.16E-01	1.00E+00
TSHZ2	-2.19E+00	2.08E-61	GMDS	-1.44E-01	1.00E+00	RFWD3	-2.95E-01	1.00E+00
BLOC1S4	-3.93E+00	2.31E-61	MSRA	-5.10E-01	1.00E+00	GBA	1.50E-01	1.00E+00
KRT8	-8.28E+00	2.37E-61	CS	3.26E-01	1.00E+00	LOC102411389	2.39E-01	1.00E+00
WDR45	-1.95E+00	2.40E-61	PLD6	-1.80E-01	1.00E+00	NADSYN1	-1.47E-01	1.00E+00
PTPRS	-1.91E+00	2.57E-61	ZNF346	1.10E-01	1.00E+00	TAF6	-1.36E-01	1.00E+00
ACBD7	3.27E+00	2.68E-61	ICK	-3.77E-01	1.00E+00	FAM210B	1.54E-01	1.00E+00
LOC112580283	8.01E+00	2.83E-61	GGA1	1.34E-01	1.00E+00	ZNF830	-1.62E-01	1.00E+00
MTSS1	2.27E+00	2.98E-61	FYCO1	1.98E-01	1.00E+00	SPATS2	1.44E-01	1.00E+00
LOC102405660	-2.38E+00	3.04E-61	ANKS6	2.24E-01	1.00E+00	SYN3	2.28E-01	1.00E+00
GPR160	6.65E+00	3.06E-61	DNA2	-2.37E-01	1.00E+00	C6H1orf226	2.57E-01	1.00E+00
LOC112586208	5.27E+00	3.26E-61	C9H5orf24	3.49E-01	1.00E+00	LOC102403213	-3.43E-01	1.00E+00
ATG13	-1.73E+00	3.28E-61	LOC112580137	1.28E-01	1.00E+00	OSBPL11	1.92E-01	1.00E+00
KCNH1	5.20E+00	3.30E-61	LIPA	-1.42E-01	1.00E+00	GRIA1	2.57E-01	1.00E+00
KHDC4	-1.85E+00	3.31E-61	RNF168	1.47E-01	1.00E+00	TMEM200A	-2.02E-01	1.00E+00
LOC102400615	6.92E+00	3.43E-61	TMEM225B	-2.58E-01	1.00E+00	SIKE1	-2.28E-01	1.00E+00
MAP3K2	-2.90E+00	3.48E-61	FAM184B	-2.76E-01	1.00E+00	PMEL	3.11E-01	1.00E+00
LOC112580215	5.21E+00	3.53E-61	LOC102392517	4.22E-01	1.00E+00	COL1A2	4.93E-01	1.00E+00
LOC112580229	6.95E+00	3.64E-61	PHACTR3	-1.08E-01	1.00E+00	YWHAH	-1.57E-01	1.00E+00
ERGIC1	-1.56E+00	3.92E-61	NR3C1	2.18E-01	1.00E+00	GTPBP4	-1.42E-01	1.00E+00
PLD1	-4.10E+00	3.96E-61	INSRR	-5.56E-01	1.00E+00	RAB12	-1.31E-01	1.00E+00
CXCL9	6.34E+00	4.30E-61	ATP8B4	1.73E-01	1.00E+00	ANXA11	1.36E-01	1.00E+00
DPF3	-3.62E+00	4.34E-61	PLA2G4D	-1.80E-01	1.00E+00	KIF11	-1.71E-01	1.00E+00
STX1A	2.22E+00	4.74E-61	LOC102400872	-1.73E-01	1.00E+00	FSHR	-1.43E-01	1.00E+00
EN2	6.70E+00	4.93E-61	ERCC6	1.49E-01	1.00E+00	GSAP	3.48E-01	1.00E+00
LOC112581595	5.95E+00	5.17E-61	ARID3A	-2.81E-01	1.00E+00	SMAD9	2.90E-01	1.00E+00
TUBE1	2.02E+00	5.38E-61	PWP2	1.34E-01	1.00E+00	TBC1D8	-1.88E-01	1.00E+00
WDR75	1.63E+00	5.42E-61	PMVK	-2.99E-	1.00E+00	SPATS2L	1.56E-01	1.00E+00

				01				
USP35	2.47E+00	5.51E-61	ADAMTS3	8.95E-01	1.00E+00	POLR3D	-1.38E-01	1.00E+00
ADAT2	-3.26E+00	5.59E-61	LOC102390720	1.45E-01	1.00E+00	ZNF804B	2.09E-01	1.00E+00
STX3	2.19E+00	5.68E-61	ARHGEF2	-4.33E-01	1.00E+00	HNRNPD	-1.70E-01	1.00E+00
TIMM44	2.03E+00	5.77E-61	CELF6	-2.05E-01	1.00E+00	UBE2T	-2.44E-01	1.00E+00
ETFB	-3.03E+00	6.19E-61	LOC102411682	7.61E-01	1.00E+00	C2CD5	-1.60E-01	1.00E+00
LOC102406518	-5.96E+00	6.29E-61	SPSB2	-2.19E-01	1.00E+00	RSRC2	-1.37E-01	1.00E+00
TRIM35	-2.23E+00	6.48E-61	RBFOX3	-3.76E-01	1.00E+00	GUCY1A1	-2.74E-01	1.00E+00
SERPING1	-4.32E+00	7.17E-61	KCTD18	-3.18E-01	1.00E+00	PARP9	1.64E-01	1.00E+00
C24H16orf58	-2.76E+00	7.23E-61	KDM5B	-2.32E-01	1.00E+00	LOC112579667	-3.50E-01	1.00E+00
EPB41L1	-3.01E+00	7.45E-61	VAV3	1.51E-01	1.00E+00	NR2F2	-1.96E-01	1.00E+00
KIT	4.18E+00	7.68E-61	STK11IP	1.53E-01	1.00E+00	C9H19orf38	-2.46E-01	1.00E+00
KIAA1143	1.60E+00	8.22E-61	MAP3K12	1.82E-01	1.00E+00	LTBR	1.45E-01	1.00E+00
EXTL3	1.78E+00	8.63E-61	CKMT2	-6.27E-01	1.00E+00	CLIC5	4.43E-01	1.00E+00
CTNNA2	-2.33E+00	8.88E-61	CDC73	-1.37E-01	1.00E+00	QSOX2	-1.45E-01	1.00E+00
ADCY3	2.67E+00	9.46E-61	DNASE1	2.69E-01	1.00E+00	LOC102402422	3.08E-01	1.00E+00
DYNC1LI2	-2.10E+00	9.48E-61	ORC6	-1.08E-01	1.00E+00	SCML2	-2.41E-01	1.00E+00
KCNC4	3.08E+00	9.48E-61	RPS4X	-2.22E-01	1.00E+00	ZNF696	-1.61E-01	1.00E+00
TPX2	2.20E+00	9.51E-61	LOC102401651	-8.46E-01	1.00E+00	PSEN1	1.46E-01	1.00E+00
ZSCAN31	3.91E+00	9.55E-61	MRPL12	-2.47E-01	1.00E+00	PTH1R	3.05E-01	1.00E+00
HSD17B11	-4.04E+00	9.68E-61	LOC112583749	5.37E-01	1.00E+00	LGR4	3.01E-01	1.00E+00
NUP37	1.89E+00	1.00E-60	RWDD3	-1.70E-01	1.00E+00	MISP3	3.04E-01	1.00E+00
DRC1	4.56E+00	1.06E-60	MPP7	1.33E-01	1.00E+00	LOC112587907	-3.59E-01	1.00E+00
QTRT1	-2.51E+00	1.09E-60	FAM102B	3.10E-01	1.00E+00	SPCS3	-1.36E-01	1.00E+00
PRRG3	-5.40E+00	1.12E-60	SCYL2	1.55E-01	1.00E+00	SCN1B	3.75E-01	1.00E+00
ZC3HAV1	-5.10E+00	1.12E-60	CNEP1R1	1.80E-01	1.00E+00	SUGP1	-1.55E-01	1.00E+00
RCN3	-3.00E+00	1.19E-60	MTNR1A	4.90E-01	1.00E+00	CST6	2.40E-01	1.00E+00
PLPPR1	3.31E+00	1.31E-60	USP49	-2.04E-01	1.00E+00	OPLAH	-1.37E-01	1.00E+00
POLR3C	1.77E+00	1.37E-60	LRP6	1.57E-01	1.00E+00	TUBGCP4	-1.49E-01	1.00E+00
KLF1	4.81E+00	1.50E-60	GOLGA2	2.02E-01	1.00E+00	TMEM56	-2.59E-01	1.00E+00
RUNX1	-4.43E+00	1.51E-60	LRRTM4	1.79E-01	1.00E+00	HOOK2	-2.65E-01	1.00E+00
ADIRF	4.92E+00	1.72E-60	KCNH2	5.28E-01	1.00E+00	BCL2L2	-1.57E-01	1.00E+00
LOC102410179	3.53E+00	1.87E-60	XPO6	1.22E-01	1.00E+00	PIF1	-2.76E-01	1.00E+00

TMEM74B	5.11E+00	1.93E-60	TMEM18	2.10E-01	1.00E+00	GPAT4	1.63E-01	1.00E+00
NHLRC3	-2.76E+00	1.95E-60	SPATA16	3.96E-01	1.00E+00	LOC102413296	1.79E-01	1.00E+00
BSN	4.44E+00	1.96E-60	OPRL1	-4.65E-01	1.00E+00	KHNYN	1.34E-01	1.00E+00
UHRF1	2.30E+00	1.97E-60	LOC112586884	-5.20E-01	1.00E+00	FAM45A	-1.51E-01	1.00E+00
ALPK3	-2.23E+00	2.08E-60	LOC102398226	-7.52E-01	1.00E+00	SEPT6	2.13E-01	1.00E+00
LOC102391122	2.81E+00	2.09E-60	ZNF274	1.48E-01	1.00E+00	TBX1	-3.44E-01	1.00E+00
TMEM189	1.79E+00	2.10E-60	LOC102404153	4.34E-01	1.00E+00	RAB3IL1	4.47E-01	1.00E+00
MAL	-8.07E+00	2.14E-60	ARAP2	-1.43E-01	1.00E+00	LOC102395245	2.12E-01	1.00E+00
ZNF333	-2.54E+00	2.37E-60	TRMT112	-1.30E-01	1.00E+00	RRAGD	-2.02E-01	1.00E+00
HEATR3	-2.16E+00	2.46E-60	TRIM2	-3.12E-01	1.00E+00	BFSP1	3.44E-01	1.00E+00
MRI1	-2.64E+00	2.47E-60	JMJD6	-1.63E-01	1.00E+00	PRUNE2	2.66E-01	1.00E+00
TMEM259	-1.84E+00	2.67E-60	LOC102398702	-4.23E-01	1.00E+00	CDC6	-2.07E-01	1.00E+00
PUSL1	2.49E+00	2.70E-60	MAP3K9	2.10E-01	1.00E+00	NABP1	-2.21E-01	1.00E+00
CCNL1	-4.31E+00	2.76E-60	NPRL2	2.13E-01	1.00E+00	APBA3	1.53E-01	1.00E+00
LOC102395815	4.39E+00	2.85E-60	NID2	1.36E-01	1.00E+00	GRIP1	2.22E-01	1.00E+00
ADD3	2.49E+00	2.86E-60	EEF2KMT	1.55E-01	1.00E+00	RFX5	-2.25E-01	1.00E+00
RASSF4	-5.89E+00	3.00E-60	ISG20L2	-5.79E-01	1.00E+00	RLIM	-1.77E-01	1.00E+00
SIX3	7.36E+00	3.00E-60	SPTY2D1	1.38E-01	1.00E+00	FGD5	2.17E-01	1.00E+00
IRF2BP2	-2.68E+00	3.36E-60	LOC112587361	-3.43E-01	1.00E+00	HOXA3	3.91E-01	1.00E+00
TUBGCP2	-2.13E+00	3.40E-60	GPRC5A	-2.04E-01	1.00E+00	CRMP1	2.20E-01	1.00E+00
CBL	3.21E+00	3.43E-60	MAK16	-3.13E-01	1.00E+00	LOC112581182	-3.02E-01	1.00E+00
LOC102391771	5.58E+00	3.45E-60	NPNT	1.16E-01	1.00E+00	PRSS23	-3.88E-01	1.00E+00
RPS10	-3.86E+00	3.86E-60	MCF2L	-4.91E-01	1.00E+00	ZMYND10	-3.35E-01	1.00E+00
ACAD9	-2.51E+00	3.88E-60	DNM3	1.91E-01	1.00E+00	VCL	1.67E-01	1.00E+00
LOC102416376	-5.89E+00	3.90E-60	MAN2B1	-1.73E-01	1.00E+00	PTPRK	1.48E-01	1.00E+00
PIAS2	1.58E+00	4.19E-60	PURG	2.23E-01	1.00E+00	NUP160	-1.57E-01	1.00E+00
CPSF6	-1.67E+00	4.20E-60	CTDSP1	-2.02E-01	1.00E+00	MRPS12	2.04E-01	1.00E+00
SH3BGRL	-6.14E+00	4.26E-60	TMEM38B	1.94E-01	1.00E+00	JMJD8	-1.59E-01	1.00E+00
MRPL22	2.01E+00	4.42E-60	GJB2	-3.99E-01	1.00E+00	PLEKHH3	2.08E-01	1.00E+00
LOC112585789	7.06E+00	4.45E-60	ZSWIM5	-2.65E-01	1.00E+00	KHDC4	-1.52E-01	1.00E+00
LOC102395800	6.54E+00	4.45E-60	MYO9A	2.03E-01	1.00E+00	TMEM218	1.71E-01	1.00E+00
HAPLN3	-5.41E+00	4.72E-60	REPS2	1.87E-01	1.00E+00	RHBDD3	1.68E-01	1.00E+00
CMC1	2.07E+00	4.84E-60	SMARCD2	1.09E-01	1.00E+00	ABCB9	-2.90E-01	1.00E+00
NMB	-8.03E+00	4.87E-60	SYT11	1.98E-01	1.00E+00	SLIT3	3.37E-01	1.00E+00

BRSK2	4.46E+00	4.90E-60	CNKSR3	-5.42E-01	1.00E+00	CD82	-1.78E-01	1.00E+00
AKIRIN2	1.63E+00	4.93E-60	HHIPL1	7.29E-01	1.00E+00	RARRES2	2.80E-01	1.00E+00
BRPF3	2.25E+00	5.00E-60	BARHL2	1.55E-01	1.00E+00	MCC	1.74E-01	1.00E+00
IMMP2L	1.82E+00	5.11E-60	KLHL2	1.93E-01	1.00E+00	GK5	-2.08E-01	1.00E+00
GINS4	1.67E+00	5.37E-60	SYTL5	1.64E-01	1.00E+00	ESS2	1.70E-01	1.00E+00
LOC112577737	8.33E+00	5.49E-60	LOC112580859	-3.47E-01	1.00E+00	TSG101	1.30E-01	1.00E+00
LOC102412418	-7.15E+00	5.53E-60	FLCN	-1.33E-01	1.00E+00	MAFK	2.17E-01	1.00E+00
LOC112585687	7.47E+00	5.55E-60	NGLY1	-1.19E-01	1.00E+00	PABPC4	-1.83E-01	1.00E+00
LOC102395360	6.94E+00	5.79E-60	WDR74	-2.10E-01	1.00E+00	LOC112578895	-3.78E-01	1.00E+00
TXNRD1	-1.60E+00	5.85E-60	VMP1	-1.58E-01	1.00E+00	TXK	2.95E-01	1.00E+00
CLIC4	1.80E+00	6.09E-60	CCNB1IP1	-2.19E-01	1.00E+00	ELOVL4	3.10E-01	1.00E+00
CHN2	-5.09E+00	6.34E-60	KIAA1549L	1.48E-01	1.00E+00	FIZ1	1.54E-01	1.00E+00
PLCD3	-4.94E+00	6.44E-60	GBA2	1.45E-01	1.00E+00	REXO2	1.58E-01	1.00E+00
UNC5D	7.26E+00	6.76E-60	ATF1	2.30E-01	1.00E+00	NDUFS6	2.04E-01	1.00E+00
CDS2	-1.51E+00	7.15E-60	LOC112586636	-1.46E-01	1.00E+00	IMPG2	2.87E-01	1.00E+00
P2RX5	3.80E+00	7.37E-60	TG	2.28E-01	1.00E+00	ARSD	-1.90E-01	1.00E+00
USP20	-2.10E+00	7.52E-60	SCN8A	1.33E-01	1.00E+00	MAN2C1	-1.58E-01	1.00E+00
CHST12	1.94E+00	7.69E-60	HAUS5	3.18E-01	1.00E+00	SH3YL1	-1.79E-01	1.00E+00
SCARB2	-1.81E+00	7.73E-60	FAM213A	1.70E-01	1.00E+00	SUZ12	-1.71E-01	1.00E+00
PCID2	-2.01E+00	7.92E-60	LMAN2	1.07E-01	1.00E+00	LOC102399664	3.82E-01	1.00E+00
TRIP10	-3.93E+00	7.96E-60	GALNT18	-6.61E-01	1.00E+00	AOC1	3.79E-01	1.00E+00
FKBP11	-3.86E+00	7.99E-60	SLC6A15	-4.25E-01	1.00E+00	SARNP	-1.65E-01	1.00E+00
LOC102415700	-2.33E+00	8.72E-60	GBX2	1.98E-01	1.00E+00	CAP1	1.59E-01	1.00E+00
LOC102409972	-4.15E+00	9.17E-60	CIITA	-1.71E-01	1.00E+00	MBIP	-1.46E-01	1.00E+00
LOC102409228	-4.30E+00	9.48E-60	RNF111	1.54E-01	1.00E+00	RPN1	-1.85E-01	1.00E+00
G2E3	-2.58E+00	9.95E-60	ZNF217	1.61E-01	1.00E+00	CDK20	-1.28E-01	1.00E+00
DNAH9	-4.89E+00	1.07E-59	ZNF22	-2.69E-01	1.00E+00	PNRC1	-2.30E-01	1.00E+00
SNX5	-1.65E+00	1.08E-59	EXOSC4	-4.31E-01	1.00E+00	VPS52	-1.28E-01	1.00E+00
HOPX	-5.33E+00	1.08E-59	MAGEA10	7.15E-01	1.00E+00	FBXW5	1.71E-01	1.00E+00
SIX5	-2.47E+00	1.10E-59	GRAMD1C	3.99E-01	1.00E+00	ESCO2	-1.82E-01	1.00E+00
CLSTN3	4.99E+00	1.15E-59	RBBP9	-5.82E-01	1.00E+00	ITPKB	-1.28E-01	1.00E+00
KAT8	-1.70E+00	1.15E-59	RPL31	-1.73E-01	1.00E+00	ACOT7	1.85E-01	1.00E+00
FAM129C	5.46E+00	1.17E-59	IRF4	-4.02E-01	1.00E+00	CCM2	1.50E-01	1.00E+00
TLDC1	1.83E+00	1.18E-59	KSR1	-2.07E-	1.00E+00	CA5A	-4.00E-	1.00E+00

				01			01	
LOC102411767	5.15E+00	1.19E-59	DARS2	-2.41E-01	1.00E+00	LOC102401704	-3.07E-01	1.00E+00
SPART	-1.71E+00	1.21E-59	KIAA1614	-3.06E-01	1.00E+00	PI16	3.38E-01	1.00E+00
LOC102389829	-4.79E+00	1.24E-59	RHBDD1	1.33E-01	1.00E+00	CDK5R1	3.30E-01	1.00E+00
CREG1	2.90E+00	1.31E-59	IFT22	1.62E-01	1.00E+00	PPP1R7	1.75E-01	1.00E+00
GBA	-2.40E+00	1.33E-59	NFKBIZ	-6.39E-01	1.00E+00	ATP13A3	-2.61E-01	1.00E+00
PAM	-5.88E+00	1.37E-59	PTPRM	-3.60E-01	1.00E+00	LZTS3	1.68E-01	1.00E+00
MYLK	-3.96E+00	1.40E-59	IL18	-6.07E-01	1.00E+00	RAB11FIP2	-1.83E-01	1.00E+00
TMEM256	-4.87E+00	1.46E-59	SLC43A3	1.44E-01	1.00E+00	PISD	1.36E-01	1.00E+00
PEA15	-1.77E+00	1.61E-59	LOC112584644	5.54E-01	1.00E+00	RND2	3.03E-01	1.00E+00
ZBTB20	-2.58E+00	1.74E-59	SNAP47	1.29E-01	1.00E+00	SLK	1.50E-01	1.00E+00
SEC11C	2.21E+00	1.80E-59	TBC1D9B	1.39E-01	1.00E+00	UNC119	-2.30E-01	1.00E+00
LDHB	-2.98E+00	1.87E-59	PNPLA4	-2.61E-01	1.00E+00	ENOX1	2.48E-01	1.00E+00
RPS15	-4.07E+00	1.90E-59	GRIN2A	-2.18E-01	1.00E+00	TLE4	1.93E-01	1.00E+00
ZWILCH	1.68E+00	1.90E-59	LOC102408909	4.99E-01	1.00E+00	CTDSP2	1.73E-01	1.00E+00
SNRPN	-3.04E+00	2.03E-59	LOC112578116	-1.52E-01	1.00E+00	CPNE9	-3.35E-01	1.00E+00
TAZ	-3.13E+00	2.16E-59	RGS22	-1.78E-01	1.00E+00	MRAS	1.37E-01	1.00E+00
RANBP6	-3.71E+00	2.20E-59	TRABD2B	6.07E-01	1.00E+00	NREP	1.44E-01	1.00E+00
NT5DC2	-2.17E+00	2.23E-59	TIMM8A	-1.22E-01	1.00E+00	MAP3K14	2.05E-01	1.00E+00
GAN	2.62E+00	2.44E-59	NDUFC2	-1.93E-01	1.00E+00	MSMO1	-1.48E-01	1.00E+00
HARS	-1.83E+00	2.51E-59	TMEM223	-2.15E-01	1.00E+00	NOP10	2.17E-01	1.00E+00
IRF2BP1	1.74E+00	2.52E-59	POLR2G	-1.50E-01	1.00E+00	TXLNG	-1.85E-01	1.00E+00
GPR75	-6.17E+00	2.60E-59	HGH1	1.98E-01	1.00E+00	NXT2	2.14E-01	1.00E+00
LOC112586243	7.64E+00	2.65E-59	LOC102414676	-3.62E-01	1.00E+00	LOC112579539	-3.19E-01	1.00E+00
EXOC7	-1.78E+00	2.65E-59	TCP11	-1.58E-01	1.00E+00	CTSA	1.55E-01	1.00E+00
RAPGEF5	3.98E+00	2.68E-59	LOC102404242	2.71E-01	1.00E+00	TMEM159	1.81E-01	1.00E+00
ATP13A2	-3.07E+00	2.71E-59	ABRAXAS1	-1.92E-01	1.00E+00	KIRREL1	-2.78E-01	1.00E+00
RPS19	-3.69E+00	2.77E-59	LOC102397029	-1.71E-01	1.00E+00	FABP5	3.28E-01	1.00E+00
CEP170B	2.79E+00	2.83E-59	LSM5	-1.94E-01	1.00E+00	URM1	1.56E-01	1.00E+00
MRGBP	1.85E+00	2.86E-59	PKLR	-3.18E-01	1.00E+00	RELN	2.86E-01	1.00E+00
TMEM68	-3.43E+00	2.90E-59	LOC112587745	-1.79E-01	1.00E+00	CPSF1	-1.41E-01	1.00E+00
NOL6	1.93E+00	3.01E-59	AIP	-1.62E-01	1.00E+00	BARD1	-2.12E-01	1.00E+00
TSPAN9	-4.23E+00	3.21E-59	SPG21	-1.52E-01	1.00E+00	LTC4S	2.79E-01	1.00E+00
MXD4	-	3.26E-59	NID1	5.16E-01	1.00E+00	TC2N	3.82E-01	1.00E+00

	1.89E+00							
DRAM2	-1.97E+00	3.53E-59	ITPRIP	1.53E-01	1.00E+00	RFK	1.58E-01	1.00E+00
RAB11FIP1	-5.28E+00	3.55E-59	LOC102415962	-3.43E-01	1.00E+00	LOC102412878	-1.78E-01	1.00E+00
LOC102411437	9.00E+00	3.58E-59	LOC102409002	1.17E-01	1.00E+00	AQR	1.54E-01	1.00E+00
LOC102392193	5.20E+00	3.62E-59	LOC112580156	-3.24E-01	1.00E+00	NRAS	1.65E-01	1.00E+00
BSDC1	1.46E+00	3.79E-59	YIF1A	1.54E-01	1.00E+00	EPS8	3.42E-01	1.00E+00
ZNF395	-1.96E+00	3.93E-59	TIE1	4.61E-01	1.00E+00	ZNF467	3.52E-01	1.00E+00
IKZF5	-3.34E+00	4.01E-59	THSD7B	1.42E-01	1.00E+00	BBS4	-1.51E-01	1.00E+00
LOC112578958	-6.40E+00	4.02E-59	TOP3B	1.33E-01	1.00E+00	CDCA8	-1.61E-01	1.00E+00
MEX3C	-2.77E+00	4.25E-59	SEPSECS	1.74E-01	1.00E+00	SOX15	2.22E-01	1.00E+00
PAXX	-4.05E+00	4.42E-59	ENKD1	2.18E-01	1.00E+00	KIF4A	-1.64E-01	1.00E+00
NYNRIN	-6.24E+00	4.44E-59	RPL36	-1.68E-01	1.00E+00	NUCB1	-1.56E-01	1.00E+00
SPR	-2.92E+00	4.52E-59	MTM1	1.30E-01	1.00E+00	MPHOSPH9	-1.48E-01	1.00E+00
HACD2	-1.62E+00	4.53E-59	G3BP2	1.20E-01	1.00E+00	SPICE1	-1.39E-01	1.00E+00
TCEA3	-5.77E+00	4.72E-59	CTBS	8.03E-01	1.00E+00	LOC102415868	1.87E-01	1.00E+00
LOC102394141	-5.03E+00	4.76E-59	FOXN3	-1.53E-01	1.00E+00	RAB17	3.05E-01	1.00E+00
YWHAZ	-2.27E+00	5.11E-59	KLF1	-2.72E-01	1.00E+00	NDUFS3	-1.80E-01	1.00E+00
LOC112586981	-3.49E+00	5.72E-59	HEBP1	1.74E-01	1.00E+00	ERMARD	-1.52E-01	1.00E+00
TNRC6C	-1.76E+00	5.75E-59	TUBB6	-2.54E-01	1.00E+00	MMP14	1.62E-01	1.00E+00
CRIM1	-2.04E+00	6.07E-59	LOC102398822	-2.29E-01	1.00E+00	ANGEL2	-1.59E-01	1.00E+00
ZBTB47	-2.95E+00	6.42E-59	TMEM216	-2.95E-01	1.00E+00	MED22	1.40E-01	1.00E+00
TNFRSF1A	-5.81E+00	6.81E-59	ROGDI	-1.70E-01	1.00E+00	HMGN5	-2.69E-01	1.00E+00
TMEM176A	-6.88E+00	7.33E-59	MTA2	-3.95E-01	1.00E+00	PKHD1L1	-3.73E-01	1.00E+00
SMG7	2.05E+00	7.40E-59	SYNPO2L	-3.64E-01	1.00E+00	SPRED1	1.89E-01	1.00E+00
HS1BP3	-5.98E+00	7.94E-59	ZKSCAN1	1.19E-01	1.00E+00	C19H5orf51	1.44E-01	1.00E+00
LOC102389646	-2.76E+00	8.04E-59	GAB1	2.19E-01	1.00E+00	CHSY3	3.27E-01	1.00E+00
MON1B	1.89E+00	8.60E-59	MYO1B	-1.20E-01	1.00E+00	ERAP2	-2.26E-01	1.00E+00
NLE1	-2.95E+00	9.41E-59	PSPH	-1.38E-01	1.00E+00	IL27RA	1.46E-01	1.00E+00
NCAM1	-4.87E+00	9.49E-59	SEC22C	1.42E-01	1.00E+00	FZD3	1.49E-01	1.00E+00
EIF3F	-2.07E+00	1.04E-58	LCP2	-4.89E-01	1.00E+00	GHR	3.23E-01	1.00E+00
ZNF18	2.09E+00	1.07E-58	FHDC1	1.81E-01	1.00E+00	C15H8orf82	-1.84E-01	1.00E+00
KIAA0232	-2.00E+00	1.10E-58	PTGES3L	-1.76E-01	1.00E+00	RBMX2	-1.42E-01	1.00E+00
LOC102405290	-6.94E+00	1.10E-58	HNRNPD	-1.06E-01	1.00E+00	EFHD2	2.20E-01	1.00E+00

VGLL4	2.06E+00	1.16E-58	LOC102401474	-4.69E-01	1.00E+00	MTRR	-1.45E-01	1.00E+00
MAATS1	2.54E+00	1.20E-58	TIFAB	8.50E-01	1.00E+00	CETN3	-1.81E-01	1.00E+00
CKAP4	-1.76E+00	1.39E-58	ITGA6	1.29E-01	1.00E+00	FBXO24	-3.12E-01	1.00E+00
GFOD1	4.62E+00	1.41E-58	LOC102393658	-1.78E-01	1.00E+00	MAPK1	-1.43E-01	1.00E+00
BTG1	-1.96E+00	1.43E-58	NWD2	-1.69E-01	1.00E+00	PCID2	-1.35E-01	1.00E+00
TARS2	-4.13E+00	1.46E-58	PSMB5	-1.50E-01	1.00E+00	NUFIP2	-1.76E-01	1.00E+00
DDX59	1.66E+00	1.51E-58	BABAM1	1.15E-01	1.00E+00	COMMD10	1.43E-01	1.00E+00
BTBD7	-2.63E+00	1.52E-58	ST3GAL4	-5.11E-01	1.00E+00	COL1A1	2.90E-01	1.00E+00
TOR2A	1.95E+00	1.62E-58	NAT14	1.64E-01	1.00E+00	IPP	-1.70E-01	1.00E+00
EXOSC10	-1.75E+00	1.74E-58	DLX2	7.56E-01	1.00E+00	RAB11FIP5	1.62E-01	1.00E+00
SF3B1	-2.00E+00	1.76E-58	DNAJC15	-1.74E-01	1.00E+00	ZFX	-1.68E-01	1.00E+00
ARHGAP25	7.28E+00	1.80E-58	ABHD13	-3.59E-01	1.00E+00	ERI2	-2.22E-01	1.00E+00
RIC8A	-2.52E+00	1.82E-58	LOC102397659	-4.04E-01	1.00E+00	FRAS1	3.71E-01	1.00E+00
KLHL12	1.61E+00	1.87E-58	LOC102406873	1.53E-01	1.00E+00	SCUBE1	2.87E-01	1.00E+00
STK4	1.81E+00	2.01E-58	MKRN2	1.04E-01	1.00E+00	GOLGA2	-1.94E-01	1.00E+00
LOC112579071	-7.56E+00	2.11E-58	PEG3	2.28E-01	1.00E+00	IQGAP1	1.82E-01	1.00E+00
FBXL7	-5.04E+00	2.15E-58	CIB2	-1.84E-01	1.00E+00	C21H3orf67	-1.65E-01	1.00E+00
TMC4	2.41E+00	2.19E-58	EIF4E	1.03E-01	1.00E+00	LOC112580624	2.90E-01	1.00E+00
KCTD10	2.04E+00	2.20E-58	LPIN2	1.33E-01	1.00E+00	GLMN	-1.61E-01	1.00E+00
MRTFA	2.19E+00	2.22E-58	MAP1LC3B	-1.25E-01	1.00E+00	POLE3	-1.44E-01	1.00E+00
LOC112584434	-6.26E+00	2.22E-58	ZNF710	2.42E-01	1.00E+00	SLC35E4	2.82E-01	1.00E+00
RGS17	-5.62E+00	2.41E-58	KAT2A	1.12E-01	1.00E+00	ARMC9	-1.44E-01	1.00E+00
WWTR1	-4.40E+00	2.49E-58	SORL1	1.48E-01	1.00E+00	NFKBIZ	-4.03E-01	1.00E+00
TMEM248	1.63E+00	2.53E-58	MAP3K6	-2.88E-01	1.00E+00	TXNDC16	3.21E-01	1.00E+00
LOC102400130	6.00E+00	2.60E-58	CEP135	2.80E-01	1.00E+00	PIK3R4	1.32E-01	1.00E+00
LOC102412117	-3.50E+00	2.61E-58	UBL3	1.62E-01	1.00E+00	TRNP1	2.97E-01	1.00E+00
HS3ST3B1	4.74E+00	2.70E-58	CPXM1	-6.76E-01	1.00E+00	MYH10	-1.62E-01	1.00E+00
DCBLD1	2.41E+00	2.71E-58	CDC42BPB	1.71E-01	1.00E+00	HLCS	-1.79E-01	1.00E+00
HSD17B1	-7.32E+00	2.82E-58	COQ3	-1.48E-01	1.00E+00	CLCC1	-1.44E-01	1.00E+00
LOC112579902	4.84E+00	2.84E-58	VPS29	-1.27E-01	1.00E+00	PPIL6	-3.32E-01	1.00E+00
CES2	-6.38E+00	2.85E-58	COQ9	1.50E-01	1.00E+00	PRKRA	-1.39E-01	1.00E+00
LOC112586251	8.09E+00	2.89E-58	LOC112581212	-5.78E-01	1.00E+00	PLPPR3	2.40E-01	1.00E+00
NEK8	-4.13E+00	3.00E-58	SLC9A5	-5.65E-01	1.00E+00	MEMO1	1.26E-01	1.00E+00
LOC102415514	-	3.43E-58	TSSK3	7.27E-01	1.00E+00	NOS3	2.27E-01	1.00E+00

	2.93E+00							
LOC112585512	5.67E+00	3.43E-58	NMD3	1.18E-01	1.00E+00	ARPC1B	2.45E-01	1.00E+00
FBXO30	3.68E+00	3.44E-58	TCL1A	1.36E-01	1.00E+00	LOC102409111	3.11E-01	1.00E+00
SIK2	-3.07E+00	3.51E-58	LOC112580349	1.34E-01	1.00E+00	DNAJC3	-1.30E-01	1.00E+00
EPHX1	-3.93E+00	3.65E-58	TTL	1.81E-01	1.00E+00	FLYWCH2	2.02E-01	1.00E+00
C1QTNF1	-7.45E+00	3.75E-58	WDHD1	-1.62E-01	1.00E+00	SGCE	-1.28E-01	1.00E+00
UCN	-6.16E+00	3.80E-58	PRC1	1.33E-01	1.00E+00	LOC112581726	3.34E-01	1.00E+00
MICALL2	-5.33E+00	3.85E-58	PCIF1	1.34E-01	1.00E+00	WBP4	-1.33E-01	1.00E+00
LOC102405958	4.07E+00	3.88E-58	NKX2-8	-1.58E-01	1.00E+00	LAMA2	2.22E-01	1.00E+00
TBC1D30	2.67E+00	4.00E-58	RPS6KA2	-3.80E-01	1.00E+00	MYCBP2	2.10E-01	1.00E+00
POU3F1	5.04E+00	4.09E-58	SHB	-2.64E-01	1.00E+00	TNS3	-1.58E-01	1.00E+00
SEMA4D	-2.24E+00	4.10E-58	EDNRA	-3.31E-01	1.00E+00	TRIM9	3.35E-01	1.00E+00
LIMK2	2.01E+00	4.14E-58	MVP	1.46E-01	1.00E+00	SLC10A6	3.93E-01	1.00E+00
UAP1L1	-2.80E+00	4.26E-58	LOC102399127	3.30E-01	1.00E+00	ITPR2	-1.36E-01	1.00E+00
LOC102409414	-2.76E+00	4.29E-58	USP43	2.07E-01	1.00E+00	TAOK2	-1.41E-01	1.00E+00
LIX1	5.32E+00	4.65E-58	LOC112581119	2.90E-01	1.00E+00	ZW10	-1.44E-01	1.00E+00
HACD4	2.09E+00	4.69E-58	ZBTB8B	2.92E-01	1.00E+00	MTX2	1.34E-01	1.00E+00
PPP1R7	-2.43E+00	4.80E-58	SRSF1	1.34E-01	1.00E+00	LOC102406739	-1.77E-01	1.00E+00
THOC2	-2.41E+00	4.89E-58	C2H6orf132	7.85E-01	1.00E+00	NOP58	-1.39E-01	1.00E+00
LOC102410915	7.97E+00	5.03E-58	NSD3	2.11E-01	1.00E+00	WDR13	-1.58E-01	1.00E+00
BEX5	-7.61E+00	5.14E-58	ADAD2	-1.35E-01	1.00E+00	PPIG	-1.26E-01	1.00E+00
NDC80	2.06E+00	5.25E-58	BCL2L12	1.09E-01	1.00E+00	ATP2C2	-2.51E-01	1.00E+00
CHID1	-2.37E+00	5.57E-58	ARID1B	1.28E-01	1.00E+00	SLC37A3	1.61E-01	1.00E+00
KCNJ8	-7.51E+00	5.85E-58	LAMP2	1.17E-01	1.00E+00	LOC102412587	3.30E-01	1.00E+00
CEP152	2.19E+00	5.99E-58	LOC102401786	1.39E-01	1.00E+00	PANK3	-1.89E-01	1.00E+00
SDHA	-2.00E+00	6.08E-58	PIGT	1.74E-01	1.00E+00	HSPB1	-2.37E-01	1.00E+00
CDC42EP1	-6.50E+00	6.39E-58	TRPV1	-1.70E-01	1.00E+00	SRP9	1.43E-01	1.00E+00
JRKL	-4.98E+00	6.54E-58	U2AF2	1.06E-01	1.00E+00	ILF3	-1.50E-01	1.00E+00
TSC1	1.92E+00	6.65E-58	RPAIN	-1.76E-01	1.00E+00	RHOA	-1.56E-01	1.00E+00
BUB1B	2.28E+00	6.89E-58	LOC102390085	2.43E-01	1.00E+00	NKIRAS2	1.25E-01	1.00E+00
MAP3K1	-3.96E+00	7.15E-58	RSPO2	1.22E-01	1.00E+00	LBH	3.40E-01	1.00E+00
REEP5	-4.51E+00	7.18E-58	MARK1	-1.85E-01	1.00E+00	PRRT3	2.45E-01	1.00E+00
ARL4C	-4.50E+00	7.67E-58	RAD23A	-1.55E-01	1.00E+00	LZTS2	1.35E-01	1.00E+00
LOC112585014	7.03E+00	7.79E-58	U2AF1	-1.32E-01	1.00E+00	LOC102402353	-2.70E-01	1.00E+00

PDLIM5	3.29E+00	7.95E-58	TRIM68	-1.54E-01	1.00E+00	TGIF1	-1.36E-01	1.00E+00
ZBED5	-2.57E+00	8.24E-58	CAST	-2.65E-01	1.00E+00	ENTPD4	-1.55E-01	1.00E+00
RILPL2	-2.48E+00	8.31E-58	PAPOLA	9.97E-02	1.00E+00	PTRHD1	-2.24E-01	1.00E+00
KIAA1549L	6.33E+00	9.42E-58	SLC24A4	-1.69E-01	1.00E+00	FOPNL	-1.75E-01	1.00E+00
HOXA1	3.43E+00	9.44E-58	DNAL4	1.72E-01	1.00E+00	FBXL7	2.17E-01	1.00E+00
IKZF2	-2.74E+00	1.08E-57	LOC102410179	-1.89E-01	1.00E+00	ARHGEF9	1.57E-01	1.00E+00
INSM2	6.44E+00	1.09E-57	PLXNA1	1.75E-01	1.00E+00	RPUSD1	-2.62E-01	1.00E+00
APC2	4.29E+00	1.13E-57	NUP37	-1.37E-01	1.00E+00	KLHL5	-1.80E-01	1.00E+00
RSL1D1	1.72E+00	1.16E-57	TUB	1.19E-01	1.00E+00	NR6A1	2.09E-01	1.00E+00
RETREG2	-1.54E+00	1.18E-57	ZMYM4	2.28E-01	1.00E+00	CMTM3	1.57E-01	1.00E+00
ADNP	-2.12E+00	1.23E-57	CPSF6	-1.23E-01	1.00E+00	HES7	3.37E-01	1.00E+00
PAAF1	-2.33E+00	1.23E-57	ADAMTSL1	1.23E-01	1.00E+00	BCOR	-2.17E-01	1.00E+00
IFI35	-5.57E+00	1.25E-57	GAREM2	-3.87E-01	1.00E+00	RBM27	-1.62E-01	1.00E+00
RBM17	-2.29E+00	1.27E-57	PLAGL2	1.69E-01	1.00E+00	LOC102394014	-3.19E-01	1.00E+00
CDAN1	-3.01E+00	1.30E-57	SLC25A16	2.68E-01	1.00E+00	STYXL1	-2.50E-01	1.00E+00
NUMBL	-2.44E+00	1.36E-57	LOC112584376	5.60E-01	1.00E+00	FBXO10	-1.50E-01	1.00E+00
IFNL3	7.58E+00	1.39E-57	LOC102405126	-2.00E-01	1.00E+00	LOC102389985	3.33E-01	1.00E+00
P4HB	-2.96E+00	1.39E-57	ZBTB6	3.21E-01	1.00E+00	AGT	-4.97E-01	1.00E+00
KLF13	-2.33E+00	1.52E-57	GPATCH8	1.70E-01	1.00E+00	FBLIM1	2.44E-01	1.00E+00
UBASH3B	3.12E+00	1.55E-57	LRP1B	-1.33E-01	1.00E+00	SLBP	-1.27E-01	1.00E+00
SLC6A8	1.66E+00	1.58E-57	PHLDB1	1.19E-01	1.00E+00	ELOVL6	-1.60E-01	1.00E+00
LOC102394286	5.60E+00	1.60E-57	LOC102408914	-2.57E-01	1.00E+00	LOC102400376	3.42E-01	1.00E+00
LSM12	-1.63E+00	1.64E-57	LOC112582250	-1.38E-01	1.00E+00	CNTROB	-1.39E-01	1.00E+00
LOC102410603	-3.68E+00	1.64E-57	GSR	1.12E-01	1.00E+00	LOC112584176	-5.61E-01	1.00E+00
LOC102390612	-2.74E+00	1.77E-57	LOC102399864	-1.21E-01	1.00E+00	ATP6V1B2	1.34E-01	1.00E+00
LOC112587745	-2.35E+00	1.85E-57	ELOVL2	2.98E-01	1.00E+00	CREB3	1.58E-01	1.00E+00
ABHD14A	-4.05E+00	1.94E-57	LOC102403725	-3.75E-01	1.00E+00	LOC102404191	2.02E-01	1.00E+00
DAP	-1.88E+00	1.96E-57	PPP2R5B	1.66E-01	1.00E+00	NTRK2	4.44E-01	1.00E+00
ARRDC3	-3.40E+00	1.99E-57	GPNMB	-8.20E-01	1.00E+00	CPT1C	3.00E-01	1.00E+00
FAM185A	-2.32E+00	2.06E-57	CACNB2	-2.08E-01	1.00E+00	GUF1	-1.42E-01	1.00E+00
ITPR1	2.83E+00	2.10E-57	LRP2	1.20E-01	1.00E+00	CPSF7	-1.26E-01	1.00E+00
SLC35D2	-4.37E+00	2.15E-57	AP3B2	1.31E-01	1.00E+00	ANGPTL6	-3.03E-01	1.00E+00
TFDP1	-1.94E+00	2.18E-57	TRIM4	-2.04E-01	1.00E+00	LOC112581215	3.00E-01	1.00E+00

ASXL1	-1.76E+00	2.27E-57	MPHOSPH6	-1.54E-01	1.00E+00	SAV1	-1.65E-01	1.00E+00
MSH5	3.96E+00	2.45E-57	ABLIM1	1.23E-01	1.00E+00	BST2	-2.20E-01	1.00E+00
LOC102415932	7.53E+00	2.46E-57	SOX21	-9.29E-01	1.00E+00	FAM161A	-1.49E-01	1.00E+00
PDGFC	-5.86E+00	2.52E-57	LOC112586460	3.09E-01	1.00E+00	TMEM41A	-1.52E-01	1.00E+00
SLC25A19	1.82E+00	2.53E-57	FKBP3	-2.38E-01	1.00E+00	CNNM2	-1.52E-01	1.00E+00
CCDC173	2.86E+00	2.66E-57	AKAP5	-7.30E-01	1.00E+00	JAGN1	1.62E-01	1.00E+00
KIF20B	2.31E+00	2.70E-57	YPEL1	-2.32E-01	1.00E+00	FAM167A	4.05E-01	1.00E+00
UBL7	-2.26E+00	2.81E-57	PRDM15	2.53E-01	1.00E+00	PAM	1.95E-01	1.00E+00
LCLAT1	1.85E+00	2.82E-57	CCDC157	2.46E-01	1.00E+00	SMPD4	-1.37E-01	1.00E+00
LOC102405260	2.28E+00	2.86E-57	LOC102404426	-2.09E-01	1.00E+00	NKTR	-1.64E-01	1.00E+00
PIGY	2.26E+00	3.12E-57	SEMA3E	-2.04E-01	1.00E+00	PGP	-1.86E-01	1.00E+00
C10H6orf203	2.62E+00	3.29E-57	TOMM5	-2.42E-01	1.00E+00	RBM43	1.60E-01	1.00E+00
HSPB7	-8.68E+00	3.38E-57	LOC102397167	-4.48E-01	1.00E+00	NFRKB	-1.37E-01	1.00E+00
LRRC14	-3.98E+00	3.48E-57	XYLT1	6.64E-01	1.00E+00	CENPH	-1.74E-01	1.00E+00
SOX4	-4.21E+00	3.48E-57	LOC112580411	-5.01E-01	1.00E+00	GABRA2	2.57E-01	1.00E+00
PLCL2	-2.62E+00	3.53E-57	AGMAT	-2.14E-01	1.00E+00	CBARP	1.69E-01	1.00E+00
ASMT	4.39E+00	3.69E-57	NLRP2	1.10E-01	1.00E+00	CCNC	1.44E-01	1.00E+00
TNFRSF12A	-6.28E+00	3.71E-57	SMAGP	-5.28E-01	1.00E+00	FAM57A	1.56E-01	1.00E+00
ALG12	-2.41E+00	3.79E-57	TCTEX1D2	-1.67E-01	1.00E+00	TMC4	-2.07E-01	1.00E+00
NCKIPSD	2.30E+00	3.89E-57	PAX8	-4.74E-01	1.00E+00	FBXL4	-1.47E-01	1.00E+00
CD9	-6.30E+00	3.92E-57	SGPP2	1.13E-01	1.00E+00	CRAMP1	-1.80E-01	1.00E+00
ACP2	-2.91E+00	4.04E-57	RBFOX2	-1.64E-01	1.00E+00	MYH9	2.07E-01	1.00E+00
BCAT2	-3.22E+00	4.17E-57	LOC102413604	-4.25E-01	1.00E+00	RNF180	2.40E-01	1.00E+00
TCEAL1	-4.87E+00	4.17E-57	TARBP1	2.14E-01	1.00E+00	DDX20	-1.31E-01	1.00E+00
LOC102399409	4.41E+00	4.23E-57	HHIP	-1.50E-01	1.00E+00	EPOP	3.10E-01	1.00E+00
MRPL9	-2.02E+00	4.24E-57	EBP	-2.01E-01	1.00E+00	LOC102416224	1.49E-01	1.00E+00
MCUB	2.13E+00	4.50E-57	C19H5orf58	-2.03E-01	1.00E+00	EHD2	1.58E-01	1.00E+00
PDCD2L	1.90E+00	4.58E-57	TACSTD2	-1.37E-01	1.00E+00	BAG2	1.68E-01	1.00E+00
SNRNP70	-2.10E+00	4.98E-57	TP53I3	-2.48E-01	1.00E+00	NGEF	1.99E-01	1.00E+00
JUND	-4.68E+00	5.01E-57	PCED1B	-3.60E-01	1.00E+00	TFG	-1.50E-01	1.00E+00
LOC102401361	-3.00E+00	5.10E-57	ZMAT3	1.40E-01	1.00E+00	LRIF1	2.25E-01	1.00E+00
ATP11A	3.21E+00	5.11E-57	GNA14	-1.12E-01	1.00E+00	QRICH1	-1.41E-01	1.00E+00
ISCA2	-	5.19E-57	S100Z	1.77E-01	1.00E+00	TM9SF3	-1.85E-	1.00E+00

	2.57E+00						01	
TXNDC12	-1.86E+00	5.35E-57	NXNL2	1.01E+00	1.00E+00	PLD1	-1.48E-01	1.00E+00
KBTBD13	8.65E+00	5.71E-57	EFTUD2	1.12E-01	1.00E+00	SH2D4A	2.92E-01	1.00E+00
CCDC36	3.43E+00	5.80E-57	XPO7	1.25E-01	1.00E+00	KCNAB1	1.96E-01	1.00E+00
SIX1	5.39E+00	5.90E-57	CCDC8	-4.81E-01	1.00E+00	CEP89	1.43E-01	1.00E+00
MAP1B	-3.15E+00	5.92E-57	LOC102409978	-6.89E-01	1.00E+00	ATG4A	2.14E-01	1.00E+00
MOSPD3	-2.73E+00	6.03E-57	GPX2	2.88E-01	1.00E+00	SETD3	-1.32E-01	1.00E+00
TTLL5	1.69E+00	6.03E-57	PGGT1B	1.59E-01	1.00E+00	SLC35G1	-3.37E-01	1.00E+00
LOC102398781	-6.67E+00	6.42E-57	ANKRD6	-2.06E-01	1.00E+00	TTC38	-1.76E-01	1.00E+00
ACTG2	-5.46E+00	6.46E-57	TSSC4	-2.19E-01	1.00E+00	FDXR	-2.33E-01	1.00E+00
ZNF469	-6.35E+00	6.75E-57	WASL	1.32E-01	1.00E+00	UVSSA	-3.59E-01	1.00E+00
DOCK7	-2.24E+00	6.94E-57	GABRA4	-4.67E-01	1.00E+00	SLC1A5	-1.51E-01	1.00E+00
GPT2	2.07E+00	7.07E-57	CAAP1	1.50E-01	1.00E+00	PSMB9	-1.84E-01	1.00E+00
LOC102393946	-2.54E+00	7.85E-57	SNRPD1	-1.39E-01	1.00E+00	CBFB	1.41E-01	1.00E+00
GPN3	1.61E+00	8.19E-57	DCP2	1.51E-01	1.00E+00	MXRA8	1.90E-01	1.00E+00
UBAC1	-1.89E+00	8.28E-57	TDG	-5.89E-01	1.00E+00	LOC112578437	3.03E-01	1.00E+00
ATP6V1FNB	4.07E+00	8.63E-57	ELF5	-5.85E-01	1.00E+00	CPEB3	-3.18E-01	1.00E+00
SMOC2	-6.25E+00	9.07E-57	CDC16	-1.68E-01	1.00E+00	PTPN2	-1.22E-01	1.00E+00
NGLY1	1.54E+00	9.80E-57	FLII	1.13E-01	1.00E+00	LOC102392737	-4.33E-01	1.00E+00
FGF17	7.04E+00	1.04E-56	PYGB	-1.30E-01	1.00E+00	CFAP43	-3.58E-01	1.00E+00
RNF215	-2.42E+00	1.08E-56	NCDN	1.39E-01	1.00E+00	C3H9orf43	-2.54E-01	1.00E+00
MSRB3	-3.27E+00	1.10E-56	RPL21	-1.17E-01	1.00E+00	TMEM132D	1.78E-01	1.00E+00
JMY	-2.26E+00	1.15E-56	LOC112578502	2.78E-01	1.00E+00	LOC112585767	-3.35E-01	1.00E+00
CD63	-2.45E+00	1.22E-56	KIDINS220	1.36E-01	1.00E+00	METTL13	-1.47E-01	1.00E+00
LOC112581224	7.27E+00	1.23E-56	POLR2K	-1.60E-01	1.00E+00	OCIAD2	1.82E-01	1.00E+00
LOC102399486	-2.72E+00	1.28E-56	ZNRF1	1.12E-01	1.00E+00	FOXA3	2.96E-01	1.00E+00
PLS3	-4.64E+00	1.29E-56	LOC102397662	-5.84E-01	1.00E+00	KCNMB4	2.15E-01	1.00E+00
ZNF527	-3.22E+00	1.36E-56	MFAP3	-3.37E-01	1.00E+00	CABLES1	3.64E-01	1.00E+00
AMHR2	-4.89E+00	1.51E-56	LOC112583870	-6.49E-01	1.00E+00	DUSP2	-4.02E-01	1.00E+00
ZDHHC9	-3.51E+00	1.52E-56	MIA	-5.36E-01	1.00E+00	ADAMTS12	4.77E-01	1.00E+00
R3HCC1	-6.96E+00	1.53E-56	DDX10	-9.74E-02	1.00E+00	SENP1	-1.58E-01	1.00E+00
GPRIN3	3.71E+00	1.58E-56	SMPD4	1.50E-01	1.00E+00	ZGRF1	-1.62E-01	1.00E+00
PPAT	-1.71E+00	1.65E-56	DKK3	1.35E-01	1.00E+00	CDKN1B	1.35E-01	1.00E+00
ATP6V1B2	1.57E+00	1.73E-56	SIDT1	-2.91E-	1.00E+00	FAM160B2	-1.27E-	1.00E+00

				01			01	
TMIGD2	5.73E+00	1.77E-56	LOC102399277	-2.09E-01	1.00E+00	WDR75	1.40E-01	1.00E+00
SLC4A3	-6.06E+00	1.86E-56	RHOU	4.03E-01	1.00E+00	LOC102413819	-1.29E-01	1.00E+00
SMS	-1.60E+00	1.87E-56	LOC112583399	-5.94E-01	1.00E+00	IL1RN	-6.35E-01	1.00E+00
DYNC1I1	3.70E+00	1.90E-56	RFK	1.47E-01	1.00E+00	ZRSR2	-1.47E-01	1.00E+00
ARL4A	1.70E+00	1.91E-56	LOC112587012	4.15E-01	1.00E+00	LOC102396286	4.01E-01	1.00E+00
LOC102413100	-3.29E+00	2.09E-56	WDR11	1.47E-01	1.00E+00	DOCK4	2.33E-01	1.00E+00
SOCS5	1.68E+00	2.23E-56	LOC112583901	1.53E-01	1.00E+00	DLG1	1.60E-01	1.00E+00
ULK1	-2.77E+00	2.31E-56	MPDU1	-1.09E-01	1.00E+00	PECR	-1.65E-01	1.00E+00
CITED4	-4.95E+00	2.31E-56	THRA	1.73E-01	1.00E+00	AMOT	2.13E-01	1.00E+00
PHACTR1	6.39E+00	2.32E-56	INPP5E	-3.77E-01	1.00E+00	LOC112586866	3.13E-01	1.00E+00
ADAM19	3.80E+00	2.34E-56	RAB11FIP5	1.50E-01	1.00E+00	PACSIN1	-1.73E-01	1.00E+00
SLC22A31	5.35E+00	2.38E-56	ARHGAP42	2.33E-01	1.00E+00	MOCOS	-3.10E-01	1.00E+00
BCL11A	-4.83E+00	2.38E-56	PBXIP1	1.11E-01	1.00E+00	LOC102414856	2.24E-01	1.00E+00
GNG5	-2.31E+00	2.44E-56	YBEY	-3.37E-01	1.00E+00	LOC102394431	2.80E-01	1.00E+00
SLC26A8	2.41E+00	2.51E-56	KLF7	1.52E-01	1.00E+00	PCDHB10	2.97E-01	1.00E+00
IRF2	-4.78E+00	2.55E-56	NCOA6	1.38E-01	1.00E+00	RSAD2	3.13E-01	1.00E+00
CRIP3	-4.67E+00	2.55E-56	MARCH10	-2.19E-01	1.00E+00	ATL2	-1.38E-01	1.00E+00
MRPS34	-3.18E+00	2.59E-56	SLC25A31	2.53E-01	1.00E+00	CCNT2	-1.57E-01	1.00E+00
GADD45A	-3.04E+00	2.94E-56	CROCC	4.40E-01	1.00E+00	FOXP2	-1.85E-01	1.00E+00
LOC102400274	-3.43E+00	3.16E-56	DEXI	-2.04E-01	1.00E+00	SELENBP1	-2.20E-01	1.00E+00
CFLAR	-2.59E+00	3.78E-56	AGK	1.29E-01	1.00E+00	LOC102392841	-2.31E-01	1.00E+00
AMD1	1.71E+00	3.88E-56	NOB1	-1.35E-01	1.00E+00	TRMT5	2.15E-01	1.00E+00
LOC102396103	1.65E+00	3.88E-56	UBALD2	1.71E-01	1.00E+00	ETFA	-1.54E-01	1.00E+00
ZMAT3	1.64E+00	4.38E-56	LOC112583748	4.00E-01	1.00E+00	EIF2B3	1.66E-01	1.00E+00
CBARP	-2.73E+00	4.45E-56	TSC22D3	-2.85E-01	1.00E+00	AP5S1	1.85E-01	1.00E+00
MARCH9	1.66E+00	4.48E-56	DYNC1I1	-1.65E-01	1.00E+00	SLC46A3	2.66E-01	1.00E+00
CDC20B	4.76E+00	4.58E-56	ADGRL2	2.16E-01	1.00E+00	FGF11	2.35E-01	1.00E+00
MAPT	3.30E+00	4.67E-56	RANBP6	-4.53E-01	1.00E+00	PRELID2	-1.66E-01	1.00E+00
MYCBPAP	-2.98E+00	4.67E-56	LOC112585729	-5.37E-01	1.00E+00	RGS20	4.15E-01	1.00E+00
HCFC1R1	-3.04E+00	4.68E-56	LOC102409686	-1.54E-01	1.00E+00	HACD3	1.24E-01	1.00E+00
PXDN	-4.86E+00	4.94E-56	MADD	1.32E-01	1.00E+00	LOC112582992	2.00E-01	1.00E+00
CDKN1B	-2.07E+00	5.10E-56	RTEL1	1.50E-01	1.00E+00	TM9SF4	-1.20E-01	1.00E+00
MOV10	-2.21E+00	5.55E-56	SF3A1	9.53E-02	1.00E+00	USP12	1.76E-01	1.00E+00

CIB3	6.07E+00	5.93E-56	TTLL5	1.34E-01	1.00E+00	CCDC103	-3.04E-01	1.00E+00
FAT1	-3.21E+00	5.94E-56	MUC4	-1.91E-01	1.00E+00	C17H4orf46	-1.75E-01	1.00E+00
SLC2A13	-3.64E+00	5.98E-56	PNLDC1	1.15E-01	1.00E+00	ABHD17A	1.57E-01	1.00E+00
TXK	2.73E+00	6.55E-56	TAF1A	1.70E-01	1.00E+00	DNAAF2	-1.75E-01	1.00E+00
MC4R	7.47E+00	6.61E-56	NOTCH3	5.51E-01	1.00E+00	TMED7	-1.31E-01	1.00E+00
ELOVL2	4.77E+00	7.08E-56	ETV4	-2.31E-01	1.00E+00	CCDC14	-1.61E-01	1.00E+00
PHF24	2.48E+00	7.35E-56	PLEKHA1	1.16E-01	1.00E+00	EIF4A1	1.96E-01	1.00E+00
CHAT	6.38E+00	7.46E-56	PRRC2A	1.39E-01	1.00E+00	LOC102399152	-2.59E-01	1.00E+00
TRUB2	-2.42E+00	7.52E-56	TARDBP	1.53E-01	1.00E+00	ZBTB49	-1.41E-01	1.00E+00
TBC1D25	1.63E+00	8.15E-56	HOXA4	1.63E-01	1.00E+00	METTL17	-1.51E-01	1.00E+00
FAM57A	-2.55E+00	8.62E-56	ZBED4	1.27E-01	1.00E+00	UBAC1	1.48E-01	1.00E+00
HHEX	3.73E+00	9.42E-56	ABCC10	2.33E-01	1.00E+00	TRMT10B	-1.73E-01	1.00E+00
LOC102390086	-3.87E+00	9.42E-56	RPS6KL1	2.40E-01	1.00E+00	CDC42SE1	1.54E-01	1.00E+00
LOC102389985	3.32E+00	1.01E-55	ZFR	1.39E-01	1.00E+00	FAM198A	3.03E-01	1.00E+00
SLC15A4	1.45E+00	1.02E-55	XKR8	1.99E-01	1.00E+00	SMOC1	-2.07E-01	1.00E+00
LOC102399263	-2.10E+00	1.05E-55	SLC16A13	1.09E-01	1.00E+00	ATP7B	-1.59E-01	1.00E+00
OSBPL1A	1.90E+00	1.06E-55	LOC102410286	4.90E-01	1.00E+00	CACNB3	1.36E-01	1.00E+00
AHI1	-2.78E+00	1.09E-55	ATXN7L2	2.25E-01	1.00E+00	OLFML3	3.36E-01	1.00E+00
TSR1	1.73E+00	1.13E-55	MYCBP2	2.02E-01	1.00E+00	SPACA9	-2.61E-01	1.00E+00
AMH	-6.13E+00	1.21E-55	PLB1	-1.44E-01	1.00E+00	FBLN1	-1.78E-01	1.00E+00
GABPA	-2.23E+00	1.35E-55	C22H18orf54	2.07E-01	1.00E+00	UGDH	1.55E-01	1.00E+00
PTPRD	2.10E+00	1.57E-55	RAC1	-1.05E-01	1.00E+00	PDHX	-1.35E-01	1.00E+00
CPSF1	-1.68E+00	1.61E-55	DERL3	-1.82E-01	1.00E+00	FAR2	2.98E-01	1.00E+00
BCL7B	1.56E+00	1.62E-55	DYNC1LI2	1.66E-01	1.00E+00	DYNC1LI1	-1.72E-01	1.00E+00
CD82	-4.08E+00	1.63E-55	ATP6V0A4	1.16E-01	1.00E+00	RNMT	-1.28E-01	1.00E+00
LOC102406463	7.42E+00	1.68E-55	USP30	1.87E-01	1.00E+00	LOC102396318	-1.93E-01	1.00E+00
AVPI1	-2.60E+00	1.70E-55	ANAPC1	1.39E-01	1.00E+00	ITGAX	5.97E-01	1.00E+00
SMC2	2.07E+00	1.70E-55	PARD3	-1.31E-01	1.00E+00	SRSF10	-1.68E-01	1.00E+00
PHF7	3.31E+00	1.73E-55	PDCD2	-1.68E-01	1.00E+00	ZNF599	2.78E-01	1.00E+00
LOC102399631	1.02E+01	1.73E-55	LOC102405614	2.23E-01	1.00E+00	MFHAS1	1.44E-01	1.00E+00
KIFC3	2.91E+00	1.76E-55	LOC112577704	6.94E-01	1.00E+00	RELB	2.42E-01	1.00E+00
PEBP1	-2.55E+00	1.96E-55	TMEM254	2.50E-01	1.00E+00	CCT7	1.68E-01	1.00E+00
ACTR6	1.65E+00	2.01E-55	RBMXL2	-1.99E-01	1.00E+00	CDKL4	3.13E-01	1.00E+00
LOC102389538	-5.27E+00	2.09E-55	LOC102402635	5.04E-01	1.00E+00	DIS3L	-1.29E-01	1.00E+00

MTMR12	2.02E+00	2.11E-55	MTCH2	-1.09E-01	1.00E+00	LOC102400242	3.31E-01	1.00E+00
GLMP	-1.66E+00	2.16E-55	PDCD10	2.46E-01	1.00E+00	NLRX1	-2.27E-01	1.00E+00
DMWD	1.63E+00	2.17E-55	PLEKHB2	-1.02E-01	1.00E+00	IFI35	2.02E-01	1.00E+00
PLEKHA5	-2.60E+00	2.26E-55	PANK4	-2.43E-01	1.00E+00	ROCK2	1.79E-01	1.00E+00
GABRG1	6.80E+00	2.44E-55	ATAD2	-2.03E-01	1.00E+00	LOC112583918	1.91E-01	1.00E+00
GLCCI1	-2.28E+00	2.48E-55	LOC102407546	-1.33E-01	1.00E+00	RREB1	-1.52E-01	1.00E+00
PLRG1	1.39E+00	2.67E-55	METTL5	-2.49E-01	1.00E+00	CBX2	1.99E-01	1.00E+00
CTBP1	-2.14E+00	2.76E-55	PPIH	-1.49E-01	1.00E+00	RAI14	1.60E-01	1.00E+00
LOC102403205	2.41E+00	2.76E-55	BCL7A	2.02E-01	1.00E+00	EMC1	-1.51E-01	1.00E+00
LOC112578199	4.89E+00	2.83E-55	LIN37	-1.51E-01	1.00E+00	DERL1	-1.36E-01	1.00E+00
PLPP3	-4.81E+00	2.86E-55	KHSRP	1.52E-01	1.00E+00	GVQW3	-3.38E-01	1.00E+00
LOC102400319	8.38E+00	2.94E-55	LOC102394362	-1.27E-01	1.00E+00	AK5	1.65E-01	1.00E+00
LOC102403514	5.36E+00	3.16E-55	ZNF503	-1.91E-01	1.00E+00	SCN2A	3.41E-01	1.00E+00
KLHDC10	-1.66E+00	3.21E-55	ZADH2	-4.81E-01	1.00E+00	PPP1R3B	2.13E-01	1.00E+00
MXRA8	-7.02E+00	3.24E-55	UBE2T	1.45E-01	1.00E+00	OPTN	-1.23E-01	1.00E+00
BCKDK	-2.05E+00	3.25E-55	MB21D2	1.01E-01	1.00E+00	DMAC1	1.84E-01	1.00E+00
NCS1	-5.25E+00	3.28E-55	THSD4	1.82E-01	1.00E+00	PLIN3	1.83E-01	1.00E+00
INTS12	1.45E+00	3.38E-55	CCR7	6.66E-01	1.00E+00	PIAS2	-1.32E-01	1.00E+00
MED12L	2.79E+00	3.54E-55	PKP4	1.13E-01	1.00E+00	NXN	1.78E-01	1.00E+00
PFKP	-4.64E+00	3.69E-55	DLD	-1.27E-01	1.00E+00	CD302	3.80E-01	1.00E+00
LOC102414721	-3.88E+00	3.70E-55	LOC102394014	-3.33E-01	1.00E+00	GMPPA	-1.48E-01	1.00E+00
LOC102397238	1.77E+00	3.77E-55	MRM2	-3.12E-01	1.00E+00	TRIOBP	1.31E-01	1.00E+00
METTL18	2.12E+00	3.89E-55	LOC102394187	2.38E-01	1.00E+00	RBFOX3	-2.94E-01	1.00E+00
RPL35	-3.65E+00	3.97E-55	LOC112581824	-2.38E-01	1.00E+00	SLC25A39	-1.77E-01	1.00E+00
GNB1L	-2.79E+00	4.01E-55	LOC112580810	-1.59E-01	1.00E+00	CAV1	3.35E-01	1.00E+00
CHDH	6.42E+00	4.16E-55	PTOV1	-1.23E-01	1.00E+00	SPATA13	3.19E-01	1.00E+00
DDX51	-2.52E+00	4.34E-55	MMADHC	-1.39E-01	1.00E+00	DLGAP5	-1.81E-01	1.00E+00
KHDRBS1	-1.63E+00	4.79E-55	KIF2A	1.17E-01	1.00E+00	SEC11A	-1.73E-01	1.00E+00
FAM161A	1.58E+00	4.89E-55	LOC112581113	-5.17E-01	1.00E+00	CCR8	1.86E-01	1.00E+00
CAPN15	-2.41E+00	4.97E-55	CHRNB2	-3.92E-01	1.00E+00	CLEC12B	3.54E-01	1.00E+00
JADE3	1.47E+00	5.05E-55	C6H1orf54	-6.05E-01	1.00E+00	BTD	-1.28E-01	1.00E+00
MEX3A	-2.97E+00	5.27E-55	NEDD1	1.12E-01	1.00E+00	CDT1	-1.88E-01	1.00E+00
ADAMTSL1	3.25E+00	5.37E-55	UBL5	-1.51E-	1.00E+00	LMO7	3.70E-01	1.00E+00

					01						
SUMO3	-1.78E+00	5.44E-55		FHL3	-8.02E-01	1.00E+00		TMEM120A	-1.91E-01	1.00E+00	
HSPG2	-5.69E+00	5.52E-55		CPA6	1.35E-01	1.00E+00		SHQ1	1.43E-01	1.00E+00	
TMEM174	9.10E+00	5.53E-55		FBXL2	-2.59E-01	1.00E+00		PANK1	-1.53E-01	1.00E+00	
NAA16	-2.46E+00	6.12E-55		PIGQ	1.63E-01	1.00E+00		SLC9C2	3.37E-01	1.00E+00	
LOC102413072	-6.26E+00	6.15E-55		AFAP1L2	1.32E-01	1.00E+00		SH3BP1	2.30E-01	1.00E+00	
SPARC	-7.23E+00	6.31E-55		TDRD6	-2.27E-01	1.00E+00		MYCBPAP	-1.43E-01	1.00E+00	
PPP6R1	-1.76E+00	6.52E-55		LOC112579728	-1.74E-01	1.00E+00		LOC102403501	2.16E-01	1.00E+00	
BAHCC1	-2.34E+00	6.73E-55		CRTAC1	-2.80E-01	1.00E+00		RIN3	2.12E-01	1.00E+00	
CACNG1	8.75E+00	6.79E-55		RACK1	-1.21E-01	1.00E+00		KIF18A	-1.87E-01	1.00E+00	
UPRT	1.39E+00	6.80E-55		PROM1	8.06E-01	1.00E+00		TMEM242	-1.53E-01	1.00E+00	
PABPC4L	-5.48E+00	6.84E-55		SLC16A1	-1.27E-01	1.00E+00		ZCCHC4	1.70E-01	1.00E+00	
RAPGEF3	2.50E+00	6.86E-55		RABIF	2.11E-01	1.00E+00		SRCAP	-1.55E-01	1.00E+00	
CRABP2	-7.06E+00	7.15E-55		DUS3L	1.18E-01	1.00E+00		LOC112585146	2.12E-01	1.00E+00	
MRPL17	-2.31E+00	7.37E-55		GGT6	4.08E-01	1.00E+00		RHPN1	-1.81E-01	1.00E+00	
GTPBP6	-3.45E+00	7.70E-55		LOC112578564	-6.11E-01	1.00E+00		LOC102391418	2.33E-01	1.00E+00	
GID4	1.50E+00	7.74E-55		TCEA3	5.39E-01	1.00E+00		HARBI1	1.64E-01	1.00E+00	
NAA25	-2.27E+00	7.95E-55		C8H7orf31	-1.89E-01	1.00E+00		SLC2A4	-2.81E-01	1.00E+00	
VAMP5	-6.15E+00	8.00E-55		GBGT1	2.39E-01	1.00E+00		SLC30A10	-3.63E-01	1.00E+00	
CAMSAP1	2.19E+00	8.58E-55		ITGAL	-5.65E-01	1.00E+00		AFAP1L2	2.77E-01	1.00E+00	
SPEF2	-3.86E+00	8.86E-55		CENPF	1.95E-01	1.00E+00		TBC1D23	-1.53E-01	1.00E+00	
HEATR4	3.55E+00	9.59E-55		APMAP	1.32E-01	1.00E+00		CACNA1H	1.87E-01	1.00E+00	
DDX4	7.90E+00	9.95E-55		RSPH10B	-4.00E-01	1.00E+00		LOC102393147	1.56E-01	1.00E+00	
PIGG	-5.16E+00	1.02E-54		FRMD3	1.05E-01	1.00E+00		FMO5	-1.25E-01	1.00E+00	
PRRG2	2.62E+00	1.07E-54		LOC102389800	-2.68E-01	1.00E+00		LOC112587527	-3.38E-01	1.00E+00	
BAIAP3	2.92E+00	1.08E-54		SPTAN1	1.68E-01	1.00E+00		CCDC69	-2.12E-01	1.00E+00	
NMT1	-1.50E+00	1.09E-54		LOC102414507	-2.13E-01	1.00E+00		KCTD12	2.45E-01	1.00E+00	
LOC102402068	-2.15E+00	1.10E-54		XPA	-1.83E-01	1.00E+00		IGF2	2.13E-01	1.00E+00	
BLVRB	-7.22E+00	1.16E-54		SPESP1	-1.07E-01	1.00E+00		MUL1	1.49E-01	1.00E+00	
LOC102404071	7.12E+00	1.21E-54		ADGRL3	1.88E-01	1.00E+00		ECH1	-1.69E-01	1.00E+00	
ANKRD27	1.46E+00	1.31E-54		PATL1	1.30E-01	1.00E+00		C24H7orf50	1.68E-01	1.00E+00	
LOC112580873	5.00E+00	1.32E-54		ETFBKMT	6.00E-01	1.00E+00		SARS	1.43E-01	1.00E+00	
FKBP5	-2.10E+00	1.34E-54		FES	-2.95E-01	1.00E+00		CLYBL	-1.66E-01	1.00E+00	
TNFAIP8	2.29E+00	1.37E-54		MORN2	-2.86E-01	1.00E+00		EPS8L2	-3.37E-01	1.00E+00	

LOC102400616	8.18E+00	1.44E-54	BRDT	1.43E-01	1.00E+00	CNIH2	2.82E-01	1.00E+00
RPL24	-2.61E+00	1.46E-54	SOD1	-1.10E-01	1.00E+00	SKA1	-1.80E-01	1.00E+00
TFAP2A	5.34E+00	1.69E-54	LOC102396414	-3.60E-01	1.00E+00	MLLT6	1.52E-01	1.00E+00
FAM204A	-1.78E+00	1.73E-54	TOP2A	1.89E-01	1.00E+00	LOC102394968	3.58E-01	1.00E+00
FYN	1.52E+00	1.73E-54	CCSAP	1.42E-01	1.00E+00	CASP8	3.32E-01	1.00E+00
DTX3	-1.66E+00	1.73E-54	NUMA1	1.58E-01	1.00E+00	DUSP10	-3.19E-01	1.00E+00
RHPN1	-4.90E+00	1.75E-54	FAM92B	-5.92E-01	1.00E+00	ARHGEF11	-1.38E-01	1.00E+00
NES	-3.44E+00	2.02E-54	DIDO1	2.44E-01	1.00E+00	SIPA1L2	-1.54E-01	1.00E+00
IFT74	1.45E+00	2.09E-54	ERAL1	1.32E-01	1.00E+00	ELMOD2	1.56E-01	1.00E+00
LOC102394731	8.88E+00	2.16E-54	TXLNA	1.22E-01	1.00E+00	COL15A1	-4.29E-01	1.00E+00
CCDC47	-1.76E+00	2.16E-54	FAM149B1	1.22E-01	1.00E+00	TBC1D5	-1.27E-01	1.00E+00
WDR34	-2.57E+00	2.28E-54	PRR30	5.79E-01	1.00E+00	ANO8	1.63E-01	1.00E+00
LOC102414331	2.47E+00	2.29E-54	SRSF3	-1.30E-01	1.00E+00	TAF12	1.62E-01	1.00E+00
GM2A	-3.08E+00	2.29E-54	ARVCF	-5.19E-01	1.00E+00	SSBP3	1.32E-01	1.00E+00
NAT9	-3.03E+00	2.30E-54	LCOR	1.77E-01	1.00E+00	NUP153	-1.70E-01	1.00E+00
DNA2	2.75E+00	2.33E-54	C2CD2	1.69E-01	1.00E+00	SPATA2L	3.00E-01	1.00E+00
ADAMTSL4	-3.28E+00	2.35E-54	LOC102407989	-3.21E-01	1.00E+00	CHPF	-1.97E-01	1.00E+00
SPERT	5.95E+00	2.39E-54	PRDM11	-3.87E-01	1.00E+00	JADE1	-1.72E-01	1.00E+00
LOC112580623	-6.31E+00	2.49E-54	IDUA	-7.84E-01	1.00E+00	GABBR1	-1.36E-01	1.00E+00
GPAM	-2.85E+00	2.51E-54	KXD1	2.21E-01	1.00E+00	RAP2C	1.47E-01	1.00E+00
TOLLIP	1.60E+00	2.54E-54	LIMK2	1.02E-01	1.00E+00	FBH1	-1.26E-01	1.00E+00
SYN2	-3.81E+00	2.54E-54	OTX2	-5.67E-01	1.00E+00	SYT3	-2.63E-01	1.00E+00
ITGA7	-5.39E+00	2.54E-54	RPS10	-1.59E-01	1.00E+00	FEM1A	1.22E-01	1.00E+00
STXBP5	-2.37E+00	2.57E-54	RNF207	-5.12E-01	1.00E+00	KHDRBS1	-1.29E-01	1.00E+00
PDXDC1	1.74E+00	2.66E-54	SYN1	-1.72E-01	1.00E+00	POLG	-1.23E-01	1.00E+00
C5H11orf54	-4.39E+00	2.77E-54	TSPAN33	1.58E-01	1.00E+00	SYK	6.57E-01	1.00E+00
RPL9	-3.77E+00	2.83E-54	CACYBP	-1.20E-01	1.00E+00	TMEM220	1.92E-01	1.00E+00
GADD45B	-2.54E+00	2.83E-54	BAIAP3	-2.06E-01	1.00E+00	BLOC1S6	-1.33E-01	1.00E+00
POP5	-3.42E+00	2.88E-54	PHF5A	-2.83E-01	1.00E+00	RNF19B	-1.95E-01	1.00E+00
LPIN2	2.07E+00	3.13E-54	SYVN1	1.04E-01	1.00E+00	LOC102406999	-1.40E-01	1.00E+00
URI1	-1.66E+00	3.20E-54	UBASH3A	1.08E-01	1.00E+00	TAAR1	3.52E-01	1.00E+00
PRKAR2B	-5.77E+00	3.23E-54	COG1	1.29E-01	1.00E+00	SAE1	-1.49E-01	1.00E+00
RNF168	2.14E+00	3.26E-54	COMMD9	-1.50E-01	1.00E+00	SIVA1	-2.25E-01	1.00E+00
LHPP	-	3.32E-54	ZCWPW2	-2.01E-	1.00E+00	TXNDC9	1.38E-01	1.00E+00

	2.44E+00			01				
TXN	-2.46E+00	3.38E-54	C11H15orf48	-2.73E-01	1.00E+00	ECHDC2	-2.12E-01	1.00E+00
PLXNA1	-2.12E+00	3.66E-54	LOC112585253	4.50E-01	1.00E+00	PAPOLA	-1.34E-01	1.00E+00
CDCA7	1.97E+00	3.70E-54	RNF31	1.01E-01	1.00E+00	LOC102416294	2.96E-01	1.00E+00
HEBP1	-2.40E+00	3.71E-54	RDM1	-3.18E-01	1.00E+00	MUC1	-2.93E-01	1.00E+00
CGGBP1	-1.54E+00	3.73E-54	ACTN4	1.18E-01	1.00E+00	OSM	-5.43E-01	1.00E+00
SNTB1	3.22E+00	3.90E-54	WDPCP	-1.29E-01	1.00E+00	MTMR7	2.39E-01	1.00E+00
ATP4B	-7.44E+00	3.92E-54	ATF6	9.92E-02	1.00E+00	MYCN	-2.54E-01	1.00E+00
LOC102405950	-5.99E+00	3.94E-54	LOC112577712	2.80E-01	1.00E+00	LOC102397911	-2.61E-01	1.00E+00
RHOB	-4.90E+00	3.97E-54	SMIM10L1	2.53E-01	1.00E+00	SLC52A2	-1.50E-01	1.00E+00
ACACA	-1.79E+00	4.16E-54	DLGAP1	-1.16E-01	1.00E+00	SLC28A1	3.02E-01	1.00E+00
LOC112585064	6.11E+00	4.16E-54	LRFN1	-1.90E-01	1.00E+00	ATXN1	-1.65E-01	1.00E+00
GUCY2C	-4.27E+00	4.27E-54	ABCE1	-1.43E-01	1.00E+00	ALDH7A1	-1.40E-01	1.00E+00
PALD1	3.57E+00	4.34E-54	AK4	1.11E-01	1.00E+00	ZBED5	1.45E-01	1.00E+00
RMDN3	1.41E+00	4.52E-54	TMEM176A	-5.89E-01	1.00E+00	MAMSTR	-3.02E-01	1.00E+00
LOC102400341	-3.57E+00	4.60E-54	SP8	-6.39E-01	1.00E+00	STK32C	3.15E-01	1.00E+00
MICAL1	2.08E+00	4.66E-54	SMIM3	-3.13E-01	1.00E+00	CIPC	1.54E-01	1.00E+00
ZNF644	-2.30E+00	4.69E-54	PDCD6IP	1.08E-01	1.00E+00	ATP23	1.46E-01	1.00E+00
SZT2	-2.24E+00	4.70E-54	MRPL48	-1.81E-01	1.00E+00	GNG7	2.20E-01	1.00E+00
GATD1	-2.44E+00	5.06E-54	ANGPTL6	-2.48E-01	1.00E+00	NEIL3	-2.12E-01	1.00E+00
LOC112577795	-2.84E+00	5.11E-54	ELK1	1.23E-01	1.00E+00	LOC102388979	1.98E-01	1.00E+00
SP140	-5.11E+00	5.27E-54	LOC102414187	-1.27E-01	1.00E+00	ACTR3B	-1.49E-01	1.00E+00
HTATSF1	-1.61E+00	5.53E-54	ADAMTS14	1.93E-01	1.00E+00	PRX	1.47E-01	1.00E+00
GPD1	-5.40E+00	5.55E-54	HSPA2	9.32E-02	1.00E+00	MACO1	-1.66E-01	1.00E+00
LOC102411606	1.85E+00	5.78E-54	CSDC2	-4.08E-01	1.00E+00	CDKN2AIP	-1.76E-01	1.00E+00
ZDHHC13	1.58E+00	6.06E-54	TRAPPC11	1.21E-01	1.00E+00	TRIM66	-2.92E-01	1.00E+00
ATOH8	-6.11E+00	6.25E-54	LOC112586835	5.25E-01	1.00E+00	VGLL2	-3.09E-01	1.00E+00
RAB13	-3.89E+00	6.32E-54	SH2D5	-2.07E-01	1.00E+00	LOC102413305	-1.41E-01	1.00E+00
FZD6	-4.96E+00	6.35E-54	LOC112586914	2.84E-01	1.00E+00	SHPK	3.03E-01	1.00E+00
SERINC4	-4.54E+00	6.44E-54	OSBPL1A	-1.40E-01	1.00E+00	TEX9	-1.61E-01	1.00E+00
KPNA1	-1.68E+00	6.64E-54	LOC112585064	-3.10E-01	1.00E+00	PRKCA	1.50E-01	1.00E+00
RCAN1	-3.28E+00	6.76E-54	EIF4E2	-9.74E-02	1.00E+00	PANK4	-1.24E-01	1.00E+00
HEBP2	-6.75E+00	6.79E-54	CRACR2A	-2.77E-01	1.00E+00	HMGB2	-2.10E-01	1.00E+00

RNF19A	-2.06E+00	6.94E-54	LOC102397853	-3.07E-01	1.00E+00	CTSD	2.13E-01	1.00E+00
LOC112580241	5.88E+00	7.01E-54	HAUS7	1.51E-01	1.00E+00	ACSS3	-1.40E-01	1.00E+00
LOC112579925	4.82E+00	7.51E-54	MAP3K4	1.47E-01	1.00E+00	ZFP36	-3.32E-01	1.00E+00
TDRD15	8.79E+00	7.64E-54	HOXA1	1.39E-01	1.00E+00	ZNF74	1.36E-01	1.00E+00
AP4M1	-2.21E+00	7.66E-54	PC	1.22E-01	1.00E+00	EIF4A2	-1.77E-01	1.00E+00
ZSCAN10	8.24E+00	8.03E-54	MLH3	1.93E-01	1.00E+00	FSCN1	2.11E-01	1.00E+00
PPP1R3D	3.36E+00	8.88E-54	MRC1	-2.12E-01	1.00E+00	SARAF	-1.30E-01	1.00E+00
TCEA2	-2.74E+00	9.03E-54	CHPT1	2.16E-01	1.00E+00	SLC25A21	3.01E-01	1.00E+00
MED27	1.67E+00	9.06E-54	LAMTOR2	-1.83E-01	1.00E+00	PCK2	-1.89E-01	1.00E+00
PCDHB1	7.76E+00	9.37E-54	TDRKH	1.12E-01	1.00E+00	SCG2	-4.00E-01	1.00E+00
EEF1D	-3.25E+00	9.71E-54	NIPA1	3.55E-01	1.00E+00	CDH13	2.90E-01	1.00E+00
FAF1	-1.57E+00	9.83E-54	LOC102389249	1.32E-01	1.00E+00	CKAP4	-1.47E-01	1.00E+00
FNDC11	-6.26E+00	1.06E-53	ATXN3	1.64E-01	1.00E+00	ERI3	-1.65E-01	1.00E+00
GRN	-6.10E+00	1.07E-53	LOC112585732	-4.09E-01	1.00E+00	PCNP	-3.99E-01	1.00E+00
TRIP12	2.00E+00	1.09E-53	TRIM65	1.93E-01	1.00E+00	ATP9A	-1.95E-01	1.00E+00
DLG4	-2.29E+00	1.11E-53	FIBP	-1.32E-01	1.00E+00	GAL3ST3	2.86E-01	1.00E+00
SDC1	-6.92E+00	1.14E-53	CEP83	1.15E-01	1.00E+00	NASP	-1.54E-01	1.00E+00
GAS7	6.35E+00	1.18E-53	PLCXD3	-4.26E-01	1.00E+00	STOX2	2.56E-01	1.00E+00
LOC102396279	5.22E+00	1.19E-53	MAFA	3.02E-01	1.00E+00	LOC102407177	1.92E-01	1.00E+00
MAPK10	1.71E+00	1.22E-53	ZNF582	3.68E-01	1.00E+00	OSBPL6	1.63E-01	1.00E+00
OAZ2	1.72E+00	1.28E-53	CCDC18	-2.19E-01	1.00E+00	GRB14	-1.98E-01	1.00E+00
FAH	-6.37E+00	1.42E-53	HAVCR2	2.76E-01	1.00E+00	RAD17	-1.39E-01	1.00E+00
KCNE5	-7.23E+00	1.48E-53	PYM1	-1.20E-01	1.00E+00	SGSM3	1.43E-01	1.00E+00
MMP14	-6.80E+00	1.48E-53	STX1A	1.29E-01	1.00E+00	OBSCN	4.27E-01	1.00E+00
TTF2	1.55E+00	1.48E-53	TSPAN4	3.71E-01	1.00E+00	PORCN	1.84E-01	1.00E+00
DHRSX	-2.48E+00	1.51E-53	RAB39B	-1.93E-01	1.00E+00	LOC102399924	-2.05E-01	1.00E+00
PIGL	-3.95E+00	1.53E-53	ABCA3	2.75E-01	1.00E+00	CCDC17	-2.35E-01	1.00E+00
LOC102413663	6.49E+00	1.59E-53	OSBP	1.16E-01	1.00E+00	XYLT1	1.91E-01	1.00E+00
LOC112584654	6.14E+00	1.59E-53	UPF3B	-1.75E-01	1.00E+00	YARS	1.30E-01	1.00E+00
SLC41A3	-2.76E+00	1.67E-53	NANS	3.62E-01	1.00E+00	LOC102402689	1.65E-01	1.00E+00
DAG1	-2.54E+00	1.67E-53	ING2	-2.78E-01	1.00E+00	REC114	2.38E-01	1.00E+00
TMEM222	-1.87E+00	1.69E-53	SEC23B	9.98E-02	1.00E+00	TAS1R3	-2.70E-01	1.00E+00
SAMD11	-5.24E+00	1.76E-53	GABRA2	9.83E-01	1.00E+00	MAVS	1.38E-01	1.00E+00
PSMD2	-1.58E+00	1.76E-53	EBAG9	-2.17E-01	1.00E+00	USP43	-2.01E-01	1.00E+00

RNF24	2.76E+00	1.86E-53	GPATCH11	-1.76E-01	1.00E+00	GLUD1	-1.23E-01	1.00E+00
NSD1	-2.34E+00	1.93E-53	JSRP1	8.21E-01	1.00E+00	MAEA	-1.23E-01	1.00E+00
TMEM168	1.60E+00	1.98E-53	WDR19	9.79E-02	1.00E+00	CDH5	-3.74E-01	1.00E+00
LOC102399617	-2.71E+00	2.07E-53	SPATA2L	-2.17E-01	1.00E+00	LOC102410803	-2.08E-01	1.00E+00
IGSF11	-1.56E+00	2.08E-53	TBXT	-4.73E-01	1.00E+00	RMDN1	-1.44E-01	1.00E+00
RAPGEF1	1.77E+00	2.14E-53	CTNNAL1	-1.25E-01	1.00E+00	TET3	2.17E-01	1.00E+00
LOC112587835	6.17E+00	2.16E-53	ACIN1	1.26E-01	1.00E+00	AP5B1	1.63E-01	1.00E+00
LOC102405773	-5.81E+00	2.23E-53	GNPDA1	-3.59E-01	1.00E+00	YDJC	3.00E-01	1.00E+00
RAB2B	1.39E+00	2.25E-53	LOC102402536	-6.59E-01	1.00E+00	MBTPS1	-1.44E-01	1.00E+00
YTHDC1	-1.50E+00	2.25E-53	TRPA1	2.41E-01	1.00E+00	ADSSL1	-1.89E-01	1.00E+00
TMEM268	-2.14E+00	2.28E-53	FANCD2	1.19E-01	1.00E+00	NPR3	2.53E-01	1.00E+00
FANCG	-2.14E+00	2.29E-53	URM1	-1.51E-01	1.00E+00	LOC112586999	2.77E-01	1.00E+00
LOC112584673	7.63E+00	2.29E-53	POLR2M	1.12E-01	1.00E+00	TCEA3	-2.20E-01	1.00E+00
SNRNP27	1.85E+00	2.35E-53	RFLNB	1.81E-01	1.00E+00	MDM1	-1.93E-01	1.00E+00
LOC112581602	8.43E+00	2.40E-53	PGM1	1.61E-01	1.00E+00	B9D2	1.98E-01	1.00E+00
MYH9	-2.46E+00	2.59E-53	CIPC	1.22E-01	1.00E+00	NRDC	-1.17E-01	1.00E+00
BNC2	1.94E+00	2.65E-53	TMEM87B	-1.66E-01	1.00E+00	ZNF282	-1.22E-01	1.00E+00
CD37	-3.71E+00	2.71E-53	BEX3	-2.73E-01	1.00E+00	ALDH1A3	-5.87E-01	1.00E+00
BTBD11	-3.64E+00	2.91E-53	ECD	-1.14E-01	1.00E+00	AKT1	1.37E-01	1.00E+00
ZFP62	-3.26E+00	2.94E-53	LMBR1	1.29E-01	1.00E+00	ZNF444	1.44E-01	1.00E+00
TEX2	1.82E+00	3.01E-53	LOC112586985	-3.90E-01	1.00E+00	BCAM	1.34E-01	1.00E+00
ADGRG7	5.91E+00	3.08E-53	PEX6	-1.66E-01	1.00E+00	NEIL2	-1.79E-01	1.00E+00
DYNC1I2	1.80E+00	3.09E-53	LOC102400624	-1.76E-01	1.00E+00	EXOC3L1	-2.33E-01	1.00E+00
WEE1	-4.15E+00	3.14E-53	PPP2R5E	1.05E-01	1.00E+00	CACNA1G	3.43E-01	1.00E+00
LRMDA	3.15E+00	3.14E-53	ETV5	1.26E-01	1.00E+00	COPG1	-1.39E-01	1.00E+00
RFC2	-2.52E+00	3.75E-53	LOC112584743	-5.01E-01	1.00E+00	PTCD3	-1.18E-01	1.00E+00
DDR1	-1.82E+00	3.80E-53	ZYX	-1.92E-01	1.00E+00	VARS	1.29E-01	1.00E+00
LEMD2	-1.95E+00	3.89E-53	RGS16	1.18E-01	1.00E+00	FLVCR1	-2.99E-01	1.00E+00
LOC102394921	3.89E+00	4.03E-53	ZNF687	1.53E-01	1.00E+00	SLC35C2	1.54E-01	1.00E+00
COL3A1	-9.38E+00	4.27E-53	TROAP	1.01E-01	1.00E+00	PSIP1	-1.29E-01	1.00E+00
VPS36	-2.29E+00	4.30E-53	TEDC2	1.19E-01	1.00E+00	TMEM163	-2.49E-01	1.00E+00
CALR	-3.55E+00	4.38E-53	LOC102411164	-1.63E-01	1.00E+00	LOC102405095	-2.15E-01	1.00E+00
FRY	1.64E+00	4.39E-53	DPYD	-5.18E-01	1.00E+00	VIPAS39	1.16E-01	1.00E+00

LOC102398213	-2.43E+00	4.57E-53	HYAL1	6.65E-01	1.00E+00	KIAA0586	1.39E-01	1.00E+00
SEPT4	-4.66E+00	4.75E-53	CEP55	-1.38E-01	1.00E+00	EPHX2	1.72E-01	1.00E+00
TBX18	5.82E+00	4.84E-53	TTLL11	-2.26E-01	1.00E+00	RHOT1	-1.30E-01	1.00E+00
ZSCAN2	-2.93E+00	5.06E-53	MGP	-4.53E-01	1.00E+00	ARL6IP1	-1.46E-01	1.00E+00
IPP	1.73E+00	5.22E-53	FSTL4	-1.54E-01	1.00E+00	IRAK3	3.70E-01	1.00E+00
SMIM17	4.96E+00	5.22E-53	STAG1	-1.82E-01	1.00E+00	LOC102399242	2.78E-01	1.00E+00
MAL2	2.33E+00	5.26E-53	ZSWIM4	2.35E-01	1.00E+00	TTLL7	1.66E-01	1.00E+00
LOC102400440	-1.51E+00	5.78E-53	MSMO1	-1.16E-01	1.00E+00	UMPS	1.45E-01	1.00E+00
TMC6	-6.51E+00	5.93E-53	FBXO48	-5.71E-01	1.00E+00	LOC112583888	1.86E-01	1.00E+00
AP5Z1	-2.74E+00	5.93E-53	SPAG4	-3.32E-01	1.00E+00	SMYD4	-1.57E-01	1.00E+00
COL5A2	-7.87E+00	6.19E-53	ITPKA	-2.44E-01	1.00E+00	RAP1A	1.24E-01	1.00E+00
SLC2A1	-4.55E+00	6.59E-53	CAPZA1	-1.16E-01	1.00E+00	HMGN4	1.40E-01	1.00E+00
LOC112580184	-7.93E+00	7.08E-53	LOC102397719	6.32E-01	1.00E+00	CFAP20	1.43E-01	1.00E+00
LOC102404549	2.55E+00	7.08E-53	STAT2	1.51E-01	1.00E+00	EZH1	-1.28E-01	1.00E+00
DNAJC10	-1.37E+00	7.09E-53	ATP5IF1	-1.42E-01	1.00E+00	NAP1L3	-2.61E-01	1.00E+00
KIF5C	5.00E+00	7.30E-53	CTNND1	1.00E-01	1.00E+00	PEX11B	2.21E-01	1.00E+00
ANKRD35	3.55E+00	7.32E-53	API5	2.24E-01	1.00E+00	COMT	-1.46E-01	1.00E+00
CACNG7	2.33E+00	7.53E-53	HDAC7	1.27E-01	1.00E+00	ZFYVE28	-3.42E-01	1.00E+00
GRIK5	-5.75E+00	7.61E-53	NRK	-1.80E-01	1.00E+00	NAIP	3.64E-01	1.00E+00
MTBP	-2.12E+00	7.63E-53	BLZF1	9.82E-02	1.00E+00	LOC102399011	-1.70E-01	1.00E+00
GTF2E2	1.61E+00	7.99E-53	POLG2	-1.12E-01	1.00E+00	SH3BGRL	1.25E-01	1.00E+00
KREMEN1	1.91E+00	7.99E-53	LOC102394049	3.60E-01	1.00E+00	MGAT4B	-1.69E-01	1.00E+00
PAX6	5.03E+00	8.32E-53	ADPRH	5.28E-01	1.00E+00	MTAP	1.35E-01	1.00E+00
LSR	-1.77E+00	8.43E-53	EPG5	1.95E-01	1.00E+00	TTN	-2.44E-01	1.00E+00
HHATL	3.51E+00	8.72E-53	BRINP3	1.40E-01	1.00E+00	USP9X	1.35E-01	1.00E+00
LOC102414936	-1.02E+01	8.83E-53	MMS19	1.23E-01	1.00E+00	FUT8	3.75E-01	1.00E+00
SPRY3	-5.80E+00	9.14E-53	CTCF	-1.25E-01	1.00E+00	RPL39	2.00E-01	1.00E+00
SERPINF1	-6.31E+00	9.16E-53	LONRF2	-3.48E-01	1.00E+00	LOC102403557	-1.82E-01	1.00E+00
ZNF365	-5.17E+00	9.21E-53	MORC4	-1.85E-01	1.00E+00	CTH	-2.09E-01	1.00E+00
SPON1	2.21E+00	9.40E-53	AP1M1	1.51E-01	1.00E+00	ALOX5AP	-2.50E-01	1.00E+00
TWF2	-2.34E+00	9.83E-53	STC1	-7.50E-01	1.00E+00	CASC1	2.62E-01	1.00E+00
CALB2	3.23E+00	9.97E-53	ATP5MPL	-1.52E-01	1.00E+00	C19H5orf34	-1.74E-01	1.00E+00
SCAMP5	4.24E+00	1.08E-52	EMC1	1.85E-01	1.00E+00	SLC35E1	-1.25E-01	1.00E+00
LOC102411911	-	1.11E-52	LOC112585719	1.77E-01	1.00E+00	LIG1	-1.64E-	1.00E+00

	1.54E+00						01	
CDK1	1.69E+00	1.11E-52	HACD3	-1.11E-01	1.00E+00	MCM4	-1.49E-01	1.00E+00
CMTM3	-2.43E+00	1.14E-52	ME1	9.34E-02	1.00E+00	CATSPER3	2.69E-01	1.00E+00
ECHDC2	2.38E+00	1.14E-52	TMEM52	1.38E-01	1.00E+00	LSP1	2.52E-01	1.00E+00
SWSAP1	2.68E+00	1.18E-52	ZC3H13	1.45E-01	1.00E+00	XAF1	1.78E-01	1.00E+00
PDE3A	6.56E+00	1.18E-52	DENND1A	1.18E-01	1.00E+00	CHD1L	-1.34E-01	1.00E+00
TRIP6	-6.26E+00	1.20E-52	ELL3	-4.63E-01	1.00E+00	INSIG2	1.73E-01	1.00E+00
MIEN1	-3.95E+00	1.21E-52	CENPB	-3.45E-01	1.00E+00	CDC14B	-2.02E-01	1.00E+00
TFDP2	-1.84E+00	1.24E-52	KALRN	1.07E-01	1.00E+00	ACOX1	1.26E-01	1.00E+00
APLP1	2.12E+00	1.31E-52	MPST	3.00E-01	1.00E+00	RUFY2	-1.61E-01	1.00E+00
RUSC2	-3.89E+00	1.31E-52	AAAS	9.09E-02	1.00E+00	ZFP36L2	-1.55E-01	1.00E+00
VWA1	-7.55E+00	1.32E-52	ANKRD40CL	-1.40E-01	1.00E+00	SET	-1.41E-01	1.00E+00
RNASET2	-1.96E+00	1.42E-52	LOC102390513	-5.55E-01	1.00E+00	TXN2	1.41E-01	1.00E+00
GOLPH3L	1.30E+00	1.46E-52	DTD1	-1.29E-01	1.00E+00	PSMB10	1.96E-01	1.00E+00
CYS1	-5.98E+00	1.55E-52	SVEP1	2.31E-01	1.00E+00	ELL2	1.55E-01	1.00E+00
LOC102389989	9.76E+00	1.57E-52	SEMA5B	-3.07E-01	1.00E+00	LOC102414453	2.82E-01	1.00E+00
MCAM	-2.20E+00	1.60E-52	POC1B	2.63E-01	1.00E+00	ZNF292	-1.58E-01	1.00E+00
HSPA1L	3.40E+00	1.60E-52	LOC102411097	4.32E-01	1.00E+00	MTHFR	-1.59E-01	1.00E+00
RASL11B	-6.41E+00	1.61E-52	FAM53A	-5.34E-01	1.00E+00	CCDC188	-2.79E-01	1.00E+00
GRASP	-5.33E+00	1.62E-52	JADE1	1.99E-01	1.00E+00	SAR1A	-1.27E-01	1.00E+00
RTL6	-2.67E+00	1.69E-52	LOC112578173	-1.75E-01	1.00E+00	NFKBIL1	1.84E-01	1.00E+00
SNX6	-1.58E+00	1.73E-52	SPATA6L	4.97E-01	1.00E+00	CNKSR2	-2.24E-01	1.00E+00
ADI1	-2.47E+00	1.74E-52	INCA1	-3.65E-01	1.00E+00	TMEM181	-1.25E-01	1.00E+00
ARAP1	-2.01E+00	1.78E-52	FCHO2	-2.23E-01	1.00E+00	MARCH11	4.21E-01	1.00E+00
ERCC1	-2.30E+00	1.79E-52	SLC40A1	1.66E-01	1.00E+00	STON1	-1.91E-01	1.00E+00
LRRC27	-2.38E+00	1.79E-52	LOC102394339	6.98E-01	1.00E+00	PTP4A2	-1.46E-01	1.00E+00
LOC102412079	-5.34E+00	1.86E-52	CHD2	1.68E-01	1.00E+00	DDR2	1.89E-01	1.00E+00
LOC112581596	8.08E+00	1.88E-52	UBXN6	1.18E-01	1.00E+00	RPA1	-1.25E-01	1.00E+00
SCARA5	-3.67E+00	1.97E-52	RMC1	1.11E-01	1.00E+00	LOC102397766	-2.70E-01	1.00E+00
POLDIP3	1.62E+00	2.02E-52	TMBIM6	9.43E-02	1.00E+00	ADD3	-1.70E-01	1.00E+00
HGFAC	-5.45E+00	2.04E-52	ATP13A3	2.52E-01	1.00E+00	FOXRED2	-2.26E-01	1.00E+00
SH3BP4	-5.87E+00	2.14E-52	SEC23IP	1.30E-01	1.00E+00	STAC	-4.24E-01	1.00E+00
KCNN4	4.65E+00	2.16E-52	AK6	-4.29E-01	1.00E+00	SENP2	-1.41E-01	1.00E+00

C5H11orf86	7.35E+00	2.25E-52	LOC102412587	-1.17E-01	1.00E+00	TP53I13	-1.89E-01	1.00E+00
ACTA2	-4.84E+00	2.30E-52	FBXL7	-6.11E-01	1.00E+00	TMSB10	2.40E-01	1.00E+00
DIABLO	-2.91E+00	2.36E-52	LOC102403482	-2.15E-01	1.00E+00	LOC102400779	-2.50E-01	1.00E+00
RANBP9	1.54E+00	2.37E-52	C9H5orf30	2.58E-01	1.00E+00	ZNF605	-1.91E-01	1.00E+00
LOC102416055	6.94E+00	2.40E-52	FAM20B	1.86E-01	1.00E+00	NUDT12	-1.65E-01	1.00E+00
RRAD	-6.99E+00	2.49E-52	NAMPT	1.10E-01	1.00E+00	ADK	-1.17E-01	1.00E+00
DOCK2	3.81E+00	2.57E-52	SSH2	1.32E-01	1.00E+00	SNIP1	-1.66E-01	1.00E+00
SMOC1	-6.04E+00	2.62E-52	PDCD2L	-1.35E-01	1.00E+00	DOCK2	-3.50E-01	1.00E+00
NVL	1.21E+00	2.64E-52	ADRA1D	3.61E-01	1.00E+00	EPHA2	-1.33E-01	1.00E+00
NDUFAF5	-3.10E+00	2.67E-52	NR6A1	1.36E-01	1.00E+00	BUB3	-1.17E-01	1.00E+00
TTC39B	-2.44E+00	2.83E-52	GTF2A2	1.55E-01	1.00E+00	NUP188	-1.29E-01	1.00E+00
MINDY4	-5.11E+00	2.83E-52	LOC112586463	-4.82E-01	1.00E+00	LMBR1	-1.27E-01	1.00E+00
LOC102412637	-6.53E+00	2.89E-52	WDR83	2.32E-01	1.00E+00	C14H20orf96	-1.93E-01	1.00E+00
CHD1	-2.94E+00	3.04E-52	RASGRP4	-3.14E-01	1.00E+00	SEC16A	-1.51E-01	1.00E+00
LOC102397659	7.58E+00	3.21E-52	CD226	-4.82E-01	1.00E+00	HNRNPH1	-1.78E-01	1.00E+00
LOC102392628	-3.46E+00	3.27E-52	PSMB1	-1.26E-01	1.00E+00	SUDS3	-1.25E-01	1.00E+00
FAM161B	2.46E+00	3.38E-52	MKS1	1.34E-01	1.00E+00	TATDN3	-1.37E-01	1.00E+00
TPM1	-4.75E+00	3.45E-52	LOC102408483	5.85E-01	1.00E+00	PABPC1	-1.78E-01	1.00E+00
RIMS2	2.84E+00	3.49E-52	CDCA4	-1.85E-01	1.00E+00	SLC30A5	-1.34E-01	1.00E+00
TBL2	1.51E+00	3.55E-52	VAC14	1.26E-01	1.00E+00	DNAJB4	-1.99E-01	1.00E+00
LOC112585619	6.26E+00	3.60E-52	SARM1	1.50E-01	1.00E+00	COMMD3	1.31E-01	1.00E+00
TMEM35A	2.69E+00	3.61E-52	PLK1	1.18E-01	1.00E+00	LOC112580286	2.57E-01	1.00E+00
TNIP3	3.61E+00	3.71E-52	STX12	9.62E-02	1.00E+00	HERC3	1.84E-01	1.00E+00
ZNF84	-3.64E+00	4.03E-52	CCNI2	-1.38E-01	1.00E+00	SEL1L3	2.74E-01	1.00E+00
TMTC1	-4.57E+00	4.12E-52	CCT6A	-3.11E-01	1.00E+00	GABARAPL2	1.36E-01	1.00E+00
EXOC1	-1.68E+00	4.27E-52	KBTBD8	1.83E-01	1.00E+00	MAP2K5	-1.31E-01	1.00E+00
NDST2	-2.14E+00	4.36E-52	CT83	2.33E-01	1.00E+00	NEK8	-1.90E-01	1.00E+00
CEACAM19	4.34E+00	4.55E-52	MKI67	2.09E-01	1.00E+00	MON1B	-1.60E-01	1.00E+00
AGBL2	1.86E+00	4.65E-52	NDUFS1	-1.56E-01	1.00E+00	MFSD2B	2.32E-01	1.00E+00
ID2	-6.07E+00	4.76E-52	TTC28	1.57E-01	1.00E+00	SGTB	3.30E-01	1.00E+00
FUOM	-3.77E+00	4.85E-52	PPL	1.34E-01	1.00E+00	LOC102405586	2.38E-01	1.00E+00
G6PD	-2.50E+00	4.86E-52	TRAPPC9	-1.07E-01	1.00E+00	ANXA2	1.81E-01	1.00E+00
TCERG1	-2.04E+00	4.97E-52	LOC102405871	1.83E-01	1.00E+00	ABCB8	-1.20E-01	1.00E+00

SPOP	1.22E+00	5.12E-52	CABYR	3.77E-01	1.00E+00	GGA3	-1.39E-01	1.00E+00
LGALS3	-3.29E+00	5.92E-52	ANXA6	-3.33E-01	1.00E+00	PLCD4	2.19E-01	1.00E+00
LOC102403613	-5.71E+00	6.27E-52	SNW1	-1.00E-01	1.00E+00	ABL1	1.39E-01	1.00E+00
ZNF567	-3.79E+00	6.31E-52	LRCH1	-1.33E-01	1.00E+00	FRS3	-2.15E-01	1.00E+00
PPP6R2	-1.85E+00	6.58E-52	LOC102402857	5.60E-01	1.00E+00	UBE2D1	1.86E-01	1.00E+00
PDE5A	3.12E+00	6.80E-52	SKIL	-6.62E-01	1.00E+00	ND2	-2.64E-01	1.00E+00
HARS2	-1.84E+00	6.84E-52	PIP5KL1	5.14E-01	1.00E+00	TRIT1	-1.26E-01	1.00E+00
FAM83F	3.92E+00	7.35E-52	ASNA1	1.31E-01	1.00E+00	LOC112585314	-2.24E-01	1.00E+00
LIMCH1	2.43E+00	7.90E-52	NCK2	1.06E-01	1.00E+00	PPIL4	-1.65E-01	1.00E+00
ZNF454	-1.86E+00	7.99E-52	WDR27	3.80E-01	1.00E+00	DGKD	-1.28E-01	1.00E+00
LOC102404294	1.63E+00	8.01E-52	OSBPL10	1.03E-01	1.00E+00	TSPAN31	1.29E-01	1.00E+00
ATRAID	-2.15E+00	8.01E-52	LOC102394237	1.19E-01	1.00E+00	NAE1	-1.26E-01	1.00E+00
BHLHE40	-4.79E+00	9.07E-52	NCOR1	1.61E-01	1.00E+00	PTBP2	-1.54E-01	1.00E+00
PRKAG2	-4.12E+00	9.42E-52	ALG9	-1.60E-01	1.00E+00	OXA1L	-1.16E-01	1.00E+00
ARHGAP42	-2.24E+00	1.01E-51	POLD4	-4.03E-01	1.00E+00	LMNA	-1.50E-01	1.00E+00
IQCH	-2.94E+00	1.05E-51	NPTX2	9.77E-02	1.00E+00	TRIM38	1.26E-01	1.00E+00
DHRS11	-2.52E+00	1.07E-51	CTNNA2	-1.56E-01	1.00E+00	ALG6	-1.91E-01	1.00E+00
BBX	-2.06E+00	1.07E-51	ARHGAP29	1.54E-01	1.00E+00	HSPBP1	1.64E-01	1.00E+00
RPL6	-2.81E+00	1.08E-51	LOC102415205	-2.17E-01	1.00E+00	LOC102397662	3.24E-01	1.00E+00
ARL6IP6	-3.89E+00	1.12E-51	CKB	-1.07E-01	1.00E+00	HS6ST1	1.52E-01	1.00E+00
LOC112584584	-5.89E+00	1.13E-51	LOC102402274	5.27E-01	1.00E+00	KCNB2	-1.57E-01	1.00E+00
ARRDC1	1.85E+00	1.15E-51	ZNF181	-1.49E-01	1.00E+00	LOC102401189	-1.59E-01	1.00E+00
MEGF10	6.18E+00	1.16E-51	CUL7	1.68E-01	1.00E+00	TMEM50B	-1.17E-01	1.00E+00
EDN1	-8.34E+00	1.16E-51	EPHB2	-2.27E-01	1.00E+00	SPSB2	2.07E-01	1.00E+00
GSTM3	3.77E+00	1.17E-51	CNIH4	-1.17E-01	1.00E+00	ALCAM	3.52E-01	1.00E+00
RCBTB2	-1.71E+00	1.18E-51	NF2	1.03E-01	1.00E+00	CD81	-1.40E-01	1.00E+00
CDH24	-3.02E+00	1.29E-51	CNBD2	-1.16E-01	1.00E+00	POLD1	-1.50E-01	1.00E+00
MED11	-4.20E+00	1.32E-51	ARHGEF5	1.12E-01	1.00E+00	COX1	-3.63E-01	1.00E+00
RIMKLB	-4.70E+00	1.40E-51	TMEM82	-6.03E-01	1.00E+00	LASP1	1.35E-01	1.00E+00
RAB9A	1.60E+00	1.40E-51	SLC9A1	1.14E-01	1.00E+00	TM9SF1	-1.21E-01	1.00E+00
LOC112580778	6.36E+00	1.47E-51	RPS5	-1.28E-01	1.00E+00	ENPP5	1.91E-01	1.00E+00
KIAA0930	-3.99E+00	1.47E-51	WIF1	1.32E-01	1.00E+00	AKAP5	2.94E-01	1.00E+00
MBTPS2	-	1.48E-51	IL23A	-2.32E-	1.00E+00	LOC112581256	2.95E-01	1.00E+00

	4.20E+00			01				
KCND1	-4.86E+00	1.74E-51	LOC102398506	1.18E-01	1.00E+00	ZBTB7B	1.58E-01	1.00E+00
ATP9A	2.23E+00	1.75E-51	NATD1	-1.32E-01	1.00E+00	DCAF6	1.20E-01	1.00E+00
SIGLEC11	6.23E+00	1.75E-51	RBM45	-1.27E-01	1.00E+00	GRIN2A	3.11E-01	1.00E+00
FUT10	-5.38E+00	1.79E-51	SURF4	9.90E-02	1.00E+00	CARMIL3	-2.77E-01	1.00E+00
TRA2A	-1.72E+00	1.80E-51	NOP58	-1.37E-01	1.00E+00	VPS36	1.41E-01	1.00E+00
C1H21orf2	-2.55E+00	1.81E-51	FAM25A	-2.52E-01	1.00E+00	MBOAT2	1.28E-01	1.00E+00
UFC1	-2.32E+00	1.82E-51	TM9SF1	-1.81E-01	1.00E+00	MPPED2	2.56E-01	1.00E+00
XKR5	-6.43E+00	1.82E-51	FAM129C	-1.05E-01	1.00E+00	MAATS1	2.50E-01	1.00E+00
FOXE3	9.26E+00	1.83E-51	E2F1	8.94E-02	1.00E+00	LOC102408113	-1.57E-01	1.00E+00
UBTD1	1.78E+00	1.89E-51	PTPN5	-2.73E-01	1.00E+00	AARS	1.23E-01	1.00E+00
IFIT5	-4.72E+00	1.89E-51	LRRC74B	5.34E-01	1.00E+00	CRYBB1	2.69E-01	1.00E+00
RBM19	-2.05E+00	1.89E-51	PHPT1	-2.07E-01	1.00E+00	LRRC1	-1.28E-01	1.00E+00
DGKD	1.66E+00	1.90E-51	MED18	-1.10E-01	1.00E+00	RWDD3	-1.57E-01	1.00E+00
LOC102406739	-3.62E+00	1.97E-51	RBM8A	-1.27E-01	1.00E+00	GID8	-3.51E-01	1.00E+00
MYBBP1A	1.86E+00	2.06E-51	DHX16	1.73E-01	1.00E+00	FDFT1	-1.36E-01	1.00E+00
SELENOP	-3.76E+00	2.12E-51	ZSCAN10	3.79E-01	1.00E+00	TEX14	-2.19E-01	1.00E+00
COG2	1.31E+00	2.15E-51	NRP2	-2.49E-01	1.00E+00	CCDC42	-3.48E-01	1.00E+00
ZER1	-2.11E+00	2.31E-51	CTU1	3.32E-01	1.00E+00	P2RX5	3.22E-01	1.00E+00
NQO2	-2.18E+00	2.61E-51	ZFAND2B	-2.38E-01	1.00E+00	TGFBR2	2.34E-01	1.00E+00
TMED5	-2.97E+00	2.61E-51	BPTF	2.12E-01	1.00E+00	PTPRN	2.44E-01	1.00E+00
CFAP58	7.24E+00	2.74E-51	ZFPM1	-2.20E-01	1.00E+00	IRF2BP2	1.51E-01	1.00E+00
IMPDH1	-2.07E+00	2.75E-51	XPO5	1.41E-01	1.00E+00	HEPACAM	3.58E-01	1.00E+00
LOC102392068	-2.35E+00	2.80E-51	SPRED1	2.68E-01	1.00E+00	STRADA	-1.11E-01	1.00E+00
UBE3B	1.57E+00	2.81E-51	TMEM196	1.71E-01	1.00E+00	ISCU	1.26E-01	1.00E+00
SSBP4	-2.07E+00	2.83E-51	PKDCC	2.23E-01	1.00E+00	LOC112584587	3.24E-01	1.00E+00
RBP4	-3.07E+00	2.93E-51	ERMP1	1.38E-01	1.00E+00	TIMELESS	-1.37E-01	1.00E+00
GPRC5D	3.90E+00	3.01E-51	JAM3	-1.05E-01	1.00E+00	TRMT13	-2.62E-01	1.00E+00
IRF3	-1.84E+00	3.01E-51	LOC112578513	2.80E-01	1.00E+00	LOC102404909	-1.84E-01	1.00E+00
ARAF	-1.62E+00	3.14E-51	ZNF572	1.53E-01	1.00E+00	B3GNT9	-2.30E-01	1.00E+00
DACH1	-5.89E+00	3.25E-51	CPSF7	-2.36E-01	1.00E+00	RBM15B	1.19E-01	1.00E+00
BMPR2	-2.30E+00	3.31E-51	GPAT3	-2.07E-01	1.00E+00	UTP23	1.80E-01	1.00E+00
PKMYT1	2.21E+00	3.47E-51	GMPPA	1.06E-01	1.00E+00	HSBP1	1.37E-01	1.00E+00

RAB21	1.50E+00	3.54E-51	C10H6orf203	-1.13E-01	1.00E+00	LOC102389532	3.08E-01	1.00E+00
GSR	1.61E+00	3.56E-51	LOC102410528	-1.00E-01	1.00E+00	TMEM200C	3.96E-01	1.00E+00
VPS72	1.41E+00	3.64E-51	CD276	1.63E-01	1.00E+00	EDC3	1.21E-01	1.00E+00
PISD	1.47E+00	3.75E-51	PIWIL1	-3.25E-01	1.00E+00	DNAJC27	-1.81E-01	1.00E+00
TMED4	-1.67E+00	3.78E-51	PROCA1	-1.05E-01	1.00E+00	LOC102399215	-1.77E-01	1.00E+00
COL24A1	4.47E+00	3.85E-51	SPEG	-1.98E-01	1.00E+00	LPGAT1	1.26E-01	1.00E+00
POM121C	1.80E+00	3.90E-51	PKP1	3.40E-01	1.00E+00	CTPS2	1.27E-01	1.00E+00
NFYA	-3.93E+00	3.97E-51	PET100	-2.08E-01	1.00E+00	TWISTNB	-1.30E-01	1.00E+00
ONECUT1	5.57E+00	4.06E-51	IMMT	9.53E-02	1.00E+00	LRP2BP	-2.74E-01	1.00E+00
ZBTB14	-4.11E+00	4.19E-51	RPLP0	-1.18E-01	1.00E+00	LOC112577691	3.54E-01	1.00E+00
PNPLA7	-2.47E+00	4.40E-51	LOC102408544	1.38E-01	1.00E+00	TFDP1	-1.27E-01	1.00E+00
RNF20	1.52E+00	4.54E-51	TMEM132B	-5.74E-01	1.00E+00	GTPBP6	1.82E-01	1.00E+00
CSRP1	-6.51E+00	4.62E-51	STK26	-3.00E-01	1.00E+00	EXO5	2.04E-01	1.00E+00
ERAP1	-6.40E+00	4.79E-51	IGSF21	-3.09E-01	1.00E+00	CCL5	5.03E-01	1.00E+00
LOC112584619	-4.40E+00	4.84E-51	ZBED8	-3.84E-01	1.00E+00	CTNNBIP1	-1.60E-01	1.00E+00
EHD1	1.66E+00	4.86E-51	PEX5L	1.34E-01	1.00E+00	TEAD2	1.21E-01	1.00E+00
ZFAND6	1.40E+00	5.05E-51	TRNP1	-2.08E-01	1.00E+00	SCAF1	-1.19E-01	1.00E+00
HS3ST1	-3.78E+00	5.13E-51	TRIM41	1.30E-01	1.00E+00	LOC102409513	2.28E-01	1.00E+00
SPAG7	-2.15E+00	5.17E-51	CAPN13	-1.23E-01	1.00E+00	NUB1	-1.29E-01	1.00E+00
LOC102403599	7.23E+00	5.40E-51	TNS3	1.54E-01	1.00E+00	SUN1	-1.38E-01	1.00E+00
RPP40	1.95E+00	5.50E-51	RPLP2	-1.58E-01	1.00E+00	LOC102395067	-3.19E-01	1.00E+00
FNDC1	5.13E+00	5.57E-51	LOC102399924	1.44E-01	1.00E+00	PFDN6	1.81E-01	1.00E+00
RASGEF1B	-3.47E+00	5.73E-51	CHAT	-2.44E-01	1.00E+00	KNTC1	-2.56E-01	1.00E+00
RPS6KA2	-3.13E+00	5.80E-51	CDKN2C	-3.45E-01	1.00E+00	SRPRA	-1.21E-01	1.00E+00
RAP2B	-2.22E+00	5.99E-51	TGDS	4.94E-01	1.00E+00	PLEKHA7	-1.20E-01	1.00E+00
ANGPTL4	-3.87E+00	6.10E-51	LY6E	-3.22E-01	1.00E+00	TBC1D31	-2.95E-01	1.00E+00
LOC112578832	7.20E+00	6.25E-51	CDKN1A	-5.51E-01	1.00E+00	LOC102401515	-2.59E-01	1.00E+00
SNCA	6.93E+00	6.78E-51	SYNRG	1.08E-01	1.00E+00	LRRC56	-2.95E-01	1.00E+00
CRHR1	6.54E+00	7.20E-51	RNF4	9.95E-02	1.00E+00	ACP1	-1.19E-01	1.00E+00
HS3ST2	4.64E+00	7.46E-51	HMGB1	-9.93E-02	1.00E+00	KMT2A	-1.74E-01	1.00E+00
C2CD4C	7.24E+00	7.55E-51	SIAH3	-1.78E-01	1.00E+00	RNF24	2.38E-01	1.00E+00
POGLUT1	-3.74E+00	7.56E-51	LOC112585163	-1.24E-01	1.00E+00	ELOF1	1.54E-01	1.00E+00
TMEM156	-4.99E+00	7.62E-51	RNF19A	1.54E-01	1.00E+00	CXXC5	1.27E-01	1.00E+00
PXT1	3.60E+00	7.79E-51	PRRC2B	1.91E-01	1.00E+00	LRWD1	-1.37E-	1.00E+00

							01	
MTA3	-1.73E+00	8.08E-51	ASNSD1	-1.14E-01	1.00E+00	FAM13B	-1.76E-01	1.00E+00
PSMB4	-2.09E+00	8.29E-51	PRKCD	2.24E-01	1.00E+00	C9H19orf70	1.89E-01	1.00E+00
DNAJC16	1.69E+00	8.45E-51	AMACR	-3.82E-01	1.00E+00	RHOV	-2.80E-01	1.00E+00
LOC102401515	3.11E+00	9.01E-51	TRNT1	-1.12E-01	1.00E+00	PPCS	-1.47E-01	1.00E+00
IL17RC	-5.11E+00	9.53E-51	TMCO1	-1.57E-01	1.00E+00	ATXN7L2	-1.54E-01	1.00E+00
MMP11	-5.22E+00	9.63E-51	ENTPD4	1.34E-01	1.00E+00	FAM78A	-1.49E-01	1.00E+00
TEX45	-6.10E+00	9.91E-51	FAM210B	-1.60E-01	1.00E+00	LOC112582091	-2.08E-01	1.00E+00
SEPT6	-3.42E+00	1.02E-50	GFM1	-2.10E-01	1.00E+00	MTMR8	1.50E-01	1.00E+00
BRAP	1.44E+00	1.06E-50	LOC102404764	-2.54E-01	1.00E+00	ATP6V1C2	-2.65E-01	1.00E+00
TRIT1	-1.54E+00	1.10E-50	LOC102392771	-4.41E-01	1.00E+00	ZFHX2	1.65E-01	1.00E+00
KIF16B	1.54E+00	1.15E-50	HMCN2	-4.37E-01	1.00E+00	SNX29	-1.46E-01	1.00E+00
OGDH	-1.36E+00	1.16E-50	LOC112580260	-4.17E-01	1.00E+00	DTNB	-1.46E-01	1.00E+00
JUP	-6.89E+00	1.16E-50	SHROOM4	-2.13E-01	1.00E+00	CENPW	-1.73E-01	1.00E+00
AKAP4	4.10E+00	1.22E-50	RALGAPA1	1.30E-01	1.00E+00	CTC1	-1.48E-01	1.00E+00
LOC112581035	5.94E+00	1.25E-50	GLS2	9.44E-02	1.00E+00	CCDC47	-1.20E-01	1.00E+00
SLC26A2	-2.07E+00	1.25E-50	LOC112586495	-2.07E-01	1.00E+00	VNN2	-3.80E-01	1.00E+00
CLASP1	-1.78E+00	1.26E-50	PARP14	-4.67E-01	1.00E+00	NCOR2	-1.37E-01	1.00E+00
ST5	-1.64E+00	1.30E-50	PPA1	9.89E-02	1.00E+00	COG2	-1.19E-01	1.00E+00
MYCN	-5.98E+00	1.48E-50	SLC37A4	-1.35E-01	1.00E+00	DBT	-1.66E-01	1.00E+00
DCUN1D2	-2.83E+00	1.49E-50	RANBP10	1.85E-01	1.00E+00	LOC102390160	-1.63E-01	1.00E+00
CRYL1	-3.74E+00	1.57E-50	UPF1	1.24E-01	1.00E+00	PALM	-1.34E-01	1.00E+00
MYDGF	-2.25E+00	1.61E-50	GTDC1	-1.38E-01	1.00E+00	NXNL2	-1.75E-01	1.00E+00
GJB3	-6.51E+00	1.62E-50	KCNH5	1.29E-01	1.00E+00	DNAJB12	-1.12E-01	1.00E+00
CAPRIN1	-1.50E+00	1.73E-50	DNAH8	2.46E-01	1.00E+00	LOC112587308	1.73E-01	1.00E+00
NDUFA10	-2.21E+00	1.78E-50	ATG4B	1.34E-01	1.00E+00	MMS22L	-1.71E-01	1.00E+00
DPH2	1.64E+00	1.83E-50	STON2	2.13E-01	1.00E+00	DHDH	2.26E-01	1.00E+00
LOC102413401	2.91E+00	1.88E-50	COL4A5	3.05E-01	1.00E+00	LOC102398033	-2.01E-01	1.00E+00
IL6R	-5.21E+00	1.92E-50	LOC102414381	-1.61E-01	1.00E+00	MAP2K7	-1.14E-01	1.00E+00
VAMP2	-2.03E+00	1.99E-50	RNF40	1.23E-01	1.00E+00	KLK12	-3.81E-01	1.00E+00
SGCD	3.75E+00	2.01E-50	CAPG	-4.19E-01	1.00E+00	SLU7	1.46E-01	1.00E+00
LOC102405586	2.38E+00	2.07E-50	HAS3	1.97E-01	1.00E+00	G2E3	-1.65E-01	1.00E+00
NR3C1	3.04E+00	2.13E-50	WDR45B	1.02E-01	1.00E+00	MFSD6	2.64E-01	1.00E+00

IL36RN	7.86E+00	2.17E-50	GFI1B	-2.52E-01	1.00E+00	AMIGO1	-1.77E-01	1.00E+00
PABPN1	-1.72E+00	2.19E-50	CWC25	1.02E-01	1.00E+00	LOC102413289	-1.30E-01	1.00E+00
HSF4	-3.15E+00	2.21E-50	TXNDC12	-1.42E-01	1.00E+00	LOC112578487	3.19E-01	1.00E+00
GRK6	1.67E+00	2.22E-50	LOC112579965	-2.65E-01	1.00E+00	ZSWIM6	1.72E-01	1.00E+00
C21H3orf14	-3.10E+00	2.29E-50	DCLRE1B	1.50E-01	1.00E+00	C9H5orf15	-1.13E-01	1.00E+00
ACAA2	-2.04E+00	2.54E-50	HTRA1	6.01E-01	1.00E+00	SMARCAD1	1.64E-01	1.00E+00
MXI1	1.65E+00	2.69E-50	LOC102411445	-3.00E-01	1.00E+00	UBE2E3	1.37E-01	1.00E+00
ABCA3	-2.56E+00	2.70E-50	ASB1	1.07E-01	1.00E+00	RBFOX2	1.24E-01	1.00E+00
DUSP10	3.48E+00	2.75E-50	STEAP3	-2.04E-01	1.00E+00	ISOC2	1.92E-01	1.00E+00
CCDC34	2.05E+00	2.76E-50	C5H1orf105	-4.15E-01	1.00E+00	TARS2	-1.13E-01	1.00E+00
KIF23	2.40E+00	2.93E-50	LOC112578528	-4.12E-01	1.00E+00	TDRD12	2.96E-01	1.00E+00
ZFAND2B	-2.62E+00	2.95E-50	RPAP1	1.22E-01	1.00E+00	TMCO4	-1.30E-01	1.00E+00
LOC112578119	-7.46E+00	2.95E-50	GTF2F1	-1.09E-01	1.00E+00	SPPL2B	-1.27E-01	1.00E+00
LOC112586532	-8.40E+00	2.99E-50	LOC102408072	4.99E-01	1.00E+00	NSD2	-1.31E-01	1.00E+00
AKAP11	2.34E+00	3.14E-50	LOC102414406	-6.34E-01	1.00E+00	NRP1	-1.72E-01	1.00E+00
LOC102398455	-7.97E+00	3.24E-50	SMAD4	2.06E-01	1.00E+00	ATP6V1G2	2.67E-01	1.00E+00
PDPN	-4.73E+00	3.32E-50	RBP4	2.79E-01	1.00E+00	WHAMM	-1.26E-01	1.00E+00
CERK	-6.43E+00	3.65E-50	TSPYL1	9.45E-02	1.00E+00	EBAG9	-1.37E-01	1.00E+00
ZBED3	-3.25E+00	3.69E-50	ZBTB33	1.14E-01	1.00E+00	PGRMC2	-1.21E-01	1.00E+00
IFFO1	1.68E+00	3.69E-50	NR0B2	2.78E-01	1.00E+00	LOC102399267	2.46E-01	1.00E+00
TMCC3	2.71E+00	3.76E-50	DPF2	1.03E-01	1.00E+00	DCK	-1.72E-01	1.00E+00
LOC102390920	5.23E+00	3.81E-50	TCEA1	1.33E-01	1.00E+00	COL6A2	1.96E-01	1.00E+00
AP1S3	2.46E+00	4.01E-50	LOC112586239	-5.00E-01	1.00E+00	LOC102403261	-3.08E-01	1.00E+00
TMEM179	6.59E+00	4.20E-50	LOC102412878	9.07E-02	1.00E+00	RWDD4	-1.31E-01	1.00E+00
HDGF	-3.55E+00	4.32E-50	SLC10A4	-4.08E-01	1.00E+00	CDPF1	-2.01E-01	1.00E+00
DRG1	1.78E+00	4.35E-50	KIAA0319	1.64E-01	1.00E+00	ARID3B	-1.60E-01	1.00E+00
THUMPD3	-1.68E+00	4.36E-50	LOC112579699	-4.70E-01	1.00E+00	INAFM2	1.54E-01	1.00E+00
EFCAB2	-3.52E+00	4.57E-50	TMEM151A	4.03E-01	1.00E+00	LOC102399535	2.15E-01	1.00E+00
DDA1	1.50E+00	4.68E-50	LOC102411708	-2.58E-01	1.00E+00	SRGAP1	-1.15E-01	1.00E+00
LOC102399988	2.95E+00	4.71E-50	DNAJC28	-2.42E-01	1.00E+00	TNFAIP8L1	1.43E-01	1.00E+00
URM1	-1.89E+00	4.82E-50	RPL18	-1.30E-01	1.00E+00	SH3BP5	2.23E-01	1.00E+00
XAF1	-5.33E+00	5.32E-50	WASF2	1.10E-01	1.00E+00	DACH1	-1.31E-01	1.00E+00
PARP6	-2.32E+00	5.73E-50	PTEN	1.45E-01	1.00E+00	HADH	-1.36E-01	1.00E+00

COQ8A	1.47E+00	5.76E-50	C22H18orf32	-1.53E-01	1.00E+00	SERPINA11	2.98E-01	1.00E+00
E2F6	1.58E+00	5.82E-50	C8A	-4.64E-01	1.00E+00	TDG	-1.44E-01	1.00E+00
MCFD2	-2.10E+00	5.89E-50	AGBL4	-4.66E-01	1.00E+00	SCAND1	1.59E-01	1.00E+00
TRAFD1	1.35E+00	5.92E-50	LEUTX	2.74E-01	1.00E+00	RSPO2	-3.15E-01	1.00E+00
SRGAP1	1.41E+00	5.94E-50	IQCM	3.42E-01	1.00E+00	UNC45A	1.23E-01	1.00E+00
GEMIN4	1.54E+00	6.02E-50	EPB41L2	1.01E-01	1.00E+00	SLC22A17	-1.41E-01	1.00E+00
CDK2AP2	2.10E+00	6.03E-50	LOC112585053	-1.02E-01	1.00E+00	GCHFR	2.68E-01	1.00E+00
LOC102396303	-5.20E+00	6.42E-50	LOC112585097	6.57E-01	1.00E+00	CCDC71	1.49E-01	1.00E+00
LOC102414812	-5.81E+00	6.55E-50	FAM120C	1.69E-01	1.00E+00	GLB1L	-1.16E-01	1.00E+00
LOC102404230	3.15E+00	7.17E-50	BCL2L13	1.54E-01	1.00E+00	POMC	2.14E-01	1.00E+00
RAD54L	1.86E+00	7.25E-50	ACTN2	2.48E-01	1.00E+00	BORCS7	-1.98E-01	1.00E+00
PLEKHB2	1.45E+00	7.25E-50	NR5A1	5.37E-01	1.00E+00	C18H16orf46	2.43E-01	1.00E+00
CLEC1B	-6.94E+00	7.63E-50	LOC112585537	-2.91E-01	1.00E+00	ARPC5L	1.20E-01	1.00E+00
LOC102409250	-1.31E+00	7.70E-50	ALKBH4	1.16E-01	1.00E+00	KLHL36	1.14E-01	1.00E+00
DCUN1D4	1.37E+00	8.16E-50	PRG4	-1.96E-01	1.00E+00	HNRNPUL1	-1.56E-01	1.00E+00
TRAPPC11	1.59E+00	8.39E-50	TECR	1.18E-01	1.00E+00	METTL21A	1.66E-01	1.00E+00
CCT8L2	6.33E+00	8.43E-50	SEC24D	1.59E-01	1.00E+00	CORO2B	-2.97E-01	1.00E+00
LOC102398554	4.93E+00	8.56E-50	LOC112585206	-3.25E-01	1.00E+00	CERS2	1.15E-01	1.00E+00
SOD2	1.41E+00	8.75E-50	RNF139	-1.17E-01	1.00E+00	SLC23A3	2.88E-01	1.00E+00
LOC102394622	-2.79E+00	8.95E-50	LOC102408933	-5.12E-01	1.00E+00	CATSPERD	-1.67E-01	1.00E+00
TNIP2	-3.60E+00	9.26E-50	TRMT61A	-1.06E-01	1.00E+00	PAQR3	1.45E-01	1.00E+00
CEBPD	-6.90E+00	9.34E-50	XRRA1	1.97E-01	1.00E+00	RASL10B	-2.86E-01	1.00E+00
PPIA	-2.49E+00	1.04E-49	LMAN2L	1.47E-01	1.00E+00	LOC102398843	-1.87E-01	1.00E+00
RTP3	-9.12E+00	1.07E-49	WBP1L	-1.76E-01	1.00E+00	TREM2	-5.81E-01	1.00E+00
CNKSR3	-4.75E+00	1.09E-49	LOC112580606	-2.34E-01	1.00E+00	PDSS1	-1.42E-01	1.00E+00
RPS5	-3.43E+00	1.09E-49	ETV6	-1.11E-01	1.00E+00	RIOK2	1.27E-01	1.00E+00
TBC1D17	-1.57E+00	1.10E-49	COL5A1	-4.88E-01	1.00E+00	FIS1	1.63E-01	1.00E+00
LOC112578238	-3.58E+00	1.11E-49	LBX1	3.99E-01	1.00E+00	TREX2	2.48E-01	1.00E+00
LOC112586997	-5.71E+00	1.12E-49	ANKRD37	2.65E-01	1.00E+00	LOC102402715	1.55E-01	1.00E+00
CD27	5.92E+00	1.17E-49	NFIB	-1.74E-01	1.00E+00	LOC102390349	-2.81E-01	1.00E+00
TMEM106B	-3.72E+00	1.18E-49	GLE1	9.03E-02	1.00E+00	LOC102403233	-1.14E-01	1.00E+00
EIF2A	-1.47E+00	1.20E-49	GPD1L	-1.68E-01	1.00E+00	RFT1	-1.38E-01	1.00E+00
ZCCHC24	-5.12E+00	1.21E-49	ZMIZ2	1.51E-01	1.00E+00	LOC112583735	-3.10E-01	1.00E+00
NRG4	2.75E+00	1.23E-49	LOC102416629	-3.32E-	1.00E+00	RPN2	-1.37E-	1.00E+00

				01			01	
LOC112581036	4.41E+00	1.24E-49	C20H15orf40	-2.40E-01	1.00E+00	GYG2	1.43E-01	1.00E+00
MPC1L	6.54E+00	1.24E-49	CCDC112	-3.59E-01	1.00E+00	YPEL3	-1.62E-01	1.00E+00
TMEM132A	-7.02E+00	1.24E-49	NUTF2	-1.05E-01	1.00E+00	SYNPO	1.20E-01	1.00E+00
LOC102391514	1.80E+00	1.34E-49	DAAM1	1.20E-01	1.00E+00	LOC102399663	1.98E-01	1.00E+00
LOC112581832	7.69E+00	1.43E-49	AP1G2	-1.41E-01	1.00E+00	APLN	3.10E-01	1.00E+00
CPT1A	-3.60E+00	1.51E-49	DCPS	-1.27E-01	1.00E+00	GFM2	-1.20E-01	1.00E+00
EFEMP2	-5.11E+00	1.54E-49	HLTF	1.94E-01	1.00E+00	ROR1	2.30E-01	1.00E+00
RPN2	-1.70E+00	1.55E-49	ZDHHC1	3.17E-01	1.00E+00	TATDN1	-1.46E-01	1.00E+00
APOM	-5.06E+00	1.60E-49	LOC102404032	9.30E-02	1.00E+00	THAP5	1.50E-01	1.00E+00
LOC112579234	2.81E+00	1.62E-49	CASP9	3.08E-01	1.00E+00	TMX3	-1.41E-01	1.00E+00
DHRS3	-7.03E+00	1.64E-49	SLC25A25	-2.54E-01	1.00E+00	CERK	1.28E-01	1.00E+00
HRCT1	3.55E+00	1.65E-49	CABCOCO1	3.64E-01	1.00E+00	SLC2A11	-2.93E-01	1.00E+00
CPQ	-5.79E+00	1.71E-49	LEMD3	1.74E-01	1.00E+00	PPP2R5A	1.26E-01	1.00E+00
LOC102394989	-5.23E+00	1.74E-49	MYO1H	-2.31E-01	1.00E+00	NHLRC1	2.35E-01	1.00E+00
HEYL	-5.45E+00	1.75E-49	TFAP4	3.72E-01	1.00E+00	CCNE1	-1.72E-01	1.00E+00
ABTB2	3.10E+00	1.78E-49	CD37	5.39E-01	1.00E+00	RAD51	-1.46E-01	1.00E+00
ZNF22	-2.18E+00	1.81E-49	UBR2	1.42E-01	1.00E+00	SF3B1	-1.52E-01	1.00E+00
CXXC5	-5.50E+00	2.02E-49	EDEM1	-2.87E-01	1.00E+00	LOC102414615	2.33E-01	1.00E+00
LOC112581589	5.48E+00	2.05E-49	LOC102411911	1.24E-01	1.00E+00	MRPL32	1.34E-01	1.00E+00
GRAMD1A	-1.43E+00	2.07E-49	HUWE1	1.87E-01	1.00E+00	POGLUT1	-1.81E-01	1.00E+00
FOCAD	-1.52E+00	2.32E-49	gen-01	3.53E-01	1.00E+00	LOC102403526	2.45E-01	1.00E+00
DHX29	-1.54E+00	2.32E-49	MAMDC2	-3.14E-01	1.00E+00	TOMM7	1.63E-01	1.00E+00
TDP2	1.48E+00	2.33E-49	MIPEP	1.30E-01	1.00E+00	MAMLD1	-1.86E-01	1.00E+00
SIGLECL1	8.09E+00	2.33E-49	AKTIP	1.12E-01	1.00E+00	CMIP	-1.37E-01	1.00E+00
DPY19L3	-1.91E+00	2.35E-49	ZNF282	-3.33E-01	1.00E+00	PACSIN2	-1.14E-01	1.00E+00
VSTM4	-5.38E+00	2.36E-49	LOC102402068	-1.86E-01	1.00E+00	LOC102399902	-2.92E-01	1.00E+00
SENP7	-2.36E+00	2.40E-49	PIR	-1.17E-01	1.00E+00	PGAM1	1.56E-01	1.00E+00
YIPF2	-1.77E+00	2.43E-49	RPS24	-1.80E-01	1.00E+00	C24H7orf26	1.26E-01	1.00E+00
ZNF646	2.07E+00	2.59E-49	ANAPC7	-9.32E-02	1.00E+00	TTC12	-1.75E-01	1.00E+00
IDUA	-6.65E+00	2.74E-49	LOC112577761	1.10E-01	1.00E+00	INHBA	-3.07E-01	1.00E+00
NT5C3A	-5.04E+00	2.75E-49	TYK2	-4.37E-01	1.00E+00	LOC112579935	2.70E-01	1.00E+00
THEM6	3.03E+00	2.83E-49	BMP15	1.34E-01	1.00E+00	HAUS1	1.34E-01	1.00E+00
RPS6KB2	-	2.90E-49	ERC2	-1.19E-	1.00E+00	SHROOM4	2.55E-01	1.00E+00

	2.33E+00			01				
ZNF140	-2.35E+00	2.92E-49	PRKD3	1.64E-01	1.00E+00	EMILIN2	3.93E-01	1.00E+00
WT1	-4.28E+00	2.94E-49	TMC2	2.72E-01	1.00E+00	FBXL2	1.88E-01	1.00E+00
TMEM126B	2.31E+00	3.03E-49	DGKH	2.15E-01	1.00E+00	REXO4	1.49E-01	1.00E+00
MAP2K1	2.01E+00	3.10E-49	KIF14	2.04E-01	1.00E+00	TDRP	-1.35E-01	1.00E+00
BRCA2	1.97E+00	3.24E-49	KLF8	-8.88E-01	1.00E+00	SCRN2	1.85E-01	1.00E+00
SLC10A3	1.46E+00	3.24E-49	TEPSIN	-1.31E-01	1.00E+00	LDAH	1.33E-01	1.00E+00
RPS6KC1	-1.60E+00	3.29E-49	MMACHC	-2.57E-01	1.00E+00	TBKBP1	1.41E-01	1.00E+00
PRDM13	6.56E+00	3.36E-49	MRRF	-1.30E-01	1.00E+00	KRAS	-1.70E-01	1.00E+00
HYI	-3.84E+00	3.41E-49	RBFOX1	-1.21E-01	1.00E+00	MBOAT7	1.32E-01	1.00E+00
PSD4	7.40E+00	3.43E-49	SH3GLB1	-1.01E-01	1.00E+00	RNF166	-1.36E-01	1.00E+00
LOC112582084	6.04E+00	3.45E-49	LOC112584451	3.04E-01	1.00E+00	SCAMP5	3.78E-01	1.00E+00
LOC102403829	-2.05E+00	3.51E-49	SLC29A3	1.92E-01	1.00E+00	SBF2	1.46E-01	1.00E+00
RGS10	3.41E+00	3.72E-49	FCGRT	-2.23E-01	1.00E+00	ARPC2	1.30E-01	1.00E+00
MMP15	2.00E+00	3.78E-49	TCP1	-8.72E-02	1.00E+00	MAFG	1.79E-01	1.00E+00
LCAT	-6.20E+00	3.87E-49	PDSS1	1.67E-01	1.00E+00	TMF1	-2.03E-01	1.00E+00
ZNF692	-2.13E+00	4.03E-49	LOC112586203	-2.08E-01	1.00E+00	ALDH1A2	-3.89E-01	1.00E+00
EED	-1.82E+00	4.26E-49	LOC102408956	-2.85E-01	1.00E+00	TEF	1.24E-01	1.00E+00
MYC	-7.32E+00	4.60E-49	LOC112578203	-3.11E-01	1.00E+00	CALCOCO1	-1.16E-01	1.00E+00
ARHGAP21	-2.58E+00	4.65E-49	LOC102413215	-3.88E-01	1.00E+00	ACTR8	-1.24E-01	1.00E+00
CCDC62	-2.53E+00	4.79E-49	KCNE1	-3.39E-01	1.00E+00	TGS1	-1.56E-01	1.00E+00
RBM47	-5.40E+00	4.81E-49	ABCD2	1.89E-01	1.00E+00	KITLG	2.90E-01	1.00E+00
WBP11	1.30E+00	5.02E-49	PLIN3	-1.61E-01	1.00E+00	MSH5	-3.25E-01	1.00E+00
LOC102413847	-2.74E+00	5.02E-49	MRPS14	-1.13E-01	1.00E+00	CCNYL1	-2.09E-01	1.00E+00
LOC102401136	-8.97E+00	5.04E-49	ACP2	3.27E-01	1.00E+00	ATP10A	2.43E-01	1.00E+00
ARL6	2.35E+00	5.16E-49	PLOD2	4.96E-01	1.00E+00	MARK3	-1.14E-01	1.00E+00
ACSF2	-4.68E+00	5.24E-49	TOPORS	1.55E-01	1.00E+00	ALDH6A1	-2.56E-01	1.00E+00
SLC12A2	2.35E+00	5.32E-49	ZSWIM7	-2.12E-01	1.00E+00	FCHSD2	-1.39E-01	1.00E+00
ITGAV	-4.89E+00	5.56E-49	SAP30L	1.08E-01	1.00E+00	ADAMTS8	-2.37E-01	1.00E+00
LOC102414965	-5.52E+00	5.66E-49	TCEAL1	6.47E-01	1.00E+00	SAP130	-1.28E-01	1.00E+00
KYNU	-5.67E+00	6.14E-49	LOC102405926	-4.97E-01	1.00E+00	ST3GAL5	1.38E-01	1.00E+00
HBA1	3.73E+00	6.50E-49	PNRC2	-1.32E-01	1.00E+00	NUPR1	2.18E-01	1.00E+00
LOC102416083	1.89E+00	6.52E-49	UBA52	-2.55E-01	1.00E+00	PARL	-1.14E-01	1.00E+00

DNAI2	-3.44E+00	6.55E-49	ABRACL	-1.51E-01	1.00E+00	PRDX4	1.68E-01	1.00E+00
KLF15	-2.37E+00	6.69E-49	SLC25A39	9.33E-02	1.00E+00	HACL1	-1.62E-01	1.00E+00
ZSWIM6	-3.36E+00	6.74E-49	LOC102395684	-4.76E-01	1.00E+00	CCNE2	-3.39E-01	1.00E+00
ATP11B	1.78E+00	6.85E-49	SEL1L	1.36E-01	1.00E+00	USP21	-1.64E-01	1.00E+00
ZYG11A	6.13E+00	6.91E-49	ZNF775	-2.31E-01	1.00E+00	ZNF316	1.18E-01	1.00E+00
SCEL	6.70E+00	6.93E-49	RASSF1	4.40E-01	1.00E+00	LOC102414507	1.88E-01	1.00E+00
KDM6A	-2.44E+00	7.34E-49	SAMD4B	1.50E-01	1.00E+00	DPYSL2	-1.27E-01	1.00E+00
ZNF232	6.51E+00	7.52E-49	MGME1	1.15E-01	1.00E+00	SNRNP48	-1.21E-01	1.00E+00
HYAL1	-4.97E+00	7.64E-49	HERC2	1.92E-01	1.00E+00	LOC112586232	3.57E-01	1.00E+00
ACVR2A	-2.88E+00	7.70E-49	LOC112585344	5.94E-01	1.00E+00	PMAIP1	-3.38E-01	1.00E+00
DNAJC6	3.34E+00	7.79E-49	AKT1	-9.69E-02	1.00E+00	MIER2	-1.24E-01	1.00E+00
NEBL	-1.93E+00	8.18E-49	ZHX2	-1.04E-01	1.00E+00	PLEKHO1	1.64E-01	1.00E+00
STAM2	-2.17E+00	8.26E-49	CDA	-1.58E-01	1.00E+00	SOBP	-1.45E-01	1.00E+00
EZR	2.50E+00	8.39E-49	NCBP2	1.19E-01	1.00E+00	NPTN	-1.19E-01	1.00E+00
CAMK1D	-2.67E+00	8.40E-49	LOC102394407	-1.03E-01	1.00E+00	GPR161	1.49E-01	1.00E+00
RPLP0	-3.38E+00	8.71E-49	NAA20	-1.03E-01	1.00E+00	ADCY2	3.08E-01	1.00E+00
ISG15	-6.78E+00	8.74E-49	SDCBP	1.09E-01	1.00E+00	LOC102396410	2.18E-01	1.00E+00
RRN3	1.59E+00	9.01E-49	C9H5orf15	-4.00E-01	1.00E+00	XDH	2.51E-01	1.00E+00
EDEM1	-2.94E+00	9.18E-49	DRC1	1.76E-01	1.00E+00	SETD5	-2.02E-01	1.00E+00
CEP250	-2.35E+00	9.19E-49	GJB5	-1.85E-01	1.00E+00	BAIAP2L1	-1.56E-01	1.00E+00
PLXDC2	-2.50E+00	9.24E-49	POLI	1.21E-01	1.00E+00	GNG10	-1.25E-01	1.00E+00
BDP1	-3.48E+00	9.25E-49	HIGD1B	4.26E-01	1.00E+00	SIL1	-1.75E-01	1.00E+00
PDGFRB	-4.66E+00	9.28E-49	RAP2C	-1.94E-01	1.00E+00	ATP5ME	2.03E-01	1.00E+00
SLC4A11	3.78E+00	9.46E-49	VSX2	1.95E-01	1.00E+00	RHNO1	-1.49E-01	1.00E+00
DPYSL3	-3.93E+00	9.87E-49	MOB1B	1.62E-01	1.00E+00	ZNF692	-1.41E-01	1.00E+00
TMEM184C	1.48E+00	9.96E-49	LOC112582279	1.35E-01	1.00E+00	FANCD2	-1.29E-01	1.00E+00
NR0B2	4.68E+00	1.03E-48	ENY2	-1.88E-01	1.00E+00	DDIT3	-2.38E-01	1.00E+00
MAGT1	-1.54E+00	1.06E-48	VMAC	-2.44E-01	1.00E+00	LOC102410484	-2.65E-01	1.00E+00
NSUN2	-1.48E+00	1.07E-48	PRMT5	8.85E-02	1.00E+00	ZNF521	1.41E-01	1.00E+00
LZTS1	-5.91E+00	1.07E-48	FAF2	-9.50E-02	1.00E+00	CERKL	2.91E-01	1.00E+00
CCL27	-5.41E+00	1.09E-48	CSMD3	-5.46E-01	1.00E+00	ARID5B	1.65E-01	1.00E+00
STAU1	1.51E+00	1.12E-48	MRPL15	-1.59E-01	1.00E+00	VAV2	1.70E-01	1.00E+00
ABCC1	1.77E+00	1.17E-48	SRRM2	1.65E-01	1.00E+00	LOC112580156	1.76E-01	1.00E+00

ST6GALNAC6	-2.40E+00	1.23E-48	LOC102414910	-1.44E-01	1.00E+00	TPCN1	-1.82E-01	1.00E+00
RGN	-6.00E+00	1.24E-48	EEF1AKMT1	-1.23E-01	1.00E+00	LOC102409679	2.46E-01	1.00E+00
DENND2C	3.39E+00	1.25E-48	RNF32	-5.11E-01	1.00E+00	PTPRJ	2.59E-01	1.00E+00
BST2	-7.22E+00	1.27E-48	LOC102415932	-3.36E-01	1.00E+00	MARVELD2	-1.99E-01	1.00E+00
UBQLN1	1.77E+00	1.28E-48	LOC112582355	-3.82E-01	1.00E+00	ARID3A	1.90E-01	1.00E+00
SLC24A5	-4.21E+00	1.31E-48	FBXL20	1.48E-01	1.00E+00	MRPL14	1.61E-01	1.00E+00
LOC102399411	7.24E+00	1.32E-48	SMPD3	-2.00E-01	1.00E+00	MIS18A	-1.78E-01	1.00E+00
GNB5	-1.80E+00	1.34E-48	LOC112586834	-1.44E-01	1.00E+00	LOC102403205	1.84E-01	1.00E+00
GULP1	-5.35E+00	1.37E-48	CAMSAP2	1.87E-01	1.00E+00	B3GALNT2	-1.29E-01	1.00E+00
RFFL	1.47E+00	1.40E-48	TMEM268	-2.34E-01	1.00E+00	TMEM39B	-1.27E-01	1.00E+00
MED12	-1.96E+00	1.47E-48	SLX4IP	-1.93E-01	1.00E+00	GADD45A	-1.93E-01	1.00E+00
LOC112582091	-3.43E+00	1.61E-48	KRI1	1.48E-01	1.00E+00	IDE	1.19E-01	1.00E+00
SERTAD3	-2.00E+00	1.64E-48	PDZD8	1.68E-01	1.00E+00	MRPL3	-1.18E-01	1.00E+00
RFX7	-2.70E+00	1.67E-48	OSER1	-1.06E-01	1.00E+00	AXIN2	-1.76E-01	1.00E+00
LOC102409101	-1.59E+00	1.74E-48	INPP5J	1.82E-01	1.00E+00	TMEM170B	-1.28E-01	1.00E+00
IL15RA	-4.64E+00	1.77E-48	GIMAP8	-3.08E-01	1.00E+00	RAD21	-1.31E-01	1.00E+00
DNTTIP2	2.07E+00	1.91E-48	ESRRA	1.76E-01	1.00E+00	BTG3	1.49E-01	1.00E+00
WFDC2	6.71E+00	1.96E-48	FOXN1	1.32E-01	1.00E+00	PCGF1	-1.59E-01	1.00E+00
SOD3	-6.66E+00	1.96E-48	TULP1	5.83E-01	1.00E+00	RPH3AL	2.35E-01	1.00E+00
UHRF1BP1L	-2.63E+00	2.06E-48	MRPL35	-2.68E-01	1.00E+00	KIAA0753	-1.44E-01	1.00E+00
LOC112580204	6.38E+00	2.06E-48	C24H7orf50	-1.40E-01	1.00E+00	LOC102390601	1.69E-01	1.00E+00
LOC112586993	5.59E+00	2.10E-48	TMEM174	-3.55E-01	1.00E+00	PABPN1	-1.33E-01	1.00E+00
C3H9orf72	1.56E+00	2.11E-48	ADAM19	1.34E-01	1.00E+00	PPP1R9A	-2.09E-01	1.00E+00
SPEG	-2.44E+00	2.12E-48	LOC102398622	-1.34E-01	1.00E+00	TBCK	1.41E-01	1.00E+00
LOC102398979	-3.23E+00	2.15E-48	GPR37	-3.11E-01	1.00E+00	HNRNPL	-1.45E-01	1.00E+00
MEFV	4.22E+00	2.20E-48	LOC102411403	-3.22E-01	1.00E+00	EXTL3	1.16E-01	1.00E+00
PLA2G4A	-6.06E+00	2.20E-48	CTTN	8.57E-02	1.00E+00	CDKL5	2.81E-01	1.00E+00
BLK	5.09E+00	2.27E-48	LSM8	-1.56E-01	1.00E+00	POLR3E	-1.14E-01	1.00E+00
RSU1	1.49E+00	2.30E-48	IPO7	-1.31E-01	1.00E+00	LOC112584570	2.46E-01	1.00E+00
LOC112581217	6.86E+00	2.30E-48	RAPGEF5	1.51E-01	1.00E+00	ZFAND4	-1.58E-01	1.00E+00
GPAA1	-1.83E+00	2.40E-48	CEMIP2	1.58E-01	1.00E+00	MAGOH	-1.32E-01	1.00E+00
CLDN1	-5.65E+00	2.51E-48	TENT4B	1.98E-01	1.00E+00	ZGPAT	1.38E-01	1.00E+00
DNAJB2	-	2.61E-48	LRRC8C	-1.29E-	1.00E+00	ANKRD52	-1.45E-	1.00E+00

	2.15E+00			01			01	
TENM3	4.11E+00	2.78E-48	JUP	-6.32E-01	1.00E+00	DHX33	-2.10E-01	1.00E+00
DKK1	9.21E+00	2.86E-48	DMAC2	-1.40E-01	1.00E+00	DCTD	-1.70E-01	1.00E+00
NEURL4	-1.65E+00	2.91E-48	LOC112587833	4.84E-01	1.00E+00	MOB1B	1.38E-01	1.00E+00
LOC112581272	5.50E+00	2.93E-48	ACSL6	-1.62E-01	1.00E+00	ERAP1	-1.21E-01	1.00E+00
JADE2	-5.72E+00	2.96E-48	FAS	1.19E+00	1.00E+00	SOWAHC	3.06E-01	1.00E+00
TP53INP1	2.31E+00	2.97E-48	DHRS12	-2.01E-01	1.00E+00	LOC112584572	2.21E-01	1.00E+00
VAV3	3.97E+00	3.01E-48	ADAMTS18	1.70E-01	1.00E+00	TFR2	1.96E-01	1.00E+00
PIP4K2A	-1.86E+00	3.20E-48	KRTCAP2	-1.40E-01	1.00E+00	SMIM12	1.59E-01	1.00E+00
TRPM3	3.05E+00	3.32E-48	KIT	1.09E-01	1.00E+00	ZNF182	1.68E-01	1.00E+00
LOC112586444	7.22E+00	3.34E-48	TAF9B	-1.63E-01	1.00E+00	OSBPL3	3.65E-01	1.00E+00
POT1	1.43E+00	3.39E-48	ALDH7A1	-9.84E-02	1.00E+00	MBD4	-1.34E-01	1.00E+00
AFF4	-4.44E+00	3.44E-48	CEP112	-1.67E-01	1.00E+00	SFXN3	1.68E-01	1.00E+00
AGPAT3	-1.37E+00	3.46E-48	FBXW7	9.63E-02	1.00E+00	MED11	1.92E-01	1.00E+00
NTN4	-4.89E+00	3.55E-48	LOC102406036	1.90E-01	1.00E+00	BTBD11	-1.32E-01	1.00E+00
PNISR	-1.91E+00	3.67E-48	PITPNC1	-1.86E-01	1.00E+00	TTC5	-1.23E-01	1.00E+00
ACY1	-4.00E+00	3.73E-48	SNX10	-3.23E-01	1.00E+00	MCCC2	-1.34E-01	1.00E+00
LOC112587947	-5.70E+00	3.77E-48	LOC102408707	-5.66E-01	1.00E+00	PHKA1	-1.17E-01	1.00E+00
TRANK1	-6.04E+00	3.77E-48	LOC102405684	2.89E-01	1.00E+00	RNF170	-1.18E-01	1.00E+00
FTH1	-4.60E+00	3.80E-48	DCAF7	1.11E-01	1.00E+00	ABHD14B	1.38E-01	1.00E+00
RPS6KA4	-5.29E+00	3.92E-48	TRAP1	9.67E-02	1.00E+00	RHBDL3	2.62E-01	1.00E+00
DNASE2	-1.87E+00	4.05E-48	INTS2	1.64E-01	1.00E+00	COQ8A	-1.21E-01	1.00E+00
YWHAQ	-1.53E+00	4.14E-48	MAPK4	-9.56E-02	1.00E+00	LCORL	-1.58E-01	1.00E+00
CAPG	-4.98E+00	4.47E-48	NUDT19	-1.97E-01	1.00E+00	SPATA6L	2.25E-01	1.00E+00
FLNA	-6.37E+00	4.63E-48	SACM1L	1.13E-01	1.00E+00	ACTG2	1.50E-01	1.00E+00
LPP	-4.79E+00	4.84E-48	ZNF445	1.30E-01	1.00E+00	CREB5	3.03E-01	1.00E+00
LMNB1	-4.01E+00	4.88E-48	VGLL4	1.01E-01	1.00E+00	MAPK10	1.50E-01	1.00E+00
LOC102396267	-5.70E+00	4.91E-48	TACR2	-3.99E-01	1.00E+00	LOC112580184	-2.49E-01	1.00E+00
REPS2	2.35E+00	4.99E-48	IFI6	-5.01E-01	1.00E+00	LOC102406545	1.40E-01	1.00E+00
LOC112578770	2.04E+00	5.17E-48	LOC112586132	-2.23E-01	1.00E+00	MAN1A1	1.59E-01	1.00E+00
TCAIM	-2.16E+00	5.17E-48	PTDSS2	9.99E-02	1.00E+00	WASF2	1.27E-01	1.00E+00
B4GAT1	-2.34E+00	5.30E-48	EMP2	-4.08E-01	1.00E+00	TRAM2	-1.55E-01	1.00E+00
FAM229B	2.82E+00	5.43E-48	TMEM14A	-3.23E-01	1.00E+00	EXD1	-2.79E-01	1.00E+00

LOC102399298	-4.77E+00	5.49E-48	ADNP	1.46E-01	1.00E+00	LOC102392464	-1.28E-01	1.00E+00
DTHD1	8.02E+00	5.57E-48	CNTN4	-2.07E-01	1.00E+00	CDH16	-3.05E-01	1.00E+00
OST4	-2.31E+00	5.76E-48	SNN	-1.66E-01	1.00E+00	GOT1	1.20E-01	1.00E+00
ANTXR1	-5.68E+00	5.94E-48	CLPTM1	8.86E-02	1.00E+00	ZNF576	1.41E-01	1.00E+00
RPL36AL	-2.75E+00	6.08E-48	C21H3orf67	-1.31E-01	1.00E+00	DCUN1D2	-1.47E-01	1.00E+00
PDE4C	5.46E+00	6.10E-48	ANKDD1B	-2.31E-01	1.00E+00	MPP6	1.54E-01	1.00E+00
LOC102395002	4.42E+00	6.22E-48	CPLX1	7.26E-01	1.00E+00	ZBTB1	-1.82E-01	1.00E+00
LOC112587012	7.54E+00	6.39E-48	RPL13	-1.22E-01	1.00E+00	DDIT4	1.75E-01	1.00E+00
CCNK	1.40E+00	6.42E-48	ZNF697	-2.77E-01	1.00E+00	ACSF3	-1.56E-01	1.00E+00
PPARD	1.52E+00	6.62E-48	NPDC1	-2.15E-01	1.00E+00	FSIP2	2.83E-01	1.00E+00
LOC102392353	-3.19E+00	6.71E-48	GPR108	-1.28E-01	1.00E+00	PCYT1A	1.58E-01	1.00E+00
LOC102389067	-5.91E+00	6.79E-48	FOXI1	-3.87E-01	1.00E+00	UBE2Z	1.11E-01	1.00E+00
DPF2	1.48E+00	6.80E-48	STARD10	-1.31E-01	1.00E+00	MRPL12	1.73E-01	1.00E+00
IL11RA	-3.29E+00	6.83E-48	SC5D	-4.70E-01	1.00E+00	SACS	2.08E-01	1.00E+00
ORC1	2.13E+00	6.93E-48	FAM178B	-1.96E-01	1.00E+00	NUDT1	2.35E-01	1.00E+00
A4GNT	6.69E+00	7.31E-48	LOC102406545	1.46E-01	1.00E+00	PRPF4B	-1.24E-01	1.00E+00
TSPAN31	-1.75E+00	7.49E-48	PTPN9	-1.31E-01	1.00E+00	SLC25A22	-1.40E-01	1.00E+00
CHST8	-6.52E+00	7.82E-48	NOL4	-1.40E-01	1.00E+00	RAB5A	-1.54E-01	1.00E+00
LOC112587811	5.02E+00	7.83E-48	CYHR1	-9.42E-02	1.00E+00	LOC102391382	-1.52E-01	1.00E+00
LOC112586394	6.37E+00	7.99E-48	ZDHHC5	1.15E-01	1.00E+00	TMEM225B	-1.30E-01	1.00E+00
LOC102414144	7.74E+00	8.21E-48	LOC102392520	-1.73E-01	1.00E+00	NIPSNAP2	-1.13E-01	1.00E+00
DRD5	7.16E+00	8.34E-48	WBP2	1.08E-01	1.00E+00	LOC102404456	-1.81E-01	1.00E+00
HBP1	1.59E+00	8.42E-48	C2H2orf66	-1.20E-01	1.00E+00	TOX4	-1.28E-01	1.00E+00
ATP10B	8.26E+00	8.43E-48	LRRC57	2.39E-01	1.00E+00	MPRIP	1.27E-01	1.00E+00
LDHD	-6.55E+00	8.87E-48	OBSCN	2.39E-01	1.00E+00	EVC	1.13E-01	1.00E+00
LOC102411142	-2.58E+00	8.90E-48	CBLN1	4.72E-01	1.00E+00	SMIM26	1.86E-01	1.00E+00
OSBPL8	-1.77E+00	8.96E-48	APRT	-2.51E-01	1.00E+00	RFFL	-1.14E-01	1.00E+00
SAMD8	1.91E+00	9.02E-48	CABLES2	8.79E-02	1.00E+00	TRADD	1.44E-01	1.00E+00
ZFP36	-4.87E+00	9.24E-48	TCFL5	-3.15E-01	1.00E+00	CPE	1.72E-01	1.00E+00
CLDND1	-2.57E+00	9.25E-48	LOC102413394	2.34E-01	1.00E+00	DSN1	-1.25E-01	1.00E+00
LOC102409002	-1.82E+00	9.47E-48	BCL2L15	1.39E-01	1.00E+00	TTC17	-1.59E-01	1.00E+00
TMED10	-1.39E+00	9.60E-48	TDRP	8.58E-02	1.00E+00	LOC102409123	2.63E-01	1.00E+00
CHSY1	-2.21E+00	9.82E-48	ERF	-1.70E-01	1.00E+00	LOC102388998	-2.24E-01	1.00E+00

IFT140	-1.61E+00	1.03E-47	HDAC9	1.20E-01	1.00E+00	G3BP2	-1.50E-01	1.00E+00
CDH7	2.70E+00	1.04E-47	TNIK	-1.79E-01	1.00E+00	SNX33	1.37E-01	1.00E+00
PATJ	1.90E+00	1.04E-47	HESX1	7.72E-01	1.00E+00	CDKN3	-1.97E-01	1.00E+00
SUV39H1	1.73E+00	1.05E-47	PRKCZ	1.59E-01	1.00E+00	FKBP1A	1.24E-01	1.00E+00
C1QTNF2	3.94E+00	1.06E-47	PROSER1	2.07E-01	1.00E+00	WDR66	2.84E-01	1.00E+00
PDIA4	-2.48E+00	1.07E-47	AQP3	-1.44E-01	1.00E+00	MRPL51	1.65E-01	1.00E+00
SMIM23	8.13E+00	1.11E-47	HPS6	-3.76E-01	1.00E+00	TCEANC2	-1.31E-01	1.00E+00
DMAC1	-2.85E+00	1.12E-47	NUDT2	-1.33E-01	1.00E+00	DUS3L	-1.41E-01	1.00E+00
FSCN1	-3.39E+00	1.14E-47	MRPL58	-1.23E-01	1.00E+00	CCNB2	-1.74E-01	1.00E+00
GBX1	5.44E+00	1.16E-47	LOC102399450	6.18E-01	1.00E+00	RMND5B	1.34E-01	1.00E+00
SDC2	-6.34E+00	1.17E-47	TMC8	1.98E-01	1.00E+00	ZMAT1	-2.01E-01	1.00E+00
KIAA1522	1.88E+00	1.24E-47	HMG20B	-1.25E-01	1.00E+00	BCR	1.23E-01	1.00E+00
SLC25A14	1.81E+00	1.27E-47	ALDOA	1.57E-01	1.00E+00	LOC112583734	-2.47E-01	1.00E+00
LOC102405095	-4.51E+00	1.28E-47	FUK	-1.13E-01	1.00E+00	LOC102401136	-2.59E-01	1.00E+00
SF3A3	1.28E+00	1.29E-47	CCDC25	1.23E-01	1.00E+00	FAM184B	2.54E-01	1.00E+00
KCNH2	-4.95E+00	1.29E-47	PAQR4	2.86E-01	1.00E+00	LOC102406738	1.31E-01	1.00E+00
FGF19	9.61E+00	1.35E-47	LOC112578542	-4.33E-01	1.00E+00	LOC112586829	1.80E-01	1.00E+00
LOC102407062	6.24E+00	1.35E-47	C12H9orf116	-3.12E-01	1.00E+00	TCHP	-1.25E-01	1.00E+00
LOC112584193	-2.83E+00	1.42E-47	SPRY4	2.82E-01	1.00E+00	HMGXB4	-1.34E-01	1.00E+00
PIP4P2	-5.39E+00	1.46E-47	IFFO2	3.81E-01	1.00E+00	MS4A8	4.56E-01	1.00E+00
TPGS2	-3.25E+00	1.50E-47	CMPK1	1.11E-01	1.00E+00	ATG16L1	-1.53E-01	1.00E+00
MGP	-4.82E+00	1.58E-47	FAM160A1	1.47E-01	1.00E+00	TLR10	-1.99E-01	1.00E+00
C18H19orf12	-2.09E+00	1.62E-47	CD320	1.03E-01	1.00E+00	TMEM263	-1.75E-01	1.00E+00
MFSD4B	2.75E+00	1.75E-47	LENG1	-1.54E-01	1.00E+00	LOC102408106	-2.25E-01	1.00E+00
OSBPL11	-2.16E+00	1.77E-47	EDC3	1.15E-01	1.00E+00	S100A13	2.01E-01	1.00E+00
HMG20B	-1.78E+00	1.79E-47	NGDN	-1.02E-01	1.00E+00	TMEM38A	2.55E-01	1.00E+00
NPAS3	2.87E+00	1.91E-47	PPP5C	-9.84E-02	1.00E+00	FASTK	-1.44E-01	1.00E+00
MYADM	-4.85E+00	1.93E-47	CLIP2	-2.34E-01	1.00E+00	LAMA1	1.55E-01	1.00E+00
ACAP1	-4.11E+00	1.95E-47	DDX49	1.21E-01	1.00E+00	ALMS1	-1.27E-01	1.00E+00
PSMB2	-1.95E+00	1.96E-47	UCKL1	-1.80E-01	1.00E+00	DNAJC13	1.51E-01	1.00E+00
N6AMT1	-4.13E+00	1.97E-47	FGF11	4.36E-01	1.00E+00	ARL6IP6	-1.46E-01	1.00E+00
PITX2	4.56E+00	1.98E-47	DYNLT3	-1.36E-01	1.00E+00	PC	-1.54E-01	1.00E+00
TOP2A	-2.34E+00	2.04E-47	C2H6orf106	9.02E-02	1.00E+00	LOC102400532	1.30E-01	1.00E+00
LOC102408113	-	2.17E-47	ASRGL1	-8.83E-	1.00E+00	ERCC6	-1.11E-	1.00E+00

	4.12E+00			02			01	
LBX1	7.78E+00	2.29E-47	PSMA6	-9.73E-02	1.00E+00	LOC102409025	-2.47E-01	1.00E+00
CNOT6	-1.80E+00	2.31E-47	FASLG	-2.29E-01	1.00E+00	MON2	-1.50E-01	1.00E+00
INTS7	1.39E+00	2.34E-47	CNNM4	1.39E-01	1.00E+00	PLEKHG4	3.36E-01	1.00E+00
CARD10	3.52E+00	2.38E-47	FAM136A	-1.57E-01	1.00E+00	DLGAP4	1.40E-01	1.00E+00
FNDC4	-5.57E+00	2.47E-47	LOC112583717	-2.00E-01	1.00E+00	LOC102393427	-1.77E-01	1.00E+00
TMBIM4	-1.44E+00	2.51E-47	UBAP2L	1.32E-01	1.00E+00	MOXD1	1.80E-01	1.00E+00
SLC38A9	1.56E+00	2.53E-47	LOC112580648	-5.55E-01	1.00E+00	LOC102396719	-1.89E-01	1.00E+00
LOC102415442	1.62E+00	2.54E-47	MLPH	3.06E-01	1.00E+00	LOC102405282	-2.17E-01	1.00E+00
ADAMTS8	-5.50E+00	2.79E-47	CENPN	1.48E-01	1.00E+00	PIEZO1	-1.32E-01	1.00E+00
LOC112580196	3.65E+00	2.80E-47	UNC45A	1.68E-01	1.00E+00	NEK4	-1.23E-01	1.00E+00
LONRF1	2.15E+00	2.90E-47	SMC5	1.34E-01	1.00E+00	LOC112579144	2.84E-01	1.00E+00
DLG2	4.38E+00	2.95E-47	PLCG1	1.90E-01	1.00E+00	GAMT	-1.81E-01	1.00E+00
BCL9	-2.34E+00	3.16E-47	LOC112578845	1.73E-01	1.00E+00	DOCK7	1.38E-01	1.00E+00
RORA	2.55E+00	3.33E-47	RAB23	-2.13E-01	1.00E+00	PCNX4	-1.53E-01	1.00E+00
SEL1L3	-6.24E+00	3.48E-47	GOLGA4	1.37E-01	1.00E+00	ENSA	1.15E-01	1.00E+00
ADAM15	-6.54E+00	3.59E-47	RIPK1	2.27E-01	1.00E+00	CLPB	-1.25E-01	1.00E+00
DERL1	1.57E+00	3.90E-47	IKZF4	2.46E-01	1.00E+00	KCNAB3	-2.42E-01	1.00E+00
FAM214B	-3.50E+00	4.03E-47	NFKBIA	-1.70E-01	1.00E+00	MAN1C1	2.96E-01	1.00E+00
LRPAP1	-2.53E+00	4.32E-47	GDA	1.01E-01	1.00E+00	RAB33B	1.81E-01	1.00E+00
C4H12orf66	2.60E+00	4.60E-47	LOC102400551	-1.94E-01	1.00E+00	LRP6	-1.38E-01	1.00E+00
FAM114A1	-5.92E+00	4.62E-47	CEPT1	-1.09E-01	1.00E+00	SERPINH1	-2.07E-01	1.00E+00
GRHL1	-2.50E+00	4.69E-47	LOC102398014	-2.99E-01	1.00E+00	GSPT1	1.16E-01	1.00E+00
LOC102394536	4.47E+00	4.74E-47	ATP7A	3.69E-01	1.00E+00	IGSF23	3.19E-01	1.00E+00
SEPT10	-2.74E+00	4.85E-47	GRIK3	-3.97E-01	1.00E+00	TRIM37	-1.15E-01	1.00E+00
NLGN4X	-5.36E+00	5.22E-47	LOC102410010	1.72E-01	1.00E+00	ZNF398	-1.43E-01	1.00E+00
LRRC8A	1.70E+00	5.56E-47	TTYH2	-5.50E-01	1.00E+00	PCOLCE	1.24E-01	1.00E+00
PLAG1	4.32E+00	5.59E-47	ARHGAP44	1.46E-01	1.00E+00	TGDS	-1.64E-01	1.00E+00
AKAP8	-1.44E+00	5.83E-47	NUMB	9.10E-02	1.00E+00	XPO6	1.20E-01	1.00E+00
MAN2B1	-2.20E+00	6.04E-47	NACA	1.14E-01	1.00E+00	TRMT10C	1.37E-01	1.00E+00
PCGF6	2.22E+00	6.19E-47	HDLBP	1.17E-01	1.00E+00	FOXP1	-1.38E-01	1.00E+00
CACNA2D1	3.72E+00	6.28E-47	SSBP3	1.07E-01	1.00E+00	RASSF2	-3.21E-01	1.00E+00
LOC102414554	3.96E+00	6.57E-47	HDHD5	-1.77E-01	1.00E+00	FITM2	-2.15E-01	1.00E+00
SOS2	-	6.66E-47	PCID2	1.49E-01	1.00E+00	FAN1	-1.12E-	1.00E+00

	2.66E+00						01	
COLQ	-4.31E+00	6.78E-47	LOC102406576	-1.14E-01	1.00E+00	NOSTRIN	-1.46E-01	1.00E+00
LOC102396030	6.48E+00	6.95E-47	SMIM4	-1.73E-01	1.00E+00	NTAN1	1.23E-01	1.00E+00
EFNB2	-3.07E+00	7.06E-47	RPS6KB2	2.15E-01	1.00E+00	NTNG1	1.46E-01	1.00E+00
LOC112584719	-5.47E+00	7.17E-47	LOC112581168	1.71E-01	1.00E+00	TOMM22	-1.41E-01	1.00E+00
LOC102398665	-5.86E+00	7.32E-47	LRRC31	-1.23E-01	1.00E+00	HECTD2	2.67E-01	1.00E+00
TRAM1L1	2.94E+00	7.55E-47	MRTO4	1.35E-01	1.00E+00	RFX1	-1.18E-01	1.00E+00
LOC112586667	-6.12E+00	8.59E-47	INTS9	9.16E-02	1.00E+00	CASC4	-1.26E-01	1.00E+00
NACC2	2.72E+00	8.82E-47	PPP3CA	1.87E-01	1.00E+00	TMEM179B	-1.34E-01	1.00E+00
CDC14B	-2.40E+00	9.41E-47	RABGEF1	9.41E-02	1.00E+00	LOC102399526	2.02E-01	1.00E+00
ZNF34	1.42E+00	9.42E-47	CFAP70	-2.50E-01	1.00E+00	WDR77	1.36E-01	1.00E+00
LOC102400151	-7.45E+00	9.49E-47	TEX12	-2.28E-01	1.00E+00	PCMTD2	-1.46E-01	1.00E+00
ARMH4	-1.51E+00	9.51E-47	COL1A2	2.78E-01	1.00E+00	LIPA	-1.31E-01	1.00E+00
SERPINA11	-7.86E+00	9.58E-47	GTPBP6	-2.97E-01	1.00E+00	PLA2G4A	1.18E-01	1.00E+00
ANKH	-2.88E+00	9.63E-47	GHR	-2.17E-01	1.00E+00	TEX2	1.34E-01	1.00E+00
ROCK2	-3.24E+00	9.87E-47	C18H19orf12	2.23E-01	1.00E+00	CXHXorf38	1.38E-01	1.00E+00
THBS3	-3.31E+00	1.00E-46	BLOC1S1	-2.02E-01	1.00E+00	RIC8A	1.12E-01	1.00E+00
C9H19orf53	-2.18E+00	1.03E-46	KIF11	1.14E-01	1.00E+00	RABEP1	1.11E-01	1.00E+00
PSMC3IP	2.34E+00	1.03E-46	LOC112582921	-2.67E-01	1.00E+00	IFT88	-1.16E-01	1.00E+00
DDX10	1.34E+00	1.06E-46	CANT1	-1.18E-01	1.00E+00	CXHXorf56	-1.32E-01	1.00E+00
PYGL	-2.25E+00	1.08E-46	LOC102396286	3.45E-01	1.00E+00	TRIM36	-3.94E-01	1.00E+00
SFSWAP	-1.37E+00	1.14E-46	BPHL	-2.60E-01	1.00E+00	MEX3C	-1.52E-01	1.00E+00
LOC112587866	5.34E+00	1.14E-46	TSHR	-3.35E-01	1.00E+00	GCH1	-1.82E-01	1.00E+00
ZNF775	-2.40E+00	1.15E-46	HSDL2	-2.48E-01	1.00E+00	FUCA1	-1.09E-01	1.00E+00
LRRN1	-5.41E+00	1.18E-46	PRKAA1	2.20E-01	1.00E+00	SLC30A4	-2.79E-01	1.00E+00
TRIM21	-5.88E+00	1.19E-46	PNPLA7	-2.57E-01	1.00E+00	ZNF280C	-1.51E-01	1.00E+00
UNC13B	1.67E+00	1.26E-46	CSRP1	6.19E-01	1.00E+00	ZNF362	-1.14E-01	1.00E+00
DNTTIP1	-1.91E+00	1.36E-46	LOC112582074	-3.58E-01	1.00E+00	TTC13	-1.17E-01	1.00E+00
HOXA4	3.31E+00	1.52E-46	ASTN1	-1.38E-01	1.00E+00	CHM	1.61E-01	1.00E+00
UBXN1	-2.15E+00	1.53E-46	ZC3HAV1	-5.56E-01	1.00E+00	HAPLN3	1.42E-01	1.00E+00
GLI3	-5.71E+00	1.61E-46	ST8SIA5	-5.06E-01	1.00E+00	KHK	-1.41E-01	1.00E+00
MYL6	-2.68E+00	1.66E-46	TMEM80	4.08E-01	1.00E+00	SUFU	1.49E-01	1.00E+00
HSDL1	1.34E+00	1.67E-46	PSMD10	-1.48E-	1.00E+00	CYTB	-2.82E-	1.00E+00

				01			01	
LOC102407231	-4.28E+00	1.73E-46	CDH8	-3.66E-01	1.00E+00	MSANTD3	1.43E-01	1.00E+00
ATG9A	-1.53E+00	1.73E-46	COPS5	-1.68E-01	1.00E+00	ZMYM5	-1.36E-01	1.00E+00
LOC102409097	-2.82E+00	1.74E-46	ATP5PB	-1.12E-01	1.00E+00	MX1	1.23E-01	1.00E+00
PSMC6	-1.28E+00	1.95E-46	KLB	2.89E-01	1.00E+00	SOX4	2.09E-01	1.00E+00
LOC112579160	-5.43E+00	1.96E-46	PTPN3	-9.38E-02	1.00E+00	HYI	1.93E-01	1.00E+00
CREB1	-1.63E+00	1.96E-46	PEX5	9.17E-02	1.00E+00	LHPP	-1.44E-01	1.00E+00
THAP11	1.66E+00	1.98E-46	NCAPG2	1.39E-01	1.00E+00	ZFYVE1	1.13E-01	1.00E+00
CXXC1	-1.89E+00	2.06E-46	FBXO9	-1.29E-01	1.00E+00	LOC102392624	1.40E-01	1.00E+00
LOC102409686	1.85E+00	2.12E-46	LOC112580129	-4.06E-01	1.00E+00	NELFCD	-1.21E-01	1.00E+00
SPTSSA	-3.11E+00	2.28E-46	POFUT1	1.80E-01	1.00E+00	HGS	-1.07E-01	1.00E+00
TLE2	-3.33E+00	2.29E-46	ITPRID2	2.27E-01	1.00E+00	EYA3	1.14E-01	1.00E+00
EIF4A2	-1.96E+00	2.32E-46	HGSNAT	1.05E-01	1.00E+00	LOC102407601	5.27E-01	1.00E+00
LOC112580274	-8.37E+00	2.33E-46	C3H17orf80	-2.07E-01	1.00E+00	REEP4	-1.54E-01	1.00E+00
SLITRK2	-4.92E+00	2.36E-46	TMEM214	9.66E-02	1.00E+00	LOC102398516	2.71E-01	1.00E+00
LOC102402091	-5.10E+00	2.41E-46	RPL36AL	-1.49E-01	1.00E+00	INHBB	-2.29E-01	1.00E+00
SARM1	2.03E+00	2.41E-46	HORMAD2	3.60E-01	1.00E+00	LOC112584308	-2.82E-01	1.00E+00
HLX	4.97E+00	2.69E-46	PSTPIP2	-3.63E-01	1.00E+00	FBXO33	1.66E-01	1.00E+00
ISOC2	-3.35E+00	2.69E-46	LOC112587405	-2.25E-01	1.00E+00	ZAR1L	-2.14E-01	1.00E+00
NIT2	-2.06E+00	2.71E-46	PRRG2	-1.31E-01	1.00E+00	GPR137	-1.31E-01	1.00E+00
CNST	-2.14E+00	2.78E-46	ATP7B	1.83E-01	1.00E+00	ATP8A1	-1.50E-01	1.00E+00
RASGRP3	-2.40E+00	2.85E-46	MRPS34	-2.15E-01	1.00E+00	ERBB4	2.27E-01	1.00E+00
TRIM59	2.09E+00	2.87E-46	NAA60	1.04E-01	1.00E+00	MARC1	-1.57E-01	1.00E+00
RAB3C	5.51E+00	2.89E-46	SATB1	1.34E-01	1.00E+00	TRNT1	1.27E-01	1.00E+00
DAB1	3.28E+00	2.92E-46	PCNX3	1.71E-01	1.00E+00	SKIDA1	-2.59E-01	1.00E+00
CRB1	4.52E+00	3.07E-46	DUSP5	1.15E-01	1.00E+00	HIPK1	-1.48E-01	1.00E+00
SH3GL2	3.54E+00	3.09E-46	LOC102394697	-2.59E-01	1.00E+00	STK36	-1.15E-01	1.00E+00
HSD17B8	-2.68E+00	3.09E-46	UBAC2	-1.19E-01	1.00E+00	RCC1	-1.25E-01	1.00E+00
LOC102402768	5.71E+00	3.50E-46	HEATR5A	-3.35E-01	1.00E+00	ERLIN1	-1.20E-01	1.00E+00
ZNF500	-2.79E+00	3.54E-46	PGPEP1L	6.13E-01	1.00E+00	ACYP1	2.14E-01	1.00E+00
HINT2	-2.97E+00	3.70E-46	TTI2	2.13E-01	1.00E+00	HPS5	1.36E-01	1.00E+00
CNDP2	-1.84E+00	3.76E-46	LOC102390495	-1.78E-01	1.00E+00	TRMT1	-1.32E-01	1.00E+00
ARMCX4	-6.15E+00	3.78E-46	ANAPC11	-9.80E-02	1.00E+00	LOC102392066	1.92E-01	1.00E+00

SRF	-1.96E+00	3.81E-46	DIS3L	-1.34E-01	1.00E+00	SIN3A	-1.11E-01	1.00E+00
CELF2	5.97E+00	4.15E-46	CEP89	1.44E-01	1.00E+00	LOC102393439	-2.86E-01	1.00E+00
PGD	-2.04E+00	4.39E-46	LOC112587187	-6.90E-01	1.00E+00	LOC112580302	-2.10E-01	1.00E+00
NEMP1	1.70E+00	4.39E-46	LOC102410981	3.66E-01	1.00E+00	SHC4	-1.37E-01	1.00E+00
PAPPA	-3.95E+00	4.52E-46	EFCAB8	-2.31E-01	1.00E+00	AK8	-1.68E-01	1.00E+00
MAP1LC3C	-8.68E+00	4.52E-46	OXSM	1.22E-01	1.00E+00	LOC102405195	1.44E-01	1.00E+00
BDH2	-6.42E+00	4.67E-46	CHCHD7	-1.14E-01	1.00E+00	ICA1L	2.70E-01	1.00E+00
RAD54L2	2.70E+00	4.76E-46	QRICH1	1.11E-01	1.00E+00	ARID5A	-3.33E-01	1.00E+00
DHX16	-1.59E+00	4.87E-46	MAN2C1	2.01E-01	1.00E+00	ERBIN	1.44E-01	1.00E+00
HBEGF	2.79E+00	4.90E-46	ERGIC1	1.20E-01	1.00E+00	ARHGDIA	1.29E-01	1.00E+00
RRAS	-4.02E+00	4.91E-46	FRG1	-1.41E-01	1.00E+00	CUX1	-1.21E-01	1.00E+00
SRRT	-1.43E+00	5.04E-46	LOC112581623	-2.37E-01	1.00E+00	ATG4B	-1.18E-01	1.00E+00
VASH2	-5.48E+00	5.10E-46	TYW5	-2.12E-01	1.00E+00	LOC112581399	2.27E-01	1.00E+00
ZDHHC4	-1.79E+00	5.21E-46	HKDC1	2.54E-01	1.00E+00	LSM14A	-1.16E-01	1.00E+00
INHA	-7.44E+00	5.25E-46	NSF	9.33E-02	1.00E+00	LIMK1	-1.15E-01	1.00E+00
LOC102410010	2.36E+00	5.32E-46	TMEM140	1.49E-01	1.00E+00	USP11	-1.04E-01	1.00E+00
PKP1	5.83E+00	5.63E-46	ATP5F1E	-1.35E-01	1.00E+00	SLC8B1	1.78E-01	1.00E+00
KLK4	-5.49E+00	5.71E-46	TEX264	-9.24E-02	1.00E+00	RRAGB	-1.31E-01	1.00E+00
LPGAT1	-2.02E+00	5.71E-46	N4BP2L1	4.28E-01	1.00E+00	RGS1	-5.71E-01	1.00E+00
LOC102414386	-2.14E+00	5.84E-46	ZNF513	-1.20E-01	1.00E+00	USF1	1.17E-01	1.00E+00
SNAP29	1.39E+00	5.92E-46	RPH3AL	9.36E-02	1.00E+00	C4H12orf45	-1.60E-01	1.00E+00
IFITM10	-6.23E+00	6.03E-46	PTPRD	8.92E-02	1.00E+00	MRPL21	1.43E-01	1.00E+00
ZNF12	-2.45E+00	6.04E-46	LOC102415736	-2.63E-01	1.00E+00	XK	1.98E-01	1.00E+00
TFCP2	-1.70E+00	6.29E-46	LOC112580666	-9.38E-02	1.00E+00	AGTRAP	-1.56E-01	1.00E+00
RPS2	-3.11E+00	6.39E-46	LOC102408709	-1.14E-01	1.00E+00	LZTFL1	-1.36E-01	1.00E+00
LOC112584542	7.85E+00	6.84E-46	POLE	1.55E-01	1.00E+00	ARHGEF33	-2.53E-01	1.00E+00
CMBL	-6.36E+00	6.84E-46	SOX30	-1.14E-01	1.00E+00	PLXND1	2.79E-01	1.00E+00
STXBP4	-2.09E+00	6.87E-46	ECE1	1.19E-01	1.00E+00	SHPRH	-2.04E-01	1.00E+00
TMEM26	-5.13E+00	6.88E-46	GPR3	-1.24E-01	1.00E+00	MORN2	-1.88E-01	1.00E+00
FBXO22	1.49E+00	7.30E-46	LOC112583843	2.18E-01	1.00E+00	ARL13B	-1.40E-01	1.00E+00
LOC112585185	5.31E+00	7.48E-46	FAM173B	-1.22E-01	1.00E+00	SFMBT1	1.31E-01	1.00E+00
POLR1E	1.52E+00	7.87E-46	MFSD6	1.30E-01	1.00E+00	COL14A1	2.98E-01	1.00E+00
SH2B2	2.92E+00	8.28E-46	CD2BP2	-1.42E-01	1.00E+00	ACTB	-2.22E-01	1.00E+00

LOC102392458	5.55E+00	8.33E-46	ZNF800	1.41E-01	1.00E+00	SLC4A4	-1.75E-01	1.00E+00
HAUS8	2.30E+00	8.43E-46	DNMT3B	1.51E-01	1.00E+00	FRAT2	-1.75E-01	1.00E+00
FAM241B	1.98E+00	8.51E-46	LOC102390601	-1.37E-01	1.00E+00	SF3A2	-1.19E-01	1.00E+00
AQP10	-6.04E+00	8.54E-46	MXD3	-1.90E-01	1.00E+00	IKZF3	2.69E-01	1.00E+00
DYSF	-3.77E+00	8.67E-46	L3HYPDH	-1.97E-01	1.00E+00	RDH13	1.54E-01	1.00E+00
ESPN	2.72E+00	8.92E-46	NCLN	9.00E-02	1.00E+00	LOC102404627	-3.75E-01	1.00E+00
IPCEF1	3.09E+00	9.18E-46	PDGFD	-2.60E-01	1.00E+00	FHOD1	-1.53E-01	1.00E+00
RMDN2	-2.80E+00	9.32E-46	BOD1	-1.28E-01	1.00E+00	PARP1	-1.14E-01	1.00E+00
PARS2	2.08E+00	9.66E-46	LOC112578770	-1.59E-01	1.00E+00	ZNF281	1.68E-01	1.00E+00
SENP3	1.29E+00	9.67E-46	LOC112579153	6.45E-01	1.00E+00	MED16	-1.11E-01	1.00E+00
LOC102408791	2.94E+00	1.02E-45	GEMIN2	-1.26E-01	1.00E+00	KDM7A	2.45E-01	1.00E+00
LOC102409679	-5.99E+00	1.04E-45	EFNA2	-2.74E-01	1.00E+00	KLHL24	1.57E-01	1.00E+00
STEAP1	-4.63E+00	1.07E-45	LOC102406086	2.15E-01	1.00E+00	MANSC1	-1.38E-01	1.00E+00
DCLK3	-5.13E+00	1.07E-45	ALDH1B1	-3.11E-01	1.00E+00	IMMT	-1.09E-01	1.00E+00
PCF11	-1.62E+00	1.09E-45	SDF2L1	-1.28E-01	1.00E+00	OTUB2	-2.51E-01	1.00E+00
MLST8	-2.41E+00	1.09E-45	LOC102410066	7.28E-01	1.00E+00	PTPN13	1.80E-01	1.00E+00
NFE2L2	-1.59E+00	1.10E-45	PIP4K2C	1.12E-01	1.00E+00	LOC102398520	3.17E-01	1.00E+00
TRIM38	-2.27E+00	1.13E-45	BTK	-2.08E-01	1.00E+00	SLC25A30	1.62E-01	1.00E+00
LOC102396855	7.04E+00	1.14E-45	GLI4	-1.41E-01	1.00E+00	LOC112586216	-1.54E-01	1.00E+00
NDUFB7	-2.61E+00	1.15E-45	RABEPK	-2.83E-01	1.00E+00	SLC7A8	2.81E-01	1.00E+00
ADAL	1.65E+00	1.15E-45	AGPAT3	1.23E-01	1.00E+00	CDKL1	-1.92E-01	1.00E+00
NYAP1	-3.99E+00	1.16E-45	SERPINH1	-5.42E-01	1.00E+00	ZC4H2	1.46E-01	1.00E+00
CUL5	-1.85E+00	1.31E-45	CYR61	-6.44E-01	1.00E+00	SLC35B2	1.10E-01	1.00E+00
LOC112579555	5.41E+00	1.35E-45	CDH1	1.52E-01	1.00E+00	TMEM138	-1.46E-01	1.00E+00
LOC102395109	-2.23E+00	1.36E-45	LOC112583579	-7.42E-01	1.00E+00	PHLPP2	-1.56E-01	1.00E+00
WIPI1	-5.48E+00	1.42E-45	STUM	7.36E-01	1.00E+00	ATP6V0B	1.19E-01	1.00E+00
PANK3	1.85E+00	1.44E-45	KCNK1	1.53E-01	1.00E+00	ZC3H3	-1.44E-01	1.00E+00
LOC102390348	-7.39E+00	1.47E-45	PXMP2	-1.52E-01	1.00E+00	MMEL1	-2.32E-01	1.00E+00
SH3PXD2B	-3.11E+00	1.48E-45	IFT20	-1.27E-01	1.00E+00	INHA	-2.81E-01	1.00E+00
TRAPPC5	-2.79E+00	1.48E-45	UBE2J2	-9.06E-02	1.00E+00	GFAP	-3.28E-01	1.00E+00
EPN2	1.53E+00	1.52E-45	PFDN1	-1.12E-01	1.00E+00	CHTF8	1.14E-01	1.00E+00
PTPN21	-2.16E+00	1.52E-45	ACTR2	8.64E-02	1.00E+00	ERP44	1.15E-01	1.00E+00

LOC112579560	4.64E+00	1.60E-45	DYNLRB2	-2.39E-01	1.00E+00	TCF7L2	-1.41E-01	1.00E+00
PPARGC1A	3.51E+00	1.65E-45	AP5S1	-1.40E-01	1.00E+00	KCTD9	-1.35E-01	1.00E+00
GPR173	-2.23E+00	1.85E-45	TOR3A	1.34E-01	1.00E+00	TSSK3	2.58E-01	1.00E+00
KIF3A	1.81E+00	1.92E-45	LOC102399011	1.18E-01	1.00E+00	SNX6	1.07E-01	1.00E+00
ARHGAP28	-5.53E+00	1.96E-45	ATP2A1	4.77E-01	1.00E+00	PSMD2	1.23E-01	1.00E+00
ZSWIM7	-2.46E+00	1.96E-45	UPB1	-4.69E-01	1.00E+00	ATP5MC3	1.29E-01	1.00E+00
LOC102393736	6.67E+00	1.97E-45	ADGRB3	1.76E-01	1.00E+00	ZNF639	-1.24E-01	1.00E+00
PTAR1	-2.58E+00	2.05E-45	GP1BA	-1.78E-01	1.00E+00	PRDX6	1.45E-01	1.00E+00
ST6GALNAC1	6.38E+00	2.17E-45	LOC112582910	-5.31E-01	1.00E+00	LOC102397046	-2.07E-01	1.00E+00
PELI1	2.03E+00	2.18E-45	LOC102397670	-2.09E-01	1.00E+00	LOC102393233	4.87E-01	1.00E+00
LOC102400844	-5.69E+00	2.19E-45	TMTC4	1.75E-01	1.00E+00	LOC102412762	2.35E-01	1.00E+00
C12H2orf40	-7.82E+00	2.19E-45	RRP7A	1.36E-01	1.00E+00	RALYL	1.89E-01	1.00E+00
RPL27	-2.93E+00	2.20E-45	SLC29A1	1.39E-01	1.00E+00	CDR1	-5.07E-01	1.00E+00
SERPINH1	-7.31E+00	2.23E-45	IL1RAPL1	-2.37E-01	1.00E+00	NDUFA4	1.68E-01	1.00E+00
GPR84	6.98E+00	2.30E-45	LOC102399929	-1.27E-01	1.00E+00	MICALL1	1.10E-01	1.00E+00
TNFAIP8L1	-2.45E+00	2.36E-45	RPL7	-2.96E-01	1.00E+00	STOML1	-1.22E-01	1.00E+00
S100A2	-7.69E+00	2.40E-45	DOT1L	1.71E-01	1.00E+00	BACH2	2.23E-01	1.00E+00
RNF121	1.37E+00	2.41E-45	TLK2	1.37E-01	1.00E+00	MEX3B	-2.01E-01	1.00E+00
RSBN1	-3.28E+00	2.45E-45	PDZD11	-1.31E-01	1.00E+00	TMEM165	-1.55E-01	1.00E+00
MESD	-1.57E+00	2.54E-45	SLC45A2	-1.57E-01	1.00E+00	TMEM245	-2.17E-01	1.00E+00
RNF219	1.51E+00	2.56E-45	SLC6A9	-5.22E-01	1.00E+00	LOC102395095	1.47E-01	1.00E+00
LOC102406990	-4.15E+00	2.56E-45	HEATR5B	1.82E-01	1.00E+00	FN3KRP	-1.62E-01	1.00E+00
SQOR	2.43E+00	2.59E-45	RNASEH2A	-1.25E-01	1.00E+00	GCNT3	3.16E-01	1.00E+00
PCNA	1.96E+00	2.76E-45	MYH9	1.40E-01	1.00E+00	SLC38A2	1.45E-01	1.00E+00
THOP1	1.83E+00	2.81E-45	FSIP2	-3.58E-01	1.00E+00	ITGA11	3.35E-01	1.00E+00
LOC102416007	2.76E+00	2.85E-45	NUDT12	-2.43E-01	1.00E+00	CCDC28A	1.58E-01	1.00E+00
CPNE9	2.96E+00	2.88E-45	TONSL	1.12E-01	1.00E+00	LOC112583111	2.88E-01	1.00E+00
VEGFC	-2.10E+00	2.94E-45	UBE3C	1.10E-01	1.00E+00	MRPS16	1.59E-01	1.00E+00
LOC112582136	-6.24E+00	2.96E-45	CDC42BPG	1.52E-01	1.00E+00	COMMD5	1.33E-01	1.00E+00
PDIK1L	-3.27E+00	3.15E-45	GAL3ST2	-3.68E-01	1.00E+00	PCOLCE2	1.09E-01	1.00E+00
GCSH	-2.46E+00	3.17E-45	LOC112584742	-3.21E-01	1.00E+00	LOC112586436	1.45E-01	1.00E+00
DALRD3	-3.23E+00	3.24E-45	CORO6	9.42E-02	1.00E+00	ACTR5	1.33E-01	1.00E+00
ADAM9	-2.56E+00	3.28E-45	MED1	1.18E-01	1.00E+00	NUP93	-1.17E-01	1.00E+00

EEF1A1	-2.22E+00	3.33E-45	HACL1	-1.34E-01	1.00E+00	TNIP2	1.52E-01	1.00E+00
PARP14	-3.76E+00	3.35E-45	PNPO	-3.64E-01	1.00E+00	ATP6	-2.60E-01	1.00E+00
PXK	1.73E+00	3.47E-45	FBXO10	1.08E-01	1.00E+00	NSMAF	-1.06E-01	1.00E+00
NOS1	5.59E+00	3.48E-45	C8H7orf25	-9.75E-02	1.00E+00	BACE1	-1.05E-01	1.00E+00
CLDN12	1.87E+00	3.52E-45	SLC9A3R2	5.28E-01	1.00E+00	DNAI1	-1.69E-01	1.00E+00
SCD	-5.32E+00	3.58E-45	TBX18	2.88E-01	1.00E+00	POT1	1.31E-01	1.00E+00
EMC2	-2.24E+00	3.71E-45	SSB	9.41E-02	1.00E+00	DPM1	-1.33E-01	1.00E+00
RFPL4A	6.97E+00	3.71E-45	TRIQK	1.43E-01	1.00E+00	PTRH2	1.59E-01	1.00E+00
HNRNPA3	-1.58E+00	3.72E-45	PEAK1	1.88E-01	1.00E+00	SLC45A2	2.52E-01	1.00E+00
FAM69A	2.15E+00	3.73E-45	HAAO	4.06E-01	1.00E+00	AAR2	1.36E-01	1.00E+00
LOC112585054	-5.03E+00	3.84E-45	ATP11A	-1.72E-01	1.00E+00	KIZ	-1.25E-01	1.00E+00
FYCO1	-1.71E+00	3.89E-45	OGFR	-1.11E-01	1.00E+00	NSMCE4A	-1.12E-01	1.00E+00
ABCD1	-2.85E+00	3.93E-45	FKBP5	-1.24E-01	1.00E+00	IER3IP1	-1.18E-01	1.00E+00
ITSN1	-1.87E+00	4.02E-45	RRP12	1.59E-01	1.00E+00	C15H8orf59	1.72E-01	1.00E+00
TPST1	1.51E+00	4.06E-45	RANGRF	-1.12E-01	1.00E+00	IL15RA	1.90E-01	1.00E+00
LOC102405384	-4.54E+00	4.13E-45	VKORC1	-1.42E-01	1.00E+00	CEBPZOS	-1.64E-01	1.00E+00
LOC102398106	1.56E+00	4.28E-45	PDHX	9.64E-02	1.00E+00	WRN	-1.40E-01	1.00E+00
TMOD1	1.70E+00	4.44E-45	NXN	-9.81E-02	1.00E+00	TMUB2	1.16E-01	1.00E+00
FURIN	1.48E+00	4.44E-45	CATSPER2	-2.62E-01	1.00E+00	PHETA2	1.59E-01	1.00E+00
SMC1A	-1.51E+00	4.45E-45	PGM3	-1.21E-01	1.00E+00	ZNF48	1.36E-01	1.00E+00
MUC4	5.22E+00	4.99E-45	CELSR1	-3.85E-01	1.00E+00	TSC22D2	-1.55E-01	1.00E+00
CCS	-2.09E+00	5.03E-45	SLC5A9	3.69E-01	1.00E+00	CFL2	1.28E-01	1.00E+00
SPSB1	-6.12E+00	5.20E-45	LOC102391411	1.51E-01	1.00E+00	SYNM	-1.61E-01	1.00E+00
LOC102394506	8.13E+00	5.21E-45	MRPS25	-1.35E-01	1.00E+00	PDGFC	1.18E-01	1.00E+00
MRPL24	-2.68E+00	5.30E-45	SHANK2	-2.35E-01	1.00E+00	UPK1B	-1.71E-01	1.00E+00
ASB1	1.82E+00	5.79E-45	EFCAB2	4.46E-01	1.00E+00	MPHOSPH6	-1.46E-01	1.00E+00
AEN	-3.21E+00	5.86E-45	NLRX1	-4.80E-01	1.00E+00	PNKP	-1.28E-01	1.00E+00
UBAC2	-1.29E+00	6.04E-45	EIF4G1	1.13E-01	1.00E+00	LOC102399489	1.45E-01	1.00E+00
LRRC41	-3.45E+00	6.05E-45	PDGFB	-4.14E-01	1.00E+00	RNF40	1.01E-01	1.00E+00
SON	-2.06E+00	6.06E-45	LOC102399587	-4.72E-01	1.00E+00	TP53	-1.07E-01	1.00E+00
PSMB6	-2.49E+00	6.12E-45	LETM2	1.71E-01	1.00E+00	UTP20	1.30E-01	1.00E+00
ABHD16A	-1.54E+00	6.24E-45	ZNF286A	1.10E-01	1.00E+00	JAKMIP3	-2.13E-01	1.00E+00
CHP1	1.36E+00	6.30E-45	EXOC3L4	-3.21E-01	1.00E+00	B3GNTL1	2.47E-01	1.00E+00

PTGR1	-6.93E+00	6.30E-45	CLU	1.11E-01	1.00E+00	UNC5C	-1.33E-01	1.00E+00
KNTC1	2.78E+00	6.52E-45	DDX50	-1.13E-01	1.00E+00	MTF1	-1.82E-01	1.00E+00
SPATS2L	-4.36E+00	6.62E-45	TLCD2	1.45E-01	1.00E+00	MCM2	-1.51E-01	1.00E+00
LOC102396662	-5.18E+00	6.66E-45	NEO1	1.50E-01	1.00E+00	CCDC9	-1.16E-01	1.00E+00
STXBP6	6.41E+00	6.72E-45	LOC112586165	5.81E-01	1.00E+00	LRRIQ3	2.22E-01	1.00E+00
LOC112587918	4.38E+00	6.94E-45	LYRM9	-2.17E-01	1.00E+00	SLC30A1	-1.35E-01	1.00E+00
LRRC38	7.40E+00	7.05E-45	ARFGEF2	1.74E-01	1.00E+00	LOC102404409	1.75E-01	1.00E+00
SPECC1	-1.96E+00	7.05E-45	RUNDC3A	-4.13E-01	1.00E+00	EFCAB10	2.45E-01	1.00E+00
COPG1	-1.62E+00	7.13E-45	LOC102389189	5.08E-01	1.00E+00	GPC2	2.34E-01	1.00E+00
SEC61A2	1.32E+00	7.24E-45	NEMF	1.04E-01	1.00E+00	RMI2	-1.80E-01	1.00E+00
DOLPP1	-2.47E+00	7.37E-45	PDIA6	9.60E-02	1.00E+00	CTIF	1.22E-01	1.00E+00
ELOVL1	-1.69E+00	7.42E-45	MUC13	4.44E-01	1.00E+00	FOXJ2	1.20E-01	1.00E+00
SART3	1.30E+00	7.44E-45	MAP10	-1.40E-01	1.00E+00	ORMDL3	1.17E-01	1.00E+00
DCHS1	-6.44E+00	7.49E-45	KIAA0895L	1.05E-01	1.00E+00	LOC102398591	1.51E-01	1.00E+00
ST13	-1.51E+00	7.81E-45	GMPS	9.79E-02	1.00E+00	HERC2	-1.35E-01	1.00E+00
CCDC50	1.35E+00	8.05E-45	YBX3	-2.20E-01	1.00E+00	CUL1	-1.02E-01	1.00E+00
SPRYD7	1.68E+00	8.13E-45	SEC22B	1.11E-01	1.00E+00	APPL2	1.18E-01	1.00E+00
STIM1	-1.41E+00	8.21E-45	LOC112578161	-4.60E-01	1.00E+00	BNIP1	-1.22E-01	1.00E+00
ARC	-4.44E+00	8.50E-45	GAK	9.88E-02	1.00E+00	PGRMC1	-1.11E-01	1.00E+00
ZMYND8	2.11E+00	8.54E-45	SFRP4	1.06E+00	1.00E+00	LOC102402015	-2.10E-01	1.00E+00
DLGAP1	1.55E+00	8.67E-45	RRN3	9.99E-02	1.00E+00	GLP2R	-3.24E-01	1.00E+00
ZADH2	-3.53E+00	8.73E-45	TAGAP	-2.21E-01	1.00E+00	LOC112581451	-2.83E-01	1.00E+00
RPP25L	-4.72E+00	8.84E-45	PCNX1	1.41E-01	1.00E+00	CASTOR2	1.13E-01	1.00E+00
TRO	-3.99E+00	8.89E-45	CUL1	1.09E-01	1.00E+00	CBFA2T3	2.71E-01	1.00E+00
COLGALT2	-5.48E+00	9.52E-45	CHST14	-2.73E-01	1.00E+00	EXT2	-1.08E-01	1.00E+00
QDPR	-1.77E+00	9.69E-45	LHX6	-1.61E-01	1.00E+00	PLP2	1.27E-01	1.00E+00
PMVK	-2.18E+00	9.69E-45	LOC112582352	3.24E-01	1.00E+00	C18H19orf12	1.23E-01	1.00E+00
LOC102407872	1.48E+00	9.76E-45	CDK10	-2.02E-01	1.00E+00	LOC102394527	-1.42E-01	1.00E+00
ZNF48	-4.74E+00	1.03E-44	ELOVL6	-1.44E-01	1.00E+00	PRKCI	-1.62E-01	1.00E+00
TLE4	1.80E+00	1.06E-44	EGFL7	-5.68E-01	1.00E+00	LOC112580443	2.49E-01	1.00E+00
LOC102416091	-4.05E+00	1.07E-44	TRIM55	-5.70E-01	1.00E+00	SCRIB	-1.12E-01	1.00E+00
KCNAB1	-3.81E+00	1.15E-44	ZSCAN2	3.17E-01	1.00E+00	EHD1	1.18E-01	1.00E+00
LOC102409673	1.98E+00	1.16E-44	PTGIS	-2.14E-01	1.00E+00	LOC112581638	2.34E-01	1.00E+00

YPEL3	1.82E+00	1.18E-44	HSBP1	-1.02E-01	1.00E+00	C19H5orf22	-1.34E-01	1.00E+00
BCOR	-2.81E+00	1.18E-44	TFDP2	-1.64E-01	1.00E+00	ANKS3	1.33E-01	1.00E+00
VPS8	1.28E+00	1.26E-44	SDC2	-6.30E-01	1.00E+00	ZNHIT3	1.62E-01	1.00E+00
DHX38	-1.79E+00	1.26E-44	METRNL	-9.70E-02	1.00E+00	ADGRA1	2.26E-01	1.00E+00
PGGHG	-5.61E+00	1.26E-44	PDF	-2.07E-01	1.00E+00	PGK1	1.50E-01	1.00E+00
C3H8orf58	3.51E+00	1.31E-44	ABCB8	1.35E-01	1.00E+00	SDC1	2.91E-01	1.00E+00
EPB41L5	1.24E+00	1.32E-44	LOC112578876	-3.08E-01	1.00E+00	FBXO38	-2.01E-01	1.00E+00
CATSPERE	-4.51E+00	1.33E-44	ACVR2A	3.35E-01	1.00E+00	RPS6KA5	-1.20E-01	1.00E+00
SPRED2	3.03E+00	1.49E-44	ZBTB18	-1.82E-01	1.00E+00	ERMP1	-1.09E-01	1.00E+00
TTC38	-2.86E+00	1.50E-44	ERI2	2.46E-01	1.00E+00	LOC102394020	2.54E-01	1.00E+00
LOC112581628	9.29E+00	1.50E-44	LOC102401999	1.06E-01	1.00E+00	TKT	-1.73E-01	1.00E+00
SEMA6C	-5.68E+00	1.50E-44	IRGQ	1.57E-01	1.00E+00	C11H15orf41	1.44E-01	1.00E+00
CHRDL1	-5.55E+00	1.53E-44	SCAF8	1.14E-01	1.00E+00	LOC112581371	3.80E-01	1.00E+00
SREBF2	-2.16E+00	1.57E-44	FRRS1	-3.05E-01	1.00E+00	CCDC66	-1.27E-01	1.00E+00
RPL18	-3.10E+00	1.58E-44	GTPBP1	-8.41E-02	1.00E+00	LRRN4CL	-1.21E-01	1.00E+00
RANGAP1	-1.84E+00	1.59E-44	LOC102407763	-4.39E-01	1.00E+00	MYRF	2.64E-01	1.00E+00
LOC102414371	-2.23E+00	1.59E-44	LOC112586481	-4.66E-01	1.00E+00	LOC102410676	-1.17E-01	1.00E+00
DFFB	-3.30E+00	1.60E-44	TJAP1	1.16E-01	1.00E+00	DYNC1H1	1.42E-01	1.00E+00
RIOK1	1.68E+00	1.67E-44	CIDEA	2.05E-01	1.00E+00	CAMTA1	1.54E-01	1.00E+00
EIF2AK1	1.33E+00	1.69E-44	ETS2	-3.63E-01	1.00E+00	MAP2K3	1.48E-01	1.00E+00
LOC102410433	-6.48E+00	1.77E-44	SERP2	-2.84E-01	1.00E+00	PARD6A	-2.25E-01	1.00E+00
LRAT	5.47E+00	1.83E-44	RAB11FIP3	9.80E-02	1.00E+00	MATR3	-1.37E-01	1.00E+00
KPNA2	1.80E+00	1.86E-44	LOC112581457	-1.22E-01	1.00E+00	TMEM131	1.28E-01	1.00E+00
G3BP2	1.88E+00	1.88E-44	OLA1	-1.96E-01	1.00E+00	LMNB1	-1.18E-01	1.00E+00
NCOA7	-3.08E+00	1.92E-44	TNIP3	-1.99E-01	1.00E+00	CEP57	-1.27E-01	1.00E+00
RPRD1B	-1.88E+00	1.96E-44	LOC112588004	-1.91E-01	1.00E+00	PAK2	1.24E-01	1.00E+00
NELFA	1.46E+00	1.97E-44	VIPAS39	-9.43E-02	1.00E+00	NME9	-2.43E-01	1.00E+00
SH2D7	3.95E+00	2.01E-44	HMGN3	-2.18E-01	1.00E+00	ROCK1	1.40E-01	1.00E+00
SLC22A3	-6.38E+00	2.08E-44	POLD2	1.15E-01	1.00E+00	PRSS50	2.60E-01	1.00E+00
SYNE4	5.50E+00	2.12E-44	EYA2	-9.38E-02	1.00E+00	MFSD4B	-2.19E-01	1.00E+00
EGFR	-2.37E+00	2.14E-44	HNRNPH1	1.33E-01	1.00E+00	LOC112585727	2.27E-01	1.00E+00
NUBP2	-2.82E+00	2.21E-44	GADD45A	-1.71E-01	1.00E+00	ACVR2A	1.38E-01	1.00E+00
POLA2	1.92E+00	2.34E-44	ARSI	3.19E-01	1.00E+00	LOC102412702	-1.68E-01	1.00E+00

LOC112583888	-4.70E+00	2.34E-44	CREB3L4	-4.14E-01	1.00E+00	GTF2H3	-1.36E-01	1.00E+00
USE1	-2.57E+00	2.51E-44	C12H2orf68	3.56E-01	1.00E+00	LOC112578471	2.33E-01	1.00E+00
CDC25B	2.25E+00	2.51E-44	UTP14A	1.06E-01	1.00E+00	WIPF1	2.59E-01	1.00E+00
LTB4R	-5.70E+00	2.67E-44	TBC1D2B	1.28E-01	1.00E+00	RBCK1	-1.46E-01	1.00E+00
MKX	-5.22E+00	2.72E-44	ETNK1	1.36E-01	1.00E+00	IFNAR2	1.31E-01	1.00E+00
SH3TC2	-3.96E+00	2.91E-44	SLC16A10	2.09E-01	1.00E+00	EVC2	1.12E-01	1.00E+00
NR5A1	-5.55E+00	3.01E-44	HMCES	8.55E-02	1.00E+00	LOC102392517	1.78E-01	1.00E+00
FERMT1	1.82E+00	3.06E-44	RAPGEF1	9.74E-02	1.00E+00	RPS27	-1.32E-01	1.00E+00
LZTS3	-1.92E+00	3.46E-44	FER	1.36E-01	1.00E+00	RABGGTB	-1.01E-01	1.00E+00
PBX4	4.97E+00	3.63E-44	CNNM2	1.21E-01	1.00E+00	EML5	-1.66E-01	1.00E+00
C2CD2	1.90E+00	3.66E-44	ALDH9A1	-9.90E-02	1.00E+00	TMCO6	-1.11E-01	1.00E+00
NUMB	2.05E+00	3.68E-44	LOC112583969	-4.36E-01	1.00E+00	LOC112579161	2.59E-01	1.00E+00
LASP1	1.49E+00	3.69E-44	CAMK2A	-6.41E-01	1.00E+00	TPRN	1.48E-01	1.00E+00
PLPP5	-1.76E+00	3.79E-44	GRK4	-2.84E-01	1.00E+00	RTN4IP1	1.24E-01	1.00E+00
LOC112580637	-6.63E+00	3.86E-44	ELK3	-2.01E-01	1.00E+00	REX1BD	1.65E-01	1.00E+00
SLC7A1	-3.17E+00	3.89E-44	MILR1	4.90E-01	1.00E+00	UBP1	-1.76E-01	1.00E+00
EYA1	6.98E+00	3.89E-44	FAM50A	-1.00E-01	1.00E+00	TRMT6	-1.26E-01	1.00E+00
LOC102413296	-5.36E+00	3.97E-44	USB1	8.62E-02	1.00E+00	ETFRF1	-1.75E-01	1.00E+00
ATAD2	-2.34E+00	4.04E-44	LOC112580012	5.19E-01	1.00E+00	LRRC71	-1.67E-01	1.00E+00
BAIAP2L1	2.01E+00	4.05E-44	PPP6R1	-1.22E-01	1.00E+00	LOC102389957	-1.41E-01	1.00E+00
C17H4orf46	-3.31E+00	4.05E-44	LMCD1	-2.62E-01	1.00E+00	MRPL30	1.39E-01	1.00E+00
MFSD5	-2.09E+00	4.07E-44	RBM4	8.71E-02	1.00E+00	HYAL2	1.14E-01	1.00E+00
CLEC14A	-5.37E+00	4.23E-44	RNF41	2.60E-01	1.00E+00	AKAP13	1.56E-01	1.00E+00
ATE1	1.22E+00	4.27E-44	SYNE4	-3.19E-01	1.00E+00	LOC102394622	1.51E-01	1.00E+00
DNAH17	1.94E+00	4.30E-44	SMUG1	2.62E-01	1.00E+00	ATP5MC1	1.55E-01	1.00E+00
B9D2	-2.94E+00	4.49E-44	DDX4	-1.18E-01	1.00E+00	MBNL1	1.34E-01	1.00E+00
INSR	-6.44E+00	4.56E-44	KHDRBS1	1.26E-01	1.00E+00	TOX2	-1.15E-01	1.00E+00
UPF3A	1.42E+00	4.63E-44	LOC102399215	2.60E-01	1.00E+00	CAVIN4	2.31E-01	1.00E+00
IQCA1L	6.50E+00	4.64E-44	HNRNPLL	-3.30E-01	1.00E+00	AP4M1	1.29E-01	1.00E+00
NKAP	1.41E+00	4.68E-44	VSTM2A	-1.72E-01	1.00E+00	RNF220	-1.09E-01	1.00E+00
RABEP1	1.27E+00	4.90E-44	PPARG	1.40E-01	1.00E+00	LOC112587008	-2.74E-01	1.00E+00
SNRPC	-2.16E+00	4.94E-44	SGPP1	-4.18E-01	1.00E+00	RAP1GDS1	1.02E-01	1.00E+00
LOC102397338	6.21E+00	4.95E-44	PAN3	1.66E-01	1.00E+00	ARMC10	1.24E-01	1.00E+00
USP13	2.49E+00	5.27E-44	LOC112586106	-3.48E-	1.00E+00	MED25	-1.11E-	1.00E+00

				01			01	
MPND	1.57E+00	5.56E-44	TMEM192	1.30E-01	1.00E+00	SYNRG	-1.01E-01	1.00E+00
LOC102403104	-5.54E+00	5.73E-44	GORASP1	9.16E-02	1.00E+00	DDX52	-1.21E-01	1.00E+00
CAPZA2	-1.35E+00	5.75E-44	CASKIN1	-3.35E-01	1.00E+00	FADD	1.37E-01	1.00E+00
PARP8	-4.63E+00	5.79E-44	DAO	6.18E-01	1.00E+00	GLE1	-1.05E-01	1.00E+00
HTD2	-2.10E+00	5.85E-44	QTRT2	1.82E-01	1.00E+00	NR5A2	-1.55E-01	1.00E+00
LOC102413176	-2.75E+00	5.86E-44	ARFIP2	1.09E-01	1.00E+00	RRAGA	1.12E-01	1.00E+00
AAMDC	-7.04E+00	5.94E-44	BRAT1	-1.72E-01	1.00E+00	CCDC137	1.35E-01	1.00E+00
RGS2	2.66E+00	6.12E-44	RMI2	-1.44E-01	1.00E+00	HSPD1	-1.41E-01	1.00E+00
SIRT1	-2.47E+00	6.21E-44	FAM53C	1.02E-01	1.00E+00	SPRED2	2.49E-01	1.00E+00
PIR	2.33E+00	6.29E-44	TAS1R1	5.63E-01	1.00E+00	LOC102400966	2.29E-01	1.00E+00
LOC102410341	-8.25E+00	6.52E-44	HSF1	9.44E-02	1.00E+00	SLC25A27	-1.31E-01	1.00E+00
LOC102392295	6.03E+00	6.58E-44	KLF2	1.00E-01	1.00E+00	LOC102406196	-2.63E-01	1.00E+00
LOC102403818	-2.12E+00	6.60E-44	CIRBP	-1.12E-01	1.00E+00	GID4	-1.17E-01	1.00E+00
ACADL	-5.19E+00	6.72E-44	GAP43	4.32E-01	1.00E+00	SPTB	1.88E-01	1.00E+00
LOC112584557	5.47E+00	6.91E-44	CD19	-3.65E-01	1.00E+00	AGPAT5	-1.24E-01	1.00E+00
EGR1	-4.96E+00	7.13E-44	UBE2G1	8.47E-02	1.00E+00	CCDC38	1.66E-01	1.00E+00
RBBP9	-3.59E+00	7.21E-44	CRBN	9.54E-02	1.00E+00	ZC3H4	-1.09E-01	1.00E+00
RUNX2	-4.29E+00	7.64E-44	RELCH	1.19E-01	1.00E+00	BLM	-1.28E-01	1.00E+00
BBS12	1.56E+00	7.72E-44	LPCAT3	1.34E-01	1.00E+00	TNKS1BP1	1.05E-01	1.00E+00
CHMP4A	-6.35E+00	7.90E-44	COQ10A	-9.15E-02	1.00E+00	MXI1	1.33E-01	1.00E+00
ANXA3	-6.27E+00	8.08E-44	SGMS1	-1.52E-01	1.00E+00	ARID1B	-1.15E-01	1.00E+00
UBALD2	-2.20E+00	8.12E-44	KCNH8	-2.04E-01	1.00E+00	TIMM8B	1.68E-01	1.00E+00
SIAH2	-1.98E+00	8.43E-44	RPS7	-1.28E-01	1.00E+00	MRPL13	1.47E-01	1.00E+00
IGSF8	-6.55E+00	8.87E-44	MTFR1L	-9.44E-02	1.00E+00	TMEM209	-1.38E-01	1.00E+00
LRRC1	-1.54E+00	9.15E-44	SCUBE2	-3.57E-01	1.00E+00	TAP2	1.20E-01	1.00E+00
NOM1	1.44E+00	9.56E-44	PABPC1	9.30E-02	1.00E+00	FAR1	-1.38E-01	1.00E+00
ZNF70	-3.17E+00	9.56E-44	ADCY9	1.20E-01	1.00E+00	ACSF2	-1.91E-01	1.00E+00
LOC102405090	7.68E+00	9.83E-44	TBC1D13	1.02E-01	1.00E+00	LOC102396662	-1.94E-01	1.00E+00
STXBP1	1.67E+00	1.00E-43	FAM20A	-4.21E-01	1.00E+00	UBXN8	-1.24E-01	1.00E+00
ZFP1	2.03E+00	1.03E-43	LOC112579960	-1.50E-01	1.00E+00	WARS2	2.06E-01	1.00E+00
LOC112580409	3.77E+00	1.08E-43	C5H1orf115	5.37E-01	1.00E+00	PARD6B	2.08E-01	1.00E+00
FANCD2	1.55E+00	1.09E-43	CCNY	1.00E-01	1.00E+00	YAP1	1.06E-01	1.00E+00
FBXO17	-4.05E+00	1.10E-43	LOC102406739	3.11E-01	1.00E+00	POMGNT2	1.36E-01	1.00E+00

GAPDHS	-4.20E+00	1.10E-43	SENP3	8.49E-02	1.00E+00	NFYA	1.28E-01	1.00E+00
SCN2A	3.07E+00	1.12E-43	MC4R	-3.30E-01	1.00E+00	C2CD2	1.74E-01	1.00E+00
LEAP2	5.16E+00	1.13E-43	SPN	4.20E-01	1.00E+00	SIRT2	1.05E-01	1.00E+00
ACTR5	-3.06E+00	1.13E-43	IFRD1	1.28E-01	1.00E+00	LOC112583954	2.97E-01	1.00E+00
LOC102412347	3.80E+00	1.20E-43	CLMP	3.72E-01	1.00E+00	SIRT7	-1.29E-01	1.00E+00
COL6A3	-6.08E+00	1.21E-43	FCRLB	3.31E-01	1.00E+00	U2AF2	-1.15E-01	1.00E+00
CALM2	-2.21E+00	1.22E-43	DDX20	-8.40E-02	1.00E+00	LOC102409612	-2.44E-01	1.00E+00
NSG2	2.56E+00	1.22E-43	ADCY2	4.28E-01	1.00E+00	ALDH16A1	1.19E-01	1.00E+00
RBM11	2.28E+00	1.30E-43	KIF13B	1.61E-01	1.00E+00	MRPS26	1.90E-01	1.00E+00
LOC102402713	-6.23E+00	1.33E-43	LOC102389871	-2.86E-01	1.00E+00	STAT3	1.48E-01	1.00E+00
SPHK2	-1.60E+00	1.34E-43	C10H6orf120	-1.19E-01	1.00E+00	LOC102402583	-1.42E-01	1.00E+00
GABRB3	3.11E+00	1.37E-43	MORF4L2	-8.89E-02	1.00E+00	AKAP4	3.09E-01	1.00E+00
RHOA	1.98E+00	1.38E-43	MFSD5	-2.24E-01	1.00E+00	FUS	-1.43E-01	1.00E+00
LOC112583626	-4.15E+00	1.43E-43	NDUFS6	-2.00E-01	1.00E+00	INPP5D	4.34E-01	1.00E+00
NRDE2	-2.43E+00	1.49E-43	OVCH1	1.19E-01	1.00E+00	PTK6	2.44E-01	1.00E+00
C5H1orf112	1.36E+00	1.51E-43	OAS2	-5.31E-01	1.00E+00	MAGEH1	-1.26E-01	1.00E+00
TNPO3	-1.25E+00	1.62E-43	CCDC73	2.36E-01	1.00E+00	XRCC2	-1.64E-01	1.00E+00
HHIPL1	-6.90E+00	1.65E-43	IGFBP2	-1.54E-01	1.00E+00	P2RX4	-1.17E-01	1.00E+00
WDR77	1.55E+00	1.66E-43	LMBR1L	1.15E-01	1.00E+00	LOC112580842	-2.76E-01	1.00E+00
KIAA1551	-2.57E+00	1.66E-43	TMEM263	1.43E-01	1.00E+00	CENPBD1	-2.08E-01	1.00E+00
ARL2BP	1.21E+00	1.70E-43	FAM222A	-2.86E-01	1.00E+00	EME2	-1.53E-01	1.00E+00
CCNF	-2.19E+00	1.71E-43	TEX30	-1.16E-01	1.00E+00	ARHGEF38	3.02E-01	1.00E+00
NFS1	-1.72E+00	1.76E-43	IL6R	-6.18E-01	1.00E+00	HMGA2	2.74E-01	1.00E+00
SLC24A2	6.53E+00	1.78E-43	WNT9B	-2.39E-01	1.00E+00	ZSCAN26	-1.52E-01	1.00E+00
STUB1	-2.03E+00	1.82E-43	ADIPOR2	-9.76E-02	1.00E+00	CCT4	1.16E-01	1.00E+00
GTPBP4	1.52E+00	1.82E-43	LOC102403557	-2.99E-01	1.00E+00	MLXIP	1.21E-01	1.00E+00
PEX7	1.45E+00	1.84E-43	BNIP1	1.27E-01	1.00E+00	MVK	-1.49E-01	1.00E+00
FBXW5	-2.09E+00	2.09E-43	PPP2R3B	8.16E-02	1.00E+00	LOC102406285	-1.82E-01	1.00E+00
ARSA	-2.12E+00	2.09E-43	EIF2B5	1.78E-01	1.00E+00	MRPS5	-1.10E-01	1.00E+00
ULK3	-2.02E+00	2.19E-43	SSR1	8.60E-02	1.00E+00	MGST3	1.53E-01	1.00E+00
ABHD2	-2.42E+00	2.22E-43	EPS8	1.03E-01	1.00E+00	LIMD1	-2.06E-01	1.00E+00
WDR53	1.89E+00	2.22E-43	MCUB	-1.08E-01	1.00E+00	ZBBX	2.51E-01	1.00E+00
BMP7	6.31E+00	2.25E-43	CA11	5.27E-01	1.00E+00	DOCK9	-1.37E-01	1.00E+00

ZNF503	-1.82E+00	2.27E-43	TRIP11	1.17E-01	1.00E+00	PLCB4	-1.10E-01	1.00E+00
ARHGAP20	-2.60E+00	2.36E-43	SCYL3	1.39E-01	1.00E+00	NAA35	-1.06E-01	1.00E+00
MRAP2	5.28E+00	2.42E-43	ADGRG7	-1.66E-01	1.00E+00	CCDC186	-1.58E-01	1.00E+00
DOCK3	1.98E+00	2.44E-43	AK5	5.28E-01	1.00E+00	LOC102404996	-1.84E-01	1.00E+00
ZNF784	-3.40E+00	2.54E-43	GDPGP1	1.62E-01	1.00E+00	LOC102413900	-2.77E-01	1.00E+00
DNMT1	1.69E+00	2.54E-43	PYURF	-1.44E-01	1.00E+00	PLEKHB1	-1.25E-01	1.00E+00
MUC13	-5.00E+00	2.55E-43	ODF2	1.01E-01	1.00E+00	COL16A1	1.82E-01	1.00E+00
NUDT13	3.27E+00	2.60E-43	KRAS	2.63E-01	1.00E+00	GNB3	-2.51E-01	1.00E+00
NKAPD1	1.34E+00	2.62E-43	PRELID2	-1.55E-01	1.00E+00	HBEGF	2.12E-01	1.00E+00
LZTS2	-1.56E+00	2.63E-43	ARMCX3	-6.54E-01	1.00E+00	GNA11	-1.02E-01	1.00E+00
PKD1	-1.75E+00	2.69E-43	LDLRAD4	1.78E-01	1.00E+00	LETM2	1.68E-01	1.00E+00
TLK1	-1.33E+00	2.74E-43	SHQ1	-1.42E-01	1.00E+00	RNF19A	-1.35E-01	1.00E+00
IL4R	-3.58E+00	2.76E-43	FSCN1	2.97E-01	1.00E+00	PML	-1.01E-01	1.00E+00
IRS2	-3.16E+00	2.77E-43	CCDC102B	-1.56E-01	1.00E+00	LOC102399988	2.48E-01	1.00E+00
MTG1	-2.11E+00	3.07E-43	RPS2	-1.19E-01	1.00E+00	EXD2	1.82E-01	1.00E+00
BUB3	1.41E+00	3.53E-43	RNF20	1.02E-01	1.00E+00	LOC112581856	-2.63E-01	1.00E+00
RFTN2	-4.62E+00	3.64E-43	HDGFL1	-2.20E-01	1.00E+00	LOC112579717	2.51E-01	1.00E+00
INPP5K	-1.75E+00	3.70E-43	TDRD15	1.81E-01	1.00E+00	ELF2	-1.17E-01	1.00E+00
C21H3orf62	-2.35E+00	3.77E-43	AP2A2	8.70E-02	1.00E+00	ZBTB22	-1.34E-01	1.00E+00
LOC112586436	-2.50E+00	3.79E-43	LOC112584546	4.06E-01	1.00E+00	POFUT1	-1.26E-01	1.00E+00
LYSMD3	-2.29E+00	3.95E-43	ULK1	-2.56E-01	1.00E+00	SETD6	-1.15E-01	1.00E+00
TRIM56	-4.64E+00	4.18E-43	GLRX5	-2.68E-01	1.00E+00	LOC112582955	-1.48E-01	1.00E+00
LOC102391904	7.53E+00	4.23E-43	NCAM2	2.76E-01	1.00E+00	ITFG1	-1.18E-01	1.00E+00
CREBRF	-2.37E+00	4.27E-43	KRIT1	2.44E-01	1.00E+00	SLC35B1	1.11E-01	1.00E+00
SYTL3	6.63E+00	4.43E-43	SV2C	-1.36E-01	1.00E+00	TGFBI	3.60E-01	1.00E+00
ARSK	-4.08E+00	4.71E-43	LOC112585577	-4.40E-01	1.00E+00	UBE2J2	-1.03E-01	1.00E+00
TBRG1	1.58E+00	4.73E-43	DENND2C	1.72E-01	1.00E+00	BRIP1	-1.43E-01	1.00E+00
LOC112584007	-3.25E+00	4.78E-43	RMND5B	9.74E-02	1.00E+00	DHFR	-1.61E-01	1.00E+00
LOC102392879	-3.82E+00	4.86E-43	ADGRG1	1.51E-01	1.00E+00	YWHAE	-1.39E-01	1.00E+00
TAOK2	-1.64E+00	4.98E-43	HSF5	1.58E-01	1.00E+00	CSF1R	4.74E-01	1.00E+00
BTG3	-4.69E+00	5.01E-43	LOC112578809	-3.65E-01	1.00E+00	LOC102409925	4.45E-01	1.00E+00
FAN1	-1.40E+00	5.08E-43	XXYLT1	-8.56E-02	1.00E+00	KDR	-2.07E-01	1.00E+00

MRC2	-5.42E+00	5.26E-43	LTBR	-4.72E-01	1.00E+00	UTP15	1.56E-01	1.00E+00
NR2F2	-2.54E+00	5.33E-43	FUT8	3.00E-01	1.00E+00	LCLAT1	1.35E-01	1.00E+00
GGCX	-2.20E+00	5.41E-43	LOC112583857	1.58E-01	1.00E+00	BHLHE40	2.05E-01	1.00E+00
CADM4	-6.11E+00	5.44E-43	SLC6A1	-1.58E-01	1.00E+00	RAB5B	1.01E-01	1.00E+00
LOC112578843	4.97E+00	5.50E-43	FAM13B	1.14E-01	1.00E+00	RCOR1	-1.97E-01	1.00E+00
EPHX2	-2.38E+00	5.97E-43	RNF212B	-3.44E-01	1.00E+00	GNAI1	2.00E-01	1.00E+00
HEATR1	1.52E+00	6.12E-43	GPT2	1.16E-01	1.00E+00	F2	2.08E-01	1.00E+00
LOC102409319	-2.51E+00	6.12E-43	LOC112586936	-3.87E-01	1.00E+00	ZBTB18	-1.26E-01	1.00E+00
FASLG	5.92E+00	6.31E-43	LOC102390828	3.68E-01	1.00E+00	TPBGL	-2.43E-01	1.00E+00
ZSCAN20	2.02E+00	6.34E-43	DUSP22	-2.41E-01	1.00E+00	SPON2	3.01E-01	1.00E+00
DCAF15	1.56E+00	6.44E-43	ANTXR1	-5.45E-01	1.00E+00	LOC112578475	2.36E-01	1.00E+00
PGAM1	-1.70E+00	6.88E-43	AP2B1	8.89E-02	1.00E+00	LOC102410316	-1.49E-01	1.00E+00
SLC9B2	2.77E+00	7.05E-43	LOC102390517	-1.49E-01	1.00E+00	GRB7	2.15E-01	1.00E+00
HAUS2	1.75E+00	7.14E-43	GEMIN7	3.39E-01	1.00E+00	SBSN	2.56E-01	1.00E+00
KIAA0825	1.50E+00	7.47E-43	SUGCT	1.81E-01	1.00E+00	AJUBA	1.08E-01	1.00E+00
CCDC80	-7.21E+00	7.52E-43	ROPN1L	-5.00E-01	1.00E+00	CABCOCO1	1.15E-01	1.00E+00
ACOX3	-1.63E+00	7.75E-43	COPS6	-8.97E-02	1.00E+00	SDHAF2	-1.28E-01	1.00E+00
KCNK15	7.24E+00	7.96E-43	ABHD14A	-4.80E-01	1.00E+00	SLC24A3	2.15E-01	1.00E+00
LOC102411916	8.04E+00	8.06E-43	DHX30	1.17E-01	1.00E+00	AP3M2	2.25E-01	1.00E+00
SRP72	-1.24E+00	8.59E-43	POLK	5.19E-01	1.00E+00	ORAI3	1.28E-01	1.00E+00
RER1	-1.49E+00	8.91E-43	CCDC12	-1.69E-01	1.00E+00	ZNF484	1.37E-01	1.00E+00
CHCHD5	-2.25E+00	9.10E-43	SSR2	1.07E-01	1.00E+00	CBX4	-1.48E-01	1.00E+00
GPR158	6.29E+00	9.59E-43	NOC3L	-9.37E-02	1.00E+00	GMCL1	-1.24E-01	1.00E+00
SNX18	-2.14E+00	9.76E-43	BANP	8.42E-02	1.00E+00	DNASE1	-1.91E-01	1.00E+00
LOC112587806	5.79E+00	1.02E-42	POLR3C	-1.06E-01	1.00E+00	SLC35A2	-1.13E-01	1.00E+00
LOC102404020	2.95E+00	1.05E-42	RPL23A	-1.88E-01	1.00E+00	WDTC1	1.08E-01	1.00E+00
TLR3	-5.51E+00	1.06E-42	DDX18	-1.28E-01	1.00E+00	PLPBP	-1.06E-01	1.00E+00
RALGAPA2	-1.64E+00	1.07E-42	TNRC6A	1.48E-01	1.00E+00	ALKBH5	-1.02E-01	1.00E+00
LOC102412021	2.84E+00	1.10E-42	NACC2	1.02E-01	1.00E+00	ANKRD13B	1.13E-01	1.00E+00
FOXP1	-1.67E+00	1.11E-42	UNG	-8.10E-02	1.00E+00	INPPL1	-1.15E-01	1.00E+00
FAM120C	-1.67E+00	1.13E-42	ENTHD1	-3.33E-01	1.00E+00	HOXD4	1.35E-01	1.00E+00
VPS33B	-1.92E+00	1.15E-42	SCPEP1	-1.53E-01	1.00E+00	ASB13	1.92E-01	1.00E+00
HOGA1	3.83E+00	1.19E-42	ELOB	-1.43E-01	1.00E+00	POLR2M	-1.13E-01	1.00E+00
ZC3H6	-4.19E+00	1.21E-42	C22H18orf63	2.56E-01	1.00E+00	CPEB4	2.36E-01	1.00E+00

LOC112586203	5.76E+00	1.30E-42	ACAA2	1.63E-01	1.00E+00	STK17A	1.88E-01	1.00E+00
LOC112578408	5.21E+00	1.38E-42	LOC112582239	-1.67E-01	1.00E+00	WDR44	1.81E-01	1.00E+00
LOC112585535	-4.74E+00	1.39E-42	MARS2	4.56E-01	1.00E+00	MINDY4	-1.28E-01	1.00E+00
CTSF	-2.45E+00	1.39E-42	LOC102396469	4.57E-01	1.00E+00	CSNK2A2	-2.14E-01	1.00E+00
NUMA1	-1.68E+00	1.43E-42	GATA6	-5.54E-01	1.00E+00	NPHP4	-1.25E-01	1.00E+00
LOC112584965	5.78E+00	1.52E-42	DGAT2	-8.72E-02	1.00E+00	PARD6G	1.28E-01	1.00E+00
PCOLCE	-2.19E+00	1.52E-42	PSIP1	9.28E-02	1.00E+00	POLR3F	1.21E-01	1.00E+00
ZNF672	-3.39E+00	1.55E-42	LOC102392879	-4.53E-01	1.00E+00	AEBP2	-1.32E-01	1.00E+00
ALDOC	-3.12E+00	1.56E-42	GRPR	6.60E-01	1.00E+00	LOC102414521	2.84E-01	1.00E+00
LOC112580852	5.43E+00	1.62E-42	LOC102403559	8.35E-02	1.00E+00	TIGAR	1.27E-01	1.00E+00
MTMR3	2.77E+00	1.62E-42	TSACC	-4.22E-01	1.00E+00	CIB1	-1.58E-01	1.00E+00
FANCE	-2.91E+00	1.64E-42	METTL4	5.00E-01	1.00E+00	POLDIP3	-9.89E-02	1.00E+00
SPEN	-1.88E+00	1.67E-42	LOC102400966	-1.44E-01	1.00E+00	CSRNP1	3.22E-01	1.00E+00
ZNF213	-3.31E+00	1.74E-42	RTRAF	-1.05E-01	1.00E+00	LOC102394430	1.77E-01	1.00E+00
DPP9	1.50E+00	1.78E-42	LOC102409097	-2.30E-01	1.00E+00	ARL2BP	9.83E-02	1.00E+00
MS4A3	6.56E+00	1.82E-42	CDYL	8.74E-02	1.00E+00	LOC102393261	-1.55E-01	1.00E+00
RPL21	-3.26E+00	1.83E-42	ABCF3	1.26E-01	1.00E+00	DDX42	-1.02E-01	1.00E+00
PEX6	-1.48E+00	1.89E-42	TRPM3	1.05E-01	1.00E+00	LOC102402188	3.15E-01	1.00E+00
HMGB1	-2.05E+00	2.01E-42	YTHDF3	1.19E-01	1.00E+00	BCAR3	-1.10E-01	1.00E+00
AMACR	-2.80E+00	2.02E-42	LOC102412347	-1.22E-01	1.00E+00	DDX6	1.12E-01	1.00E+00
MGST1	-5.83E+00	2.04E-42	LOC112579901	-1.70E-01	1.00E+00	PIP4K2B	9.90E-02	1.00E+00
C9H5orf46	7.73E+00	2.04E-42	STRA6	4.85E-01	1.00E+00	PIK3IP1	1.32E-01	1.00E+00
MOCS1	1.79E+00	2.12E-42	ANXA11	8.70E-02	1.00E+00	AASS	-1.65E-01	1.00E+00
TMEM44	-4.47E+00	2.15E-42	RRP8	1.31E-01	1.00E+00	HTT	-1.04E-01	1.00E+00
NOL11	-1.72E+00	2.29E-42	SLC9A9	-2.00E-01	1.00E+00	LOC112579218	-3.11E-01	1.00E+00
MRPL23	-2.92E+00	2.29E-42	LOC112586971	5.48E-01	1.00E+00	PNPLA6	-1.35E-01	1.00E+00
ZNF410	-1.32E+00	2.43E-42	LOC102403795	-1.65E-01	1.00E+00	EIF4A3	-1.13E-01	1.00E+00
LOC112585187	-6.47E+00	2.60E-42	ANKRD12	1.94E-01	1.00E+00	CCDC91	-1.07E-01	1.00E+00
ACAD8	-1.93E+00	2.67E-42	IL10RA	3.91E-01	1.00E+00	XAB2	-1.20E-01	1.00E+00
SLC43A2	-1.55E+00	2.69E-42	ELMSAN1	-1.22E-01	1.00E+00	ISLR	-1.84E-01	1.00E+00
PPM1E	3.38E+00	2.82E-42	ZC3H18	1.02E-01	1.00E+00	SDHA	-1.30E-01	1.00E+00
PDLIM4	-5.15E+00	2.85E-42	CNPY3	1.67E-01	1.00E+00	RALY	1.35E-01	1.00E+00
MRS2	-1.77E+00	2.93E-42	PPM1K	-3.57E-01	1.00E+00	ACD	-1.42E-01	1.00E+00

MORN4	-1.82E+00	3.01E-42	KLHDC2	-8.50E-02	1.00E+00	BCAT2	-1.72E-01	1.00E+00
TMEM125	5.28E+00	3.15E-42	CCDC86	1.11E-01	1.00E+00	OTUD5	1.10E-01	1.00E+00
LOC102390160	-2.61E+00	3.20E-42	EDA	1.26E-01	1.00E+00	MEF2A	1.32E-01	1.00E+00
FREM1	4.63E+00	3.21E-42	XRCC2	-1.36E-01	1.00E+00	DFFB	-1.54E-01	1.00E+00
PRX	-5.94E+00	3.26E-42	LOC112577758	1.15E-01	1.00E+00	ANKRD37	2.58E-01	1.00E+00
TTC8	-2.57E+00	3.66E-42	NARF	7.84E-02	1.00E+00	CAVIN1	1.52E-01	1.00E+00
LOC102392066	2.08E+00	3.81E-42	NAA50	1.37E-01	1.00E+00	DENND6B	-1.35E-01	1.00E+00
TESK2	1.81E+00	3.85E-42	EFHC1	1.20E-01	1.00E+00	CLSTN1	-1.26E-01	1.00E+00
CFL2	1.38E+00	4.04E-42	BEND4	-1.41E-01	1.00E+00	GPANK1	1.46E-01	1.00E+00
TMEM220	-3.65E+00	4.07E-42	EFEMP1	-2.49E-01	1.00E+00	SGSH	-1.21E-01	1.00E+00
CANX	-1.72E+00	4.23E-42	RAB22A	-8.11E-02	1.00E+00	MSH4	2.49E-01	1.00E+00
MISP3	2.63E+00	4.53E-42	LOC102414397	2.63E-01	1.00E+00	TTBK1	2.00E-01	1.00E+00
LOC112583968	6.00E+00	4.56E-42	PIANP	-3.94E-01	1.00E+00	RTP3	2.37E-01	1.00E+00
MIGA1	-2.74E+00	5.00E-42	UROC1	-1.98E-01	1.00E+00	ADAM32	2.24E-01	1.00E+00
ABI3BP	4.19E+00	5.34E-42	PPCDC	-1.53E-01	1.00E+00	ZFP92	-2.69E-01	1.00E+00
RELN	-5.58E+00	5.36E-42	USP28	1.24E-01	1.00E+00	ZNF436	-1.30E-01	1.00E+00
ARHGAP10	-1.28E+00	5.46E-42	CFAP58	-1.41E-01	1.00E+00	TTC1	-1.09E-01	1.00E+00
LOC102394267	-5.35E+00	5.78E-42	PDCD11	1.22E-01	1.00E+00	STAT6	1.35E-01	1.00E+00
TIA1	-5.48E+00	5.87E-42	PIBF1	-2.08E-01	1.00E+00	HAUS7	1.39E-01	1.00E+00
KCMF1	1.21E+00	5.88E-42	ZNF674	-2.48E-01	1.00E+00	TMEM135	-1.19E-01	1.00E+00
LOC102398514	7.26E+00	5.92E-42	LOC112584700	-2.95E-01	1.00E+00	CLSTN2	1.30E-01	1.00E+00
LOC102390320	7.12E+00	5.92E-42	TMEM238	2.22E-01	1.00E+00	NME4	-1.50E-01	1.00E+00
LOC102409265	4.90E+00	6.00E-42	ZDHHC9	-3.47E-01	1.00E+00	SPINT2	-1.45E-01	1.00E+00
EGLN3	3.16E+00	6.03E-42	NFRKB	1.22E-01	1.00E+00	ATP6V1H	1.07E-01	1.00E+00
IQSEC2	1.61E+00	6.07E-42	CDK1	-8.18E-02	1.00E+00	CDK16	-1.04E-01	1.00E+00
FSTL3	-3.49E+00	6.19E-42	NEDD8	-1.11E-01	1.00E+00	DVL1	1.03E-01	1.00E+00
LOC102394398	-2.87E+00	6.53E-42	RFC3	-9.28E-02	1.00E+00	CERCAM	-1.27E-01	1.00E+00
PRTFDC1	-4.15E+00	6.79E-42	CEP170	3.30E-01	1.00E+00	DEUP1	2.77E-01	1.00E+00
UBA2	-1.23E+00	6.90E-42	LOC102413401	-2.08E-01	1.00E+00	SERINC1	1.11E-01	1.00E+00
LOC102407015	3.82E+00	7.02E-42	DPY19L3	-1.88E-01	1.00E+00	TEX30	-1.51E-01	1.00E+00
CRBN	1.24E+00	7.04E-42	CDKN3	-2.57E-01	1.00E+00	LOC102409360	-1.18E-01	1.00E+00
SMAD7	-4.13E+00	7.04E-42	KCTD20	-1.62E-01	1.00E+00	LOC112587329	-2.54E-01	1.00E+00
SNX8	-1.62E+00	7.08E-42	BOLA1	-1.67E-01	1.00E+00	CSDC2	1.19E-01	1.00E+00

LOC102409389	9.52E+00	7.13E-42	ATP1A3	3.68E-01	1.00E+00	STC2	2.74E-01	1.00E+00
ZNF821	-1.87E+00	7.14E-42	KIAA0513	1.37E-01	1.00E+00	TMEM176B	-1.40E-01	1.00E+00
IGFBP4	-5.79E+00	7.20E-42	LPCAT4	1.75E-01	1.00E+00	LGI4	-2.18E-01	1.00E+00
CPT1B	-3.05E+00	7.42E-42	DMRT2	-1.28E-01	1.00E+00	WDR92	-1.21E-01	1.00E+00
SLC1A6	-7.09E+00	7.62E-42	LOC102393672	-9.94E-02	1.00E+00	RBM3	-1.24E-01	1.00E+00
DCHS2	1.85E+00	7.66E-42	TNFSF8	5.69E-01	1.00E+00	MED4	-9.51E-02	1.00E+00
ELMO3	-3.79E+00	7.68E-42	LIG1	1.15E-01	1.00E+00	LOC112583635	-2.82E-01	1.00E+00
LOC102416331	1.18E+00	7.69E-42	UBIAD1	-1.87E-01	1.00E+00	HP1BP3	-1.15E-01	1.00E+00
CHTF8	-1.46E+00	7.72E-42	ATP6V1F	-1.14E-01	1.00E+00	MRPL47	-1.42E-01	1.00E+00
DACT1	-3.73E+00	7.76E-42	GABBR2	-2.06E-01	1.00E+00	GALNT7	-1.46E-01	1.00E+00
FAM118A	-2.52E+00	8.11E-42	EXOSC3	-1.43E-01	1.00E+00	SCAF8	-9.76E-02	1.00E+00
PDLIM3	-4.61E+00	8.21E-42	PRMT7	-1.13E-01	1.00E+00	EIF4ENIF1	-1.09E-01	1.00E+00
NUP107	1.25E+00	8.30E-42	CRADD	-1.60E-01	1.00E+00	NMT2	-1.09E-01	1.00E+00
LOC102388954	-4.00E+00	8.38E-42	PYGM	3.40E-01	1.00E+00	LOC112579189	-2.57E-01	1.00E+00
PANX3	5.30E+00	8.49E-42	AFF2	-3.82E-01	1.00E+00	RAB15	-1.47E-01	1.00E+00
LOC102389337	-6.91E+00	8.84E-42	TBC1D8B	-1.76E-01	1.00E+00	ND5	1.71E-01	1.00E+00
CASC3	1.33E+00	9.16E-42	RGS21	-3.88E-01	1.00E+00	SH3RF1	1.46E-01	1.00E+00
MOB1A	-2.80E+00	9.44E-42	SELENOT	1.12E-01	1.00E+00	TRMT11	-1.23E-01	1.00E+00
UBL4A	-2.77E+00	9.93E-42	CREB1	1.46E-01	1.00E+00	LYPD5	-2.26E-01	1.00E+00
MOSPD2	1.68E+00	1.04E-41	PLSCR3	-1.32E-01	1.00E+00	CEP19	-2.37E-01	1.00E+00
MAP9	-2.82E+00	1.06E-41	CMTM4	1.13E-01	1.00E+00	LOC112586898	2.11E-01	1.00E+00
LOC102393808	-3.34E+00	1.13E-41	UBR7	8.77E-02	1.00E+00	SEC31B	-1.22E-01	1.00E+00
CIDEA	4.43E+00	1.13E-41	FBXO7	9.35E-02	1.00E+00	SGO2	-1.32E-01	1.00E+00
NDOR1	-1.69E+00	1.15E-41	HAL	1.16E-01	1.00E+00	DEPDC7	-2.26E-01	1.00E+00
ADGRL2	-1.85E+00	1.22E-41	NR2C1	1.23E-01	1.00E+00	MSANTD2	-1.21E-01	1.00E+00
USP7	1.52E+00	1.23E-41	MEAF6	-1.71E-01	1.00E+00	MFSD3	-1.71E-01	1.00E+00
TMTC2	1.94E+00	1.26E-41	TAF6L	1.80E-01	1.00E+00	SPAG17	2.59E-01	1.00E+00
FKBP7	-6.82E+00	1.27E-41	STRADA	8.54E-02	1.00E+00	IGSF8	1.08E-01	1.00E+00
PGRMC1	-1.34E+00	1.27E-41	TJP1	1.29E-01	1.00E+00	HSD17B1	-2.53E-01	1.00E+00
LOC112583894	4.52E+00	1.28E-41	ZNF212	9.01E-02	1.00E+00	MICU2	-1.09E-01	1.00E+00
DBNDD1	-2.03E+00	1.30E-41	RPL3	-9.30E-02	1.00E+00	NDST1	1.16E-01	1.00E+00
LOC102395519	-4.94E+00	1.32E-41	TLE2	3.22E-01	1.00E+00	SLC43A2	1.19E-01	1.00E+00
EDIL3	-2.52E+00	1.34E-41	IL17RA	-3.88E-01	1.00E+00	PODXL	1.73E-01	1.00E+00

MIF	-3.03E+00	1.36E-41	KIF7	1.68E-01	1.00E+00	LOC102404545	4.09E-01	1.00E+00
APOA1	-7.45E+00	1.37E-41	PITPNM1	-2.94E-01	1.00E+00	CCT6B	-1.63E-01	1.00E+00
LOC112577683	-4.97E+00	1.41E-41	GPKOW	-1.18E-01	1.00E+00	INTS2	-1.58E-01	1.00E+00
LOC102416276	-5.22E+00	1.42E-41	DNAJA1	-7.97E-02	1.00E+00	CD99L2	-9.68E-02	1.00E+00
ZDHHC21	-2.40E+00	1.43E-41	SLC35A2	1.26E-01	1.00E+00	LOC102411351	-2.34E-01	1.00E+00
FAM186B	3.18E+00	1.44E-41	LOC102394527	1.57E-01	1.00E+00	ALDH18A1	-1.02E-01	1.00E+00
LAMTOR5	-2.30E+00	1.44E-41	SLC7A1	2.75E-01	1.00E+00	DCDC2B	-2.63E-01	1.00E+00
LOC112585546	6.91E+00	1.48E-41	SMOC2	-4.84E-01	1.00E+00	HEATR3	1.14E-01	1.00E+00
GPR180	-3.23E+00	1.48E-41	STYX	-2.38E-01	1.00E+00	ATP5F1D	1.39E-01	1.00E+00
C17H12orf76	1.86E+00	1.54E-41	ASCC1	8.98E-02	1.00E+00	CALU	-1.34E-01	1.00E+00
LOC112587959	6.68E+00	1.55E-41	LNX1	-4.06E-01	1.00E+00	DIS3L2	-1.12E-01	1.00E+00
PFKL	-5.26E+00	1.57E-41	SDHAF3	1.85E-01	1.00E+00	TULP4	1.41E-01	1.00E+00
WDR55	-1.89E+00	1.60E-41	FDX1	-1.01E-01	1.00E+00	SORT1	-1.05E-01	1.00E+00
ADSSL1	-3.33E+00	1.61E-41	LOC102398019	-3.38E-01	1.00E+00	RHOQ	-1.75E-01	1.00E+00
FAM117B	1.61E+00	1.68E-41	TRAK1	1.33E-01	1.00E+00	GPR45	2.45E-01	1.00E+00
SEMA4G	-2.65E+00	1.73E-41	FTCDNL1	-3.14E-01	1.00E+00	HELB	1.74E-01	1.00E+00
LRSAM1	1.69E+00	1.81E-41	ZNF830	-1.02E-01	1.00E+00	TRIB1	2.41E-01	1.00E+00
RAB17	-5.95E+00	1.86E-41	SSSCA1	-1.14E-01	1.00E+00	SLIT1	2.27E-01	1.00E+00
ABHD1	2.14E+00	1.92E-41	CCDC85B	-2.81E-01	1.00E+00	ZHX3	1.61E-01	1.00E+00
LOC112579955	-5.77E+00	1.96E-41	TCF7L1	2.25E-01	1.00E+00	TOLLIP	1.02E-01	1.00E+00
LOC102405154	-6.95E+00	1.98E-41	LACTB2	-1.57E-01	1.00E+00	ETV3	-1.80E-01	1.00E+00
DDX6	-1.62E+00	1.98E-41	MARCH3	2.12E-01	1.00E+00	CCDC174	-1.19E-01	1.00E+00
FZD7	-4.75E+00	2.07E-41	MRGPRF	5.70E-01	1.00E+00	CAMLG	-1.15E-01	1.00E+00
TYW3	-5.97E+00	2.07E-41	BICDL2	1.77E-01	1.00E+00	VAV1	5.07E-01	1.00E+00
CABCOCO1	-2.89E+00	2.18E-41	IQCK	-3.41E-01	1.00E+00	LRP3	-1.54E-01	1.00E+00
TCIRG1	-2.82E+00	2.32E-41	ADGRG3	5.52E-01	1.00E+00	LOC102406326	2.47E-01	1.00E+00
LOC102394043	6.65E+00	2.33E-41	RNF152	-3.95E-01	1.00E+00	RAD51D	-1.37E-01	1.00E+00
EMILIN1	-2.36E+00	2.34E-41	LOC102410383	-4.40E-01	1.00E+00	EXOSC2	-1.12E-01	1.00E+00
KMT5C	2.07E+00	2.50E-41	YAP1	-1.66E-01	1.00E+00	CLK1	-1.14E-01	1.00E+00
ARMC9	-1.99E+00	2.51E-41	LOC112577656	5.02E-01	1.00E+00	NUMA1	-1.12E-01	1.00E+00
EPN1	-1.66E+00	2.51E-41	PNKD	-1.82E-01	1.00E+00	SLC1A7	2.43E-01	1.00E+00
ACVR2B	-2.74E+00	2.53E-41	CPSF4	-8.63E-02	1.00E+00	R3HCC1	1.95E-01	1.00E+00
TDRD9	5.31E+00	2.62E-41	TIA1	-5.05E-	1.00E+00	ISCA1	1.02E-01	1.00E+00

				01				
UVRAG	1.35E+00	2.64E-41	MICALL1	8.17E-02	1.00E+00	SCUBE3	-2.12E-01	1.00E+00
LOC112586550	5.70E+00	2.66E-41	PLK3	9.24E-02	1.00E+00	TSGA10IP	-2.81E-01	1.00E+00
AMOTL1	1.67E+00	2.69E-41	GRM4	-2.91E-01	1.00E+00	SLC38A9	1.27E-01	1.00E+00
LOC102407763	-2.98E+00	2.79E-41	ITCH	1.31E-01	1.00E+00	ACSBG1	-1.59E-01	1.00E+00
LOC102392841	2.07E+00	2.83E-41	CASTOR2	-8.14E-02	1.00E+00	LOC112580198	-2.60E-01	1.00E+00
GPN2	1.39E+00	2.83E-41	SREBF1	-2.08E-01	1.00E+00	CDKN1A	2.31E-01	1.00E+00
MGMT	2.36E+00	2.93E-41	LOC112584673	3.26E-01	1.00E+00	SERINC5	-1.36E-01	1.00E+00
GRB14	-6.06E+00	2.94E-41	SELENOF	-9.23E-02	1.00E+00	UBR5	-1.16E-01	1.00E+00
ARL4D	4.60E+00	2.94E-41	DNMBP	1.28E-01	1.00E+00	CC2D1A	-1.09E-01	1.00E+00
DNAAF3	-2.90E+00	3.02E-41	IQGAP2	1.38E-01	1.00E+00	BOLA2B	1.50E-01	1.00E+00
PRKD1	-1.78E+00	3.06E-41	NPRL3	1.21E-01	1.00E+00	NCF4	3.09E-01	1.00E+00
LOC102394430	-4.66E+00	3.06E-41	GLIPR2	-2.92E-01	1.00E+00	SULF1	1.85E-01	1.00E+00
SLC13A3	-4.32E+00	3.06E-41	MTUS1	8.53E-02	1.00E+00	LTBP2	3.26E-01	1.00E+00
RNF166	1.49E+00	3.10E-41	UBR4	1.35E-01	1.00E+00	PWP2	1.09E-01	1.00E+00
CTU2	-2.46E+00	3.10E-41	LRWD1	1.53E-01	1.00E+00	CLBA1	1.48E-01	1.00E+00
SPRYD3	-1.30E+00	3.40E-41	IFNGR2	-2.91E-01	1.00E+00	SLC9A3R2	-1.97E-01	1.00E+00
METAP1D	-2.53E+00	3.54E-41	ARID5B	8.89E-02	1.00E+00	MIA3	-1.12E-01	1.00E+00
SOCS3	-5.50E+00	3.64E-41	C16H11orf96	-2.28E-01	1.00E+00	TNFAIP1	1.15E-01	1.00E+00
AP3B2	1.62E+00	3.74E-41	LOC102411048	3.20E-01	1.00E+00	IGF2R	-1.60E-01	1.00E+00
INTS5	1.54E+00	3.95E-41	FABP3	1.75E-01	1.00E+00	HK1	1.16E-01	1.00E+00
GIN1	-2.69E+00	3.98E-41	SAR1B	1.41E-01	1.00E+00	ENG	-2.69E-01	1.00E+00
C19H5orf58	2.62E+00	4.10E-41	TAF6	1.16E-01	1.00E+00	LGALS4	-2.24E-01	1.00E+00
IDH3G	-1.93E+00	4.16E-41	RAB2B	-1.00E-01	1.00E+00	ARHGEF28	-1.21E-01	1.00E+00
S1PR1	-6.22E+00	4.37E-41	MAN1C1	-1.07E-01	1.00E+00	TBC1D9B	-9.48E-02	1.00E+00
GSPT2	-3.04E+00	4.39E-41	KLF3	-2.46E-01	1.00E+00	MUT	-9.87E-02	1.00E+00
LOC102394491	-6.03E+00	4.41E-41	NPHP1	-2.40E-01	1.00E+00	SSX2IP	1.37E-01	1.00E+00
PPM1L	2.70E+00	4.53E-41	MGAT4A	9.19E-02	1.00E+00	BAG5	1.68E-01	1.00E+00
PRPF40B	-1.59E+00	4.58E-41	MRPL37	9.44E-02	1.00E+00	SCARB2	-1.21E-01	1.00E+00
MAP7	-2.45E+00	4.61E-41	FAM208A	1.53E-01	1.00E+00	SMAD2	-1.25E-01	1.00E+00
LOC102398394	-7.14E+00	4.89E-41	LOC102391011	1.39E-01	1.00E+00	TCTE3	-2.38E-01	1.00E+00
LOC102408974	4.79E+00	5.15E-41	SAMM50	-1.03E-01	1.00E+00	TLN1	1.24E-01	1.00E+00
TTC33	-3.54E+00	5.22E-41	C2H2orf88	5.03E-01	1.00E+00	RRAGC	1.17E-01	1.00E+00
HERC4	-	5.23E-41	LOC112580918	2.06E-01	1.00E+00	CRISPLD1	-2.82E-	1.00E+00

	1.32E+00						01	
TMEM171	4.53E+00	5.41E-41	RDH5	-3.65E-01	1.00E+00	SYCE1L	-2.04E-01	1.00E+00
KIAA2013	-1.74E+00	5.87E-41	PCDH10	7.33E-01	1.00E+00	KAZALD1	-2.16E-01	1.00E+00
TBATA	-7.39E+00	5.93E-41	MID1	-9.57E-02	1.00E+00	NUDT13	2.57E-01	1.00E+00
LOC112578002	3.45E+00	5.95E-41	LRRIQ1	-3.23E-01	1.00E+00	PRR13	1.15E-01	1.00E+00
PCDH11X	-4.87E+00	5.98E-41	PRKCG	2.46E-01	1.00E+00	ALKBH8	1.43E-01	1.00E+00
PDE4D	3.32E+00	6.11E-41	LOC112582189	-2.39E-01	1.00E+00	LOC102414909	4.62E-01	1.00E+00
EIF4E	1.38E+00	6.13E-41	CCNI	7.95E-02	1.00E+00	VEZT	-1.03E-01	1.00E+00
MOCS2	-1.44E+00	6.15E-41	CCDC61	-1.13E-01	1.00E+00	LOC102414448	-1.24E-01	1.00E+00
FKBP1A	-1.45E+00	6.22E-41	LOC102397166	-1.36E-01	1.00E+00	ANKRD23	-1.25E-01	1.00E+00
LOC102390265	1.83E+00	6.32E-41	ACOX3	1.63E-01	1.00E+00	RPP38	1.30E-01	1.00E+00
CLCN6	-2.20E+00	6.39E-41	ARFGEF1	1.11E-01	1.00E+00	LOC102396564	1.42E-01	1.00E+00
GTF2H1	1.14E+00	6.68E-41	ZNF668	1.22E-01	1.00E+00	TAF4B	-1.56E-01	1.00E+00
COL4A5	-2.91E+00	6.96E-41	NYNRIN	-7.21E-01	1.00E+00	TMEM156	-1.44E-01	1.00E+00
ARNT2	-3.10E+00	7.03E-41	TUSC3	1.15E-01	1.00E+00	DCAF8	-1.01E-01	1.00E+00
C5H11orf68	-2.70E+00	7.21E-41	DNPH1	-1.30E-01	1.00E+00	DNMT3A	-1.08E-01	1.00E+00
C7H4orf36	3.60E+00	7.23E-41	DDX56	9.89E-02	1.00E+00	ZNF664	1.07E-01	1.00E+00
SARNP	-2.67E+00	7.25E-41	GLA	2.27E-01	1.00E+00	EPM2AIP1	-1.23E-01	1.00E+00
MAPK1	1.47E+00	7.33E-41	MSH2	-9.93E-02	1.00E+00	PTPRM	-1.04E-01	1.00E+00
MZT2B	-3.59E+00	7.62E-41	PRR15L	-5.71E-01	1.00E+00	MCOLN1	-1.05E-01	1.00E+00
ACTN1	2.42E+00	7.93E-41	ARID3B	9.31E-02	1.00E+00	MEIS2	1.43E-01	1.00E+00
TPPP	3.80E+00	8.03E-41	DIP2C	-2.00E-01	1.00E+00	SYT17	-1.28E-01	1.00E+00
HSD17B14	-5.09E+00	8.66E-41	GPM6B	-1.23E-01	1.00E+00	LOC102391441	1.47E-01	1.00E+00
NR5A2	-6.56E+00	8.78E-41	RIC1	1.20E-01	1.00E+00	CRTC2	-9.74E-02	1.00E+00
KIF27	-2.43E+00	8.96E-41	CACNG4	5.06E-01	1.00E+00	FMC1	1.44E-01	1.00E+00
PER2	3.65E+00	9.39E-41	BMP7	2.41E-01	1.00E+00	LOC102391376	1.32E-01	1.00E+00
IMPDH2	-1.97E+00	9.56E-41	ZNHIT2	-1.16E-01	1.00E+00	CFAP99	-2.31E-01	1.00E+00
RARRES2	-6.67E+00	9.57E-41	SMARCAD1	1.07E-01	1.00E+00	TET2	-1.39E-01	1.00E+00
ISPD	1.70E+00	9.65E-41	LOC102392508	-4.40E-01	1.00E+00	SAMD13	-2.25E-01	1.00E+00
ERMARD	-1.63E+00	9.74E-41	NAP1L1	1.08E-01	1.00E+00	ARSG	1.35E-01	1.00E+00
gen-01	-2.82E+00	9.79E-41	THRB	-3.15E-01	1.00E+00	WNK3	1.91E-01	1.00E+00
LOC102390560	-3.35E+00	1.01E-40	TERF2IP	-1.13E-01	1.00E+00	TSEN34	-1.29E-01	1.00E+00
KMT2B	1.70E+00	1.03E-40	CLCN2	-2.39E-01	1.00E+00	MAP10	-2.36E-01	1.00E+00
CENPBD1	2.14E+00	1.05E-40	KCTD17	1.59E-01	1.00E+00	ZNF554	2.43E-01	1.00E+00

LOC102396276	1.72E+00	1.08E-40	SLC39A5	-4.47E-01	1.00E+00	ST6GALNAC3	1.81E-01	1.00E+00
CMTM6	-1.57E+00	1.09E-40	KDM2A	1.24E-01	1.00E+00	SHANK1	-1.71E-01	1.00E+00
POGK	-1.55E+00	1.10E-40	CCDC40	-1.97E-01	1.00E+00	DCP2	-1.06E-01	1.00E+00
SMCHD1	-2.05E+00	1.10E-40	PPARD	8.99E-02	1.00E+00	C4H1orf198	1.21E-01	1.00E+00
MRPL18	1.77E+00	1.13E-40	LOC102389632	-3.13E-01	1.00E+00	SH3GLB1	1.08E-01	1.00E+00
EPS15	1.76E+00	1.15E-40	DHX33	2.79E-01	1.00E+00	SLC14A1	2.53E-01	1.00E+00
ZFP37	-3.76E+00	1.15E-40	ATP8	-3.16E-01	1.00E+00	PTDSS1	-9.86E-02	1.00E+00
ENO2	-6.58E+00	1.21E-40	KLF9	-5.66E-01	1.00E+00	CCDC121	-2.15E-01	1.00E+00
NTNG1	-5.63E+00	1.21E-40	DNM1	1.93E-01	1.00E+00	DDIAS	-1.43E-01	1.00E+00
CCNG1	1.27E+00	1.21E-40	LOC102391189	-9.00E-02	1.00E+00	LOC112585085	-2.25E-01	1.00E+00
GADD45G	-7.28E+00	1.23E-40	UBE2D1	1.63E-01	1.00E+00	FGD6	2.75E-01	1.00E+00
C17H12orf43	1.59E+00	1.25E-40	CSDE1	9.41E-02	1.00E+00	PRR14	-1.10E-01	1.00E+00
MOB3A	1.38E+00	1.26E-40	VEGFA	6.10E-01	1.00E+00	LOC102411827	-1.27E-01	1.00E+00
EMX2	-6.14E+00	1.28E-40	NCOR2	2.40E-01	1.00E+00	SS18	-1.06E-01	1.00E+00
HMGCL	-2.74E+00	1.37E-40	A1CF	-1.01E-01	1.00E+00	OSTM1	1.34E-01	1.00E+00
HERPUD1	-4.50E+00	1.39E-40	SAP30BP	8.12E-02	1.00E+00	SMU1	9.52E-02	1.00E+00
FKBP15	1.17E+00	1.42E-40	C6H1orf50	-1.13E-01	1.00E+00	RSU1	9.83E-02	1.00E+00
TMED7	-1.39E+00	1.45E-40	PLP2	1.78E-01	1.00E+00	ARL15	1.64E-01	1.00E+00
DLGAP3	2.69E+00	1.51E-40	P4HTM	-2.74E-01	1.00E+00	STK35	-1.02E-01	1.00E+00
NXT2	1.99E+00	1.52E-40	CELF1	9.40E-02	1.00E+00	PLA2G2C	2.41E-01	1.00E+00
MIPEP	-1.38E+00	1.54E-40	ZBTB17	1.32E-01	1.00E+00	C15H8orf88	-1.61E-01	1.00E+00
APPL2	1.30E+00	1.57E-40	PFDN5	-1.38E-01	1.00E+00	LOC102391229	1.35E-01	1.00E+00
SCD5	-4.22E+00	1.58E-40	RNF146	-7.88E-02	1.00E+00	RPS6KC1	-9.46E-02	1.00E+00
DHRS7	-4.61E+00	1.60E-40	DENR	-2.37E-01	1.00E+00	LOC102416007	-2.17E-01	1.00E+00
NBL1	-5.71E+00	1.71E-40	CNRIP1	9.23E-02	1.00E+00	IFIH1	1.76E-01	1.00E+00
KCNK7	3.72E+00	1.74E-40	LOC102391973	2.09E-01	1.00E+00	LOC102394190	1.26E-01	1.00E+00
ZBTB1	-4.72E+00	1.76E-40	NT5C3B	-1.66E-01	1.00E+00	SNRNP35	1.70E-01	1.00E+00
HHIP	3.73E+00	1.77E-40	FAM174B	-2.58E-01	1.00E+00	MAPRE2	-1.25E-01	1.00E+00
JSRP1	-7.22E+00	1.84E-40	DHX35	9.94E-02	1.00E+00	DPY19L2	2.92E-01	1.00E+00
ERBIN	-2.43E+00	1.95E-40	MYH7B	1.12E-01	1.00E+00	MED7	1.20E-01	1.00E+00
TFRC	-1.72E+00	1.96E-40	HMCN1	-1.06E-01	1.00E+00	MYOZ1	-1.99E-01	1.00E+00
CAV1	-7.48E+00	1.96E-40	ATL3	1.22E-01	1.00E+00	SLC2A1	1.69E-01	1.00E+00
SLC7A2	1.55E+00	2.00E-40	CIR1	9.49E-02	1.00E+00	SAMD8	1.36E-01	1.00E+00
RERGL	7.39E+00	2.03E-40	ZNF143	8.58E-02	1.00E+00	R3HCC1L	1.14E-01	1.00E+00

DPY19L4	-1.96E+00	2.04E-40	CDC37	8.49E-02	1.00E+00	ANKRD34B	2.51E-01	1.00E+00
IRF8	6.68E+00	2.11E-40	LOC112581642	-3.55E-01	1.00E+00	USP49	-2.19E-01	1.00E+00
TCP11L2	1.68E+00	2.15E-40	THEM6	-1.36E-01	1.00E+00	FEZ2	-1.07E-01	1.00E+00
LOC102395425	4.48E+00	2.15E-40	LOC102392861	-2.02E-01	1.00E+00	RPE	-1.20E-01	1.00E+00
CCDC71	-2.91E+00	2.20E-40	DNAJB1	-9.17E-02	1.00E+00	ERN2	3.02E-01	1.00E+00
ARHGEF6	-5.35E+00	2.20E-40	LOC102397898	-1.08E-01	1.00E+00	MZT2B	1.97E-01	1.00E+00
LOC112587814	-2.36E+00	2.33E-40	TOMM7	-1.31E-01	1.00E+00	SUGP2	-1.20E-01	1.00E+00
LOC102395271	-6.46E+00	2.41E-40	ZBTB44	1.56E-01	1.00E+00	ALDOC	-1.48E-01	1.00E+00
LOC102398033	-4.64E+00	2.47E-40	LOC112586805	2.22E-01	1.00E+00	LAMC3	1.99E-01	1.00E+00
C12H2orf42	1.71E+00	2.48E-40	LOC102405334	2.87E-01	1.00E+00	PPP2R3B	-1.36E-01	1.00E+00
EXOSC6	-2.52E+00	2.50E-40	TWSG1	3.34E-01	1.00E+00	LOC102398945	-2.42E-01	1.00E+00
SV2A	-3.48E+00	2.54E-40	LOC102416262	-2.77E-01	1.00E+00	OAZ2	1.01E-01	1.00E+00
ARHGEF1	-1.63E+00	2.61E-40	INPP1	-1.66E-01	1.00E+00	SAFB2	-9.82E-02	1.00E+00
MARCKS	-6.50E+00	2.90E-40	LOC112583705	-4.70E-01	1.00E+00	TUBA4A	-2.92E-01	1.00E+00
LOC102405126	2.11E+00	3.00E-40	TRMT10A	1.23E-01	1.00E+00	PHKA2	1.23E-01	1.00E+00
TRIM14	-3.70E+00	3.00E-40	SALL2	2.60E-01	1.00E+00	GNA13	1.24E-01	1.00E+00
GDF11	-5.05E+00	3.03E-40	LOC102406753	1.76E-01	1.00E+00	DMAC2	1.12E-01	1.00E+00
TAL2	3.07E+00	3.03E-40	ND5	-1.89E-01	1.00E+00	UXS1	-9.90E-02	1.00E+00
RSRC2	-1.19E+00	3.14E-40	SQOR	8.26E-02	1.00E+00	NDUFAF4	1.41E-01	1.00E+00
CAND1	1.38E+00	3.22E-40	RAB30	9.05E-02	1.00E+00	LOC112587746	1.46E-01	1.00E+00
CDR2	1.82E+00	3.35E-40	PDIA3	8.88E-02	1.00E+00	ATF7IP	-1.52E-01	1.00E+00
FAM19A2	6.62E+00	3.37E-40	COL8A1	3.67E-01	1.00E+00	REC8	-2.55E-01	1.00E+00
ISYNA1	-3.18E+00	3.42E-40	DDX19A	9.32E-02	1.00E+00	NUBP2	1.53E-01	1.00E+00
S100A11	-6.75E+00	3.44E-40	ZNF34	-9.78E-02	1.00E+00	ATP5S	-1.40E-01	1.00E+00
RBM24	-4.14E+00	3.51E-40	AGTRAP	1.41E-01	1.00E+00	KIF17	-1.78E-01	1.00E+00
LOC112583745	-5.62E+00	3.52E-40	PISD	-8.10E-02	1.00E+00	ZNF503	1.16E-01	1.00E+00
ZC3HAV1L	-4.92E+00	3.52E-40	C20H14orf28	5.24E-01	1.00E+00	PANK2	-9.77E-02	1.00E+00
LOC102395010	-5.85E+00	3.60E-40	TSKU	1.01E-01	1.00E+00	STN1	1.25E-01	1.00E+00
HIGD2A	1.91E+00	3.61E-40	LOC102395153	1.27E-01	1.00E+00	RPL12	1.90E-01	1.00E+00
CFAP46	-2.86E+00	3.65E-40	TRIM15	-4.07E-01	1.00E+00	PLS1	2.81E-01	1.00E+00
LOC102407934	-6.76E+00	3.67E-40	LOC102411662	-1.17E-01	1.00E+00	POLR2I	1.55E-01	1.00E+00
KANK1	-2.33E+00	4.00E-40	SGCD	7.86E-02	1.00E+00	ALS2CR12	-1.79E-01	1.00E+00
FUCA2	-3.25E+00	4.04E-40	ALG12	-1.28E-01	1.00E+00	HGSNAT	-9.35E-02	1.00E+00

DUSP14	2.15E+00	4.11E-40	ALKBH5	1.06E-01	1.00E+00	L1CAM	-4.37E-01	1.00E+00
NLK	1.70E+00	4.19E-40	ABI3BP	1.35E-01	1.00E+00	WWP1	-1.01E-01	1.00E+00
SLC27A6	-4.68E+00	4.19E-40	ODF4	1.13E-01	1.00E+00	RIBC1	-1.73E-01	1.00E+00
RSRC1	-1.49E+00	4.24E-40	PET117	2.08E-01	1.00E+00	LOC102389658	1.76E-01	1.00E+00
ITGB5	-1.76E+00	4.30E-40	DDOST	9.90E-02	1.00E+00	CLIC4	1.14E-01	1.00E+00
LOC112583851	4.01E+00	4.32E-40	LOC112580127	2.58E-01	1.00E+00	CD48	-5.07E-01	1.00E+00
ARTN	-5.05E+00	4.37E-40	BRSK1	-1.70E-01	1.00E+00	ZNF146	1.24E-01	1.00E+00
HORMAD1	-3.51E+00	4.44E-40	EMP1	4.09E-01	1.00E+00	ACSL4	1.22E-01	1.00E+00
CDC123	1.37E+00	4.66E-40	PERP	4.22E-01	1.00E+00	IL10RB	-1.13E-01	1.00E+00
NUDT19	-1.76E+00	4.66E-40	TXNDC16	1.53E-01	1.00E+00	ARID1A	-1.34E-01	1.00E+00
USHBP1	-3.50E+00	4.90E-40	GSN	-2.96E-01	1.00E+00	PIGH	-1.53E-01	1.00E+00
KIRREL1	-6.54E+00	4.94E-40	PATL2	8.95E-02	1.00E+00	RGS11	2.24E-01	1.00E+00
SORD	-4.89E+00	5.19E-40	YIPF2	-1.55E-01	1.00E+00	UBE2O	9.82E-02	1.00E+00
TNFSF18	4.02E+00	5.78E-40	CDK3	7.66E-01	1.00E+00	RRNAD1	-1.14E-01	1.00E+00
GATA6	-6.10E+00	5.89E-40	PYROXD1	-2.04E-01	1.00E+00	LOC102409452	-1.16E-01	1.00E+00
LHFPL5	5.87E+00	5.91E-40	IFNL3	3.00E-01	1.00E+00	PLEKHM1	1.19E-01	1.00E+00
HNRNPUL1	-1.94E+00	6.04E-40	ARL13B	1.18E-01	1.00E+00	LOC112585077	-2.70E-01	1.00E+00
TRERF1	-2.50E+00	6.21E-40	CMSS1	-6.44E-01	1.00E+00	IQCN	1.99E-01	1.00E+00
SUDS3	1.27E+00	6.24E-40	TPX2	8.62E-02	1.00E+00	AP4S1	1.31E-01	1.00E+00
PYURF	1.95E+00	6.29E-40	APOBR	-3.85E-01	1.00E+00	CRYAB	-2.38E-01	1.00E+00
GJB2	6.06E+00	6.52E-40	ABHD1	1.65E-01	1.00E+00	LOC112584706	2.36E-01	1.00E+00
FBXO33	2.07E+00	6.73E-40	MAPKBP1	1.29E-01	1.00E+00	OBSL1	-1.94E-01	1.00E+00
TAB1	1.31E+00	7.01E-40	SYT3	4.72E-01	1.00E+00	KALRN	1.68E-01	1.00E+00
EMSY	-3.77E+00	7.18E-40	DENND2A	1.12E-01	1.00E+00	GABBR2	2.69E-01	1.00E+00
MBD3	-1.80E+00	7.23E-40	ALDOC	2.38E-01	1.00E+00	TOM1	1.05E-01	1.00E+00
TTC25	-3.75E+00	7.87E-40	MSI2	-1.56E-01	1.00E+00	TSPAN3	-1.08E-01	1.00E+00
ZDHHC14	-1.77E+00	8.00E-40	STARD8	2.81E-01	1.00E+00	ALS2	-1.22E-01	1.00E+00
LOC112585767	-5.69E+00	8.15E-40	SUMO3	-1.26E-01	1.00E+00	PCSK7	9.75E-02	1.00E+00
KPNB1	-1.88E+00	8.26E-40	LOC102401184	3.34E-01	1.00E+00	AAED1	-1.33E-01	1.00E+00
SIGLEC10	-4.50E+00	8.28E-40	ZNF32	-1.41E-01	1.00E+00	SPOCK1	-1.22E-01	1.00E+00
CPD	-1.80E+00	8.75E-40	COMMD5	-2.19E-01	1.00E+00	HOMER2	-1.33E-01	1.00E+00
MGARP	-5.07E+00	8.88E-40	EMC10	-1.14E-01	1.00E+00	TMEM268	-1.03E-01	1.00E+00
TSTA3	-1.99E+00	9.04E-40	CLDN23	4.60E-01	1.00E+00	TRMT10A	-1.60E-01	1.00E+00
PDCD7	1.47E+00	9.04E-40	NEURL1B	-3.11E-	1.00E+00	ATP1B1	-1.19E-	1.00E+00

				01			01	
VPS41	1.19E+00	9.20E-40	RPL5	-9.49E-02	1.00E+00	LOC102411134	2.17E-01	1.00E+00
LOC102414738	-6.43E+00	9.47E-40	LOC102411827	7.99E-02	1.00E+00	NDUFA12	1.31E-01	1.00E+00
LOC102413519	-3.00E+00	9.60E-40	HNRNPUL2	-8.77E-02	1.00E+00	SLC25A1	-1.51E-01	1.00E+00
KLHL17	-4.70E+00	1.00E-39	ESF1	-1.46E-01	1.00E+00	CEP152	-1.38E-01	1.00E+00
POLA1	1.37E+00	1.03E-39	CPN1	-1.59E-01	1.00E+00	CLUAP1	-1.02E-01	1.00E+00
RNF157	-1.48E+00	1.04E-39	SGTB	1.27E-01	1.00E+00	CHMP6	1.25E-01	1.00E+00
LOC102395720	5.38E+00	1.06E-39	DPH7	-1.17E-01	1.00E+00	LOC102411696	-2.21E-01	1.00E+00
GNL2	1.20E+00	1.07E-39	ICE1	1.76E-01	1.00E+00	CDK12	-1.25E-01	1.00E+00
FGF5	5.63E+00	1.17E-39	KDELR1	9.54E-02	1.00E+00	HAS2	2.60E-01	1.00E+00
CTHRC1	-7.68E+00	1.18E-39	NT5C2	9.30E-02	1.00E+00	TMEM167B	1.03E-01	1.00E+00
ZNF292	-2.42E+00	1.19E-39	UNC13B	1.17E-01	1.00E+00	FBXO7	-1.06E-01	1.00E+00
LOC112586833	2.30E+00	1.26E-39	CNIH1	1.00E-01	1.00E+00	GCC1	1.42E-01	1.00E+00
KCTD8	-4.32E+00	1.31E-39	LOC112582017	-2.36E-01	1.00E+00	LOC102410221	-1.85E-01	1.00E+00
BRD7	1.25E+00	1.33E-39	LYRM2	1.91E-01	1.00E+00	CWC25	-1.25E-01	1.00E+00
RNF222	4.67E+00	1.34E-39	CLUH	9.26E-02	1.00E+00	DLG3	-9.48E-02	1.00E+00
HIBADH	-1.27E+00	1.34E-39	RIMBP2	2.46E-01	1.00E+00	CCDC92B	2.25E-01	1.00E+00
GRIP2	7.84E+00	1.44E-39	SENP5	-9.31E-02	1.00E+00	SULF2	2.45E-01	1.00E+00
AMPD3	4.09E+00	1.47E-39	RAB3A	-1.30E-01	1.00E+00	CLINT1	-1.26E-01	1.00E+00
NOC3L	1.13E+00	1.50E-39	KNL1	1.47E-01	1.00E+00	KIAA0319	-2.15E-01	1.00E+00
ARMCX1	-6.08E+00	1.52E-39	GANAB	1.08E-01	1.00E+00	ZNF283	-1.83E-01	1.00E+00
SAMHD1	1.41E+00	1.53E-39	TRAPPC12	-1.49E-01	1.00E+00	CAP2	1.25E-01	1.00E+00
KIAA0319	2.58E+00	1.55E-39	DPM2	1.45E-01	1.00E+00	CENPL	-1.40E-01	1.00E+00
ITPKC	-1.93E+00	1.66E-39	LOC102409358	-1.22E-01	1.00E+00	MIEF1	1.10E-01	1.00E+00
ZBTB42	-5.99E+00	1.76E-39	CCDC198	6.26E-01	1.00E+00	LOC112582098	2.63E-01	1.00E+00
LOC102398530	-1.79E+00	1.79E-39	CENPX	1.43E-01	1.00E+00	AKAP8L	-1.19E-01	1.00E+00
CSNK1G2	-1.43E+00	1.81E-39	GPC3	8.62E-02	1.00E+00	DGKI	1.75E-01	1.00E+00
JAK3	-5.98E+00	1.82E-39	LOC112582058	1.86E-01	1.00E+00	SFT2D3	-1.57E-01	1.00E+00
GSG1L	-3.71E+00	1.84E-39	PORCN	1.40E-01	1.00E+00	CERS6	3.01E-01	1.00E+00
OTULINL	-1.97E+00	1.87E-39	KMT2D	2.42E-01	1.00E+00	CDC42SE2	1.00E-01	1.00E+00
RARS	1.49E+00	1.90E-39	GTF3C6	-1.39E-01	1.00E+00	YTHDC2	-1.50E-01	1.00E+00
CITED1	-6.61E+00	1.97E-39	RNPEPL1	1.23E-01	1.00E+00	FBXO31	1.13E-01	1.00E+00
MED15	1.44E+00	2.01E-39	LOC112584695	-3.26E-01	1.00E+00	LOC102408258	-2.17E-01	1.00E+00

METTL2A	-2.68E+00	2.07E-39	SLC12A2	1.41E-01	1.00E+00	FBXO30	-2.20E-01	1.00E+00
RBMS2	-1.29E+00	2.13E-39	RUNDC3B	-1.29E-01	1.00E+00	LOC112585315	-2.95E-01	1.00E+00
ELOVL7	2.21E+00	2.15E-39	LOC112582084	2.64E-01	1.00E+00	SEPT1	-1.93E-01	1.00E+00
TENM2	4.24E+00	2.17E-39	SLC6A6	-2.85E-01	1.00E+00	SMUG1	1.05E-01	1.00E+00
RPL19	-2.52E+00	2.23E-39	SHC1	9.59E-02	1.00E+00	LOC102416136	2.20E-01	1.00E+00
G3BP1	-1.45E+00	2.26E-39	CCDC152	-3.04E-01	1.00E+00	NUP107	-1.07E-01	1.00E+00
FOXI1	6.76E+00	2.27E-39	TARSL2	-1.33E-01	1.00E+00	PHF19	-1.09E-01	1.00E+00
TUFT1	1.78E+00	2.36E-39	LOXL3	6.74E-01	1.00E+00	ZFP14	1.62E-01	1.00E+00
POU4F3	7.83E+00	2.37E-39	LOC112585227	1.23E-01	1.00E+00	SLC16A11	-1.83E-01	1.00E+00
CCDC12	-1.74E+00	2.41E-39	DGKD	-1.03E-01	1.00E+00	RNF145	-1.07E-01	1.00E+00
PHC2	-1.53E+00	2.48E-39	ABHD16B	2.88E-01	1.00E+00	NUBPL	1.30E-01	1.00E+00
ABCB8	-1.46E+00	2.49E-39	LOC102396296	2.64E-01	1.00E+00	STARD6	-2.11E-01	1.00E+00
CLEC4F	5.14E+00	2.56E-39	STARD3NL	-1.46E-01	1.00E+00	OTUD3	-1.20E-01	1.00E+00
LOC112582213	8.25E+00	2.59E-39	MAP2K2	1.00E-01	1.00E+00	LOC102404070	2.40E-01	1.00E+00
LOC102412652	-3.60E+00	2.59E-39	NPR1	3.83E-01	1.00E+00	SAMD10	2.18E-01	1.00E+00
ANKRD34A	2.76E+00	2.61E-39	LOC102397881	1.04E-01	1.00E+00	TSPAN17	9.97E-02	1.00E+00
MFSD10	-1.71E+00	2.64E-39	ZNF835	-2.82E-01	1.00E+00	LYL1	-2.48E-01	1.00E+00
PARD3	-1.23E+00	2.64E-39	NLRP5	1.04E-01	1.00E+00	LOC102394634	-2.05E-01	1.00E+00
HIVEP1	-2.31E+00	2.65E-39	CRB3	1.24E-01	1.00E+00	LOC102394379	2.55E-01	1.00E+00
TMEM109	1.39E+00	2.66E-39	MED26	-1.24E-01	1.00E+00	GFOD2	1.37E-01	1.00E+00
LOC112582106	6.09E+00	2.73E-39	SLC7A11	1.75E-01	1.00E+00	ZFPM2	1.13E-01	1.00E+00
DSCAML1	7.11E+00	2.74E-39	GPCPD1	-1.09E-01	1.00E+00	SOS1	1.21E-01	1.00E+00
DDB2	1.27E+00	2.76E-39	LOC112580389	-6.82E-01	1.00E+00	POLD3	-1.03E-01	1.00E+00
LOC102397911	2.43E+00	2.99E-39	TUT7	-1.41E-01	1.00E+00	ZC3HAV1	-1.39E-01	1.00E+00
DNLZ	-2.46E+00	3.00E-39	RPL32	-1.43E-01	1.00E+00	FAM89A	1.88E-01	1.00E+00
LOC102390251	4.51E+00	3.01E-39	NDUFAF2	-1.85E-01	1.00E+00	NMNAT2	1.07E-01	1.00E+00
HMGA1	2.49E+00	3.12E-39	LRRC4C	-1.54E-01	1.00E+00	DOCK5	-1.11E-01	1.00E+00
LOC112578687	5.02E+00	3.12E-39	TAB1	9.21E-02	1.00E+00	GAK	1.01E-01	1.00E+00
TGDS	-3.27E+00	3.13E-39	SPPL3	1.52E-01	1.00E+00	LOC112577660	-2.20E-01	1.00E+00
LOC112578703	6.08E+00	3.15E-39	GLYR1	9.07E-02	1.00E+00	PTPRA	-9.11E-02	1.00E+00
RNF123	-1.40E+00	3.19E-39	DET1	1.14E-01	1.00E+00	ELL3	-2.27E-01	1.00E+00
SEC31B	1.43E+00	3.21E-39	RPL14	-1.10E-01	1.00E+00	SENP7	-1.42E-01	1.00E+00
CPXM1	-6.61E+00	3.21E-39	ITGB3	3.31E-01	1.00E+00	TBC1D4	1.12E-01	1.00E+00
DOLK	1.48E+00	3.23E-39	KIAA1210	2.02E-01	1.00E+00	TMEM87B	1.29E-01	1.00E+00

LOC112584592	4.00E+00	3.24E-39	STARD9	-1.50E-01	1.00E+00	UBE3B	-9.09E-02	1.00E+00
LOC112582974	6.21E+00	3.25E-39	ZGPAT	-2.91E-01	1.00E+00	C12H9orf16	2.08E-01	1.00E+00
SMG8	1.52E+00	3.28E-39	TAS1R3	-2.52E-01	1.00E+00	EPC1	-9.07E-02	1.00E+00
LOC102416551	7.93E+00	3.28E-39	KLRG2	-2.45E-01	1.00E+00	LOC112581642	1.79E-01	1.00E+00
ATP2A2	1.93E+00	3.30E-39	PRSS35	1.48E-01	1.00E+00	RAB3IP	-1.12E-01	1.00E+00
SGTB	2.97E+00	3.31E-39	LOC112586630	-3.97E-01	1.00E+00	APMAP	-1.10E-01	1.00E+00
LTB	-5.76E+00	3.33E-39	GNAI1	-1.30E-01	1.00E+00	LOC102404549	-1.91E-01	1.00E+00
MZT1	-4.10E+00	3.43E-39	ARMC2	-1.05E-01	1.00E+00	ALG8	-1.14E-01	1.00E+00
TMEM63A	-1.95E+00	3.46E-39	MYO5C	-1.15E-01	1.00E+00	LOC102395160	1.24E-01	1.00E+00
IDH3A	-1.36E+00	3.86E-39	LOC112580793	-1.37E-01	1.00E+00	PLCH1	2.53E-01	1.00E+00
LOC102400719	7.53E+00	4.08E-39	C5H1orf53	-2.16E-01	1.00E+00	ARHGAP44	1.42E-01	1.00E+00
LOC102398382	-4.23E+00	4.08E-39	XPR1	1.02E-01	1.00E+00	MLF2	1.09E-01	1.00E+00
JAKMIP3	-4.11E+00	4.56E-39	LOC112585225	-1.29E-01	1.00E+00	LOC112579672	-2.77E-01	1.00E+00
ERP29	-2.17E+00	4.62E-39	ZSCAN20	1.53E-01	1.00E+00	RAB28	-9.91E-02	1.00E+00
EIF2B1	1.56E+00	4.64E-39	PDZD3	-3.92E-01	1.00E+00	CENPJ	-1.12E-01	1.00E+00
CRHR2	4.60E+00	4.77E-39	SPACA9	3.72E-01	1.00E+00	UFM1	1.27E-01	1.00E+00
RITA1	1.46E+00	4.82E-39	CTIF	-3.33E-01	1.00E+00	DCDC1	1.92E-01	1.00E+00
OSTC	-1.91E+00	4.82E-39	STX4	-1.73E-01	1.00E+00	PPIL3	1.26E-01	1.00E+00
MTUS2	5.18E+00	4.85E-39	ZNF322	-4.26E-01	1.00E+00	NQO1	-1.79E-01	1.00E+00
KIDINS220	1.77E+00	5.02E-39	NEFH	3.01E-01	1.00E+00	COQ10B	-1.78E-01	1.00E+00
METTL15	-2.03E+00	5.09E-39	LOC112579967	-4.47E-01	1.00E+00	RAB3GAP1	-1.01E-01	1.00E+00
GATD3A	-1.89E+00	5.55E-39	DRAM2	-1.80E-01	1.00E+00	ZNF326	-1.14E-01	1.00E+00
KCNE4	-7.18E+00	5.69E-39	ZNF239	2.37E-01	1.00E+00	EIF4B	-1.23E-01	1.00E+00
ADCY7	5.22E+00	5.83E-39	JPT1	-9.78E-02	1.00E+00	ANKRD28	1.18E-01	1.00E+00
TSPYL1	1.31E+00	5.85E-39	VEPH1	-2.25E-01	1.00E+00	LOC112587006	-2.39E-01	1.00E+00
ARL14EP	1.56E+00	5.97E-39	AMOTL2	-6.28E-01	1.00E+00	DDX5	-1.57E-01	1.00E+00
MRM3	1.40E+00	6.10E-39	C9H19orf70	-1.30E-01	1.00E+00	POMK	-1.06E-01	1.00E+00
UBE2F	1.29E+00	6.15E-39	SLC25A27	-9.70E-02	1.00E+00	CAMSAP1	1.14E-01	1.00E+00
CDKL5	2.59E+00	6.16E-39	FAM89B	-1.28E-01	1.00E+00	PKD1	-1.15E-01	1.00E+00
RPS20	-2.67E+00	6.21E-39	VTI1B	-1.22E-01	1.00E+00	PTPN12	1.40E-01	1.00E+00
ST8SIA3	6.91E+00	6.22E-39	UTP25	8.37E-02	1.00E+00	SLC19A1	1.58E-01	1.00E+00
JUN	-4.37E+00	6.50E-39	SLC14A2	-2.86E-01	1.00E+00	CDK3	1.27E-01	1.00E+00
SPG21	1.43E+00	6.60E-39	ZFP1	1.52E-01	1.00E+00	LOC102414738	-2.44E-01	1.00E+00

EEF1E1	-2.09E+00	6.85E-39	CTDP1	1.25E-01	1.00E+00	PROM1	1.53E-01	1.00E+00
MCM6	1.16E+00	6.87E-39	CMBL	-5.34E-01	1.00E+00	DCP1B	-1.23E-01	1.00E+00
DPP3	-1.44E+00	6.88E-39	EPHA5	7.22E-01	1.00E+00	SKA3	-1.29E-01	1.00E+00
FRMD8	2.05E+00	7.07E-39	ELL2	1.24E-01	1.00E+00	BASP1	1.40E-01	1.00E+00
ASRGL1	1.45E+00	7.23E-39	GLTP	9.28E-02	1.00E+00	RASIP1	-1.97E-01	1.00E+00
SLIT3	2.87E+00	7.30E-39	PMM1	-1.45E-01	1.00E+00	EIF2A	-9.83E-02	1.00E+00
PRRT4	2.77E+00	7.89E-39	LOC112587915	-2.29E-01	1.00E+00	BZW2	-9.65E-02	1.00E+00
PCSK4	-3.83E+00	9.09E-39	SPSB1	-5.40E-01	1.00E+00	ZC3HAV1L	-1.67E-01	1.00E+00
ATP5PO	-2.48E+00	9.20E-39	UTF1	4.80E-01	1.00E+00	SMS	9.82E-02	1.00E+00
SSC4D	3.56E+00	9.75E-39	LOC112582347	3.32E-01	1.00E+00	PSMA1	1.09E-01	1.00E+00
MAP3K9	3.03E+00	9.95E-39	ING1	1.00E-01	1.00E+00	RALB	9.92E-02	1.00E+00
LOC112584978	4.00E+00	1.02E-38	VPS39	1.22E-01	1.00E+00	SNX24	-1.08E-01	1.00E+00
ZBTB39	2.14E+00	1.03E-38	CDH17	6.15E-01	1.00E+00	ARMC2	2.29E-01	1.00E+00
FGGY	1.71E+00	1.03E-38	PLEKHM1	1.42E-01	1.00E+00	TMEM182	1.74E-01	1.00E+00
DPYSL2	-6.20E+00	1.04E-38	LOC102390535	-4.79E-01	1.00E+00	MAP3K21	-1.30E-01	1.00E+00
MCEE	-2.99E+00	1.05E-38	LOC102399409	-2.44E-01	1.00E+00	ELP6	-1.78E-01	1.00E+00
CDK5R1	2.60E+00	1.05E-38	CILP	9.46E-02	1.00E+00	LOC112586835	-2.46E-01	1.00E+00
LOC112587647	-6.44E+00	1.08E-38	TGIF1	-2.56E-01	1.00E+00	NUDT19	-1.03E-01	1.00E+00
LOC102395796	-2.30E+00	1.09E-38	C1H3orf58	4.09E-01	1.00E+00	DZIP1	-9.47E-02	1.00E+00
TAAR1	-7.32E+00	1.11E-38	HERC1	1.66E-01	1.00E+00	OSBPL2	-9.10E-02	1.00E+00
PLEC	-2.56E+00	1.11E-38	ZNF385A	-2.05E-01	1.00E+00	ZNF623	1.24E-01	1.00E+00
MAFA	3.47E+00	1.12E-38	HMGB2	-9.58E-02	1.00E+00	RHOD	2.23E-01	1.00E+00
GNA13	1.60E+00	1.15E-38	SNX29	-8.45E-02	1.00E+00	PLA2G12A	-1.19E-01	1.00E+00
NCEH1	-5.65E+00	1.21E-38	LOC102397700	1.59E-01	1.00E+00	HIRIP3	-1.33E-01	1.00E+00
C21H3orf18	-3.40E+00	1.22E-38	MCAT	1.70E-01	1.00E+00	LOC102390802	-1.42E-01	1.00E+00
LOC102390601	1.75E+00	1.25E-38	XRCC5	9.94E-02	1.00E+00	LOC112579536	-2.32E-01	1.00E+00
ANKRD23	-2.29E+00	1.29E-38	FN3KRP	-1.24E-01	1.00E+00	CANX	-1.31E-01	1.00E+00
WASF2	1.50E+00	1.29E-38	AKAP12	1.60E-01	1.00E+00	SEC23A	-1.39E-01	1.00E+00
GALNT7	-2.17E+00	1.32E-38	ENOPH1	1.08E-01	1.00E+00	TCFL5	1.59E-01	1.00E+00
GTF3C6	-1.42E+00	1.34E-38	EXOSC5	2.01E-01	1.00E+00	ITGAE	1.39E-01	1.00E+00
WNT6	-7.14E+00	1.35E-38	LOC102392405	-2.09E-01	1.00E+00	IVNS1ABP	-1.85E-01	1.00E+00
LOC112578474	7.13E+00	1.37E-38	CROCC2	2.90E-01	1.00E+00	STKLD1	2.13E-01	1.00E+00
BAZ2B	-2.07E+00	1.38E-38	LOC102404484	-1.99E-01	1.00E+00	FOXK2	-9.21E-02	1.00E+00
LOC102404996	-5.34E+00	1.38E-38	ECHS1	8.15E-02	1.00E+00	MTBP	-1.11E-01	1.00E+00

NDUFS7	-1.93E+00	1.39E-38	DNAJC1	-9.72E-02	1.00E+00	DOCK6	-9.93E-02	1.00E+00
FAM227A	-7.71E+00	1.39E-38	ZNF777	1.05E-01	1.00E+00	PRPF3	-9.09E-02	1.00E+00
C8G	-4.32E+00	1.40E-38	TMTC3	1.43E-01	1.00E+00	PARP2	-1.15E-01	1.00E+00
IGF2BP1	5.33E+00	1.41E-38	WDR77	1.25E-01	1.00E+00	LOC112582115	1.26E-01	1.00E+00
ASCL3	7.03E+00	1.44E-38	DCUN1D2	2.84E-01	1.00E+00	THOC2	-1.39E-01	1.00E+00
RNF217	-2.24E+00	1.44E-38	VCPIP1	1.23E-01	1.00E+00	UBE2Q2	-9.37E-02	1.00E+00
ANKRD1	-6.24E+00	1.47E-38	ZDHHC17	2.57E-01	1.00E+00	SMARCA2	-1.03E-01	1.00E+00
LAMTOR1	-1.61E+00	1.49E-38	KPNA7	1.05E-01	1.00E+00	NUP62CL	-1.36E-01	1.00E+00
LOC102396266	9.08E+00	1.52E-38	TYRO3	-4.11E-01	1.00E+00	LOC102413318	-2.75E-01	1.00E+00
SEPT2	-1.26E+00	1.64E-38	TSPAN31	-1.25E-01	1.00E+00	TATDN2	1.03E-01	1.00E+00
YIPF6	1.33E+00	1.64E-38	ARL6IP4	-1.53E-01	1.00E+00	WT1	-1.28E-01	1.00E+00
C15H8orf33	-2.14E+00	1.65E-38	INO80C	-1.41E-01	1.00E+00	AFDN	-1.33E-01	1.00E+00
LOC102414896	6.39E+00	1.67E-38	SRXN1	-1.82E-01	1.00E+00	FAM178B	2.60E-01	1.00E+00
SV2C	4.42E+00	1.68E-38	SOX1	-2.33E-01	1.00E+00	TYROBP	-4.42E-01	1.00E+00
LOC112584418	5.18E+00	1.73E-38	AAR2	1.13E-01	1.00E+00	RNF111	-1.38E-01	1.00E+00
AFF1	1.82E+00	1.73E-38	REC114	-1.18E-01	1.00E+00	CC2D2A	-1.21E-01	1.00E+00
KLHL26	-3.01E+00	1.73E-38	THADA	1.23E-01	1.00E+00	KCNN4	3.18E-01	1.00E+00
GNAI1	2.20E+00	1.84E-38	BEND3	4.80E-01	1.00E+00	RIDA	1.48E-01	1.00E+00
KLHDC1	2.12E+00	2.02E-38	PKD1	1.29E-01	1.00E+00	ANK3	-1.63E-01	1.00E+00
PIK3IP1	-2.15E+00	2.13E-38	ZMYM5	8.54E-02	1.00E+00	GUCD1	1.07E-01	1.00E+00
PRRX1	-3.71E+00	2.14E-38	SLC25A30	1.07E-01	1.00E+00	HAUS2	-1.38E-01	1.00E+00
RPL38	-2.59E+00	2.15E-38	VARS	-1.25E-01	1.00E+00	LOC112581423	-2.08E-01	1.00E+00
PLOD2	-4.76E+00	2.26E-38	LOC112583681	-4.02E-01	1.00E+00	LOC112583621	-2.28E-01	1.00E+00
ICOS	6.77E+00	2.29E-38	SLC5A2	-3.27E-01	1.00E+00	NAXD	1.01E-01	1.00E+00
NAE1	1.22E+00	2.31E-38	ST8SIA2	-2.44E-01	1.00E+00	LOC102408337	-2.04E-01	1.00E+00
ADPRH	-3.65E+00	2.46E-38	KCNA7	-2.28E-01	1.00E+00	GTF2F2	9.47E-02	1.00E+00
ATR	-2.04E+00	2.48E-38	ERN2	2.52E-01	1.00E+00	LOC102404595	-2.59E-01	1.00E+00
WNK1	1.91E+00	2.49E-38	GAS2L2	2.88E-01	1.00E+00	LOC102390363	-1.02E-01	1.00E+00
LOC102404507	3.88E+00	2.56E-38	RAD51AP1	8.41E-02	1.00E+00	TRAF7	-1.02E-01	1.00E+00
FGL1	-6.27E+00	2.61E-38	ACOT11	-2.08E-01	1.00E+00	HDAC11	-2.15E-01	1.00E+00
IQSEC1	1.81E+00	2.65E-38	ASIC2	-5.12E-01	1.00E+00	ZNF518A	-1.60E-01	1.00E+00
LOC112580319	3.66E+00	2.74E-38	ERO1A	-1.60E-01	1.00E+00	FLII	-9.63E-02	1.00E+00
NOL3	-1.93E+00	2.75E-38	RPAP2	-9.07E-02	1.00E+00	YEATS4	-1.20E-01	1.00E+00

NDRG4	2.39E+00	2.76E-38	ZNF384	9.45E-02	1.00E+00	RNASEH2B	-1.29E-01	1.00E+00
ARHGAP32	-2.22E+00	2.85E-38	DST	-2.38E-01	1.00E+00	LOC102402779	-1.42E-01	1.00E+00
HYAL2	-1.50E+00	2.86E-38	NMNAT2	-2.33E-01	1.00E+00	LOC102410458	2.34E-01	1.00E+00
NLGN2	-3.83E+00	2.97E-38	OXNAD1	2.68E-01	1.00E+00	PRICKLE3	-1.57E-01	1.00E+00
PHLPP2	2.00E+00	3.01E-38	MTMR8	1.84E-01	1.00E+00	LOC112583863	2.15E-01	1.00E+00
LOC102408370	2.67E+00	3.15E-38	EAF2	-3.39E-01	1.00E+00	PDE9A	1.31E-01	1.00E+00
CEBPB	-3.97E+00	3.18E-38	C15H8orf89	-2.32E-01	1.00E+00	GALT	-1.68E-01	1.00E+00
CCNYL1	2.19E+00	3.23E-38	SEPHS2	-9.48E-02	1.00E+00	HSPA12B	-2.03E-01	1.00E+00
KIAA0753	1.44E+00	3.31E-38	GNAT3	4.18E-01	1.00E+00	SURF4	-9.87E-02	1.00E+00
TARBP1	-1.77E+00	3.44E-38	CHSY1	1.72E-01	1.00E+00	CARMIL2	-2.34E-01	1.00E+00
NAV3	6.02E+00	3.48E-38	ANKRD45	-2.81E-01	1.00E+00	LOC102410930	3.77E-01	1.00E+00
KIF17	2.04E+00	3.63E-38	FAN1	-1.12E-01	1.00E+00	MYO9B	1.03E-01	1.00E+00
LOC112587909	7.95E+00	3.77E-38	LOC102406480	1.05E-01	1.00E+00	ACTN1	1.82E-01	1.00E+00
CAMK2D	2.10E+00	3.83E-38	LOC102407141	-5.09E-01	1.00E+00	AK4	1.14E-01	1.00E+00
LMCD1	-3.33E+00	3.88E-38	TPI1	-1.35E-01	1.00E+00	PER1	-1.09E-01	1.00E+00
C9H19orf44	-2.28E+00	3.91E-38	CISD2	-1.18E-01	1.00E+00	FBP1	-1.57E-01	1.00E+00
RWDD1	-2.37E+00	4.02E-38	SNRNP200	1.18E-01	1.00E+00	GPN1	-9.69E-02	1.00E+00
NMRAL1	-2.50E+00	4.02E-38	DENND5B	-1.23E-01	1.00E+00	PLPP5	-1.04E-01	1.00E+00
ZC3H12C	-2.40E+00	4.04E-38	PIAS3	1.60E-01	1.00E+00	LOC102410179	-2.51E-01	1.00E+00
ACAA1	-2.07E+00	4.06E-38	COX2	-2.26E-01	1.00E+00	MBD3	1.23E-01	1.00E+00
CLK3	-1.29E+00	4.20E-38	MEGF9	9.73E-02	1.00E+00	LDB1	-1.03E-01	1.00E+00
LRP8	-7.31E+00	4.21E-38	LOC112577789	-1.40E-01	1.00E+00	LOC112583712	-1.25E-01	1.00E+00
PLD2	-4.71E+00	4.22E-38	BRWD3	1.34E-01	1.00E+00	LOC102391313	2.18E-01	1.00E+00
SMIM7	-1.60E+00	4.29E-38	EPDR1	7.30E-01	1.00E+00	LAMTOR1	1.08E-01	1.00E+00
TMPO	-1.90E+00	4.39E-38	CCDC78	2.68E-01	1.00E+00	TIMM21	-1.09E-01	1.00E+00
RTN4	-1.29E+00	4.52E-38	NUDT21	-7.14E-02	1.00E+00	LOC112586663	-2.22E-01	1.00E+00
DDX24	1.31E+00	4.57E-38	CBX3	-2.49E-01	1.00E+00	ATR	-1.20E-01	1.00E+00
LOC112583612	5.44E+00	4.58E-38	CAMKK2	2.11E-01	1.00E+00	DOCK3	1.50E-01	1.00E+00
ZNF687	-1.38E+00	4.58E-38	LOC112585683	3.92E-01	1.00E+00	RBBP4	-8.81E-02	1.00E+00
DUSP28	-2.29E+00	4.58E-38	METAP1D	2.53E-01	1.00E+00	CFAP97	-1.20E-01	1.00E+00
LOC102412030	8.35E+00	4.71E-38	MCM2	8.08E-02	1.00E+00	ATAD5	-1.43E-01	1.00E+00
LOC112583129	-6.19E+00	4.87E-38	CFB	1.28E-01	1.00E+00	DESI1	-1.04E-01	1.00E+00
LOC102410806	4.88E+00	4.96E-38	OCA2	2.06E-01	1.00E+00	CHMP1A	9.86E-02	1.00E+00
SLC12A1	3.03E+00	5.07E-38	CEP162	2.08E-01	1.00E+00	FAM98C	1.41E-01	1.00E+00

GABRA5	5.75E+00	5.09E-38	PRKAR1B	5.48E-01	1.00E+00	EIF1B	1.02E-01	1.00E+00
LOC102398374	-4.42E+00	5.30E-38	TOX	2.30E-01	1.00E+00	TMEM158	-2.17E-01	1.00E+00
TSPYL4	-1.50E+00	5.39E-38	UBC	9.43E-02	1.00E+00	STIM1	-9.32E-02	1.00E+00
LOC102402606	1.28E+00	5.48E-38	CBX7	-2.95E-01	1.00E+00	TPRG1L	1.25E-01	1.00E+00
SDC4	-6.29E+00	5.54E-38	LOC102408857	1.74E-01	1.00E+00	CUL9	-9.33E-02	1.00E+00
NFIC	-1.40E+00	5.55E-38	CPEB3	1.30E-01	1.00E+00	MTERF4	1.15E-01	1.00E+00
INTS1	-1.46E+00	5.68E-38	NISCH	9.98E-02	1.00E+00	LOC102411789	-2.59E-01	1.00E+00
CSPP1	1.40E+00	5.79E-38	LOC112578742	-4.79E-01	1.00E+00	METTL25	-1.48E-01	1.00E+00
NBR1	1.52E+00	5.87E-38	LOC102395095	-2.40E-01	1.00E+00	SUB1	1.05E-01	1.00E+00
PPP1R42	-6.16E+00	5.92E-38	GSPT1	1.03E-01	1.00E+00	RASSF7	1.62E-01	1.00E+00
FUT4	-3.19E+00	6.00E-38	AURKAIP1	-1.42E-01	1.00E+00	MIGA1	1.47E-01	1.00E+00
RPA2	1.45E+00	6.36E-38	CLCN4	1.04E-01	1.00E+00	RGS17	1.04E-01	1.00E+00
SCARF1	-4.38E+00	6.56E-38	PLXNB2	-1.84E-01	1.00E+00	LOC102411403	2.53E-01	1.00E+00
CSRNP2	1.67E+00	6.65E-38	TP73	-4.87E-01	1.00E+00	IQCE	-1.00E-01	1.00E+00
RALA	1.17E+00	6.93E-38	LOC102395906	-2.64E-01	1.00E+00	LOC102409914	-1.12E-01	1.00E+00
NR1D2	-4.25E+00	7.01E-38	LRRC29	2.29E-01	1.00E+00	COLGALT1	-1.39E-01	1.00E+00
EFNA3	4.10E+00	7.11E-38	TLN2	1.18E-01	1.00E+00	PLA2G3	-1.67E-01	1.00E+00
RIN2	-5.59E+00	7.27E-38	TPPP	-2.22E-01	1.00E+00	NAMPT	-1.50E-01	1.00E+00
GTPBP8	-2.86E+00	7.38E-38	CALM3	9.20E-02	1.00E+00	WDR91	-9.92E-02	1.00E+00
LRRC29	3.84E+00	7.48E-38	LIG4	2.18E-01	1.00E+00	EIF4E	-1.06E-01	1.00E+00
RPL7	-3.32E+00	7.67E-38	TMEM37	-3.11E-01	1.00E+00	OIP5	-2.31E-01	1.00E+00
LOC112578016	-3.93E+00	7.69E-38	METTL21A	1.24E-01	1.00E+00	PER3	2.14E-01	1.00E+00
LOC102395276	1.55E+00	7.71E-38	KIF6	-2.06E-01	1.00E+00	ARFGEF1	-1.15E-01	1.00E+00
SH3GL3	-4.67E+00	7.89E-38	SETD7	-1.81E-01	1.00E+00	ST3GAL2	-1.10E-01	1.00E+00
TDRD12	2.85E+00	8.05E-38	ZNF335	1.47E-01	1.00E+00	LOC102402202	-2.25E-01	1.00E+00
LAMC3	-5.78E+00	8.37E-38	TCP11L2	-1.05E-01	1.00E+00	HHIP	-3.04E-01	1.00E+00
C4H12orf60	3.84E+00	8.87E-38	LOC102398059	-1.34E-01	1.00E+00	EIF6	1.24E-01	1.00E+00
SELP	-8.01E+00	8.98E-38	FER1L5	-2.43E-01	1.00E+00	NUF2	-1.39E-01	1.00E+00
LOC102406175	1.96E+00	9.07E-38	KIAA1324	-4.28E-01	1.00E+00	SNX8	9.78E-02	1.00E+00
RBM20	4.89E+00	9.68E-38	POLR3A	1.01E-01	1.00E+00	ITIH4	1.43E-01	1.00E+00
EIF3G	-2.11E+00	9.97E-38	LOC102409482	4.70E-01	1.00E+00	FAM124A	-2.02E-01	1.00E+00
NHLRC1	-5.78E+00	1.01E-37	DCLK2	8.16E-02	1.00E+00	MYCL	1.86E-01	1.00E+00
STRADB	1.33E+00	1.04E-37	CEP126	-4.25E-01	1.00E+00	PRDM2	-1.18E-01	1.00E+00

PTCD3	-1.29E+00	1.05E-37	LITAF	-3.35E-01	1.00E+00	LOC112587901	2.11E-01	1.00E+00
LOC102396408	6.15E+00	1.06E-37	SLC12A5	-9.79E-02	1.00E+00	CSTF1	1.14E-01	1.00E+00
ACTG1	-2.72E+00	1.13E-37	UNC80	-1.49E-01	1.00E+00	EPB41L1	1.24E-01	1.00E+00
AHNAK	-4.99E+00	1.15E-37	ATAD5	2.32E-01	1.00E+00	SAP25	-1.74E-01	1.00E+00
RRP12	-1.65E+00	1.19E-37	PDHB	-1.43E-01	1.00E+00	CCDC62	-1.08E-01	1.00E+00
LOC102398353	-2.18E+00	1.23E-37	EHMT2	8.09E-02	1.00E+00	CASP9	-1.39E-01	1.00E+00
LOC102394332	-5.55E+00	1.25E-37	CREB3	1.37E-01	1.00E+00	GATD3A	-1.30E-01	1.00E+00
POLR1C	-1.87E+00	1.26E-37	ASAP2	8.58E-02	1.00E+00	NUP214	-9.08E-02	1.00E+00
MEGF11	5.18E+00	1.31E-37	LOC112586993	-2.47E-01	1.00E+00	LOC112579997	2.93E-01	1.00E+00
FHL3	-5.62E+00	1.32E-37	LOC112581162	1.97E-01	1.00E+00	TMEM143	-1.26E-01	1.00E+00
LOC102399664	-7.49E+00	1.38E-37	NUP133	7.78E-02	1.00E+00	RNASEL	2.26E-01	1.00E+00
RNF182	-3.30E+00	1.41E-37	MPZL3	9.26E-02	1.00E+00	LOC102391408	1.14E-01	1.00E+00
RBL1	-2.96E+00	1.44E-37	LOC112577737	-2.94E-01	1.00E+00	TLR4	-5.02E-01	1.00E+00
LOC102409528	-5.68E+00	1.49E-37	PPP1R13B	1.04E-01	1.00E+00	RRBP1	-1.24E-01	1.00E+00
LOC112580303	5.44E+00	1.51E-37	FBXW8	-1.07E-01	1.00E+00	LOC102402106	2.55E-01	1.00E+00
CTXN1	-3.13E+00	1.56E-37	PIM3	-8.85E-02	1.00E+00	FAM50A	-1.05E-01	1.00E+00
GLRA1	5.96E+00	1.61E-37	TET3	-1.53E-01	1.00E+00	KAT6B	-1.37E-01	1.00E+00
LOC112585131	6.56E+00	1.62E-37	LOC112582182	-4.60E-01	1.00E+00	PBXIP1	9.83E-02	1.00E+00
TXLNB	3.69E+00	1.62E-37	SPPL2B	9.94E-02	1.00E+00	ZEB2	1.18E-01	1.00E+00
MAVS	-2.31E+00	1.72E-37	PCCB	-8.38E-02	1.00E+00	PLOD3	-1.01E-01	1.00E+00
CDV3	-1.12E+00	1.73E-37	LGALS3BP	-4.85E-01	1.00E+00	SYNC	1.50E-01	1.00E+00
USP12	-2.30E+00	1.75E-37	ECT2	9.70E-02	1.00E+00	RASGRF2	2.80E-01	1.00E+00
LOC102416020	-8.54E+00	1.80E-37	WDR88	5.47E-01	1.00E+00	ASB15	-2.03E-01	1.00E+00
PFKFB3	-3.91E+00	1.87E-37	LOC102392741	3.77E-01	1.00E+00	FHIT	1.50E-01	1.00E+00
KNG1	6.47E+00	1.89E-37	MTMR4	1.20E-01	1.00E+00	LOC102395477	-2.01E-01	1.00E+00
LRRC2	-6.42E+00	1.90E-37	HDAC1	-1.02E-01	1.00E+00	RRS1	1.26E-01	1.00E+00
TAGLN2	-3.23E+00	1.96E-37	SDR42E1	1.30E-01	1.00E+00	PPP1R21	-1.03E-01	1.00E+00
ZNF529	2.49E+00	2.01E-37	SMIM17	-2.61E-01	1.00E+00	GFM1	-1.21E-01	1.00E+00
PRR15	-6.06E+00	2.04E-37	MPV17L2	-1.17E-01	1.00E+00	SEMA5B	2.74E-01	1.00E+00
BICRA	1.75E+00	2.05E-37	SIM2	-4.12E-01	1.00E+00	LOC102394892	-1.96E-01	1.00E+00
NGDN	1.41E+00	2.05E-37	ATP6V0E2	3.89E-01	1.00E+00	CRTC1	-1.05E-01	1.00E+00
RGMA	-3.85E+00	2.16E-37	GLP2R	-4.00E-01	1.00E+00	SLC3A2	-1.19E-01	1.00E+00
DHX34	-	2.26E-37	ATP6V0A2	1.35E-01	1.00E+00	PDGFA	1.06E-01	1.00E+00

	1.90E+00							
LOC102405118	4.50E+00	2.28E-37	RBAK	2.40E-01	1.00E+00	EHD4	1.83E-01	1.00E+00
LOC112581397	6.49E+00	2.34E-37	LOC102393350	1.73E-01	1.00E+00	KBTBD8	2.09E-01	1.00E+00
TSPAN15	-4.07E+00	2.37E-37	ASH2L	-7.43E-02	1.00E+00	HEXIM1	1.07E-01	1.00E+00
SRSF2	-1.50E+00	2.49E-37	PPP4R1	9.70E-02	1.00E+00	LOC102394692	1.51E-01	1.00E+00
CKS2	1.72E+00	2.50E-37	LOC112584654	-2.93E-01	1.00E+00	SLC25A3	1.05E-01	1.00E+00
MTHFD1L	-5.85E+00	2.50E-37	SELENOO	-1.79E-01	1.00E+00	P4HTM	-1.37E-01	1.00E+00
CTNNB1	-1.36E+00	2.62E-37	FBLN2	1.33E-01	1.00E+00	ATP2C1	1.06E-01	1.00E+00
RNF5	-1.76E+00	2.64E-37	ANP32A	-9.12E-02	1.00E+00	LNX1	2.11E-01	1.00E+00
LOC102410528	1.88E+00	2.65E-37	MACROD1	-1.19E-01	1.00E+00	MAML3	-1.05E-01	1.00E+00
LOC102416262	-2.64E+00	2.71E-37	SNX25	8.48E-02	1.00E+00	LOC102392359	-1.94E-01	1.00E+00
RFWD3	2.74E+00	2.75E-37	HMGCS1	9.29E-02	1.00E+00	TPM3	-1.08E-01	1.00E+00
CHRNB2	5.08E+00	2.77E-37	FNTA	-1.11E-01	1.00E+00	PSMA5	1.13E-01	1.00E+00
C1QTNF6	-6.58E+00	2.84E-37	TATDN1	1.20E-01	1.00E+00	GEMIN6	1.17E-01	1.00E+00
PLEK2	2.00E+00	2.94E-37	BTG1	-7.76E-02	1.00E+00	AP3B2	-1.30E-01	1.00E+00
LOC112584611	-4.72E+00	3.00E-37	OPA3	1.40E-01	1.00E+00	MRPL18	1.17E-01	1.00E+00
ZNF177	-2.01E+00	3.05E-37	UBE3A	1.27E-01	1.00E+00	GAS2L1	1.13E-01	1.00E+00
LOC102406199	-1.55E+00	3.25E-37	TMOD3	-1.38E-01	1.00E+00	ACHE	-2.07E-01	1.00E+00
LOC112581457	6.84E+00	3.27E-37	AKT1S1	9.89E-02	1.00E+00	LMO4	-8.82E-02	1.00E+00
ZNF182	1.73E+00	3.30E-37	FAM124A	-1.16E-01	1.00E+00	ETFDH	-1.00E-01	1.00E+00
SNAP47	1.16E+00	3.37E-37	GRM8	1.60E-01	1.00E+00	STOM	1.07E-01	1.00E+00
BCCIP	-1.58E+00	3.39E-37	TPRA1	-1.39E-01	1.00E+00	ZNF768	1.02E-01	1.00E+00
LOC102403881	-6.86E+00	3.53E-37	PFKFB4	1.25E-01	1.00E+00	IL17D	-2.02E-01	1.00E+00
POC1A	1.97E+00	3.64E-37	DHX37	1.18E-01	1.00E+00	RYK	-1.13E-01	1.00E+00
LOC112582327	-2.81E+00	3.67E-37	LOC112577891	-5.19E-01	1.00E+00	LOC102415974	-9.29E-02	1.00E+00
LOC102398503	5.44E+00	3.77E-37	EIF3I	8.55E-02	1.00E+00	NAALADL1	1.38E-01	1.00E+00
IQCC	-2.46E+00	3.78E-37	PLCD4	2.94E-01	1.00E+00	SOGA3	2.86E-01	1.00E+00
CELF3	4.77E+00	3.80E-37	CNTNAP2	8.93E-02	1.00E+00	LETMD1	-9.90E-02	1.00E+00
LRRC61	-2.70E+00	3.84E-37	MTFP1	-1.63E-01	1.00E+00	LOC102395100	1.16E-01	1.00E+00
CCNY	-1.06E+00	3.93E-37	TP53INP1	1.12E-01	1.00E+00	TTLL12	-1.09E-01	1.00E+00
XRCC6	1.33E+00	4.03E-37	LHX3	-2.29E-01	1.00E+00	NIPAL2	1.23E-01	1.00E+00
ABHD16B	3.98E+00	4.04E-37	WASHC1	-8.17E-02	1.00E+00	AJAP1	-3.92E-01	1.00E+00
LOC102391792	4.98E+00	4.14E-37	KLHL29	-1.84E-01	1.00E+00	LOC102415727	4.30E-01	1.00E+00
NELL2	-1.99E+00	4.15E-37	AMPD2	-1.26E-01	1.00E+00	EXOSC5	1.42E-01	1.00E+00

ASTN1	5.00E+00	4.15E-37	ZNF746	1.00E-01	1.00E+00	LOC102406357	1.68E-01	1.00E+00
LOC102393147	-2.41E+00	4.32E-37	CD58	8.78E-02	1.00E+00	LOC112587025	-2.17E-01	1.00E+00
LOC102402338	8.29E+00	4.37E-37	SNAPIN	-9.89E-02	1.00E+00	VPS8	8.79E-02	1.00E+00
BCL7C	-2.59E+00	4.40E-37	NEURL2	-2.92E-01	1.00E+00	ZMYM2	-1.16E-01	1.00E+00
IL22RA1	-5.73E+00	4.49E-37	ITGA4	-1.02E-01	1.00E+00	LOC102398125	2.59E-01	1.00E+00
MAP3K3	-1.35E+00	4.60E-37	SBF1	9.08E-02	1.00E+00	PRRX1	1.59E-01	1.00E+00
MTX1	-2.19E+00	4.62E-37	PELP1	1.44E-01	1.00E+00	CDK7	1.09E-01	1.00E+00
MAP2K5	-1.69E+00	4.65E-37	LOC102391171	-1.37E-01	1.00E+00	LOC102415277	-3.14E-01	1.00E+00
LOC112581189	-9.09E+00	4.75E-37	ago-03	-2.36E-01	1.00E+00	BCHE	2.61E-01	1.00E+00
LPAR1	-3.92E+00	4.80E-37	ZBTB47	2.43E-01	1.00E+00	NCAPH2	-1.16E-01	1.00E+00
FAM173A	-3.41E+00	4.94E-37	DENND4B	1.62E-01	1.00E+00	PI4K2A	9.49E-02	1.00E+00
QSER1	-1.62E+00	4.96E-37	LOC112582773	-6.39E-01	1.00E+00	EVI5L	9.62E-02	1.00E+00
LOC102388979	-4.73E+00	5.07E-37	LOC102399397	1.31E-01	1.00E+00	PDE7A	1.14E-01	1.00E+00
PTRHD1	2.07E+00	5.21E-37	LOC112579428	2.64E-01	1.00E+00	TIMM17A	1.03E-01	1.00E+00
FAF2	-1.95E+00	5.27E-37	LOC102412418	-5.58E-01	1.00E+00	DUS1L	1.27E-01	1.00E+00
AMT	-1.74E+00	5.28E-37	OXT	-2.64E-01	1.00E+00	TMEM205	1.40E-01	1.00E+00
IGFBP1	-6.79E+00	5.72E-37	OTUD7B	9.44E-02	1.00E+00	LOC102397708	1.27E-01	1.00E+00
LOC102391491	-2.82E+00	5.81E-37	ARHGEF33	9.43E-02	1.00E+00	LRFN1	-1.36E-01	1.00E+00
HSPA5	-4.07E+00	5.88E-37	LOC112586491	1.71E-01	1.00E+00	ISCA2	1.14E-01	1.00E+00
RIN3	-3.26E+00	5.94E-37	GAD1	1.24E-01	1.00E+00	PHF12	-1.05E-01	1.00E+00
SIDT2	1.82E+00	6.09E-37	SDF2	-2.16E-01	1.00E+00	CCDC87	-2.21E-01	1.00E+00
SLC2A4RG	-2.86E+00	6.17E-37	YPEL3	-9.50E-02	1.00E+00	LOC112577767	-2.08E-01	1.00E+00
RFC5	1.25E+00	6.49E-37	SAFB2	9.59E-02	1.00E+00	ERICH3	-1.25E-01	1.00E+00
STX17	1.20E+00	6.85E-37	RETREG3	-7.85E-02	1.00E+00	GAREM1	1.18E-01	1.00E+00
SIPA1	1.70E+00	7.12E-37	LSMEM1	-3.69E-01	1.00E+00	DPP8	-1.22E-01	1.00E+00
RHPN2	2.63E+00	7.23E-37	EGLN2	-9.33E-02	1.00E+00	LOC102406990	-1.11E-01	1.00E+00
ACVR1	1.95E+00	7.31E-37	FAM189A2	1.11E-01	1.00E+00	CCNJL	-1.62E-01	1.00E+00
FJX1	-6.23E+00	7.38E-37	ZW10	-9.88E-02	1.00E+00	HACE1	1.11E-01	1.00E+00
LGI4	-4.64E+00	7.41E-37	AP1AR	1.52E-01	1.00E+00	PIGA	-1.40E-01	1.00E+00
ALB	-7.49E+00	7.46E-37	ABT1	-8.00E-02	1.00E+00	MIOX	2.26E-01	1.00E+00
ACOT11	3.28E+00	8.05E-37	PTGR2	-1.12E-01	1.00E+00	SLC9A8	-1.04E-01	1.00E+00
OVOL3	5.16E+00	8.32E-37	KCNN4	-1.39E-01	1.00E+00	ARHGAP45	-2.09E-01	1.00E+00
FIP1L1	1.24E+00	8.38E-37	LOC102406790	3.18E-01	1.00E+00	COQ2	-1.16E-01	1.00E+00

PEX2	1.58E+00	8.44E-37	CCDC7	-8.39E-02	1.00E+00	NFATC1	-3.24E-01	1.00E+00
LOC102399836	7.62E+00	8.44E-37	SMIM19	-2.70E-01	1.00E+00	VWCE	2.07E-01	1.00E+00
LARP7	1.29E+00	8.56E-37	PAGE4	-4.73E-01	1.00E+00	HOXC6	-2.65E-01	1.00E+00
GGCT	1.98E+00	8.70E-37	SFSWAP	1.09E-01	1.00E+00	LOC112580647	2.33E-01	1.00E+00
DNAJC18	1.19E+00	8.99E-37	KIF1C	1.20E-01	1.00E+00	ZNF593	1.51E-01	1.00E+00
ZFYVE26	1.51E+00	9.03E-37	ANGEL2	-1.50E-01	1.00E+00	LOC102398735	2.14E-01	1.00E+00
LOC102410316	-5.19E+00	9.06E-37	LRRK1	-1.53E-01	1.00E+00	CCDC68	-1.24E-01	1.00E+00
LOC102406308	1.93E+00	9.18E-37	PROB1	3.58E-01	1.00E+00	HSBP1L1	-1.56E-01	1.00E+00
ITGB1	-3.85E+00	9.43E-37	TMEM220	-4.89E-01	1.00E+00	PAX8	1.78E-01	1.00E+00
TEX10	1.16E+00	9.49E-37	CUL5	1.65E-01	1.00E+00	SYNGAP1	-1.35E-01	1.00E+00
OPHN1	-3.10E+00	1.00E-36	ZBTB34	3.50E-01	1.00E+00	COL7A1	-2.08E-01	1.00E+00
LOC102406481	-5.43E+00	1.07E-36	SWI5	-1.02E-01	1.00E+00	XRCC3	-1.51E-01	1.00E+00
CRKL	1.33E+00	1.13E-36	DPY19L2	-3.35E-01	1.00E+00	NDOR1	-9.93E-02	1.00E+00
LOC112586457	4.91E+00	1.14E-36	LOC112578420	-5.73E-01	1.00E+00	PYURF	-1.51E-01	1.00E+00
SLC29A2	-2.32E+00	1.15E-36	LOC102402353	2.31E-01	1.00E+00	MMP25	3.09E-01	1.00E+00
TRPM7	-1.77E+00	1.15E-36	CSNK2A2	-2.90E-01	1.00E+00	CASP6	9.56E-02	1.00E+00
CCM2	-1.68E+00	1.18E-36	BCL2L14	-1.06E-01	1.00E+00	SCAF11	1.05E-01	1.00E+00
KIZ	1.26E+00	1.18E-36	TSPAN13	9.62E-02	1.00E+00	P4HB	-1.66E-01	1.00E+00
SLC5A8	-2.50E+00	1.19E-36	DENND2D	2.99E-01	1.00E+00	YBX1	-1.12E-01	1.00E+00
NELL1	5.63E+00	1.21E-36	RPS16	2.04E-01	1.00E+00	ST5	-9.79E-02	1.00E+00
AARS2	-2.22E+00	1.21E-36	DPP8	-9.58E-02	1.00E+00	LOC102402536	-1.36E-01	1.00E+00
LYPD4	6.51E+00	1.21E-36	LRRC46	2.72E-01	1.00E+00	HERPUD2	-1.15E-01	1.00E+00
TMEM74	3.38E+00	1.22E-36	NLRC5	1.16E-01	1.00E+00	FAM107A	-1.24E-01	1.00E+00
CPM	2.15E+00	1.25E-36	IDH3G	-1.09E-01	1.00E+00	SMIM7	1.04E-01	1.00E+00
RANBP17	-1.62E+00	1.29E-36	CALCR	1.44E-01	1.00E+00	DHX37	1.01E-01	1.00E+00
SELENOM	-2.59E+00	1.29E-36	MCM6	7.47E-02	1.00E+00	ABCF2	8.98E-02	1.00E+00
ZNF652	1.74E+00	1.31E-36	PRICKLE1	1.44E-01	1.00E+00	TMEM260	9.82E-02	1.00E+00
MEIS1	-4.59E+00	1.34E-36	SCAF11	1.16E-01	1.00E+00	SPTBN1	1.25E-01	1.00E+00
SPC24	-2.27E+00	1.49E-36	HILPDA	-1.24E-01	1.00E+00	SAP30L	9.97E-02	1.00E+00
ORMDL2	-1.94E+00	1.50E-36	CPTP	8.96E-02	1.00E+00	AKTIP	-1.80E-01	1.00E+00
BMP3	5.89E+00	1.52E-36	FGD4	-2.16E-01	1.00E+00	SGSM1	-1.70E-01	1.00E+00
ZNF280C	-4.81E+00	1.52E-36	SYCP3	-1.04E-01	1.00E+00	KIAA1958	-9.33E-02	1.00E+00
PAK1	1.68E+00	1.53E-36	VAMP3	-7.49E-02	1.00E+00	ZFYVE27	8.90E-02	1.00E+00

TAF9B	1.93E+00	1.54E-36	MOK	-2.29E-01	1.00E+00	SIN3B	-9.14E-02	1.00E+00
PRKACA	-1.87E+00	1.62E-36	DUSP28	2.17E-01	1.00E+00	ZNF687	-9.50E-02	1.00E+00
RNF216	1.24E+00	1.65E-36	WAS	-1.82E-01	1.00E+00	PYGB	9.64E-02	1.00E+00
FBXL5	1.18E+00	1.66E-36	NEB	1.38E-01	1.00E+00	PRDM5	-1.05E-01	1.00E+00
P4HTM	-2.60E+00	1.75E-36	CHD1L	-1.04E-01	1.00E+00	PHF10	1.14E-01	1.00E+00
HEXIM2	3.10E+00	1.78E-36	FBXO38	1.31E-01	1.00E+00	VPS53	1.12E-01	1.00E+00
SPATS1	3.22E+00	1.79E-36	MFF	-8.83E-02	1.00E+00	PLXNB1	1.06E-01	1.00E+00
PSMB8	-2.56E+00	1.81E-36	SUDS3	8.21E-02	1.00E+00	SAR1B	-1.29E-01	1.00E+00
HLTF	-1.89E+00	1.81E-36	LOC112580313	-2.97E-01	1.00E+00	LYSMD2	2.21E-01	1.00E+00
CD99L2	-1.67E+00	1.82E-36	ELL	8.69E-02	1.00E+00	IL23A	1.30E-01	1.00E+00
CCDC138	-1.62E+00	1.84E-36	FAM163B	6.43E-01	1.00E+00	UBN2	-1.29E-01	1.00E+00
PDE9A	-5.26E+00	1.85E-36	LOC102396207	3.88E-01	1.00E+00	SPSB1	1.01E-01	1.00E+00
FAM131A	-2.77E+00	1.86E-36	PDIA2	-2.77E-01	1.00E+00	PSMA6	1.07E-01	1.00E+00
FAM193A	1.39E+00	1.86E-36	FMN1	1.20E-01	1.00E+00	LOC102408471	-1.64E-01	1.00E+00
MOXD1	-3.61E+00	1.86E-36	MFSD2A	7.48E-02	1.00E+00	LOC112580815	2.28E-01	1.00E+00
ZFP90	2.37E+00	1.91E-36	ERI1	-1.10E-01	1.00E+00	AXDND1	-1.64E-01	1.00E+00
TRIM37	1.14E+00	1.93E-36	MCOLN2	6.27E-01	1.00E+00	TACC1	1.11E-01	1.00E+00
LOC102393048	-5.70E+00	1.94E-36	EBF3	-9.14E-02	1.00E+00	AS3MT	2.14E-01	1.00E+00
COMMD7	-1.69E+00	1.95E-36	NPW	-4.08E-01	1.00E+00	SCAI	-1.24E-01	1.00E+00
PDE4DIP	1.33E+00	1.95E-36	PDE7B	2.24E-01	1.00E+00	GPR19	-2.17E-01	1.00E+00
TRIM9	2.82E+00	1.96E-36	WRN	1.26E-01	1.00E+00	AREL1	9.31E-02	1.00E+00
NCKAP5	3.84E+00	1.97E-36	FSCN3	-3.70E-01	1.00E+00	PPIP5K2	-1.20E-01	1.00E+00
LOC102403295	5.43E+00	2.00E-36	NDRG1	-4.12E-01	1.00E+00	TRIP12	9.21E-02	1.00E+00
YDJC	2.59E+00	2.05E-36	WDSUB1	-8.24E-02	1.00E+00	CT55	-2.42E-01	1.00E+00
TMEM176B	-6.99E+00	2.09E-36	BHLHE22	-5.45E-01	1.00E+00	LYRM4	-1.24E-01	1.00E+00
RBM7	-2.56E+00	2.14E-36	CCNB3	1.06E-01	1.00E+00	TINAGL1	2.99E-01	1.00E+00
NOL9	1.52E+00	2.16E-36	OSGEPL1	2.03E-01	1.00E+00	TMEM102	-2.02E-01	1.00E+00
LOC102390802	-2.18E+00	2.22E-36	LRRC61	-2.72E-01	1.00E+00	WNT11	-2.04E-01	1.00E+00
SORT1	-1.33E+00	2.24E-36	STK11	-1.14E-01	1.00E+00	ZFAND3	-8.95E-02	1.00E+00
PLPPR3	1.95E+00	2.28E-36	GRID2	-1.09E-01	1.00E+00	TSPAN13	9.28E-02	1.00E+00
LOC102389178	3.72E+00	2.28E-36	TMEM132A	-5.43E-01	1.00E+00	CATSPERG	1.24E-01	1.00E+00
RAB5A	1.55E+00	2.29E-36	TNRC6C	1.14E-01	1.00E+00	LOC112583109	-1.99E-01	1.00E+00
KLF9	-5.11E+00	2.37E-36	DHDDS	7.66E-02	1.00E+00	ZRANB3	-1.03E-01	1.00E+00

EFNA1	-3.60E+00	2.37E-36	MAPRE1	1.06E-01	1.00E+00	RABEPK	-1.31E-01	1.00E+00
HTRA1	-5.70E+00	2.37E-36	LOC112579981	2.73E-01	1.00E+00	CNGA3	2.17E-01	1.00E+00
RPRD1A	-1.24E+00	2.38E-36	MED14	1.30E-01	1.00E+00	KRT1	2.31E-01	1.00E+00
CCDC186	1.86E+00	2.50E-36	C11H15orf41	-9.46E-02	1.00E+00	CD68	-3.41E-01	1.00E+00
PAQR9	-6.22E+00	2.51E-36	SMC4	-9.62E-02	1.00E+00	RARA	-9.50E-02	1.00E+00
CAPN13	7.48E+00	2.57E-36	WDR24	9.07E-02	1.00E+00	LOC102391491	-1.56E-01	1.00E+00
NOG	-6.36E+00	2.61E-36	MAGI3	-1.44E-01	1.00E+00	TOR2A	1.07E-01	1.00E+00
GCDH	-1.53E+00	2.63E-36	MMP17	-2.95E-01	1.00E+00	MRE11	-1.06E-01	1.00E+00
ASB9	-6.94E+00	2.63E-36	NKAIN2	1.38E-01	1.00E+00	TARBP1	-9.40E-02	1.00E+00
LRMP	5.98E+00	2.71E-36	BPNT1	-7.99E-02	1.00E+00	SDHC	1.03E-01	1.00E+00
NOTUM	-4.53E+00	2.73E-36	CERS3	1.32E-01	1.00E+00	LOC102399969	1.63E-01	1.00E+00
TPM2	-6.19E+00	2.74E-36	PLEKHH2	5.86E-01	1.00E+00	MARK1	-9.93E-02	1.00E+00
LOC112581891	4.81E+00	2.75E-36	LOC112580318	-4.72E-01	1.00E+00	PPP1R3D	2.39E-01	1.00E+00
MTDH	-1.23E+00	2.83E-36	DOCK4	-1.06E-01	1.00E+00	LOC112582053	-1.06E-01	1.00E+00
LOC102391841	6.08E+00	2.84E-36	CRTC2	7.91E-02	1.00E+00	OPHN1	1.64E-01	1.00E+00
ADAMTS1	-6.40E+00	2.87E-36	TAF2	1.27E-01	1.00E+00	GABARAP	1.27E-01	1.00E+00
TIMM22	-1.99E+00	2.87E-36	CCDC38	-1.81E-01	1.00E+00	AR	-9.76E-02	1.00E+00
PER1	1.69E+00	2.89E-36	TIMM13	-1.06E-01	1.00E+00	IFT43	1.27E-01	1.00E+00
CSGALNACT1	-2.67E+00	2.90E-36	DZIP1L	1.71E-01	1.00E+00	CD86	4.68E-01	1.00E+00
VPS28	-1.95E+00	2.93E-36	RAB8B	1.19E-01	1.00E+00	RGMB	1.11E-01	1.00E+00
TPH2	6.02E+00	3.02E-36	MLLT6	1.79E-01	1.00E+00	PDPR	-9.68E-02	1.00E+00
GAPDH	-2.99E+00	3.02E-36	PLCG2	1.32E-01	1.00E+00	CD274	-2.56E-01	1.00E+00
TAGLN	-2.99E+00	3.13E-36	C17H4orf46	4.02E-01	1.00E+00	C1H3orf58	1.11E-01	1.00E+00
LOC112582330	-5.93E+00	3.14E-36	PSME3	8.69E-02	1.00E+00	TTC3	-9.53E-02	1.00E+00
SIN3A	1.33E+00	3.18E-36	FKBP2	-1.25E-01	1.00E+00	SEC61A1	-1.08E-01	1.00E+00
RPL4	-3.07E+00	3.19E-36	C8G	5.04E-01	1.00E+00	MAGED1	-9.64E-02	1.00E+00
MMADHC	-1.29E+00	3.23E-36	NDUFS5	-1.04E-01	1.00E+00	AGRN	9.66E-02	1.00E+00
HYKK	2.02E+00	3.34E-36	CDH24	3.01E-01	1.00E+00	MAL	-2.08E-01	1.00E+00
C9H5orf30	-2.00E+00	3.36E-36	KIAA1211	8.39E-02	1.00E+00	VSTM4	1.94E-01	1.00E+00
TMEM86A	2.16E+00	3.37E-36	CLEC4G	-9.83E-02	1.00E+00	SNRPB2	-8.83E-02	1.00E+00
CBR4	-3.39E+00	3.43E-36	C22H18orf25	9.40E-02	1.00E+00	LMNTD2	-1.99E-01	1.00E+00
LOC102411827	1.55E+00	3.63E-36	ENTPD5	1.10E-01	1.00E+00	BCO2	3.15E-01	1.00E+00
EXOC6B	-1.31E+00	3.65E-36	BTG3	4.83E-01	1.00E+00	AP1S2	8.92E-02	1.00E+00

SYNM	-2.50E+00	3.66E-36	REEP5	-3.52E-01	1.00E+00	NLK	-1.16E-01	1.00E+00
PSMB3	-2.15E+00	3.76E-36	CWF19L2	-9.94E-02	1.00E+00	SH3GL2	2.50E-01	1.00E+00
BUD23	1.61E+00	3.81E-36	LIPT1	-1.85E-01	1.00E+00	SLC45A4	-1.32E-01	1.00E+00
TTC37	-1.30E+00	3.96E-36	WFIKKN1	2.51E-01	1.00E+00	PSMD12	1.03E-01	1.00E+00
TMEM240	-5.73E+00	4.05E-36	TREX2	-3.11E-01	1.00E+00	SUCLG1	1.15E-01	1.00E+00
TTC21B	1.15E+00	4.11E-36	MRC2	3.89E-01	1.00E+00	AKIRIN2	-9.90E-02	1.00E+00
LOC112585206	-2.96E+00	4.14E-36	TMEM171	2.39E-01	1.00E+00	TUBB4B	-1.81E-01	1.00E+00
C11H14orf119	1.41E+00	4.17E-36	NUDT7	-3.21E-01	1.00E+00	CCNI	-1.01E-01	1.00E+00
ZC4H2	-2.13E+00	4.25E-36	CRIPT	-8.62E-02	1.00E+00	LOC102393522	-1.55E-01	1.00E+00
ELOVL6	-1.63E+00	4.34E-36	LOC102415336	4.65E-01	1.00E+00	UBIAD1	-1.14E-01	1.00E+00
LOC112582248	5.85E+00	4.43E-36	RHPN1	4.71E-01	1.00E+00	HES1	1.48E-01	1.00E+00
NAA20	1.32E+00	4.43E-36	MOB2	-1.14E-01	1.00E+00	CCNT1	-1.13E-01	1.00E+00
SULF1	-5.81E+00	4.49E-36	RIIAD1	-1.02E-01	1.00E+00	VSIG10	-1.03E-01	1.00E+00
C24H16orf96	-2.78E+00	4.55E-36	FBXO25	-7.02E-02	1.00E+00	SSC5D	-2.16E-01	1.00E+00
PQLC1	-2.62E+00	4.58E-36	LOC112584592	-1.10E-01	1.00E+00	LOC112586211	2.56E-01	1.00E+00
IGSF9B	2.93E+00	4.76E-36	GABPB2	1.42E-01	1.00E+00	LOC102403265	-1.55E-01	1.00E+00
RPL15	-1.80E+00	4.79E-36	MRPL22	-9.59E-02	1.00E+00	ZBTB2	-1.23E-01	1.00E+00
LOC112583701	3.59E+00	4.85E-36	ZC3H12C	-2.48E-01	1.00E+00	EPHB2	9.56E-02	1.00E+00
LOC112585112	-4.63E+00	4.93E-36	C7H4orf17	-1.51E-01	1.00E+00	ANXA7	-1.00E-01	1.00E+00
SORBS3	-1.61E+00	5.01E-36	NSUN2	1.08E-01	1.00E+00	CLEC2L	-2.17E-01	1.00E+00
ARL16	-1.58E+00	5.05E-36	KLHL18	9.48E-02	1.00E+00	EXTL2	-9.99E-02	1.00E+00
LOC102414397	4.98E+00	5.13E-36	PRR5	-1.61E-01	1.00E+00	MAGI1	-1.18E-01	1.00E+00
LOC112580195	5.52E+00	5.19E-36	INIP	-9.22E-02	1.00E+00	STEAP2	1.15E-01	1.00E+00
TLL2	-4.30E+00	5.20E-36	PHEX	-1.54E-01	1.00E+00	PIP5K1C	-9.32E-02	1.00E+00
ZNF414	-1.89E+00	5.20E-36	LUZP1	2.05E-01	1.00E+00	TMIE	2.16E-01	1.00E+00
LOC112579251	-3.17E+00	5.25E-36	FAM126A	1.36E-01	1.00E+00	LOC102400622	2.02E-01	1.00E+00
AGAP2	2.12E+00	5.28E-36	GOLGA7	7.84E-02	1.00E+00	THTPA	1.12E-01	1.00E+00
HMGN5	-4.88E+00	5.35E-36	ATL1	-1.91E-01	1.00E+00	MFAP3L	1.06E-01	1.00E+00
GBE1	-1.82E+00	5.36E-36	LOC102389143	3.85E-01	1.00E+00	GPR155	1.50E-01	1.00E+00
SLC30A2	-7.04E+00	5.39E-36	SPRY1	1.87E-01	1.00E+00	TMX2	-8.89E-02	1.00E+00
LOC112586233	3.37E+00	5.40E-36	WNK2	1.63E-01	1.00E+00	KDM4A	-9.37E-02	1.00E+00
CHEK1	1.64E+00	5.41E-36	AKR1E2	-6.03E-01	1.00E+00	MYO15A	-2.34E-01	1.00E+00
BRD1	1.67E+00	5.63E-36	SGO1	7.59E-02	1.00E+00	DOK4	-1.90E-01	1.00E+00

LOC112581649	4.83E+00	5.67E-36	ABCF2	7.30E-02	1.00E+00	AKNA	-1.69E-01	1.00E+00
AOC1	-9.09E+00	5.67E-36	SHARPIN	9.10E-02	1.00E+00	LOC102407981	2.26E-01	1.00E+00
AHCYL1	-1.11E+00	5.78E-36	CD109	1.53E-01	1.00E+00	TMEM19	-1.73E-01	1.00E+00
DPP8	1.58E+00	5.92E-36	TMEM159	1.07E-01	1.00E+00	WDR4	1.23E-01	1.00E+00
LEF1	2.51E+00	5.93E-36	MRPL17	-1.37E-01	1.00E+00	CBR4	-1.53E-01	1.00E+00
FAM170A	4.64E+00	6.11E-36	TMEM63B	9.12E-02	1.00E+00	DAGLB	-9.05E-02	1.00E+00
PCDHB7	-5.01E+00	6.13E-36	CHAC1	7.13E-02	1.00E+00	B3GALT6	-1.13E-01	1.00E+00
PURA	-2.86E+00	6.29E-36	LOC102392615	-1.60E-01	1.00E+00	DGKH	-1.65E-01	1.00E+00
UHRF1BP1	-1.65E+00	7.03E-36	WDR25	-1.17E-01	1.00E+00	GOPC	-1.16E-01	1.00E+00
RGS12	-1.48E+00	7.13E-36	FAM131A	3.33E-01	1.00E+00	EGR1	-2.42E-01	1.00E+00
GTF2H4	-1.86E+00	7.14E-36	ABL1	1.03E-01	1.00E+00	B4GALT1	-9.77E-02	1.00E+00
C9H19orf66	2.24E+00	7.21E-36	WDR1	-8.15E-02	1.00E+00	CREG1	-1.77E-01	1.00E+00
LOC112585309	-7.23E+00	7.23E-36	CDK2AP2	7.40E-02	1.00E+00	FAM126A	1.56E-01	1.00E+00
NEURL2	-2.55E+00	7.24E-36	NARS2	-1.35E-01	1.00E+00	MLH1	-9.38E-02	1.00E+00
RENBP	-6.63E+00	7.31E-36	IGSF22	-4.52E-01	1.00E+00	RTN4	1.03E-01	1.00E+00
NT5M	-2.20E+00	7.36E-36	TRPV4	2.55E-01	1.00E+00	SRA1	1.12E-01	1.00E+00
LOC112583727	2.90E+00	7.43E-36	LOC102413997	-3.99E-01	1.00E+00	DUSP3	-1.42E-01	1.00E+00
GRSF1	1.14E+00	7.62E-36	ZDHHC23	1.05E-01	1.00E+00	FBXW11	1.29E-01	1.00E+00
LOC112579125	4.52E+00	7.83E-36	HEATR4	-1.95E-01	1.00E+00	METAP2	-8.89E-02	1.00E+00
PAQR6	-4.18E+00	7.90E-36	LOC112581441	3.54E-01	1.00E+00	CC2D1B	9.57E-02	1.00E+00
ADAMTS18	5.92E+00	7.92E-36	LOC112584542	-3.37E-01	1.00E+00	EIF2AK4	1.06E-01	1.00E+00
PLEKHO2	-1.76E+00	8.39E-36	OTUD4	1.20E-01	1.00E+00	ARPC5	9.23E-02	1.00E+00
TOR1B	-1.29E+00	8.54E-36	PACSIN3	3.75E-01	1.00E+00	ZMYM6	-1.18E-01	1.00E+00
CLCN2	-2.29E+00	8.59E-36	RGP1	8.92E-02	1.00E+00	IRX5	1.23E-01	1.00E+00
GALNT2	-1.28E+00	8.63E-36	LOC102400686	-3.69E-01	1.00E+00	TPR	9.62E-02	1.00E+00
HADHA	-1.41E+00	8.72E-36	SPRED2	1.16E-01	1.00E+00	OTUD4	-1.37E-01	1.00E+00
DUSP6	-2.16E+00	9.15E-36	NHP2	-1.23E-01	1.00E+00	C18H19orf47	-1.12E-01	1.00E+00
ADCK1	1.69E+00	9.44E-36	LOC112578733	-3.94E-01	1.00E+00	SPAG8	-1.75E-01	1.00E+00
RNF7	-1.51E+00	9.75E-36	PM20D2	7.67E-01	1.00E+00	IL16	2.93E-01	1.00E+00
CHEK2	-1.72E+00	1.07E-35	WDR4	-1.71E-01	1.00E+00	PPP2R5B	1.01E-01	1.00E+00
ERMAP	2.71E+00	1.09E-35	COLGALT1	2.38E-01	1.00E+00	TWSG1	1.16E-01	1.00E+00
TNIK	-1.87E+00	1.09E-35	ZZZ3	1.07E-01	1.00E+00	DNM2	-9.27E-02	1.00E+00
KLHL31	-5.29E+00	1.18E-35	RAD51	-7.61E-02	1.00E+00	CHST14	-9.71E-02	1.00E+00

INTS4	1.11E+00	1.20E-35	ACOX2	2.99E-01	1.00E+00	PURG	1.29E-01	1.00E+00
PIGV	1.47E+00	1.26E-35	ASL	-3.82E-01	1.00E+00	LOC102394989	1.30E-01	1.00E+00
DNAH11	1.92E+00	1.33E-35	DNAJC24	2.49E-01	1.00E+00	AGFG2	-1.01E-01	1.00E+00
USP25	1.46E+00	1.46E-35	SLC10A3	8.31E-02	1.00E+00	MOCS2	-9.92E-02	1.00E+00
DHH	-5.01E+00	1.49E-35	AKAP1	8.30E-02	1.00E+00	HYDIN	-2.06E-01	1.00E+00
ZNF467	3.04E+00	1.50E-35	NIPSNAP1	-1.39E-01	1.00E+00	TRPM2	4.46E-01	1.00E+00
DDAH1	-5.07E+00	1.54E-35	CTBP2	7.92E-02	1.00E+00	TMEM45B	-2.60E-01	1.00E+00
NME4	-5.28E+00	1.54E-35	XPC	1.19E-01	1.00E+00	C9H19orf24	1.51E-01	1.00E+00
SPATA2	1.35E+00	1.62E-35	LOC102392464	1.01E-01	1.00E+00	CIAO2B	1.11E-01	1.00E+00
GCAT	2.47E+00	1.65E-35	TMEM260	-1.72E-01	1.00E+00	LOC102407917	1.81E-01	1.00E+00
TAF6	-1.31E+00	1.68E-35	SMURF2	7.33E-02	1.00E+00	MSMB	-2.39E-01	1.00E+00
CPB2	4.45E+00	1.69E-35	GRIA1	2.21E-01	1.00E+00	KIAA0513	-1.02E-01	1.00E+00
SMARCAD1	1.58E+00	1.73E-35	ZNF592	1.03E-01	1.00E+00	PET117	-1.34E-01	1.00E+00
ORAI2	-3.05E+00	1.73E-35	LDLR	-3.47E-01	1.00E+00	ARMCX4	1.64E-01	1.00E+00
POPDC3	-8.54E+00	1.74E-35	FITM2	2.58E-01	1.00E+00	RPS19BP1	1.22E-01	1.00E+00
EYA3	1.19E+00	1.77E-35	WDR75	8.62E-02	1.00E+00	URB2	1.39E-01	1.00E+00
WBP1L	-1.48E+00	1.82E-35	LOC102392455	2.40E-01	1.00E+00	THAP9	-1.17E-01	1.00E+00
UNC5CL	3.62E+00	1.86E-35	LOC112585629	-5.28E-01	1.00E+00	LATS1	-2.06E-01	1.00E+00
LOC112578876	5.99E+00	1.98E-35	SCRN1	2.07E-01	1.00E+00	CREB3L2	-1.16E-01	1.00E+00
ATP6AP1	-1.29E+00	2.02E-35	OXCT1	-1.52E-01	1.00E+00	IRAK4	1.31E-01	1.00E+00
CCL22	5.15E+00	2.03E-35	HAUS6	-7.06E-02	1.00E+00	ACOX3	-9.18E-02	1.00E+00
MROH7	3.51E+00	2.09E-35	SYTL2	1.19E-01	1.00E+00	FAM69B	-2.36E-01	1.00E+00
GAR1	1.46E+00	2.21E-35	PRKD1	1.53E-01	1.00E+00	GAA	-9.89E-02	1.00E+00
USP28	1.43E+00	2.39E-35	DNAJB5	-4.26E-01	1.00E+00	RBBP9	-1.34E-01	1.00E+00
SERTAD1	-2.69E+00	2.43E-35	SOAT1	7.54E-02	1.00E+00	SIK3	-8.95E-02	1.00E+00
GAB2	1.73E+00	2.49E-35	SIGLEC11	-1.58E-01	1.00E+00	ZC2HC1A	-1.30E-01	1.00E+00
RAB26	-2.97E+00	2.52E-35	PEX26	1.55E-01	1.00E+00	SGPP1	-1.17E-01	1.00E+00
PRDM4	1.18E+00	2.55E-35	LOC102393439	-2.36E-01	1.00E+00	LRSAM1	-1.05E-01	1.00E+00
TNIP1	-1.42E+00	2.60E-35	NDUFS2	-1.17E-01	1.00E+00	LOC102392550	-1.32E-01	1.00E+00
EAPP	1.33E+00	2.66E-35	MSN	1.53E-01	1.00E+00	NMB	1.87E-01	1.00E+00
SLC4A8	-2.13E+00	2.69E-35	PLA2G4A	-6.51E-01	1.00E+00	GTPBP3	-9.89E-02	1.00E+00
CA8	-2.54E+00	2.75E-35	PXK	1.07E-01	1.00E+00	SSRP1	-1.08E-01	1.00E+00
AKR1E2	-5.29E+00	2.78E-35	MET	2.41E-01	1.00E+00	MAPK14	8.76E-02	1.00E+00
PIMREG	-	2.81E-35	STK40	1.13E-01	1.00E+00	NPHP3	-1.21E-	1.00E+00

	2.59E+00						01	
ID1	-5.50E+00	2.81E-35	SPRY2	1.27E-01	1.00E+00	PRMT3	1.05E-01	1.00E+00
SYVN1	1.20E+00	2.82E-35	RENBP	-6.47E-01	1.00E+00	DNAH9	-1.39E-01	1.00E+00
ADSS	1.53E+00	2.84E-35	RPS19	-1.11E-01	1.00E+00	CRLS1	-9.77E-02	1.00E+00
CHRAC1	-2.02E+00	2.89E-35	ARHGEF37	1.09E-01	1.00E+00	ARRDC1	-1.12E-01	1.00E+00
PLA2G1B	-7.32E+00	2.92E-35	ATP5MG	-1.68E-01	1.00E+00	DHX9	-1.12E-01	1.00E+00
SYNC	-4.67E+00	3.00E-35	POU3F1	2.29E-01	1.00E+00	ABCC9	-8.73E-02	1.00E+00
GATM	-6.14E+00	3.12E-35	LOC112582229	2.14E-01	1.00E+00	TMBIM4	8.53E-02	1.00E+00
ANLN	1.56E+00	3.22E-35	PLA1A	-1.67E-01	1.00E+00	IGF1R	-1.06E-01	1.00E+00
SETX	1.19E+00	3.28E-35	IPO8	-2.52E-01	1.00E+00	SLCO2B1	2.64E-01	1.00E+00
SEC14L1	1.80E+00	3.31E-35	ARHGAP24	-1.06E-01	1.00E+00	PCED1A	-1.29E-01	1.00E+00
TNRC6A	1.96E+00	3.32E-35	LOC102409319	-1.41E-01	1.00E+00	ERCC1	-1.19E-01	1.00E+00
HMBOX1	1.89E+00	3.37E-35	GUCA1C	-2.77E-01	1.00E+00	LOC112586957	-2.41E-01	1.00E+00
PBLD	2.99E+00	3.42E-35	UBN1	1.14E-01	1.00E+00	IL17RA	1.16E-01	1.00E+00
SYNPR	-4.67E+00	3.45E-35	NSMF	4.91E-01	1.00E+00	CORO1B	-1.03E-01	1.00E+00
KLC2	-1.28E+00	3.47E-35	MAP3K1	-3.27E-01	1.00E+00	VAPA	-8.82E-02	1.00E+00
WTAP	-1.23E+00	3.49E-35	ALDH3A2	-1.28E-01	1.00E+00	MBLAC2	-1.66E-01	1.00E+00
ABCG4	5.16E+00	3.52E-35	ADD1	7.87E-02	1.00E+00	HDHD3	1.61E-01	1.00E+00
CFAP54	-2.79E+00	3.54E-35	ENDOV	9.59E-02	1.00E+00	ASXL1	-9.14E-02	1.00E+00
MGAT5	2.29E+00	3.55E-35	LOC102401265	-1.17E-01	1.00E+00	LOC102407694	-1.14E-01	1.00E+00
LOC102389323	3.65E+00	3.62E-35	FMC1	-2.46E-01	1.00E+00	LOC112584448	2.14E-01	1.00E+00
LOC102403813	6.01E+00	3.65E-35	TLCD1	-1.23E-01	1.00E+00	LOC102407763	1.41E-01	1.00E+00
SPINT2	-1.80E+00	3.90E-35	FAM120B	1.25E-01	1.00E+00	PPFIBP2	1.24E-01	1.00E+00
CHCHD7	1.65E+00	4.08E-35	ROMO1	-1.26E-01	1.00E+00	CDK13	-9.97E-02	1.00E+00
FIG4	1.07E+00	4.09E-35	UBE2L3	-8.22E-02	1.00E+00	RFC5	-9.30E-02	1.00E+00
FAM126B	1.85E+00	4.25E-35	FRAT2	1.11E-01	1.00E+00	LOC102403487	-1.66E-01	1.00E+00
LOC102414664	6.84E+00	4.29E-35	IL27RA	-4.84E-01	1.00E+00	TADA1	-1.11E-01	1.00E+00
MAD2L1	1.63E+00	4.43E-35	SLC7A2	1.06E-01	1.00E+00	IL18BP	-1.04E-01	1.00E+00
THUMPD2	-1.64E+00	4.54E-35	PAK3	1.17E-01	1.00E+00	SINHCAF	1.28E-01	1.00E+00
C7H4orf19	5.74E+00	4.67E-35	UBE2J1	-2.60E-01	1.00E+00	PAPSS2	1.08E-01	1.00E+00
TATDN3	-2.15E+00	4.67E-35	PPM1F	-2.53E-01	1.00E+00	UTP25	9.69E-02	1.00E+00
UPF2	1.18E+00	4.73E-35	GRIK5	5.56E-01	1.00E+00	ZCCHC8	-1.16E-01	1.00E+00
KLHL33	-6.03E+00	4.80E-35	KBTBD3	1.26E-01	1.00E+00	DPH7	1.05E-01	1.00E+00

FAM178B	2.77E+00	4.93E-35	ROCK1	1.01E-01	1.00E+00	DDX24	-9.05E-02	1.00E+00
MEX3B	-4.04E+00	4.97E-35	LOC102408254	1.21E-01	1.00E+00	FER1L5	1.52E-01	1.00E+00
C6H1orf109	1.53E+00	5.08E-35	NECTIN3	1.05E-01	1.00E+00	DARS	8.33E-02	1.00E+00
TMEM221	-5.40E+00	5.15E-35	LOC112584601	-5.08E-01	1.00E+00	LOC102400686	1.17E-01	1.00E+00
NEFM	-7.67E+00	5.17E-35	TM4SF20	-1.50E-01	1.00E+00	SMURF1	1.37E-01	1.00E+00
LOC102402191	4.39E+00	5.27E-35	TMEM161B	-1.50E-01	1.00E+00	FZD5	-1.30E-01	1.00E+00
BCL2L13	-1.40E+00	5.30E-35	INSL3	7.38E-01	1.00E+00	INSR	1.21E-01	1.00E+00
BBIP1	1.42E+00	5.31E-35	POLR3B	1.36E-01	1.00E+00	PRR5	-1.22E-01	1.00E+00
KAT2B	-1.73E+00	5.40E-35	PPIL4	-1.15E-01	1.00E+00	TCP11L2	1.40E-01	1.00E+00
HADHB	-1.18E+00	5.59E-35	LOC112581440	4.38E-01	1.00E+00	USP24	9.68E-02	1.00E+00
MFSD11	1.29E+00	5.59E-35	EOGT	-1.98E-01	1.00E+00	FBXL12	-1.25E-01	1.00E+00
CCDC22	1.38E+00	5.63E-35	AK9	3.77E-01	1.00E+00	MRM1	1.11E-01	1.00E+00
RAVER1	-1.30E+00	5.72E-35	HBS1L	-1.32E-01	1.00E+00	LOC112582308	-2.07E-01	1.00E+00
LOC102415279	-3.49E+00	5.84E-35	DNAJC6	1.25E-01	1.00E+00	CHST2	1.38E-01	1.00E+00
CMPK2	-2.68E+00	5.91E-35	ANKRD17	1.22E-01	1.00E+00	NXPH4	1.82E-01	1.00E+00
CBLN1	7.65E+00	5.91E-35	RNF222	-2.74E-01	1.00E+00	RBM23	-8.57E-02	1.00E+00
FAM241A	-3.33E+00	5.95E-35	THEGL	2.09E-01	1.00E+00	FAM229B	-2.01E-01	1.00E+00
GMEB2	-1.20E+00	6.05E-35	LOC102410024	-2.17E-01	1.00E+00	SESN2	9.60E-02	1.00E+00
COL18A1	-7.64E+00	6.17E-35	RPS6KA3	9.54E-02	1.00E+00	PAQR6	-1.92E-01	1.00E+00
LY6G5B	-4.04E+00	6.23E-35	MAP4K2	1.38E-01	1.00E+00	NFATC4	-1.06E-01	1.00E+00
MRPS22	-1.32E+00	6.37E-35	SERINC3	8.86E-02	1.00E+00	TCAIM	-1.09E-01	1.00E+00
SCML1	-3.39E+00	6.54E-35	ABCA2	1.36E-01	1.00E+00	NEK7	-1.12E-01	1.00E+00
NUAK1	-4.79E+00	6.57E-35	LOC112584177	-3.17E-01	1.00E+00	BAMBI	-1.72E-01	1.00E+00
FRYL	1.64E+00	6.78E-35	FOXR1	9.80E-02	1.00E+00	CCDC127	1.05E-01	1.00E+00
ZDHHC15	-2.99E+00	7.05E-35	KLHL24	-2.94E-01	1.00E+00	TMEM14A	8.62E-02	1.00E+00
LOC102389468	1.77E+00	7.36E-35	TRIP4	7.40E-02	1.00E+00	STEAP3	1.54E-01	1.00E+00
MAEA	1.17E+00	7.41E-35	NGRN	-1.89E-01	1.00E+00	LCP1	3.05E-01	1.00E+00
LOC102396893	-7.06E+00	7.50E-35	TMEM147	-1.05E-01	1.00E+00	MAMDC4	-2.13E-01	1.00E+00
GSC2	3.60E+00	7.56E-35	BCAR3	1.13E-01	1.00E+00	SUMF1	-8.47E-02	1.00E+00
LOC102392630	-6.46E+00	7.57E-35	TYSND1	1.51E-01	1.00E+00	WBP11	-8.64E-02	1.00E+00
LOC102395807	-1.36E+00	7.58E-35	LOC102400587	1.64E-01	1.00E+00	SCLY	2.32E-01	1.00E+00
BCAR3	-1.40E+00	7.90E-35	SREK1IP1	1.24E-01	1.00E+00	AEN	1.11E-01	1.00E+00
FRS2	-1.63E+00	8.04E-35	CASKIN2	1.10E-01	1.00E+00	TNFAIP2	-3.58E-01	1.00E+00
EXOC4	1.24E+00	8.05E-35	PDRG1	9.99E-02	1.00E+00	EXOSC7	-1.03E-	1.00E+00

							01	
RABL3	1.20E+00	8.09E-35	ATP23	-1.97E-01	1.00E+00	RAD23B	-9.08E-02	1.00E+00
MYB	-5.51E+00	8.09E-35	ATP6V1B2	7.65E-02	1.00E+00	ALAD	-1.35E-01	1.00E+00
PUS7	-2.64E+00	8.13E-35	CCNYL1	1.01E-01	1.00E+00	ANKLE2	-9.86E-02	1.00E+00
PDK4	-5.86E+00	8.21E-35	GPR137B	-1.03E-01	1.00E+00	SPATA6	1.46E-01	1.00E+00
ZSWIM3	2.60E+00	8.51E-35	KPNA2	-8.17E-02	1.00E+00	SATB1	1.23E-01	1.00E+00
DHX30	-1.37E+00	8.52E-35	TLX3	-3.89E-01	1.00E+00	LOC102389068	1.32E-01	1.00E+00
HAT1	1.46E+00	8.61E-35	LYPLA2	9.80E-02	1.00E+00	APBB3	-1.24E-01	1.00E+00
EIF2S3	-1.47E+00	8.79E-35	POU3F2	1.87E-01	1.00E+00	TMED8	9.67E-02	1.00E+00
ZBTB12	-2.05E+00	8.82E-35	CFAP298	-7.64E-02	1.00E+00	TAF7L	1.93E-01	1.00E+00
C12H2orf68	-2.69E+00	9.25E-35	CEP170B	9.52E-02	1.00E+00	SAMD1	1.07E-01	1.00E+00
LOC112586228	5.10E+00	9.25E-35	ELF2	2.44E-01	1.00E+00	LOC102397700	-1.44E-01	1.00E+00
ECHDC3	-4.62E+00	9.50E-35	CKAP5	1.13E-01	1.00E+00	VPS26A	-1.93E-01	1.00E+00
EPCAM	7.79E+00	9.68E-35	NAALAD2	3.34E-01	1.00E+00	TGIF2	-9.01E-02	1.00E+00
EDA	1.54E+00	9.70E-35	INHA	-3.98E-01	1.00E+00	NOTCH1	-2.70E-01	1.00E+00
FREM2	-6.47E+00	1.04E-34	HOXD8	-1.20E-01	1.00E+00	ODR4	9.31E-02	1.00E+00
LOC112586215	-3.39E+00	1.06E-34	SLC35F1	-2.95E-01	1.00E+00	ASB6	9.50E-02	1.00E+00
PTPN23	-1.63E+00	1.07E-34	NDUFAB1	-1.03E-01	1.00E+00	LOC102392047	-1.19E-01	1.00E+00
CPEB2	-3.32E+00	1.08E-34	CCDC173	1.58E-01	1.00E+00	COQ9	-1.08E-01	1.00E+00
YRDC	-3.07E+00	1.08E-34	RTF1	-8.91E-02	1.00E+00	DCAF10	1.05E-01	1.00E+00
CBX2	1.71E+00	1.10E-34	PNKP	-9.18E-02	1.00E+00	SOD2	-8.68E-02	1.00E+00
MOB3C	-2.71E+00	1.13E-34	FOS	-5.77E-01	1.00E+00	MFNG	3.02E-01	1.00E+00
SCARB1	-5.38E+00	1.14E-34	EIF1B	-8.38E-02	1.00E+00	ZNF286A	1.01E-01	1.00E+00
SIGMAR1	-2.12E+00	1.15E-34	C5H1orf167	2.67E-01	1.00E+00	KHSRP	-9.48E-02	1.00E+00
LRRC7	-4.73E+00	1.17E-34	TCEANC2	1.08E-01	1.00E+00	FANCE	-1.19E-01	1.00E+00
AFG3L2	1.23E+00	1.22E-34	TSC1	1.11E-01	1.00E+00	LOC102396291	2.10E-01	1.00E+00
TST	-3.24E+00	1.22E-34	GTF2H5	-1.20E-01	1.00E+00	STRN4	-8.63E-02	1.00E+00
GPR3	3.99E+00	1.23E-34	SEC23A	1.51E-01	1.00E+00	INTS3	-8.44E-02	1.00E+00
LOC112586985	5.13E+00	1.23E-34	TMUB2	-3.42E-01	1.00E+00	SNX22	-2.17E-01	1.00E+00
RASGRP1	2.42E+00	1.24E-34	SRP14	-1.27E-01	1.00E+00	SAYSD1	1.91E-01	1.00E+00
HUWE1	-1.97E+00	1.26E-34	FZD8	-4.49E-01	1.00E+00	WNT9B	-1.90E-01	1.00E+00
LUC7L3	-1.40E+00	1.26E-34	LOC102409360	8.29E-02	1.00E+00	SALL2	9.59E-02	1.00E+00
HARBI1	1.42E+00	1.28E-34	USP14	8.65E-02	1.00E+00	CD247	1.19E-01	1.00E+00

PC	-1.76E+00	1.29E-34	ITGA9	-1.17E-01	1.00E+00	PTPMT1	-1.66E-01	1.00E+00
MPPE1	-2.37E+00	1.29E-34	DHODH	-1.38E-01	1.00E+00	ADCY4	-2.65E-01	1.00E+00
BMI1	-2.60E+00	1.30E-34	PPID	-1.56E-01	1.00E+00	ZBTB39	-1.50E-01	1.00E+00
NGRN	-2.11E+00	1.30E-34	SORBS2	-2.07E-01	1.00E+00	PXMP2	-1.44E-01	1.00E+00
STAMBPL1	-6.07E+00	1.34E-34	PRIM1	1.42E-01	1.00E+00	ENPP6	-2.83E-01	1.00E+00
LOC102394692	-4.07E+00	1.34E-34	LOC102407015	-2.19E-01	1.00E+00	ACBD4	-1.38E-01	1.00E+00
RASA3	-3.23E+00	1.35E-34	NAA38	-1.20E-01	1.00E+00	HUWE1	-1.21E-01	1.00E+00
NDNF	-6.59E+00	1.38E-34	NOXA1	5.03E-01	1.00E+00	LOC102398506	-1.96E-01	1.00E+00
TEAD4	-4.02E+00	1.43E-34	ATP6V0A1	-1.28E-01	1.00E+00	COP1	8.52E-02	1.00E+00
MFAP1	1.11E+00	1.57E-34	MORC2	8.33E-02	1.00E+00	LOC102401360	2.11E-01	1.00E+00
ARPP21	6.31E+00	1.61E-34	LOC102389096	3.09E-01	1.00E+00	KIF22	-1.37E-01	1.00E+00
RNLS	-2.24E+00	1.67E-34	MTF2	9.79E-02	1.00E+00	ZNF592	-8.51E-02	1.00E+00
PDLIM7	-1.85E+00	1.71E-34	EIF3M	-8.41E-02	1.00E+00	LOC102409999	3.74E-01	1.00E+00
SRGAP3	3.14E+00	1.71E-34	FAM187A	3.76E-01	1.00E+00	RBM34	1.15E-01	1.00E+00
POU1F1	6.26E+00	1.71E-34	C1QL4	5.10E-01	1.00E+00	CTSC	2.96E-01	1.00E+00
CD2BP2	-1.84E+00	1.77E-34	CLASRP	9.24E-02	1.00E+00	WDR17	-1.66E-01	1.00E+00
ASAH1	-1.72E+00	1.84E-34	LOC102414107	-3.40E-01	1.00E+00	MED12	-1.06E-01	1.00E+00
LOC102416503	-6.93E+00	1.85E-34	CARD10	9.37E-02	1.00E+00	NUP50	-8.35E-02	1.00E+00
TRMO	1.74E+00	1.89E-34	CBFA2T3	-5.40E-01	1.00E+00	TRAK2	1.21E-01	1.00E+00
CCDC116	3.43E+00	1.92E-34	ERP29	-1.10E-01	1.00E+00	HIBADH	-9.01E-02	1.00E+00
LOC112581168	6.20E+00	1.92E-34	CDC26	-1.23E-01	1.00E+00	THNSL1	1.61E-01	1.00E+00
ITPRIPL2	-3.33E+00	1.97E-34	AKIRIN2	7.61E-02	1.00E+00	GOLPH3	-1.12E-01	1.00E+00
DGKZ	1.29E+00	2.02E-34	TDRD10	-9.30E-02	1.00E+00	LOC102412968	3.09E-01	1.00E+00
DNAJC9	1.56E+00	2.04E-34	LOC102390617	2.15E-01	1.00E+00	LOC102414434	2.21E-01	1.00E+00
ASNSD1	1.41E+00	2.06E-34	PAIP1	1.00E-01	1.00E+00	SESN3	-1.26E-01	1.00E+00
RBM43	-5.78E+00	2.15E-34	STOM	-1.04E-01	1.00E+00	MOSPD2	1.27E-01	1.00E+00
NRBP1	-1.17E+00	2.22E-34	RBM5	-8.26E-02	1.00E+00	RNASEH2A	-1.53E-01	1.00E+00
ARL6IP4	-2.20E+00	2.23E-34	LOC102411789	-2.08E-01	1.00E+00	ACACA	-9.14E-02	1.00E+00
GGT5	5.05E+00	2.25E-34	USP12	-2.10E-01	1.00E+00	MYO1B	-1.17E-01	1.00E+00
SIRT3	1.82E+00	2.32E-34	LOC102403153	1.16E-01	1.00E+00	METTL14	-9.28E-02	1.00E+00
TAF1A	1.82E+00	2.32E-34	MIER3	1.36E-01	1.00E+00	LOC102409836	-2.37E-01	1.00E+00
CBX7	-2.51E+00	2.35E-34	TEX2	9.64E-02	1.00E+00	SEMA4B	-8.82E-02	1.00E+00
CRIPT	1.33E+00	2.38E-34	LARP1	1.13E-01	1.00E+00	BORCS6	1.35E-01	1.00E+00
S1PR5	-5.77E+00	2.40E-34	LOC112585002	-3.49E-01	1.00E+00	C15H8orf76	-1.04E-01	1.00E+00

ZBTB43	3.07E+00	2.48E-34	PQBP1	-1.02E-01	1.00E+00	PFDN1	1.23E-01	1.00E+00
LOC102410588	-6.52E+00	2.51E-34	TFIP11	7.47E-02	1.00E+00	AASDH	-1.10E-01	1.00E+00
C14H20orf96	-4.30E+00	2.59E-34	DAAM2	1.34E-01	1.00E+00	MRPS27	-8.47E-02	1.00E+00
PASK	-1.55E+00	2.66E-34	POLE3	-9.64E-02	1.00E+00	G3BP1	-9.95E-02	1.00E+00
LOC112581879	-3.56E+00	2.70E-34	SMPD1	-3.72E-01	1.00E+00	CETN2	-1.03E-01	1.00E+00
TAF13	-1.82E+00	2.82E-34	CEBPA	-4.24E-01	1.00E+00	JRK	1.04E-01	1.00E+00
AACS	1.36E+00	2.84E-34	LOC102389990	-4.19E-01	1.00E+00	OSBPL8	-9.95E-02	1.00E+00
SYNJ2BP	1.27E+00	2.90E-34	RAD9A	1.02E-01	1.00E+00	MRPS21	1.12E-01	1.00E+00
SMIM24	4.70E+00	3.06E-34	PSMC4	1.12E-01	1.00E+00	PARP14	-1.27E-01	1.00E+00
LRRC42	1.14E+00	3.16E-34	SCYL1	-7.93E-02	1.00E+00	PPIE	1.34E-01	1.00E+00
LOC102416510	-9.77E+00	3.19E-34	NUBPL	1.32E-01	1.00E+00	TRIM45	-1.08E-01	1.00E+00
PHYHD1	-3.26E+00	3.27E-34	SOBP	-1.89E-01	1.00E+00	EPHA7	2.53E-01	1.00E+00
GCC2	1.74E+00	3.35E-34	BCKDK	1.51E-01	1.00E+00	NSUN2	8.82E-02	1.00E+00
PHGDH	-6.89E+00	3.35E-34	COPS7A	7.86E-02	1.00E+00	FLNA	1.29E-01	1.00E+00
GLOD4	1.38E+00	3.35E-34	PARP1	8.07E-02	1.00E+00	SELENOW	1.34E-01	1.00E+00
C1H3orf70	1.60E+00	3.40E-34	SH3YL1	-1.19E-01	1.00E+00	DNALI1	-2.20E-01	1.00E+00
LOC102410303	3.22E+00	3.60E-34	LOC112579707	-1.09E-01	1.00E+00	KPNA5	-2.27E-01	1.00E+00
LOC112586860	-8.02E+00	3.71E-34	FGF18	-2.64E-01	1.00E+00	NOTCH2	-1.22E-01	1.00E+00
MTMR14	1.25E+00	3.77E-34	SFT2D1	-1.13E-01	1.00E+00	ZFYVE19	-1.36E-01	1.00E+00
CCDC127	1.28E+00	3.88E-34	LOC112578240	4.40E-01	1.00E+00	OCRL	-9.37E-02	1.00E+00
CD59	1.57E+00	3.94E-34	LOC102409942	1.60E-01	1.00E+00	PRMT6	8.94E-02	1.00E+00
ALG6	1.72E+00	3.97E-34	FOXK1	1.64E-01	1.00E+00	GAB2	-1.04E-01	1.00E+00
PPP1CA	-1.57E+00	3.98E-34	PLAA	8.13E-02	1.00E+00	TACR3	2.36E-01	1.00E+00
OVCH2	4.07E+00	3.98E-34	LOC102410603	-1.10E-01	1.00E+00	DNAJC2	-8.84E-02	1.00E+00
LOC102415329	5.93E+00	4.10E-34	TCF4	1.13E-01	1.00E+00	TSKU	1.05E-01	1.00E+00
RTCB	1.01E+00	4.11E-34	GPR68	-2.32E-01	1.00E+00	DYNC2H1	9.94E-02	1.00E+00
TRAPPC10	-1.72E+00	4.12E-34	SHMT2	9.48E-02	1.00E+00	POLR1A	8.79E-02	1.00E+00
LAMTOR3	1.10E+00	4.13E-34	BICD2	1.34E-01	1.00E+00	AZI2	-7.98E-02	1.00E+00
SNX10	-2.41E+00	4.16E-34	IKBKB	1.07E-01	1.00E+00	MB21D2	1.07E-01	1.00E+00
SOBP	-1.93E+00	4.24E-34	FRZB	8.97E-02	1.00E+00	LOC102392879	-1.17E-01	1.00E+00
NFXL1	1.74E+00	4.41E-34	FBXL3	1.12E-01	1.00E+00	CSNK1G2	1.03E-01	1.00E+00
LOC102394020	2.46E+00	4.69E-34	CPNE8	6.95E-01	1.00E+00	SEC24D	-1.16E-01	1.00E+00
GMPPB	-2.36E+00	4.75E-34	DOCK5	1.22E-01	1.00E+00	NSRP1	-8.82E-02	1.00E+00
TMEM208	-2.05E+00	4.85E-34	MAST2	9.54E-02	1.00E+00	KLK11	2.73E-01	1.00E+00

BAG2	1.42E+00	4.88E-34	KRT72	5.54E-01	1.00E+00	LOC102392001	-1.18E-01	1.00E+00
FIGNL1	1.43E+00	4.97E-34	LOC102408106	-2.19E-01	1.00E+00	NUBP1	-1.14E-01	1.00E+00
DDX5	-1.80E+00	5.02E-34	ANGPT4	-3.41E-01	1.00E+00	RNF114	-8.31E-02	1.00E+00
RC3H1	-1.71E+00	5.17E-34	MKNK1	8.19E-02	1.00E+00	LOC102410885	-1.86E-01	1.00E+00
LOC102411787	-4.94E+00	5.19E-34	NME6	-1.99E-01	1.00E+00	NR1D2	1.35E-01	1.00E+00
MRPS30	1.50E+00	5.19E-34	PLS3	-3.58E-01	1.00E+00	HEY2	-1.22E-01	1.00E+00
SLC44A5	2.87E+00	5.20E-34	TRABD	-1.07E-01	1.00E+00	PCGF2	-8.34E-02	1.00E+00
ADD2	3.21E+00	5.26E-34	NMU	5.28E-01	1.00E+00	LOC112584435	-1.95E-01	1.00E+00
VIM	-6.58E+00	5.29E-34	CDH7	1.10E-01	1.00E+00	GNL3	-9.29E-02	1.00E+00
TRPS1	-1.76E+00	5.47E-34	MCEE	-3.27E-01	1.00E+00	TRIQK	-1.05E-01	1.00E+00
MAP3K7	-1.51E+00	5.53E-34	CASQ2	3.75E-01	1.00E+00	POLL	-1.03E-01	1.00E+00
ATF1	-1.61E+00	5.56E-34	MEX3D	-1.89E-01	1.00E+00	LOC112585799	1.23E-01	1.00E+00
LOC112578269	-4.26E+00	5.57E-34	LOC102407589	3.04E-01	1.00E+00	NEDD1	-1.07E-01	1.00E+00
IER2	-6.17E+00	5.64E-34	TEX261	1.11E-01	1.00E+00	CASQ1	1.62E-01	1.00E+00
ISG20	-6.79E+00	5.92E-34	G6PD	-9.63E-02	1.00E+00	NCDN	8.46E-02	1.00E+00
TFAP4	-2.90E+00	5.92E-34	KREMEN1	1.02E-01	1.00E+00	NOL12	1.03E-01	1.00E+00
PLK1	1.92E+00	5.95E-34	TDP1	7.58E-02	1.00E+00	COMMD7	1.07E-01	1.00E+00
LOC102405614	2.50E+00	5.98E-34	MAIP1	-1.10E-01	1.00E+00	PCDH11X	1.95E-01	1.00E+00
METTL1	-2.54E+00	5.98E-34	LOC102410470	2.74E-01	1.00E+00	COLGALT2	-1.38E-01	1.00E+00
COL6A1	-6.68E+00	6.00E-34	TIAL1	-8.51E-02	1.00E+00	LOC102412461	-9.12E-02	1.00E+00
ARSI	-3.24E+00	6.51E-34	EFCAB3	3.72E-01	1.00E+00	NAPG	-8.37E-02	1.00E+00
TDRD6	2.25E+00	6.62E-34	LOC102396919	-1.50E-01	1.00E+00	GRHPR	-1.17E-01	1.00E+00
MMAA	1.57E+00	6.74E-34	MBD5	-1.33E-01	1.00E+00	PDRG1	-1.13E-01	1.00E+00
GGT6	5.13E+00	6.80E-34	SORCS1	-1.05E-01	1.00E+00	MTHFD2L	1.23E-01	1.00E+00
VIP	8.23E+00	6.87E-34	FBL	-7.90E-02	1.00E+00	RETSAT	-1.57E-01	1.00E+00
STRN4	-1.37E+00	7.11E-34	NOL3	-1.40E-01	1.00E+00	APELA	-2.25E-01	1.00E+00
KDM1B	2.76E+00	7.65E-34	E2F4	6.72E-02	1.00E+00	VAMP1	-1.17E-01	1.00E+00
RAB33B	-3.32E+00	7.65E-34	STS	3.52E-01	1.00E+00	LOC102407908	-2.24E-01	1.00E+00
TBC1D19	-2.25E+00	7.99E-34	SF3A2	8.09E-02	1.00E+00	AURKB	-1.37E-01	1.00E+00
SUGP2	-1.58E+00	8.16E-34	G6PC3	1.92E-01	1.00E+00	ADNP	-1.16E-01	1.00E+00
LOC112580666	1.47E+00	8.23E-34	FAM207A	-1.72E-01	1.00E+00	MED13L	-1.31E-01	1.00E+00
EDEM2	1.25E+00	8.35E-34	SMIM26	-1.64E-01	1.00E+00	XRCC1	-1.12E-01	1.00E+00
CTSO	-	8.44E-34	FAM107B	8.05E-02	1.00E+00	LOC102393823	1.73E-01	1.00E+00

	1.74E+00							
LOC112586495	2.52E+00	8.54E-34	CLINT1	1.06E-01	1.00E+00	COPS7B	-8.47E-02	1.00E+00
CHCHD1	-2.43E+00	8.68E-34	GPI	8.17E-02	1.00E+00	FTO	-8.03E-02	1.00E+00
INHBA	-6.25E+00	8.71E-34	OLAH	1.08E-01	1.00E+00	MRPL10	-9.69E-02	1.00E+00
LIN7C	-1.88E+00	8.93E-34	EHD3	3.72E-01	1.00E+00	BCAS2	-1.02E-01	1.00E+00
BCL2L12	1.39E+00	9.10E-34	BNIP2	-1.40E-01	1.00E+00	LOC102397082	-1.17E-01	1.00E+00
LOC112581576	-4.76E+00	9.21E-34	RNF122	3.49E-01	1.00E+00	FIGN	1.39E-01	1.00E+00
FOXL2	-6.07E+00	9.21E-34	CRTC3	9.86E-02	1.00E+00	LOC112582986	-1.97E-01	1.00E+00
LOC102400568	-2.25E+00	9.26E-34	OGDH	8.66E-02	1.00E+00	IRF2BP1	9.37E-02	1.00E+00
GJA5	-4.62E+00	9.28E-34	ADAM23	-4.27E-01	1.00E+00	ENTPD5	-1.18E-01	1.00E+00
OGA	-1.26E+00	9.35E-34	VCPKMT	2.21E-01	1.00E+00	MCM6	-8.56E-02	1.00E+00
FOXRED2	2.10E+00	9.41E-34	WDR44	1.92E-01	1.00E+00	DNAJB6	8.23E-02	1.00E+00
SPG11	-1.40E+00	9.68E-34	PTK7	1.67E-01	1.00E+00	TRIM23	-1.44E-01	1.00E+00
LOC102391231	-6.21E+00	9.83E-34	AMIGO1	9.68E-02	1.00E+00	SLC39A2	1.17E-01	1.00E+00
MYPOP	-2.83E+00	9.85E-34	CCDC66	-8.72E-02	1.00E+00	MPIG6B	-1.78E-01	1.00E+00
ZBTB8B	3.18E+00	9.95E-34	TBXAS1	-1.53E-01	1.00E+00	CDC16	8.54E-02	1.00E+00
DPP6	-2.18E+00	1.01E-33	TMEM17	-3.38E-01	1.00E+00	LRRC74B	-2.46E-01	1.00E+00
VPS53	1.28E+00	1.04E-33	LETM1	8.32E-02	1.00E+00	RPAP1	-8.18E-02	1.00E+00
SUMF2	-3.97E+00	1.04E-33	SUGP2	1.11E-01	1.00E+00	MYMK	2.12E-01	1.00E+00
ATP12A	4.79E+00	1.07E-33	RAI1	9.69E-02	1.00E+00	LNPK	-1.22E-01	1.00E+00
PALM	-1.38E+00	1.07E-33	SDHAF2	-1.22E-01	1.00E+00	NANS	1.50E-01	1.00E+00
RSAD2	-5.35E+00	1.09E-33	CEP152	1.33E-01	1.00E+00	ZKSCAN5	-9.31E-02	1.00E+00
SEC22C	1.80E+00	1.09E-33	OPCML	2.14E-01	1.00E+00	LOC102389267	2.13E-01	1.00E+00
DAPL1	3.36E+00	1.13E-33	ARL2	-1.12E-01	1.00E+00	SURF2	1.12E-01	1.00E+00
MAGEE2	-8.04E+00	1.15E-33	LOC112582123	4.47E-01	1.00E+00	MVD	-1.28E-01	1.00E+00
SKA2	-1.97E+00	1.16E-33	PRDM10	2.94E-01	1.00E+00	SLC46A1	1.13E-01	1.00E+00
LOC102392615	-1.59E+00	1.16E-33	COG2	-6.92E-02	1.00E+00	CEP68	-9.08E-02	1.00E+00
PDGFRL	-3.19E+00	1.19E-33	L3MBTL2	1.22E-01	1.00E+00	LOC102413919	1.93E-01	1.00E+00
SINHCAF	-1.83E+00	1.19E-33	SNRPB2	-7.91E-02	1.00E+00	NARS2	1.05E-01	1.00E+00
LOC102403202	2.79E+00	1.22E-33	FNTB	-1.10E-01	1.00E+00	LOC102397167	2.86E-01	1.00E+00
TNFRSF6B	-3.29E+00	1.23E-33	LOC102390463	3.73E-01	1.00E+00	TMEM256	1.18E-01	1.00E+00
ATF3	-5.11E+00	1.24E-33	SSU72	-1.10E-01	1.00E+00	PPP1R3C	2.09E-01	1.00E+00
LOC112582921	3.52E+00	1.26E-33	PRR14L	1.96E-01	1.00E+00	CALD1	1.34E-01	1.00E+00
BRD2	-	1.27E-33	ERLEC1	7.08E-02	1.00E+00	DAAM1	-1.37E-	1.00E+00

	1.64E+00						01	
LOC102390349	-4.79E+00	1.28E-33	TYMS	-8.23E-02	1.00E+00	LOC102408254	1.16E-01	1.00E+00
LOC112579941	2.52E+00	1.35E-33	RAB3C	8.36E-02	1.00E+00	LOC112583719	2.06E-01	1.00E+00
COL28A1	5.02E+00	1.43E-33	WDR37	1.37E-01	1.00E+00	ALDH3B1	1.95E-01	1.00E+00
TMEM200A	-5.71E+00	1.43E-33	CD82	3.44E-01	1.00E+00	LOC102395153	1.26E-01	1.00E+00
LOC102406790	5.00E+00	1.48E-33	RAD54L	-7.92E-02	1.00E+00	FXYD2	-2.24E-01	1.00E+00
TTYH2	-3.82E+00	1.64E-33	TMEM102	3.99E-01	1.00E+00	GPX6	-2.46E-01	1.00E+00
CLIC2	-6.22E+00	1.65E-33	TBC1D9	4.35E-01	1.00E+00	REPS2	1.68E-01	1.00E+00
MYO7A	4.04E+00	1.68E-33	PBDC1	-9.19E-02	1.00E+00	AIDA	8.94E-02	1.00E+00
TMEM79	-1.40E+00	1.70E-33	MTSS1L	1.00E-01	1.00E+00	NRXN3	1.05E-01	1.00E+00
AOAH	5.72E+00	1.71E-33	LOC112581723	4.47E-01	1.00E+00	LOC112584566	-1.94E-01	1.00E+00
TBC1D9	-4.96E+00	1.71E-33	TWF2	-1.60E-01	1.00E+00	GRN	-1.10E-01	1.00E+00
DOCK5	1.57E+00	1.72E-33	SNRNP25	-1.07E-01	1.00E+00	XPNPEP3	-1.05E-01	1.00E+00
SAMD14	2.52E+00	1.76E-33	SNTA1	2.34E-01	1.00E+00	RELT	-1.34E-01	1.00E+00
GPR61	4.21E+00	1.77E-33	MSRB2	2.72E-01	1.00E+00	CAND2	1.03E-01	1.00E+00
LOC102406545	1.55E+00	1.83E-33	SH3GL1	7.17E-02	1.00E+00	TLE2	-9.72E-02	1.00E+00
PIGN	1.07E+00	1.83E-33	PTF1A	-4.81E-01	1.00E+00	R3HDM2	-1.31E-01	1.00E+00
CCSER1	4.65E+00	1.83E-33	CATSPERG	3.87E-01	1.00E+00	FSD2	-1.89E-01	1.00E+00
TBC1D24	1.24E+00	1.86E-33	TBC1D2	-1.14E-01	1.00E+00	ITGB1BP1	9.26E-02	1.00E+00
WDR83OS	-1.95E+00	1.90E-33	MAPK8IP2	9.61E-02	1.00E+00	OLFML2B	1.19E-01	1.00E+00
MYEF2	-3.16E+00	1.93E-33	PGLS	-1.57E-01	1.00E+00	CFAP300	1.30E-01	1.00E+00
SLC25A38	1.20E+00	2.00E-33	ZNF248	1.15E-01	1.00E+00	SETD9	-1.76E-01	1.00E+00
KIF26B	4.61E+00	2.01E-33	LOC112582248	3.12E-01	1.00E+00	INTS14	-9.55E-02	1.00E+00
LHFPL3	5.61E+00	2.03E-33	ST13	-7.86E-02	1.00E+00	PLPP1	9.29E-02	1.00E+00
IL4I1	-2.48E+00	2.05E-33	SAMD15	2.14E-01	1.00E+00	KATNA1	-8.98E-02	1.00E+00
COMMD10	-1.53E+00	2.08E-33	LOC102398843	-2.23E-01	1.00E+00	ACOT8	1.16E-01	1.00E+00
ABHD11	-2.13E+00	2.13E-33	LOC112577686	-2.31E-01	1.00E+00	CLTC	1.07E-01	1.00E+00
RRAGD	1.68E+00	2.18E-33	SOX6	-2.59E-01	1.00E+00	SUGCT	1.34E-01	1.00E+00
LOC102399541	3.07E+00	2.25E-33	FRY	9.42E-02	1.00E+00	MAP3K5	-1.21E-01	1.00E+00
LOC112581531	-8.22E+00	2.26E-33	LOC102390363	6.90E-02	1.00E+00	C17H12orf65	1.32E-01	1.00E+00
EXOC3L4	4.32E+00	2.29E-33	NOS1	-1.22E-01	1.00E+00	ETHE1	1.08E-01	1.00E+00
SLC18B1	-4.40E+00	2.30E-33	HEATR6	9.75E-02	1.00E+00	EPAS1	1.35E-01	1.00E+00
FKBP2	-2.08E+00	2.31E-33	SSRP1	7.01E-02	1.00E+00	FAM169A	-9.13E-02	1.00E+00
PSTPIP1	4.49E+00	2.34E-33	PHF19	-1.27E-	1.00E+00	AVL9	1.26E-01	1.00E+00

				01				
PALLD	-1.14E+00	2.46E-33	CEP76	1.95E-01	1.00E+00	STK38	1.06E-01	1.00E+00
LOC102396564	-2.23E+00	2.46E-33	ZDHHC18	8.06E-02	1.00E+00	DCAF17	-1.31E-01	1.00E+00
USP39	-1.25E+00	2.50E-33	DCTN2	-1.47E-01	1.00E+00	IL1RAP	-1.24E-01	1.00E+00
SEC24A	-1.77E+00	2.53E-33	DOP1A	-1.37E-01	1.00E+00	DZIP1L	-1.55E-01	1.00E+00
PLK4	-1.54E+00	2.53E-33	LOC102399077	3.12E-01	1.00E+00	TOPBP1	-9.25E-02	1.00E+00
NSL1	1.76E+00	2.57E-33	DTX1	-1.66E-01	1.00E+00	PAK4	-9.65E-02	1.00E+00
LOC112583816	-5.60E+00	2.57E-33	EPHB6	3.58E-01	1.00E+00	LOC102402539	2.87E-01	1.00E+00
LOC102391952	-3.29E+00	2.59E-33	SLC6A16	-4.91E-01	1.00E+00	LOC102414052	1.23E-01	1.00E+00
LOC102406344	5.97E+00	2.65E-33	EIF4G2	8.12E-02	1.00E+00	LOC102390188	1.59E-01	1.00E+00
TFIP11	1.18E+00	2.77E-33	FRMD5	7.51E-02	1.00E+00	ZNF684	2.01E-01	1.00E+00
USP6NL	1.67E+00	2.83E-33	PCSK4	-3.77E-01	1.00E+00	SIRT4	-1.22E-01	1.00E+00
UBR3	-1.59E+00	3.01E-33	SPATS1	1.97E-01	1.00E+00	BIRC3	-1.29E-01	1.00E+00
PDGFRA	-6.09E+00	3.19E-33	CCNJ	1.65E-01	1.00E+00	FKBP11	-1.34E-01	1.00E+00
IPO11	-1.33E+00	3.22E-33	PPARGC1B	2.23E-01	1.00E+00	GDF1	-2.20E-01	1.00E+00
RBM15B	-1.35E+00	3.25E-33	PARVB	3.01E-01	1.00E+00	LOC102392561	-1.56E-01	1.00E+00
LOC102404784	-6.00E+00	3.27E-33	MAP2K4	7.70E-02	1.00E+00	LOC112579607	-1.11E-01	1.00E+00
UBXN2A	-2.60E+00	3.51E-33	ZNF385B	-1.29E-01	1.00E+00	CHN1	-1.27E-01	1.00E+00
TMX4	-1.30E+00	3.54E-33	DCAF1	1.10E-01	1.00E+00	NPY5R	2.05E-01	1.00E+00
CAT	-1.26E+00	3.58E-33	RHOC	8.36E-02	1.00E+00	ECM1	2.14E-01	1.00E+00
KLHL21	1.27E+00	3.58E-33	PCGF1	-1.09E-01	1.00E+00	PIK3CD	2.02E-01	1.00E+00
PTX4	7.22E+00	3.65E-33	ADGRB2	-1.67E-01	1.00E+00	LOC112585206	-1.57E-01	1.00E+00
SAMD15	2.74E+00	3.73E-33	FAM151B	1.62E-01	1.00E+00	ZC3H18	-8.45E-02	1.00E+00
ARL1	-1.09E+00	3.94E-33	CD99L2	-1.44E-01	1.00E+00	C2H6orf141	1.39E-01	1.00E+00
LOC102400242	-8.19E+00	3.95E-33	LONP1	9.38E-02	1.00E+00	INTS6	-9.55E-02	1.00E+00
CCDC189	-3.44E+00	4.02E-33	FAM234B	1.08E-01	1.00E+00	GLS	1.08E-01	1.00E+00
SLC5A9	5.98E+00	4.06E-33	FZD2	-2.54E-01	1.00E+00	LOC102412332	-2.00E-01	1.00E+00
MIOX	-5.77E+00	4.07E-33	CBR3	-1.19E-01	1.00E+00	CBY1	-9.22E-02	1.00E+00
APTX	1.54E+00	4.29E-33	EFNB1	9.65E-02	1.00E+00	C1GALT1	-1.30E-01	1.00E+00
GPR1	4.85E+00	4.44E-33	KCTD6	-1.91E-01	1.00E+00	THOC6	1.23E-01	1.00E+00
NPHP1	-1.85E+00	4.45E-33	PRPF4B	1.29E-01	1.00E+00	FRZB	-2.13E-01	1.00E+00
MPP3	3.43E+00	4.51E-33	PRDM6	-9.25E-02	1.00E+00	UBE2U	1.86E-01	1.00E+00
PRKCG	-2.39E+00	4.51E-33	LOC102406738	1.05E-01	1.00E+00	SMAGP	2.38E-01	1.00E+00

METTL27	-4.45E+00		4.54E-33	IRF2BPL	9.86E-02	1.00E+00	MCM5	-1.34E-01	1.00E+00
KATNB1	1.27E+00		4.58E-33	PCP2	-1.96E-01	1.00E+00	FXYD5	2.40E-01	1.00E+00
STRADA	1.09E+00		4.66E-33	NCAPH	7.35E-02	1.00E+00	ZCWPW2	1.24E-01	1.00E+00
CSMD1	-1.98E+00		4.94E-33	PTP4A1	8.22E-02	1.00E+00	DHX32	8.58E-02	1.00E+00
FCN3	5.61E+00		4.99E-33	QRICH2	-1.46E-01	1.00E+00	GATAD1	-1.40E-01	1.00E+00
TNK1	2.12E+00		5.05E-33	TFDP1	-1.28E-01	1.00E+00	SMPD2	-1.23E-01	1.00E+00
LOC102404894	-5.16E+00		5.07E-33	CSAD	1.98E-01	1.00E+00	HSD17B4	-8.85E-02	1.00E+00
AXIN2	1.62E+00		5.20E-33	CNIH3	-1.55E-01	1.00E+00	MLX	-9.10E-02	1.00E+00
LOC112583909	5.11E+00		5.22E-33	AIFM3	-4.55E-01	1.00E+00	RBAK	9.95E-02	1.00E+00
SAFB2	-1.18E+00		5.31E-33	GABRG1	1.15E-01	1.00E+00	LOC112582355	1.71E-01	1.00E+00
PRKAA2	1.76E+00		5.31E-33	TXN	1.31E-01	1.00E+00	SSBP2	1.63E-01	1.00E+00
KLHDC8B	-4.66E+00		5.49E-33	LOC102414273	-2.11E-01	1.00E+00	ABLIM2	-1.86E-01	1.00E+00
GDI1	-1.21E+00		5.57E-33	MIOS	9.19E-02	1.00E+00	IMPA1	9.29E-02	1.00E+00
MUM1L1	-5.38E+00		5.58E-33	VPS41	8.12E-02	1.00E+00	LOC112584582	-1.68E-01	1.00E+00
ERLIN2	-1.31E+00		5.67E-33	PTPN23	-1.50E-01	1.00E+00	C3H17orf97	-2.28E-01	1.00E+00
KLHL28	-2.61E+00		5.68E-33	ATXN10	6.71E-02	1.00E+00	C5H11orf68	1.08E-01	1.00E+00
TP53I11	-4.08E+00		5.80E-33	TFB2M	-2.57E-01	1.00E+00	SLC31A2	1.97E-01	1.00E+00
C5H1orf174	-1.80E+00		5.85E-33	ZNF532	1.25E-01	1.00E+00	GGNBP2	-8.62E-02	1.00E+00
SYAP1	1.19E+00		6.06E-33	TIMM29	-1.00E-01	1.00E+00	ZBTB41	-1.14E-01	1.00E+00
LOC102411322	-9.63E+00		6.08E-33	GTF2F2	-6.76E-02	1.00E+00	SUCO	-9.41E-02	1.00E+00
LOC112583686	-1.69E+00		6.16E-33	BAX	1.06E-01	1.00E+00	SPARC	1.44E-01	1.00E+00
MPHOSPH6	1.62E+00		6.24E-33	GPRIN3	-1.66E-01	1.00E+00	TNFAIP8L3	1.15E-01	1.00E+00
CCDC171	1.76E+00		6.54E-33	BCCIP	-9.89E-02	1.00E+00	LOC102415240	-2.34E-01	1.00E+00
FMO4	-3.40E+00		6.60E-33	LOC102414041	2.06E-01	1.00E+00	CCPG1	-1.21E-01	1.00E+00
ACP1	1.17E+00		6.65E-33	C2H6orf10	-3.00E-01	1.00E+00	PDE6D	1.10E-01	1.00E+00
AVL9	1.62E+00		6.73E-33	SIAH2	-1.68E-01	1.00E+00	SF3B4	-8.25E-02	1.00E+00
PLPPR2	1.74E+00		6.77E-33	CENPI	-1.32E-01	1.00E+00	PRIM2	-1.01E-01	1.00E+00
USP53	-2.92E+00		6.86E-33	BMS1	-8.39E-02	1.00E+00	PTGES3	-9.75E-02	1.00E+00
FBXO7	1.12E+00		7.19E-33	MPZ	-3.56E-01	1.00E+00	TNRC18	-1.05E-01	1.00E+00
FAM189B	-1.25E+00		7.34E-33	TBP	7.72E-02	1.00E+00	SFT2D1	-8.60E-02	1.00E+00
NCL	-1.88E+00		7.37E-33	CPXM2	1.67E-01	1.00E+00	RAB27A	1.61E-01	1.00E+00
LOC102407663	-3.19E+00		7.41E-33	EYA3	8.75E-02	1.00E+00	PDZD9	-2.01E-01	1.00E+00
ATP5MF	-2.15E+00		7.46E-33	MED10	-9.39E-02	1.00E+00	LGALS9	1.88E-01	1.00E+00

LOC112583298	5.80E+00	7.52E-33	BTBD19	-2.11E-01	1.00E+00	LOC112587920	-2.02E-01	1.00E+00
LOC102404904	5.15E+00	7.57E-33	THAP1	1.06E-01	1.00E+00	DUSP19	-1.48E-01	1.00E+00
STRA6	-5.44E+00	7.59E-33	NSMCE2	-9.14E-02	1.00E+00	TRIM47	-1.03E-01	1.00E+00
LOC102399387	-5.31E+00	7.76E-33	KANSL1L	1.96E-01	1.00E+00	SERAC1	1.41E-01	1.00E+00
NDUFB8	-1.53E+00	7.90E-33	MRPS7	8.78E-02	1.00E+00	ATXN2	-8.61E-02	1.00E+00
SPECC1L	1.18E+00	7.91E-33	PELI3	-2.63E-01	1.00E+00	ACOT9	1.16E-01	1.00E+00
CTGF	-6.41E+00	8.19E-33	LOC102389244	2.51E-01	1.00E+00	PRPF19	-1.27E-01	1.00E+00
UFSP1	-4.67E+00	8.23E-33	IARS2	1.07E-01	1.00E+00	ITPRIPL1	1.12E-01	1.00E+00
RBM25	-1.35E+00	8.48E-33	ESRP1	7.98E-02	1.00E+00	DCP1A	-1.18E-01	1.00E+00
PLA2G3	-3.37E+00	8.50E-33	NTRK3	-4.54E-01	1.00E+00	ACER3	1.05E-01	1.00E+00
USP37	1.65E+00	8.66E-33	BCR	1.20E-01	1.00E+00	IQCD	1.77E-01	1.00E+00
RPL28	-3.19E+00	8.78E-33	RAB27B	-1.06E-01	1.00E+00	PIP4K2A	9.35E-02	1.00E+00
LOC102414273	-2.14E+00	8.79E-33	NUP205	9.00E-02	1.00E+00	LOC102414331	1.58E-01	1.00E+00
CNOT6L	1.59E+00	8.83E-33	BLMH	6.96E-02	1.00E+00	PDCL	-1.23E-01	1.00E+00
F2R	-2.95E+00	9.15E-33	NOL11	1.59E-01	1.00E+00	PRPF40A	-8.50E-02	1.00E+00
PLSCR5	-4.28E+00	9.31E-33	LOC112586943	-2.79E-01	1.00E+00	HPSE	-1.50E-01	1.00E+00
STRBP	1.16E+00	9.32E-33	NDUFA9	-8.98E-02	1.00E+00	FDPS	-1.16E-01	1.00E+00
LOC112581378	5.28E+00	9.45E-33	BBS4	1.01E-01	1.00E+00	PDP1	-1.32E-01	1.00E+00
DNAJC21	1.18E+00	9.53E-33	ZNF512B	1.09E-01	1.00E+00	AMMECR1L	-1.11E-01	1.00E+00
SRPK3	-4.19E+00	9.66E-33	RNLS	-2.54E-01	1.00E+00	HERC5	1.85E-01	1.00E+00
ZNF641	1.99E+00	9.66E-33	LOC112587937	-1.70E-01	1.00E+00	YLPM1	-8.95E-02	1.00E+00
PABPC4	-1.62E+00	9.71E-33	OLIG1	-9.07E-02	1.00E+00	STARD4	-1.74E-01	1.00E+00
AZIN1	-1.37E+00	9.93E-33	BET1	-9.84E-02	1.00E+00	ST7	1.03E-01	1.00E+00
SLC47A2	2.75E+00	1.01E-32	LSM11	7.96E-02	1.00E+00	NOP2	9.08E-02	1.00E+00
LOC102401704	-3.76E+00	1.03E-32	TAP1	-1.54E-01	1.00E+00	CLP1	1.05E-01	1.00E+00
ENTPD5	1.48E+00	1.04E-32	UBA7	-2.47E-01	1.00E+00	CLDN3	-1.92E-01	1.00E+00
PPIP5K2	1.61E+00	1.04E-32	VPS35	7.30E-02	1.00E+00	CROCC	-1.03E-01	1.00E+00
TMEM242	1.32E+00	1.05E-32	SECISBP2	1.19E-01	1.00E+00	ARMC6	1.09E-01	1.00E+00
GPC5	2.69E+00	1.05E-32	CLSTN2	-1.02E-01	1.00E+00	TOE1	-1.06E-01	1.00E+00
PLK2	2.25E+00	1.07E-32	LOC102395556	-3.83E-01	1.00E+00	SLC25A13	-8.47E-02	1.00E+00
PRPF19	-1.79E+00	1.08E-32	CFAP52	4.83E-01	1.00E+00	GIPC1	9.80E-02	1.00E+00
FRRS1L	-3.21E+00	1.10E-32	FASTK	1.07E-01	1.00E+00	LOC102402465	1.36E-01	1.00E+00
ESCO2	1.61E+00	1.11E-32	PTGES3	-1.14E-01	1.00E+00	LOC112581897	-1.77E-01	1.00E+00

MAP10	2.29E+00	1.11E-32	VAMP1	2.76E-01	1.00E+00	NCOA2	-1.11E-01	1.00E+00
METTL23	-2.11E+00	1.11E-32	NTM	-2.38E-01	1.00E+00	SEPT11	1.01E-01	1.00E+00
PAOX	-1.99E+00	1.15E-32	LOC102410181	-2.08E-01	1.00E+00	TRDMT1	-1.06E-01	1.00E+00
P4HA3	-2.14E+00	1.16E-32	SLC5A10	-2.90E-01	1.00E+00	LOC102411142	-1.24E-01	1.00E+00
PIEZO1	-2.54E+00	1.18E-32	DCHS1	-5.41E-01	1.00E+00	ZNF70	1.45E-01	1.00E+00
SEC23B	1.19E+00	1.20E-32	ALDH18A1	8.70E-02	1.00E+00	STX2	1.18E-01	1.00E+00
LOC112582058	3.92E+00	1.20E-32	ERCC2	8.84E-02	1.00E+00	CNOT10	-8.48E-02	1.00E+00
LOC112586914	3.12E+00	1.21E-32	AFF3	-1.11E-01	1.00E+00	QPCTL	-1.19E-01	1.00E+00
UBXN2B	1.77E+00	1.22E-32	C2H6orf222	-2.65E-01	1.00E+00	ZNF358	-1.25E-01	1.00E+00
LOC102406999	-6.89E+00	1.24E-32	GHRL	-1.79E-01	1.00E+00	DOLPP1	-1.03E-01	1.00E+00
TRMT44	-1.78E+00	1.24E-32	TMEM128	1.98E-01	1.00E+00	CCDC105	-2.15E-01	1.00E+00
LOC102397881	1.35E+00	1.27E-32	SYNE2	-6.69E-02	1.00E+00	LOC102401654	1.91E-01	1.00E+00
AHNAK2	-4.56E+00	1.28E-32	ALK	-2.68E-01	1.00E+00	ELAC2	9.58E-02	1.00E+00
DCAKD	1.36E+00	1.33E-32	LOC112585535	4.17E-01	1.00E+00	LOC102400287	9.66E-02	1.00E+00
LOC112577668	-4.49E+00	1.33E-32	ACOT6	3.73E-01	1.00E+00	PCDH18	1.67E-01	1.00E+00
RB1	-2.61E+00	1.34E-32	LOC112580820	3.22E-01	1.00E+00	LIPH	1.90E-01	1.00E+00
LRRC49	-1.98E+00	1.43E-32	ASB11	1.01E-01	1.00E+00	WDFY3	-1.10E-01	1.00E+00
HSDL2	-1.88E+00	1.44E-32	UBN2	1.35E-01	1.00E+00	EXOSC6	1.32E-01	1.00E+00
LOC112582305	-4.16E+00	1.48E-32	BCL2L1	6.73E-02	1.00E+00	COPA	-1.01E-01	1.00E+00
KCNAB3	-4.74E+00	1.50E-32	ENSA	7.59E-02	1.00E+00	TOP3A	-8.82E-02	1.00E+00
ZKSCAN2	1.72E+00	1.51E-32	LOC102390176	1.26E-01	1.00E+00	NAP1L4	-8.27E-02	1.00E+00
WDR4	-1.91E+00	1.52E-32	LOC112585052	3.69E-01	1.00E+00	MCM3	-1.21E-01	1.00E+00
PLEKHA1	1.65E+00	1.60E-32	ABCC1	1.01E-01	1.00E+00	GRIPAP1	8.10E-02	1.00E+00
RPS13	-2.39E+00	1.60E-32	TRIM27	-2.36E-01	1.00E+00	JSRP1	-1.77E-01	1.00E+00
PEAK1	-1.81E+00	1.65E-32	RAB4A	1.64E-01	1.00E+00	SMARCC2	-8.67E-02	1.00E+00
GPR153	-2.71E+00	1.68E-32	ABHD14B	-2.53E-01	1.00E+00	DHX35	-9.17E-02	1.00E+00
RBBP8	1.03E+00	1.73E-32	LHX4	-7.34E-02	1.00E+00	ZC3H6	-1.40E-01	1.00E+00
LOC112585666	5.08E+00	1.73E-32	C19H5orf34	-1.11E-01	1.00E+00	RNF2	-8.83E-02	1.00E+00
GPR37	5.04E+00	1.78E-32	LOC102413663	8.49E-02	1.00E+00	EFL1	8.22E-02	1.00E+00
DHCR7	-1.41E+00	1.82E-32	PPARA	1.45E-01	1.00E+00	STAB1	2.53E-01	1.00E+00
SHANK3	1.52E+00	1.92E-32	ADGRF3	2.99E-01	1.00E+00	SLC44A4	-2.44E-01	1.00E+00
CXHXorf56	1.46E+00	1.92E-32	LOC112577799	-1.15E-01	1.00E+00	FAM76B	-1.23E-01	1.00E+00
UBP1	-2.03E+00	1.92E-32	LOC102403840	3.53E-01	1.00E+00	SMARCD2	-9.45E-02	1.00E+00
LOC102391847	-	1.93E-32	LOC112583991	-2.19E-	1.00E+00	PER2	-2.49E-	1.00E+00

	4.83E+00			01			01	
LOC112586994	6.38E+00	1.93E-32	FBXL4	7.55E-02	1.00E+00	LOC102404511	2.03E-01	1.00E+00
TNS1	-2.23E+00	1.99E-32	CEP57L1	-1.03E-01	1.00E+00	CPTP	1.00E-01	1.00E+00
NOXA1	-4.08E+00	2.02E-32	SLC5A8	2.49E-01	1.00E+00	EBP	-1.50E-01	1.00E+00
LOC112587329	-5.09E+00	2.07E-32	PPFIBP1	7.68E-02	1.00E+00	CHADL	-1.75E-01	1.00E+00
STC1	-7.57E+00	2.11E-32	POLR2L	-1.08E-01	1.00E+00	PDZRN3	2.04E-01	1.00E+00
ANAPC4	1.14E+00	2.16E-32	TNRC6B	1.13E-01	1.00E+00	MSRA	-8.67E-02	1.00E+00
MPI	-1.93E+00	2.22E-32	TRMT10B	-1.09E-01	1.00E+00	NUDT6	-8.90E-02	1.00E+00
LOC102406480	1.46E+00	2.22E-32	HOMEZ	9.08E-02	1.00E+00	RPL15	1.10E-01	1.00E+00
CCDC112	-2.46E+00	2.23E-32	LOC112583737	2.42E-01	1.00E+00	PFKFB3	1.05E-01	1.00E+00
AADAT	-2.99E+00	2.27E-32	PDGFRA	-6.07E-01	1.00E+00	TRAPPC2	1.10E-01	1.00E+00
CXHXorf36	4.76E+00	2.36E-32	LOC112582299	-1.82E-01	1.00E+00	KMT2E	-1.14E-01	1.00E+00
SNAI2	-7.63E+00	2.37E-32	NAPB	-1.93E-01	1.00E+00	SMYD5	9.60E-02	1.00E+00
YIPF5	1.07E+00	2.47E-32	MSRB3	-2.57E-01	1.00E+00	PAAF1	9.64E-02	1.00E+00
COMTD1	-3.04E+00	2.48E-32	PITX3	2.57E-01	1.00E+00	NPC1	-1.19E-01	1.00E+00
WSB2	1.16E+00	2.50E-32	LOC102407805	1.20E-01	1.00E+00	LURAP1	-1.53E-01	1.00E+00
TIMMDC1	-1.70E+00	2.52E-32	LOC112580128	-1.01E-01	1.00E+00	BAD	-1.21E-01	1.00E+00
LRCH2	-4.60E+00	2.55E-32	PLPBP	1.09E-01	1.00E+00	LOC102403202	-2.29E-01	1.00E+00
ICE1	-1.64E+00	2.63E-32	GALNT17	3.64E-01	1.00E+00	FAM111B	1.96E-01	1.00E+00
LOC112579908	5.66E+00	2.63E-32	MYBL1	-1.87E-01	1.00E+00	MAP3K20	1.12E-01	1.00E+00
EBF4	-1.89E+00	2.66E-32	RBPJ	7.86E-02	1.00E+00	FCRL5	2.32E-01	1.00E+00
THAP3	-2.22E+00	2.68E-32	LOC102409492	1.42E-01	1.00E+00	TPI1	1.41E-01	1.00E+00
DBP	-2.30E+00	2.69E-32	LRIF1	-9.45E-02	1.00E+00	DNAJC21	-8.46E-02	1.00E+00
FAM217A	3.56E+00	2.74E-32	RABGAP1	8.32E-02	1.00E+00	LOC102389096	1.30E-01	1.00E+00
TENT5D	5.77E+00	2.77E-32	RCN2	1.06E-01	1.00E+00	KATNAL1	-9.69E-02	1.00E+00
PRMT9	-1.38E+00	2.79E-32	NEURL4	1.15E-01	1.00E+00	PMS2	-9.30E-02	1.00E+00
SNAP25	2.51E+00	2.79E-32	PDHA1	1.00E-01	1.00E+00	SLC44A1	-9.04E-02	1.00E+00
DNAJB14	-2.14E+00	2.85E-32	PABPN1L	-7.80E-02	1.00E+00	CADM1	-1.23E-01	1.00E+00
CCDC88A	1.62E+00	2.87E-32	PDZRN3	-9.93E-02	1.00E+00	HDLBP	-1.12E-01	1.00E+00
LOC102390926	6.28E+00	2.87E-32	THAP6	-1.67E-01	1.00E+00	C2CD3	8.88E-02	1.00E+00
ZMYM4	-1.69E+00	3.00E-32	ARMH4	9.00E-02	1.00E+00	SYNE1	1.15E-01	1.00E+00
PDIA3	-1.93E+00	3.03E-32	DNAAF4	-1.27E-01	1.00E+00	FBXL5	8.59E-02	1.00E+00
SLC39A4	-4.66E+00	3.06E-32	LIMD1	4.70E-01	1.00E+00	LOC102413519	-1.44E-01	1.00E+00
CDH1	3.39E+00	3.22E-32	COPS9	-1.34E-	1.00E+00	WDR45B	-8.56E-	1.00E+00

				01			02	
DR1	-1.79E+00	3.27E-32	NUP62	-8.04E-02	1.00E+00	HSPBAP1	1.11E-01	1.00E+00
BHLHE22	-4.60E+00	3.27E-32	SVOPL	1.15E-01	1.00E+00	C6H1orf189	1.74E-01	1.00E+00
WDR83	-2.10E+00	3.27E-32	LOC102405068	-3.35E-01	1.00E+00	DALRD3	-1.17E-01	1.00E+00
PAPSS2	-5.33E+00	3.32E-32	PHOSPHO2	-3.66E-01	1.00E+00	RGMA	-1.54E-01	1.00E+00
ARHGAP24	3.86E+00	3.38E-32	MSH3	-1.03E-01	1.00E+00	PXYLP1	1.16E-01	1.00E+00
TMEM56	-2.28E+00	3.41E-32	LHX8	7.34E-02	1.00E+00	ND3	-1.85E-01	1.00E+00
NSMCE4A	1.15E+00	3.70E-32	BTF3L4	-1.12E-01	1.00E+00	TLR5	-1.95E-01	1.00E+00
ERCC4	1.49E+00	3.71E-32	LOC102406463	-2.80E-01	1.00E+00	RNF14	-9.00E-02	1.00E+00
SRD5A1	-4.94E+00	3.73E-32	ASB6	8.54E-02	1.00E+00	LOC102411613	-1.70E-01	1.00E+00
TECTA	4.05E+00	3.81E-32	DTD2	-2.34E-01	1.00E+00	SLC12A4	8.66E-02	1.00E+00
KIAA1191	1.24E+00	3.84E-32	ELOF1	-1.03E-01	1.00E+00	ZBTB8OS	-9.55E-02	1.00E+00
C9H19orf71	-2.88E+00	3.96E-32	ZNF599	-3.29E-01	1.00E+00	LOC102399157	-1.29E-01	1.00E+00
SETDB1	1.22E+00	4.02E-32	IQGAP1	1.59E-01	1.00E+00	CNIH1	-8.16E-02	1.00E+00
ZC3H3	1.59E+00	4.06E-32	MRPL40	-1.16E-01	1.00E+00	EMC2	-1.15E-01	1.00E+00
BMP6	2.60E+00	4.18E-32	PHYKPL	-3.72E-01	1.00E+00	DEDD2	9.73E-02	1.00E+00
AHSG	-5.39E+00	4.24E-32	LOC112584001	-2.72E-01	1.00E+00	LOC102405384	1.40E-01	1.00E+00
LOC102411226	-4.03E+00	4.39E-32	MYSM1	1.17E-01	1.00E+00	AKR1B1	-1.66E-01	1.00E+00
AXL	-6.94E+00	4.39E-32	LOC102392581	1.39E-01	1.00E+00	SOAT1	-9.54E-02	1.00E+00
PKN3	-2.40E+00	4.39E-32	PPM1B	8.55E-02	1.00E+00	LOC102398966	1.31E-01	1.00E+00
PRICKLE1	1.78E+00	4.49E-32	LOC102401890	-1.58E-01	1.00E+00	ZSWIM9	1.33E-01	1.00E+00
INHBB	-6.79E+00	4.53E-32	FKTN	-1.45E-01	1.00E+00	ORC2	-1.18E-01	1.00E+00
DCC	5.20E+00	4.53E-32	MICAL3	1.09E-01	1.00E+00	LAMB3	1.98E-01	1.00E+00
FASTK	-1.66E+00	4.58E-32	ARL5B	1.53E-01	1.00E+00	RCOR2	-1.36E-01	1.00E+00
ZNF142	-1.31E+00	4.63E-32	FURIN	8.29E-02	1.00E+00	IKZF4	-1.32E-01	1.00E+00
MARK2	1.19E+00	4.71E-32	MAFG	7.73E-02	1.00E+00	SLC52A3	-1.99E-01	1.00E+00
IGDCC4	-5.13E+00	4.76E-32	HTT	1.51E-01	1.00E+00	IQCH	1.28E-01	1.00E+00
SOSTDC1	6.17E+00	4.77E-32	CDKL2	9.12E-02	1.00E+00	ATP6V1F	1.17E-01	1.00E+00
RBM8A	-1.41E+00	4.81E-32	APOA4	3.37E-01	1.00E+00	GNB1L	-1.32E-01	1.00E+00
RAN	-1.63E+00	4.84E-32	LOC112578895	-4.35E-01	1.00E+00	CCDC173	-1.83E-01	1.00E+00
SLC27A2	-3.27E+00	4.90E-32	LOC102397708	2.73E-01	1.00E+00	PARP6	-9.31E-02	1.00E+00
PHF23	-1.24E+00	4.92E-32	SAMHD1	1.02E-01	1.00E+00	ILVBL	9.14E-02	1.00E+00
NEFH	4.35E+00	4.96E-32	CLHC1	1.58E-01	1.00E+00	ZNF821	-9.34E-02	1.00E+00

PFKM	-1.81E+00	5.02E-32	SUPT5H	7.45E-02	1.00E+00	CPPED1	-9.83E-02	1.00E+00
USP8	1.20E+00	5.06E-32	MPC1	-9.84E-02	1.00E+00	RSRC1	-8.67E-02	1.00E+00
CISD3	-3.48E+00	5.10E-32	VWDE	-3.97E-01	1.00E+00	DMTF1	-8.72E-02	1.00E+00
LOC112578055	1.81E+00	5.21E-32	TBKBP1	-1.48E-01	1.00E+00	KLF5	2.39E-01	1.00E+00
MSMO1	-1.27E+00	5.35E-32	GRSF1	6.69E-02	1.00E+00	LOC112585344	-3.47E-01	1.00E+00
PTBP3	-1.51E+00	5.78E-32	ZC3H4	1.97E-01	1.00E+00	MTMR2	-9.69E-02	1.00E+00
DGKG	5.80E+00	6.01E-32	TFEB	-2.14E-01	1.00E+00	TRAM1L1	2.01E-01	1.00E+00
GRK3	1.72E+00	6.08E-32	NT5C	-3.42E-01	1.00E+00	JAM3	8.35E-02	1.00E+00
ARHGAP33	-2.79E+00	6.14E-32	ATM	-1.36E-01	1.00E+00	PDCD7	1.02E-01	1.00E+00
TMEM177	-2.80E+00	6.22E-32	RARB	8.16E-02	1.00E+00	JUNB	-2.50E-01	1.00E+00
LPAR2	2.19E+00	6.52E-32	TMEM248	7.63E-02	1.00E+00	LOC102392068	9.37E-02	1.00E+00
LRP4	-2.35E+00	6.63E-32	EXOSC9	-7.46E-02	1.00E+00	LIPT2	-2.17E-01	1.00E+00
CRISPLD2	-3.19E+00	6.74E-32	LOC112581171	-1.44E-01	1.00E+00	FAM102B	1.67E-01	1.00E+00
XIAP	1.96E+00	6.91E-32	PPIL2	1.06E-01	1.00E+00	ADRB3	2.08E-01	1.00E+00
LOC112579116	2.67E+00	7.52E-32	KCNA5	-7.86E-02	1.00E+00	TSNAX	-9.95E-02	1.00E+00
NCAPH2	-1.67E+00	7.68E-32	PSMA1	-1.48E-01	1.00E+00	NUFIP1	-9.93E-02	1.00E+00
KLHL15	-1.73E+00	7.70E-32	SQSTM1	7.47E-02	1.00E+00	ZNF829	-1.27E-01	1.00E+00
YPEL1	-1.78E+00	7.72E-32	SCN11A	2.98E-01	1.00E+00	HYPK	1.24E-01	1.00E+00
NAPG	-1.63E+00	7.89E-32	OARD1	9.78E-02	1.00E+00	LOC102402392	1.02E-01	1.00E+00
LOC112586516	5.77E+00	7.92E-32	TOMM40L	9.25E-02	1.00E+00	MRPL53	-7.60E-02	1.00E+00
FRMD5	1.64E+00	8.01E-32	HMGCL	2.36E-01	1.00E+00	CFLAR	9.95E-02	1.00E+00
SLC25A30	1.58E+00	8.12E-32	DNAJB6	-7.06E-02	1.00E+00	DHRS12	1.73E-01	1.00E+00
NOVA1	-1.68E+00	8.16E-32	THOC6	-9.31E-02	1.00E+00	SNX15	1.17E-01	1.00E+00
STARD3NL	-1.24E+00	8.33E-32	ZNF706	7.37E-02	1.00E+00	POC1A	-1.19E-01	1.00E+00
CISH	-3.36E+00	8.34E-32	CC2D1A	7.76E-02	1.00E+00	C20H14orf132	1.06E-01	1.00E+00
LOC102413316	4.52E+00	8.44E-32	LOC102406570	-2.64E-01	1.00E+00	HAUS4	-1.13E-01	1.00E+00
SUPT20H	-1.48E+00	8.59E-32	GCM2	3.86E-01	1.00E+00	ATIC	8.10E-02	1.00E+00
METAP1	-1.37E+00	8.75E-32	LOC112577774	-1.09E-01	1.00E+00	NFIX	-1.33E-01	1.00E+00
CYTL1	-8.66E+00	8.82E-32	GLMN	-9.48E-02	1.00E+00	LOC102390045	-1.92E-01	1.00E+00
RUBCNL	4.75E+00	8.96E-32	PHKA2	8.60E-02	1.00E+00	NDUFAB1	1.10E-01	1.00E+00
GRPEL1	1.36E+00	9.14E-32	TEAD1	9.63E-02	1.00E+00	PTPRH	1.69E-01	1.00E+00
C4H12orf4	1.15E+00	9.31E-32	RABL6	8.60E-02	1.00E+00	BRD3	-9.15E-02	1.00E+00
ASDURF	2.15E+00	9.41E-32	GSTZ1	-1.72E-01	1.00E+00	POLR3K	-8.85E-02	1.00E+00
MGAT4B	-1.64E+00	9.41E-32	TSPAN18	-2.14E-01	1.00E+00	SRPK2	-7.94E-02	1.00E+00

OXSM	1.50E+00	9.51E-32	BHLHA9	2.30E-01	1.00E+00	EIF2AK2	1.02E-01	1.00E+00
PTPN13	1.81E+00	9.58E-32	RABEP2	-1.40E-01	1.00E+00	RBM10	-9.72E-02	1.00E+00
TMEM17	-2.46E+00	9.73E-32	MRPS12	-1.60E-01	1.00E+00	ARNT	-9.77E-02	1.00E+00
CFAP36	-1.62E+00	1.01E-31	LOC102406144	-4.72E-01	1.00E+00	ZNF25	-1.45E-01	1.00E+00
NXPH3	-4.70E+00	1.02E-31	OVCH2	-2.58E-01	1.00E+00	LOC112587814	1.11E-01	1.00E+00
LRRFIP1	-2.50E+00	1.04E-31	PPP1CA	9.39E-02	1.00E+00	BTK	-3.68E-01	1.00E+00
PLIN4	-5.47E+00	1.05E-31	TIMM44	6.93E-02	1.00E+00	ARAP3	2.37E-01	1.00E+00
RAPSN	-2.33E+00	1.09E-31	VPS37A	-1.08E-01	1.00E+00	NLGN1	1.25E-01	1.00E+00
KDM4A	1.22E+00	1.09E-31	ZBED3	-2.78E-01	1.00E+00	MTMR14	8.82E-02	1.00E+00
PREX1	3.76E+00	1.15E-31	CSTF3	-7.54E-02	1.00E+00	MSL3	-8.13E-02	1.00E+00
TRAM2	1.85E+00	1.23E-31	HEXIM2	-1.74E-01	1.00E+00	GFRA3	-1.89E-01	1.00E+00
LOC112582329	6.91E+00	1.25E-31	TNFRSF25	2.24E-01	1.00E+00	SEMA6D	-1.38E-01	1.00E+00
MEX3D	-2.38E+00	1.25E-31	LOC102405065	1.37E-01	1.00E+00	ZDHHC6	-8.66E-02	1.00E+00
HPS1	-1.49E+00	1.27E-31	FAM162A	-1.12E-01	1.00E+00	FAM53C	1.21E-01	1.00E+00
LAMA1	-5.79E+00	1.27E-31	PRPS1	1.42E-01	1.00E+00	MORC4	-9.33E-02	1.00E+00
TOM1L2	-1.62E+00	1.37E-31	C15H8orf34	-1.47E-01	1.00E+00	LOC112587947	-1.83E-01	1.00E+00
LOC102408933	8.29E+00	1.41E-31	RFTN1	1.94E-01	1.00E+00	CYYR1	-2.72E-01	1.00E+00
LOC102397143	-6.74E+00	1.42E-31	TSPAN7	7.18E-02	1.00E+00	CDC7	-9.33E-02	1.00E+00
COL6A5	5.15E+00	1.43E-31	MROH1	-2.48E-01	1.00E+00	SNAPC1	-1.10E-01	1.00E+00
PFKFB4	-1.54E+00	1.44E-31	TNN	-1.48E-01	1.00E+00	IFNGR1	8.53E-02	1.00E+00
ATXN7	1.60E+00	1.44E-31	FMNL3	9.73E-02	1.00E+00	ME2	8.31E-02	1.00E+00
ARHGEF39	-2.23E+00	1.46E-31	PRPF19	7.53E-02	1.00E+00	RITA1	1.06E-01	1.00E+00
PRSS56	5.63E+00	1.47E-31	CCDC116	-1.81E-01	1.00E+00	HDAC5	-8.59E-02	1.00E+00
GFM1	-1.59E+00	1.47E-31	NSUN7	-1.15E-01	1.00E+00	ANXA5	1.14E-01	1.00E+00
MCPH1	1.11E+00	1.50E-31	RPS27	-2.97E-01	1.00E+00	NCOA7	-1.15E-01	1.00E+00
LOC102389957	-2.33E+00	1.55E-31	CXXC5	-4.30E-01	1.00E+00	CDC25A	-9.16E-02	1.00E+00
ZDHHC20	-1.48E+00	1.59E-31	PCP4L1	-1.39E-01	1.00E+00	LOC102404507	-2.50E-01	1.00E+00
MEI4	-2.52E+00	1.62E-31	HPCAL1	1.03E-01	1.00E+00	TBCD	-8.57E-02	1.00E+00
RAB39B	2.52E+00	1.67E-31	ZNF140	-2.17E-01	1.00E+00	TSPAN14	-8.36E-02	1.00E+00
S100A14	-5.08E+00	1.67E-31	LOC102410583	1.20E-01	1.00E+00	PPP1R1B	-2.26E-01	1.00E+00
LOC102392605	-1.44E+00	1.69E-31	RAB21	8.15E-02	1.00E+00	NT5M	1.15E-01	1.00E+00
ATP7A	-3.25E+00	1.71E-31	LOC102398037	1.76E-01	1.00E+00	FGFR2	2.16E-01	1.00E+00
P2RY2	-5.69E+00	1.73E-31	SHROOM3	8.94E-02	1.00E+00	OGDH	7.98E-02	1.00E+00

CAVIN4	-4.03E+00	1.78E-31	LOC102397908	-1.97E-01	1.00E+00	CRIPT	8.99E-02	1.00E+00
ANO7	-4.37E+00	1.82E-31	SLC4A5	-3.47E-01	1.00E+00	RNFT1	-9.38E-02	1.00E+00
ESD	1.22E+00	1.85E-31	SNX13	-1.01E-01	1.00E+00	RNF8	-8.79E-02	1.00E+00
EXOC8	1.37E+00	1.90E-31	DLG5	1.22E-01	1.00E+00	SFN	1.91E-01	1.00E+00
LOC112587010	7.02E+00	1.91E-31	FOXD2	1.74E-01	1.00E+00	XPOT	-8.06E-02	1.00E+00
SEC62	1.05E+00	1.94E-31	OXTR	-2.33E-01	1.00E+00	KLHDC2	-7.64E-02	1.00E+00
MEPCE	1.42E+00	1.97E-31	OFD1	-9.55E-02	1.00E+00	TSPAN33	1.89E-01	1.00E+00
C11H15orf61	2.01E+00	1.99E-31	ORC4	-7.94E-02	1.00E+00	LTB	-1.73E-01	1.00E+00
MCTS1	-2.04E+00	2.10E-31	NR2F1	-2.33E-01	1.00E+00	DDX56	-8.79E-02	1.00E+00
IL1RAP	-5.30E+00	2.16E-31	PLS1	6.87E-02	1.00E+00	TBCB	9.70E-02	1.00E+00
IQGAP2	1.69E+00	2.19E-31	LOC102393166	-1.35E-01	1.00E+00	BAIAP2L2	1.81E-01	1.00E+00
OPTN	1.07E+00	2.25E-31	NDNF	-5.47E-01	1.00E+00	DISP1	9.51E-02	1.00E+00
LOC102392359	1.95E+00	2.35E-31	FAM241B	-9.46E-02	1.00E+00	DAG1	-1.27E-01	1.00E+00
CNGA2	-8.33E+00	2.48E-31	TMEM178A	1.14E-01	1.00E+00	PTN	-1.07E-01	1.00E+00
FKBP14	-1.42E+00	2.50E-31	LOC102393576	1.90E-01	1.00E+00	EXOC2	-7.61E-02	1.00E+00
ICA1	-1.40E+00	2.51E-31	SLC47A1	-1.00E-01	1.00E+00	ZNHIT6	-9.12E-02	1.00E+00
LRP12	-4.26E+00	2.56E-31	CEP97	9.58E-02	1.00E+00	GPR180	-1.14E-01	1.00E+00
DNAJC15	-1.43E+00	2.57E-31	DCC	7.41E-02	1.00E+00	GNPTG	-1.01E-01	1.00E+00
BICC1	4.60E+00	2.57E-31	UBE3B	8.43E-02	1.00E+00	PAIP2B	-9.38E-02	1.00E+00
FKBP9	-1.87E+00	2.59E-31	LOC112587357	-2.51E-01	1.00E+00	TRAPPC10	-1.15E-01	1.00E+00
ANKRD34B	-6.90E+00	2.63E-31	MRPS9	-9.43E-02	1.00E+00	MRPS11	1.20E-01	1.00E+00
HOXA3	2.92E+00	2.69E-31	R3HDM4	-1.15E-01	1.00E+00	LOC102404032	1.96E-01	1.00E+00
UBA52	-2.29E+00	2.79E-31	LOC102410676	1.02E-01	1.00E+00	C22H18orf54	1.29E-01	1.00E+00
LOC102410971	-3.39E+00	2.79E-31	TOLLIP	7.14E-02	1.00E+00	TIMM22	1.03E-01	1.00E+00
PEX1	1.95E+00	2.87E-31	RCOR1	-1.08E-01	1.00E+00	INTS1	-8.24E-02	1.00E+00
QSOX1	-2.05E+00	2.88E-31	CFAP43	2.02E-01	1.00E+00	NDUFC1	1.05E-01	1.00E+00
BLCAP	1.04E+00	2.92E-31	LYAR	-7.35E-02	1.00E+00	ACTR3	8.91E-02	1.00E+00
TENT4A	1.38E+00	2.94E-31	LOC102393972	8.65E-02	1.00E+00	SNX21	1.24E-01	1.00E+00
LOC112586947	3.34E+00	2.98E-31	PLEKHB1	-1.18E-01	1.00E+00	LOC102400151	1.35E-01	1.00E+00
LOC112586935	4.69E+00	3.00E-31	ISPD	-9.78E-02	1.00E+00	LOC112582281	-8.61E-02	1.00E+00
CCDC137	1.32E+00	3.00E-31	ARGLU1	-9.88E-02	1.00E+00	SEC22C	1.37E-01	1.00E+00
LOC102403745	-3.46E+00	3.09E-31	NOC2L	1.30E-01	1.00E+00	HFM1	1.64E-01	1.00E+00
GLCE	-1.81E+00	3.18E-31	ACTG1	8.39E-02	1.00E+00	FAM110C	2.03E-01	1.00E+00

FAM229A	-5.26E+00	3.19E-31	PPCS	-7.81E-02	1.00E+00	CDS2	-7.83E-02	1.00E+00
MDGA1	-2.29E+00	3.22E-31	MARCH6	8.91E-02	1.00E+00	POLM	-1.16E-01	1.00E+00
ANXA1	-6.23E+00	3.39E-31	P3H3	-3.64E-01	1.00E+00	APRT	1.19E-01	1.00E+00
SC5D	-4.10E+00	3.46E-31	SAC3D1	-2.85E-01	1.00E+00	PMM2	-8.48E-02	1.00E+00
LGALS9	-5.34E+00	3.50E-31	RBM15	1.23E-01	1.00E+00	ARPC3	9.33E-02	1.00E+00
PELI2	1.49E+00	3.59E-31	NDUFV3	-7.69E-02	1.00E+00	NAA10	1.18E-01	1.00E+00
MYO5A	-1.40E+00	3.64E-31	TUBGCP2	-9.49E-02	1.00E+00	RPS27L	1.18E-01	1.00E+00
TLX3	6.36E+00	3.64E-31	RFX1	9.96E-02	1.00E+00	STT3A	-9.11E-02	1.00E+00
JAKMIP2	-1.52E+00	3.65E-31	DHDH	-3.15E-01	1.00E+00	YWHAG	9.27E-02	1.00E+00
MYOCD	-5.19E+00	3.66E-31	EGR4	1.86E-01	1.00E+00	C18H19orf48	9.25E-02	1.00E+00
FAM20B	-1.46E+00	3.68E-31	LOC112582913	-2.25E-01	1.00E+00	BAK1	9.36E-02	1.00E+00
ETV2	-5.59E+00	3.68E-31	GFRA1	-3.80E-01	1.00E+00	SLC30A7	-1.05E-01	1.00E+00
MCM3	1.95E+00	3.84E-31	DNAI1	-1.81E-01	1.00E+00	SPRYD3	7.56E-02	1.00E+00
KCTD1	-1.80E+00	3.85E-31	IZUMO4	2.65E-01	1.00E+00	INSYN1	1.62E-01	1.00E+00
DCLK1	-4.45E+00	3.87E-31	TBC1D25	8.66E-02	1.00E+00	EMC6	1.11E-01	1.00E+00
CXCL12	-3.24E+00	3.89E-31	LOC112579692	-3.99E-01	1.00E+00	NDUFA13	1.33E-01	1.00E+00
ANKRD11	-1.33E+00	4.05E-31	NHLRC2	-1.17E-01	1.00E+00	NT5DC1	-1.06E-01	1.00E+00
EP400	-1.92E+00	4.26E-31	MAPK12	-2.89E-01	1.00E+00	ADAT1	-1.60E-01	1.00E+00
LOC102402668	6.13E+00	4.32E-31	PRMT1	-7.18E-02	1.00E+00	GPATCH4	1.54E-01	1.00E+00
SRM	-1.77E+00	4.48E-31	D2HGDH	-9.09E-02	1.00E+00	MAP7	-1.46E-01	1.00E+00
ABRA	6.24E+00	4.59E-31	DCLRE1C	1.03E-01	1.00E+00	CDKN2AIPNL	-1.19E-01	1.00E+00
STARD9	-2.18E+00	4.65E-31	FBXO28	1.84E-01	1.00E+00	DMWD	8.69E-02	1.00E+00
NFATC1	3.70E+00	4.69E-31	MRPL32	-9.12E-02	1.00E+00	COBL	2.23E-01	1.00E+00
ILKAP	-1.43E+00	4.90E-31	NIPA2	1.15E-01	1.00E+00	WASHC5	1.04E-01	1.00E+00
APOOL	-5.35E+00	5.04E-31	ITGB1BP1	-8.51E-02	1.00E+00	HILPDA	1.14E-01	1.00E+00
LOC102408092	3.44E+00	5.05E-31	LOC102401360	-3.60E-01	1.00E+00	TCTN3	-9.42E-02	1.00E+00
NDUFB9	1.94E+00	5.08E-31	HEATR1	1.05E-01	1.00E+00	USP7	-8.17E-02	1.00E+00
TECR	-1.80E+00	5.14E-31	IZUMO1R	8.60E-02	1.00E+00	CLMN	1.80E-01	1.00E+00
LOC112585629	-3.68E+00	5.22E-31	UBXN4	6.94E-02	1.00E+00	NR1H3	1.19E-01	1.00E+00
CD70	-6.31E+00	5.24E-31	CAPRIN1	-7.20E-02	1.00E+00	LOC102407124	-8.54E-02	1.00E+00
CERS2	1.17E+00	5.42E-31	LOC112584636	3.58E-01	1.00E+00	SPDYA	1.65E-01	1.00E+00
LOC102397766	2.29E+00	5.46E-31	MAT2A	-6.85E-02	1.00E+00	LOC112584610	-1.85E-01	1.00E+00
LSM14B	1.15E+00	5.51E-31	FAM227B	-1.07E-	1.00E+00	LOC112586887	-1.92E-	1.00E+00

				01			01	
WWC2	1.79E+00	5.54E-31	UBR1	1.74E-01	1.00E+00	C1H3orf38	-9.22E-02	1.00E+00
NSMF	-3.60E+00	5.65E-31	C12H2orf92	5.25E-01	1.00E+00	LOC102398227	1.62E-01	1.00E+00
LOC112579236	1.76E+00	5.77E-31	KDM5C	9.19E-02	1.00E+00	FANCF	1.79E-01	1.00E+00
LOC102394634	-5.27E+00	5.88E-31	SPINK5	-9.57E-02	1.00E+00	IFT172	-9.53E-02	1.00E+00
POLR1B	1.27E+00	5.95E-31	SLC39A14	9.58E-02	1.00E+00	CPNE5	1.84E-01	1.00E+00
TNFRSF1B	-2.90E+00	5.96E-31	KIF3B	8.70E-02	1.00E+00	LOC102411606	1.00E-01	1.00E+00
DIS3L2	-1.50E+00	6.04E-31	OPHN1	-3.19E-01	1.00E+00	LOC102393117	1.20E-01	1.00E+00
ZZEF1	-1.62E+00	6.12E-31	ELF3	-1.06E-01	1.00E+00	DCLRE1A	1.08E-01	1.00E+00
LOC112580423	5.93E+00	6.17E-31	BBS5	1.00E-01	1.00E+00	MANEAL	-1.86E-01	1.00E+00
LOC102410221	-4.13E+00	6.20E-31	EIF5B	-8.28E-02	1.00E+00	FAM114A2	-8.25E-02	1.00E+00
SEMA3C	-4.99E+00	6.26E-31	LOC112577616	-4.02E-01	1.00E+00	GZF1	-9.30E-02	1.00E+00
PCDHB10	-5.18E+00	6.36E-31	CDK12	1.45E-01	1.00E+00	IFIT5	1.17E-01	1.00E+00
GYPC	-5.18E+00	6.51E-31	WISP3	1.25E-01	1.00E+00	TNIP3	2.19E-01	1.00E+00
AP3D1	-1.14E+00	6.60E-31	CDC42BPA	9.33E-02	1.00E+00	DDX47	8.44E-02	1.00E+00
LOC102401474	6.24E+00	6.92E-31	ANKRD23	-2.26E-01	1.00E+00	C4H12orf50	-1.91E-01	1.00E+00
KLC4	-1.31E+00	7.19E-31	PXYLP1	1.42E-01	1.00E+00	RALGAPA2	-9.07E-02	1.00E+00
PPP1R14A	-5.08E+00	7.21E-31	ATP6V1E1	8.11E-02	1.00E+00	NHSL1	1.19E-01	1.00E+00
LENG9	-4.40E+00	7.27E-31	RDH13	2.36E-01	1.00E+00	PGAP3	-8.68E-02	1.00E+00
FBP1	-3.30E+00	7.36E-31	SNX19	7.08E-02	1.00E+00	RBM8A	9.18E-02	1.00E+00
TMEM65	-1.83E+00	7.38E-31	NOL8	1.09E-01	1.00E+00	KCTD20	-7.87E-02	1.00E+00
TGFA	3.39E+00	7.38E-31	CBLB	-9.60E-02	1.00E+00	LOC112583838	2.12E-01	1.00E+00
CEP55	-1.34E+00	7.43E-31	XRCC3	2.17E-01	1.00E+00	HAUS3	9.09E-02	1.00E+00
ACCS	-3.06E+00	7.56E-31	CD302	8.96E-02	1.00E+00	RCOR3	-9.80E-02	1.00E+00
SCARA3	-6.10E+00	7.58E-31	TNKS2	1.52E-01	1.00E+00	SELL	1.15E-01	1.00E+00
RNF183	3.89E+00	7.59E-31	VTA1	8.01E-02	1.00E+00	LOC102389646	-1.12E-01	1.00E+00
CHAC1	1.87E+00	8.26E-31	CCDC50	8.07E-02	1.00E+00	TMEM248	7.55E-02	1.00E+00
AIP	-1.57E+00	8.28E-31	PALM3	1.12E-01	1.00E+00	MTPAP	8.99E-02	1.00E+00
MSL3	1.07E+00	8.42E-31	LOC102404507	-2.00E-01	1.00E+00	SLC6A6	-3.65E-01	1.00E+00
MCOLN1	-1.21E+00	8.50E-31	AXIN2	9.52E-02	1.00E+00	KANK3	-1.70E-01	1.00E+00
VPS25	-1.67E+00	8.62E-31	DNAJB4	1.74E-01	1.00E+00	TMEM238	-1.44E-01	1.00E+00
MRNIP	1.41E+00	8.65E-31	CLEC16A	7.34E-02	1.00E+00	PLCXD2	-1.41E-01	1.00E+00
C9H1orf35	-1.81E+00	8.66E-31	LLPH	-9.61E-02	1.00E+00	NFXL1	-1.30E-01	1.00E+00
SSR1	-	8.93E-31	SYNE1	2.02E-01	1.00E+00	AKT2	-7.75E-	1.00E+00

	1.15E+00						02	
ARMC10	1.63E+00	8.93E-31	DRC3	-2.86E-01	1.00E+00	PRDM4	8.46E-02	1.00E+00
CRTAM	5.40E+00	9.12E-31	EIF4EBP3	-1.46E-01	1.00E+00	TNRC6A	-1.15E-01	1.00E+00
SLC10A4	5.30E+00	9.33E-31	GLO1	-8.11E-02	1.00E+00	NACC1	-2.08E-01	1.00E+00
P2RX4	-1.48E+00	9.80E-31	ACAT2	-6.92E-02	1.00E+00	CEP164	8.78E-02	1.00E+00
EIF4G1	-1.28E+00	9.81E-31	MON1A	1.82E-01	1.00E+00	CSTF2	9.69E-02	1.00E+00
PACS1	-1.34E+00	9.84E-31	STX2	1.08E-01	1.00E+00	MTO1	-9.44E-02	1.00E+00
LOC112581826	4.37E+00	9.86E-31	GSE1	8.47E-02	1.00E+00	MLH3	1.26E-01	1.00E+00
C19H5orf49	-4.69E+00	1.02E-30	CCDC28B	-1.64E-01	1.00E+00	CACYBP	-9.33E-02	1.00E+00
IP6K2	1.28E+00	1.04E-30	ANKS1B	1.16E-01	1.00E+00	SNRNP27	9.35E-02	1.00E+00
LOC112579121	-4.95E+00	1.04E-30	MAPK10	-8.62E-02	1.00E+00	RPL7L1	1.08E-01	1.00E+00
LOC112583749	6.49E+00	1.06E-30	VEGFC	1.49E-01	1.00E+00	AAK1	1.47E-01	1.00E+00
EMD	-1.32E+00	1.07E-30	KCTD3	7.13E-02	1.00E+00	RAB3A	-2.05E-01	1.00E+00
RFC1	1.03E+00	1.07E-30	LOC112582121	-1.72E-01	1.00E+00	TAF1B	-9.73E-02	1.00E+00
ZNF385B	-1.97E+00	1.08E-30	TFB1M	7.70E-02	1.00E+00	LOC112581189	-2.24E-01	1.00E+00
CCDC115	-1.31E+00	1.09E-30	NFATC3	1.28E-01	1.00E+00	PROS1	-9.70E-02	1.00E+00
LOC112585698	-4.04E+00	1.12E-30	TCEA2	-1.64E-01	1.00E+00	DTX3L	2.11E-01	1.00E+00
RSPH9	-2.14E+00	1.13E-30	ACADVL	1.10E-01	1.00E+00	TMEM161A	-1.05E-01	1.00E+00
MKI67	-1.93E+00	1.13E-30	PLAG1	2.01E-01	1.00E+00	PPP1R14A	-2.08E-01	1.00E+00
LAYN	4.17E+00	1.14E-30	LOC102395887	8.97E-02	1.00E+00	LOC102403241	1.06E-01	1.00E+00
CDK10	-1.92E+00	1.14E-30	PGAM1	-6.63E-02	1.00E+00	HDGFL3	-1.77E-01	1.00E+00
LSM6	-1.86E+00	1.17E-30	LOC102416301	-1.70E-01	1.00E+00	LOC102413915	-9.29E-02	1.00E+00
RGS21	6.28E+00	1.18E-30	SH3BGRL2	1.14E-01	1.00E+00	AP2S1	1.18E-01	1.00E+00
FGF14	-2.99E+00	1.18E-30	USF1	-1.86E-01	1.00E+00	SCAMP2	-7.69E-02	1.00E+00
CAVIN3	-3.51E+00	1.19E-30	CDK5RAP3	8.03E-02	1.00E+00	MSH2	-7.96E-02	1.00E+00
IDNK	-2.83E+00	1.21E-30	CACHD1	1.08E-01	1.00E+00	STX4	1.13E-01	1.00E+00
NUP43	1.45E+00	1.23E-30	RHOBTB3	-2.34E-01	1.00E+00	RPP25L	1.03E-01	1.00E+00
LOC112586216	1.83E+00	1.25E-30	HOGA1	-2.15E-01	1.00E+00	DDX21	8.72E-02	1.00E+00
LOC102402422	2.09E+00	1.26E-30	LOC112586394	2.58E-01	1.00E+00	DRC3	-1.89E-01	1.00E+00
TBX1	-6.20E+00	1.28E-30	MVD	1.59E-01	1.00E+00	TMEM53	1.26E-01	1.00E+00
UBXN11	-2.40E+00	1.29E-30	RPS15	-1.11E-01	1.00E+00	SLC28A2	2.28E-01	1.00E+00
XPR1	1.34E+00	1.29E-30	EDF1	-8.06E-02	1.00E+00	LOC102390612	1.11E-01	1.00E+00
EEF1B2	-2.10E+00	1.31E-30	EWSR1	-1.19E-01	1.00E+00	MARCH7	-1.09E-01	1.00E+00
RPP21	1.86E+00	1.33E-30	ZNF283	1.49E-01	1.00E+00	KLHL13	1.61E-01	1.00E+00
RPP30	1.24E+00	1.33E-30	CD2AP	1.54E-01	1.00E+00	TESK2	1.37E-01	1.00E+00

PLPP1	-1.45E+00	1.34E-30	C1QTNF2	-1.40E-01	1.00E+00	SLC35G2	2.04E-01	1.00E+00
CCDC88B	-3.55E+00	1.34E-30	MIB2	1.64E-01	1.00E+00	LOC112586657	1.80E-01	1.00E+00
TBC1D4	-1.53E+00	1.35E-30	CDC42SE2	7.18E-02	1.00E+00	FKTN	-1.08E-01	1.00E+00
FGFR2	2.80E+00	1.43E-30	CNOT1	1.14E-01	1.00E+00	AP4B1	-1.13E-01	1.00E+00
METAP2	1.08E+00	1.43E-30	NOLC1	7.01E-02	1.00E+00	MUTYH	-1.08E-01	1.00E+00
MCTP2	4.12E+00	1.46E-30	LOC112586515	-1.93E-01	1.00E+00	ERO1B	-8.81E-02	1.00E+00
YWHAE	-1.41E+00	1.49E-30	LOC102408084	-2.88E-01	1.00E+00	STARD8	1.76E-01	1.00E+00
EFTUD2	1.27E+00	1.50E-30	LOC112582052	-2.20E-01	1.00E+00	SAC3D1	1.46E-01	1.00E+00
LHX9	5.32E+00	1.54E-30	ABTB2	-1.00E-01	1.00E+00	ST6GALNAC4	1.02E-01	1.00E+00
SSTR1	-4.92E+00	1.57E-30	ROBO2	1.03E-01	1.00E+00	RAB8A	8.51E-02	1.00E+00
EPS8	2.82E+00	1.61E-30	TSR1	7.93E-02	1.00E+00	HBS1L	-7.85E-02	1.00E+00
NIP7	1.79E+00	1.66E-30	LOC102389468	-7.03E-02	1.00E+00	ABCA3	-9.56E-02	1.00E+00
TRAPPC6A	-3.20E+00	1.66E-30	CPSF3	8.10E-02	1.00E+00	SPNS1	-7.52E-02	1.00E+00
GCKR	4.26E+00	1.67E-30	DAPL1	-1.28E-01	1.00E+00	ZNF644	9.63E-02	1.00E+00
PTPRC	-5.27E+00	1.71E-30	LRRC38	2.34E-01	1.00E+00	R3HDM4	-8.17E-02	1.00E+00
RALYL	-4.44E+00	1.74E-30	ACACB	1.58E-01	1.00E+00	PHYHD1	-1.20E-01	1.00E+00
SESN2	1.04E+00	1.81E-30	LOC112578089	-4.13E-01	1.00E+00	LOC112585148	1.37E-01	1.00E+00
AK6	-2.82E+00	1.83E-30	EIF4EBP1	-1.24E-01	1.00E+00	ERGIC2	-7.74E-02	1.00E+00
ACSBG1	-5.83E+00	1.84E-30	LOC102407761	7.33E-02	1.00E+00	KMT2D	-1.33E-01	1.00E+00
TSR3	-2.07E+00	1.89E-30	NUBP1	9.79E-02	1.00E+00	ARRDC4	-9.54E-02	1.00E+00
CSRNP3	-3.52E+00	1.98E-30	LPCAT2	1.09E-01	1.00E+00	COG1	-8.27E-02	1.00E+00
AK5	-4.35E+00	2.00E-30	PTGR1	-4.78E-01	1.00E+00	TGFBR1	-1.73E-01	1.00E+00
PCDH10	-5.57E+00	2.07E-30	LOC102404636	-1.92E-01	1.00E+00	SPATA5L1	-8.69E-02	1.00E+00
LOC102407915	-6.26E+00	2.08E-30	ROBO1	1.05E-01	1.00E+00	SLC25A53	-1.13E-01	1.00E+00
GLYCTK	-4.49E+00	2.13E-30	AKR1B1	1.02E-01	1.00E+00	GAS8	-1.34E-01	1.00E+00
PGRMC2	-1.11E+00	2.13E-30	ATP1A1	8.08E-02	1.00E+00	TBRG4	9.08E-02	1.00E+00
PRELID1	-1.44E+00	2.18E-30	ELAC1	-2.15E-01	1.00E+00	LOC112586900	-3.77E-01	1.00E+00
COTL1	-3.11E+00	2.19E-30	SYNGAP1	-1.22E-01	1.00E+00	MRPS25	1.32E-01	1.00E+00
BPHL	-2.26E+00	2.22E-30	FAM160B2	1.04E-01	1.00E+00	HIVEP1	-1.12E-01	1.00E+00
SSH1	1.30E+00	2.26E-30	DHX9	-1.11E-01	1.00E+00	GLUL	8.95E-02	1.00E+00
ZNF449	1.50E+00	2.28E-30	TMEM160	-1.54E-01	1.00E+00	TEX49	-1.61E-01	1.00E+00
TMEM140	1.45E+00	2.30E-30	LHPP	-1.41E-01	1.00E+00	WDR97	-1.20E-01	1.00E+00

LOC102405714	-3.92E+00	2.37E-30	MSANTD2	-2.20E-01	1.00E+00	IQCB1	-1.02E-01	1.00E+00
MRAS	-1.35E+00	2.44E-30	CSK	1.37E-01	1.00E+00	RIOK1	-1.16E-01	1.00E+00
CRB2	3.15E+00	2.46E-30	SLC51A	-2.81E-01	1.00E+00	LOC102409828	-1.43E-01	1.00E+00
FCHSD1	1.59E+00	2.49E-30	BORCS7	-4.53E-01	1.00E+00	MYOM2	-1.16E-01	1.00E+00
PADI2	3.82E+00	2.61E-30	PEX13	-1.88E-01	1.00E+00	DUSP16	9.59E-02	1.00E+00
CCDC172	6.50E+00	2.63E-30	FN3K	-2.21E-01	1.00E+00	PTS	1.02E-01	1.00E+00
POLR3GL	-2.00E+00	2.65E-30	SDC4	-4.68E-01	1.00E+00	EPHA4	1.97E-01	1.00E+00
LOC102399277	2.19E+00	2.66E-30	ANTXR2	-3.20E-01	1.00E+00	TELO2	-9.54E-02	1.00E+00
GIT1	1.09E+00	2.73E-30	CFAP54	-3.01E-01	1.00E+00	RMND5A	-9.31E-02	1.00E+00
RSF1	-1.43E+00	2.73E-30	POLR3F	-3.59E-01	1.00E+00	CHERP	-8.68E-02	1.00E+00
LOC102402392	-1.85E+00	2.76E-30	RAP2A	1.88E-01	1.00E+00	RNF214	-9.52E-02	1.00E+00
TBXT	5.95E+00	2.76E-30	SNCA	-8.51E-02	1.00E+00	SLC18B1	1.20E-01	1.00E+00
HGD	3.45E+00	3.02E-30	PRDM4	8.31E-02	1.00E+00	WDR1	9.21E-02	1.00E+00
FBXL13	3.07E+00	3.02E-30	PIEZO1	2.19E-01	1.00E+00	LOC102408240	-7.86E-02	1.00E+00
LOC112585664	6.79E+00	3.06E-30	GDI1	6.95E-02	1.00E+00	TRIM8	9.97E-02	1.00E+00
IQCN	-4.44E+00	3.08E-30	LOC112586908	-1.81E-01	1.00E+00	SLC38A1	1.11E-01	1.00E+00
HSPA2	1.28E+00	3.10E-30	MTUS2	8.74E-02	1.00E+00	LOC112587492	-2.76E-01	1.00E+00
CHST4	6.57E+00	3.13E-30	LOC112586663	2.39E-01	1.00E+00	STX6	9.07E-02	1.00E+00
LOC112580357	7.14E+00	3.16E-30	CAPN1	7.86E-02	1.00E+00	ZMYND11	-8.30E-02	1.00E+00
ZNF367	-3.60E+00	3.20E-30	TEAD2	1.85E-01	1.00E+00	ACTR2	1.04E-01	1.00E+00
LOC102403358	3.22E+00	3.49E-30	GYS1	1.07E-01	1.00E+00	SNX1	-7.42E-02	1.00E+00
LOC112583857	-1.53E+00	3.55E-30	MICU2	-1.01E-01	1.00E+00	KLHL28	-1.45E-01	1.00E+00
LRRC75A	-4.08E+00	3.56E-30	LOC112584624	-1.67E-01	1.00E+00	GLB1	-7.99E-02	1.00E+00
TBC1D8B	-1.48E+00	3.74E-30	LOC102415697	3.06E-01	1.00E+00	LOC112578166	-2.62E-01	1.00E+00
GCH1	1.77E+00	3.75E-30	ZMYND19	-8.65E-02	1.00E+00	FAM216A	-1.55E-01	1.00E+00
MAP4K5	1.31E+00	3.77E-30	SESN1	-1.13E-01	1.00E+00	ADCK1	1.13E-01	1.00E+00
LOC112578471	-5.19E+00	3.85E-30	WDR70	-1.01E-01	1.00E+00	RIPK3	2.97E-01	1.00E+00
LOC102408293	5.70E+00	3.91E-30	SH3RF1	1.30E-01	1.00E+00	EDRF1	9.67E-02	1.00E+00
LOC112581736	5.23E+00	3.99E-30	KRBA1	5.25E-01	1.00E+00	GABPB1	8.43E-02	1.00E+00
PHF21A	-1.17E+00	4.03E-30	CD27	-2.15E-01	1.00E+00	CHMP2B	9.50E-02	1.00E+00
LOC102401265	1.83E+00	4.07E-30	HNRNPM	-7.76E-02	1.00E+00	CDCA2	-1.11E-01	1.00E+00
ZNRF2	1.44E+00	4.11E-30	BAHD1	8.61E-02	1.00E+00	PTRH1	1.68E-01	1.00E+00
SAPCD2	-2.49E+00	4.15E-30	FEZ2	-9.78E-02	1.00E+00	PGAP1	-1.14E-01	1.00E+00
FXR2	1.00E+00	4.26E-30	APLP2	8.10E-02	1.00E+00	LOC102408956	-8.71E-02	1.00E+00

SLC16A14	-5.45E+00	4.34E-30	FMNL1	1.18E-01	1.00E+00	UBAP2L	-8.74E-02	1.00E+00
ARL13B	1.32E+00	4.38E-30	CLIP3	1.10E-01	1.00E+00	BICD2	-9.50E-02	1.00E+00
VAMP8	-2.42E+00	4.44E-30	CNKSR1	7.15E-02	1.00E+00	AGPAT3	7.37E-02	1.00E+00
KIAA0391	1.11E+00	4.46E-30	GATA2	7.82E-02	1.00E+00	SLC25A10	-1.00E-01	1.00E+00
CORO1C	-1.28E+00	4.48E-30	FZD5	1.21E-01	1.00E+00	LOC102388956	-1.17E-01	1.00E+00
LOC102409669	-5.66E+00	4.50E-30	TSC2	9.73E-02	1.00E+00	USP1	-8.30E-02	1.00E+00
SRGN	-6.54E+00	4.51E-30	BRSK2	1.94E-01	1.00E+00	LOC112586285	1.92E-01	1.00E+00
LOC102391307	-1.39E+00	4.53E-30	OGG1	9.18E-02	1.00E+00	GOSR1	8.56E-02	1.00E+00
LOC102399152	-3.65E+00	4.53E-30	POT1	8.38E-02	1.00E+00	UBR4	-8.62E-02	1.00E+00
TSPYL5	-2.09E+00	4.66E-30	EIF4G3	9.71E-02	1.00E+00	HSPA13	-1.03E-01	1.00E+00
FAM184B	2.36E+00	4.81E-30	LOC102410561	2.05E-01	1.00E+00	PREB	8.29E-02	1.00E+00
FLAD1	1.56E+00	4.85E-30	C3H9orf3	-6.88E-02	1.00E+00	LOC102407989	1.89E-01	1.00E+00
MLH3	1.77E+00	4.87E-30	TMEM131L	9.41E-02	1.00E+00	RANBP17	-8.72E-02	1.00E+00
DUSP7	1.73E+00	4.90E-30	TMEM9	8.98E-02	1.00E+00	CTDP1	8.24E-02	1.00E+00
VWA8	-1.44E+00	5.01E-30	MAD1L1	6.43E-02	1.00E+00	ZBTB11	-1.12E-01	1.00E+00
LOC102398333	-7.53E+00	5.04E-30	ECSIT	1.45E-01	1.00E+00	PNPLA4	-1.11E-01	1.00E+00
MARC1	-4.60E+00	5.08E-30	CLDND1	2.08E-01	1.00E+00	FUZ	-1.20E-01	1.00E+00
STN1	1.34E+00	5.16E-30	CDIP1	8.04E-02	1.00E+00	C9H5orf24	1.16E-01	1.00E+00
TBC1D21	-5.34E+00	5.36E-30	UBE2W	-1.03E-01	1.00E+00	IER5	-1.32E-01	1.00E+00
ADCY5	4.17E+00	5.41E-30	RPRD1A	9.93E-02	1.00E+00	ZNF207	-8.71E-02	1.00E+00
GLRX2	-3.32E+00	5.49E-30	LOC102400341	-8.54E-02	1.00E+00	RASGEF1B	-1.38E-01	1.00E+00
TEX261	-1.41E+00	5.61E-30	MS4A13	-3.61E-01	1.00E+00	KLHL8	9.55E-02	1.00E+00
ERO1B	-1.36E+00	5.67E-30	BEST2	3.72E-01	1.00E+00	SLC22A20P	2.11E-01	1.00E+00
ZNF804B	1.59E+00	5.69E-30	SNURF	2.65E-01	1.00E+00	SLCO3A1	-7.97E-02	1.00E+00
SLC26A5	4.76E+00	5.72E-30	POMK	-1.40E-01	1.00E+00	STX16	8.62E-02	1.00E+00
MSRB1	-2.32E+00	5.74E-30	TMEM9B	-7.70E-02	1.00E+00	HSDL2	-9.94E-02	1.00E+00
KCNG3	-2.73E+00	5.93E-30	SPOCD1	-3.46E-01	1.00E+00	ZNF181	9.26E-02	1.00E+00
LOC112583978	4.44E+00	6.02E-30	TBC1D10B	7.82E-02	1.00E+00	LOC102401307	-3.41E-01	1.00E+00
BTC	-5.90E+00	6.02E-30	CPB2	1.92E-01	1.00E+00	SSH2	1.36E-01	1.00E+00
C9H5orf24	-2.45E+00	6.03E-30	UBR5	1.21E-01	1.00E+00	TFB2M	8.79E-02	1.00E+00
TRPC1	-3.48E+00	6.18E-30	NXF1	-7.54E-02	1.00E+00	MCM9	-9.24E-02	1.00E+00
UBA3	-1.07E+00	6.26E-30	RAMAC	-6.77E-02	1.00E+00	LOC102393808	1.28E-01	1.00E+00
C15H8orf37	-2.85E+00	6.41E-30	SERAC1	-1.31E-01	1.00E+00	SMARCE1	8.25E-02	1.00E+00

PRRT3	-2.68E+00	6.51E-30	FAT3	7.54E-02	1.00E+00	ANAPC7	-8.22E-02	1.00E+00
EXOSC9	1.27E+00	6.52E-30	KCNJ6	8.83E-02	1.00E+00	KEAP1	-8.48E-02	1.00E+00
PPP1R12B	1.38E+00	6.53E-30	TNS1	1.85E-01	1.00E+00	SOCS5	-9.66E-02	1.00E+00
TAF2	-1.47E+00	6.58E-30	TRMT12	8.29E-02	1.00E+00	LOC102408518	-2.02E-01	1.00E+00
ITIH5	-5.43E+00	6.91E-30	PSMD4	-7.33E-02	1.00E+00	PCBD2	9.99E-02	1.00E+00
KDELR1	-1.36E+00	6.97E-30	FCGBP	4.97E-01	1.00E+00	LRRC14	9.75E-02	1.00E+00
P3H2	-6.50E+00	7.00E-30	CXHXorf58	-1.50E-01	1.00E+00	PJA1	-8.50E-02	1.00E+00
LOC102394017	5.20E+00	7.18E-30	SLC6A8	8.69E-02	1.00E+00	DHRS7B	-1.10E-01	1.00E+00
LCN6	-5.45E+00	7.30E-30	PCDHB1	2.64E-01	1.00E+00	LOC112580421	1.21E-01	1.00E+00
GIGYF2	1.19E+00	7.38E-30	LOC112585799	2.21E-01	1.00E+00	ABCB10	-1.28E-01	1.00E+00
SMC3	-2.84E+00	7.63E-30	SLC26A11	1.63E-01	1.00E+00	MTM1	-1.42E-01	1.00E+00
COPS7B	1.00E+00	7.82E-30	LOC112586853	4.49E-01	1.00E+00	MFF	-7.44E-02	1.00E+00
KRT10	-4.91E+00	7.85E-30	KLF13	-1.48E-01	1.00E+00	ZNRF3	1.03E-01	1.00E+00
CAPZB	-1.60E+00	7.97E-30	DLX6	1.08E-01	1.00E+00	HCN2	1.43E-01	1.00E+00
TATDN2	1.12E+00	8.34E-30	CCDC58	-1.24E-01	1.00E+00	LMF2	-8.77E-02	1.00E+00
ZNFX1	-1.67E+00	8.52E-30	CPD	1.04E-01	1.00E+00	FGFR3	1.96E-01	1.00E+00
RABIF	-1.46E+00	8.57E-30	TMEM11	-9.46E-02	1.00E+00	MRPL9	-8.78E-02	1.00E+00
SETD1A	1.34E+00	8.66E-30	SH3BP5	8.48E-02	1.00E+00	SLC35E3	-9.63E-02	1.00E+00
PUS7L	1.42E+00	8.91E-30	TBC1D4	1.35E-01	1.00E+00	FAM219B	-1.21E-01	1.00E+00
PTPN18	-2.35E+00	8.96E-30	AP2A1	8.16E-02	1.00E+00	CLK3	-7.44E-02	1.00E+00
E2F4	1.20E+00	9.00E-30	ZFC3H1	1.67E-01	1.00E+00	GPR137B	1.36E-01	1.00E+00
TSSC4	-2.01E+00	9.02E-30	NAA35	-6.78E-02	1.00E+00	SMARCAL1	7.36E-02	1.00E+00
CENPH	-2.52E+00	9.04E-30	CCDC3	7.12E-02	1.00E+00	WDR27	1.06E-01	1.00E+00
POLB	1.12E+00	9.10E-30	ARID4B	-1.06E-01	1.00E+00	EXOSC1	-9.70E-02	1.00E+00
SEMA5A	-5.23E+00	9.19E-30	UCHL5	-9.00E-02	1.00E+00	DVL3	-8.37E-02	1.00E+00
RPS27A	-3.68E+00	9.19E-30	FAM117B	9.14E-02	1.00E+00	TRPS1	9.46E-02	1.00E+00
ARL3	-2.17E+00	9.21E-30	LOC112581638	-4.55E-01	1.00E+00	SLC11A2	-8.63E-02	1.00E+00
TESK1	-1.20E+00	9.79E-30	SAR1A	-7.12E-02	1.00E+00	MAL2	-1.53E-01	1.00E+00
ANAPC1	1.19E+00	1.01E-29	HSPBAP1	-1.52E-01	1.00E+00	NAA15	7.85E-02	1.00E+00
EMB	3.62E+00	1.03E-29	LOC112581176	-3.13E-01	1.00E+00	PTPRF	-9.11E-02	1.00E+00
RPS29	-2.77E+00	1.04E-29	LOC102412813	-3.34E-01	1.00E+00	MTPN	-1.53E-01	1.00E+00
LRRCC1	-1.04E+00	1.05E-29	BCL7C	-1.55E-01	1.00E+00	NAA16	-1.13E-01	1.00E+00
PPDPF	-	1.09E-29	PLEK2	-1.02E-	1.00E+00	PLIN5	2.37E-01	1.00E+00

	1.62E+00			01				
UBAP1L	3.26E+00	1.13E-29	ASPSCR1	-8.48E-02	1.00E+00	DDX10	8.22E-02	1.00E+00
LOC102398591	-4.61E+00	1.13E-29	C14H20orf194	-9.20E-02	1.00E+00	ADGRE1	3.24E-01	1.00E+00
FBXL16	4.08E+00	1.14E-29	RBM3	-1.31E-01	1.00E+00	CDC37L1	-9.35E-02	1.00E+00
FANCD2OS	5.69E+00	1.15E-29	GUCY2F	-3.36E-01	1.00E+00	LAGE3	-1.05E-01	1.00E+00
RNF144A	-5.89E+00	1.15E-29	ICMT	8.87E-02	1.00E+00	KLHDC1	-1.49E-01	1.00E+00
AAED1	-2.41E+00	1.16E-29	RBM24	-3.39E-01	1.00E+00	TTC9C	-9.52E-02	1.00E+00
NTMT1	-1.89E+00	1.22E-29	SLC35B4	-8.18E-02	1.00E+00	ARFGEF3	-1.78E-01	1.00E+00
SPAG1	-2.53E+00	1.23E-29	RIT1	-6.66E-02	1.00E+00	YTHDC1	-8.48E-02	1.00E+00
PSMC4	-1.32E+00	1.23E-29	ARL8B	-6.79E-02	1.00E+00	FUT10	8.83E-02	1.00E+00
SORCS3	5.86E+00	1.24E-29	MAP1A	1.66E-01	1.00E+00	MAPK8IP3	7.73E-02	1.00E+00
XDH	-3.20E+00	1.25E-29	GTF2E2	7.98E-02	1.00E+00	ITGA2B	-1.60E-01	1.00E+00
CHRM3	2.41E+00	1.26E-29	PTPN1	-7.28E-02	1.00E+00	TMEM134	-1.29E-01	1.00E+00
LOC102394666	-2.16E+00	1.27E-29	PRRG4	-1.42E-01	1.00E+00	TM2D2	-8.13E-02	1.00E+00
LOC112586534	-4.36E+00	1.27E-29	LOC102391562	2.04E-01	1.00E+00	WARS	8.13E-02	1.00E+00
ANKRD50	-5.15E+00	1.27E-29	DACT1	-3.65E-01	1.00E+00	COQ5	9.22E-02	1.00E+00
GPA33	5.90E+00	1.28E-29	LOC112578167	3.27E-01	1.00E+00	TRIB3	-2.01E-01	1.00E+00
NRXN1	4.93E+00	1.29E-29	FKBP6	9.09E-02	1.00E+00	FER	9.88E-02	1.00E+00
GPRC5C	2.95E+00	1.29E-29	GANC	2.20E-01	1.00E+00	MRPL27	1.33E-01	1.00E+00
LOC112586879	6.13E+00	1.30E-29	VRTN	-1.43E-01	1.00E+00	NAA20	7.73E-02	1.00E+00
ID4	-3.59E+00	1.31E-29	MACROD2	-8.61E-02	1.00E+00	LMTK2	1.35E-01	1.00E+00
MTPN	2.07E+00	1.31E-29	LOC102401686	1.34E-01	1.00E+00	SPR	-1.11E-01	1.00E+00
NAGLU	-4.41E+00	1.32E-29	LYRM1	-1.46E-01	1.00E+00	GPN3	-9.31E-02	1.00E+00
FTSJ3	1.25E+00	1.32E-29	OPTN	-6.86E-02	1.00E+00	GLRX2	1.26E-01	1.00E+00
RPS7	-2.71E+00	1.32E-29	PLA2G2F	3.04E-01	1.00E+00	MYOM3	1.53E-01	1.00E+00
RRAGB	-2.09E+00	1.32E-29	ZNF398	1.10E-01	1.00E+00	KLHL26	-1.13E-01	1.00E+00
LOC112579143	6.16E+00	1.34E-29	ATRN	1.04E-01	1.00E+00	TK1	-1.20E-01	1.00E+00
NAP1L1	-1.32E+00	1.34E-29	CENPT	-7.27E-02	1.00E+00	EXOSC8	-1.06E-01	1.00E+00
NPR3	-5.32E+00	1.36E-29	CREG2	2.87E-01	1.00E+00	PLCXD1	-1.17E-01	1.00E+00
GNMT	1.94E+00	1.41E-29	CSNK1G3	1.25E-01	1.00E+00	LOC102409673	-1.17E-01	1.00E+00
AATF	-1.61E+00	1.47E-29	RNF25	-7.84E-02	1.00E+00	KIF27	-9.20E-02	1.00E+00
DDX31	-9.89E-01	1.50E-29	DENND6A	2.56E-01	1.00E+00	NUP43	-1.02E-01	1.00E+00
MSMB	-6.78E+00	1.51E-29	GOLGA3	1.32E-01	1.00E+00	MDH1B	-1.18E-01	1.00E+00
CD3E	5.14E+00	1.55E-29	STPG1	4.27E-01	1.00E+00	RSBN1L	-9.30E-	1.00E+00

							02	
SLC9A3R2	-3.72E+00	1.58E-29	ZNF646	1.26E-01	1.00E+00	PANX2	1.69E-01	1.00E+00
MAP1LC3A	-1.74E+00	1.60E-29	PCDH9	1.72E-01	1.00E+00	HEATR5B	-1.35E-01	1.00E+00
LYL1	-5.66E+00	1.68E-29	PLG	-2.42E-01	1.00E+00	ARHGAP8	-1.27E-01	1.00E+00
PSKH1	1.39E+00	1.69E-29	RWDD4	-1.25E-01	1.00E+00	GIGYF1	-8.30E-02	1.00E+00
LOC102389131	8.13E+00	1.71E-29	PTPDC1	4.67E-01	1.00E+00	EGFR	9.96E-02	1.00E+00
TRMT1	-1.86E+00	1.75E-29	INTS12	7.42E-02	1.00E+00	CLK4	-8.36E-02	1.00E+00
NALCN	-4.81E+00	1.78E-29	LCT	2.80E-01	1.00E+00	AP1G1	8.19E-02	1.00E+00
STK25	-1.59E+00	1.78E-29	AARS2	1.98E-01	1.00E+00	TM6SF1	8.68E-02	1.00E+00
LOC102411942	4.69E+00	1.81E-29	INAFM1	-1.46E-01	1.00E+00	CHTOP	-8.97E-02	1.00E+00
ECHDC1	-1.32E+00	1.84E-29	CFAP65	-2.17E-01	1.00E+00	PPP1CA	9.28E-02	1.00E+00
LOC102411883	-5.06E+00	1.85E-29	LOC112583743	-5.90E-01	1.00E+00	DNAJC7	-8.51E-02	1.00E+00
LOC112579629	4.15E+00	1.85E-29	C4H12orf73	-1.05E-01	1.00E+00	C5H11orf95	-8.97E-02	1.00E+00
FASTKD3	1.82E+00	1.86E-29	LOC102392547	-1.92E-01	1.00E+00	CLUH	-7.85E-02	1.00E+00
UBE4A	-1.20E+00	1.87E-29	LATS2	2.58E-01	1.00E+00	TMEM63B	8.09E-02	1.00E+00
PNMA6E	4.74E+00	1.93E-29	STAT1	7.96E-02	1.00E+00	ARL5A	8.54E-02	1.00E+00
CLBA1	-2.07E+00	1.95E-29	GPR84	1.45E-01	1.00E+00	GMFG	-3.18E-01	1.00E+00
ZCCHC12	1.82E+00	2.02E-29	SHROOM2	8.81E-02	1.00E+00	C22H18orf32	8.48E-02	1.00E+00
PPP2R2B	4.74E+00	2.03E-29	CRY2	-1.06E-01	1.00E+00	THBD	-3.03E-01	1.00E+00
VSTM2A	3.58E+00	2.07E-29	ELP4	-1.22E-01	1.00E+00	ATXN7L1	-8.90E-02	1.00E+00
JDP2	-5.06E+00	2.07E-29	CNPY2	-8.09E-02	1.00E+00	ACADS	-9.26E-02	1.00E+00
TMEM136	-4.53E+00	2.09E-29	MRPL20	-8.68E-02	1.00E+00	NPL	3.60E-01	1.00E+00
PRMT6	-1.12E+00	2.10E-29	SMC2	8.97E-02	1.00E+00	FLAD1	-1.01E-01	1.00E+00
DUSP1	-2.89E+00	2.17E-29	PNPLA6	-8.95E-02	1.00E+00	LAPTM4A	-8.05E-02	1.00E+00
LOC102400283	-6.25E+00	2.18E-29	AP2S1	-1.11E-01	1.00E+00	FAM110A	-1.33E-01	1.00E+00
LOC102408258	2.02E+00	2.23E-29	MUC19	2.20E-01	1.00E+00	ALG12	1.04E-01	1.00E+00
LOC112586503	-6.33E+00	2.25E-29	CCDC146	-8.14E-02	1.00E+00	LOC102399617	1.03E-01	1.00E+00
NOP16	-2.18E+00	2.25E-29	IP6K2	-6.93E-02	1.00E+00	LOC102411901	8.75E-02	1.00E+00
WARS2	1.76E+00	2.30E-29	SEC62	-7.08E-02	1.00E+00	VPS35L	7.41E-02	1.00E+00
NCSTN	-1.03E+00	2.32E-29	PIP4P1	-8.47E-02	1.00E+00	CTBP2	7.70E-02	1.00E+00
LOC112585052	-3.30E+00	2.34E-29	LOC102416251	2.41E-01	1.00E+00	GRWD1	9.76E-02	1.00E+00
LRCH4	1.12E+00	2.41E-29	LOC102398554	-1.21E-01	1.00E+00	MYH6	2.02E-01	1.00E+00
FAM76A	-1.92E+00	2.46E-29	OOSP2	-4.38E-01	1.00E+00	LOC102412047	1.92E-01	1.00E+00
LOC112578799	6.89E+00	2.51E-29	PGAP1	1.28E-01	1.00E+00	HAUS8	-1.18E-01	1.00E+00

LOC102410916	-3.90E+00	2.53E-29	MEIOB	-3.41E-01	1.00E+00	TYW5	-1.22E-01	1.00E+00
TENM4	2.22E+00	2.57E-29	BCAT2	-1.72E-01	1.00E+00	AHSA2P	8.81E-02	1.00E+00
PLSCR4	-5.41E+00	2.59E-29	LOC102400631	-1.41E-01	1.00E+00	SNRNP40	-7.52E-02	1.00E+00
COL6A2	-4.74E+00	2.73E-29	DDIAS	8.64E-02	1.00E+00	CHMP2A	1.04E-01	1.00E+00
LOC102400714	-1.50E+00	2.74E-29	RGS7	1.79E-01	1.00E+00	SEMA4D	-7.86E-02	1.00E+00
LOC102411899	4.54E+00	2.77E-29	SRPRB	-1.16E-01	1.00E+00	RBBP6	-9.36E-02	1.00E+00
CAMSAP2	-1.74E+00	2.78E-29	CHRM3	9.28E-02	1.00E+00	RIMKLB	-7.97E-02	1.00E+00
KLHL36	1.01E+00	2.82E-29	GSTA4	-2.80E-01	1.00E+00	DAZAP1	-7.51E-02	1.00E+00
EGF	5.23E+00	2.87E-29	HOXA9	-2.06E-01	1.00E+00	DHX30	-7.93E-02	1.00E+00
KIF21A	-4.66E+00	2.89E-29	LOC102389538	3.48E-01	1.00E+00	FZD4	-1.62E-01	1.00E+00
LOC102398227	-4.92E+00	2.95E-29	PCP4	-4.69E-01	1.00E+00	NCKAP1L	3.55E-01	1.00E+00
LOC112586630	5.79E+00	3.01E-29	PTGES2	-1.86E-01	1.00E+00	RGN	-1.65E-01	1.00E+00
LSM10	1.51E+00	3.02E-29	MAP2K5	1.53E-01	1.00E+00	CCDC88B	-1.23E-01	1.00E+00
LOC112587937	5.44E+00	3.02E-29	PPP1R9A	-1.53E-01	1.00E+00	GALC	1.85E-01	1.00E+00
ALYREF	-1.63E+00	3.06E-29	SMARCD1	7.77E-02	1.00E+00	LOC102414386	-1.00E-01	1.00E+00
UTP4	1.05E+00	3.11E-29	LOC112587765	2.15E-01	1.00E+00	ARHGEF18	-7.41E-02	1.00E+00
GCNT1	1.45E+00	3.22E-29	GCFC2	1.41E-01	1.00E+00	SLFNL1	-1.61E-01	1.00E+00
LOC102390247	3.68E+00	3.25E-29	LYSMD2	1.81E-01	1.00E+00	GDPD3	1.59E-01	1.00E+00
MINPP1	-2.03E+00	3.33E-29	DAXX	1.00E-01	1.00E+00	SELENOK	-1.02E-01	1.00E+00
ZNF557	-2.20E+00	3.34E-29	PHYHIPL	-7.41E-02	1.00E+00	LOC102415700	-1.05E-01	1.00E+00
ALDH1B1	3.15E+00	3.36E-29	AASDHPPT	8.02E-02	1.00E+00	ZC3HC1	8.62E-02	1.00E+00
HNRNPH2	-1.09E+00	3.41E-29	CROT	-6.88E-02	1.00E+00	SPSB4	1.78E-01	1.00E+00
LOC102415752	-1.97E+00	3.43E-29	ATOX1	-1.35E-01	1.00E+00	CIDEB	1.00E-01	1.00E+00
LOC112583862	-5.57E+00	3.48E-29	SSR3	7.83E-02	1.00E+00	ARL6IP4	1.22E-01	1.00E+00
LOC112580906	5.71E+00	3.51E-29	RALGPS2	1.05E-01	1.00E+00	AK1	1.38E-01	1.00E+00
LOC102401443	2.04E+00	3.57E-29	HSPB6	-1.56E-01	1.00E+00	C1GALT1C1	-8.43E-02	1.00E+00
PLAT	-2.74E+00	3.60E-29	ID3	-9.90E-02	1.00E+00	EXOC3	-7.22E-02	1.00E+00
USP24	-1.62E+00	3.62E-29	PXDC1	3.67E-01	1.00E+00	DEK	-8.23E-02	1.00E+00
RSPH14	3.89E+00	3.77E-29	NUDT3	-6.87E-02	1.00E+00	MRPL28	1.19E-01	1.00E+00
CMTM5	-4.70E+00	3.77E-29	LOC102409347	6.38E-02	1.00E+00	NBAS	-7.17E-02	1.00E+00
FOSL2	-1.72E+00	3.78E-29	LOC112581451	-1.96E-01	1.00E+00	MALL	-1.81E-01	1.00E+00
LOC112582182	7.58E+00	3.87E-29	YTHDF1	7.12E-02	1.00E+00	ABHD1	1.62E-01	1.00E+00
CHKA	-1.15E+00	3.88E-29	NSUN6	7.57E-02	1.00E+00	SCYL2	1.01E-01	1.00E+00
LOC102389068	-	3.89E-29	IGFLR1	-1.28E-	1.00E+00	CASK	9.05E-02	1.00E+00

	2.24E+00			01				
WNK4	2.51E+00	3.96E-29	RPP25L	-4.82E-01	1.00E+00	LYN	3.34E-01	1.00E+00
GJC1	1.19E+00	3.98E-29	IMPG2	4.33E-01	1.00E+00	PAXBP1	-9.51E-02	1.00E+00
LOC112580684	4.39E+00	4.01E-29	CPT1B	3.24E-01	1.00E+00	TSTD1	1.14E-01	1.00E+00
TTI1	1.20E+00	4.10E-29	RPS21	-1.96E-01	1.00E+00	RRAS2	9.44E-02	1.00E+00
RPS15A	-3.83E+00	4.11E-29	PRPSAP1	-6.80E-02	1.00E+00	PIBF1	9.62E-02	1.00E+00
HELLS	1.15E+00	4.24E-29	ANKRD13A	8.36E-02	1.00E+00	TYW3	1.04E-01	1.00E+00
LOC112579564	5.47E+00	4.30E-29	PHF20L1	-2.08E-01	1.00E+00	FAM20B	-8.15E-02	1.00E+00
LOC102398949	3.46E+00	4.32E-29	LOC112579979	-3.77E-01	1.00E+00	ANP32A	-1.00E-01	1.00E+00
LOC112583766	6.97E+00	4.35E-29	ABCD1	1.45E-01	1.00E+00	C21H3orf14	-1.15E-01	1.00E+00
LOC102398127	4.84E+00	4.35E-29	GPSM2	1.23E-01	1.00E+00	RCN1	-9.10E-02	1.00E+00
LOC102414928	-5.80E+00	4.58E-29	ELOVL5	7.07E-02	1.00E+00	VCPIP1	1.14E-01	1.00E+00
PRR36	1.82E+00	4.79E-29	TMEM219	8.91E-02	1.00E+00	LOC102391085	-9.90E-02	1.00E+00
SLC35G2	2.50E+00	4.89E-29	FANCG	-1.48E-01	1.00E+00	BANP	1.01E-01	1.00E+00
USH2A	6.13E+00	4.90E-29	B3GALT6	8.44E-02	1.00E+00	ADGRA2	1.52E-01	1.00E+00
KIF19	-5.03E+00	4.92E-29	MRPL46	1.35E-01	1.00E+00	PUS1	1.05E-01	1.00E+00
FBXL15	-2.65E+00	4.96E-29	TNK2	7.93E-02	1.00E+00	VPS28	-1.08E-01	1.00E+00
LOC102404419	-8.11E+00	4.97E-29	DLG1	1.09E-01	1.00E+00	KMT2C	-1.10E-01	1.00E+00
VPS39	-1.06E+00	4.98E-29	LOC112582206	1.80E-01	1.00E+00	NSL1	-1.09E-01	1.00E+00
NEK9	-1.64E+00	4.99E-29	LOC102393852	7.44E-02	1.00E+00	PCNX3	-7.49E-02	1.00E+00
HTT	-1.54E+00	5.03E-29	NSUN3	9.34E-02	1.00E+00	ABR	-7.16E-02	1.00E+00
ANKS3	-1.55E+00	5.08E-29	TSHZ2	-1.23E-01	1.00E+00	VCAN	-1.52E-01	1.00E+00
BAG5	-2.35E+00	5.32E-29	PTPN12	2.07E-01	1.00E+00	FBN1	1.23E-01	1.00E+00
FAM166B	-5.25E+00	5.39E-29	STMP1	-7.50E-02	1.00E+00	STAT1	9.64E-02	1.00E+00
LOC102395434	5.05E+00	5.42E-29	HM13	7.95E-02	1.00E+00	MIEN1	1.15E-01	1.00E+00
ZMYND19	1.74E+00	5.46E-29	OSMR	-1.95E-01	1.00E+00	RAMP1	-1.99E-01	1.00E+00
TRNAR-ACG-12	6.45E+00	5.47E-29	TBC1D15	7.13E-02	1.00E+00	RPL24	1.13E-01	1.00E+00
KLK5	-8.68E+00	5.50E-29	VDAC2	-1.01E-01	1.00E+00	WDR55	8.99E-02	1.00E+00
ACSL5	4.19E+00	5.61E-29	LRFN3	2.10E-01	1.00E+00	LATS2	-1.61E-01	1.00E+00
CHD6	-1.50E+00	5.65E-29	TNNI3	-3.35E-01	1.00E+00	PLEKHG1	1.03E-01	1.00E+00
USP49	1.96E+00	5.71E-29	LOC102403358	-2.34E-01	1.00E+00	RNPC3	-1.36E-01	1.00E+00
SLC30A7	-1.86E+00	5.76E-29	LOC112582272	-2.58E-01	1.00E+00	STRIP1	7.52E-02	1.00E+00
KIAA0408	-3.87E+00	5.77E-29	LOC102400360	3.33E-01	1.00E+00	GMEB2	-7.37E-02	1.00E+00
CACYBP	-1.30E+00	5.80E-29	PCBP3	-3.27E-01	1.00E+00	TMX1	-7.75E-02	1.00E+00

WDR27	-2.65E+00	5.81E-29	LOC102413429	-3.77E-01	1.00E+00	KDM1A	7.45E-02	1.00E+00
PCGF5	1.29E+00	5.98E-29	LOC112584007	3.61E-01	1.00E+00	EMID1	1.29E-01	1.00E+00
DHRS12	2.09E+00	6.00E-29	SEZ6L	2.32E-01	1.00E+00	ITGA10	-1.52E-01	1.00E+00
ZNF446	-2.20E+00	6.02E-29	SCAND1	-1.10E-01	1.00E+00	NCL	-1.18E-01	1.00E+00
CFP	-4.16E+00	6.09E-29	CLGN	-9.91E-02	1.00E+00	LOC102395897	-9.34E-02	1.00E+00
PPIF	1.59E+00	6.19E-29	LOC112584193	-1.28E-01	1.00E+00	KIAA1109	-1.11E-01	1.00E+00
RHBG	-4.52E+00	6.22E-29	PLTP	-4.20E-01	1.00E+00	PDPN	1.30E-01	1.00E+00
LOC102413318	-6.69E+00	6.34E-29	RPL26	-1.61E-01	1.00E+00	LOC102394468	1.57E-01	1.00E+00
GLUD1	-1.07E+00	6.34E-29	TMC5	4.62E-01	1.00E+00	SNX16	-1.23E-01	1.00E+00
TEFM	1.72E+00	6.65E-29	DEPDC5	8.77E-02	1.00E+00	BAX	9.65E-02	1.00E+00
LRRC32	3.41E+00	6.68E-29	KCNK7	-1.45E-01	1.00E+00	TMC8	-1.47E-01	1.00E+00
LOC102399535	-3.22E+00	6.76E-29	IGSF9B	1.12E-01	1.00E+00	SPC25	-1.35E-01	1.00E+00
LOC112581646	4.41E+00	6.77E-29	LOC112587870	-1.46E-01	1.00E+00	SSR1	-8.50E-02	1.00E+00
C5H11orf95	-1.50E+00	6.88E-29	LOC112583970	1.47E-01	1.00E+00	CAB39	7.59E-02	1.00E+00
MTERF3	1.08E+00	7.08E-29	LOC112581736	2.74E-01	1.00E+00	PRCP	8.72E-02	1.00E+00
LOC112579940	-5.57E+00	7.09E-29	PUS7	2.88E-01	1.00E+00	TBCCD1	-8.63E-02	1.00E+00
OLFML1	4.08E+00	7.10E-29	TAB2	9.43E-02	1.00E+00	ITFG2	-8.94E-02	1.00E+00
PBXIP1	-1.09E+00	7.18E-29	ADGRD2	-4.21E-01	1.00E+00	PPIB	-1.34E-01	1.00E+00
PAPOLA	1.08E+00	7.18E-29	C9H19orf66	-1.04E-01	1.00E+00	LOC102397769	-1.77E-01	1.00E+00
RNF135	-5.34E+00	7.27E-29	FAM189B	9.99E-02	1.00E+00	MAP4K5	9.68E-02	1.00E+00
REELD1	-5.01E+00	7.57E-29	PTCD3	-9.55E-02	1.00E+00	B4GALT6	1.13E-01	1.00E+00
RPL23A	-1.86E+00	7.60E-29	LOC102415917	-9.43E-02	1.00E+00	USP45	-1.76E-01	1.00E+00
DIS3L	-1.17E+00	7.71E-29	ACVR1	9.87E-02	1.00E+00	GGA2	7.34E-02	1.00E+00
ABHD6	1.29E+00	7.88E-29	FGD2	1.27E-01	1.00E+00	PPP5C	-8.45E-02	1.00E+00
PNPLA2	-1.67E+00	8.18E-29	MTERF3	-9.40E-02	1.00E+00	DNLZ	1.20E-01	1.00E+00
PTER	-4.04E+00	8.24E-29	ZNF12	1.97E-01	1.00E+00	TP53RK	9.06E-02	1.00E+00
DNM2	-1.10E+00	8.26E-29	EXT2	-6.38E-02	1.00E+00	C6H1orf43	-8.60E-02	1.00E+00
IQANK1	-3.53E+00	8.33E-29	ENTPD6	8.27E-02	1.00E+00	CCNG1	-8.04E-02	1.00E+00
MMS19	1.14E+00	8.38E-29	EIF3E	-6.94E-02	1.00E+00	DNAJC14	7.24E-02	1.00E+00
DAB2IP	1.51E+00	8.56E-29	TP63	1.21E-01	1.00E+00	STX18	8.19E-02	1.00E+00
SLC25A42	1.64E+00	8.57E-29	JAKMIP2	1.44E-01	1.00E+00	C24H16orf96	-9.94E-02	1.00E+00
ASAP2	1.17E+00	8.61E-29	ISL2	-1.45E-01	1.00E+00	PPP2CA	-7.94E-02	1.00E+00
MARVELD1	-5.51E+00	8.61E-29	RAD52	-7.54E-02	1.00E+00	ESRP2	8.78E-02	1.00E+00
LOC102399369	-	8.67E-29	PPEF1	-9.11E-	1.00E+00	LOC102405595	2.05E-01	1.00E+00

	3.28E+00			02				
TMEM160	2.46E+00	8.71E-29	INSM1	5.05E-01	1.00E+00	LOC112584575	1.66E-01	1.00E+00
NIFK	1.06E+00	8.81E-29	RPS9	-1.02E-01	1.00E+00	TPRKB	-9.72E-02	1.00E+00
FAM45A	-1.28E+00	8.87E-29	SLC6A11	-1.56E-01	1.00E+00	SYNJ2BP	-7.97E-02	1.00E+00
LOC102403789	-1.66E+00	9.11E-29	C15H8orf88	2.13E-01	1.00E+00	TKFC	-9.81E-02	1.00E+00
TPRA1	-1.52E+00	9.15E-29	PIGF	-1.58E-01	1.00E+00	TEN1	9.49E-02	1.00E+00
PRKG1	3.37E+00	9.16E-29	DNAH11	-6.64E-02	1.00E+00	FGFR1OP	-8.56E-02	1.00E+00
C9H19orf25	-2.45E+00	9.23E-29	SLC35C1	1.03E-01	1.00E+00	PUDP	-8.37E-02	1.00E+00
GCN1	-1.28E+00	9.24E-29	LOC102414721	-2.73E-01	1.00E+00	KIAA1191	-8.34E-02	1.00E+00
LOC112587477	7.33E+00	9.29E-29	PAM16	1.21E-01	1.00E+00	NUP58	-9.83E-02	1.00E+00
JUNB	-6.66E+00	9.38E-29	NEURL3	-9.39E-02	1.00E+00	TUBB	1.23E-01	1.00E+00
HHIPL2	4.79E+00	9.46E-29	CARS2	-7.78E-02	1.00E+00	GJA4	1.23E-01	1.00E+00
SAP130	1.30E+00	9.68E-29	MRPS18C	-1.01E-01	1.00E+00	C2H6orf10	2.04E-01	1.00E+00
SVOPL	1.77E+00	9.84E-29	KLF4	1.25E-01	1.00E+00	FRK	-1.07E-01	1.00E+00
LRP1B	5.83E+00	9.89E-29	ZNF518A	1.37E-01	1.00E+00	LOC102408905	3.37E-01	1.00E+00
PLOD1	-6.51E+00	1.03E-28	GFAP	2.17E-01	1.00E+00	ZBTB12	-9.49E-02	1.00E+00
PTPRN	-4.52E+00	1.03E-28	CEP104	-9.22E-02	1.00E+00	ZNF260	1.10E-01	1.00E+00
TOR1AIP1	-2.15E+00	1.03E-28	CALCOCO2	6.75E-02	1.00E+00	LOC102415243	-1.66E-01	1.00E+00
PGBD2	-3.33E+00	1.04E-28	SSC5D	-1.55E-01	1.00E+00	SON	-1.09E-01	1.00E+00
LOC112585148	-4.46E+00	1.06E-28	REEP4	7.69E-02	1.00E+00	QRSL1	8.70E-02	1.00E+00
ERF	-1.84E+00	1.06E-28	LOC102405260	8.55E-02	1.00E+00	NHLRC3	-9.63E-02	1.00E+00
LOC112585160	-4.50E+00	1.08E-28	GDAP2	6.49E-02	1.00E+00	MPP1	8.99E-02	1.00E+00
FIS1	-1.76E+00	1.08E-28	LOC112578777	1.80E-01	1.00E+00	OPRL1	-2.17E-01	1.00E+00
FKBP8	-1.61E+00	1.10E-28	NOX5	1.40E-01	1.00E+00	EARS2	-1.47E-01	1.00E+00
PIWIL1	6.43E+00	1.15E-28	MAP3K3	1.16E-01	1.00E+00	C1D	-7.37E-02	1.00E+00
CH25H	-4.96E+00	1.16E-28	REXO2	-8.24E-02	1.00E+00	TRIM2	-8.36E-02	1.00E+00
NISCH	1.21E+00	1.17E-28	ZGRF1	-1.17E-01	1.00E+00	AK3	7.86E-02	1.00E+00
UBR4	-1.37E+00	1.17E-28	MRM3	-7.68E-02	1.00E+00	MFSD13A	-1.01E-01	1.00E+00
FAM180B	-4.62E+00	1.17E-28	LOC112582010	-1.21E-01	1.00E+00	MLYCD	1.04E-01	1.00E+00
TBX6	1.91E+00	1.18E-28	KCTD1	-1.32E-01	1.00E+00	LOC102404419	2.06E-01	1.00E+00
FAM92A	-1.47E+00	1.19E-28	PHF3	-1.44E-01	1.00E+00	RUSC1	-8.72E-02	1.00E+00
LOC102405455	3.47E+00	1.20E-28	LOC102407725	1.68E-01	1.00E+00	CDC20	-1.43E-01	1.00E+00
UNK	1.29E+00	1.22E-28	SLC4A1AP	6.68E-02	1.00E+00	LOC112586445	-1.56E-01	1.00E+00

SPRYD4	-1.88E+00	1.24E-28	HBA1	1.55E-01	1.00E+00	TES	-1.44E-01	1.00E+00
LOC102399077	4.38E+00	1.27E-28	PLCXD2	6.10E-01	1.00E+00	CCDC155	-1.81E-01	1.00E+00
FZD2	3.50E+00	1.27E-28	LOC102405290	-6.23E-01	1.00E+00	LOC102389307	-1.67E-01	1.00E+00
TSACC	5.19E+00	1.29E-28	NCKAP1L	-2.36E-01	1.00E+00	AHSA1	8.22E-02	1.00E+00
MIA	-3.19E+00	1.30E-28	LOC102391771	2.17E-01	1.00E+00	SPRY3	1.26E-01	1.00E+00
FASTKD1	-1.50E+00	1.31E-28	ADSSL1	-3.30E-01	1.00E+00	ATP9B	-7.57E-02	1.00E+00
YEATS2	-1.50E+00	1.32E-28	TET2	-8.71E-02	1.00E+00	LOC102416626	-2.29E-01	1.00E+00
LOC102405233	-7.51E+00	1.34E-28	SPAG16	-2.21E-01	1.00E+00	PNO1	7.76E-02	1.00E+00
OAS1	-4.95E+00	1.37E-28	MTTP	3.47E-01	1.00E+00	EMP2	-7.47E-02	1.00E+00
RNASEH2B	-1.77E+00	1.38E-28	PFKP	3.09E-01	1.00E+00	JCAD	1.01E-01	1.00E+00
UBE2QL1	-4.33E+00	1.38E-28	PDZRN4	1.51E-01	1.00E+00	RPGR	1.05E-01	1.00E+00
VILL	-5.80E+00	1.40E-28	CHRNA1	-1.41E-01	1.00E+00	KIDINS220	-8.55E-02	1.00E+00
PHKA2	1.49E+00	1.41E-28	AS3MT	-1.28E-01	1.00E+00	ING5	-9.41E-02	1.00E+00
ZXDC	-1.38E+00	1.45E-28	GPR180	3.00E-01	1.00E+00	TRPA1	1.59E-01	1.00E+00
LOC102404867	-4.83E+00	1.45E-28	AMHR2	-4.02E-01	1.00E+00	NUDT18	1.18E-01	1.00E+00
SHMT2	-1.43E+00	1.48E-28	SS18L1	-2.63E-01	1.00E+00	HOXC8	2.45E-01	1.00E+00
EFHD1	1.84E+00	1.50E-28	DCTN6	-1.03E-01	1.00E+00	DNAH1	-1.54E-01	1.00E+00
CIAO3	-1.45E+00	1.50E-28	HNRNPDL	8.16E-02	1.00E+00	KCNIP2	-2.42E-01	1.00E+00
PYGO1	-1.78E+00	1.50E-28	TENT5C	9.23E-02	1.00E+00	LOC102397090	-1.73E-01	1.00E+00
PRKD2	-1.83E+00	1.54E-28	LOC102394286	2.01E-01	1.00E+00	EED	-7.59E-02	1.00E+00
LOC102408787	6.00E+00	1.58E-28	PCYOX1L	1.59E-01	1.00E+00	ARAP1	-7.60E-02	1.00E+00
TAF1	-2.24E+00	1.60E-28	DOC2B	1.26E-01	1.00E+00	MMAA	-9.95E-02	1.00E+00
LOC102416251	4.20E+00	1.63E-28	SLC25A20	-8.56E-02	1.00E+00	RASGRP1	1.69E-01	1.00E+00
GAS2L2	5.63E+00	1.63E-28	RILPL1	-9.21E-02	1.00E+00	VWA8	-7.31E-02	1.00E+00
KANSL1L	-2.09E+00	1.66E-28	ATG2B	1.42E-01	1.00E+00	RANBP3L	2.14E-01	1.00E+00
PCSK2	-3.37E+00	1.67E-28	JMJD1C	-8.78E-02	1.00E+00	LOC112587546	-1.77E-01	1.00E+00
ERCC6L	1.40E+00	1.67E-28	STK35	7.19E-02	1.00E+00	MIB1	9.92E-02	1.00E+00
GCNA	-3.89E+00	1.70E-28	GALNT3	-2.48E-01	1.00E+00	P2RY2	1.78E-01	1.00E+00
ATP5ME	-2.25E+00	1.73E-28	CFAP161	4.73E-01	1.00E+00	CUL4B	-1.01E-01	1.00E+00
RSAD1	-3.17E+00	1.76E-28	TRA2A	8.90E-02	1.00E+00	SRSF7	-8.60E-02	1.00E+00
SERPINB1	-5.28E+00	1.77E-28	PRAG1	1.19E-01	1.00E+00	RPS2	-1.49E-01	1.00E+00
NKAIN1	2.69E+00	1.82E-28	CCDC178	3.04E-01	1.00E+00	LMBR1L	-9.06E-02	1.00E+00
UNC93B1	-	1.85E-28	KLHL26	-3.26E-	1.00E+00	SEPHS2	7.90E-02	1.00E+00

	4.02E+00			01				
LOC112578798	-1.79E+00	1.86E-28	CCDC57	1.17E-01	1.00E+00	ARHGAP39	-9.16E-02	1.00E+00
SLC7A14	4.53E+00	1.86E-28	IFT88	-7.25E-02	1.00E+00	ABI1	-8.07E-02	1.00E+00
BORCS7	-3.18E+00	1.89E-28	LOC102389575	8.41E-02	1.00E+00	SLC4A5	-2.30E-01	1.00E+00
SYT16	4.90E+00	1.89E-28	LOC102398085	-3.71E-01	1.00E+00	NDUFS1	-7.52E-02	1.00E+00
LOC112579148	2.93E+00	1.93E-28	WNT9A	-2.15E-01	1.00E+00	LUC7L	-8.68E-02	1.00E+00
LENG8	-1.76E+00	1.95E-28	ARHGAP21	1.75E-01	1.00E+00	DIABLO	9.01E-02	1.00E+00
MGAM2	8.00E+00	1.95E-28	TENT4A	-1.11E-01	1.00E+00	ZBTB25	-1.24E-01	1.00E+00
KIRREL3	-2.50E+00	2.02E-28	C24H16orf58	-2.11E-01	1.00E+00	PKP2	-9.77E-02	1.00E+00
KLB	2.85E+00	2.05E-28	STAG2	-1.70E-01	1.00E+00	NUP35	-8.77E-02	1.00E+00
RTEL1	-1.32E+00	2.09E-28	PJVK	-2.79E-01	1.00E+00	CA2	1.50E-01	1.00E+00
P2RX2	4.17E+00	2.13E-28	LOC102403961	-1.86E-01	1.00E+00	KCNG3	1.42E-01	1.00E+00
LOC112580904	7.78E+00	2.15E-28	LOC102399180	-2.92E-01	1.00E+00	FAM184A	-9.13E-02	1.00E+00
FGB	3.21E+00	2.17E-28	MUS81	8.13E-02	1.00E+00	ATP13A1	-7.35E-02	1.00E+00
TMEM119	3.75E+00	2.22E-28	CEP19	-2.51E-01	1.00E+00	PFAS	-9.13E-02	1.00E+00
AKNA	-3.94E+00	2.25E-28	ZNF132	-1.20E-01	1.00E+00	KMT5B	-8.41E-02	1.00E+00
LOC102403213	-5.64E+00	2.27E-28	DGKA	9.61E-02	1.00E+00	BRCA2	-1.10E-01	1.00E+00
ATF5	-2.55E+00	2.30E-28	ZNF316	2.42E-01	1.00E+00	TIAL1	-7.13E-02	1.00E+00
ZNF25	-4.61E+00	2.37E-28	TINF2	6.22E-02	1.00E+00	TBC1D19	8.58E-02	1.00E+00
LOC112581374	-7.58E+00	2.39E-28	FOXP2	-2.35E-01	1.00E+00	TSPAN7	1.22E-01	1.00E+00
PLG	3.74E+00	2.43E-28	LOC112582094	-3.29E-01	1.00E+00	ABCA1	2.25E-01	1.00E+00
WFDC1	-2.97E+00	2.45E-28	RPP21	9.75E-02	1.00E+00	GINS3	-1.13E-01	1.00E+00
GLT8D1	-1.34E+00	2.45E-28	TMEM165	1.50E-01	1.00E+00	PRICKLE4	-1.30E-01	1.00E+00
MTMR10	-1.29E+00	2.45E-28	TRPC4AP	6.94E-02	1.00E+00	LOC112580305	-1.20E-01	1.00E+00
LOC112585315	-6.29E+00	2.48E-28	UBE2V2	-8.15E-02	1.00E+00	ANKFY1	9.18E-02	1.00E+00
KDM8	-2.05E+00	2.49E-28	NAXE	-9.05E-02	1.00E+00	CASC3	-7.19E-02	1.00E+00
BMS1	1.00E+00	2.59E-28	AMBRA1	7.22E-02	1.00E+00	ADAM15	-9.70E-02	1.00E+00
CBLC	-4.19E+00	2.63E-28	PUF60	-8.25E-02	1.00E+00	ZDHHC21	-9.59E-02	1.00E+00
FGF22	-5.14E+00	2.70E-28	NBN	-7.18E-02	1.00E+00	SCN4B	-1.97E-01	1.00E+00
LOC102397769	-4.76E+00	2.75E-28	REXO4	9.89E-02	1.00E+00	ATG2A	-7.75E-02	1.00E+00
TG	2.49E+00	2.77E-28	ERGIC2	6.65E-02	1.00E+00	PGS1	7.64E-02	1.00E+00
NT5C	-3.00E+00	2.79E-28	CCPG1	1.05E-01	1.00E+00	ADAMTSL2	-1.93E-01	1.00E+00
MRTO4	-1.67E+00	2.79E-28	RALYL	4.43E-01	1.00E+00	MAPK15	1.87E-01	1.00E+00

LOC112578267	-3.83E+00	2.82E-28	LOC102398966	-1.30E-01	1.00E+00	ABHD5	1.20E-01	1.00E+00
IL17B	3.43E+00	2.85E-28	CREBRF	-1.82E-01	1.00E+00	SNAPIN	-9.40E-02	1.00E+00
TRPM5	3.98E+00	2.85E-28	ADAMTS12	-7.86E-02	1.00E+00	MRTFB	-1.24E-01	1.00E+00
GPATCH2	-1.44E+00	2.86E-28	ZNF410	-9.46E-02	1.00E+00	GOLPH3L	7.34E-02	1.00E+00
CDCA7L	-1.48E+00	2.86E-28	THRAP3	9.25E-02	1.00E+00	CD164	-8.91E-02	1.00E+00
HDGFL3	-3.09E+00	2.98E-28	DHX8	9.27E-02	1.00E+00	TBC1D25	8.61E-02	1.00E+00
LOC112581162	2.61E+00	3.01E-28	NDUFB10	-1.14E-01	1.00E+00	DRAM1	2.88E-01	1.00E+00
LOC102395067	-5.97E+00	3.07E-28	RMI1	1.40E-01	1.00E+00	PHF7	-1.90E-01	1.00E+00
LOC112581758	4.79E+00	3.12E-28	FCHSD1	1.17E-01	1.00E+00	MARK4	-7.79E-02	1.00E+00
DCBLD2	-2.70E+00	3.32E-28	PPT1	6.86E-02	1.00E+00	LOC102401061	-1.91E-01	1.00E+00
RSBN1L	-1.38E+00	3.40E-28	PLA2G3	-3.93E-01	1.00E+00	C9H5orf30	-9.49E-02	1.00E+00
CEP131	-1.42E+00	3.40E-28	PAWR	1.15E-01	1.00E+00	PRPSAP1	7.13E-02	1.00E+00
LOC112580677	6.51E+00	3.47E-28	FZD9	3.61E-01	1.00E+00	ARHGEF1	-7.11E-02	1.00E+00
MUC19	6.33E+00	3.53E-28	TMEM120A	1.17E-01	1.00E+00	MARCH5	8.20E-02	1.00E+00
LOC112583846	5.30E+00	3.53E-28	LRR1	-8.90E-02	1.00E+00	ITPR1	-1.23E-01	1.00E+00
ZNF768	-1.28E+00	3.55E-28	C4H22orf46	1.49E-01	1.00E+00	HTRA3	2.47E-01	1.00E+00
DQX1	-4.95E+00	3.60E-28	TIAF1	-1.75E-01	1.00E+00	NDUFAF2	-1.12E-01	1.00E+00
TMEM9B	1.16E+00	3.66E-28	G3BP1	7.47E-02	1.00E+00	TIMM10	1.09E-01	1.00E+00
LOC112580302	-2.62E+00	3.78E-28	UBXN1	-1.11E-01	1.00E+00	SPEN	1.02E-01	1.00E+00
EIF4E2	1.16E+00	3.78E-28	KIF3C	7.97E-02	1.00E+00	ZCCHC14	-9.62E-02	1.00E+00
LOC102393747	6.48E+00	3.89E-28	CARNMT1	8.91E-02	1.00E+00	LOC102408349	-1.91E-01	1.00E+00
LOC112581119	2.84E+00	3.93E-28	SCP2	-1.27E-01	1.00E+00	RAE1	7.50E-02	1.00E+00
KRT79	-6.44E+00	3.96E-28	TACC3	9.23E-02	1.00E+00	TAF5	-8.75E-02	1.00E+00
MLIP	5.12E+00	4.15E-28	MALL	6.02E-01	1.00E+00	KIAA1024L	-1.26E-01	1.00E+00
LOC112580166	5.01E+00	4.17E-28	VPS53	8.25E-02	1.00E+00	DYNLT1	-1.05E-01	1.00E+00
LRRC56	-5.14E+00	4.17E-28	PMEL	-1.83E-01	1.00E+00	TOMM5	7.86E-02	1.00E+00
ABCG5	2.59E+00	4.27E-28	VPS4B	8.71E-02	1.00E+00	CNPPD1	7.43E-02	1.00E+00
SLC6A7	3.91E+00	4.28E-28	KCNH1	7.53E-02	1.00E+00	SPRED3	1.76E-01	1.00E+00
LOC102407045	2.94E+00	4.31E-28	KIN	7.19E-02	1.00E+00	LOC102396011	-2.02E-01	1.00E+00
SPCS2	-1.29E+00	4.31E-28	LOC112579236	-8.62E-02	1.00E+00	ARNTL	-1.21E-01	1.00E+00
LOC112583634	3.16E+00	4.33E-28	WTAP	-1.05E-01	1.00E+00	CHRNE	-1.43E-01	1.00E+00
AMOTL2	-5.81E+00	4.37E-28	C9H1orf35	-1.23E-01	1.00E+00	UHMK1	1.17E-01	1.00E+00
PPP3CB	-1.02E+00	4.38E-28	FGFR1OP	-6.79E-02	1.00E+00	NFYC	-7.62E-02	1.00E+00
CHPT1	-	4.39E-28	RBM26	7.67E-02	1.00E+00	ATP6V0E1	-9.31E-	1.00E+00

	1.46E+00						02	
BLOC1S1	-2.16E+00	4.39E-28	LOC112582946	-2.38E-01	1.00E+00	SETD1A	-7.96E-02	1.00E+00
LOC102405323	-5.73E+00	4.45E-28	EXOC7	9.63E-02	1.00E+00	TAB1	-7.42E-02	1.00E+00
LOC112579428	4.13E+00	4.55E-28	FERMT3	-1.40E-01	1.00E+00	RAP2A	-7.56E-02	1.00E+00
MAGED1	-6.01E+00	4.58E-28	ANP32E	-9.00E-02	1.00E+00	ATP6V0D1	8.43E-02	1.00E+00
SYN1	2.04E+00	4.62E-28	C1GALT1C1	-9.74E-02	1.00E+00	TRAPPC5	1.25E-01	1.00E+00
AKAP6	3.76E+00	4.66E-28	CCAR1	6.91E-02	1.00E+00	ZNF713	1.25E-01	1.00E+00
CYTH1	1.10E+00	4.68E-28	ZNF367	3.50E-01	1.00E+00	TSC22D3	-9.54E-02	1.00E+00
PLCE1	3.05E+00	4.79E-28	MACO1	8.12E-02	1.00E+00	PCLAF	-1.03E-01	1.00E+00
SLC48A1	1.25E+00	4.91E-28	CAMLG	-8.20E-02	1.00E+00	C1H3orf70	9.32E-02	1.00E+00
CDT1	1.63E+00	5.04E-28	ZNF394	1.97E-01	1.00E+00	LOC112578768	-1.61E-01	1.00E+00
LST1	-6.45E+00	5.06E-28	TTLL12	7.30E-02	1.00E+00	LGMN	-1.87E-01	1.00E+00
DYDC2	2.48E+00	5.07E-28	PBX1	-9.08E-02	1.00E+00	ZNF280D	-1.00E-01	1.00E+00
CD164	-1.25E+00	5.12E-28	GTF3C1	6.70E-02	1.00E+00	SIRT6	1.00E-01	1.00E+00
ZNF280D	-1.75E+00	5.33E-28	FAM19A3	-3.70E-01	1.00E+00	NKAPL	-9.49E-02	1.00E+00
SPIDR	1.08E+00	5.36E-28	LOC112582129	-8.05E-02	1.00E+00	KDM5B	7.91E-02	1.00E+00
TMPRSS5	5.52E+00	5.44E-28	AKAP8L	8.69E-02	1.00E+00	FKBP10	-1.01E-01	1.00E+00
YIPF3	-1.27E+00	5.49E-28	THAP11	6.22E-02	1.00E+00	PHB	1.12E-01	1.00E+00
TSEN15	-2.12E+00	5.57E-28	CENPJ	7.50E-02	1.00E+00	HNRNPUL2	7.32E-02	1.00E+00
PLEKHA8	-1.19E+00	5.62E-28	ACBD3	9.14E-02	1.00E+00	NCEH1	-9.93E-02	1.00E+00
PPM1N	3.20E+00	5.69E-28	BCDIN3D	-2.37E-01	1.00E+00	DAD1	1.04E-01	1.00E+00
RAP2C	-1.41E+00	5.71E-28	MORN1	1.56E-01	1.00E+00	SDE2	-9.08E-02	1.00E+00
DKK3	1.85E+00	6.16E-28	CAPN5	-3.25E-01	1.00E+00	TIGD2	9.47E-02	1.00E+00
LSM2	1.32E+00	6.16E-28	ILVBL	-1.19E-01	1.00E+00	ZNF404	1.64E-01	1.00E+00
LOC102397482	-6.47E+00	6.24E-28	RHBDD3	1.50E-01	1.00E+00	UROC1	-1.13E-01	1.00E+00
LOC102410667	-8.50E+00	6.27E-28	DHRS7B	1.17E-01	1.00E+00	INPP5K	-7.28E-02	1.00E+00
CCDC125	-2.71E+00	6.43E-28	LOC102409355	-1.66E-01	1.00E+00	SEC61B	1.21E-01	1.00E+00
GUCD1	1.26E+00	6.62E-28	CELF5	-1.99E-01	1.00E+00	RMDN2	8.71E-02	1.00E+00
LOC112578438	-4.81E+00	6.72E-28	NELFE	6.58E-02	1.00E+00	CUTC	8.93E-02	1.00E+00
EIF5A	-1.48E+00	7.02E-28	UTP23	8.46E-02	1.00E+00	SUOX	-9.58E-02	1.00E+00
IL13RA1	1.26E+00	7.14E-28	LOC102403617	1.26E-01	1.00E+00	LOC102401686	-1.39E-01	1.00E+00
PRTG	-2.39E+00	7.23E-28	ASF1B	6.99E-02	1.00E+00	WDR35	8.98E-02	1.00E+00
ATXN7L3B	-1.03E+00	7.44E-28	PITPNA	-6.24E-02	1.00E+00	GPSM2	-8.44E-02	1.00E+00

GPR26	5.16E+00	7.46E-28	TCAIM	-1.75E-01	1.00E+00	LOC102404930	8.38E-02	1.00E+00
RTTN	1.33E+00	7.68E-28	POLR2D	-9.60E-02	1.00E+00	WWTR1	-9.88E-02	1.00E+00
MRPL15	-1.22E+00	7.79E-28	ST3GAL1	-4.01E-01	1.00E+00	GALNT10	1.19E-01	1.00E+00
ZNF197	-1.58E+00	7.89E-28	RNF212	-1.08E-01	1.00E+00	PIGF	-1.02E-01	1.00E+00
GBA2	-1.07E+00	7.96E-28	STYXL1	2.34E-01	1.00E+00	PRRC2B	-9.62E-02	1.00E+00
PPP1R1B	2.89E+00	8.12E-28	OTC	-2.91E-01	1.00E+00	DNAJC9	-1.01E-01	1.00E+00
SNCAIP	3.99E+00	8.53E-28	ZFAND1	1.29E-01	1.00E+00	STT3B	-7.76E-02	1.00E+00
MACC1	4.27E+00	8.59E-28	MOSPD2	-6.28E-02	1.00E+00	NUP54	9.32E-02	1.00E+00
DNAAF1	3.97E+00	8.61E-28	DDB1	7.70E-02	1.00E+00	HMOX2	1.00E-01	1.00E+00
MPLKIP	-2.60E+00	8.68E-28	SMS	-8.62E-02	1.00E+00	PIAS1	-7.87E-02	1.00E+00
LOC102411649	1.76E+00	8.75E-28	CDC45	1.00E-01	1.00E+00	LAPTM5	-3.58E-01	1.00E+00
FAM83H	-2.15E+00	8.85E-28	PCGF5	-8.87E-02	1.00E+00	PSMD8	9.98E-02	1.00E+00
TTI2	-1.46E+00	8.87E-28	HSD17B8	-1.35E-01	1.00E+00	MYOF	9.34E-02	1.00E+00
CEMIP	-3.85E+00	9.01E-28	LSS	9.89E-02	1.00E+00	PLCD1	-1.02E-01	1.00E+00
NWD2	6.49E+00	9.06E-28	ZNF276	1.10E-01	1.00E+00	OLA1	7.76E-02	1.00E+00
ADAMTS12	3.96E+00	9.17E-28	ZBTB5	7.31E-02	1.00E+00	GPCPD1	-1.09E-01	1.00E+00
ATP2C1	1.15E+00	9.22E-28	EPC2	-1.20E-01	1.00E+00	HSD3B7	1.23E-01	1.00E+00
SUPT3H	-1.45E+00	9.43E-28	NINJ1	1.15E-01	1.00E+00	RAB7A	-7.53E-02	1.00E+00
SLC25A41	-4.69E+00	9.55E-28	ZRANB1	9.01E-02	1.00E+00	CENPF	-9.85E-02	1.00E+00
POLR2L	-2.04E+00	9.80E-28	LAMTOR5	-1.65E-01	1.00E+00	LOC102410120	-2.35E-01	1.00E+00
CHD2	-1.32E+00	9.98E-28	LOC112580607	1.14E-01	1.00E+00	LOC102406024	8.76E-02	1.00E+00
ELMO1	-4.31E+00	1.00E-27	LOC112580412	-5.87E-01	1.00E+00	KRIT1	-1.75E-01	1.00E+00
RXRB	-1.23E+00	1.01E-27	CSNK2B	7.59E-02	1.00E+00	ECPAS	7.11E-02	1.00E+00
APEX1	-1.23E+00	1.07E-27	HS3ST3B1	-1.52E-01	1.00E+00	AP5Z1	8.26E-02	1.00E+00
PMEPA1	-2.07E+00	1.07E-27	CBY1	-1.70E-01	1.00E+00	PRDM11	-1.37E-01	1.00E+00
PTPN7	-4.72E+00	1.09E-27	NDC80	-6.73E-02	1.00E+00	NBDY	8.53E-02	1.00E+00
LOC102399270	-2.39E+00	1.09E-27	AURKB	7.92E-02	1.00E+00	TNPO2	-6.88E-02	1.00E+00
SRPRA	-9.97E-01	1.10E-27	LOC102415513	-1.93E-01	1.00E+00	UNC93B1	1.19E-01	1.00E+00
SLC30A6	-1.62E+00	1.10E-27	AMD1	6.48E-02	1.00E+00	MELK	-9.51E-02	1.00E+00
EFS	-4.89E+00	1.11E-27	ZBTB38	2.21E-01	1.00E+00	SLC25A16	-1.38E-01	1.00E+00
SPATA9	-3.46E+00	1.12E-27	LOC112586947	1.93E-01	1.00E+00	LOC112580623	-1.45E-01	1.00E+00
LOC112579660	5.38E+00	1.13E-27	HAUS1	8.64E-02	1.00E+00	ARFIP2	-8.80E-02	1.00E+00
MDH1B	-3.76E+00	1.15E-27	ZNF644	1.57E-01	1.00E+00	ZSCAN25	-9.40E-02	1.00E+00

LOC102401601	3.23E+00	1.18E-27	HMBOX1	8.37E-02	1.00E+00	LOC102402027	-9.35E-02	1.00E+00
MTHFD2	1.58E+00	1.19E-27	LOC102403789	9.22E-02	1.00E+00	PRPF18	7.75E-02	1.00E+00
RNF14	9.92E-01	1.20E-27	KCNA3	-3.47E-01	1.00E+00	DTD1	8.65E-02	1.00E+00
KDM3B	-2.48E+00	1.23E-27	CIP2A	1.14E-01	1.00E+00	RPS21	-1.19E-01	1.00E+00
GIPC3	-6.87E+00	1.24E-27	TAF1	1.90E-01	1.00E+00	GSDMD	2.92E-01	1.00E+00
KLHL9	-1.67E+00	1.25E-27	P2RX5	-1.38E-01	1.00E+00	TSR1	-9.04E-02	1.00E+00
LOC112578003	7.12E+00	1.25E-27	TBC1D31	1.97E-01	1.00E+00	LOC102389727	1.48E-01	1.00E+00
LOC102395590	3.82E+00	1.27E-27	LOC112587866	2.48E-01	1.00E+00	PIP4K2C	6.92E-02	1.00E+00
TOR3A	-1.63E+00	1.30E-27	CPNE3	-3.70E-01	1.00E+00	LOC112587745	-1.03E-01	1.00E+00
C22H18orf63	5.74E+00	1.32E-27	GFOD2	1.96E-01	1.00E+00	LOC112582305	-1.43E-01	1.00E+00
CKB	2.77E+00	1.32E-27	AGBL1	-1.45E-01	1.00E+00	POLR3H	1.13E-01	1.00E+00
IPO4	-1.32E+00	1.32E-27	KANSL2	-8.58E-02	1.00E+00	SLC9A1	9.47E-02	1.00E+00
NECAP2	-1.19E+00	1.37E-27	COL4A3BP	7.78E-02	1.00E+00	OCEL1	1.02E-01	1.00E+00
CD320	2.02E+00	1.38E-27	ZFAND2A	-7.88E-02	1.00E+00	MYBL2	-1.02E-01	1.00E+00
LOC102407686	1.12E+00	1.39E-27	PRCP	-1.18E-01	1.00E+00	ENO3	1.36E-01	1.00E+00
GINS1	-2.52E+00	1.39E-27	VDAC3	-8.26E-02	1.00E+00	FAM171B	1.12E-01	1.00E+00
MDFIC	-1.90E+00	1.40E-27	SNUPN	-7.69E-02	1.00E+00	MRPL15	-7.37E-02	1.00E+00
PIP5K1C	1.22E+00	1.42E-27	STRN3	8.08E-02	1.00E+00	CCT3	9.94E-02	1.00E+00
LOC112585091	-6.95E+00	1.44E-27	LOC102395607	1.05E-01	1.00E+00	BCORL1	-1.24E-01	1.00E+00
PRPF4B	-1.27E+00	1.44E-27	PGD	-6.57E-02	1.00E+00	LOC112580246	1.60E-01	1.00E+00
ZNF532	-1.38E+00	1.45E-27	PLCB4	-2.17E-01	1.00E+00	FRMD5	-1.05E-01	1.00E+00
SBSN	-5.59E+00	1.46E-27	KIAA2013	-9.05E-02	1.00E+00	IKZF2	-8.50E-02	1.00E+00
LPL	-6.11E+00	1.47E-27	ZNF575	1.05E-01	1.00E+00	CISD1	1.11E-01	1.00E+00
LOC102390734	4.81E+00	1.48E-27	TGFB3	2.32E-01	1.00E+00	LOC102412637	8.28E-02	1.00E+00
SSPN	-5.15E+00	1.51E-27	ANXA4	-3.38E-01	1.00E+00	GYPC	1.47E-01	1.00E+00
GRAMD1C	-2.18E+00	1.51E-27	DDHD2	7.85E-02	1.00E+00	C4H12orf29	-8.97E-02	1.00E+00
LMBR1	-1.11E+00	1.53E-27	LOC102389879	-2.83E-01	1.00E+00	KCTD2	6.97E-02	1.00E+00
SRP54	-9.53E-01	1.53E-27	STRBP	7.71E-02	1.00E+00	AKT3	8.97E-02	1.00E+00
MANBA	1.36E+00	1.55E-27	RELB	1.68E-01	1.00E+00	PTGR2	-8.90E-02	1.00E+00
NLRX1	-2.83E+00	1.55E-27	SLC4A11	-1.68E-01	1.00E+00	FBXO15	-1.16E-01	1.00E+00
LOC112583969	5.51E+00	1.55E-27	ATP8B1	-2.25E-01	1.00E+00	LCP2	3.39E-01	1.00E+00
DISP2	5.07E+00	1.58E-27	MEX3A	-1.83E-01	1.00E+00	COX3	-1.92E-01	1.00E+00
ALX3	4.60E+00	1.59E-27	ROR1	1.09E-01	1.00E+00	LOC112582089	1.65E-01	1.00E+00
BRINP3	6.50E+00	1.62E-27	RARG	-3.36E-01	1.00E+00	HESX1	-1.40E-01	1.00E+00

MMP16	-4.25E+00	1.63E-27	CREB3L1	-1.61E-01	1.00E+00	CTPS1	-7.41E-02	1.00E+00
ACO1	-1.35E+00	1.69E-27	SYNM	2.23E-01	1.00E+00	ZNF772	1.07E-01	1.00E+00
LOC112582263	7.58E+00	1.74E-27	FNDC1	1.36E-01	1.00E+00	FAF2	1.11E-01	1.00E+00
PNMA5	7.62E+00	1.76E-27	COPZ1	7.53E-02	1.00E+00	OSBPL9	-7.98E-02	1.00E+00
TTF1	1.03E+00	1.76E-27	PTTG1	-8.88E-02	1.00E+00	METTL27	-1.66E-01	1.00E+00
SNAPC1	-1.76E+00	1.78E-27	ZNF202	-1.56E-01	1.00E+00	LOC102391695	2.98E-01	1.00E+00
TMEM232	2.17E+00	1.78E-27	LOC112580807	-8.85E-02	1.00E+00	LOC112581234	-1.79E-01	1.00E+00
HAUS7	-1.64E+00	1.79E-27	TAF5	-7.36E-02	1.00E+00	LOC102408209	1.96E-01	1.00E+00
MAF1	-1.44E+00	1.81E-27	SNAP25	-1.20E-01	1.00E+00	LOC102404484	9.30E-02	1.00E+00
ARNTL	1.70E+00	1.82E-27	CHURC1	6.61E-02	1.00E+00	L3HYPDH	1.25E-01	1.00E+00
IFNAR2	-6.05E+00	1.83E-27	NDUFA12	-1.28E-01	1.00E+00	CYREN	8.29E-02	1.00E+00
RASAL1	-5.38E+00	1.87E-27	TBC1D17	1.03E-01	1.00E+00	CCDC18	-9.03E-02	1.00E+00
DLST	1.14E+00	1.89E-27	CXHXorf56	-7.37E-02	1.00E+00	LOC102409528	-1.71E-01	1.00E+00
KLF5	2.86E+00	1.90E-27	LOC102415619	1.04E-01	1.00E+00	LCA5	1.93E-01	1.00E+00
NIPSNAP1	-1.21E+00	1.95E-27	RUVBL1	-7.08E-02	1.00E+00	CRIP3	-1.37E-01	1.00E+00
CCDC148	2.87E+00	1.96E-27	ITPA	-9.60E-02	1.00E+00	LOC102403267	-3.89E-01	1.00E+00
LOC112580260	4.57E+00	1.99E-27	ORMDL2	1.41E-01	1.00E+00	PHLPP1	7.78E-02	1.00E+00
LOC102396645	5.90E+00	1.99E-27	LLGL2	8.05E-02	1.00E+00	LOC102401619	1.90E-01	1.00E+00
LOC112587417	-6.13E+00	1.99E-27	PGAP2	-1.48E-01	1.00E+00	FZD9	1.47E-01	1.00E+00
LRRC71	-1.93E+00	2.00E-27	FGFR2	1.62E-01	1.00E+00	NMRK1	-9.69E-02	1.00E+00
TRIB2	-5.81E+00	2.00E-27	LAMB4	7.34E-02	1.00E+00	IMMP2L	8.77E-02	1.00E+00
FAT3	5.76E+00	2.07E-27	SOGA1	9.84E-02	1.00E+00	DIAPH2	8.99E-02	1.00E+00
LOC112583918	-4.12E+00	2.07E-27	RPP40	-1.08E-01	1.00E+00	REM2	1.00E-01	1.00E+00
FBH1	-1.03E+00	2.08E-27	NUDT16L1	-1.04E-01	1.00E+00	BCL7B	-8.05E-02	1.00E+00
CSMD2	-1.67E+00	2.12E-27	ASPDH	-1.75E-01	1.00E+00	TBP	-8.09E-02	1.00E+00
FOXE1	6.93E+00	2.13E-27	LOC102412117	3.00E-01	1.00E+00	PCSK1N	1.24E-01	1.00E+00
AGBL3	-3.73E+00	2.19E-27	ELOVL7	-1.33E-01	1.00E+00	LOC102402227	9.02E-02	1.00E+00
PUM3	1.10E+00	2.19E-27	KIF3A	9.44E-02	1.00E+00	MAP7D2	-7.97E-02	1.00E+00
CREB3L1	-2.46E+00	2.20E-27	CBX8	6.86E-02	1.00E+00	SLC35C1	1.07E-01	1.00E+00
LOC112580688	5.55E+00	2.20E-27	POMGNT2	-9.86E-02	1.00E+00	LOC102402179	1.36E-01	1.00E+00
CFAP206	3.03E+00	2.20E-27	GCNT3	-1.35E-01	1.00E+00	MYORG	-1.59E-01	1.00E+00
METTL8	1.28E+00	2.22E-27	VPS13A	1.35E-01	1.00E+00	LOC112580349	1.73E-01	1.00E+00
USP16	8.92E-01	2.31E-27	NPM2	-8.69E-02	1.00E+00	SLC11A1	-3.46E-01	1.00E+00
LOC102415310	-5.57E+00	2.34E-27	SFXN2	-1.33E-01	1.00E+00	TBC1D15	-9.12E-02	1.00E+00
SARS2	-1.64E+00	2.47E-27	MTMR10	-1.22E-01	1.00E+00	LOC102408791	1.87E-01	1.00E+00

LOC112585628	-5.29E+00	2.49E-27	NME4	4.82E-01	1.00E+00	PNRC2	-8.48E-02	1.00E+00
CYYR1	-5.85E+00	2.50E-27	ALX3	-2.93E-01	1.00E+00	ALDH9A1	-7.67E-02	1.00E+00
LOC112579903	4.41E+00	2.51E-27	FAM71E1	-1.54E-01	1.00E+00	RECQL4	-1.10E-01	1.00E+00
GNE	-1.65E+00	2.65E-27	ACSM1	-2.07E-01	1.00E+00	MYBL1	-1.21E-01	1.00E+00
WNT3	5.80E+00	2.67E-27	CNPY4	-1.72E-01	1.00E+00	RRP1	8.44E-02	1.00E+00
SIDT1	3.88E+00	2.74E-27	GRIN2D	5.44E-01	1.00E+00	ACAD10	-7.77E-02	1.00E+00
LOC112584451	3.08E+00	2.74E-27	ESR2	8.50E-02	1.00E+00	PHYKPL	-9.92E-02	1.00E+00
MAF	-5.88E+00	2.84E-27	TAF5L	6.03E-02	1.00E+00	PCGF3	7.31E-02	1.00E+00
TMPRSS9	2.08E+00	2.85E-27	BRINP2	7.57E-02	1.00E+00	KIF19	-9.15E-02	1.00E+00
DENND4A	1.22E+00	2.90E-27	LOC112584965	-2.54E-01	1.00E+00	TTYH1	-1.76E-01	1.00E+00
LOC102396042	-2.30E+00	2.91E-27	ADAL	8.86E-02	1.00E+00	CIAO2A	-9.74E-02	1.00E+00
SLC13A5	-5.97E+00	2.94E-27	TMED4	-6.85E-02	1.00E+00	ACAP2	1.25E-01	1.00E+00
GRM4	5.24E+00	3.04E-27	PHF14	7.76E-02	1.00E+00	COG4	7.44E-02	1.00E+00
SLC35E4	-3.50E+00	3.06E-27	EIF4H	5.83E-02	1.00E+00	DPCD	-1.06E-01	1.00E+00
SLC39A8	-5.22E+00	3.07E-27	NDUFB1	-1.23E-01	1.00E+00	TMEM145	1.44E-01	1.00E+00
LOC112578666	4.59E+00	3.13E-27	COMMD2	-9.24E-02	1.00E+00	OCIAD1	-7.23E-02	1.00E+00
TRAF3IP3	2.19E+00	3.13E-27	PARP4	-2.14E-01	1.00E+00	LOC102394835	-1.98E-01	1.00E+00
KLC3	2.82E+00	3.14E-27	MELK	7.20E-02	1.00E+00	LOC102415829	-7.52E-02	1.00E+00
CEP95	1.39E+00	3.22E-27	PDE1C	-1.49E-01	1.00E+00	COQ4	-9.56E-02	1.00E+00
LOC112579999	6.19E+00	3.29E-27	CUTA	-8.16E-02	1.00E+00	TOMM40	8.93E-02	1.00E+00
ANP32A	-1.40E+00	3.45E-27	LOC102392628	-9.53E-02	1.00E+00	KCNE4	1.62E-01	1.00E+00
ATP5F1A	-1.63E+00	3.56E-27	BMPR1A	-6.51E-02	1.00E+00	UIMC1	7.19E-02	1.00E+00
LOC112582255	-5.02E+00	3.67E-27	PLEKHF2	-8.09E-02	1.00E+00	LOC102409615	1.78E-01	1.00E+00
FBXO8	1.25E+00	3.70E-27	EXOSC8	-7.84E-02	1.00E+00	IDNK	-1.18E-01	1.00E+00
SEMA6B	-4.41E+00	3.71E-27	MBTPS2	-3.23E-01	1.00E+00	MYL6	1.27E-01	1.00E+00
FAM136A	-1.44E+00	3.75E-27	CHEK1	-8.59E-02	1.00E+00	HSPB6	1.48E-01	1.00E+00
ANKRD12	-1.75E+00	3.76E-27	CATSPER3	2.09E-01	1.00E+00	ATG13	7.38E-02	1.00E+00
NGFR	2.96E+00	3.88E-27	IL10RB	2.89E-01	1.00E+00	LOC112580679	1.75E-01	1.00E+00
LOC102411786	-3.19E+00	3.97E-27	EXOC1	9.97E-02	1.00E+00	LOC102407934	-1.10E-01	1.00E+00
MPDZ	-1.56E+00	4.03E-27	LOC102406662	-9.76E-02	1.00E+00	DMAP1	9.25E-02	1.00E+00
TMEM50A	-1.09E+00	4.20E-27	POP4	9.91E-02	1.00E+00	KDM3B	-1.19E-01	1.00E+00
LOC102414615	-3.63E+00	4.22E-27	TRIM36	1.79E-01	1.00E+00	BCKDK	-7.19E-02	1.00E+00
ALG3	-1.72E+00	4.25E-27	MTG2	1.54E-01	1.00E+00	LDOC1	1.28E-01	1.00E+00

COPZ1	-1.24E+00	4.27E-27	MPPE1	2.19E-01	1.00E+00	INO80C	8.85E-02	1.00E+00
PRKAG3	-6.40E+00	4.37E-27	COPB1	-6.44E-02	1.00E+00	RNF181	1.17E-01	1.00E+00
GFM2	1.03E+00	4.49E-27	LOC102394398	3.02E-01	1.00E+00	RIOX2	9.18E-02	1.00E+00
LOC102395838	-3.09E+00	4.50E-27	AGBL5	1.10E-01	1.00E+00	PURA	-8.63E-02	1.00E+00
HSP90AB1	-1.90E+00	4.50E-27	PTPRO	1.65E-01	1.00E+00	GMEB1	-7.84E-02	1.00E+00
LOC102394311	5.65E+00	4.54E-27	PEF1	1.28E-01	1.00E+00	TEAD3	-9.13E-02	1.00E+00
THBD	-8.51E+00	4.57E-27	CDH13	1.06E-01	1.00E+00	GOLGB1	7.19E-02	1.00E+00
KLHL2	-1.49E+00	4.64E-27	UBTD1	8.21E-02	1.00E+00	SEC24C	-6.88E-02	1.00E+00
WDR48	9.52E-01	4.66E-27	ALKBH7	-1.10E-01	1.00E+00	VAPB	-8.03E-02	1.00E+00
MS4A1	4.29E+00	4.74E-27	LOC112581779	3.69E-01	1.00E+00	XRRA1	-1.23E-01	1.00E+00
SLC25A43	-3.44E+00	4.82E-27	INSM2	2.31E-01	1.00E+00	BLOC1S2	8.92E-02	1.00E+00
SLC49A3	-1.43E+00	4.89E-27	CSNK1G1	-7.07E-02	1.00E+00	NPC2	1.06E-01	1.00E+00
C17H5orf52	1.98E+00	4.89E-27	BAG6	6.90E-02	1.00E+00	PUS10	-9.37E-02	1.00E+00
LOC102404166	-4.53E+00	4.91E-27	GPLD1	9.52E-02	1.00E+00	SNAPC5	-1.48E-01	1.00E+00
MAN2A1	-1.54E+00	4.93E-27	ZNF804B	9.48E-02	1.00E+00	GTSE1	-9.34E-02	1.00E+00
UNC119	2.06E+00	5.00E-27	PAK4	8.67E-02	1.00E+00	SAMD12	1.09E-01	1.00E+00
DHPS	1.52E+00	5.06E-27	UBXN2B	8.59E-02	1.00E+00	NRG4	1.66E-01	1.00E+00
LOC102403897	-3.98E+00	5.09E-27	PLEKHG1	7.76E-02	1.00E+00	SHMT2	-8.77E-02	1.00E+00
FBXL3	1.39E+00	5.11E-27	PRKAA2	-1.09E-01	1.00E+00	FBXW4	9.39E-02	1.00E+00
PPP1R21	1.11E+00	5.15E-27	ZNF711	-4.68E-01	1.00E+00	LRRC2	8.40E-02	1.00E+00
LOC112587819	3.32E+00	5.34E-27	NWD1	-4.79E-01	1.00E+00	UBXN4	-6.78E-02	1.00E+00
ZNF428	-2.39E+00	5.48E-27	LOC112580626	-1.46E-01	1.00E+00	INTS13	7.97E-02	1.00E+00
LOC102394445	3.94E+00	5.52E-27	LOC102395334	-1.53E-01	1.00E+00	CEBPD	-1.36E-01	1.00E+00
LOC102399215	-1.98E+00	5.53E-27	LOC102405586	1.27E-01	1.00E+00	RAB33A	1.63E-01	1.00E+00
LOC102393680	-5.92E+00	5.58E-27	LEF1	1.39E-01	1.00E+00	RPL38	-1.26E-01	1.00E+00
COL1A2	-4.41E+00	5.60E-27	ANK1	-1.80E-01	1.00E+00	CCDC8	-1.02E-01	1.00E+00
LOC102404242	4.28E+00	5.61E-27	LOC102402583	-1.71E-01	1.00E+00	ST7L	-8.96E-02	1.00E+00
LOC102402411	-8.86E+00	5.78E-27	TRIM56	3.94E-01	1.00E+00	ZNF789	-1.28E-01	1.00E+00
HK2	-4.07E+00	5.95E-27	ASB7	-1.42E-01	1.00E+00	MRS2	-8.16E-02	1.00E+00
MRPL35	-1.94E+00	5.95E-27	SEPT6	2.78E-01	1.00E+00	URI1	-7.10E-02	1.00E+00
WNT9A	2.99E+00	5.95E-27	ZBTB9	9.74E-02	1.00E+00	DEF6	1.53E-01	1.00E+00
TRAPPC2L	-2.75E+00	6.13E-27	SERPINE3	-3.45E-01	1.00E+00	AP2M1	9.10E-02	1.00E+00
GK2	7.03E+00	6.13E-27	LOC102414025	-3.23E-01	1.00E+00	LOC102416651	-9.40E-02	1.00E+00
LOC102404801	-	6.13E-27	NUF2	6.81E-02	1.00E+00	LOC102406576	-1.00E-	1.00E+00

	6.39E+00						01	
TSPAN32	-5.69E+00	6.17E-27	YWHAQ	7.01E-02	1.00E+00	NAF1	-9.08E-02	1.00E+00
NAALAD2	-3.05E+00	6.20E-27	PKM	8.77E-02	1.00E+00	LOC102397908	-1.69E-01	1.00E+00
RAD51C	1.21E+00	6.28E-27	LOC102402465	-1.10E-01	1.00E+00	LOC112578805	-1.69E-01	1.00E+00
ACOT13	-1.66E+00	6.35E-27	TPD52L2	1.07E-01	1.00E+00	VRK3	-7.40E-02	1.00E+00
ROGDI	-1.88E+00	6.50E-27	TMEM106C	-9.72E-02	1.00E+00	RABL2B	-7.38E-02	1.00E+00
SYNPO2L	3.48E+00	6.55E-27	LOC102397010	1.79E-01	1.00E+00	HHEX	1.98E-01	1.00E+00
LOC112587797	4.78E+00	6.60E-27	RRAGB	-2.05E-01	1.00E+00	BLOC1S5	8.09E-02	1.00E+00
SLC46A1	1.20E+00	6.63E-27	NMRAL1	-1.47E-01	1.00E+00	LARGE1	7.02E-02	1.00E+00
LOC112583101	-5.00E+00	6.74E-27	PICALM	6.81E-02	1.00E+00	LOC112578706	2.12E-01	1.00E+00
PSMD3	-1.33E+00	7.00E-27	ARHGAP11A	1.21E-01	1.00E+00	MEN1	-7.49E-02	1.00E+00
SHISA4	-2.61E+00	7.29E-27	GCLC	-1.42E-01	1.00E+00	MLLT3	-8.02E-02	1.00E+00
TPCN2	-1.68E+00	7.31E-27	UIMC1	6.39E-02	1.00E+00	UPF2	-7.03E-02	1.00E+00
PRDM10	-2.32E+00	7.35E-27	PAPPA	-3.17E-01	1.00E+00	DEF8	8.28E-02	1.00E+00
SPATA5L1	-1.24E+00	7.45E-27	RPSA	-1.02E-01	1.00E+00	MTRF1L	-7.93E-02	1.00E+00
STARD5	-4.68E+00	7.50E-27	FOXC2	-1.93E-01	1.00E+00	FXR1	-6.87E-02	1.00E+00
PRKAR1B	-4.22E+00	7.65E-27	CNPPD1	-8.42E-02	1.00E+00	TSTD2	-8.95E-02	1.00E+00
TGM2	-6.19E+00	7.72E-27	MYT1L	-2.41E-01	1.00E+00	ZNF394	9.37E-02	1.00E+00
LOC112583973	4.14E+00	7.74E-27	SEM1	-8.01E-02	1.00E+00	CEP162	-9.33E-02	1.00E+00
SCG5	-1.52E+00	7.79E-27	UFM1	-9.24E-02	1.00E+00	MAGIX	-1.40E-01	1.00E+00
LOC112585216	-5.88E+00	7.89E-27	LOC102409480	-2.28E-01	1.00E+00	SYNGR2	-7.57E-02	1.00E+00
CEP162	-1.75E+00	8.02E-27	DBT	-2.10E-01	1.00E+00	CTSZ	-1.58E-01	1.00E+00
PSAP	-1.55E+00	8.02E-27	VRK1	-6.39E-02	1.00E+00	TRIAP1	-9.67E-02	1.00E+00
LOC112578050	4.71E+00	8.04E-27	CHRAC1	-1.40E-01	1.00E+00	TCAF2	-1.06E-01	1.00E+00
HS3ST6	3.55E+00	8.07E-27	LOC102402496	3.72E-01	1.00E+00	TFE3	8.00E-02	1.00E+00
ARL8B	1.14E+00	8.09E-27	LOC102408974	-1.80E-01	1.00E+00	SMPDL3A	2.99E-01	1.00E+00
PLAC9	-5.68E+00	8.18E-27	KIF22	6.50E-02	1.00E+00	EPG5	-1.06E-01	1.00E+00
GUK1	-1.65E+00	8.24E-27	TCTN3	-9.03E-02	1.00E+00	THOC1	-6.84E-02	1.00E+00
PLEKHD1	-2.26E+00	8.39E-27	CAPN3	-1.74E-01	1.00E+00	TMEM9B	7.73E-02	1.00E+00
DZANK1	-3.68E+00	8.43E-27	PLCD1	-3.43E-01	1.00E+00	POLR3GL	-9.27E-02	1.00E+00
TMEM25	-2.75E+00	8.44E-27	LOC112587819	2.50E-01	1.00E+00	CKS1B	-1.12E-01	1.00E+00
CLCN3	-9.84E-01	8.77E-27	CIB1	7.05E-02	1.00E+00	METTL23	-1.06E-01	1.00E+00
PI4KB	9.21E-01	8.78E-27	MARCH8	9.08E-02	1.00E+00	CNN3	1.01E-01	1.00E+00
LOC102408184	1.23E+00	9.52E-27	PDE6A	-2.67E-	1.00E+00	RBM26	-8.15E-	1.00E+00

				01			02	
ZDHHC16	-1.44E+00	9.55E-27	TEC	-2.11E-01	1.00E+00	JAKMIP2	-8.44E-02	1.00E+00
NUP62CL	-1.92E+00	9.87E-27	AMOTL1	8.12E-02	1.00E+00	LOC102394597	-1.07E-01	1.00E+00
GPR108	-1.27E+00	1.02E-26	LOC112582071	-3.16E-01	1.00E+00	TXNDC11	-7.09E-02	1.00E+00
AGPAT1	1.02E+00	1.04E-26	PCF11	9.84E-02	1.00E+00	SPI1	-3.24E-01	1.00E+00
TP63	5.69E+00	1.07E-26	LOC112586445	-2.00E-01	1.00E+00	TRAF1	-8.55E-02	1.00E+00
STARD13	-3.08E+00	1.09E-26	HNRNPH3	-7.55E-02	1.00E+00	FBXO22	8.14E-02	1.00E+00
ZBTB41	-1.85E+00	1.09E-26	UBA5	-9.60E-02	1.00E+00	BAG4	9.94E-02	1.00E+00
TBC1D12	-2.32E+00	1.11E-26	FMO1	3.66E-01	1.00E+00	LOC102413847	1.13E-01	1.00E+00
SLC28A2	-7.95E+00	1.11E-26	ARHGEF9	1.02E-01	1.00E+00	USP30	-9.65E-02	1.00E+00
TGS1	1.25E+00	1.12E-26	RUFY1	-8.03E-02	1.00E+00	ARHGEF16	1.69E-01	1.00E+00
MRPL3	-1.12E+00	1.14E-26	CACTIN	8.86E-02	1.00E+00	LOC102412117	-1.11E-01	1.00E+00
ETS2	-2.99E+00	1.16E-26	METTL23	-1.45E-01	1.00E+00	SEC24A	-1.04E-01	1.00E+00
FGD6	2.86E+00	1.16E-26	ALDH4A1	-8.70E-02	1.00E+00	P3H1	-8.42E-02	1.00E+00
LOC112587008	-5.00E+00	1.19E-26	DAZAP2	6.33E-02	1.00E+00	GSE1	-1.22E-01	1.00E+00
RNF103	-1.43E+00	1.20E-26	PAN2	9.38E-02	1.00E+00	FOLH1B	1.48E-01	1.00E+00
LOC112579958	-5.06E+00	1.21E-26	CLCN6	1.72E-01	1.00E+00	MGAT3	-1.58E-01	1.00E+00
FLCN	1.17E+00	1.24E-26	FAM110B	-9.60E-02	1.00E+00	RECQL	-1.08E-01	1.00E+00
SNRPD2	-2.10E+00	1.25E-26	DECR2	1.17E-01	1.00E+00	MARCKS	-7.11E-02	1.00E+00
ASB15	-5.24E+00	1.28E-26	NUP214	8.77E-02	1.00E+00	CARS2	9.50E-02	1.00E+00
LOC112587923	5.40E+00	1.34E-26	IGDCC4	-4.56E-01	1.00E+00	NAP1L1	-7.97E-02	1.00E+00
TPST2	1.28E+00	1.34E-26	SYBU	-1.53E-01	1.00E+00	IGSF11	-7.95E-02	1.00E+00
YBEY	-2.43E+00	1.36E-26	SLC38A1	-4.68E-01	1.00E+00	PAIP1	6.83E-02	1.00E+00
LOC112583752	6.76E+00	1.38E-26	SPIC	-8.89E-02	1.00E+00	LOC112581204	1.71E-01	1.00E+00
FTO	-9.95E-01	1.43E-26	CHMP4A	-5.45E-01	1.00E+00	MCM7	-9.50E-02	1.00E+00
SPAST	-2.03E+00	1.43E-26	TTLL3	1.06E-01	1.00E+00	FAM217B	-1.92E-01	1.00E+00
NME3	-2.31E+00	1.45E-26	LOC112583851	-1.94E-01	1.00E+00	RNF38	8.55E-02	1.00E+00
GPRC5A	2.59E+00	1.46E-26	C24H16orf72	6.59E-02	1.00E+00	PTPN6	-2.42E-01	1.00E+00
LOXL3	-5.74E+00	1.49E-26	PHF23	-8.06E-02	1.00E+00	NSMF	-1.14E-01	1.00E+00
TMEM67	-3.13E+00	1.50E-26	ZNF142	9.88E-02	1.00E+00	SMG6	6.63E-02	1.00E+00
ARHGAP15	-4.80E+00	1.52E-26	ASB4	-2.81E-01	1.00E+00	E2F3	9.39E-02	1.00E+00
LOC102390188	1.83E+00	1.53E-26	KIF2C	6.05E-02	1.00E+00	SYCP3	-1.82E-01	1.00E+00
LOC102404378	3.99E+00	1.54E-26	PPP1R21	-9.09E-	1.00E+00	SMARCD3	-8.87E-	1.00E+00

				02			02	
VCPIP1	1.46E+00	1.55E-26	LOC112580299	1.80E-01	1.00E+00	ZNF185	1.50E-01	1.00E+00
SERINC5	-1.30E+00	1.59E-26	MTFR2	-6.34E-02	1.00E+00	ZKSCAN4	-1.30E-01	1.00E+00
TSG101	1.01E+00	1.62E-26	SARNP	-2.11E-01	1.00E+00	CCAR2	7.38E-02	1.00E+00
LOC112581585	6.94E+00	1.62E-26	TEX10	6.43E-02	1.00E+00	ABHD3	2.04E-01	1.00E+00
GSDMB	-5.23E+00	1.63E-26	PLRG1	6.19E-02	1.00E+00	LRRC8B	1.23E-01	1.00E+00
EIF2AK2	-1.54E+00	1.64E-26	TSTD3	-1.87E-01	1.00E+00	MRM2	8.97E-02	1.00E+00
ENSA	1.14E+00	1.65E-26	DNAH17	9.75E-02	1.00E+00	TSHZ2	-8.15E-02	1.00E+00
PMS2	-1.29E+00	1.70E-26	MFGE8	1.19E-01	1.00E+00	TK2	-8.33E-02	1.00E+00
CNIH1	1.02E+00	1.70E-26	LEKR1	2.78E-01	1.00E+00	FCER1G	-2.65E-01	1.00E+00
PRICKLE3	-3.56E+00	1.71E-26	LOC112587808	-4.00E-01	1.00E+00	JAM2	7.59E-02	1.00E+00
GABRA3	5.74E+00	1.71E-26	HINT2	1.75E-01	1.00E+00	PHRF1	-6.66E-02	1.00E+00
LOC102415243	-5.32E+00	1.71E-26	ZC4H2	-1.97E-01	1.00E+00	UCK1	-7.86E-02	1.00E+00
TFE3	1.14E+00	1.76E-26	MRPS35	-7.64E-02	1.00E+00	MED12L	1.57E-01	1.00E+00
XYLT1	-3.97E+00	1.79E-26	RMND5A	-7.13E-02	1.00E+00	C8G	-1.10E-01	1.00E+00
VAMP1	-2.15E+00	1.80E-26	COQ7	-9.49E-02	1.00E+00	ZNF236	-1.09E-01	1.00E+00
BNIP3L	-1.06E+00	1.81E-26	MEF2D	9.07E-02	1.00E+00	DBP	1.09E-01	1.00E+00
LOC102406357	-4.50E+00	1.88E-26	RYR1	2.12E-01	1.00E+00	PPARA	-1.02E-01	1.00E+00
LACTB	-1.32E+00	1.89E-26	ANKIB1	1.06E-01	1.00E+00	ADAM9	1.06E-01	1.00E+00
BLZF1	1.16E+00	1.91E-26	LOC102414856	2.11E-01	1.00E+00	DHX58	8.82E-02	1.00E+00
HSD17B12	1.01E+00	1.96E-26	SPATA5	7.63E-02	1.00E+00	RCCD1	-1.08E-01	1.00E+00
NUB1	9.51E-01	1.96E-26	LOC102393963	-3.39E-01	1.00E+00	MARCH8	-7.51E-02	1.00E+00
LOC102397724	4.34E+00	1.97E-26	LOC102394582	-5.95E-02	1.00E+00	GGCX	-7.84E-02	1.00E+00
LOC112578475	-3.83E+00	1.97E-26	UBE2E3	-7.31E-02	1.00E+00	ZNF184	9.63E-02	1.00E+00
LOC102393616	6.03E+00	2.03E-26	SPATA20	-1.84E-01	1.00E+00	MED27	-7.59E-02	1.00E+00
LOC112581850	3.60E+00	2.03E-26	CCSER2	1.05E-01	1.00E+00	NMNAT1	7.39E-02	1.00E+00
LOC102403883	5.28E+00	2.05E-26	PSD2	-8.93E-02	1.00E+00	CEBPA	-1.91E-01	1.00E+00
NDUFA1	-1.84E+00	2.06E-26	TAF12	-7.93E-02	1.00E+00	GLT8D1	-7.76E-02	1.00E+00
KLF6	-2.98E+00	2.07E-26	PHTF2	-1.60E-01	1.00E+00	GNB5	-7.90E-02	1.00E+00
LOC112583775	4.42E+00	2.08E-26	KCNMA1	-7.18E-02	1.00E+00	ZNF513	7.41E-02	1.00E+00
TGFBR3L	-2.66E+00	2.10E-26	FOXK2	5.68E-02	1.00E+00	RASAL1	1.25E-01	1.00E+00
SLC38A5	-5.59E+00	2.12E-26	MCCC2	-2.53E-01	1.00E+00	RTTN	-8.88E-02	1.00E+00
LOC102395151	-6.69E+00	2.14E-26	MYO1A	2.96E-01	1.00E+00	PREX1	2.38E-01	1.00E+00
LOC102415205	2.18E+00	2.14E-26	ABCA13	-2.48E-01	1.00E+00	SEMA6A	1.20E-01	1.00E+00

UFD1	-1.37E+00	2.16E-26	LOC102400687	-4.07E-01	1.00E+00	LOC112581846	-1.97E-01	1.00E+00
DNHD1	-4.11E+00	2.27E-26	UBOX5	9.01E-02	1.00E+00	LLPH	1.04E-01	1.00E+00
KDM2A	-1.17E+00	2.28E-26	CPSF1	7.87E-02	1.00E+00	LRP10	7.45E-02	1.00E+00
DENND3	1.76E+00	2.30E-26	PAXX	-2.78E-01	1.00E+00	AIMP1	-9.46E-02	1.00E+00
TMEM69	1.17E+00	2.39E-26	CDK9	5.96E-02	1.00E+00	FNIP1	9.63E-02	1.00E+00
LOC102414504	-6.53E+00	2.39E-26	SPTLC1	8.85E-02	1.00E+00	FABP4	-2.46E-01	1.00E+00
IL11	4.28E+00	2.40E-26	LOC102395100	1.33E-01	1.00E+00	CCAR1	-7.70E-02	1.00E+00
MLPH	4.46E+00	2.44E-26	CSRNP2	6.51E-02	1.00E+00	IWS1	-6.99E-02	1.00E+00
NDUFB3	-1.67E+00	2.49E-26	NEDD9	4.85E-01	1.00E+00	ARF5	8.93E-02	1.00E+00
OTUD1	-2.29E+00	2.52E-26	LOC112585568	-1.89E-01	1.00E+00	TENT4A	-7.16E-02	1.00E+00
NCR3	-5.16E+00	2.52E-26	LOC102393427	1.59E-01	1.00E+00	B4GALT2	7.59E-02	1.00E+00
KDSR	-1.32E+00	2.54E-26	LOC112580673	-1.69E-01	1.00E+00	STYX	-1.06E-01	1.00E+00
SLC45A1	3.43E+00	2.57E-26	TARS2	2.78E-01	1.00E+00	ZSCAN22	1.38E-01	1.00E+00
LUM	-5.61E+00	2.59E-26	LAYN	2.41E-01	1.00E+00	HMGN1	-7.33E-02	1.00E+00
MED13	-1.60E+00	2.66E-26	UBXN10	-2.14E-01	1.00E+00	CDK4	7.84E-02	1.00E+00
HKDC1	3.59E+00	2.73E-26	LY6K	4.42E-01	1.00E+00	ANKRD40	7.67E-02	1.00E+00
MTERF1	1.60E+00	2.73E-26	CUL3	6.90E-02	1.00E+00	CTNS	1.05E-01	1.00E+00
CLEC2B	-5.58E+00	2.76E-26	CREB3L2	1.34E-01	1.00E+00	OVCH1	-8.49E-02	1.00E+00
LOC102402119	-3.53E+00	2.79E-26	SEMA6D	5.19E-01	1.00E+00	PDCD11	6.59E-02	1.00E+00
DTYMK	-1.95E+00	2.81E-26	USP37	1.04E-01	1.00E+00	PPP1R14B	8.45E-02	1.00E+00
BCL2L2	-1.25E+00	2.81E-26	BARD1	1.14E-01	1.00E+00	EFEMP1	-1.04E-01	1.00E+00
AASDH	1.09E+00	2.91E-26	MPP1	-9.64E-02	1.00E+00	LOC102399387	-1.66E-01	1.00E+00
RNF6	1.25E+00	2.92E-26	GZMM	-3.20E-01	1.00E+00	FAM206A	7.74E-02	1.00E+00
ECM1	-4.24E+00	2.97E-26	FGGY	7.42E-02	1.00E+00	LOC102390897	1.76E-01	1.00E+00
LOC102390746	-4.24E+00	3.03E-26	ABCB7	1.05E-01	1.00E+00	ADCK2	-8.51E-02	1.00E+00
LOC102406753	1.92E+00	3.04E-26	NR1I3	1.42E-01	1.00E+00	ZBTB24	-9.19E-02	1.00E+00
LOC102393350	-1.60E+00	3.05E-26	CCDC47	1.15E-01	1.00E+00	POLR2B	7.10E-02	1.00E+00
SOCS4	-1.24E+00	3.05E-26	UBQLN1	7.11E-02	1.00E+00	TMLHE	8.26E-02	1.00E+00
LOC112585333	-5.26E+00	3.10E-26	PABPC1L	7.75E-02	1.00E+00	WDCP	-1.10E-01	1.00E+00
LOC102393576	2.97E+00	3.13E-26	PTGS2	1.14E-01	1.00E+00	MRPL46	-1.02E-01	1.00E+00
LOC102401061	-4.98E+00	3.14E-26	SUPT4H1	-2.53E-01	1.00E+00	LOC102410234	-9.40E-02	1.00E+00
CEP70	1.16E+00	3.16E-26	C1H21orf2	-1.55E-01	1.00E+00	ARHGEF12	1.04E-01	1.00E+00
FZD8	-3.43E+00	3.18E-26	POLDIP3	6.45E-02	1.00E+00	BAZ1B	6.92E-02	1.00E+00
DPH7	-	3.38E-26	FAM173A	-2.38E-	1.00E+00	LCTL	-1.58E-	1.00E+00

	1.32E+00			01			01	
LOC102402894	5.00E+00	3.43E-26	LOC102402231	1.25E-01	1.00E+00	POP5	-1.01E-01	1.00E+00
LGALS1	-2.37E+00	3.46E-26	RPL15	-9.51E-02	1.00E+00	RAB40C	8.06E-02	1.00E+00
SLC22A17	-5.82E+00	3.56E-26	SLC22A17	5.48E-01	1.00E+00	YKT6	7.41E-02	1.00E+00
DYDC1	2.34E+00	3.62E-26	TMEM170B	1.55E-01	1.00E+00	BLZF1	-8.93E-02	1.00E+00
OTUD7A	3.11E+00	3.62E-26	MINOS1	-8.84E-02	1.00E+00	SMOC2	1.03E-01	1.00E+00
LOC112583901	1.43E+00	3.63E-26	COPS8	-6.59E-02	1.00E+00	HSF4	1.13E-01	1.00E+00
HMGCR	-2.07E+00	3.66E-26	SLC10A7	-9.21E-02	1.00E+00	IL11RA	-1.14E-01	1.00E+00
FBXL22	4.22E+00	3.72E-26	RAB3B	-9.75E-02	1.00E+00	OGFOD3	8.78E-02	1.00E+00
LSP1	-6.04E+00	3.74E-26	POFUT2	1.19E-01	1.00E+00	KPNA3	7.32E-02	1.00E+00
EML5	-1.68E+00	3.80E-26	CKM	-2.62E-01	1.00E+00	SEC22A	9.48E-02	1.00E+00
SLC9B1	1.20E+00	3.80E-26	ALPK3	1.30E-01	1.00E+00	GNPDA1	1.33E-01	1.00E+00
HABP4	-9.94E-01	3.87E-26	GABRG3	-1.07E-01	1.00E+00	TBC1D21	-1.63E-01	1.00E+00
MX1	-6.41E+00	3.87E-26	SESN3	1.61E-01	1.00E+00	PIH1D2	1.14E-01	1.00E+00
PSME4	-1.42E+00	3.89E-26	TET1	-2.56E-01	1.00E+00	SLC35A1	-7.38E-02	1.00E+00
LOC102403863	-4.79E+00	4.02E-26	RAB3GAP2	8.30E-02	1.00E+00	TXNRD3	-1.02E-01	1.00E+00
ZNF319	1.66E+00	4.03E-26	PAPSS1	7.88E-02	1.00E+00	ASNS	7.41E-02	1.00E+00
ALS2CR12	-4.46E+00	4.09E-26	CWF19L1	9.77E-02	1.00E+00	PTPN23	6.83E-02	1.00E+00
SNX16	1.55E+00	4.10E-26	ARL14EP	-6.98E-02	1.00E+00	GREM1	-1.59E-01	1.00E+00
NFKBIZ	-5.14E+00	4.14E-26	LOC112585135	1.30E-01	1.00E+00	PHPT1	1.22E-01	1.00E+00
TRIM36	3.70E+00	4.27E-26	POLA1	7.89E-02	1.00E+00	PSENEN	8.81E-02	1.00E+00
SERHL2	2.69E+00	4.31E-26	APCDD1L	-4.20E-01	1.00E+00	FAM168B	6.33E-02	1.00E+00
DOK1	-2.28E+00	4.43E-26	ASXL1	1.06E-01	1.00E+00	BAZ2B	-1.05E-01	1.00E+00
PIGBOS1	-2.19E+00	4.50E-26	PES1	-6.58E-02	1.00E+00	FUBP1	-7.58E-02	1.00E+00
ITIH4	-5.24E+00	4.50E-26	NLRP12	4.46E-01	1.00E+00	RPL18A	-1.72E-01	1.00E+00
LIMD1	-4.01E+00	4.50E-26	PCBD2	1.62E-01	1.00E+00	RAB19	1.75E-01	1.00E+00
C2H2orf76	1.45E+00	4.53E-26	CDK13	1.38E-01	1.00E+00	MRPL41	1.11E-01	1.00E+00
LOC112579217	-5.94E+00	4.75E-26	S100B	-3.16E-01	1.00E+00	LOC112580132	1.38E-01	1.00E+00
CD4	4.26E+00	4.80E-26	SLC20A2	1.36E-01	1.00E+00	DNAJC15	-7.40E-02	1.00E+00
NOL12	1.38E+00	5.02E-26	SLC12A3	-1.73E-01	1.00E+00	FOLR2	-3.26E-01	1.00E+00
MLLT10	-1.50E+00	5.18E-26	LOC112583612	2.39E-01	1.00E+00	CAMKMT	-8.32E-02	1.00E+00
LOC112579179	3.50E+00	5.32E-26	ZNF7	9.68E-02	1.00E+00	LOC102402119	-1.29E-01	1.00E+00
RPRM	4.62E+00	5.34E-26	TMEM88B	1.94E-01	1.00E+00	PDIA6	-9.89E-02	1.00E+00
TRIM15	5.20E+00	5.34E-26	ATP6V0D1	6.96E-02	1.00E+00	DENND4B	-7.73E-02	1.00E+00

OTX2	6.00E+00	5.41E-26	LOC102413519	2.54E-01	1.00E+00	RPP21	1.15E-01	1.00E+00
C3	-4.76E+00	5.55E-26	PRPF6	6.09E-02	1.00E+00	HARS	6.88E-02	1.00E+00
PAK3	1.39E+00	5.61E-26	UBXN2A	-2.38E-01	1.00E+00	SEC31A	-7.38E-02	1.00E+00
RAB40C	1.20E+00	5.64E-26	PANK1	7.60E-02	1.00E+00	PTPA	7.70E-02	1.00E+00
ELK3	3.02E+00	5.69E-26	SHLD1	-1.38E-01	1.00E+00	RBKS	9.17E-02	1.00E+00
WDR31	1.20E+00	5.89E-26	ATP8B3	4.91E-01	1.00E+00	RPL37	-1.40E-01	1.00E+00
FST	-6.01E+00	5.98E-26	GSK3B	-7.37E-02	1.00E+00	PINK1	7.40E-02	1.00E+00
GPR137B	1.56E+00	6.08E-26	LOC112579418	1.34E-01	1.00E+00	ST3GAL1	-8.43E-02	1.00E+00
LOC112586211	-5.69E+00	6.18E-26	TGS1	8.17E-02	1.00E+00	DDA1	7.87E-02	1.00E+00
CREB5	2.54E+00	6.21E-26	ULK2	8.70E-02	1.00E+00	TRIM13	7.26E-02	1.00E+00
KRT4	3.76E+00	6.35E-26	NOVA2	-3.35E-01	1.00E+00	WRAP73	-9.42E-02	1.00E+00
NF2	1.20E+00	6.36E-26	FAM98A	-9.58E-02	1.00E+00	POP1	8.12E-02	1.00E+00
NAPRT	-2.35E+00	6.40E-26	MAGI1	8.38E-02	1.00E+00	SNRNP200	7.33E-02	1.00E+00
LOC112582308	-4.67E+00	6.45E-26	USP4	-7.81E-02	1.00E+00	FASTKD3	-1.25E-01	1.00E+00
JMJD6	-1.26E+00	6.55E-26	MRPL3	-8.32E-02	1.00E+00	ELP5	8.03E-02	1.00E+00
LOC112580793	-1.27E+00	6.67E-26	ECHDC2	1.06E-01	1.00E+00	EIF3D	-9.56E-02	1.00E+00
SRR	1.05E+00	6.71E-26	NFATC2	-9.93E-02	1.00E+00	MTOR	-6.77E-02	1.00E+00
LOC112583681	5.78E+00	6.75E-26	ARMH3	5.80E-02	1.00E+00	UGGT1	-7.70E-02	1.00E+00
ZNF444	-1.48E+00	6.79E-26	IGF2R	1.12E-01	1.00E+00	RFC1	-6.92E-02	1.00E+00
STX2	1.32E+00	6.84E-26	NUDT8	1.89E-01	1.00E+00	NDUFV1	9.69E-02	1.00E+00
LDOC1	-2.45E+00	6.89E-26	RAD21	7.07E-02	1.00E+00	ASL	1.50E-01	1.00E+00
MICU2	-1.20E+00	7.00E-26	CUX1	-1.10E-01	1.00E+00	RNLS	9.34E-02	1.00E+00
NSUN3	1.28E+00	7.09E-26	LOC112583621	-1.18E-01	1.00E+00	RCN3	-9.99E-02	1.00E+00
RAB33A	-4.62E+00	7.24E-26	SLC25A19	7.66E-02	1.00E+00	PREPL	-1.54E-01	1.00E+00
WRAP53	-1.24E+00	7.25E-26	CHD8	1.17E-01	1.00E+00	IRF2	-7.14E-02	1.00E+00
CDKN2AIPNL	-1.85E+00	7.26E-26	HMGCR	-7.13E-02	1.00E+00	ZNF304	-8.35E-02	1.00E+00
SIAE	-1.25E+00	7.27E-26	SPATA6	1.53E-01	1.00E+00	PHF8	9.80E-02	1.00E+00
AAAS	1.18E+00	7.44E-26	ATXN7L1	7.41E-02	1.00E+00	UAP1	-6.63E-02	1.00E+00
GABRG2	6.08E+00	7.51E-26	ETV1	-1.19E-01	1.00E+00	ZBTB9	-8.44E-02	1.00E+00
METTL5	-1.54E+00	7.56E-26	ORAI2	2.29E-01	1.00E+00	TMEM116	9.67E-02	1.00E+00
PPFIA2	3.48E+00	7.72E-26	TFRC	1.03E-01	1.00E+00	MYO6	-8.12E-02	1.00E+00
DCN	-6.10E+00	7.72E-26	NR2C2AP	1.10E-01	1.00E+00	FAM149B1	-7.15E-02	1.00E+00
IL17D	1.93E+00	7.81E-26	LOC102411198	-1.57E-01	1.00E+00	FPGS	-8.69E-02	1.00E+00
EFCAB11	-	7.87E-26	RNF13	7.62E-02	1.00E+00	PGAM2	1.54E-01	1.00E+00

	1.97E+00							
BATF2	5.15E+00	7.92E-26	ZNF18	7.44E-02	1.00E+00	CIART	1.09E-01	1.00E+00
TOMM40	-1.35E+00	8.17E-26	DACH2	-1.40E-01	1.00E+00	CIRBP	-9.09E-02	1.00E+00
RABEP2	-1.52E+00	8.41E-26	LOC102393534	3.48E-01	1.00E+00	ITGB1	1.03E-01	1.00E+00
NT5DC1	1.33E+00	8.45E-26	CCDC71	-2.31E-01	1.00E+00	LOC102396788	-3.19E-01	1.00E+00
TOX3	4.29E+00	8.69E-26	SLC25A22	6.63E-02	1.00E+00	ANKRD49	8.54E-02	1.00E+00
ATP7B	-1.42E+00	8.71E-26	CNTNAP4	2.60E-01	1.00E+00	RPTOR	-6.27E-02	1.00E+00
ODF2L	-1.39E+00	9.26E-26	TTC38	2.52E-01	1.00E+00	FBXO6	9.18E-02	1.00E+00
KATNA1	-1.17E+00	9.34E-26	PPP4C	8.92E-02	1.00E+00	MTSS1L	8.48E-02	1.00E+00
LOC112580649	-5.04E+00	9.51E-26	SCARA5	2.28E-01	1.00E+00	PRRC2A	-7.86E-02	1.00E+00
SESN3	-1.92E+00	9.52E-26	KLHL7	-7.22E-02	1.00E+00	HSD17B11	8.41E-02	1.00E+00
HCFC1	-1.87E+00	9.72E-26	MMAB	-9.23E-02	1.00E+00	FBXO21	-7.56E-02	1.00E+00
LOC102392935	7.43E+00	9.76E-26	RPA3	-7.73E-02	1.00E+00	RGR	-1.56E-01	1.00E+00
DTWD1	1.30E+00	9.76E-26	POU2F1	7.14E-02	1.00E+00	CCNY	-6.34E-02	1.00E+00
TMEM132D	-5.68E+00	9.83E-26	DNAJC14	6.20E-02	1.00E+00	SRCIN1	1.67E-01	1.00E+00
LGALS7	3.82E+00	9.87E-26	UTP3	1.12E-01	1.00E+00	LOC112580414	-2.68E-01	1.00E+00
TNS3	-1.37E+00	9.95E-26	ASH1L	1.26E-01	1.00E+00	C2H6orf106	6.46E-02	1.00E+00
LOC112585182	2.61E+00	1.00E-25	LOC112585111	-4.54E-01	1.00E+00	DDX17	-9.83E-02	1.00E+00
FAM122B	1.28E+00	1.00E-25	LOC112581533	-1.87E-01	1.00E+00	DERA	7.89E-02	1.00E+00
LRRC24	-1.46E+00	1.02E-25	PDIA4	7.91E-02	1.00E+00	CACNG7	1.36E-01	1.00E+00
S1PR3	-6.52E+00	1.03E-25	TTC32	2.52E-01	1.00E+00	PPP1R35	9.79E-02	1.00E+00
DCP2	-1.13E+00	1.04E-25	EEA1	9.69E-02	1.00E+00	NEURL4	-6.83E-02	1.00E+00
LOC112583863	-4.78E+00	1.04E-25	LOC102400615	-2.00E-01	1.00E+00	TBK1	-8.85E-02	1.00E+00
LOC112580259	3.53E+00	1.05E-25	LOC112582243	-4.81E-01	1.00E+00	NCBP2-AS2	-1.37E-01	1.00E+00
WHAMM	9.82E-01	1.06E-25	LOC102406199	-9.16E-02	1.00E+00	TUBA8	1.75E-01	1.00E+00
PDZD4	3.69E+00	1.06E-25	NAGPA	-8.74E-02	1.00E+00	GPATCH8	-8.39E-02	1.00E+00
FAM47E	1.78E+00	1.08E-25	RABGGTB	1.06E-01	1.00E+00	UTP14A	7.11E-02	1.00E+00
LOC112582320	4.24E+00	1.10E-25	ZNF277	-1.02E-01	1.00E+00	PPP4R4	1.65E-01	1.00E+00
RIBC1	1.74E+00	1.12E-25	NSMAF	7.09E-02	1.00E+00	CEP63	-7.48E-02	1.00E+00
LANCL2	-1.26E+00	1.13E-25	EZH1	7.77E-02	1.00E+00	PLD4	-3.54E-01	1.00E+00
OSR2	2.93E+00	1.13E-25	CLPTM1L	5.69E-02	1.00E+00	CLASP2	-8.09E-02	1.00E+00
EIF2S1	-1.08E+00	1.13E-25	MAPK6	1.03E-01	1.00E+00	NECTIN2	7.02E-02	1.00E+00
TBC1D8	-1.40E+00	1.14E-25	LOC112577713	-8.85E-02	1.00E+00	ZNF287	-1.67E-01	1.00E+00
AP4S1	-	1.15E-25	NRXN2	1.33E-01	1.00E+00	PRKCSH	-1.08E-	1.00E+00

	1.80E+00						01	
ZFHX3	1.71E+00	1.16E-25	EEF1A1	-6.30E-02	1.00E+00	FBRS	-6.69E-02	1.00E+00
LOC102390415	-3.66E+00	1.20E-25	NCF1	-1.95E-01	1.00E+00	CXXC1	-8.02E-02	1.00E+00
EXOSC5	-2.01E+00	1.22E-25	WNT10B	-2.85E-01	1.00E+00	MTIF3	-7.25E-02	1.00E+00
LOC102397046	-4.10E+00	1.22E-25	COL13A1	3.51E-01	1.00E+00	RNF4	-7.00E-02	1.00E+00
LOC112579699	4.68E+00	1.26E-25	RNF181	-1.29E-01	1.00E+00	TMEM249	-1.66E-01	1.00E+00
ZNF805	-1.45E+00	1.28E-25	FGF19	-2.79E-01	1.00E+00	SSNA1	1.05E-01	1.00E+00
LOC102388998	-3.61E+00	1.34E-25	ALDH1L2	1.56E-01	1.00E+00	LMTK3	-1.39E-01	1.00E+00
SERPINE1	-2.30E+00	1.36E-25	MTCL1	-9.46E-02	1.00E+00	PHF11	-6.41E-02	1.00E+00
LOC102394835	-7.87E+00	1.38E-25	ACRV1	3.82E-01	1.00E+00	LOC102400551	1.50E-01	1.00E+00
TREX1	-3.66E+00	1.42E-25	GAPDHS	-4.07E-01	1.00E+00	PGM1	-6.90E-02	1.00E+00
LOC102397166	5.45E+00	1.44E-25	HNRNPA2B1	8.81E-02	1.00E+00	B3GAT3	9.17E-02	1.00E+00
LOC102398188	-3.39E+00	1.44E-25	MAD2L1	-6.91E-02	1.00E+00	LOC102390415	-1.35E-01	1.00E+00
BCORL1	1.93E+00	1.46E-25	CALM2	1.23E-01	1.00E+00	RNF139	-8.11E-02	1.00E+00
RPA1	1.09E+00	1.47E-25	UBTF	-1.15E-01	1.00E+00	LOC102389249	-6.32E-02	1.00E+00
SLC38A3	2.66E+00	1.49E-25	PEBP4	-2.04E-01	1.00E+00	LOC102411445	1.72E-01	1.00E+00
WBP4	9.84E-01	1.50E-25	NPFFR1	3.85E-01	1.00E+00	HMGXB3	6.88E-02	1.00E+00
C18H16orf70	1.02E+00	1.51E-25	CTSV	2.98E-01	1.00E+00	PCM1	-8.13E-02	1.00E+00
LOC112586230	4.01E+00	1.53E-25	THAP8	1.16E-01	1.00E+00	LOC102401443	-1.54E-01	1.00E+00
TLR6	-4.38E+00	1.56E-25	MGMT	-6.58E-02	1.00E+00	KIF3B	7.86E-02	1.00E+00
INSIG2	1.32E+00	1.56E-25	CRCP	1.46E-01	1.00E+00	GPS1	-9.95E-02	1.00E+00
LOC102404409	-2.50E+00	1.59E-25	TIGAR	1.14E-01	1.00E+00	NEURL1B	-1.01E-01	1.00E+00
SPHAR	-3.40E+00	1.60E-25	MRPS11	-8.74E-02	1.00E+00	PPM1F	7.37E-02	1.00E+00
LOC102390919	4.56E+00	1.60E-25	IQGAP3	1.14E-01	1.00E+00	PCGF5	8.57E-02	1.00E+00
CCDC15	1.09E+00	1.60E-25	CTNNA1	6.62E-02	1.00E+00	MYO9A	8.89E-02	1.00E+00
THNSL1	1.72E+00	1.60E-25	MIEF2	-1.01E-01	1.00E+00	ADAR	8.63E-02	1.00E+00
LOC102408093	1.21E+00	1.65E-25	BRCA2	8.48E-02	1.00E+00	NDUFB3	9.19E-02	1.00E+00
RBAK	-1.80E+00	1.69E-25	CALB2	-1.24E-01	1.00E+00	C5H1orf174	8.31E-02	1.00E+00
LARP1B	1.18E+00	1.74E-25	OTUD6B	7.04E-02	1.00E+00	MAPK9	-6.86E-02	1.00E+00
CCDC174	-2.12E+00	1.75E-25	PLEKHA5	-1.73E-01	1.00E+00	MED13	8.22E-02	1.00E+00
MORN2	-2.16E+00	1.77E-25	TM9SF3	6.70E-02	1.00E+00	DNAI2	-1.17E-01	1.00E+00
RNF208	-5.78E+00	1.80E-25	ITFG1	-8.63E-02	1.00E+00	LOC102391124	-1.97E-01	1.00E+00
GDPD3	-4.05E+00	1.82E-25	FANCC	1.02E-01	1.00E+00	TCF20	7.04E-02	1.00E+00
MRPL50	-1.82E+00	1.86E-25	SLC19A3	4.42E-01	1.00E+00	LOC102411226	-1.03E-01	1.00E+00

STK38	-1.43E+00	1.88E-25	MTHFD2	-7.99E-02	1.00E+00	RLF	-6.89E-02	1.00E+00
ARHGEF4	-3.46E+00	1.88E-25	UNC50	-1.14E-01	1.00E+00	RGL1	1.11E-01	1.00E+00
LRCH3	1.45E+00	1.88E-25	GALNS	2.56E-01	1.00E+00	BRD1	-8.50E-02	1.00E+00
ZNF576	-1.56E+00	1.89E-25	RBM19	1.27E-01	1.00E+00	PSMB6	1.01E-01	1.00E+00
DOK7	-4.36E+00	1.91E-25	MESP2	-1.89E-01	1.00E+00	IFFO1	-7.80E-02	1.00E+00
MAP4K4	-1.09E+00	1.92E-25	SLC25A53	-6.57E-02	1.00E+00	CXCL16	-1.02E-01	1.00E+00
ALDH6A1	-3.44E+00	1.96E-25	PPP2R2B	-8.82E-02	1.00E+00	ZBTB8A	1.31E-01	1.00E+00
LOC112586639	6.08E+00	1.96E-25	LOC102398106	-7.57E-02	1.00E+00	CCDC82	-7.57E-02	1.00E+00
EML3	-1.50E+00	1.96E-25	IMMP2L	-7.41E-02	1.00E+00	SRBD1	-6.79E-02	1.00E+00
LOC112584442	4.17E+00	1.96E-25	NDUFA3	-1.04E-01	1.00E+00	STAU2	-6.74E-02	1.00E+00
NDP	-6.70E+00	1.97E-25	AP1S1	-8.22E-02	1.00E+00	ZCCHC2	-1.01E-01	1.00E+00
WDHD1	-1.20E+00	2.09E-25	LOC112581104	-3.84E-01	1.00E+00	LOC102412418	1.10E-01	1.00E+00
ARL9	-2.18E+00	2.09E-25	FOXRED2	9.27E-02	1.00E+00	TBX3	1.85E-01	1.00E+00
TLR10	-5.59E+00	2.11E-25	ELOVL1	9.10E-02	1.00E+00	DEPDC5	-7.75E-02	1.00E+00
DYNC1H1	-1.53E+00	2.13E-25	TSG101	6.56E-02	1.00E+00	PNPLA8	7.83E-02	1.00E+00
PTPRE	-4.27E+00	2.13E-25	ISYNA1	-1.51E-01	1.00E+00	LOC102399593	1.58E-01	1.00E+00
AIFM1	-1.26E+00	2.18E-25	ACBD4	-1.50E-01	1.00E+00	LOC102390463	1.48E-01	1.00E+00
LOC102410920	1.48E+00	2.20E-25	CCDC82	8.10E-02	1.00E+00	LOC102389028	9.84E-02	1.00E+00
UBAP2	1.10E+00	2.29E-25	LOC102390920	-1.04E-01	1.00E+00	XPR1	-8.44E-02	1.00E+00
GOLGA3	1.63E+00	2.30E-25	ZXDC	-1.08E-01	1.00E+00	STIP1	7.79E-02	1.00E+00
EEF1AKMT2	-2.04E+00	2.31E-25	PNPLA8	8.14E-02	1.00E+00	NPRL3	-7.35E-02	1.00E+00
NCKAP5L	1.19E+00	2.34E-25	IGF2BP2	8.45E-02	1.00E+00	GAR1	-8.03E-02	1.00E+00
ZNF668	1.23E+00	2.38E-25	ACAD11	-1.81E-01	1.00E+00	MIEF2	9.74E-02	1.00E+00
LOC112585316	-5.66E+00	2.38E-25	RASGEF1A	7.52E-02	1.00E+00	RRM2B	-1.02E-01	1.00E+00
SCLY	2.35E+00	2.41E-25	TOP1	1.01E-01	1.00E+00	YBEY	1.06E-01	1.00E+00
R3HDM4	-1.11E+00	2.42E-25	CCDC134	8.87E-02	1.00E+00	LOC102413450	-2.08E-01	1.00E+00
FRMD7	2.96E+00	2.54E-25	CFAP57	-3.17E-01	1.00E+00	LOC112585160	-1.31E-01	1.00E+00
RCC2	-1.04E+00	2.55E-25	RASL11B	-3.18E-01	1.00E+00	LOC102396583	3.30E-01	1.00E+00
LOC112579701	5.19E+00	2.64E-25	ZBED5	-1.60E-01	1.00E+00	XPO5	7.21E-02	1.00E+00
UNC13D	-3.65E+00	2.75E-25	CITED1	-5.06E-01	1.00E+00	LOXL1	-2.29E-01	1.00E+00
LOC102410286	-3.14E+00	2.83E-25	SLC1A1	3.00E-01	1.00E+00	MITF	1.55E-01	1.00E+00
B4GALNT4	-2.00E+00	2.88E-25	TMEM50B	9.12E-02	1.00E+00	LOC102391307	7.02E-02	1.00E+00
LOC102398760	5.12E+00	3.02E-25	LOC112585093	-1.20E-01	1.00E+00	LOC112583923	1.44E-01	1.00E+00

COQ4	-1.57E+00	3.04E-25	VCP	6.21E-02	1.00E+00	CRTAP	-7.79E-02	1.00E+00
LOC112580692	6.25E+00	3.14E-25	TMEM134	-1.09E-01	1.00E+00	NARF	-6.98E-02	1.00E+00
PKIA	-2.59E+00	3.14E-25	ATL2	-6.88E-02	1.00E+00	PIAS4	-7.21E-02	1.00E+00
LOC112587789	4.39E+00	3.19E-25	FXR1	6.07E-02	1.00E+00	RRAS	1.18E-01	1.00E+00
ANP32E	-9.58E-01	3.24E-25	NKAPD1	6.18E-02	1.00E+00	NGDN	-7.76E-02	1.00E+00
RABGAP1L	1.06E+00	3.25E-25	PRELID3B	-1.10E-01	1.00E+00	STX10	-1.07E-01	1.00E+00
FIBP	-1.40E+00	3.32E-25	LOC112578820	-1.53E-01	1.00E+00	GEMIN2	-7.58E-02	1.00E+00
RABEPK	-2.06E+00	3.40E-25	LFNG	-1.69E-01	1.00E+00	INTS9	-7.11E-02	1.00E+00
LOC112581357	-6.04E+00	3.40E-25	ABHD2	1.08E-01	1.00E+00	HNRNPU	-8.90E-02	1.00E+00
MYO16	3.59E+00	3.43E-25	IL11RA	-2.58E-01	1.00E+00	LOC102397580	7.36E-02	1.00E+00
ZNF605	-1.97E+00	3.44E-25	LOC112584557	-1.99E-01	1.00E+00	TSC22D1	1.11E-01	1.00E+00
LOC102402992	-4.52E+00	3.57E-25	PDCL3	-2.35E-01	1.00E+00	USP14	6.83E-02	1.00E+00
PRDM8	2.71E+00	3.60E-25	LOC112586949	-2.69E-01	1.00E+00	SHROOM2	-9.79E-02	1.00E+00
GINS2	1.30E+00	3.62E-25	G2E3	-1.48E-01	1.00E+00	FAH	-1.50E-01	1.00E+00
LOC112587785	-2.36E+00	3.67E-25	STXBP2	-7.99E-02	1.00E+00	EIF2S3	-8.16E-02	1.00E+00
AXDND1	1.81E+00	3.68E-25	UBE2B	-7.31E-02	1.00E+00	PYCR1	-1.02E-01	1.00E+00
COL27A1	-3.14E+00	3.71E-25	LMLN	1.26E-01	1.00E+00	FAM166B	1.42E-01	1.00E+00
LOC112577707	-2.06E+00	3.75E-25	DDAH2	1.02E-01	1.00E+00	OLFML2A	1.58E-01	1.00E+00
NDUFA2	-1.61E+00	3.76E-25	TUSC2	-2.38E-01	1.00E+00	RHEB	6.63E-02	1.00E+00
TFF1	-7.64E+00	3.79E-25	CRYZL1	-1.27E-01	1.00E+00	CHSY1	-8.18E-02	1.00E+00
TTLL11	2.14E+00	3.81E-25	PSMD6	7.37E-02	1.00E+00	ZNF407	-7.65E-02	1.00E+00
TCEAL9	-5.22E+00	3.83E-25	SLC38A9	-8.06E-02	1.00E+00	YPEL4	-1.36E-01	1.00E+00
CRAMP1	1.43E+00	3.89E-25	KLF10	-5.28E-01	1.00E+00	SEM1	1.00E-01	1.00E+00
CCNA2	1.27E+00	3.90E-25	GPC4	6.64E-02	1.00E+00	TPGS1	9.09E-02	1.00E+00
ACSL6	1.92E+00	3.93E-25	SLC25A46	1.37E-01	1.00E+00	ACSL6	-1.48E-01	1.00E+00
LOC112582352	5.80E+00	3.98E-25	ANAPC15	-6.74E-02	1.00E+00	SP1	-7.12E-02	1.00E+00
LRFN3	-2.06E+00	3.99E-25	TMEM185A	-8.45E-02	1.00E+00	WDR53	9.84E-02	1.00E+00
PDZD3	5.17E+00	4.02E-25	HELLS	6.58E-02	1.00E+00	ECHS1	-9.05E-02	1.00E+00
DAAM2	1.67E+00	4.06E-25	LOC112580268	3.98E-01	1.00E+00	LOC102397346	-1.67E-01	1.00E+00
UBE2O	-1.09E+00	4.49E-25	SGSM2	5.89E-02	1.00E+00	NIPAL1	1.58E-01	1.00E+00
HHLA2	1.51E+00	4.50E-25	LOC102410517	-7.42E-02	1.00E+00	ZBTB4	-7.75E-02	1.00E+00
THAP5	1.26E+00	4.58E-25	SHOX2	1.38E-01	1.00E+00	GTPBP10	-7.93E-02	1.00E+00
HTR5A	5.52E+00	4.62E-25	STK3	-1.07E-01	1.00E+00	SRI	7.72E-02	1.00E+00

LOC102403234	-5.82E+00	4.64E-25	MYO10	9.57E-02	1.00E+00	FES	1.57E-01	1.00E+00
HIVEP2	-4.51E+00	4.75E-25	NDUFA5	-1.08E-01	1.00E+00	PITPNM2	1.08E-01	1.00E+00
VCPKMT	-1.82E+00	4.75E-25	RPS28	-1.06E-01	1.00E+00	LOC102390517	-8.50E-02	1.00E+00
IRAK1	-1.07E+00	4.78E-25	RMDN2	-2.45E-01	1.00E+00	ABCG5	1.82E-01	1.00E+00
TRAF3	1.09E+00	4.83E-25	CGNL1	1.08E-01	1.00E+00	RFESD	1.33E-01	1.00E+00
GNS	1.72E+00	4.88E-25	PTPRB	-2.36E-01	1.00E+00	ATP5MPL	9.84E-02	1.00E+00
RPN1	-1.39E+00	4.92E-25	EDN3	-3.34E-01	1.00E+00	YBX3	8.97E-02	1.00E+00
HTR6	-4.08E+00	4.96E-25	PNMA8A	-2.98E-01	1.00E+00	GCN1	-6.55E-02	1.00E+00
LOC102401106	2.38E+00	4.97E-25	E2F2	8.86E-02	1.00E+00	MCRIP1	8.45E-02	1.00E+00
SACS	-2.14E+00	4.97E-25	ENPP1	7.76E-02	1.00E+00	RBM38	-8.99E-02	1.00E+00
CRTAC1	3.22E+00	5.00E-25	PCYT2	-1.61E-01	1.00E+00	MTRF1	1.14E-01	1.00E+00
C3H9orf84	-4.55E+00	5.00E-25	PRKAG2	2.40E-01	1.00E+00	FAM208B	-9.35E-02	1.00E+00
LOC102404203	3.14E+00	5.03E-25	CCDC151	-1.40E-01	1.00E+00	LOC112587821	1.50E-01	1.00E+00
LOC112582336	5.91E+00	5.09E-25	CEBPZOS	-1.46E-01	1.00E+00	EIF1	-1.02E-01	1.00E+00
PLEKHA3	-1.46E+00	5.14E-25	GLI2	-1.79E-01	1.00E+00	UPRT	6.79E-02	1.00E+00
SEMA4F	-1.09E+00	5.29E-25	MRPS15	-1.01E-01	1.00E+00	PRMT7	-7.30E-02	1.00E+00
NPLOC4	9.52E-01	5.36E-25	ZNF524	-1.12E-01	1.00E+00	PLEKHB2	6.82E-02	1.00E+00
ZNF157	-3.36E+00	5.63E-25	ALS2CL	-2.35E-01	1.00E+00	MED24	-6.34E-02	1.00E+00
NEURL1	3.41E+00	5.76E-25	AURKA	7.47E-02	1.00E+00	TMEM104	7.44E-02	1.00E+00
SLC37A2	-6.45E+00	5.87E-25	EIF2B2	9.18E-02	1.00E+00	LYRM2	-9.52E-02	1.00E+00
NPR1	-3.04E+00	5.94E-25	SPOCK2	1.76E-01	1.00E+00	ENPP2	1.51E-01	1.00E+00
CLDN11	-7.53E+00	6.09E-25	DALRD3	-2.25E-01	1.00E+00	FBLN5	1.32E-01	1.00E+00
FAM83C	3.38E+00	6.15E-25	ZFYVE16	-1.26E-01	1.00E+00	FAM181B	1.61E-01	1.00E+00
HSP90B1	-2.14E+00	6.16E-25	LOC102409265	-2.05E-01	1.00E+00	ACYP2	9.99E-02	1.00E+00
LOC102416327	2.38E+00	6.32E-25	MITF	1.72E-01	1.00E+00	BTBD19	1.52E-01	1.00E+00
LOC102404258	-5.35E+00	6.35E-25	ZNF76	7.21E-02	1.00E+00	ZCCHC17	-9.08E-02	1.00E+00
HILPDA	1.41E+00	6.46E-25	CNTN5	1.23E-01	1.00E+00	EXOG	-9.68E-02	1.00E+00
ALKBH1	-1.31E+00	6.50E-25	TMCO6	7.18E-02	1.00E+00	C14H20orf27	7.94E-02	1.00E+00
ZNF79	-1.36E+00	6.55E-25	LOC112578031	-3.52E-01	1.00E+00	DCTN6	-6.24E-02	1.00E+00
LOC102402672	1.96E+00	6.59E-25	CRYAB	-1.79E-01	1.00E+00	PPIA	1.04E-01	1.00E+00
CHUK	1.14E+00	6.64E-25	FAM3C	-2.06E-01	1.00E+00	ABTB1	7.19E-02	1.00E+00
TMEM206	-1.43E+00	6.72E-25	MARK3	-6.51E-02	1.00E+00	C7H4orf3	6.94E-02	1.00E+00
BRMS1	-1.43E+00	6.83E-25	TRIB1	-1.47E-01	1.00E+00	TACC3	-9.55E-02	1.00E+00
RRP8	1.23E+00	6.83E-25	CCDC63	-2.51E-	1.00E+00	ADAM11	-1.57E-	1.00E+00

				01			01	
DYRK4	4.15E+00	6.93E-25	LOC102398382	-3.02E-01	1.00E+00	RTN3	-6.39E-02	1.00E+00
DHTKD1	-1.31E+00	6.99E-25	TENM1	9.94E-02	1.00E+00	ADGRE5	9.33E-02	1.00E+00
LOC102402718	5.65E+00	7.11E-25	MTX3	-1.45E-01	1.00E+00	TMED4	-7.62E-02	1.00E+00
EEF1A2	-4.91E+00	7.24E-25	CASZ1	1.94E-01	1.00E+00	MTMR12	-9.54E-02	1.00E+00
USP10	8.94E-01	7.31E-25	KMT5B	-5.96E-02	1.00E+00	TLE6	-1.05E-01	1.00E+00
RARA	-1.35E+00	7.36E-25	CGN	8.48E-02	1.00E+00	LOC102396503	-1.04E-01	1.00E+00
LOC102407141	7.88E+00	7.37E-25	RNASEH1	-9.27E-02	1.00E+00	ACBD7	1.49E-01	1.00E+00
CRTC2	9.90E-01	7.37E-25	TMEM184B	9.77E-02	1.00E+00	CCDC93	6.62E-02	1.00E+00
PACSIN3	-2.75E+00	7.58E-25	LOC112578005	-1.06E-01	1.00E+00	FAM104A	-1.23E-01	1.00E+00
LOC102397448	4.32E+00	7.60E-25	CEP85L	-1.71E-01	1.00E+00	EVA1A	1.63E-01	1.00E+00
LOC112585299	-4.15E+00	7.66E-25	RHBDL3	-3.41E-01	1.00E+00	SEC63	6.53E-02	1.00E+00
BET1	1.03E+00	7.72E-25	FDX2	-8.18E-02	1.00E+00	ING4	-7.04E-02	1.00E+00
MATN2	-2.70E+00	7.76E-25	ERCC3	5.35E-02	1.00E+00	EMC10	-7.67E-02	1.00E+00
LOC112587874	-4.35E+00	7.77E-25	LOC102406142	2.54E-01	1.00E+00	SHB	-1.07E-01	1.00E+00
C5H11orf24	1.15E+00	7.84E-25	VSIG10L	-2.61E-01	1.00E+00	MICU3	-1.30E-01	1.00E+00
PTCH1	-1.69E+00	8.04E-25	NDUFB3	-1.21E-01	1.00E+00	EMC4	7.54E-02	1.00E+00
FUCA1	-1.08E+00	8.04E-25	AGAP3	7.98E-02	1.00E+00	CUEDC2	9.62E-02	1.00E+00
GCM2	5.15E+00	8.09E-25	TRAF7	5.74E-02	1.00E+00	SNX3	6.57E-02	1.00E+00
KCNG4	-3.31E+00	8.14E-25	SAMD11	4.18E-01	1.00E+00	ZNF674	-9.46E-02	1.00E+00
PSMG3	-1.72E+00	8.17E-25	PRSS50	1.21E-01	1.00E+00	BRD7	-6.39E-02	1.00E+00
CDK3	-5.10E+00	8.19E-25	MFSD8	-6.94E-02	1.00E+00	PEBP1	-1.05E-01	1.00E+00
SNF8	-1.45E+00	8.23E-25	WDR45	1.09E-01	1.00E+00	MTLN	1.20E-01	1.00E+00
NUP153	1.51E+00	8.31E-25	CTDNEP1	6.50E-02	1.00E+00	TRAFD1	6.53E-02	1.00E+00
LOC102409570	1.23E+00	8.38E-25	GCAT	7.85E-02	1.00E+00	SWT1	-7.69E-02	1.00E+00
CUL1	-9.17E-01	8.40E-25	ATP6V1C1	-6.09E-02	1.00E+00	SLC25A32	7.60E-02	1.00E+00
C5	4.35E+00	8.56E-25	RRAGD	1.01E-01	1.00E+00	ISPD	1.01E-01	1.00E+00
PSMC2	-1.19E+00	8.57E-25	YAE1	-8.39E-02	1.00E+00	ZNF277	-7.43E-02	1.00E+00
MPC2	-1.64E+00	8.78E-25	LOC112583635	1.61E-01	1.00E+00	GULP1	1.08E-01	1.00E+00
SVEP1	2.92E+00	8.89E-25	UTP18	-5.92E-02	1.00E+00	CYSTM1	-7.19E-02	1.00E+00
APOD	-3.49E+00	9.06E-25	P2RX2	-2.35E-01	1.00E+00	RASA3	-1.14E-01	1.00E+00
HTATIP2	1.36E+00	9.25E-25	SLX4	8.98E-02	1.00E+00	GTF2E1	8.07E-02	1.00E+00
LOC102394298	-5.39E+00	9.29E-25	MAP6D1	-1.97E-01	1.00E+00	CCDC159	-1.47E-01	1.00E+00
EDAR	3.69E+00	9.61E-25	EPHA6	-1.84E-01	1.00E+00	LOC102389189	-1.42E-01	1.00E+00

SPINK13	-5.78E+00	9.86E-25	LOC102395792	9.46E-02	1.00E+00	UBL3	8.26E-02	1.00E+00
RILPL1	-1.01E+00	9.87E-25	LOC102402715	-9.37E-02	1.00E+00	DYNC2LI1	-7.28E-02	1.00E+00
HOXD8	1.40E+00	9.91E-25	ZBTB49	6.08E-02	1.00E+00	RAB11B	-6.54E-02	1.00E+00
BMPER	-5.41E+00	9.91E-25	LOC102391948	-3.53E-01	1.00E+00	SPPL3	-1.07E-01	1.00E+00
STXBP2	-1.21E+00	1.00E-24	ZNF174	5.63E-02	1.00E+00	LOC102401621	-2.28E-01	1.00E+00
INSL3	-6.67E+00	1.03E-24	YPEL5	-6.72E-02	1.00E+00	PHF13	-7.95E-02	1.00E+00
EIF4H	-1.18E+00	1.04E-24	SLC6A20	-8.60E-02	1.00E+00	ACAT1	-8.42E-02	1.00E+00
LOC102397454	1.44E+00	1.04E-24	MDGA1	1.63E-01	1.00E+00	MIB2	-7.33E-02	1.00E+00
TIMM8B	-1.87E+00	1.07E-24	SRR	-7.81E-02	1.00E+00	LOC102399225	-1.53E-01	1.00E+00
LIAS	1.16E+00	1.08E-24	DPP7	-2.40E-01	1.00E+00	BCLAF1	-1.07E-01	1.00E+00
LOC102395814	-1.54E+00	1.10E-24	TMEM98	-3.25E-01	1.00E+00	SPAG1	-8.88E-02	1.00E+00
MBTD1	-2.02E+00	1.13E-24	AGPAT5	-9.89E-02	1.00E+00	LOC102410010	1.41E-01	1.00E+00
ZNF789	-4.05E+00	1.15E-24	TSGA10	1.68E-01	1.00E+00	ACO2	-7.59E-02	1.00E+00
GTPBP1	1.03E+00	1.17E-24	LOC102389178	1.77E-01	1.00E+00	NSD3	-7.49E-02	1.00E+00
LOC112585588	5.38E+00	1.18E-24	ZNF608	1.27E-01	1.00E+00	POGK	6.88E-02	1.00E+00
USP43	1.74E+00	1.19E-24	ZNF653	-1.09E-01	1.00E+00	LOC102393855	9.13E-02	1.00E+00
LOC102407589	3.33E+00	1.19E-24	PDLIM3	-2.91E-01	1.00E+00	NR2F6	-8.62E-02	1.00E+00
LOC102413667	-4.90E+00	1.20E-24	POLR1A	8.64E-02	1.00E+00	IFITM5	-1.21E-01	1.00E+00
TEKT2	-4.35E+00	1.22E-24	KCNB2	-3.19E-01	1.00E+00	HINT2	1.06E-01	1.00E+00
C5H1orf159	-1.89E+00	1.23E-24	NAPSA	1.58E-01	1.00E+00	KBTBD11	1.50E-01	1.00E+00
FOXC1	4.04E+00	1.28E-24	DEDD2	9.77E-02	1.00E+00	MEPCE	7.76E-02	1.00E+00
SERF2	-1.89E+00	1.28E-24	KCNQ4	1.64E-01	1.00E+00	CCDC157	-1.24E-01	1.00E+00
ABCB7	-1.09E+00	1.28E-24	FLNA	-3.42E-01	1.00E+00	HSP90AB1	-1.17E-01	1.00E+00
LOC112584562	2.84E+00	1.29E-24	PKIG	-1.09E-01	1.00E+00	PEX5	6.42E-02	1.00E+00
ZBTB32	3.57E+00	1.30E-24	ARL15	6.89E-02	1.00E+00	VPS33A	-7.23E-02	1.00E+00
C2H2orf69	-1.99E+00	1.30E-24	HCFC1R1	-1.65E-01	1.00E+00	LOC102398213	8.42E-02	1.00E+00
MFSD14A	-1.33E+00	1.32E-24	ZBTB1	-4.26E-01	1.00E+00	ZNF174	8.67E-02	1.00E+00
FOXP2	-2.32E+00	1.32E-24	CENPP	8.68E-02	1.00E+00	TPM2	-1.20E-01	1.00E+00
LAMB1	-2.18E+00	1.36E-24	FSTL3	-1.59E-01	1.00E+00	MPLKIP	-9.73E-02	1.00E+00
MEIKIN	5.10E+00	1.39E-24	CBX6	9.04E-02	1.00E+00	SEPHS1	-6.94E-02	1.00E+00
SCAND1	-1.58E+00	1.42E-24	MTERF1	-1.07E-01	1.00E+00	PRKAG1	-6.63E-02	1.00E+00
TOB1	-1.98E+00	1.42E-24	TMEM138	1.17E-01	1.00E+00	SNX19	6.17E-02	1.00E+00
LOC102408429	-3.79E+00	1.44E-24	C17H12orf43	-6.43E-02	1.00E+00	PITPNB	6.04E-02	1.00E+00

STAB1	-3.51E+00	1.45E-24	STT3B	7.66E-02	1.00E+00	GSPT2	7.34E-02	1.00E+00
SAYSD1	2.14E+00	1.49E-24	ZFP42	4.13E-01	1.00E+00	ASH1L	-9.42E-02	1.00E+00
FAM174B	2.81E+00	1.51E-24	LOC112583859	-2.76E-01	1.00E+00	LOC102404358	8.08E-02	1.00E+00
MARCH4	-3.37E+00	1.55E-24	GTF2H3	2.44E-01	1.00E+00	CEP97	-9.66E-02	1.00E+00
LOC102397289	3.32E+00	1.58E-24	TBCB	-6.16E-02	1.00E+00	TMEM223	8.63E-02	1.00E+00
ARHGEF18	-1.18E+00	1.61E-24	LOC102399663	-1.09E-01	1.00E+00	NSG1	1.47E-01	1.00E+00
TMEM131L	1.09E+00	1.63E-24	GNB1L	-1.02E-01	1.00E+00	SH3GLB2	-7.30E-02	1.00E+00
SERTAD2	-3.69E+00	1.63E-24	PRR13	-6.99E-02	1.00E+00	CDK17	9.44E-02	1.00E+00
RASL11A	-2.91E+00	1.63E-24	TMEM241	-1.26E-01	1.00E+00	LOC102398768	1.51E-01	1.00E+00
COX2	-3.30E+00	1.69E-24	TRIM3	6.91E-02	1.00E+00	LOC102397684	8.79E-02	1.00E+00
GMPR2	9.46E-01	1.70E-24	LOC112578408	-2.05E-01	1.00E+00	DHODH	9.21E-02	1.00E+00
LOC102397580	9.91E-01	1.76E-24	LOC102412769	-2.02E-01	1.00E+00	UBE2K	6.32E-02	1.00E+00
LOC112587869	4.87E+00	1.81E-24	LONRF3	1.59E-01	1.00E+00	ZNF609	-8.03E-02	1.00E+00
TIGD5	-1.67E+00	1.87E-24	BABAM2	-6.89E-02	1.00E+00	ZNF697	-1.29E-01	1.00E+00
ZNF81	-2.51E+00	1.93E-24	RGS11	2.15E-01	1.00E+00	ENTPD3	1.64E-01	1.00E+00
LOC112587821	-4.10E+00	1.95E-24	ZMYM2	7.90E-02	1.00E+00	ABHD11	1.01E-01	1.00E+00
NASP	1.50E+00	1.96E-24	USP8	6.54E-02	1.00E+00	FAM193B	-6.52E-02	1.00E+00
MCM4	1.34E+00	1.96E-24	SMC1B	-6.95E-02	1.00E+00	BIRC6	9.06E-02	1.00E+00
ADAM17	-1.27E+00	1.99E-24	LOC112584692	3.19E-01	1.00E+00	COL18A1	-9.68E-02	1.00E+00
TRIP11	1.34E+00	2.01E-24	RAB18	-6.49E-02	1.00E+00	DHX36	-7.19E-02	1.00E+00
FAM234A	-1.35E+00	2.07E-24	LOC112581397	-1.70E-01	1.00E+00	MAGEL2	-1.06E-01	1.00E+00
TIGD2	1.24E+00	2.08E-24	MCU	-6.29E-02	1.00E+00	CITED4	-1.25E-01	1.00E+00
LOC112584667	3.05E+00	2.10E-24	LOC112583669	-2.85E-01	1.00E+00	TMEM128	-8.54E-02	1.00E+00
SCG2	-6.75E+00	2.10E-24	USP7	7.35E-02	1.00E+00	PPL	1.65E-01	1.00E+00
NUCB2	-1.71E+00	2.10E-24	TMEM169	7.69E-02	1.00E+00	PSMC6	6.33E-02	1.00E+00
HSD17B10	-1.70E+00	2.16E-24	AXDND1	-1.26E-01	1.00E+00	DYNLRB1	9.11E-02	1.00E+00
HINFP	9.79E-01	2.19E-24	MAPK3	7.97E-02	1.00E+00	LOC112585628	-1.50E-01	1.00E+00
LOC102399426	-7.75E+00	2.25E-24	PTDSS1	-6.37E-02	1.00E+00	GTF3C1	-6.17E-02	1.00E+00
ZIC2	4.90E+00	2.27E-24	SSC4D	-1.87E-01	1.00E+00	TICAM2	-7.66E-02	1.00E+00
CHRD	-2.39E+00	2.33E-24	ND6	-1.55E-01	1.00E+00	RUFY1	6.39E-02	1.00E+00
KLK10	-4.59E+00	2.34E-24	HEY1	6.82E-02	1.00E+00	SLC25A14	-9.95E-02	1.00E+00
LOC102404254	-8.16E+00	2.35E-24	LOC102393901	-7.35E-02	1.00E+00	TEK	1.77E-01	1.00E+00

LOC112583838	-5.48E+00	2.36E-24	DYNC1H1	1.01E-01	1.00E+00	LOC102398530	-6.98E-02	1.00E+00
UPK3B	-4.16E+00	2.43E-24	TRDMT1	1.21E-01	1.00E+00	LOC112580253	-1.54E-01	1.00E+00
CDK2AP1	1.16E+00	2.43E-24	LOC102394020	-1.31E-01	1.00E+00	LSM11	1.07E-01	1.00E+00
KHK	-1.91E+00	2.51E-24	RSF1	1.13E-01	1.00E+00	LOC102390176	-1.07E-01	1.00E+00
LOC112581256	-5.75E+00	2.52E-24	ATMIN	5.77E-02	1.00E+00	AADACL3	1.51E-01	1.00E+00
LOC112578124	1.85E+00	2.52E-24	MOCS2	-1.07E-01	1.00E+00	ARL3	9.77E-02	1.00E+00
NUP50	-9.36E-01	2.60E-24	ND2	-1.37E-01	1.00E+00	NT5C3A	-7.87E-02	1.00E+00
GUCA2A	5.32E+00	2.62E-24	MIA2	7.58E-02	1.00E+00	TOGARAM1	-8.74E-02	1.00E+00
IRAK3	2.67E+00	2.66E-24	HOOK3	7.93E-02	1.00E+00	ZNRD1	8.27E-02	1.00E+00
EHMT1	-1.10E+00	2.68E-24	CCDC115	1.08E-01	1.00E+00	KCNC4	-1.48E-01	1.00E+00
DLL4	-6.53E+00	2.71E-24	SPATA7	9.82E-02	1.00E+00	RPGRIP1L	9.83E-02	1.00E+00
CSAD	-1.62E+00	2.73E-24	HLF	6.94E-02	1.00E+00	NDN	8.13E-02	1.00E+00
DCTPP1	-2.23E+00	2.79E-24	LOC112585715	4.15E-01	1.00E+00	RRAD	1.02E-01	1.00E+00
GBGT1	-1.57E+00	2.82E-24	ASAP1	7.24E-02	1.00E+00	HTATIP2	8.22E-02	1.00E+00
LOC102402751	-6.79E+00	2.86E-24	PRPF40B	8.66E-02	1.00E+00	LOC102416057	-1.90E-01	1.00E+00
OVOL1	8.22E+00	2.87E-24	ARHGEF12	1.06E-01	1.00E+00	XPO7	-7.22E-02	1.00E+00
PPP2R5E	9.57E-01	2.87E-24	ARHGEF3	-8.88E-02	1.00E+00	LOC102410583	8.20E-02	1.00E+00
PROKR1	-6.09E+00	2.88E-24	CADPS2	5.53E-02	1.00E+00	MARCH6	-7.98E-02	1.00E+00
GNRHR	-5.96E+00	2.95E-24	UPF3A	-7.71E-02	1.00E+00	PTEN	6.15E-02	1.00E+00
SERINC1	-1.06E+00	2.95E-24	RSPO3	2.69E-01	1.00E+00	PHTF2	-9.22E-02	1.00E+00
PPP6R3	-1.03E+00	2.99E-24	RABEP1	-6.69E-02	1.00E+00	CLEC2B	-1.35E-01	1.00E+00
MLYCD	-2.16E+00	3.00E-24	LOC112578778	-4.95E-01	1.00E+00	MTFP1	1.09E-01	1.00E+00
CYR61	-5.70E+00	3.00E-24	LOC102415348	-1.12E-01	1.00E+00	IMMP1L	-7.70E-02	1.00E+00
LOC102409478	-2.40E+00	3.02E-24	ANKRD13D	-1.70E-01	1.00E+00	EIF4E3	8.87E-02	1.00E+00
PLEKHG5	-2.25E+00	3.02E-24	ZC3H8	-7.76E-02	1.00E+00	PRMT5	-6.61E-02	1.00E+00
LOC102398768	-4.97E+00	3.05E-24	HERPUD2	9.91E-02	1.00E+00	DPAGT1	-6.95E-02	1.00E+00
SLC38A1	-4.59E+00	3.06E-24	SAE1	5.72E-02	1.00E+00	LOC112583668	-1.50E-01	1.00E+00
SLC35C1	1.36E+00	3.09E-24	IQCN	-3.79E-01	1.00E+00	FAM131A	-8.30E-02	1.00E+00
LOC102404191	-2.19E+00	3.13E-24	THAP5	-9.94E-02	1.00E+00	PIGK	-6.64E-02	1.00E+00
PGPEP1	-2.80E+00	3.14E-24	CD3D	2.91E-01	1.00E+00	METTL24	-1.16E-01	1.00E+00
TOMM40L	1.14E+00	3.16E-24	HIC2	6.20E-02	1.00E+00	CCDC59	-8.05E-02	1.00E+00
SYNGR3	2.75E+00	3.21E-24	THUMPD3	-1.05E-01	1.00E+00	FZD7	9.37E-02	1.00E+00
ARV1	-	3.21E-24	KARS	-7.04E-	1.00E+00	ADGRA3	-8.45E-	1.00E+00

	2.42E+00			02			02	
THBS2	-8.24E+00	3.27E-24	DGCR6L	-8.59E-02	1.00E+00	MTFR1L	-6.46E-02	1.00E+00
TTC14	-1.43E+00	3.28E-24	RBM41	-1.17E-01	1.00E+00	USP48	-6.15E-02	1.00E+00
DOCK4	1.64E+00	3.29E-24	LOC112587597	3.83E-01	1.00E+00	SELENOF	-6.61E-02	1.00E+00
DSTN	1.50E+00	3.31E-24	GJA1	1.22E-01	1.00E+00	S100PBP	-6.58E-02	1.00E+00
CLTB	-1.46E+00	3.35E-24	TOMM34	6.62E-02	1.00E+00	ZNF569	-7.24E-02	1.00E+00
ADGRG6	-3.29E+00	3.35E-24	CCDC22	-5.94E-02	1.00E+00	GOLT1B	7.16E-02	1.00E+00
BORCS6	-2.04E+00	3.36E-24	MVB12A	1.63E-01	1.00E+00	EDF1	9.61E-02	1.00E+00
ZNF331	-1.92E+00	3.36E-24	LMBRD2	1.19E-01	1.00E+00	CMTM4	-7.75E-02	1.00E+00
SF3B4	9.97E-01	3.40E-24	FIS1	-1.02E-01	1.00E+00	ATG4D	-7.92E-02	1.00E+00
CCDC90B	-1.22E+00	3.44E-24	STAMBP	8.05E-02	1.00E+00	IDH2	-7.08E-02	1.00E+00
HOXD3	2.21E+00	3.54E-24	RASGRF2	-2.23E-01	1.00E+00	TMEM150A	9.87E-02	1.00E+00
PA2G4	-1.45E+00	3.54E-24	DNAH1	-1.84E-01	1.00E+00	DNAJC8	-6.87E-02	1.00E+00
TRUB1	-1.57E+00	3.57E-24	MGST2	9.73E-02	1.00E+00	FGL1	-1.48E-01	1.00E+00
PRSS55	-4.46E+00	3.58E-24	RSBN1L	-1.16E-01	1.00E+00	UBE2G1	6.41E-02	1.00E+00
DDHD1	1.14E+00	3.64E-24	N4BP3	-3.78E-01	1.00E+00	USE1	-9.82E-02	1.00E+00
NKIRAS2	-1.14E+00	3.74E-24	YWHAZ	6.53E-02	1.00E+00	EIF4EBP3	1.05E-01	1.00E+00
TNFAIP1	-1.21E+00	3.76E-24	DHX58	-5.66E-02	1.00E+00	CHURC1	6.81E-02	1.00E+00
CHRM5	-3.30E+00	3.82E-24	CCNO	-7.73E-02	1.00E+00	MAPKAPK5	-1.55E-01	1.00E+00
TMEM263	-1.53E+00	3.84E-24	FAM229B	-9.12E-02	1.00E+00	CISD2	6.98E-02	1.00E+00
ENOX2	-1.40E+00	3.85E-24	GXYLT2	3.78E-01	1.00E+00	NOSIP	9.31E-02	1.00E+00
IQCK	-2.07E+00	3.85E-24	MCM4	6.26E-02	1.00E+00	TNPO1	-7.46E-02	1.00E+00
LOC112584636	5.86E+00	3.91E-24	EVI5L	-7.05E-02	1.00E+00	DTX2	7.06E-02	1.00E+00
LOC102389244	-2.03E+00	3.93E-24	PTPA	6.02E-02	1.00E+00	ASPSCR1	-9.10E-02	1.00E+00
FAM124A	1.87E+00	3.95E-24	HIRIP3	8.11E-02	1.00E+00	GABPA	-7.36E-02	1.00E+00
LOC102403238	3.61E+00	3.98E-24	C3H9orf43	-2.41E-01	1.00E+00	DND1	1.04E-01	1.00E+00
PDS5B	-1.19E+00	4.04E-24	SLC25A44	5.76E-02	1.00E+00	WNK1	-7.50E-02	1.00E+00
UTP14A	-1.06E+00	4.07E-24	LOC102393517	-8.10E-02	1.00E+00	BMP8A	1.64E-01	1.00E+00
SUCLA2	9.97E-01	4.08E-24	SAP130	8.68E-02	1.00E+00	EXOC6B	-6.05E-02	1.00E+00
ARHGAP45	-4.14E+00	4.11E-24	ARL9	-1.92E-01	1.00E+00	LOC102410118	-9.04E-02	1.00E+00
LOC112578203	5.86E+00	4.17E-24	LRRC71	-1.36E-01	1.00E+00	ARHGAP35	-7.24E-02	1.00E+00
FAM104A	1.64E+00	4.24E-24	MFN1	6.57E-02	1.00E+00	ICE1	-7.36E-02	1.00E+00
POSTN	-	4.24E-24	GINS4	-6.20E-	1.00E+00	COASY	-8.68E-	1.00E+00

	6.89E+00			02			02	
NCF2	4.41E+00	4.24E-24	SENP1	-1.23E-01	1.00E+00	CEP78	-7.19E-02	1.00E+00
PHPT1	-2.12E+00	4.26E-24	CBX2	8.56E-02	1.00E+00	SLC4A1AP	-6.18E-02	1.00E+00
TNFRSF11A	3.08E+00	4.35E-24	TMCO4	-1.14E-01	1.00E+00	NSD1	-7.67E-02	1.00E+00
SNUPN	1.26E+00	4.41E-24	TES	-7.75E-02	1.00E+00	CEP72	-9.12E-02	1.00E+00
STON2	3.14E+00	4.41E-24	LOC102411494	-2.29E-01	1.00E+00	RNF126	7.20E-02	1.00E+00
CALML4	-2.66E+00	4.47E-24	SWAP70	-7.76E-02	1.00E+00	AGTPBP1	1.04E-01	1.00E+00
TSPAN17	-1.24E+00	4.50E-24	LIAS	7.39E-02	1.00E+00	FANCC	6.95E-02	1.00E+00
NMT2	-1.31E+00	4.53E-24	OBSL1	-1.07E-01	1.00E+00	MOB3B	1.43E-01	1.00E+00
SPATA18	-4.72E+00	4.53E-24	ZDHHC16	-1.11E-01	1.00E+00	SNX27	7.35E-02	1.00E+00
RPS21	-2.03E+00	4.58E-24	TULP3	5.85E-02	1.00E+00	LMF1	-7.02E-02	1.00E+00
DHRS1	-1.30E+00	4.66E-24	CACNG7	8.50E-02	1.00E+00	NOM1	6.33E-02	1.00E+00
CEP128	1.20E+00	4.72E-24	DDX59	6.39E-02	1.00E+00	EEF1A2	-1.52E-01	1.00E+00
TTC16	2.44E+00	4.72E-24	TMEM41B	-1.10E-01	1.00E+00	USP28	-7.21E-02	1.00E+00
ATG4B	-1.14E+00	4.80E-24	ZPR1	7.47E-02	1.00E+00	MTG1	-8.81E-02	1.00E+00
C20H15orf40	-1.64E+00	4.84E-24	PPM1E	9.53E-02	1.00E+00	COX2	-1.65E-01	1.00E+00
PYROXD1	-1.52E+00	4.87E-24	EIF2S3	-6.40E-02	1.00E+00	ORC3	-7.37E-02	1.00E+00
LOC102411578	2.94E+00	4.89E-24	LGALS4	-7.97E-02	1.00E+00	BAZ2A	-9.03E-02	1.00E+00
GP5	4.55E+00	4.92E-24	XRCC4	8.59E-02	1.00E+00	AGAP1	-7.39E-02	1.00E+00
DNMT3B	1.70E+00	4.93E-24	ZRANB3	5.84E-02	1.00E+00	MAP3K3	-6.78E-02	1.00E+00
DENND5B	-1.08E+00	5.00E-24	PA2G4	-5.59E-02	1.00E+00	SELPLG	3.01E-01	1.00E+00
DBX2	4.86E+00	5.07E-24	FST	4.37E-01	1.00E+00	RAD9A	-7.66E-02	1.00E+00
CDH4	4.45E+00	5.07E-24	SNED1	1.57E-01	1.00E+00	HMG20B	7.20E-02	1.00E+00
MORC1	5.98E+00	5.09E-24	TTC39A	-7.43E-02	1.00E+00	COQ8B	7.40E-02	1.00E+00
EIF3I	-1.43E+00	5.14E-24	BUB1B	7.08E-02	1.00E+00	RAB9B	1.44E-01	1.00E+00
PRIM2	1.11E+00	5.18E-24	SEMA4F	-9.54E-02	1.00E+00	CCDC57	8.36E-02	1.00E+00
OCIAD2	-6.29E+00	5.36E-24	ECT2L	-2.35E-01	1.00E+00	TUBD1	1.28E-01	1.00E+00
GNG11	-5.98E+00	5.44E-24	LOC102396260	1.20E-01	1.00E+00	CD44	-2.97E-01	1.00E+00
STXBP3	1.13E+00	5.61E-24	SLC16A8	-3.99E-01	1.00E+00	KCTD6	-8.48E-02	1.00E+00
LOC112578768	-3.81E+00	5.62E-24	ARMH1	-2.53E-01	1.00E+00	GALNT9	1.10E-01	1.00E+00
SNRPA1	1.14E+00	5.80E-24	NVL	-5.55E-02	1.00E+00	LOC102396606	1.45E-01	1.00E+00
LOC102400830	-1.30E+00	5.84E-24	ANKRD34A	-1.69E-01	1.00E+00	MRPL34	1.03E-01	1.00E+00
UBE2H	1.06E+00	5.92E-24	TFG	5.74E-02	1.00E+00	SLC12A2	-1.14E-01	1.00E+00

CCDC105	-4.84E+00	6.18E-24	MYBL2	5.98E-02	1.00E+00	HTR6	1.21E-01	1.00E+00
VANGL1	1.39E+00	6.19E-24	TMEM125	2.44E-01	1.00E+00	NRF1	-7.11E-02	1.00E+00
HAGH	1.44E+00	6.21E-24	LONP2	-7.60E-02	1.00E+00	RNF44	9.77E-02	1.00E+00
PRIM1	-1.44E+00	6.25E-24	LOC112581262	-3.20E-01	1.00E+00	SMC1A	-6.30E-02	1.00E+00
NFE2L3	-1.43E+00	6.27E-24	MNT	8.18E-02	1.00E+00	CPSF2	-6.13E-02	1.00E+00
CATSPERD	1.25E+00	6.38E-24	GBE1	1.36E-01	1.00E+00	TCF3	6.83E-02	1.00E+00
MYO6	-1.27E+00	6.42E-24	BCAS2	-8.13E-02	1.00E+00	DIP2C	6.37E-02	1.00E+00
LOC102404070	-4.42E+00	6.43E-24	SEC24B	8.06E-02	1.00E+00	TXNL1	-6.73E-02	1.00E+00
NINJ2	3.81E+00	6.53E-24	NRBF2	-1.49E-01	1.00E+00	UBA5	-7.79E-02	1.00E+00
PIF1	-3.83E+00	6.55E-24	RC3H2	1.41E-01	1.00E+00	MYSM1	-1.02E-01	1.00E+00
INSIG1	-2.12E+00	6.71E-24	NUP43	-9.31E-02	1.00E+00	MTA2	6.37E-02	1.00E+00
RBM5	-1.04E+00	6.77E-24	TLE6	7.79E-02	1.00E+00	SGF29	-1.01E-01	1.00E+00
POU2F2	-3.08E+00	6.81E-24	ITPR2	-1.68E-01	1.00E+00	PXN	-7.11E-02	1.00E+00
LOC102407094	-6.17E+00	7.03E-24	AZIN1	7.36E-02	1.00E+00	CRNKL1	6.21E-02	1.00E+00
ACOX2	-2.17E+00	7.07E-24	USP46	6.84E-02	1.00E+00	KCNQ3	-1.54E-01	1.00E+00
LOC102409132	-1.64E+00	7.15E-24	RALY	-5.97E-02	1.00E+00	TMEM222	-6.80E-02	1.00E+00
ASB14	-4.04E+00	7.21E-24	HDAC5	1.15E-01	1.00E+00	USP22	-7.50E-02	1.00E+00
FBLIM1	1.71E+00	7.22E-24	COL9A3	-3.00E-01	1.00E+00	ANKRD33B	-1.33E-01	1.00E+00
NOS3	-4.69E+00	7.41E-24	CDK14	-8.91E-02	1.00E+00	OSGIN2	-8.29E-02	1.00E+00
LOC102397090	-2.72E+00	7.59E-24	LOC112583974	-1.48E-01	1.00E+00	NPHP1	-8.08E-02	1.00E+00
GAB3	-3.73E+00	7.72E-24	LOC102393546	-2.53E-01	1.00E+00	AIMP2	7.92E-02	1.00E+00
ARHGEF3	1.64E+00	7.72E-24	NT5DC2	-7.98E-02	1.00E+00	TBL2	-6.53E-02	1.00E+00
LGI3	-3.97E+00	7.73E-24	TAPBPL	-2.43E-01	1.00E+00	CSTF3	-6.23E-02	1.00E+00
CABYR	-2.09E+00	7.75E-24	SETD5	1.32E-01	1.00E+00	TIAF1	1.37E-01	1.00E+00
MKKS	-1.23E+00	7.78E-24	LPCAT1	7.27E-02	1.00E+00	EIF4EBP1	-9.48E-02	1.00E+00
PRSS22	6.35E+00	7.91E-24	KCTD5	5.94E-02	1.00E+00	TACSTD2	-1.69E-01	1.00E+00
SLC3A2	1.27E+00	7.91E-24	IRF7	-8.25E-02	1.00E+00	FBXO36	1.15E-01	1.00E+00
MZB1	3.42E+00	7.95E-24	SLC22A20P	2.64E-01	1.00E+00	NDRG4	-1.28E-01	1.00E+00
MANEA	1.07E+00	8.01E-24	CCDC150	-1.88E-01	1.00E+00	CCDC32	7.90E-02	1.00E+00
PHF11	-8.81E-01	8.19E-24	LOC112586855	-3.75E-01	1.00E+00	MTFMT	-7.73E-02	1.00E+00
LOC102401659	-2.62E+00	8.41E-24	MTMR14	-5.28E-02	1.00E+00	DCUN1D5	-6.58E-02	1.00E+00
SUN3	4.19E+00	8.46E-24	GSDMC	-1.52E-01	1.00E+00	ZNF140	8.19E-02	1.00E+00
CWC27	-	8.47E-24	ITGA10	-1.34E-	1.00E+00	PLOD2	7.65E-02	1.00E+00

	1.78E+00			01				
EEF2K	1.04E+00	8.52E-24	BIRC6	1.44E-01	1.00E+00	LOC102412874	8.14E-02	1.00E+00
SYNGR1	-1.29E+00	8.53E-24	AKAP8	-8.66E-02	1.00E+00	LOC102401181	-7.74E-02	1.00E+00
LOC112581846	2.51E+00	8.69E-24	CRLS1	-8.65E-02	1.00E+00	SUSD6	6.97E-02	1.00E+00
GMFB	-1.36E+00	8.70E-24	LOC102399398	-8.60E-02	1.00E+00	ZNF449	9.76E-02	1.00E+00
DONSON	9.78E-01	8.90E-24	TMEM65	-1.50E-01	1.00E+00	CCDC151	9.13E-02	1.00E+00
TAF12	1.25E+00	8.99E-24	LOC102401181	-7.90E-02	1.00E+00	ATP5PD	9.19E-02	1.00E+00
TJP3	-3.67E+00	9.01E-24	SNRNP70	-7.86E-02	1.00E+00	LOC102410412	-1.55E-01	1.00E+00
ITGA6	-1.41E+00	9.08E-24	TPCN2	-1.51E-01	1.00E+00	RAB32	-8.32E-02	1.00E+00
LOC102404595	2.64E+00	9.14E-24	PPIB	-6.76E-02	1.00E+00	LOC102407546	8.27E-02	1.00E+00
KRAS	-1.92E+00	9.15E-24	CLTRN	-2.26E-01	1.00E+00	SART3	-6.18E-02	1.00E+00
LOC112579693	5.00E+00	9.23E-24	LGR4	1.25E-01	1.00E+00	FIGNL1	-8.28E-02	1.00E+00
RPS6KB1	9.11E-01	9.27E-24	SLC25A33	-8.11E-02	1.00E+00	HERC1	7.97E-02	1.00E+00
LOC102395095	-1.77E+00	9.36E-24	IER5L	9.84E-02	1.00E+00	EFCAB8	-1.19E-01	1.00E+00
SKIL	-4.66E+00	9.43E-24	LOC102413646	4.21E-01	1.00E+00	MPC1	-7.43E-02	1.00E+00
LOC102402274	6.06E+00	9.52E-24	ELOA	1.54E-01	1.00E+00	TERB1	-1.62E-01	1.00E+00
ERP27	-6.47E+00	1.01E-23	SNX6	-8.82E-02	1.00E+00	EIF2AK1	-5.93E-02	1.00E+00
SLC30A1	-1.38E+00	1.01E-23	OTOGL	1.62E-01	1.00E+00	TRAPPC3	8.18E-02	1.00E+00
ITFG1	-1.07E+00	1.03E-23	LOC102401017	-6.34E-02	1.00E+00	SPEF1	-1.21E-01	1.00E+00
OAZ1	-1.46E+00	1.04E-23	SLCO2A1	-2.49E-01	1.00E+00	SRC	-6.29E-02	1.00E+00
LOC112587382	-3.16E+00	1.04E-23	LOC102392256	2.97E-01	1.00E+00	DONSON	-6.76E-02	1.00E+00
CCSER2	1.44E+00	1.05E-23	LOC102415752	1.19E-01	1.00E+00	ADO	6.60E-02	1.00E+00
CACNA1C	2.39E+00	1.05E-23	GOT1	7.14E-02	1.00E+00	APH1A	-6.16E-02	1.00E+00
EIF2AK4	1.09E+00	1.06E-23	POP1	-7.46E-02	1.00E+00	PDHA1	-1.06E-01	1.00E+00
SPATC1L	-3.78E+00	1.08E-23	TAOK2	9.34E-02	1.00E+00	LOC102392771	1.29E-01	1.00E+00
RAI14	1.34E+00	1.11E-23	HIKESHI	9.77E-02	1.00E+00	TANK	6.78E-02	1.00E+00
PRDX1	1.68E+00	1.12E-23	LOC102410932	-2.15E-01	1.00E+00	LOC102402730	1.50E-01	1.00E+00
NDUFB11	-1.54E+00	1.15E-23	SNX24	-7.57E-02	1.00E+00	GGPS1	-7.09E-02	1.00E+00
NOP58	-1.11E+00	1.17E-23	SLTM	1.00E-01	1.00E+00	ARMC3	1.74E-01	1.00E+00
SLC10A1	-3.97E+00	1.21E-23	PLEC	1.51E-01	1.00E+00	LOC102401224	-1.28E-01	1.00E+00
RCOR3	-1.29E+00	1.22E-23	DCBLD1	7.57E-02	1.00E+00	POLE4	8.35E-02	1.00E+00
EPHA2	-1.18E+00	1.24E-23	ABHD18	2.00E-01	1.00E+00	SPOPL	-1.47E-01	1.00E+00
TRIP13	1.20E+00	1.26E-23	CRELD2	7.82E-02	1.00E+00	STXBP1	8.12E-02	1.00E+00
C2CD3	-1.39E+00	1.29E-23	SLITRK6	9.57E-02	1.00E+00	OARD1	-8.24E-02	1.00E+00

RSPH3	-1.69E+00	1.32E-23	PLPPR2	1.08E-01	1.00E+00	RAF1	6.05E-02	1.00E+00
ELFN2	4.09E+00	1.34E-23	DUSP8	1.33E-01	1.00E+00	WASF1	-7.44E-02	1.00E+00
LOC102410383	5.32E+00	1.36E-23	PIH1D2	-8.94E-02	1.00E+00	LOC102416276	-1.24E-01	1.00E+00
FAM207A	-2.00E+00	1.36E-23	MLN	-7.89E-02	1.00E+00	NFAT5	-9.68E-02	1.00E+00
PDE1B	2.45E+00	1.37E-23	HPX	-2.17E-01	1.00E+00	CYTH2	5.79E-02	1.00E+00
POLK	-3.36E+00	1.37E-23	HPS1	-1.18E-01	1.00E+00	CARNMT1	8.64E-02	1.00E+00
AIMP1	-1.50E+00	1.41E-23	ATXN7L3	3.92E-01	1.00E+00	LOC102413321	1.57E-01	1.00E+00
PAG1	3.39E+00	1.44E-23	WWC1	-8.16E-02	1.00E+00	SECISBP2	8.97E-02	1.00E+00
ABCD2	5.86E+00	1.47E-23	IRF2	-3.37E-01	1.00E+00	SOWAHB	1.07E-01	1.00E+00
LOC102410373	1.63E+00	1.47E-23	LAPTM4B	-9.14E-02	1.00E+00	GPR107	-6.53E-02	1.00E+00
TIMELESS	1.19E+00	1.48E-23	CARD11	-1.80E-01	1.00E+00	PIK3CB	8.08E-02	1.00E+00
ELP1	9.87E-01	1.49E-23	ABHD3	-2.21E-01	1.00E+00	PBX1	7.21E-02	1.00E+00
CCNJL	-2.93E+00	1.53E-23	PMPCA	5.52E-02	1.00E+00	MAN1A2	-8.04E-02	1.00E+00
ATP6V0B	-1.11E+00	1.54E-23	FOXE1	3.32E-01	1.00E+00	CCR1	2.98E-01	1.00E+00
LOC102390176	1.39E+00	1.56E-23	DLX5	-3.15E-01	1.00E+00	GNPAT	-5.77E-02	1.00E+00
DNALI1	2.19E+00	1.56E-23	SNRPD2	-9.21E-02	1.00E+00	LOC102405552	-1.58E-01	1.00E+00
ITGB8	-5.27E+00	1.61E-23	EGLN3	-6.37E-02	1.00E+00	CEP57L1	1.05E-01	1.00E+00
LOC102410132	1.99E+00	1.64E-23	C6H1orf109	-8.06E-02	1.00E+00	PSMB3	1.00E-01	1.00E+00
CDON	-1.75E+00	1.70E-23	RAB3IL1	-1.68E-01	1.00E+00	PTCHD4	1.29E-01	1.00E+00
LOC112585335	-5.68E+00	1.71E-23	CERS2	-5.66E-02	1.00E+00	FGF12	6.04E-02	1.00E+00
LOC102400922	3.81E+00	1.72E-23	AKNAD1	-1.02E-01	1.00E+00	LOC102411603	-1.36E-01	1.00E+00
OCEL1	1.34E+00	1.73E-23	DRC7	7.57E-02	1.00E+00	ITPA	-8.41E-02	1.00E+00
ABCC3	-1.93E+00	1.74E-23	SIRT6	1.18E-01	1.00E+00	SEC61A2	-6.31E-02	1.00E+00
CLPS	7.86E+00	1.75E-23	ITPRIPL2	-2.88E-01	1.00E+00	CSRNP2	9.44E-02	1.00E+00
RDH13	-1.88E+00	1.75E-23	EFCAB14	-6.26E-02	1.00E+00	LOC102398488	-1.03E-01	1.00E+00
LOC112585314	-5.70E+00	1.78E-23	ARHGAP19	8.97E-02	1.00E+00	CNEP1R1	-7.89E-02	1.00E+00
NDFIP1	9.34E-01	1.78E-23	LOC102395384	6.36E-02	1.00E+00	CHCHD3	6.26E-02	1.00E+00
LOC112586805	2.81E+00	1.80E-23	CCDC36	-1.24E-01	1.00E+00	CCT5	-8.16E-02	1.00E+00
TMEM205	-1.93E+00	1.81E-23	E2F6	-6.33E-02	1.00E+00	AATK	-1.49E-01	1.00E+00
RAB28	8.80E-01	1.84E-23	KCNA4	3.75E-01	1.00E+00	LOC102389244	1.02E-01	1.00E+00
FSCN3	4.19E+00	1.84E-23	LOC112587813	3.27E-01	1.00E+00	ATM	9.37E-02	1.00E+00
CASP8	-4.53E+00	1.85E-23	MRPL34	-8.46E-02	1.00E+00	C5H1orf53	-1.23E-01	1.00E+00
ITPKA	-1.88E+00	1.90E-23	STX16	1.53E-01	1.00E+00	LOC102414455	8.12E-02	1.00E+00

ATP5PF	-1.50E+00	1.98E-23	LOC102408538	3.58E-01	1.00E+00	FAM120B	-6.75E-02	1.00E+00
CCNG2	-1.39E+00	2.01E-23	METTL6	-7.22E-02	1.00E+00	LOC102402606	6.19E-02	1.00E+00
LOC102409439	6.66E+00	2.05E-23	LOC102399270	2.27E-01	1.00E+00	ATRAID	-7.71E-02	1.00E+00
CD101	-3.67E+00	2.07E-23	SNCAIP	1.31E-01	1.00E+00	NDUFB4	8.27E-02	1.00E+00
LOC102401107	-3.42E+00	2.10E-23	SGTA	7.37E-02	1.00E+00	VEGFB	-8.20E-02	1.00E+00
LOC102393261	1.50E+00	2.12E-23	GALC	1.60E-01	1.00E+00	LOC102411217	1.59E-01	1.00E+00
CDKN3	-2.24E+00	2.16E-23	ZFYVE27	7.56E-02	1.00E+00	CTTNBP2	-1.13E-01	1.00E+00
PIGA	-1.82E+00	2.20E-23	IRS2	1.95E-01	1.00E+00	CBX8	-1.00E-01	1.00E+00
MYLIP	1.26E+00	2.23E-23	GRAP2	-1.95E-01	1.00E+00	WDR81	-6.68E-02	1.00E+00
CELSR2	1.38E+00	2.28E-23	KAZALD1	1.60E-01	1.00E+00	KIAA1549	-6.20E-02	1.00E+00
YIPF1	-1.52E+00	2.29E-23	PPFIA2	-7.78E-02	1.00E+00	SPTBN5	-1.45E-01	1.00E+00
FBXO9	1.06E+00	2.31E-23	MPND	-7.12E-02	1.00E+00	SARM1	9.89E-02	1.00E+00
ARID5A	2.87E+00	2.32E-23	NUP160	9.01E-02	1.00E+00	CEP112	-6.54E-02	1.00E+00
MACO1	1.13E+00	2.32E-23	NFE2	2.87E-01	1.00E+00	LOC102409686	9.41E-02	1.00E+00
SUSD2	3.90E+00	2.38E-23	UBE2L6	-1.31E-01	1.00E+00	LHFPL2	1.79E-01	1.00E+00
GDNF	2.92E+00	2.39E-23	HS6ST2	-2.53E-01	1.00E+00	RBM42	7.62E-02	1.00E+00
SH2D4A	-4.74E+00	2.40E-23	LOC102399600	-3.08E-01	1.00E+00	LOC102395796	8.98E-02	1.00E+00
WDR35	-1.37E+00	2.47E-23	UBE2V1	-5.79E-02	1.00E+00	LOC102392363	-1.64E-01	1.00E+00
LOC112582250	6.06E+00	2.59E-23	FAM186B	1.60E-01	1.00E+00	LOC112583654	1.53E-01	1.00E+00
RINT1	-1.38E+00	2.66E-23	CMC1	-5.85E-02	1.00E+00	GPBP1	-5.92E-02	1.00E+00
DCDC2B	-4.20E+00	2.67E-23	RHEBL1	-7.08E-02	1.00E+00	NRTN	-1.26E-01	1.00E+00
TNFSF11	5.03E+00	2.71E-23	DPH5	-8.58E-02	1.00E+00	DCPS	-8.63E-02	1.00E+00
KIAA0355	1.28E+00	2.73E-23	FGF16	-8.86E-02	1.00E+00	CINP	8.13E-02	1.00E+00
LYSMD4	1.34E+00	2.73E-23	DNM2	6.19E-02	1.00E+00	SIMC1	-6.91E-02	1.00E+00
IVD	-1.41E+00	2.77E-23	EFHC2	1.43E-01	1.00E+00	LOC112577668	1.38E-01	1.00E+00
FBXO41	3.25E+00	2.86E-23	WDR61	-6.85E-02	1.00E+00	RAB5C	6.07E-02	1.00E+00
SNX15	1.40E+00	2.91E-23	TBCD	9.79E-02	1.00E+00	FERMT3	-2.22E-01	1.00E+00
PLSCR3	-1.13E+00	2.92E-23	SLC39A7	8.89E-02	1.00E+00	PURB	6.20E-02	1.00E+00
ART1	5.02E+00	2.94E-23	TRAF3IP1	8.92E-02	1.00E+00	ZNF746	6.64E-02	1.00E+00
DMPK	-2.12E+00	2.98E-23	LOC112583836	8.31E-02	1.00E+00	KLHDC4	-8.46E-02	1.00E+00
ATF6	8.75E-01	3.01E-23	PYCR1	-3.32E-01	1.00E+00	LOC102408340	1.05E-01	1.00E+00
LOC112580820	3.53E+00	3.13E-23	ZNF391	-2.97E-01	1.00E+00	SNAPC4	6.81E-02	1.00E+00
REPS1	8.62E-01	3.18E-23	DENND4C	-8.90E-02	1.00E+00	NONO	-7.36E-02	1.00E+00
RCSD1	-	3.20E-23	GKAP1	-5.97E-	1.00E+00	VRK2	6.50E-02	1.00E+00

	3.10E+00				02			
CLMN	-3.42E+00	3.27E-23	ADAMTS13	1.65E-01	1.00E+00	LOC102399099	1.54E-01	1.00E+00
ATPAF1	-1.10E+00	3.41E-23	SDHD	-7.34E-02	1.00E+00	RABIF	6.96E-02	1.00E+00
TMEM161B	-1.39E+00	3.42E-23	KRTCAP3	-8.90E-02	1.00E+00	GGA1	-6.35E-02	1.00E+00
HDAC9	3.40E+00	3.43E-23	DDRGK1	-7.54E-02	1.00E+00	HRCT1	1.71E-01	1.00E+00
WDR17	1.75E+00	3.48E-23	GALK2	-7.35E-02	1.00E+00	CLIP2	6.68E-02	1.00E+00
PXMP4	1.70E+00	3.62E-23	TBC1D16	-1.52E-01	1.00E+00	HEATR5A	1.12E-01	1.00E+00
LOC102409828	1.78E+00	3.64E-23	MEFV	1.02E-01	1.00E+00	AMFR	-6.62E-02	1.00E+00
USP1	1.04E+00	3.67E-23	STRN4	9.48E-02	1.00E+00	IMPACT	-8.31E-02	1.00E+00
CEP126	-2.78E+00	3.75E-23	DISP1	-9.37E-02	1.00E+00	AP3D1	-6.45E-02	1.00E+00
LOC112583735	-4.61E+00	3.79E-23	MNX1	1.19E-01	1.00E+00	SARDH	-1.93E-01	1.00E+00
FARP1	9.47E-01	3.83E-23	HIBCH	-1.02E-01	1.00E+00	SNRNP70	7.68E-02	1.00E+00
NADSYN1	-5.67E+00	3.89E-23	PPP4R3B	-8.10E-02	1.00E+00	ORMDL1	1.72E-01	1.00E+00
LOC112577753	-2.92E+00	3.93E-23	AIFM1	-8.97E-02	1.00E+00	NCR3LG1	-8.88E-02	1.00E+00
PLCXD2	-4.96E+00	3.93E-23	MYOZ3	-6.85E-02	1.00E+00	B9D1	-8.69E-02	1.00E+00
PPP1R3C	2.13E+00	3.99E-23	USP22	6.92E-02	1.00E+00	ATP4B	1.39E-01	1.00E+00
LOC112586999	-4.00E+00	4.14E-23	ZYG11B	-1.16E-01	1.00E+00	MSC	2.23E-01	1.00E+00
KAZN	2.43E+00	4.14E-23	RAB9A	5.66E-02	1.00E+00	PAXX	-1.01E-01	1.00E+00
ATP5S	-2.72E+00	4.32E-23	DUSP18	1.91E-01	1.00E+00	SENP6	-6.79E-02	1.00E+00
CCDC68	-6.23E+00	4.35E-23	PLEKHD1	-1.64E-01	1.00E+00	PRKACA	-6.39E-02	1.00E+00
GREB1	-5.35E+00	4.39E-23	HSF4	-2.67E-01	1.00E+00	TMA16	-8.26E-02	1.00E+00
MTNR1A	4.22E+00	4.40E-23	MPHOSPH9	6.49E-02	1.00E+00	RING1	7.06E-02	1.00E+00
SLC6A11	5.78E+00	4.41E-23	IRF6	6.29E-02	1.00E+00	LOC112587311	1.27E-01	1.00E+00
CKAP5	1.35E+00	4.41E-23	ATG4C	-1.07E-01	1.00E+00	HACD1	1.08E-01	1.00E+00
ARID4A	-2.44E+00	4.52E-23	LOC102407968	-1.36E-01	1.00E+00	ZFP69B	-1.46E-01	1.00E+00
DCAF13	9.29E-01	4.53E-23	PFDN4	-7.97E-02	1.00E+00	RPIA	-7.46E-02	1.00E+00
ATP6V0E2	-2.51E+00	4.55E-23	LOC102400714	-9.62E-02	1.00E+00	GCSH	-6.67E-02	1.00E+00
CLEC12B	-5.68E+00	4.56E-23	MST1R	-1.10E-01	1.00E+00	CFAP46	-8.64E-02	1.00E+00
LOC112586431	6.71E+00	4.57E-23	LOC112578060	1.29E-01	1.00E+00	POLD2	-9.11E-02	1.00E+00
PCBP4	-1.43E+00	4.60E-23	PAX3	7.66E-02	1.00E+00	NOP53	8.48E-02	1.00E+00
LOC102401116	-4.54E+00	4.64E-23	S100A13	-3.95E-01	1.00E+00	SKAP1	-9.61E-02	1.00E+00
EPC1	8.92E-01	4.65E-23	LOC102399577	-2.63E-01	1.00E+00	TBX6	1.32E-01	1.00E+00
PYGM	3.61E+00	4.67E-23	GNG5	-8.59E-02	1.00E+00	LOC112578124	1.29E-01	1.00E+00
LOC112581215	2.26E+00	4.78E-23	LOC112581566	1.58E-01	1.00E+00	SPTBN2	1.51E-01	1.00E+00

MED28	-1.57E+00	4.79E-23	DHRS13	-1.57E-01	1.00E+00	NKAP	7.66E-02	1.00E+00
LOC102392561	-4.48E+00	4.83E-23	LOC102414203	-1.95E-01	1.00E+00	SYNE3	-8.18E-02	1.00E+00
PIH1D2	1.38E+00	4.83E-23	MARVELD1	4.10E-01	1.00E+00	HAVCR2	-1.13E-01	1.00E+00
MAX	-1.19E+00	4.92E-23	SETD1B	1.11E-01	1.00E+00	TBC1D1	6.79E-02	1.00E+00
LOC112579106	2.85E+00	4.95E-23	MAML1	1.21E-01	1.00E+00	CHST7	1.76E-01	1.00E+00
GSX1	4.48E+00	4.97E-23	MMP28	-2.33E-01	1.00E+00	FAM185A	-7.58E-02	1.00E+00
BCL9L	-1.59E+00	4.99E-23	CHFR	5.89E-02	1.00E+00	QARS	6.67E-02	1.00E+00
HYPK	-1.61E+00	5.06E-23	RNF138	-1.59E-01	1.00E+00	SLC15A1	1.28E-01	1.00E+00
GATA4	-4.46E+00	5.10E-23	CEACAM19	-1.41E-01	1.00E+00	AARS2	-8.11E-02	1.00E+00
JOSD2	-1.97E+00	5.12E-23	TICAM1	-1.76E-01	1.00E+00	BTC	-7.76E-02	1.00E+00
ATP8A2	-1.77E+00	5.29E-23	RELA	7.27E-02	1.00E+00	LOC112579582	1.33E-01	1.00E+00
TOP2B	-1.18E+00	5.37E-23	SYP	-1.22E-01	1.00E+00	MMACHC	-8.95E-02	1.00E+00
FAIM2	3.21E+00	5.43E-23	MSX1	-7.64E-02	1.00E+00	MRPL55	9.30E-02	1.00E+00
TIAM1	3.55E+00	5.46E-23	C4H12orf29	1.24E-01	1.00E+00	MBD6	-6.41E-02	1.00E+00
TLR8	5.38E+00	5.48E-23	FTL	-7.85E-02	1.00E+00	CLCN3	-5.86E-02	1.00E+00
LOC112581444	2.28E+00	5.54E-23	LOC102398760	-2.79E-01	1.00E+00	TDP2	6.91E-02	1.00E+00
FNIP1	-1.45E+00	5.63E-23	SLITRK2	3.97E-01	1.00E+00	RAMAC	-7.20E-02	1.00E+00
LOC112587471	3.54E+00	5.81E-23	WDR5B	2.09E-01	1.00E+00	C16H11orf58	5.88E-02	1.00E+00
LOC112579930	2.41E+00	5.82E-23	SHISA2	-2.21E-01	1.00E+00	NBEAL1	-1.11E-01	1.00E+00
C6H1orf210	-5.01E+00	5.84E-23	PIN1	-9.90E-02	1.00E+00	SPATA7	-6.47E-02	1.00E+00
CNOT3	1.12E+00	6.21E-23	ITM2B	-1.12E-01	1.00E+00	LMNB2	-7.75E-02	1.00E+00
LOC112587870	4.08E+00	6.23E-23	ANKRD27	7.04E-02	1.00E+00	PPDPF	8.39E-02	1.00E+00
VPS13D	-1.41E+00	6.26E-23	CNIH2	-2.85E-01	1.00E+00	DPH1	-6.95E-02	1.00E+00
FLRT2	-3.26E+00	6.36E-23	SLC2A1	-2.30E-01	1.00E+00	LOC102399865	1.19E-01	1.00E+00
MX2	-5.73E+00	6.50E-23	MOB3B	-2.48E-01	1.00E+00	TBC1D14	5.73E-02	1.00E+00
APOO	1.19E+00	6.51E-23	PODNL1	4.33E-01	1.00E+00	ADIPOR1	-6.02E-02	1.00E+00
OPRD1	4.49E+00	6.63E-23	NCOA2	1.15E-01	1.00E+00	TMEM147	-8.55E-02	1.00E+00
ARFIP2	-1.24E+00	6.63E-23	NOTCH4	-2.13E-01	1.00E+00	ZNF445	-7.55E-02	1.00E+00
LOC112585772	4.07E+00	6.74E-23	WDFY2	-1.34E-01	1.00E+00	GTF2B	6.32E-02	1.00E+00
LOC102407618	-1.86E+00	6.77E-23	ATP1B1	5.63E-02	1.00E+00	LOC112586916	1.48E-01	1.00E+00
USP47	-8.97E-01	6.84E-23	LAGE3	-1.08E-01	1.00E+00	POLG2	-6.41E-02	1.00E+00
FLT3LG	-3.56E+00	6.85E-23	C12H2orf81	-4.15E-01	1.00E+00	LOC102399369	1.22E-01	1.00E+00
TNFAIP3	-2.16E+00	6.95E-23	ATP13A2	1.79E-01	1.00E+00	SMIM10L1	5.69E-02	1.00E+00

AUTS2	1.56E+00	6.99E-23	ANO4	-5.93E-02	1.00E+00	FKBP1B	1.28E-01	1.00E+00
PTCHD4	-4.74E+00	6.99E-23	LOC102407787	1.37E-01	1.00E+00	WDR89	8.25E-02	1.00E+00
PTPDC1	-3.21E+00	7.03E-23	SEBOX	1.76E-01	1.00E+00	DNAJC16	-7.60E-02	1.00E+00
DGKE	-1.74E+00	7.10E-23	TBC1D20	-7.32E-02	1.00E+00	CYFIP2	8.82E-02	1.00E+00
EFCAB7	-1.66E+00	7.20E-23	ACSBG1	3.84E-01	1.00E+00	YIF1A	-7.24E-02	1.00E+00
VAC14	-1.03E+00	7.22E-23	DYRK2	-1.61E-01	1.00E+00	GPD2	-9.68E-02	1.00E+00
MAPK9	-1.06E+00	7.26E-23	PEX7	7.92E-02	1.00E+00	PPIL1	-6.76E-02	1.00E+00
NDUFV1	-1.54E+00	7.36E-23	KIAA1109	-1.19E-01	1.00E+00	CCDC36	1.61E-01	1.00E+00
LOC112583715	6.69E+00	7.48E-23	NBEAL1	-1.83E-01	1.00E+00	PHKG2	-6.23E-02	1.00E+00
LOC102391628	6.33E+00	7.54E-23	CPE	-2.13E-01	1.00E+00	CCNB1IP1	-1.03E-01	1.00E+00
RNF11	-1.33E+00	7.60E-23	LOC102409738	-1.59E-01	1.00E+00	GNMT	1.21E-01	1.00E+00
ITGB6	5.35E+00	7.63E-23	TFE3	6.29E-02	1.00E+00	CDK2AP2	-8.48E-02	1.00E+00
ATP8B4	4.40E+00	7.77E-23	HOXD9	-9.37E-02	1.00E+00	YRDC	7.13E-02	1.00E+00
MDM1	1.38E+00	7.79E-23	TLR8	1.19E-01	1.00E+00	LOC102412561	1.57E-01	1.00E+00
LOC112583906	4.70E+00	7.83E-23	TRPV5	-2.98E-01	1.00E+00	RAPGEF6	-8.43E-02	1.00E+00
PHF8	-1.44E+00	7.92E-23	TBPL2	-1.02E-01	1.00E+00	PKNOX1	6.38E-02	1.00E+00
CEBPA	-3.54E+00	8.02E-23	CADPS	8.73E-02	1.00E+00	TMEM123	-7.98E-02	1.00E+00
CDK20	-9.46E-01	8.04E-23	LOC102397448	-2.61E-01	1.00E+00	MON1A	7.82E-02	1.00E+00
LOC102405097	-6.59E+00	8.06E-23	BAG5	-1.87E-01	1.00E+00	FAM117B	-7.41E-02	1.00E+00
CHPF2	-1.16E+00	8.20E-23	LOC102402713	-4.70E-01	1.00E+00	SDHAF4	8.77E-02	1.00E+00
CATSPER4	-5.20E+00	8.24E-23	PODXL	7.99E-02	1.00E+00	GPR108	-6.87E-02	1.00E+00
SLC4A4	1.58E+00	8.25E-23	SPIN1	1.16E-01	1.00E+00	NDRG1	8.18E-02	1.00E+00
ORC4	9.07E-01	8.27E-23	RAB11B	7.06E-02	1.00E+00	BRAF	-6.69E-02	1.00E+00
LOC102415277	-6.89E+00	8.49E-23	NSDHL	-5.64E-02	1.00E+00	BIN2	3.13E-01	1.00E+00
PRKAG1	9.31E-01	8.56E-23	ACSL4	6.90E-02	1.00E+00	LOC102403745	9.33E-02	1.00E+00
TMEM170B	-1.47E+00	8.57E-23	CTSD	-5.46E-02	1.00E+00	SLC6A14	-1.50E-01	1.00E+00
SLC25A47	2.95E+00	8.65E-23	UFD1	-7.42E-02	1.00E+00	NME7	6.73E-02	1.00E+00
RPS27L	-1.61E+00	8.74E-23	ERICH6	2.00E-01	1.00E+00	EFCAB11	-8.13E-02	1.00E+00
DERL3	2.50E+00	8.78E-23	LAMC3	3.91E-01	1.00E+00	COG8	8.07E-02	1.00E+00
SCN5A	1.85E+00	8.95E-23	TMEM106B	2.44E-01	1.00E+00	CES2	-1.25E-01	1.00E+00
NDUFS3	-1.61E+00	8.97E-23	PPP1R12C	-7.17E-02	1.00E+00	POGZ	-7.61E-02	1.00E+00
FGFR1OP2	-1.90E+00	9.05E-23	SUMF1	7.49E-02	1.00E+00	DNAJC5	6.30E-02	1.00E+00
LOC112580833	-4.22E+00	9.17E-23	FOXC1	2.50E-01	1.00E+00	ABRAXAS2	7.01E-02	1.00E+00
DNAJC24	-	9.27E-23	ACVRL1	1.89E-01	1.00E+00	PDXP	9.46E-02	1.00E+00

	1.87E+00							
ETV6	1.23E+00	9.47E-23	IFRD2	-1.52E-01	1.00E+00	UBQLN4	-5.96E-02	1.00E+00
LOC102392517	-2.51E+00	9.49E-23	IGSF3	-7.06E-02	1.00E+00	DNAJC11	-5.81E-02	1.00E+00
TSKS	2.40E+00	9.49E-23	ARPC4	5.93E-02	1.00E+00	LOC102394770	-9.19E-02	1.00E+00
CCND2	-5.28E+00	9.51E-23	SPCS3	5.34E-02	1.00E+00	DST	9.10E-02	1.00E+00
BIRC3	-1.95E+00	9.61E-23	LOC112585512	-1.78E-01	1.00E+00	LOC102406518	-9.82E-02	1.00E+00
NOP2	1.05E+00	9.73E-23	BCL2L10	-6.19E-02	1.00E+00	ZCRB1	7.23E-02	1.00E+00
LOC112585883	4.68E+00	9.95E-23	FAM171A2	3.49E-01	1.00E+00	DLGAP3	-1.35E-01	1.00E+00
LOC102394407	-8.88E-01	9.99E-23	CLN5	-1.17E-01	1.00E+00	TRMT2A	8.01E-02	1.00E+00
TRAIP	1.49E+00	1.00E-22	COL25A1	-1.45E-01	1.00E+00	DDX41	-6.79E-02	1.00E+00
CCDC69	-3.07E+00	1.01E-22	CDCA7L	-8.82E-02	1.00E+00	THPO	-1.38E-01	1.00E+00
ACAP3	-1.47E+00	1.01E-22	RAPH1	6.70E-02	1.00E+00	LOC102405614	-1.50E-01	1.00E+00
TBXAS1	1.86E+00	1.04E-22	PRMT9	-9.65E-02	1.00E+00	RAP1B	6.75E-02	1.00E+00
ESRRG	4.84E+00	1.04E-22	FOXO1	7.48E-02	1.00E+00	MAP1LC3A	-7.66E-02	1.00E+00
ACTR1A	-9.39E-01	1.05E-22	PRPF39	1.09E-01	1.00E+00	SERTAD3	7.25E-02	1.00E+00
LOC102398431	-4.64E+00	1.05E-22	PRRT3	-2.38E-01	1.00E+00	PUM2	6.85E-02	1.00E+00
SAMD4A	-2.96E+00	1.09E-22	DNASE2	-1.10E-01	1.00E+00	SYNGR1	6.88E-02	1.00E+00
KCNQ1	2.00E+00	1.10E-22	GPR137C	1.49E-01	1.00E+00	C2H6orf62	6.35E-02	1.00E+00
TRPC7	6.42E+00	1.12E-22	METRN	-3.01E-01	1.00E+00	DTWD2	1.53E-01	1.00E+00
ABCG1	-6.01E+00	1.15E-22	CHID1	8.59E-02	1.00E+00	DHX8	6.03E-02	1.00E+00
LOC112580462	-2.15E+00	1.15E-22	PAXBP1	1.24E-01	1.00E+00	NECAP1	-7.22E-02	1.00E+00
LOC102404341	-4.78E+00	1.16E-22	AKAP6	1.11E-01	1.00E+00	RAB35	-6.03E-02	1.00E+00
LOC102389532	-4.56E+00	1.17E-22	FLVCR1	1.77E-01	1.00E+00	PHETA1	-7.31E-02	1.00E+00
LOC102400529	-4.03E+00	1.19E-22	PFKL	3.14E-01	1.00E+00	ENTR1	-6.18E-02	1.00E+00
RAD23A	-1.26E+00	1.20E-22	TROVE2	-1.22E-01	1.00E+00	GMPR	-1.09E-01	1.00E+00
LOC112577778	4.75E+00	1.20E-22	JAZF1	-5.78E-02	1.00E+00	ACADM	-6.39E-02	1.00E+00
LOC102394968	-5.04E+00	1.21E-22	ICAM4	-1.59E-01	1.00E+00	RTN2	7.81E-02	1.00E+00
LOC102411789	2.54E+00	1.21E-22	USP11	5.39E-02	1.00E+00	BBC3	-1.23E-01	1.00E+00
METTL21A	1.20E+00	1.22E-22	EHHADH	1.73E-01	1.00E+00	SLC16A10	-9.85E-02	1.00E+00
RNF207	4.55E+00	1.25E-22	FREM1	9.24E-02	1.00E+00	C4H12orf4	6.92E-02	1.00E+00
SMAD9	-2.96E+00	1.25E-22	LOC102411003	-9.28E-02	1.00E+00	ELMO3	-9.90E-02	1.00E+00
WNT5A	-2.80E+00	1.25E-22	FAHD1	-8.16E-02	1.00E+00	LOC112578454	-7.06E-02	1.00E+00
LOC102390270	-6.81E+00	1.28E-22	SIRT7	-7.60E-02	1.00E+00	MIER1	-8.55E-02	1.00E+00

HID1	1.38E+00	1.30E-22	TRIM52	-6.61E-02	1.00E+00	DENND2C	-1.71E-01	1.00E+00
CCR10	-5.40E+00	1.31E-22	WDR5	5.75E-02	1.00E+00	NDUFS7	8.06E-02	1.00E+00
LOC112581613	5.18E+00	1.32E-22	RAB9B	-2.18E-01	1.00E+00	PSMB4	7.91E-02	1.00E+00
RNPEPL1	-1.28E+00	1.35E-22	CLN6	1.89E-01	1.00E+00	LOC102398643	-1.47E-01	1.00E+00
TANGO6	-1.24E+00	1.39E-22	ZSWIM1	-2.41E-01	1.00E+00	NFIC	6.31E-02	1.00E+00
RALGPS1	-1.45E+00	1.39E-22	MED13	1.23E-01	1.00E+00	HMGN3	-7.78E-02	1.00E+00
CSF1	-4.22E+00	1.42E-22	CCDC59	7.43E-02	1.00E+00	BLVRA	-7.12E-02	1.00E+00
CPE	-2.07E+00	1.42E-22	INTS6L	-2.43E-01	1.00E+00	DNAJC1	7.20E-02	1.00E+00
SRP68	1.03E+00	1.42E-22	C19H5orf22	-1.00E-01	1.00E+00	TRIM52	-7.24E-02	1.00E+00
OLFML3	-4.40E+00	1.43E-22	DUSP27	-4.10E-01	1.00E+00	LOC102401265	-1.15E-01	1.00E+00
UBN1	1.16E+00	1.44E-22	PRDM5	7.71E-02	1.00E+00	MANBAL	-7.44E-02	1.00E+00
VDAC3	9.04E-01	1.46E-22	RGS17	-3.80E-01	1.00E+00	LOC112585227	1.20E-01	1.00E+00
DAGLB	-1.05E+00	1.46E-22	SH2B1	6.28E-02	1.00E+00	NPM1	-8.65E-02	1.00E+00
TMEM223	-1.54E+00	1.48E-22	RPL34	-1.18E-01	1.00E+00	LOC102414812	8.50E-02	1.00E+00
FPGS	-1.34E+00	1.48E-22	LOC112586164	-3.32E-01	1.00E+00	MINDY3	-7.07E-02	1.00E+00
YIF1A	-1.28E+00	1.52E-22	HOXC4	-1.93E-01	1.00E+00	ATP2A1	1.36E-01	1.00E+00
CPLANE2	-1.89E+00	1.53E-22	BDNF	-1.35E-01	1.00E+00	SERPINC1	1.54E-01	1.00E+00
DCAF7	9.60E-01	1.53E-22	AVL9	7.61E-02	1.00E+00	PLAA	-5.75E-02	1.00E+00
ADAMTS13	6.58E+00	1.56E-22	MTHFD1	6.13E-02	1.00E+00	LOC102415948	1.45E-01	1.00E+00
TRNT1	1.11E+00	1.56E-22	SAMD1	-1.64E-01	1.00E+00	SLC13A3	-1.06E-01	1.00E+00
SYPL1	1.13E+00	1.58E-22	LOC112577606	8.95E-02	1.00E+00	CHCHD10	-1.09E-01	1.00E+00
PROB1	-2.49E+00	1.58E-22	NOSTRIN	-8.96E-02	1.00E+00	CHST12	-7.63E-02	1.00E+00
EFNA5	-1.55E+00	1.60E-22	ANXA1	-4.14E-01	1.00E+00	AGA	-1.22E-01	1.00E+00
STAR	4.43E+00	1.62E-22	GATAD1	-1.90E-01	1.00E+00	LRP12	1.00E-01	1.00E+00
LOC102406498	-5.44E+00	1.64E-22	TRIM50	-7.90E-02	1.00E+00	FAM3A	-7.36E-02	1.00E+00
SLC40A1	2.23E+00	1.64E-22	PARP12	-7.59E-02	1.00E+00	LOC102409355	1.20E-01	1.00E+00
LOC102390054	1.38E+00	1.65E-22	B4GALT5	8.00E-02	1.00E+00	TMEM67	-1.03E-01	1.00E+00
SLC30A9	-1.05E+00	1.67E-22	CCNG1	-5.65E-02	1.00E+00	TROVE2	9.92E-02	1.00E+00
HACE1	1.03E+00	1.67E-22	OLFML2A	-1.67E-01	1.00E+00	GUK1	-8.98E-02	1.00E+00
CCNH	1.01E+00	1.68E-22	LOC102409034	-3.00E-01	1.00E+00	RIIAD1	-1.33E-01	1.00E+00
NECAP1	-1.13E+00	1.68E-22	SHISA9	-1.94E-01	1.00E+00	EPHX3	8.82E-02	1.00E+00
TTC31	-3.29E+00	1.69E-22	BTAF1	7.98E-02	1.00E+00	FAM160A2	5.80E-02	1.00E+00
LURAP1L	-	1.69E-22	MPHOSPH10	-8.35E-	1.00E+00	NDUFAF5	-6.41E-	1.00E+00

	3.63E+00			02			02	
LOC102402730	-4.13E+00	1.69E-22	LOC112580131	-2.43E-01	1.00E+00	TIA1	-8.05E-02	1.00E+00
NUDT12	-1.67E+00	1.69E-22	PSD	2.65E-01	1.00E+00	KANK1	-8.91E-02	1.00E+00
PRKCZ	1.43E+00	1.69E-22	UNC5C	-6.62E-02	1.00E+00	LOC102392581	9.47E-02	1.00E+00
EIF2B4	-1.16E+00	1.72E-22	BBC3	-2.02E-01	1.00E+00	ELP3	6.34E-02	1.00E+00
COX1	-3.73E+00	1.72E-22	CHRD	-2.45E-01	1.00E+00	DMPK	-9.74E-02	1.00E+00
LOC112578530	5.13E+00	1.73E-22	CIAPIN1	6.10E-02	1.00E+00	PHACTR4	7.12E-02	1.00E+00
FEM1B	-1.05E+00	1.75E-22	TPRKB	-1.52E-01	1.00E+00	DDX1	-5.80E-02	1.00E+00
SHISA2	-2.22E+00	1.78E-22	LOC112586500	2.60E-01	1.00E+00	LOC102400624	-6.66E-02	1.00E+00
GNAI2	-1.04E+00	1.80E-22	HMGA1	5.91E-02	1.00E+00	MCF2L	1.66E-01	1.00E+00
ZNF277	-1.22E+00	1.82E-22	PLPP1	-1.25E-01	1.00E+00	PCSK5	1.04E-01	1.00E+00
MAGI2	3.33E+00	1.83E-22	YEATS2	7.64E-02	1.00E+00	ACER2	1.37E-01	1.00E+00
NME7	9.89E-01	1.86E-22	RAD51B	-8.64E-02	1.00E+00	SKIV2L	5.74E-02	1.00E+00
PRDX4	-1.53E+00	1.86E-22	RNF125	6.14E-01	1.00E+00	ANO10	8.18E-02	1.00E+00
MARCH1	-3.61E+00	1.89E-22	LOC102408653	-2.14E-01	1.00E+00	THOP1	7.21E-02	1.00E+00
LOC112582955	-2.16E+00	1.92E-22	NFKBID	2.90E-01	1.00E+00	JARID2	8.67E-02	1.00E+00
TSC2	-1.05E+00	1.93E-22	PSTPIP1	9.12E-02	1.00E+00	CNP	6.69E-02	1.00E+00
PROCR	3.93E+00	1.94E-22	COMMD3	-8.14E-02	1.00E+00	LOC102392263	7.75E-02	1.00E+00
LOC102398059	-1.66E+00	1.95E-22	LOC112580601	-9.57E-02	1.00E+00	RTF1	-5.79E-02	1.00E+00
SLC25A51	9.80E-01	1.96E-22	LOC102395720	1.76E-01	1.00E+00	LZIC	-6.33E-02	1.00E+00
ZFP14	1.41E+00	1.97E-22	SIRT4	1.33E-01	1.00E+00	UBE2Q1	5.75E-02	1.00E+00
A3GALT2	-3.98E+00	1.99E-22	SLC19A2	6.23E-02	1.00E+00	U2AF1L4	-7.02E-02	1.00E+00
LOC102394580	9.19E-01	2.05E-22	SMAD2	5.30E-02	1.00E+00	LRRK2	1.06E-01	1.00E+00
CCT6B	-2.29E+00	2.07E-22	ARL4A	-6.34E-02	1.00E+00	TMEM259	-7.05E-02	1.00E+00
SORCS1	5.00E+00	2.09E-22	INO80D	1.05E-01	1.00E+00	SLC41A3	7.14E-02	1.00E+00
LOC102391549	2.37E+00	2.11E-22	UBA1	5.97E-02	1.00E+00	LOC112578811	7.15E-02	1.00E+00
SH2D6	-4.10E+00	2.12E-22	C3H9orf85	-1.87E-01	1.00E+00	CDCA3	-1.03E-01	1.00E+00
MAPRE3	1.03E+00	2.14E-22	CHST1	2.39E-01	1.00E+00	LOC112580462	1.03E-01	1.00E+00
REX1BD	-1.79E+00	2.14E-22	LOC112584582	-2.45E-01	1.00E+00	DTD2	-1.18E-01	1.00E+00
GPR107	-1.16E+00	2.21E-22	MED24	-5.78E-02	1.00E+00	LOC102407094	-7.22E-02	1.00E+00
UBE2D2	-1.04E+00	2.23E-22	LOC112585772	-2.71E-01	1.00E+00	ARPP19	5.96E-02	1.00E+00
ATF4	-1.59E+00	2.26E-22	NUP85	-6.66E-02	1.00E+00	SELENOT	-5.78E-02	1.00E+00
NUDT18	-2.04E+00	2.27E-22	FAM107A	-1.63E-01	1.00E+00	LOC102394078	1.69E-01	1.00E+00
THRA	-1.24E+00	2.39E-22	FAM129A	8.13E-02	1.00E+00	JDP2	-1.43E-01	1.00E+00
MED23	-1.86E+00	2.40E-22	DDX39A	6.46E-02	1.00E+00	RAD50	-5.99E-02	1.00E+00

MRPL57	-2.52E+00	2.48E-22	RAB40C	5.85E-02	1.00E+00	PXDC1	-8.40E-02	1.00E+00
CTSC	3.30E+00	2.50E-22	LOC102416484	2.20E-01	1.00E+00	UBXN11	-9.23E-02	1.00E+00
SUPT5H	8.78E-01	2.61E-22	MBLAC1	-1.51E-01	1.00E+00	SLC7A6OS	-7.82E-02	1.00E+00
HSPA6	-4.56E+00	2.68E-22	VARS2	9.33E-02	1.00E+00	DPP7	7.31E-02	1.00E+00
PRMT3	-1.30E+00	2.69E-22	ARHGAP5	9.21E-02	1.00E+00	LOC112585747	1.23E-01	1.00E+00
LOC112578208	6.10E+00	2.71E-22	LOC102398677	4.33E-01	1.00E+00	RNF168	9.56E-02	1.00E+00
CCDC13	4.33E+00	2.74E-22	MED6	-7.04E-02	1.00E+00	HNRNPH3	-6.34E-02	1.00E+00
SGCB	-2.18E+00	2.75E-22	ZNF281	1.11E-01	1.00E+00	LOC102391189	-6.98E-02	1.00E+00
OTUD4	1.46E+00	2.77E-22	GPAT4	-5.23E-02	1.00E+00	NDUFAF6	-6.81E-02	1.00E+00
ZNF41	-3.77E+00	2.77E-22	SHTN1	5.32E-02	1.00E+00	KARS	-7.22E-02	1.00E+00
LACTBL1	4.25E+00	2.85E-22	SOS2	-1.72E-01	1.00E+00	CDC5L	-5.90E-02	1.00E+00
SHANK1	-2.71E+00	2.92E-22	YTHDF2	-7.42E-02	1.00E+00	FMNL1	-2.31E-01	1.00E+00
ATP6V0E1	-1.29E+00	2.93E-22	GLB1L	3.11E-01	1.00E+00	CNDP2	7.30E-02	1.00E+00
LOC112586919	2.35E+00	2.94E-22	SRPK1	-7.31E-02	1.00E+00	LOC102410132	-1.30E-01	1.00E+00
LOC102401786	1.04E+00	2.98E-22	LOC112580692	-2.32E-01	1.00E+00	KIF13A	-7.12E-02	1.00E+00
SLC24A4	5.62E+00	3.00E-22	ARRB1	9.85E-02	1.00E+00	LYRM1	9.03E-02	1.00E+00
LOC102408209	2.45E+00	3.05E-22	C16H11orf74	-8.40E-02	1.00E+00	NT5C	1.01E-01	1.00E+00
OOSP2	6.70E+00	3.09E-22	NGFR	1.61E-01	1.00E+00	CAGE1	-1.03E-01	1.00E+00
SCAP	-1.13E+00	3.17E-22	PDE6D	5.84E-02	1.00E+00	MYD88	-6.89E-02	1.00E+00
DDX50	-1.89E+00	3.19E-22	LOC102413479	1.43E-01	1.00E+00	NUP133	6.23E-02	1.00E+00
KMT2A	-1.86E+00	3.20E-22	CAMK4	-2.16E-01	1.00E+00	USP35	7.44E-02	1.00E+00
HDX	-4.68E+00	3.23E-22	LOC112581371	3.47E-01	1.00E+00	DQX1	-8.60E-02	1.00E+00
DUSP11	-1.26E+00	3.26E-22	KTN1	-6.75E-02	1.00E+00	ANAPC4	-5.98E-02	1.00E+00
PADI3	5.05E+00	3.32E-22	PSPC1	8.68E-02	1.00E+00	SPIRE2	-8.55E-02	1.00E+00
RAD51B	1.44E+00	3.40E-22	CIZ1	5.92E-02	1.00E+00	KDM4C	7.60E-02	1.00E+00
LOC112582356	-2.00E+00	3.41E-22	CEBPG	-8.36E-02	1.00E+00	SEMA4C	7.81E-02	1.00E+00
FKTN	-1.34E+00	3.59E-22	ZNF829	1.28E-01	1.00E+00	NSUN5	-7.80E-02	1.00E+00
LURAP1	-5.46E+00	3.60E-22	PANK2	-8.40E-02	1.00E+00	IDH1	-9.72E-02	1.00E+00
FAM13B	1.29E+00	3.64E-22	PPP1R18	-4.66E-01	1.00E+00	DNTTIP1	7.05E-02	1.00E+00
LOC102416294	-3.96E+00	3.65E-22	NRAP	2.31E-01	1.00E+00	DCTPP1	1.01E-01	1.00E+00
GCNT4	2.82E+00	3.70E-22	DHX29	-9.05E-02	1.00E+00	LTBP3	6.44E-02	1.00E+00
ETNK1	-1.30E+00	3.72E-22	TRPS1	-1.35E-01	1.00E+00	LMBRD2	-9.95E-02	1.00E+00
MTFR2	1.29E+00	3.74E-22	HAUS3	1.10E-01	1.00E+00	PLAGL1	-9.22E-02	1.00E+00

EGR3	1.10E+00	3.77E-22	DPAGT1	8.68E-02	1.00E+00	LOC102390765	1.39E-01	1.00E+00
LZTFL1	-1.55E+00	3.89E-22	SLC35F6	-7.88E-02	1.00E+00	LOC102414209	9.65E-02	1.00E+00
ASXL2	1.44E+00	3.91E-22	NABP2	-9.73E-02	1.00E+00	RNF113A	8.88E-02	1.00E+00
RHOF	-1.77E+00	3.93E-22	LOC112586975	2.69E-01	1.00E+00	MXD3	1.12E-01	1.00E+00
STAC	-4.73E+00	4.02E-22	DTWD1	-7.27E-02	1.00E+00	LOC102415513	6.99E-02	1.00E+00
TRIAP1	1.29E+00	4.04E-22	LOC102397911	7.78E-02	1.00E+00	C18H16orf70	-7.14E-02	1.00E+00
PPIL1	-1.28E+00	4.08E-22	DNAI2	-1.75E-01	1.00E+00	BBS10	-1.14E-01	1.00E+00
WSB1	-1.43E+00	4.10E-22	RAB3IP	5.36E-02	1.00E+00	LOC102395109	-7.69E-02	1.00E+00
LMAN2	-1.29E+00	4.33E-22	LOC112587471	2.35E-01	1.00E+00	C2H2orf76	-8.27E-02	1.00E+00
LOC102402779	-5.08E+00	4.38E-22	CTPS2	8.42E-02	1.00E+00	TMOD3	1.07E-01	1.00E+00
BACH2	-3.62E+00	4.46E-22	ATP5MC3	-6.46E-02	1.00E+00	CCDC112	7.73E-02	1.00E+00
NETO1	4.32E+00	4.47E-22	LOC112586432	-3.49E-01	1.00E+00	UBA7	-7.87E-02	1.00E+00
LOC102397082	-1.54E+00	4.51E-22	COQ8A	-5.98E-02	1.00E+00	DTX3	-6.07E-02	1.00E+00
PDP2	1.24E+00	4.59E-22	EPC1	6.19E-02	1.00E+00	CA8	9.35E-02	1.00E+00
ANKRD40	9.28E-01	4.65E-22	KAT8	-8.45E-02	1.00E+00	ZBTB43	-1.64E-01	1.00E+00
LOC102406860	-5.26E+00	4.67E-22	MAPK8IP1	7.51E-02	1.00E+00	REXO5	-6.53E-02	1.00E+00
MRPL27	-2.03E+00	4.72E-22	BRD2	5.86E-02	1.00E+00	EIF3A	-7.49E-02	1.00E+00
LOC112586853	6.77E+00	4.75E-22	LMOD3	5.48E-02	1.00E+00	NUDT16L1	9.31E-02	1.00E+00
C6H1orf123	-1.61E+00	4.86E-22	LOC102408791	-9.63E-02	1.00E+00	TM9SF2	-6.33E-02	1.00E+00
EIF2B2	-1.17E+00	4.86E-22	ZNF200	6.71E-02	1.00E+00	FRRS1L	-1.14E-01	1.00E+00
FITM1	-3.67E+00	4.95E-22	POLB	6.44E-02	1.00E+00	DNM1L	-8.64E-02	1.00E+00
BCL3	-1.74E+00	4.99E-22	CMPK2	2.43E-01	1.00E+00	ITPR3	1.34E-01	1.00E+00
LOC102396820	-4.51E+00	5.06E-22	WNT16	3.72E-01	1.00E+00	PTPN1	6.48E-02	1.00E+00
TTC23L	2.76E+00	5.10E-22	EPHA2	8.79E-02	1.00E+00	CEP85L	1.01E-01	1.00E+00
ARMCX3	-4.65E+00	5.22E-22	RASGRP2	-8.77E-02	1.00E+00	ACSM3	1.56E-01	1.00E+00
AMPD2	-1.06E+00	5.26E-22	GRM7	-8.35E-02	1.00E+00	LOC102398703	2.38E-01	1.00E+00
ZNF35	-1.31E+00	5.28E-22	UBE2I	-5.69E-02	1.00E+00	AURKA	-8.49E-02	1.00E+00
MSS51	-4.21E+00	5.28E-22	LOC112586833	-1.03E-01	1.00E+00	TCTN1	-5.80E-02	1.00E+00
ASNA1	-1.42E+00	5.31E-22	ZC3H7B	1.36E-01	1.00E+00	DLG5	8.45E-02	1.00E+00
LOC112577760	-2.00E+00	5.39E-22	AOAH	1.35E-01	1.00E+00	TMEM267	-1.12E-01	1.00E+00
SETD5	-1.88E+00	5.52E-22	TPM3	-5.67E-02	1.00E+00	ZCCHC3	-5.57E-02	1.00E+00
TAF7L	-3.22E+00	5.57E-22	LOC102389323	-1.77E-01	1.00E+00	SP110	-8.83E-02	1.00E+00
CDC42EP3	-4.26E+00	5.60E-22	CHM	8.76E-02	1.00E+00	DPYSL3	1.31E-01	1.00E+00
LOC102415240	-	5.77E-22	RGS3	1.03E-01	1.00E+00	RPP40	-8.44E-	1.00E+00

	5.10E+00						02	
SASH1	-4.64E+00	5.83E-22	ALG5	1.39E-01	1.00E+00	TCEAL1	-6.74E-02	1.00E+00
RBM27	1.24E+00	5.83E-22	RNF103	1.18E-01	1.00E+00	RHOBTB3	8.01E-02	1.00E+00
LOC112585751	2.33E+00	5.85E-22	FRS2	-1.03E-01	1.00E+00	RASAL3	-1.62E-01	1.00E+00
MAPK15	-3.93E+00	5.88E-22	PSMD11	5.37E-02	1.00E+00	NDUFS8	9.05E-02	1.00E+00
ARG2	1.42E+00	5.99E-22	MSH5	9.60E-02	1.00E+00	TTLL9	1.35E-01	1.00E+00
UBR5	-1.25E+00	6.00E-22	MTMR1	-5.71E-02	1.00E+00	ETFBKMT	1.31E-01	1.00E+00
MYCBP	1.82E+00	6.00E-22	RND2	8.38E-02	1.00E+00	MTSS1	8.94E-02	1.00E+00
GALNT13	5.49E+00	6.15E-22	HSPA9	5.46E-02	1.00E+00	LOC112581738	1.24E-01	1.00E+00
WHRN	-3.84E+00	6.15E-22	NFATC4	-6.46E-02	1.00E+00	SLC48A1	-7.33E-02	1.00E+00
ALKBH4	1.07E+00	6.19E-22	CXCL13	-4.43E-01	1.00E+00	NOXO1	1.49E-01	1.00E+00
HUNK	-1.76E+00	6.29E-22	TIMM9	5.41E-02	1.00E+00	TCEANC	1.05E-01	1.00E+00
PM20D2	-5.13E+00	6.34E-22	SRF	7.96E-02	1.00E+00	FTL	-1.04E-01	1.00E+00
GBF1	1.52E+00	6.34E-22	QSER1	1.07E-01	1.00E+00	CAMK2D	1.13E-01	1.00E+00
SPRN	-2.64E+00	6.38E-22	SLC25A40	7.23E-02	1.00E+00	NECTIN3	-6.75E-02	1.00E+00
LOC112584475	2.68E+00	6.38E-22	ZBTB41	1.56E-01	1.00E+00	ZFP91	-5.41E-02	1.00E+00
RNF181	-1.83E+00	6.43E-22	CDC7	6.68E-02	1.00E+00	GRAMD1A	-5.65E-02	1.00E+00
EIF2S2	-1.40E+00	6.43E-22	MMS22L	5.71E-02	1.00E+00	PHF1	-5.84E-02	1.00E+00
KIF7	-1.56E+00	6.43E-22	LOC112578814	-2.84E-01	1.00E+00	BTBD9	-5.62E-02	1.00E+00
LOC102399180	-2.18E+00	6.48E-22	LOC102414386	-1.15E-01	1.00E+00	COL5A1	8.26E-02	1.00E+00
SGTA	1.08E+00	7.01E-22	LOC112587909	2.63E-01	1.00E+00	PLEKHH2	-1.09E-01	1.00E+00
CCNC	-1.23E+00	7.15E-22	LOC102392019	-4.31E-01	1.00E+00	HAT1	-6.94E-02	1.00E+00
ZNF185	-3.09E+00	7.20E-22	ZNF577	-1.23E-01	1.00E+00	OTOA	1.56E-01	1.00E+00
FHAD1	4.00E+00	7.45E-22	UBFD1	7.02E-02	1.00E+00	BORCS8	1.04E-01	1.00E+00
MAFF	-3.55E+00	7.48E-22	CMTM7	-8.53E-02	1.00E+00	RB1CC1	6.34E-02	1.00E+00
ARSJ	-5.60E+00	7.50E-22	ESYT1	8.87E-02	1.00E+00	BEND7	7.13E-02	1.00E+00
FOSB	-4.33E+00	7.58E-22	ATF7IP	-7.98E-02	1.00E+00	NDUFAF7	-7.27E-02	1.00E+00
FAM71E1	1.94E+00	7.66E-22	EPHA10	6.76E-02	1.00E+00	WIPI2	-5.95E-02	1.00E+00
MYOC	3.98E+00	7.66E-22	LOC102408576	-2.44E-01	1.00E+00	LOC112585719	-1.44E-01	1.00E+00
EBF1	4.05E+00	7.68E-22	CPEB1	6.88E-02	1.00E+00	VSTM5	1.20E-01	1.00E+00
VARS2	-1.25E+00	7.76E-22	NKAP	-7.12E-02	1.00E+00	MRPS18C	7.32E-02	1.00E+00
LOC112579536	-4.22E+00	7.91E-22	SFXN4	-6.73E-02	1.00E+00	DYNC1I2	5.89E-02	1.00E+00
NPTX2	2.83E+00	8.03E-22	INO80B	1.63E-01	1.00E+00	CHRM2	6.58E-02	1.00E+00
LOC102408997	-1.38E+00	8.15E-22	LOC102415868	-1.45E-01	1.00E+00	PPM1A	-6.10E-02	1.00E+00
PTPRH	-3.58E+00	8.44E-22	IGF2BP1	-1.84E-01	1.00E+00	TM2D3	-6.16E-02	1.00E+00

LOC102392178	-4.87E+00	8.54E-22	OS9	6.58E-02	1.00E+00	EIF3E	-8.01E-02	1.00E+00
TNN	1.68E+00	8.55E-22	METTL17	-3.18E-01	1.00E+00	LOC102392446	-6.52E-02	1.00E+00
TRAF3IP1	1.14E+00	8.57E-22	FDFT1	-6.96E-02	1.00E+00	C5H11orf24	6.85E-02	1.00E+00
TMEM80	-2.48E+00	8.75E-22	NFE2L2	-8.28E-02	1.00E+00	LOC112581029	-1.64E-01	1.00E+00
BRSK1	-1.37E+00	8.76E-22	TTC12	-1.45E-01	1.00E+00	CEP70	-6.45E-02	1.00E+00
VTI1A	7.99E-01	8.88E-22	LVRN	3.65E-01	1.00E+00	TMBIM6	6.12E-02	1.00E+00
POLR3D	9.75E-01	9.04E-22	TCN2	3.47E-01	1.00E+00	EGR2	1.98E-01	1.00E+00
LOC112581176	4.99E+00	9.07E-22	SLC7A7	-5.52E-02	1.00E+00	RBP4	-8.12E-02	1.00E+00
LOC102414434	-4.71E+00	9.09E-22	SEPT10	-1.78E-01	1.00E+00	DHTKD1	-7.07E-02	1.00E+00
FNDC8	3.59E+00	9.22E-22	TAPBP	-7.76E-02	1.00E+00	SH2B3	-9.25E-02	1.00E+00
TRAP1	1.39E+00	9.29E-22	LOC102405522	-2.62E-01	1.00E+00	TTC23L	1.43E-01	1.00E+00
HTRA3	-6.89E+00	9.44E-22	BCAR1	-6.90E-02	1.00E+00	CEPT1	-6.41E-02	1.00E+00
ZNF394	-1.74E+00	9.44E-22	RAPGEFL1	7.84E-02	1.00E+00	SYNE2	-8.90E-02	1.00E+00
LOC112579418	2.94E+00	9.50E-22	ACAD10	-1.81E-01	1.00E+00	HMGB1	-8.94E-02	1.00E+00
FGF23	-3.18E+00	9.66E-22	LOC112578474	-2.61E-01	1.00E+00	L3MBTL2	-7.41E-02	1.00E+00
SASS6	1.30E+00	9.68E-22	TERF1	1.38E-01	1.00E+00	SEC23IP	-6.79E-02	1.00E+00
NUP54	1.14E+00	1.00E-21	AVPI1	1.02E-01	1.00E+00	LOC102414041	-1.16E-01	1.00E+00
NKX2-2	6.93E+00	1.00E-21	PDGFRB	-2.54E-01	1.00E+00	RNASET2	6.34E-02	1.00E+00
SLC35E3	1.04E+00	1.02E-21	EFHB	-1.15E-01	1.00E+00	NIPBL	7.54E-02	1.00E+00
LOC102399929	1.25E+00	1.02E-21	SLC47A2	-1.56E-01	1.00E+00	LOC112586282	1.21E-01	1.00E+00
LOC102409513	-5.19E+00	1.03E-21	SLC39A3	1.65E-01	1.00E+00	ARHGEF10	-6.25E-02	1.00E+00
NACC1	-4.57E+00	1.04E-21	DDX31	-5.44E-02	1.00E+00	FAM160B1	-9.83E-02	1.00E+00
MAN1A2	-1.29E+00	1.04E-21	CCNF	1.24E-01	1.00E+00	GLYR1	-5.84E-02	1.00E+00
NR2E3	7.55E+00	1.07E-21	YIPF4	1.16E-01	1.00E+00	CACUL1	5.85E-02	1.00E+00
LOC102410999	-8.66E+00	1.12E-21	TBRG4	6.29E-02	1.00E+00	MZF1	6.17E-02	1.00E+00
LOC112586866	2.01E+00	1.12E-21	AAGAB	4.95E-02	1.00E+00	GPI	8.69E-02	1.00E+00
DISC1	3.79E+00	1.13E-21	SLC1A5	5.85E-02	1.00E+00	TRAPPC8	7.80E-02	1.00E+00
ARHGAP19	1.46E+00	1.14E-21	C15H8orf82	1.15E-01	1.00E+00	CLTB	7.45E-02	1.00E+00
CLDN3	-3.11E+00	1.15E-21	CACNB3	-9.17E-02	1.00E+00	ITGA3	-6.34E-02	1.00E+00
SCUBE1	-3.48E+00	1.16E-21	LHX2	6.83E-02	1.00E+00	KXD1	7.52E-02	1.00E+00
ABCD4	-1.17E+00	1.17E-21	KLHL21	6.99E-02	1.00E+00	SPAG7	-6.99E-02	1.00E+00
NICN1	-1.12E+00	1.17E-21	TFAP2B	-5.49E-02	1.00E+00	NIFK	5.83E-02	1.00E+00
ITGB3	-2.84E+00	1.17E-21	ARF5	6.49E-02	1.00E+00	DSG2	-7.37E-02	1.00E+00
USP22	1.24E+00	1.19E-21	HK2	2.95E-01	1.00E+00	TTBK2	-8.57E-02	1.00E+00

LOC112579690	5.11E+00	1.20E-21	CPLANE2	-1.68E-01	1.00E+00	FDX1	6.46E-02	1.00E+00
XPNPEP3	-1.44E+00	1.20E-21	NBR1	6.79E-02	1.00E+00	RBBP8	-5.62E-02	1.00E+00
GFPT1	1.01E+00	1.21E-21	ADCY7	8.81E-02	1.00E+00	BIRC5	-8.21E-02	1.00E+00
ARHGAP1	-9.91E-01	1.22E-21	LOC102398169	-6.88E-02	1.00E+00	TBL1X	5.41E-02	1.00E+00
LOC102390323	5.87E+00	1.23E-21	DDB2	-6.97E-02	1.00E+00	LTBP4	-9.97E-02	1.00E+00
TMOD4	-3.84E+00	1.23E-21	PARP3	-3.04E-01	1.00E+00	CTDSPL2	-6.56E-02	1.00E+00
GRIPAP1	9.31E-01	1.26E-21	CELF3	1.66E-01	1.00E+00	HNRNPA1	-7.65E-02	1.00E+00
ZNF711	-4.43E+00	1.28E-21	LOC102414221	2.06E-01	1.00E+00	DMXL2	-8.81E-02	1.00E+00
EEA1	1.72E+00	1.29E-21	MCTS1	1.38E-01	1.00E+00	KLHDC8B	-1.00E-01	1.00E+00
LOC112580183	-5.44E+00	1.31E-21	RSAD1	3.38E-01	1.00E+00	MCAT	6.21E-02	1.00E+00
MPZ	4.03E+00	1.34E-21	ISL1	1.01E-01	1.00E+00	WDR86	-1.43E-01	1.00E+00
FGD2	4.20E+00	1.35E-21	POC5	-6.31E-02	1.00E+00	GRIN2D	1.04E-01	1.00E+00
MYO3B	5.17E+00	1.37E-21	LOC102398488	6.42E-02	1.00E+00	ATG14	-6.87E-02	1.00E+00
PODNL1	-4.39E+00	1.37E-21	FZD10	3.64E-01	1.00E+00	HSCB	8.52E-02	1.00E+00
AGPAT5	-1.09E+00	1.38E-21	INTS10	-6.46E-02	1.00E+00	GRB2	5.54E-02	1.00E+00
LOC102415281	-3.20E+00	1.40E-21	EMILIN2	-6.99E-02	1.00E+00	DCBLD1	-1.06E-01	1.00E+00
POLR2I	1.56E+00	1.41E-21	TFAP2A	1.60E-01	1.00E+00	GGH	6.81E-02	1.00E+00
ASCC1	8.90E-01	1.42E-21	PIGN	6.21E-02	1.00E+00	MRPL43	6.98E-02	1.00E+00
LOC112586189	4.91E+00	1.43E-21	DUSP15	-2.04E-01	1.00E+00	PLK2	1.15E-01	1.00E+00
DARS	-9.64E-01	1.44E-21	SUGT1	-6.23E-02	1.00E+00	PXK	8.10E-02	1.00E+00
CRELD2	-1.74E+00	1.46E-21	FXYD5	-3.70E-01	1.00E+00	CRKL	6.10E-02	1.00E+00
LOC102409906	5.01E+00	1.47E-21	METTL2A	-1.98E-01	1.00E+00	APIP	-7.87E-02	1.00E+00
AP1M1	-1.15E+00	1.49E-21	LOC112582281	-2.12E-01	1.00E+00	GPALPP1	-6.49E-02	1.00E+00
CNOT9	-9.09E-01	1.50E-21	AGPAT1	4.92E-02	1.00E+00	ENO1	1.05E-01	1.00E+00
LOC102406687	-1.60E+00	1.54E-21	NLRC4	-1.48E-01	1.00E+00	USP20	5.84E-02	1.00E+00
TMEM159	1.15E+00	1.55E-21	FZD3	7.52E-02	1.00E+00	LOC102400274	7.82E-02	1.00E+00
LRIG1	-1.52E+00	1.60E-21	CHMP2B	-6.78E-02	1.00E+00	YIPF5	-5.53E-02	1.00E+00
LOC102409773	4.73E+00	1.62E-21	LOC102401342	-1.59E-01	1.00E+00	DARS2	-8.45E-02	1.00E+00
LOC102402583	-1.49E+00	1.62E-21	MIDN	-2.26E-01	1.00E+00	MBD2	5.61E-02	1.00E+00
TP53I3	1.81E+00	1.63E-21	GLIS3	7.79E-02	1.00E+00	TIPRL	-5.99E-02	1.00E+00
FAM171B	-1.54E+00	1.65E-21	PSMC2	-6.95E-02	1.00E+00	PAK1	9.14E-02	1.00E+00
COL21A1	-5.00E+00	1.70E-21	TMEM92	5.53E-02	1.00E+00	UBOX5	6.96E-02	1.00E+00
EDN3	5.23E+00	1.75E-21	ACADM	-7.05E-02	1.00E+00	GRK2	5.98E-02	1.00E+00

PLCD4	-2.30E+00	1.76E-21	RPUSD2	-8.90E-02	1.00E+00	PIP4P2	-7.39E-02	1.00E+00
TTLL7	-2.94E+00	1.87E-21	DTNBP1	-8.97E-02	1.00E+00	ANKIB1	8.81E-02	1.00E+00
PPM1M	-2.07E+00	1.89E-21	SEPT1	-7.32E-02	1.00E+00	MED29	9.61E-02	1.00E+00
RSL24D1	9.74E-01	1.93E-21	RERGL	-2.55E-01	1.00E+00	MMP24OS	-1.15E-01	1.00E+00
TMF1	-1.66E+00	1.93E-21	OCLN	-5.91E-02	1.00E+00	KIAA0825	7.38E-02	1.00E+00
ZBTB25	-1.82E+00	1.99E-21	GGA2	5.41E-02	1.00E+00	SYNJ2	1.49E-01	1.00E+00
MAGEF1	-1.70E+00	2.00E-21	CPT2	-1.65E-01	1.00E+00	AIFM2	7.22E-02	1.00E+00
GSTA1	-6.49E+00	2.01E-21	GPN2	5.90E-02	1.00E+00	ZBTB44	-6.61E-02	1.00E+00
LOC102394106	-4.45E+00	2.09E-21	LYRM7	-1.16E-01	1.00E+00	PDZRN4	-8.36E-02	1.00E+00
DLEU7	-6.02E+00	2.14E-21	DNAL1	-1.38E-01	1.00E+00	TANGO6	-6.68E-02	1.00E+00
OVGP1	-3.58E+00	2.16E-21	ITGB1	2.28E-01	1.00E+00	TENM4	1.24E-01	1.00E+00
LOC112584536	4.73E+00	2.16E-21	C12H2orf42	8.61E-02	1.00E+00	PEA15	6.42E-02	1.00E+00
FBXW9	-3.35E+00	2.19E-21	LOC102410303	-1.55E-01	1.00E+00	TCP11	1.36E-01	1.00E+00
COQ10A	9.45E-01	2.21E-21	BRD4	6.83E-02	1.00E+00	TARS	5.74E-02	1.00E+00
USP32	1.83E+00	2.23E-21	TBC1D23	-8.07E-02	1.00E+00	SLC16A5	-8.18E-02	1.00E+00
CALN1	4.51E+00	2.35E-21	HSCB	-8.00E-02	1.00E+00	ACTR6	6.77E-02	1.00E+00
TAOK3	1.14E+00	2.38E-21	ASB8	6.14E-02	1.00E+00	TMEM192	-7.31E-02	1.00E+00
F8	-3.03E+00	2.39E-21	LDB1	5.62E-02	1.00E+00	PPID	6.04E-02	1.00E+00
LOC112578054	5.58E+00	2.41E-21	NECAP1	7.92E-02	1.00E+00	SIX5	-6.53E-02	1.00E+00
TNFRSF21	-5.37E+00	2.42E-21	LOC102401704	-1.96E-01	1.00E+00	NSF	7.78E-02	1.00E+00
LRIG2	-1.39E+00	2.43E-21	LOC112582329	2.92E-01	1.00E+00	RPL27A	-8.57E-02	1.00E+00
CDIP1	-9.22E-01	2.45E-21	MLKL	-7.71E-02	1.00E+00	PABPC4L	-1.02E-01	1.00E+00
REC8	2.17E+00	2.52E-21	ZNF782	-2.62E-01	1.00E+00	HDAC1	-5.44E-02	1.00E+00
LOC112587187	-4.84E+00	2.54E-21	TNPO2	7.97E-02	1.00E+00	ENDOV	-6.50E-02	1.00E+00
SUSD5	2.79E+00	2.55E-21	GNL3	-6.92E-02	1.00E+00	ATP7A	-9.92E-02	1.00E+00
DCAF12	8.36E-01	2.58E-21	LOC102411360	3.43E-01	1.00E+00	MASTL	-1.04E-01	1.00E+00
B3GALNT1	-1.53E+00	2.59E-21	TMEM168	7.30E-02	1.00E+00	TTLL11	1.35E-01	1.00E+00
POLR2A	1.42E+00	2.60E-21	REV3L	9.59E-02	1.00E+00	PJA2	-7.54E-02	1.00E+00
F2	-3.63E+00	2.64E-21	NDUFAF3	-1.13E-01	1.00E+00	RAPSN	8.15E-02	1.00E+00
LOC102409066	-4.75E+00	2.66E-21	MIER1	-1.49E-01	1.00E+00	LOC102408946	-1.43E-01	1.00E+00
MBD6	1.17E+00	2.66E-21	SPARC	-3.03E-01	1.00E+00	PRMT2	6.50E-02	1.00E+00
LIMK1	-1.27E+00	2.72E-21	ALCAM	3.07E-01	1.00E+00	AGGF1	-5.51E-02	1.00E+00
LOC102402708	-1.32E+00	2.76E-21	LOC112587651	3.86E-01	1.00E+00	TMEM161B	-7.61E-02	1.00E+00

RUBCN	-1.32E+00	2.77E-21	LINS1	6.94E-02	1.00E+00	UCP2	-1.80E-01	1.00E+00
SYTL4	1.13E+00	2.80E-21	TPPP3	-1.24E-01	1.00E+00	LOC102405658	-6.65E-02	1.00E+00
WNT11	-4.17E+00	2.81E-21	LOC102401082	-3.44E-01	1.00E+00	LOC102409912	-5.59E-02	1.00E+00
TTC28	1.32E+00	2.84E-21	OLFML2B	3.16E-01	1.00E+00	KCND3	-1.49E-01	1.00E+00
LOC112580132	1.62E+00	2.86E-21	DIS3	-6.48E-02	1.00E+00	LOC102411461	-5.64E-02	1.00E+00
SYT11	-1.38E+00	2.87E-21	PCNX2	-1.71E-01	1.00E+00	FOXO3	6.55E-02	1.00E+00
DNAJB5	-2.81E+00	2.89E-21	CCNDBP1	7.13E-02	1.00E+00	ZC3H10	6.88E-02	1.00E+00
FOXN3	-1.08E+00	2.95E-21	CCDC181	-6.49E-02	1.00E+00	WDR47	9.92E-02	1.00E+00
BCAP29	1.02E+00	2.95E-21	LOC102402269	-1.71E-01	1.00E+00	TARBP2	-8.32E-02	1.00E+00
GRIP1	-2.21E+00	3.01E-21	DDX5	5.40E-02	1.00E+00	BRD9	-5.49E-02	1.00E+00
DDN	2.73E+00	3.02E-21	FRMD4A	9.02E-02	1.00E+00	B4GALT4	6.20E-02	1.00E+00
IPO9	-8.61E-01	3.08E-21	SLC24A1	1.31E-01	1.00E+00	SSH3	6.51E-02	1.00E+00
BARX2	5.55E+00	3.10E-21	DNAJC30	7.25E-02	1.00E+00	LOC102393658	1.08E-01	1.00E+00
LOC112580893	5.70E+00	3.13E-21	SPON1	-6.38E-02	1.00E+00	KDM6A	-7.68E-02	1.00E+00
GTF2H3	-2.34E+00	3.14E-21	ALDOB	-1.07E-01	1.00E+00	LOC102415186	-6.07E-02	1.00E+00
DDC	5.01E+00	3.21E-21	SGSM3	5.21E-02	1.00E+00	LOC102400587	-1.22E-01	1.00E+00
PRRG1	-1.64E+00	3.23E-21	STK4	7.90E-02	1.00E+00	LOC102399179	-7.70E-02	1.00E+00
PHLDB2	-4.31E+00	3.23E-21	TMEM116	1.24E-01	1.00E+00	ARMCX1	-6.94E-02	1.00E+00
VPS29	9.52E-01	3.26E-21	SLC30A7	1.56E-01	1.00E+00	DPP4	6.94E-02	1.00E+00
GALNT14	5.08E+00	3.28E-21	PARS2	1.01E-01	1.00E+00	SCCPDH	-5.93E-02	1.00E+00
MRPS9	-1.08E+00	3.29E-21	MYL2	2.73E-01	1.00E+00	HSPA1L	-1.44E-01	1.00E+00
CRY1	1.13E+00	3.30E-21	MRAS	-8.85E-02	1.00E+00	CCDC130	-6.80E-02	1.00E+00
COPS8	8.65E-01	3.32E-21	LSR	8.46E-02	1.00E+00	LANCL2	6.51E-02	1.00E+00
CASKIN1	-2.30E+00	3.32E-21	TAL2	1.46E-01	1.00E+00	LOC102411786	-8.91E-02	1.00E+00
MORF4L1	-9.87E-01	3.39E-21	PAQR8	-2.07E-01	1.00E+00	LCMT1	-6.52E-02	1.00E+00
FGFR3	-4.44E+00	3.46E-21	TOR1B	8.45E-02	1.00E+00	CCDC88A	-7.52E-02	1.00E+00
LOC102411593	-1.15E+00	3.59E-21	LOC112581832	2.19E-01	1.00E+00	YIPF3	-6.44E-02	1.00E+00
LOC102397183	5.38E+00	3.61E-21	C24H16orf96	-2.37E-01	1.00E+00	GALM	-6.94E-02	1.00E+00
VGLL2	-3.54E+00	3.62E-21	C1QBP	-7.67E-02	1.00E+00	SHROOM1	1.32E-01	1.00E+00
DYRK1B	9.71E-01	3.66E-21	ASXL2	8.18E-02	1.00E+00	CREM	7.15E-02	1.00E+00
ITPK1	-1.24E+00	3.75E-21	RIMS1	1.46E-01	1.00E+00	LOC102410630	-1.55E-01	1.00E+00
ERBB3	-2.43E+00	3.86E-21	MAP3K7CL	-1.19E-01	1.00E+00	D2HGDH	-6.81E-02	1.00E+00
LOC112577706	2.13E+00	3.87E-21	ADAMTS10	3.14E-01	1.00E+00	PEX11G	-9.12E-02	1.00E+00
C4H12orf40	6.01E+00	3.95E-21	SLC12A8	6.27E-02	1.00E+00	NUP98	-6.33E-02	1.00E+00

PANK2	-9.28E-01	3.96E-21	BOD1L1	-1.87E-01	1.00E+00	USP38	-8.74E-02	1.00E+00
UBE2M	-1.18E+00	3.97E-21	TWISTNB	1.21E-01	1.00E+00	FAM71A	2.79E-01	1.00E+00
GEM	-5.97E+00	4.01E-21	WDR7	9.80E-02	1.00E+00	PEX3	8.98E-02	1.00E+00
TMSB10	-1.96E+00	4.04E-21	C2H6orf223	-2.85E-01	1.00E+00	RBL1	-1.02E-01	1.00E+00
JPH4	4.88E+00	4.06E-21	SMIM23	-2.30E-01	1.00E+00	PPT1	-6.58E-02	1.00E+00
PAX8	-2.79E+00	4.06E-21	JAK3	3.15E-01	1.00E+00	RANBP6	7.91E-02	1.00E+00
DROSHA	8.84E-01	4.10E-21	SRP68	5.13E-02	1.00E+00	B4GALNT4	7.65E-02	1.00E+00
GLI2	3.97E+00	4.14E-21	CIB4	-4.26E-01	1.00E+00	UBE2G2	5.83E-02	1.00E+00
SIM1	5.65E+00	4.18E-21	BAIAP2	5.06E-02	1.00E+00	ACOT12	-1.52E-01	1.00E+00
IFT52	-1.15E+00	4.27E-21	LOC112578899	3.34E-01	1.00E+00	SCYL3	-5.79E-02	1.00E+00
ACAD11	-1.60E+00	4.35E-21	CDON	-1.49E-01	1.00E+00	RXYLT1	-5.57E-02	1.00E+00
LOC112579082	3.77E+00	4.42E-21	RRM2	1.21E-01	1.00E+00	ZNF189	-5.94E-02	1.00E+00
LOC102401424	-4.27E+00	4.60E-21	ESPN	-1.06E-01	1.00E+00	KCNAB2	1.09E-01	1.00E+00
FAM43B	-6.64E+00	4.69E-21	ODF2L	1.07E-01	1.00E+00	LOC112579160	1.20E-01	1.00E+00
FCGBP	-4.67E+00	4.69E-21	RUSC1	-2.36E-01	1.00E+00	MAPK6	9.24E-02	1.00E+00
LINGO2	3.94E+00	4.71E-21	SUMO1	-6.32E-02	1.00E+00	LOC102411911	-6.22E-02	1.00E+00
PSD	-2.24E+00	4.71E-21	KCTD10	5.50E-02	1.00E+00	MYLIP	-7.21E-02	1.00E+00
GTF2IRD1	-1.51E+00	4.74E-21	MARCKSL1	-8.38E-02	1.00E+00	C22H18orf25	6.32E-02	1.00E+00
PRSS12	2.27E+00	4.81E-21	SLK	9.56E-02	1.00E+00	MAP6D1	8.90E-02	1.00E+00
CHD3	-4.14E+00	4.91E-21	LARGE2	-3.06E-01	1.00E+00	ABL2	-1.18E-01	1.00E+00
LOC112587770	5.30E+00	4.93E-21	SERBP1	-5.98E-02	1.00E+00	SMIM8	-1.04E-01	1.00E+00
SLF2	-1.18E+00	4.94E-21	LOC102391382	-1.71E-01	1.00E+00	SFSWAP	-5.29E-02	1.00E+00
IFT80	-1.80E+00	5.06E-21	LOC112586994	2.57E-01	1.00E+00	TBPL1	-6.21E-02	1.00E+00
RAPGEF6	1.24E+00	5.13E-21	EHBP1L1	1.27E-01	1.00E+00	FUT11	1.29E-01	1.00E+00
C24H7orf26	9.62E-01	5.14E-21	SFR1	-7.56E-02	1.00E+00	TRIP10	6.67E-02	1.00E+00
LOC112587920	-4.34E+00	5.20E-21	SH3D19	-8.70E-02	1.00E+00	SSSCA1	6.77E-02	1.00E+00
PNPLA8	9.34E-01	5.20E-21	LOC102404258	4.37E-01	1.00E+00	CLNS1A	6.13E-02	1.00E+00
FOXO1	-1.28E+00	5.24E-21	PPP1R1B	-1.14E-01	1.00E+00	ARMC4	9.12E-02	1.00E+00
BRD4	-9.19E-01	5.26E-21	AADACL3	-1.33E-01	1.00E+00	BECN1	-5.47E-02	1.00E+00
CNIH4	1.07E+00	5.26E-21	HMGN2	8.28E-02	1.00E+00	SLC25A23	-7.57E-02	1.00E+00
LOC102399924	1.46E+00	5.27E-21	PTP4A3	2.91E-01	1.00E+00	EIF3K	-7.69E-02	1.00E+00
PPP3CA	-1.39E+00	5.28E-21	SIN3B	6.49E-02	1.00E+00	CS	-1.53E-01	1.00E+00
LOC112577670	-3.07E+00	5.29E-21	ADAMTSL4	2.69E-01	1.00E+00	PDE4A	1.75E-01	1.00E+00
LCORL	-	5.30E-21	GLRX2	-2.78E-	1.00E+00	TADA3	6.03E-02	1.00E+00

	1.34E+00			01				
SSBP2	1.66E+00	5.36E-21	SNTB2	6.83E-02	1.00E+00	HMGN2	9.95E-02	1.00E+00
PELI3	2.78E+00	5.41E-21	LOC102412377	-2.77E-01	1.00E+00	HCST	-1.35E-01	1.00E+00
YES1	1.37E+00	5.42E-21	LOC102403870	8.01E-02	1.00E+00	PPM1B	-6.42E-02	1.00E+00
FRMD4B	2.36E+00	5.43E-21	STX5	6.79E-02	1.00E+00	SLC27A2	-9.85E-02	1.00E+00
CALCOCO2	9.66E-01	5.57E-21	SMARCC1	7.96E-02	1.00E+00	CREBL2	9.64E-02	1.00E+00
ZCCHC14	-1.60E+00	5.63E-21	SYNGR4	-1.86E-01	1.00E+00	PARD3	-5.59E-02	1.00E+00
LYPLA2	-1.04E+00	5.64E-21	MCRS1	6.48E-02	1.00E+00	LAMTOR5	6.79E-02	1.00E+00
LOC102392428	-5.86E+00	5.75E-21	NECTIN2	-3.77E-01	1.00E+00	ZBED9	-1.24E-01	1.00E+00
LOC112582377	5.70E+00	6.14E-21	TRMT1L	6.22E-02	1.00E+00	LOC102390265	-9.02E-02	1.00E+00
LOC112580624	-3.40E+00	6.28E-21	NDUFB4	-7.95E-02	1.00E+00	LTN1	9.06E-02	1.00E+00
FAM171A2	-2.98E+00	6.31E-21	SARS	-6.39E-02	1.00E+00	GBE1	6.81E-02	1.00E+00
UBA1	1.04E+00	6.35E-21	EGR3	6.08E-02	1.00E+00	PLEKHM3	-8.27E-02	1.00E+00
LOC112580883	4.99E+00	6.44E-21	LOC102410567	2.12E-01	1.00E+00	PDLIM2	1.36E-01	1.00E+00
TMEM158	-3.69E+00	6.52E-21	IBTK	7.59E-02	1.00E+00	MAP2K1	7.71E-02	1.00E+00
ALKBH3	-1.66E+00	6.57E-21	HSDL1	6.78E-02	1.00E+00	UCHL5	5.86E-02	1.00E+00
PP2D1	-2.36E+00	6.57E-21	LOC102397082	1.01E-01	1.00E+00	MAP9	-8.16E-02	1.00E+00
LOC102403423	2.48E+00	6.63E-21	SIK3	9.33E-02	1.00E+00	TMEM168	6.94E-02	1.00E+00
LOC102409533	-3.45E+00	6.67E-21	PAF1	-4.90E-02	1.00E+00	NMT1	5.58E-02	1.00E+00
PSIP1	1.06E+00	6.67E-21	TRIM24	-6.96E-02	1.00E+00	CEP104	-7.72E-02	1.00E+00
LOC112586460	4.85E+00	6.69E-21	PTAR1	-1.64E-01	1.00E+00	YIPF4	-1.11E-01	1.00E+00
RGS9	1.80E+00	6.71E-21	WDR33	5.02E-02	1.00E+00	NCF2	-2.53E-01	1.00E+00
CFAP300	1.34E+00	6.74E-21	VILL	3.91E-01	1.00E+00	EIF4EBP2	5.98E-02	1.00E+00
LANCL1	8.33E-01	6.79E-21	AHCYL1	-7.29E-02	1.00E+00	RRM1	-5.66E-02	1.00E+00
F5	-2.60E+00	6.84E-21	LARP4B	6.34E-02	1.00E+00	CRYL1	7.12E-02	1.00E+00
ZC3H12B	-4.35E+00	7.15E-21	LOC102404909	7.92E-02	1.00E+00	RPUSD3	-6.51E-02	1.00E+00
KCNE1	3.09E+00	7.19E-21	FKBP1A	-6.43E-02	1.00E+00	CILP2	1.43E-01	1.00E+00
ZNF623	1.35E+00	7.31E-21	UNCX	8.83E-02	1.00E+00	CMYA5	1.57E-01	1.00E+00
ARHGEF19	-2.51E+00	7.31E-21	GNG4	7.41E-02	1.00E+00	LOC112580307	-1.41E-01	1.00E+00
EPHA8	4.84E+00	7.40E-21	TXN2	-6.77E-02	1.00E+00	LOC112579236	-9.07E-02	1.00E+00
SNAP23	-1.07E+00	7.47E-21	TTI1	6.97E-02	1.00E+00	GNPTAB	-7.03E-02	1.00E+00
SYTL5	5.52E+00	7.53E-21	LNP1	8.04E-02	1.00E+00	CTTNBP2NL	-1.20E-01	1.00E+00
STAC2	-5.01E+00	7.69E-21	WWC3	-1.02E-01	1.00E+00	GCC2	-7.10E-02	1.00E+00
CARD14	2.98E+00	7.77E-21	LRRC45	8.37E-02	1.00E+00	TTC26	-7.68E-02	1.00E+00
MAP2	2.13E+00	7.79E-21	ATP6V1A	7.62E-02	1.00E+00	HEATR1	6.04E-02	1.00E+00

USP50	-4.12E+00	7.86E-21	TMEM190	-2.07E-01	1.00E+00	CHST8	-8.87E-02	1.00E+00
LOC112583954	-5.14E+00	7.96E-21	IFT122	9.60E-02	1.00E+00	CENPI	-1.07E-01	1.00E+00
TBCA	-1.52E+00	8.29E-21	SLC12A9	1.07E-01	1.00E+00	ZNF770	-9.64E-02	1.00E+00
TNFRSF19	-5.05E+00	8.35E-21	VPS13B	5.46E-02	1.00E+00	RPL23A	7.74E-02	1.00E+00
ARHGEF28	-1.09E+00	8.40E-21	KIF16B	6.92E-02	1.00E+00	P4HA1	6.34E-02	1.00E+00
LOC112584695	3.03E+00	8.45E-21	NOD1	-7.68E-02	1.00E+00	LOC102394580	-6.34E-02	1.00E+00
GAS2	2.22E+00	8.45E-21	P2RX4	-9.83E-02	1.00E+00	FUT4	-8.08E-02	1.00E+00
SETDB2	1.02E+00	8.54E-21	LOC102395770	6.02E-02	1.00E+00	XYLT2	-6.30E-02	1.00E+00
LRRC9	-2.42E+00	8.79E-21	LOC102416306	3.49E-01	1.00E+00	CFAP61	1.21E-01	1.00E+00
CORIN	2.23E+00	8.91E-21	PRKAR1A	5.11E-02	1.00E+00	RFX3	8.55E-02	1.00E+00
DNM3	1.72E+00	8.92E-21	NAA15	7.88E-02	1.00E+00	UFSP2	5.60E-02	1.00E+00
EMC7	-9.87E-01	9.00E-21	CD81	6.22E-02	1.00E+00	FPGT	-9.16E-02	1.00E+00
NAA10	-1.48E+00	9.27E-21	CCDC32	-7.10E-02	1.00E+00	LOC112585811	-1.42E-01	1.00E+00
LOC112587824	-5.33E+00	9.36E-21	SCRN2	1.52E-01	1.00E+00	MKNK1	-7.41E-02	1.00E+00
RARS2	1.17E+00	9.47E-21	ZNF436	5.98E-02	1.00E+00	DNASE1L1	-6.91E-02	1.00E+00
POLD4	-2.52E+00	9.64E-21	COL27A1	-2.48E-01	1.00E+00	RNF31	-5.54E-02	1.00E+00
PINK1	-1.04E+00	9.68E-21	BAK1	-1.26E-01	1.00E+00	LOC112587597	-1.63E-01	1.00E+00
TMEM225B	1.63E+00	9.70E-21	TRAF1	8.20E-02	1.00E+00	CLDND1	-6.63E-02	1.00E+00
ANGPT1	-4.49E+00	1.00E-20	RBMX2	-9.06E-02	1.00E+00	EEF2KMT	-7.68E-02	1.00E+00
CLDN6	4.99E+00	1.00E-20	BLOC1S6	1.66E-01	1.00E+00	KCTD8	9.66E-02	1.00E+00
COMMD3	-1.09E+00	1.00E-20	RPRM	-2.55E-01	1.00E+00	BCL9	7.00E-02	1.00E+00
RHBDD1	9.57E-01	1.02E-20	LOC102415279	2.83E-01	1.00E+00	LOC112578119	1.28E-01	1.00E+00
PCCA	-8.90E-01	1.02E-20	ELMOD3	1.50E-01	1.00E+00	SLC35D2	1.02E-01	1.00E+00
ADAMTS6	-3.15E+00	1.03E-20	SEC61A1	5.30E-02	1.00E+00	ZNF584	-7.93E-02	1.00E+00
LOC102414052	1.21E+00	1.05E-20	METTL15	-1.52E-01	1.00E+00	YTHDF1	-5.81E-02	1.00E+00
LOC102390045	-3.91E+00	1.06E-20	LOC102416288	-6.63E-02	1.00E+00	PINX1	7.03E-02	1.00E+00
RNF10	1.07E+00	1.06E-20	TRPM7	1.15E-01	1.00E+00	ZSWIM7	-7.84E-02	1.00E+00
PSMG4	-1.79E+00	1.06E-20	BCL9	1.24E-01	1.00E+00	SMAD4	-6.14E-02	1.00E+00
LOC112580272	5.97E+00	1.07E-20	SNX21	1.31E-01	1.00E+00	FH	5.77E-02	1.00E+00
LOC112585771	2.95E+00	1.08E-20	ADRA2C	-1.77E-01	1.00E+00	MECP2	-5.11E-02	1.00E+00
CHMP5	8.47E-01	1.08E-20	TXNL1	-5.18E-02	1.00E+00	CTU1	1.06E-01	1.00E+00
CD34	-3.86E+00	1.09E-20	CHST8	-3.25E-01	1.00E+00	EPRS	5.68E-02	1.00E+00
IWS1	8.06E-01	1.10E-20	UTP20	7.90E-02	1.00E+00	TRAF3IP1	-7.03E-02	1.00E+00
TNFRSF25	2.26E+00	1.10E-20	LOC112585664	-2.85E-01	1.00E+00	LOC102394850	-8.34E-02	1.00E+00

MRPL44	9.88E-01	1.13E-20	LOC112579148	-1.75E-01	1.00E+00	LOC102397670	-7.11E-02	1.00E+00
LOC112586469	3.97E+00	1.14E-20	GPATCH3	6.85E-02	1.00E+00	SOS2	7.63E-02	1.00E+00
AQP6	5.07E+00	1.14E-20	CCIN	1.34E-01	1.00E+00	GOLGA4	-6.85E-02	1.00E+00
PARD3B	1.02E+00	1.17E-20	WDFY3	9.89E-02	1.00E+00	LOC102407805	-8.02E-02	1.00E+00
C8B	5.03E+00	1.23E-20	MUT	7.01E-02	1.00E+00	NDUFB9	8.13E-02	1.00E+00
FAM205C	2.99E+00	1.24E-20	NDUFAF8	-9.83E-02	1.00E+00	EMP1	1.57E-01	1.00E+00
RAB32	1.29E+00	1.26E-20	ESCO1	1.00E-01	1.00E+00	ARHGEF10L	6.02E-02	1.00E+00
APELA	-4.90E+00	1.27E-20	ELF4	1.22E-01	1.00E+00	PTPN9	6.41E-02	1.00E+00
LOC112584971	7.73E+00	1.31E-20	MADCAM1	-1.16E-01	1.00E+00	GPN2	6.52E-02	1.00E+00
LOC102410932	3.76E+00	1.33E-20	RMDN3	6.22E-02	1.00E+00	HECTD4	7.50E-02	1.00E+00
CXXC4	1.78E+00	1.35E-20	CHTF18	-6.08E-02	1.00E+00	LRP1	-7.65E-02	1.00E+00
FMNL3	1.68E+00	1.36E-20	LOC102401601	7.69E-02	1.00E+00	LOC102397454	9.00E-02	1.00E+00
MRPL41	-1.67E+00	1.37E-20	ZDHHC6	-6.79E-02	1.00E+00	SRM	7.37E-02	1.00E+00
LOC112587829	-4.52E+00	1.40E-20	LOC112584448	-1.78E-01	1.00E+00	ANKDD1B	1.31E-01	1.00E+00
TSPAN11	-3.27E+00	1.41E-20	NR1D2	-2.96E-01	1.00E+00	STMP1	5.40E-02	1.00E+00
LOC102402159	-5.28E+00	1.42E-20	LOC112578157	3.62E-01	1.00E+00	SLC25A35	-1.19E-01	1.00E+00
PPARGC1B	-2.03E+00	1.42E-20	MCF2	-8.01E-02	1.00E+00	ATF5	8.12E-02	1.00E+00
MYPN	4.70E+00	1.44E-20	MATN3	-2.35E-01	1.00E+00	EIF3L	6.29E-02	1.00E+00
LOC112578899	6.03E+00	1.45E-20	PAK1IP1	-1.40E-01	1.00E+00	WWP2	-5.42E-02	1.00E+00
PRR16	4.09E+00	1.45E-20	PTPN13	5.97E-02	1.00E+00	NUP85	-6.39E-02	1.00E+00
TRABD	-1.21E+00	1.45E-20	OXSR1	7.36E-02	1.00E+00	NCKAP5L	5.94E-02	1.00E+00
EVI5	-1.41E+00	1.48E-20	DPPA5	-5.87E-02	1.00E+00	NAPA	-6.23E-02	1.00E+00
IRGQ	-1.28E+00	1.50E-20	ENO1	8.15E-02	1.00E+00	ZCCHC10	7.43E-02	1.00E+00
P2RY1	2.83E+00	1.51E-20	SURF6	-9.02E-02	1.00E+00	HINT3	-6.16E-02	1.00E+00
PFN2	-9.22E-01	1.51E-20	PLSCR4	-4.12E-01	1.00E+00	MIER3	-8.25E-02	1.00E+00
BMP2K	-1.06E+00	1.52E-20	LOC112580132	1.24E-01	1.00E+00	SAT1	-9.05E-02	1.00E+00
NPAS2	-1.61E+00	1.53E-20	LOC102407694	1.83E-01	1.00E+00	ESCO1	7.48E-02	1.00E+00
LRRC3	-3.83E+00	1.53E-20	LOC102411461	5.86E-02	1.00E+00	PRDX1	7.70E-02	1.00E+00
ACTR3	-1.00E+00	1.55E-20	ULK3	1.29E-01	1.00E+00	HELQ	-6.90E-02	1.00E+00
NFATC4	1.09E+00	1.57E-20	MGAT5B	5.20E-02	1.00E+00	RIC3	9.39E-02	1.00E+00
LOC102412869	-3.78E+00	1.59E-20	ACP1	-5.40E-02	1.00E+00	KMT2B	-5.60E-02	1.00E+00
TRPV2	-4.03E+00	1.62E-20	TTF2	-5.57E-02	1.00E+00	LOC102399825	-9.37E-02	1.00E+00
ANO3	-4.16E+00	1.63E-20	TMEM161A	-1.58E-01	1.00E+00	ZNF446	-6.83E-02	1.00E+00
VMP1	-1.14E+00	1.64E-20	KLHL36	5.36E-02	1.00E+00	TNRC6B	-6.60E-02	1.00E+00

SLC35B3	-1.12E+00	1.67E-20	PEX11B	1.04E-01	1.00E+00	C2H2orf69	-8.32E-02	1.00E+00
LOC102416629	2.96E+00	1.69E-20	CCNE1	-8.21E-02	1.00E+00	KLHDC9	-1.27E-01	1.00E+00
TADA3	9.84E-01	1.70E-20	RAB5A	5.13E-02	1.00E+00	NUDCD3	5.50E-02	1.00E+00
LOC102401766	-1.75E+00	1.75E-20	C23H10orf143	-8.97E-02	1.00E+00	LOC102414221	1.70E-01	1.00E+00
GZMM	3.44E+00	1.79E-20	PCNT	-5.96E-02	1.00E+00	NELFE	7.00E-02	1.00E+00
PPP1R2	1.10E+00	1.80E-20	PDAP1	-5.81E-02	1.00E+00	KLF15	-7.18E-02	1.00E+00
CERS1	-1.60E+00	1.86E-20	VAPA	-4.71E-02	1.00E+00	GMDS	-7.33E-02	1.00E+00
ACSL1	-2.28E+00	1.90E-20	MYOZ1	-1.04E-01	1.00E+00	TMED2	-5.70E-02	1.00E+00
CTSH	-4.15E+00	1.91E-20	DPCD	-7.70E-02	1.00E+00	REELD1	-1.32E-01	1.00E+00
TMEM250	9.38E-01	1.92E-20	UGT8	1.81E-01	1.00E+00	CA14	-1.16E-01	1.00E+00
LEKR1	-2.17E+00	1.92E-20	EHBP1	5.50E-02	1.00E+00	PRTFDC1	-7.34E-02	1.00E+00
KMT2D	-2.09E+00	1.93E-20	GPAT2	7.41E-02	1.00E+00	OSBPL1A	6.72E-02	1.00E+00
CDKN1A	-3.51E+00	1.98E-20	GMCL1	7.97E-02	1.00E+00	C1H21orf91	1.04E-01	1.00E+00
AKIRIN1	-1.19E+00	1.99E-20	ATG7	-6.56E-02	1.00E+00	BCL2	1.15E-01	1.00E+00
LOC112578234	2.92E+00	2.05E-20	ADAMTS15	3.08E-01	1.00E+00	MAML1	-7.17E-02	1.00E+00
LOC112578420	8.11E+00	2.06E-20	RBM28	7.68E-02	1.00E+00	LOC102409132	-6.40E-02	1.00E+00
COL4A6	-2.00E+00	2.11E-20	COPS3	4.96E-02	1.00E+00	NFE2L1	-7.73E-02	1.00E+00
LOC102392405	-1.71E+00	2.13E-20	LOC112584668	-2.60E-01	1.00E+00	ZYG11B	7.29E-02	1.00E+00
VPS37D	1.40E+00	2.13E-20	ITSN2	7.49E-02	1.00E+00	LOC102407787	-9.38E-02	1.00E+00
NOL4	4.95E+00	2.13E-20	PPFIBP2	-5.27E-02	1.00E+00	LOC102402714	-8.76E-02	1.00E+00
RHOV	-3.57E+00	2.16E-20	LOC102402624	6.39E-02	1.00E+00	DTNBP1	7.88E-02	1.00E+00
PRKCE	-1.32E+00	2.16E-20	LOC102411593	5.15E-02	1.00E+00	MOB2	7.43E-02	1.00E+00
POLR2C	8.55E-01	2.19E-20	DOCK7	-1.14E-01	1.00E+00	PSMF1	-5.63E-02	1.00E+00
PIGH	-2.46E+00	2.20E-20	ATP5MF	-8.20E-02	1.00E+00	NDUFB11	7.80E-02	1.00E+00
CARHSP1	-1.49E+00	2.26E-20	CEP44	8.57E-02	1.00E+00	FNDC5	-1.24E-01	1.00E+00
EMILIN2	3.02E+00	2.26E-20	DNAJC17	9.05E-02	1.00E+00	ADPGK	-5.63E-02	1.00E+00
NUP98	1.14E+00	2.30E-20	CYTH2	-4.99E-02	1.00E+00	LOC102412079	7.86E-02	1.00E+00
TMEM59L	-3.03E+00	2.30E-20	SCG5	-1.06E-01	1.00E+00	TJAP1	-6.05E-02	1.00E+00
ARL5A	-1.31E+00	2.34E-20	C4H1orf131	1.08E-01	1.00E+00	CEP126	8.00E-02	1.00E+00
NKPD1	-2.14E+00	2.35E-20	SH3GLB2	-7.33E-02	1.00E+00	DOLK	-6.87E-02	1.00E+00
TMEM39A	8.75E-01	2.41E-20	SH2D7	-1.53E-01	1.00E+00	LOC102408963	-2.40E-01	1.00E+00
FAM222A	2.38E+00	2.43E-20	FOXO4	9.59E-02	1.00E+00	LOC102405577	-6.75E-02	1.00E+00
M6PR	-8.72E-	2.44E-20	TOPBP1	7.80E-02	1.00E+00	ARRB1	-1.30E-	1.00E+00

	01						01	
SRRM4	6.31E+00	2.45E-20	SLC36A4	1.80E-01	1.00E+00	HMBS	7.39E-02	1.00E+00
ANO5	6.30E+00	2.48E-20	MRPL27	-1.26E-01	1.00E+00	LOC102405871	7.15E-02	1.00E+00
RAB6B	1.17E+00	2.52E-20	DLL3	-1.03E-01	1.00E+00	MRPL35	-6.99E-02	1.00E+00
LOC102409830	7.33E+00	2.56E-20	LOC102414896	-2.36E-01	1.00E+00	MYO10	6.42E-02	1.00E+00
LOC102408518	-3.90E+00	2.57E-20	SHANK1	2.67E-01	1.00E+00	LOC112582960	-1.28E-01	1.00E+00
ACVRL1	2.47E+00	2.63E-20	RBM34	-9.00E-02	1.00E+00	ZNF319	8.85E-02	1.00E+00
MSC	-8.06E+00	2.65E-20	DOCK1	7.55E-02	1.00E+00	TPP1	-6.27E-02	1.00E+00
PEBP4	3.48E+00	2.67E-20	RRAGA	4.88E-02	1.00E+00	RANGRF	-1.03E-01	1.00E+00
DMXL2	1.20E+00	2.67E-20	ELP2	6.36E-02	1.00E+00	LOC112583737	1.25E-01	1.00E+00
LOC102416136	-3.62E+00	2.67E-20	LOC102410920	9.86E-02	1.00E+00	CCNQ	-6.96E-02	1.00E+00
DNMT3L	-6.82E+00	2.70E-20	EPB41L1	-1.96E-01	1.00E+00	LOC102390198	1.44E-01	1.00E+00
ATP5F1E	-1.53E+00	2.85E-20	NRIP3	6.84E-02	1.00E+00	PPOX	-6.24E-02	1.00E+00
PIM2	-1.70E+00	2.85E-20	GPR62	-3.37E-01	1.00E+00	CAPN10	-7.10E-02	1.00E+00
RAP1A	8.68E-01	2.88E-20	CIAO2B	8.29E-02	1.00E+00	STK40	6.75E-02	1.00E+00
CTDSPL2	-1.11E+00	2.93E-20	SF3B2	-5.00E-02	1.00E+00	GFER	8.37E-02	1.00E+00
PCSK5	1.41E+00	2.95E-20	PLA2G6	8.09E-02	1.00E+00	FAM120C	-6.69E-02	1.00E+00
GKAP1	-1.65E+00	3.00E-20	DSEL	1.66E-01	1.00E+00	GRSF1	-5.88E-02	1.00E+00
CDKN2B	-4.60E+00	3.02E-20	LOC102397580	6.13E-02	1.00E+00	LOC102409101	-5.66E-02	1.00E+00
MOSMO	-2.40E+00	3.03E-20	WDFY1	-5.56E-02	1.00E+00	PIMREG	-8.70E-02	1.00E+00
ISM1	2.10E+00	3.05E-20	POGZ	7.34E-02	1.00E+00	ACAP1	-7.50E-02	1.00E+00
LOC112581779	-2.56E+00	3.06E-20	CSKMT	-1.28E-01	1.00E+00	FDXACB1	-1.11E-01	1.00E+00
PPP1R3E	-2.50E+00	3.09E-20	STARD3	-1.42E-01	1.00E+00	MEI4	9.76E-02	1.00E+00
RALGPS2	-1.17E+00	3.20E-20	SEPHS1	1.07E-01	1.00E+00	COPS7A	-6.02E-02	1.00E+00
PDS5A	-1.05E+00	3.20E-20	LOC112587806	1.62E-01	1.00E+00	LOC102416218	-7.92E-02	1.00E+00
SLC17A8	4.34E+00	3.21E-20	CRYBG3	-5.78E-02	1.00E+00	CCDC40	1.11E-01	1.00E+00
MSH2	8.63E-01	3.21E-20	TMEM107	-6.68E-02	1.00E+00	PMPCA	6.91E-02	1.00E+00
UBXN6	1.41E+00	3.22E-20	TXNDC5	-7.43E-02	1.00E+00	ZNF354A	-6.15E-02	1.00E+00
CDC23	-1.18E+00	3.24E-20	MTO1	-7.67E-02	1.00E+00	LOC112581879	-9.62E-02	1.00E+00
SLCO2B1	-4.44E+00	3.25E-20	MYO6	9.03E-02	1.00E+00	TCN2	-6.13E-02	1.00E+00
CERS3	5.55E+00	3.26E-20	TGFBR3L	-2.73E-01	1.00E+00	SSBP1	-6.18E-02	1.00E+00
TARDBP	-1.01E+00	3.33E-20	UFC1	-8.59E-02	1.00E+00	GPR63	1.06E-01	1.00E+00
MRPL55	-1.53E+00	3.37E-20	C8B	2.98E-01	1.00E+00	DSE	-5.56E-02	1.00E+00
MYD88	1.05E+00	3.38E-20	CTH	-7.68E-	1.00E+00	RUVBL1	7.52E-02	1.00E+00

				02				
ND3	-2.31E+00	3.40E-20	FBXO8	-8.81E-02	1.00E+00	GLI4	8.42E-02	1.00E+00
TNFRSF18	-4.88E+00	3.40E-20	USP39	-8.43E-02	1.00E+00	KDM3A	-6.03E-02	1.00E+00
ERCC8	-1.47E+00	3.44E-20	LOC102397476	1.92E-01	1.00E+00	NABP2	-6.96E-02	1.00E+00
ARHGAP17	-9.97E-01	3.45E-20	ERMAP	-1.36E-01	1.00E+00	TBC1D20	-5.32E-02	1.00E+00
CDC45	-1.26E+00	3.45E-20	TAF1C	6.36E-02	1.00E+00	HOXC9	1.34E-01	1.00E+00
TXN2	-1.09E+00	3.50E-20	USP24	1.19E-01	1.00E+00	CALHM2	-1.16E-01	1.00E+00
STYX	-1.69E+00	3.51E-20	BMP2K	8.31E-02	1.00E+00	LOC102389337	-1.40E-01	1.00E+00
VASH1	-1.84E+00	3.55E-20	SEMA3B	-1.91E-01	1.00E+00	ZNF774	1.18E-01	1.00E+00
PKP3	5.34E+00	3.57E-20	CDCA2	-7.21E-02	1.00E+00	ASB11	8.15E-02	1.00E+00
RNF138	-1.56E+00	3.59E-20	LOC102391307	1.05E-01	1.00E+00	TICAM1	1.06E-01	1.00E+00
GSTZ1	-1.77E+00	3.59E-20	FAM129B	5.46E-02	1.00E+00	TMEM69	-6.80E-02	1.00E+00
CIAO2B	-1.26E+00	3.63E-20	CDKL3	3.02E-01	1.00E+00	MYO1D	1.02E-01	1.00E+00
TMEM39B	9.13E-01	3.64E-20	ATP13A1	8.34E-02	1.00E+00	ATP8B2	8.22E-02	1.00E+00
STK10	-1.95E+00	3.64E-20	NSUN4	8.84E-02	1.00E+00	RASSF1	7.03E-02	1.00E+00
GNB2	-1.25E+00	3.64E-20	MIS18A	-5.11E-02	1.00E+00	BAG1	5.50E-02	1.00E+00
PFN4	3.24E+00	3.75E-20	HABP4	7.23E-02	1.00E+00	INPP4B	9.16E-02	1.00E+00
LOC102410728	-4.35E+00	3.75E-20	MED23	1.51E-01	1.00E+00	DDX55	-6.31E-02	1.00E+00
TRIB3	-3.49E+00	3.77E-20	ACTA2	-2.84E-01	1.00E+00	LOC102408914	-1.21E-01	1.00E+00
TRAF5	3.62E+00	3.83E-20	MYO5A	8.07E-02	1.00E+00	CCDC117	-5.70E-02	1.00E+00
IRAK4	1.13E+00	3.91E-20	VIRMA	7.33E-02	1.00E+00	HIPK3	-6.75E-02	1.00E+00
LAMTOR2	-1.84E+00	3.98E-20	EXOG	-6.51E-02	1.00E+00	ACBD5	-9.54E-02	1.00E+00
IL20RB	4.85E+00	4.03E-20	VPS13C	1.40E-01	1.00E+00	TBC1D10A	7.19E-02	1.00E+00
CA5A	-4.81E+00	4.13E-20	LOC112579922	2.87E-01	1.00E+00	TMEM98	-6.70E-02	1.00E+00
ZNF609	-1.36E+00	4.15E-20	C2CD2L	-9.80E-02	1.00E+00	SIK1	1.34E-01	1.00E+00
LOC112584572	-5.75E+00	4.27E-20	QRSL1	7.24E-02	1.00E+00	QTRT2	-7.11E-02	1.00E+00
GK5	-1.61E+00	4.33E-20	DCTN1	5.54E-02	1.00E+00	COL4A5	-9.00E-02	1.00E+00
ACOXL	3.43E+00	4.35E-20	CLCF1	1.60E-01	1.00E+00	SH2D3C	-1.25E-01	1.00E+00
TPD52	7.95E-01	4.40E-20	RFC1	5.36E-02	1.00E+00	SAMD15	-1.29E-01	1.00E+00
LOC102399489	1.13E+00	4.40E-20	AP3B1	5.35E-02	1.00E+00	UNC13B	5.43E-02	1.00E+00
LOC112585046	5.59E+00	4.42E-20	MND1	-7.75E-02	1.00E+00	ENDOG	-8.96E-02	1.00E+00
PRKG2	4.68E+00	4.46E-20	IGFBP6	3.16E-01	1.00E+00	DKKL1	-9.76E-02	1.00E+00
ZNF569	-1.18E+00	4.57E-20	KYAT3	-9.67E-02	1.00E+00	FBXL8	1.10E-01	1.00E+00
LOC102390842	-1.08E+00	4.59E-20	LOC102409297	3.24E-01	1.00E+00	USP5	-5.73E-02	1.00E+00

NDUFA3	-1.56E+00	4.60E-20	GTF3A	1.31E-01	1.00E+00	PLEKHA3	-7.27E-02	1.00E+00
PARM1	-4.92E+00	4.64E-20	NUDCD2	-9.28E-02	1.00E+00	SRP68	-5.59E-02	1.00E+00
LOC112587016	-3.65E+00	4.66E-20	PIGZ	-1.54E-01	1.00E+00	PFKL	7.45E-02	1.00E+00
ZC3H8	8.87E-01	4.68E-20	MRPL13	-7.13E-02	1.00E+00	HNRNPF	-6.44E-02	1.00E+00
FOXM1	1.34E+00	4.70E-20	MRPL51	-7.22E-02	1.00E+00	CAPZA1	5.48E-02	1.00E+00
LOC112585722	3.77E+00	4.70E-20	SLC20A1	6.65E-02	1.00E+00	LYRM9	-6.31E-02	1.00E+00
GPM6A	-4.79E+00	4.87E-20	FAM83F	-1.59E-01	1.00E+00	LOC102394666	8.35E-02	1.00E+00
LOC102410270	2.03E+00	4.98E-20	BICRAL	9.62E-02	1.00E+00	PSMA7	7.78E-02	1.00E+00
AGAP1	1.09E+00	4.98E-20	C7H4orf36	-1.58E-01	1.00E+00	LIG4	9.82E-02	1.00E+00
KCNV2	5.22E+00	5.03E-20	KCNAB2	-2.54E-01	1.00E+00	IGFBP4	1.24E-01	1.00E+00
LOC102410583	-1.27E+00	5.11E-20	REL	-1.43E-01	1.00E+00	CTDSPL	6.09E-02	1.00E+00
ALDH5A1	1.58E+00	5.13E-20	MRAP2	-1.07E-01	1.00E+00	NAT9	6.84E-02	1.00E+00
FOS	-6.23E+00	5.25E-20	CHL1	6.40E-02	1.00E+00	MMAB	-9.20E-02	1.00E+00
GNG2	-2.22E+00	5.26E-20	ARPC5L	-5.91E-02	1.00E+00	LOC112581797	1.31E-01	1.00E+00
ME1	8.07E-01	5.30E-20	FGF5	-2.00E-01	1.00E+00	LSM2	-6.23E-02	1.00E+00
LOC112587900	5.06E+00	5.32E-20	MCM3AP	7.19E-02	1.00E+00	TRNAU1AP	-6.25E-02	1.00E+00
TXNDC16	1.85E+00	5.36E-20	ZNF713	-1.24E-01	1.00E+00	DDX23	-5.57E-02	1.00E+00
MRPL32	1.05E+00	5.39E-20	BMPR2	1.21E-01	1.00E+00	FAM107B	-5.70E-02	1.00E+00
LOC102394761	-4.16E+00	5.42E-20	LRSAM1	-8.15E-02	1.00E+00	TLDC1	-6.54E-02	1.00E+00
GLIS3	3.53E+00	5.49E-20	GSTO2	-2.65E-01	1.00E+00	PDE6G	-1.39E-01	1.00E+00
SH3BP5L	9.50E-01	5.55E-20	PIP5K1B	-5.90E-02	1.00E+00	LOC112581186	1.32E-01	1.00E+00
LOC112580602	4.21E+00	5.56E-20	RNF141	7.80E-02	1.00E+00	METTL1	7.09E-02	1.00E+00
KNL1	1.71E+00	5.61E-20	ACD	5.11E-02	1.00E+00	AUNIP	-8.58E-02	1.00E+00
MKRN1	-1.20E+00	5.63E-20	DDR1	6.72E-02	1.00E+00	NDST2	-5.92E-02	1.00E+00
MIGA2	8.98E-01	5.65E-20	CHTF8	-7.08E-02	1.00E+00	CFAP52	-1.42E-01	1.00E+00
TMEM258	-1.47E+00	5.69E-20	LOC102396145	-4.10E-01	1.00E+00	MRPS28	-6.97E-02	1.00E+00
CSGALNACT2	-1.39E+00	5.74E-20	B4GALT6	1.19E-01	1.00E+00	LRP4	-6.36E-02	1.00E+00
LOC102415619	-1.42E+00	5.75E-20	NTNG2	2.71E-01	1.00E+00	ZC3H12C	-7.50E-02	1.00E+00
COPG2	7.84E-01	5.78E-20	LOC112588008	1.08E-01	1.00E+00	PABPC5	-1.43E-01	1.00E+00
LOC102408422	-2.73E+00	5.80E-20	SH3BP2	1.96E-01	1.00E+00	TPRA1	-6.51E-02	1.00E+00
RHOJ	3.93E+00	5.80E-20	HCLS1	-3.36E-01	1.00E+00	SFXN5	-8.28E-02	1.00E+00
DHRS7B	-1.47E+00	6.06E-20	MAPK8	9.94E-02	1.00E+00	MRPS17	9.19E-02	1.00E+00
LTBP1	-1.21E+00	6.10E-20	C15H8orf37	-1.93E-01	1.00E+00	CHIC2	6.59E-02	1.00E+00

LOC112580156	-1.58E+00	6.31E-20	ALG1	1.11E-01	1.00E+00	KHDRBS3	1.21E-01	1.00E+00
NAPB	-1.55E+00	6.33E-20	LOC102393242	-1.91E-01	1.00E+00	LOC102405308	1.91E-01	1.00E+00
SLC51A	3.80E+00	6.52E-20	SPIRE1	-6.92E-02	1.00E+00	PRKAB1	5.44E-02	1.00E+00
TM4SF1	-7.22E+00	6.55E-20	MTPN	5.09E-02	1.00E+00	LOC112580268	1.30E-01	1.00E+00
SLC1A1	3.68E+00	6.64E-20	C9H19orf53	-7.99E-02	1.00E+00	UEVLD	-7.61E-02	1.00E+00
SLC2A11	-4.12E+00	6.69E-20	TOPAZ1	-1.05E-01	1.00E+00	AKAP12	6.99E-02	1.00E+00
TULP4	1.44E+00	6.69E-20	TRDN	3.30E-01	1.00E+00	LOC102389879	-1.25E-01	1.00E+00
P2RX3	4.14E+00	6.84E-20	DEAF1	-9.17E-02	1.00E+00	TSPYL5	-5.29E-02	1.00E+00
ZNF496	-1.03E+00	6.86E-20	NDFIP1	-5.06E-02	1.00E+00	FAM214B	7.56E-02	1.00E+00
GALNT12	-1.86E+00	6.87E-20	STIMATE	6.08E-02	1.00E+00	MADD	-5.31E-02	1.00E+00
NOP9	9.75E-01	7.00E-20	DBF4	-6.99E-02	1.00E+00	NFE2L2	5.99E-02	1.00E+00
KLF17	6.61E+00	7.00E-20	SIPA1	-7.18E-02	1.00E+00	EPM2A	1.07E-01	1.00E+00
CNOT1	1.20E+00	7.02E-20	CLUAP1	5.48E-02	1.00E+00	ZSCAN2	6.27E-02	1.00E+00
UTP20	1.05E+00	7.06E-20	APOLD1	2.55E-01	1.00E+00	KLHDC10	5.10E-02	1.00E+00
COPS4	9.17E-01	7.33E-20	SLC10A6	2.76E-01	1.00E+00	MOSMO	-9.83E-02	1.00E+00
TMX1	-9.06E-01	7.34E-20	SRM	1.03E-01	1.00E+00	CBX1	5.24E-02	1.00E+00
ZAN	-4.34E+00	7.42E-20	TMEM156	-3.60E-01	1.00E+00	PIGX	7.57E-02	1.00E+00
ACADSB	-1.06E+00	7.63E-20	SCARB2	6.42E-02	1.00E+00	DENND4A	-6.60E-02	1.00E+00
NSDHL	1.15E+00	7.63E-20	TUBGCP6	9.97E-02	1.00E+00	NBEA	9.21E-02	1.00E+00
QPRT	-6.17E+00	7.66E-20	IFIT3	-1.43E-01	1.00E+00	DUSP28	-7.28E-02	1.00E+00
EFEMP1	-1.76E+00	7.66E-20	MOGAT1	6.30E-02	1.00E+00	MATK	-1.73E-01	1.00E+00
TBX4	3.99E+00	7.70E-20	LRRC58	-1.65E-01	1.00E+00	GNG11	1.53E-01	1.00E+00
IL2RB	-4.25E+00	7.84E-20	AGAP1	5.45E-02	1.00E+00	RBM47	1.10E-01	1.00E+00
TPH1	4.74E+00	8.00E-20	ZC2HC1C	-1.00E-01	1.00E+00	POLI	-5.88E-02	1.00E+00
LOC112579939	-4.22E+00	8.09E-20	HERC4	7.37E-02	1.00E+00	TUBGCP2	-6.67E-02	1.00E+00
MOSPD1	1.30E+00	8.21E-20	PDS5A	8.50E-02	1.00E+00	MSRB1	8.16E-02	1.00E+00
SPRED1	-1.63E+00	8.47E-20	PPP1R10	5.62E-02	1.00E+00	LOC102400351	-1.98E-01	1.00E+00
ETAA1	-9.63E-01	8.51E-20	LOC112581146	-2.42E-01	1.00E+00	NDUFA10	7.43E-02	1.00E+00
SRP14	-1.34E+00	8.79E-20	GZMA	3.62E-01	1.00E+00	LOC102398867	-1.48E-01	1.00E+00
MATN3	3.11E+00	8.79E-20	LOC102394519	-3.25E-01	1.00E+00	TPST2	7.06E-02	1.00E+00
DCP1A	-1.37E+00	8.81E-20	VAMP4	9.32E-02	1.00E+00	SHARPIN	7.30E-02	1.00E+00
LOC112580601	5.62E+00	8.82E-20	ARAP1	1.19E-01	1.00E+00	TAOK3	-5.99E-02	1.00E+00
SYNGR4	2.59E+00	8.86E-20	PPP4R4	7.74E-02	1.00E+00	LOC102395684	1.10E-01	1.00E+00
NKAIN2	4.81E+00	8.91E-20	LOC112583752	-2.96E-01	1.00E+00	LOC102404275	1.40E-01	1.00E+00

CERS4	3.19E+00	8.92E-20	CHRNA5	-1.15E-01	1.00E+00	NR2C2AP	9.23E-02	1.00E+00
ZKSCAN4	-2.86E+00	9.30E-20	ADAMTS19	-6.40E-02	1.00E+00	FBXO11	6.66E-02	1.00E+00
DMTN	-2.98E+00	9.63E-20	APPL2	-5.80E-02	1.00E+00	GPR75	1.00E-01	1.00E+00
VIRMA	-9.02E-01	9.73E-20	FBXL16	-2.17E-01	1.00E+00	HADHB	5.62E-02	1.00E+00
CACNA1D	1.98E+00	9.87E-20	DICER1	8.18E-02	1.00E+00	FXR2	4.93E-02	1.00E+00
TMEM255B	-5.55E+00	9.87E-20	LOC102395897	-1.05E-01	1.00E+00	USP13	-1.06E-01	1.00E+00
NUP214	9.11E-01	9.97E-20	GFER	-8.67E-02	1.00E+00	APOD	-1.34E-01	1.00E+00
CCDC121	1.69E+00	1.01E-19	SLC35A3	1.58E-01	1.00E+00	ARRDC3	-9.69E-02	1.00E+00
E2F3	-2.05E+00	1.01E-19	RWDD2B	-6.47E-02	1.00E+00	PSRC1	-8.12E-02	1.00E+00
LOC112586240	4.53E+00	1.02E-19	DNAJB14	1.38E-01	1.00E+00	FARP2	-6.69E-02	1.00E+00
NIPA2	-9.68E-01	1.04E-19	WWP2	6.64E-02	1.00E+00	RRM2	-7.13E-02	1.00E+00
ZNF572	-1.02E+00	1.05E-19	PDLIM2	7.18E-02	1.00E+00	PATJ	-8.04E-02	1.00E+00
ST6GALNAC3	-1.93E+00	1.06E-19	SAXO1	5.93E-02	1.00E+00	DCDC2C	-1.45E-01	1.00E+00
NDUFAF1	-1.27E+00	1.09E-19	MED22	-5.33E-02	1.00E+00	MRGBP	-6.54E-02	1.00E+00
MLF2	-1.01E+00	1.10E-19	BCL2L2	8.68E-02	1.00E+00	PPP1CB	6.34E-02	1.00E+00
ARHGAP36	-6.97E+00	1.13E-19	LRCH2	3.76E-01	1.00E+00	ANO7	-1.02E-01	1.00E+00
CFAP69	-2.22E+00	1.13E-19	UBE2C	5.17E-02	1.00E+00	DNPH1	9.56E-02	1.00E+00
REEP6	2.86E+00	1.13E-19	IL13RA1	6.27E-02	1.00E+00	MOK	-8.83E-02	1.00E+00
GUCA1C	4.24E+00	1.15E-19	ZIC3	1.48E-01	1.00E+00	CIP2A	-7.03E-02	1.00E+00
LOC112580129	5.11E+00	1.15E-19	CDK17	-7.92E-02	1.00E+00	PPP1R1A	-1.07E-01	1.00E+00
KHSRP	1.16E+00	1.19E-19	MAL2	7.69E-02	1.00E+00	KPNB1	-7.43E-02	1.00E+00
TMCO5A	4.87E+00	1.20E-19	LOC102399157	-1.51E-01	1.00E+00	FN1	1.40E-01	1.00E+00
FANCM	1.25E+00	1.21E-19	KCTD8	-3.39E-01	1.00E+00	NDUFAF3	-8.34E-02	1.00E+00
IGSF1	-4.24E+00	1.21E-19	TBCA	-7.68E-02	1.00E+00	SUV39H2	-6.57E-02	1.00E+00
BUD13	8.27E-01	1.22E-19	ITPR3	1.29E-01	1.00E+00	FASTKD2	-5.62E-02	1.00E+00
PHLPP1	9.67E-01	1.24E-19	SLC25A32	-7.22E-02	1.00E+00	LOC102409148	6.79E-02	1.00E+00
SPAG4	2.46E+00	1.33E-19	PRSS56	-7.80E-02	1.00E+00	LEMD2	5.81E-02	1.00E+00
BARX1	2.48E+00	1.33E-19	LOC102415015	1.39E-01	1.00E+00	SRRD	-1.17E-01	1.00E+00
NFIB	2.69E+00	1.34E-19	BANF1	-5.32E-02	1.00E+00	ANKRD54	-5.99E-02	1.00E+00
CDC7	9.33E-01	1.37E-19	LOC102408590	3.29E-01	1.00E+00	RTCB	5.02E-02	1.00E+00
RND3	-6.75E+00	1.37E-19	DHRS11	1.33E-01	1.00E+00	VKORC1L1	-5.61E-02	1.00E+00
LOC102404358	-1.26E+00	1.39E-19	FAM241A	-2.95E-01	1.00E+00	FBRSL1	-5.46E-02	1.00E+00
SELENOT	-8.70E-01	1.41E-19	MRPL24	-1.23E-01	1.00E+00	TMEM131L	5.78E-02	1.00E+00

LOC102413264	-1.18E+00	1.51E-19	NUFIP1	5.21E-02	1.00E+00	LARP1	-5.80E-02	1.00E+00
SNX1	-7.51E-01	1.52E-19	DRG1	5.41E-02	1.00E+00	PIGP	-8.34E-02	1.00E+00
GJB1	3.70E+00	1.52E-19	NRG3	-1.03E-01	1.00E+00	DCAF16	1.13E-01	1.00E+00
ASH1L	-1.42E+00	1.56E-19	LOC102404490	5.69E-02	1.00E+00	RFC4	-7.70E-02	1.00E+00
OLFM1	5.32E+00	1.57E-19	LYSMD3	-1.38E-01	1.00E+00	PPP6R1	-5.88E-02	1.00E+00
DDX21	-9.83E-01	1.61E-19	CAPN10	8.29E-02	1.00E+00	FGR	-2.22E-01	1.00E+00
DGKA	-1.02E+00	1.61E-19	SPOP	4.63E-02	1.00E+00	CSNK1A1	-5.98E-02	1.00E+00
PDRG1	1.07E+00	1.61E-19	ADAMTSL5	-7.29E-02	1.00E+00	ANAPC5	-6.56E-02	1.00E+00
LOC112579153	7.79E+00	1.63E-19	TBCE	-7.34E-02	1.00E+00	TRPC4AP	-5.25E-02	1.00E+00
BEST1	3.02E+00	1.63E-19	FAM83D	5.26E-02	1.00E+00	ABCC5	-6.02E-02	1.00E+00
SNTN	-4.72E+00	1.65E-19	HTATSF1	6.64E-02	1.00E+00	SGPL1	1.22E-01	1.00E+00
CREB3	-1.23E+00	1.65E-19	SUGP1	-9.61E-02	1.00E+00	SOCS4	-6.18E-02	1.00E+00
TBX5	3.09E+00	1.65E-19	MYOC	2.03E-01	1.00E+00	SEC24B	6.72E-02	1.00E+00
LOC102406721	5.51E+00	1.68E-19	TIMM8B	-9.78E-02	1.00E+00	TRMT1L	-5.37E-02	1.00E+00
AMFR	-1.13E+00	1.70E-19	R3HCC1L	8.33E-02	1.00E+00	ATG9A	5.42E-02	1.00E+00
ADAP2	-5.19E+00	1.71E-19	RAI2	-1.66E-01	1.00E+00	ARL14EP	-6.29E-02	1.00E+00
ATP6V1H	8.04E-01	1.72E-19	LOC112587318	4.45E-01	1.00E+00	KCNJ6	6.03E-02	1.00E+00
ATG7	9.25E-01	1.74E-19	PTBP1	5.72E-02	1.00E+00	SPTAN1	6.31E-02	1.00E+00
UBL5	-1.49E+00	1.74E-19	KLK15	-3.91E-01	1.00E+00	HSPG2	-7.83E-02	1.00E+00
LOC112584612	3.09E+00	1.77E-19	PI4KA	9.90E-02	1.00E+00	MCL1	-9.70E-02	1.00E+00
PHOSPHO1	4.81E+00	1.77E-19	KIAA1257	-8.93E-02	1.00E+00	POLE2	-8.08E-02	1.00E+00
LOC112584610	-3.47E+00	1.78E-19	SYNE3	-1.48E-01	1.00E+00	KIAA0355	-6.09E-02	1.00E+00
RPS6	-2.05E+00	1.80E-19	GPR39	5.13E-02	1.00E+00	HRH2	2.34E-01	1.00E+00
C5H1orf115	-3.80E+00	1.83E-19	ASDURF	1.13E-01	1.00E+00	MTHFSD	-7.27E-02	1.00E+00
LOC102404666	-5.49E+00	1.84E-19	TMEM181	7.18E-02	1.00E+00	MARCH2	-6.99E-02	1.00E+00
LOC112581627	5.15E+00	1.84E-19	BZW1	-5.80E-02	1.00E+00	PPP6C	-6.24E-02	1.00E+00
ADAM32	-2.93E+00	1.84E-19	LOC112586657	2.48E-01	1.00E+00	ZSCAN31	-9.23E-02	1.00E+00
SUMF1	-8.77E-01	1.86E-19	ABHD12	-6.21E-02	1.00E+00	LOC112584719	1.19E-01	1.00E+00
AVEN	-1.24E+00	1.89E-19	STX7	6.59E-02	1.00E+00	GK	8.18E-02	1.00E+00
PAK5	3.78E+00	1.90E-19	MCL1	-7.69E-02	1.00E+00	CITED1	9.03E-02	1.00E+00
RBL2	1.30E+00	1.92E-19	LOC112587851	1.35E-01	1.00E+00	POLR3C	-5.66E-02	1.00E+00
HAPLN1	4.10E+00	1.94E-19	STX17	-5.34E-02	1.00E+00	AP5M1	-6.87E-02	1.00E+00
LEPROTL1	-8.91E-01	1.99E-19	PPFIA1	8.02E-02	1.00E+00	QRICH2	1.18E-01	1.00E+00

LOC102403160	-1.46E+00	2.04E-19	KHK	-1.48E-01	1.00E+00	PPM1D	7.48E-02	1.00E+00
ENTR1	8.50E-01	2.07E-19	TMEM163	-4.56E-02	1.00E+00	GRK6	-6.33E-02	1.00E+00
IFNLR1	-1.98E+00	2.09E-19	IRAK4	-7.97E-02	1.00E+00	B4GALT3	-5.30E-02	1.00E+00
TMCO4	-1.24E+00	2.13E-19	RNF220	-5.20E-02	1.00E+00	SPEF2	-8.38E-02	1.00E+00
CTC1	9.81E-01	2.13E-19	BEAN1	-1.85E-01	1.00E+00	TRIP11	-7.23E-02	1.00E+00
PSMD5	7.96E-01	2.14E-19	LOC112587811	1.71E-01	1.00E+00	LOC102405290	-7.13E-02	1.00E+00
TMEM108	2.63E+00	2.17E-19	C7H4orf3	-8.55E-02	1.00E+00	TMTC2	7.93E-02	1.00E+00
LOC112585146	-1.92E+00	2.28E-19	DBN1	-6.02E-02	1.00E+00	LOC102409972	9.46E-02	1.00E+00
NRP2	-2.08E+00	2.32E-19	MECOM	-1.27E-01	1.00E+00	DHX16	-5.04E-02	1.00E+00
ESR1	-5.03E+00	2.33E-19	LOC112586812	1.56E-01	1.00E+00	HDHD5	7.85E-02	1.00E+00
PTPRT	2.68E+00	2.34E-19	SMURF1	6.18E-02	1.00E+00	FAM49B	-5.24E-02	1.00E+00
CABP2	3.04E+00	2.39E-19	MYO18B	6.13E-02	1.00E+00	LOC112580384	1.17E-01	1.00E+00
HTR1F	-5.17E+00	2.41E-19	LOC112578672	-2.33E-01	1.00E+00	MSS51	8.12E-02	1.00E+00
FCRL5	-4.62E+00	2.43E-19	BACH2	-3.45E-01	1.00E+00	BMP1	-6.17E-02	1.00E+00
TMEM64	-1.48E+00	2.46E-19	BTF3	-5.30E-02	1.00E+00	EIF1AD	7.02E-02	1.00E+00
OTUB2	2.17E+00	2.47E-19	PLSCR5	3.28E-01	1.00E+00	KAT2A	5.59E-02	1.00E+00
SMIM14	1.13E+00	2.52E-19	OTULINL	1.14E-01	1.00E+00	POLRMT	-5.42E-02	1.00E+00
ATCAY	4.36E+00	2.55E-19	TOX4	6.22E-02	1.00E+00	SCP2	-5.31E-02	1.00E+00
LOC102400013	-6.22E+00	2.57E-19	ZFP69B	7.98E-02	1.00E+00	ZFP37	-9.99E-02	1.00E+00
LOC112578872	3.53E+00	2.59E-19	LOC112586997	-2.67E-01	1.00E+00	LOC102394761	9.61E-02	1.00E+00
SNX22	2.21E+00	2.60E-19	NEK7	9.96E-02	1.00E+00	LOC112584711	1.31E-01	1.00E+00
LOC102416267	-4.61E+00	2.60E-19	LOC102405090	-2.26E-01	1.00E+00	TNNT1	1.18E-01	1.00E+00
SNX21	-1.66E+00	2.61E-19	LARP1B	6.57E-02	1.00E+00	PRKCZ	7.95E-02	1.00E+00
OBSCN	3.03E+00	2.66E-19	LIMA1	4.80E-02	1.00E+00	PNKD	8.10E-02	1.00E+00
AIDA	8.87E-01	2.66E-19	FOXQ1	-1.70E-01	1.00E+00	LOC102415185	-1.13E-01	1.00E+00
CENPF	1.50E+00	2.66E-19	RAB6B	-4.98E-02	1.00E+00	NEK1	6.67E-02	1.00E+00
SLC31A1	-1.02E+00	2.71E-19	PPAT	-8.62E-02	1.00E+00	CTTN	5.62E-02	1.00E+00
SESN1	1.16E+00	2.72E-19	PSMG4	-8.83E-02	1.00E+00	TXNL4A	-6.61E-02	1.00E+00
MAP1LC3B	9.28E-01	2.73E-19	LOC102413806	-7.50E-02	1.00E+00	LOC102393972	-4.92E-02	1.00E+00
CNOT2	-8.55E-01	2.77E-19	HDHD2	4.78E-02	1.00E+00	IQUB	1.18E-01	1.00E+00
IMPG2	-2.70E+00	2.86E-19	EFCAB11	1.46E-01	1.00E+00	CNNM4	-7.30E-02	1.00E+00
PALMD	-6.53E+00	2.92E-19	CHP1	4.73E-02	1.00E+00	PDK2	-5.66E-02	1.00E+00
LOC102406330	-4.93E+00	2.94E-19	CSRP2	6.68E-02	1.00E+00	PTAR1	7.22E-02	1.00E+00
RAB36	-	2.95E-19	SYPL1	-6.72E-	1.00E+00	LOC112585732	1.22E-01	1.00E+00

	4.41E+00			02				
EPM2A	-3.97E+00	3.01E-19	CCDC137	-6.47E-02	1.00E+00	ZNF408	5.42E-02	1.00E+00
ADARB1	1.09E+00	3.04E-19	LOC102411226	-3.41E-01	1.00E+00	ATG101	-7.12E-02	1.00E+00
MAP2K3	-1.25E+00	3.08E-19	ARIH2	6.78E-02	1.00E+00	SUSD1	1.71E-01	1.00E+00
SDR39U1	-2.10E+00	3.10E-19	CDCA7	-6.27E-02	1.00E+00	APEH	-5.37E-02	1.00E+00
KIF2B	6.76E+00	3.12E-19	FBXO6	9.99E-02	1.00E+00	SYT9	5.94E-02	1.00E+00
ARL2	-1.36E+00	3.17E-19	LOC102411696	6.22E-01	1.00E+00	LOC102413816	-1.31E-01	1.00E+00
FZD9	-2.81E+00	3.19E-19	C24H16orf45	-2.30E-01	1.00E+00	LOC102404003	-6.28E-02	1.00E+00
GRM7	3.47E+00	3.21E-19	ZNF518B	8.49E-02	1.00E+00	CD74	2.22E-01	1.00E+00
SYT6	4.14E+00	3.21E-19	TIPARP	9.71E-02	1.00E+00	CLTA	-6.70E-02	1.00E+00
PPP4C	-1.23E+00	3.25E-19	LOC112581589	-1.74E-01	1.00E+00	RNGTT	5.54E-02	1.00E+00
PXDC1	-2.86E+00	3.25E-19	LOC112586244	9.81E-02	1.00E+00	ABHD15	1.24E-01	1.00E+00
POLR2J	1.30E+00	3.35E-19	LOC112579487	-6.83E-02	1.00E+00	WDR24	-6.02E-02	1.00E+00
NPHP3	-3.15E+00	3.38E-19	SNRPA1	-5.43E-02	1.00E+00	MGMT	-9.08E-02	1.00E+00
SERGEF	-2.21E+00	3.40E-19	PIK3C2A	1.13E-01	1.00E+00	SNX17	5.81E-02	1.00E+00
SLC10A2	5.22E+00	3.42E-19	PKN2	1.29E-01	1.00E+00	LOC112578005	1.10E-01	1.00E+00
ZBTB7C	-3.69E+00	3.48E-19	LOC112579082	2.34E-01	1.00E+00	LOC102414992	1.53E-01	1.00E+00
NYX	3.42E+00	3.48E-19	CFAP20	-5.51E-02	1.00E+00	LOC112579993	-2.85E-01	1.00E+00
CD200	1.81E+00	3.52E-19	NDUFA7	7.59E-02	1.00E+00	ZNF668	-7.11E-02	1.00E+00
ST3GAL6	8.97E-01	3.53E-19	C11H14orf39	2.22E-01	1.00E+00	NKD1	1.17E-01	1.00E+00
RBBP4	-8.03E-01	3.55E-19	LOC112586800	3.01E-01	1.00E+00	AGPAT2	-1.45E-01	1.00E+00
MST1	-1.71E+00	3.70E-19	C17H4orf51	3.51E-01	1.00E+00	GFPT1	-5.47E-02	1.00E+00
PHF12	1.07E+00	3.71E-19	WDR59	8.29E-02	1.00E+00	MCM10	-6.31E-02	1.00E+00
SCAF11	-1.00E+00	3.71E-19	PTCD2	-9.58E-02	1.00E+00	HPS1	-5.14E-02	1.00E+00
TFR2	-1.87E+00	3.72E-19	IL4R	-2.08E-01	1.00E+00	SPG21	5.47E-02	1.00E+00
LOC102398125	-4.98E+00	3.75E-19	CHIC1	3.14E-01	1.00E+00	LOC112579071	1.15E-01	1.00E+00
CDPF1	-2.21E+00	3.81E-19	MAK	-2.60E-01	1.00E+00	CFL1	7.84E-02	1.00E+00
LOC102393522	-2.67E+00	3.81E-19	REST	-2.11E-01	1.00E+00	AKAP10	-6.14E-02	1.00E+00
LOC102409611	5.27E+00	3.83E-19	SSH3	5.96E-02	1.00E+00	LOC102389167	1.19E-01	1.00E+00
ZMYM2	1.10E+00	3.85E-19	NUMBL	-1.31E-01	1.00E+00	CSNK1D	5.06E-02	1.00E+00
FGF11	-2.37E+00	3.92E-19	RSPH14	1.78E-01	1.00E+00	SLC27A6	-1.14E-01	1.00E+00
LOC102405064	-4.21E+00	3.93E-19	AGPS	5.62E-02	1.00E+00	MRPL37	-5.89E-02	1.00E+00
LOC102403557	-1.99E+00	4.05E-19	MIF4GD	6.83E-02	1.00E+00	LOC102405684	1.08E-01	1.00E+00
WDR60	-1.25E+00	4.24E-19	ZNF641	9.96E-02	1.00E+00	MAP3K2	-5.84E-02	1.00E+00

TEKT3	3.12E+00	4.25E-19	ZNF449	7.79E-02	1.00E+00	TANGO2	6.97E-02	1.00E+00
PITRM1	-9.47E-01	4.26E-19	TBC1D1	5.18E-02	1.00E+00	NET1	4.90E-02	1.00E+00
NOX5	2.33E+00	4.26E-19	LOC112587814	1.50E-01	1.00E+00	FBXL17	5.40E-02	1.00E+00
METTL9	-9.81E-01	4.28E-19	LOC102394843	-2.58E-01	1.00E+00	GPBP1L1	5.29E-02	1.00E+00
HTR1B	-5.72E+00	4.46E-19	OAZ1	-5.31E-02	1.00E+00	NOTUM	-1.14E-01	1.00E+00
GVQW3	-4.26E+00	4.49E-19	CCDC192	-1.19E-01	1.00E+00	DNAJA3	-5.70E-02	1.00E+00
MFAP2	-4.02E+00	4.51E-19	MS4A3	-1.98E-01	1.00E+00	ACTR1A	5.17E-02	1.00E+00
CCR8	1.20E+00	4.52E-19	ZSWIM6	-1.87E-01	1.00E+00	OTULINL	6.23E-02	1.00E+00
LOC112584574	-4.20E+00	4.54E-19	USP38	-8.76E-02	1.00E+00	ABCA5	-6.36E-02	1.00E+00
KLHL29	2.14E+00	4.57E-19	CCDC28A	-1.42E-01	1.00E+00	ASAP1	8.35E-02	1.00E+00
LOC112586217	2.95E+00	4.61E-19	GAMT	-2.44E-01	1.00E+00	CENPO	-7.68E-02	1.00E+00
U2AF1	-1.19E+00	4.71E-19	CEP350	-8.68E-02	1.00E+00	ELAVL1	4.99E-02	1.00E+00
C12H9orf16	-2.69E+00	4.80E-19	FGFR1	9.03E-02	1.00E+00	PCNX1	-5.52E-02	1.00E+00
LHFPL2	2.26E+00	4.82E-19	DYNC2H1	-1.20E-01	1.00E+00	LOC102416376	1.07E-01	1.00E+00
LOC102393569	-5.03E+00	4.86E-19	PI4KB	4.62E-02	1.00E+00	USP6NL	7.77E-02	1.00E+00
USF2	-1.04E+00	4.91E-19	SRRT	5.22E-02	1.00E+00	POLR2K	-7.14E-02	1.00E+00
USP15	8.59E-01	4.94E-19	LOC102413071	-8.59E-02	1.00E+00	PEX16	-7.42E-02	1.00E+00
CRACR2A	-1.67E+00	4.95E-19	MEIS2	-1.27E-01	1.00E+00	PPP2R3A	7.61E-02	1.00E+00
DEPTOR	1.85E+00	4.98E-19	VMA21	1.05E-01	1.00E+00	CSRNP3	-7.35E-02	1.00E+00
LOC112584643	4.21E+00	4.99E-19	LOC102405716	-1.80E-01	1.00E+00	CCP110	-9.30E-02	1.00E+00
LOC102403725	-2.00E+00	5.01E-19	ST6GALNAC3	-1.60E-01	1.00E+00	PKD1L2	6.37E-02	1.00E+00
LOC102392771	-2.56E+00	5.05E-19	WSB1	6.45E-02	1.00E+00	CLEC14A	-1.13E-01	1.00E+00
RPUSD3	1.18E+00	5.12E-19	TBC1D22A	-7.03E-02	1.00E+00	ABT1	5.15E-02	1.00E+00
TMEM37	-2.85E+00	5.21E-19	FAM199X	8.83E-02	1.00E+00	HEG1	6.27E-02	1.00E+00
REM2	1.27E+00	5.21E-19	ZNF235	-6.48E-02	1.00E+00	GPR173	-7.08E-02	1.00E+00
TUT1	-1.31E+00	5.21E-19	SELENOI	7.06E-02	1.00E+00	DEPTOR	1.20E-01	1.00E+00
TRIM45	-1.29E+00	5.25E-19	DUSP7	-9.80E-02	1.00E+00	DTWD1	-6.71E-02	1.00E+00
ENTPD3	-3.81E+00	5.28E-19	UCK1	5.90E-02	1.00E+00	PRR15	-5.68E-02	1.00E+00
CDNF	-3.11E+00	5.36E-19	RELN	-2.31E-01	1.00E+00	ETV2	-1.26E-01	1.00E+00
DHX33	-1.92E+00	5.43E-19	LOC102395360	1.51E-01	1.00E+00	BUD23	-6.09E-02	1.00E+00
RUFY3	-8.37E-01	5.47E-19	EIF2B1	-6.58E-02	1.00E+00	TMEM160	-1.03E-01	1.00E+00
SLC16A3	-1.94E+00	5.49E-19	KLHDC1	6.75E-02	1.00E+00	CAV2	-1.07E-01	1.00E+00
RAMP3	-8.24E+00	5.49E-19	DMBX1	1.66E-01	1.00E+00	RPL36AL	8.65E-02	1.00E+00

FHL5	5.50E+00	5.55E-19	SUPV3L1	-6.74E-02	1.00E+00	RBX1	5.25E-02	1.00E+00
LOC112578658	4.81E+00	5.59E-19	RNF170	-7.85E-02	1.00E+00	MYO5A	-6.41E-02	1.00E+00
MKLN1	7.54E-01	5.64E-19	CUEDC2	-9.66E-02	1.00E+00	ARID4B	-7.27E-02	1.00E+00
SLC25A32	9.00E-01	5.69E-19	MYO18A	-6.95E-02	1.00E+00	HECW1	5.20E-02	1.00E+00
HOXC8	-5.23E+00	5.69E-19	CACNA1A	-1.32E-01	1.00E+00	GMPS	-4.95E-02	1.00E+00
NOB1	-1.25E+00	5.73E-19	PLEKHO1	-7.75E-02	1.00E+00	ING3	-5.88E-02	1.00E+00
RPE	9.52E-01	5.74E-19	LOC102408340	-7.08E-02	1.00E+00	GMPR2	-5.48E-02	1.00E+00
NDUFV2	-1.10E+00	5.84E-19	CBARP	1.63E-01	1.00E+00	MNS1	-8.72E-02	1.00E+00
MMEL1	-4.07E+00	5.88E-19	HNRNPL	7.98E-02	1.00E+00	ARL8B	5.80E-02	1.00E+00
THAP4	-1.14E+00	5.99E-19	EME1	1.04E-01	1.00E+00	FRMD8	9.75E-02	1.00E+00
LOC102401452	6.89E+00	5.99E-19	POLRMT	8.46E-02	1.00E+00	RPA3	-7.41E-02	1.00E+00
MAP3K8	-5.72E+00	6.06E-19	METTL25	1.14E-01	1.00E+00	CARS	5.36E-02	1.00E+00
DHX37	9.68E-01	6.22E-19	RPS6KA5	6.70E-02	1.00E+00	GRAP2	1.63E-01	1.00E+00
ITGA5	-3.61E+00	6.26E-19	THNSL2	2.31E-01	1.00E+00	TREX1	9.90E-02	1.00E+00
PTF1A	6.07E+00	6.35E-19	PSMD14	-6.36E-02	1.00E+00	OMA1	6.78E-02	1.00E+00
LOC102396108	3.13E+00	6.42E-19	RNF114	6.25E-02	1.00E+00	EML4	-6.04E-02	1.00E+00
FAAP100	-1.31E+00	6.49E-19	PRELID3A	5.65E-02	1.00E+00	CIAO3	6.06E-02	1.00E+00
PBDC1	1.15E+00	6.51E-19	AFG1L	9.59E-02	1.00E+00	M6PR	4.82E-02	1.00E+00
FN3K	2.15E+00	6.54E-19	ATG9A	6.87E-02	1.00E+00	ZNF775	6.60E-02	1.00E+00
FYB2	1.78E+00	6.55E-19	PPP1R15A	-5.30E-02	1.00E+00	UBXN1	6.75E-02	1.00E+00
LOC102400551	1.61E+00	6.56E-19	DAGLA	-7.36E-02	1.00E+00	PRR19	1.20E-01	1.00E+00
NEMF	7.93E-01	6.66E-19	HEXDC	-1.35E-01	1.00E+00	MAPK7	-5.39E-02	1.00E+00
CWC15	9.50E-01	6.70E-19	STAMBPL1	3.93E-01	1.00E+00	MEF2D	-7.06E-02	1.00E+00
LOC112586285	-4.50E+00	6.70E-19	ADO	-6.84E-02	1.00E+00	EMILIN1	7.49E-02	1.00E+00
LOC112585799	-1.53E+00	6.79E-19	MBD3	6.17E-02	1.00E+00	RPGRIP1	-7.21E-02	1.00E+00
ZNF638	-9.34E-01	6.91E-19	ABCB10	-8.72E-02	1.00E+00	ANKLE1	-1.07E-01	1.00E+00
CFTR	4.34E+00	6.97E-19	SMCHD1	-1.21E-01	1.00E+00	GRIA3	9.43E-02	1.00E+00
FBLL1	2.14E+00	7.01E-19	NR1D1	8.76E-02	1.00E+00	EP300	-7.00E-02	1.00E+00
ZNF608	-1.68E+00	7.02E-19	MRPS31	6.42E-02	1.00E+00	SPATA2	-6.17E-02	1.00E+00
AP1G1	-8.41E-01	7.03E-19	TSR2	-5.92E-02	1.00E+00	KIAA0319L	4.91E-02	1.00E+00
CAP1	-9.44E-01	7.06E-19	PRADC1	-8.59E-02	1.00E+00	TMEM17	-8.29E-02	1.00E+00
KSR2	4.36E+00	7.12E-19	RBM23	8.44E-02	1.00E+00	PRKX	8.06E-02	1.00E+00
KCNH5	4.72E+00	7.20E-19	LOC112586215	2.00E-01	1.00E+00	CPSF3	4.94E-02	1.00E+00
TAF15	-1.15E+00	7.22E-19	CFL2	-5.08E-02	1.00E+00	SUPT7L	-6.11E-02	1.00E+00

SDK2	4.11E+00	7.29E-19	LOC102414052	8.32E-02	1.00E+00	DMXL1	-8.11E-02	1.00E+00
LOC112587915	2.77E+00	7.39E-19	C19H5orf49	3.32E-01	1.00E+00	THUMPD3	5.07E-02	1.00E+00
HES4	-2.27E+00	7.45E-19	LOC112581795	1.13E-01	1.00E+00	LOC102415675	-5.50E-02	1.00E+00
HERC2	-1.34E+00	7.45E-19	ITGB5	5.27E-02	1.00E+00	VHL	7.64E-02	1.00E+00
MED6	8.41E-01	7.55E-19	NRG2	4.78E-02	1.00E+00	LOC102400830	6.89E-02	1.00E+00
CST6	1.62E+00	7.58E-19	DOHH	-7.96E-02	1.00E+00	RBM28	5.30E-02	1.00E+00
GRM3	4.96E+00	7.59E-19	BRI3	-8.67E-02	1.00E+00	CCDC120	7.19E-02	1.00E+00
UBE2J2	8.64E-01	7.91E-19	TADA2B	1.19E-01	1.00E+00	RPS6KB1	-5.14E-02	1.00E+00
CD80	-3.51E+00	8.05E-19	LOC102404003	5.38E-02	1.00E+00	C21H3orf22	-1.18E-01	1.00E+00
BFSP1	2.03E+00	8.08E-19	ENO4	-1.19E-01	1.00E+00	MRPS2	6.77E-02	1.00E+00
WDR38	-2.82E+00	8.22E-19	RPS6KA1	9.88E-02	1.00E+00	ATXN1L	7.77E-02	1.00E+00
PLEKHA2	-3.08E+00	8.25E-19	SUMO2	-5.48E-02	1.00E+00	SGCB	8.46E-02	1.00E+00
LMNTD2	-3.11E+00	8.28E-19	PPP2R3A	-1.08E-01	1.00E+00	ago-02	-6.04E-02	1.00E+00
SLC25A34	-4.06E+00	8.47E-19	DNAAF2	1.04E-01	1.00E+00	LOC102403795	-5.25E-02	1.00E+00
LOC112585729	5.07E+00	8.55E-19	LOC112587112	2.09E-01	1.00E+00	NOG	1.25E-01	1.00E+00
SF3B3	-7.71E-01	8.56E-19	SUPT6H	6.09E-02	1.00E+00	FAM189B	-5.00E-02	1.00E+00
LOC102408090	-1.33E+00	9.04E-19	VPS50	7.66E-02	1.00E+00	ZC2HC1B	1.27E-01	1.00E+00
POLN	-1.26E+00	9.28E-19	ESRRB	-1.67E-01	1.00E+00	CCDC125	-8.42E-02	1.00E+00
CDR1	-7.58E+00	9.45E-19	SPDYA	1.17E-01	1.00E+00	TMEM141	8.98E-02	1.00E+00
RMDN1	9.36E-01	9.52E-19	SLF2	8.39E-02	1.00E+00	PALLD	-5.41E-02	1.00E+00
RPL7A	-1.89E+00	9.53E-19	DVL3	5.28E-02	1.00E+00	EPSTI1	-6.65E-02	1.00E+00
SCML2	1.50E+00	9.54E-19	CIART	-5.21E-02	1.00E+00	TYRO3	5.42E-02	1.00E+00
GABBR2	2.25E+00	9.55E-19	LOC112577692	1.18E-01	1.00E+00	PPP3CC	-5.01E-02	1.00E+00
FXYD5	-3.79E+00	9.64E-19	SLC46A1	6.66E-02	1.00E+00	WDR26	6.39E-02	1.00E+00
TMEM41A	-1.12E+00	9.67E-19	WLS	5.14E-02	1.00E+00	RSAD1	7.07E-02	1.00E+00
CLCN5	1.32E+00	9.86E-19	KMT5C	5.18E-02	1.00E+00	GATAD2B	-5.98E-02	1.00E+00
VCAN	-5.12E+00	1.00E-18	STAT3	7.16E-02	1.00E+00	SCFD2	6.00E-02	1.00E+00
AKAIN1	1.84E+00	1.02E-18	USP34	8.60E-02	1.00E+00	ANKRD17	-6.46E-02	1.00E+00
TNK2	8.97E-01	1.03E-18	APOBEC2	1.56E-01	1.00E+00	PDCD10	5.24E-02	1.00E+00
SLC35F6	-8.41E-01	1.05E-18	LOC112580253	2.45E-01	1.00E+00	LOC102390371	1.02E-01	1.00E+00
YTHDC2	-1.72E+00	1.07E-18	NHS	-5.04E-02	1.00E+00	CD58	5.39E-02	1.00E+00
DDX42	-8.14E-01	1.08E-18	EMX2	-4.26E-01	1.00E+00	EFCAB14	-4.75E-02	1.00E+00
CALU	-1.13E+00	1.10E-18	LOC102413798	9.01E-02	1.00E+00	HPS6	-9.60E-02	1.00E+00
TFG	9.54E-01	1.11E-18	PARG	6.45E-02	1.00E+00	CNPY4	-6.17E-	1.00E+00

							02	
GRAMD2B	-1.10E+00	1.13E-18	VPS26B	5.15E-02	1.00E+00	FKBP7	5.96E-02	1.00E+00
LOC112578166	-4.75E+00	1.15E-18	CCDC88C	6.99E-02	1.00E+00	LOC102403928	-1.19E-01	1.00E+00
ATP13A3	-1.96E+00	1.16E-18	GRB10	-8.04E-02	1.00E+00	WDR41	-4.89E-02	1.00E+00
PHF13	1.03E+00	1.19E-18	FARP2	8.19E-02	1.00E+00	SCAMP1	-5.93E-02	1.00E+00
LOC102411445	2.29E+00	1.20E-18	EMC4	-9.78E-02	1.00E+00	MRPL48	7.55E-02	1.00E+00
PI4KA	-1.27E+00	1.25E-18	ERAS	-1.96E-01	1.00E+00	FXYD3	1.02E-01	1.00E+00
SAMD13	-4.67E+00	1.26E-18	CCT2	-5.11E-02	1.00E+00	LOC102409683	1.29E-01	1.00E+00
ABLIM1	1.07E+00	1.27E-18	LTA4H	2.85E-01	1.00E+00	TMEM9	-5.55E-02	1.00E+00
LRFN5	-3.62E+00	1.27E-18	LCMT1	-6.28E-02	1.00E+00	CTNNBL1	-6.76E-02	1.00E+00
LOC112587293	6.24E+00	1.28E-18	QPCTL	7.42E-02	1.00E+00	NOD2	1.59E-01	1.00E+00
FBLN7	-3.89E+00	1.29E-18	PEX1	8.30E-02	1.00E+00	LSM12	4.84E-02	1.00E+00
PABPC1	-1.21E+00	1.31E-18	C1H3orf33	8.76E-02	1.00E+00	BCL10	7.57E-02	1.00E+00
LOC102405684	-2.09E+00	1.31E-18	LOC102414209	-1.42E-01	1.00E+00	PEG3	-7.09E-02	1.00E+00
SPOUT1	1.19E+00	1.31E-18	LOC102395519	3.03E-01	1.00E+00	LARP7	-5.25E-02	1.00E+00
LOC102411401	-5.04E+00	1.32E-18	B3GNT9	1.73E-01	1.00E+00	KRR1	-6.85E-02	1.00E+00
CIPC	9.46E-01	1.33E-18	LOC112579515	-1.62E-01	1.00E+00	RAD54B	-6.97E-02	1.00E+00
C2CD4A	3.17E+00	1.33E-18	LOC112580206	-2.09E-01	1.00E+00	COMMD1	6.77E-02	1.00E+00
NPC2	1.30E+00	1.33E-18	CASTOR1	5.24E-02	1.00E+00	IHH	-9.14E-02	1.00E+00
CARD11	2.99E+00	1.34E-18	DYSF	1.77E-01	1.00E+00	C9H19orf53	6.99E-02	1.00E+00
PTGS2	-2.01E+00	1.34E-18	LOC112579930	-1.50E-01	1.00E+00	PEX26	-6.00E-02	1.00E+00
SVOP	-2.25E+00	1.35E-18	LOC102404191	1.87E-01	1.00E+00	CCT2	5.70E-02	1.00E+00
MCTP1	3.76E+00	1.37E-18	PBRM1	1.06E-01	1.00E+00	NCALD	1.38E-01	1.00E+00
ITGAE	1.29E+00	1.37E-18	RPS20	-6.23E-02	1.00E+00	POLR2E	-6.94E-02	1.00E+00
CA11	-3.36E+00	1.38E-18	FIGNL1	-5.58E-02	1.00E+00	RAB1A	-5.67E-02	1.00E+00
CEP135	-1.70E+00	1.42E-18	ODR4	-7.34E-02	1.00E+00	LOC102393133	8.58E-02	1.00E+00
HPS3	1.39E+00	1.43E-18	MRPS28	-7.56E-02	1.00E+00	IMPDH1	-5.93E-02	1.00E+00
HOXC9	1.92E+00	1.43E-18	SLC31A1	7.11E-02	1.00E+00	LOC102403078	1.15E-01	1.00E+00
CENPO	1.31E+00	1.43E-18	GPN3	-6.04E-02	1.00E+00	TBC1D10B	-5.25E-02	1.00E+00
LOC102394142	-4.27E+00	1.45E-18	PNMA6E	2.09E-01	1.00E+00	MYH7B	-8.06E-02	1.00E+00
ING3	-1.10E+00	1.48E-18	GART	6.44E-02	1.00E+00	C12H9orf116	-9.66E-02	1.00E+00
NUTM1	2.88E+00	1.50E-18	SLC39A13	-8.42E-02	1.00E+00	UFSP1	9.74E-02	1.00E+00
UMPS	-1.19E+00	1.52E-18	LOC102409453	-9.98E-02	1.00E+00	LOC102405833	7.54E-02	1.00E+00
SSC5D	1.79E+00	1.54E-18	SMARCA5	6.63E-02	1.00E+00	PHF2	6.90E-02	1.00E+00

CCDC61	1.12E+00	1.55E-18	GPA33	1.24E-01	1.00E+00	MRNIP	-6.76E-02	1.00E+00
LOC102390535	5.28E+00	1.63E-18	TMEM74	1.63E-01	1.00E+00	MYCBP	1.14E-01	1.00E+00
PLBD1	-4.47E+00	1.64E-18	CYLD	-7.03E-02	1.00E+00	CDK10	-7.20E-02	1.00E+00
ADAMDEC1	-4.76E+00	1.66E-18	KIAA1211L	-7.52E-02	1.00E+00	FAM71E1	1.16E-01	1.00E+00
DENND2D	3.54E+00	1.68E-18	SMG8	7.61E-02	1.00E+00	ZBTB38	1.00E-01	1.00E+00
LOC112583531	4.27E+00	1.70E-18	HBEGF	-5.19E-02	1.00E+00	SPP1	-2.02E-01	1.00E+00
LOC112587794	5.35E+00	1.87E-18	ZC3H12D	2.76E-01	1.00E+00	CRLF2	-2.49E-01	1.00E+00
ENPP1	1.44E+00	1.88E-18	CCNG2	8.86E-02	1.00E+00	ASNA1	6.80E-02	1.00E+00
UBXN10	2.20E+00	1.88E-18	CA8	-1.54E-01	1.00E+00	NCOR1	-5.60E-02	1.00E+00
TBC1D9B	-8.47E-01	1.88E-18	KCNAB1	2.77E-01	1.00E+00	FAM204A	-5.20E-02	1.00E+00
SLC18A2	1.15E+00	1.88E-18	RTKN2	5.65E-02	1.00E+00	LOC112581895	-6.00E-02	1.00E+00
NF1	-1.22E+00	1.89E-18	WDR31	-7.19E-02	1.00E+00	WDR83OS	7.04E-02	1.00E+00
LOC102407046	4.71E+00	1.90E-18	SEPT2	-4.88E-02	1.00E+00	FAM89B	6.35E-02	1.00E+00
MTF2	1.00E+00	1.96E-18	CERS6	2.08E-01	1.00E+00	RPL5	-8.75E-02	1.00E+00
NEUROD4	4.98E+00	1.97E-18	SCML4	2.23E-01	1.00E+00	FOXN3	-5.37E-02	1.00E+00
RPUSD1	1.51E+00	2.01E-18	BTBD9	4.71E-02	1.00E+00	UTP3	-6.02E-02	1.00E+00
DOCK9	-1.33E+00	2.05E-18	ZFP64	5.27E-02	1.00E+00	CCDC181	1.15E-01	1.00E+00
GRWD1	-1.20E+00	2.09E-18	FAM69A	-7.60E-02	1.00E+00	RAB8B	6.07E-02	1.00E+00
LOC102413952	-6.15E+00	2.13E-18	CFAP36	-7.10E-02	1.00E+00	CHORDC1	4.98E-02	1.00E+00
TBCC	8.38E-01	2.16E-18	SPRN	-2.00E-01	1.00E+00	HEXDC	-6.41E-02	1.00E+00
COPS2	-8.13E-01	2.17E-18	SNX11	4.43E-02	1.00E+00	RSPH10B	1.04E-01	1.00E+00
ROPN1	4.65E+00	2.19E-18	MTLN	-1.26E-01	1.00E+00	TMEM35B	1.40E-01	1.00E+00
TMLHE	-1.23E+00	2.20E-18	SUB1	-5.67E-02	1.00E+00	RETREG3	4.51E-02	1.00E+00
CELF5	2.54E+00	2.22E-18	ANKRA2	7.02E-02	1.00E+00	KAT2B	-6.60E-02	1.00E+00
LOC102392624	-1.17E+00	2.22E-18	CLIC6	2.58E-01	1.00E+00	NCBP1	-5.51E-02	1.00E+00
PTPRA	-7.57E-01	2.23E-18	RASIP1	-1.16E-01	1.00E+00	YIPF1	5.47E-02	1.00E+00
TXLNA	8.90E-01	2.25E-18	LOC112580858	-2.39E-01	1.00E+00	PGM2L1	5.70E-02	1.00E+00
PRICKLE4	1.39E+00	2.25E-18	RIOK2	-8.35E-02	1.00E+00	POLR2H	6.58E-02	1.00E+00
LOC102400049	5.71E+00	2.31E-18	CRTC1	5.86E-02	1.00E+00	INTS4	-5.34E-02	1.00E+00
ETV1	2.43E+00	2.33E-18	IQSEC1	8.01E-02	1.00E+00	DDX58	8.15E-02	1.00E+00
LOC112582076	-2.86E+00	2.33E-18	NIF3L1	-5.31E-02	1.00E+00	FUNDC2	5.51E-02	1.00E+00
NUDC	-1.35E+00	2.33E-18	RBM33	-5.67E-02	1.00E+00	PAIP2	-1.20E-01	1.00E+00
HOMEZ	9.41E-01	2.34E-18	MED29	-9.61E-02	1.00E+00	NCOA6	-5.93E-02	1.00E+00
CCDC102B	4.64E+00	2.40E-18	LOC112586499	1.22E-01	1.00E+00	TEX10	-4.74E-	1.00E+00

							02	
AGA	-4.17E+00	2.41E-18	MARC2	-1.63E-01	1.00E+00	RAPGEF3	-1.05E-01	1.00E+00
OGFOD1	8.67E-01	2.43E-18	GNL1	-5.25E-02	1.00E+00	TAOK1	5.15E-02	1.00E+00
TNC	-4.28E+00	2.44E-18	NDUFA8	-5.70E-02	1.00E+00	DNAL4	5.95E-02	1.00E+00
LRRK2	-1.84E+00	2.47E-18	LSM10	6.41E-02	1.00E+00	SLC9A7	-1.14E-01	1.00E+00
TRMT12	1.06E+00	2.49E-18	VKORC1L1	-8.40E-02	1.00E+00	SNRPN	-5.75E-02	1.00E+00
LOC112586164	4.31E+00	2.49E-18	ATG5	-4.53E-02	1.00E+00	MCUR1	6.20E-02	1.00E+00
PPM1D	-1.53E+00	2.49E-18	FAM163A	5.95E-02	1.00E+00	LOC102389674	2.34E-01	1.00E+00
VAV2	1.23E+00	2.51E-18	LOC102392450	1.51E-01	1.00E+00	CRY1	6.22E-02	1.00E+00
IDS	-1.17E+00	2.56E-18	NRF1	6.94E-02	1.00E+00	TMEM251	7.19E-02	1.00E+00
NDUFA13	-1.53E+00	2.57E-18	RYBP	7.10E-02	1.00E+00	LOC102393963	1.30E-01	1.00E+00
DENND2A	1.01E+00	2.59E-18	MSANTD3	9.83E-02	1.00E+00	UPF1	-5.11E-02	1.00E+00
LHX5	7.17E+00	2.67E-18	FGF12	-1.67E-01	1.00E+00	TRIM26	-5.18E-02	1.00E+00
PGM2	-2.66E+00	2.71E-18	KNSTRN	-5.54E-02	1.00E+00	FGF2	7.42E-02	1.00E+00
TSPOAP1	-3.68E+00	2.76E-18	TUBB	-5.91E-02	1.00E+00	REST	-1.00E-01	1.00E+00
LOC102401468	-1.32E+00	2.76E-18	RAPGEF4	-5.44E-02	1.00E+00	TRIM50	-1.16E-01	1.00E+00
MRM2	-1.62E+00	2.76E-18	ARMC6	7.72E-02	1.00E+00	COMMD8	-5.98E-02	1.00E+00
GID8	-4.89E+00	2.82E-18	UMAD1	1.53E-01	1.00E+00	TRPT1	-9.38E-02	1.00E+00
LOC112578076	4.48E+00	2.84E-18	RSRP1	1.61E-01	1.00E+00	GAPDH	1.08E-01	1.00E+00
LOC112586991	-4.97E+00	2.85E-18	VDR	7.10E-02	1.00E+00	BRPF3	-6.46E-02	1.00E+00
LOC102393700	-4.09E+00	2.96E-18	SYCP2	7.46E-02	1.00E+00	SLC1A4	9.48E-02	1.00E+00
CSNK1G1	8.91E-01	3.07E-18	LOC102393150	-1.51E-01	1.00E+00	LEKR1	7.90E-02	1.00E+00
LOC112582272	2.83E+00	3.11E-18	CNTF	-3.40E-01	1.00E+00	MTERF3	-5.17E-02	1.00E+00
PARP16	7.84E-01	3.14E-18	LOC102413280	-6.84E-02	1.00E+00	VRK1	-6.04E-02	1.00E+00
DUSP15	-2.10E+00	3.14E-18	TM2D3	-5.88E-02	1.00E+00	WDR3	-7.69E-02	1.00E+00
ADPRHL2	-1.62E+00	3.18E-18	CKS1B	-6.11E-02	1.00E+00	AVEN	-6.06E-02	1.00E+00
FBXL8	-3.70E+00	3.24E-18	FAT2	-3.41E-01	1.00E+00	CARF	-8.79E-02	1.00E+00
CEP72	1.30E+00	3.25E-18	DMD	-5.67E-02	1.00E+00	ZNF34	5.21E-02	1.00E+00
PTGES	-5.12E+00	3.28E-18	RAB12	1.30E-01	1.00E+00	FAM173A	-9.31E-02	1.00E+00
FMNL1	3.20E+00	3.29E-18	MAPKAPK3	-7.72E-02	1.00E+00	HABP4	-4.86E-02	1.00E+00
SKIV2L	-8.41E-01	3.31E-18	MOB3A	4.68E-02	1.00E+00	AKIRIN1	-6.45E-02	1.00E+00
MRPL28	-1.50E+00	3.38E-18	SPC25	-5.13E-02	1.00E+00	LSM1	6.62E-02	1.00E+00
LOC112582191	2.12E+00	3.52E-18	ZNF584	-1.30E-01	1.00E+00	SMOX	-6.51E-02	1.00E+00

IRAK1BP1	-1.88E+00		3.53E-18	DCAF12	4.87E-02	1.00E+00	ARTN	-8.98E-02	1.00E+00
LOC102396469	4.05E+00		3.58E-18	PKNOX2	-1.46E-01	1.00E+00	PDCD4	-5.56E-02	1.00E+00
TMEM151A	3.04E+00		3.60E-18	TMIGD2	-1.61E-01	1.00E+00	LOC102402269	-1.14E-01	1.00E+00
RTN1	4.34E+00		3.66E-18	NAP1L4	4.57E-02	1.00E+00	KLHL42	-5.54E-02	1.00E+00
LOC102416288	-1.50E+00		3.73E-18	LCMT2	-7.07E-02	1.00E+00	NOC3L	5.12E-02	1.00E+00
SLAIN2	9.31E-01		3.79E-18	ZBTB10	-7.69E-02	1.00E+00	BOD1L1	-6.61E-02	1.00E+00
SNRPB2	8.15E-01		3.81E-18	KIF5A	-9.50E-02	1.00E+00	SH2B1	4.98E-02	1.00E+00
ABHD18	-1.88E+00		3.87E-18	GOLPH3L	-4.59E-02	1.00E+00	TTC33	-6.98E-02	1.00E+00
TP53I13	-1.52E+00		3.89E-18	SLC38A6	1.73E-01	1.00E+00	PDCD6	4.93E-02	1.00E+00
LOC102415373	-3.41E+00		3.96E-18	LOC112581570	1.23E-01	1.00E+00	SLC26A2	5.72E-02	1.00E+00
LOC102391189	-9.57E-01		3.98E-18	LOC102414144	-2.05E-01	1.00E+00	ATP11B	-6.17E-02	1.00E+00
BRK1	-1.09E+00		4.02E-18	NKX6-2	-6.59E-02	1.00E+00	PEAK1	6.75E-02	1.00E+00
LHB	2.70E+00		4.05E-18	LOC112586458	-1.68E-01	1.00E+00	PPP4R3B	-5.54E-02	1.00E+00
BDNF	2.35E+00		4.08E-18	LOC112586509	-1.43E-01	1.00E+00	NSG2	1.11E-01	1.00E+00
ACHE	-3.63E+00		4.10E-18	KIAA1551	1.08E-01	1.00E+00	MAGEE2	1.23E-01	1.00E+00
ARID2	-1.55E+00		4.11E-18	NRG4	-9.57E-02	1.00E+00	CAPZB	-6.97E-02	1.00E+00
GALNT10	1.22E+00		4.18E-18	ST3GAL5	-1.97E-01	1.00E+00	DENND1B	5.90E-02	1.00E+00
INSC	5.72E+00		4.20E-18	LOC112581132	-9.28E-02	1.00E+00	NLRC5	-1.45E-01	1.00E+00
LOC112581566	4.98E+00		4.22E-18	FAM206A	7.72E-02	1.00E+00	LAMTOR3	5.18E-02	1.00E+00
ELOVL5	9.71E-01		4.25E-18	FKBP14	9.37E-02	1.00E+00	DCLRE1C	8.92E-02	1.00E+00
ZWINT	-1.41E+00		4.37E-18	LOC102394446	-7.22E-02	1.00E+00	LEMD3	-6.88E-02	1.00E+00
LOC102402523	3.20E+00		4.39E-18	KAT2B	9.28E-02	1.00E+00	EIF3H	-5.72E-02	1.00E+00
DYRK1A	-8.40E-01		4.43E-18	LOC112579179	-1.76E-01	1.00E+00	LOC102392521	-8.29E-02	1.00E+00
LOC102398037	4.57E+00		4.53E-18	SLC48A1	-6.28E-02	1.00E+00	INTS10	-5.25E-02	1.00E+00
ZFP42	6.28E+00		4.53E-18	LOC102397135	2.61E-01	1.00E+00	ANKRD13D	-7.51E-02	1.00E+00
ZBTB9	9.64E-01		4.54E-18	OSCP1	-1.22E-01	1.00E+00	C10H6orf203	-8.49E-02	1.00E+00
ZMAT1	-2.51E+00		4.56E-18	GNMT	9.84E-02	1.00E+00	KCTD5	-5.35E-02	1.00E+00
SLC2A10	-5.19E+00		4.59E-18	GATAD2A	5.96E-02	1.00E+00	MSRB2	7.77E-02	1.00E+00
JAM2	-5.71E+00		4.64E-18	CHCHD5	1.13E-01	1.00E+00	CHCHD7	-6.59E-02	1.00E+00
OTP	3.57E+00		4.70E-18	MED9	9.05E-02	1.00E+00	NUP62	-5.26E-02	1.00E+00
DOP1A	1.43E+00		4.72E-18	CHMP3	-5.49E-02	1.00E+00	BLOC1S3	8.24E-02	1.00E+00
PMPCA	-1.08E+00		4.94E-18	LOC112585613	-2.06E-01	1.00E+00	C16H11orf49	-5.84E-02	1.00E+00
MAPKAP1	7.76E-01		5.08E-18	SLC12A4	-1.66E-01	1.00E+00	EIF4H	-6.20E-02	1.00E+00

NCOR1	1.19E+00	5.20E-18	CUBN	3.71E-01	1.00E+00	BTBD2	6.17E-02	1.00E+00
RASSF7	-2.08E+00	5.21E-18	LOC112580628	-1.97E-01	1.00E+00	ERMAP	-1.13E-01	1.00E+00
LOC112578483	-1.88E+00	5.22E-18	UPK1B	-1.10E-01	1.00E+00	CARM1	-4.96E-02	1.00E+00
TMEM86B	-3.62E+00	5.23E-18	SPTLC3	-1.85E-01	1.00E+00	CACHD1	6.47E-02	1.00E+00
CHRM1	4.55E+00	5.25E-18	ALDH1A3	-4.14E-01	1.00E+00	AUH	-5.43E-02	1.00E+00
CSE1L	7.59E-01	5.27E-18	LOC112578811	-6.96E-02	1.00E+00	NDUFA9	-5.34E-02	1.00E+00
GPX1	-1.43E+00	5.32E-18	NPHP4	-2.22E-01	1.00E+00	POLH	-5.57E-02	1.00E+00
GREM1	2.02E+00	5.33E-18	LOC102403587	-1.39E-01	1.00E+00	TMED10	5.31E-02	1.00E+00
FOXQ1	2.80E+00	5.34E-18	CCDC87	-6.51E-02	1.00E+00	VDAC2	5.71E-02	1.00E+00
RHNO1	-1.37E+00	5.40E-18	ENDOD1	-1.09E-01	1.00E+00	PPP1R26	5.85E-02	1.00E+00
METTL13	-1.08E+00	5.49E-18	YAF2	1.02E-01	1.00E+00	ASMTL	-6.71E-02	1.00E+00
SMARCB1	-1.11E+00	5.66E-18	ADNP2	5.90E-02	1.00E+00	PSMD3	-5.72E-02	1.00E+00
MCF2L2	-1.63E+00	5.66E-18	ATP2C2	6.76E-02	1.00E+00	ANKRD13C	5.94E-02	1.00E+00
HJURP	1.17E+00	5.67E-18	LRRFIP2	-5.00E-02	1.00E+00	LOC102400568	7.10E-02	1.00E+00
PORCN	-1.42E+00	5.69E-18	PBLD	-7.99E-02	1.00E+00	ARHGAP18	6.42E-02	1.00E+00
LOC102392047	-1.57E+00	5.73E-18	KIAA1958	7.99E-02	1.00E+00	LOC102407804	8.38E-02	1.00E+00
LOC102394431	1.56E+00	5.82E-18	KDM4C	-6.27E-02	1.00E+00	LIN52	-5.78E-02	1.00E+00
ATP5MD	-1.27E+00	5.82E-18	LRRC15	-2.18E-01	1.00E+00	LOC102416515	-9.13E-02	1.00E+00
LOC112579549	4.55E+00	5.82E-18	ZCCHC17	-6.46E-02	1.00E+00	SMAP1	-4.63E-02	1.00E+00
ZNF354A	8.11E-01	5.86E-18	RIN1	-1.63E-01	1.00E+00	SPACA6	-7.61E-02	1.00E+00
ANKRD45	-1.71E+00	5.93E-18	LOC102412461	4.58E-02	1.00E+00	SLC2A3	8.76E-02	1.00E+00
LOC102412583	-3.99E+00	5.99E-18	PROCR	-1.99E-01	1.00E+00	TAB3	-6.34E-02	1.00E+00
MYO1C	-1.23E+00	5.99E-18	TMEM43	4.88E-02	1.00E+00	ALB	1.23E-01	1.00E+00
KCNK6	3.08E+00	6.04E-18	CCDC107	-8.00E-02	1.00E+00	BRWD3	-7.21E-02	1.00E+00
LOC112585727	-3.11E+00	6.05E-18	NOS3	-4.27E-01	1.00E+00	GTF2H5	-6.76E-02	1.00E+00
PRR13	9.63E-01	6.11E-18	GCA	8.63E-02	1.00E+00	TMEM47	5.31E-02	1.00E+00
AGPS	8.64E-01	6.20E-18	LOC102399179	-1.09E-01	1.00E+00	RUBCNL	-2.06E-01	1.00E+00
INKA2	2.19E+00	6.24E-18	LOC112580677	1.46E-01	1.00E+00	FSD1L	-1.01E-01	1.00E+00
EPHA7	2.20E+00	6.30E-18	LOC112578030	-2.82E-01	1.00E+00	GPS2	-6.01E-02	1.00E+00
MAMSTR	1.94E+00	6.31E-18	LOC102394124	8.43E-02	1.00E+00	ISG15	6.89E-02	1.00E+00
GRIA1	-1.90E+00	6.35E-18	LOC102389719	6.41E-02	1.00E+00	LOC102391847	1.05E-01	1.00E+00
CCDC71L	-1.93E+00	6.37E-18	KAT6A	-9.11E-02	1.00E+00	LOC102408093	6.18E-02	1.00E+00
ANKRD55	-2.87E+00	6.47E-18	FAM217B	-2.40E-01	1.00E+00	LOC102408709	-5.38E-02	1.00E+00

SLC2A3	-3.82E+00	6.49E-18	SLC9A6	-4.98E-02	1.00E+00	SOD1	-6.13E-02	1.00E+00
ACSS3	-1.19E+00	6.55E-18	AKAP10	5.24E-02	1.00E+00	JMJD7	-1.04E-01	1.00E+00
C20H14orf28	-2.57E+00	6.69E-18	LYPD5	-1.64E-01	1.00E+00	CREB1	5.37E-02	1.00E+00
PRR29	3.38E+00	6.72E-18	ZPBP	-1.20E-01	1.00E+00	EIF5A2	7.25E-02	1.00E+00
SPINDOC	-9.26E-01	6.72E-18	TSPAN5	-4.67E-02	1.00E+00	TLK2	4.87E-02	1.00E+00
HIKESHI	-1.16E+00	6.74E-18	SDHA	-4.83E-02	1.00E+00	REEP5	-4.99E-02	1.00E+00
DFFA	-8.43E-01	6.86E-18	GPR155	9.83E-02	1.00E+00	LOC112578567	9.54E-02	1.00E+00
WWOX	1.25E+00	6.95E-18	LOC102398505	-2.43E-01	1.00E+00	RNF146	4.68E-02	1.00E+00
MFSD3	-1.82E+00	6.97E-18	ANKRD35	-1.26E-01	1.00E+00	PIK3CA	1.05E-01	1.00E+00
OTOGL	4.05E+00	7.06E-18	NDUFV2	-5.51E-02	1.00E+00	LOC102404764	-1.19E-01	1.00E+00
LOC102392019	-5.78E+00	7.17E-18	LTBP1	4.83E-02	1.00E+00	VPS41	4.98E-02	1.00E+00
PNN	-8.97E-01	7.20E-18	COL1A1	-2.68E-01	1.00E+00	METTL8	6.04E-02	1.00E+00
LLGL1	-1.14E+00	7.23E-18	APPL1	9.54E-02	1.00E+00	PCBP2	-6.04E-02	1.00E+00
ZNF699	2.11E+00	7.35E-18	SPTSSA	1.70E-01	1.00E+00	PHKB	-5.72E-02	1.00E+00
LOC112578161	5.01E+00	7.42E-18	LTBP2	2.61E-01	1.00E+00	TMOD1	6.85E-02	1.00E+00
LOC112587058	6.96E+00	7.60E-18	SCRIB	-7.11E-02	1.00E+00	MAPRE3	5.50E-02	1.00E+00
ITPRIP	1.40E+00	7.64E-18	WARS	5.38E-02	1.00E+00	MYBBP1A	-4.93E-02	1.00E+00
AADACL3	1.72E+00	7.71E-18	MAP3K11	9.14E-02	1.00E+00	ABCC10	-5.64E-02	1.00E+00
LOC112587492	-5.15E+00	7.76E-18	NPHS2	1.45E-01	1.00E+00	LOC102395706	6.43E-02	1.00E+00
LMTK3	1.63E+00	7.81E-18	CABP7	1.00E-01	1.00E+00	AP1AR	-6.64E-02	1.00E+00
GRXCR1	-3.21E+00	7.81E-18	LOC112584678	2.07E-01	1.00E+00	ATXN7L3B	4.99E-02	1.00E+00
LOC112585093	4.38E+00	7.85E-18	BTBD7	-1.40E-01	1.00E+00	TACC2	5.90E-02	1.00E+00
LOC112585163	-1.62E+00	7.85E-18	CIT	6.10E-02	1.00E+00	VDAC1	-5.40E-02	1.00E+00
ANAPC2	-1.10E+00	7.86E-18	GMNN	-5.85E-02	1.00E+00	CDH24	-6.46E-02	1.00E+00
USO1	1.00E+00	8.06E-18	UGDH	-1.07E-01	1.00E+00	LOC102399270	8.61E-02	1.00E+00
KCTD2	7.53E-01	8.06E-18	ATP6V1G1	-6.87E-02	1.00E+00	CNST	-6.10E-02	1.00E+00
PRAG1	3.62E+00	8.12E-18	TACC2	-1.97E-01	1.00E+00	MAP3K4	-4.59E-02	1.00E+00
ARHGAP12	1.00E+00	8.14E-18	NBAS	6.03E-02	1.00E+00	ZNF219	5.63E-02	1.00E+00
TMEM88	-3.64E+00	8.14E-18	ZNF692	-9.23E-02	1.00E+00	SPHK2	4.70E-02	1.00E+00
MXD3	-1.82E+00	8.15E-18	HK1	5.44E-02	1.00E+00	ACP6	-7.07E-02	1.00E+00
LOC112581262	3.54E+00	8.19E-18	MESD	5.75E-02	1.00E+00	THADA	-5.21E-02	1.00E+00
NT5E	1.68E+00	8.27E-18	LOC102392521	-1.47E-01	1.00E+00	YARS2	5.48E-02	1.00E+00
ZNF577	1.43E+00	8.27E-18	PTGES	4.63E-01	1.00E+00	P3H4	6.93E-02	1.00E+00

MED7	1.06E+00	8.29E-18	SNRPF	-5.61E-02	1.00E+00	FEM1B	4.88E-02	1.00E+00
TMEM33	1.27E+00	8.31E-18	CDK6	9.77E-02	1.00E+00	SPINDOC	-4.96E-02	1.00E+00
LOC102410635	3.08E+00	8.37E-18	LOC102408379	-6.73E-02	1.00E+00	KDM4B	4.63E-02	1.00E+00
LOC102392085	-1.21E+00	8.41E-18	DISC1	-7.67E-02	1.00E+00	GRXCR1	9.59E-02	1.00E+00
SRA1	-1.19E+00	8.51E-18	CCDC174	1.65E-01	1.00E+00	LOC102404009	4.76E-02	1.00E+00
FAM189A2	1.18E+00	8.55E-18	C22H18orf21	-7.80E-02	1.00E+00	THAP4	5.83E-02	1.00E+00
ACOT8	-1.20E+00	8.64E-18	GFM2	-5.74E-02	1.00E+00	ELMSAN1	-7.35E-02	1.00E+00
SCNN1G	5.60E+00	8.67E-18	ETFDH	6.44E-02	1.00E+00	ANAPC15	-7.75E-02	1.00E+00
NDUFV3	8.23E-01	8.76E-18	SUPT16H	-9.10E-02	1.00E+00	CLGN	-5.82E-02	1.00E+00
LOC102391382	-1.66E+00	8.85E-18	PI4K2A	-5.87E-02	1.00E+00	MID1	8.87E-02	1.00E+00
LOC112580609	4.69E+00	8.85E-18	STK17B	-2.95E-01	1.00E+00	DENND5B	5.23E-02	1.00E+00
NDUFA4	-1.44E+00	8.85E-18	LOC102415561	1.53E-01	1.00E+00	COMTD1	7.58E-02	1.00E+00
LOC112579531	-6.93E+00	8.95E-18	C16H11orf16	5.17E-02	1.00E+00	LOC112577761	1.17E-01	1.00E+00
GDPD1	-1.27E+00	8.97E-18	MYNN	-6.87E-02	1.00E+00	TIMM8A	5.89E-02	1.00E+00
LOC102399479	-5.03E+00	9.06E-18	FNDC8	2.03E-01	1.00E+00	FBXO3	5.40E-02	1.00E+00
LOC102412844	1.37E+00	9.30E-18	NDUFA4	-6.78E-02	1.00E+00	ATP6V0A1	5.00E-02	1.00E+00
VWA7	1.96E+00	9.30E-18	JPH1	-6.17E-02	1.00E+00	RAD18	4.98E-02	1.00E+00
GMEB1	-1.18E+00	9.34E-18	SLC5A1	4.89E-02	1.00E+00	ARL9	-6.94E-02	1.00E+00
PEX13	-1.56E+00	9.56E-18	ARL4D	-1.63E-01	1.00E+00	TUBGCP6	-5.09E-02	1.00E+00
GPALPP1	-1.20E+00	9.62E-18	HMGXB4	7.67E-02	1.00E+00	PSMB5	6.68E-02	1.00E+00
ASXL3	1.74E+00	9.80E-18	THUMPD1	5.83E-02	1.00E+00	SENP3	-4.53E-02	1.00E+00
PADI1	4.29E+00	9.80E-18	LOC112578454	7.94E-02	1.00E+00	LOC102389829	8.56E-02	1.00E+00
LOC112587796	3.72E+00	9.83E-18	LOC102404294	4.53E-02	1.00E+00	RNF103	5.65E-02	1.00E+00
EFHB	1.44E+00	9.93E-18	SLC7A14	1.30E-01	1.00E+00	EEF1AKMT2	-6.10E-02	1.00E+00
ENO4	1.65E+00	9.95E-18	ING3	9.67E-02	1.00E+00	PDCD2L	-6.42E-02	1.00E+00
LOC102415264	-4.00E+00	1.00E-17	PPP1R11	6.21E-02	1.00E+00	EEA1	-9.40E-02	1.00E+00
EPRS	-7.82E-01	1.02E-17	LOC102406196	-3.52E-01	1.00E+00	PPP1R3E	7.21E-02	1.00E+00
CCDC3	-1.46E+00	1.02E-17	FOXP3	-2.28E-01	1.00E+00	LINS1	6.24E-02	1.00E+00
ST8SIA2	5.78E+00	1.03E-17	VPS36	1.17E-01	1.00E+00	CBX3	-5.12E-02	1.00E+00
LOC112580246	-3.80E+00	1.05E-17	TMEM262	-2.36E-01	1.00E+00	ATP5MF	7.85E-02	1.00E+00
ICAM3	-2.14E+00	1.05E-17	LRRC8D	1.22E-01	1.00E+00	ADGRV1	-6.14E-02	1.00E+00
TRIM8	1.03E+00	1.06E-17	PUS1	6.15E-02	1.00E+00	UBTD2	5.96E-02	1.00E+00
DMAP1	-1.20E+00	1.06E-17	NAXD	-4.59E-02	1.00E+00	ANP32E	-4.56E-02	1.00E+00

ZNF614	-1.65E+00	1.07E-17	LOC102413919	2.64E-01	1.00E+00	EHMT1	-4.95E-02	1.00E+00
PYM1	1.26E+00	1.08E-17	CHPF	5.07E-02	1.00E+00	MALSU1	-6.99E-02	1.00E+00
TRIM17	2.81E+00	1.08E-17	INCENP	-4.38E-02	1.00E+00	MAZ	-6.24E-02	1.00E+00
GLRB	-4.21E+00	1.08E-17	GUK1	-5.83E-02	1.00E+00	ZNF205	5.89E-02	1.00E+00
LOC102389267	-3.66E+00	1.09E-17	LRGUK	-2.61E-01	1.00E+00	ZNF641	-9.18E-02	1.00E+00
PLA2G7	2.16E+00	1.10E-17	FAM180A	2.74E-01	1.00E+00	TTC7B	-5.65E-02	1.00E+00
LOC112579072	-2.94E+00	1.11E-17	LOC102395706	-9.90E-02	1.00E+00	EDEM2	5.10E-02	1.00E+00
SAMD7	6.10E+00	1.12E-17	SMIM13	-1.56E-01	1.00E+00	ASXL2	7.52E-02	1.00E+00
SLC38A4	-4.98E+00	1.14E-17	MXI1	5.18E-02	1.00E+00	LOC102399010	1.21E-01	1.00E+00
PTGIS	2.72E+00	1.18E-17	RAD50	5.27E-02	1.00E+00	SP3	-6.11E-02	1.00E+00
PCLO	4.65E+00	1.20E-17	SKIDA1	-2.86E-01	1.00E+00	DOK5	-6.97E-02	1.00E+00
ATG16L2	-1.56E+00	1.22E-17	LOC102395276	-4.91E-02	1.00E+00	PSMG3	7.15E-02	1.00E+00
ENDOV	8.60E-01	1.23E-17	RAB5B	5.02E-02	1.00E+00	AP1G2	-5.29E-02	1.00E+00
CEP57	-1.15E+00	1.25E-17	CFAP77	-2.47E-01	1.00E+00	FAM118B	-4.55E-02	1.00E+00
LOC102402635	4.34E+00	1.31E-17	SOX12	-8.51E-02	1.00E+00	PLEKHA1	7.34E-02	1.00E+00
LOC112584702	-3.42E+00	1.34E-17	FRMPD3	-2.04E-01	1.00E+00	GIT1	4.53E-02	1.00E+00
AKT2	-8.06E-01	1.34E-17	RPS19BP1	-8.35E-02	1.00E+00	ZFC3H1	-6.02E-02	1.00E+00
LYVE1	-6.91E+00	1.42E-17	PRTFDC1	3.09E-01	1.00E+00	TMEM8A	-5.39E-02	1.00E+00
ANGEL1	9.23E-01	1.42E-17	RNF17	-5.91E-02	1.00E+00	TBX19	-5.54E-02	1.00E+00
CPXM2	3.92E+00	1.43E-17	ANKRD53	-1.12E-01	1.00E+00	DDRGK1	-6.86E-02	1.00E+00
IK	9.52E-01	1.44E-17	LOC102397338	-8.84E-02	1.00E+00	WDR18	5.11E-02	1.00E+00
LOC112584414	2.41E+00	1.46E-17	LIMCH1	5.93E-02	1.00E+00	IER3	8.39E-02	1.00E+00
SLC39A14	-1.20E+00	1.46E-17	ZWILCH	4.55E-02	1.00E+00	GALE	-5.42E-02	1.00E+00
MYOM3	-3.41E+00	1.48E-17	DDO	1.52E-01	1.00E+00	PRDM15	6.82E-02	1.00E+00
STUM	-4.26E+00	1.52E-17	ARL6	9.74E-02	1.00E+00	LAMTOR4	7.24E-02	1.00E+00
DEUP1	2.00E+00	1.53E-17	LOC102408113	-2.59E-01	1.00E+00	TCF12	6.30E-02	1.00E+00
FAS	-5.73E+00	1.56E-17	FOCAD	6.85E-02	1.00E+00	PRRC2C	-7.33E-02	1.00E+00
PHLDA1	-2.17E+00	1.59E-17	RCN1	9.63E-02	1.00E+00	CDC37	5.79E-02	1.00E+00
LOC102410187	-4.10E+00	1.65E-17	KIF15	9.06E-02	1.00E+00	TNKS2	7.10E-02	1.00E+00
LOC112587556	2.38E+00	1.67E-17	NUDT6	4.45E-02	1.00E+00	TRIM44	4.95E-02	1.00E+00
IL10RA	-2.62E+00	1.68E-17	MBD4	-4.53E-02	1.00E+00	PDSS2	5.52E-02	1.00E+00
PROM1	-4.40E+00	1.69E-17	BMPR1B	5.55E-02	1.00E+00	TSSK2	-1.12E-01	1.00E+00
ANXA10	-4.49E+00	1.70E-17	GOLM1	-6.22E-02	1.00E+00	ADAMTS19	-5.17E-02	1.00E+00

MAB21L3	3.94E+00	1.80E-17	MCM10	5.70E-02	1.00E+00	WFIKKN1	-1.07E-01	1.00E+00
BAD	-1.42E+00	1.83E-17	LOC102404009	1.32E-01	1.00E+00	UBE2L3	-5.08E-02	1.00E+00
CXHXorf65	6.46E+00	1.84E-17	GPRC5D	-1.22E-01	1.00E+00	UBE2V2	-4.81E-02	1.00E+00
CNN3	-1.26E+00	1.85E-17	SF3B5	-8.14E-02	1.00E+00	KANSL1L	7.74E-02	1.00E+00
ARHGEF10L	-9.17E-01	1.90E-17	PTP4A2	4.39E-02	1.00E+00	ITGB3BP	9.87E-02	1.00E+00
LOC112580915	3.63E+00	1.91E-17	MAPK13	-7.51E-02	1.00E+00	DTYMK	8.02E-02	1.00E+00
MLLT1	7.95E-01	1.93E-17	TTC16	1.55E-01	1.00E+00	LOC102398188	9.15E-02	1.00E+00
KDELR3	-1.03E+00	1.94E-17	SRI	-6.82E-02	1.00E+00	LOC102415205	1.16E-01	1.00E+00
LOC112581451	2.08E+00	1.94E-17	FAM111B	-2.10E-01	1.00E+00	DBF4	-5.36E-02	1.00E+00
LOC112578656	3.89E+00	1.98E-17	TPCN1	-5.25E-02	1.00E+00	SPINK13	-1.03E-01	1.00E+00
ADAMTS3	-4.47E+00	1.98E-17	FBF1	1.02E-01	1.00E+00	NPTX1	1.14E-01	1.00E+00
PPP1R15A	-2.37E+00	2.04E-17	PRSS53	5.45E-02	1.00E+00	JTB	6.30E-02	1.00E+00
LOC102406090	-2.71E+00	2.08E-17	ZNF784	-2.17E-01	1.00E+00	IL17RC	-6.09E-02	1.00E+00
LCA5	2.06E+00	2.08E-17	P4HB	5.23E-02	1.00E+00	PGM3	5.11E-02	1.00E+00
LOC112587006	-3.62E+00	2.11E-17	CORO1C	-7.77E-02	1.00E+00	C3H17orf58	8.33E-02	1.00E+00
CHFR	7.17E-01	2.12E-17	ZNF408	6.45E-02	1.00E+00	AMN1	-5.35E-02	1.00E+00
CCNE1	1.09E+00	2.15E-17	SCOC	7.37E-02	1.00E+00	LOC102411804	5.80E-02	1.00E+00
CSRP3	-7.27E+00	2.16E-17	RNASEK	-8.38E-02	1.00E+00	CCDC51	5.78E-02	1.00E+00
LOC102414630	-3.62E+00	2.18E-17	PARM1	4.00E-01	1.00E+00	EIF2B2	-5.30E-02	1.00E+00
LOC112581781	-3.35E+00	2.22E-17	SMAP1	5.73E-02	1.00E+00	TMEM62	6.27E-02	1.00E+00
DPM3	-1.45E+00	2.24E-17	PHLDB3	1.05E-01	1.00E+00	RCE1	5.49E-02	1.00E+00
FNTA	-8.40E-01	2.28E-17	PRMT3	-1.03E-01	1.00E+00	PNMA2	1.35E-01	1.00E+00
CFAP161	-2.93E+00	2.29E-17	ZNF467	1.27E-01	1.00E+00	TMEM30A	5.44E-02	1.00E+00
PRAM1	-2.23E+00	2.29E-17	TEX9	-6.48E-02	1.00E+00	OSBPL7	5.11E-02	1.00E+00
LOC102391462	5.53E+00	2.31E-17	RXRB	-6.46E-02	1.00E+00	DEAF1	6.22E-02	1.00E+00
LOC112586103	-3.60E+00	2.38E-17	MTERF4	-6.33E-02	1.00E+00	ARFGAP1	5.77E-02	1.00E+00
GLDC	1.19E+00	2.38E-17	FAM104A	8.54E-02	1.00E+00	TRIM28	-6.63E-02	1.00E+00
MBOAT7	-9.30E-01	2.42E-17	ZNF624	8.18E-02	1.00E+00	NUP155	4.89E-02	1.00E+00
AK3	-8.68E-01	2.43E-17	LOC112578889	-2.14E-01	1.00E+00	LOC102415348	-7.35E-02	1.00E+00
INTU	-1.60E+00	2.44E-17	LOC112584706	-1.95E-01	1.00E+00	LOC112584700	1.14E-01	1.00E+00
RTN2	-1.39E+00	2.46E-17	LOC102390919	1.38E-01	1.00E+00	NOL9	-5.90E-02	1.00E+00
ABCC8	-2.96E+00	2.48E-17	ZNF37A	-7.43E-02	1.00E+00	CDC123	5.00E-02	1.00E+00
ZNF263	-1.44E+00	2.56E-17	KCNIP1	-2.05E-01	1.00E+00	PLCG2	1.21E-01	1.00E+00

HCFC2	1.25E+00	2.57E-17	EPHX3	-8.01E-02	1.00E+00	ROMO1	7.71E-02	1.00E+00
LOC112582052	4.40E+00	2.57E-17	PRDM8	-1.54E-01	1.00E+00	NDUFB6	6.37E-02	1.00E+00
WAS	4.07E+00	2.58E-17	LOC102411167	-1.03E-01	1.00E+00	DDX39A	-6.42E-02	1.00E+00
PPP1R37	-8.98E-01	2.72E-17	FN1	-7.75E-02	1.00E+00	PBDC1	6.07E-02	1.00E+00
RIOK2	-1.01E+00	2.72E-17	RAP2B	1.24E-01	1.00E+00	CCDC65	-9.67E-02	1.00E+00
DTX4	-4.48E+00	2.73E-17	GCDH	-8.80E-02	1.00E+00	TTF1	5.04E-02	1.00E+00
LOC102393384	9.94E-01	2.78E-17	TOM1L2	9.97E-02	1.00E+00	OXNAD1	4.66E-02	1.00E+00
TIPIN	-8.49E-01	2.78E-17	ARHGAP27	-1.74E-01	1.00E+00	LOC102402708	5.25E-02	1.00E+00
SP8	5.50E+00	2.79E-17	MAN2A2	1.81E-01	1.00E+00	KLC4	-5.01E-02	1.00E+00
LOC112583725	3.59E+00	2.81E-17	STK36	6.41E-02	1.00E+00	C11H15orf48	1.47E-01	1.00E+00
UPF3B	-1.02E+00	2.82E-17	LAP3	-5.23E-02	1.00E+00	EXOSC9	-5.27E-02	1.00E+00
CELF1	8.26E-01	2.86E-17	ETF1	-6.59E-02	1.00E+00	AMOTL2	-7.40E-02	1.00E+00
DDX46	-8.34E-01	2.88E-17	LOC112581891	-1.77E-01	1.00E+00	LOC102413667	1.19E-01	1.00E+00
LOC112585310	-4.23E+00	2.91E-17	YLPM1	7.19E-02	1.00E+00	CPT2	4.55E-02	1.00E+00
LOC102413645	-5.87E+00	2.91E-17	PRICKLE3	3.27E-01	1.00E+00	HEBP2	8.40E-02	1.00E+00
GPX3	-1.23E+00	2.94E-17	ABHD4	4.46E-02	1.00E+00	RELCH	5.48E-02	1.00E+00
ZCCHC8	-1.12E+00	2.97E-17	IREB2	7.34E-02	1.00E+00	LOC102412869	-8.83E-02	1.00E+00
DCAF8	8.43E-01	3.02E-17	KLHDC8B	-3.58E-01	1.00E+00	RNPEPL1	5.63E-02	1.00E+00
ASPSCR1	-1.22E+00	3.02E-17	CNBP	-4.75E-02	1.00E+00	NDUFS4	-6.88E-02	1.00E+00
MYOM2	-3.25E+00	3.03E-17	CIAO2A	-1.08E-01	1.00E+00	N4BP2L1	-5.66E-02	1.00E+00
PTPN22	-3.21E+00	3.03E-17	COPZ2	-7.41E-02	1.00E+00	UBE2N	4.60E-02	1.00E+00
PRLR	-2.44E+00	3.04E-17	PPM1G	4.43E-02	1.00E+00	ZMAT3	5.69E-02	1.00E+00
LOC112581768	-2.88E+00	3.05E-17	LOC112578933	1.91E-01	1.00E+00	LOC112581450	-7.44E-02	1.00E+00
UBE2I	9.16E-01	3.14E-17	RNPC3	6.43E-02	1.00E+00	PTTG1	-7.37E-02	1.00E+00
C21H3orf22	-3.82E+00	3.15E-17	LOC102393048	4.39E-01	1.00E+00	TIE1	-1.02E-01	1.00E+00
PITX3	2.31E+00	3.16E-17	TMEM150A	4.84E-02	1.00E+00	HPS3	-8.32E-02	1.00E+00
C23H10orf90	4.23E+00	3.17E-17	PRPF38B	7.52E-02	1.00E+00	TMEM109	4.90E-02	1.00E+00
CDIPT	-1.25E+00	3.23E-17	ANKRD42	-7.10E-02	1.00E+00	RGS22	7.25E-02	1.00E+00
CROCC2	2.40E+00	3.27E-17	SUSD6	6.70E-02	1.00E+00	LOC102402993	-2.24E-01	1.00E+00
NKX1-2	4.41E+00	3.29E-17	XK	1.18E-01	1.00E+00	MRPL50	6.21E-02	1.00E+00
COG1	-9.12E-01	3.31E-17	MYCBPAP	-1.47E-01	1.00E+00	NDUFA8	6.09E-02	1.00E+00
DUOX1	-4.70E+00	3.34E-17	TNFSF11	1.76E-01	1.00E+00	MRPL39	-4.75E-02	1.00E+00
LOC102409034	4.67E+00	3.38E-17	PHRF1	5.81E-02	1.00E+00	EPB41	4.93E-02	1.00E+00
PREP	-1.20E+00	3.38E-17	ABHD10	-6.93E-02	1.00E+00	NAB1	6.24E-02	1.00E+00

ME3	4.09E+00	3.39E-17	PHETA1	-1.02E-01	1.00E+00	MRPS14	-6.30E-02	1.00E+00
ABCF1	1.01E+00	3.40E-17	BORCS6	1.51E-01	1.00E+00	ZNF462	-5.97E-02	1.00E+00
CASZ1	-1.98E+00	3.42E-17	PLGRKT	-1.01E-01	1.00E+00	MAPK13	8.33E-02	1.00E+00
LOC102400587	1.63E+00	3.43E-17	DNAJC16	6.12E-02	1.00E+00	LOC112581440	-1.10E-01	1.00E+00
ACVR1B	-1.69E+00	3.44E-17	KIF13A	-1.05E-01	1.00E+00	SDF2	5.40E-02	1.00E+00
MEGF6	-4.97E+00	3.46E-17	NPTXR	1.76E-01	1.00E+00	CHD4	7.99E-02	1.00E+00
TCF7	-1.14E+00	3.48E-17	LMX1B	-5.73E-02	1.00E+00	TACR2	1.30E-01	1.00E+00
TOMM22	-1.15E+00	3.49E-17	MDN1	9.76E-02	1.00E+00	TRAF2	5.75E-02	1.00E+00
ATG9B	-2.10E+00	3.57E-17	TXNRD3	-8.69E-02	1.00E+00	REV3L	6.34E-02	1.00E+00
CDH11	-3.09E+00	3.60E-17	SLC35A5	6.28E-02	1.00E+00	PTCD1	5.62E-02	1.00E+00
GRP	3.31E+00	3.61E-17	CSPG5	-7.03E-02	1.00E+00	CEP76	-6.91E-02	1.00E+00
EXT2	-7.53E-01	3.61E-17	SPECC1	-8.77E-02	1.00E+00	FOXO1	-6.53E-02	1.00E+00
DEXI	-1.16E+00	3.66E-17	LOC102391440	-1.59E-01	1.00E+00	CLDN12	-6.61E-02	1.00E+00
IFRD1	-1.66E+00	3.66E-17	DUSP16	5.56E-02	1.00E+00	LOC112585524	-1.19E-01	1.00E+00
LOC112584679	2.84E+00	3.66E-17	MFSD11	5.38E-02	1.00E+00	ECD	4.69E-02	1.00E+00
PANX2	-4.68E+00	3.80E-17	FAM216A	-1.18E-01	1.00E+00	LOC102406175	-7.88E-02	1.00E+00
NR4A1	-2.25E+00	3.84E-17	EML5	-8.60E-02	1.00E+00	LOC102397524	-9.52E-02	1.00E+00
LOC102415561	2.55E+00	4.01E-17	MYH15	5.51E-02	1.00E+00	CHUK	-5.77E-02	1.00E+00
ZNF276	-9.90E-01	4.02E-17	LOC102416345	1.02E-01	1.00E+00	BTRC	4.91E-02	1.00E+00
LOC102409836	-4.72E+00	4.02E-17	CCSER1	-6.84E-02	1.00E+00	WDR45	-4.86E-02	1.00E+00
ATAD1	-9.01E-01	4.05E-17	LOC112580864	4.22E-01	1.00E+00	APEX1	5.32E-02	1.00E+00
LOC102415914	4.79E+00	4.09E-17	BYSL	4.40E-02	1.00E+00	ZNF438	6.85E-02	1.00E+00
FBXO38	1.51E+00	4.10E-17	MCPH1	-4.43E-02	1.00E+00	ITGB5	-6.56E-02	1.00E+00
MMP17	-1.96E+00	4.21E-17	LIFR	-1.20E-01	1.00E+00	PLK3	-5.93E-02	1.00E+00
RRP1	9.92E-01	4.25E-17	CRAMP1	6.47E-02	1.00E+00	LOC102398046	-1.08E-01	1.00E+00
LOC112578933	3.39E+00	4.27E-17	LOC112581585	2.64E-01	1.00E+00	RFXANK	-5.43E-02	1.00E+00
FIBIN	-5.94E+00	4.28E-17	LOC102389548	-5.62E-02	1.00E+00	AK2	-5.08E-02	1.00E+00
ZFYVE9	8.32E-01	4.37E-17	GDE1	-7.04E-02	1.00E+00	PSMG2	4.94E-02	1.00E+00
LOC102407446	4.77E+00	4.37E-17	MRPL53	-6.85E-02	1.00E+00	PSMA3	5.26E-02	1.00E+00
CCT8	1.03E+00	4.44E-17	DNTTIP2	4.67E-02	1.00E+00	MCRS1	5.04E-02	1.00E+00
NOP56	-1.25E+00	4.47E-17	BAZ2B	-8.78E-02	1.00E+00	NADK	4.73E-02	1.00E+00
LOC102414187	-7.92E-01	4.56E-17	AQP6	-2.61E-01	1.00E+00	VPS29	4.44E-02	1.00E+00
KIFAP3	8.02E-01	4.62E-17	ZNF26	-1.48E-01	1.00E+00	CALM3	-7.83E-02	1.00E+00

NOX4	-4.00E+00	4.64E-17	C3H9orf72	6.48E-02	1.00E+00	RGL2	-4.71E-02	1.00E+00
SCAF4	-1.05E+00	4.74E-17	IL17D	7.84E-02	1.00E+00	CROT	5.75E-02	1.00E+00
ACADS	-1.03E+00	4.75E-17	CTSO	9.91E-02	1.00E+00	ATP5F1B	6.78E-02	1.00E+00
PGM5	3.17E+00	4.78E-17	CD47	-9.16E-02	1.00E+00	LOC112585054	9.79E-02	1.00E+00
PCDHB5	-3.67E+00	4.78E-17	LOC112580467	1.63E-01	1.00E+00	LAMC1	5.19E-02	1.00E+00
LOC102403795	-1.03E+00	4.79E-17	PIK3CA	-1.11E-01	1.00E+00	LOC102397977	1.48E-01	1.00E+00
BCAP31	-1.25E+00	4.79E-17	SEMA3G	5.78E-02	1.00E+00	PDE6C	-1.07E-01	1.00E+00
RAMP2	-4.14E+00	4.83E-17	PTX4	2.36E-01	1.00E+00	LOC102395807	4.87E-02	1.00E+00
GCFC2	-1.26E+00	4.85E-17	AIMP2	5.26E-02	1.00E+00	TET1	-6.34E-02	1.00E+00
IGF2	-6.60E+00	4.92E-17	ASB9	4.81E-01	1.00E+00	KIAA0232	5.12E-02	1.00E+00
SEMA7A	-1.42E+00	4.95E-17	GTF2A1	1.23E-01	1.00E+00	UBTF	4.91E-02	1.00E+00
PTGDR2	3.93E+00	4.95E-17	RNF7	-8.13E-02	1.00E+00	ECHDC1	-5.47E-02	1.00E+00
WDR19	9.04E-01	4.97E-17	ANKAR	2.42E-01	1.00E+00	SELENOM	-7.75E-02	1.00E+00
LOC112581562	3.20E+00	5.04E-17	LOC112579513	2.64E-01	1.00E+00	HENMT1	9.64E-02	1.00E+00
C5AR2	-4.53E+00	5.05E-17	TUSC1	-1.17E-01	1.00E+00	TMCC1	5.04E-02	1.00E+00
MSRB2	-1.95E+00	5.07E-17	UBR3	8.44E-02	1.00E+00	KIF5B	6.61E-02	1.00E+00
SLC23A1	2.18E+00	5.07E-17	GABRG2	8.53E-02	1.00E+00	XBP1	-7.58E-02	1.00E+00
LOC112582918	3.22E+00	5.08E-17	ICAM3	-2.03E-01	1.00E+00	ARF1	-5.61E-02	1.00E+00
TMEM260	-1.19E+00	5.14E-17	MRPL52	-6.64E-02	1.00E+00	OSMR	1.05E-01	1.00E+00
TMX3	-1.44E+00	5.18E-17	LOC102397454	-7.85E-02	1.00E+00	PRG4	-1.23E-01	1.00E+00
ALDH1L1	-4.10E+00	5.21E-17	LOC102403234	4.28E-01	1.00E+00	DGUOK	4.44E-02	1.00E+00
PHTF2	-1.40E+00	5.34E-17	LOC102395458	3.85E-01	1.00E+00	WDR54	-7.48E-02	1.00E+00
UROD	-1.39E+00	5.35E-17	STK25	-6.68E-02	1.00E+00	LOC102397476	9.26E-02	1.00E+00
SLC14A1	-3.46E+00	5.38E-17	MYO7A	-1.40E-01	1.00E+00	AXIN1	4.66E-02	1.00E+00
RASAL2	1.14E+00	5.41E-17	CFAP97D1	-2.61E-01	1.00E+00	CNOT4	-5.08E-02	1.00E+00
KCNK3	-5.10E+00	5.42E-17	E2F7	-1.34E-01	1.00E+00	NDUFV2	-5.63E-02	1.00E+00
ZSCAN23	-1.12E+00	5.44E-17	THAP4	6.11E-02	1.00E+00	MBNL3	-1.07E-01	1.00E+00
PLPBP	-9.10E-01	5.60E-17	PPP1R9B	7.78E-02	1.00E+00	ZSCAN20	7.02E-02	1.00E+00
SNX9	1.02E+00	5.62E-17	AP4E1	-1.30E-01	1.00E+00	CADPS	7.50E-02	1.00E+00
MMP2	-3.75E+00	5.63E-17	COMMD1	1.12E-01	1.00E+00	UBTD1	5.80E-02	1.00E+00
PPIE	-1.52E+00	5.72E-17	BET1L	8.75E-02	1.00E+00	LOC112585078	-7.98E-02	1.00E+00
BRI3	-1.34E+00	5.73E-17	OAZ2	-4.58E-02	1.00E+00	SUN2	5.42E-02	1.00E+00
MAP2K2	-9.62E-	5.77E-17	KLF6	9.64E-02	1.00E+00	LOC102390113	-5.54E-	1.00E+00

	01						02	
CCDC92B	-3.98E+00	5.79E-17	ZC3H10	-6.26E-02	1.00E+00	TRMU	6.03E-02	1.00E+00
DLD	-7.65E-01	5.83E-17	UNC5CL	-1.43E-01	1.00E+00	LOC102399298	-8.04E-02	1.00E+00
ZNF236	-1.46E+00	5.96E-17	NOP9	4.83E-02	1.00E+00	VPS37C	5.85E-02	1.00E+00
KIAA1328	8.68E-01	6.04E-17	ADAP1	5.38E-02	1.00E+00	DLST	-4.54E-02	1.00E+00
CSK	-1.06E+00	6.05E-17	SUMF2	-2.82E-01	1.00E+00	METTL2A	-4.81E-02	1.00E+00
CXCR5	4.23E+00	6.05E-17	BNIPL	1.52E-01	1.00E+00	OPA1	4.63E-02	1.00E+00
HOMER2	-1.34E+00	6.06E-17	SOWAHA	2.55E-01	1.00E+00	ABHD14A	-6.50E-02	1.00E+00
NEDD9	-4.39E+00	6.16E-17	JAG1	-8.07E-02	1.00E+00	POLR2L	8.00E-02	1.00E+00
NDUFA12	-1.36E+00	6.27E-17	PDIA5	4.14E-02	1.00E+00	IPO4	-4.61E-02	1.00E+00
RBM44	5.52E+00	6.31E-17	MARCH9	4.93E-02	1.00E+00	FUNDC1	-5.51E-02	1.00E+00
LOC112582343	-4.04E+00	6.35E-17	SH2B2	-9.08E-02	1.00E+00	CCDC191	-5.32E-02	1.00E+00
ZNF26	-1.51E+00	6.39E-17	ASNS	-5.97E-02	1.00E+00	RARS2	-5.72E-02	1.00E+00
ARID5B	1.15E+00	6.41E-17	ANKS3	-8.31E-02	1.00E+00	RPL26L1	-5.84E-02	1.00E+00
PDP1	1.20E+00	6.44E-17	LTO1	4.89E-02	1.00E+00	SERP1	-4.23E-02	1.00E+00
ZNF597	4.95E+00	6.57E-17	USP10	4.40E-02	1.00E+00	DIRC2	-5.95E-02	1.00E+00
B3GALT1	2.29E+00	6.65E-17	ABCC11	-2.94E-01	1.00E+00	SLC44A2	-4.60E-02	1.00E+00
TRPC3	-3.97E+00	6.77E-17	GNPTAB	-1.27E-01	1.00E+00	SENP8	6.33E-02	1.00E+00
ZNF696	-1.13E+00	6.77E-17	AVEN	-7.16E-02	1.00E+00	OXCT1	4.73E-02	1.00E+00
AKIP1	1.75E+00	6.96E-17	LGR5	2.24E-01	1.00E+00	ARF6	5.82E-02	1.00E+00
APBB1	-8.39E-01	7.29E-17	SMLR1	3.07E-01	1.00E+00	LOC102399344	-6.75E-02	1.00E+00
TCEANC2	9.30E-01	7.31E-17	IL4I1	-1.75E-01	1.00E+00	FAF1	-4.49E-02	1.00E+00
LOC102389376	-9.77E-01	7.38E-17	HS3ST6	1.97E-01	1.00E+00	RPS6KA3	6.50E-02	1.00E+00
CHMP4B	-9.14E-01	7.49E-17	LOC102408782	2.43E-01	1.00E+00	GSDME	5.94E-02	1.00E+00
MRPS18C	1.05E+00	7.53E-17	KANK1	1.09E-01	1.00E+00	VPS37A	5.66E-02	1.00E+00
STK32A	4.88E+00	7.64E-17	TADA1	4.77E-02	1.00E+00	LARP6	5.87E-02	1.00E+00
RBFOX2	-9.59E-01	7.66E-17	TIMM50	-6.15E-02	1.00E+00	LOC102406480	5.61E-02	1.00E+00
EGR2	-4.90E+00	7.85E-17	TMEM39B	5.39E-02	1.00E+00	ARHGAP25	-1.97E-01	1.00E+00
KLF10	-5.17E+00	8.47E-17	CLK4	-7.67E-02	1.00E+00	LOC102395582	-5.66E-02	1.00E+00
HAAO	2.90E+00	8.49E-17	SPATA5L1	-8.53E-02	1.00E+00	AGAP3	-4.76E-02	1.00E+00
GDE1	-1.01E+00	8.58E-17	EVL	5.87E-02	1.00E+00	BRMS1L	-5.90E-02	1.00E+00
LOC112579967	4.33E+00	8.60E-17	ZNF473	9.89E-02	1.00E+00	STK31	6.56E-02	1.00E+00
MECR	-1.48E+00	8.72E-17	PTPRQ	4.81E-02	1.00E+00	AFF2	-4.89E-02	1.00E+00
STK32B	-3.83E+00	8.72E-17	LRMP	-7.69E-02	1.00E+00	CWF19L1	4.71E-02	1.00E+00

C2CD5	-1.07E+00	8.77E-17	LOC112584566	-1.11E-01	1.00E+00	ARVCF	6.22E-02	1.00E+00
TTC3	-9.05E-01	8.84E-17	LOC112584414	1.68E-01	1.00E+00	ANKRD45	-6.85E-02	1.00E+00
LOC112583734	1.57E+00	8.89E-17	LOC102388954	2.80E-01	1.00E+00	PAQR8	1.03E-01	1.00E+00
DENND4B	-1.15E+00	9.10E-17	EIF3F	-5.12E-02	1.00E+00	LOC102395812	6.15E-02	1.00E+00
ECT2L	2.41E+00	9.12E-17	LOC102410112	2.65E-01	1.00E+00	LOC102412021	-1.15E-01	1.00E+00
ANAPC15	1.41E+00	9.27E-17	BCL6	7.76E-02	1.00E+00	GPATCH1	5.08E-02	1.00E+00
DUSP12	8.61E-01	9.37E-17	SRSF12	6.94E-02	1.00E+00	XRCC6	-4.96E-02	1.00E+00
FUNDC2	-1.02E+00	9.46E-17	LOC112579234	-8.40E-02	1.00E+00	FAIM	5.32E-02	1.00E+00
CDC42BPG	1.32E+00	9.46E-17	TMED10	4.46E-02	1.00E+00	IRF5	-1.83E-01	1.00E+00
LOC102392881	6.67E+00	9.79E-17	B9D2	-1.31E-01	1.00E+00	BYSL	4.94E-02	1.00E+00
CENPL	1.15E+00	9.93E-17	SLC25A15	-4.98E-02	1.00E+00	TTC23	5.50E-02	1.00E+00
CTSK	-5.92E+00	1.02E-16	MAP3K13	7.90E-02	1.00E+00	TMEM50A	4.56E-02	1.00E+00
UBE2Z	7.06E-01	1.02E-16	LOC112587552	-1.73E-01	1.00E+00	SEC62	-4.46E-02	1.00E+00
MAMLD1	-4.28E+00	1.03E-16	AMT	8.01E-02	1.00E+00	LOC102390746	9.81E-02	1.00E+00
CDH16	-3.32E+00	1.03E-16	ACVR2B	-1.55E-01	1.00E+00	CLN3	-5.01E-02	1.00E+00
PLA1A	1.98E+00	1.04E-16	APC2	-1.27E-01	1.00E+00	ABCF3	4.41E-02	1.00E+00
PPP1R3B	-1.91E+00	1.05E-16	CAMTA1	5.21E-02	1.00E+00	DOK1	-6.58E-02	1.00E+00
TERF1	-1.28E+00	1.05E-16	ATP9A	5.46E-02	1.00E+00	SNF8	6.06E-02	1.00E+00
LOC102401726	6.28E+00	1.07E-16	NBEAL2	1.09E-01	1.00E+00	RAPH1	8.20E-02	1.00E+00
SCGN	4.64E+00	1.07E-16	PCBP1	4.79E-02	1.00E+00	VDAC3	5.10E-02	1.00E+00
LOC102397010	-1.44E+00	1.07E-16	ARAF	-6.29E-02	1.00E+00	CRAT	4.69E-02	1.00E+00
BAIAP2L2	-3.14E+00	1.10E-16	LOC112580789	-7.80E-02	1.00E+00	LOC102409250	4.32E-02	1.00E+00
LOC112581727	-5.00E+00	1.10E-16	ZNF70	2.11E-01	1.00E+00	RXRA	4.92E-02	1.00E+00
MFNG	-5.36E+00	1.13E-16	PDE8A	-4.41E-02	1.00E+00	DET1	-5.73E-02	1.00E+00
EPHB1	3.74E+00	1.14E-16	CRNKL1	-4.12E-02	1.00E+00	GIMAP8	1.16E-01	1.00E+00
NGB	7.30E+00	1.15E-16	CNPY1	1.22E-01	1.00E+00	NIF3L1	6.71E-02	1.00E+00
CCDC170	-3.55E+00	1.15E-16	RPP38	-1.08E-01	1.00E+00	RNF157	4.65E-02	1.00E+00
MYORG	-3.27E+00	1.16E-16	GPR27	-9.88E-02	1.00E+00	MYL6B	-6.83E-02	1.00E+00
C18H19orf54	1.14E+00	1.21E-16	SPSB3	6.59E-02	1.00E+00	HELZ	-5.56E-02	1.00E+00
EPB41L4A	-9.02E-01	1.22E-16	LOC102412417	-4.51E-02	1.00E+00	BSCL2	-4.36E-02	1.00E+00
SAMD5	-2.29E+00	1.23E-16	TMEM8B	1.53E-01	1.00E+00	MAML2	6.54E-02	1.00E+00
ETFA	-9.91E-01	1.25E-16	DIP2B	8.55E-02	1.00E+00	LRRC45	-6.68E-02	1.00E+00
MADD	8.66E-01	1.26E-16	MTRF1	-9.29E-02	1.00E+00	SIGLEC10	8.28E-02	1.00E+00
LOC102412332	-4.49E+00	1.29E-16	TRUB1	9.73E-02	1.00E+00	TRPM7	-5.79E-02	1.00E+00

PDSS2	8.61E-01	1.30E-16	LOC102390323	-2.76E-01	1.00E+00	SHISA5	-4.64E-02	1.00E+00
MAP4K3	-1.54E+00	1.32E-16	B3GNTL1	5.92E-02	1.00E+00	PLAUR	9.76E-02	1.00E+00
STOM	-9.15E-01	1.32E-16	MPC2	-1.02E-01	1.00E+00	AHCTF1	-5.16E-02	1.00E+00
QKI	-1.19E+00	1.33E-16	TTC7A	5.09E-02	1.00E+00	LOC102392703	-1.00E-01	1.00E+00
TJP1	1.08E+00	1.34E-16	SGCB	1.55E-01	1.00E+00	LOC112587016	-9.98E-02	1.00E+00
FNDC3B	-1.17E+00	1.35E-16	BHMG1	-2.42E-01	1.00E+00	LOC112587829	9.88E-02	1.00E+00
UCK2	-1.22E+00	1.44E-16	TNPO3	5.69E-02	1.00E+00	LOC102399277	-1.03E-01	1.00E+00
LOC112578445	5.14E+00	1.45E-16	HOXA3	8.27E-02	1.00E+00	AEBP1	-6.57E-02	1.00E+00
CKM	-2.60E+00	1.45E-16	SEC61G	-1.01E-01	1.00E+00	PPIL2	4.62E-02	1.00E+00
LRRC46	2.18E+00	1.46E-16	LOC112578208	-2.75E-01	1.00E+00	ANKRD44	9.28E-02	1.00E+00
LOC102413997	4.13E+00	1.46E-16	TSPYL2	6.02E-02	1.00E+00	LARP4	-6.00E-02	1.00E+00
LOC112582100	-3.52E+00	1.50E-16	SEC22A	5.37E-02	1.00E+00	N4BP1	-4.67E-02	1.00E+00
NAIF1	-1.44E+00	1.52E-16	MLLT10	8.89E-02	1.00E+00	SLC12A6	5.44E-02	1.00E+00
TEX264	9.66E-01	1.53E-16	LOC112578078	5.19E-01	1.00E+00	YPEL1	5.79E-02	1.00E+00
LYPD6B	4.86E+00	1.57E-16	REEP1	-1.30E-01	1.00E+00	RNF13	4.57E-02	1.00E+00
DIRC2	-1.36E+00	1.58E-16	SLBP	4.76E-02	1.00E+00	ATF7	5.38E-02	1.00E+00
LOC102391085	-1.08E+00	1.60E-16	EI24	5.08E-02	1.00E+00	CENPX	8.38E-02	1.00E+00
ZNF704	-2.59E+00	1.60E-16	XPNPEP1	-4.94E-02	1.00E+00	PNMA1	-6.24E-02	1.00E+00
FUZ	-1.53E+00	1.60E-16	NECAB3	-1.39E-01	1.00E+00	EHD3	1.06E-01	1.00E+00
FAM200A	-3.31E+00	1.61E-16	PIK3IP1	1.28E-01	1.00E+00	RNF10	-4.73E-02	1.00E+00
EML1	-1.24E+00	1.62E-16	LOC102403707	4.65E-01	1.00E+00	KYAT3	-5.71E-02	1.00E+00
ZFC3H1	-1.39E+00	1.64E-16	KIAA0825	-5.64E-02	1.00E+00	DCUN1D3	6.39E-02	1.00E+00
LOC102395100	1.29E+00	1.66E-16	FAM13A	5.28E-02	1.00E+00	ZNFX1	-6.25E-02	1.00E+00
VIPR1	3.44E+00	1.68E-16	ASGR1	-1.53E-01	1.00E+00	GNAI3	-4.35E-02	1.00E+00
COP1	-7.57E-01	1.69E-16	FLOT1	-4.65E-02	1.00E+00	PSMD10	5.03E-02	1.00E+00
CPPED1	-1.21E+00	1.70E-16	EPAS1	4.26E-02	1.00E+00	AGPS	5.05E-02	1.00E+00
LOC102405199	-4.96E+00	1.70E-16	SENP6	4.77E-02	1.00E+00	GOT2	-4.71E-02	1.00E+00
LOC112582939	5.77E+00	1.70E-16	SLC25A29	1.30E-01	1.00E+00	NR1D1	-6.00E-02	1.00E+00
LOC102416299	-2.98E+00	1.71E-16	ADAMTS4	-2.20E-01	1.00E+00	LOC102398374	-9.59E-02	1.00E+00
ANP32B	-1.21E+00	1.75E-16	CNTD2	8.00E-02	1.00E+00	CHD1	-6.33E-02	1.00E+00
LOC102402010	3.52E+00	1.76E-16	PODXL2	1.36E-01	1.00E+00	POMT1	-4.96E-02	1.00E+00
NUBP1	1.16E+00	1.76E-16	ZNF10	2.50E-01	1.00E+00	ZNF483	-1.04E-01	1.00E+00
LOC112580279	-	1.77E-16	CFAP44	5.53E-02	1.00E+00	CTSV	-4.98E-	1.00E+00

	5.51E+00						02	
AFF3	1.58E+00	1.80E-16	AKIRIN1	-6.59E-02	1.00E+00	LOC112582189	-5.89E-02	1.00E+00
STAT1	-9.23E-01	1.80E-16	C19H5orf47	-1.01E-01	1.00E+00	LOC112583463	-1.13E-01	1.00E+00
UBN2	1.25E+00	1.84E-16	DNAH2	-2.28E-01	1.00E+00	RTL9	-9.69E-02	1.00E+00
GON7	1.02E+00	1.87E-16	FBXO31	5.51E-02	1.00E+00	FDX2	6.53E-02	1.00E+00
C1QL4	5.46E+00	1.88E-16	ABI2	3.98E-02	1.00E+00	PIAS3	-5.63E-02	1.00E+00
ACBD5	-1.58E+00	1.90E-16	TBX15	5.17E-02	1.00E+00	DEGS2	1.10E-01	1.00E+00
AAK1	1.47E+00	1.91E-16	KDM1B	1.16E-01	1.00E+00	ZNF618	-5.05E-02	1.00E+00
LOC112583974	5.08E+00	1.94E-16	CEP68	4.23E-02	1.00E+00	MAP1S	5.22E-02	1.00E+00
ANKRD29	-2.66E+00	1.94E-16	FMO4	-2.58E-01	1.00E+00	RBM12B	-6.02E-02	1.00E+00
SH3BP2	-1.99E+00	1.97E-16	WDR54	-7.23E-02	1.00E+00	TCIM	9.29E-02	1.00E+00
COL11A2	-2.86E+00	1.99E-16	CACNG5	-1.24E-01	1.00E+00	CDC23	-5.32E-02	1.00E+00
TOX	-1.68E+00	2.00E-16	CXXC4	1.03E-01	1.00E+00	BRINP1	-1.48E-01	1.00E+00
PARVG	3.86E+00	2.03E-16	ZNF219	-1.24E-01	1.00E+00	MRPL24	6.98E-02	1.00E+00
MFSD12	-1.76E+00	2.03E-16	PIGU	-6.06E-02	1.00E+00	SNRPD1	6.97E-02	1.00E+00
FARS2	-1.02E+00	2.03E-16	KCNJ10	3.24E-01	1.00E+00	SNTN	-1.11E-01	1.00E+00
PEX11B	1.21E+00	2.04E-16	C17H5orf52	1.15E-01	1.00E+00	NR3C1	-9.92E-02	1.00E+00
TIMM8A	9.56E-01	2.04E-16	CCDC62	-1.38E-01	1.00E+00	UBQLN2	-6.05E-02	1.00E+00
RAB11B	-8.49E-01	2.05E-16	ANKRD31	1.57E-01	1.00E+00	LRPAP1	-7.47E-02	1.00E+00
EPDR1	-5.16E+00	2.05E-16	NHEJ1	1.09E-01	1.00E+00	C15H8orf37	-7.38E-02	1.00E+00
GGACT	-2.61E+00	2.05E-16	SOCS5	4.94E-02	1.00E+00	FBXO4	-6.44E-02	1.00E+00
LOC102394124	1.07E+00	2.10E-16	CFL1	-4.65E-02	1.00E+00	LOC102395337	9.88E-02	1.00E+00
C6H1orf216	-3.17E+00	2.11E-16	ADGRA3	-6.68E-02	1.00E+00	PIGY	7.09E-02	1.00E+00
NUDT7	-2.07E+00	2.11E-16	LOC102391313	6.58E-02	1.00E+00	ECSIT	6.93E-02	1.00E+00
SAMD4B	1.02E+00	2.17E-16	USP16	4.14E-02	1.00E+00	PPP3CB	-4.33E-02	1.00E+00
YLPM1	-9.03E-01	2.17E-16	ZNF184	-5.53E-02	1.00E+00	LOC102398348	-6.30E-02	1.00E+00
LOC112579192	2.93E+00	2.19E-16	TIGD2	-6.72E-02	1.00E+00	RUNDC1	-6.76E-02	1.00E+00
NAA50	1.25E+00	2.22E-16	LOC102416032	-3.77E-01	1.00E+00	SLC38A7	5.12E-02	1.00E+00
SPDYA	1.47E+00	2.23E-16	TRPC7	7.83E-02	1.00E+00	RNF219	-5.58E-02	1.00E+00
ZNF830	9.53E-01	2.23E-16	LOC102410132	1.13E-01	1.00E+00	SBNO1	5.98E-02	1.00E+00
LOC102395053	4.02E+00	2.23E-16	SIGMAR1	-6.03E-02	1.00E+00	NOP56	6.26E-02	1.00E+00
LOC102404231	3.24E+00	2.27E-16	LOC102398755	9.19E-02	1.00E+00	ING1	6.06E-02	1.00E+00
DCUN1D1	-8.67E-01	2.27E-16	AGTPBP1	-9.50E-02	1.00E+00	CHKB	-5.85E-02	1.00E+00
SUPT7L	9.29E-01	2.28E-16	SEPT11	-5.41E-02	1.00E+00	LOC112577706	-1.06E-01	1.00E+00

LOC112581204	-3.28E+00	2.30E-16	TMED2	-7.22E-02	1.00E+00	HECTD1	5.59E-02	1.00E+00
QPCT	1.98E+00	2.31E-16	RRAGC	-4.42E-02	1.00E+00	CHRD	6.93E-02	1.00E+00
MTERF4	9.81E-01	2.32E-16	ADAM15	-4.12E-01	1.00E+00	STXBP2	-5.07E-02	1.00E+00
GSTCD	-1.69E+00	2.33E-16	USHBP1	1.89E-01	1.00E+00	JADE2	-5.70E-02	1.00E+00
REXO2	1.09E+00	2.38E-16	PPP3CB	6.37E-02	1.00E+00	FERMT1	-6.86E-02	1.00E+00
AKAP9	-1.36E+00	2.45E-16	GFI1	-8.86E-02	1.00E+00	PEX14	-4.47E-02	1.00E+00
HDC	2.65E+00	2.50E-16	FASTKD1	1.05E-01	1.00E+00	LOC102392787	-2.31E-01	1.00E+00
ABHD4	7.14E-01	2.52E-16	PARN	4.44E-02	1.00E+00	TBC1D24	4.63E-02	1.00E+00
RASL12	3.40E+00	2.53E-16	POU1F1	2.06E-01	1.00E+00	BHLHE41	8.12E-02	1.00E+00
C24H16orf89	2.99E+00	2.53E-16	ACAA1	5.75E-02	1.00E+00	RAB9A	-5.26E-02	1.00E+00
LOC102398746	-4.39E+00	2.56E-16	LOC102402959	-1.22E-01	1.00E+00	LOC102390908	-8.18E-02	1.00E+00
LOC112579592	3.15E+00	2.60E-16	SF3A3	-4.18E-02	1.00E+00	NFIL3	-9.48E-02	1.00E+00
LAMTOR4	-1.32E+00	2.63E-16	CAB39L	1.07E-01	1.00E+00	HARS2	-4.59E-02	1.00E+00
CSNK1D	7.22E-01	2.69E-16	FAM76B	1.28E-01	1.00E+00	SUPT20H	-4.62E-02	1.00E+00
LOC102398905	8.55E-01	2.75E-16	PSMG2	1.18E-01	1.00E+00	SNTB2	-4.69E-02	1.00E+00
SLC7A5	-1.28E+00	2.77E-16	PSMA2	-1.34E-01	1.00E+00	LOC102408597	-6.80E-02	1.00E+00
BEAN1	2.26E+00	2.79E-16	DDIT3	7.47E-02	1.00E+00	TBCA	-6.65E-02	1.00E+00
COL16A1	-1.52E+00	2.80E-16	KAT5	-5.51E-02	1.00E+00	ZNF212	5.36E-02	1.00E+00
ATP1B2	-4.34E+00	2.81E-16	LOC102415700	7.11E-02	1.00E+00	CBR3	1.22E-01	1.00E+00
NRTN	-2.12E+00	2.86E-16	LOC112581229	-5.94E-02	1.00E+00	MAN2A2	5.82E-02	1.00E+00
CASP3	-9.73E-01	2.88E-16	DDX41	-5.35E-02	1.00E+00	SPART	-4.85E-02	1.00E+00
APLP2	-1.12E+00	2.88E-16	FAM171B	-9.33E-02	1.00E+00	BBS5	-6.78E-02	1.00E+00
LOC112583825	2.45E+00	2.89E-16	COMMD6	9.97E-02	1.00E+00	GRAMD1C	-6.08E-02	1.00E+00
PRRC1	1.36E+00	2.89E-16	KIF20B	6.61E-02	1.00E+00	CEP44	-5.42E-02	1.00E+00
RFXANK	-1.07E+00	2.90E-16	AGAP2	-8.90E-02	1.00E+00	NHP2	6.70E-02	1.00E+00
CCDC152	4.09E+00	2.94E-16	SYNJ2	7.11E-02	1.00E+00	CHML	1.55E-01	1.00E+00
C9H19orf70	-1.43E+00	2.99E-16	SNRNP40	1.26E-01	1.00E+00	NEGR1	7.18E-02	1.00E+00
LOC112578689	-3.98E+00	3.10E-16	LOC102389850	-7.12E-02	1.00E+00	KIFC1	-6.14E-02	1.00E+00
MAP2K6	-1.97E+00	3.27E-16	LOC102411916	-2.00E-01	1.00E+00	LYPLA1	4.34E-02	1.00E+00
SCAI	-1.33E+00	3.32E-16	LOC112578530	-1.05E-01	1.00E+00	PDK3	-5.61E-02	1.00E+00
PPME1	9.69E-01	3.33E-16	SLC6A14	2.87E-01	1.00E+00	TMEM265	7.66E-02	1.00E+00
GABRA4	4.62E+00	3.39E-16	IQCH	1.10E-01	1.00E+00	AP3B1	4.38E-02	1.00E+00
DUSP22	-1.56E+00	3.46E-16	POLR2F	-6.95E-02	1.00E+00	S1PR1	9.69E-02	1.00E+00
FAM184A	8.49E-01	3.51E-16	MCCC1	-5.22E-02	1.00E+00	MTFR2	6.01E-02	1.00E+00

RIT1	8.33E-01	3.51E-16	SFN	-9.12E-02	1.00E+00	LOC112585571	1.11E-01	1.00E+00
INSM1	6.00E+00	3.61E-16	ABCD3	6.02E-02	1.00E+00	ZRANB2	-5.11E-02	1.00E+00
SENP1	-1.07E+00	3.63E-16	TIMELESS	6.09E-02	1.00E+00	CGRRF1	6.14E-02	1.00E+00
DCUN1D5	8.25E-01	3.69E-16	LOC112579974	2.88E-01	1.00E+00	TIMP2	-6.40E-02	1.00E+00
FARP2	1.07E+00	3.72E-16	SEC61B	1.40E-01	1.00E+00	TOR1A	5.13E-02	1.00E+00
KIF1A	-3.13E+00	3.86E-16	VRK3	8.01E-02	1.00E+00	LOC102390842	4.61E-02	1.00E+00
DGKQ	-1.32E+00	3.91E-16	TNFRSF11A	-1.30E-01	1.00E+00	PCBP1	-5.15E-02	1.00E+00
MRPL53	-8.33E-01	3.92E-16	PLXNB1	1.14E-01	1.00E+00	NR1H2	4.68E-02	1.00E+00
LOC112581125	4.38E+00	3.95E-16	RIPK2	-8.94E-02	1.00E+00	OGFOD1	4.76E-02	1.00E+00
ZBTB8OS	-9.88E-01	4.00E-16	DDX47	-4.02E-02	1.00E+00	NECTIN1	-4.45E-02	1.00E+00
TGOLN2	8.04E-01	4.06E-16	LRRC36	-2.29E-01	1.00E+00	NAPEPLD	-4.45E-02	1.00E+00
DTX3L	-5.20E+00	4.09E-16	DYNLL2	-5.12E-02	1.00E+00	ZNF629	4.65E-02	1.00E+00
SDCCAG8	-1.14E+00	4.10E-16	CDX1	-8.47E-02	1.00E+00	LOC102400625	-7.99E-02	1.00E+00
LOC102407908	-3.80E+00	4.12E-16	CCDC30	-1.03E-01	1.00E+00	MBD5	5.73E-02	1.00E+00
PHF14	-7.37E-01	4.15E-16	TANC2	7.93E-02	1.00E+00	TFF2	6.61E-02	1.00E+00
ARF3	8.76E-01	4.19E-16	CLNS1A	-1.13E-01	1.00E+00	MPPE1	-5.18E-02	1.00E+00
RTF1	-6.88E-01	4.19E-16	KCTD2	3.84E-02	1.00E+00	LOC102394142	-1.10E-01	1.00E+00
BIRC6	-1.42E+00	4.25E-16	LOC102402675	-2.72E-01	1.00E+00	CRYZ	4.43E-02	1.00E+00
ATF7IP	-1.16E+00	4.26E-16	OCEL1	5.58E-02	1.00E+00	SLC25A4	-5.60E-02	1.00E+00
ZNF706	9.59E-01	4.28E-16	LOC112587746	-1.04E-01	1.00E+00	GAPVD1	-4.58E-02	1.00E+00
SUCNR1	-5.25E+00	4.38E-16	FIGLA	-5.64E-02	1.00E+00	PKNOX2	1.18E-01	1.00E+00
LOC102388984	-4.06E+00	4.47E-16	CHD7	9.96E-02	1.00E+00	ANKRD53	7.78E-02	1.00E+00
NUP62	9.35E-01	4.49E-16	AP4B1	-7.46E-02	1.00E+00	POLR3G	-8.13E-02	1.00E+00
CALM3	-1.43E+00	4.51E-16	ACAP2	-8.26E-02	1.00E+00	ITSN2	6.16E-02	1.00E+00
EDN2	-5.54E+00	4.52E-16	LOC102396318	9.87E-02	1.00E+00	MRI1	-5.56E-02	1.00E+00
GANC	2.18E+00	4.55E-16	RFX3	1.12E-01	1.00E+00	LOC102414919	5.69E-02	1.00E+00
KMT2C	1.35E+00	4.57E-16	LOC102411649	-8.02E-02	1.00E+00	LOC102391952	-9.08E-02	1.00E+00
HSPBP1	1.13E+00	4.64E-16	CCDC65	-4.64E-02	1.00E+00	LOC102398226	-5.68E-02	1.00E+00
CLDN23	4.61E+00	4.66E-16	TSNAX	5.91E-02	1.00E+00	STARD3	-4.41E-02	1.00E+00
PTBP1	-9.74E-01	4.70E-16	FZR1	5.47E-02	1.00E+00	LOC112582356	7.85E-02	1.00E+00
LOC102403501	1.24E+00	4.74E-16	PCDH15	1.32E-01	1.00E+00	SLC15A3	-2.28E-01	1.00E+00
FANCB	1.63E+00	4.76E-16	PIK3R1	7.62E-02	1.00E+00	LAPTM4B	-8.09E-02	1.00E+00
LOC112578528	5.44E+00	4.84E-16	LOC112578440	-1.25E-01	1.00E+00	ZFR	-5.09E-02	1.00E+00

TIMM10	-1.53E+00	4.84E-16	ANKRD40	5.91E-02	1.00E+00	SYNCRIP	-4.44E-02	1.00E+00
COPS6	-1.24E+00	4.91E-16	UTP4	-4.55E-02	1.00E+00	LRIG2	5.91E-02	1.00E+00
ZNRD1	-1.21E+00	4.97E-16	PLAT	-1.22E-01	1.00E+00	ATG7	-5.02E-02	1.00E+00
ADGRD1	3.51E+00	5.13E-16	LOC102404378	1.88E-01	1.00E+00	CCDC61	5.64E-02	1.00E+00
LOC102397719	4.80E+00	5.18E-16	PPIP5K1	7.86E-02	1.00E+00	PLAU	-1.12E-01	1.00E+00
UBE3D	9.95E-01	5.18E-16	SLAIN1	-5.09E-02	1.00E+00	RELL1	7.62E-02	1.00E+00
CHRDL2	3.82E+00	5.22E-16	LOC112583531	-2.47E-01	1.00E+00	NOL11	4.36E-02	1.00E+00
TMEM262	3.35E+00	5.24E-16	RNF8	4.61E-02	1.00E+00	ESYT2	-4.41E-02	1.00E+00
EPHA5	-4.59E+00	5.26E-16	EIF3J	6.64E-02	1.00E+00	NDUFAF1	4.90E-02	1.00E+00
RBMX2	-9.35E-01	5.28E-16	NAA10	-6.47E-02	1.00E+00	NRG2	1.01E-01	1.00E+00
SOX5	-1.35E+00	5.33E-16	DDC	-9.05E-02	1.00E+00	LAMB4	8.76E-02	1.00E+00
SIRT5	9.60E-01	5.37E-16	LOC102415270	7.20E-02	1.00E+00	PA2G4	6.30E-02	1.00E+00
LOC102395477	1.62E+00	5.39E-16	HMMR	6.87E-02	1.00E+00	HNRNPA0	-4.48E-02	1.00E+00
LOC102410360	-4.96E+00	5.41E-16	WDR6	-1.26E-01	1.00E+00	HTRA4	-5.43E-02	1.00E+00
LOC112585226	2.59E+00	5.49E-16	NALCN	-2.78E-01	1.00E+00	LOC112578269	-7.59E-02	1.00E+00
KLF16	9.80E-01	5.58E-16	DEF6	-7.90E-02	1.00E+00	RPL36A	7.21E-02	1.00E+00
MAPK12	2.65E+00	5.67E-16	UROS	7.05E-02	1.00E+00	TMEM170A	4.69E-02	1.00E+00
GGPS1	8.34E-01	5.80E-16	ECI1	-8.83E-02	1.00E+00	YPEL2	-6.30E-02	1.00E+00
LOC112587476	4.86E+00	6.05E-16	PAXIP1	7.57E-02	1.00E+00	METTL4	-1.04E-01	1.00E+00
STK17B	-3.14E+00	6.05E-16	TMED5	-1.26E-01	1.00E+00	PALB2	-4.95E-02	1.00E+00
LOC112583751	4.04E+00	6.23E-16	APOA2	-7.80E-02	1.00E+00	TMEM120B	-6.35E-02	1.00E+00
LOC112586640	-4.43E+00	6.28E-16	TTC26	9.47E-02	1.00E+00	LOC112578798	-5.88E-02	1.00E+00
ALDH9A1	-8.42E-01	6.42E-16	SAXO2	2.08E-01	1.00E+00	VPS13A	5.88E-02	1.00E+00
TK1	1.24E+00	6.48E-16	HNRNPH2	-6.69E-02	1.00E+00	CRK	4.94E-02	1.00E+00
LONRF3	-1.44E+00	6.56E-16	LSM1	-6.71E-02	1.00E+00	HK3	-1.11E-01	1.00E+00
MAP3K11	-1.03E+00	6.68E-16	LAMB3	-1.40E-01	1.00E+00	SRSF1	-4.73E-02	1.00E+00
ARHGAP26	1.27E+00	6.72E-16	LOC102410549	-1.18E-01	1.00E+00	ATF1	-4.60E-02	1.00E+00
ABCA5	9.73E-01	6.74E-16	ABCC5	9.19E-02	1.00E+00	UBL5	6.56E-02	1.00E+00
FOLH1B	-3.28E+00	6.84E-16	TRAPPC2L	1.98E-01	1.00E+00	METTL6	4.58E-02	1.00E+00
NPM1	-1.11E+00	6.86E-16	HNRNPA3	4.48E-02	1.00E+00	CLIP1	5.77E-02	1.00E+00
CLU	-1.52E+00	7.00E-16	SLC27A4	1.00E-01	1.00E+00	SNX25	-4.86E-02	1.00E+00
MRPS12	-1.47E+00	7.04E-16	LOC112587898	1.62E-01	1.00E+00	MAP3K15	4.85E-02	1.00E+00
TMEM216	-1.63E+00	7.07E-16	HSF2BP	-5.35E-02	1.00E+00	NRM	-6.98E-02	1.00E+00
SLC22A23	-	7.09E-16	MTMR7	-4.52E-	1.00E+00	SLC7A2	5.70E-02	1.00E+00

	1.69E+00			02				
PIM3	1.04E+00	7.09E-16	KLHL11	8.82E-02	1.00E+00	TBCE	-4.33E-02	1.00E+00
SGK1	-6.41E+00	7.10E-16	FUCA2	-2.35E-01	1.00E+00	RNFT2	-6.53E-02	1.00E+00
RHOG	-1.13E+00	7.17E-16	OTUD7A	-7.47E-02	1.00E+00	VPS72	4.24E-02	1.00E+00
ATF7IP2	9.46E-01	7.32E-16	ANPEP	-2.25E-01	1.00E+00	DIXDC1	6.31E-02	1.00E+00
EMILIN3	-3.84E+00	7.33E-16	LTN1	8.31E-02	1.00E+00	PPP2R5D	-4.55E-02	1.00E+00
MYL7	5.28E+00	7.33E-16	PPP1R12B	5.71E-02	1.00E+00	WDR78	-6.78E-02	1.00E+00
INTS11	-1.04E+00	7.34E-16	RAB36	4.03E-01	1.00E+00	MECR	-6.52E-02	1.00E+00
LOC112578484	2.82E+00	7.37E-16	PIGX	-5.58E-02	1.00E+00	RNF185	4.17E-02	1.00E+00
YTHDF3	1.02E+00	7.40E-16	LOC102388956	-1.12E-01	1.00E+00	C6H1orf123	6.46E-02	1.00E+00
NLRC4	2.98E+00	7.42E-16	ILKAP	-6.26E-02	1.00E+00	KRBA2	-6.15E-02	1.00E+00
LRRC28	-9.46E-01	7.46E-16	SIX3	-1.51E-01	1.00E+00	RAD51C	-5.40E-02	1.00E+00
DPY19L1	-9.68E-01	7.57E-16	ACTR8	-5.61E-02	1.00E+00	PFDN5	8.20E-02	1.00E+00
FGF18	2.24E+00	7.58E-16	CAPS2	1.74E-01	1.00E+00	BLOC1S1	7.03E-02	1.00E+00
LOC112582163	5.54E+00	7.65E-16	ZNF8	1.33E-01	1.00E+00	LOC102411280	7.84E-02	1.00E+00
IRF4	2.22E+00	7.74E-16	RNF217	-1.36E-01	1.00E+00	P3H3	-5.55E-02	1.00E+00
ERICH6	3.05E+00	7.84E-16	NKIRAS2	6.96E-02	1.00E+00	LOC102407872	-5.14E-02	1.00E+00
LOC112586514	4.70E+00	7.85E-16	DNAJC18	-5.31E-02	1.00E+00	RAD52	-4.17E-02	1.00E+00
RHBDL1	-2.54E+00	7.94E-16	KATNA1	7.66E-02	1.00E+00	TRPM3	9.81E-02	1.00E+00
MYO1A	3.64E+00	7.95E-16	TM7SF3	-6.00E-02	1.00E+00	ODF3L2	-1.30E-01	1.00E+00
LOC102400532	9.64E-01	8.03E-16	STK33	7.52E-02	1.00E+00	ADI1	-5.71E-02	1.00E+00
LOC112585747	-2.60E+00	8.19E-16	ZMYND11	-5.42E-02	1.00E+00	TRPM4	8.72E-02	1.00E+00
JAKMIP1	-2.81E+00	8.26E-16	LOC112584643	-1.41E-01	1.00E+00	E2F2	-7.61E-02	1.00E+00
CACNB1	-1.84E+00	8.34E-16	LOC112582320	-1.80E-01	1.00E+00	LOC112578002	-1.22E-01	1.00E+00
UBE3A	-8.65E-01	8.48E-16	LOC102408184	-5.64E-02	1.00E+00	PAN3	-5.13E-02	1.00E+00
UGGT2	-1.40E+00	8.65E-16	PMEPA1	-8.76E-02	1.00E+00	IREB2	-5.08E-02	1.00E+00
AIMP2	1.03E+00	8.84E-16	FOXO6	1.00E-01	1.00E+00	CNNM3	-4.27E-02	1.00E+00
GPX6	-3.70E+00	8.91E-16	INHBA	-2.44E-01	1.00E+00	GMIP	9.29E-02	1.00E+00
LOC102414209	-1.51E+00	8.94E-16	PDE3B	6.06E-02	1.00E+00	CEP83	-4.42E-02	1.00E+00
HDAC1	-7.57E-01	8.98E-16	GPR179	7.88E-02	1.00E+00	YES1	5.83E-02	1.00E+00
LOC102408349	-4.20E+00	9.00E-16	LOC102397734	1.72E-01	1.00E+00	CYTH1	-4.77E-02	1.00E+00
PDHX	7.16E-01	9.24E-16	RAB33B	-2.31E-01	1.00E+00	MORC2	-4.35E-02	1.00E+00
ARHGEF9	1.01E+00	9.27E-16	LDLRAP1	5.24E-02	1.00E+00	KTN1	4.53E-02	1.00E+00
MAFK	-	9.39E-16	EPRS	-4.93E-	1.00E+00	XKRX	7.56E-02	1.00E+00

	1.23E+00			02				
CIT	1.19E+00	9.46E-16	CES4A	-2.77E-01	1.00E+00	DACT1	-8.75E-02	1.00E+00
XAB2	9.65E-01	9.50E-16	CNTLN	-9.64E-02	1.00E+00	TOP2B	4.52E-02	1.00E+00
LOC112583591	4.54E+00	9.53E-16	PTPRN2	-7.69E-02	1.00E+00	MAPK3	4.71E-02	1.00E+00
TIGD3	-3.09E+00	9.61E-16	RTN4	5.06E-02	1.00E+00	LOC102397361	-4.98E-02	1.00E+00
GEMIN7	-1.88E+00	9.63E-16	ZNF407	-6.48E-02	1.00E+00	CNTFR	-8.77E-02	1.00E+00
QRICH1	8.43E-01	9.72E-16	ZNF813	-7.36E-02	1.00E+00	C1QB	-1.96E-01	1.00E+00
ZHX1	-2.17E+00	9.78E-16	LAMA2	5.96E-02	1.00E+00	CDK9	4.77E-02	1.00E+00
LOC102405376	4.85E+00	9.88E-16	BLOC1S5	-4.84E-02	1.00E+00	CCDC25	4.09E-02	1.00E+00
CPTP	9.03E-01	9.97E-16	NCAPD3	5.36E-02	1.00E+00	LOC112581441	-9.03E-02	1.00E+00
EAF1	9.52E-01	1.02E-15	LOC112583654	-2.15E-01	1.00E+00	MYPOP	8.10E-02	1.00E+00
LOC102407809	-6.02E+00	1.04E-15	IL1R1	-5.88E-02	1.00E+00	PRKD3	7.12E-02	1.00E+00
WASHC5	8.76E-01	1.04E-15	GIPC1	-1.18E-01	1.00E+00	PCIF1	-4.24E-02	1.00E+00
CENPI	1.34E+00	1.04E-15	P3H1	7.06E-02	1.00E+00	KLHL3	1.25E-01	1.00E+00
ASB16	2.22E+00	1.04E-15	SIRT2	8.42E-02	1.00E+00	C17H12orf49	-5.16E-02	1.00E+00
TGM1	1.83E+00	1.05E-15	THBS4	-4.83E-02	1.00E+00	TAF5L	-4.62E-02	1.00E+00
FNDC3A	9.66E-01	1.08E-15	CCR8	-7.33E-02	1.00E+00	C4H1orf131	-5.44E-02	1.00E+00
DUSP2	-4.84E+00	1.09E-15	GSTM3	-5.76E-02	1.00E+00	EIF4G3	4.69E-02	1.00E+00
PFDN5	-1.64E+00	1.12E-15	RNF180	-5.10E-02	1.00E+00	CTSF	-5.88E-02	1.00E+00
BAMBI	-2.06E+00	1.12E-15	VRK2	-5.59E-02	1.00E+00	UBR3	-5.66E-02	1.00E+00
MTLN	-1.76E+00	1.12E-15	MAMDC4	3.06E-01	1.00E+00	EFNA2	-8.69E-02	1.00E+00
GALNT15	-3.57E+00	1.12E-15	FLAD1	-5.60E-02	1.00E+00	PRELID1	5.28E-02	1.00E+00
NHSL1	1.18E+00	1.13E-15	TUBGCP5	6.26E-02	1.00E+00	CLEC1B	-7.70E-02	1.00E+00
ECT2	1.07E+00	1.14E-15	LOC102403646	-5.63E-02	1.00E+00	CIB2	-5.97E-02	1.00E+00
OSTM1	-1.34E+00	1.14E-15	LOC102395312	-6.78E-02	1.00E+00	VAMP8	6.93E-02	1.00E+00
LOC102411461	-7.35E-01	1.15E-15	EMC8	-5.78E-02	1.00E+00	CCDC22	5.07E-02	1.00E+00
RAB19	-3.26E+00	1.16E-15	RPL11	-6.50E-02	1.00E+00	C9H1orf35	-5.60E-02	1.00E+00
CCDC9	-9.13E-01	1.18E-15	RREB1	-5.58E-02	1.00E+00	VCP	-5.36E-02	1.00E+00
SPIN4	-5.09E+00	1.21E-15	LOC102401813	-5.35E-02	1.00E+00	VPS13B	-5.06E-02	1.00E+00
LOC102398735	-3.59E+00	1.21E-15	ADRM1	4.23E-02	1.00E+00	ZNF276	-4.67E-02	1.00E+00
DIAPH3	7.73E-01	1.21E-15	LOC102393747	-2.34E-01	1.00E+00	NEDD8	5.67E-02	1.00E+00
BRF2	9.83E-01	1.22E-15	ABCF1	-5.20E-02	1.00E+00	FEN1	5.59E-02	1.00E+00
GLDN	-5.50E+00	1.22E-15	ZDHHC12	7.35E-02	1.00E+00	RASSF9	1.25E-01	1.00E+00

CCDC28A	-1.37E+00	1.22E-15	TGFBR3	5.76E-02	1.00E+00	GLS2	9.37E-02	1.00E+00
WDR25	1.03E+00	1.23E-15	DPYSL5	-1.53E-01	1.00E+00	SCFD1	-4.23E-02	1.00E+00
CLTRN	-1.61E+00	1.24E-15	LOC112586282	-2.37E-01	1.00E+00	TUSC3	-3.93E-02	1.00E+00
FAM208A	-1.02E+00	1.26E-15	LRP2BP	1.41E-01	1.00E+00	STARD3NL	-4.24E-02	1.00E+00
DDX60	1.78E+00	1.27E-15	IQCB1	4.20E-02	1.00E+00	DDX54	4.42E-02	1.00E+00
TTLL3	-1.09E+00	1.27E-15	CDH3	5.05E-02	1.00E+00	GNG12	5.80E-02	1.00E+00
NDUFB10	-1.45E+00	1.28E-15	LOC112582336	-6.12E-02	1.00E+00	ZBTB47	4.33E-02	1.00E+00
ARVCF	-2.59E+00	1.30E-15	CDC20	3.87E-02	1.00E+00	BCCIP	5.06E-02	1.00E+00
S100A4	-6.53E+00	1.33E-15	MAT1A	-2.11E-01	1.00E+00	APPL1	-1.04E-01	1.00E+00
LRRC52	4.61E+00	1.35E-15	OGFOD3	-6.40E-02	1.00E+00	EXOSC4	-1.05E-01	1.00E+00
LOC102413806	-1.36E+00	1.35E-15	RNF26	5.47E-02	1.00E+00	LOC112578438	-1.04E-01	1.00E+00
PSMD6	-7.55E-01	1.37E-15	MCMBP	4.86E-02	1.00E+00	PRPF8	-4.59E-02	1.00E+00
FAM91A1	-1.27E+00	1.37E-15	ZNF35	-9.85E-02	1.00E+00	TNK2	4.24E-02	1.00E+00
SRCAP	1.40E+00	1.38E-15	MTOR	-5.03E-02	1.00E+00	CENPC	-5.02E-02	1.00E+00
KIAA2012	2.90E+00	1.38E-15	SFXN1	-8.53E-02	1.00E+00	SLC38A8	1.14E-01	1.00E+00
GPIHBP1	-4.87E+00	1.40E-15	LOC102416201	-6.60E-02	1.00E+00	LOC102396428	9.34E-02	1.00E+00
FAM160A2	6.99E-01	1.40E-15	HOXB7	-1.04E-01	1.00E+00	UGCG	-5.63E-02	1.00E+00
LOC102399099	-4.12E+00	1.41E-15	SCNN1A	-7.74E-02	1.00E+00	INTS7	-4.64E-02	1.00E+00
ALDH1A1	-4.80E+00	1.44E-15	PRKD2	-1.15E-01	1.00E+00	STAM2	-5.66E-02	1.00E+00
GFPT2	-2.16E+00	1.44E-15	EZH2	-4.21E-02	1.00E+00	PKN3	-5.70E-02	1.00E+00
HDHD3	-1.89E+00	1.44E-15	CCNK	4.54E-02	1.00E+00	KDM2A	-4.67E-02	1.00E+00
WDFY2	-1.31E+00	1.45E-15	LOC102391650	-2.59E-01	1.00E+00	CLPTM1	-4.62E-02	1.00E+00
LOC112582355	-1.75E+00	1.47E-15	FRAS1	7.01E-02	1.00E+00	ZZZ3	-5.35E-02	1.00E+00
CUL2	-8.09E-01	1.49E-15	LOC102410316	-2.80E-01	1.00E+00	FGFRL1	-4.79E-02	1.00E+00
KDM7A	-2.33E+00	1.50E-15	LOC102412844	7.19E-02	1.00E+00	ZNF451	-4.48E-02	1.00E+00
IFNGR1	9.05E-01	1.52E-15	CALCOCO1	5.34E-02	1.00E+00	ZNHIT2	-7.33E-02	1.00E+00
PLAU	-5.08E+00	1.55E-15	RNF34	4.10E-02	1.00E+00	SAFB	-4.58E-02	1.00E+00
LOC102397479	-7.11E+00	1.55E-15	HACD1	1.11E-01	1.00E+00	LOC102416331	-4.65E-02	1.00E+00
LOC102395499	-1.26E+00	1.57E-15	STIL	1.03E-01	1.00E+00	BPTF	-6.17E-02	1.00E+00
EHBP1	-1.25E+00	1.58E-15	NKX3-1	-4.78E-02	1.00E+00	LOC112578267	-9.35E-02	1.00E+00
LMX1A	3.43E+00	1.63E-15	LBH	-1.09E-01	1.00E+00	CD276	-4.43E-02	1.00E+00
SEMA3E	4.19E+00	1.63E-15	NES	1.05E-01	1.00E+00	GLRX3	-4.93E-02	1.00E+00
MPPED2	-	1.64E-15	CDC25C	-4.15E-	1.00E+00	HTD2	4.64E-02	1.00E+00

	3.09E+00			02				
LOC102404498	1.64E+00	1.65E-15	LIPH	1.02E-01	1.00E+00	CORIN	1.00E-01	1.00E+00
LOC112578060	4.09E+00	1.68E-15	FTSJ3	4.62E-02	1.00E+00	LOC102416262	6.18E-02	1.00E+00
TRDMT1	-1.01E+00	1.69E-15	VPS8	4.46E-02	1.00E+00	SHISA2	-7.09E-02	1.00E+00
LOC112583779	2.42E+00	1.70E-15	PLCL1	1.33E-01	1.00E+00	B4GALT7	5.22E-02	1.00E+00
LOC112585317	-5.37E+00	1.73E-15	HAS2	-2.09E-01	1.00E+00	ENTPD6	-5.67E-02	1.00E+00
TMEM199	-2.88E+00	1.73E-15	SH3BP1	-5.94E-02	1.00E+00	LLGL2	-6.98E-02	1.00E+00
LOC112581803	1.96E+00	1.73E-15	C2H2orf69	-1.57E-01	1.00E+00	RASL11B	-7.57E-02	1.00E+00
LOC102390023	5.39E+00	1.80E-15	LRRC52	1.96E-01	1.00E+00	KCMF1	-4.71E-02	1.00E+00
YTHDF1	7.29E-01	1.82E-15	LOC112578141	3.15E-01	1.00E+00	PDCD5	6.39E-02	1.00E+00
PAIP2	-2.70E+00	1.85E-15	DCHS2	-4.90E-02	1.00E+00	YWHAQ	4.56E-02	1.00E+00
NTF4	3.80E+00	1.85E-15	SETX	-4.77E-02	1.00E+00	PRKRIP1	5.15E-02	1.00E+00
CHAF1B	9.51E-01	1.85E-15	TRNAU1AP	-6.32E-02	1.00E+00	EEFSEC	6.04E-02	1.00E+00
LOC112578811	9.39E-01	1.86E-15	GP5	-2.05E-01	1.00E+00	CSRP2	-5.26E-02	1.00E+00
KRR1	-1.05E+00	1.90E-15	NIPBL	-8.93E-02	1.00E+00	ECI1	-6.27E-02	1.00E+00
KBTBD11	1.51E+00	1.90E-15	CLPB	7.48E-02	1.00E+00	PACS1	4.44E-02	1.00E+00
PTS	-1.25E+00	1.90E-15	STK16	-5.37E-02	1.00E+00	PHLDB3	4.61E-02	1.00E+00
C1H4orf47	-1.30E+00	1.91E-15	DYDC1	-8.26E-02	1.00E+00	TTC27	-4.00E-02	1.00E+00
MPP1	-1.02E+00	1.96E-15	TMPRSS9	8.82E-02	1.00E+00	ATAD2B	-6.57E-02	1.00E+00
LOC102395334	-1.55E+00	1.98E-15	EMC2	-7.21E-02	1.00E+00	LRRC3	9.39E-02	1.00E+00
LOC102396410	-3.41E+00	1.98E-15	CDH12	-9.41E-02	1.00E+00	TXNDC5	-5.16E-02	1.00E+00
CMTM8	1.36E+00	1.98E-15	ZDHHC3	5.44E-02	1.00E+00	SIX4	6.99E-02	1.00E+00
LOXL2	-3.21E+00	2.00E-15	AFDN	5.98E-02	1.00E+00	SLC39A9	4.65E-02	1.00E+00
KCTD16	4.52E+00	2.01E-15	TEKT1	-7.37E-02	1.00E+00	ZNF79	-4.36E-02	1.00E+00
IL17RD	-1.29E+00	2.02E-15	CFTR	7.20E-02	1.00E+00	BRF2	5.89E-02	1.00E+00
LATS1	-2.44E+00	2.02E-15	DPYSL4	-1.78E-01	1.00E+00	CCDC114	-7.64E-02	1.00E+00
KCNA4	-3.05E+00	2.02E-15	ESCO2	7.10E-02	1.00E+00	SCRN3	-6.91E-02	1.00E+00
SLC43A1	-2.54E+00	2.05E-15	SETD3	4.90E-02	1.00E+00	ZC3H12A	1.14E-01	1.00E+00
VPS50	9.24E-01	2.07E-15	WHAMM	5.11E-02	1.00E+00	PYM1	6.11E-02	1.00E+00
TLK2	-7.97E-01	2.07E-15	PMS2	8.60E-02	1.00E+00	RAB4A	-6.31E-02	1.00E+00
TMEM106C	-1.24E+00	2.10E-15	MPP6	5.83E-02	1.00E+00	GRAMD2B	4.62E-02	1.00E+00
ZNF260	-1.44E+00	2.12E-15	ARHGEF4	2.65E-01	1.00E+00	UBE2V1	4.27E-02	1.00E+00
LOC112577686	4.64E+00	2.14E-15	RUNX3	-1.21E-01	1.00E+00	RNPS1	-4.58E-02	1.00E+00
AKAP7	-1.51E+00	2.17E-15	LOC112578487	1.96E-01	1.00E+00	ACAA2	-4.77E-02	1.00E+00
ARHGEF38	2.17E+00	2.20E-15	LOC102389989	1.76E-01	1.00E+00	CA11	-7.92E-02	1.00E+00

BCKDHA	-1.12E+00	2.21E-15	DNAJC7	-4.12E-02	1.00E+00	MNAT1	4.52E-02	1.00E+00
BAG6	-8.17E-01	2.29E-15	DDX27	5.08E-02	1.00E+00	RASGRP3	-4.99E-02	1.00E+00
RNASEH2C	-1.58E+00	2.32E-15	LOC102398213	-9.29E-02	1.00E+00	PLXNA3	4.33E-02	1.00E+00
HTR3A	-6.25E+00	2.36E-15	DPF3	-1.47E-01	1.00E+00	MFSD1	5.13E-02	1.00E+00
THSD7B	3.45E+00	2.38E-15	RFC2	-8.90E-02	1.00E+00	STUM	6.44E-02	1.00E+00
SBNO2	-1.18E+00	2.38E-15	RASAL2	6.29E-02	1.00E+00	RPAIN	6.43E-02	1.00E+00
LOC112585597	3.16E+00	2.43E-15	FBXL5	4.18E-02	1.00E+00	CMSS1	-5.36E-02	1.00E+00
ZFP57	-2.70E+00	2.47E-15	PEX14	-4.01E-02	1.00E+00	TIGD3	9.41E-02	1.00E+00
SNX27	-9.85E-01	2.48E-15	FEM1C	5.53E-02	1.00E+00	LOC102395698	-4.22E-02	1.00E+00
WDR24	9.10E-01	2.50E-15	LIN52	6.97E-02	1.00E+00	SLC2A8	-4.70E-02	1.00E+00
SHISAL1	4.12E+00	2.54E-15	RNF223	-9.77E-02	1.00E+00	MUM1L1	-5.29E-02	1.00E+00
GUCY1A1	-3.56E+00	2.56E-15	GUF1	5.98E-02	1.00E+00	VPS50	-4.44E-02	1.00E+00
PKP4	8.23E-01	2.59E-15	NOC4L	5.21E-02	1.00E+00	EHBP1L1	1.48E-01	1.00E+00
PDCD6	-1.04E+00	2.61E-15	PKD2	-1.89E-01	1.00E+00	DNM1	7.26E-02	1.00E+00
CAPN10	-1.12E+00	2.66E-15	SLC8A1	4.27E-02	1.00E+00	LOC102404294	-4.30E-02	1.00E+00
APOE	-5.99E+00	2.69E-15	CDS2	-6.45E-02	1.00E+00	SERGEF	-6.53E-02	1.00E+00
LOC112583896	4.33E+00	2.69E-15	LOC102412900	1.05E-01	1.00E+00	OTULIN	4.04E-02	1.00E+00
FAM187A	3.23E+00	2.72E-15	MTREX	6.06E-02	1.00E+00	WDR60	-4.98E-02	1.00E+00
FCRLA	5.59E+00	2.72E-15	LOC102391228	-9.41E-02	1.00E+00	PDHB	-4.36E-02	1.00E+00
GET4	-1.08E+00	2.77E-15	CNOT7	-5.37E-02	1.00E+00	FBXO25	-4.39E-02	1.00E+00
ACYP1	1.39E+00	2.85E-15	IFT80	-1.21E-01	1.00E+00	OVCA2	5.71E-02	1.00E+00
PAF1	9.28E-01	2.86E-15	ARHGEF26	-5.26E-02	1.00E+00	CAND1	-4.38E-02	1.00E+00
LOC112581858	-3.50E+00	2.87E-15	LOC112584513	1.98E-01	1.00E+00	DNA2	-7.98E-02	1.00E+00
MMP19	-3.50E+00	2.93E-15	ANKRD13B	5.71E-02	1.00E+00	GTF3C6	-4.24E-02	1.00E+00
NOP10	-1.50E+00	2.98E-15	MAF1	4.58E-02	1.00E+00	SUMO2	-4.83E-02	1.00E+00
L3MBTL1	-2.14E+00	2.98E-15	LOC112587796	2.29E-01	1.00E+00	TSHZ1	-5.23E-02	1.00E+00
LOC102399336	-2.44E+00	3.08E-15	GLUD1	5.36E-02	1.00E+00	DDX3X	-6.45E-02	1.00E+00
LOC112578444	1.26E+00	3.10E-15	AGBL2	-6.50E-02	1.00E+00	LCMT2	8.96E-02	1.00E+00
OLFM2	2.71E+00	3.16E-15	SGSM1	1.22E-01	1.00E+00	ENPP4	-6.68E-02	1.00E+00
SIPA1L2	-1.14E+00	3.20E-15	LOC102393946	6.30E-02	1.00E+00	NEK10	8.55E-02	1.00E+00
CLCN1	5.19E+00	3.24E-15	LSM7	-6.45E-02	1.00E+00	THOC3	-4.87E-02	1.00E+00
AFMID	-1.37E+00	3.26E-15	PEX2	-4.73E-02	1.00E+00	GRPEL1	4.71E-02	1.00E+00
TBC1D22A	-9.75E-01	3.27E-15	SARDH	-1.37E-01	1.00E+00	HPGDS	1.30E-01	1.00E+00

LOC102392520	-1.38E+00	3.28E-15	LOC102394141	-1.84E-01	1.00E+00	SNRPD2	7.55E-02	1.00E+00
SLCO1A2	4.71E+00	3.31E-15	BCOR	1.18E-01	1.00E+00	IBTK	-4.37E-02	1.00E+00
SLC25A27	8.40E-01	3.31E-15	LOC112586917	-1.57E-01	1.00E+00	TXNRD1	3.89E-02	1.00E+00
U2AF2	9.38E-01	3.42E-15	RBMX	5.14E-02	1.00E+00	ELOC	4.34E-02	1.00E+00
MRPL4	9.74E-01	3.43E-15	NDUFA11	-5.83E-02	1.00E+00	MIF4GD	-4.94E-02	1.00E+00
CETN3	1.12E+00	3.47E-15	MYH11	-1.21E-01	1.00E+00	TMED3	-5.72E-02	1.00E+00
CTTNBP2	1.33E+00	3.49E-15	ATP2A2	6.28E-02	1.00E+00	ZNF598	-4.22E-02	1.00E+00
ZSCAN26	1.15E+00	3.55E-15	TAF11	-5.80E-02	1.00E+00	UBE2A	4.39E-02	1.00E+00
LOC102400858	1.01E+00	3.55E-15	EPS15L1	-4.74E-02	1.00E+00	LOC112580864	-7.76E-02	1.00E+00
XRN1	-1.39E+00	3.59E-15	LOC102409773	1.33E-01	1.00E+00	DOP1A	6.88E-02	1.00E+00
LOC112586931	4.60E+00	3.60E-15	SEC24A	-7.73E-02	1.00E+00	MOSPD3	-6.09E-02	1.00E+00
AAMP	-8.01E-01	3.60E-15	ZNF358	8.52E-02	1.00E+00	CRIM1	5.44E-02	1.00E+00
ARFGAP1	-9.89E-01	3.70E-15	CACNA1C	1.01E-01	1.00E+00	GLOD4	4.74E-02	1.00E+00
AEBP2	-1.00E+00	3.81E-15	BAZ1B	4.86E-02	1.00E+00	LOC102409978	1.03E-01	1.00E+00
LOC102398843	-1.34E+00	3.88E-15	SPOCK1	-5.09E-02	1.00E+00	CMPK1	-4.40E-02	1.00E+00
SEMA6D	-4.19E+00	3.91E-15	NDUFAF4	-4.18E-02	1.00E+00	PFN2	4.47E-02	1.00E+00
ZNF483	1.67E+00	3.98E-15	CRYL1	1.89E-01	1.00E+00	CAMSAP3	4.97E-02	1.00E+00
PRKAB1	8.72E-01	3.99E-15	EIF3B	-3.71E-02	1.00E+00	ATG12	-4.13E-02	1.00E+00
UBE2T	1.43E+00	4.08E-15	FCF1	-8.57E-02	1.00E+00	ME1	-3.96E-02	1.00E+00
LOC112585524	-3.54E+00	4.14E-15	NPL	-1.59E-01	1.00E+00	LOC112577606	-6.48E-02	1.00E+00
DPH3	-1.11E+00	4.27E-15	LOC102396444	-9.69E-02	1.00E+00	LOC112581780	9.82E-02	1.00E+00
GTF3C1	7.29E-01	4.29E-15	TPM2	2.58E-01	1.00E+00	LOC102403045	-9.41E-02	1.00E+00
MSI1	9.99E-01	4.38E-15	ZIC2	2.12E-01	1.00E+00	WDR7	4.84E-02	1.00E+00
LOC102413141	-1.14E+00	4.42E-15	AP3M2	4.74E-02	1.00E+00	DNAH2	1.16E-01	1.00E+00
LOC112580628	3.76E+00	4.43E-15	MINDY1	4.85E-02	1.00E+00	TYSND1	5.09E-02	1.00E+00
UACA	8.93E-01	4.44E-15	PDLIM4	-2.63E-01	1.00E+00	LOC102406426	1.05E-01	1.00E+00
IFT20	-9.80E-01	4.58E-15	SLC35A4	6.41E-02	1.00E+00	NRDE2	-4.57E-02	1.00E+00
ZNF674	-1.29E+00	4.58E-15	SZT2	-8.94E-02	1.00E+00	IPO5	4.61E-02	1.00E+00
LY6K	4.76E+00	4.59E-15	DOCK3	6.55E-02	1.00E+00	RPS6KA1	8.28E-02	1.00E+00
ZBTB21	-1.54E+00	4.79E-15	PLEKHJ1	-6.36E-02	1.00E+00	CRLF3	-4.87E-02	1.00E+00
TREM1	-6.07E+00	4.83E-15	NR2E3	2.79E-01	1.00E+00	SKA2	-5.19E-02	1.00E+00
PPP2R5B	-8.74E-01	4.92E-15	EPS8L2	-2.60E-01	1.00E+00	TIMM44	4.71E-02	1.00E+00
LOC112586106	4.89E+00	4.99E-15	SLC39A11	7.56E-02	1.00E+00	TMEM51	9.00E-02	1.00E+00
TRMT112	1.26E+00	4.99E-15	UBB	-7.01E-02	1.00E+00	DNTTIP2	-6.20E-02	1.00E+00

HIBCH	-9.20E-01	5.05E-15	PCNA	-4.42E-02	1.00E+00	SMIM19	-4.49E-02	1.00E+00
OSER1	8.88E-01	5.09E-15	MAP3K5	1.27E-01	1.00E+00	BPGM	5.36E-02	1.00E+00
RBM42	9.94E-01	5.20E-15	PKD1L2	-5.76E-02	1.00E+00	UNC50	-4.80E-02	1.00E+00
DLG5	-1.20E+00	5.22E-15	CDH26	2.26E-01	1.00E+00	LOC102390935	-6.65E-02	1.00E+00
ZNF507	-1.16E+00	5.25E-15	KCNMB4	5.16E-02	1.00E+00	NME3	7.22E-02	1.00E+00
MBD5	-1.02E+00	5.27E-15	LRFN2	2.96E-01	1.00E+00	PTCH1	-5.80E-02	1.00E+00
GRM1	1.68E+00	5.38E-15	CTDSPL	-1.24E-01	1.00E+00	RAMP2	-1.28E-01	1.00E+00
LOC102410120	-4.40E+00	5.43E-15	LNPK	6.66E-02	1.00E+00	CA9	-8.36E-02	1.00E+00
PEX16	-1.34E+00	5.49E-15	ADSS	4.18E-02	1.00E+00	SNAPC2	5.01E-02	1.00E+00
KPNA6	7.63E-01	5.52E-15	FDPS	5.76E-02	1.00E+00	GPATCH11	4.50E-02	1.00E+00
STK11	-9.03E-01	5.56E-15	SLC22A5	-6.98E-02	1.00E+00	LOC102397527	-9.03E-02	1.00E+00
TCF25	-8.87E-01	5.59E-15	GPATCH2	8.60E-02	1.00E+00	USP16	-3.89E-02	1.00E+00
CREG2	2.40E+00	5.61E-15	CADM3	1.32E-01	1.00E+00	SH3BGRL3	5.69E-02	1.00E+00
LOC102393767	2.52E+00	5.68E-15	LRRN4	-1.08E-01	1.00E+00	MED18	6.35E-02	1.00E+00
COQ5	-1.04E+00	5.74E-15	LOC102405919	-2.25E-01	1.00E+00	SLC5A2	-5.40E-02	1.00E+00
NUDT16	-1.34E+00	5.74E-15	GALM	-2.52E-01	1.00E+00	ITGB4	-1.06E-01	1.00E+00
MAP4K2	1.14E+00	5.77E-15	MAGT1	5.92E-02	1.00E+00	CXXC4	9.61E-02	1.00E+00
LAMA2	1.27E+00	5.80E-15	PTRH2	5.67E-02	1.00E+00	STK11	4.42E-02	1.00E+00
CDC27	8.87E-01	5.80E-15	LOC112585626	8.40E-02	1.00E+00	SLC13A5	-1.09E-01	1.00E+00
RDH14	8.10E-01	5.81E-15	OSBP2	-8.39E-02	1.00E+00	LOC102407057	4.22E-02	1.00E+00
PGF	-3.54E+00	5.83E-15	SPTBN4	1.54E-01	1.00E+00	DUSP23	8.78E-02	1.00E+00
ACSL3	8.70E-01	5.89E-15	WDR82	4.56E-02	1.00E+00	TNPO3	3.91E-02	1.00E+00
PTRH1	-3.39E+00	5.90E-15	DYNC1LI1	-6.05E-02	1.00E+00	SMARCC1	4.95E-02	1.00E+00
ATF2	-7.45E-01	6.05E-15	KLC2	5.91E-02	1.00E+00	LOC102409758	-9.92E-02	1.00E+00
SMARCD2	8.90E-01	6.17E-15	HINFP	4.59E-02	1.00E+00	TMEM64	5.84E-02	1.00E+00
NFKBIL1	-1.40E+00	6.21E-15	SELENOM	-1.02E-01	1.00E+00	USP27X	6.04E-02	1.00E+00
DEF8	8.84E-01	6.23E-15	TMEM59	-5.51E-02	1.00E+00	SPATA24	-5.50E-02	1.00E+00
LAMP2	-8.17E-01	6.34E-15	MED12L	5.39E-02	1.00E+00	LARS2	-4.02E-02	1.00E+00
PRPF6	8.68E-01	6.34E-15	LOC112578238	1.90E-01	1.00E+00	MRPS36	5.02E-02	1.00E+00
MATK	-3.69E+00	6.39E-15	GTF2A1L	-3.95E-02	1.00E+00	KCTD15	6.27E-02	1.00E+00
BAHD1	9.32E-01	6.43E-15	ONECUT2	6.81E-02	1.00E+00	UBE2R2	-4.12E-02	1.00E+00
LOC102395341	-1.10E+00	6.61E-15	ARFRP1	-7.60E-02	1.00E+00	RECQL5	4.82E-02	1.00E+00
SLC27A5	1.46E+00	6.62E-15	RUNX1	-1.63E-01	1.00E+00	MTDH	-4.39E-02	1.00E+00
FAM19A1	4.26E+00	6.62E-15	ZNF527	1.71E-01	1.00E+00	BRD4	-4.29E-02	1.00E+00
KLF11	8.51E-01	6.72E-15	LOC112578154	2.29E-01	1.00E+00	SF3B6	5.45E-02	1.00E+00

LOC102398505	3.27E+00	6.84E-15	JMY	9.20E-02	1.00E+00	CELSR3	-4.68E-02	1.00E+00
PER3	-2.29E+00	6.87E-15	CHST4	-2.19E-01	1.00E+00	LOC102405491	9.25E-02	1.00E+00
IST1	6.84E-01	6.87E-15	SRSF7	-4.63E-02	1.00E+00	NUP37	-5.06E-02	1.00E+00
HIF1AN	-7.34E-01	6.87E-15	NICN1	-6.61E-02	1.00E+00	CMC2	6.26E-02	1.00E+00
AGL	-2.08E+00	6.90E-15	LOC112580682	-2.43E-01	1.00E+00	ADAP1	-1.41E-01	1.00E+00
BLMH	7.51E-01	7.12E-15	NCOA4	4.18E-02	1.00E+00	NFKBIB	6.04E-02	1.00E+00
ANK1	3.34E+00	7.14E-15	SMC3	1.60E-01	1.00E+00	EPHB3	9.71E-02	1.00E+00
IRAK2	1.53E+00	7.14E-15	C4H12orf10	-6.16E-02	1.00E+00	NENF	6.06E-02	1.00E+00
LOC112583836	-9.55E-01	7.23E-15	FLOT2	5.35E-02	1.00E+00	MEGF8	4.38E-02	1.00E+00
CANT1	8.73E-01	7.30E-15	LOC112581279	-3.43E-01	1.00E+00	LOC102409332	-5.01E-02	1.00E+00
MRPL30	-1.25E+00	7.38E-15	GPRIN2	-3.73E-01	1.00E+00	SOGA1	-6.49E-02	1.00E+00
SLC25A26	8.30E-01	7.39E-15	STAM	1.04E-01	1.00E+00	SYT5	7.05E-02	1.00E+00
ZSWIM5	1.69E+00	7.51E-15	ADAM22	-2.10E-01	1.00E+00	LOC112584001	9.22E-02	1.00E+00
BMT2	-1.14E+00	7.55E-15	LOC112577767	2.15E-01	1.00E+00	LOC102396276	-6.15E-02	1.00E+00
GOLIM4	9.90E-01	7.57E-15	ATP5MC2	-6.63E-02	1.00E+00	HMGCS2	-1.07E-01	1.00E+00
LOC112577787	2.11E+00	7.58E-15	FBXL22	-1.88E-01	1.00E+00	GLO1	-4.50E-02	1.00E+00
CYSRT1	-2.61E+00	7.79E-15	N4BP2L2	5.04E-02	1.00E+00	BEND3	-8.23E-02	1.00E+00
LOC102396583	-6.73E+00	7.93E-15	ESYT3	-5.69E-02	1.00E+00	VLDLR	-4.86E-02	1.00E+00
LOC102393170	-3.14E+00	7.93E-15	ARHGEF1	-8.03E-02	1.00E+00	SMIM11A	-7.03E-02	1.00E+00
SIRPA	-3.68E+00	7.95E-15	IMP4	-4.22E-02	1.00E+00	SFR1	-5.87E-02	1.00E+00
POP1	8.11E-01	7.97E-15	LOC102414455	-1.28E-01	1.00E+00	COQ3	4.18E-02	1.00E+00
MCMBP	7.36E-01	8.06E-15	KCNC3	7.55E-02	1.00E+00	SIAH1	-5.73E-02	1.00E+00
CDKL4	-2.52E+00	8.06E-15	SCMH1	5.71E-02	1.00E+00	LOC102397198	7.08E-02	1.00E+00
PLA2G2F	4.46E+00	8.09E-15	EN2	-1.47E-01	1.00E+00	LOC102398755	7.30E-02	1.00E+00
HPSE	-3.20E+00	8.11E-15	HCN3	5.98E-02	1.00E+00	GPR68	9.00E-02	1.00E+00
DUSP19	1.16E+00	8.13E-15	LOC112586469	-2.15E-01	1.00E+00	KLRG2	8.41E-02	1.00E+00
MBLAC2	-1.72E+00	8.37E-15	EXOC3L1	7.14E-02	1.00E+00	LEPROT	-4.94E-02	1.00E+00
LOC102408954	4.37E+00	8.38E-15	AASDH	4.87E-02	1.00E+00	ATXN7L3	4.18E-02	1.00E+00
LOC112586983	2.81E+00	8.41E-15	PPP1R3D	6.39E-02	1.00E+00	COPS6	6.08E-02	1.00E+00
LOC102400779	-2.30E+00	8.45E-15	ADARB1	-6.10E-02	1.00E+00	PARG	4.62E-02	1.00E+00
NCALD	-2.86E+00	8.46E-15	RPS13	1.29E-01	1.00E+00	CPED1	-5.53E-02	1.00E+00
ARHGEF7	6.33E-01	8.50E-15	HSPA4L	-3.98E-02	1.00E+00	TLR2	1.58E-01	1.00E+00
ST14	-3.35E+00	8.51E-15	ACOT9	-4.00E-02	1.00E+00	C12H9orf78	-4.59E-02	1.00E+00
LOC102408538	3.65E+00	8.55E-15	HTRA4	-7.71E-02	1.00E+00	DECR2	-5.99E-02	1.00E+00

SPAG8	-1.99E+00	8.55E-15	YPEL2	5.03E-02	1.00E+00	ADCY9	4.19E-02	1.00E+00
LAMP1	-9.32E-01	8.68E-15	LOC102399526	-2.50E-01	1.00E+00	KLHL21	-4.78E-02	1.00E+00
RGS22	1.04E+00	9.07E-15	IWS1	4.74E-02	1.00E+00	FAM192A	-4.73E-02	1.00E+00
ADAMTS19	-7.96E-01	9.30E-15	FAM155A	-1.59E-01	1.00E+00	LOC102402068	-4.77E-02	1.00E+00
NDEL1	1.00E+00	9.40E-15	XAB2	4.20E-02	1.00E+00	KIF3C	4.79E-02	1.00E+00
ZNF438	9.47E-01	9.49E-15	PCLAF	-1.54E-01	1.00E+00	LOC102405841	-9.88E-02	1.00E+00
NKAPL	-1.06E+00	9.61E-15	VIT	-2.35E-01	1.00E+00	ITM2B	4.62E-02	1.00E+00
TMED3	-1.15E+00	9.61E-15	PPIC	-1.95E-01	1.00E+00	MPV17	4.44E-02	1.00E+00
LOC102413215	-1.76E+00	9.67E-15	RAVER2	-9.64E-02	1.00E+00	CCSER2	5.47E-02	1.00E+00
SUMO2	9.94E-01	9.77E-15	MYL6	-4.91E-02	1.00E+00	GTF2A1	-4.63E-02	1.00E+00
SCN3A	-4.19E+00	9.85E-15	EPHX1	1.57E-01	1.00E+00	PAPOLG	5.73E-02	1.00E+00
LOC102403261	-3.36E+00	9.92E-15	COPE	-4.39E-02	1.00E+00	LOC102402751	-1.18E-01	1.00E+00
LOC102400624	-9.08E-01	9.96E-15	SPAST	4.16E-02	1.00E+00	TCEA2	-5.74E-02	1.00E+00
LOC102396998	-1.06E+00	1.00E-14	LOC112586104	-1.02E-01	1.00E+00	HAX1	-5.44E-02	1.00E+00
LOC102390492	3.57E+00	1.02E-14	SUOX	-5.17E-02	1.00E+00	PSME4	4.82E-02	1.00E+00
RNF150	-1.89E+00	1.03E-14	PINX1	5.12E-02	1.00E+00	FAM207A	6.73E-02	1.00E+00
LOC112585593	4.64E+00	1.04E-14	GATD3A	-5.34E-02	1.00E+00	HTR1F	-1.23E-01	1.00E+00
TRPT1	1.35E+00	1.04E-14	LOC102409570	-5.90E-02	1.00E+00	MOB1A	4.51E-02	1.00E+00
RAD1	-1.46E+00	1.05E-14	MTDH	-5.51E-02	1.00E+00	KCNJ8	-8.71E-02	1.00E+00
LOC102394594	1.50E+00	1.06E-14	PHKA1	-8.87E-02	1.00E+00	FZR1	-4.14E-02	1.00E+00
LOC112578742	3.84E+00	1.07E-14	MAU2	4.24E-02	1.00E+00	PI4KB	-3.75E-02	1.00E+00
TMC2	2.38E+00	1.10E-14	UBE4B	5.62E-02	1.00E+00	THEMIS2	-1.44E-01	1.00E+00
LOC112587833	3.36E+00	1.11E-14	MPZL1	7.63E-02	1.00E+00	DNAAF4	-5.62E-02	1.00E+00
LGR6	-2.84E+00	1.14E-14	CARHSP1	6.32E-02	1.00E+00	LOC102400385	-6.17E-02	1.00E+00
LOC112583856	4.06E+00	1.15E-14	CLEC11A	1.31E-01	1.00E+00	ITCH	5.71E-02	1.00E+00
CD68	-4.31E+00	1.16E-14	TMIGD1	-6.60E-02	1.00E+00	C16H11orf16	-7.91E-02	1.00E+00
NPM3	1.59E+00	1.16E-14	LOC102393823	-1.91E-01	1.00E+00	BDP1	-6.75E-02	1.00E+00
FBXL20	1.03E+00	1.16E-14	EXT1	-3.68E-02	1.00E+00	ASTE1	-5.49E-02	1.00E+00
PIGM	1.13E+00	1.17E-14	POLA2	4.22E-02	1.00E+00	POLD4	6.66E-02	1.00E+00
IL1R1	1.38E+00	1.17E-14	LOC102401224	2.05E-01	1.00E+00	LSM14B	-4.02E-02	1.00E+00
KLK12	-2.93E+00	1.19E-14	SUCLG1	-5.83E-02	1.00E+00	ZNF12	-5.17E-02	1.00E+00
EFCAB10	1.79E+00	1.20E-14	PGM2	2.39E-01	1.00E+00	COG5	-4.21E-02	1.00E+00
SPACA9	-1.97E+00	1.23E-14	RYR3	-6.41E-02	1.00E+00	LOC102392339	-5.21E-02	1.00E+00

SLC12A9	-9.61E-01	1.25E-14	ZSCAN26	-7.15E-02	1.00E+00	LY6G5B	-6.24E-02	1.00E+00
MRPS23	-1.17E+00	1.25E-14	KLHL32	-4.05E-02	1.00E+00	COTL1	8.90E-02	1.00E+00
TMEFF1	-9.70E-01	1.27E-14	ENGASE	-2.30E-01	1.00E+00	METTL5	-4.87E-02	1.00E+00
URB1	-1.22E+00	1.31E-14	LOC102406414	2.69E-01	1.00E+00	RBBP7	-4.03E-02	1.00E+00
LOC112585542	1.91E+00	1.31E-14	LOC102399988	-8.26E-02	1.00E+00	SAMD4B	4.38E-02	1.00E+00
FAM210A	-9.49E-01	1.34E-14	TAZ	-1.14E-01	1.00E+00	TNFAIP3	-7.35E-02	1.00E+00
SLC46A3	-2.01E+00	1.34E-14	LOC102402422	-7.68E-02	1.00E+00	HMG20A	-4.04E-02	1.00E+00
ZFHX2	1.17E+00	1.34E-14	SOCS7	5.89E-02	1.00E+00	FAM98B	-4.48E-02	1.00E+00
WDR43	-7.05E-01	1.36E-14	RPUSD1	-7.72E-02	1.00E+00	NHEJ1	-6.19E-02	1.00E+00
CHORDC1	-7.69E-01	1.36E-14	NRTN	2.02E-01	1.00E+00	VPS4B	-4.99E-02	1.00E+00
CLOCK	1.02E+00	1.37E-14	SEZ6	5.26E-02	1.00E+00	FABP3	7.46E-02	1.00E+00
KPTN	-1.33E+00	1.38E-14	SKOR2	-6.10E-02	1.00E+00	RSPRY1	5.37E-02	1.00E+00
LOC102416310	-3.49E+00	1.38E-14	CLYBL	-6.33E-02	1.00E+00	LOC102415736	-8.44E-02	1.00E+00
SNX19	6.79E-01	1.38E-14	HOMER1	8.89E-02	1.00E+00	RORC	1.22E-01	1.00E+00
TGM7	2.81E+00	1.39E-14	MIGA1	1.40E-01	1.00E+00	MAGI3	-6.92E-02	1.00E+00
RICTOR	1.20E+00	1.44E-14	YWHAB	-3.66E-02	1.00E+00	KCTD3	-4.42E-02	1.00E+00
IER5L	1.54E+00	1.44E-14	AP2M1	4.31E-02	1.00E+00	RPS28	7.38E-02	1.00E+00
TMTC4	-1.20E+00	1.44E-14	ARMC8	-4.90E-02	1.00E+00	RRN3	-4.94E-02	1.00E+00
TNFRSF13B	2.61E+00	1.45E-14	KRT4	-1.73E-01	1.00E+00	C3H17orf53	-9.32E-02	1.00E+00
STARD6	-2.06E+00	1.45E-14	CCDC71L	-1.34E-01	1.00E+00	HSPA14	-3.80E-02	1.00E+00
PTPMT1	-1.67E+00	1.48E-14	LOC102393522	2.38E-01	1.00E+00	NELL2	5.39E-02	1.00E+00
KBTBD4	-1.04E+00	1.50E-14	TSPAN11	1.71E-01	1.00E+00	HNRNPH2	3.95E-02	1.00E+00
TMEM8B	-1.57E+00	1.51E-14	ZC3H3	4.85E-02	1.00E+00	WDPCP	-5.92E-02	1.00E+00
RBM12B	-1.01E+00	1.55E-14	LOC112582960	-1.52E-01	1.00E+00	MED6	4.14E-02	1.00E+00
LOC112586957	-3.15E+00	1.59E-14	SYNCRIP	5.04E-02	1.00E+00	LOC102410728	-9.22E-02	1.00E+00
SPPL2A	-1.09E+00	1.59E-14	DNAAF5	6.64E-02	1.00E+00	ZEB1	7.35E-02	1.00E+00
SNRNP25	1.32E+00	1.60E-14	NR3C2	4.40E-02	1.00E+00	EIF2S1	4.24E-02	1.00E+00
SRPK1	-7.26E-01	1.61E-14	LRRC49	-1.17E-01	1.00E+00	ZFPL1	-4.49E-02	1.00E+00
ZSCAN22	-2.96E+00	1.61E-14	C19H5orf51	6.45E-02	1.00E+00	LOC102399577	-9.25E-02	1.00E+00
LOC102415513	-1.40E+00	1.62E-14	NAV3	4.22E-02	1.00E+00	FAM155B	-8.81E-02	1.00E+00
LOC112583996	-2.76E+00	1.62E-14	SPART	-6.51E-02	1.00E+00	LOC102394013	1.46E-01	1.00E+00
FAM89B	-1.02E+00	1.64E-14	ZNF330	-4.98E-02	1.00E+00	ZNF577	-6.68E-02	1.00E+00
UPB1	3.28E+00	1.65E-14	DNALI1	-7.42E-02	1.00E+00	CDK5RAP1	-4.26E-02	1.00E+00
LOC112587112	2.52E+00	1.66E-14	LOC112582314	2.48E-01	1.00E+00	CD36	9.64E-02	1.00E+00

FAM32A	-1.11E+00	1.66E-14	KCNIP4	6.81E-02	1.00E+00	UBAP1	-3.95E-02	1.00E+00
LOC102410092	-6.84E+00	1.67E-14	HPS5	-6.00E-02	1.00E+00	GSKIP	4.85E-02	1.00E+00
SFR1	1.04E+00	1.68E-14	C1QTNF1	-2.60E-01	1.00E+00	CHST15	5.43E-02	1.00E+00
GDF1	-3.19E+00	1.74E-14	KDM3A	-5.45E-02	1.00E+00	C16H11orf1	9.59E-02	1.00E+00
IKZF3	2.29E+00	1.80E-14	ERG28	7.55E-02	1.00E+00	LOC102410846	-5.72E-02	1.00E+00
LOC102410981	2.22E+00	1.81E-14	GET4	-5.51E-02	1.00E+00	EIF1AX	6.16E-02	1.00E+00
RIBC2	4.27E+00	1.82E-14	CHORDC1	5.40E-02	1.00E+00	ELOVL1	4.20E-02	1.00E+00
POU6F1	-2.78E+00	1.82E-14	TPRN	-5.84E-02	1.00E+00	RPL6	-6.83E-02	1.00E+00
POLL	-9.84E-01	1.83E-14	CCDC90B	-7.35E-02	1.00E+00	LGI3	-8.91E-02	1.00E+00
RFK	9.22E-01	1.83E-14	LOC102390023	2.82E-01	1.00E+00	TXNIP	-4.66E-02	1.00E+00
SNTB2	-7.45E-01	1.84E-14	COMMD8	-5.68E-02	1.00E+00	LIMA1	1.14E-01	1.00E+00
LOC102416626	-4.17E+00	1.84E-14	ZNF862	-6.10E-02	1.00E+00	ABCB7	3.99E-02	1.00E+00
ACO2	-8.52E-01	1.85E-14	STX8	4.27E-02	1.00E+00	S100A4	1.25E-01	1.00E+00
SUPV3L1	8.95E-01	1.88E-14	BLM	4.19E-02	1.00E+00	S100A1	8.04E-02	1.00E+00
RNASE6	-5.93E+00	1.93E-14	HYAL2	-6.81E-02	1.00E+00	PIGBOS1	5.68E-02	1.00E+00
LOC112583811	-3.36E+00	1.93E-14	PRIMPOL	3.62E-02	1.00E+00	RNF41	-6.19E-02	1.00E+00
ZRANB1	7.77E-01	1.95E-14	LOC112583856	2.25E-01	1.00E+00	CCDC115	-3.85E-02	1.00E+00
ZNF239	-1.33E+00	1.96E-14	NIPSNAP2	4.82E-02	1.00E+00	STAU1	-4.02E-02	1.00E+00
NTRK1	-4.00E+00	2.05E-14	PDSS2	5.16E-02	1.00E+00	TMEM234	-6.00E-02	1.00E+00
LOC112586434	3.23E+00	2.06E-14	GATD1	7.99E-02	1.00E+00	TNIP1	4.11E-02	1.00E+00
LOC102393430	2.76E+00	2.08E-14	LOC102404456	1.21E-01	1.00E+00	ZNF653	4.55E-02	1.00E+00
EHHADH	1.85E+00	2.09E-14	NDUFS3	7.50E-02	1.00E+00	GHDC	-5.25E-02	1.00E+00
SLC9A8	-8.74E-01	2.13E-14	LOC112583923	-2.05E-01	1.00E+00	NSA2	-4.55E-02	1.00E+00
LPCAT1	-8.16E-01	2.16E-14	ACOT7	-6.88E-02	1.00E+00	ANO4	-5.56E-02	1.00E+00
IFI44L	-4.37E+00	2.16E-14	BRD3OS	-1.05E-01	1.00E+00	LOC102395334	6.52E-02	1.00E+00
NFE2	3.15E+00	2.17E-14	MCTP2	6.38E-02	1.00E+00	LOC112587551	-9.44E-02	1.00E+00
FBXO32	-1.25E+00	2.22E-14	PKN3	1.52E-01	1.00E+00	BFAR	-3.95E-02	1.00E+00
SLC30A5	-8.47E-01	2.22E-14	COQ10B	-5.47E-02	1.00E+00	HDDC2	5.05E-02	1.00E+00
CTSZ	-1.66E+00	2.23E-14	CHTOP	-3.66E-02	1.00E+00	LOC102412527	-6.04E-02	1.00E+00
C18H16orf95	2.96E+00	2.24E-14	LOC112579227	1.83E-01	1.00E+00	NEU3	-9.07E-02	1.00E+00
CEP170	-2.03E+00	2.28E-14	EPB42	-7.46E-02	1.00E+00	LOC102415288	-1.29E-01	1.00E+00
UBIAD1	1.15E+00	2.29E-14	LMO7	-5.69E-02	1.00E+00	RHOF	-6.10E-02	1.00E+00
KIF11	9.49E-01	2.33E-14	HNRNPK	4.88E-02	1.00E+00	NVL	3.77E-02	1.00E+00
KLHDC3	-8.44E-01	2.33E-14	IER5	-1.38E-01	1.00E+00	LOC102389468	-5.55E-02	1.00E+00

CCDC30	-1.19E+00	2.36E-14	STRIP1	4.19E-02	1.00E+00	RIT1	-4.71E-02	1.00E+00
ZNHIT1	-1.38E+00	2.38E-14	CDKL5	-7.90E-02	1.00E+00	B3GALNT1	5.89E-02	1.00E+00
ERCC6L2	-8.21E-01	2.40E-14	VPS37C	-9.58E-02	1.00E+00	TMEM199	-9.54E-02	1.00E+00
LOC102411134	-2.24E+00	2.43E-14	ATP5MC1	-6.25E-02	1.00E+00	INCA1	7.64E-02	1.00E+00
SLC25A20	9.62E-01	2.44E-14	GAS8	9.58E-02	1.00E+00	LOC112579558	-8.03E-02	1.00E+00
PPM1A	6.93E-01	2.44E-14	FNDC10	7.14E-02	1.00E+00	MAD2L1BP	-5.78E-02	1.00E+00
SLC16A8	4.68E+00	2.46E-14	ZNF25	3.01E-01	1.00E+00	LOC112578122	-9.65E-02	1.00E+00
ARSE	-5.36E+00	2.47E-14	TRA2B	-7.06E-02	1.00E+00	HDGFL2	5.31E-02	1.00E+00
PTPRO	3.51E+00	2.49E-14	SULF2	4.66E-02	1.00E+00	RHOBTB1	4.10E-02	1.00E+00
MTTP	3.31E+00	2.51E-14	TMEM67	-1.81E-01	1.00E+00	RMI1	4.90E-02	1.00E+00
LOC102390532	2.39E+00	2.52E-14	OVOL3	-1.46E-01	1.00E+00	SNURF	-6.26E-02	1.00E+00
MTFP1	-1.51E+00	2.53E-14	LOC112581846	7.60E-02	1.00E+00	PLA2R1	2.01E-01	1.00E+00
DEAF1	-1.08E+00	2.54E-14	LOC112582930	5.48E-02	1.00E+00	LOC102410254	8.20E-02	1.00E+00
POLR3H	1.18E+00	2.55E-14	LOC102394190	-7.26E-02	1.00E+00	MUS81	-4.39E-02	1.00E+00
ZNF461	-1.16E+00	2.61E-14	LOC102399418	-1.75E-01	1.00E+00	SPTLC2	3.80E-02	1.00E+00
GAPVD1	-7.40E-01	2.66E-14	ABCC9	-6.49E-02	1.00E+00	TMEM136	-5.49E-02	1.00E+00
AGTPBP1	1.19E+00	2.78E-14	GRB7	8.98E-02	1.00E+00	C5H1orf74	7.36E-02	1.00E+00
NR2C2	8.96E-01	2.80E-14	ARHGAP31	1.19E-01	1.00E+00	PHF6	5.14E-02	1.00E+00
BMF	-1.43E+00	2.82E-14	HS3ST1	-1.78E-01	1.00E+00	LOC112582076	7.59E-02	1.00E+00
NPRL2	-1.18E+00	2.83E-14	MATR3	-4.36E-02	1.00E+00	ZFP64	4.21E-02	1.00E+00
RAB3GAP1	7.37E-01	2.84E-14	ZBBX	-1.84E-01	1.00E+00	MTUS1	5.55E-02	1.00E+00
LOC112579227	4.34E+00	2.87E-14	ATP6V1C2	-2.50E-01	1.00E+00	IK	-4.19E-02	1.00E+00
LOC102407762	2.61E+00	2.90E-14	VAPB	-3.96E-02	1.00E+00	SDF2L1	-6.96E-02	1.00E+00
TMEM115	-8.65E-01	2.90E-14	LOC112582115	5.34E-02	1.00E+00	MOB3C	-5.45E-02	1.00E+00
SFI1	-9.98E-01	2.94E-14	C9H5orf63	-1.93E-01	1.00E+00	LMLN	-5.47E-02	1.00E+00
ZNF169	-1.51E+00	2.94E-14	IGFBP3	-7.37E-02	1.00E+00	INO80	-3.76E-02	1.00E+00
BTBD19	1.68E+00	2.94E-14	PDE12	-6.40E-02	1.00E+00	DPH2	4.81E-02	1.00E+00
ZNF202	-1.20E+00	2.94E-14	STXBP6	-5.45E-02	1.00E+00	MAP2K4	-3.79E-02	1.00E+00
MANSC1	-1.75E+00	3.00E-14	MED20	5.22E-02	1.00E+00	TMEM150C	1.02E-01	1.00E+00
ATP6	-2.16E+00	3.02E-14	LANCL2	-5.46E-02	1.00E+00	SEC11C	5.58E-02	1.00E+00
DIS3	7.54E-01	3.09E-14	MRPL11	5.85E-02	1.00E+00	SENP5	-4.55E-02	1.00E+00
MTPAP	7.15E-01	3.12E-14	PDE1B	5.15E-02	1.00E+00	BCKDHA	-5.17E-02	1.00E+00
ATP6V0A2	1.21E+00	3.13E-14	LOC102412511	-5.41E-02	1.00E+00	DZANK1	6.12E-02	1.00E+00

LOC102396788	-6.06E+00	3.15E-14	LOC112579694	2.72E-01	1.00E+00	PNMA8A	8.68E-02	1.00E+00
POLE3	9.12E-01	3.18E-14	PWWP2B	-1.95E-01	1.00E+00	VSIG10L	7.62E-02	1.00E+00
MPC1	8.22E-01	3.18E-14	LOC112585588	1.86E-01	1.00E+00	DIMT1	-4.97E-02	1.00E+00
LOC112578241	-2.48E+00	3.18E-14	ALKBH3	-9.55E-02	1.00E+00	PDGFRL	-6.55E-02	1.00E+00
UBC	-1.17E+00	3.19E-14	RTN4IP1	-6.32E-02	1.00E+00	TJP1	-4.81E-02	1.00E+00
CBS	1.21E+00	3.20E-14	ZNF703	6.82E-02	1.00E+00	RNF11	4.99E-02	1.00E+00
SIX4	-4.71E+00	3.24E-14	CRY1	6.18E-02	1.00E+00	LOC102399966	6.90E-02	1.00E+00
CCDC194	-4.05E+00	3.24E-14	C3H9orf64	-5.45E-02	1.00E+00	CHST10	4.63E-02	1.00E+00
NSRP1	8.11E-01	3.25E-14	GJA4	4.70E-02	1.00E+00	SMIM27	8.59E-02	1.00E+00
LOC102402714	-1.66E+00	3.27E-14	LOC102393424	-3.99E-02	1.00E+00	BSG	-6.49E-02	1.00E+00
EXOSC7	-1.02E+00	3.27E-14	OMA1	4.17E-02	1.00E+00	ARF4	-5.62E-02	1.00E+00
EZH2	7.52E-01	3.27E-14	MAVS	-1.24E-01	1.00E+00	ASB8	4.47E-02	1.00E+00
LOC102410484	-2.64E+00	3.38E-14	RABL2B	4.51E-02	1.00E+00	GLTP	4.22E-02	1.00E+00
AHSA2P	-9.63E-01	3.39E-14	KDM6A	-9.39E-02	1.00E+00	APLP2	5.43E-02	1.00E+00
SLC29A3	1.32E+00	3.41E-14	NFAT5	1.25E-01	1.00E+00	CASD1	5.46E-02	1.00E+00
HOXC10	4.05E+00	3.42E-14	RBSN	-1.12E-01	1.00E+00	PPME1	-5.09E-02	1.00E+00
LOC102415015	2.85E+00	3.44E-14	SLC44A5	7.89E-02	1.00E+00	MAP1LC3B	4.79E-02	1.00E+00
DTNBP1	1.21E+00	3.46E-14	INA	-5.45E-02	1.00E+00	CHAF1B	-5.08E-02	1.00E+00
LOC102415270	-9.60E-01	3.49E-14	FIP1L1	4.04E-02	1.00E+00	SMDT1	5.93E-02	1.00E+00
TAC1	6.99E+00	3.49E-14	QARS	-5.53E-02	1.00E+00	FKRP	-5.18E-02	1.00E+00
C4H12orf54	5.93E+00	3.57E-14	UHRF1BP1	-7.94E-02	1.00E+00	RPS3	-7.70E-02	1.00E+00
ERLEC1	6.43E-01	3.61E-14	LOC112581738	-1.57E-01	1.00E+00	COPS5	3.99E-02	1.00E+00
BTF3L4	-8.67E-01	3.68E-14	EFHD1	6.52E-02	1.00E+00	ATRX	5.79E-02	1.00E+00
MIEF1	8.07E-01	3.76E-14	ADGRA1	4.04E-01	1.00E+00	TEAD1	4.81E-02	1.00E+00
LOC102410358	-4.75E+00	3.77E-14	MPHOSPH8	4.22E-02	1.00E+00	ZC3H13	4.46E-02	1.00E+00
MSANTD1	-3.75E+00	3.78E-14	TEK	3.99E-02	1.00E+00	PPM1G	-4.75E-02	1.00E+00
LOC112582314	3.42E+00	3.79E-14	SMARCB1	4.33E-02	1.00E+00	TSEN54	-4.63E-02	1.00E+00
KLF7	9.51E-01	3.82E-14	CENPE	5.94E-02	1.00E+00	LRRC8D	3.81E-02	1.00E+00
AKAP14	-3.11E+00	3.82E-14	LOC112583962	1.02E-01	1.00E+00	HSF5	-9.08E-02	1.00E+00
SLC30A10	-4.06E+00	3.84E-14	ABCB9	1.17E-01	1.00E+00	ODF2L	4.96E-02	1.00E+00
TRMT10C	-1.07E+00	3.92E-14	KCNG3	-1.80E-01	1.00E+00	NKPD1	7.45E-02	1.00E+00
AJM1	2.83E+00	3.94E-14	CDKN2D	1.39E-01	1.00E+00	LOC102410721	6.85E-02	1.00E+00
TTC7B	-1.25E+00	3.98E-14	SETBP1	-1.86E-01	1.00E+00	CAPN1	-4.06E-02	1.00E+00
PDAP1	-1.04E+00	4.04E-14	CCDC85A	-7.60E-02	1.00E+00	SLC26A11	-5.18E-02	1.00E+00
FOXB1	7.13E+00	4.09E-14	TSPAN9	2.27E-01	1.00E+00	CBL	6.65E-02	1.00E+00

DNAI1	-1.42E+00	4.12E-14	ATP6V1D	-5.84E-02	1.00E+00	LYSMD1	-4.99E-02	1.00E+00
CLUH	8.04E-01	4.14E-14	HUS1	-5.20E-02	1.00E+00	TMEM246	-4.02E-02	1.00E+00
LOC112579226	-3.14E+00	4.15E-14	APBB3	-1.58E-01	1.00E+00	LOC102389062	-6.99E-02	1.00E+00
LOC112580307	-3.25E+00	4.16E-14	UBE2U	-5.90E-02	1.00E+00	CEBPG	4.16E-02	1.00E+00
CCER2	-3.11E+00	4.16E-14	FBXO43	-8.86E-02	1.00E+00	TRMT12	4.99E-02	1.00E+00
ZC3H10	8.33E-01	4.17E-14	C1D	-1.01E-01	1.00E+00	HPCAL1	4.13E-02	1.00E+00
MAST3	1.22E+00	4.19E-14	CPM	5.31E-02	1.00E+00	KLK5	-1.21E-01	1.00E+00
LYAR	7.88E-01	4.23E-14	ZNF691	3.90E-02	1.00E+00	DCAF5	3.89E-02	1.00E+00
TWNK	-1.19E+00	4.31E-14	LOC102412874	1.85E-01	1.00E+00	TDRD5	-9.95E-02	1.00E+00
OXR1	-1.14E+00	4.32E-14	SLC2A4RG	-1.38E-01	1.00E+00	TMEM186	5.92E-02	1.00E+00
FAM120B	-8.26E-01	4.33E-14	HIVEP3	-8.21E-02	1.00E+00	LOC102405126	-7.96E-02	1.00E+00
NPFFR1	4.51E+00	4.42E-14	NEU1	-1.20E-01	1.00E+00	LOC112581779	6.93E-02	1.00E+00
LOC102394892	-1.67E+00	4.49E-14	CCDC142	1.19E-01	1.00E+00	EFCAB2	-5.05E-02	1.00E+00
MRPS7	1.01E+00	4.51E-14	PITX1	-1.11E-01	1.00E+00	UROS	-5.91E-02	1.00E+00
ADRA1D	2.33E+00	4.58E-14	LOC112587308	-9.51E-02	1.00E+00	LOC112578958	-6.60E-02	1.00E+00
RPAIN	1.20E+00	4.58E-14	TCF7	7.50E-02	1.00E+00	ZDHHC3	-3.68E-02	1.00E+00
TAS1R1	3.72E+00	4.60E-14	ILDR1	4.31E-02	1.00E+00	SIT1	1.08E-01	1.00E+00
AMPH	1.52E+00	4.68E-14	LOC112587446	-2.04E-01	1.00E+00	LOC112586128	8.54E-02	1.00E+00
IGSF9	-4.29E+00	4.70E-14	UFL1	4.93E-02	1.00E+00	RBM45	-3.82E-02	1.00E+00
LOC112586816	-4.44E+00	4.73E-14	SOHLH1	6.66E-02	1.00E+00	NOVA1	-4.50E-02	1.00E+00
UBE2R2	7.65E-01	4.81E-14	DHCR7	-4.55E-02	1.00E+00	RAPGEF2	-3.80E-02	1.00E+00
LOC112581805	-4.97E+00	4.86E-14	STAM2	9.00E-02	1.00E+00	USP32	7.72E-02	1.00E+00
LDLRAP1	7.57E-01	4.93E-14	ABHD8	-4.73E-02	1.00E+00	E2F5	-5.46E-02	1.00E+00
BRAT1	-1.07E+00	4.93E-14	ARL6IP1	-3.86E-02	1.00E+00	JKAMP	4.49E-02	1.00E+00
AIF1	-5.54E+00	5.04E-14	VCAN	2.87E-01	1.00E+00	NAIF1	-5.30E-02	1.00E+00
DDX19A	6.80E-01	5.07E-14	DCTN5	-5.90E-02	1.00E+00	SELENON	3.80E-02	1.00E+00
CCDC59	9.53E-01	5.13E-14	NKTR	-6.21E-02	1.00E+00	TOR3A	5.21E-02	1.00E+00
SLC16A12	-4.06E+00	5.26E-14	LOC102399489	-5.75E-02	1.00E+00	TMEM185A	-4.05E-02	1.00E+00
TMEM150C	-2.46E+00	5.31E-14	LOC102405932	1.74E-01	1.00E+00	RAD54L2	-6.59E-02	1.00E+00
LOC112579539	-3.45E+00	5.36E-14	RPRD1B	9.11E-02	1.00E+00	TFPT	6.05E-02	1.00E+00
HK3	-2.77E+00	5.37E-14	FNDC7	-7.72E-02	1.00E+00	MYDGF	5.37E-02	1.00E+00
DOK3	-3.02E+00	5.44E-14	ARHGAP4	-1.05E-01	1.00E+00	GET4	-4.93E-02	1.00E+00
RFC4	1.11E+00	5.49E-14	LARGE1	4.44E-02	1.00E+00	BRMS1	4.75E-02	1.00E+00

ZBBX	-2.37E+00	5.50E-14	TEF	-5.22E-02	1.00E+00	MREG	-1.01E-01	1.00E+00
CXCL13	6.09E+00	5.67E-14	MRPL55	-5.84E-02	1.00E+00	SMC5	-5.27E-02	1.00E+00
VPS18	8.17E-01	5.81E-14	SGMS2	-4.17E-02	1.00E+00	LOC112585115	1.01E-01	1.00E+00
PDIA5	1.05E+00	5.82E-14	LOC112587959	1.72E-01	1.00E+00	LOC102394237	-9.00E-02	1.00E+00
MFSD1	7.96E-01	5.91E-14	SNX27	-7.26E-02	1.00E+00	MED21	-4.42E-02	1.00E+00
C9H5orf63	-1.87E+00	5.98E-14	GNPNAT1	-5.07E-02	1.00E+00	KRTCAP2	5.67E-02	1.00E+00
S100PBP	-8.30E-01	5.99E-14	BMP4	-6.47E-02	1.00E+00	HORMAD1	-7.92E-02	1.00E+00
LOC112580779	-3.31E+00	6.00E-14	LOC112585634	-8.35E-02	1.00E+00	ZC2HC1C	-8.05E-02	1.00E+00
LOC102416032	6.47E+00	6.05E-14	EME2	1.26E-01	1.00E+00	PTDSS2	4.04E-02	1.00E+00
OFD1	7.57E-01	6.07E-14	HDAC4	7.46E-02	1.00E+00	RICTOR	6.07E-02	1.00E+00
TMEFF2	3.60E+00	6.16E-14	IER3IP1	6.51E-02	1.00E+00	PIM1	8.46E-02	1.00E+00
DET1	8.69E-01	6.33E-14	C6H1orf87	-1.26E-01	1.00E+00	LOC112584193	-6.53E-02	1.00E+00
CAMTA1	1.05E+00	6.33E-14	FGF20	-1.04E-01	1.00E+00	LSM3	5.94E-02	1.00E+00
ESRRA	-9.91E-01	6.40E-14	DZANK1	-2.85E-01	1.00E+00	TCAP	-5.78E-02	1.00E+00
LOC102395589	4.81E+00	6.51E-14	BMT2	8.96E-02	1.00E+00	WDR61	4.65E-02	1.00E+00
DHX40	7.73E-01	6.52E-14	N4BP1	-6.55E-02	1.00E+00	DCLK3	5.83E-02	1.00E+00
LOC112583654	-2.25E+00	6.55E-14	SLC29A4	-4.53E-02	1.00E+00	DNAJC17	-5.04E-02	1.00E+00
LOC112582172	1.45E+00	6.59E-14	LOC112578445	-2.65E-01	1.00E+00	C2H6orf89	-3.73E-02	1.00E+00
PUS1	-1.07E+00	6.70E-14	ZNF23	-5.79E-02	1.00E+00	DDHD2	-4.63E-02	1.00E+00
ATP2B3	-2.77E+00	6.77E-14	GNA11	-5.91E-02	1.00E+00	CDIPT	4.75E-02	1.00E+00
C1QBP	-1.07E+00	6.79E-14	ALMS1	-6.34E-02	1.00E+00	TSTA3	-5.05E-02	1.00E+00
LOC112580379	3.39E+00	6.91E-14	MTIF3	-9.17E-02	1.00E+00	PCF11	-4.40E-02	1.00E+00
ABHD17B	7.56E-01	7.00E-14	RAPGEF3	6.78E-02	1.00E+00	LOC102392405	5.49E-02	1.00E+00
TRIR	-1.14E+00	7.01E-14	SH3D21	-1.25E-01	1.00E+00	NTMT1	-5.53E-02	1.00E+00
LOC102401621	-4.15E+00	7.08E-14	ATG16L2	1.20E-01	1.00E+00	IFT81	4.96E-02	1.00E+00
POLR2M	7.66E-01	7.08E-14	DAGLB	-6.51E-02	1.00E+00	TRAF3	4.28E-02	1.00E+00
ASAP3	1.80E+00	7.09E-14	SLC9A7	-1.95E-01	1.00E+00	PIR	-6.39E-02	1.00E+00
LOC102412912	-3.70E+00	7.09E-14	LGALS1	-4.95E-02	1.00E+00	RNF141	6.13E-02	1.00E+00
CD83	-4.34E+00	7.21E-14	HSPA12A	1.16E-01	1.00E+00	GPM6B	-5.71E-02	1.00E+00
COL12A1	-5.66E+00	7.24E-14	SCLT1	3.87E-02	1.00E+00	ZADH2	4.19E-02	1.00E+00
ANKRD44	1.41E+00	7.27E-14	ATP8B2	-8.03E-02	1.00E+00	RAB30	3.69E-02	1.00E+00
LOC102399794	3.65E+00	7.29E-14	HBP1	3.69E-02	1.00E+00	SMG1	-5.52E-02	1.00E+00
ISLR2	-3.32E+00	7.37E-14	PLPPR3	-6.23E-02	1.00E+00	C4H12orf57	-7.05E-02	1.00E+00
LOC112580305	-1.62E+00	7.41E-14	NGB	-3.06E-01	1.00E+00	RSPH3	-5.49E-02	1.00E+00

TRPV4	1.68E+00	7.43E-14	C5H11orf95	-7.67E-02	1.00E+00	API5	-7.32E-02	1.00E+00
HPGDS	-4.83E+00	7.47E-14	RAD18	-3.72E-02	1.00E+00	CD2BP2	5.03E-02	1.00E+00
LPIN3	-1.24E+00	7.57E-14	PDCD1	-2.41E-01	1.00E+00	NEFM	9.07E-02	1.00E+00
LOC112578805	-3.05E+00	7.74E-14	SIM1	-1.23E-01	1.00E+00	PLPP2	1.07E-01	1.00E+00
ACTN2	-1.33E+00	7.75E-14	RBM11	7.46E-02	1.00E+00	DUSP8	-1.03E-01	1.00E+00
SPOCD1	-2.78E+00	7.76E-14	HECTD2	9.81E-02	1.00E+00	TMEM208	5.29E-02	1.00E+00
LOC112578654	4.92E+00	7.81E-14	FKRP	1.51E-01	1.00E+00	ZBTB6	-9.77E-02	1.00E+00
UTF1	3.63E+00	7.83E-14	LOC102393839	3.86E-02	1.00E+00	ZNF197	-4.55E-02	1.00E+00
METRNL	1.51E+00	8.07E-14	EMILIN1	-8.99E-02	1.00E+00	LGR6	-8.45E-02	1.00E+00
TSHZ3	-2.56E+00	8.13E-14	PCMT1	5.61E-02	1.00E+00	PITPNA	3.65E-02	1.00E+00
PIK3CB	-1.08E+00	8.18E-14	FBLN5	-5.30E-02	1.00E+00	LOC102409492	-7.86E-02	1.00E+00
FAM167A	-4.06E+00	8.19E-14	FXR2	3.72E-02	1.00E+00	GDAP2	4.20E-02	1.00E+00
PARVA	-8.77E-01	8.30E-14	PNPLA2	-7.54E-02	1.00E+00	MTMR3	-7.75E-02	1.00E+00
TMC8	1.16E+00	8.36E-14	IPO5	5.50E-02	1.00E+00	NSUN7	-5.57E-02	1.00E+00
TTC9	-2.55E+00	8.41E-14	ENO3	-1.25E-01	1.00E+00	PAFAH1B2	3.66E-02	1.00E+00
LOC102393439	1.78E+00	8.42E-14	TMEM179B	-8.87E-02	1.00E+00	ATE1	3.99E-02	1.00E+00
RORB	3.93E+00	8.54E-14	LOC112586879	-9.54E-02	1.00E+00	SPRYD7	5.30E-02	1.00E+00
PHIP	-1.07E+00	8.78E-14	LOC112586123	-5.42E-02	1.00E+00	LOC102410181	9.61E-02	1.00E+00
TGFB1	-1.47E+00	9.00E-14	ABCG8	6.50E-02	1.00E+00	ZNF862	4.67E-02	1.00E+00
DNM1L	9.95E-01	9.06E-14	WRAP53	5.46E-02	1.00E+00	ELF1	7.22E-02	1.00E+00
LOC112582913	1.98E+00	9.11E-14	FAM102A	1.19E-01	1.00E+00	LOC102412910	-7.77E-02	1.00E+00
XRCC2	1.08E+00	9.13E-14	MANBA	-4.40E-02	1.00E+00	NFX1	-3.77E-02	1.00E+00
ZNF391	2.61E+00	9.23E-14	LOC112581895	-6.50E-02	1.00E+00	TEKT2	6.43E-02	1.00E+00
TRMT2B	-2.31E+00	9.48E-14	C24H16orf89	1.89E-01	1.00E+00	DRG2	-4.25E-02	1.00E+00
LOC112580206	2.42E+00	9.61E-14	PAK1	-5.37E-02	1.00E+00	DDX27	-4.16E-02	1.00E+00
MSANTD3	1.10E+00	9.61E-14	C18H16orf46	5.05E-02	1.00E+00	USP10	-3.77E-02	1.00E+00
RDX	8.95E-01	9.70E-14	FBN1	-9.19E-02	1.00E+00	TRMO	-6.40E-02	1.00E+00
TLR2	2.70E+00	9.71E-14	HDAC6	9.29E-02	1.00E+00	TOP3B	-4.16E-02	1.00E+00
NOSTRIN	9.88E-01	9.81E-14	FAM221A	2.55E-01	1.00E+00	ERP27	-6.68E-02	1.00E+00
CFAP65	4.13E+00	9.85E-14	EMSY	-1.81E-01	1.00E+00	TMEM18	-4.43E-02	1.00E+00
SIK1	-2.01E+00	9.91E-14	ANO10	3.98E-02	1.00E+00	TOM1L1	4.39E-02	1.00E+00
PARD6B	1.42E+00	9.98E-14	ZNF446	1.33E-01	1.00E+00	CDK6	-3.97E-02	1.00E+00
DGAT1	-	1.00E-13	UBL7	8.00E-02	1.00E+00	PM20D2	-6.29E-	1.00E+00

	1.03E+00						02	
AQR	-9.99E-01	1.01E-13	MCRIP1	4.61E-02	1.00E+00	RNF6	4.95E-02	1.00E+00
LBP	-3.94E+00	1.02E-13	KLK12	1.33E-01	1.00E+00	CCDC163	-9.44E-02	1.00E+00
LOC102404482	-7.18E+00	1.02E-13	GCHFR	1.25E-01	1.00E+00	TDRD7	-4.51E-02	1.00E+00
LOC112578513	2.24E+00	1.02E-13	PHIP	7.43E-02	1.00E+00	SEPT7	-4.11E-02	1.00E+00
ATXN3	9.00E-01	1.03E-13	GPR161	5.99E-02	1.00E+00	ORAI1	-6.10E-02	1.00E+00
GHITM	7.52E-01	1.04E-13	ACCS	-1.90E-01	1.00E+00	ZNF215	-9.49E-02	1.00E+00
LOC102395684	-1.81E+00	1.04E-13	GRM1	-7.29E-02	1.00E+00	TOMM20	4.64E-02	1.00E+00
NET1	-7.10E-01	1.07E-13	EIF2S2	-4.23E-02	1.00E+00	MINPP1	4.96E-02	1.00E+00
CGRRF1	-1.65E+00	1.07E-13	LOC102401116	-3.25E-01	1.00E+00	GKAP1	-6.71E-02	1.00E+00
LOC112587551	-2.07E+00	1.07E-13	RUFY2	6.02E-02	1.00E+00	C5H1orf21	4.69E-02	1.00E+00
UBE2W	-7.22E-01	1.09E-13	PIDD1	1.75E-01	1.00E+00	TUT1	4.30E-02	1.00E+00
KDM6B	-1.41E+00	1.12E-13	LOC102403233	3.63E-02	1.00E+00	TTC37	4.03E-02	1.00E+00
GPR155	-1.29E+00	1.12E-13	LOC102396719	7.79E-02	1.00E+00	ZFP1	7.01E-02	1.00E+00
DUS1L	-1.17E+00	1.13E-13	LOC112580609	-2.19E-01	1.00E+00	LOC102406199	4.15E-02	1.00E+00
GALNT11	7.79E-01	1.14E-13	LOC112584978	1.35E-01	1.00E+00	TPD52	-3.76E-02	1.00E+00
EFCAB8	-1.54E+00	1.14E-13	AKT2	4.66E-02	1.00E+00	WIZ	3.75E-02	1.00E+00
C6H1orf189	-1.87E+00	1.14E-13	TRIM28	3.61E-02	1.00E+00	KNSTRN	-5.26E-02	1.00E+00
CCDC106	9.78E-01	1.15E-13	TRPM6	6.71E-02	1.00E+00	SLMAP	-4.24E-02	1.00E+00
GXYLT2	-2.70E+00	1.16E-13	LIX1L	7.96E-02	1.00E+00	LOC102407985	-8.90E-02	1.00E+00
AREL1	7.92E-01	1.18E-13	DUS2	-1.02E-01	1.00E+00	CHMP4B	4.20E-02	1.00E+00
GPR176	-3.18E+00	1.18E-13	RAB6A	-4.85E-02	1.00E+00	LOC102409778	-1.69E-01	1.00E+00
LOC112585072	-2.30E+00	1.21E-13	SCAMP3	3.98E-02	1.00E+00	LOC102390435	5.62E-02	1.00E+00
LOC102393242	2.75E+00	1.22E-13	CUX2	5.21E-02	1.00E+00	NLE1	-4.93E-02	1.00E+00
SERP2	-1.42E+00	1.25E-13	SPATA2	5.43E-02	1.00E+00	TMEM229B	-3.62E-02	1.00E+00
CNTROB	7.90E-01	1.26E-13	TNFAIP3	1.22E-01	1.00E+00	KLHL9	-4.61E-02	1.00E+00
ZNF771	-1.21E+00	1.27E-13	RAP1B	-3.89E-02	1.00E+00	SPATA20	8.98E-02	1.00E+00
PRDX3	-9.26E-01	1.27E-13	IFNAR2	-3.64E-01	1.00E+00	MTF2	-4.69E-02	1.00E+00
SMAD2	-8.36E-01	1.28E-13	MAP7D1	-9.16E-02	1.00E+00	LOC112581533	-8.47E-02	1.00E+00
FLOT1	-7.92E-01	1.30E-13	CASC4	1.08E-01	1.00E+00	RRP8	-4.64E-02	1.00E+00
SF3B2	-8.39E-01	1.30E-13	SAFB	5.09E-02	1.00E+00	ZFP41	-7.42E-02	1.00E+00
FADS6	-2.94E+00	1.31E-13	ARRDC2	-4.54E-02	1.00E+00	STBD1	4.44E-02	1.00E+00
VGLL3	-	1.31E-13	DCBLD2	6.72E-02	1.00E+00	ZDHHC17	-5.34E-	1.00E+00

	5.86E+00						02	
SYT2	1.64E+00	1.31E-13	MEIS3	1.86E-01	1.00E+00	APOM	-7.44E-02	1.00E+00
ANKZF1	-9.45E-01	1.32E-13	DIAPH3	3.81E-02	1.00E+00	HSF2	-3.93E-02	1.00E+00
ARID1B	-7.65E-01	1.33E-13	LOC102393430	-1.31E-01	1.00E+00	NCOA1	-4.89E-02	1.00E+00
SLC7A6OS	-1.01E+00	1.34E-13	SUSD2	1.48E-01	1.00E+00	LOC102402897	5.93E-02	1.00E+00
PADI4	-2.96E+00	1.35E-13	RGS14	-1.64E-01	1.00E+00	EMSY	-5.36E-02	1.00E+00
MTURN	-1.04E+00	1.35E-13	IQCA1L	-1.30E-01	1.00E+00	FYCO1	3.80E-02	1.00E+00
IQCD	-2.92E+00	1.36E-13	STT3A	-3.74E-02	1.00E+00	EPS15L1	-3.49E-02	1.00E+00
LOC102399500	2.19E+00	1.36E-13	LOC102401932	4.29E-02	1.00E+00	LOC102403497	4.60E-02	1.00E+00
BCL7A	-1.01E+00	1.37E-13	RNF19B	-3.87E-02	1.00E+00	ZNF557	5.62E-02	1.00E+00
C15H8orf34	3.12E+00	1.38E-13	NFKBIL1	7.95E-02	1.00E+00	ATP10D	-5.76E-02	1.00E+00
MED19	-9.45E-01	1.38E-13	DONSON	5.20E-02	1.00E+00	ZNF691	4.70E-02	1.00E+00
TIMM29	8.31E-01	1.38E-13	USE1	-8.48E-02	1.00E+00	CSPG5	-8.06E-02	1.00E+00
ZBTB45	-1.62E+00	1.40E-13	P4HA1	-4.88E-02	1.00E+00	PSMC3	5.74E-02	1.00E+00
ESRRB	3.43E+00	1.41E-13	LOC112580614	-1.06E-01	1.00E+00	ATP6AP1	-3.99E-02	1.00E+00
SLC23A3	-2.83E+00	1.41E-13	BCL3	9.71E-02	1.00E+00	LOC102402769	6.89E-02	1.00E+00
LOC112585578	1.34E+00	1.42E-13	LOC102391122	-6.33E-02	1.00E+00	COPS2	-3.84E-02	1.00E+00
FBL	-1.20E+00	1.43E-13	LOC112582989	-6.51E-02	1.00E+00	PPP1R36	8.59E-02	1.00E+00
REEP4	1.04E+00	1.45E-13	SH2D4B	1.66E-01	1.00E+00	MKRN2	3.80E-02	1.00E+00
GLYR1	7.30E-01	1.45E-13	LOC112577787	-1.18E-01	1.00E+00	POM121C	-4.33E-02	1.00E+00
KRT1	-3.67E+00	1.45E-13	BCL2	-7.95E-02	1.00E+00	SLC12A1	9.27E-02	1.00E+00
GOLGB1	-7.69E-01	1.45E-13	PDK1	-7.89E-02	1.00E+00	GPR3	1.34E-01	1.00E+00
STXBP5L	1.33E+00	1.47E-13	KATNAL1	4.98E-02	1.00E+00	AUTS2	5.72E-02	1.00E+00
CCDC192	2.51E+00	1.48E-13	ZNF684	-1.41E-01	1.00E+00	EEF1G	-6.75E-02	1.00E+00
GRB7	1.18E+00	1.48E-13	FBXW9	2.33E-01	1.00E+00	CYLD	4.38E-02	1.00E+00
PUS10	-1.33E+00	1.51E-13	SHC4	7.52E-02	1.00E+00	GNL2	-3.65E-02	1.00E+00
DUSP4	-4.30E+00	1.52E-13	C16H11orf58	5.18E-02	1.00E+00	CD46	4.61E-02	1.00E+00
RASSF2	-2.73E+00	1.52E-13	NEK1	-6.58E-02	1.00E+00	CCR10	-9.95E-02	1.00E+00
IGSF3	-9.10E-01	1.53E-13	LOC112587954	-6.87E-02	1.00E+00	MAD1L1	4.83E-02	1.00E+00
ATP13A1	-8.53E-01	1.53E-13	LOC112581562	1.82E-01	1.00E+00	GNL1	3.82E-02	1.00E+00
LOC102406576	-1.09E+00	1.53E-13	PLA2G12B	9.68E-02	1.00E+00	UTP18	3.69E-02	1.00E+00
ENY2	-9.07E-01	1.54E-13	EXOC5	7.14E-02	1.00E+00	FAM174A	-4.73E-02	1.00E+00
CHMP7	7.70E-01	1.54E-13	TAPT1	-7.08E-02	1.00E+00	AURKAIP1	5.85E-02	1.00E+00
CCDC120	1.02E+00	1.55E-13	KIAA1522	5.26E-02	1.00E+00	PMM1	4.55E-02	1.00E+00

TNKS2	-1.22E+00	1.55E-13	BBS7	-1.10E-01	1.00E+00	APPBP2	4.10E-02	1.00E+00
C12H2orf49	8.02E-01	1.56E-13	C2H2orf76	-5.71E-02	1.00E+00	EIF4E2	4.02E-02	1.00E+00
SYN3	1.20E+00	1.56E-13	LOC112577753	2.06E-01	1.00E+00	RTKN	-8.40E-02	1.00E+00
TRIL	-3.47E+00	1.59E-13	MARCH4	2.40E-01	1.00E+00	LOC112582363	-4.44E-02	1.00E+00
RERE	-1.01E+00	1.59E-13	SPR	8.27E-02	1.00E+00	ELP4	4.71E-02	1.00E+00
NRM	-1.56E+00	1.60E-13	BORCS5	5.11E-02	1.00E+00	TBL3	4.83E-02	1.00E+00
LOC102407546	-1.04E+00	1.63E-13	LOC102405833	-5.40E-02	1.00E+00	PHF5A	4.56E-02	1.00E+00
CACNG8	6.04E+00	1.69E-13	BTBD3	5.37E-02	1.00E+00	C3H17orf49	-8.16E-02	1.00E+00
LOC102410987	-5.21E+00	1.70E-13	FSCB	1.31E-01	1.00E+00	TBC1D8B	4.73E-02	1.00E+00
SNED1	-1.26E+00	1.70E-13	NPM3	-6.40E-02	1.00E+00	C4H12orf73	-5.19E-02	1.00E+00
LOC112579513	3.42E+00	1.70E-13	ATP6V1H	-3.79E-02	1.00E+00	CRCP	-5.14E-02	1.00E+00
WDR54	1.20E+00	1.72E-13	LRRC41	1.45E-01	1.00E+00	LOC112578241	-7.38E-02	1.00E+00
ARFGEF2	1.14E+00	1.73E-13	GPS1	-7.63E-02	1.00E+00	SLX4	-4.01E-02	1.00E+00
CRAT	-7.33E-01	1.79E-13	AAMP	-3.93E-02	1.00E+00	CUL3	3.70E-02	1.00E+00
SPON2	-3.89E+00	1.81E-13	BEST1	-1.65E-01	1.00E+00	GNG5	-4.98E-02	1.00E+00
GPNMB	-4.68E+00	1.83E-13	LOC112580259	-1.59E-01	1.00E+00	ATG4C	4.76E-02	1.00E+00
DLAT	7.95E-01	1.86E-13	GIN1	1.58E-01	1.00E+00	PKLR	-7.99E-02	1.00E+00
LOC102400086	2.48E+00	1.86E-13	PDCD6	-7.23E-02	1.00E+00	OSER1	-4.17E-02	1.00E+00
GALNT9	1.22E+00	1.87E-13	CARMIL3	1.10E-01	1.00E+00	FAM126B	5.57E-02	1.00E+00
LOC102399533	-2.40E+00	1.88E-13	LOC112584612	-1.65E-01	1.00E+00	FZD8	-5.93E-02	1.00E+00
TOMM20	-1.05E+00	1.90E-13	EIF1	-3.60E-02	1.00E+00	LOC102413141	-3.78E-02	1.00E+00
NID2	1.60E+00	1.97E-13	LOC102409300	-1.52E-01	1.00E+00	ZNF583	4.53E-02	1.00E+00
ABLIM2	-1.91E+00	1.98E-13	SLC16A3	1.12E-01	1.00E+00	ZNF384	-3.51E-02	1.00E+00
CDC42EP2	-4.30E+00	1.99E-13	LOC102409025	-1.32E-01	1.00E+00	LYSMD4	5.39E-02	1.00E+00
TEN1	-1.02E+00	2.01E-13	RAB35	3.79E-02	1.00E+00	TTC8	-5.16E-02	1.00E+00
PKIG	-1.20E+00	2.07E-13	SLC9B1	4.69E-02	1.00E+00	ISG20L2	4.02E-02	1.00E+00
ZMYND10	-3.33E+00	2.08E-13	LOC102392193	-7.16E-02	1.00E+00	PRAM1	-7.42E-02	1.00E+00
DVL1	-7.66E-01	2.09E-13	FBXO41	-1.32E-01	1.00E+00	FRY	-4.22E-02	1.00E+00
ZNF574	8.90E-01	2.09E-13	GNG7	-2.27E-01	1.00E+00	SLIRP	6.10E-02	1.00E+00
ARHGAP30	-4.09E+00	2.09E-13	GAS2	-8.85E-02	1.00E+00	UBE2B	4.11E-02	1.00E+00
LOC102414203	3.79E+00	2.10E-13	LOC102412400	1.70E-01	1.00E+00	KREMEN1	4.55E-02	1.00E+00
PLEKHF1	-2.74E+00	2.14E-13	APCDD1	1.59E-01	1.00E+00	SIAH2	-4.62E-02	1.00E+00
ASB12	-3.74E+00	2.16E-13	BVES	6.50E-02	1.00E+00	IPPK	4.40E-02	1.00E+00

NDUFC2	-1.12E+00	2.18E-13	RPS6	-1.30E-01	1.00E+00	TMEM132A	-4.85E-02	1.00E+00
KIF26A	4.43E+00	2.20E-13	MIF	-6.80E-02	1.00E+00	CYTIP	1.31E-01	1.00E+00
KAT5	-7.87E-01	2.20E-13	ACSL3	-4.82E-02	1.00E+00	DHRS11	-4.37E-02	1.00E+00
SOCS1	1.87E+00	2.23E-13	MAPK14	-3.69E-02	1.00E+00	C12H2orf68	4.32E-02	1.00E+00
NUTF2	-9.32E-01	2.25E-13	KCTD13	4.18E-02	1.00E+00	RNF187	-5.00E-02	1.00E+00
NAA15	-7.30E-01	2.25E-13	SAP30	-4.96E-02	1.00E+00	HSPB11	-5.22E-02	1.00E+00
LOC112579922	2.90E+00	2.30E-13	DSE	7.11E-02	1.00E+00	RNASEH2C	6.67E-02	1.00E+00
TMEM41B	-1.27E+00	2.31E-13	LOC102407062	-7.28E-02	1.00E+00	FOSL1	9.96E-02	1.00E+00
KIAA2026	-1.51E+00	2.32E-13	WRAP73	4.53E-02	1.00E+00	LOC112580313	-8.31E-02	1.00E+00
STEAP2	-9.94E-01	2.34E-13	EGR1	-1.17E-01	1.00E+00	UBE2W	-3.65E-02	1.00E+00
MAPK1IP1L	-3.38E+00	2.35E-13	EHD1	3.45E-02	1.00E+00	ZNF524	5.44E-02	1.00E+00
RAB3GAP2	8.47E-01	2.36E-13	BIN2	-5.85E-02	1.00E+00	STK3	-3.45E-02	1.00E+00
PAH	4.26E+00	2.36E-13	LOC102408240	4.10E-02	1.00E+00	CIZ1	-3.55E-02	1.00E+00
HM13	-7.53E-01	2.36E-13	L3MBTL1	-1.91E-01	1.00E+00	TJP3	8.55E-02	1.00E+00
ELF5	3.43E+00	2.38E-13	LOC102400130	6.82E-02	1.00E+00	TGOLN2	3.80E-02	1.00E+00
LOC112580443	-2.64E+00	2.39E-13	RNF166	4.81E-02	1.00E+00	SPIRE1	4.79E-02	1.00E+00
CCDC18	-1.04E+00	2.40E-13	ARL2BP	3.60E-02	1.00E+00	HMCES	-4.22E-02	1.00E+00
LOC112578567	-2.97E+00	2.42E-13	EPOR	-7.75E-02	1.00E+00	CCT6A	-3.44E-02	1.00E+00
POMT2	7.75E-01	2.48E-13	BACH1	-8.82E-02	1.00E+00	EDIL3	5.92E-02	1.00E+00
DBF4	7.69E-01	2.49E-13	MFSD2B	2.34E-01	1.00E+00	PPP1R8	-3.74E-02	1.00E+00
MICALL1	6.71E-01	2.51E-13	C5H1orf159	-1.12E-01	1.00E+00	PAOX	-4.47E-02	1.00E+00
PTGR2	8.02E-01	2.57E-13	ACAD9	6.49E-02	1.00E+00	SEMA4A	1.27E-01	1.00E+00
ITPR3	1.41E+00	2.57E-13	LOC112586431	2.25E-01	1.00E+00	CTU2	4.69E-02	1.00E+00
LOC112586232	-4.23E+00	2.58E-13	DEK	-7.44E-02	1.00E+00	NCOA5	-3.47E-02	1.00E+00
DNAH12	-2.07E+00	2.61E-13	FHL5	1.16E-01	1.00E+00	KAT5	-3.87E-02	1.00E+00
LRRN4CL	-9.76E-01	2.64E-13	LOC102407057	4.59E-02	1.00E+00	ALG14	-4.30E-02	1.00E+00
SPIB	1.76E+00	2.72E-13	SELENOK	-4.84E-02	1.00E+00	PRKAB2	-4.55E-02	1.00E+00
CISD2	7.82E-01	2.74E-13	LOC112585766	1.54E-01	1.00E+00	ZBTB45	-6.63E-02	1.00E+00
NIPA1	-1.63E+00	2.76E-13	LOC102405658	-1.09E-01	1.00E+00	VBP1	-3.89E-02	1.00E+00
NSUN5	-1.09E+00	2.80E-13	POLR1E	-3.81E-02	1.00E+00	LOC112580666	-4.53E-02	1.00E+00
XPO4	-7.81E-01	2.81E-13	PIGL	1.66E-01	1.00E+00	STARD5	6.44E-02	1.00E+00
SLC38A6	-1.53E+00	2.81E-13	ARFGAP1	-4.58E-02	1.00E+00	SRSF4	-3.61E-02	1.00E+00
SPATA13	-3.10E+00	2.81E-13	RPL7A	-4.48E-02	1.00E+00	NCK2	3.64E-02	1.00E+00
LOC112587361	2.37E+00	2.89E-13	PAAF1	-8.63E-	1.00E+00	FOXK1	-3.95E-	1.00E+00

				02			02	
GALK1	-1.28E+00	2.97E-13	CCDC13	-1.93E-01	1.00E+00	DKC1	-4.11E-02	1.00E+00
LOC102410803	1.27E+00	2.99E-13	CCDC148	-8.17E-02	1.00E+00	LOC102395641	4.77E-02	1.00E+00
FXYD2	-2.99E+00	2.99E-13	DNAJC5	-3.46E-02	1.00E+00	LOC102414936	-9.31E-02	1.00E+00
LOC112586884	2.96E+00	3.00E-13	LOC112584679	-1.45E-01	1.00E+00	PHF20L1	-4.75E-02	1.00E+00
KCTD18	-1.30E+00	3.04E-13	ETS1	8.22E-02	1.00E+00	TOMM20L	-6.25E-02	1.00E+00
UBXN7	7.47E-01	3.06E-13	SELENOH	-7.02E-02	1.00E+00	LOC102393336	-7.81E-02	1.00E+00
ORMDL3	8.37E-01	3.07E-13	SNX8	-6.36E-02	1.00E+00	PPM1H	5.06E-02	1.00E+00
MYL2	3.13E+00	3.07E-13	RTL6	1.10E-01	1.00E+00	SND1	-3.57E-02	1.00E+00
ARX	2.85E+00	3.08E-13	TXNRD2	8.28E-02	1.00E+00	LOC102390617	6.06E-02	1.00E+00
CS	1.86E+00	3.11E-13	RHPN2	8.97E-02	1.00E+00	RERE	-4.49E-02	1.00E+00
CMPK1	6.66E-01	3.15E-13	PLOD1	2.90E-01	1.00E+00	SPC24	5.82E-02	1.00E+00
LOC112587820	-1.27E+00	3.16E-13	PTPRA	4.28E-02	1.00E+00	DDB1	-3.76E-02	1.00E+00
GABRG3	3.35E+00	3.17E-13	ATP2B1	5.16E-02	1.00E+00	POLR2G	5.21E-02	1.00E+00
C11H15orf48	2.17E+00	3.18E-13	P2RX1	2.39E-01	1.00E+00	RSL24D1	3.72E-02	1.00E+00
PMP22	2.61E+00	3.20E-13	NTN4	-2.49E-01	1.00E+00	RMC1	4.58E-02	1.00E+00
CEP112	-8.93E-01	3.27E-13	ZNHIT6	4.10E-02	1.00E+00	STK4	4.36E-02	1.00E+00
TRIM66	1.48E+00	3.33E-13	SRSF6	-6.42E-02	1.00E+00	ITM2C	4.53E-02	1.00E+00
RCC1	-8.37E-01	3.35E-13	NETO2	3.80E-02	1.00E+00	RPS26	-6.99E-02	1.00E+00
LOC112586445	1.71E+00	3.42E-13	DIAPH1	5.24E-02	1.00E+00	ETFB	5.89E-02	1.00E+00
ZNF660	-1.84E+00	3.43E-13	BBS1	1.40E-01	1.00E+00	PSMC3IP	-5.82E-02	1.00E+00
LOC102394014	1.89E+00	3.44E-13	KDM5A	-6.10E-02	1.00E+00	WDR5	-3.84E-02	1.00E+00
KIF22	1.16E+00	3.44E-13	KPNB1	6.48E-02	1.00E+00	SMURF2	4.13E-02	1.00E+00
TRMT1L	6.87E-01	3.46E-13	PLEKHG7	-3.67E-02	1.00E+00	GON7	-4.72E-02	1.00E+00
NOL10	6.45E-01	3.47E-13	AGMO	-1.30E-01	1.00E+00	LOC102411003	-3.76E-02	1.00E+00
LOC102414992	-4.83E+00	3.49E-13	MTCH1	4.25E-02	1.00E+00	SIPA1	-5.49E-02	1.00E+00
C15H8orf88	-1.32E+00	3.52E-13	WSB2	3.79E-02	1.00E+00	EIF3F	-4.64E-02	1.00E+00
SFMBT1	8.14E-01	3.64E-13	MED8	-5.55E-02	1.00E+00	ALG5	-5.01E-02	1.00E+00
ECHS1	-1.00E+00	3.65E-13	REV1	4.97E-02	1.00E+00	ZNF638	3.76E-02	1.00E+00
KLF8	-3.60E+00	3.73E-13	RRP15	5.10E-02	1.00E+00	MAN2B1	-5.33E-02	1.00E+00
LOC102406013	4.24E+00	3.75E-13	LOC112578687	-1.29E-01	1.00E+00	LOC102416013	3.91E-02	1.00E+00
NKX2-5	4.63E+00	3.76E-13	RNMT	-4.48E-02	1.00E+00	RPF2	-3.75E-02	1.00E+00
WFIKKN1	1.67E+00	3.77E-13	EGFR	-8.79E-02	1.00E+00	PPP1R13B	-5.27E-02	1.00E+00
LOC112579993	-8.26E+00	3.77E-13	APEH	-9.56E-02	1.00E+00	C16H11orf71	-6.27E-02	1.00E+00
LOC102410181	1.69E+00	3.83E-13	APLF	-3.69E-	1.00E+00	KCTD21	6.20E-02	1.00E+00

				02				
LOC112579607	-1.05E+00	3.87E-13	C3H9orf152	-9.74E-02	1.00E+00	SPCS2	-4.04E-02	1.00E+00
LOC112580131	3.38E+00	3.88E-13	SRD5A3	-5.74E-02	1.00E+00	RALA	3.47E-02	1.00E+00
NRAP	2.43E+00	3.89E-13	TMEM109	3.72E-02	1.00E+00	QKI	4.76E-02	1.00E+00
LOC102413515	-3.30E+00	3.90E-13	RABL3	-4.03E-02	1.00E+00	CHCHD1	5.52E-02	1.00E+00
B3GNT9	-1.44E+00	3.93E-13	SUV39H2	-3.63E-02	1.00E+00	POLDIP2	-4.29E-02	1.00E+00
CARD9	-2.90E+00	4.16E-13	LOC102410939	-5.72E-02	1.00E+00	SMG8	4.74E-02	1.00E+00
UFL1	7.97E-01	4.18E-13	SEC61A2	3.89E-02	1.00E+00	RPA2	4.04E-02	1.00E+00
CFI	-3.70E+00	4.20E-13	C6H1orf194	7.24E-02	1.00E+00	CACNA2D3	-8.66E-02	1.00E+00
LOC102411682	5.10E+00	4.24E-13	IFT74	3.75E-02	1.00E+00	JMJD6	3.96E-02	1.00E+00
C6H1orf87	3.96E+00	4.25E-13	LRP5	-1.94E-01	1.00E+00	MINOS1	-5.91E-02	1.00E+00
COL11A1	-4.01E+00	4.31E-13	LOC112580616	-2.34E-01	1.00E+00	NEDD9	4.40E-02	1.00E+00
LOC102410885	-1.89E+00	4.32E-13	LOC112585749	-5.34E-02	1.00E+00	AHSG	8.67E-02	1.00E+00
LOC112580428	2.21E+00	4.32E-13	IDE	-5.66E-02	1.00E+00	LOC102391011	-5.25E-02	1.00E+00
PITX1	-1.68E+00	4.36E-13	ATP4A	1.73E-01	1.00E+00	EXO1	-5.00E-02	1.00E+00
SNX29	8.69E-01	4.38E-13	PNISR	-4.92E-02	1.00E+00	FSTL1	-3.96E-02	1.00E+00
THAP6	1.22E+00	4.42E-13	FOXL2	2.68E-01	1.00E+00	ERBB3	-7.68E-02	1.00E+00
HTR2A	3.27E+00	4.45E-13	TOX2	-2.35E-01	1.00E+00	MVB12A	4.80E-02	1.00E+00
DZIP3	-9.67E-01	4.50E-13	SLC35C2	-6.19E-02	1.00E+00	EPS15	5.15E-02	1.00E+00
DBT	-1.51E+00	4.52E-13	DBX1	5.25E-02	1.00E+00	ERCC6L	-4.70E-02	1.00E+00
ZNF697	-1.67E+00	4.54E-13	TMX1	5.27E-02	1.00E+00	FBXW9	5.81E-02	1.00E+00
LOC102391387	2.67E+00	4.54E-13	LOC102408234	-1.57E-01	1.00E+00	NDFIP1	-3.47E-02	1.00E+00
CEPT1	6.73E-01	4.54E-13	LOC112578881	2.12E-01	1.00E+00	LRRC9	7.26E-02	1.00E+00
SMARCE1	-7.27E-01	4.61E-13	DMAC1	-1.04E-01	1.00E+00	CDKN1C	6.47E-02	1.00E+00
NDUFA5	-1.02E+00	4.73E-13	PSMC1	-3.69E-02	1.00E+00	SBDS	3.59E-02	1.00E+00
RABL6	-8.42E-01	4.81E-13	HHIPL2	1.57E-01	1.00E+00	TMEM35A	8.14E-02	1.00E+00
VAMP3	7.31E-01	4.81E-13	MYH6	5.93E-02	1.00E+00	PRRG2	7.02E-02	1.00E+00
LOC112586135	-3.01E+00	4.91E-13	DGAT1	-6.09E-02	1.00E+00	HYAL1	-4.98E-02	1.00E+00
SEC31A	-7.56E-01	4.93E-13	POLR3K	-4.48E-02	1.00E+00	ATPAF2	-5.10E-02	1.00E+00
ATXN2	-8.73E-01	4.94E-13	BAIAP2L1	3.88E-02	1.00E+00	FMR1	-5.03E-02	1.00E+00
EBP	-1.42E+00	4.96E-13	C17H12orf49	-4.00E-02	1.00E+00	PRNP	6.36E-02	1.00E+00
LDAH	-8.82E-01	4.96E-13	MFSD13A	3.66E-02	1.00E+00	ADAM12	6.87E-02	1.00E+00
NAV2	1.18E+00	4.99E-13	HAGHL	-6.86E-02	1.00E+00	LOC112580427	4.12E-02	1.00E+00
DNAH8	3.82E+00	5.00E-13	LOC112579927	-4.12E-02	1.00E+00	RBM7	5.18E-02	1.00E+00

TBCEL	8.15E-01	5.05E-13	NAIF1	-1.03E-01	1.00E+00	PIGM	-6.76E-02	1.00E+00
LOC102411450	2.76E+00	5.13E-13	B3GNT4	6.05E-02	1.00E+00	SPAST	-6.65E-02	1.00E+00
GFRA3	1.48E+00	5.15E-13	NUB1	-4.53E-02	1.00E+00	PSEN2	-4.23E-02	1.00E+00
LMOD2	-6.35E+00	5.16E-13	HAGH	-4.21E-02	1.00E+00	LOC102407011	-7.98E-02	1.00E+00
CDC42SE1	7.80E-01	5.20E-13	TAP2	1.89E-01	1.00E+00	CUTA	-4.50E-02	1.00E+00
ZC3H12A	-2.10E+00	5.23E-13	DHRSX	9.68E-02	1.00E+00	LOC112581898	6.87E-02	1.00E+00
ENPP4	-1.99E+00	5.25E-13	EFL1	-4.55E-02	1.00E+00	SRP72	-3.45E-02	1.00E+00
MERTK	-1.48E+00	5.28E-13	SCLY	6.83E-02	1.00E+00	ERCC4	4.78E-02	1.00E+00
FRAS1	1.84E+00	5.30E-13	CFAP99	2.65E-01	1.00E+00	IQCK	5.34E-02	1.00E+00
EIF1AX	-1.13E+00	5.40E-13	C4H12orf75	5.63E-02	1.00E+00	ANKRD11	4.06E-02	1.00E+00
RCL1	9.09E-01	5.44E-13	DLST	3.68E-02	1.00E+00	DDB2	3.72E-02	1.00E+00
THUMPD1	7.22E-01	5.49E-13	NDUFAF1	-8.26E-02	1.00E+00	DHX40	-4.24E-02	1.00E+00
CA14	-1.75E+00	5.65E-13	PRKRIP1	-3.70E-02	1.00E+00	SRPRB	-3.48E-02	1.00E+00
POLE4	-1.13E+00	5.73E-13	PTK2	4.10E-02	1.00E+00	TMEM88	6.99E-02	1.00E+00
NPW	2.72E+00	5.74E-13	DUSP3	4.89E-02	1.00E+00	FAM180B	7.90E-02	1.00E+00
C1QA	-5.74E+00	5.79E-13	ORMDL3	-4.00E-02	1.00E+00	MCEE	5.59E-02	1.00E+00
LOC112579661	3.47E+00	5.87E-13	ELP3	-5.49E-02	1.00E+00	SNRPG	-4.95E-02	1.00E+00
DNAJC19	-1.09E+00	6.19E-13	NFYA	-1.75E-01	1.00E+00	TXNDC15	3.89E-02	1.00E+00
LOC102412400	4.92E+00	6.24E-13	KIAA1143	-3.85E-02	1.00E+00	C4H12orf66	8.43E-02	1.00E+00
TMTC3	-1.13E+00	6.24E-13	CD40LG	2.05E-01	1.00E+00	DNAJA2	-3.56E-02	1.00E+00
KIAA0040	1.73E+00	6.25E-13	CAPZB	3.51E-02	1.00E+00	LOC112584667	1.07E-01	1.00E+00
TPP2	8.79E-01	6.37E-13	NUDT1	9.10E-02	1.00E+00	ERCC6L2	-4.12E-02	1.00E+00
ALG8	8.13E-01	6.40E-13	MACF1	7.15E-02	1.00E+00	LOC102416239	9.70E-02	1.00E+00
GALNT1	-6.75E-01	6.47E-13	LOC112581611	8.51E-02	1.00E+00	IFT74	3.86E-02	1.00E+00
BCDIN3D	-1.34E+00	6.60E-13	TXNDC11	3.45E-02	1.00E+00	JUP	-3.77E-02	1.00E+00
ACER3	8.35E-01	6.65E-13	ARFGAP2	3.95E-02	1.00E+00	LOC102402877	-4.87E-02	1.00E+00
GUCA1A	-3.01E+00	6.67E-13	SLC16A5	5.90E-02	1.00E+00	WDR43	-3.45E-02	1.00E+00
ZSCAN29	6.58E-01	6.70E-13	LOC102405118	1.33E-01	1.00E+00	RHBDF1	4.20E-02	1.00E+00
C1RL	-4.42E+00	6.70E-13	TBC1D30	5.33E-02	1.00E+00	RINT1	-4.05E-02	1.00E+00
SLU7	8.86E-01	6.76E-13	GFRA3	9.25E-02	1.00E+00	FGD3	1.55E-01	1.00E+00
BCL2L11	-1.34E+00	6.77E-13	CCT5	-3.66E-02	1.00E+00	IP6K1	3.50E-02	1.00E+00
UBOX5	8.10E-01	6.85E-13	HES4	-1.14E-01	1.00E+00	UHRF2	4.02E-02	1.00E+00
CASQ2	3.14E+00	6.85E-13	POLQ	6.89E-02	1.00E+00	UCHL3	4.72E-02	1.00E+00
WDR41	-6.26E-01	7.02E-13	CRTAM	-1.49E-01	1.00E+00	C16H11orf74	-5.17E-02	1.00E+00
LOC102389530	-2.04E+00	7.06E-13	NAT10	4.34E-02	1.00E+00	PSMD11	3.41E-02	1.00E+00

PCSK6	-1.37E+00	7.13E-13	FYB2	6.59E-02	1.00E+00	ARHGAP21	4.51E-02	1.00E+00
ATP23	-1.16E+00	7.40E-13	ITGB4	6.28E-02	1.00E+00	MPG	5.14E-02	1.00E+00
MED20	7.35E-01	7.40E-13	LOC112587923	-1.14E-01	1.00E+00	LOC102406090	-6.92E-02	1.00E+00
ERN2	2.21E+00	7.46E-13	RNF144A	-2.94E-01	1.00E+00	LOC102404976	6.74E-02	1.00E+00
FARSA	-7.37E-01	7.65E-13	RSRC1	-6.36E-02	1.00E+00	KIAA0408	8.72E-02	1.00E+00
FN1	-1.83E+00	7.67E-13	COMTD1	-1.56E-01	1.00E+00	LOC102410470	5.21E-02	1.00E+00
TNRC6B	9.17E-01	7.70E-13	ARHGEF40	-5.51E-02	1.00E+00	LOC102393778	5.81E-02	1.00E+00
ZNF397	1.04E+00	7.77E-13	AJUBA	1.35E-01	1.00E+00	HNRNPA3	-4.21E-02	1.00E+00
LOC102416255	-6.14E+00	7.80E-13	THUMPD2	-8.78E-02	1.00E+00	SLC25A11	3.99E-02	1.00E+00
SLC50A1	-1.43E+00	7.89E-13	ABAT	4.47E-02	1.00E+00	SETD2	-3.98E-02	1.00E+00
LOC112579144	-3.00E+00	7.92E-13	KHDRBS3	4.70E-02	1.00E+00	FKBP14	3.78E-02	1.00E+00
LOC102413900	-3.64E+00	8.02E-13	SCAI	-9.36E-02	1.00E+00	CNBP	-4.44E-02	1.00E+00
SFRP2	-3.07E+00	8.05E-13	ZCCHC3	5.06E-02	1.00E+00	LCOR	-4.09E-02	1.00E+00
RASD1	-1.93E+00	8.17E-13	TRIOBP	3.69E-02	1.00E+00	CLPTM1L	-3.78E-02	1.00E+00
MOCOS	1.61E+00	8.54E-13	FOXJ3	5.19E-02	1.00E+00	MED1	5.21E-02	1.00E+00
SCAMP1	9.00E-01	8.55E-13	FAM219B	-9.43E-02	1.00E+00	ICE2	-5.09E-02	1.00E+00
CNTNAP5	-3.16E+00	8.74E-13	APLP1	-4.52E-02	1.00E+00	TMOD4	-7.48E-02	1.00E+00
LOC112580299	4.11E+00	8.78E-13	MYEF2	-1.28E-01	1.00E+00	NUDT3	-3.24E-02	1.00E+00
RSPO4	-4.01E+00	8.79E-13	EIF2AK4	-4.31E-02	1.00E+00	SPCS1	4.18E-02	1.00E+00
CORO1A	-2.86E+00	8.94E-13	PSMD1	-3.39E-02	1.00E+00	CARD9	-1.00E-01	1.00E+00
DOK6	3.08E+00	8.94E-13	PTPRF	1.44E-01	1.00E+00	ARHGEF25	5.10E-02	1.00E+00
PKD2L1	-2.28E+00	8.97E-13	SLC35E3	4.99E-02	1.00E+00	KBTBD3	5.38E-02	1.00E+00
SLC41A2	9.29E-01	9.03E-13	LRPPRC	-5.60E-02	1.00E+00	HTATSF1	3.73E-02	1.00E+00
TIPARP	-1.56E+00	9.04E-13	LOC112587902	-6.32E-02	1.00E+00	SMARCA4	-3.62E-02	1.00E+00
SHISA5	-8.53E-01	9.06E-13	CNOT8	5.14E-02	1.00E+00	GNE	-4.62E-02	1.00E+00
LOC102414436	2.97E+00	9.15E-13	RFX7	1.04E-01	1.00E+00	TCIRG1	5.90E-02	1.00E+00
TRMT2A	-1.02E+00	9.20E-13	PPP6R3	-5.03E-02	1.00E+00	XRN2	-3.89E-02	1.00E+00
ANKLE1	-3.91E+00	9.32E-13	CAP1	3.51E-02	1.00E+00	CHID1	4.62E-02	1.00E+00
CHCHD6	1.58E+00	9.51E-13	NBEA	8.20E-02	1.00E+00	YWHAB	3.93E-02	1.00E+00
LAMC2	-1.72E+00	9.58E-13	UTP11	-4.74E-02	1.00E+00	IL17RD	-4.76E-02	1.00E+00
ATP5PB	-8.38E-01	9.59E-13	ATF7IP2	-4.54E-02	1.00E+00	RBM6	3.30E-02	1.00E+00
LOC102393672	6.69E-01	9.65E-13	POP5	-1.40E-01	1.00E+00	NEFL	1.29E-01	1.00E+00
BIN2	3.49E+00	9.70E-13	ZNHIT3	-6.43E-02	1.00E+00	TMEM243	4.64E-02	1.00E+00
SCN1B	-	9.80E-13	OSBPL7	-9.32E-	1.00E+00	TRIM33	-4.21E-	1.00E+00

	4.13E+00			02			02	
CNEP1R1	9.03E-01	9.84E-13	PSMG3	-6.13E-02	1.00E+00	SPTLC1	-3.92E-02	1.00E+00
LOC102406005	4.25E+00	9.88E-13	UHRF2	4.51E-02	1.00E+00	LOC102393839	-4.04E-02	1.00E+00
GPATCH11	-8.36E-01	9.94E-13	MLC1	2.26E-01	1.00E+00	LOC112583698	-1.04E-01	1.00E+00
LOC112586811	4.02E+00	1.00E-12	NRIP1	-1.31E-01	1.00E+00	LAP3	-3.61E-02	1.00E+00
CASP9	-1.35E+00	1.02E-12	PLEKHM2	4.05E-02	1.00E+00	PTBP3	3.74E-02	1.00E+00
TIMM10B	-9.63E-01	1.03E-12	CNGA3	5.52E-02	1.00E+00	AP3M1	3.84E-02	1.00E+00
CHRNB1	-1.72E+00	1.04E-12	LRBA	5.14E-02	1.00E+00	NFE2L3	4.32E-02	1.00E+00
NDUFAF7	8.05E-01	1.05E-12	ZNF292	1.04E-01	1.00E+00	LIPT1	-7.19E-02	1.00E+00
IHH	-4.82E+00	1.05E-12	LZTR1	9.49E-02	1.00E+00	MRPS34	5.62E-02	1.00E+00
PIH1D1	-1.14E+00	1.07E-12	PHKB	-4.81E-02	1.00E+00	HDAC2	3.63E-02	1.00E+00
LOC112584743	2.91E+00	1.08E-12	GNB1	3.62E-02	1.00E+00	DDX39B	-4.32E-02	1.00E+00
LOC102402326	-5.15E+00	1.08E-12	EPHB1	-7.42E-02	1.00E+00	NOTCH4	-6.95E-02	1.00E+00
ACSM1	3.69E+00	1.09E-12	GNG12	3.82E-02	1.00E+00	C1H4orf47	-4.86E-02	1.00E+00
CEBPZOS	-1.12E+00	1.11E-12	ROCK2	-1.26E-01	1.00E+00	PLEKHA5	4.31E-02	1.00E+00
ARMH1	-1.84E+00	1.12E-12	C24H16orf91	-6.57E-02	1.00E+00	IPO13	-3.27E-02	1.00E+00
KIAA1257	-1.12E+00	1.12E-12	HGD	1.14E-01	1.00E+00	METTL18	5.45E-02	1.00E+00
RNF212B	2.33E+00	1.14E-12	SMYD3	-5.12E-02	1.00E+00	SEMA4F	-3.63E-02	1.00E+00
LOC102404841	-4.25E+00	1.15E-12	LOC102390086	1.57E-01	1.00E+00	TEFM	5.99E-02	1.00E+00
CAMSAP3	9.48E-01	1.15E-12	RAF1	3.55E-02	1.00E+00	GSTK1	8.92E-02	1.00E+00
JOSD1	-8.28E-01	1.16E-12	SP4	-9.11E-02	1.00E+00	RAN	4.73E-02	1.00E+00
NDUFB4	-1.03E+00	1.16E-12	STIM1	5.13E-02	1.00E+00	LOC112585751	-9.40E-02	1.00E+00
CC2D2B	-3.54E+00	1.17E-12	LOC102408090	6.36E-02	1.00E+00	ERF	-5.60E-02	1.00E+00
IFT57	-8.21E-01	1.17E-12	LOC112578850	-1.58E-01	1.00E+00	GXYLT1	3.96E-02	1.00E+00
SMARCA4	-7.68E-01	1.17E-12	GAS2L3	-9.21E-02	1.00E+00	ANKRD50	5.13E-02	1.00E+00
COL7A1	-2.01E+00	1.17E-12	TMX4	4.62E-02	1.00E+00	FCGRT	-3.27E-02	1.00E+00
GGA3	-8.25E-01	1.22E-12	ZDHHC13	3.67E-02	1.00E+00	NUMB	-5.78E-02	1.00E+00
LOC112584513	3.90E+00	1.22E-12	LOC102407446	2.04E-01	1.00E+00	EZH2	-3.51E-02	1.00E+00
DUS4L	-1.16E+00	1.23E-12	RABGGTA	-1.33E-01	1.00E+00	NT5DC2	-4.34E-02	1.00E+00
C1GALT1	-1.10E+00	1.25E-12	GORASP2	3.17E-02	1.00E+00	NKD2	-8.64E-02	1.00E+00
LOC102400351	-4.81E+00	1.26E-12	RPAP3	-4.76E-02	1.00E+00	ADAMTSL4	5.47E-02	1.00E+00
SIVA1	1.40E+00	1.26E-12	RSU1	-4.02E-02	1.00E+00	EDEM3	-4.54E-02	1.00E+00
UGDH	-1.00E+00	1.27E-12	TKTL1	-4.74E-02	1.00E+00	CHP1	3.73E-02	1.00E+00

CLN5	-9.91E-01	1.29E-12	CDC5L	4.57E-02	1.00E+00	SEC14L1	-5.08E-02	1.00E+00
ADAMTS15	-2.32E+00	1.29E-12	TAB3	5.38E-02	1.00E+00	MSL1	-3.48E-02	1.00E+00
SUMO1	7.87E-01	1.30E-12	BCO2	2.74E-01	1.00E+00	RALGAPA1	4.89E-02	1.00E+00
LOC102408072	3.07E+00	1.30E-12	GDNF	-9.41E-02	1.00E+00	ASDURF	-7.69E-02	1.00E+00
SUPT16H	-8.62E-01	1.30E-12	AGFG1	3.53E-02	1.00E+00	ARL1	3.33E-02	1.00E+00
ZYG11B	-9.94E-01	1.33E-12	CBS	4.92E-02	1.00E+00	RNF7	3.73E-02	1.00E+00
RAF1	6.55E-01	1.35E-12	SLC2A3	-2.19E-01	1.00E+00	TPST1	-4.02E-02	1.00E+00
MAU2	7.57E-01	1.36E-12	SUCLA2	4.05E-02	1.00E+00	LOC112584574	6.63E-02	1.00E+00
BOLA3	-1.07E+00	1.38E-12	MPI	8.07E-02	1.00E+00	THG1L	3.90E-02	1.00E+00
KCNJ11	-2.73E+00	1.38E-12	MRPL23	-6.52E-02	1.00E+00	LOC102410356	5.01E-02	1.00E+00
LOC112578482	1.49E+00	1.40E-12	INPPL1	5.10E-02	1.00E+00	PCGF6	6.78E-02	1.00E+00
ZNF37A	1.31E+00	1.41E-12	KDM3B	-1.18E-01	1.00E+00	CABP1	-7.02E-02	1.00E+00
NBEA	-1.27E+00	1.41E-12	ZNF613	8.14E-02	1.00E+00	ATP5PO	5.85E-02	1.00E+00
KRT5	5.74E+00	1.42E-12	PHF10	5.93E-02	1.00E+00	PAFAH1B1	3.41E-02	1.00E+00
GNB4	-1.76E+00	1.45E-12	IPO9	4.38E-02	1.00E+00	UBXN6	-4.62E-02	1.00E+00
LOC112586458	2.26E+00	1.45E-12	ADORA2B	-4.09E-02	1.00E+00	STUB1	4.92E-02	1.00E+00
CFAP97D1	3.33E+00	1.48E-12	TMEM45B	-1.82E-01	1.00E+00	USP53	-5.10E-02	1.00E+00
LOC102411680	5.10E+00	1.49E-12	PFN1	4.38E-02	1.00E+00	TRIO	4.72E-02	1.00E+00
FAM169B	1.53E+00	1.50E-12	POLR1B	-4.58E-02	1.00E+00	LOC112581200	8.89E-02	1.00E+00
LOC102400360	2.59E+00	1.50E-12	SH2D1B	-1.77E-01	1.00E+00	NFKBID	-4.43E-02	1.00E+00
TADA2B	-1.13E+00	1.51E-12	TTC23L	1.29E-01	1.00E+00	PATL1	-3.41E-02	1.00E+00
NEFL	2.70E+00	1.52E-12	PSMD7	6.63E-02	1.00E+00	RAB11A	3.54E-02	1.00E+00
LOC112580392	2.73E+00	1.53E-12	TMEM104	6.79E-02	1.00E+00	OGT	6.38E-02	1.00E+00
LOC102414406	4.01E+00	1.53E-12	CNTD1	1.19E-01	1.00E+00	MRPS30	4.07E-02	1.00E+00
PPP1R9B	-8.84E-01	1.54E-12	FBXO4	-7.10E-02	1.00E+00	LRTM2	-8.67E-02	1.00E+00
LOC112587102	4.15E+00	1.55E-12	DYNC2LI1	-5.61E-02	1.00E+00	CSMD1	-5.45E-02	1.00E+00
ECI2	9.99E-01	1.55E-12	CCDC92	4.87E-02	1.00E+00	TMEM68	4.50E-02	1.00E+00
MRPL20	1.03E+00	1.56E-12	BCL10	-3.83E-02	1.00E+00	MSR1	1.58E-01	1.00E+00
GLRA4	4.65E+00	1.58E-12	ARHGEF6	2.31E-01	1.00E+00	MEA1	-6.88E-02	1.00E+00
STOX2	-3.29E+00	1.58E-12	ARNTL2	1.57E-01	1.00E+00	PKIB	1.11E-01	1.00E+00
MROH8	2.52E+00	1.59E-12	RAB37	7.80E-02	1.00E+00	LOC102401361	5.60E-02	1.00E+00
LSM14A	-7.33E-01	1.61E-12	LOC112577778	-1.64E-01	1.00E+00	KIF3A	-4.46E-02	1.00E+00
CCDC81	2.98E+00	1.62E-12	LOC112587010	-1.87E-01	1.00E+00	MAP3K7	-3.91E-02	1.00E+00
SQLE	-6.68E-01	1.68E-12	ABCA7	1.49E-01	1.00E+00	PRDX2	5.04E-02	1.00E+00
THPO	-2.36E+00	1.71E-12	PNMA5	2.05E-01	1.00E+00	LOC102398191	-9.31E-02	1.00E+00
PARP11	-8.96E-	1.72E-12	DVL2	4.33E-02	1.00E+00	RNF25	-3.93E-	1.00E+00

	01						02	
GOSR1	6.62E-01	1.73E-12	LOC102401766	1.07E-01	1.00E+00	PLXNB2	-3.86E-02	1.00E+00
KCNS3	3.83E+00	1.75E-12	SARAF	5.52E-02	1.00E+00	ABCG1	1.05E-01	1.00E+00
CDCA3	1.24E+00	1.76E-12	PCCA	-4.56E-02	1.00E+00	NCLN	-3.80E-02	1.00E+00
TMEM234	-1.35E+00	1.76E-12	SENP8	3.72E-02	1.00E+00	ARHGAP4	-1.18E-01	1.00E+00
COPS9	-1.24E+00	1.76E-12	PIGV	4.58E-02	1.00E+00	LOC112583996	8.23E-02	1.00E+00
KIF1B	-9.47E-01	1.77E-12	THEMIS2	2.01E-01	1.00E+00	DRAM2	-3.48E-02	1.00E+00
MRPS26	-1.43E+00	1.80E-12	DLK2	7.44E-02	1.00E+00	ARAF	-3.42E-02	1.00E+00
ANKRD9	-1.24E+00	1.80E-12	RNF183	-1.19E-01	1.00E+00	FAAP24	5.81E-02	1.00E+00
CCDC159	-2.39E+00	1.80E-12	ZBTB48	5.48E-02	1.00E+00	ZNF10	7.23E-02	1.00E+00
PRPS2	-8.87E-01	1.81E-12	FAM83C	1.41E-01	1.00E+00	SCG3	-9.40E-02	1.00E+00
PDE2A	2.05E+00	1.81E-12	EGF	-9.70E-02	1.00E+00	ZBED3	4.59E-02	1.00E+00
SIPA1L1	6.56E-01	1.84E-12	IST1	-3.53E-02	1.00E+00	MRPL22	4.06E-02	1.00E+00
IRS4	3.50E+00	1.84E-12	WASHC5	4.13E-02	1.00E+00	DIAPH1	-3.45E-02	1.00E+00
CXADR	-1.17E+00	1.86E-12	GSG1	-6.83E-02	1.00E+00	TAF11	3.85E-02	1.00E+00
LCA5L	-1.13E+00	1.87E-12	RFXANK	5.20E-02	1.00E+00	LOC112584611	6.39E-02	1.00E+00
PAFAH1B3	-1.15E+00	1.88E-12	MANEA	-4.56E-02	1.00E+00	CAPN15	-4.22E-02	1.00E+00
PACRGL	-9.27E-01	1.89E-12	TRIP6	2.48E-01	1.00E+00	SORBS2	-4.09E-02	1.00E+00
LOC102397179	-2.51E+00	1.89E-12	MRPS21	-4.56E-02	1.00E+00	SMAD3	-5.26E-02	1.00E+00
LOC112578174	3.09E+00	1.91E-12	MPP3	1.11E-01	1.00E+00	AVPI1	5.03E-02	1.00E+00
PRKAR2A	-6.80E-01	1.91E-12	KIF1B	-5.52E-02	1.00E+00	KLHL22	-4.05E-02	1.00E+00
LOC102399526	-2.24E+00	1.95E-12	EMX1	2.28E-01	1.00E+00	C23H10orf143	4.77E-02	1.00E+00
LOC102406087	2.54E+00	1.96E-12	LOC102394731	-1.42E-01	1.00E+00	ADPRH	4.29E-02	1.00E+00
CD2AP	-1.11E+00	1.98E-12	ACTR3B	5.82E-02	1.00E+00	CEBPZ	-3.31E-02	1.00E+00
LOC102406311	1.89E+00	2.02E-12	ACADSB	4.52E-02	1.00E+00	SMARCD1	-3.14E-02	1.00E+00
LOC102396318	-1.23E+00	2.04E-12	LOC102391418	-6.07E-02	1.00E+00	CUL5	-3.95E-02	1.00E+00
IMMP1L	7.61E-01	2.07E-12	DNAJC3	3.42E-02	1.00E+00	LOC112586254	-4.53E-02	1.00E+00
ZNF19	1.14E+00	2.10E-12	NCOA3	-7.31E-02	1.00E+00	SLC2A13	4.43E-02	1.00E+00
CFL1	-1.05E+00	2.12E-12	SCAMP2	3.24E-02	1.00E+00	LETM1	3.47E-02	1.00E+00
NOCT	1.00E+00	2.14E-12	PRDX1	4.41E-02	1.00E+00	SPIN1	-3.48E-02	1.00E+00
CHRNE	1.15E+00	2.15E-12	JADE3	-3.72E-02	1.00E+00	RPL4	-7.70E-02	1.00E+00
BRD3OS	-1.21E+00	2.19E-12	ZNF511	-6.33E-02	1.00E+00	LOC112580153	-6.04E-02	1.00E+00
MYBPC3	-3.08E+00	2.19E-12	MATN2	1.47E-01	1.00E+00	SLC35A5	-4.34E-02	1.00E+00

SYBU	2.01E+00	2.22E-12	LOC102406878	1.64E-01	1.00E+00	GDPD1	-3.89E-02	1.00E+00
SIM2	3.64E+00	2.26E-12	PGRMC2	4.83E-02	1.00E+00	PIGN	3.62E-02	1.00E+00
PEG3	-1.14E+00	2.26E-12	MPC1L	1.34E-01	1.00E+00	NTPCR	-3.81E-02	1.00E+00
CNBP	-8.84E-01	2.31E-12	FEN1	4.34E-02	1.00E+00	NRIP1	-4.42E-02	1.00E+00
GRIN2D	-3.26E+00	2.31E-12	SPIB	-1.27E-01	1.00E+00	MRPL20	4.59E-02	1.00E+00
NPY5R	-3.15E+00	2.33E-12	MBNL2	-1.06E-01	1.00E+00	PAM16	5.25E-02	1.00E+00
KCNC1	3.10E+00	2.33E-12	C1QL3	1.84E-01	1.00E+00	LPCAT1	3.49E-02	1.00E+00
MGST3	-1.19E+00	2.34E-12	LOC112580319	7.75E-02	1.00E+00	SERINC3	-4.57E-02	1.00E+00
PAFAH2	-1.26E+00	2.35E-12	SLC12A1	-9.60E-02	1.00E+00	LOC102394049	-6.60E-02	1.00E+00
LOC112580887	4.95E+00	2.35E-12	TUBG2	3.84E-02	1.00E+00	MTMR1	-3.79E-02	1.00E+00
POLR1A	7.37E-01	2.38E-12	LOC102395369	-1.17E-01	1.00E+00	LOC112586860	-8.16E-02	1.00E+00
LOC102396260	-8.89E-01	2.39E-12	UBE2M	4.35E-02	1.00E+00	ADM5	-1.06E-01	1.00E+00
MPHOSPH8	6.17E-01	2.44E-12	LOC102391792	-1.13E-01	1.00E+00	ARHGAP22	-6.05E-02	1.00E+00
KIAA1841	-1.36E+00	2.49E-12	LOC112578871	1.78E-01	1.00E+00	AFTPH	-4.59E-02	1.00E+00
LOC112586856	3.11E+00	2.51E-12	TMEM42	-5.01E-02	1.00E+00	GPX4	5.20E-02	1.00E+00
SPARCL1	-3.69E+00	2.52E-12	SLC18A2	6.31E-02	1.00E+00	MED19	3.97E-02	1.00E+00
MSL1	6.48E-01	2.53E-12	TMEM232	-6.57E-02	1.00E+00	LOC102414288	-4.20E-02	1.00E+00
MEP1A	4.00E+00	2.54E-12	SRP9	5.54E-02	1.00E+00	TAF3	4.32E-02	1.00E+00
ZBED9	-2.87E+00	2.55E-12	ZNF583	3.75E-02	1.00E+00	PUF60	-4.21E-02	1.00E+00
LOC112587808	3.21E+00	2.60E-12	APH1B	3.72E-02	1.00E+00	G6PC3	-4.13E-02	1.00E+00
BORA	9.13E-01	2.62E-12	EIF2AK1	-3.53E-02	1.00E+00	NUDT8	-6.91E-02	1.00E+00
CERKL	1.58E+00	2.62E-12	LOC102407663	1.24E-01	1.00E+00	KATNB1	-3.57E-02	1.00E+00
OSGIN2	8.48E-01	2.63E-12	LAPTM4A	3.51E-02	1.00E+00	SLC30A9	-3.94E-02	1.00E+00
HCST	-2.40E+00	2.64E-12	LOC112578872	9.20E-02	1.00E+00	LOC102410373	-5.34E-02	1.00E+00
LOC112579183	3.50E+00	2.65E-12	BSPRY	-4.19E-02	1.00E+00	ALPK2	7.90E-02	1.00E+00
LOC102389167	-2.51E+00	2.67E-12	ZNF787	-1.19E-01	1.00E+00	SLC5A8	4.26E-02	1.00E+00
LOC102392741	2.88E+00	2.67E-12	SLC30A1	-7.09E-02	1.00E+00	GPHN	3.94E-02	1.00E+00
EXOSC8	9.22E-01	2.71E-12	BOLA2B	-5.56E-02	1.00E+00	NANOS1	5.70E-02	1.00E+00
FARSB	9.35E-01	2.73E-12	SLC35B2	8.64E-02	1.00E+00	TAPT1	-4.62E-02	1.00E+00
LOC102414362	-3.52E+00	2.75E-12	ATP5ME	-6.62E-02	1.00E+00	IL13RA1	-3.97E-02	1.00E+00
UCHL1	1.12E+00	2.75E-12	SLC27A3	-1.09E-01	1.00E+00	GPX2	-7.97E-02	1.00E+00
TINAG	-7.11E+00	2.79E-12	HEXIM1	-1.26E-01	1.00E+00	ETF1	-4.07E-02	1.00E+00
LOC102391124	-3.83E+00	2.81E-12	GRAMD1B	4.31E-02	1.00E+00	PSMA4	4.46E-02	1.00E+00

PLIN5	-3.62E+00	2.81E-12	GNL3L	-1.27E-01	1.00E+00	LOC102413100	5.53E-02	1.00E+00
GAS2L3	-1.37E+00	2.82E-12	ARPC5	4.10E-02	1.00E+00	DNAJC18	-3.83E-02	1.00E+00
LOC102407968	-1.33E+00	2.87E-12	TMEM53	-5.69E-02	1.00E+00	VANGL2	8.07E-02	1.00E+00
ADD1	7.19E-01	2.88E-12	LOC112586516	1.24E-01	1.00E+00	TMEM59	-3.51E-02	1.00E+00
ENO3	-1.57E+00	2.92E-12	CCDC17	-9.77E-02	1.00E+00	TRAF6	4.30E-02	1.00E+00
TSN	7.36E-01	2.92E-12	LOC102398394	2.70E-01	1.00E+00	ZDHHC24	-3.85E-02	1.00E+00
ATL1	2.01E+00	2.94E-12	BCKDHA	-5.09E-02	1.00E+00	LOC102403333	5.62E-02	1.00E+00
UBE2K	6.42E-01	2.98E-12	B3GNT3	-5.18E-02	1.00E+00	LOC102400440	-3.23E-02	1.00E+00
ABHD8	7.94E-01	2.99E-12	SEMA3C	-2.55E-01	1.00E+00	RDX	3.76E-02	1.00E+00
LOC102392023	1.59E+00	3.04E-12	OSR1	-1.70E-01	1.00E+00	OXR1	-4.80E-02	1.00E+00
LOC112587024	-3.35E+00	3.07E-12	SLC6A4	4.44E-02	1.00E+00	RRP15	4.07E-02	1.00E+00
TANK	6.89E-01	3.15E-12	NUTM1	1.53E-01	1.00E+00	WDR48	-3.38E-02	1.00E+00
FBXO2	-2.26E+00	3.21E-12	ZRSR2	-5.17E-02	1.00E+00	LOXL2	1.03E-01	1.00E+00
EME2	-1.34E+00	3.26E-12	SLC35G2	8.79E-02	1.00E+00	RCN2	-3.56E-02	1.00E+00
SLC6A17	-4.78E+00	3.27E-12	POGK	-7.25E-02	1.00E+00	ZFYVE9	3.35E-02	1.00E+00
PLEKHH2	-3.33E+00	3.30E-12	IRF8	3.40E-02	1.00E+00	ZMYND19	4.96E-02	1.00E+00
FAM129B	9.92E-01	3.36E-12	LOC112585882	-1.20E-01	1.00E+00	C3H9orf3	3.71E-02	1.00E+00
CCNL2	-6.89E-01	3.36E-12	LOC102397874	-1.41E-01	1.00E+00	SYCE2	6.83E-02	1.00E+00
LOC112581234	-2.83E+00	3.40E-12	LOC102413337	-1.01E-01	1.00E+00	GATD1	-4.24E-02	1.00E+00
NDUFAF3	-1.29E+00	3.41E-12	FOXO3	-5.36E-02	1.00E+00	TAF8	-4.72E-02	1.00E+00
USP4	-6.06E-01	3.46E-12	DPY19L1	5.10E-02	1.00E+00	C1H21orf58	-8.18E-02	1.00E+00
UBA7	-1.42E+00	3.47E-12	TDRD9	4.36E-02	1.00E+00	MRVI1	-5.94E-02	1.00E+00
SLC35A2	-7.49E-01	3.49E-12	TM2D1	4.90E-02	1.00E+00	LOC102403789	-4.55E-02	1.00E+00
LOC102414482	1.30E+00	3.52E-12	ARSJ	-3.57E-01	1.00E+00	GIN1	-4.56E-02	1.00E+00
PIK3R1	-9.67E-01	3.66E-12	HAP1	1.52E-01	1.00E+00	SMPD1	3.45E-02	1.00E+00
HESX1	-2.75E+00	3.67E-12	LOC102409250	4.39E-02	1.00E+00	LOC102389932	7.50E-02	1.00E+00
CEP44	8.46E-01	3.68E-12	LOC102396030	1.25E-01	1.00E+00	HOXC4	7.73E-02	1.00E+00
MALSU1	1.12E+00	3.69E-12	LOC112585257	-5.19E-02	1.00E+00	KIAA1107	5.19E-02	1.00E+00
NDUFB5	-9.82E-01	3.70E-12	PRMT8	1.88E-01	1.00E+00	CDC42BPB	-3.50E-02	1.00E+00
NRBF2	-1.20E+00	3.74E-12	CCNL2	3.92E-02	1.00E+00	LOC112581449	5.51E-02	1.00E+00
TGFBI	-2.48E+00	3.75E-12	LOC102397654	-5.94E-02	1.00E+00	IPO11	-3.33E-02	1.00E+00
NT5C2	6.25E-01	3.79E-12	COG5	-3.10E-02	1.00E+00	COPZ1	-3.82E-02	1.00E+00
LOC102389856	1.41E+00	3.83E-12	LOC102391272	-4.08E-	1.00E+00	TMEM126A	4.82E-02	1.00E+00

				02				
LOC102407805	-1.09E+00	3.87E-12	ELAC2	4.39E-02	1.00E+00	SECISBP2L	5.49E-02	1.00E+00
TMEM249	-2.70E+00	3.90E-12	LOC112586188	8.95E-02	1.00E+00	NOL7	-3.81E-02	1.00E+00
MRGPRF	4.40E+00	3.92E-12	PFKFB1	1.20E-01	1.00E+00	ZNF518B	4.07E-02	1.00E+00
DLC1	-2.85E+00	3.95E-12	M6PR	4.40E-02	1.00E+00	MXD4	3.16E-02	1.00E+00
LOC102415544	2.93E+00	4.02E-12	SLC6A7	-1.23E-01	1.00E+00	LOC102404867	7.27E-02	1.00E+00
FAM198A	-3.07E+00	4.02E-12	PLEKHG6	2.47E-01	1.00E+00	QTRT1	-4.43E-02	1.00E+00
EXD3	-1.03E+00	4.04E-12	INF2	2.84E-01	1.00E+00	COL4A6	-5.92E-02	1.00E+00
LOC102408707	3.96E+00	4.14E-12	FKBP9	5.42E-02	1.00E+00	ZNF148	-3.84E-02	1.00E+00
LOC102414856	-1.52E+00	4.15E-12	LINGO3	-7.08E-02	1.00E+00	ANXA4	-3.80E-02	1.00E+00
ACP5	-2.96E+00	4.19E-12	CFD	1.79E-01	1.00E+00	SUPT16H	3.20E-02	1.00E+00
IFT122	-8.71E-01	4.40E-12	MINK1	3.76E-02	1.00E+00	RCBTB1	-3.33E-02	1.00E+00
LOC112581856	-2.48E+00	4.48E-12	TIPIN	4.84E-02	1.00E+00	FAM135A	5.29E-02	1.00E+00
NKG7	-5.46E+00	4.63E-12	PTPMT1	-7.49E-02	1.00E+00	MBNL2	-3.55E-02	1.00E+00
TMEM18	-9.57E-01	4.64E-12	RWDD2A	-3.12E-02	1.00E+00	UBA2	-3.30E-02	1.00E+00
CENPC	7.63E-01	4.64E-12	ACBD6	8.39E-02	1.00E+00	FGD4	6.33E-02	1.00E+00
KRI1	-8.26E-01	4.75E-12	C2CD4C	1.34E-01	1.00E+00	KAT8	-3.28E-02	1.00E+00
HNRNPUL2	-6.37E-01	4.81E-12	CARD14	1.31E-01	1.00E+00	WEE1	-4.01E-02	1.00E+00
COMP	2.53E+00	4.82E-12	PPEF2	4.55E-02	1.00E+00	RARRES1	7.89E-02	1.00E+00
UTP15	9.17E-01	4.84E-12	KLHL15	7.46E-02	1.00E+00	PLA2G15	-4.52E-02	1.00E+00
BBOF1	1.73E+00	4.89E-12	DDX54	3.86E-02	1.00E+00	ARL8A	-3.35E-02	1.00E+00
TMEM53	1.04E+00	4.91E-12	RSPH9	1.16E-01	1.00E+00	PDS5A	3.54E-02	1.00E+00
DHFR	1.04E+00	4.94E-12	PEG10	6.67E-02	1.00E+00	N4BP2L2	-3.21E-02	1.00E+00
LOC102405841	-3.47E+00	4.99E-12	SMAD7	-1.68E-01	1.00E+00	LOC102411979	5.32E-02	1.00E+00
MRPS5	-8.04E-01	5.01E-12	LOC102414554	8.67E-02	1.00E+00	PIN4	4.95E-02	1.00E+00
HR	-3.44E+00	5.04E-12	C17H4orf33	4.05E-02	1.00E+00	C3H9orf84	8.18E-02	1.00E+00
NPEPPS	-7.55E-01	5.07E-12	RAB8A	4.17E-02	1.00E+00	CDC42BPA	-3.76E-02	1.00E+00
LOC102404484	-1.07E+00	5.08E-12	RIMS3	4.53E-02	1.00E+00	SH3TC1	-8.94E-02	1.00E+00
GRIN2A	1.58E+00	5.27E-12	IQSEC3	1.81E-01	1.00E+00	PSMB7	4.75E-02	1.00E+00
LOC112579706	3.10E+00	5.36E-12	LOC112583715	2.09E-01	1.00E+00	IPCEF1	8.06E-02	1.00E+00
RAD23B	6.90E-01	5.37E-12	GMIP	1.18E-01	1.00E+00	CD24	-7.44E-02	1.00E+00
IMPA1	7.32E-01	5.42E-12	LOC112578679	9.61E-02	1.00E+00	GDI2	-3.73E-02	1.00E+00
EPHA4	1.72E+00	5.42E-12	PPP2R2A	3.76E-02	1.00E+00	OXSR1	3.30E-02	1.00E+00
LOC112584587	-3.12E+00	5.46E-12	LOC102390247	1.13E-01	1.00E+00	PLRG1	-3.49E-02	1.00E+00
SFXN5	-1.24E+00	5.51E-12	ARSA	5.22E-02	1.00E+00	PTTG1IP	3.25E-02	1.00E+00

LOC102401224	-1.76E+00	5.52E-12	LHX1	1.02E-01	1.00E+00	TIMM10B	3.97E-02	1.00E+00
SERINC2	2.91E+00	5.52E-12	VPS33A	-4.63E-02	1.00E+00	NT5C3B	-4.19E-02	1.00E+00
TBC1D2	9.42E-01	5.52E-12	CFAP61	-1.11E-01	1.00E+00	PHF24	7.10E-02	1.00E+00
ZBTB2	8.30E-01	5.57E-12	LOC102398050	9.03E-02	1.00E+00	VPS13D	-3.26E-02	1.00E+00
DENND5A	-8.26E-01	5.59E-12	CDH5	-1.29E-01	1.00E+00	MED14	4.29E-02	1.00E+00
ADAMTS17	1.25E+00	5.70E-12	GNB3	-1.30E-01	1.00E+00	LRIG1	-5.17E-02	1.00E+00
MAP3K15	7.74E-01	5.72E-12	CHPF2	5.41E-02	1.00E+00	CDKN2D	-4.54E-02	1.00E+00
IL23A	-1.06E+00	5.75E-12	ZNF792	5.11E-02	1.00E+00	B3GALT4	-5.25E-02	1.00E+00
RRS1	8.96E-01	5.82E-12	CKAP4	3.62E-02	1.00E+00	LOC112583101	6.74E-02	1.00E+00
CCDC24	-2.00E+00	5.85E-12	FAM172A	5.86E-02	1.00E+00	CCT8	3.68E-02	1.00E+00
GGH	-1.29E+00	5.86E-12	SRRM1	1.13E-01	1.00E+00	LRCH2	5.26E-02	1.00E+00
IL3RA	-2.96E+00	5.95E-12	MARK4	4.11E-02	1.00E+00	IDUA	-3.64E-02	1.00E+00
CPNE8	-3.68E+00	5.98E-12	TMEM217	-1.52E-01	1.00E+00	CNOT9	-3.08E-02	1.00E+00
SNAPIN	9.08E-01	6.03E-12	LOC102397558	-6.44E-02	1.00E+00	TMEM230	-3.34E-02	1.00E+00
FCHO1	1.23E+00	6.08E-12	DHX34	9.27E-02	1.00E+00	PSMD4	4.67E-02	1.00E+00
PHKB	-7.10E-01	6.10E-12	PSMD9	-3.10E-02	1.00E+00	LIN28B	-5.48E-02	1.00E+00
NUP188	7.81E-01	6.15E-12	THOP1	-3.52E-02	1.00E+00	KIAA1671	4.08E-02	1.00E+00
FCRLB	1.84E+00	6.22E-12	PDLIM5	4.14E-02	1.00E+00	RXRB	3.50E-02	1.00E+00
BBS7	-1.24E+00	6.32E-12	COG8	6.34E-02	1.00E+00	TSPYL1	-3.76E-02	1.00E+00
MOCS3	9.13E-01	6.41E-12	AP1G1	-4.37E-02	1.00E+00	TMEM11	-3.95E-02	1.00E+00
ARCN1	-6.60E-01	6.43E-12	CDK5RAP2	3.35E-02	1.00E+00	EMC7	3.34E-02	1.00E+00
PTPN4	-9.10E-01	6.50E-12	VAMP7	-3.23E-02	1.00E+00	SLC39A7	-4.05E-02	1.00E+00
WRAP73	8.86E-01	6.71E-12	LOC112585666	-1.27E-01	1.00E+00	MESD	-3.57E-02	1.00E+00
LOC102410337	-7.05E+00	6.78E-12	MTFMT	6.46E-02	1.00E+00	RRP7A	3.93E-02	1.00E+00
TRIM28	-9.47E-01	6.93E-12	LOC112581897	-1.37E-01	1.00E+00	ARMCX2	-4.07E-02	1.00E+00
TNXB	-2.61E+00	6.93E-12	DFFA	4.86E-02	1.00E+00	PUSL1	5.89E-02	1.00E+00
DPAGT1	7.91E-01	7.14E-12	LOC102402010	-1.60E-01	1.00E+00	AGFG1	3.37E-02	1.00E+00
LOC112586128	-2.53E+00	7.14E-12	LRRC24	-6.30E-02	1.00E+00	MAP3K13	5.79E-02	1.00E+00
C11H14orf39	4.69E+00	7.20E-12	ESYT2	3.88E-02	1.00E+00	MORC3	-5.22E-02	1.00E+00
CDYL2	9.72E-01	7.24E-12	CDX2	-5.18E-02	1.00E+00	CEP128	4.16E-02	1.00E+00
POLR2B	-6.24E-01	7.25E-12	NKIRAS1	-4.40E-02	1.00E+00	PSMD1	3.24E-02	1.00E+00
ENOPH1	-7.56E-01	7.27E-12	SPEF1	1.47E-01	1.00E+00	LOC112577707	5.10E-02	1.00E+00
GPR27	1.18E+00	7.28E-12	SIK1	9.36E-02	1.00E+00	E4F1	-4.08E-02	1.00E+00

LOC112580239	-4.85E+00	7.29E-12	MOGS	7.49E-02	1.00E+00	PNPLA1	-8.78E-02	1.00E+00
LOC102391650	3.97E+00	7.33E-12	TBX5	-1.52E-01	1.00E+00	LOC102412417	3.53E-02	1.00E+00
KCNAB2	-1.98E+00	7.35E-12	BRMS1L	4.19E-02	1.00E+00	AKT1S1	-3.67E-02	1.00E+00
ERC1	7.14E-01	7.47E-12	LRRC6	7.13E-02	1.00E+00	SFI1	-3.84E-02	1.00E+00
TMEM209	7.66E-01	7.52E-12	OSTM1	-9.01E-02	1.00E+00	TSFM	3.92E-02	1.00E+00
GPX2	1.45E+00	7.53E-12	KLHL42	-4.86E-02	1.00E+00	LOC102397881	3.77E-02	1.00E+00
MFSD2B	-2.16E+00	7.56E-12	TM9SF2	-3.13E-02	1.00E+00	CDC34	-3.62E-02	1.00E+00
SPATA7	-8.12E-01	7.57E-12	VEZT	-7.21E-02	1.00E+00	LOC102408561	-7.24E-02	1.00E+00
KCNK13	1.72E+00	7.61E-12	RASA2	-8.21E-02	1.00E+00	PLS3	3.69E-02	1.00E+00
MMACHC	-1.18E+00	7.82E-12	TMUB1	-6.19E-02	1.00E+00	DNAJC19	3.96E-02	1.00E+00
PRKRA	6.44E-01	7.92E-12	SYT2	-7.40E-02	1.00E+00	AP2A2	3.19E-02	1.00E+00
SCN9A	-1.50E+00	8.09E-12	ITFG2	-5.15E-02	1.00E+00	MFN1	-3.50E-02	1.00E+00
MAGEL2	-5.08E+00	8.26E-12	PPP1R3E	-1.61E-01	1.00E+00	YAF2	-3.95E-02	1.00E+00
WASHC3	1.03E+00	8.33E-12	MARF1	5.30E-02	1.00E+00	INTU	-5.41E-02	1.00E+00
TARSL2	8.71E-01	8.39E-12	POMT1	6.28E-02	1.00E+00	SOCS7	4.55E-02	1.00E+00
MVK	-1.00E+00	8.39E-12	SH3BGRL3	-6.42E-02	1.00E+00	ANKMY1	-7.78E-02	1.00E+00
LOC112577691	-2.95E+00	8.39E-12	LOC112583917	-1.22E-01	1.00E+00	TMEM86B	8.10E-02	1.00E+00
LOC112579214	4.31E+00	8.40E-12	LUC7L3	-3.84E-02	1.00E+00	ATP5MG	-4.28E-02	1.00E+00
LOC112584711	1.63E+00	8.41E-12	GMEB2	-4.92E-02	1.00E+00	AP4E1	-3.64E-02	1.00E+00
LOC102403587	1.46E+00	8.44E-12	AP4M1	-6.66E-02	1.00E+00	RFC2	-4.34E-02	1.00E+00
MAGEA10	4.19E+00	8.44E-12	TTC33	-1.95E-01	1.00E+00	UHRF1BP1L	4.32E-02	1.00E+00
BAX	-9.03E-01	8.54E-12	POLR3E	6.81E-02	1.00E+00	OGFOD2	4.87E-02	1.00E+00
PLEKHG2	1.07E+00	8.64E-12	RNF123	-4.81E-02	1.00E+00	ABHD8	3.84E-02	1.00E+00
LOC112581131	2.20E+00	8.67E-12	ECHDC1	-6.38E-02	1.00E+00	RIF1	4.59E-02	1.00E+00
SPATA24	-1.06E+00	8.69E-12	SMARCC2	5.97E-02	1.00E+00	UBA3	-3.10E-02	1.00E+00
LOC102402959	3.17E+00	8.76E-12	LOC102399865	-1.12E-01	1.00E+00	ST3GAL3	3.36E-02	1.00E+00
NUDT2	1.10E+00	8.91E-12	LOC112579201	-1.95E-01	1.00E+00	PLCG1	3.77E-02	1.00E+00
CARD6	-3.96E+00	8.94E-12	NAGK	4.59E-02	1.00E+00	CD70	-8.78E-02	1.00E+00
STYXL1	-1.57E+00	9.47E-12	TCIRG1	-1.04E-01	1.00E+00	ARHGAP27	1.12E-01	1.00E+00
TRPC5	3.12E+00	9.73E-12	METAP1	6.21E-02	1.00E+00	OAF	-4.16E-02	1.00E+00
GHR	1.49E+00	9.83E-12	IMPA2	-8.02E-02	1.00E+00	RPL22	-6.00E-02	1.00E+00
MUS81	7.82E-01	1.00E-11	PQLC3	-1.92E-01	1.00E+00	SYTL4	-3.90E-02	1.00E+00
MLC1	4.36E+00	1.01E-11	TMEM233	-2.31E-	1.00E+00	LOC102404894	-4.69E-	1.00E+00

				01			02	
VDAC1	-7.06E-01	1.04E-11	NLGN3	-2.07E-01	1.00E+00	GHITM	-3.21E-02	1.00E+00
CTCF	-6.58E-01	1.04E-11	UBE2G2	4.48E-02	1.00E+00	ITGAM	-9.42E-02	1.00E+00
LOC112587651	3.84E+00	1.04E-11	GMFB	6.75E-02	1.00E+00	ASB12	6.92E-02	1.00E+00
ADGRV1	8.84E-01	1.05E-11	RPGRIP1	-3.74E-02	1.00E+00	PYCR2	3.86E-02	1.00E+00
HYDIN	1.38E+00	1.05E-11	CYFIP1	-5.07E-02	1.00E+00	EDC4	-3.07E-02	1.00E+00
TDRD3	-6.27E-01	1.07E-11	RHBDD2	7.95E-02	1.00E+00	MRPL16	-3.80E-02	1.00E+00
LOC102392737	-4.46E+00	1.09E-11	ATG101	-4.09E-02	1.00E+00	ACTR10	-3.11E-02	1.00E+00
WDR89	9.42E-01	1.09E-11	LOC102395499	-4.71E-02	1.00E+00	BTBD3	-5.58E-02	1.00E+00
UBTD2	-9.04E-01	1.10E-11	HORMAD1	1.11E-01	1.00E+00	LOC102413394	-4.75E-02	1.00E+00
FAAP20	-1.09E+00	1.10E-11	MOSPD1	-5.54E-02	1.00E+00	IRF8	-1.46E-01	1.00E+00
LOC112581423	-2.79E+00	1.10E-11	GCSH	9.98E-02	1.00E+00	LOC102411662	5.67E-02	1.00E+00
LUZP2	3.35E+00	1.14E-11	ONECUT1	-1.01E-01	1.00E+00	LOC102401932	-5.90E-02	1.00E+00
LOC102392736	-7.97E-01	1.15E-11	TMF1	-6.14E-02	1.00E+00	NPLOC4	-3.03E-02	1.00E+00
LOC112583917	2.38E+00	1.15E-11	WIPF1	-8.89E-02	1.00E+00	AGK	-3.60E-02	1.00E+00
LOC112583743	5.64E+00	1.16E-11	FAM210A	6.37E-02	1.00E+00	STMN1	4.42E-02	1.00E+00
LOC112582094	2.37E+00	1.17E-11	SHANK3	5.01E-02	1.00E+00	LOC112585084	-5.64E-02	1.00E+00
WDR78	-1.13E+00	1.20E-11	ADA	-1.50E-01	1.00E+00	CILP	7.37E-02	1.00E+00
SDHC	8.14E-01	1.22E-11	PIP5K1C	4.76E-02	1.00E+00	CNTD2	-6.49E-02	1.00E+00
INTS6	-7.08E-01	1.23E-11	LOC112585751	-8.52E-02	1.00E+00	C10H6orf163	-7.67E-02	1.00E+00
LOC102405308	-4.22E+00	1.24E-11	NR2F6	-9.21E-02	1.00E+00	PSMC2	3.63E-02	1.00E+00
PPP1R9A	-1.33E+00	1.25E-11	LOC102399272	-8.71E-02	1.00E+00	UBQLN1	3.54E-02	1.00E+00
APAF1	1.08E+00	1.28E-11	SMC1A	-5.09E-02	1.00E+00	BABAM2	3.25E-02	1.00E+00
CHCHD3	6.58E-01	1.30E-11	LRPAP1	-4.12E-02	1.00E+00	TRAPPC2L	3.81E-02	1.00E+00
GRID1	2.11E+00	1.32E-11	GINS3	-3.85E-02	1.00E+00	PPARG	-3.69E-02	1.00E+00
LOC102402179	-2.23E+00	1.33E-11	NIFK	-3.44E-02	1.00E+00	MARK2	3.24E-02	1.00E+00
RBM15	-8.24E-01	1.33E-11	SLC7A5	6.56E-02	1.00E+00	RPRD2	-3.35E-02	1.00E+00
LOC102394334	6.46E+00	1.34E-11	LRRC40	8.28E-02	1.00E+00	TRAPPC6A	5.20E-02	1.00E+00
LOC112581797	-3.19E+00	1.35E-11	MEA1	-8.37E-02	1.00E+00	BACE2	4.18E-02	1.00E+00
BCKDHB	-8.13E-01	1.35E-11	PRKAR2A	-4.36E-02	1.00E+00	RFTN2	-5.45E-02	1.00E+00
YTHDF2	-6.87E-01	1.35E-11	QRFPR	-1.39E-01	1.00E+00	DNAJA4	3.20E-02	1.00E+00
GINM1	6.21E-01	1.36E-11	SYNJ1	4.90E-02	1.00E+00	SMO	-3.25E-02	1.00E+00
SLC39A9	-8.29E-01	1.37E-11	SFMBT2	7.63E-02	1.00E+00	LOC102396331	7.43E-02	1.00E+00
CYTH2	5.86E-01	1.40E-11	ZC3H7A	7.78E-02	1.00E+00	ZNF285	-7.07E-	1.00E+00

							02	
HDAC4	-9.90E-01	1.40E-11	TMX2	3.38E-02	1.00E+00	KCNJ11	-7.48E-02	1.00E+00
WDR26	7.97E-01	1.40E-11	XYLB	-1.57E-01	1.00E+00	ACTN4	3.55E-02	1.00E+00
EDF1	-1.05E+00	1.41E-11	RECQL	6.58E-02	1.00E+00	CDC42EP5	-8.26E-02	1.00E+00
CHD7	-1.12E+00	1.41E-11	METAP2	-3.09E-02	1.00E+00	KIF1B	4.31E-02	1.00E+00
EBF2	3.71E+00	1.44E-11	SGPL1	-5.94E-02	1.00E+00	GARS	3.09E-02	1.00E+00
GAREM2	-1.42E+00	1.44E-11	ZNF74	1.85E-01	1.00E+00	PACRGL	-3.60E-02	1.00E+00
POLI	6.82E-01	1.45E-11	PARP8	2.13E-01	1.00E+00	EEF1E1	3.91E-02	1.00E+00
CDC42BPB	-7.94E-01	1.49E-11	CFAP45	-1.70E-01	1.00E+00	LGALS8	6.16E-02	1.00E+00
C1R	-3.92E+00	1.50E-11	CAPZA2	-5.05E-02	1.00E+00	TPM1	-6.06E-02	1.00E+00
MRPL46	-1.16E+00	1.51E-11	TRNAR-ACG-12	-1.80E-01	1.00E+00	WBP2	-3.06E-02	1.00E+00
SMAD5	-1.19E+00	1.54E-11	PEX10	4.64E-02	1.00E+00	IDS	-3.95E-02	1.00E+00
CSF1R	3.06E+00	1.54E-11	DAG1	5.08E-02	1.00E+00	WASHC4	4.36E-02	1.00E+00
FCHO2	-1.29E+00	1.55E-11	CDC42EP5	-1.98E-01	1.00E+00	RYBP	-3.37E-02	1.00E+00
TMEM128	-1.14E+00	1.56E-11	LOC102413681	9.22E-02	1.00E+00	LOC112585257	5.34E-02	1.00E+00
RNASEH2A	-1.23E+00	1.59E-11	LRRC20	5.90E-02	1.00E+00	LOC102397029	4.29E-02	1.00E+00
LOC112580616	3.85E+00	1.60E-11	MRPL14	4.34E-02	1.00E+00	C3H9orf72	4.03E-02	1.00E+00
TBC1D10B	7.08E-01	1.60E-11	KLF5	6.28E-02	1.00E+00	PDGFD	9.04E-02	1.00E+00
HSPB11	8.72E-01	1.62E-11	MARVELD2	4.36E-02	1.00E+00	EPDR1	3.46E-02	1.00E+00
LOC112581642	-1.43E+00	1.63E-11	RPGR	-6.28E-02	1.00E+00	ZNF660	-5.39E-02	1.00E+00
LOC112587335	3.01E+00	1.64E-11	TCP11L1	-3.47E-02	1.00E+00	CENPB	3.96E-02	1.00E+00
MROH5	2.81E+00	1.68E-11	ADCY6	-1.42E-01	1.00E+00	DPY19L4	3.93E-02	1.00E+00
MTMR6	7.22E-01	1.71E-11	MTHFR	9.30E-02	1.00E+00	TMEM219	4.64E-02	1.00E+00
ZFP3	9.74E-01	1.72E-11	ANKRD39	-7.54E-02	1.00E+00	PEX13	4.79E-02	1.00E+00
CYSTM1	7.18E-01	1.73E-11	FGF17	-1.22E-01	1.00E+00	SLF1	-4.63E-02	1.00E+00
RNF32	-1.81E+00	1.74E-11	STBD1	-5.19E-02	1.00E+00	PDE7B	3.16E-02	1.00E+00
SECISBP2L	1.07E+00	1.75E-11	GPX4	-4.29E-02	1.00E+00	CTDSP1	2.97E-02	1.00E+00
ITGAM	-3.52E+00	1.76E-11	PRSS2	4.11E-02	1.00E+00	TOMM34	-4.16E-02	1.00E+00
ADAMTS14	-1.44E+00	1.76E-11	SEMA4A	5.04E-02	1.00E+00	GALNT6	-1.41E-01	1.00E+00
RLF	-8.25E-01	1.77E-11	LSM2	3.57E-02	1.00E+00	TYMS	3.67E-02	1.00E+00
FGD1	6.92E-01	1.77E-11	VHL	-4.07E-02	1.00E+00	FAM213B	-7.11E-02	1.00E+00
TXNIP	-1.48E+00	1.77E-11	LOC102407045	-7.85E-02	1.00E+00	SLC2A4RG	-4.73E-02	1.00E+00
LOC112578056	2.40E+00	1.81E-11	MTPAP	-4.17E-02	1.00E+00	MRPS22	-3.05E-02	1.00E+00
ASIP	2.36E+00	1.81E-11	NARS	-3.59E-02	1.00E+00	USO1	3.84E-02	1.00E+00
SERPINB5	3.77E+00	1.82E-11	TMA16	-5.76E-	1.00E+00	NUDCD1	3.97E-02	1.00E+00

				02				
SNRNP200	-7.97E-01	1.82E-11	LOC112580421	-4.89E-02	1.00E+00	PMS1	3.94E-02	1.00E+00
EVA1B	-1.92E+00	1.82E-11	DBNL	-3.84E-02	1.00E+00	PTGER4	-1.36E-01	1.00E+00
MRPL16	-8.74E-01	1.84E-11	SERPINB5	-1.71E-01	1.00E+00	AMMECR1	-3.92E-02	1.00E+00
GFRA4	1.82E+00	1.85E-11	RASD1	1.13E-01	1.00E+00	FOXP4	3.15E-02	1.00E+00
LOC102408857	3.26E+00	1.89E-11	SRP54	4.26E-02	1.00E+00	NAA38	5.64E-02	1.00E+00
NTRK2	-3.30E+00	1.90E-11	ZNF326	-9.55E-02	1.00E+00	ZNF703	4.90E-02	1.00E+00
CDH8	-1.84E+00	1.90E-11	PRDX4	-4.66E-02	1.00E+00	VARS2	-3.85E-02	1.00E+00
RASGEF1C	2.07E+00	1.93E-11	MTRF1L	4.75E-02	1.00E+00	HEATR6	3.10E-02	1.00E+00
CD40LG	2.87E+00	1.94E-11	TBC1D8	-3.88E-02	1.00E+00	LOC112578502	4.94E-02	1.00E+00
RHOT2	-7.55E-01	1.95E-11	ZNF580	1.26E-01	1.00E+00	KLHL18	3.62E-02	1.00E+00
KIF4A	8.22E-01	1.97E-11	LOC102411437	-1.22E-01	1.00E+00	PRPS1	-3.12E-02	1.00E+00
LOC112581399	-2.96E+00	1.98E-11	CA14	1.04E-01	1.00E+00	CLCN7	-3.05E-02	1.00E+00
MRPS35	7.17E-01	1.99E-11	MYO1C	5.29E-02	1.00E+00	ANKZF1	-3.46E-02	1.00E+00
LOC102401543	3.48E+00	2.01E-11	ABHD11	6.31E-02	1.00E+00	CHCHD5	3.95E-02	1.00E+00
SMPD2	-1.16E+00	2.03E-11	RORA	3.16E-02	1.00E+00	ZNF879	4.14E-02	1.00E+00
RNF225	-2.37E+00	2.14E-11	SAAL1	6.10E-02	1.00E+00	LOC112583711	-6.77E-02	1.00E+00
LOC102411603	-2.30E+00	2.15E-11	ZNF704	-1.79E-01	1.00E+00	DOC2B	9.54E-02	1.00E+00
ABRACL	1.04E+00	2.16E-11	PRNP	-5.50E-02	1.00E+00	TFAP4	-4.58E-02	1.00E+00
CD6	2.96E+00	2.18E-11	SLC38A11	2.05E-01	1.00E+00	PYCR3	4.20E-02	1.00E+00
NUDT16L1	-1.14E+00	2.29E-11	WDR47	4.03E-02	1.00E+00	TXNRD2	-4.52E-02	1.00E+00
WISP1	-3.74E+00	2.34E-11	AXIN1	3.55E-02	1.00E+00	RBSN	-3.01E-02	1.00E+00
ANKEF1	3.02E+00	2.36E-11	EMC7	-4.05E-02	1.00E+00	GIGYF2	-3.36E-02	1.00E+00
CNGA3	1.47E+00	2.37E-11	DTX2	3.42E-02	1.00E+00	DCLRE1B	3.99E-02	1.00E+00
RABL2B	-6.52E-01	2.37E-11	PDE5A	-4.98E-02	1.00E+00	COPS8	-2.96E-02	1.00E+00
SPSB3	-7.47E-01	2.41E-11	PRPF38A	-3.68E-02	1.00E+00	CNOT8	-3.04E-02	1.00E+00
AMBRA1	6.50E-01	2.50E-11	CMTM3	-8.43E-02	1.00E+00	RAC2	1.42E-01	1.00E+00
CDK12	9.89E-01	2.51E-11	LIN9	8.49E-02	1.00E+00	ZPBP2	6.83E-02	1.00E+00
INAVA	-1.64E+00	2.52E-11	OSGIN2	-4.12E-02	1.00E+00	JADE3	3.56E-02	1.00E+00
UBR2	-8.40E-01	2.53E-11	STMN3	-1.63E-01	1.00E+00	DPY30	3.87E-02	1.00E+00
LOC112578741	4.10E+00	2.54E-11	CPA1	3.45E-02	1.00E+00	CDK8	-3.47E-02	1.00E+00
ABAT	1.16E+00	2.56E-11	LOC102409389	1.62E-01	1.00E+00	FAM53B	-3.24E-02	1.00E+00
CC2D2A	-1.91E+00	2.56E-11	FMR1	4.92E-02	1.00E+00	RPS10	-6.41E-02	1.00E+00
PRKX	-1.11E+00	2.57E-11	ZEB1	7.61E-02	1.00E+00	COPG2	-3.08E-02	1.00E+00
SH2D3C	1.39E+00	2.58E-11	AZIN2	-3.80E-02	1.00E+00	KLHL20	-4.46E-02	1.00E+00

RAC1	-6.78E-01	2.59E-11	FKBPL	-1.51E-01	1.00E+00	ERI1	3.45E-02	1.00E+00
C16H11orf94	2.09E+00	2.62E-11	WDR46	-4.00E-02	1.00E+00	E2F4	-3.26E-02	1.00E+00
GSKIP	7.79E-01	2.62E-11	SPACA6	1.77E-01	1.00E+00	DCAF7	-3.27E-02	1.00E+00
RIMBP2	2.78E+00	2.62E-11	CPQ	-2.32E-01	1.00E+00	LOC112586215	6.08E-02	1.00E+00
LONP2	-6.53E-01	2.62E-11	SF3B3	-3.29E-02	1.00E+00	BTF3	-3.63E-02	1.00E+00
CDKL1	1.07E+00	2.63E-11	WDR41	-4.03E-02	1.00E+00	LOC112585163	-5.59E-02	1.00E+00
SLC22A15	1.90E+00	2.65E-11	CAVIN2	-1.33E-01	1.00E+00	ABHD10	-3.66E-02	1.00E+00
LOC102389850	-7.50E-01	2.65E-11	WNK4	9.60E-02	1.00E+00	CTDNEP1	-3.59E-02	1.00E+00
WIZ	7.42E-01	2.65E-11	ZAR1	4.97E-02	1.00E+00	PPP2R1A	-3.99E-02	1.00E+00
AP1B1	6.55E-01	2.68E-11	IFNAR1	-1.09E-01	1.00E+00	ERICH1	-4.03E-02	1.00E+00
TMX2	5.82E-01	2.72E-11	DSTN	-4.07E-02	1.00E+00	ATP5F1C	4.02E-02	1.00E+00
LOC112581898	-1.54E+00	2.72E-11	LOC112582327	-1.24E-01	1.00E+00	GSTZ1	-4.84E-02	1.00E+00
ZNF521	7.82E-01	2.75E-11	ZFX	5.97E-02	1.00E+00	MFSD12	5.56E-02	1.00E+00
KCNT1	2.23E+00	2.78E-11	KLHDC3	-3.17E-02	1.00E+00	LOC102410024	-3.13E-02	1.00E+00
LOC112587873	8.01E+00	2.79E-11	CRELD1	5.53E-02	1.00E+00	DUS4L	-4.64E-02	1.00E+00
TCF20	7.93E-01	2.79E-11	PLK4	5.37E-02	1.00E+00	EFR3A	-4.00E-02	1.00E+00
CSRNP1	-2.33E+00	2.80E-11	RANBP9	3.31E-02	1.00E+00	STAG1	-3.14E-02	1.00E+00
PCGF1	9.22E-01	2.83E-11	GFRA2	1.32E-01	1.00E+00	EDNRA	4.97E-02	1.00E+00
MAT2A	-6.99E-01	2.83E-11	SNX4	7.47E-02	1.00E+00	DCAF1	-3.41E-02	1.00E+00
JMJD4	-7.67E-01	2.83E-11	SGCE	-6.68E-02	1.00E+00	BRPF1	-3.21E-02	1.00E+00
C5H1orf167	1.76E+00	2.85E-11	POLE2	3.50E-02	1.00E+00	LOC112581444	7.30E-02	1.00E+00
TPR	-7.48E-01	2.86E-11	CIAO3	5.18E-02	1.00E+00	LGALSL	4.11E-02	1.00E+00
LOC112581533	1.34E+00	2.88E-11	RCC1	4.63E-02	1.00E+00	TRIM41	3.26E-02	1.00E+00
LOC112580864	-3.75E+00	2.89E-11	TRIM44	-4.09E-02	1.00E+00	NCK1	3.15E-02	1.00E+00
SYT14	3.28E+00	2.89E-11	LOC112579910	-4.52E-02	1.00E+00	MFSD9	-3.79E-02	1.00E+00
LOC112584575	-2.70E+00	2.91E-11	ADAM11	1.36E-01	1.00E+00	KCNN3	9.17E-02	1.00E+00
TMEM187	-1.93E+00	2.94E-11	ZNF311	1.15E-01	1.00E+00	CTNND1	-3.62E-02	1.00E+00
LOC102394013	-3.34E+00	2.94E-11	NDST2	-7.54E-02	1.00E+00	FMNL2	3.54E-02	1.00E+00
LOC102389674	-6.38E+00	2.95E-11	WIPI2	3.04E-02	1.00E+00	C4H12orf56	7.33E-02	1.00E+00
HMMR	-1.00E+00	2.97E-11	SPRYD7	4.70E-02	1.00E+00	PIP4P1	-3.24E-02	1.00E+00
RNF125	-3.88E+00	2.99E-11	ZMIZ1	-1.46E-01	1.00E+00	LIN9	-5.46E-02	1.00E+00
ABCC6	-2.97E+00	3.00E-11	PPP1R3B	-1.35E-01	1.00E+00	LOC102405199	9.62E-02	1.00E+00
LOC112582089	-3.01E+00	3.02E-11	FAM169B	-8.44E-02	1.00E+00	C14H20orf194	-4.37E-02	1.00E+00
C24H7orf61	-	3.03E-11	PON1	-1.44E-	1.00E+00	SMAD1	4.13E-02	1.00E+00

	2.11E+00			01				
ZNF404	1.37E+00	3.04E-11	RARS	-3.59E-02	1.00E+00	ZNF567	-4.54E-02	1.00E+00
STX11	-2.56E+00	3.04E-11	IFFO1	4.29E-02	1.00E+00	LOC112583836	-3.49E-02	1.00E+00
MINDY3	7.36E-01	3.04E-11	LOC112582199	-1.37E-01	1.00E+00	SELENOI	-3.60E-02	1.00E+00
PKNOX1	-7.50E-01	3.05E-11	RNF157	5.11E-02	1.00E+00	BID	4.04E-02	1.00E+00
KIF12	3.27E+00	3.11E-11	FAM160A2	3.41E-02	1.00E+00	KDSR	3.17E-02	1.00E+00
LOC112587399	4.06E+00	3.11E-11	PALLD	3.58E-02	1.00E+00	ZNF777	3.22E-02	1.00E+00
BEND4	1.03E+00	3.13E-11	MRGBP	2.96E-02	1.00E+00	TARSL2	-4.28E-02	1.00E+00
DLX5	2.96E+00	3.14E-11	PLCB1	-5.64E-02	1.00E+00	XPO1	3.13E-02	1.00E+00
DMXL1	-1.30E+00	3.21E-11	LOC112578658	1.87E-01	1.00E+00	FGFR1	3.15E-02	1.00E+00
PODXL	1.09E+00	3.23E-11	DLG4	-7.92E-02	1.00E+00	BARX1	-8.78E-02	1.00E+00
KXD1	-9.86E-01	3.23E-11	CT55	2.26E-01	1.00E+00	DBR1	-3.63E-02	1.00E+00
TMEM200C	-3.08E+00	3.27E-11	RFX6	-5.86E-02	1.00E+00	CD72	-7.53E-02	1.00E+00
SLC38A11	3.71E+00	3.33E-11	TMEM187	-1.69E-01	1.00E+00	DPH3	-4.16E-02	1.00E+00
ENTPD7	-1.52E+00	3.36E-11	WNT3	-8.40E-02	1.00E+00	CGAS	-5.93E-02	1.00E+00
SEC23A	-1.04E+00	3.39E-11	FAM214B	1.75E-01	1.00E+00	INAFM1	-4.25E-02	1.00E+00
PRELID2	9.19E-01	3.40E-11	LOC112586549	7.05E-02	1.00E+00	SLC25A38	-3.14E-02	1.00E+00
ATP5IF1	-1.07E+00	3.41E-11	NUP50	-4.53E-02	1.00E+00	LOC102416336	-8.38E-02	1.00E+00
LOC112580198	-2.66E+00	3.43E-11	LOC102390842	5.28E-02	1.00E+00	FERMT2	6.80E-02	1.00E+00
LOC102390278	1.76E+00	3.43E-11	RPL10	5.09E-02	1.00E+00	RBM11	6.74E-02	1.00E+00
SUN1	7.83E-01	3.44E-11	MMP24	1.57E-01	1.00E+00	WDR31	-4.23E-02	1.00E+00
TUT7	-8.95E-01	3.49E-11	CSGALNACT2	7.79E-02	1.00E+00	SHKBP1	-3.27E-02	1.00E+00
LOC112581794	-3.22E+00	3.62E-11	HMOX1	-7.06E-02	1.00E+00	LOC102389632	6.34E-02	1.00E+00
MEF2C	7.81E-01	3.63E-11	CES5A	1.18E-01	1.00E+00	F8	-5.97E-02	1.00E+00
MTX3	1.44E+00	3.65E-11	FOXRED1	-6.71E-02	1.00E+00	LOC102393048	3.59E-02	1.00E+00
NR4A2	-2.87E+00	3.67E-11	GFOD1	4.31E-02	1.00E+00	OGFRL1	-3.18E-02	1.00E+00
LOC102409683	-3.22E+00	3.67E-11	MTX1	-7.20E-02	1.00E+00	RAB6A	3.23E-02	1.00E+00
LOC102395458	4.80E+00	3.71E-11	POLL	-5.45E-02	1.00E+00	LOC102396103	3.10E-02	1.00E+00
FERMT3	2.27E+00	3.71E-11	C21H3orf62	-9.30E-02	1.00E+00	HECTD3	3.19E-02	1.00E+00
LOC112585683	3.41E+00	3.73E-11	LRRC32	1.08E-01	1.00E+00	SSB	-3.05E-02	1.00E+00
TRPM1	3.15E+00	3.81E-11	COPA	3.31E-02	1.00E+00	VPS54	3.21E-02	1.00E+00
EMX1	3.37E+00	3.82E-11	ACADS	-4.12E-02	1.00E+00	LOC102415373	-7.83E-02	1.00E+00
NEDD1	7.23E-01	3.82E-11	SRP72	-4.36E-02	1.00E+00	TAZ	-4.09E-02	1.00E+00
CERCAM	-9.07E-01	3.83E-11	KPNA3	4.31E-02	1.00E+00	BCL2L1	3.25E-02	1.00E+00

SGIP1	3.83E+00	3.86E-11	PRICKLE2	4.97E-02	1.00E+00	SACM1L	-3.20E-02	1.00E+00
PHC3	8.00E-01	3.93E-11	YPEL4	-3.40E-02	1.00E+00	PAFAH1B3	4.66E-02	1.00E+00
LOC102397551	4.51E+00	3.95E-11	PREP	-6.77E-02	1.00E+00	UBE2H	3.49E-02	1.00E+00
SFT2D2	1.45E+00	3.98E-11	FTO	4.41E-02	1.00E+00	COQ6	-3.56E-02	1.00E+00
ETV4	1.41E+00	4.00E-11	SLC19A1	-9.23E-02	1.00E+00	CCNG2	4.40E-02	1.00E+00
PLA2G2C	-2.06E+00	4.07E-11	KDM1A	-3.37E-02	1.00E+00	FIG4	3.22E-02	1.00E+00
VWA3B	2.74E+00	4.07E-11	FNDC3A	3.61E-02	1.00E+00	STK33	-4.81E-02	1.00E+00
MALL	-3.54E+00	4.08E-11	TMEM150B	1.71E-01	1.00E+00	ELP1	3.19E-02	1.00E+00
TAF1B	6.89E-01	4.14E-11	SAMD12	-3.50E-02	1.00E+00	RERG	4.09E-02	1.00E+00
LOC112584689	1.75E+00	4.24E-11	ZNF638	-4.60E-02	1.00E+00	PPP1R12A	-5.29E-02	1.00E+00
LOC112585749	1.30E+00	4.29E-11	COL23A1	-1.76E-01	1.00E+00	NPDC1	7.57E-02	1.00E+00
ERI2	-1.29E+00	4.32E-11	ISOC1	5.03E-02	1.00E+00	LOC112579251	-4.74E-02	1.00E+00
ENDOG	-1.16E+00	4.36E-11	LY6G6C	1.58E-01	1.00E+00	PAFAH2	-3.54E-02	1.00E+00
DLEC1	-7.97E-01	4.40E-11	XKR6	-1.32E-01	1.00E+00	MAF1	3.90E-02	1.00E+00
MICU1	-6.51E-01	4.42E-11	NELL2	6.82E-02	1.00E+00	RBMS2	3.11E-02	1.00E+00
C1QB	-5.50E+00	4.45E-11	LYPD1	3.70E-02	1.00E+00	SLC29A4	-3.74E-02	1.00E+00
SLC4A7	-1.53E+00	4.48E-11	RBMS2	-5.22E-02	1.00E+00	UCK2	3.67E-02	1.00E+00
MS4A8	-4.21E+00	4.48E-11	NEBL	-5.56E-02	1.00E+00	ZNF106	-3.48E-02	1.00E+00
SLK	-7.97E-01	4.52E-11	CENPV	-1.63E-01	1.00E+00	CMC4	4.98E-02	1.00E+00
NDFIP2	-1.05E+00	4.53E-11	PPOX	-8.07E-02	1.00E+00	LOC102413215	-4.54E-02	1.00E+00
FRMPD3	3.68E+00	4.53E-11	RRBP1	4.72E-02	1.00E+00	GATB	-2.97E-02	1.00E+00
FAM155A	-1.95E+00	4.57E-11	TNRC18	-5.04E-02	1.00E+00	NEK6	-9.70E-02	1.00E+00
GLRX3	-8.03E-01	4.58E-11	EIF1AD	-5.21E-02	1.00E+00	HCRTR1	9.34E-02	1.00E+00
NDUFS5	-1.22E+00	4.59E-11	CDC123	-3.21E-02	1.00E+00	NOL8	-3.17E-02	1.00E+00
LOC112586887	1.65E+00	4.64E-11	NEUROD4	-2.01E-01	1.00E+00	CBX5	-3.10E-02	1.00E+00
LOC102394078	-3.43E+00	4.77E-11	CMTR1	4.27E-02	1.00E+00	DCTN3	3.60E-02	1.00E+00
ASIC4	-1.30E+00	4.78E-11	SFMBT1	4.96E-02	1.00E+00	TMEFF1	-3.22E-02	1.00E+00
DUSP16	7.18E-01	4.79E-11	SHISA5	-5.37E-02	1.00E+00	BTBD1	3.26E-02	1.00E+00
LOC102397135	3.38E+00	4.87E-11	PPP1CB	4.45E-02	1.00E+00	TDRD3	-2.99E-02	1.00E+00
USP40	-7.61E-01	4.90E-11	THOC5	-3.51E-02	1.00E+00	FRRS1	-6.63E-02	1.00E+00
LOC112583845	3.93E+00	4.99E-11	ZCWPW1	3.29E-02	1.00E+00	AAAS	-3.39E-02	1.00E+00
RASA2	-1.28E+00	5.10E-11	MFSD10	6.47E-02	1.00E+00	TWF1	-3.87E-02	1.00E+00

ROR1	-1.33E+00	5.16E-11	TMEM126B	4.16E-02	1.00E+00	YTHDF3	-3.93E-02	1.00E+00
LOC102399865	-1.61E+00	5.17E-11	KRBA2	-6.89E-02	1.00E+00	PPP4R2	-3.97E-02	1.00E+00
LOC112582114	-3.05E+00	5.17E-11	EIF2B3	4.99E-02	1.00E+00	ZBED8	3.66E-02	1.00E+00
PLEKHG6	-2.58E+00	5.22E-11	LOC112583593	-6.97E-02	1.00E+00	CFAP161	4.93E-02	1.00E+00
HIPK2	-1.06E+00	5.22E-11	HS3ST2	-6.09E-02	1.00E+00	ZNF202	-4.09E-02	1.00E+00
SRPK2	6.01E-01	5.26E-11	MYLK	-1.44E-01	1.00E+00	ALG3	-4.24E-02	1.00E+00
LIX1L	-8.90E-01	5.28E-11	ISCA1	-3.71E-02	1.00E+00	SAT2	4.52E-02	1.00E+00
LOC112587552	3.44E+00	5.44E-11	ZNF414	6.61E-02	1.00E+00	ZC3H8	-3.28E-02	1.00E+00
LOC112586657	-1.80E+00	5.58E-11	CSRNP3	1.66E-01	1.00E+00	RAMP3	-1.22E-01	1.00E+00
C24H16orf72	-7.97E-01	5.63E-11	ETHE1	9.14E-02	1.00E+00	GNAI2	-3.28E-02	1.00E+00
LOC102399593	-2.13E+00	5.67E-11	BSG	-3.39E-02	1.00E+00	IFI30	-1.06E-01	1.00E+00
B3GNT3	-1.04E+00	5.71E-11	NECAP2	-4.43E-02	1.00E+00	TRUB2	-3.31E-02	1.00E+00
CMIP	8.32E-01	5.72E-11	ATP6V1B1	4.92E-02	1.00E+00	MRPS9	-3.33E-02	1.00E+00
BNIPL	1.71E+00	5.77E-11	SYT17	4.78E-02	1.00E+00	ZNF37A	-5.79E-02	1.00E+00
TNNT1	-2.21E+00	5.79E-11	LOC112580619	-2.64E-01	1.00E+00	PTOV1	-4.24E-02	1.00E+00
SYCE2	1.30E+00	5.80E-11	LOC102399969	3.64E-02	1.00E+00	ASCC1	-2.96E-02	1.00E+00
ADGRF3	1.97E+00	5.81E-11	CLNK	-1.71E-01	1.00E+00	SPAAR	7.38E-02	1.00E+00
LOC102402227	-9.62E-01	5.89E-11	SPCS1	-3.62E-02	1.00E+00	LARP1B	-4.06E-02	1.00E+00
JHY	3.65E+00	5.93E-11	NABP1	3.39E-02	1.00E+00	WIPF2	-3.60E-02	1.00E+00
NME6	-1.23E+00	5.95E-11	KDM2B	-5.04E-02	1.00E+00	ZSWIM1	-6.35E-02	1.00E+00
ZNF281	-1.03E+00	5.99E-11	TPR	5.06E-02	1.00E+00	LOC112582074	-8.91E-02	1.00E+00
LOC102397602	2.16E+00	6.01E-11	ARFGEF3	3.06E-02	1.00E+00	LOC102407140	4.78E-02	1.00E+00
CCDC114	-1.43E+00	6.21E-11	LOC112586105	8.04E-02	1.00E+00	ECHDC3	-5.07E-02	1.00E+00
LOC112585077	-3.36E+00	6.30E-11	SEPT5	6.05E-02	1.00E+00	CD3EAP	5.19E-02	1.00E+00
MON1A	-1.11E+00	6.41E-11	IFT46	-5.48E-02	1.00E+00	PNPT1	-3.12E-02	1.00E+00
ZNF536	-1.03E+00	6.41E-11	STAT6	-1.46E-01	1.00E+00	CLTRN	5.32E-02	1.00E+00
TRAPPC12	-7.84E-01	6.44E-11	FGF2	7.66E-02	1.00E+00	RNF112	8.03E-02	1.00E+00
CHD1L	6.55E-01	6.47E-11	SLC7A6OS	7.20E-02	1.00E+00	ZNF135	-4.36E-02	1.00E+00
EOGT	-1.09E+00	6.58E-11	TRIM62	-8.84E-02	1.00E+00	MDH2	3.83E-02	1.00E+00
LOC112584692	2.35E+00	6.59E-11	LOC112585187	-2.26E-01	1.00E+00	TRAP1	-3.93E-02	1.00E+00
DUS2	-1.07E+00	6.60E-11	DHPS	3.35E-02	1.00E+00	TMUB1	-4.54E-02	1.00E+00
SERTM1	3.03E+00	6.74E-11	GAL	-8.83E-02	1.00E+00	ICK	-3.88E-02	1.00E+00
CACNA1A	1.68E+00	6.75E-11	SLC17A5	4.91E-02	1.00E+00	BLVRB	-5.02E-	1.00E+00

							02	
ADGRL4	-3.65E+00	6.91E-11	NDUFB11	-4.89E-02	1.00E+00	OGG1	4.20E-02	1.00E+00
TMEM135	-8.79E-01	7.08E-11	GJB4	-5.15E-02	1.00E+00	KLHDC3	3.40E-02	1.00E+00
F2RL1	1.28E+00	7.10E-11	PMP22	6.82E-02	1.00E+00	CDK5RAP2	-3.32E-02	1.00E+00
LOC102392387	-2.50E+00	7.13E-11	ZNF570	1.03E-01	1.00E+00	LOC102415917	-3.88E-02	1.00E+00
TSKU	8.09E-01	7.13E-11	LOC112580379	-1.51E-01	1.00E+00	LOC112584584	-5.52E-02	1.00E+00
CD244	-4.96E+00	7.24E-11	KCTD15	8.36E-02	1.00E+00	IPO7	-3.67E-02	1.00E+00
SEMA6A	-2.13E+00	7.28E-11	TMEM51	2.92E-02	1.00E+00	ZUP1	3.15E-02	1.00E+00
LOC102391440	3.85E+00	7.29E-11	LOC102403818	-4.18E-02	1.00E+00	PTGFR	9.96E-02	1.00E+00
LOC102397734	2.54E+00	7.31E-11	TAGLN2	8.68E-02	1.00E+00	IGSF3	-4.00E-02	1.00E+00
APCDD1	-2.03E+00	7.35E-11	CLSTN1	-4.92E-02	1.00E+00	APEX2	3.60E-02	1.00E+00
RTN4RL1	2.29E+00	7.47E-11	REXO1	7.15E-02	1.00E+00	SRP54	-2.89E-02	1.00E+00
WWC1	9.23E-01	7.47E-11	AES	-8.19E-02	1.00E+00	FAM214A	-3.75E-02	1.00E+00
RFX3	-1.15E+00	7.54E-11	DGCR8	4.29E-02	1.00E+00	VIRMA	-2.83E-02	1.00E+00
TCP1	-7.10E-01	7.59E-11	ACTR5	-1.32E-01	1.00E+00	CDNF	-6.27E-02	1.00E+00
ACBD6	-1.16E+00	7.72E-11	MCM7	-2.89E-02	1.00E+00	LOC102413515	8.00E-02	1.00E+00
ARMC3	-3.73E+00	7.73E-11	LOC102414482	8.00E-02	1.00E+00	OSCP1	5.33E-02	1.00E+00
KCTD12	-4.30E+00	7.74E-11	EXD2	9.02E-02	1.00E+00	SLC5A10	-6.35E-02	1.00E+00
ENPP5	9.32E-01	7.74E-11	HDC	-1.35E-01	1.00E+00	NAPRT	-3.64E-02	1.00E+00
SCAPER	7.09E-01	7.85E-11	FPGS	-4.94E-02	1.00E+00	CCDC28B	-4.81E-02	1.00E+00
ATP4A	2.15E+00	7.86E-11	DCAF11	-8.85E-02	1.00E+00	HADHA	-3.55E-02	1.00E+00
C1H21orf91	-1.86E+00	7.87E-11	LOC102390188	4.79E-02	1.00E+00	HS2ST1	6.32E-02	1.00E+00
KCNQ3	-2.33E+00	7.93E-11	SERINC2	1.20E-01	1.00E+00	COPE	4.54E-02	1.00E+00
PARG	6.50E-01	7.94E-11	LOC112583909	-1.34E-01	1.00E+00	RPL7A	6.12E-02	1.00E+00
HEPH	-3.05E+00	7.99E-11	CCT4	3.79E-02	1.00E+00	LOC102407761	-3.08E-02	1.00E+00
LOC102406142	-1.57E+00	7.99E-11	ARHGEF15	-1.77E-01	1.00E+00	GPD1L	3.84E-02	1.00E+00
DDX11	7.67E-01	8.10E-11	EIF5A2	7.95E-02	1.00E+00	ARHGAP6	2.94E-02	1.00E+00
UNC80	2.17E+00	8.11E-11	HNF1B	5.32E-02	1.00E+00	LOC112583810	7.18E-02	1.00E+00
GPX4	-1.06E+00	8.35E-11	LOC102410118	9.11E-02	1.00E+00	LOC112585691	7.12E-02	1.00E+00
MTMR8	-9.53E-01	8.48E-11	RUFY3	4.23E-02	1.00E+00	DAB2	5.11E-02	1.00E+00
LOC102408576	-1.61E+00	8.49E-11	LOC112586856	-1.58E-01	1.00E+00	MAD2L2	-4.97E-02	1.00E+00
GATAD1	-1.27E+00	8.56E-11	CD40	-6.40E-02	1.00E+00	EIF2S2	-4.27E-02	1.00E+00
RAB1A	6.33E-01	8.63E-11	ST3GAL3	-5.03E-02	1.00E+00	SART1	-3.17E-02	1.00E+00

LOC112583719	-2.36E+00	8.64E-11	RAMP1	-1.24E-01	1.00E+00	METRN	6.35E-02	1.00E+00
C6H1orf162	-3.92E+00	8.67E-11	ZNF428	-9.01E-02	1.00E+00	MZT1	-4.66E-02	1.00E+00
GFRA2	-1.71E+00	8.76E-11	FAT1	9.25E-02	1.00E+00	ZNF711	-4.77E-02	1.00E+00
ANKRD13D	-1.08E+00	8.77E-11	KLC1	-4.12E-02	1.00E+00	GAB1	-5.63E-02	1.00E+00
ATP6V1A	-8.98E-01	8.80E-11	CDADC1	4.51E-02	1.00E+00	LOC102405334	-7.21E-02	1.00E+00
PCYT1B	9.02E-01	8.83E-11	CNN1	-9.41E-02	1.00E+00	LOC102405323	-7.61E-02	1.00E+00
MTMR4	-8.17E-01	8.90E-11	GNB5	-5.51E-02	1.00E+00	SLC23A2	-3.94E-02	1.00E+00
TAGAP	1.94E+00	8.94E-11	TSPAN3	-3.09E-02	1.00E+00	COL6A1	8.86E-02	1.00E+00
LOC102412044	1.18E+00	8.99E-11	PSMB10	-1.10E-01	1.00E+00	ZNF792	4.35E-02	1.00E+00
OGFOD3	-9.09E-01	9.07E-11	CCDC127	-4.62E-02	1.00E+00	GAREM2	5.43E-02	1.00E+00
PLAUR	1.33E+00	9.17E-11	LRMDA	3.76E-02	1.00E+00	SMIM4	-4.98E-02	1.00E+00
ADGRL3	2.38E+00	9.18E-11	NAF1	-5.12E-02	1.00E+00	C24H16orf72	-4.04E-02	1.00E+00
HIPK1	-9.73E-01	9.20E-11	PPP1CC	-3.28E-02	1.00E+00	SQSTM1	4.15E-02	1.00E+00
LOC102407985	1.77E+00	9.24E-11	GHITM	3.26E-02	1.00E+00	PRAF2	-3.63E-02	1.00E+00
WNK3	1.10E+00	9.37E-11	NR2F2	1.15E-01	1.00E+00	KIAA1147	-7.62E-02	1.00E+00
SAC3D1	-1.52E+00	9.54E-11	INSIG1	-3.67E-02	1.00E+00	WDR34	-4.29E-02	1.00E+00
LOC102394597	-9.88E-01	9.55E-11	ZNF207	2.86E-02	1.00E+00	EMG1	4.39E-02	1.00E+00
PDXK	-6.43E-01	9.58E-11	NUP155	3.63E-02	1.00E+00	PCDHB5	7.38E-02	1.00E+00
CACNA1B	-1.40E+00	9.60E-11	ATPAF1	-4.36E-02	1.00E+00	MIF	5.76E-02	1.00E+00
ZNF473	1.27E+00	9.77E-11	NPTN	-3.67E-02	1.00E+00	TNNI3	6.52E-02	1.00E+00
LOC112587311	-1.57E+00	9.79E-11	LOC112579693	-1.38E-01	1.00E+00	CPT1B	-4.07E-02	1.00E+00
LOC112587638	3.81E+00	9.81E-11	GSS	-8.75E-02	1.00E+00	DUS2	-3.65E-02	1.00E+00
CCDC150	-1.22E+00	1.00E-10	LOC102401659	1.74E-01	1.00E+00	DENND6A	3.73E-02	1.00E+00
AP2M1	-8.50E-01	1.00E-10	MLH1	3.53E-02	1.00E+00	NAA25	-3.77E-02	1.00E+00
LAMB3	1.44E+00	1.00E-10	TIMM10	7.57E-02	1.00E+00	ZNF41	4.47E-02	1.00E+00
ARL10	6.48E-01	1.01E-10	NSG2	-6.24E-02	1.00E+00	APLF	-4.11E-02	1.00E+00
RHBDF1	8.07E-01	1.03E-10	FUBP1	3.52E-02	1.00E+00	ZNF3	3.22E-02	1.00E+00
N4BP3	-2.08E+00	1.04E-10	LAMA4	-1.16E-01	1.00E+00	SULT1C4	-7.64E-02	1.00E+00
LOC102413789	1.74E+00	1.04E-10	OSBPL2	3.80E-02	1.00E+00	INF2	1.08E-01	1.00E+00
NEK11	-1.30E+00	1.04E-10	NFKBIE	3.28E-02	1.00E+00	ZNF331	-3.80E-02	1.00E+00
C6H1orf43	-7.47E-01	1.05E-10	INAFM2	-8.41E-02	1.00E+00	CMTM5	-6.60E-02	1.00E+00
NAT16	-1.70E+00	1.06E-10	INTS7	3.88E-02	1.00E+00	ZNF200	6.30E-02	1.00E+00
SETD1B	9.26E-01	1.06E-10	ZNF263	8.46E-02	1.00E+00	KIAA1614	-2.96E-02	1.00E+00

TRMT61A	1.04E+00	1.07E-10	SPTBN1	-7.05E-02	1.00E+00	DHX15	-3.19E-02	1.00E+00
SCRN1	-1.17E+00	1.09E-10	LOC112587928	-8.23E-02	1.00E+00	EMC9	6.02E-02	1.00E+00
CCNI	-6.23E-01	1.10E-10	LOC102392339	-6.57E-02	1.00E+00	ATXN7	-4.53E-02	1.00E+00
IGSF5	-3.36E+00	1.10E-10	LOC102400086	-1.26E-01	1.00E+00	LENG1	4.93E-02	1.00E+00
NGEF	-3.30E+00	1.10E-10	LOC102391904	1.57E-01	1.00E+00	MAPKAP1	-2.82E-02	1.00E+00
C10H6orf120	7.50E-01	1.11E-10	PYGO1	-8.14E-02	1.00E+00	AUP1	-3.14E-02	1.00E+00
CIAO1	-1.17E+00	1.13E-10	WNT7A	6.99E-02	1.00E+00	LOC112581745	6.98E-02	1.00E+00
PTDSS2	6.97E-01	1.14E-10	NTPCR	3.83E-02	1.00E+00	ZNF699	7.99E-02	1.00E+00
GLG1	-8.84E-01	1.14E-10	CLN8	-4.45E-02	1.00E+00	BRI3	-3.95E-02	1.00E+00
SLC36A4	-1.55E+00	1.14E-10	EIF4A3	-3.27E-02	1.00E+00	PET100	5.57E-02	1.00E+00
REST	-1.48E+00	1.16E-10	MAPKAP1	2.84E-02	1.00E+00	FAM199X	7.39E-02	1.00E+00
UGT8	3.42E+00	1.17E-10	C4H12orf40	7.20E-02	1.00E+00	MARVELD1	-4.07E-02	1.00E+00
EXOC2	5.43E-01	1.17E-10	AKR1A1	4.92E-02	1.00E+00	HLF	-4.24E-02	1.00E+00
NELFB	-7.41E-01	1.18E-10	HTR2A	-1.31E-01	1.00E+00	LOC102409097	4.12E-02	1.00E+00
ERRFI1	-1.35E+00	1.18E-10	LOC102415675	-5.08E-02	1.00E+00	ZPBP	-6.21E-02	1.00E+00
BBS4	7.31E-01	1.18E-10	RGS9	6.53E-02	1.00E+00	COMMD2	3.60E-02	1.00E+00
PCBP1	7.98E-01	1.20E-10	ITPR1	5.18E-02	1.00E+00	TMX4	-3.47E-02	1.00E+00
MGME1	7.86E-01	1.21E-10	ZDHHC8	5.59E-02	1.00E+00	USF2	-3.15E-02	1.00E+00
HSPA12A	1.72E+00	1.24E-10	MAP3K15	3.90E-02	1.00E+00	ATP6V1G1	-2.92E-02	1.00E+00
EHMT2	7.16E-01	1.26E-10	MSL3	3.34E-02	1.00E+00	EBNA1BP2	4.15E-02	1.00E+00
VSIG4	-5.29E+00	1.26E-10	LOC112580365	9.08E-02	1.00E+00	PLD2	-4.49E-02	1.00E+00
C19H5orf51	-7.06E-01	1.27E-10	MOCS3	-4.86E-02	1.00E+00	LOC102398732	7.08E-02	1.00E+00
TMEM245	-1.29E+00	1.29E-10	SLC22A16	-7.78E-02	1.00E+00	TASP1	3.82E-02	1.00E+00
PCNP	-2.62E+00	1.30E-10	LOC102394334	2.89E-01	1.00E+00	FAM98A	2.79E-02	1.00E+00
LOC112581726	-3.26E+00	1.30E-10	EIF4E3	7.33E-02	1.00E+00	SCG5	3.24E-02	1.00E+00
FAM50A	7.68E-01	1.32E-10	LOC102412805	-3.00E-02	1.00E+00	RGS16	-8.98E-02	1.00E+00
LOC112582910	3.95E+00	1.35E-10	RUVBL2	3.37E-02	1.00E+00	SARS2	-3.80E-02	1.00E+00
GNG7	-2.13E+00	1.37E-10	TMEM230	4.26E-02	1.00E+00	INIP	-3.05E-02	1.00E+00
C18H19orf47	7.61E-01	1.37E-10	LOC112580302	1.45E-01	1.00E+00	ITGB1BP2	5.61E-02	1.00E+00
IKZF4	-1.10E+00	1.38E-10	SLC25A11	-3.31E-02	1.00E+00	SWI5	3.72E-02	1.00E+00
USP21	8.36E-01	1.39E-10	LOC102408093	-4.68E-02	1.00E+00	SDCCAG8	3.14E-02	1.00E+00
WNT7B	-3.15E+00	1.46E-10	PPM1A	3.37E-02	1.00E+00	LOC102397215	-5.37E-02	1.00E+00
TRIM63	-3.79E+00	1.50E-10	CCDC117	3.17E-02	1.00E+00	KIF2A	3.11E-02	1.00E+00
LOC102416571	5.75E+00	1.52E-10	PEX11A	4.12E-02	1.00E+00	FBXW7	3.30E-02	1.00E+00

KCNA3	3.17E+00	1.55E-10	ZBTB12	8.73E-02	1.00E+00	FITM1	5.33E-02	1.00E+00
NEGR1	-1.14E+00	1.56E-10	PABPC5	-1.50E-01	1.00E+00	DELE1	-2.84E-02	1.00E+00
TUSC2	1.98E+00	1.58E-10	TNFSF18	5.53E-02	1.00E+00	MRTO4	3.98E-02	1.00E+00
SRSF4	-5.99E-01	1.58E-10	NBDY	1.44E-01	1.00E+00	VAMP7	3.07E-02	1.00E+00
LFNG	-1.36E+00	1.58E-10	PDE6C	-3.23E-02	1.00E+00	DESI2	3.13E-02	1.00E+00
SCUBE3	-3.16E+00	1.62E-10	FANCD2OS	-1.41E-01	1.00E+00	TAP1	3.29E-02	1.00E+00
RNPC3	1.07E+00	1.62E-10	C6H1orf43	3.77E-02	1.00E+00	RNF217	3.66E-02	1.00E+00
LOC112578030	4.50E+00	1.63E-10	TTC39B	8.44E-02	1.00E+00	MOV10	-2.82E-02	1.00E+00
LOC112577712	3.45E+00	1.65E-10	AP4S1	-9.07E-02	1.00E+00	PARK7	4.24E-02	1.00E+00
CALHM2	-2.17E+00	1.66E-10	POLR2E	-3.34E-02	1.00E+00	NCBP2	3.05E-02	1.00E+00
ADCY4	-3.00E+00	1.67E-10	SFT2D2	6.88E-02	1.00E+00	ZNF771	-4.13E-02	1.00E+00
MYL10	2.46E+00	1.69E-10	TTC17	4.23E-02	1.00E+00	FXYD1	4.97E-02	1.00E+00
LOC102398014	3.56E+00	1.69E-10	PRRC1	-4.43E-02	1.00E+00	RAP2B	-3.43E-02	1.00E+00
IGIP	-3.84E+00	1.70E-10	PHC3	3.56E-02	1.00E+00	C20H14orf28	5.62E-02	1.00E+00
PARD6G	-9.26E-01	1.71E-10	RGS12	-4.70E-02	1.00E+00	R3HDM1	-2.92E-02	1.00E+00
PDE4A	1.92E+00	1.74E-10	SPCS2	-4.25E-02	1.00E+00	RIC1	4.48E-02	1.00E+00
NAALADL2	1.15E+00	1.75E-10	GCN1	5.34E-02	1.00E+00	TNFRSF1B	4.66E-02	1.00E+00
ABCF3	7.50E-01	1.77E-10	SENP7	-7.54E-02	1.00E+00	ADNP2	3.74E-02	1.00E+00
HDAC10	-1.06E+00	1.77E-10	ESRP2	4.84E-02	1.00E+00	DEDD	2.73E-02	1.00E+00
GPR68	1.36E+00	1.78E-10	TBPL1	-3.10E-02	1.00E+00	PPP1R3F	4.98E-02	1.00E+00
FAM76B	-1.24E+00	1.78E-10	PAQR5	-3.98E-02	1.00E+00	DNAH11	4.48E-02	1.00E+00
PEX26	-8.84E-01	1.79E-10	MZT2B	-1.05E-01	1.00E+00	LYPLA2	-2.94E-02	1.00E+00
UCP2	-4.61E+00	1.80E-10	IFT81	6.19E-02	1.00E+00	CACNB1	-4.71E-02	1.00E+00
CR2	-4.09E+00	1.81E-10	PRKRA	-4.00E-02	1.00E+00	ANXA6	3.89E-02	1.00E+00
LOC112585337	-4.14E+00	1.81E-10	CALM1	3.11E-02	1.00E+00	PTGES3L	5.55E-02	1.00E+00
LOC112581152	-3.16E+00	1.82E-10	DPM1	4.77E-02	1.00E+00	HYKK	5.96E-02	1.00E+00
IGSF22	3.04E+00	1.84E-10	LOC102389376	5.38E-02	1.00E+00	UPF3A	3.59E-02	1.00E+00
SNX24	7.16E-01	1.84E-10	LOC112579865	5.93E-02	1.00E+00	PRCC	-2.82E-02	1.00E+00
B4GALNT1	2.10E+00	1.84E-10	PIM1	1.37E-01	1.00E+00	AP1S3	6.02E-02	1.00E+00
LOC102411403	1.98E+00	1.84E-10	EPOP	-3.93E-02	1.00E+00	NEMP1	-3.84E-02	1.00E+00
ZNF782	-1.78E+00	1.86E-10	PRKCH	3.80E-02	1.00E+00	PGR	-3.76E-02	1.00E+00
PIKFYVE	-1.42E+00	1.87E-10	NEU3	-7.11E-02	1.00E+00	GPATCH2	3.40E-02	1.00E+00
C5H1orf105	1.76E+00	1.88E-10	ZNF285	1.46E-01	1.00E+00	LOC102399450	-7.04E-02	1.00E+00
FAM180A	3.52E+00	1.88E-10	ZBTB8OS	5.41E-02	1.00E+00	LOC102409480	-6.35E-02	1.00E+00
VRK2	5.99E-01	1.89E-10	TENM3	2.96E-02	1.00E+00	LOC102408728	-6.25E-	1.00E+00

							02	
TMCO3	-8.03E-01	1.92E-10	BCKDHB	4.90E-02	1.00E+00	LOC102394298	-7.24E-02	1.00E+00
PNKD	-1.13E+00	1.92E-10	LOC102400533	1.49E-01	1.00E+00	DYNLL2	-3.08E-02	1.00E+00
CLEC2L	-2.50E+00	1.93E-10	ARF1	3.02E-02	1.00E+00	C6H1orf109	-4.31E-02	1.00E+00
FAM168A	-7.22E-01	1.95E-10	ATG10	1.43E-01	1.00E+00	RRP1B	2.72E-02	1.00E+00
KLHL3	-2.62E+00	1.98E-10	ALDH16A1	-1.11E-01	1.00E+00	NISCH	-2.80E-02	1.00E+00
THAP7	-9.91E-01	1.98E-10	AZI2	5.73E-02	1.00E+00	LOC102410920	-5.04E-02	1.00E+00
NDUFAF8	-1.32E+00	1.99E-10	LCTL	-2.07E-01	1.00E+00	L2HGDH	6.10E-02	1.00E+00
LOC102404153	1.71E+00	1.99E-10	PSMD12	-3.77E-02	1.00E+00	PSMC5	3.85E-02	1.00E+00
LOC102399242	-1.80E+00	2.00E-10	SYT14	8.49E-02	1.00E+00	THEM6	-6.32E-02	1.00E+00
TMEM8A	-1.13E+00	2.01E-10	ZMAT2	3.12E-02	1.00E+00	RAD51B	4.46E-02	1.00E+00
CWC22	-6.33E-01	2.01E-10	LOC102390987	5.89E-02	1.00E+00	C8H7orf25	-3.96E-02	1.00E+00
RPF2	-7.30E-01	2.02E-10	RAC2	-1.96E-01	1.00E+00	BCL7C	4.82E-02	1.00E+00
ADCY9	6.91E-01	2.04E-10	ACER3	4.36E-02	1.00E+00	SNRNP25	4.61E-02	1.00E+00
WDR7	-8.10E-01	2.05E-10	SFI1	-4.94E-02	1.00E+00	CNPY3	-4.30E-02	1.00E+00
PLCG1	-9.58E-01	2.05E-10	IL11	1.21E-01	1.00E+00	LOC102410270	6.74E-02	1.00E+00
SERPINI1	-1.93E+00	2.06E-10	RAB5C	3.12E-02	1.00E+00	RWDD2B	-2.94E-02	1.00E+00
EGFLAM	-3.18E+00	2.08E-10	LOC112580193	2.05E-01	1.00E+00	LOC112583686	-3.40E-02	1.00E+00
FBXL19	7.29E-01	2.09E-10	SYNGR3	1.11E-01	1.00E+00	A4GALT	-4.31E-02	1.00E+00
ELP2	5.73E-01	2.10E-10	OVOL1	-1.97E-01	1.00E+00	ENGASE	-3.77E-02	1.00E+00
CENPM	1.12E+00	2.13E-10	LOC112581879	1.69E-01	1.00E+00	METTL15	3.30E-02	1.00E+00
SMPDL3A	-4.36E+00	2.16E-10	LOXL1	-6.00E-02	1.00E+00	LRRC8A	3.11E-02	1.00E+00
ARPP19	-8.04E-01	2.16E-10	TMEM167B	-6.10E-02	1.00E+00	PEX1	-5.50E-02	1.00E+00
ENTHD1	2.37E+00	2.17E-10	RIF1	5.49E-02	1.00E+00	LRRFIP2	-2.79E-02	1.00E+00
AGER	-4.19E+00	2.22E-10	LAMA1	2.06E-01	1.00E+00	RP2	4.61E-02	1.00E+00
LOC112587901	-2.50E+00	2.27E-10	MERTK	6.62E-02	1.00E+00	AFG1L	-4.08E-02	1.00E+00
RNF2	6.73E-01	2.31E-10	MCUR1	-3.47E-02	1.00E+00	ETS1	5.25E-02	1.00E+00
FBRS	6.36E-01	2.32E-10	LOC112580842	1.00E-01	1.00E+00	DGCR6L	4.64E-02	1.00E+00
DDX41	-7.32E-01	2.35E-10	SEPT7	3.30E-02	1.00E+00	PHF23	2.95E-02	1.00E+00
NKAIN3	-3.04E+00	2.35E-10	ZNF438	5.05E-02	1.00E+00	GAPDHS	3.40E-02	1.00E+00
LOC102401360	-1.83E+00	2.38E-10	EPB41L3	-5.96E-02	1.00E+00	TOMM70	-2.64E-02	1.00E+00
RBM4	7.56E-01	2.38E-10	BBX	-5.70E-02	1.00E+00	SRFBP1	-4.06E-02	1.00E+00
VKORC1	-9.37E-01	2.40E-10	SPATS2L	-1.85E-01	1.00E+00	ZNF382	-4.68E-02	1.00E+00
FKBP4	-	2.41E-10	PTCH1	7.01E-02	1.00E+00	SLC27A5	-6.13E-	1.00E+00

	1.02E+00						02	
CCNDBP1	6.42E-01	2.41E-10	PDLIM7	-5.12E-02	1.00E+00	BICDL1	-3.59E-02	1.00E+00
LOC102390363	-5.95E-01	2.42E-10	MOSMO	-1.62E-01	1.00E+00	THUMPD2	-3.19E-02	1.00E+00
OTC	3.61E+00	2.43E-10	NDUFA10	-4.04E-02	1.00E+00	PRICKLE2	3.96E-02	1.00E+00
LOC102399969	-1.14E+00	2.45E-10	ZNRD1	-6.11E-02	1.00E+00	MYO5C	-6.43E-02	1.00E+00
PSMB1	-9.90E-01	2.47E-10	LOC102392890	3.08E-02	1.00E+00	CCDC134	-3.66E-02	1.00E+00
VPS4A	-6.46E-01	2.47E-10	PUDP	-4.68E-02	1.00E+00	LSM8	3.77E-02	1.00E+00
SRD5A3	8.01E-01	2.48E-10	LOC112580166	-1.14E-01	1.00E+00	HAS1	-7.47E-02	1.00E+00
ZRANB2	-6.77E-01	2.48E-10	GPR61	1.28E-01	1.00E+00	PLBD2	-3.41E-02	1.00E+00
ANKLE2	7.74E-01	2.50E-10	TRAPPC6A	1.47E-01	1.00E+00	LOC102392710	4.51E-02	1.00E+00
SRBD1	5.59E-01	2.50E-10	FBXL19	4.10E-02	1.00E+00	ARMC1	-2.85E-02	1.00E+00
MUC1	-3.64E+00	2.51E-10	GEMIN8	4.04E-02	1.00E+00	NDUFA1	3.67E-02	1.00E+00
INTS10	-6.75E-01	2.53E-10	NIPAL1	1.42E-01	1.00E+00	VEZF1	3.48E-02	1.00E+00
SLC30A4	1.39E+00	2.55E-10	KLHL28	-9.93E-02	1.00E+00	MEAF6	2.80E-02	1.00E+00
LOC112583966	3.39E+00	2.55E-10	LOC102397090	-1.10E-01	1.00E+00	C5AR2	4.72E-02	1.00E+00
PHF6	-8.17E-01	2.56E-10	PSMB2	-4.49E-02	1.00E+00	FANCM	-3.58E-02	1.00E+00
SPIRE1	7.61E-01	2.57E-10	C2CD3	7.11E-02	1.00E+00	CEMIP	-6.96E-02	1.00E+00
BACH1	-1.42E+00	2.61E-10	RGS20	1.27E-01	1.00E+00	ZRANB1	3.11E-02	1.00E+00
MTG2	-9.79E-01	2.62E-10	BFAR	3.28E-02	1.00E+00	TSGA10	-4.62E-02	1.00E+00
LOC112587007	-4.19E+00	2.75E-10	LOC102396111	1.39E-01	1.00E+00	SLC10A1	6.76E-02	1.00E+00
PTPRB	2.29E+00	2.75E-10	LOC112586550	-1.18E-01	1.00E+00	MTMR6	3.34E-02	1.00E+00
LOC112578057	1.85E+00	2.76E-10	RNF167	3.09E-02	1.00E+00	PPCDC	-4.23E-02	1.00E+00
NSUN7	8.40E-01	2.79E-10	ARPC1A	3.21E-02	1.00E+00	GSTCD	4.35E-02	1.00E+00
BAZ1B	6.52E-01	2.81E-10	SYT7	1.24E-01	1.00E+00	EGLN1	2.96E-02	1.00E+00
LRWD1	-8.05E-01	2.83E-10	CRHBP	9.74E-02	1.00E+00	MPDU1	-3.40E-02	1.00E+00
ZNF512	-5.40E-01	2.87E-10	TSEN54	-6.77E-02	1.00E+00	DNAJC12	6.08E-02	1.00E+00
TP53RK	7.32E-01	2.88E-10	NPLOC4	-2.99E-02	1.00E+00	HOOK1	-4.62E-02	1.00E+00
LOC102404636	-1.20E+00	2.89E-10	HNRNPR	-3.86E-02	1.00E+00	LOC102398273	-4.01E-02	1.00E+00
MAP6D1	-1.22E+00	2.90E-10	RBM18	3.09E-02	1.00E+00	TGFBRAP1	-2.96E-02	1.00E+00
NIPAL1	-2.02E+00	2.90E-10	LOC112586251	4.76E-02	1.00E+00	LOC112581825	5.39E-02	1.00E+00
LOC102399011	8.55E-01	2.95E-10	CDC37L1	3.22E-02	1.00E+00	RALGPS1	3.36E-02	1.00E+00
CLCC1	8.40E-01	2.97E-10	LOC102410254	1.59E-01	1.00E+00	LOC102406570	5.60E-02	1.00E+00
ROCK1	7.66E-01	3.01E-10	TWIST1	1.36E-01	1.00E+00	DHX29	-3.05E-02	1.00E+00
WNT5B	-2.57E+00	3.05E-10	ZFYVE21	-3.92E-02	1.00E+00	STXBP5	4.19E-02	1.00E+00

SLC25A40	6.22E-01	3.05E-10	PRKCE	4.36E-02	1.00E+00	LOC102409228	4.07E-02	1.00E+00
CYTB	-1.77E+00	3.06E-10	FAM19A5	-1.69E-01	1.00E+00	SNAPC3	-2.80E-02	1.00E+00
NDUFB2	-7.58E-01	3.10E-10	CYFIP2	3.20E-02	1.00E+00	LOC102392628	-5.79E-02	1.00E+00
KCTD6	-1.01E+00	3.10E-10	NANOS1	-3.61E-02	1.00E+00	AMDHD2	3.63E-02	1.00E+00
COMMD6	-9.68E-01	3.13E-10	LOC102393305	4.46E-02	1.00E+00	PDZD8	3.58E-02	1.00E+00
ZDHHC1	-1.30E+00	3.17E-10	LOC112587556	-1.11E-01	1.00E+00	PRPF4	-2.90E-02	1.00E+00
LOC102414910	7.65E-01	3.18E-10	ANAPC4	3.04E-02	1.00E+00	PRMT1	-4.15E-02	1.00E+00
LOC112580458	-2.86E+00	3.20E-10	CA2	1.08E-01	1.00E+00	CCDC30	3.98E-02	1.00E+00
LENG1	1.14E+00	3.21E-10	KIFC1	3.26E-02	1.00E+00	MALT1	3.11E-02	1.00E+00
LOC102396414	-1.44E+00	3.22E-10	DUSP19	-5.69E-02	1.00E+00	PRELID3B	-2.95E-02	1.00E+00
PTH1R	-3.46E+00	3.23E-10	B4GALT4	-3.42E-02	1.00E+00	STRN3	3.47E-02	1.00E+00
RC3H2	-1.18E+00	3.25E-10	YIPF3	-3.60E-02	1.00E+00	LOC102399864	3.75E-02	1.00E+00
LOC102405595	1.75E+00	3.25E-10	DCP1A	6.39E-02	1.00E+00	PITHD1	3.06E-02	1.00E+00
COPRS	9.75E-01	3.29E-10	IL1R2	-3.78E-02	1.00E+00	CD320	-4.96E-02	1.00E+00
ICAM5	1.62E+00	3.32E-10	LOC112579500	-2.42E-01	1.00E+00	LOC102399590	6.66E-02	1.00E+00
LCT	-1.37E+00	3.33E-10	GGPS1	-4.00E-02	1.00E+00	SLC9A6	-3.95E-02	1.00E+00
POP7	8.97E-01	3.34E-10	BCLAF1	-5.82E-02	1.00E+00	WDR11	-3.31E-02	1.00E+00
SFRP5	-5.87E+00	3.44E-10	IL18BP	1.11E-01	1.00E+00	KPNA7	-9.57E-02	1.00E+00
BFSP2	2.97E+00	3.46E-10	LOC102394597	-3.12E-02	1.00E+00	MTA3	-2.76E-02	1.00E+00
DUS3L	7.73E-01	3.52E-10	COG7	-4.58E-02	1.00E+00	CPNE3	3.47E-02	1.00E+00
LARS	-5.24E-01	3.54E-10	ALKBH8	-5.38E-02	1.00E+00	GNB1	3.21E-02	1.00E+00
LOC102399267	-1.83E+00	3.54E-10	AUP1	1.32E-01	1.00E+00	EIF5B	-2.93E-02	1.00E+00
LOC102397198	-1.38E+00	3.56E-10	THYN1	-3.84E-02	1.00E+00	EPHX1	-4.26E-02	1.00E+00
GRB10	1.11E+00	3.56E-10	SLC35G1	1.10E-01	1.00E+00	MRRF	3.87E-02	1.00E+00
RIOK3	9.20E-01	3.57E-10	ZNF48	2.19E-01	1.00E+00	ERCC3	-2.61E-02	1.00E+00
CCDC63	3.51E+00	3.60E-10	LOC102407618	-9.10E-02	1.00E+00	HSD17B8	4.34E-02	1.00E+00
GAL	-1.69E+00	3.67E-10	JOSD2	7.43E-02	1.00E+00	NDUFC2	-3.45E-02	1.00E+00
SATB2	1.39E+00	3.69E-10	RAB24	-4.95E-02	1.00E+00	LOC112579940	6.81E-02	1.00E+00
HCRTR1	-3.37E+00	3.75E-10	AMN	-1.00E-01	1.00E+00	KIF21A	4.11E-02	1.00E+00
AMDHD1	2.35E+00	3.77E-10	RNF10	3.31E-02	1.00E+00	FLOT1	3.04E-02	1.00E+00
FOXJ2	-7.33E-01	3.78E-10	KIF4A	-3.44E-02	1.00E+00	TNFAIP8	5.38E-02	1.00E+00
PGP	-9.20E-01	3.78E-10	BCAP31	-4.16E-02	1.00E+00	SPINT1	-7.18E-02	1.00E+00
NR4A3	-4.41E+00	3.78E-10	LOC102395271	-2.48E-01	1.00E+00	CYS1	-3.03E-02	1.00E+00
PSMF1	-6.05E-01	3.81E-10	ING4	-3.70E-02	1.00E+00	EDA	3.74E-02	1.00E+00

AP2S1	-1.02E+00	3.82E-10	LOC112578903	-6.02E-02	1.00E+00	LOC102408576	-5.58E-02	1.00E+00
UBA5	-7.16E-01	3.84E-10	SLC3A2	2.85E-02	1.00E+00	FAM227B	-4.74E-02	1.00E+00
ZW10	6.48E-01	3.86E-10	GOLIM4	-3.73E-02	1.00E+00	LOC102404498	-6.36E-02	1.00E+00
DOT1L	-9.07E-01	3.88E-10	AK8	-5.80E-02	1.00E+00	EIF5A	3.70E-02	1.00E+00
MIER3	-9.01E-01	3.89E-10	PCYOX1	7.87E-02	1.00E+00	ZFAND6	-2.86E-02	1.00E+00
EN1	3.09E+00	3.91E-10	TRIP10	-1.44E-01	1.00E+00	CDK5RAP3	-3.51E-02	1.00E+00
PCNX2	2.16E+00	3.92E-10	LOC102390765	-1.02E-01	1.00E+00	LNPEP	-3.41E-02	1.00E+00
LOC102412560	4.19E+00	3.92E-10	DMTF1	3.11E-02	1.00E+00	DRAP1	3.92E-02	1.00E+00
TMEM102	-1.77E+00	3.96E-10	POLR3H	3.59E-02	1.00E+00	WRAP53	-3.05E-02	1.00E+00
LARP4	-7.99E-01	3.98E-10	TOM1L1	8.89E-02	1.00E+00	LOC102389800	-4.05E-02	1.00E+00
TTBK1	1.55E+00	3.98E-10	PDE2A	1.00E-01	1.00E+00	SASS6	-3.75E-02	1.00E+00
PRCP	-8.15E-01	4.03E-10	SULT1C4	1.98E-01	1.00E+00	PIK3AP1	7.55E-02	1.00E+00
LOC102403347	1.94E+00	4.03E-10	SLC35F4	-7.28E-02	1.00E+00	PSMC1	3.44E-02	1.00E+00
RHOT1	6.02E-01	4.05E-10	LOC102389646	-6.02E-02	1.00E+00	RPS16	-4.05E-02	1.00E+00
CCR5	3.43E+00	4.06E-10	LOC102402779	2.17E-01	1.00E+00	SLC2A6	-9.32E-02	1.00E+00
LRCH1	8.31E-01	4.13E-10	ICOS	-6.24E-02	1.00E+00	STX1A	4.66E-02	1.00E+00
SEC61B	-1.16E+00	4.13E-10	RPL7L1	-3.01E-02	1.00E+00	TPD52L2	-3.50E-02	1.00E+00
NBEAL1	-1.44E+00	4.13E-10	ZNF771	-5.19E-02	1.00E+00	FAM189A2	-3.90E-02	1.00E+00
ACSL4	-7.04E-01	4.14E-10	PCSK5	4.23E-02	1.00E+00	ZAN	-5.86E-02	1.00E+00
TYROBP	-5.30E+00	4.15E-10	KIF18A	3.10E-02	1.00E+00	DLD	2.73E-02	1.00E+00
MOB3B	-1.57E+00	4.16E-10	MYCBP	7.40E-02	1.00E+00	GEMIN8	-3.42E-02	1.00E+00
PKNOX2	1.65E+00	4.16E-10	S1PR2	-1.29E-01	1.00E+00	SCMH1	2.67E-02	1.00E+00
LOC102411351	-2.96E+00	4.16E-10	FAIM	-7.90E-02	1.00E+00	LOC112579707	5.58E-02	1.00E+00
KBTBD2	1.04E+00	4.22E-10	BAG3	-4.68E-02	1.00E+00	SDHD	2.70E-02	1.00E+00
SEC24C	-5.28E-01	4.25E-10	SERHL2	9.97E-02	1.00E+00	LOC102410092	1.31E-01	1.00E+00
TTLL12	-7.41E-01	4.28E-10	LOC112578484	-1.17E-01	1.00E+00	GOLGA3	-3.72E-02	1.00E+00
CNPY2	9.39E-01	4.36E-10	CIDEC	8.79E-02	1.00E+00	LSG1	-2.94E-02	1.00E+00
ZNF106	8.00E-01	4.36E-10	EPHB4	-1.60E-01	1.00E+00	DAGLA	5.91E-02	1.00E+00
PWP2	6.46E-01	4.41E-10	SLC41A2	4.47E-02	1.00E+00	STXBP4	3.22E-02	1.00E+00
INPP5A	-1.12E+00	4.41E-10	PPIE	-6.09E-02	1.00E+00	PRPF6	-2.96E-02	1.00E+00
LOC112578762	2.75E+00	4.44E-10	CCDC125	1.66E-01	1.00E+00	C12H2orf42	-4.29E-02	1.00E+00
NMNAT1	6.73E-01	4.62E-10	PRR36	-5.75E-02	1.00E+00	EGLN2	2.87E-02	1.00E+00
RSPO3	3.69E+00	4.77E-10	PRRG1	7.98E-02	1.00E+00	ATG3	-2.54E-02	1.00E+00

PARP1	6.63E-01	4.78E-10	CCDC166	-1.62E-01	1.00E+00	CEACAM1	-1.13E-01	1.00E+00
PMEL	1.34E+00	4.80E-10	NUDT5	4.91E-02	1.00E+00	NAGPA	-3.17E-02	1.00E+00
ZFPL1	6.98E-01	4.81E-10	LOC102389034	-1.27E-01	1.00E+00	SEL1L	-3.60E-02	1.00E+00
KCNA2	1.99E+00	4.81E-10	TPRG1L	-3.42E-02	1.00E+00	INPP5F	-3.81E-02	1.00E+00
LOC112586943	3.69E+00	4.88E-10	LOC102404027	1.49E-01	1.00E+00	TNKS	3.53E-02	1.00E+00
LOC102395698	-7.22E-01	4.88E-10	DDX21	4.39E-02	1.00E+00	PPWD1	3.09E-02	1.00E+00
ago-03	-1.32E+00	4.91E-10	ARHGAP26	-4.18E-02	1.00E+00	LOC102399857	-8.28E-02	1.00E+00
SLC39A3	-1.27E+00	4.94E-10	LOC102399344	3.88E-02	1.00E+00	ZNF32	3.79E-02	1.00E+00
LOC102400860	-3.95E+00	4.95E-10	NUFIP2	5.11E-02	1.00E+00	IKBKG	-2.86E-02	1.00E+00
RAB11FIP5	7.15E-01	4.96E-10	ITPKB	1.22E-01	1.00E+00	USP46	2.66E-02	1.00E+00
CAPN14	3.78E+00	4.97E-10	TMEM265	7.78E-02	1.00E+00	LOC102404490	-3.38E-02	1.00E+00
FPGT	-1.47E+00	5.00E-10	LOC102415281	-1.93E-01	1.00E+00	AMER1	-3.63E-02	1.00E+00
TRAF4	8.17E-01	5.03E-10	DHTKD1	-5.71E-02	1.00E+00	REEP3	-3.68E-02	1.00E+00
MREG	-3.25E+00	5.04E-10	LOC112579555	1.12E-01	1.00E+00	VPS26B	2.92E-02	1.00E+00
LOC102416057	-5.52E+00	5.05E-10	CRIM1	-4.23E-02	1.00E+00	ENKUR	4.65E-02	1.00E+00
NABP2	-9.19E-01	5.07E-10	RBL1	1.06E-01	1.00E+00	NIP7	-4.92E-02	1.00E+00
MRPS6	-9.42E-01	5.14E-10	GLB1	1.10E-01	1.00E+00	PIK3C2A	-4.01E-02	1.00E+00
LOC112585704	1.86E+00	5.17E-10	RNFT2	3.88E-02	1.00E+00	THAP7	-3.70E-02	1.00E+00
LOC102409467	-5.61E+00	5.18E-10	ABLIM2	1.21E-01	1.00E+00	C1R	-8.35E-02	1.00E+00
NXNL2	-3.97E+00	5.21E-10	PPP4R3A	-3.86E-02	1.00E+00	SCLT1	-4.85E-02	1.00E+00
ADGRE5	8.65E-01	5.21E-10	RABGAP1L	3.00E-02	1.00E+00	PPP1R12C	3.12E-02	1.00E+00
NPHS1	3.19E+00	5.22E-10	STEAP2	-6.49E-02	1.00E+00	ND4L	4.66E-02	1.00E+00
HIP1R	7.09E-01	5.28E-10	BUB1	3.63E-02	1.00E+00	PWP1	2.55E-02	1.00E+00
AMMECR1	-1.06E+00	5.29E-10	LOC102408976	3.74E-02	1.00E+00	LOC102411787	-5.70E-02	1.00E+00
PRMT5	6.29E-01	5.47E-10	CCT8	-3.12E-02	1.00E+00	PPP1R13L	-3.26E-02	1.00E+00
LOC112580847	3.59E+00	5.56E-10	ACADL	-2.25E-01	1.00E+00	LOC112578770	-5.08E-02	1.00E+00
ALDH3B1	-1.94E+00	5.61E-10	SERF2	-5.09E-02	1.00E+00	EXOC7	-2.71E-02	1.00E+00
DDO	1.65E+00	5.64E-10	CCNB2	3.62E-02	1.00E+00	SLC25A20	-3.31E-02	1.00E+00
LOC102393534	2.35E+00	5.64E-10	LOC102402736	4.00E-02	1.00E+00	PI4K2B	-4.40E-02	1.00E+00
ZNF275	-9.62E-01	5.65E-10	FTH1	1.09E-01	1.00E+00	C24H16orf91	3.48E-02	1.00E+00
VSTM2L	1.70E+00	5.66E-10	LOC102403599	-1.16E-01	1.00E+00	ALKBH2	3.80E-02	1.00E+00
IQUB	-2.15E+00	5.67E-10	DDX11	-3.84E-02	1.00E+00	FAM161B	-5.55E-02	1.00E+00
LOC102404764	1.38E+00	5.72E-10	TFCP2	-5.76E-02	1.00E+00	MARCH10	3.39E-02	1.00E+00
TBC1D16	-	5.73E-10	CLCN3	-4.15E-	1.00E+00	SYT2	-6.18E-	1.00E+00

	1.02E+00			02			02	
ARHGEF12	-9.43E-01	5.78E-10	MYO5B	2.98E-02	1.00E+00	POC1B	-3.22E-02	1.00E+00
LAMA4	2.00E+00	5.81E-10	RAB13	1.05E-01	1.00E+00	RDM1	-6.73E-02	1.00E+00
CCDC97	9.41E-01	5.84E-10	XPNPEP3	7.21E-02	1.00E+00	LOC102401786	2.54E-02	1.00E+00
CPSF2	6.22E-01	5.91E-10	LOC112579996	-7.34E-02	1.00E+00	PLGRKT	-4.31E-02	1.00E+00
SLC22A4	2.48E+00	5.96E-10	NDE1	-6.81E-02	1.00E+00	AP1B1	2.68E-02	1.00E+00
KCNH8	2.88E+00	6.18E-10	LOC112582213	-1.43E-01	1.00E+00	WDR6	-2.59E-02	1.00E+00
GPATCH3	7.28E-01	6.20E-10	PHF24	-4.99E-02	1.00E+00	TIMM29	-3.14E-02	1.00E+00
PLXNC1	1.11E+00	6.37E-10	SKA1	3.32E-02	1.00E+00	LOC112583596	-5.80E-02	1.00E+00
NECTIN3	6.30E-01	6.40E-10	PPWD1	4.60E-02	1.00E+00	XPC	-2.71E-02	1.00E+00
SDS	-5.01E+00	6.42E-10	MED27	-3.12E-02	1.00E+00	HEBP1	3.65E-02	1.00E+00
FRG1	7.95E-01	6.43E-10	ZNF609	5.91E-02	1.00E+00	TMEM8B	-3.29E-02	1.00E+00
ATP2A3	-3.37E+00	6.49E-10	LOC102399541	-6.47E-02	1.00E+00	ZMAT2	2.72E-02	1.00E+00
TEX49	-1.73E+00	6.54E-10	HADHB	-2.96E-02	1.00E+00	STAT2	3.69E-02	1.00E+00
NPDC1	1.40E+00	6.56E-10	PAGR1	4.11E-02	1.00E+00	CDYL	-3.05E-02	1.00E+00
TSHR	3.56E+00	6.73E-10	LTV1	-2.79E-02	1.00E+00	FOXO4	3.63E-02	1.00E+00
FAM174A	-8.41E-01	6.78E-10	LOC102401468	-7.31E-02	1.00E+00	NDUFA2	-3.75E-02	1.00E+00
JADE1	-9.53E-01	6.82E-10	MRPL28	5.02E-02	1.00E+00	SLAIN2	2.91E-02	1.00E+00
UTP3	-8.44E-01	6.92E-10	TTC21B	-3.27E-02	1.00E+00	PBRM1	-4.94E-02	1.00E+00
SUSD4	1.07E+00	6.99E-10	PARP16	3.38E-02	1.00E+00	C1QBP	-3.55E-02	1.00E+00
LOC102394187	1.07E+00	7.02E-10	CD200	2.70E-02	1.00E+00	IQANK1	5.01E-02	1.00E+00
FAR2	-1.95E+00	7.16E-10	LRRC8E	3.81E-02	1.00E+00	RFTN1	4.37E-02	1.00E+00
GUCY2F	4.24E+00	7.22E-10	NBL1	1.60E-01	1.00E+00	ARMC5	3.28E-02	1.00E+00
SOWAHC	-2.86E+00	7.23E-10	TRAPPC4	-4.30E-02	1.00E+00	LOC102405233	5.25E-02	1.00E+00
NCOA1	9.18E-01	7.23E-10	COQ2	4.98E-02	1.00E+00	SIK2	4.32E-02	1.00E+00
CNNM4	7.96E-01	7.34E-10	RPL27A	-1.15E-01	1.00E+00	SLC25A43	4.08E-02	1.00E+00
LOC112585754	2.18E+00	7.35E-10	PICK1	-4.87E-02	1.00E+00	CEP250	3.27E-02	1.00E+00
LOC102402769	1.11E+00	7.41E-10	IPO4	5.08E-02	1.00E+00	TTC21B	2.94E-02	1.00E+00
ANKRD33B	-1.61E+00	7.47E-10	LHX9	1.25E-01	1.00E+00	USP3	2.83E-02	1.00E+00
LOC102405552	-2.44E+00	7.49E-10	DPP6	-3.95E-02	1.00E+00	OSBP	-2.89E-02	1.00E+00
TRMT13	-1.60E+00	7.52E-10	LETMD1	-3.85E-02	1.00E+00	PTPN11	4.19E-02	1.00E+00
ZNF165	1.20E+00	7.53E-10	SALL3	5.74E-02	1.00E+00	SETD1B	-3.39E-02	1.00E+00
CAB39	6.05E-01	7.62E-10	YBX1	-9.40E-02	1.00E+00	MRPL23	-5.12E-02	1.00E+00
BEND3	-1.83E+00	7.69E-10	MRE11	-3.77E-02	1.00E+00	ULK4	3.17E-02	1.00E+00

PIANP	3.47E+00	7.69E-10	AUH	-4.26E-02	1.00E+00	SF3B2	-3.12E-02	1.00E+00
PRRT1	-2.81E+00	7.76E-10	FBXO3	-3.48E-02	1.00E+00	C17H12orf76	-3.97E-02	1.00E+00
UBE2C	-9.95E-01	7.79E-10	NSUN5	5.17E-02	1.00E+00	RBM22	-2.63E-02	1.00E+00
KCNJ2	-2.49E+00	7.88E-10	INTU	9.20E-02	1.00E+00	ALG9	-2.80E-02	1.00E+00
ALG5	-9.84E-01	7.89E-10	GRIK2	4.11E-02	1.00E+00	STX7	3.14E-02	1.00E+00
NCBP2	-6.66E-01	7.97E-10	PPP3CC	6.67E-02	1.00E+00	TWF2	3.33E-02	1.00E+00
LOC102393741	2.91E+00	8.01E-10	LOC102411578	-7.24E-02	1.00E+00	EEF1B2	-4.53E-02	1.00E+00
ATM	-9.30E-01	8.14E-10	THSD1	-4.40E-02	1.00E+00	SPSB3	-2.69E-02	1.00E+00
TSPAN3	-6.24E-01	8.35E-10	MGAM2	7.69E-02	1.00E+00	ITSN1	3.16E-02	1.00E+00
SVBP	-1.13E+00	8.39E-10	EFNB2	-8.65E-02	1.00E+00	MAP1A	3.36E-02	1.00E+00
MAFB	-3.79E+00	8.41E-10	TMEM164	-5.82E-02	1.00E+00	TSN	-2.67E-02	1.00E+00
MECOM	3.05E+00	8.41E-10	DTYMK	-4.45E-02	1.00E+00	ARHGAP5	-3.98E-02	1.00E+00
PPIL2	-7.11E-01	8.49E-10	HECTD3	-4.43E-02	1.00E+00	SNRPC	-3.60E-02	1.00E+00
LOC102398252	-3.25E+00	8.55E-10	IL18R1	-1.48E-01	1.00E+00	PIK3R1	3.43E-02	1.00E+00
LOC102392363	-2.27E+00	8.57E-10	PIP5K1A	-1.10E-01	1.00E+00	ZNF740	-3.38E-02	1.00E+00
NFYC	6.44E-01	8.61E-10	C17H12orf76	4.53E-02	1.00E+00	LOC112581142	5.84E-02	1.00E+00
COIL	7.07E-01	8.63E-10	GPT	1.79E-01	1.00E+00	KIRREL3	4.53E-02	1.00E+00
PDCD5	9.91E-01	8.67E-10	CENPK	-5.49E-02	1.00E+00	LOC102413798	-3.30E-02	1.00E+00
RIPOR2	2.34E+00	8.74E-10	LOC102392736	-2.84E-02	1.00E+00	LOC112581447	4.86E-02	1.00E+00
STX5	-6.81E-01	8.77E-10	LOC102403241	-3.77E-02	1.00E+00	IBA57	3.61E-02	1.00E+00
LOC112578167	2.31E+00	8.82E-10	NDUFB5	-4.74E-02	1.00E+00	MRPL49	2.85E-02	1.00E+00
LOC102397346	-2.21E+00	8.97E-10	LOC102394044	2.04E-01	1.00E+00	ATG2B	3.70E-02	1.00E+00
TRMT10A	8.76E-01	9.05E-10	CAP2	1.57E-01	1.00E+00	UBXN7	3.06E-02	1.00E+00
BTK	2.63E+00	9.07E-10	LOC112585691	1.42E-01	1.00E+00	PTK2B	-1.26E-01	1.00E+00
SLC52A3	-2.66E+00	9.10E-10	DKK1	1.23E-01	1.00E+00	COG6	4.04E-02	1.00E+00
ALG14	8.14E-01	9.12E-10	ZNF300	7.44E-02	1.00E+00	TUT7	3.68E-02	1.00E+00
PHC1	6.93E-01	9.14E-10	TMEM62	6.46E-02	1.00E+00	CISH	-5.76E-02	1.00E+00
TMCO6	5.90E-01	9.25E-10	LOC112587477	-1.64E-01	1.00E+00	SFXN1	-4.43E-02	1.00E+00
PPM1G	7.67E-01	9.37E-10	IDH3A	-4.54E-02	1.00E+00	MAP4K4	2.84E-02	1.00E+00
ACBD4	-1.12E+00	9.41E-10	ZNF135	5.85E-02	1.00E+00	STXBP3	-3.46E-02	1.00E+00
LOC102409360	6.82E-01	9.49E-10	HAUS4	4.07E-02	1.00E+00	MAX	2.53E-02	1.00E+00
ELF4	1.43E+00	9.64E-10	LOC102400274	1.17E-01	1.00E+00	SDF4	-3.60E-02	1.00E+00
NACAD	1.44E+00	9.67E-10	TENT5B	3.43E-02	1.00E+00	ARHGAP17	2.58E-02	1.00E+00
KLK11	-2.61E+00	9.72E-10	IFT43	7.74E-02	1.00E+00	DAZAP2	-2.58E-02	1.00E+00

LHFPL6	-2.23E+00	9.75E-10	FAM98C	6.01E-02	1.00E+00	INKA1	-6.95E-02	1.00E+00
CARF	-1.44E+00	9.81E-10	CXXC1	4.26E-02	1.00E+00	GDI1	2.84E-02	1.00E+00
LOC112583668	-2.37E+00	9.86E-10	CFLAR	8.22E-02	1.00E+00	RRP12	-2.60E-02	1.00E+00
CLASP2	-7.27E-01	9.91E-10	WDYHV1	2.93E-02	1.00E+00	THRA	-2.73E-02	1.00E+00
ND1	-1.51E+00	9.93E-10	LOC102392068	8.38E-02	1.00E+00	SMIM13	4.66E-02	1.00E+00
EAF2	2.16E+00	1.02E-09	CARD9	1.86E-01	1.00E+00	PLEKHA8	-3.02E-02	1.00E+00
PRSS23	1.73E+00	1.02E-09	NIT2	-6.75E-02	1.00E+00	ARMH3	2.71E-02	1.00E+00
CDR2L	-1.02E+00	1.02E-09	DGKG	-4.09E-02	1.00E+00	CCDC92	-3.55E-02	1.00E+00
FAM135B	3.32E+00	1.02E-09	CISD3	-1.37E-01	1.00E+00	RNF122	6.21E-02	1.00E+00
DNAJC1	6.96E-01	1.04E-09	ZNF232	-1.12E-01	1.00E+00	TBCC	-2.83E-02	1.00E+00
LPCAT3	-8.92E-01	1.04E-09	SLC46A3	-1.55E-01	1.00E+00	PARP11	3.12E-02	1.00E+00
GRM8	2.85E+00	1.04E-09	CXHXorf36	-9.44E-02	1.00E+00	KMT5A	-2.56E-02	1.00E+00
LOC102390996	2.97E+00	1.04E-09	ZFP62	-1.20E-01	1.00E+00	HAGHL	4.27E-02	1.00E+00
MRPL33	-8.62E-01	1.04E-09	TASP1	-5.47E-02	1.00E+00	BPNT1	2.79E-02	1.00E+00
LOC112582347	2.76E+00	1.05E-09	LOC112584418	-4.11E-02	1.00E+00	SIRPA	8.87E-02	1.00E+00
PECAM1	-3.28E+00	1.05E-09	RXYLT1	-1.19E-01	1.00E+00	UBE2M	-3.12E-02	1.00E+00
YAE1	8.12E-01	1.06E-09	MAPRE3	-3.92E-02	1.00E+00	LOC102389530	5.57E-02	1.00E+00
TTLL8	4.62E+00	1.06E-09	INPP5B	-5.02E-02	1.00E+00	SLC47A1	-3.36E-02	1.00E+00
MAIP1	6.96E-01	1.06E-09	BRAF	3.78E-02	1.00E+00	AADAT	-3.22E-02	1.00E+00
VPS13B	6.42E-01	1.06E-09	RBM15B	4.81E-02	1.00E+00	LOC102414277	5.00E-02	1.00E+00
CHST10	-7.45E-01	1.06E-09	SDHAF1	-5.52E-02	1.00E+00	LOC102415442	3.04E-02	1.00E+00
CCDC9B	1.54E+00	1.07E-09	ACSS2	8.10E-02	1.00E+00	TMEM80	-5.10E-02	1.00E+00
FUT11	-2.95E+00	1.09E-09	WDR83OS	-4.76E-02	1.00E+00	AAMP	2.76E-02	1.00E+00
MUT	-5.99E-01	1.09E-09	CXCL9	8.97E-02	1.00E+00	CRADD	3.28E-02	1.00E+00
LOC102416515	-1.77E+00	1.10E-09	AHSA2P	-5.29E-02	1.00E+00	DTX4	7.88E-02	1.00E+00
CDH5	1.99E+00	1.10E-09	LOC102403983	-3.65E-02	1.00E+00	SS18L1	-6.44E-02	1.00E+00
OCRL	6.20E-01	1.10E-09	SYPL2	-2.15E-01	1.00E+00	ULK2	-3.27E-02	1.00E+00
RBFOX1	2.77E+00	1.11E-09	CHMP1A	3.27E-02	1.00E+00	HUNK	-2.79E-02	1.00E+00
PCED1A	-9.64E-01	1.11E-09	FANCI	4.02E-02	1.00E+00	CMTM6	-2.62E-02	1.00E+00
PFKFB1	2.70E+00	1.12E-09	LOC112579116	-6.58E-02	1.00E+00	IFT46	-3.07E-02	1.00E+00
WDR82	-5.45E-01	1.13E-09	ZKSCAN5	3.85E-02	1.00E+00	SREK1IP1	-3.34E-02	1.00E+00
THOC6	9.00E-01	1.13E-09	TRIM45	6.60E-02	1.00E+00	DBNL	-2.62E-02	1.00E+00
TCFL5	-	1.14E-09	ULK4	-3.34E-	1.00E+00	ROBO1	3.48E-02	1.00E+00

	1.29E+00			02				
DGKB	3.40E+00	1.15E-09	WAPL	4.62E-02	1.00E+00	MCM3AP	-2.35E-02	1.00E+00
USP34	8.56E-01	1.15E-09	HMGN1	-5.49E-02	1.00E+00	MPC2	3.23E-02	1.00E+00
PHLDA2	2.29E+00	1.18E-09	NDUFS4	-4.83E-02	1.00E+00	LHCGR	-1.06E-01	1.00E+00
KBTBD3	8.54E-01	1.18E-09	RARS2	3.31E-02	1.00E+00	ICAM3	-4.89E-02	1.00E+00
PHYH	-8.78E-01	1.18E-09	PARP9	-1.29E-01	1.00E+00	LRRCC1	-2.45E-02	1.00E+00
PHAX	5.77E-01	1.21E-09	NYX	-8.73E-02	1.00E+00	PDE4DIP	-2.64E-02	1.00E+00
ZFHX4	3.19E+00	1.21E-09	LRRC43	-1.53E-01	1.00E+00	PTPN18	4.09E-02	1.00E+00
DEDD2	-7.67E-01	1.21E-09	GAPDH	-4.36E-02	1.00E+00	RBM41	3.70E-02	1.00E+00
C5H1orf74	9.60E-01	1.25E-09	NFE2L1	3.58E-02	1.00E+00	NDUFB5	3.20E-02	1.00E+00
HAVCR2	1.26E+00	1.26E-09	PRR15	2.30E-01	1.00E+00	COQ7	3.61E-02	1.00E+00
TSR2	7.67E-01	1.26E-09	BRIP1	-3.28E-02	1.00E+00	ADAMDEC1	6.94E-02	1.00E+00
RARRES1	1.42E+00	1.27E-09	PHF7	-6.21E-02	1.00E+00	FBXL18	2.93E-02	1.00E+00
LOC112581200	-2.48E+00	1.27E-09	RPLP1	6.70E-02	1.00E+00	TLK1	-2.55E-02	1.00E+00
NPEPL1	6.81E-01	1.27E-09	VPS18	-3.85E-02	1.00E+00	BEX5	5.68E-02	1.00E+00
ATP8B3	-2.35E+00	1.27E-09	NASP	3.14E-02	1.00E+00	SLC25A46	-2.83E-02	1.00E+00
LOC102415948	-2.03E+00	1.28E-09	SCAMP4	3.51E-02	1.00E+00	PIK3C3	2.72E-02	1.00E+00
EIF3M	-7.65E-01	1.29E-09	MNAT1	-3.98E-02	1.00E+00	DYNLRB2	5.22E-02	1.00E+00
ERAS	-1.54E+00	1.29E-09	KIAA1324L	4.27E-02	1.00E+00	PPP2R5E	2.81E-02	1.00E+00
SYT10	4.04E+00	1.31E-09	ABHD17B	3.69E-02	1.00E+00	PIH1D1	-3.52E-02	1.00E+00
PDZD11	-8.68E-01	1.32E-09	LOC102391647	3.64E-02	1.00E+00	VPS25	-3.36E-02	1.00E+00
LOC112586936	3.80E+00	1.33E-09	SLC35B1	3.09E-02	1.00E+00	KIF16B	2.98E-02	1.00E+00
LOC102415483	2.72E+00	1.33E-09	CELSR2	5.00E-02	1.00E+00	ARHGAP30	1.02E-01	1.00E+00
PIK3C2G	1.86E+00	1.33E-09	ACER1	1.12E-01	1.00E+00	TLCD2	7.10E-02	1.00E+00
GNB1	-6.67E-01	1.34E-09	RNPS1	2.85E-02	1.00E+00	GFRA2	-5.59E-02	1.00E+00
RECQL	-9.69E-01	1.35E-09	NOP53	4.22E-02	1.00E+00	SMAD6	5.37E-02	1.00E+00
YWHAB	-6.04E-01	1.36E-09	SLC7A3	-3.98E-02	1.00E+00	DYDC1	6.44E-02	1.00E+00
SCMH1	-6.09E-01	1.37E-09	SEMA5A	-2.24E-01	1.00E+00	RPL11	4.94E-02	1.00E+00
LOC102406450	-2.81E+00	1.37E-09	LOC102398867	-6.50E-02	1.00E+00	KLHL31	2.74E-02	1.00E+00
RNF130	-6.74E-01	1.37E-09	HSD17B12	3.17E-02	1.00E+00	NCOA4	2.96E-02	1.00E+00
COL25A1	2.64E+00	1.38E-09	DDX42	-3.43E-02	1.00E+00	CPOX	3.01E-02	1.00E+00
ABCB10	9.41E-01	1.38E-09	KCNC2	-1.10E-01	1.00E+00	LOC102396414	-4.19E-02	1.00E+00
DGCR6L	-1.06E+00	1.40E-09	MTMR11	4.69E-02	1.00E+00	CDC42	2.84E-02	1.00E+00
SAMM50	-7.41E-01	1.40E-09	EMD	3.61E-02	1.00E+00	C9H5orf63	4.69E-02	1.00E+00

FGF13	-2.97E+00	1.40E-09	LOC102407231	-1.74E-01	1.00E+00	TRIM3	-2.82E-02	1.00E+00
ATP1A3	1.96E+00	1.43E-09	GLT8D1	4.14E-02	1.00E+00	SPTLC3	6.24E-02	1.00E+00
SRL	-2.76E+00	1.44E-09	METTL24	9.87E-02	1.00E+00	KPNA6	-2.29E-02	1.00E+00
GDF10	-3.90E+00	1.46E-09	STXBP4	-7.27E-02	1.00E+00	CDKAL1	2.64E-02	1.00E+00
SFT2D1	-6.46E-01	1.47E-09	SLC9A3R1	3.93E-02	1.00E+00	MAP4K1	5.30E-02	1.00E+00
ZNF835	-1.26E+00	1.47E-09	NIPAL3	7.80E-02	1.00E+00	CCDC106	3.17E-02	1.00E+00
CCAR1	-5.73E-01	1.47E-09	UGGT1	6.75E-02	1.00E+00	LOC102408435	2.98E-02	1.00E+00
GSDMD	-4.84E+00	1.47E-09	WDR81	5.79E-02	1.00E+00	BMP2	-6.53E-02	1.00E+00
LOC112586836	3.17E+00	1.48E-09	LOC102403160	-3.25E-02	1.00E+00	PSMD6	-2.44E-02	1.00E+00
MRPL42	7.43E-01	1.49E-09	TMEM129	6.61E-02	1.00E+00	IAH1	-3.07E-02	1.00E+00
LOC102400645	2.21E+00	1.50E-09	WT1	1.67E-01	1.00E+00	LOC102393350	-3.57E-02	1.00E+00
LOC102406878	2.02E+00	1.53E-09	LOC102391462	1.86E-01	1.00E+00	FBXO45	5.00E-02	1.00E+00
SNURF	-1.42E+00	1.53E-09	GIT2	3.38E-02	1.00E+00	CCDC107	-4.36E-02	1.00E+00
ROMO1	-1.09E+00	1.54E-09	CNTNAP1	1.22E-01	1.00E+00	CTNNAL1	-2.37E-02	1.00E+00
LOC102415736	1.20E+00	1.54E-09	RALGAPA2	-5.28E-02	1.00E+00	NAAA	-6.55E-02	1.00E+00
LRRC8B	1.01E+00	1.55E-09	CMYA5	4.80E-02	1.00E+00	TSR3	-3.58E-02	1.00E+00
OTOF	2.11E+00	1.56E-09	LOC102416114	7.99E-02	1.00E+00	ABHD16A	-2.58E-02	1.00E+00
KLHDC2	-4.85E-01	1.58E-09	LOC112585503	4.99E-02	1.00E+00	RNF167	-2.87E-02	1.00E+00
LOC102416352	-8.64E-01	1.58E-09	GPX1	4.88E-02	1.00E+00	ATN1	-2.80E-02	1.00E+00
LOC102409297	3.71E+00	1.59E-09	NHSL1	4.61E-02	1.00E+00	POLR1E	2.73E-02	1.00E+00
DHX8	-5.91E-01	1.61E-09	TTC3	-4.28E-02	1.00E+00	EIF4G2	4.06E-02	1.00E+00
JAK2	-1.11E+00	1.62E-09	C16H11orf1	1.04E-01	1.00E+00	LEPROTL1	2.34E-02	1.00E+00
YIPF4	1.12E+00	1.62E-09	NKRF	-8.43E-02	1.00E+00	INVS	-2.45E-02	1.00E+00
KIAA1324	2.44E+00	1.64E-09	PLXNA2	-5.88E-02	1.00E+00	ACOT13	-3.24E-02	1.00E+00
SLC26A4	3.34E+00	1.65E-09	PLCB3	-1.09E-01	1.00E+00	ZC3H14	-2.44E-02	1.00E+00
PTPN1	5.55E-01	1.66E-09	LOC112581414	2.05E-01	1.00E+00	ST13	2.84E-02	1.00E+00
ATP6V1F	-9.14E-01	1.68E-09	MEGF11	-3.52E-02	1.00E+00	FURIN	2.48E-02	1.00E+00
THSD4	1.83E+00	1.68E-09	LOC112578706	1.39E-01	1.00E+00	UFD1	-3.05E-02	1.00E+00
SPP1	-4.16E+00	1.69E-09	GRWD1	4.24E-02	1.00E+00	RPL10	-4.52E-02	1.00E+00
ATP5F1C	-7.93E-01	1.69E-09	C18H16orf95	1.23E-01	1.00E+00	CAD	-2.56E-02	1.00E+00
LIN37	8.88E-01	1.71E-09	LOC102409332	2.88E-02	1.00E+00	HSPA4L	3.06E-02	1.00E+00
LOC102394379	-2.21E+00	1.72E-09	CSNK1A1	3.34E-02	1.00E+00	LOC102411097	-6.23E-02	1.00E+00
ANKRD13C	-7.88E-01	1.72E-09	LOC102394445	7.07E-02	1.00E+00	TRIM21	2.68E-02	1.00E+00
TMEM150B	2.08E+00	1.72E-09	GTF2I	3.95E-02	1.00E+00	RBFA	2.68E-02	1.00E+00

COL9A3	2.48E+00	1.73E-09	OPLAH	-1.57E-01	1.00E+00	C17H12orf43	3.10E-02	1.00E+00
SLC44A3	7.26E-01	1.75E-09	EIF5	-3.20E-02	1.00E+00	SNRPB	-3.04E-02	1.00E+00
DDX39A	-8.38E-01	1.77E-09	CARF	9.42E-02	1.00E+00	POLR2C	2.37E-02	1.00E+00
CDH23	1.98E+00	1.78E-09	UCK2	5.02E-02	1.00E+00	SNX12	2.49E-02	1.00E+00
LOC112582986	-1.91E+00	1.78E-09	GZF1	6.37E-02	1.00E+00	ARHGAP1	2.57E-02	1.00E+00
MPRIP	-6.98E-01	1.81E-09	MRPS16	-3.63E-02	1.00E+00	UNKL	3.05E-02	1.00E+00
ARFGEF1	-7.21E-01	1.81E-09	MLF1	2.95E-02	1.00E+00	FYTTD1	-2.82E-02	1.00E+00
MCC	7.75E-01	1.83E-09	LOC102403165	1.94E-01	1.00E+00	RPS15	-5.34E-02	1.00E+00
LOC112579135	2.35E+00	1.83E-09	FBRS	3.57E-02	1.00E+00	FZD6	-4.31E-02	1.00E+00
TSC22D2	9.15E-01	1.84E-09	PTCRA	1.63E-01	1.00E+00	CALM1	2.95E-02	1.00E+00
NUP85	-7.10E-01	1.84E-09	LOC112586228	-6.11E-02	1.00E+00	TMEM189	-2.98E-02	1.00E+00
CLPB	-7.23E-01	1.85E-09	CENPQ	2.97E-02	1.00E+00	SETD4	-2.81E-02	1.00E+00
SLC14A2	3.23E+00	1.85E-09	DNAH7	1.81E-01	1.00E+00	GTF2I	2.51E-02	1.00E+00
LOC112585756	-1.77E+00	1.86E-09	EPHA7	6.92E-02	1.00E+00	SNAI1	-8.75E-02	1.00E+00
TEX15	-1.63E+00	1.88E-09	PAFAH1B3	-3.99E-02	1.00E+00	STIMATE	3.01E-02	1.00E+00
PHLDB1	-9.49E-01	1.90E-09	SUFU	-4.23E-02	1.00E+00	LYAR	-2.69E-02	1.00E+00
IFI30	-2.35E+00	1.91E-09	CMTM8	-3.88E-02	1.00E+00	DHRSX	2.89E-02	1.00E+00
MACROD1	-1.18E+00	1.91E-09	COL6A5	5.65E-02	1.00E+00	TSHZ3	3.81E-02	1.00E+00
LOC102389807	2.44E+00	1.92E-09	ZCCHC7	2.53E-02	1.00E+00	PHYH	3.18E-02	1.00E+00
MID2	-1.82E+00	1.94E-09	SIRT5	-3.48E-02	1.00E+00	GUCY1B1	4.36E-02	1.00E+00
CARD19	-7.35E-01	1.94E-09	CHRNB1	1.21E-01	1.00E+00	ANKS6	-3.40E-02	1.00E+00
GTF2F1	8.86E-01	1.96E-09	FBH1	3.20E-02	1.00E+00	PCSK1	4.39E-02	1.00E+00
CNPPD1	-6.34E-01	1.96E-09	PAPPA2	-7.06E-02	1.00E+00	POMGNT1	2.55E-02	1.00E+00
RAB14	-2.58E+00	1.97E-09	LOC102397527	1.08E-01	1.00E+00	ZMYM4	3.07E-02	1.00E+00
LOC112579515	3.28E+00	1.98E-09	DCAF15	-2.65E-02	1.00E+00	PREP	-3.26E-02	1.00E+00
TIMM21	6.95E-01	2.01E-09	RAP1A	-3.62E-02	1.00E+00	RAD23A	-2.83E-02	1.00E+00
EIF6	-8.35E-01	2.02E-09	LOC102405958	7.74E-02	1.00E+00	PHLDA1	-5.67E-02	1.00E+00
LYRM2	-1.01E+00	2.03E-09	USP25	-3.93E-02	1.00E+00	NAA40	-2.71E-02	1.00E+00
GRID2	3.13E+00	2.05E-09	RHNO1	6.10E-02	1.00E+00	RBM33	-2.62E-02	1.00E+00
ARHGAP11A	-9.16E-01	2.05E-09	OTX1	6.22E-02	1.00E+00	CFAP36	-3.18E-02	1.00E+00
ZNF804A	3.31E+00	2.07E-09	IRAK3	-6.90E-02	1.00E+00	GAN	-5.06E-02	1.00E+00
FBXL12	-9.39E-01	2.07E-09	LOC112579135	-1.22E-01	1.00E+00	PLEK2	3.90E-02	1.00E+00
XPO5	6.86E-01	2.13E-09	LOC102409452	4.55E-02	1.00E+00	LOC112584551	5.89E-02	1.00E+00
ARMC6	-8.43E-01	2.15E-09	TBC1D14	-3.09E-02	1.00E+00	RNF208	-4.25E-02	1.00E+00

FAM219B	-9.51E-01	2.16E-09	SRSF5	-5.18E-02	1.00E+00	PLBD1	-7.86E-02	1.00E+00
LOC102389794	-2.00E+00	2.16E-09	BIRC5	-2.86E-02	1.00E+00	STAM	-3.04E-02	1.00E+00
ago-02	7.44E-01	2.17E-09	LOC102389262	7.26E-02	1.00E+00	IARS2	-2.39E-02	1.00E+00
KDM1A	5.66E-01	2.17E-09	LAD1	3.14E-02	1.00E+00	LOC102415752	-3.55E-02	1.00E+00
TCF4	9.11E-01	2.22E-09	TRAIP	3.64E-02	1.00E+00	MRPL52	3.86E-02	1.00E+00
HEPACAM	-3.51E+00	2.22E-09	RELL2	5.37E-02	1.00E+00	ZSCAN29	2.48E-02	1.00E+00
SCUBE2	1.78E+00	2.24E-09	PREPL	3.94E-02	1.00E+00	NR4A2	-7.33E-02	1.00E+00
CCT2	7.15E-01	2.24E-09	LOC112580847	9.03E-02	1.00E+00	EMB	8.35E-02	1.00E+00
ATP1B1	6.39E-01	2.27E-09	NOL6	3.99E-02	1.00E+00	PPP2R3C	2.55E-02	1.00E+00
PINX1	7.39E-01	2.30E-09	VIPR1	-1.51E-01	1.00E+00	SDCBP	-3.80E-02	1.00E+00
LOC112583635	1.55E+00	2.31E-09	LOC102392936	1.16E-01	1.00E+00	CCDC85A	4.82E-02	1.00E+00
VRK3	-7.97E-01	2.32E-09	MYH10	4.35E-02	1.00E+00	FZD1	3.21E-02	1.00E+00
CRTC1	6.32E-01	2.33E-09	LOC102414448	-7.62E-02	1.00E+00	ERCC8	-3.16E-02	1.00E+00
TANC2	-8.27E-01	2.34E-09	AFTPH	5.40E-02	1.00E+00	MLLT10	-3.15E-02	1.00E+00
CAPN6	-3.60E+00	2.37E-09	RSL1D1	-2.74E-02	1.00E+00	CCDC58	3.32E-02	1.00E+00
RMND1	7.84E-01	2.39E-09	GARS	-2.56E-02	1.00E+00	RTRAF	3.54E-02	1.00E+00
SLC16A11	1.10E+00	2.39E-09	RECQL5	4.65E-02	1.00E+00	ENOPH1	-2.49E-02	1.00E+00
LOC112580332	2.58E+00	2.39E-09	GTPBP8	1.15E-01	1.00E+00	PPP2R2A	2.58E-02	1.00E+00
ZNF445	7.15E-01	2.42E-09	TMC6	-2.19E-01	1.00E+00	TNIK	-3.40E-02	1.00E+00
RANBP3L	-3.84E+00	2.44E-09	PSMC5	-4.22E-02	1.00E+00	TRAPPC4	2.69E-02	1.00E+00
DEPDC1	-9.70E-01	2.45E-09	WDCP	-3.17E-02	1.00E+00	TFB1M	-3.25E-02	1.00E+00
UBE2G2	-6.26E-01	2.47E-09	LOC112582116	-1.45E-01	1.00E+00	BCLAF3	3.33E-02	1.00E+00
ZNF710	9.84E-01	2.50E-09	CYTH1	3.04E-02	1.00E+00	UVRAG	3.04E-02	1.00E+00
FRK	-1.27E+00	2.51E-09	SIAE	-4.97E-02	1.00E+00	SYNPO2	6.89E-02	1.00E+00
LOC102410713	3.08E+00	2.52E-09	LOC102409828	5.80E-02	1.00E+00	AKAP9	-3.25E-02	1.00E+00
POLR2E	-8.23E-01	2.53E-09	RXRA	9.93E-02	1.00E+00	GPR27	-4.97E-02	1.00E+00
EMG1	-9.75E-01	2.55E-09	LIX1	6.44E-02	1.00E+00	ZNF532	-2.96E-02	1.00E+00
IL1RAPL2	2.54E+00	2.63E-09	EFNA1	-1.51E-01	1.00E+00	LOC102403897	5.56E-02	1.00E+00
CRK	6.13E-01	2.64E-09	DBX2	1.03E-01	1.00E+00	LOC102408414	6.37E-02	1.00E+00
SCRN2	-1.18E+00	2.66E-09	BTG4	-3.74E-02	1.00E+00	LOC102416267	6.71E-02	1.00E+00
AP3S1	6.93E-01	2.67E-09	GALNT7	6.59E-02	1.00E+00	BRAP	2.77E-02	1.00E+00
LOC102398418	2.64E+00	2.67E-09	CERKL	5.36E-02	1.00E+00	EIPR1	-3.51E-02	1.00E+00
NXPH4	-1.50E+00	2.67E-09	CATSPERB	1.26E-01	1.00E+00	TMEM241	3.54E-02	1.00E+00
LOC102400303	-5.28E+00	2.68E-09	LOC102408435	5.61E-02	1.00E+00	LOC112587024	6.15E-02	1.00E+00
SYCE1L	-1.51E+00	2.68E-09	TBC1D5	-3.34E-02	1.00E+00	NMD3	2.88E-02	1.00E+00

BEST2	2.10E+00	2.69E-09	MAP7	2.78E-02	1.00E+00	ZNF496	-2.35E-02	1.00E+00
LOC112579981	1.51E+00	2.73E-09	SLC30A5	-4.34E-02	1.00E+00	LOC102411924	-4.80E-02	1.00E+00
LOC112585035	-3.79E+00	2.75E-09	SRRM3	1.03E-01	1.00E+00	LRRC8E	4.54E-02	1.00E+00
LOC112583963	-2.36E+00	2.77E-09	CCR5	7.84E-02	1.00E+00	SHOC2	-2.92E-02	1.00E+00
SARDH	1.85E+00	2.78E-09	SMKR1	-7.55E-02	1.00E+00	TIMM17B	-3.30E-02	1.00E+00
LRRC4B	1.71E+00	2.80E-09	LOC112578843	-9.29E-02	1.00E+00	FAM32A	3.22E-02	1.00E+00
WASF1	-7.34E-01	2.85E-09	CLTA	-2.95E-02	1.00E+00	EFHB	4.40E-02	1.00E+00
XPO6	6.45E-01	2.87E-09	FOSB	5.66E-02	1.00E+00	ASIC4	-4.43E-02	1.00E+00
SMYD5	6.80E-01	2.93E-09	LMNB2	-4.32E-02	1.00E+00	MKX	-2.47E-02	1.00E+00
IGF1R	-6.90E-01	2.94E-09	FBXW4	-3.66E-02	1.00E+00	LOC112579116	-6.31E-02	1.00E+00
CDCA4	-8.78E-01	2.96E-09	BRK1	-3.58E-02	1.00E+00	LOC102413280	-2.89E-02	1.00E+00
PKLR	1.21E+00	2.96E-09	SLC25A1	2.88E-02	1.00E+00	NUP210L	-2.97E-02	1.00E+00
LOC112583892	-3.00E+00	2.97E-09	SELENOW	3.40E-02	1.00E+00	KIAA1522	3.32E-02	1.00E+00
ZBTB44	-7.55E-01	2.99E-09	SSBP1	3.23E-02	1.00E+00	TMEM59L	5.68E-02	1.00E+00
LOC112578542	3.51E+00	2.99E-09	NFKBIB	-4.66E-02	1.00E+00	PROB1	4.08E-02	1.00E+00
ZNHIT2	9.96E-01	3.01E-09	USP2	4.39E-02	1.00E+00	SELENOO	-2.98E-02	1.00E+00
LOC112587813	3.44E+00	3.03E-09	CYREN	-4.18E-02	1.00E+00	VAMP4	2.90E-02	1.00E+00
PI4K2B	-9.82E-01	3.03E-09	NXT2	-4.23E-02	1.00E+00	HNRNPC	-2.89E-02	1.00E+00
HDLBP	-8.18E-01	3.08E-09	TBL3	3.54E-02	1.00E+00	AMD1	2.63E-02	1.00E+00
PLCL1	-1.29E+00	3.11E-09	GPR45	-1.44E-01	1.00E+00	UBALD1	-3.51E-02	1.00E+00
SLC12A6	7.37E-01	3.13E-09	AMZ2	3.29E-02	1.00E+00	ZNF8	2.53E-02	1.00E+00
LOC112579667	1.49E+00	3.16E-09	FUZ	-6.95E-02	1.00E+00	CELF1	-2.46E-02	1.00E+00
ND4	-1.45E+00	3.19E-09	MCF2L2	-8.97E-02	1.00E+00	PTP4A3	-5.80E-02	1.00E+00
DLGAP2	2.12E+00	3.23E-09	DENND5A	4.58E-02	1.00E+00	NPEPPS	2.72E-02	1.00E+00
MYO9A	8.76E-01	3.29E-09	LOC112581760	-1.13E-01	1.00E+00	XG	-2.84E-02	1.00E+00
BACE2	-9.25E-01	3.29E-09	PDS5B	4.77E-02	1.00E+00	ZNF213	3.52E-02	1.00E+00
LOC112579582	-4.63E+00	3.29E-09	NLE1	8.05E-02	1.00E+00	LRPPRC	-2.27E-02	1.00E+00
LOC112585571	-2.50E+00	3.29E-09	PAPSS2	-1.75E-01	1.00E+00	RPL23	-4.68E-02	1.00E+00
RBM23	-7.09E-01	3.32E-09	LOC102393420	-8.24E-02	1.00E+00	MAN2A1	3.60E-02	1.00E+00
CES5A	-1.48E+00	3.34E-09	CENPC	3.75E-02	1.00E+00	SERBP1	-2.99E-02	1.00E+00
LOC102395337	-2.98E+00	3.34E-09	RBM12B	4.18E-02	1.00E+00	MTREX	2.88E-02	1.00E+00
NXT1	8.59E-01	3.37E-09	MKX	-1.55E-01	1.00E+00	LOC112577789	-3.23E-02	1.00E+00
AURKAIP1	-1.05E+00	3.39E-09	MARCH2	-4.28E-02	1.00E+00	LOC102408579	-5.58E-02	1.00E+00

HEATR5A	-1.29E+00	3.40E-09	ZBTB32	9.71E-02	1.00E+00	LOC102396998	2.86E-02	1.00E+00
TMEM182	-1.59E+00	3.40E-09	ALPK1	9.50E-02	1.00E+00	REXO1	-2.42E-02	1.00E+00
FYTTD1	-6.47E-01	3.41E-09	AP3S1	4.31E-02	1.00E+00	HYAL3	-5.10E-02	1.00E+00
KLHL35	2.25E+00	3.42E-09	ADI1	5.80E-02	1.00E+00	FEM1C	2.97E-02	1.00E+00
SMIM8	1.10E+00	3.44E-09	CHCHD3	-2.66E-02	1.00E+00	LOC112578483	3.00E-02	1.00E+00
LOC112583639	-5.12E+00	3.49E-09	B3GLCT	3.70E-02	1.00E+00	PDE12	-3.89E-02	1.00E+00
ADO	5.82E-01	3.49E-09	MRTFB	5.35E-02	1.00E+00	CCND2	3.20E-02	1.00E+00
RNASEL	1.21E+00	3.60E-09	GREM1	-6.82E-02	1.00E+00	SFXN4	3.82E-02	1.00E+00
ITGA8	1.28E+00	3.69E-09	TRIM26	3.86E-02	1.00E+00	LOC102414910	2.79E-02	1.00E+00
ARPC5	-5.66E-01	3.70E-09	LOC102391170	1.45E-01	1.00E+00	UBE4A	2.66E-02	1.00E+00
LIN7B	-1.16E+00	3.75E-09	GEM	-2.56E-01	1.00E+00	NKRF	-4.72E-02	1.00E+00
SRI	-7.50E-01	3.77E-09	CC2D1B	4.52E-02	1.00E+00	GBGT1	-2.85E-02	1.00E+00
ARID1A	-9.49E-01	3.77E-09	FANCF	3.15E-02	1.00E+00	SPIDR	-2.55E-02	1.00E+00
CADPS2	6.05E-01	3.79E-09	LRIG3	5.38E-02	1.00E+00	ADGRB3	-3.73E-02	1.00E+00
SEMA5B	1.65E+00	3.80E-09	FKBP1B	-8.84E-02	1.00E+00	SLC24A1	4.68E-02	1.00E+00
FAM24A	2.84E+00	3.82E-09	TPGS1	-8.40E-02	1.00E+00	MPDZ	-3.25E-02	1.00E+00
RBM45	-5.69E-01	3.83E-09	CPOX	6.06E-02	1.00E+00	CCDC189	-3.05E-02	1.00E+00
ST7	-7.71E-01	3.83E-09	LOC102411680	2.09E-01	1.00E+00	PDIK1L	-2.95E-02	1.00E+00
S100A9	-5.23E+00	3.83E-09	CAD	-1.10E-01	1.00E+00	MTMR9	-3.99E-02	1.00E+00
LOC102393493	2.35E+00	3.91E-09	TMTC1	-1.34E-01	1.00E+00	IFT27	-3.74E-02	1.00E+00
LOC102403840	3.22E+00	3.92E-09	CDK2AP1	-2.78E-02	1.00E+00	VPS39	2.19E-02	1.00E+00
ITGA10	9.93E-01	3.95E-09	NR4A1	2.92E-02	1.00E+00	HINFP	2.52E-02	1.00E+00
RAP1B	5.83E-01	3.99E-09	LOC102410373	-2.97E-02	1.00E+00	TENT2	2.52E-02	1.00E+00
FANCC	-6.68E-01	4.02E-09	SALL4	4.69E-02	1.00E+00	CCDC33	5.46E-02	1.00E+00
SH3RF3	-2.65E+00	4.02E-09	WDR12	4.63E-02	1.00E+00	LOC112585112	-3.44E-02	1.00E+00
DLGAP4	6.81E-01	4.08E-09	FAXC	6.73E-02	1.00E+00	PXMP4	-4.60E-02	1.00E+00
LOC112584582	-1.67E+00	4.08E-09	MRPS22	-4.53E-02	1.00E+00	PEPD	2.29E-02	1.00E+00
PAFAH1B1	-5.26E-01	4.10E-09	LRP1	-9.38E-02	1.00E+00	PCYT1B	-3.32E-02	1.00E+00
TMA16	-8.96E-01	4.11E-09	SIPA1L3	-2.80E-02	1.00E+00	KIAA0895L	-4.83E-02	1.00E+00
PTPN11	9.33E-01	4.14E-09	RPS29	1.13E-01	1.00E+00	COPRS	-3.50E-02	1.00E+00
HPD	3.03E+00	4.18E-09	BCAS3	-2.75E-02	1.00E+00	LOC102410971	-4.20E-02	1.00E+00
SNX3	5.72E-01	4.20E-09	DOLK	3.13E-02	1.00E+00	ARRDC2	-7.27E-02	1.00E+00
DUSP3	8.44E-01	4.21E-09	LOC112580423	1.37E-01	1.00E+00	PGGT1B	-2.83E-02	1.00E+00
TMC7	1.81E+00	4.21E-09	RBM38	2.77E-02	1.00E+00	HMBOX1	-3.98E-	1.00E+00

							02	
CABLES1	-2.88E+00	4.22E-09	LOC112579560	-6.10E-02	1.00E+00	TNFRSF1A	2.61E-02	1.00E+00
RRM2B	1.00E+00	4.25E-09	PPP1R36	-1.04E-01	1.00E+00	TAX1BP1	-2.65E-02	1.00E+00
IL21R	-3.66E+00	4.34E-09	TMEM209	-3.58E-02	1.00E+00	POLR3A	-2.23E-02	1.00E+00
LOC112587836	2.99E+00	4.37E-09	EEF1AKMT2	9.52E-02	1.00E+00	FCHSD1	3.56E-02	1.00E+00
LOC112584546	-1.28E+00	4.40E-09	MARVELD3	4.05E-02	1.00E+00	RUFY3	-2.16E-02	1.00E+00
ADAMTS4	2.44E+00	4.43E-09	GCKR	-8.03E-02	1.00E+00	TBC1D9	-3.13E-02	1.00E+00
PDK2	6.39E-01	4.47E-09	CCDC96	1.25E-01	1.00E+00	HACD2	-2.30E-02	1.00E+00
ZNF622	6.71E-01	4.47E-09	UBXN7	2.98E-02	1.00E+00	THRAP3	2.48E-02	1.00E+00
TEAD3	7.22E-01	4.50E-09	FBXL6	4.24E-02	1.00E+00	DCAF12	2.20E-02	1.00E+00
PIP4P1	5.77E-01	4.51E-09	CRB1	-5.61E-02	1.00E+00	CDH6	-3.96E-02	1.00E+00
ARHGDIA	-6.63E-01	4.51E-09	COQ6	-4.76E-02	1.00E+00	TRAPPC1	3.17E-02	1.00E+00
UPP1	-4.94E+00	4.53E-09	TICRR	4.35E-02	1.00E+00	TMEM43	-2.30E-02	1.00E+00
TRPV5	3.97E+00	4.57E-09	GSX1	1.46E-01	1.00E+00	PIGL	3.30E-02	1.00E+00
MTMR11	7.69E-01	4.58E-09	SLCO1A2	-1.26E-01	1.00E+00	TTI2	-2.71E-02	1.00E+00
DDX3X	9.46E-01	4.59E-09	NDUFA1	6.81E-02	1.00E+00	TNS1	3.65E-02	1.00E+00
NSD2	-6.21E-01	4.60E-09	LOC112580272	-1.72E-01	1.00E+00	CIC	2.67E-02	1.00E+00
NEK3	7.28E-01	4.68E-09	PRUNE1	4.39E-02	1.00E+00	XPNPEP1	-2.17E-02	1.00E+00
ZNF653	-7.51E-01	4.70E-09	NAA16	-5.96E-02	1.00E+00	BLOC1S4	3.05E-02	1.00E+00
TTC19	6.40E-01	4.72E-09	EIPR1	3.43E-02	1.00E+00	GOLGA7	2.30E-02	1.00E+00
HPX	1.44E+00	4.72E-09	MRPL4	2.80E-02	1.00E+00	SMCR8	3.84E-02	1.00E+00
ELP5	-7.48E-01	4.74E-09	KIAA2026	-8.30E-02	1.00E+00	NELFB	2.56E-02	1.00E+00
SOGA1	-9.07E-01	4.75E-09	DKKL1	-1.87E-01	1.00E+00	SLC36A4	4.68E-02	1.00E+00
DENND1A	-6.19E-01	4.77E-09	C16H11orf94	1.11E-01	1.00E+00	CEP290	-2.87E-02	1.00E+00
BPTF	-1.00E+00	4.77E-09	PCGF3	-3.12E-02	1.00E+00	CAMK1D	-2.55E-02	1.00E+00
TIMM50	-8.24E-01	4.83E-09	INSYN1	-1.05E-01	1.00E+00	FAM151B	-3.45E-02	1.00E+00
LOC112584742	1.48E+00	4.87E-09	GTPBP2	-3.10E-02	1.00E+00	TSC1	-2.58E-02	1.00E+00
LOC112579895	2.95E+00	4.93E-09	ECH1	5.46E-02	1.00E+00	LOC112586640	6.66E-02	1.00E+00
TMEM117	1.21E+00	4.98E-09	ZCCHC4	-5.21E-02	1.00E+00	RFX7	3.09E-02	1.00E+00
ERI3	-8.49E-01	5.07E-09	GREB1L	-6.21E-02	1.00E+00	COPS9	3.56E-02	1.00E+00
FAM155B	-2.49E+00	5.07E-09	TBC1D22B	3.18E-02	1.00E+00	IQCC	3.31E-02	1.00E+00
PI16	-2.48E+00	5.13E-09	LOC102393616	-1.42E-01	1.00E+00	DDX50	-4.33E-02	1.00E+00
LEPROT	-6.92E-01	5.13E-09	USP48	-2.91E-02	1.00E+00	NTHL1	-3.86E-02	1.00E+00
EML2	6.81E-01	5.29E-09	BNIP3	3.55E-02	1.00E+00	MTFR1	-3.19E-02	1.00E+00
HSPB6	1.04E+00	5.35E-09	PPIG	-2.60E-02	1.00E+00	RPP30	2.66E-02	1.00E+00

DYNLRB1	-9.25E-01	5.46E-09	LOC112585619	7.48E-02	1.00E+00	C15H8orf33	-2.71E-02	1.00E+00
LOC102411280	-1.30E+00	5.46E-09	KLHL31	1.73E-01	1.00E+00	LOC102411649	-3.83E-02	1.00E+00
RIF1	-8.54E-01	5.47E-09	DCAF4	6.89E-02	1.00E+00	SLC25A5	-2.62E-02	1.00E+00
DLG3	-5.67E-01	5.54E-09	SURF2	4.97E-02	1.00E+00	LOC102403818	3.34E-02	1.00E+00
ZMYM1	-9.26E-01	5.56E-09	SRSF10	2.68E-02	1.00E+00	PPHLN1	-2.31E-02	1.00E+00
C8A	3.73E+00	5.59E-09	CTSA	4.89E-02	1.00E+00	CPNE2	6.23E-02	1.00E+00
SLC2A8	-6.67E-01	5.63E-09	LOC112584718	1.35E-01	1.00E+00	TEX11	2.61E-02	1.00E+00
AAR2	7.35E-01	5.75E-09	DENND1B	-2.60E-02	1.00E+00	DYNC1LI2	2.88E-02	1.00E+00
LOC102399010	1.35E+00	5.76E-09	VOPP1	-8.71E-02	1.00E+00	MKNK2	-2.32E-02	1.00E+00
WFIKKN2	-2.41E+00	5.89E-09	PREX2	-1.13E-01	1.00E+00	CARD19	2.52E-02	1.00E+00
FBXO3	5.48E-01	5.91E-09	RDH12	4.17E-02	1.00E+00	CDK2AP1	-2.50E-02	1.00E+00
LOC102413852	3.26E+00	5.95E-09	CCDC77	-1.07E-01	1.00E+00	BNIP2	-2.79E-02	1.00E+00
LOC102412702	-1.35E+00	5.96E-09	KDR	8.99E-02	1.00E+00	GTF2A2	-2.63E-02	1.00E+00
RIC8B	-6.15E-01	6.02E-09	MAP1LC3A	5.11E-02	1.00E+00	MARF1	3.05E-02	1.00E+00
TMEM190	1.51E+00	6.03E-09	LOC112581555	1.90E-01	1.00E+00	CCDC12	2.48E-02	1.00E+00
VPS26A	-1.37E+00	6.07E-09	GPSM1	1.24E-01	1.00E+00	CELSR1	6.16E-02	1.00E+00
JMJD8	-8.11E-01	6.12E-09	MLLT11	-2.71E-02	1.00E+00	SPRY1	4.86E-02	1.00E+00
CD24	-4.44E+00	6.22E-09	LOC102413289	-2.92E-02	1.00E+00	LOC102403617	3.32E-02	1.00E+00
NIPBL	-8.72E-01	6.43E-09	GPN1	-3.59E-02	1.00E+00	WAS	1.11E-01	1.00E+00
WDR74	-9.19E-01	6.59E-09	DCAF8	3.09E-02	1.00E+00	DCX	4.63E-02	1.00E+00
ID3	1.23E+00	6.66E-09	OCIAD1	-4.72E-02	1.00E+00	RNF5	2.73E-02	1.00E+00
LOC112585287	2.74E+00	6.66E-09	DDX28	2.92E-02	1.00E+00	CFAP126	5.65E-02	1.00E+00
CRY2	-6.92E-01	6.70E-09	ZFP90	5.96E-02	1.00E+00	USP33	-3.03E-02	1.00E+00
LOC102399225	-2.39E+00	6.76E-09	GIPR	4.48E-02	1.00E+00	CPXM1	-4.14E-02	1.00E+00
LOC102406662	1.03E+00	6.79E-09	CNTFR	1.48E-01	1.00E+00	DHX57	2.32E-02	1.00E+00
LOC102391941	3.87E+00	6.79E-09	LOC112583615	-1.63E-01	1.00E+00	MAT2B	-2.64E-02	1.00E+00
LOC102408782	2.00E+00	6.87E-09	DCTN4	2.70E-02	1.00E+00	DNAAF3	-3.27E-02	1.00E+00
YARS2	-7.22E-01	6.92E-09	LOC112586225	-1.13E-01	1.00E+00	ZNF318	-3.03E-02	1.00E+00
LOC102389189	-1.75E+00	6.93E-09	ATF2	-3.57E-02	1.00E+00	LOC112579958	5.61E-02	1.00E+00
ARHGEF25	-1.24E+00	6.98E-09	ZSCAN29	-3.25E-02	1.00E+00	PRKAR2A	-2.29E-02	1.00E+00
THNSL2	-1.53E+00	7.01E-09	TOE1	-4.46E-02	1.00E+00	FTCDNL1	5.97E-02	1.00E+00
PDSS1	-7.85E-01	7.04E-09	LOC112578056	-1.27E-01	1.00E+00	ZDHHC18	-2.87E-02	1.00E+00
ALMS1	7.21E-01	7.04E-09	EPHA4	-5.76E-02	1.00E+00	TEDC2	3.03E-02	1.00E+00
SPRY2	1.18E+00	7.05E-09	PREX1	4.22E-02	1.00E+00	ZNF622	-2.58E-	1.00E+00

							02	
C6H1orf122	-1.00E+00	7.08E-09	CELF4	3.43E-02	1.00E+00	RHBDD2	-2.36E-02	1.00E+00
EGLN2	-6.35E-01	7.09E-09	SWT1	-6.64E-02	1.00E+00	SPOUT1	-3.16E-02	1.00E+00
ADGRD2	2.58E+00	7.09E-09	PLEKHA8	-4.14E-02	1.00E+00	BLMH	2.27E-02	1.00E+00
FAT4	-2.93E+00	7.09E-09	UBE2K	-2.64E-02	1.00E+00	ABHD12	-2.78E-02	1.00E+00
LOC112586509	1.86E+00	7.18E-09	ZFHX3	4.34E-02	1.00E+00	ZNF572	-2.52E-02	1.00E+00
DYNC1LI1	7.25E-01	7.20E-09	LOC102402897	-4.69E-02	1.00E+00	DXO	-3.13E-02	1.00E+00
LOC112581227	-2.82E+00	7.30E-09	METTL18	-3.14E-02	1.00E+00	LOC102399180	-4.11E-02	1.00E+00
SIGLEC1	-5.20E+00	7.35E-09	LOC102395477	-3.64E-02	1.00E+00	CCDC78	-4.05E-02	1.00E+00
TINAGL1	-5.05E+00	7.38E-09	RNF187	1.33E-01	1.00E+00	FAM234A	2.66E-02	1.00E+00
SLC9A9	1.84E+00	7.41E-09	C4H12orf4	-3.34E-02	1.00E+00	ALDH1L2	-3.28E-02	1.00E+00
SAMSN1	-4.32E+00	7.42E-09	RNF43	4.14E-02	1.00E+00	ARHGAP10	-2.24E-02	1.00E+00
ZNF511	-1.07E+00	7.45E-09	NTAN1	-4.02E-02	1.00E+00	ELMOD3	-2.32E-02	1.00E+00
LOC112578527	4.38E+00	7.47E-09	LMNA	-3.91E-02	1.00E+00	LOC102410528	-3.16E-02	1.00E+00
LOC112580209	-3.29E+00	7.50E-09	RHEB	-3.13E-02	1.00E+00	LOC112579184	-5.47E-02	1.00E+00
LOC102399397	-9.79E-01	7.54E-09	PTTG1IP	-2.65E-02	1.00E+00	CDK1	-2.43E-02	1.00E+00
NUDT1	-1.41E+00	7.68E-09	OVOL2	-2.12E-01	1.00E+00	PYGO1	2.90E-02	1.00E+00
LOC112581142	-3.46E+00	7.69E-09	FERMT2	-9.74E-02	1.00E+00	SCYL1	-2.44E-02	1.00E+00
RXFP2	3.66E+00	7.70E-09	ZC3H6	-1.63E-01	1.00E+00	POU2F1	2.87E-02	1.00E+00
RNF122	-1.77E+00	7.72E-09	LOC112579667	-9.03E-02	1.00E+00	MRM3	2.56E-02	1.00E+00
FAM53A	2.99E+00	7.96E-09	LOC102405806	-1.22E-01	1.00E+00	SLC25A12	2.59E-02	1.00E+00
LOC102409778	-3.35E+00	8.00E-09	TICAM2	1.63E-01	1.00E+00	TP53I3	4.55E-02	1.00E+00
ETFBKMT	-1.79E+00	8.04E-09	SAMD4A	1.18E-01	1.00E+00	KLF12	2.79E-02	1.00E+00
TEAD1	7.15E-01	8.07E-09	ARIH1	2.87E-02	1.00E+00	RHOG	-2.77E-02	1.00E+00
LOC102414731	-2.80E+00	8.10E-09	CHGB	-6.54E-02	1.00E+00	RNF121	2.32E-02	1.00E+00
LOC102405833	-9.32E-01	8.16E-09	ELP1	3.45E-02	1.00E+00	ZBTB20	-2.86E-02	1.00E+00
CASK	-6.42E-01	8.23E-09	ZNF333	6.84E-02	1.00E+00	ALPK3	-2.13E-02	1.00E+00
SYNE3	-1.08E+00	8.27E-09	CTSB	2.65E-02	1.00E+00	DDR1	2.70E-02	1.00E+00
SAR1B	-8.03E-01	8.59E-09	LOC102402392	6.60E-02	1.00E+00	ABHD13	2.74E-02	1.00E+00
SPPL2B	-6.43E-01	8.67E-09	CBR4	-1.33E-01	1.00E+00	C3H9orf64	3.41E-02	1.00E+00
OPA3	7.42E-01	8.79E-09	LOC112584562	-5.57E-02	1.00E+00	LOC112587928	-3.53E-02	1.00E+00
SYT7	1.87E+00	8.81E-09	FUNDC2	-3.79E-02	1.00E+00	FAM3C	2.47E-02	1.00E+00
LOC112580412	4.59E+00	8.84E-09	BARX1	-6.79E-	1.00E+00	BBS7	3.09E-02	1.00E+00

				02				
ARFGAP2	-6.16E-01	8.85E-09	CHN1	-2.75E-02	1.00E+00	ERC2	-3.99E-02	1.00E+00
SRRM2	-1.02E+00	9.35E-09	ZNF280D	6.21E-02	1.00E+00	GSK3B	2.90E-02	1.00E+00
MYSM1	-8.77E-01	9.40E-09	ZNF423	9.60E-02	1.00E+00	YJU2	-2.86E-02	1.00E+00
MRPS11	-8.88E-01	9.40E-09	PRKG2	7.96E-02	1.00E+00	RPL27	-4.26E-02	1.00E+00
LOC102407601	-4.51E+00	9.40E-09	POLN	-4.86E-02	1.00E+00	NAT10	2.19E-02	1.00E+00
WNT16	2.96E+00	9.40E-09	AEN	-1.07E-01	1.00E+00	IRF2BPL	-3.74E-02	1.00E+00
ZFAT	7.05E-01	9.53E-09	FAM92A	4.47E-02	1.00E+00	OSGEPL1	3.00E-02	1.00E+00
CFAP298	6.75E-01	9.56E-09	NKX1-2	-1.59E-01	1.00E+00	CCS	-2.82E-02	1.00E+00
APBA3	6.24E-01	9.58E-09	APEX2	-5.01E-02	1.00E+00	LOC102391413	-5.79E-02	1.00E+00
LETM1	-5.63E-01	9.61E-09	KDM4B	3.74E-02	1.00E+00	USF3	-3.91E-02	1.00E+00
TTC29	1.80E+00	9.67E-09	CSNK1D	2.38E-02	1.00E+00	KPNA4	2.18E-02	1.00E+00
LNX1	-1.42E+00	9.80E-09	BFSP1	-7.26E-02	1.00E+00	TTPAL	-4.23E-02	1.00E+00
IFT81	-7.53E-01	9.81E-09	CDH6	-7.20E-02	1.00E+00	UBN1	2.40E-02	1.00E+00
SLC25A12	6.35E-01	9.81E-09	LOC102403309	-1.50E-01	1.00E+00	WDR82	-2.32E-02	1.00E+00
LOC102398703	2.35E+00	9.84E-09	TACC1	4.99E-02	1.00E+00	C6H1orf54	4.79E-02	1.00E+00
RNF145	5.92E-01	9.98E-09	CCDC188	-1.10E-01	1.00E+00	LOC102407744	2.42E-02	1.00E+00
LOC102413450	-2.35E+00	1.01E-08	LOC102402866	6.33E-02	1.00E+00	CLN8	2.95E-02	1.00E+00
P2RX1	2.67E+00	1.01E-08	LOC112586216	5.15E-02	1.00E+00	UBA6	-2.30E-02	1.00E+00
FBXW8	-6.40E-01	1.02E-08	LOC112585704	-1.19E-01	1.00E+00	UBA1	-2.29E-02	1.00E+00
LOC112584551	1.30E+00	1.02E-08	RCBTB1	-2.43E-02	1.00E+00	DBNDD1	-2.95E-02	1.00E+00
SF3B5	-1.05E+00	1.03E-08	MAP9	1.08E-01	1.00E+00	MIS12	3.39E-02	1.00E+00
SNX14	-5.67E-01	1.03E-08	NRM	-8.00E-02	1.00E+00	CD2AP	3.11E-02	1.00E+00
AASDHPPT	-5.06E-01	1.03E-08	LOC112585609	6.79E-02	1.00E+00	LMAN2	-2.90E-02	1.00E+00
VEGFA	-2.87E+00	1.04E-08	RNASEL	6.00E-02	1.00E+00	ABHD18	3.19E-02	1.00E+00
EIF4EBP2	6.60E-01	1.04E-08	PCYT1B	5.14E-02	1.00E+00	MBP	-1.13E-01	1.00E+00
SERP1	4.93E-01	1.05E-08	JUN	-5.93E-02	1.00E+00	MOCS3	3.46E-02	1.00E+00
PIP4K2C	-4.90E-01	1.06E-08	STAP2	-4.00E-02	1.00E+00	ARHGEF26	-5.92E-02	1.00E+00
RAP1GAP	1.84E+00	1.07E-08	UBP1	5.08E-02	1.00E+00	SNAP23	-2.39E-02	1.00E+00
KDM5C	-5.80E-01	1.08E-08	TMEM74B	-8.22E-02	1.00E+00	MGAT2	-2.84E-02	1.00E+00
TBKBP1	-8.11E-01	1.08E-08	LOC112578878	1.16E-01	1.00E+00	ARMCX3	-3.14E-02	1.00E+00
LOC102406111	-4.11E+00	1.10E-08	INTS8	-3.98E-02	1.00E+00	GEMIN4	2.48E-02	1.00E+00
SPATA20	1.22E+00	1.13E-08	MRPL39	3.52E-02	1.00E+00	AAMDC	3.74E-02	1.00E+00
CCDC167	1.10E+00	1.14E-08	TFAM	1.66E-01	1.00E+00	LDHD	4.11E-02	1.00E+00

CRTC3	-5.67E-01	1.15E-08	EVC2	-1.12E-01	1.00E+00	AKAP11	-3.00E-02	1.00E+00
LOC112583748	1.77E+00	1.15E-08	ATR	-6.59E-02	1.00E+00	TAPBPL	2.77E-02	1.00E+00
MFSD14B	5.95E-01	1.16E-08	SYT16	3.94E-02	1.00E+00	SLC39A1	2.16E-02	1.00E+00
LOC102403941	-1.47E+00	1.16E-08	LSG1	-2.33E-02	1.00E+00	RPS6KA4	-2.65E-02	1.00E+00
WDR91	5.38E-01	1.17E-08	DDX58	-4.50E-02	1.00E+00	DHPS	-2.95E-02	1.00E+00
HECA	7.42E-01	1.17E-08	MRPL38	-3.02E-02	1.00E+00	PLA1A	5.82E-02	1.00E+00
XIRP2	4.86E+00	1.18E-08	ZFPL1	2.75E-02	1.00E+00	TADA2A	-2.28E-02	1.00E+00
THRB	-1.57E+00	1.19E-08	KCTD9	2.59E-02	1.00E+00	UTP11	2.28E-02	1.00E+00
SNU13	7.57E-01	1.19E-08	FAF1	3.57E-02	1.00E+00	FAM208A	2.54E-02	1.00E+00
GRIK4	3.20E+00	1.20E-08	TK2	4.02E-02	1.00E+00	PSMD5	-2.19E-02	1.00E+00
STK33	8.35E-01	1.20E-08	AK2	3.61E-02	1.00E+00	ACTL6A	-2.25E-02	1.00E+00
VNN2	-3.12E+00	1.21E-08	LIMD2	-4.16E-02	1.00E+00	NANP	-3.05E-02	1.00E+00
TMEM167B	-7.13E-01	1.22E-08	KIF26A	-1.83E-01	1.00E+00	BET1L	2.12E-02	1.00E+00
LOC102393972	-5.41E-01	1.23E-08	MLIP	3.99E-02	1.00E+00	LOC102399398	-2.38E-02	1.00E+00
PTTG1IP	-5.09E-01	1.24E-08	LOC112586639	1.44E-01	1.00E+00	HDGF	-2.67E-02	1.00E+00
ZBTB46	1.64E+00	1.25E-08	APPBP2	-3.01E-02	1.00E+00	ADAMTS9	-3.66E-02	1.00E+00
PPP1R12A	-1.47E+00	1.25E-08	KAT7	2.67E-02	1.00E+00	LAT2	-8.77E-02	1.00E+00
TBCE	-5.89E-01	1.26E-08	LOC112587399	8.80E-02	1.00E+00	RAB1B	2.62E-02	1.00E+00
FCF1	-8.46E-01	1.27E-08	EIF6	-3.47E-02	1.00E+00	ACAD11	3.37E-02	1.00E+00
CXCR4	1.76E+00	1.30E-08	TMCC3	3.01E-02	1.00E+00	FAM47E	4.63E-02	1.00E+00
LOC112583111	1.44E+00	1.31E-08	C16H11orf49	-6.02E-02	1.00E+00	PCMT1	-2.50E-02	1.00E+00
LOC102410630	-3.15E+00	1.31E-08	SALL1	8.34E-02	1.00E+00	CHMP5	2.06E-02	1.00E+00
TFEC	-3.54E+00	1.34E-08	LCA5L	6.41E-02	1.00E+00	TMEM240	-5.13E-02	1.00E+00
PTPA	-6.37E-01	1.35E-08	LOC102407011	-1.00E-01	1.00E+00	PECAM1	8.62E-02	1.00E+00
NUF2	8.04E-01	1.35E-08	IMMP1L	3.72E-02	1.00E+00	MPST	3.39E-02	1.00E+00
SAMD10	-2.16E+00	1.35E-08	YWHAE	2.32E-02	1.00E+00	LOC102415603	-3.40E-02	1.00E+00
BABAM2	5.54E-01	1.37E-08	ESRRG	4.02E-02	1.00E+00	LOC112586859	-5.47E-02	1.00E+00
BMP2	-3.75E+00	1.37E-08	RETSAT	-7.93E-02	1.00E+00	POLK	4.34E-02	1.00E+00
BHMG1	2.08E+00	1.39E-08	ZBTB14	-1.56E-01	1.00E+00	HDDC3	-3.88E-02	1.00E+00
TMEM198	1.23E+00	1.40E-08	GPD2	-8.46E-02	1.00E+00	PABPC1L	-3.59E-02	1.00E+00
NCKAP1	-6.75E-01	1.40E-08	BCAP29	3.77E-02	1.00E+00	APOLD1	7.43E-02	1.00E+00
LOC102411988	-1.53E+00	1.41E-08	SLC25A17	-3.55E-02	1.00E+00	IQCG	5.04E-02	1.00E+00
RAB1B	-6.88E-01	1.43E-08	KLHL13	-7.40E-02	1.00E+00	KDM6B	3.91E-02	1.00E+00
TBC1D20	-5.21E-	1.43E-08	CEP290	7.61E-02	1.00E+00	ABHD4	2.06E-02	1.00E+00

	01							
LGALS8	9.90E-01	1.43E-08	TBC1D32	-6.40E-02	1.00E+00	TOP1	-3.46E-02	1.00E+00
FHOD1	-9.48E-01	1.43E-08	FAM177A1	-8.49E-02	1.00E+00	POLR3B	2.42E-02	1.00E+00
COPE	-8.95E-01	1.43E-08	GGACT	1.70E-01	1.00E+00	CMC1	2.87E-02	1.00E+00
HPS6	-1.34E+00	1.43E-08	TMEM50A	3.37E-02	1.00E+00	ELP2	-2.17E-02	1.00E+00
SLC17A9	-3.90E+00	1.45E-08	LOC102408370	4.25E-02	1.00E+00	MED10	-2.45E-02	1.00E+00
FRMD6	1.73E+00	1.45E-08	IKZF5	9.34E-02	1.00E+00	DDIT4L	-5.27E-02	1.00E+00
ZFYVE21	6.79E-01	1.46E-08	LOC102411899	3.78E-02	1.00E+00	CASP8AP2	-3.38E-02	1.00E+00
EIF3E	-7.59E-01	1.46E-08	NLGN1	-1.43E-01	1.00E+00	SV2A	-3.70E-02	1.00E+00
LOC102414092	4.32E+00	1.49E-08	CHST7	-1.09E-01	1.00E+00	SMKR1	-3.35E-02	1.00E+00
LOC112583962	4.10E+00	1.49E-08	TRHDE	-6.37E-02	1.00E+00	LOC102391411	2.43E-02	1.00E+00
CCDC28B	-1.01E+00	1.50E-08	CHRDL2	-1.44E-01	1.00E+00	EVI5	-3.04E-02	1.00E+00
ARID4B	-7.51E-01	1.50E-08	CEP57	4.90E-02	1.00E+00	UTP6	2.36E-02	1.00E+00
SEC13	7.07E-01	1.51E-08	FUCA1	-3.72E-02	1.00E+00	ADAL	-2.98E-02	1.00E+00
TMEM54	-2.86E+00	1.52E-08	C2H6orf226	4.54E-02	1.00E+00	RHOU	4.82E-02	1.00E+00
SF3A2	6.45E-01	1.53E-08	SMIM24	6.26E-02	1.00E+00	SF3B3	-2.16E-02	1.00E+00
LOC112584601	2.88E+00	1.54E-08	ROR2	9.52E-02	1.00E+00	STX5	2.27E-02	1.00E+00
STMP1	5.45E-01	1.56E-08	NUDCD1	-2.82E-02	1.00E+00	ATP5PB	2.44E-02	1.00E+00
ZFYVE16	-9.43E-01	1.57E-08	LOC102391376	-6.23E-02	1.00E+00	TRPC1	3.84E-02	1.00E+00
HOOK1	9.72E-01	1.57E-08	PPA2	-8.11E-02	1.00E+00	F2R	-4.19E-02	1.00E+00
RASGRP4	2.02E+00	1.59E-08	DTL	3.40E-02	1.00E+00	DDX28	-2.94E-02	1.00E+00
ZNF592	6.03E-01	1.62E-08	TMEM203	3.76E-02	1.00E+00	FAM222B	2.59E-02	1.00E+00
KDM4C	6.16E-01	1.63E-08	TEX11	3.83E-02	1.00E+00	TYK2	-3.13E-02	1.00E+00
LMO4	4.97E-01	1.63E-08	C3H17orf58	-3.68E-02	1.00E+00	SEPSECS	2.82E-02	1.00E+00
SF3A1	6.06E-01	1.63E-08	HECTD1	-3.36E-02	1.00E+00	WAPL	2.10E-02	1.00E+00
LOC112585882	2.24E+00	1.63E-08	PARK7	-2.94E-02	1.00E+00	PROSER2	-2.99E-02	1.00E+00
CARMIL3	1.25E+00	1.69E-08	PHKG2	-7.16E-02	1.00E+00	SFT2D2	-5.10E-02	1.00E+00
SPI1	-3.24E+00	1.70E-08	TMEM200A	2.31E-01	1.00E+00	RNF144B	-3.54E-02	1.00E+00
LOC102416013	-6.08E-01	1.70E-08	DARS	-4.02E-02	1.00E+00	UTP4	2.27E-02	1.00E+00
C1H21orf58	-1.92E+00	1.72E-08	ASAH1	4.93E-02	1.00E+00	THAP2	-2.74E-02	1.00E+00
SLC25A39	-8.07E-01	1.73E-08	PCSK6	7.37E-02	1.00E+00	LOC102409010	3.69E-02	1.00E+00
COG6	9.37E-01	1.77E-08	DBP	7.14E-02	1.00E+00	NDUFS5	3.77E-02	1.00E+00
AFAP1	-1.28E+00	1.79E-08	CNKSR2	-6.33E-02	1.00E+00	PFDN2	2.62E-02	1.00E+00
SLC25A36	-7.04E-	1.80E-08	NPR2	-6.63E-	1.00E+00	HOXD8	3.21E-02	1.00E+00

	01			02				
CHTOP	-6.24E-01	1.80E-08	LRP12	-1.63E-01	1.00E+00	POLR2J	-3.00E-02	1.00E+00
ARMC5	-7.81E-01	1.81E-08	LOC102392359	5.38E-02	1.00E+00	TTI1	2.50E-02	1.00E+00
VPS35L	-5.08E-01	1.82E-08	SYN3	4.27E-02	1.00E+00	TSR2	-2.67E-02	1.00E+00
ABHD12	-7.07E-01	1.82E-08	LOC112578054	-8.67E-02	1.00E+00	CTR9	-2.15E-02	1.00E+00
LOC102411979	8.48E-01	1.83E-08	UTRN	-8.82E-02	1.00E+00	GARNL3	2.78E-02	1.00E+00
FZD3	-6.44E-01	1.83E-08	TRAPPC1	3.70E-02	1.00E+00	DMBT1	1.22E-01	1.00E+00
CAMK2B	1.29E+00	1.84E-08	BRD9	5.55E-02	1.00E+00	ZDHHC9	2.29E-02	1.00E+00
DRAM1	-2.84E+00	1.85E-08	AFAP1	-5.84E-02	1.00E+00	ZBTB21	-3.69E-02	1.00E+00
PCP4	-2.99E+00	1.86E-08	HIPK2	4.68E-02	1.00E+00	MED23	-3.14E-02	1.00E+00
METTL6	5.92E-01	1.86E-08	PNPLA3	6.36E-02	1.00E+00	PPM1M	-2.80E-02	1.00E+00
NDUFA4L2	-2.97E+00	1.86E-08	KCTD16	5.97E-02	1.00E+00	POLR1B	-2.50E-02	1.00E+00
DDRGK1	8.17E-01	1.87E-08	AP3D1	-2.85E-02	1.00E+00	NFU1	-2.60E-02	1.00E+00
SYNGR2	-6.51E-01	1.87E-08	FAM84A	-1.29E-01	1.00E+00	ZNF300	4.12E-02	1.00E+00
SEC22B	-5.32E-01	1.89E-08	C18H16orf74	-3.21E-02	1.00E+00	SKIL	4.09E-02	1.00E+00
LRRC40	-1.05E+00	1.91E-08	CSTF2	-2.93E-02	1.00E+00	SSR3	2.05E-02	1.00E+00
ENTPD1	-2.92E+00	1.92E-08	CRYBB1	7.85E-02	1.00E+00	RAB18	-2.25E-02	1.00E+00
FKBPL	-1.34E+00	1.95E-08	SLC38A2	8.61E-02	1.00E+00	SPECC1L	2.15E-02	1.00E+00
ACAN	-2.51E+00	1.97E-08	TIMM17A	-2.72E-02	1.00E+00	CHFR	-2.06E-02	1.00E+00
HEG1	-8.99E-01	1.97E-08	PEX12	-2.62E-02	1.00E+00	GORASP1	-2.12E-02	1.00E+00
LOC112577767	-1.46E+00	1.98E-08	FRMD4B	4.14E-02	1.00E+00	PEX11A	4.10E-02	1.00E+00
IZUMO4	1.53E+00	1.99E-08	CREBZF	6.44E-02	1.00E+00	CSAD	2.64E-02	1.00E+00
ADCY1	-3.91E+00	2.00E-08	SERTAD1	-5.33E-02	1.00E+00	KIAA1328	2.41E-02	1.00E+00
LOC112580859	3.17E+00	2.00E-08	RPL24	-4.25E-02	1.00E+00	HOXD3	-5.26E-02	1.00E+00
LOC102398313	-2.89E+00	2.01E-08	PANX3	9.82E-02	1.00E+00	BBIP1	-2.49E-02	1.00E+00
KATNAL1	5.73E-01	2.01E-08	CDK7	-3.02E-02	1.00E+00	HSD17B12	-2.24E-02	1.00E+00
HEATR5B	1.03E+00	2.01E-08	TMEM35B	-4.76E-02	1.00E+00	OTUD6B	-2.16E-02	1.00E+00
MXD1	-1.45E+00	2.02E-08	CDK5RAP1	-2.45E-02	1.00E+00	ACP2	-2.41E-02	1.00E+00
FANCI	6.50E-01	2.04E-08	RTF2	-3.05E-02	1.00E+00	FNTB	-2.18E-02	1.00E+00
LOC102415727	-5.28E+00	2.05E-08	LOC112578798	-6.98E-02	1.00E+00	ARSK	-4.39E-02	1.00E+00
C4H12orf45	-1.39E+00	2.07E-08	ARNT2	-9.57E-02	1.00E+00	FCF1	-2.54E-02	1.00E+00
WFDC3	-1.15E+00	2.08E-08	GPBP1	-2.72E-02	1.00E+00	EEF1A1	-3.43E-02	1.00E+00
LOC102398867	1.39E+00	2.10E-08	SCN9A	-7.96E-02	1.00E+00	VAC14	2.17E-02	1.00E+00

CD46	-6.78E-01	2.12E-08	PTPRCAP	1.33E-01	1.00E+00	METTL26	3.20E-02	1.00E+00
NOTCH2	9.01E-01	2.14E-08	ARHGEF28	3.09E-02	1.00E+00	SNAP29	2.19E-02	1.00E+00
PHF3	-7.98E-01	2.14E-08	PLA2G7	6.16E-02	1.00E+00	TAF10	-3.02E-02	1.00E+00
LOC112578031	2.40E+00	2.17E-08	LOC102404904	1.11E-01	1.00E+00	MINDY2	-3.60E-02	1.00E+00
SDHAF2	-7.70E-01	2.17E-08	HELB	-4.38E-02	1.00E+00	THAP1	-2.48E-02	1.00E+00
LDB1	5.42E-01	2.19E-08	ENOX1	-3.23E-02	1.00E+00	LOC102409570	-2.97E-02	1.00E+00
THG1L	5.59E-01	2.19E-08	RALA	-2.36E-02	1.00E+00	LOC102402624	2.13E-02	1.00E+00
BCL2	1.11E+00	2.24E-08	APC	4.76E-02	1.00E+00	CCDC43	2.34E-02	1.00E+00
ZFYVE28	-2.11E+00	2.27E-08	CC2D2A	-1.17E-01	1.00E+00	KIAA0391	2.26E-02	1.00E+00
VIPAS39	4.80E-01	2.29E-08	ZBTB25	8.04E-02	1.00E+00	LIN7A	4.48E-02	1.00E+00
LOC112579161	-2.82E+00	2.30E-08	MRPS2	4.05E-02	1.00E+00	CACTIN	2.09E-02	1.00E+00
LOC102404060	1.61E+00	2.31E-08	PFKFB3	1.20E-01	1.00E+00	PPIH	2.89E-02	1.00E+00
PPP1R12C	7.18E-01	2.32E-08	MAGEF1	7.12E-02	1.00E+00	SDAD1	-2.27E-02	1.00E+00
IQSEC3	2.75E+00	2.32E-08	PHGDH	-1.93E-01	1.00E+00	CCDC102A	-2.78E-02	1.00E+00
PIK3R4	-5.55E-01	2.33E-08	DLX4	3.63E-02	1.00E+00	POPDC3	5.31E-02	1.00E+00
HSPA4	5.79E-01	2.33E-08	SNRNP35	4.08E-02	1.00E+00	TTLL1	-2.53E-02	1.00E+00
LOC102390198	-2.57E+00	2.33E-08	VWA8	5.07E-02	1.00E+00	HCK	-9.09E-02	1.00E+00
LOC102402675	2.34E+00	2.35E-08	SLC25A4	4.32E-02	1.00E+00	PCDH10	3.78E-02	1.00E+00
LOC112577692	1.02E+00	2.35E-08	CCBE1	4.11E-02	1.00E+00	APOOL	-2.10E-02	1.00E+00
LOC112587832	3.53E+00	2.39E-08	PYCR2	3.19E-02	1.00E+00	KIF1A	5.12E-02	1.00E+00
GTF2A1	-9.76E-01	2.40E-08	TMEM106A	3.97E-02	1.00E+00	DDX51	2.66E-02	1.00E+00
SEPSECS	7.28E-01	2.42E-08	OTUB1	-2.49E-02	1.00E+00	ABRAXAS1	-2.16E-02	1.00E+00
LOC102401651	3.14E+00	2.42E-08	CBX1	-2.57E-02	1.00E+00	DSTN	-2.65E-02	1.00E+00
SAR1A	-5.27E-01	2.42E-08	FAM184A	-2.80E-02	1.00E+00	LPAR2	4.70E-02	1.00E+00
LOC102413816	-1.78E+00	2.43E-08	NFKB2	3.72E-02	1.00E+00	LOC102394582	-2.03E-02	1.00E+00
MYMK	-2.87E+00	2.43E-08	SELENOP	-5.40E-02	1.00E+00	CHRM3	4.94E-02	1.00E+00
LOC102397527	-1.46E+00	2.48E-08	LOC112582130	3.57E-02	1.00E+00	GALNT2	2.20E-02	1.00E+00
ZKSCAN1	-7.81E-01	2.51E-08	MED4	-2.80E-02	1.00E+00	TRAPPC11	-2.50E-02	1.00E+00
SELENOW	-8.93E-01	2.54E-08	TMEM115	2.97E-02	1.00E+00	TFIP11	-2.06E-02	1.00E+00
ASL	-1.41E+00	2.54E-08	AGT	-6.98E-02	1.00E+00	PRDM10	-3.78E-02	1.00E+00
LOC112581441	-1.41E+00	2.55E-08	LOC112586818	-8.86E-02	1.00E+00	FMO4	2.91E-02	1.00E+00
TTC27	5.01E-01	2.55E-08	PEX16	4.76E-02	1.00E+00	PPP2R1B	2.24E-02	1.00E+00
LOC112586543	9.21E-01	2.56E-08	PNN	-2.32E-02	1.00E+00	SLC38A10	-2.51E-02	1.00E+00
RNF139	5.73E-01	2.56E-08	SS18	4.89E-02	1.00E+00	LOC102395480	-8.74E-02	1.00E+00
PDE1C	2.79E+00	2.57E-08	BECN1	2.96E-02	1.00E+00	GTF3A	-2.51E-	1.00E+00

							02	
LOC102392581	8.05E-01	2.58E-08	TIMM22	5.94E-02	1.00E+00	LIN37	2.97E-02	1.00E+00
ZNF580	-1.69E+00	2.60E-08	TMEM242	3.17E-02	1.00E+00	PGBD1	-2.30E-02	1.00E+00
TMEM251	7.48E-01	2.60E-08	NCBP3	-2.70E-02	1.00E+00	OGA	-2.27E-02	1.00E+00
CARS	-5.82E-01	2.67E-08	MIA3	2.72E-02	1.00E+00	ACAA1	-3.14E-02	1.00E+00
NPTXR	1.51E+00	2.68E-08	TARBP2	-3.19E-02	1.00E+00	CDH23	-6.68E-02	1.00E+00
TLR5	-2.24E+00	2.70E-08	NSD2	2.68E-02	1.00E+00	MRPL38	2.53E-02	1.00E+00
SLC39A11	-7.29E-01	2.70E-08	NOP10	-4.97E-02	1.00E+00	CTNNA2	2.74E-02	1.00E+00
LOC102409452	-6.74E-01	2.78E-08	SELL	-5.31E-02	1.00E+00	ZNF333	2.62E-02	1.00E+00
EVA1A	-2.46E+00	2.83E-08	PSEN2	4.90E-02	1.00E+00	AGPAT1	2.10E-02	1.00E+00
LOC112586282	-1.71E+00	2.84E-08	EPHA8	-1.28E-01	1.00E+00	GTF3C5	2.27E-02	1.00E+00
NEK4	-6.09E-01	2.87E-08	KHNYN	1.40E-01	1.00E+00	MTMR10	2.18E-02	1.00E+00
LOC102408914	1.23E+00	2.93E-08	MORN4	5.08E-02	1.00E+00	SERPINE3	4.82E-02	1.00E+00
C12H2orf73	2.50E+00	2.96E-08	BDH2	-2.13E-01	1.00E+00	TMEM203	2.66E-02	1.00E+00
LOC102396291	-2.01E+00	2.97E-08	LRIG1	4.29E-02	1.00E+00	YWHAZ	3.05E-02	1.00E+00
TOB2	-1.02E+00	3.06E-08	SAP18	2.71E-02	1.00E+00	LARP4B	-2.02E-02	1.00E+00
MGAT1	-5.25E-01	3.06E-08	TUBE1	-3.05E-02	1.00E+00	EEF2	-3.64E-02	1.00E+00
EXOSC3	-7.93E-01	3.07E-08	EN1	-1.30E-01	1.00E+00	PTGES2	-2.94E-02	1.00E+00
RRP15	6.52E-01	3.07E-08	IKZF3	-9.49E-02	1.00E+00	NOP14	2.25E-02	1.00E+00
SMYD4	8.14E-01	3.09E-08	LOC102399369	1.43E-01	1.00E+00	TM2D1	-2.16E-02	1.00E+00
UGP2	4.93E-01	3.09E-08	INPP5A	6.88E-02	1.00E+00	LOC102406763	2.69E-02	1.00E+00
UBQLN2	-6.90E-01	3.10E-08	TMEM141	-7.07E-02	1.00E+00	RAC3	-3.50E-02	1.00E+00
SUCLG2	-5.72E-01	3.12E-08	LOC102404976	-3.08E-02	1.00E+00	BMP2K	-2.38E-02	1.00E+00
CYGB	-2.68E+00	3.12E-08	LRRC10	-1.66E-01	1.00E+00	BICRA	-2.82E-02	1.00E+00
RASGRF1	3.02E+00	3.14E-08	LOC102393257	5.56E-02	1.00E+00	UBXN2B	-3.60E-02	1.00E+00
ALDH4A1	-6.55E-01	3.15E-08	WDR13	2.56E-02	1.00E+00	MORN1	-2.75E-02	1.00E+00
LOC102391810	2.60E+00	3.16E-08	KIAA0930	-1.31E-01	1.00E+00	NUDC	3.05E-02	1.00E+00
ATP5MC3	-6.73E-01	3.19E-08	FAM135A	-3.62E-02	1.00E+00	EFHC1	-2.82E-02	1.00E+00
TNS2	-6.98E-01	3.21E-08	LATS1	1.04E-01	1.00E+00	LOC112584602	5.51E-02	1.00E+00
LOC112587907	-2.59E+00	3.22E-08	PRPF8	-3.97E-02	1.00E+00	PLEKHG6	3.41E-02	1.00E+00
ENKD1	-8.80E-01	3.29E-08	LIF	-8.95E-02	1.00E+00	ICMT	-2.95E-02	1.00E+00
LOC102409122	-2.73E+00	3.31E-08	LOC102406005	1.70E-01	1.00E+00	TUFM	2.75E-02	1.00E+00
CCDC175	3.14E+00	3.31E-08	EIF4A2	3.41E-02	1.00E+00	LOC102408544	4.92E-02	1.00E+00
DNAJB9	-6.33E-01	3.37E-08	MAPKAPK5	9.58E-02	1.00E+00	SNU13	-2.56E-02	1.00E+00

FABP5	1.32E+00	3.38E-08	LSM4	-3.76E-02	1.00E+00	METTL9	2.08E-02	1.00E+00
LOC112586257	1.80E+00	3.39E-08	PRKCSH	3.28E-02	1.00E+00	C1RL	6.73E-02	1.00E+00
EIF1	-8.15E-01	3.40E-08	LAMP1	2.41E-02	1.00E+00	SLC50A1	-3.54E-02	1.00E+00
SMARCA2	-7.04E-01	3.40E-08	KPNA1	-4.47E-02	1.00E+00	SIPA1L3	-2.54E-02	1.00E+00
SLC35A3	-1.14E+00	3.53E-08	ITGA1	-5.54E-02	1.00E+00	CCDC167	3.70E-02	1.00E+00
COX3	-1.64E+00	3.56E-08	LOC102414136	-3.83E-02	1.00E+00	ZNF646	-2.56E-02	1.00E+00
ACTR3B	-7.46E-01	3.63E-08	LOC112578863	-4.20E-02	1.00E+00	KANSL1	-2.18E-02	1.00E+00
SETD7	-9.99E-01	3.64E-08	LRRC1	-3.90E-02	1.00E+00	IL9R	-4.40E-02	1.00E+00
TMEM104	-7.04E-01	3.64E-08	TMEM100	5.42E-02	1.00E+00	LOC102410433	-4.77E-02	1.00E+00
CNTN3	1.66E+00	3.70E-08	JKAMP	-2.95E-02	1.00E+00	HNRNPK	-2.21E-02	1.00E+00
SEC23IP	-5.87E-01	3.73E-08	ZSCAN25	-5.14E-02	1.00E+00	LOC112582279	4.06E-02	1.00E+00
CPNE7	1.99E+00	3.75E-08	JAGN1	-3.64E-02	1.00E+00	LOC112579930	-4.89E-02	1.00E+00
EDC4	5.37E-01	3.76E-08	LOC102391514	-3.09E-02	1.00E+00	VOPP1	-2.12E-02	1.00E+00
DCAF5	-5.97E-01	3.76E-08	RAPSN	-7.11E-02	1.00E+00	RB1	2.86E-02	1.00E+00
SPSB2	9.03E-01	3.77E-08	TRAPPC8	2.95E-02	1.00E+00	MGRN1	-2.13E-02	1.00E+00
GRK4	-1.10E+00	3.82E-08	PFKM	-5.92E-02	1.00E+00	BLCAP	2.04E-02	1.00E+00
LOC102402015	-1.36E+00	3.83E-08	FAR1	-4.58E-02	1.00E+00	MSH3	-2.13E-02	1.00E+00
LYSMD2	1.15E+00	3.84E-08	LOC112585722	-1.15E-01	1.00E+00	RUVBL2	3.02E-02	1.00E+00
SPTBN5	-2.15E+00	3.86E-08	EIF3A	2.82E-02	1.00E+00	CCHCR1	-2.98E-02	1.00E+00
EMC8	6.04E-01	3.87E-08	MTAP	3.19E-02	1.00E+00	RAB21	2.17E-02	1.00E+00
PCNX1	-6.41E-01	3.93E-08	THAP9	2.48E-02	1.00E+00	RTF2	-2.75E-02	1.00E+00
GSAP	-1.89E+00	3.93E-08	AHI1	7.19E-02	1.00E+00	LOC102399336	4.94E-02	1.00E+00
DRD2	4.05E+00	3.94E-08	TRIO	-2.64E-02	1.00E+00	MYNN	2.56E-02	1.00E+00
ADCY2	-1.97E+00	4.10E-08	FAM204A	3.78E-02	1.00E+00	LOC102406450	4.84E-02	1.00E+00
BBS9	-9.63E-01	4.13E-08	MARS	2.33E-02	1.00E+00	TAF13	2.26E-02	1.00E+00
LOC112584472	-2.81E+00	4.15E-08	ERI3	-3.54E-02	1.00E+00	PEX6	1.98E-02	1.00E+00
VPS33A	-5.92E-01	4.21E-08	RPP14	1.03E-01	1.00E+00	COQ10A	2.05E-02	1.00E+00
RFXAP	7.20E-01	4.25E-08	LOC102410803	-5.04E-02	1.00E+00	TP53BP2	2.28E-02	1.00E+00
MMP24	2.08E+00	4.26E-08	HECA	3.40E-02	1.00E+00	KIAA0895	-3.08E-02	1.00E+00
ZCCHC2	8.76E-01	4.34E-08	ACOT13	-5.76E-02	1.00E+00	TRIM27	1.82E-02	1.00E+00
LOC102390371	-1.27E+00	4.40E-08	TECPR1	4.25E-02	1.00E+00	PSMG1	2.37E-02	1.00E+00
SELENOI	5.76E-01	4.44E-08	ZNF512	-3.01E-02	1.00E+00	RPL18	-4.02E-02	1.00E+00
OAT	6.24E-01	4.53E-08	LOC112583852	1.17E-01	1.00E+00	LOC102401659	-3.60E-02	1.00E+00

CAMKK2	-1.23E+00	4.55E-08	MTBP	5.81E-02	1.00E+00	NOB1	2.69E-02	1.00E+00
RNF165	-2.15E+00	4.55E-08	C3H8orf58	-7.01E-02	1.00E+00	NNT	-2.31E-02	1.00E+00
CTNNA3	2.00E+00	4.56E-08	SYT9	2.32E-02	1.00E+00	GLMP	-1.92E-02	1.00E+00
PROSER2	-9.58E-01	4.60E-08	OAF	-3.94E-02	1.00E+00	LPP	3.04E-02	1.00E+00
TMEM214	-5.98E-01	4.64E-08	SEMA4D	-5.91E-02	1.00E+00	AES	2.73E-02	1.00E+00
ELOC	-6.72E-01	4.64E-08	SBK2	-2.78E-02	1.00E+00	SAPCD2	-3.43E-02	1.00E+00
PHETA1	-8.28E-01	4.64E-08	ATRNL1	6.65E-02	1.00E+00	KLF16	2.30E-02	1.00E+00
MAPK13	9.76E-01	4.64E-08	SVIL	3.07E-02	1.00E+00	LOC112582945	-4.00E-02	1.00E+00
FGF9	-1.63E+00	4.68E-08	INKA2	-7.94E-02	1.00E+00	LOC102402796	3.57E-02	1.00E+00
WDFY3	8.15E-01	4.69E-08	LAMTOR1	3.56E-02	1.00E+00	STK24	-1.87E-02	1.00E+00
ZBTB6	1.38E+00	4.70E-08	PPM1H	-2.56E-02	1.00E+00	MTCH2	1.91E-02	1.00E+00
SMAD6	1.04E+00	4.72E-08	NEIL3	2.98E-02	1.00E+00	USP39	1.90E-02	1.00E+00
RGS14	2.06E+00	4.72E-08	LOC112583810	1.80E-01	1.00E+00	UXT	3.42E-02	1.00E+00
PLD4	-4.90E+00	4.77E-08	SHISAL1	-8.35E-02	1.00E+00	SYTL2	6.14E-02	1.00E+00
AMN1	-5.61E-01	4.84E-08	DCUN1D4	2.48E-02	1.00E+00	WDR93	-3.64E-02	1.00E+00
C8H7orf57	1.67E+00	4.89E-08	EEFSEC	3.37E-02	1.00E+00	MLLT1	1.85E-02	1.00E+00
LOC102392936	3.38E+00	4.91E-08	CLIC4	2.69E-02	1.00E+00	NCKAP1	2.47E-02	1.00E+00
HIPK4	-1.37E+00	4.92E-08	LOC112587770	-4.61E-02	1.00E+00	DDHD1	-2.35E-02	1.00E+00
IRF1	-2.30E+00	4.95E-08	LRRC4B	8.69E-02	1.00E+00	ASB14	-4.58E-02	1.00E+00
LOC112581745	-2.39E+00	4.98E-08	TSKS	-7.27E-02	1.00E+00	EXOSC3	2.61E-02	1.00E+00
IQGAP1	-9.12E-01	5.13E-08	LOC102408293	7.63E-02	1.00E+00	RIPK1	-3.14E-02	1.00E+00
UBE2L3	6.01E-01	5.22E-08	ARHGDIA	2.27E-02	1.00E+00	CNTNAP4	4.98E-02	1.00E+00
LOC112585111	3.80E+00	5.24E-08	AHCYL2	3.18E-02	1.00E+00	LOC102413072	4.44E-02	1.00E+00
CILP2	-2.10E+00	5.30E-08	WDR20	-3.27E-02	1.00E+00	GSDMB	4.82E-02	1.00E+00
LOC112581735	1.40E+00	5.31E-08	BEND6	-1.09E-01	1.00E+00	ZFAND2B	-2.56E-02	1.00E+00
CSDE1	6.88E-01	5.34E-08	PRKN	4.60E-02	1.00E+00	GPM6A	5.89E-02	1.00E+00
LOC102413646	-2.53E+00	5.35E-08	DEDD	2.24E-02	1.00E+00	CCNDBP1	2.04E-02	1.00E+00
C4H1orf131	-7.69E-01	5.38E-08	TMEM131	4.07E-02	1.00E+00	IL17RE	-3.98E-02	1.00E+00
GZMA	3.31E+00	5.40E-08	LOC102394017	-1.13E-01	1.00E+00	DYDC2	-5.05E-02	1.00E+00
NUP58	7.13E-01	5.40E-08	ATP6AP1L	1.84E-01	1.00E+00	PHF20	-2.26E-02	1.00E+00
NDUFS4	-8.53E-01	5.48E-08	PSMB3	-3.46E-02	1.00E+00	XRN1	2.88E-02	1.00E+00
ECSIT	8.81E-01	5.52E-08	BORCS8	7.73E-02	1.00E+00	ZNF613	2.61E-02	1.00E+00
LOC102412762	-1.31E+00	5.53E-08	CUL9	-3.23E-02	1.00E+00	PUS7	-2.56E-02	1.00E+00
WDR88	2.72E+00	5.53E-08	LOC102414664	4.57E-02	1.00E+00	GTF3C2	2.00E-02	1.00E+00
FAM20A	-1.74E+00	5.54E-08	LOC112582918	-9.59E-02	1.00E+00	CARMIL1	-2.40E-02	1.00E+00

SGPL1	1.17E+00	5.54E-08	RAB5IF	-4.05E-02	1.00E+00	NDUFB8	2.38E-02	1.00E+00
RPUSD4	-5.65E-01	5.55E-08	TBCCD1	1.20E-01	1.00E+00	LOC102411578	6.22E-02	1.00E+00
PHOSPHO2	1.87E+00	5.56E-08	PKIA	8.55E-02	1.00E+00	PARP16	-2.03E-02	1.00E+00
SENP6	-5.16E-01	5.66E-08	DUPD1	-5.99E-02	1.00E+00	RPL3	-4.07E-02	1.00E+00
VKORC1L1	-6.40E-01	5.74E-08	MRI1	-5.57E-02	1.00E+00	LOC102416404	-2.70E-02	1.00E+00
RNF4	5.25E-01	5.75E-08	DERL1	2.65E-02	1.00E+00	CSKMT	3.52E-02	1.00E+00
TAF6L	-8.27E-01	5.77E-08	MCM9	3.31E-02	1.00E+00	CBFA2T2	-2.56E-02	1.00E+00
LOC102415697	3.89E+00	5.83E-08	SKA3	-3.24E-02	1.00E+00	LOC112581781	-3.55E-02	1.00E+00
RGP1	5.37E-01	5.83E-08	HSPG2	1.13E-01	1.00E+00	CIR1	-2.16E-02	1.00E+00
TBC1D23	-6.42E-01	5.93E-08	KIZ	-2.85E-02	1.00E+00	ZFYVE16	3.14E-02	1.00E+00
GLIPR1	-2.38E+00	5.99E-08	TAOK1	-5.57E-02	1.00E+00	LOC102391122	4.34E-02	1.00E+00
GRIA3	-1.06E+00	6.10E-08	ADAMTS9	6.49E-02	1.00E+00	SLC39A4	4.42E-02	1.00E+00
SAXO2	-1.40E+00	6.13E-08	KBTBD13	9.28E-02	1.00E+00	LOC102413806	-3.08E-02	1.00E+00
FOXJ1	-3.09E+00	6.15E-08	UXS1	3.55E-02	1.00E+00	LOC102390690	2.73E-02	1.00E+00
C2H6orf222	1.82E+00	6.16E-08	DCAF16	6.96E-02	1.00E+00	LOC102397151	-4.72E-02	1.00E+00
LOC102408478	-2.58E+00	6.18E-08	FKBP11	-9.76E-02	1.00E+00	TRIM11	2.00E-02	1.00E+00
SCNN1B	3.46E+00	6.24E-08	LOC112579925	5.86E-02	1.00E+00	PAPSS1	-1.98E-02	1.00E+00
SLC22A7	-3.71E+00	6.32E-08	CD46	3.36E-02	1.00E+00	FSD1	3.79E-02	1.00E+00
LOC112585115	-2.31E+00	6.32E-08	LOC102396420	8.51E-02	1.00E+00	SV2B	-7.33E-02	1.00E+00
DMGDH	2.60E+00	6.36E-08	SLC39A6	2.83E-02	1.00E+00	DERL2	-2.20E-02	1.00E+00
LOC112580193	4.17E+00	6.37E-08	SIVA1	-4.29E-02	1.00E+00	NOP9	-2.13E-02	1.00E+00
NSMCE1	-7.33E-01	6.39E-08	LOC102406175	3.25E-02	1.00E+00	PQLC2	2.59E-02	1.00E+00
UBR1	-1.10E+00	6.39E-08	LOC112585593	-1.61E-01	1.00E+00	EBF4	-2.41E-02	1.00E+00
DECR2	7.67E-01	6.41E-08	LOC102411767	7.99E-02	1.00E+00	PPFIA1	-2.03E-02	1.00E+00
SLC15A3	-4.67E+00	6.43E-08	SSH1	3.32E-02	1.00E+00	DNAJB14	2.99E-02	1.00E+00
COBL	1.32E+00	6.45E-08	KPTN	5.29E-02	1.00E+00	TRAPPC6B	2.24E-02	1.00E+00
PUM2	5.98E-01	6.46E-08	COBL	5.84E-02	1.00E+00	LOC102397238	-2.89E-02	1.00E+00
LOC102393752	-3.12E+00	6.48E-08	ITM2C	-4.83E-02	1.00E+00	EPC2	2.20E-02	1.00E+00
LOC112586900	-5.01E+00	6.48E-08	PWWP2A	-3.43E-02	1.00E+00	AASDHPPT	1.84E-02	1.00E+00
LOC102407467	7.60E-01	6.57E-08	BPGM	2.64E-02	1.00E+00	CEP350	2.66E-02	1.00E+00
LRP2BP	1.25E+00	6.57E-08	AIMP1	-3.69E-02	1.00E+00	LACTB2	-2.52E-02	1.00E+00
FOXP4	-6.45E-01	6.65E-08	DOK5	2.65E-02	1.00E+00	ANAPC1	2.04E-02	1.00E+00
RIMKLA	6.37E-01	6.67E-08	NINJ2	6.12E-02	1.00E+00	WRNIP1	-2.19E-02	1.00E+00

HECTD3	-6.42E-01	6.73E-08	PRELID1	2.86E-02	1.00E+00	CEP170	3.20E-02	1.00E+00
LOC112584448	1.26E+00	6.76E-08	SDF4	-3.70E-02	1.00E+00	PHF14	-1.83E-02	1.00E+00
RETN	-4.04E+00	6.80E-08	LOC112586200	-4.99E-02	1.00E+00	DCAF13	1.91E-02	1.00E+00
SLIT1	1.47E+00	6.90E-08	LOC112580893	-1.44E-01	1.00E+00	LOC112578240	4.67E-02	1.00E+00
GOLPH3	6.79E-01	6.96E-08	ZNF260	6.45E-02	1.00E+00	LOC102405660	-2.65E-02	1.00E+00
SLC11A1	-4.41E+00	6.96E-08	SIAH1	-3.19E-02	1.00E+00	DNAH17	-2.79E-02	1.00E+00
LOC102391695	-4.17E+00	7.15E-08	ELMOD1	6.89E-02	1.00E+00	VTA1	2.02E-02	1.00E+00
CDH17	-2.14E+00	7.15E-08	PRPF40A	2.30E-02	1.00E+00	LOC102406662	3.24E-02	1.00E+00
ERGIC2	4.72E-01	7.22E-08	DPH6	4.26E-02	1.00E+00	LOC102392455	3.43E-02	1.00E+00
PAPPA2	3.25E+00	7.22E-08	FBXO22	-3.14E-02	1.00E+00	KDM8	2.71E-02	1.00E+00
KIAA1958	-5.78E-01	7.32E-08	TRIM9	-6.03E-02	1.00E+00	RASSF3	-3.38E-02	1.00E+00
CSRP2	-6.94E-01	7.33E-08	HDAC10	-4.36E-02	1.00E+00	SNRPA	-2.45E-02	1.00E+00
UNKL	-7.45E-01	7.37E-08	SND1	2.59E-02	1.00E+00	LIN7C	-2.43E-02	1.00E+00
MAGI1	7.22E-01	7.39E-08	ARHGEF19	1.28E-01	1.00E+00	CNOT6	-2.18E-02	1.00E+00
LOC102400385	-1.03E+00	7.47E-08	STRADB	-2.81E-02	1.00E+00	SETDB2	2.42E-02	1.00E+00
ZNF575	-7.77E-01	7.49E-08	DCUN1D3	5.95E-02	1.00E+00	LOC102407467	-2.67E-02	1.00E+00
PSMD11	4.73E-01	7.56E-08	KIAA0040	-3.81E-02	1.00E+00	UBR2	2.54E-02	1.00E+00
BHLHE41	-2.72E+00	7.61E-08	RAB27A	2.29E-02	1.00E+00	TMEM65	2.59E-02	1.00E+00
LIF	-1.59E+00	7.62E-08	CHRM1	1.44E-01	1.00E+00	SNED1	2.98E-02	1.00E+00
PSME2	-8.15E-01	7.64E-08	TSTD1	-4.46E-02	1.00E+00	FAM177A1	-2.11E-02	1.00E+00
LOC102392703	-1.74E+00	7.77E-08	TDRD12	-5.52E-02	1.00E+00	ZNF704	-4.17E-02	1.00E+00
MID1	-1.01E+00	7.94E-08	TXNRD1	-3.60E-02	1.00E+00	NAXE	2.44E-02	1.00E+00
FNTB	-5.52E-01	7.94E-08	PAX5	-1.67E-01	1.00E+00	PTPRCAP	4.22E-02	1.00E+00
XRCC4	6.12E-01	7.97E-08	CHN2	-1.15E-01	1.00E+00	PDAP1	2.54E-02	1.00E+00
DOHH	-7.79E-01	7.99E-08	MEF2C	3.50E-02	1.00E+00	BCAP31	2.62E-02	1.00E+00
GGA2	4.90E-01	8.23E-08	FNDC5	1.12E-01	1.00E+00	SYMPK	-1.90E-02	1.00E+00
LHCGR	-2.87E+00	8.25E-08	RACGAP1	2.82E-02	1.00E+00	KLK10	-4.63E-02	1.00E+00
GRB2	-4.55E-01	8.27E-08	SMIM15	-5.02E-02	1.00E+00	SUPT6H	1.78E-02	1.00E+00
MTRF1	7.42E-01	8.35E-08	GABPA	5.66E-02	1.00E+00	ETAA1	2.29E-02	1.00E+00
LOC102412461	5.43E-01	8.40E-08	LOC102403202	5.47E-02	1.00E+00	SLC33A1	2.37E-02	1.00E+00
RCAN2	-2.12E+00	8.40E-08	ALG3	-5.00E-02	1.00E+00	MFSD14B	-2.22E-02	1.00E+00
EIF4A3	-5.81E-01	8.47E-08	SLC44A2	3.14E-02	1.00E+00	NAV1	2.05E-02	1.00E+00
FNBP1	-1.19E+00	8.59E-08	GEMIN6	-4.85E-02	1.00E+00	STK25	2.40E-02	1.00E+00

CCR7	2.86E+00	8.65E-08	RNF113A	-4.22E-02	1.00E+00	SLC3A1	-2.09E-02	1.00E+00
ALOX5	-2.76E+00	8.67E-08	MAB21L3	-1.07E-01	1.00E+00	LOC112586986	-3.68E-02	1.00E+00
C12H2orf81	3.09E+00	8.73E-08	STAT4	1.06E-01	1.00E+00	LOC112587028	-7.46E-02	1.00E+00
LPIN1	-1.04E+00	8.85E-08	SEC63	-3.69E-02	1.00E+00	SERPING1	2.34E-02	1.00E+00
GEMIN8	6.92E-01	8.90E-08	DENND6B	-5.84E-02	1.00E+00	ZDHHC5	-1.98E-02	1.00E+00
RALGAPA1	7.06E-01	8.92E-08	SYT10	6.31E-02	1.00E+00	RASA1	2.31E-02	1.00E+00
HP1BP3	-5.41E-01	8.94E-08	NLRP14	-2.89E-02	1.00E+00	PHC3	-2.60E-02	1.00E+00
ARHGAP4	1.80E+00	9.00E-08	TSHZ3	-1.36E-01	1.00E+00	FBXO8	-2.24E-02	1.00E+00
TRPC4AP	5.22E-01	9.04E-08	ARMC7	2.30E-02	1.00E+00	LRRC58	6.28E-02	1.00E+00
PRDM2	8.26E-01	9.10E-08	CMTM6	-3.93E-02	1.00E+00	PSMD7	-2.19E-02	1.00E+00
SFRP4	-3.86E+00	9.13E-08	COPS4	-2.36E-02	1.00E+00	ALDH4A1	-2.16E-02	1.00E+00
LOC102403707	-3.39E+00	9.23E-08	PIH1D1	-3.90E-02	1.00E+00	CDO1	-1.87E-02	1.00E+00
TMEM167A	5.36E-01	9.25E-08	F13A1	2.04E-01	1.00E+00	PRTG	-3.00E-02	1.00E+00
TMEM147	-7.98E-01	9.35E-08	ADCY8	-7.62E-02	1.00E+00	GLCCI1	2.73E-02	1.00E+00
PPP1R14D	2.22E+00	9.37E-08	PASK	-4.30E-02	1.00E+00	GGACT	3.29E-02	1.00E+00
LOC112581808	2.00E+00	9.40E-08	EXD3	5.23E-02	1.00E+00	LOC102416423	-4.78E-02	1.00E+00
LOC112583913	-2.99E+00	9.44E-08	IQCC	7.09E-02	1.00E+00	PPP2R5C	-1.77E-02	1.00E+00
PLEKHM1	6.62E-01	9.46E-08	MSI1	3.81E-02	1.00E+00	INTS12	-1.99E-02	1.00E+00
TUBB2A	8.28E-01	9.53E-08	TIPRL	-3.52E-02	1.00E+00	TMEM231	2.15E-02	1.00E+00
LOC102397670	-8.52E-01	9.55E-08	SOD2	2.13E-02	1.00E+00	ZNF410	1.72E-02	1.00E+00
ZNF598	5.51E-01	9.58E-08	LOC102414911	-9.18E-02	1.00E+00	DCAKD	-2.22E-02	1.00E+00
SLC15A5	3.28E+00	9.62E-08	UBE4A	-3.25E-02	1.00E+00	FBXO28	-2.31E-02	1.00E+00
WWP2	5.12E-01	9.63E-08	NIN	-4.39E-02	1.00E+00	ADSL	-1.86E-02	1.00E+00
E2F5	-9.65E-01	9.67E-08	B9D1	-6.56E-02	1.00E+00	DNAJC4	-3.01E-02	1.00E+00
ANKRD66	3.06E+00	9.87E-08	STXBP5	-2.53E-02	1.00E+00	SLC37A2	-6.69E-02	1.00E+00
DDIT3	-1.12E+00	9.91E-08	LOC112586852	1.30E-01	1.00E+00	POP7	-2.60E-02	1.00E+00
CLIC5	-2.69E+00	1.00E-07	CBFA2T2	3.36E-02	1.00E+00	ZNF526	2.66E-02	1.00E+00
ING4	-5.69E-01	1.02E-07	KIAA1217	2.31E-02	1.00E+00	HOOK3	-2.12E-02	1.00E+00
TMEM217	1.87E+00	1.02E-07	SEC24C	2.52E-02	1.00E+00	LOC102402857	-4.59E-02	1.00E+00
ATP5MPL	-8.63E-01	1.02E-07	ZHX1	-1.08E-01	1.00E+00	EIF3M	-2.33E-02	1.00E+00
GLT1D1	2.71E+00	1.03E-07	GALNT2	-3.11E-02	1.00E+00	MRPS10	-1.96E-02	1.00E+00
DDX56	5.75E-01	1.03E-07	LDHB	-2.99E-02	1.00E+00	DHRS13	-3.86E-02	1.00E+00
PAPSS1	-5.16E-01	1.04E-07	SLC11A2	3.18E-02	1.00E+00	FBXW2	1.88E-02	1.00E+00

TLCD2	1.41E+00	1.06E-07	GGNBP2	2.28E-02	1.00E+00	FBXL3	-2.71E-02	1.00E+00
MIA3	5.21E-01	1.06E-07	ZHX3	3.17E-02	1.00E+00	PDPK1	2.83E-02	1.00E+00
HTR1D	2.57E+00	1.11E-07	DOCK8	3.77E-02	1.00E+00	NPHS2	4.39E-02	1.00E+00
CABP1	-2.84E+00	1.11E-07	PRIM2	2.39E-02	1.00E+00	NSUN6	2.26E-02	1.00E+00
WDR72	-2.66E+00	1.12E-07	C3H17orf49	8.14E-02	1.00E+00	LOC102402736	4.05E-02	1.00E+00
CCPG1	-7.59E-01	1.12E-07	FUBP3	-6.35E-02	1.00E+00	CRBN	1.81E-02	1.00E+00
ANKRD28	-7.12E-01	1.12E-07	TDP2	-3.11E-02	1.00E+00	ANKRD27	-2.10E-02	1.00E+00
WDR36	-6.45E-01	1.13E-07	GNL2	-2.05E-02	1.00E+00	SLC6A8	2.07E-02	1.00E+00
PFN1	-7.41E-01	1.14E-07	NFYB	2.96E-02	1.00E+00	LOC102408184	-2.26E-02	1.00E+00
CCDC33	-1.81E+00	1.15E-07	PIAS4	2.21E-02	1.00E+00	TREM1	7.94E-02	1.00E+00
NAXD	-5.44E-01	1.15E-07	NUAK1	-1.44E-01	1.00E+00	OSR2	-5.74E-02	1.00E+00
ABL2	1.22E+00	1.15E-07	RALGAPB	3.53E-02	1.00E+00	ZNF787	-2.43E-02	1.00E+00
SDCBP	7.38E-01	1.15E-07	KCNJ11	1.64E-01	1.00E+00	PRSS55	4.40E-02	1.00E+00
PPP1R36	1.37E+00	1.15E-07	ATP5PD	-2.64E-02	1.00E+00	SLC25A26	-1.80E-02	1.00E+00
LOC112580450	-2.91E+00	1.17E-07	FADS6	-1.80E-01	1.00E+00	DPM3	2.73E-02	1.00E+00
L1CAM	2.27E+00	1.18E-07	TRIB2	1.59E-01	1.00E+00	EML2	2.14E-02	1.00E+00
CD3D	2.50E+00	1.19E-07	STUB1	2.82E-02	1.00E+00	UBE3C	-1.93E-02	1.00E+00
MTHFR	-9.16E-01	1.19E-07	LHFPL3	2.77E-02	1.00E+00	FAM210A	2.06E-02	1.00E+00
ZFP36L1	1.35E+00	1.20E-07	USP44	2.79E-02	1.00E+00	BAHCC1	-2.22E-02	1.00E+00
PERP	-1.45E+00	1.21E-07	SPNS1	-3.81E-02	1.00E+00	CASP7	2.35E-02	1.00E+00
FIGNL2	-1.74E+00	1.21E-07	TRIP12	3.55E-02	1.00E+00	CSNK2B	2.23E-02	1.00E+00
TERF2IP	5.38E-01	1.23E-07	KRT19	1.42E-01	1.00E+00	SLC39A6	-2.22E-02	1.00E+00
EFNA2	1.08E+00	1.23E-07	PRKCA	2.14E-02	1.00E+00	RBM4	-2.05E-02	1.00E+00
SREK1	-5.86E-01	1.25E-07	SLC30A4	5.79E-02	1.00E+00	AKAP14	4.51E-02	1.00E+00
COASY	7.59E-01	1.26E-07	SLC23A1	-7.69E-02	1.00E+00	PCYT2	2.64E-02	1.00E+00
LOC112585085	-2.00E+00	1.28E-07	LOC102392703	1.58E-01	1.00E+00	RNF20	-2.05E-02	1.00E+00
TACC1	-7.33E-01	1.29E-07	LOC102402398	-3.46E-02	1.00E+00	DPM2	2.38E-02	1.00E+00
CHTF18	7.67E-01	1.30E-07	SPHK1	-4.69E-02	1.00E+00	PSMD9	1.91E-02	1.00E+00
LOC112587815	3.20E+00	1.32E-07	METTL3	3.45E-02	1.00E+00	ATOH8	3.83E-02	1.00E+00
MRPS27	5.18E-01	1.35E-07	GTF2H4	-5.04E-02	1.00E+00	ARMC7	-2.33E-02	1.00E+00
NTM	2.50E+00	1.37E-07	RNF5	4.27E-02	1.00E+00	LOC102410517	-1.72E-02	1.00E+00
LOC102396444	-9.68E-01	1.38E-07	FAM53B	-2.29E-02	1.00E+00	ALKBH7	2.76E-02	1.00E+00
LOC102415288	1.65E+00	1.39E-07	PPARGC1A	-3.78E-02	1.00E+00	CASTOR1	-4.01E-02	1.00E+00
OCA2	2.29E+00	1.40E-07	ITGAE	-3.10E-02	1.00E+00	NCR3	3.87E-02	1.00E+00

PPIH	-7.89E-01	1.40E-07	BOLA3	-3.93E-02	1.00E+00	GATAD2A	1.72E-02	1.00E+00
ZNF382	-9.55E-01	1.40E-07	DNAH12	-9.69E-02	1.00E+00	MOGS	-2.18E-02	1.00E+00
GMNC	2.91E+00	1.41E-07	HTR7	8.96E-02	1.00E+00	RHBDL1	3.72E-02	1.00E+00
CCDC180	3.17E+00	1.43E-07	LCP1	4.60E-02	1.00E+00	C21H3orf62	-2.28E-02	1.00E+00
TENT5A	-1.66E+00	1.44E-07	LOC112577635	-9.57E-02	1.00E+00	HNRNPLL	-2.08E-02	1.00E+00
LOC102410567	3.42E+00	1.44E-07	ABI1	2.33E-02	1.00E+00	C15H8orf89	3.18E-02	1.00E+00
CAPN1	-5.18E-01	1.45E-07	LOC102405195	-4.80E-02	1.00E+00	ERGIC1	-1.75E-02	1.00E+00
LYPD5	-1.33E+00	1.46E-07	TMEM189	2.31E-02	1.00E+00	TFCP2	-1.93E-02	1.00E+00
PIGZ	-1.01E+00	1.47E-07	FARSB	-2.26E-02	1.00E+00	PPP4C	2.29E-02	1.00E+00
LOC112579717	-2.36E+00	1.48E-07	RBBP8	2.16E-02	1.00E+00	MACF1	-2.88E-02	1.00E+00
RASIP1	1.03E+00	1.48E-07	LOC102404010	-2.69E-02	1.00E+00	SMARCB1	-2.27E-02	1.00E+00
TIFAB	3.73E+00	1.49E-07	ALKBH2	2.89E-02	1.00E+00	SPRTN	-2.40E-02	1.00E+00
FDX2	7.82E-01	1.50E-07	SLC9A8	3.73E-02	1.00E+00	TBC1D13	1.81E-02	1.00E+00
FAM43A	1.17E+00	1.50E-07	NAGLU	-1.74E-01	1.00E+00	HNRNPR	-1.86E-02	1.00E+00
DBR1	6.33E-01	1.50E-07	DLGAP4	2.66E-02	1.00E+00	FGD1	1.81E-02	1.00E+00
UBE4B	-5.78E-01	1.52E-07	KLHL22	-5.87E-02	1.00E+00	TOR1AIP1	-2.31E-02	1.00E+00
LOC112578154	1.96E+00	1.53E-07	COLEC12	3.32E-02	1.00E+00	BTF3L4	-1.82E-02	1.00E+00
UROC1	-9.22E-01	1.53E-07	LOC102392624	-3.58E-02	1.00E+00	NDUFA6	2.71E-02	1.00E+00
LRP11	-1.01E+00	1.54E-07	LOXL2	1.32E-01	1.00E+00	EDN2	5.99E-02	1.00E+00
LOC102406738	6.32E-01	1.55E-07	TAGLN	4.82E-02	1.00E+00	RORA	-3.34E-02	1.00E+00
HSBP1L1	-1.07E+00	1.56E-07	LOC102395698	-3.41E-02	1.00E+00	PHB2	2.52E-02	1.00E+00
TUT4	-7.99E-01	1.57E-07	LOC112580462	9.51E-02	1.00E+00	LOC112587105	7.05E-02	1.00E+00
TROAP	7.35E-01	1.60E-07	PDXK	2.49E-02	1.00E+00	MRPS35	-1.92E-02	1.00E+00
PON1	3.58E+00	1.62E-07	TTPAL	-6.49E-02	1.00E+00	LOC102395519	3.21E-02	1.00E+00
YIF1B	-7.93E-01	1.63E-07	NKX1-1	-7.61E-02	1.00E+00	XPA	-2.13E-02	1.00E+00
L3HYPDH	9.17E-01	1.63E-07	NXT1	-2.44E-02	1.00E+00	LOC102409942	-3.10E-02	1.00E+00
TMEM218	-7.36E-01	1.65E-07	LOC102389840	-9.53E-02	1.00E+00	RHOBTB2	1.90E-02	1.00E+00
CCBE1	-9.58E-01	1.65E-07	CAPNS1	2.67E-02	1.00E+00	FCHO2	-3.17E-02	1.00E+00
BTAF1	7.73E-01	1.67E-07	ZFP92	9.89E-02	1.00E+00	LOC102411316	-3.88E-02	1.00E+00
TMEM71	-3.51E+00	1.68E-07	EIF3H	2.23E-02	1.00E+00	SNW1	-1.76E-02	1.00E+00
POMC	-1.11E+00	1.73E-07	PRDM16	1.15E-01	1.00E+00	SNRPF	3.04E-02	1.00E+00
XRCC3	-9.75E-01	1.74E-07	FIZ1	-7.67E-02	1.00E+00	VAMP2	-2.06E-02	1.00E+00
LOC102399551	-7.20E-01	1.74E-07	VPS13D	-4.90E-02	1.00E+00	LOC112584596	3.14E-02	1.00E+00
GTF2A2	6.63E-01	1.76E-07	HMGN4	-3.13E-	1.00E+00	LOC112585253	-4.54E-	1.00E+00

				02			02	
CCDC82	5.29E-01	1.76E-07	TMSB10	3.05E-02	1.00E+00	ELOB	2.83E-02	1.00E+00
TPRG1L	-7.06E-01	1.80E-07	RIPOR1	-4.31E-02	1.00E+00	ZDHHC12	2.37E-02	1.00E+00
CIB1	-8.23E-01	1.83E-07	RSRC2	2.40E-02	1.00E+00	RPS12	-2.95E-02	1.00E+00
GMIP	-1.25E+00	1.83E-07	FNBP1	6.62E-02	1.00E+00	APOBEC2	-3.86E-02	1.00E+00
ETFDH	-5.51E-01	1.84E-07	LOC102398979	-7.70E-02	1.00E+00	ORMDL2	2.19E-02	1.00E+00
CCL5	-3.86E+00	1.85E-07	EIF4E1B	-3.29E-02	1.00E+00	GPATCH2L	-2.31E-02	1.00E+00
HOXC6	-1.84E+00	1.86E-07	WDR48	2.45E-02	1.00E+00	NPNT	2.65E-02	1.00E+00
HAS2	1.84E+00	1.86E-07	GCC1	-2.42E-02	1.00E+00	NOL6	-1.96E-02	1.00E+00
UCHL5	-5.50E-01	1.88E-07	ALPL	-1.25E-01	1.00E+00	SYT1	4.10E-02	1.00E+00
AKT1	-5.85E-01	1.89E-07	MAPRE2	-2.43E-02	1.00E+00	LOC112577646	6.58E-02	1.00E+00
EIF3K	-7.34E-01	1.89E-07	SNX1	2.33E-02	1.00E+00	UGP2	1.78E-02	1.00E+00
GTF3C5	5.72E-01	1.90E-07	LOC112587025	-1.73E-01	1.00E+00	ZNF614	2.55E-02	1.00E+00
LOC102399202	-3.71E+00	1.93E-07	MSRB1	-6.22E-02	1.00E+00	DNAJC28	-3.24E-02	1.00E+00
ISOC1	-5.60E-01	1.94E-07	TTYH3	-1.39E-01	1.00E+00	ANKRD13A	-1.99E-02	1.00E+00
HMGN2	-9.96E-01	1.94E-07	GIGYF1	4.72E-02	1.00E+00	TRMT61B	3.87E-02	1.00E+00
MACROD2	6.21E-01	1.97E-07	ARHGAP10	2.60E-02	1.00E+00	LOC102400013	-5.72E-02	1.00E+00
SAP18	5.72E-01	1.98E-07	NEPRO	4.28E-02	1.00E+00	STX12	1.95E-02	1.00E+00
TLR7	-3.46E+00	1.99E-07	LOC102399836	-1.10E-01	1.00E+00	RSPO4	-4.37E-02	1.00E+00
GPRIN2	3.50E+00	2.00E-07	EXO1	-2.80E-02	1.00E+00	TBC1D17	1.67E-02	1.00E+00
GPN1	-4.99E-01	2.01E-07	NT5DC3	6.84E-02	1.00E+00	CYFIP1	1.78E-02	1.00E+00
LOC112581565	3.35E+00	2.01E-07	BUD31	-2.62E-02	1.00E+00	ILF2	2.00E-02	1.00E+00
C24H16orf45	2.37E+00	2.02E-07	APBA3	2.92E-02	1.00E+00	MCM8	-2.84E-02	1.00E+00
TLR4	-5.29E+00	2.04E-07	NME9	1.09E-01	1.00E+00	ZBTB7A	1.99E-02	1.00E+00
SLC24A1	9.42E-01	2.06E-07	SPPL2A	4.27E-02	1.00E+00	LRCH3	2.56E-02	1.00E+00
GABARAPL2	5.69E-01	2.09E-07	PDGFA	-2.75E-02	1.00E+00	ADD1	-1.81E-02	1.00E+00
LOC102409999	-4.51E+00	2.11E-07	CYTH3	-2.46E-02	1.00E+00	SMTN	1.96E-02	1.00E+00
LOC102401393	-6.51E-01	2.11E-07	TRIT1	-3.86E-02	1.00E+00	TBXAS1	-3.89E-02	1.00E+00
EIF5	-6.45E-01	2.12E-07	PRRT4	-5.43E-02	1.00E+00	TTC25	-2.84E-02	1.00E+00
PRKCD	-8.72E-01	2.12E-07	MYOM2	1.40E-01	1.00E+00	SDK1	-3.86E-02	1.00E+00
CHST1	1.58E+00	2.13E-07	LOC102413100	-1.04E-01	1.00E+00	ARPC1A	1.81E-02	1.00E+00
AOC2	1.08E+00	2.15E-07	RBM10	-3.37E-02	1.00E+00	LOC102398050	1.89E-02	1.00E+00
RPH3A	3.23E+00	2.15E-07	CHKA	2.63E-02	1.00E+00	ADGRL2	-2.00E-02	1.00E+00
LOC112581365	1.87E+00	2.16E-07	RAD9B	-1.65E-	1.00E+00	ENTPD1	-4.16E-	1.00E+00

				01			02	
OSR1	1.86E+00	2.19E-07	EGLN1	2.99E-02	1.00E+00	MRPS33	2.52E-02	1.00E+00
ANKRD39	-8.25E-01	2.20E-07	LDAH	-4.44E-02	1.00E+00	CD14	8.08E-02	1.00E+00
CDH26	2.63E+00	2.20E-07	SPAG8	-1.03E-01	1.00E+00	MED15	-1.76E-02	1.00E+00
LIPT1	1.05E+00	2.22E-07	LYN	-9.24E-02	1.00E+00	LSM5	2.65E-02	1.00E+00
PODXL2	-1.25E+00	2.24E-07	PRKACA	5.45E-02	1.00E+00	CACFD1	-2.21E-02	1.00E+00
SNRNP35	9.27E-01	2.24E-07	CINP	3.43E-02	1.00E+00	FAM136A	-2.09E-02	1.00E+00
LOC112583093	-2.83E+00	2.24E-07	NADSYN1	2.38E-01	1.00E+00	SPX	-3.62E-02	1.00E+00
SLC2A6	1.79E+00	2.25E-07	UAP1L1	-6.21E-02	1.00E+00	ZNF461	2.64E-02	1.00E+00
BCL6	-7.63E-01	2.25E-07	HJURP	-2.51E-02	1.00E+00	POFUT2	2.34E-02	1.00E+00
KIAA0556	-6.03E-01	2.26E-07	LOC112585182	6.12E-02	1.00E+00	TGFBR3	-2.14E-02	1.00E+00
ATRX	-8.70E-01	2.26E-07	EFNA3	6.89E-02	1.00E+00	FAM193A	-1.80E-02	1.00E+00
MRPL45	-6.32E-01	2.29E-07	ELP5	3.08E-02	1.00E+00	MAFB	-8.48E-02	1.00E+00
LOC112582095	-3.51E+00	2.29E-07	LOC112580815	-4.31E-02	1.00E+00	MFAP3	-2.47E-02	1.00E+00
CCDC153	1.79E+00	2.30E-07	MDH2	2.25E-02	1.00E+00	TAF1A	2.74E-02	1.00E+00
FAM3D	2.58E+00	2.30E-07	SYT1	5.01E-02	1.00E+00	LYRM7	-2.75E-02	1.00E+00
REEP1	1.50E+00	2.30E-07	CKAP2L	3.41E-02	1.00E+00	ZFYVE26	2.27E-02	1.00E+00
LOC102414136	-7.17E-01	2.31E-07	LOC102416551	1.05E-01	1.00E+00	C3H17orf80	-2.18E-02	1.00E+00
BRICD5	-1.94E+00	2.31E-07	CLSPN	3.06E-02	1.00E+00	MED28	-2.31E-02	1.00E+00
CTPS1	4.99E-01	2.31E-07	UNC5D	2.20E-02	1.00E+00	BCAS3	2.02E-02	1.00E+00
LRRC73	-1.91E+00	2.34E-07	EQTN	4.53E-02	1.00E+00	HIBCH	1.68E-02	1.00E+00
IFT43	-9.46E-01	2.34E-07	RTN4RL2	-1.45E-01	1.00E+00	AFMID	-2.22E-02	1.00E+00
FAM98C	-8.63E-01	2.36E-07	LOC102390996	-9.09E-02	1.00E+00	GTF2E2	-1.82E-02	1.00E+00
IL18R1	3.30E+00	2.36E-07	POLR3D	2.22E-02	1.00E+00	GCNT1	-2.43E-02	1.00E+00
LOC102414166	-3.10E+00	2.40E-07	LOC102406285	5.79E-02	1.00E+00	RPL8	-3.87E-02	1.00E+00
VASP	-6.01E-01	2.43E-07	ABCD4	-4.17E-02	1.00E+00	TTC4	1.80E-02	1.00E+00
UHMK1	8.12E-01	2.45E-07	ANGEL1	3.05E-02	1.00E+00	SLC5A5	-4.41E-02	1.00E+00
GPRC5B	1.24E+00	2.45E-07	COQ4	4.21E-02	1.00E+00	ESF1	-1.86E-02	1.00E+00
HMGA2	-1.81E+00	2.46E-07	NTNG1	-1.58E-01	1.00E+00	TMEM79	-1.85E-02	1.00E+00
ATG12	4.81E-01	2.47E-07	PHB2	2.33E-02	1.00E+00	LOC102399409	4.56E-02	1.00E+00
RBM22	4.45E-01	2.47E-07	CATSPERD	3.05E-02	1.00E+00	TLR6	3.67E-02	1.00E+00
PRDM11	-1.21E+00	2.52E-07	SUPT20H	-4.32E-02	1.00E+00	SNX13	2.45E-02	1.00E+00
CALCOCO1	-5.12E-01	2.53E-07	SLC4A8	5.18E-02	1.00E+00	SPAG9	-2.14E-02	1.00E+00
ADAM12	9.17E-01	2.54E-07	XRN2	-2.36E-02	1.00E+00	C3H9orf85	-3.28E-02	1.00E+00
PITPNM3	1.88E+00	2.56E-07	LOC112581646	-9.42E-	1.00E+00	TPP2	-2.02E-	1.00E+00

				02			02	
LOC112586852	3.39E+00	2.56E-07	LAMTOR3	-2.26E-02	1.00E+00	C3H17orf75	-2.05E-02	1.00E+00
DUSP8	-1.42E+00	2.57E-07	PIWIL2	-2.96E-02	1.00E+00	THAP3	2.51E-02	1.00E+00
ZBTB38	-1.31E+00	2.58E-07	KIAA0556	-3.95E-02	1.00E+00	WASHC1	2.07E-02	1.00E+00
NIPAL4	2.10E+00	2.59E-07	MRPL44	-2.75E-02	1.00E+00	LOC102392861	-2.92E-02	1.00E+00
CDC73	-5.13E-01	2.64E-07	LOC112586464	8.99E-02	1.00E+00	SVBP	2.57E-02	1.00E+00
BBC3	-1.20E+00	2.69E-07	CPS1	-3.22E-02	1.00E+00	PAGR1	-2.72E-02	1.00E+00
CASD1	6.43E-01	2.71E-07	GTF3C4	-5.78E-02	1.00E+00	AFF4	-2.76E-02	1.00E+00
LOC112578437	-2.79E+00	2.72E-07	KIAA1328	3.00E-02	1.00E+00	RNF32	3.77E-02	1.00E+00
XRCC5	-5.45E-01	2.75E-07	SSBP4	4.98E-02	1.00E+00	LOC112582139	3.82E-02	1.00E+00
ZNF10	-1.46E+00	2.76E-07	LOC102411901	-8.88E-02	1.00E+00	UBA52	2.58E-02	1.00E+00
HNRNPC	-6.09E-01	2.76E-07	ACOXL	3.87E-02	1.00E+00	LOC112581735	-4.25E-02	1.00E+00
TRIM62	1.21E+00	2.76E-07	PKN1	3.03E-02	1.00E+00	MTX1	-2.38E-02	1.00E+00
CACNA1G	-2.56E+00	2.77E-07	TIMM10B	-4.17E-02	1.00E+00	RNF115	-1.79E-02	1.00E+00
TRIM67	2.02E+00	2.82E-07	IRF2BP2	-4.79E-02	1.00E+00	UROD	2.57E-02	1.00E+00
DCX	-1.33E+00	2.83E-07	BRD8	2.13E-02	1.00E+00	ZDHHC2	-1.88E-02	1.00E+00
ZFYVE19	7.96E-01	2.83E-07	MYO9B	3.76E-02	1.00E+00	ACSL5	6.64E-02	1.00E+00
ZNF7	-5.85E-01	2.86E-07	PRTG	8.87E-02	1.00E+00	SCPEP1	-1.77E-02	1.00E+00
ACOT7	-7.48E-01	2.88E-07	B4GALT7	7.34E-02	1.00E+00	BMPR1A	-1.67E-02	1.00E+00
WIPI2	5.07E-01	2.93E-07	RAD23B	-2.29E-02	1.00E+00	CCDC88C	-2.25E-02	1.00E+00
DRD3	2.87E+00	2.94E-07	ERCC4	3.43E-02	1.00E+00	LOC102398394	-1.95E-02	1.00E+00
SMG1	8.27E-01	2.94E-07	FAU	3.37E-02	1.00E+00	ATXN10	1.65E-02	1.00E+00
ASB3	5.64E-01	2.95E-07	ZNF41	-1.80E-01	1.00E+00	METTL3	-1.69E-02	1.00E+00
LOC102398273	-8.84E-01	2.96E-07	SPINDOC	2.80E-02	1.00E+00	IQSEC2	-2.47E-02	1.00E+00
APOBR	-1.33E+00	2.98E-07	OSBPL3	2.70E-02	1.00E+00	ACADVL	1.99E-02	1.00E+00
E2F8	-7.93E-01	3.01E-07	CDK4	5.06E-02	1.00E+00	THUMPD1	-1.95E-02	1.00E+00
SOGA3	-2.19E+00	3.05E-07	LOC112579067	-1.24E-01	1.00E+00	PHIP	2.18E-02	1.00E+00
LAPTM4A	-4.76E-01	3.14E-07	ARHGAP39	3.40E-02	1.00E+00	LOC102399019	-4.20E-02	1.00E+00
KCNIP4	-9.31E-01	3.14E-07	PBX4	3.83E-02	1.00E+00	VMA21	2.20E-02	1.00E+00
TMCO1	5.96E-01	3.15E-07	KDSR	-4.42E-02	1.00E+00	MGAT4A	3.82E-02	1.00E+00
LOC112580673	1.57E+00	3.20E-07	ROM1	-3.92E-02	1.00E+00	PDCL3	-1.85E-02	1.00E+00
BCLAF3	6.52E-01	3.23E-07	AMH	-1.02E-01	1.00E+00	AP2B1	1.79E-02	1.00E+00
CCDC134	6.70E-01	3.25E-07	TRPT1	4.09E-02	1.00E+00	LY9	8.83E-02	1.00E+00
ARL5B	-8.93E-	3.25E-07	LOC112581224	-7.27E-	1.00E+00	PPP1R37	1.74E-02	1.00E+00

	01			02				
SDHAF3	8.59E-01	3.26E-07	CGAS	-2.97E-02	1.00E+00	MMP19	-5.10E-02	1.00E+00
FXR1	-4.51E-01	3.28E-07	PAOX	5.63E-02	1.00E+00	LOC102392615	1.76E-02	1.00E+00
LMOD1	-2.12E+00	3.29E-07	NDRG3	-2.82E-02	1.00E+00	SRMS	2.78E-02	1.00E+00
DCAF17	8.08E-01	3.29E-07	ALG2	-2.96E-02	1.00E+00	TRIM24	1.86E-02	1.00E+00
PDE7A	-6.43E-01	3.32E-07	SMARCE1	2.27E-02	1.00E+00	SLF2	2.02E-02	1.00E+00
MRPS33	-7.96E-01	3.32E-07	BBS9	5.81E-02	1.00E+00	SLC15A4	-1.65E-02	1.00E+00
CD72	1.16E+00	3.38E-07	OGFRL1	-4.86E-02	1.00E+00	QSER1	1.89E-02	1.00E+00
LOC102404275	1.27E+00	3.39E-07	DBNDD2	5.18E-02	1.00E+00	TEX261	1.83E-02	1.00E+00
GRIA4	-2.16E+00	3.42E-07	CTXN3	-4.46E-02	1.00E+00	MRPS15	2.46E-02	1.00E+00
NDUFB6	7.90E-01	3.42E-07	STAG3	4.45E-02	1.00E+00	STK39	2.04E-02	1.00E+00
TBCB	6.13E-01	3.43E-07	PLEKHA3	4.81E-02	1.00E+00	JPT2	-1.61E-02	1.00E+00
RAB5IF	-7.59E-01	3.47E-07	LOC112581825	5.23E-02	1.00E+00	LOC102404784	-4.35E-02	1.00E+00
VSTM2B	1.99E+00	3.49E-07	ADCY5	6.25E-02	1.00E+00	SUPT5H	1.55E-02	1.00E+00
PSMA3	6.12E-01	3.50E-07	ZDHHC21	6.64E-02	1.00E+00	ANKRD46	-1.88E-02	1.00E+00
SCOC	5.64E-01	3.52E-07	NANP	-4.78E-02	1.00E+00	NDUFA7	-2.13E-02	1.00E+00
LOC112583463	-1.99E+00	3.56E-07	TCF25	-2.14E-02	1.00E+00	SLC7A7	3.32E-02	1.00E+00
ZNF613	-7.92E-01	3.62E-07	SNIP1	-3.33E-02	1.00E+00	ATF2	-1.80E-02	1.00E+00
PRRC2C	-9.26E-01	3.63E-07	ANKS1A	2.68E-02	1.00E+00	KLF3	-2.32E-02	1.00E+00
TALDO1	-7.81E-01	3.63E-07	LOC112583775	-5.36E-02	1.00E+00	EMX2	1.67E-02	1.00E+00
LOC102402465	-9.01E-01	3.67E-07	LOC102416224	-3.27E-02	1.00E+00	ADAMTS1	4.44E-02	1.00E+00
ALKBH6	-8.98E-01	3.69E-07	EDAR	-7.88E-02	1.00E+00	TMEM221	-3.43E-02	1.00E+00
SLC24A3	-1.21E+00	3.72E-07	ZBTB3	6.14E-02	1.00E+00	IST1	1.67E-02	1.00E+00
PIGU	6.33E-01	3.72E-07	SRBD1	-2.59E-02	1.00E+00	CERS1	-2.51E-02	1.00E+00
LOC112586464	2.38E+00	3.75E-07	PDGFRL	-1.07E-01	1.00E+00	UBFD1	-1.64E-02	1.00E+00
CREM	5.85E-01	3.81E-07	IQSEC2	-2.99E-02	1.00E+00	MAST3	3.06E-02	1.00E+00
B3GALT4	-8.81E-01	3.84E-07	TMEM186	-4.95E-02	1.00E+00	NQO2	-1.89E-02	1.00E+00
LOC102403309	2.23E+00	3.85E-07	NR4A2	1.57E-01	1.00E+00	LOC112579121	-3.85E-02	1.00E+00
TJAP1	5.41E-01	3.87E-07	SERTAD2	-1.39E-01	1.00E+00	LOC112585503	-3.23E-02	1.00E+00
CD209	-3.55E+00	3.87E-07	FBXO32	-3.29E-02	1.00E+00	LCAT	-2.11E-02	1.00E+00
LOC112584566	1.04E+00	3.87E-07	RALB	-2.17E-02	1.00E+00	CWC22	1.81E-02	1.00E+00
C3H9orf3	5.77E-01	3.87E-07	POU4F1	-5.57E-02	1.00E+00	SHANK3	-2.75E-02	1.00E+00
DAPP1	-2.28E+00	3.89E-07	RRP9	-2.46E-02	1.00E+00	U2AF1	-2.11E-02	1.00E+00
SSR3	-4.89E-	3.90E-07	PSMD5	-2.01E-	1.00E+00	LOC112581369	-4.08E-	1.00E+00

	01			02			02	
SCRN3	-1.07E+00	3.93E-07	TANGO6	-4.38E-02	1.00E+00	CD53	7.95E-02	1.00E+00
MARCH11	-2.06E+00	3.94E-07	DNAJC2	2.31E-02	1.00E+00	KCNT1	5.89E-02	1.00E+00
SNTG2	-3.37E+00	3.97E-07	RASL11A	-7.97E-02	1.00E+00	BORCS5	1.91E-02	1.00E+00
QTRT2	-6.94E-01	3.99E-07	LOC112584602	-1.12E-01	1.00E+00	SP2	2.06E-02	1.00E+00
SUSD6	6.60E-01	4.05E-07	ANKRD49	2.38E-02	1.00E+00	PPA1	-2.20E-02	1.00E+00
ASB7	-6.79E-01	4.05E-07	TAF4B	-4.79E-02	1.00E+00	SLC18A2	2.71E-02	1.00E+00
SLC19A1	-9.98E-01	4.07E-07	TMED1	-6.05E-02	1.00E+00	KSR1	2.21E-02	1.00E+00
PPIL3	-8.62E-01	4.09E-07	FBXL13	-5.27E-02	1.00E+00	GCNA	-2.45E-02	1.00E+00
PPP4R2	6.70E-01	4.11E-07	RABAC1	3.67E-02	1.00E+00	CSNK1G1	-2.05E-02	1.00E+00
LOC112584718	2.04E+00	4.14E-07	F3	3.72E-02	1.00E+00	NCF1	-5.42E-02	1.00E+00
PLIN2	8.16E-01	4.16E-07	TERT	-3.33E-02	1.00E+00	ZNF397	-2.16E-02	1.00E+00
SUOX	6.03E-01	4.17E-07	TKFC	3.03E-02	1.00E+00	SLC29A3	2.95E-02	1.00E+00
LOC102398572	2.29E+00	4.18E-07	AKAP7	-6.22E-02	1.00E+00	NOXA1	-2.53E-02	1.00E+00
C24H16orf91	-7.67E-01	4.18E-07	RBM22	-2.23E-02	1.00E+00	THOC5	1.69E-02	1.00E+00
LOC102414521	-2.81E+00	4.19E-07	MRVI1	2.12E-02	1.00E+00	USP25	2.10E-02	1.00E+00
CD226	3.05E+00	4.19E-07	SMO	4.69E-02	1.00E+00	SMCHD1	-1.90E-02	1.00E+00
PQLC3	-1.67E+00	4.21E-07	DCAF10	-8.48E-02	1.00E+00	PLEKHJ1	2.34E-02	1.00E+00
MARCH10	7.91E-01	4.22E-07	DGKB	-6.06E-02	1.00E+00	IDH3A	-1.62E-02	1.00E+00
LAS1L	-6.36E-01	4.23E-07	ART1	-1.10E-01	1.00E+00	PPT2	2.17E-02	1.00E+00
AGPAT2	-2.50E+00	4.24E-07	GUCY1B1	7.47E-02	1.00E+00	PSMD14	1.58E-02	1.00E+00
ZFYVE27	-4.91E-01	4.31E-07	TRMT2A	-3.06E-02	1.00E+00	SAP30BP	-1.56E-02	1.00E+00
ATP8	-1.24E+00	4.37E-07	LOC112586644	-1.42E-01	1.00E+00	OXSM	-2.23E-02	1.00E+00
LRG1	-2.38E+00	4.39E-07	GSKIP	-3.42E-02	1.00E+00	GOLIM4	-2.22E-02	1.00E+00
NPY1R	-2.19E+00	4.39E-07	MRPS6	-3.24E-02	1.00E+00	RPL28	2.87E-02	1.00E+00
ABCD3	5.40E-01	4.43E-07	SRGN	1.42E-01	1.00E+00	PNPO	3.47E-02	1.00E+00
TACR3	-1.85E+00	4.43E-07	CDT1	-1.90E-02	1.00E+00	APBB1IP	6.12E-02	1.00E+00
LOC102393517	-6.79E-01	4.45E-07	KCNT1	5.45E-02	1.00E+00	ATF6	1.62E-02	1.00E+00
JCAD	-7.66E-01	4.46E-07	NECTIN4	9.45E-02	1.00E+00	GRK3	-2.59E-02	1.00E+00
NUDT5	-7.38E-01	4.51E-07	ITGB6	-3.60E-02	1.00E+00	SLC2A10	-2.40E-02	1.00E+00
LOC102393427	-9.06E-01	4.54E-07	VDAC1	-2.11E-02	1.00E+00	SLC7A1	-2.71E-02	1.00E+00
GABRA2	-2.87E+00	4.54E-07	SRPRA	2.24E-02	1.00E+00	KPTN	2.33E-02	1.00E+00
ENAH	5.26E-01	4.56E-07	RBBP6	-4.74E-02	1.00E+00	CFAP298	-1.90E-02	1.00E+00

TRIQK	5.96E-01	4.72E-07	LOC102405282	-7.12E-02	1.00E+00	DUSP22	2.26E-02	1.00E+00
LOC102416239	-2.87E+00	4.73E-07	HINT3	9.27E-02	1.00E+00	ZNF45	2.00E-02	1.00E+00
PCGF3	4.37E-01	4.75E-07	LMO3	2.00E-02	1.00E+00	SNX14	-1.61E-02	1.00E+00
PSENEN	-7.30E-01	4.76E-07	DENND1C	2.39E-02	1.00E+00	DEXI	1.83E-02	1.00E+00
REXO5	-5.57E-01	4.78E-07	P3H4	6.18E-02	1.00E+00	ORC4	-1.71E-02	1.00E+00
LOC102389307	-1.64E+00	4.82E-07	ZNF805	-4.25E-02	1.00E+00	WDSUB1	-1.56E-02	1.00E+00
TMEM138	-7.67E-01	4.83E-07	GALNT6	1.57E-01	1.00E+00	SEPT2	-1.75E-02	1.00E+00
CCDC85B	-1.34E+00	4.83E-07	PATZ1	-3.57E-02	1.00E+00	SPATC1L	3.15E-02	1.00E+00
ZNF664	-6.00E-01	4.84E-07	SYNGR1	-3.77E-02	1.00E+00	TBC1D22A	-1.88E-02	1.00E+00
SLC1A7	-2.53E+00	4.88E-07	PMAIP1	5.73E-02	1.00E+00	IFT57	-1.79E-02	1.00E+00
PRUNE1	-5.67E-01	4.89E-07	LOC102400319	-8.33E-02	1.00E+00	OSBPL5	1.58E-02	1.00E+00
SLC22A18	-2.18E+00	4.92E-07	ANXA7	2.32E-02	1.00E+00	LOC102395880	2.64E-02	1.00E+00
RYR1	1.27E+00	4.94E-07	ABRAXAS2	2.03E-02	1.00E+00	YPEL5	-1.98E-02	1.00E+00
MYO1E	-7.57E-01	4.98E-07	LOC102405455	7.26E-02	1.00E+00	IGFBP7	2.09E-02	1.00E+00
KLF2	1.79E+00	5.03E-07	TOM1	2.20E-02	1.00E+00	LOC112582114	4.36E-02	1.00E+00
LOC102389028	-8.16E-01	5.04E-07	LOC102416331	2.31E-02	1.00E+00	MOB3A	1.75E-02	1.00E+00
LOC112586971	4.32E+00	5.06E-07	CDK20	2.64E-02	1.00E+00	CFAP69	-2.45E-02	1.00E+00
LOC102390085	1.14E+00	5.08E-07	CD3E	-3.81E-02	1.00E+00	GLYCTK	2.60E-02	1.00E+00
SYS1	-5.74E-01	5.09E-07	STK38	-4.18E-02	1.00E+00	USP37	-2.23E-02	1.00E+00
SPOCK1	5.48E-01	5.09E-07	MEPCE	1.97E-02	1.00E+00	ERAL1	-2.02E-02	1.00E+00
LOC112583859	2.76E+00	5.12E-07	URB1	5.22E-02	1.00E+00	CORO1A	5.20E-02	1.00E+00
FAM69B	-1.93E+00	5.19E-07	LOC102403810	5.56E-02	1.00E+00	MAK16	3.10E-02	1.00E+00
LOC102399600	3.68E+00	5.20E-07	LOC112581883	4.04E-02	1.00E+00	FAM172A	-2.01E-02	1.00E+00
LOC112587308	-8.60E-01	5.22E-07	UBAP1L	6.63E-02	1.00E+00	LTA4H	-1.72E-02	1.00E+00
PHB2	-7.32E-01	5.24E-07	VPS37B	2.31E-02	1.00E+00	SBF1	-1.64E-02	1.00E+00
LRRC4C	2.05E+00	5.28E-07	CDHR1	3.44E-02	1.00E+00	SERF2	-2.59E-02	1.00E+00
WDR86	-1.37E+00	5.44E-07	LOC102412047	-4.67E-02	1.00E+00	ZNF329	1.69E-02	1.00E+00
LOC112587546	-1.48E+00	5.46E-07	TXK	3.08E-02	1.00E+00	TXNL4B	-1.87E-02	1.00E+00
LRTM2	-2.70E+00	5.47E-07	SPAG17	-2.24E-02	1.00E+00	BRD3OS	2.38E-02	1.00E+00
UBE2D1	-8.15E-01	5.48E-07	UTP15	3.09E-02	1.00E+00	LYST	-2.53E-02	1.00E+00
DRG2	-5.84E-01	5.51E-07	GABARAPL2	-2.15E-02	1.00E+00	TNFRSF4	3.67E-02	1.00E+00
FBXL6	-8.41E-01	5.54E-07	ADCK5	4.47E-02	1.00E+00	MYO18B	-4.26E-02	1.00E+00
PHF19	-6.68E-01	5.54E-07	DTHD1	-2.84E-02	1.00E+00	KANSL3	-1.51E-02	1.00E+00

CD44	-4.72E+00	5.60E-07	TMEM30B	3.37E-02	1.00E+00	PPAT	1.47E-02	1.00E+00
GMPPA	-6.02E-01	5.65E-07	DEPDC7	-2.35E-02	1.00E+00	WWOX	2.17E-02	1.00E+00
LOC102392787	-4.73E+00	5.66E-07	PSMD3	2.46E-02	1.00E+00	LOC102409318	3.84E-02	1.00E+00
GOSR2	-5.42E-01	5.70E-07	RBCK1	-5.87E-02	1.00E+00	CALML4	2.65E-02	1.00E+00
COPB1	4.38E-01	5.70E-07	AIG1	4.51E-02	1.00E+00	CHRAC1	-2.08E-02	1.00E+00
SEPT7	-4.68E-01	5.73E-07	CFAP69	-9.08E-02	1.00E+00	NDUFV3	-1.53E-02	1.00E+00
EFNA4	-1.25E+00	5.76E-07	SETD6	6.14E-02	1.00E+00	BOLA3	2.16E-02	1.00E+00
LOC102416651	-7.89E-01	5.80E-07	ISCA2	6.55E-02	1.00E+00	MED30	1.88E-02	1.00E+00
BMPR1A	4.22E-01	5.83E-07	DMXL2	-2.31E-02	1.00E+00	SP4	-2.72E-02	1.00E+00
LOC112582252	2.73E+00	5.85E-07	PPP3R1	2.66E-02	1.00E+00	SIRT1	-2.29E-02	1.00E+00
TAS1R3	9.86E-01	5.86E-07	CCDC93	1.80E-02	1.00E+00	PIGQ	1.75E-02	1.00E+00
C7H4orf50	2.19E+00	5.87E-07	IRS4	-1.29E-01	1.00E+00	ID4	3.64E-02	1.00E+00
OSM	-3.94E+00	5.88E-07	RRM2B	4.79E-02	1.00E+00	VSIG4	-7.37E-02	1.00E+00
LOC112580414	-2.54E+00	5.88E-07	BAG2	-2.56E-02	1.00E+00	PANX1	-1.76E-02	1.00E+00
C18H16orf87	-1.94E+00	5.92E-07	MESP1	-6.00E-02	1.00E+00	PICK1	1.91E-02	1.00E+00
TRHDE	3.11E+00	6.01E-07	LOC112583698	-8.38E-02	1.00E+00	MTX3	-4.00E-02	1.00E+00
KIF1BP	-5.47E-01	6.13E-07	CDK19	-3.26E-02	1.00E+00	ANKRD16	2.12E-02	1.00E+00
COQ9	-6.67E-01	6.22E-07	DTWD2	-8.05E-02	1.00E+00	CHMP3	-1.65E-02	1.00E+00
C17H22orf39	-7.94E-01	6.25E-07	DDA1	-2.39E-02	1.00E+00	FUOM	-2.43E-02	1.00E+00
MSR1	-3.82E+00	6.37E-07	AAED1	-8.16E-02	1.00E+00	QDPR	-1.84E-02	1.00E+00
LOC102408542	-2.85E+00	6.43E-07	TSGA10IP	9.11E-02	1.00E+00	DROSHA	-1.61E-02	1.00E+00
IARS	4.78E-01	6.44E-07	AMDHD1	-1.20E-01	1.00E+00	PKN2	-2.12E-02	1.00E+00
THAP12	-1.70E+00	6.45E-07	NEK3	-3.20E-02	1.00E+00	SCAP	-1.80E-02	1.00E+00
TMEM9	-5.80E-01	6.46E-07	WDR91	2.74E-02	1.00E+00	EIF4G1	1.75E-02	1.00E+00
NCBP3	4.42E-01	6.61E-07	RNF126	2.46E-02	1.00E+00	RELA	1.82E-02	1.00E+00
LOC102398515	-2.91E+00	6.62E-07	UBE2Z	1.86E-02	1.00E+00	FBXO2	-3.53E-02	1.00E+00
CPSF4	5.11E-01	6.63E-07	RRP1	2.23E-02	1.00E+00	ASAP3	4.19E-02	1.00E+00
LOC102400054	2.67E+00	6.63E-07	MOS	-2.39E-02	1.00E+00	CENPM	-2.65E-02	1.00E+00
LOC102389262	-9.91E-01	6.67E-07	FAM192A	-2.18E-02	1.00E+00	ZNF84	-2.65E-02	1.00E+00
CBX4	-6.91E-01	6.69E-07	CYSTM1	2.33E-02	1.00E+00	PPP1R16A	-1.75E-02	1.00E+00
UNC5A	1.71E+00	6.73E-07	SNRPC	3.38E-02	1.00E+00	CWC27	-2.66E-02	1.00E+00
LOC112582243	3.26E+00	6.76E-07	TTC37	-3.26E-02	1.00E+00	LRRC42	-1.58E-02	1.00E+00
MARCH5	-5.50E-01	6.76E-07	TMEFF2	-5.55E-02	1.00E+00	RANBP9	-1.79E-02	1.00E+00

WTIP	-6.22E-01	6.77E-07	MYO1D	-2.36E-02	1.00E+00	SIPA1L1	1.64E-02	1.00E+00
LOC112578149	-3.58E+00	6.79E-07	LRP11	-5.82E-02	1.00E+00	DGCR8	-1.65E-02	1.00E+00
FBXO21	-5.09E-01	6.81E-07	SLC44A1	5.46E-02	1.00E+00	FAU	-2.89E-02	1.00E+00
SDF2L1	8.97E-01	6.86E-07	ITIH2	-1.37E-01	1.00E+00	RWDD2A	-2.82E-02	1.00E+00
MSN	-7.99E-01	6.94E-07	MAP2K3	3.24E-02	1.00E+00	SRD5A3	-2.04E-02	1.00E+00
LOC112585223	3.42E+00	6.94E-07	LOC102410356	2.59E-02	1.00E+00	MCTS1	-2.07E-02	1.00E+00
TMEM154	2.80E+00	6.94E-07	POLR2B	-2.50E-02	1.00E+00	SLC38A6	-2.37E-02	1.00E+00
SART1	-5.33E-01	6.97E-07	GK	4.37E-02	1.00E+00	LOC102415739	3.77E-02	1.00E+00
CLIC6	3.34E+00	7.02E-07	LAMA3	2.26E-02	1.00E+00	UBE2D3	-1.60E-02	1.00E+00
LOC112579065	-8.48E-01	7.08E-07	PHYH	-4.47E-02	1.00E+00	CIAO1	2.65E-02	1.00E+00
LOC112578809	1.51E+00	7.32E-07	ZNFX1	-4.44E-02	1.00E+00	KIAA1143	1.81E-02	1.00E+00
GRPR	3.01E+00	7.33E-07	MRPL33	3.22E-02	1.00E+00	SERPINB1	2.52E-02	1.00E+00
TTC17	6.09E-01	7.33E-07	TTK	2.01E-02	1.00E+00	STARD10	-2.99E-02	1.00E+00
AP1S1	-6.87E-01	7.36E-07	LOC102389068	5.53E-02	1.00E+00	LOC102393672	-1.64E-02	1.00E+00
TRIM23	-8.57E-01	7.37E-07	INTS14	8.22E-02	1.00E+00	FAM162A	2.41E-02	1.00E+00
GPCPD1	6.53E-01	7.38E-07	KIFAP3	-2.40E-02	1.00E+00	ZNF570	2.48E-02	1.00E+00
LRRTM4	2.83E+00	7.50E-07	MAFK	3.14E-02	1.00E+00	LOC102398622	-2.44E-02	1.00E+00
LOC112579951	1.41E+00	7.51E-07	RGL2	7.36E-02	1.00E+00	EPB41L5	-1.53E-02	1.00E+00
SIL1	-7.67E-01	7.51E-07	SP6	2.11E-02	1.00E+00	USP34	-1.85E-02	1.00E+00
LOC112582086	2.72E+00	7.72E-07	DCAF13	2.32E-02	1.00E+00	DPF2	1.42E-02	1.00E+00
FAM189A1	1.68E+00	7.80E-07	MEX3B	1.28E-01	1.00E+00	TUBG2	-2.08E-02	1.00E+00
TMEM62	-6.61E-01	7.83E-07	PMM2	-2.18E-02	1.00E+00	PRRT1	3.84E-02	1.00E+00
BCAS1	3.05E+00	7.88E-07	LOC112583873	-1.47E-01	1.00E+00	MMADHC	-1.52E-02	1.00E+00
DMKN	-1.40E+00	7.92E-07	AKAP9	-4.78E-02	1.00E+00	PARP4	1.89E-02	1.00E+00
GFOD2	-1.02E+00	7.93E-07	FAM43A	-4.65E-02	1.00E+00	PGD	2.18E-02	1.00E+00
GLRA3	2.97E+00	7.94E-07	PSMD13	-2.17E-02	1.00E+00	RPL21	-3.41E-02	1.00E+00
AP4B1	6.16E-01	7.98E-07	RIPK4	4.32E-02	1.00E+00	PFDN4	-1.93E-02	1.00E+00
PMM1	-6.77E-01	8.02E-07	LOC102399336	1.13E-01	1.00E+00	CTNNB1	-1.72E-02	1.00E+00
BRINP1	-2.47E+00	8.03E-07	RTTN	3.21E-02	1.00E+00	DNAJC10	-1.49E-02	1.00E+00
LOC102412769	-1.28E+00	8.03E-07	GAS6	7.48E-02	1.00E+00	LOC102401106	-3.89E-02	1.00E+00
EXOC6	5.38E-01	8.07E-07	DNAAF3	6.22E-02	1.00E+00	FRS2	1.95E-02	1.00E+00
RPP14	-1.34E+00	8.10E-07	EEF1AKMT3	-7.37E-02	1.00E+00	LOC102394187	-2.82E-02	1.00E+00
SPCS3	4.85E-01	8.10E-07	DAB2IP	2.96E-02	1.00E+00	LOC102397317	-2.59E-02	1.00E+00

LOC102416218	7.19E-01	8.12E-07	ME3	4.31E-02	1.00E+00	CNOT11	2.98E-02	1.00E+00
TGFB2	-2.57E+00	8.12E-07	TESK1	-2.57E-02	1.00E+00	CAPRIN1	-1.78E-02	1.00E+00
LOC102402218	-2.71E+00	8.21E-07	EIF4ENIF1	2.51E-02	1.00E+00	LZTR1	-1.53E-02	1.00E+00
RTCA	-4.98E-01	8.23E-07	LOC102409010	4.07E-02	1.00E+00	RPL32	3.23E-02	1.00E+00
LETMD1	4.72E-01	8.26E-07	LOC102412960	-9.40E-02	1.00E+00	C1H3orf33	1.89E-02	1.00E+00
MIB1	6.61E-01	8.28E-07	GLP1R	4.96E-02	1.00E+00	SAG	-3.13E-02	1.00E+00
FRAT1	-1.24E+00	8.29E-07	HARS2	-4.00E-02	1.00E+00	KDELR3	1.65E-02	1.00E+00
PKHD1L1	-1.97E+00	8.30E-07	FAM193B	4.87E-02	1.00E+00	AHCY	1.85E-02	1.00E+00
DDIT4L	-1.36E+00	8.41E-07	LOC102408997	2.05E-02	1.00E+00	NACA	1.59E-02	1.00E+00
CAAP1	5.14E-01	8.45E-07	FOXA3	-1.26E-01	1.00E+00	PCMTD1	-1.78E-02	1.00E+00
AHCTF1	-7.25E-01	8.57E-07	LOC102395109	5.88E-02	1.00E+00	ABCA7	-3.39E-02	1.00E+00
LOC102404604	2.64E+00	8.58E-07	NDOR1	-3.34E-02	1.00E+00	ACP4	-2.64E-02	1.00E+00
MRE11	4.94E-01	8.60E-07	DDHD1	-2.68E-02	1.00E+00	PHEX	2.05E-02	1.00E+00
LOC112578089	-1.76E+00	8.66E-07	LOC102410234	3.02E-02	1.00E+00	FKBP8	2.11E-02	1.00E+00
RASSF9	-1.82E+00	8.68E-07	LOC102394043	8.44E-02	1.00E+00	GCAT	-3.10E-02	1.00E+00
SLC27A4	-7.83E-01	8.70E-07	DNAJC4	3.16E-02	1.00E+00	BICRAL	2.01E-02	1.00E+00
TMEM143	-7.84E-01	8.72E-07	RAP1GAP2	-9.51E-02	1.00E+00	NEK9	2.01E-02	1.00E+00
RILP	-1.91E+00	8.74E-07	PTMA	3.01E-02	1.00E+00	LOC112581858	-3.15E-02	1.00E+00
LOC112587328	-2.03E+00	8.85E-07	PJA1	4.64E-02	1.00E+00	TIFA	-3.41E-02	1.00E+00
TARBP2	7.49E-01	8.91E-07	LOC112582563	1.32E-01	1.00E+00	EIF3I	2.06E-02	1.00E+00
SNAI1	1.71E+00	8.97E-07	UBE2R2	2.39E-02	1.00E+00	MRPL40	2.35E-02	1.00E+00
LOC102398702	2.72E+00	9.01E-07	LOC102394061	6.36E-02	1.00E+00	PODXL2	2.95E-02	1.00E+00
LOC102411924	-1.25E+00	9.16E-07	HRCT1	-3.32E-02	1.00E+00	ANXA1	2.90E-02	1.00E+00
ADGRA1	-2.82E+00	9.20E-07	CDKN1B	-4.66E-02	1.00E+00	ADAT2	-1.96E-02	1.00E+00
ZBTB3	1.10E+00	9.29E-07	LOC102390612	4.23E-02	1.00E+00	SCAMP3	-1.47E-02	1.00E+00
LOC102398414	-1.12E+00	9.33E-07	PANX1	2.54E-02	1.00E+00	LOC102395607	-2.49E-02	1.00E+00
EBAG9	-6.88E-01	9.41E-07	LOC102404275	6.55E-02	1.00E+00	HSD17B10	2.30E-02	1.00E+00
FOLR2	-5.10E+00	9.51E-07	ARL1	2.66E-02	1.00E+00	ASB1	2.06E-02	1.00E+00
TRMU	-6.85E-01	9.59E-07	ST7L	3.08E-02	1.00E+00	ANKRD12	1.94E-02	1.00E+00
LOC102390908	-1.16E+00	9.60E-07	CTNNB1	2.42E-02	1.00E+00	GNB2	1.94E-02	1.00E+00
SLX1A	1.74E+00	9.78E-07	HTATIP2	-2.92E-02	1.00E+00	YIPF6	-1.73E-02	1.00E+00
LOC102402796	-9.98E-01	9.83E-07	STRA8	1.07E-01	1.00E+00	ORC5	-1.77E-02	1.00E+00
FLT4	2.24E+00	9.92E-07	RIC8A	5.10E-02	1.00E+00	MAP4K3	2.37E-02	1.00E+00
IFT27	-9.54E-01	9.94E-07	ARF3	1.93E-02	1.00E+00	NDUFB10	2.40E-02	1.00E+00

LOC102389249	5.18E-01	1.00E-06	LOC112583961	1.04E-01	1.00E+00	RPF1	1.69E-02	1.00E+00
ADAM2	3.44E+00	1.00E-06	NDFIP2	4.56E-02	1.00E+00	PRKAA1	1.96E-02	1.00E+00
CDH13	1.11E+00	1.02E-06	PARP11	3.79E-02	1.00E+00	ARCN1	-1.62E-02	1.00E+00
CD74	-2.24E+00	1.02E-06	ZNF772	2.21E-02	1.00E+00	LOC102416345	1.70E-02	1.00E+00
FLNB	-7.57E-01	1.03E-06	TEKT3	-7.21E-02	1.00E+00	C4H12orf10	1.94E-02	1.00E+00
LOC102393233	-3.56E+00	1.03E-06	LOC112579167	-4.51E-02	1.00E+00	ZBTB16	-3.04E-02	1.00E+00
SENP2	-5.42E-01	1.04E-06	SHLD2	2.82E-02	1.00E+00	NMRAL1	-2.38E-02	1.00E+00
LOC102401017	-7.42E-01	1.04E-06	PROM2	5.96E-02	1.00E+00	ZHX1	-2.57E-02	1.00E+00
NDST1	5.51E-01	1.05E-06	RP2	-4.90E-02	1.00E+00	TMEM60	1.99E-02	1.00E+00
CYTIP	-2.90E+00	1.05E-06	PTPN6	7.01E-02	1.00E+00	PIN1	2.08E-02	1.00E+00
SWI5	6.26E-01	1.05E-06	EML1	3.96E-02	1.00E+00	WASL	-1.47E-02	1.00E+00
SELPLG	-4.21E+00	1.06E-06	MFAP3L	4.03E-02	1.00E+00	KPNA1	-1.57E-02	1.00E+00
KPNA5	-1.53E+00	1.07E-06	TRMO	-3.00E-02	1.00E+00	RAB22A	-1.44E-02	1.00E+00
AP5M1	5.56E-01	1.07E-06	LOC102399010	-5.20E-02	1.00E+00	LOC102391562	4.54E-02	1.00E+00
GLI1	-3.00E+00	1.08E-06	HSPA12B	-4.92E-02	1.00E+00	ZFAT	2.00E-02	1.00E+00
HCK	-2.94E+00	1.08E-06	MKLN1	2.11E-02	1.00E+00	ZNF248	-2.31E-02	1.00E+00
CDKN2D	-1.13E+00	1.09E-06	LOC112581602	-7.98E-02	1.00E+00	KRI1	-1.64E-02	1.00E+00
MED25	-5.05E-01	1.09E-06	MZF1	-4.14E-02	1.00E+00	PCYOX1	-1.56E-02	1.00E+00
TBX2	-2.54E+00	1.10E-06	RBBP7	2.14E-02	1.00E+00	DPYSL4	3.84E-02	1.00E+00
UNC79	2.37E+00	1.10E-06	IBSP	3.96E-02	1.00E+00	BET1	-1.56E-02	1.00E+00
ZNF287	-1.26E+00	1.14E-06	GGCX	5.44E-02	1.00E+00	LOC102412511	-1.48E-02	1.00E+00
ACADM	-5.20E-01	1.15E-06	SMIM12	2.71E-02	1.00E+00	TXNDC12	1.52E-02	1.00E+00
DDX23	-4.89E-01	1.15E-06	LOC102402796	-5.19E-02	1.00E+00	ATP5MD	-1.89E-02	1.00E+00
RAB2A	4.18E-01	1.16E-06	BMP3	7.17E-02	1.00E+00	LOC112584702	-3.43E-02	1.00E+00
LOC112578849	1.36E+00	1.17E-06	TOGARAM1	-3.05E-02	1.00E+00	BBS12	1.92E-02	1.00E+00
TCEA1	5.20E-01	1.19E-06	NIPAL4	-1.08E-01	1.00E+00	EEPD1	2.28E-02	1.00E+00
CFAP43	1.28E+00	1.19E-06	LOC112583298	-8.01E-02	1.00E+00	NPM3	-2.84E-02	1.00E+00
THTPA	5.73E-01	1.20E-06	MFSD9	-2.05E-02	1.00E+00	ZNF428	-2.47E-02	1.00E+00
PPP2R5D	5.20E-01	1.21E-06	DIMT1	-2.01E-02	1.00E+00	HM13	-1.45E-02	1.00E+00
LOC112586829	7.16E-01	1.21E-06	DBNDD1	-4.36E-02	1.00E+00	LOC102395792	1.76E-02	1.00E+00
RAI2	1.15E+00	1.21E-06	PIK3CB	4.33E-02	1.00E+00	LRFN3	-1.95E-02	1.00E+00
DDX27	-5.51E-01	1.22E-06	ZNF304	1.77E-02	1.00E+00	USP15	-1.53E-02	1.00E+00
PWWP2B	-1.44E+00	1.22E-06	BRCA1	-2.24E-02	1.00E+00	LOC102397179	3.51E-02	1.00E+00

GPM6B	-7.08E-01	1.22E-06	NAP1L3	-9.29E-02	1.00E+00	EMD	-1.60E-02	1.00E+00
CCDC39	1.04E+00	1.23E-06	AK1	3.43E-02	1.00E+00	MED8	-1.77E-02	1.00E+00
RBPJ	5.20E-01	1.23E-06	BICC1	3.85E-02	1.00E+00	C6H1orf162	-5.77E-02	1.00E+00
CCDC96	1.45E+00	1.23E-06	C6H1orf189	-1.00E-01	1.00E+00	PSAP	-2.17E-02	1.00E+00
LOC112586855	2.74E+00	1.24E-06	PITHD1	1.96E-02	1.00E+00	CNOT7	1.45E-02	1.00E+00
RPS6KA1	9.32E-01	1.24E-06	SMG6	2.33E-02	1.00E+00	GOLM1	1.51E-02	1.00E+00
LOC112586859	-2.89E+00	1.24E-06	MAST3	4.20E-02	1.00E+00	SP140	-1.60E-02	1.00E+00
LOC112586540	1.43E+00	1.26E-06	SIL1	2.26E-02	1.00E+00	LOC102401468	-1.94E-02	1.00E+00
LOC112583711	9.21E-01	1.29E-06	GPATCH2L	3.11E-02	1.00E+00	TMEM87A	-1.77E-02	1.00E+00
TULP1	2.22E+00	1.29E-06	SEMA4B	-5.30E-02	1.00E+00	LOC102394124	1.61E-02	1.00E+00
RELL2	7.73E-01	1.31E-06	NEXN	2.71E-02	1.00E+00	AFG3L2	-1.37E-02	1.00E+00
EMC9	-1.19E+00	1.31E-06	ETV3	-3.63E-02	1.00E+00	THAP11	1.68E-02	1.00E+00
TTBK2	-7.85E-01	1.32E-06	TMEM135	4.23E-02	1.00E+00	ERGIC3	-1.72E-02	1.00E+00
STRA8	2.00E+00	1.32E-06	DTX3	3.04E-02	1.00E+00	LOC102396111	-3.04E-02	1.00E+00
LOC112584642	2.21E+00	1.34E-06	HOXB4	7.32E-02	1.00E+00	LOC102395814	-1.77E-02	1.00E+00
TXNDC11	4.62E-01	1.36E-06	APBB2	-2.24E-02	1.00E+00	DGKZ	1.48E-02	1.00E+00
ESF1	-5.68E-01	1.36E-06	CASK	2.58E-02	1.00E+00	TLN2	2.19E-02	1.00E+00
NCAPD3	5.87E-01	1.38E-06	LAMTOR4	-2.68E-02	1.00E+00	LOC102414731	3.07E-02	1.00E+00
IGHMBP2	4.91E-01	1.39E-06	LOC102402778	-2.53E-02	1.00E+00	TCP11L1	1.75E-02	1.00E+00
PREB	-5.35E-01	1.41E-06	FANCL	1.98E-02	1.00E+00	LOC102408997	-1.99E-02	1.00E+00
LOC112581279	2.68E+00	1.41E-06	TNFRSF6B	9.66E-02	1.00E+00	LAMP1	-1.76E-02	1.00E+00
LOC102408414	-2.12E+00	1.41E-06	RPS6KC1	-3.40E-02	1.00E+00	NEMF	-1.37E-02	1.00E+00
MARS	4.72E-01	1.42E-06	SEMA4G	5.30E-02	1.00E+00	LOC102393517	1.75E-02	1.00E+00
LOC102412813	1.53E+00	1.43E-06	MST1	-5.88E-02	1.00E+00	C9H19orf44	1.80E-02	1.00E+00
CTNNBL1	-6.34E-01	1.43E-06	RTCB	1.83E-02	1.00E+00	GPKOW	-1.61E-02	1.00E+00
SLC26A10	1.43E+00	1.43E-06	LOC112587753	1.86E-01	1.00E+00	MTHFD1	1.47E-02	1.00E+00
CCL25	-1.27E+00	1.44E-06	GPR173	4.50E-02	1.00E+00	DPH6	-1.76E-02	1.00E+00
SP4	-8.73E-01	1.46E-06	JCAD	4.49E-02	1.00E+00	CHD7	1.85E-02	1.00E+00
TSPAN5	5.87E-01	1.46E-06	FAM185A	5.10E-02	1.00E+00	PDCD2	1.47E-02	1.00E+00
TRIM65	-7.65E-01	1.48E-06	HSPA14	2.03E-02	1.00E+00	PEX10	-1.82E-02	1.00E+00
SPEF1	-1.17E+00	1.48E-06	PRLR	8.62E-02	1.00E+00	RPS7	-3.05E-02	1.00E+00
C2CD2L	-6.33E-01	1.50E-06	HEXA	-2.52E-02	1.00E+00	C6H1orf122	-2.10E-02	1.00E+00
SSB	4.37E-01	1.51E-06	UBL4A	-5.38E-02	1.00E+00	NBEAL2	1.81E-02	1.00E+00
RARG	-1.36E+00	1.51E-06	TARS	-2.15E-02	1.00E+00	PRKCH	-3.24E-02	1.00E+00

BOD1	5.50E-01	1.53E-06	FAM110A	-2.00E-02	1.00E+00	KCTD18	1.79E-02	1.00E+00
LOC112580614	1.85E+00	1.54E-06	CDC42SE1	1.99E-02	1.00E+00	PRR36	-2.68E-02	1.00E+00
PDLIM1	8.27E-01	1.54E-06	LOC112583701	-6.53E-02	1.00E+00	RASA4B	1.70E-02	1.00E+00
AGBL5	-5.89E-01	1.55E-06	TTF1	-2.59E-02	1.00E+00	PTER	2.02E-02	1.00E+00
DDX54	-5.06E-01	1.56E-06	P4HA3	-4.32E-02	1.00E+00	NUDT16	-2.10E-02	1.00E+00
VLDLR	5.66E-01	1.56E-06	DHX36	-1.98E-02	1.00E+00	SUMO1	-1.49E-02	1.00E+00
DAPK3	-6.60E-01	1.57E-06	CCNE2	-1.18E-01	1.00E+00	TMEM201	-1.49E-02	1.00E+00
CCDC124	-7.46E-01	1.57E-06	KNOP1	2.75E-02	1.00E+00	MBTPS2	1.73E-02	1.00E+00
LOC102399312	-1.55E+00	1.58E-06	PALM	2.35E-02	1.00E+00	SAMD5	3.19E-02	1.00E+00
CDC16	-5.69E-01	1.58E-06	GSC2	-6.62E-02	1.00E+00	LOC102401393	1.69E-02	1.00E+00
SKA3	6.19E-01	1.58E-06	TTLL7	-1.03E-01	1.00E+00	PSME2	-2.02E-02	1.00E+00
CENPK	7.30E-01	1.58E-06	NOL4L	3.08E-02	1.00E+00	LOC112581760	3.41E-02	1.00E+00
TP53INP2	-1.31E+00	1.60E-06	SNRPB	2.43E-02	1.00E+00	STARD7	1.37E-02	1.00E+00
DNPEP	-6.90E-01	1.62E-06	SLC26A8	3.08E-02	1.00E+00	OST4	1.98E-02	1.00E+00
DDR2	-8.37E-01	1.63E-06	PLPP5	-3.57E-02	1.00E+00	DENND5A	1.60E-02	1.00E+00
CUL4B	5.92E-01	1.63E-06	RFC5	-1.88E-02	1.00E+00	LOC102409358	1.57E-02	1.00E+00
LOC112586481	1.60E+00	1.63E-06	RBM17	-4.98E-02	1.00E+00	RGS2	2.51E-02	1.00E+00
ABCA4	7.42E-01	1.65E-06	PMPCB	-5.05E-02	1.00E+00	AGBL2	1.91E-02	1.00E+00
HECTD4	6.33E-01	1.66E-06	MACC1	6.41E-02	1.00E+00	C12H2orf40	-3.26E-02	1.00E+00
PRPF38A	4.16E-01	1.66E-06	EXOC8	-2.73E-02	1.00E+00	SGTA	-1.36E-02	1.00E+00
CD86	-3.15E+00	1.69E-06	DOP1B	2.42E-02	1.00E+00	COG7	-1.44E-02	1.00E+00
LOC102391973	9.70E-01	1.70E-06	PBX3	2.25E-02	1.00E+00	LOC102395556	3.06E-02	1.00E+00
DNAJC8	5.17E-01	1.71E-06	LOC102404409	9.87E-02	1.00E+00	TAMM41	-1.84E-02	1.00E+00
PTPN5	-1.11E+00	1.72E-06	PTPRJ	-5.18E-02	1.00E+00	RPL31	-2.91E-02	1.00E+00
RP2	-8.08E-01	1.73E-06	MKKS	-3.00E-02	1.00E+00	NOL3	-1.70E-02	1.00E+00
PLTP	-1.91E+00	1.73E-06	YIPF1	4.71E-02	1.00E+00	LOC102394594	3.00E-02	1.00E+00
UBE2S	-8.32E-01	1.74E-06	FEM1A	3.09E-02	1.00E+00	MFN2	-1.34E-02	1.00E+00
ADGRE1	-3.53E+00	1.74E-06	PSMC3IP	-2.08E-02	1.00E+00	MFAP1	1.32E-02	1.00E+00
DNAJA3	5.27E-01	1.76E-06	NDUFAF5	6.95E-02	1.00E+00	MRPL17	1.77E-02	1.00E+00
GNAI3	4.06E-01	1.76E-06	CDKL1	3.89E-02	1.00E+00	FAAP100	-1.76E-02	1.00E+00
SIRPB2	2.74E+00	1.76E-06	LOC102392697	-3.91E-02	1.00E+00	MIPEP	1.37E-02	1.00E+00
LOC102397700	6.80E-01	1.77E-06	CCDC43	2.93E-02	1.00E+00	HSD17B14	2.94E-02	1.00E+00
APCDD1L	2.24E+00	1.77E-06	LOC102401139	-2.86E-02	1.00E+00	LOC102408966	1.48E-02	1.00E+00
AGPAT4	-	1.78E-06	DCUN1D5	-2.41E-	1.00E+00	MANEA	-1.75E-	1.00E+00

	2.26E+00			02			02	
KLHL5	-6.60E-01	1.79E-06	GUCA2A	1.04E-01	1.00E+00	ATP1A1	-1.82E-02	1.00E+00
CLN8	-6.61E-01	1.82E-06	ZFAND3	1.65E-02	1.00E+00	ATP1B2	3.24E-02	1.00E+00
NUP160	5.56E-01	1.82E-06	ASZ1	2.97E-02	1.00E+00	AGL	-2.94E-02	1.00E+00
TOX4	4.75E-01	1.82E-06	LOC112577650	-2.97E-02	1.00E+00	DNASE2	-1.48E-02	1.00E+00
DCTN6	-4.93E-01	1.82E-06	LOC102390387	-1.39E-01	1.00E+00	SMG7	-1.42E-02	1.00E+00
LOC102413915	-6.16E-01	1.83E-06	PCM1	-3.34E-02	1.00E+00	DDOST	1.66E-02	1.00E+00
SDE2	5.57E-01	1.85E-06	LBR	-3.11E-02	1.00E+00	TTLL5	1.73E-02	1.00E+00
PSMG1	6.07E-01	1.85E-06	MALRD1	1.77E-02	1.00E+00	RPAP2	1.71E-02	1.00E+00
NDUFA8	6.60E-01	1.86E-06	ZNF740	5.59E-02	1.00E+00	VIM	2.76E-02	1.00E+00
PRPF8	-5.93E-01	1.86E-06	BAZ1A	-3.02E-02	1.00E+00	PIGU	-1.53E-02	1.00E+00
BRD3	-5.24E-01	1.87E-06	NCAPD2	-5.34E-02	1.00E+00	COL4A4	1.79E-02	1.00E+00
LOC112585715	2.96E+00	1.89E-06	ALG11	2.07E-02	1.00E+00	GBA2	1.31E-02	1.00E+00
TSEN34	-6.62E-01	1.89E-06	ZNF382	4.91E-02	1.00E+00	EDNRB	6.01E-02	1.00E+00
DXO	-7.54E-01	1.90E-06	BTD	5.16E-02	1.00E+00	ZNF800	-1.73E-02	1.00E+00
AHSA1	5.37E-01	1.90E-06	CCDC158	-7.95E-02	1.00E+00	PGBD2	-2.74E-02	1.00E+00
FBXO36	-8.89E-01	1.94E-06	PKMYT1	2.40E-02	1.00E+00	EZR	-1.67E-02	1.00E+00
ITGA2B	-1.26E+00	1.95E-06	FASTKD2	-1.83E-02	1.00E+00	S100B	-3.54E-02	1.00E+00
LOC112582960	-1.24E+00	1.96E-06	DMRT3	-3.98E-02	1.00E+00	SLC30A6	-1.55E-02	1.00E+00
DCDC2C	-2.05E+00	1.96E-06	POLH	5.56E-02	1.00E+00	LOC112580637	3.04E-02	1.00E+00
LOC112578394	-3.34E+00	1.98E-06	ATP12A	-6.99E-02	1.00E+00	SLC25A6	-2.54E-02	1.00E+00
MYO10	6.23E-01	1.98E-06	PNMA1	-3.65E-02	1.00E+00	SMIM15	-1.45E-02	1.00E+00
POLR2H	-7.23E-01	2.00E-06	LOC102398198	-5.20E-02	1.00E+00	ARIH2	-1.23E-02	1.00E+00
KMT2E	-5.91E-01	2.00E-06	LOC102402027	-1.32E-01	1.00E+00	SUSD4	2.50E-02	1.00E+00
ARHGEF40	-6.16E-01	2.00E-06	CLBA1	-6.87E-02	1.00E+00	PPEF1	-2.10E-02	1.00E+00
ADSL	-4.85E-01	2.00E-06	FAM205C	5.93E-02	1.00E+00	LOC102397739	-3.96E-02	1.00E+00
ARPC1B	8.74E-01	2.01E-06	ITGAV	-8.24E-02	1.00E+00	NIT1	-1.79E-02	1.00E+00
BAG3	-7.67E-01	2.02E-06	KANK3	5.92E-02	1.00E+00	EIF3B	1.42E-02	1.00E+00
LOC102390463	-1.22E+00	2.02E-06	VWA2	2.71E-02	1.00E+00	DR1	1.58E-02	1.00E+00
CCZ1	5.19E-01	2.02E-06	EIF2AK3	-5.59E-02	1.00E+00	HAGH	1.78E-02	1.00E+00
ZNF829	-7.40E-01	2.05E-06	KBTBD4	4.19E-02	1.00E+00	ERN1	-2.03E-02	1.00E+00
LOC102398747	-2.19E+00	2.07E-06	LOC102395220	-1.12E-01	1.00E+00	RPL14	2.84E-02	1.00E+00
PPP2R1B	4.80E-01	2.07E-06	GPRC5B	5.12E-02	1.00E+00	ARFGEF2	-1.93E-02	1.00E+00
ANKRD31	1.68E+00	2.09E-06	LOC102390532	5.08E-02	1.00E+00	ATP6V0C	-1.55E-	1.00E+00

							02	
SH3YL1	5.88E-01	2.10E-06	GRP	-8.53E-02	1.00E+00	C1QC	6.93E-02	1.00E+00
AP1G2	-5.59E-01	2.11E-06	TMEM69	2.40E-02	1.00E+00	MAP2K2	1.47E-02	1.00E+00
HTR7	1.84E+00	2.11E-06	TADA3	1.75E-02	1.00E+00	RDH14	1.59E-02	1.00E+00
BANK1	-2.48E+00	2.11E-06	ATP5PF	-3.31E-02	1.00E+00	TMEM167A	-1.33E-02	1.00E+00
LOC102399663	8.61E-01	2.14E-06	RILPL2	-4.48E-02	1.00E+00	INO80E	1.38E-02	1.00E+00
TMEM50B	-4.73E-01	2.17E-06	TANGO2	2.92E-02	1.00E+00	LAMC2	-2.45E-02	1.00E+00
ZBTB10	-6.14E-01	2.19E-06	FASTKD3	-3.40E-02	1.00E+00	LOC112583626	-2.44E-02	1.00E+00
BRAF	-4.97E-01	2.20E-06	ARHGAP20	-5.50E-02	1.00E+00	RPS14	-2.57E-02	1.00E+00
MINOS1	-8.46E-01	2.21E-06	LOC102412652	-1.00E-01	1.00E+00	SMG9	-1.47E-02	1.00E+00
EPPK1	-3.33E+00	2.21E-06	WARS2	3.45E-02	1.00E+00	LOC102397143	2.52E-02	1.00E+00
ATP6V1D	-4.98E-01	2.22E-06	PLXNA3	8.34E-02	1.00E+00	MFSD5	1.48E-02	1.00E+00
EXOC5	-6.97E-01	2.23E-06	ABCC8	1.38E-01	1.00E+00	ARF3	1.38E-02	1.00E+00
BOLA2B	-7.78E-01	2.25E-06	DRD2	-9.15E-02	1.00E+00	TRAM1	1.31E-02	1.00E+00
ZNF317	6.93E-01	2.27E-06	GAS7	2.84E-02	1.00E+00	GCFC2	-1.92E-02	1.00E+00
PDE10A	-2.39E+00	2.34E-06	RASA4B	3.14E-02	1.00E+00	NUTF2	1.67E-02	1.00E+00
QPCTL	-7.20E-01	2.34E-06	LOC112587873	-2.05E-01	1.00E+00	FAM219A	1.66E-02	1.00E+00
B3GLCT	-5.31E-01	2.36E-06	ZNF614	5.77E-02	1.00E+00	SPTSSA	-1.64E-02	1.00E+00
LOC112582115	6.04E-01	2.37E-06	SBDS	2.24E-02	1.00E+00	LOC102406337	-1.56E-02	1.00E+00
ADGRG3	2.59E+00	2.38E-06	CHRDL1	1.06E-01	1.00E+00	LOC112578149	4.97E-02	1.00E+00
RCE1	5.83E-01	2.40E-06	TBX6	4.29E-02	1.00E+00	CGGBP1	-1.31E-02	1.00E+00
CTDSP2	6.02E-01	2.40E-06	TBRG1	2.27E-02	1.00E+00	SGMS1	-1.64E-02	1.00E+00
MBLAC1	-1.03E+00	2.41E-06	MON2	2.33E-02	1.00E+00	PCDHB8	-3.22E-02	1.00E+00
CKAP2	-5.68E-01	2.42E-06	CDAN1	6.21E-02	1.00E+00	RANGAP1	1.62E-02	1.00E+00
LOC102395369	1.08E+00	2.42E-06	MCC	2.07E-02	1.00E+00	ZNF7	1.42E-02	1.00E+00
ADAMTS2	-1.24E+00	2.42E-06	RETREG2	-2.48E-02	1.00E+00	ZBTB40	-1.50E-02	1.00E+00
DAO	-2.09E+00	2.44E-06	RNF216	-2.00E-02	1.00E+00	GALNT1	1.23E-02	1.00E+00
DNAJC28	7.93E-01	2.46E-06	COL4A2	-9.42E-02	1.00E+00	PRKAR1A	1.36E-02	1.00E+00
COPB2	4.36E-01	2.47E-06	C7H4orf50	-7.15E-02	1.00E+00	RAB24	-1.62E-02	1.00E+00
PUDP	5.35E-01	2.47E-06	HDAC8	5.48E-02	1.00E+00	MAP4	1.62E-02	1.00E+00
NLGN3	-1.55E+00	2.48E-06	ZEB2	-6.93E-02	1.00E+00	ZBTB48	1.63E-02	1.00E+00
TRIOBP	4.90E-01	2.50E-06	PDE7A	3.50E-02	1.00E+00	DCUN1D1	-1.45E-02	1.00E+00
WWP1	4.09E-01	2.50E-06	ALKBH1	-3.75E-02	1.00E+00	SOX5	1.92E-02	1.00E+00
GMNN	5.61E-01	2.51E-06	LOC112579157	2.21E-02	1.00E+00	ANAPC11	2.02E-02	1.00E+00

LOC112581212	-1.41E+00	2.52E-06	ZNF148	-4.38E-02	1.00E+00	PRAG1	-5.62E-02	1.00E+00
SLX4	-5.23E-01	2.53E-06	NT5E	3.45E-02	1.00E+00	IGHMBP2	1.40E-02	1.00E+00
MAK	1.67E+00	2.53E-06	ZMYM1	-4.34E-02	1.00E+00	DOCK8	-1.93E-02	1.00E+00
TBC1D13	4.12E-01	2.53E-06	IRF1	-2.62E-02	1.00E+00	COL4A3BP	1.40E-02	1.00E+00
JTB	-6.48E-01	2.55E-06	ORC5	-2.26E-02	1.00E+00	PITPNM1	-1.87E-02	1.00E+00
RNF185	4.98E-01	2.55E-06	BHLHE41	-1.63E-01	1.00E+00	CEP131	-1.39E-02	1.00E+00
OXSR1	-4.39E-01	2.56E-06	TELO2	-5.29E-02	1.00E+00	CXCR4	3.59E-02	1.00E+00
LOC102407989	1.29E+00	2.56E-06	PITPNM3	8.92E-02	1.00E+00	UBL7	1.44E-02	1.00E+00
CCNT1	-6.34E-01	2.58E-06	NRBP1	-1.94E-02	1.00E+00	CBX7	-1.66E-02	1.00E+00
PTP4A1	-5.41E-01	2.59E-06	ADRA2A	6.17E-02	1.00E+00	LOC102398746	1.91E-02	1.00E+00
LOC102401813	-6.76E-01	2.66E-06	LOC102403295	7.43E-02	1.00E+00	LOC102398106	1.56E-02	1.00E+00
PLCB1	9.70E-01	2.66E-06	ABHD5	5.03E-02	1.00E+00	RPL30	2.47E-02	1.00E+00
GOT1	4.58E-01	2.68E-06	TANC1	-2.56E-02	1.00E+00	HCFC2	2.12E-02	1.00E+00
PTEN	-4.64E-01	2.70E-06	EFHD2	-2.50E-02	1.00E+00	LOC102403153	1.57E-02	1.00E+00
C3H9orf43	-1.28E+00	2.72E-06	CSPP1	1.81E-02	1.00E+00	TECPR1	1.52E-02	1.00E+00
DENND4C	-7.31E-01	2.73E-06	ARHGEF11	-5.73E-02	1.00E+00	TRMT2B	-2.90E-02	1.00E+00
YWHAH	-5.34E-01	2.73E-06	DEPDC1B	-2.27E-02	1.00E+00	EXOSC10	-1.51E-02	1.00E+00
SDHD	-4.58E-01	2.73E-06	MOSPD3	3.43E-02	1.00E+00	PPP6R3	1.34E-02	1.00E+00
CCDC166	1.86E+00	2.75E-06	GJC1	2.51E-02	1.00E+00	LOC102400025	-1.19E-02	1.00E+00
VWDE	2.33E+00	2.75E-06	LOC112581581	-5.07E-02	1.00E+00	ZNF143	1.66E-02	1.00E+00
PRICKLE2	6.55E-01	2.77E-06	LOC102401361	3.24E-02	1.00E+00	LOC112586992	-3.06E-02	1.00E+00
SNTG1	-2.96E+00	2.79E-06	TMEM120B	1.66E-02	1.00E+00	LOC112583695	3.21E-02	1.00E+00
NOL4L	-5.45E-01	2.80E-06	TAL1	5.33E-02	1.00E+00	CDV3	1.23E-02	1.00E+00
LOC102396606	-2.68E+00	2.80E-06	DDX23	1.98E-02	1.00E+00	ZNF454	1.32E-02	1.00E+00
CTDP1	-5.50E-01	2.81E-06	FYN	2.11E-02	1.00E+00	UBE3D	1.61E-02	1.00E+00
RHOC	-6.13E-01	2.83E-06	TEFM	2.91E-02	1.00E+00	LOC102393569	3.12E-02	1.00E+00
CRYZL1	-6.35E-01	2.84E-06	ABLIM3	2.58E-02	1.00E+00	GEMIN5	1.37E-02	1.00E+00
SRPRB	-5.15E-01	2.88E-06	LMO1	-2.58E-02	1.00E+00	ZFP62	-1.90E-02	1.00E+00
UBE2L6	8.61E-01	2.88E-06	F2RL1	4.25E-02	1.00E+00	GPD1	1.72E-02	1.00E+00
ITGA1	7.40E-01	2.88E-06	INSIG2	2.59E-02	1.00E+00	UFL1	-1.63E-02	1.00E+00
SUSD1	-2.93E+00	2.90E-06	PCK2	9.41E-02	1.00E+00	COG3	-1.69E-02	1.00E+00
STK36	-4.67E-01	2.91E-06	IFI30	-7.54E-02	1.00E+00	MPP7	2.09E-02	1.00E+00
LOC102414221	1.43E+00	2.92E-06	GGA3	2.89E-02	1.00E+00	MIOS	-1.37E-02	1.00E+00

LOC102401307	-4.09E+00	2.93E-06	PLA2G12A	-2.23E-02	1.00E+00	LRRC75A	-1.38E-02	1.00E+00
ADCK2	-6.29E-01	2.95E-06	CEP164	2.50E-02	1.00E+00	LOC112580793	-1.29E-02	1.00E+00
FICD	8.40E-01	2.97E-06	STIP1	1.69E-02	1.00E+00	PEX19	1.30E-02	1.00E+00
ATRIP	-6.04E-01	2.97E-06	VWF	5.72E-02	1.00E+00	LOC102394106	2.57E-02	1.00E+00
LOC102391948	-1.83E+00	2.97E-06	SP3	5.20E-02	1.00E+00	CSNK1G3	-1.49E-02	1.00E+00
LOC102408708	1.90E+00	3.00E-06	MSH6	2.47E-02	1.00E+00	LOC102389123	-3.39E-02	1.00E+00
CASP2	4.73E-01	3.01E-06	GNB2	-2.04E-02	1.00E+00	GSR	1.44E-02	1.00E+00
LGMN	1.11E+00	3.02E-06	PRKCQ	-2.06E-02	1.00E+00	LOC102392425	-2.13E-02	1.00E+00
EREG	-3.94E+00	3.02E-06	RPL28	-8.15E-02	1.00E+00	LOC112587015	-3.17E-02	1.00E+00
LOC112577891	2.23E+00	3.03E-06	RASGRP3	-5.03E-02	1.00E+00	DDX25	-2.80E-02	1.00E+00
SKAP1	-8.90E-01	3.04E-06	LOC102396108	-7.53E-02	1.00E+00	TCOF1	1.27E-02	1.00E+00
HK1	-5.22E-01	3.10E-06	IQCG	-9.84E-02	1.00E+00	LOC102416288	2.06E-02	1.00E+00
EDNRA	-9.66E-01	3.10E-06	ZNF483	5.21E-02	1.00E+00	TALDO1	-1.89E-02	1.00E+00
CEBPZ	4.02E-01	3.12E-06	EIF4B	2.08E-02	1.00E+00	GATC	-1.63E-02	1.00E+00
PTK2B	-3.51E+00	3.19E-06	NAT9	-6.68E-02	1.00E+00	ARHGDIB	4.89E-02	1.00E+00
FOXP3	1.38E+00	3.19E-06	PTK6	1.15E-01	1.00E+00	LOC102411593	-1.56E-02	1.00E+00
POLR3K	5.00E-01	3.21E-06	ZNF177	5.14E-02	1.00E+00	SNX7	-1.33E-02	1.00E+00
LOC112584176	-3.20E+00	3.22E-06	PABPN1	-2.09E-02	1.00E+00	EFHC2	2.03E-02	1.00E+00
LOC112581854	-1.52E+00	3.22E-06	REX1BD	-3.64E-02	1.00E+00	FAM8A1	1.35E-02	1.00E+00
LOC102410254	-1.19E+00	3.22E-06	LOC102415842	2.61E-02	1.00E+00	CCSAP	1.59E-02	1.00E+00
LOC102411696	-2.82E+00	3.25E-06	ARMC12	-6.41E-02	1.00E+00	RILP	-2.68E-02	1.00E+00
ATP6V1C2	-1.68E+00	3.28E-06	RFX5	-1.86E-02	1.00E+00	GDPD5	3.12E-02	1.00E+00
INO80C	-6.66E-01	3.30E-06	CPPED1	-3.47E-02	1.00E+00	SRR	1.40E-02	1.00E+00
PCDH17	-1.80E+00	3.32E-06	MCM5	-1.66E-02	1.00E+00	C19H5orf49	-2.21E-02	1.00E+00
TCOF1	-5.84E-01	3.42E-06	OSTF1	-4.98E-02	1.00E+00	TMEM185B	1.30E-02	1.00E+00
STX7	5.35E-01	3.44E-06	AFG3L2	1.91E-02	1.00E+00	BRAT1	-1.49E-02	1.00E+00
DVL3	4.69E-01	3.45E-06	ERG	1.13E-01	1.00E+00	BAP1	1.20E-02	1.00E+00
GABRB2	2.38E+00	3.52E-06	ARRDC4	4.67E-02	1.00E+00	LOC112579694	-3.02E-02	1.00E+00
PDK1	-6.67E-01	3.53E-06	PITPNM2	3.32E-02	1.00E+00	COMMD4	1.87E-02	1.00E+00
TTLL9	-1.72E+00	3.54E-06	SLC1A6	-1.19E-01	1.00E+00	APP	1.75E-02	1.00E+00
LOC112584700	1.11E+00	3.57E-06	VEGFB	4.86E-02	1.00E+00	TRO	2.11E-02	1.00E+00
FZD10	2.03E+00	3.61E-06	PTPRS	2.81E-02	1.00E+00	NSMCE1	1.58E-02	1.00E+00
DHDH	-1.19E+00	3.64E-06	REM2	-3.60E-02	1.00E+00	LOC112586543	2.29E-02	1.00E+00
DNAJC17	-6.89E-	3.71E-06	IMPAD1	-3.46E-	1.00E+00	ZNF835	-1.77E-	1.00E+00

	01			02			02	
RIPK3	-2.90E+00	3.72E-06	MMP16	1.28E-01	1.00E+00	ANKRD10	1.41E-02	1.00E+00
HELB	7.51E-01	3.72E-06	CNOT2	2.72E-02	1.00E+00	ZNF22	-1.41E-02	1.00E+00
B3GALT6	5.64E-01	3.78E-06	GPR83	1.42E-01	1.00E+00	MKLN1	-1.20E-02	1.00E+00
ARRDC5	2.71E+00	3.78E-06	PDZD4	6.49E-02	1.00E+00	SRSF9	-1.33E-02	1.00E+00
OLFML2A	9.92E-01	3.81E-06	LOC102395589	4.93E-02	1.00E+00	TSTD3	-2.42E-02	1.00E+00
CCNB1IP1	7.45E-01	3.81E-06	PDE4D	-1.94E-02	1.00E+00	TMEM178B	1.58E-02	1.00E+00
ADAMTSL2	-1.57E+00	3.83E-06	VPS54	3.77E-02	1.00E+00	LOC112585756	-2.76E-02	1.00E+00
TMEM185B	4.49E-01	3.85E-06	CCT6B	6.88E-02	1.00E+00	SUCLA2	1.31E-02	1.00E+00
TFPI2	-3.53E+00	3.86E-06	CD274	-8.47E-02	1.00E+00	PKMYT1	-1.83E-02	1.00E+00
SLC9A7	-1.38E+00	3.86E-06	CLTC	2.39E-02	1.00E+00	POLA2	-1.78E-02	1.00E+00
PSTPIP2	-1.25E+00	3.87E-06	TMEM222	-3.23E-02	1.00E+00	DIP2B	-1.67E-02	1.00E+00
LOC102409318	-1.53E+00	3.88E-06	CDKN2AIP	-2.52E-02	1.00E+00	FAM83A	-2.34E-02	1.00E+00
MND1	7.44E-01	3.90E-06	SLC45A4	-1.77E-02	1.00E+00	SCN9A	-2.17E-02	1.00E+00
PID1	-7.51E-01	3.92E-06	TRAF3IP3	-4.27E-02	1.00E+00	MPI	-1.67E-02	1.00E+00
FBN3	2.39E+00	3.92E-06	ZNF593	-3.72E-02	1.00E+00	C20H15orf40	-1.38E-02	1.00E+00
CTBS	2.68E+00	3.97E-06	LMBRD1	-1.72E-02	1.00E+00	OXT	-2.77E-02	1.00E+00
PLLP	2.47E+00	3.98E-06	LOC102394590	-4.34E-02	1.00E+00	UBAC2	-1.13E-02	1.00E+00
ARMC4	-9.31E-01	3.99E-06	MEGF8	5.75E-02	1.00E+00	ZNF527	1.52E-02	1.00E+00
B4GALT6	-7.54E-01	4.01E-06	ADCY10	-7.94E-02	1.00E+00	LOC112583963	2.86E-02	1.00E+00
LOC112583584	-2.90E+00	4.03E-06	TTC5	-2.68E-02	1.00E+00	TLR7	4.74E-02	1.00E+00
SMIM13	-9.94E-01	4.03E-06	LOC102411942	-7.46E-02	1.00E+00	DAP3	1.28E-02	1.00E+00
CTDNEP1	-5.26E-01	4.05E-06	LOC112580384	-5.64E-02	1.00E+00	CITED2	1.38E-02	1.00E+00
LOC102408803	-1.66E+00	4.07E-06	ARSG	1.03E-01	1.00E+00	PIM3	1.61E-02	1.00E+00
URB2	7.28E-01	4.09E-06	ZNF618	-1.98E-02	1.00E+00	DCTN1	1.25E-02	1.00E+00
HSPA12B	8.95E-01	4.12E-06	GPX7	9.69E-02	1.00E+00	RTCA	1.16E-02	1.00E+00
C1QL3	1.74E+00	4.14E-06	MGST3	3.13E-02	1.00E+00	PPP4R3A	-1.15E-02	1.00E+00
LOC112581361	-2.13E+00	4.16E-06	ADAMTS7	3.52E-02	1.00E+00	BMT2	1.53E-02	1.00E+00
ARMC8	-4.55E-01	4.18E-06	TRIM46	9.48E-02	1.00E+00	IMP4	1.52E-02	1.00E+00
FAM160B1	8.51E-01	4.26E-06	C9H5orf46	8.14E-02	1.00E+00	STIM2	1.44E-02	1.00E+00
LRRIQ3	7.97E-01	4.27E-06	ITGB3BP	-9.16E-02	1.00E+00	SUPV3L1	1.41E-02	1.00E+00
SP1	4.68E-01	4.29E-06	ARPC2	1.65E-02	1.00E+00	GSS	1.61E-02	1.00E+00
LOXL1	1.49E+00	4.33E-06	GTSE1	2.23E-02	1.00E+00	SERTAD1	-2.42E-02	1.00E+00
FZR1	5.04E-01	4.39E-06	THSD7A	4.88E-02	1.00E+00	MRPL45	1.33E-02	1.00E+00

HOXA5	2.35E+00	4.41E-06	CCDC81	7.80E-02	1.00E+00	FANCB	-2.51E-02	1.00E+00
LOC102406426	-2.27E+00	4.43E-06	TXNL4A	-4.09E-02	1.00E+00	BSDC1	1.26E-02	1.00E+00
PAN3	-6.27E-01	4.44E-06	RANGAP1	2.45E-02	1.00E+00	UNC13D	-2.19E-02	1.00E+00
CNIH2	-1.33E+00	4.45E-06	DLG2	1.83E-02	1.00E+00	RNF215	1.36E-02	1.00E+00
ELP3	-5.05E-01	4.46E-06	EPB41L4B	-1.84E-02	1.00E+00	GADL1	-1.76E-02	1.00E+00
RELCH	-4.79E-01	4.47E-06	WBP11	-1.63E-02	1.00E+00	SPRYD4	-1.74E-02	1.00E+00
SLCO2A1	1.63E+00	4.49E-06	INKA1	-1.07E-01	1.00E+00	UBE2F	-1.20E-02	1.00E+00
IMPA2	9.16E-01	4.51E-06	CDK2	-2.51E-02	1.00E+00	HHLA2	1.88E-02	1.00E+00
LOC102390765	1.06E+00	4.52E-06	LOXL4	4.44E-02	1.00E+00	RPS17	-2.72E-02	1.00E+00
TMEM141	-1.00E+00	4.53E-06	DCX	-8.55E-02	1.00E+00	ELK1	-1.34E-02	1.00E+00
LOC112584709	1.81E+00	4.53E-06	LOC102398945	7.87E-02	1.00E+00	RANBP1	-2.03E-02	1.00E+00
HIVEP3	1.15E+00	4.58E-06	TMEM251	3.19E-02	1.00E+00	TLCD1	2.72E-02	1.00E+00
COMMD2	5.77E-01	4.59E-06	ZMAT1	9.41E-02	1.00E+00	POLR2D	1.38E-02	1.00E+00
EDNRB	-3.23E+00	4.62E-06	LOC102409495	3.52E-02	1.00E+00	LOC112583129	2.83E-02	1.00E+00
PSMA4	-6.25E-01	4.65E-06	RNF6	2.70E-02	1.00E+00	BAG6	-1.27E-02	1.00E+00
POLR2D	-5.80E-01	4.66E-06	NAAA	-4.21E-02	1.00E+00	POP4	-1.16E-02	1.00E+00
LOC112580467	1.21E+00	4.69E-06	ITGA2	-3.74E-02	1.00E+00	SH3D21	2.63E-02	1.00E+00
GALNT3	-1.23E+00	4.73E-06	DYM	1.69E-02	1.00E+00	MTERF1	1.88E-02	1.00E+00
FAAP24	7.69E-01	4.73E-06	MFSD3	-4.86E-02	1.00E+00	FAHD1	1.58E-02	1.00E+00
BORCS8	-9.58E-01	4.75E-06	C16H11orf52	-4.98E-02	1.00E+00	LOC102402161	2.36E-02	1.00E+00
RNGTT	4.23E-01	4.75E-06	LOC102404595	-5.08E-02	1.00E+00	MIPOL1	-1.96E-02	1.00E+00
CMKLR1	-3.18E+00	4.75E-06	SPHK2	-2.99E-02	1.00E+00	BMS1	1.11E-02	1.00E+00
PRRX2	-2.51E+00	4.79E-06	CACNB1	7.92E-02	1.00E+00	PIP5K1B	2.93E-02	1.00E+00
PRR12	-5.11E-01	4.79E-06	SORBS1	-1.69E-02	1.00E+00	UNC119B	1.26E-02	1.00E+00
LBR	9.06E-01	4.81E-06	NAT16	8.79E-02	1.00E+00	FARS2	-1.30E-02	1.00E+00
LOC112587817	-3.25E+00	4.85E-06	ZNF865	3.06E-02	1.00E+00	MRPL19	1.49E-02	1.00E+00
PGM2L1	-5.73E-01	4.87E-06	ZNF165	-4.14E-02	1.00E+00	BNIP3L	1.29E-02	1.00E+00
WDR33	-4.01E-01	4.88E-06	ASPH	-5.22E-02	1.00E+00	LRRC40	-1.90E-02	1.00E+00
TCTA	-6.90E-01	4.89E-06	LOC112581182	8.21E-02	1.00E+00	YIF1B	-1.61E-02	1.00E+00
CENPX	-9.13E-01	4.92E-06	PLIN2	-1.94E-02	1.00E+00	ATP5F1A	1.81E-02	1.00E+00
FGD3	-3.83E+00	4.93E-06	CLIP4	-3.17E-02	1.00E+00	TMEM214	1.28E-02	1.00E+00
OIP5	1.11E+00	4.94E-06	KCNC1	7.99E-02	1.00E+00	FNIP2	2.16E-02	1.00E+00
ENTPD6	-6.40E-01	4.98E-06	EPB41L5	1.83E-02	1.00E+00	DCTN2	-1.27E-02	1.00E+00

MBOAT2	5.66E-01	5.06E-06	ZC3H14	-1.85E-02	1.00E+00	CPNE4	2.12E-02	1.00E+00
LOC102415115	1.51E+00	5.10E-06	PITRM1	2.09E-02	1.00E+00	ZDHHC16	-1.36E-02	1.00E+00
SPATA16	2.14E+00	5.10E-06	LOC102411666	6.07E-02	1.00E+00	RCBTB2	1.16E-02	1.00E+00
SFXN2	-7.63E-01	5.10E-06	VASP	-2.54E-02	1.00E+00	RGS10	3.05E-02	1.00E+00
LOC102405522	2.37E+00	5.13E-06	EPHB3	-1.01E-01	1.00E+00	PRR14L	1.33E-02	1.00E+00
ZC3H15	4.04E-01	5.15E-06	KHDC3L	2.30E-02	1.00E+00	FRMPD1	-2.23E-02	1.00E+00
ADRB3	-2.74E+00	5.18E-06	LOC102412116	-1.06E-01	1.00E+00	RNF144A	-1.09E-02	1.00E+00
GTPBP10	4.97E-01	5.22E-06	LOC112580852	-6.72E-02	1.00E+00	VPS35	-1.26E-02	1.00E+00
PTK6	-1.46E+00	5.24E-06	LOC112582363	4.15E-02	1.00E+00	LOC112585542	2.72E-02	1.00E+00
NBAS	4.39E-01	5.27E-06	ATP6AP2	2.27E-02	1.00E+00	LOC102408429	2.52E-02	1.00E+00
GALC	1.00E+00	5.29E-06	SKA2	-4.02E-02	1.00E+00	C22H18orf21	1.42E-02	1.00E+00
SKP2	-9.45E-01	5.29E-06	LARS	-2.09E-02	1.00E+00	CDCA4	-1.51E-02	1.00E+00
SCAMP2	3.83E-01	5.32E-06	MTX2	4.97E-02	1.00E+00	RPS3A	-2.52E-02	1.00E+00
MED1	6.32E-01	5.32E-06	NUDCD3	-2.66E-02	1.00E+00	LIX1L	-1.42E-02	1.00E+00
LOC112577606	-7.53E-01	5.34E-06	WFDC3	-4.92E-02	1.00E+00	LOC112577635	-2.48E-02	1.00E+00
ZNF3	-6.17E-01	5.37E-06	RNH1	-2.83E-02	1.00E+00	ZCWPW1	-1.77E-02	1.00E+00
CAGE1	-9.24E-01	5.43E-06	HSD17B1	8.32E-02	1.00E+00	LCN6	2.68E-02	1.00E+00
C22H18orf21	-5.90E-01	5.49E-06	SQLE	-1.79E-02	1.00E+00	LIMS1	1.35E-02	1.00E+00
NLN	-5.63E-01	5.53E-06	PTPRE	1.21E-01	1.00E+00	SIGIRR	-2.92E-02	1.00E+00
CCDC78	-1.01E+00	5.57E-06	LOC102414371	-2.21E-02	1.00E+00	LOC102392520	-1.74E-02	1.00E+00
PTGER4	-2.67E+00	5.59E-06	EIF2B4	2.31E-02	1.00E+00	LOC102406330	3.00E-02	1.00E+00
LOC102394582	-3.95E-01	5.64E-06	ASCC2	2.36E-02	1.00E+00	SIGMAR1	-1.87E-02	1.00E+00
KIAA1024L	-1.02E+00	5.66E-06	FBXO16	-8.30E-02	1.00E+00	RAB3GAP2	1.38E-02	1.00E+00
PPP2R3C	4.30E-01	5.74E-06	DKK2	1.04E-01	1.00E+00	GSG1L	1.67E-02	1.00E+00
TSPAN12	1.75E+00	5.77E-06	MMP23B	-9.91E-02	1.00E+00	NSUN4	1.26E-02	1.00E+00
TNPO1	-5.05E-01	5.81E-06	RBX1	2.32E-02	1.00E+00	TDRD6	-2.33E-02	1.00E+00
MAP2K7	4.24E-01	5.82E-06	CGGBP1	-2.56E-02	1.00E+00	CEP120	-1.35E-02	1.00E+00
MYO9B	-5.40E-01	5.84E-06	SLC35B3	-3.39E-02	1.00E+00	ZC3H12D	2.64E-02	1.00E+00
SNRPD1	-7.44E-01	5.85E-06	ARRDC1	-1.77E-02	1.00E+00	MDGA1	-1.58E-02	1.00E+00
CDC5L	4.30E-01	5.87E-06	RRNAD1	7.92E-02	1.00E+00	SSR2	-1.57E-02	1.00E+00
NR1H2	5.10E-01	5.88E-06	MSH4	6.26E-02	1.00E+00	NDUFB2	-1.21E-02	1.00E+00
CNTFR	-1.24E+00	6.00E-06	UBXN8	-3.03E-02	1.00E+00	AIP	1.41E-02	1.00E+00
ZNF32	-7.00E-01	6.05E-06	SMAD6	3.15E-02	1.00E+00	KIF1C	1.11E-02	1.00E+00

TAF3	5.49E-01	6.06E-06	CCZ1	-2.57E-02	1.00E+00	FAM131C	2.55E-02	1.00E+00
PFDN6	-7.52E-01	6.07E-06	SMG9	2.26E-02	1.00E+00	IGFLR1	-1.66E-02	1.00E+00
RPP38	-7.72E-01	6.10E-06	RPN1	1.63E-02	1.00E+00	CSE1L	-1.08E-02	1.00E+00
UBE2B	-4.65E-01	6.15E-06	FAM49B	-2.02E-02	1.00E+00	LOC102404482	-5.66E-02	1.00E+00
RIMS1	1.99E+00	6.17E-06	PIKFYVE	3.77E-02	1.00E+00	TM4SF20	1.66E-02	1.00E+00
MORC2	4.42E-01	6.19E-06	LOC112579706	7.27E-02	1.00E+00	KIN	1.38E-02	1.00E+00
NR2C2AP	7.88E-01	6.27E-06	SMYD5	2.22E-02	1.00E+00	CCDC73	-1.56E-02	1.00E+00
C14H20orf27	5.41E-01	6.29E-06	PEMT	-6.11E-02	1.00E+00	C11H15orf61	1.88E-02	1.00E+00
TRAPPC13	-4.28E-01	6.34E-06	CWC22	-2.61E-02	1.00E+00	JMJD1C	1.84E-02	1.00E+00
IPO13	-4.49E-01	6.34E-06	FDXR	7.21E-02	1.00E+00	LTO1	1.26E-02	1.00E+00
LRFN2	2.37E+00	6.38E-06	MRPL16	2.36E-02	1.00E+00	LYSMD3	1.45E-02	1.00E+00
LCOR	-5.69E-01	6.39E-06	CASR	-2.08E-02	1.00E+00	FAM13A	1.16E-02	1.00E+00
NFKBIA	-7.43E-01	6.40E-06	ND4L	-4.59E-02	1.00E+00	IRAK1BP1	-2.00E-02	1.00E+00
SIGIRR	1.22E+00	6.43E-06	NUPL2	-3.13E-02	1.00E+00	CLEC3B	2.46E-02	1.00E+00
NAP1L3	-1.10E+00	6.43E-06	GPALPP1	-3.71E-02	1.00E+00	SMARCA5	-1.33E-02	1.00E+00
PLAGL2	6.69E-01	6.44E-06	BID	-3.93E-02	1.00E+00	GALK1	-1.75E-02	1.00E+00
TMEM87B	-6.11E-01	6.44E-06	PRKCB	-1.09E-01	1.00E+00	GPAA1	1.24E-02	1.00E+00
TXNDC9	4.75E-01	6.45E-06	ABCB4	8.07E-02	1.00E+00	EHHADH	-2.82E-02	1.00E+00
PIK3C3	-4.21E-01	6.46E-06	BMP6	2.09E-02	1.00E+00	RPS19	2.25E-02	1.00E+00
TBRG4	-5.28E-01	6.50E-06	CDC42EP4	2.78E-02	1.00E+00	FIP1L1	1.03E-02	1.00E+00
CTSS	-4.22E+00	6.51E-06	SPG11	-2.96E-02	1.00E+00	ATOX1	1.95E-02	1.00E+00
METTL24	-9.80E-01	6.54E-06	SUZ12	4.42E-02	1.00E+00	TBC1D2	1.64E-02	1.00E+00
HMGCS2	-2.46E+00	6.55E-06	SLX1A	-1.01E-01	1.00E+00	CAPNS1	1.55E-02	1.00E+00
SBK1	7.73E-01	6.58E-06	GNAI3	1.67E-02	1.00E+00	FAM91A1	-1.72E-02	1.00E+00
PSMD4	-6.61E-01	6.67E-06	POU5F1	1.88E-02	1.00E+00	ACY1	-1.61E-02	1.00E+00
TRIM55	2.30E+00	6.67E-06	LOC112586436	-5.90E-02	1.00E+00	FNBP1L	1.35E-02	1.00E+00
C6H1orf54	-1.59E+00	6.73E-06	DOCK2	-2.12E-02	1.00E+00	MRPL1	1.27E-02	1.00E+00
GART	-4.62E-01	6.74E-06	TM7SF2	3.79E-02	1.00E+00	ADA	-3.15E-02	1.00E+00
TOGARAM1	5.63E-01	6.80E-06	HOMER3	2.31E-02	1.00E+00	DUSP12	-1.26E-02	1.00E+00
LOC112586917	1.21E+00	6.83E-06	UBE2S	-2.04E-02	1.00E+00	SERPINI1	-2.68E-02	1.00E+00
ASS1	8.88E-01	6.85E-06	ASS1	2.56E-02	1.00E+00	LOC112584619	1.93E-02	1.00E+00
CKMT2	-1.69E+00	6.85E-06	SEMA7A	2.15E-02	1.00E+00	TGFBR3L	-1.96E-02	1.00E+00
MRPS15	-7.29E-01	6.86E-06	SDHAF4	-3.08E-02	1.00E+00	STOML2	-1.46E-02	1.00E+00
LOC102394446	-6.73E-	6.87E-06	PDIK1L	-7.81E-	1.00E+00	RPL29	2.33E-02	1.00E+00

	01			02				
PRCC	-4.32E-01	6.90E-06	CARD19	-2.71E-02	1.00E+00	PEX7	1.41E-02	1.00E+00
CNTRL	-6.42E-01	6.91E-06	LOC102402523	7.48E-02	1.00E+00	ZZEF1	-1.34E-02	1.00E+00
LOC102412047	9.89E-01	6.96E-06	KCND2	5.89E-02	1.00E+00	RPL13A	-1.88E-02	1.00E+00
ANKS1A	-4.53E-01	7.01E-06	HECW2	7.04E-02	1.00E+00	CRTC3	1.21E-02	1.00E+00
AGGF1	4.02E-01	7.04E-06	LRP10	-3.65E-02	1.00E+00	DNAL1	2.74E-02	1.00E+00
SURF4	4.49E-01	7.07E-06	CDK5	-2.38E-02	1.00E+00	ATP6V1D	-1.06E-02	1.00E+00
LOC112585344	-2.58E+00	7.12E-06	SMAD1	-2.53E-02	1.00E+00	LRRC28	-1.11E-02	1.00E+00
NDUFA11	-6.22E-01	7.13E-06	USP9X	2.35E-02	1.00E+00	FBXO42	1.27E-02	1.00E+00
GPATCH8	6.01E-01	7.16E-06	LACTB	2.85E-02	1.00E+00	PRRT4	-2.59E-02	1.00E+00
LOC102406736	-1.29E+00	7.24E-06	NAA40	2.76E-02	1.00E+00	KBTBD2	1.91E-02	1.00E+00
NCDN	-4.69E-01	7.29E-06	LOC102403205	-2.62E-02	1.00E+00	MED17	-1.13E-02	1.00E+00
SLC25A1	-7.12E-01	7.31E-06	RCC1L	1.99E-02	1.00E+00	CTSH	-3.62E-02	1.00E+00
LOC102389800	-8.54E-01	7.32E-06	LOC102401654	-3.14E-02	1.00E+00	LOC102400341	-2.27E-02	1.00E+00
INSYN1	-1.06E+00	7.33E-06	RNF185	2.53E-02	1.00E+00	ARFIP1	-1.31E-02	1.00E+00
SAG	-1.16E+00	7.33E-06	TRIP13	2.07E-02	1.00E+00	FGD2	4.76E-02	1.00E+00
UBE2A	-5.04E-01	7.37E-06	CENPO	1.61E-02	1.00E+00	LMBRD1	-1.09E-02	1.00E+00
LOC112582131	4.59E+00	7.43E-06	LOC102395796	-4.10E-02	1.00E+00	THAP6	-2.10E-02	1.00E+00
F13A1	-3.18E+00	7.46E-06	CTBP1	2.56E-02	1.00E+00	B3GLCT	1.20E-02	1.00E+00
RNF41	-8.48E-01	7.46E-06	LOC102395480	1.08E-01	1.00E+00	RPL26	1.48E-02	1.00E+00
LAPTM5	-4.25E+00	7.46E-06	NPEPL1	-1.92E-02	1.00E+00	APBB1	-1.09E-02	1.00E+00
LOC112587898	1.82E+00	7.51E-06	RIOK3	-2.28E-02	1.00E+00	KIAA1841	1.50E-02	1.00E+00
RAB3D	4.59E-01	7.53E-06	PRPF4	-1.62E-02	1.00E+00	HIF1AN	-1.04E-02	1.00E+00
CEP85L	-8.36E-01	7.54E-06	SLMAP	2.32E-02	1.00E+00	HSPA9	-1.23E-02	1.00E+00
LOC102415336	1.95E+00	7.55E-06	WDR89	-2.76E-02	1.00E+00	WDR38	-2.21E-02	1.00E+00
NOVA2	1.52E+00	7.55E-06	TECPR2	2.65E-02	1.00E+00	ANAPC2	1.26E-02	1.00E+00
LOC112585078	8.34E-01	7.56E-06	HOXA7	-3.09E-02	1.00E+00	TADA2B	1.55E-02	1.00E+00
PAQR8	-1.00E+00	7.57E-06	AACS	2.17E-02	1.00E+00	CDIP1	-1.05E-02	1.00E+00
POLD1	-5.69E-01	7.66E-06	WIPI1	1.20E-01	1.00E+00	CCDC9B	2.73E-02	1.00E+00
TROVE2	-7.66E-01	7.73E-06	IMPDH1	3.30E-02	1.00E+00	CTSS	-4.72E-02	1.00E+00
WDTC1	5.69E-01	7.75E-06	DNLZ	3.44E-02	1.00E+00	CACNB2	-1.87E-02	1.00E+00
LOC102407427	-2.00E+00	7.81E-06	LOC102399825	4.87E-02	1.00E+00	RABGAP1L	-1.18E-02	1.00E+00
CASP8AP2	7.95E-01	7.81E-06	RYK	-1.78E-02	1.00E+00	ANAPC10	-1.46E-02	1.00E+00

DRP2	-2.25E+00	7.81E-06	TRIM13	1.62E-02	1.00E+00	GRPEL2	-1.18E-02	1.00E+00
IL20RA	-2.33E+00	7.81E-06	TERB1	2.23E-02	1.00E+00	ECI2	-1.51E-02	1.00E+00
TRAPPC1	-6.60E-01	7.89E-06	YRDC	8.17E-02	1.00E+00	GJA1	-1.73E-02	1.00E+00
POLR2F	-7.01E-01	7.91E-06	BRD7	1.65E-02	1.00E+00	ULK1	-1.12E-02	1.00E+00
STT3A	-4.70E-01	7.96E-06	HIP1R	-1.95E-02	1.00E+00	LOC102389850	-1.14E-02	1.00E+00
PRMT2	-6.07E-01	7.98E-06	KIF17	-2.92E-02	1.00E+00	HTR3A	-4.27E-02	1.00E+00
NPHS2	1.07E+00	8.01E-06	DPF1	-2.68E-02	1.00E+00	GYS1	1.34E-02	1.00E+00
MYH10	6.19E-01	8.13E-06	NRXN1	-1.82E-02	1.00E+00	GDE1	1.18E-02	1.00E+00
REV1	4.99E-01	8.15E-06	RIMS4	3.02E-02	1.00E+00	CNPY2	1.49E-02	1.00E+00
LLPH	-6.86E-01	8.27E-06	TFR2	-4.90E-02	1.00E+00	DDX19A	1.03E-02	1.00E+00
NELFE	5.76E-01	8.28E-06	MRPL45	-2.62E-02	1.00E+00	NHLRC2	1.43E-02	1.00E+00
FMR1	5.88E-01	8.41E-06	MRPL21	-2.30E-02	1.00E+00	TIMMDC1	1.28E-02	1.00E+00
TMEM126A	-7.47E-01	8.44E-06	MRPL41	-2.89E-02	1.00E+00	LOC102401184	-2.02E-02	1.00E+00
ZNF639	-4.63E-01	8.47E-06	PRSS12	2.36E-02	1.00E+00	METAP1D	-1.26E-02	1.00E+00
BCR	-5.65E-01	8.50E-06	LOC102403514	-2.50E-02	1.00E+00	LOC112577683	-2.19E-02	1.00E+00
SCYL3	4.85E-01	8.51E-06	CCDC126	3.25E-02	1.00E+00	KIF21B	1.22E-02	1.00E+00
CHST7	1.36E+00	8.57E-06	TCTA	-3.10E-02	1.00E+00	LOC102402992	2.32E-02	1.00E+00
RAP1GDS1	-4.27E-01	8.58E-06	MRPL2	-2.36E-02	1.00E+00	RBL2	-1.57E-02	1.00E+00
LOC112584524	1.26E+00	8.71E-06	LOC102393261	2.89E-02	1.00E+00	LOC102413646	1.87E-02	1.00E+00
HSPA8	-8.61E-01	8.73E-06	CXHXorf38	-3.12E-02	1.00E+00	ATP5F1E	1.58E-02	1.00E+00
SPAG5	5.81E-01	8.85E-06	FBXO24	6.72E-02	1.00E+00	KBTBD4	-1.24E-02	1.00E+00
CTPS2	-4.85E-01	8.93E-06	ADAM12	-3.27E-02	1.00E+00	NOC2L	1.19E-02	1.00E+00
LNP1	6.17E-01	8.99E-06	LOC102400779	9.25E-02	1.00E+00	MPHOSPH8	9.71E-03	1.00E+00
TMEM38B	6.01E-01	9.03E-06	EPB41L4A	1.80E-02	1.00E+00	C21H3orf20	1.87E-02	1.00E+00
GLRX	9.28E-01	9.08E-06	TTLL9	1.22E-01	1.00E+00	ERBB2	1.04E-02	1.00E+00
PDCD6IP	-4.42E-01	9.09E-06	ERLIN1	-1.76E-02	1.00E+00	ZNF852	-2.12E-02	1.00E+00
LOC102398966	-7.67E-01	9.26E-06	SHD	2.74E-02	1.00E+00	LOC102406111	2.68E-02	1.00E+00
TMIE	-1.68E+00	9.28E-06	NSD1	-3.64E-02	1.00E+00	ARID2	-1.38E-02	1.00E+00
RASGRF2	1.39E+00	9.49E-06	RGMB	2.68E-02	1.00E+00	CHIC1	2.34E-02	1.00E+00
RNF167	5.21E-01	9.57E-06	IL17RC	1.27E-01	1.00E+00	SPHAR	2.26E-02	1.00E+00
ERICH6B	2.75E+00	9.59E-06	LYSMD1	3.28E-02	1.00E+00	TMEM258	1.37E-02	1.00E+00
SLCO4A1	-1.88E+00	9.61E-06	FAM32A	-2.35E-02	1.00E+00	RANBP10	-9.95E-03	1.00E+00
ABCA10	-1.68E+00	9.76E-06	SNAP91	-2.02E-02	1.00E+00	HSF1	1.09E-02	1.00E+00
ANGEL2	-5.73E-01	9.83E-06	LOC112580264	-4.83E-02	1.00E+00	ABHD17B	-1.18E-02	1.00E+00
MS4A13	2.74E+00	9.87E-06	PIGO	-3.83E-02	1.00E+00	MRPL2	-1.56E-02	1.00E+00

DARS2	-7.67E-01	9.89E-06	NPM1	-1.64E-02	1.00E+00	FBLN2	2.93E-02	1.00E+00
LIN54	-7.55E-01	1.00E-05	ZBTB4	3.92E-02	1.00E+00	TNFSF13	-2.76E-02	1.00E+00
PCMTD1	4.77E-01	1.01E-05	LOC102396645	8.64E-02	1.00E+00	SUPT4H1	-1.15E-02	1.00E+00
SMIM30	-6.19E-01	1.01E-05	LIN28A	2.71E-02	1.00E+00	ATXN3	-1.23E-02	1.00E+00
PLA2R1	-3.36E+00	1.01E-05	SCCPDH	-2.08E-02	1.00E+00	SNX2	9.46E-03	1.00E+00
HMGXB4	5.29E-01	1.02E-05	ACTR1A	1.91E-02	1.00E+00	C12H2orf49	1.13E-02	1.00E+00
SSBP1	4.94E-01	1.03E-05	TAF1D	5.66E-02	1.00E+00	ARHGAP42	-1.13E-02	1.00E+00
ACSM3	-1.61E+00	1.03E-05	DDX55	-2.38E-02	1.00E+00	ND4	-2.04E-02	1.00E+00
LOC112586463	2.56E+00	1.04E-05	SCFD1	-1.63E-02	1.00E+00	LOC102416301	-1.93E-02	1.00E+00
ZCCHC9	4.48E-01	1.04E-05	SART3	1.80E-02	1.00E+00	BUD31	-1.30E-02	1.00E+00
DCTN5	-4.72E-01	1.04E-05	C18H16orf70	2.05E-02	1.00E+00	EFTUD2	1.02E-02	1.00E+00
DDX58	6.98E-01	1.05E-05	ZNF19	-3.21E-02	1.00E+00	PSMG4	1.73E-02	1.00E+00
ZNF311	-9.87E-01	1.06E-05	VPS37D	2.94E-02	1.00E+00	DCTN4	1.14E-02	1.00E+00
RGS7	-8.12E-01	1.06E-05	PARL	1.79E-02	1.00E+00	MCMBP	-1.06E-02	1.00E+00
NAB1	-6.28E-01	1.08E-05	WDR62	-2.27E-02	1.00E+00	S1PR2	1.24E-02	1.00E+00
PHACTR2	-1.19E+00	1.09E-05	CDCP1	-3.47E-02	1.00E+00	FBXO46	1.18E-02	1.00E+00
LOC102396331	-2.19E+00	1.09E-05	IPO11	-2.93E-02	1.00E+00	RAB2A	9.54E-03	1.00E+00
PFDN4	5.81E-01	1.10E-05	SLC45A1	4.63E-02	1.00E+00	LOC112577692	2.02E-02	1.00E+00
SEMA4B	-7.69E-01	1.10E-05	SART1	-1.84E-02	1.00E+00	SSR4	1.65E-02	1.00E+00
ZSWIM1	-1.05E+00	1.11E-05	GPC6	3.31E-02	1.00E+00	CFAP54	1.64E-02	1.00E+00
POLR2K	6.26E-01	1.12E-05	YEATS4	1.68E-02	1.00E+00	AKR1A1	1.29E-02	1.00E+00
ZNF217	7.70E-01	1.12E-05	GALNT10	-2.53E-02	1.00E+00	SF3A1	-1.06E-02	1.00E+00
CYREN	5.18E-01	1.12E-05	APOO	-2.25E-02	1.00E+00	SLC37A4	1.03E-02	1.00E+00
KANSL2	-4.19E-01	1.13E-05	POLD1	-1.90E-02	1.00E+00	DHDDS	-1.03E-02	1.00E+00
GPAT3	-8.00E-01	1.14E-05	ZDHHC14	2.99E-02	1.00E+00	PPFIA3	-1.98E-02	1.00E+00
YY1	-3.73E-01	1.14E-05	COL16A1	3.54E-02	1.00E+00	WDR83	-1.43E-02	1.00E+00
FOXK2	3.64E-01	1.14E-05	LOC112586962	-3.32E-02	1.00E+00	TESK1	-1.03E-02	1.00E+00
HORMAD2	1.37E+00	1.15E-05	CASD1	2.23E-02	1.00E+00	CORO1C	1.04E-02	1.00E+00
ROBO2	-6.84E-01	1.15E-05	TRIM59	2.40E-02	1.00E+00	ENOSF1	-1.77E-02	1.00E+00
RGS1	-4.79E+00	1.16E-05	NOP16	-4.63E-02	1.00E+00	TEX264	-1.17E-02	1.00E+00
TMUB1	-7.37E-01	1.17E-05	EYA1	1.66E-02	1.00E+00	ZSWIM3	-2.36E-02	1.00E+00
SMYD2	5.60E-01	1.19E-05	BUB3	-1.64E-02	1.00E+00	LYPLAL1	-1.57E-02	1.00E+00
CCDC38	7.27E-01	1.19E-05	NELFCD	-1.80E-02	1.00E+00	SLC35F5	-1.16E-02	1.00E+00
EEF2KMT	6.16E-01	1.19E-05	TP53INP2	-3.90E-	1.00E+00	VKORC1	1.35E-02	1.00E+00

				02				
CCDC58	6.80E-01	1.20E-05	C23H10orf90	-3.93E-02	1.00E+00	RABGAP1	-1.05E-02	1.00E+00
RTL9	-1.77E+00	1.20E-05	LOC102405390	-2.08E-02	1.00E+00	THEM4	-1.19E-02	1.00E+00
HMGB3	-6.65E-01	1.20E-05	CCDC88B	1.06E-01	1.00E+00	ZCCHC7	-1.16E-02	1.00E+00
FAM110C	-2.17E+00	1.23E-05	ASIC4	-4.88E-02	1.00E+00	TRABD	1.17E-02	1.00E+00
CDHR3	1.25E+00	1.23E-05	LOC102416327	4.45E-02	1.00E+00	KAT14	-9.52E-03	1.00E+00
HSF2	-6.99E-01	1.23E-05	CDC6	-1.66E-02	1.00E+00	SF3B5	1.60E-02	1.00E+00
LOC112582978	-8.74E-01	1.24E-05	NPAS3	-2.00E-02	1.00E+00	CWF19L2	-1.06E-02	1.00E+00
CWF19L1	-4.78E-01	1.24E-05	SLC25A23	-2.97E-02	1.00E+00	TRIM54	2.77E-02	1.00E+00
LOC102411360	2.49E+00	1.25E-05	HAPLN4	4.67E-02	1.00E+00	GADD45GIP1	1.66E-02	1.00E+00
EPHX3	6.31E-01	1.25E-05	RNF14	1.50E-02	1.00E+00	CUL4A	9.50E-03	1.00E+00
LOC102391170	1.45E+00	1.26E-05	KHDC4	-2.64E-02	1.00E+00	POLB	-1.02E-02	1.00E+00
MAMDC2	2.31E+00	1.28E-05	MLLT3	2.37E-02	1.00E+00	PRELID3A	-1.31E-02	1.00E+00
CIZ1	-4.09E-01	1.28E-05	ARFIP1	-1.53E-02	1.00E+00	MBLAC1	1.76E-02	1.00E+00
PCNT	-4.16E-01	1.28E-05	MOCOS	4.61E-02	1.00E+00	ALDOA	1.61E-02	1.00E+00
SLC35A4	-5.34E-01	1.29E-05	ATCAY	-5.74E-02	1.00E+00	NIM1K	-2.20E-02	1.00E+00
WNT10B	1.36E+00	1.29E-05	LOC102399966	-2.11E-02	1.00E+00	VPS45	-9.98E-03	1.00E+00
ANKDD1B	8.74E-01	1.29E-05	LOC102403813	-7.44E-02	1.00E+00	PHC1	1.02E-02	1.00E+00
MTMR9	6.95E-01	1.30E-05	PPP2R3C	2.05E-02	1.00E+00	TOB2	1.84E-02	1.00E+00
RGS11	1.09E+00	1.31E-05	WDR76	1.73E-02	1.00E+00	MKKS	1.09E-02	1.00E+00
WASL	4.34E-01	1.32E-05	KANK2	2.73E-02	1.00E+00	RABL3	1.16E-02	1.00E+00
NXPH1	2.19E+00	1.32E-05	ACOX1	1.93E-02	1.00E+00	DIS3	1.16E-02	1.00E+00
MFF	3.95E-01	1.33E-05	NCL	-2.00E-02	1.00E+00	DGKE	-1.32E-02	1.00E+00
PTPRCAP	-1.31E+00	1.34E-05	SYTL3	-2.97E-02	1.00E+00	LOC112587647	-2.33E-02	1.00E+00
TCF19	5.75E-01	1.35E-05	TDRD3	2.04E-02	1.00E+00	UFC1	1.32E-02	1.00E+00
DLX2	2.32E+00	1.35E-05	LOC102399794	8.09E-02	1.00E+00	DYRK2	-1.10E-02	1.00E+00
PHRF1	4.24E-01	1.35E-05	RHOD	2.40E-02	1.00E+00	LOC102393546	-2.17E-02	1.00E+00
LOC102415739	-1.43E+00	1.36E-05	ACACA	-2.51E-02	1.00E+00	KAT6A	-1.27E-02	1.00E+00
LOC102412561	-1.30E+00	1.37E-05	GATA4	1.06E-01	1.00E+00	CACNA1S	2.31E-02	1.00E+00
ADM5	1.25E+00	1.37E-05	COL24A1	-2.39E-02	1.00E+00	KDELC1	-1.01E-02	1.00E+00
NACA	-4.77E-01	1.37E-05	SPAG5	1.70E-02	1.00E+00	FAM227A	2.55E-02	1.00E+00
PNMA2	-1.70E+00	1.38E-05	COLQ	-9.28E-02	1.00E+00	MND1	-1.53E-02	1.00E+00
BEND6	1.34E+00	1.38E-05	RSL24D1	-1.77E-02	1.00E+00	PSME3	9.16E-03	1.00E+00
METTL4	-1.59E+00	1.38E-05	ATG4A	2.20E-02	1.00E+00	AMZ2	1.20E-02	1.00E+00
IGSF21	2.37E+00	1.38E-05	ATF6B	3.59E-02	1.00E+00	RNF34	9.97E-03	1.00E+00

LOC102389034	3.15E+00	1.41E-05	WDR35	3.34E-02	1.00E+00	OFD1	-1.11E-02	1.00E+00
SNX31	-2.86E+00	1.41E-05	C7H4orf19	-3.05E-02	1.00E+00	PRR3	1.20E-02	1.00E+00
SLC2A12	-2.07E+00	1.42E-05	OTP	-7.80E-02	1.00E+00	CNOT2	-9.36E-03	1.00E+00
MMP1	-4.07E+00	1.43E-05	MAMSTR	-3.40E-02	1.00E+00	ALG11	1.17E-02	1.00E+00
ICAM1	1.87E+00	1.43E-05	DNAJB9	2.32E-02	1.00E+00	LOC102411494	-1.90E-02	1.00E+00
LOC102413919	-1.35E+00	1.43E-05	APOOL	1.31E-01	1.00E+00	AP3S1	1.14E-02	1.00E+00
UPF1	4.67E-01	1.45E-05	NEK11	-5.20E-02	1.00E+00	TLR3	-2.11E-02	1.00E+00
MYRF	1.02E+00	1.45E-05	MLXIP	-3.43E-02	1.00E+00	LOC112581227	-2.43E-02	1.00E+00
CCDC51	-5.88E-01	1.46E-05	LOC112584442	7.48E-02	1.00E+00	LOC112582071	-2.40E-02	1.00E+00
CBLB	-6.01E-01	1.46E-05	KIAA1107	-3.36E-02	1.00E+00	ESPN	-2.13E-02	1.00E+00
TRMT6	4.92E-01	1.47E-05	TTC21A	-3.06E-02	1.00E+00	NDUFS2	-1.34E-02	1.00E+00
DTD1	-5.51E-01	1.48E-05	GNPAT	1.83E-02	1.00E+00	LOC102395341	-1.29E-02	1.00E+00
PREX2	2.64E+00	1.48E-05	FRS3	5.32E-02	1.00E+00	WBP2NL	1.84E-02	1.00E+00
ZNF283	7.44E-01	1.48E-05	C5H1orf174	-3.80E-02	1.00E+00	ZNF26	1.38E-02	1.00E+00
AGFG1	3.79E-01	1.51E-05	GINS2	-1.81E-02	1.00E+00	HOMEZ	1.14E-02	1.00E+00
RREB1	-4.82E-01	1.52E-05	MRPL36	-2.37E-02	1.00E+00	LOC102411167	-1.45E-02	1.00E+00
PRMT1	-6.59E-01	1.53E-05	LOC102390897	-9.58E-02	1.00E+00	LOC112586667	-1.44E-02	1.00E+00
CAPS2	1.24E+00	1.57E-05	STK31	-1.55E-02	1.00E+00	TTC19	-1.04E-02	1.00E+00
LY9	-4.49E+00	1.59E-05	CCDC34	1.83E-02	1.00E+00	MRPL11	1.30E-02	1.00E+00
SOX21	3.57E+00	1.59E-05	PHF11	-1.98E-02	1.00E+00	RAB3B	2.30E-02	1.00E+00
ROBO4	-1.46E+00	1.60E-05	RRS1	1.94E-02	1.00E+00	EMC8	1.01E-02	1.00E+00
USP42	6.10E-01	1.62E-05	NETO1	4.16E-02	1.00E+00	CDK19	1.21E-02	1.00E+00
NCOA2	-6.42E-01	1.62E-05	RHBDF1	-2.60E-02	1.00E+00	PPTC7	-1.17E-02	1.00E+00
LOC102409316	-2.65E+00	1.62E-05	GCLM	2.16E-02	1.00E+00	ZFP69	1.35E-02	1.00E+00
GLRX5	-9.75E-01	1.63E-05	PSMC6	1.91E-02	1.00E+00	RPS20	-1.81E-02	1.00E+00
PRNP	-7.28E-01	1.63E-05	ZNF461	-3.96E-02	1.00E+00	SLC7A11	1.30E-02	1.00E+00
ZNF513	-4.60E-01	1.64E-05	DDX60	-3.89E-02	1.00E+00	EEF1D	-1.85E-02	1.00E+00
MIOS	4.32E-01	1.64E-05	CHGA	-2.95E-02	1.00E+00	C5H1orf112	1.09E-02	1.00E+00
BARD1	6.57E-01	1.65E-05	LOC102392710	3.01E-02	1.00E+00	ORC6	1.35E-02	1.00E+00
SSX2IP	5.40E-01	1.66E-05	NEDD4	-3.72E-02	1.00E+00	APCDD1	1.99E-02	1.00E+00
LOC112578790	-2.38E+00	1.66E-05	BBS12	2.08E-02	1.00E+00	CBLC	-2.20E-02	1.00E+00
PDHA1	6.55E-01	1.67E-05	PMS1	-1.85E-02	1.00E+00	TMCO1	1.09E-02	1.00E+00
DCTN3	-5.57E-01	1.67E-05	HVCN1	-3.55E-02	1.00E+00	CEP295	-9.91E-03	1.00E+00

AMZ1	-1.21E+00	1.67E-05	C6H1orf56	3.18E-02	1.00E+00	GPATCH3	-1.17E-02	1.00E+00
PTK2	-3.84E-01	1.68E-05	SRGAP1	1.52E-02	1.00E+00	SNX11	-1.07E-02	1.00E+00
LOC102407981	1.26E+00	1.68E-05	PLEKHA4	-7.27E-02	1.00E+00	KAT7	-9.53E-03	1.00E+00
SIMC1	4.99E-01	1.69E-05	LOC102396279	5.07E-02	1.00E+00	DPP6	-1.61E-02	1.00E+00
LOC102401256	-1.94E+00	1.70E-05	RANBP17	-3.23E-02	1.00E+00	COMMD6	1.22E-02	1.00E+00
CEP41	4.87E-01	1.70E-05	TSN	-1.75E-02	1.00E+00	LOC102398252	-2.25E-02	1.00E+00
MTREX	4.88E-01	1.71E-05	ATIC	1.45E-02	1.00E+00	LOC102402218	2.47E-02	1.00E+00
LOC102413767	-1.97E+00	1.72E-05	LOC102389727	-4.60E-02	1.00E+00	FNDC3A	-1.27E-02	1.00E+00
PRR30	-1.34E+00	1.72E-05	TWF1	3.14E-02	1.00E+00	LOC102390560	-1.61E-02	1.00E+00
LOC102414107	1.47E+00	1.73E-05	UBA2	-2.03E-02	1.00E+00	DYNLT3	1.08E-02	1.00E+00
CABP7	7.74E-01	1.75E-05	ITPK1	-2.02E-02	1.00E+00	CBX6	1.60E-02	1.00E+00
CREBBP	-6.60E-01	1.76E-05	EHD2	9.77E-02	1.00E+00	LOC102404801	2.51E-02	1.00E+00
ABI3	2.14E+00	1.76E-05	TRAF4	1.72E-02	1.00E+00	PSMB1	1.36E-02	1.00E+00
LOC102397476	-9.44E-01	1.77E-05	REXO5	-2.49E-02	1.00E+00	ACER1	-1.86E-02	1.00E+00
LOC102414909	-4.42E+00	1.77E-05	PGAM5	-1.62E-02	1.00E+00	MASP2	-1.69E-02	1.00E+00
ZNF524	-7.01E-01	1.79E-05	LOC112578567	1.03E-01	1.00E+00	ARL6	-1.41E-02	1.00E+00
ACTR10	-4.35E-01	1.79E-05	GK5	4.85E-02	1.00E+00	TRAPPC9	-9.73E-03	1.00E+00
LOC112581734	-2.48E+00	1.79E-05	CASP3	-2.68E-02	1.00E+00	TMEM206	9.28E-03	1.00E+00
FAM133A	1.38E+00	1.80E-05	LCLAT1	-2.25E-02	1.00E+00	LOC112580649	-1.22E-02	1.00E+00
SNX12	4.36E-01	1.81E-05	LOC102394921	3.97E-02	1.00E+00	WDR25	-1.26E-02	1.00E+00
RPS4X	-7.17E-01	1.81E-05	JPH4	-8.72E-02	1.00E+00	ZBTB10	1.24E-02	1.00E+00
OSTF1	-8.78E-01	1.83E-05	HSPA8	1.74E-02	1.00E+00	ABCE1	1.15E-02	1.00E+00
LY6G6C	1.63E+00	1.84E-05	BRF2	-2.30E-02	1.00E+00	TM7SF2	-1.50E-02	1.00E+00
PRKN	1.36E+00	1.85E-05	TUFT1	-1.87E-02	1.00E+00	SMAD5	1.10E-02	1.00E+00
PICK1	-5.85E-01	1.85E-05	COMT	4.54E-02	1.00E+00	STX8	1.24E-02	1.00E+00
BPNT1	4.53E-01	1.86E-05	SLC30A9	2.12E-02	1.00E+00	TOM1L2	8.79E-03	1.00E+00
VCL	-5.33E-01	1.86E-05	SRA1	-2.34E-02	1.00E+00	TIRAP	1.07E-02	1.00E+00
RETREG1	8.72E-01	1.86E-05	ZDHHC7	1.84E-02	1.00E+00	MDN1	1.24E-02	1.00E+00
SCML4	2.76E+00	1.86E-05	VCL	2.09E-02	1.00E+00	SMG5	-8.84E-03	1.00E+00
LONRF2	-8.95E-01	1.86E-05	ADCYAP1	-3.64E-02	1.00E+00	TULP2	1.35E-02	1.00E+00
TREM2	-3.73E+00	1.87E-05	WWP1	1.80E-02	1.00E+00	LOC102398210	1.46E-02	1.00E+00
SH2D4B	1.31E+00	1.87E-05	BRIX1	-1.91E-02	1.00E+00	CALM2	-1.15E-02	1.00E+00
TXNL1	-4.31E-01	1.89E-05	PRMT6	1.98E-02	1.00E+00	ABCD3	1.01E-02	1.00E+00
LOC102406285	-7.69E-	1.90E-05	CELSR3	1.75E-02	1.00E+00	TOR1B	8.84E-03	1.00E+00

	01							
S100A8	-4.54E+00	1.90E-05	MRPS10	-1.54E-02	1.00E+00	LOC102411496	1.86E-02	1.00E+00
CBX5	5.22E-01	1.90E-05	ZNF569	3.19E-02	1.00E+00	RFC3	-1.23E-02	1.00E+00
LOC112580682	1.91E+00	1.90E-05	MTHFD1L	-1.17E-01	1.00E+00	C21H3orf18	-1.72E-02	1.00E+00
IFIT2	1.22E+00	1.91E-05	MKRN1	-1.42E-02	1.00E+00	MPZL3	-1.64E-02	1.00E+00
MRPS21	-5.63E-01	1.94E-05	ZNRF3	3.26E-02	1.00E+00	EXD3	-1.07E-02	1.00E+00
SCAMP4	-4.87E-01	1.95E-05	LOC112580228	8.56E-02	1.00E+00	LOC112581531	-2.23E-02	1.00E+00
TRIM16	-2.20E+00	1.96E-05	TEN1	2.58E-02	1.00E+00	AAGAB	8.63E-03	1.00E+00
CAPS	-1.71E+00	1.97E-05	B4GALT2	1.57E-02	1.00E+00	ANAPC13	-1.44E-02	1.00E+00
AUH	4.46E-01	1.98E-05	MYDGF	2.18E-02	1.00E+00	SNX31	1.13E-02	1.00E+00
RGS19	1.06E+00	2.01E-05	XBP1	-1.57E-02	1.00E+00	SRPX2	1.87E-02	1.00E+00
LOC112580411	1.22E+00	2.02E-05	C14H20orf27	-1.67E-02	1.00E+00	ZNF839	-1.06E-02	1.00E+00
CSMD3	-1.99E+00	2.06E-05	FGFRL1	4.58E-02	1.00E+00	EIF3G	1.40E-02	1.00E+00
STPG2	2.24E+00	2.07E-05	QDPR	2.18E-02	1.00E+00	KIAA2026	1.39E-02	1.00E+00
HAX1	6.06E-01	2.10E-05	PPP1R14D	-9.40E-02	1.00E+00	ELOVL7	-1.84E-02	1.00E+00
CIB4	2.60E+00	2.12E-05	HIGD2A	1.73E-02	1.00E+00	NACAD	2.06E-02	1.00E+00
AGBL1	2.16E+00	2.12E-05	ASB16	-4.16E-02	1.00E+00	CAB39L	1.18E-02	1.00E+00
SLC26A11	-6.17E-01	2.13E-05	CACNG8	1.26E-01	1.00E+00	SEC23B	9.81E-03	1.00E+00
PDGFA	4.34E-01	2.15E-05	MIS12	1.72E-02	1.00E+00	LOC102408653	-2.03E-02	1.00E+00
FAHD1	5.81E-01	2.20E-05	RAB15	-1.74E-02	1.00E+00	LRRC27	1.05E-02	1.00E+00
LOC112583698	-1.46E+00	2.20E-05	EXOSC1	-2.18E-02	1.00E+00	TIMM13	-1.09E-02	1.00E+00
UGCG	-6.28E-01	2.22E-05	EIF3D	1.92E-02	1.00E+00	RPS8	1.95E-02	1.00E+00
LOC112579672	-1.82E+00	2.23E-05	DNAJC9	2.11E-02	1.00E+00	ASB7	-1.05E-02	1.00E+00
GLIPR2	-1.05E+00	2.23E-05	ADIPOR1	1.44E-02	1.00E+00	RAI1	1.08E-02	1.00E+00
ATXN10	-3.73E-01	2.23E-05	CACNA1B	-3.18E-02	1.00E+00	STAMBP	9.46E-03	1.00E+00
LOC102409615	-1.44E+00	2.24E-05	UROD	-2.35E-02	1.00E+00	IFT80	1.47E-02	1.00E+00
VIPR2	-3.08E+00	2.24E-05	CHMP7	1.37E-02	1.00E+00	GTF3C3	-1.29E-02	1.00E+00
ACTRT3	-8.56E-01	2.24E-05	YBX2	-1.79E-02	1.00E+00	LOC112579226	1.64E-02	1.00E+00
HIGD1B	1.33E+00	2.25E-05	SPDL1	1.51E-02	1.00E+00	SHLD2	1.25E-02	1.00E+00
EEF1AKMT3	-9.27E-01	2.27E-05	MAP4K1	-2.27E-02	1.00E+00	LOC112578444	1.50E-02	1.00E+00
ARF6	5.03E-01	2.29E-05	VPS26C	1.94E-02	1.00E+00	FOCAD	-9.14E-03	1.00E+00
LOC112585150	2.01E+00	2.30E-05	LOC102395631	-1.23E-01	1.00E+00	LOC112583901	1.35E-02	1.00E+00
LOC102402269	-1.09E+00	2.31E-05	PTRHD1	-2.39E-02	1.00E+00	SNX18	-1.18E-02	1.00E+00
UXS1	4.66E-01	2.31E-05	YY1	-1.58E-02	1.00E+00	CSNK2A1	8.21E-03	1.00E+00

LMBRD2	-7.23E-01	2.32E-05	TMEM167A	1.61E-02	1.00E+00	LOC102400529	-1.89E-02	1.00E+00
HOMER1	-7.06E-01	2.33E-05	ILF3	1.96E-02	1.00E+00	TSPYL4	-1.02E-02	1.00E+00
LOC102398520	-1.43E+00	2.33E-05	CERS1	-4.09E-02	1.00E+00	SAXO2	-1.84E-02	1.00E+00
ZC3H12D	-1.35E+00	2.33E-05	TMEM175	3.65E-02	1.00E+00	TMEM184C	9.39E-03	1.00E+00
LOC102403267	-3.79E+00	2.35E-05	GMPR2	-1.72E-02	1.00E+00	TSPAN6	-1.08E-02	1.00E+00
CDK16	-4.19E-01	2.37E-05	THOC3	1.51E-02	1.00E+00	SLC25A19	1.12E-02	1.00E+00
PTCD1	-5.56E-01	2.38E-05	ZNF507	-3.18E-02	1.00E+00	BRCC3	-8.64E-03	1.00E+00
ZNF184	4.90E-01	2.41E-05	GRB2	-1.64E-02	1.00E+00	MARS	-8.46E-03	1.00E+00
CEACAM1	-2.48E+00	2.41E-05	DPH3	-2.49E-02	1.00E+00	RFXAP	1.07E-02	1.00E+00
SGMS1	-5.39E-01	2.42E-05	GPANK1	1.61E-02	1.00E+00	APC	1.27E-02	1.00E+00
TRAPPC4	-5.25E-01	2.43E-05	SMCR8	3.04E-02	1.00E+00	MTG2	1.14E-02	1.00E+00
MANEAL	-1.01E+00	2.44E-05	KITLG	2.81E-02	1.00E+00	DOK3	2.14E-02	1.00E+00
FTSJ1	7.35E-01	2.44E-05	LOC102409478	4.44E-02	1.00E+00	TMED5	1.44E-02	1.00E+00
BAALC	1.04E+00	2.46E-05	AGFG2	-2.16E-02	1.00E+00	CREBBP	-1.19E-02	1.00E+00
COPZ2	-6.38E-01	2.46E-05	TKT	-3.18E-02	1.00E+00	LOC102409316	1.69E-02	1.00E+00
PDE6G	-1.72E+00	2.46E-05	DNAJC13	-2.43E-02	1.00E+00	FARSA	8.70E-03	1.00E+00
MDFI	1.87E+00	2.46E-05	TMEM127	-1.96E-02	1.00E+00	BRWD1	9.07E-03	1.00E+00
ARF4	-5.62E-01	2.46E-05	STOML1	-6.71E-02	1.00E+00	LOC112583093	2.23E-02	1.00E+00
LOC102409925	-3.01E+00	2.47E-05	EIF5A	1.62E-02	1.00E+00	ZNF341	1.06E-02	1.00E+00
PPP2R1A	-5.48E-01	2.52E-05	KIAA0753	-1.82E-02	1.00E+00	CEP135	-1.29E-02	1.00E+00
CHIC1	-1.48E+00	2.54E-05	LOC102394892	4.19E-02	1.00E+00	SESN1	1.23E-02	1.00E+00
LOC102413819	-4.24E-01	2.55E-05	FZD4	-1.66E-02	1.00E+00	FKBP2	1.32E-02	1.00E+00
ZSWIM4	-9.18E-01	2.56E-05	SYNGR2	1.94E-02	1.00E+00	C2H6orf226	-1.33E-02	1.00E+00
LOC112580679	-1.62E+00	2.58E-05	PGRMC1	1.85E-02	1.00E+00	GNL3L	-8.02E-03	1.00E+00
NAF1	-5.16E-01	2.59E-05	LOC102403853	-6.73E-02	1.00E+00	IDH3G	-1.13E-02	1.00E+00
LOC102406612	2.28E+00	2.59E-05	SNAPC2	-2.69E-02	1.00E+00	SH3BGRL2	-1.60E-02	1.00E+00
NCF4	-2.70E+00	2.60E-05	C2CD4A	-5.73E-02	1.00E+00	FUCA2	-1.23E-02	1.00E+00
IQCG	-1.21E+00	2.60E-05	FMO5	-1.75E-02	1.00E+00	SYNJ1	1.41E-02	1.00E+00
INF2	-2.29E+00	2.61E-05	BLCAP	-1.60E-02	1.00E+00	ACRV1	-2.17E-02	1.00E+00
SKI	5.34E-01	2.62E-05	PPP2R5A	-4.99E-02	1.00E+00	SCARF1	-1.63E-02	1.00E+00
PIK3AP1	1.06E+00	2.62E-05	NDST3	8.69E-02	1.00E+00	SCAPER	-9.80E-03	1.00E+00
LOC112586545	-3.11E+00	2.63E-05	ALAD	1.88E-02	1.00E+00	RPS18	1.65E-02	1.00E+00
CIAO2A	-6.96E-	2.64E-05	ERICH3	5.30E-02	1.00E+00	BROX	1.17E-02	1.00E+00

	01							
HMGCS1	-5.09E-01	2.65E-05	TRNAG-GCC-12	4.73E-02	1.00E+00	LOC102389376	-9.38E-03	1.00E+00
LOC102398046	-1.69E+00	2.68E-05	FLNB	-2.11E-02	1.00E+00	SNUPN	-1.02E-02	1.00E+00
KNSTRN	5.95E-01	2.69E-05	CNP	-1.58E-02	1.00E+00	LOC112587785	1.21E-02	1.00E+00
IDE	-4.46E-01	2.70E-05	ANK2	1.62E-02	1.00E+00	ARL6IP5	8.35E-03	1.00E+00
HEY2	5.57E-01	2.71E-05	LOC112587293	-8.91E-02	1.00E+00	SLC22A5	1.01E-02	1.00E+00
SYPL2	2.15E+00	2.72E-05	ATXN7L3B	1.85E-02	1.00E+00	SPG7	-9.59E-03	1.00E+00
TSTD2	-5.56E-01	2.72E-05	LOC112582097	3.27E-02	1.00E+00	XIAP	-1.41E-02	1.00E+00
CRLF2	-3.73E+00	2.74E-05	LOC112586983	-6.81E-02	1.00E+00	BANF1	1.30E-02	1.00E+00
OTULIN	-4.90E-01	2.74E-05	ATP5F1A	1.56E-02	1.00E+00	WDR70	8.65E-03	1.00E+00
LOC102409758	-1.81E+00	2.74E-05	TRIM47	1.12E-01	1.00E+00	MTCH1	8.26E-03	1.00E+00
WDR46	-5.62E-01	2.75E-05	MGARP	-9.55E-02	1.00E+00	HNRNPM	1.09E-02	1.00E+00
MRPS16	6.49E-01	2.81E-05	NOP56	2.01E-02	1.00E+00	TPGS2	-9.25E-03	1.00E+00
LOC102400859	-1.92E+00	2.82E-05	LOC112586867	2.87E-02	1.00E+00	CCDC171	-1.36E-02	1.00E+00
CACNG4	1.60E+00	2.83E-05	PINK1	1.98E-02	1.00E+00	CEP41	-1.04E-02	1.00E+00
SCLT1	-7.58E-01	2.87E-05	IFT27	3.95E-02	1.00E+00	EPN1	-1.02E-02	1.00E+00
ARHGDIB	-2.32E+00	2.87E-05	PIGG	8.94E-02	1.00E+00	NUDT5	1.12E-02	1.00E+00
CEP295	-5.06E-01	2.88E-05	LOC112580153	8.80E-02	1.00E+00	LOC112580209	1.95E-02	1.00E+00
LOC112578850	1.17E+00	2.89E-05	TMC4	-1.64E-02	1.00E+00	SFRP2	3.13E-02	1.00E+00
LOC102394061	9.17E-01	2.91E-05	CRYBB2	3.22E-02	1.00E+00	EIF2B5	8.30E-03	1.00E+00
STX8	5.95E-01	2.92E-05	RTN2	-3.65E-02	1.00E+00	RCL1	-1.07E-02	1.00E+00
LOC102404456	-7.40E-01	2.95E-05	PLBD2	-2.43E-02	1.00E+00	TOMM40L	-9.30E-03	1.00E+00
RALGAPB	5.61E-01	2.96E-05	ODC1	-1.76E-02	1.00E+00	LOC102413681	1.44E-02	1.00E+00
GPS2	-5.63E-01	2.98E-05	GREB1	1.06E-01	1.00E+00	QPRT	-1.30E-02	1.00E+00
TMEM63B	4.31E-01	3.01E-05	TMPRSS3	3.64E-02	1.00E+00	LOC102412652	-1.12E-02	1.00E+00
LSM1	5.99E-01	3.03E-05	SNX7	-5.43E-02	1.00E+00	AP1M1	8.62E-03	1.00E+00
ESR2	6.66E-01	3.03E-05	ELOVL4	-2.54E-02	1.00E+00	NAA50	-1.27E-02	1.00E+00
SULT2B1	1.52E+00	3.04E-05	USPL1	6.78E-02	1.00E+00	RASA2	1.52E-02	1.00E+00
EPHB6	1.35E+00	3.05E-05	DGKK	-1.16E-01	1.00E+00	CABLES2	-9.37E-03	1.00E+00
PAN2	-4.57E-01	3.09E-05	ADIRF	3.56E-02	1.00E+00	ENOX2	9.88E-03	1.00E+00
LOC112583923	-1.09E+00	3.11E-05	CAV2	-7.91E-02	1.00E+00	OAZ1	1.17E-02	1.00E+00
LOC102412960	1.16E+00	3.11E-05	CDC23	2.80E-02	1.00E+00	CPLANE1	-9.20E-03	1.00E+00
ARPC3	-4.89E-01	3.11E-05	WIPF3	-5.62E-02	1.00E+00	MIGA2	8.38E-03	1.00E+00

SNAPC5	9.86E-01	3.12E-05	NUCB1	2.13E-02	1.00E+00	ATL3	-9.28E-03	1.00E+00
LOC102399019	-1.29E+00	3.14E-05	TOMM20	2.58E-02	1.00E+00	SNRPD3	-1.28E-02	1.00E+00
CTNNAL1	-3.75E-01	3.14E-05	PCGF6	2.75E-02	1.00E+00	DLGAP1	-9.92E-03	1.00E+00
SET	4.86E-01	3.14E-05	PALB2	1.52E-02	1.00E+00	VSTM2L	2.19E-02	1.00E+00
ABHD5	-6.54E-01	3.16E-05	CCDC153	6.86E-02	1.00E+00	TBC1D12	-1.19E-02	1.00E+00
LOC102399825	-7.22E-01	3.18E-05	LOC112584570	5.11E-02	1.00E+00	LOC102395369	1.81E-02	1.00E+00
NSA2	-5.27E-01	3.18E-05	PQLC2	-3.00E-02	1.00E+00	LOC102403613	1.29E-02	1.00E+00
TRPM4	-9.28E-01	3.20E-05	ADGRE5	2.10E-02	1.00E+00	CAPZA2	7.77E-03	1.00E+00
JARID2	6.58E-01	3.21E-05	FBXW5	-2.75E-02	1.00E+00	IFT22	9.07E-03	1.00E+00
CHERP	-5.91E-01	3.23E-05	ITGA8	-2.58E-02	1.00E+00	GTPBP8	8.79E-03	1.00E+00
SEC24D	-5.53E-01	3.23E-05	LOC112584382	-3.74E-02	1.00E+00	C24H16orf71	2.06E-02	1.00E+00
MEIOB	1.50E+00	3.25E-05	ABCC3	-5.30E-02	1.00E+00	LSM7	-1.38E-02	1.00E+00
ZNF248	6.06E-01	3.25E-05	ATG9B	6.23E-02	1.00E+00	MRPS7	1.02E-02	1.00E+00
NATD1	5.29E-01	3.26E-05	RIMKLA	-1.40E-02	1.00E+00	MAP3K12	-8.30E-03	1.00E+00
PAK4	-5.16E-01	3.30E-05	ZAR1L	1.51E-02	1.00E+00	ANKMY2	-1.74E-02	1.00E+00
RUFY2	5.28E-01	3.30E-05	AMER1	2.17E-02	1.00E+00	ZFYVE21	-9.77E-03	1.00E+00
ENTPD4	4.99E-01	3.32E-05	LOC102403713	-2.10E-02	1.00E+00	NDUFB7	1.22E-02	1.00E+00
QRFPR	1.20E+00	3.33E-05	CHD9	-4.75E-02	1.00E+00	LOC102415369	-1.41E-02	1.00E+00
SPTLC3	-1.18E+00	3.37E-05	RBM39	1.39E-02	1.00E+00	COLQ	1.32E-02	1.00E+00
NAMPT	5.84E-01	3.37E-05	UNC79	5.34E-02	1.00E+00	PPA2	9.78E-03	1.00E+00
TSPAN19	2.37E+00	3.44E-05	VPS4A	1.45E-02	1.00E+00	SRPK1	-7.72E-03	1.00E+00
NPTX1	-1.82E+00	3.47E-05	PPP1R3F	2.40E-02	1.00E+00	HIC2	1.54E-02	1.00E+00
DNAJA2	3.80E-01	3.49E-05	EEF1E1	-3.69E-02	1.00E+00	ATAD1	8.02E-03	1.00E+00
TBCK	5.08E-01	3.51E-05	TRMT11	-1.84E-02	1.00E+00	LOC102389969	-1.64E-02	1.00E+00
MYT1L	2.27E+00	3.52E-05	RNASET2	-2.80E-02	1.00E+00	MFSD11	-9.94E-03	1.00E+00
ANKFN1	-2.31E+00	3.53E-05	LMNB1	-6.20E-02	1.00E+00	NOL10	7.67E-03	1.00E+00
FNDC10	-6.09E-01	3.55E-05	CDC34	-1.71E-02	1.00E+00	MITD1	-1.28E-02	1.00E+00
SH2D1B	2.36E+00	3.55E-05	AP1M2	1.34E-02	1.00E+00	DUT	-1.51E-02	1.00E+00
RDH10	-4.86E-01	3.56E-05	PDCL	-4.19E-02	1.00E+00	EXOC8	1.06E-02	1.00E+00
IL9R	-1.32E+00	3.59E-05	AMPH	3.15E-02	1.00E+00	NFYB	-8.17E-03	1.00E+00
NEO1	-4.87E-01	3.60E-05	PSMA4	1.76E-02	1.00E+00	KIAA0556	8.56E-03	1.00E+00
SS18L1	-1.09E+00	3.61E-05	LRCH4	1.36E-02	1.00E+00	THAP8	-1.04E-02	1.00E+00
DIXDC1	-7.03E-01	3.63E-05	ASPHD2	-1.99E-02	1.00E+00	NEK3	1.04E-02	1.00E+00

OVOL2	3.17E+00	3.63E-05	KCNV2	-8.01E-02	1.00E+00	ZDHHC20	9.29E-03	1.00E+00
THADA	-4.34E-01	3.64E-05	HSH2D	2.71E-02	1.00E+00	PSMC4	-8.41E-03	1.00E+00
CDSN	-1.35E+00	3.64E-05	TMEM35A	2.53E-02	1.00E+00	LOC102410603	-1.60E-02	1.00E+00
ND2	-1.02E+00	3.65E-05	TSTD2	2.90E-02	1.00E+00	THYN1	-1.13E-02	1.00E+00
IFIT3	9.87E-01	3.66E-05	SNRNP27	-1.71E-02	1.00E+00	LOC102415514	-1.25E-02	1.00E+00
RPA3	-6.14E-01	3.68E-05	RAB3D	-1.81E-02	1.00E+00	COPZ2	1.12E-02	1.00E+00
EEFSEC	-6.20E-01	3.68E-05	GPAM	4.26E-02	1.00E+00	C2CD2L	9.98E-03	1.00E+00
LOC112586992	-1.61E+00	3.69E-05	LOC102416055	-5.32E-02	1.00E+00	TNFAIP8L2	3.08E-02	1.00E+00
LOC112587318	3.15E+00	3.69E-05	SHCBP1	-1.32E-02	1.00E+00	PFN1	-1.01E-02	1.00E+00
TIE1	-9.43E-01	3.73E-05	SPAAR	5.90E-02	1.00E+00	INO80B	-9.58E-03	1.00E+00
NHEJ1	-6.32E-01	3.73E-05	FAM174A	2.87E-02	1.00E+00	BTBD7	8.22E-03	1.00E+00
L3MBTL3	-5.73E-01	3.76E-05	URI1	-2.54E-02	1.00E+00	LOC102399272	-1.03E-02	1.00E+00
LOC112584001	9.60E-01	3.80E-05	WDR60	-3.25E-02	1.00E+00	SNTA1	1.45E-02	1.00E+00
LOC102416423	1.15E+00	3.80E-05	POP7	-1.63E-02	1.00E+00	ACBD6	9.86E-03	1.00E+00
DUSP18	-8.03E-01	3.82E-05	SCN4B	-2.25E-02	1.00E+00	ZNF335	8.01E-03	1.00E+00
CLPTM1	4.34E-01	3.88E-05	RCOR3	-2.75E-02	1.00E+00	YME1L1	7.27E-03	1.00E+00
CFAP61	9.73E-01	3.89E-05	THNSL1	2.87E-02	1.00E+00	MRC2	-1.22E-02	1.00E+00
VSTM5	7.50E-01	3.92E-05	BNIP3L	-1.29E-02	1.00E+00	SLC9B1	9.44E-03	1.00E+00
XK	7.49E-01	3.93E-05	TCEANC	3.97E-02	1.00E+00	LOC102396260	-8.30E-03	1.00E+00
MED13L	-6.12E-01	3.94E-05	LOC102406763	1.68E-02	1.00E+00	KDM2B	8.61E-03	1.00E+00
SRFBP1	5.94E-01	3.96E-05	LOC112578482	-3.74E-02	1.00E+00	GTPBP2	-1.00E-02	1.00E+00
TAF7	4.09E-01	4.02E-05	CSNK1G2	-1.28E-02	1.00E+00	VPS37D	1.33E-02	1.00E+00
GSK3A	-3.84E-01	4.02E-05	PHC2	1.65E-02	1.00E+00	CLCN4	-1.35E-02	1.00E+00
FBXL2	-7.17E-01	4.04E-05	ABHD17C	1.36E-02	1.00E+00	CFD	-2.81E-02	1.00E+00
LOC102403487	6.89E-01	4.07E-05	BBIP1	-1.76E-02	1.00E+00	TECPR2	8.09E-03	1.00E+00
SLC4A9	1.89E+00	4.20E-05	LOC112583679	-2.78E-02	1.00E+00	WDFY2	1.13E-02	1.00E+00
LOC112581186	-1.16E+00	4.21E-05	KIF23	1.87E-02	1.00E+00	SDR39U1	1.28E-02	1.00E+00
LOC112583579	2.24E+00	4.23E-05	TTC1	-1.72E-02	1.00E+00	MCPH1	-7.41E-03	1.00E+00
TYW5	-6.59E-01	4.30E-05	PAQR3	-2.44E-02	1.00E+00	PTGR1	1.41E-02	1.00E+00
CYTH4	1.91E+00	4.31E-05	ZNF362	2.02E-02	1.00E+00	VEGFA	-1.21E-02	1.00E+00
LOC112578778	-1.63E+00	4.31E-05	LOC102404230	-3.40E-02	1.00E+00	NUDT2	1.16E-02	1.00E+00
TMEM11	5.54E-01	4.32E-05	SSUH2	-2.13E-02	1.00E+00	SLC4A2	7.32E-03	1.00E+00

LOC102412511	-4.31E-01	4.38E-05	WDR78	4.15E-02	1.00E+00	SUMF2	-9.44E-03	1.00E+00
FAM89A	7.43E-01	4.42E-05	TMEM70	-4.09E-02	1.00E+00	MRPS23	-9.88E-03	1.00E+00
PATZ1	-4.59E-01	4.42E-05	VSTM2L	-4.95E-02	1.00E+00	CMTR1	-7.22E-03	1.00E+00
ITPRIPL1	-6.06E-01	4.49E-05	GCNA	1.00E-01	1.00E+00	DHX34	9.48E-03	1.00E+00
ATG4D	-4.90E-01	4.61E-05	TAF1B	1.82E-02	1.00E+00	JMJD4	-7.87E-03	1.00E+00
FAM102B	9.53E-01	4.63E-05	CARS	-1.68E-02	1.00E+00	DUSP15	1.24E-02	1.00E+00
OTOA	-2.08E+00	4.64E-05	PIK3R4	2.02E-02	1.00E+00	LOC102416310	1.85E-02	1.00E+00
LOXL4	-1.03E+00	4.64E-05	LOC112586829	-3.02E-02	1.00E+00	PPIP5K1	-1.25E-02	1.00E+00
XPC	-4.89E-01	4.74E-05	FKBP8	1.59E-02	1.00E+00	ARIH1	7.28E-03	1.00E+00
CHMP1A	4.46E-01	4.77E-05	LOC102394862	-4.71E-02	1.00E+00	ANTXR1	9.68E-03	1.00E+00
WDR1	-4.53E-01	4.80E-05	MAEA	-1.32E-02	1.00E+00	ALKBH3	1.05E-02	1.00E+00
NKD2	-1.32E+00	4.81E-05	COPB2	-1.38E-02	1.00E+00	TERF2IP	-7.06E-03	1.00E+00
CWF19L2	3.84E-01	4.83E-05	GPBAR1	-2.21E-02	1.00E+00	LOC102406873	-1.07E-02	1.00E+00
LOC112578672	1.51E+00	4.88E-05	SYTL4	-2.21E-02	1.00E+00	PDF	1.17E-02	1.00E+00
FAM71A	-3.84E+00	4.88E-05	GCNT1	1.91E-02	1.00E+00	ZNF23	9.31E-03	1.00E+00
SYNJ1	6.24E-01	4.89E-05	ZNF804A	5.65E-02	1.00E+00	TMEM175	8.21E-03	1.00E+00
LOC112585765	-1.27E+00	4.90E-05	ACTL8	1.65E-02	1.00E+00	GTF3C4	-1.79E-02	1.00E+00
CCNJ	1.08E+00	4.91E-05	LEPROTL1	1.99E-02	1.00E+00	CHMP4A	1.13E-02	1.00E+00
ATG10	-1.10E+00	4.91E-05	ZACN	4.35E-02	1.00E+00	PUM1	-1.03E-02	1.00E+00
FEZ2	4.10E-01	4.92E-05	TRIM21	1.22E-01	1.00E+00	ATG5	7.86E-03	1.00E+00
MIPOL1	6.64E-01	4.97E-05	XRCC6	1.27E-02	1.00E+00	NSFL1C	-1.01E-02	1.00E+00
PDCD1	1.67E+00	5.01E-05	SRCIN1	2.10E-02	1.00E+00	LOC102404010	8.34E-03	1.00E+00
GAL3ST2	1.14E+00	5.02E-05	TDRD7	-1.42E-02	1.00E+00	MED20	7.69E-03	1.00E+00
PDF	-7.44E-01	5.04E-05	PCDH19	-8.21E-02	1.00E+00	SYNPR	9.58E-03	1.00E+00
LOC102408234	1.34E+00	5.10E-05	XRN1	-3.43E-02	1.00E+00	TIMM50	9.59E-03	1.00E+00
ORMDL1	1.11E+00	5.10E-05	LOC102392066	2.69E-02	1.00E+00	FBL	-1.15E-02	1.00E+00
ST7L	-5.03E-01	5.12E-05	LRRC47	-2.40E-02	1.00E+00	QPCT	-1.99E-02	1.00E+00
LOC102408728	-1.07E+00	5.12E-05	LOC102410978	4.76E-02	1.00E+00	XYLB	1.57E-02	1.00E+00
TRPM2	-3.63E+00	5.14E-05	MGAT1	-1.58E-02	1.00E+00	ENKD1	-9.64E-03	1.00E+00
DCTN1	-4.21E-01	5.15E-05	RAB4B	-2.02E-02	1.00E+00	LOC112580833	-2.19E-02	1.00E+00
ACOX1	-3.80E-01	5.19E-05	ARMT1	-5.84E-02	1.00E+00	SEC13	-8.14E-03	1.00E+00
ALDH1A3	-2.66E+00	5.20E-05	THBS1	-3.67E-02	1.00E+00	PIGG	8.17E-03	1.00E+00
MOB1B	-5.07E-01	5.21E-05	DAPK3	1.50E-02	1.00E+00	DPY19L3	-8.39E-03	1.00E+00
MYO1G	-	5.22E-05	KCNQ3	9.24E-02	1.00E+00	PPP1CC	-6.91E-	1.00E+00

	4.20E+00						03	
ZBTB18	-5.85E-01	5.23E-05	PSMC3	2.02E-02	1.00E+00	CNOT6L	8.26E-03	1.00E+00
MTIF2	5.33E-01	5.28E-05	MAN1A2	2.46E-02	1.00E+00	RNASEK	-8.65E-03	1.00E+00
INCENP	4.95E-01	5.29E-05	CRYBG1	2.11E-02	1.00E+00	NUDT22	-1.19E-02	1.00E+00
CIC	4.57E-01	5.31E-05	LOC112578525	-2.91E-02	1.00E+00	LOC112587382	-1.22E-02	1.00E+00
LOC102401184	-9.53E-01	5.32E-05	LOC102391085	1.60E-02	1.00E+00	UAP1L1	8.47E-03	1.00E+00
C21H3orf67	4.74E-01	5.37E-05	STXBP5L	-1.77E-02	1.00E+00	GALK2	-8.33E-03	1.00E+00
HOXB4	1.38E+00	5.42E-05	OSGEP	3.52E-02	1.00E+00	ABCF1	7.39E-03	1.00E+00
RBFOX3	1.02E+00	5.42E-05	GPC1	3.74E-02	1.00E+00	SLC24A5	7.39E-03	1.00E+00
ERICH1	-5.73E-01	5.43E-05	NPHS1	9.17E-02	1.00E+00	MRPL44	-7.95E-03	1.00E+00
STEAP3	6.85E-01	5.52E-05	LOC112587789	-6.44E-02	1.00E+00	PTPN21	8.07E-03	1.00E+00
MED10	4.63E-01	5.55E-05	CERK	-1.11E-01	1.00E+00	MORF4L2	-7.76E-03	1.00E+00
LOC102389990	-1.25E+00	5.57E-05	WASHC3	2.35E-02	1.00E+00	HPRT1	-7.82E-03	1.00E+00
LOC112577988	-2.47E+00	5.61E-05	PPP1R8	-1.89E-02	1.00E+00	CMAS	6.51E-03	1.00E+00
LOC102407761	4.21E-01	5.62E-05	WWTR1	-6.68E-02	1.00E+00	FNTA	6.77E-03	1.00E+00
FLII	4.21E-01	5.65E-05	ARID5A	1.72E-02	1.00E+00	COPB2	6.98E-03	1.00E+00
TULP2	-6.77E-01	5.68E-05	ADCK2	2.29E-02	1.00E+00	CRISPLD2	-1.49E-02	1.00E+00
MKS1	4.59E-01	5.73E-05	SLC24A5	6.38E-02	1.00E+00	RTL6	-8.36E-03	1.00E+00
LOC102402188	-2.77E+00	5.75E-05	IL6ST	-2.82E-02	1.00E+00	BTBD10	-8.41E-03	1.00E+00
DNPH1	7.54E-01	5.79E-05	SEMA3F	4.37E-02	1.00E+00	NCOA3	-9.06E-03	1.00E+00
U2AF1L4	-5.38E-01	5.81E-05	ORC3	1.34E-02	1.00E+00	ATP6V1C1	-6.88E-03	1.00E+00
FOXO4	-6.01E-01	5.82E-05	PTCH2	-1.73E-02	1.00E+00	ERAS	1.46E-02	1.00E+00
CETN2	4.79E-01	5.84E-05	GEMIN5	-1.92E-02	1.00E+00	GPNMB	-3.06E-02	1.00E+00
LOC112580253	-1.06E+00	5.84E-05	GBA	2.94E-02	1.00E+00	SHMT1	-1.43E-02	1.00E+00
LOC112578078	3.33E+00	5.84E-05	DDN	4.83E-02	1.00E+00	KLHL12	6.90E-03	1.00E+00
LOC112581638	-1.36E+00	5.88E-05	CRHR1	4.17E-02	1.00E+00	TMEM106B	1.01E-02	1.00E+00
PGAP3	-4.40E-01	5.94E-05	HSP90AB1	1.44E-02	1.00E+00	LOC102400303	-3.31E-02	1.00E+00
NAAA	9.87E-01	6.00E-05	ZNF444	-2.32E-02	1.00E+00	MUC4	-2.58E-02	1.00E+00
PIK3C2A	-6.89E-01	6.01E-05	SLC4A7	4.28E-02	1.00E+00	GGT7	-1.51E-02	1.00E+00
TTL	-5.21E-01	6.02E-05	FAM214A	1.77E-02	1.00E+00	RPS6KB2	8.47E-03	1.00E+00
PPIP5K1	6.21E-01	6.06E-05	CDC14B	2.25E-02	1.00E+00	CASKIN2	-7.90E-03	1.00E+00
TSPAN13	4.04E-01	6.09E-05	ITPRIPL1	-3.07E-02	1.00E+00	LSM4	9.88E-03	1.00E+00
ABCC10	-5.85E-01	6.13E-05	AADAT	-6.07E-02	1.00E+00	FYN	7.59E-03	1.00E+00
ARID3A	-7.62E-01	6.19E-05	INPP4A	1.59E-02	1.00E+00	RBPMS	-7.61E-03	1.00E+00

LOC112586916	-1.24E+00	6.22E-05	SF3B1	1.66E-02	1.00E+00	FAM241B	9.74E-03	1.00E+00
LOC102407764	2.32E+00	6.23E-05	LOC102400830	-1.38E-02	1.00E+00	LOC102398665	7.98E-03	1.00E+00
ZNF667	-5.93E-01	6.31E-05	MLX	1.65E-02	1.00E+00	NLGN2	-7.92E-03	1.00E+00
DNAJA4	4.64E-01	6.36E-05	HSD11B1L	-3.95E-02	1.00E+00	ZNF865	9.12E-03	1.00E+00
COL15A1	-2.11E+00	6.44E-05	NFX1	-1.84E-02	1.00E+00	AHCYL1	-6.47E-03	1.00E+00
BANF1	-6.43E-01	6.53E-05	LOC112586827	-5.35E-02	1.00E+00	TFAM	-7.72E-03	1.00E+00
DCPS	-5.72E-01	6.55E-05	CPT1A	5.30E-02	1.00E+00	IPMK	1.13E-02	1.00E+00
LOC102397739	-1.88E+00	6.65E-05	MBTPS1	-2.38E-02	1.00E+00	OSTC	-8.50E-03	1.00E+00
LOC102405871	6.16E-01	6.65E-05	ARX	6.43E-02	1.00E+00	LOC102413767	1.89E-02	1.00E+00
POLE	5.53E-01	6.75E-05	WBP2NL	-4.55E-02	1.00E+00	ATP11A	-1.36E-02	1.00E+00
LOC102403559	5.00E-01	6.78E-05	LGALS7	5.57E-02	1.00E+00	SLC35B4	9.45E-03	1.00E+00
TARS	-3.59E-01	6.80E-05	MAD2L2	1.85E-02	1.00E+00	DCAF11	6.97E-03	1.00E+00
LOC112585732	-1.15E+00	6.81E-05	MMD	-2.01E-02	1.00E+00	STRADB	-7.33E-03	1.00E+00
SPTBN2	-1.74E+00	6.88E-05	KIF1BP	-1.97E-02	1.00E+00	MRPL58	8.78E-03	1.00E+00
ADRM1	4.76E-01	6.90E-05	SLC41A3	-3.70E-02	1.00E+00	COL5A3	2.45E-02	1.00E+00
SLC25A11	4.53E-01	6.94E-05	PKIB	-4.48E-02	1.00E+00	WDR49	-1.49E-02	1.00E+00
HECTD2	8.79E-01	6.97E-05	DNAH10	-6.34E-02	1.00E+00	INTS8	6.19E-03	1.00E+00
EIF4A1	-6.14E-01	7.08E-05	RBM42	1.30E-02	1.00E+00	ARHGEF7	6.35E-03	1.00E+00
KIAA0100	5.18E-01	7.10E-05	ACTRT3	4.62E-02	1.00E+00	ADRM1	-7.67E-03	1.00E+00
GEMIN6	-6.01E-01	7.11E-05	TP53RK	-1.86E-02	1.00E+00	BICD1	9.67E-03	1.00E+00
CCDC198	3.01E+00	7.11E-05	LOC102410806	5.14E-02	1.00E+00	KLF7	-9.04E-03	1.00E+00
CD53	-3.83E+00	7.24E-05	SPOUT1	-1.70E-02	1.00E+00	RSF1	7.02E-03	1.00E+00
CES4A	1.70E+00	7.25E-05	TBC1D10A	-1.29E-02	1.00E+00	RPLP2	-1.41E-02	1.00E+00
LOC112580858	1.28E+00	7.29E-05	FILIP1	2.51E-02	1.00E+00	GLRB	-1.05E-02	1.00E+00
AK1	7.13E-01	7.29E-05	GRHPR	1.54E-02	1.00E+00	STAG2	8.32E-03	1.00E+00
ARPC1A	4.16E-01	7.37E-05	ZNF768	-1.86E-02	1.00E+00	PTCD2	8.06E-03	1.00E+00
GCHFR	-9.40E-01	7.38E-05	LOC102415483	-7.46E-02	1.00E+00	DLAT	-8.06E-03	1.00E+00
LOC102398348	-5.59E-01	7.49E-05	ZFP14	-2.22E-02	1.00E+00	LOC102408422	-1.50E-02	1.00E+00
WDR44	-7.89E-01	7.50E-05	KIAA1024L	-4.16E-02	1.00E+00	CATSPER2	1.39E-02	1.00E+00
LOC102415072	-2.21E+00	7.52E-05	LOC102408954	8.26E-02	1.00E+00	LOC102406624	-1.70E-02	1.00E+00
PLEKHM2	-3.90E-01	7.55E-05	PGK1	-2.42E-02	1.00E+00	HERC4	6.91E-03	1.00E+00
ANAPC7	3.91E-01	7.57E-05	AOC2	3.73E-02	1.00E+00	ZMYM1	-9.08E-03	1.00E+00
CHKB	-5.29E-01	7.63E-05	MICAL1	-1.82E-02	1.00E+00	DYM	6.37E-03	1.00E+00

TM2D3	4.02E-01	7.65E-05	PHOX2A	-2.81E-02	1.00E+00	PLEKHG2	8.52E-03	1.00E+00
HSPA9	-4.33E-01	7.72E-05	NKD1	5.64E-02	1.00E+00	IFT140	6.43E-03	1.00E+00
TRAM1	3.35E-01	7.72E-05	LOC112580883	-3.97E-02	1.00E+00	PWWP2A	7.75E-03	1.00E+00
NDUFA7	-5.53E-01	7.82E-05	GRAMD1A	-1.78E-02	1.00E+00	RACK1	-1.20E-02	1.00E+00
LOC112579201	1.49E+00	7.82E-05	NEDD4L	1.37E-02	1.00E+00	DDX18	7.92E-03	1.00E+00
LOC102416345	-5.81E-01	7.86E-05	ZNF331	4.51E-02	1.00E+00	CCDC85C	-6.75E-03	1.00E+00
APPBP2	-3.86E-01	7.95E-05	SLC25A10	-1.70E-02	1.00E+00	LOC102401256	2.34E-02	1.00E+00
TUBA4A	1.13E+00	7.96E-05	HOPX	6.25E-02	1.00E+00	RMDN3	-6.66E-03	1.00E+00
SLC35B1	4.28E-01	7.97E-05	LOC112581804	-2.71E-02	1.00E+00	NDUFA5	-7.77E-03	1.00E+00
LOC112586459	3.03E+00	7.98E-05	TCF19	1.76E-02	1.00E+00	ARL10	-6.51E-03	1.00E+00
ANO1	-1.31E+00	8.04E-05	MUL1	1.62E-02	1.00E+00	NR2C1	7.73E-03	1.00E+00
LOC112580647	-1.37E+00	8.07E-05	NUBP2	-3.53E-02	1.00E+00	LOC102393166	-1.02E-02	1.00E+00
TTPAL	-7.26E-01	8.13E-05	LARP4	2.11E-02	1.00E+00	MRPL4	-7.89E-03	1.00E+00
EHBP1L1	1.36E+00	8.17E-05	UBA6	-1.74E-02	1.00E+00	RPL19	-1.17E-02	1.00E+00
CCDC184	-1.83E+00	8.19E-05	HSPA13	-4.78E-02	1.00E+00	LOC112577774	1.01E-02	1.00E+00
ANKRD54	-4.77E-01	8.20E-05	SIGIRR	-4.84E-02	1.00E+00	TCEA1	6.16E-03	1.00E+00
GADD45GIP1	-7.41E-01	8.21E-05	ENTPD7	-4.24E-02	1.00E+00	ARL5B	-1.06E-02	1.00E+00
GRAMD1B	4.85E-01	8.27E-05	LGALSL	-5.76E-02	1.00E+00	KLF10	-1.50E-02	1.00E+00
NDUFAF6	-4.47E-01	8.28E-05	NOL7	2.10E-02	1.00E+00	PRPF38A	6.58E-03	1.00E+00
TM7SF3	3.98E-01	8.31E-05	LOC112587311	-6.53E-02	1.00E+00	SAMM50	7.61E-03	1.00E+00
ZC3H14	-3.48E-01	8.32E-05	LOC112581898	-5.94E-02	1.00E+00	SLC1A6	-1.14E-02	1.00E+00
HECTD1	-4.65E-01	8.36E-05	FSHR	-2.52E-02	1.00E+00	KCNN1	1.61E-02	1.00E+00
DNAJC30	4.98E-01	8.41E-05	C17H12orf65	-2.33E-02	1.00E+00	LOC102402131	1.03E-02	1.00E+00
GADL1	5.65E-01	8.48E-05	OGFOD2	-1.98E-02	1.00E+00	PSD	1.18E-02	1.00E+00
RAB7A	4.12E-01	8.51E-05	LOC102392605	-1.32E-02	1.00E+00	NRBP1	-6.54E-03	1.00E+00
CCDC130	4.86E-01	8.53E-05	PTPN2	-1.38E-02	1.00E+00	PHAX	-6.36E-03	1.00E+00
ENPEP	-1.16E+00	8.53E-05	ERGIC3	-2.02E-02	1.00E+00	LOC102404139	-1.26E-02	1.00E+00
LEMD3	-5.76E-01	8.57E-05	ANP32B	1.86E-02	1.00E+00	LOC102411883	-1.65E-02	1.00E+00
SEC61G	-6.82E-01	8.58E-05	INTS11	-1.68E-02	1.00E+00	STK19	-6.31E-03	1.00E+00
RAB9B	9.38E-01	8.58E-05	RER1	1.71E-02	1.00E+00	FOXN2	1.62E-02	1.00E+00
METTL3	-4.09E-01	8.61E-05	LOC102404231	6.42E-02	1.00E+00	C11H14orf119	7.75E-03	1.00E+00
CLSTN2	4.63E-01	8.62E-05	LOC102407213	4.19E-02	1.00E+00	NDC1	-7.45E-03	1.00E+00
ZNF777	-4.24E-01	8.64E-05	WNT5B	8.56E-02	1.00E+00	OS9	-8.31E-03	1.00E+00

ATL3	4.08E-01	8.65E-05	RPUSD4	-1.49E-02	1.00E+00	DHX38	-6.13E-03	1.00E+00
COL8A1	1.38E+00	8.67E-05	B4GALNT4	4.21E-02	1.00E+00	POLN	8.07E-03	1.00E+00
ADAMTS20	-1.87E+00	8.70E-05	GALNT1	1.80E-02	1.00E+00	MAIP1	-7.55E-03	1.00E+00
ASTE1	-6.19E-01	8.71E-05	LOC112586645	-7.43E-02	1.00E+00	VPS26C	-6.54E-03	1.00E+00
EME1	-5.74E-01	8.86E-05	RNF169	-1.53E-02	1.00E+00	PDCD6IP	-6.74E-03	1.00E+00
RNF128	1.23E+00	8.87E-05	CD72	-3.51E-02	1.00E+00	RCC1L	7.60E-03	1.00E+00
MMP9	-2.60E+00	8.90E-05	DUS4L	3.63E-02	1.00E+00	UGGT2	-1.06E-02	1.00E+00
LOC102395220	1.57E+00	8.95E-05	LOC102392085	2.96E-02	1.00E+00	NDRG3	-6.07E-03	1.00E+00
SMC4	4.47E-01	8.95E-05	ZNF197	-3.20E-02	1.00E+00	CMPK2	8.36E-03	1.00E+00
FRMD4A	6.27E-01	8.96E-05	LOC112580229	-4.35E-02	1.00E+00	TBCEL	7.90E-03	1.00E+00
ACER1	-8.04E-01	9.02E-05	LAMA5	2.28E-02	1.00E+00	LOC102397010	-8.45E-03	1.00E+00
GARNL3	-5.43E-01	9.03E-05	UVSSA	3.52E-02	1.00E+00	PDE3B	-1.76E-02	1.00E+00
ADAMTS9	-6.45E-01	9.04E-05	GRIN3B	3.38E-02	1.00E+00	KNOP1	6.66E-03	1.00E+00
LOC102412968	-2.76E+00	9.06E-05	NUP35	1.24E-02	1.00E+00	RPS24	1.39E-02	1.00E+00
PHB	-6.20E-01	9.10E-05	GSG1L	6.40E-02	1.00E+00	CTNNA1	-7.05E-03	1.00E+00
LOC102411446	-2.80E+00	9.13E-05	SLC25A35	2.21E-02	1.00E+00	NKG7	-2.74E-02	1.00E+00
LOC102408240	3.96E-01	9.14E-05	SLC3A1	-3.92E-02	1.00E+00	TMEM38B	8.07E-03	1.00E+00
CEP76	-7.22E-01	9.14E-05	PCOLCE	-3.30E-02	1.00E+00	BEX3	-8.46E-03	1.00E+00
SMKR1	-7.09E-01	9.16E-05	PGP	1.45E-02	1.00E+00	PGGHG	7.16E-03	1.00E+00
CDKN2AIP	-5.10E-01	9.16E-05	STN1	-1.49E-02	1.00E+00	DGCR2	6.83E-03	1.00E+00
ADAMTS5	-1.61E+00	9.23E-05	RAN	-1.45E-02	1.00E+00	RPL36	1.17E-02	1.00E+00
LOC112578814	1.15E+00	9.26E-05	MRPS36	-1.48E-02	1.00E+00	ZNF18	8.34E-03	1.00E+00
PXMP2	-6.92E-01	9.27E-05	SEC11C	-1.94E-02	1.00E+00	ZNF512	5.53E-03	1.00E+00
LOC112579979	1.64E+00	9.32E-05	ORAI1	-1.63E-02	1.00E+00	ZC3H15	5.79E-03	1.00E+00
LOC102395309	2.46E+00	9.66E-05	MGAT2	1.86E-02	1.00E+00	CENPQ	-8.81E-03	1.00E+00
TMEM134	6.52E-01	9.71E-05	LOC102413670	2.52E-02	1.00E+00	LOC112581570	-1.42E-02	1.00E+00
LOC112583729	2.80E+00	9.72E-05	NEK9	2.50E-02	1.00E+00	CDC26	-9.41E-03	1.00E+00
LOC102392256	1.87E+00	9.73E-05	TMEM201	1.60E-02	1.00E+00	NIPA2	-5.59E-03	1.00E+00
SDAD1	-3.96E-01	9.74E-05	PTPRT	1.34E-02	1.00E+00	RPL10A	1.13E-02	1.00E+00
LOC102393150	8.21E-01	9.89E-05	PUM1	-2.62E-02	1.00E+00	BABAM1	-6.69E-03	1.00E+00
ANK3	6.14E-01	9.95E-05	CLDN6	7.65E-02	1.00E+00	LOC102409438	-3.41E-02	1.00E+00
BFAR	-3.43E-01	9.95E-05	PSRC1	1.61E-02	1.00E+00	ENHO	1.66E-02	1.00E+00
VPS45	3.81E-01	9.99E-05	IFT172	3.58E-02	1.00E+00	SLC25A41	1.40E-02	1.00E+00

LOC102403617	-6.28E-01	1.00E-04	FADD	1.75E-02	1.00E+00	GRIK5	5.72E-03	1.00E+00
HAUS1	3.99E-01	1.02E-04	CHKB	1.34E-02	1.00E+00	ZNF330	-7.43E-03	1.00E+00
LOC102411053	-1.19E+00	1.03E-04	CCNQ	-1.44E-02	1.00E+00	ZNF385A	-1.05E-02	1.00E+00
RIPOR3	-1.19E+00	1.03E-04	DCUN1D1	1.96E-02	1.00E+00	LOC102393946	8.37E-03	1.00E+00
ZNF207	-3.75E-01	1.04E-04	SSR4	-1.98E-02	1.00E+00	ASRGL1	-6.58E-03	1.00E+00
PSMB7	-5.99E-01	1.04E-04	GPATCH1	1.49E-02	1.00E+00	CLPX	6.87E-03	1.00E+00
TMEM233	2.86E+00	1.05E-04	PRDX2	1.54E-02	1.00E+00	LOC102416083	-7.70E-03	1.00E+00
PRR5	-5.91E-01	1.06E-04	LASP1	1.23E-02	1.00E+00	USP19	5.60E-03	1.00E+00
HERPUD2	-4.90E-01	1.07E-04	LOC112584672	4.41E-02	1.00E+00	CD37	-8.03E-03	1.00E+00
LOC102410470	-8.74E-01	1.08E-04	CDS1	-1.86E-02	1.00E+00	EHBP1	8.81E-03	1.00E+00
LOC112582964	1.50E+00	1.08E-04	LOC102392446	2.41E-02	1.00E+00	RASSF4	8.08E-03	1.00E+00
EXD2	-7.45E-01	1.08E-04	USF2	1.67E-02	1.00E+00	PMP22	-2.14E-02	1.00E+00
DDX47	3.78E-01	1.08E-04	APLN	-6.95E-02	1.00E+00	PNLDC1	1.30E-02	1.00E+00
NRF1	-4.31E-01	1.08E-04	ATF4	1.33E-02	1.00E+00	LAMTOR2	-1.03E-02	1.00E+00
TBC1D5	-3.62E-01	1.09E-04	PPP1R13L	1.52E-02	1.00E+00	USP8	6.45E-03	1.00E+00
SLC7A11	-6.13E-01	1.10E-04	ACO1	-2.48E-02	1.00E+00	ST3GAL6	-6.57E-03	1.00E+00
CCDC40	7.30E-01	1.11E-04	LOC112586257	-6.45E-02	1.00E+00	TMEM70	-7.05E-03	1.00E+00
USP33	4.67E-01	1.11E-04	LOC112583751	5.24E-02	1.00E+00	PDZD11	-7.49E-03	1.00E+00
LOC112587462	-1.25E+00	1.11E-04	TRIM66	2.81E-02	1.00E+00	TTLL4	5.67E-03	1.00E+00
RHBDF2	6.73E-01	1.11E-04	MRPL54	-1.85E-02	1.00E+00	CAMSAP2	-7.71E-03	1.00E+00
FABP4	-3.19E+00	1.11E-04	DROSHA	1.51E-02	1.00E+00	LOC102411708	9.06E-03	1.00E+00
CAV2	-1.22E+00	1.11E-04	CRK	1.46E-02	1.00E+00	APLP1	-9.19E-03	1.00E+00
NMU	2.08E+00	1.12E-04	DOCK10	3.96E-02	1.00E+00	USP4	-5.07E-03	1.00E+00
LOC102398732	-1.80E+00	1.13E-04	CHST11	-1.33E-02	1.00E+00	FLYWCH1	-6.29E-03	1.00E+00
LOC112584308	-1.77E+00	1.14E-04	ASCL3	-5.71E-02	1.00E+00	DNAJC30	-7.24E-03	1.00E+00
ZFR	-4.26E-01	1.14E-04	NYAP1	-6.65E-02	1.00E+00	RAB20	-1.74E-02	1.00E+00
C12H9orf78	-4.63E-01	1.15E-04	TMEM64	3.23E-02	1.00E+00	LOC102394398	8.53E-03	1.00E+00
LOC112586489	2.78E+00	1.15E-04	ZNF696	2.84E-02	1.00E+00	APOE	9.65E-03	1.00E+00
ARHGAP5	-5.63E-01	1.15E-04	HSPBP1	1.17E-02	1.00E+00	C21H3orf49	-9.25E-03	1.00E+00
RAD21	4.29E-01	1.15E-04	GAL3ST3	-5.01E-02	1.00E+00	TAF9B	9.92E-03	1.00E+00
LOC112587372	1.91E+00	1.16E-04	SINHCAF	-2.58E-02	1.00E+00	RPL35	-1.16E-02	1.00E+00
NFIX	-8.24E-01	1.16E-04	SPC24	-2.06E-02	1.00E+00	ATP6V0A4	1.31E-02	1.00E+00
NSMAF	3.52E-01	1.16E-04	HARS	-2.12E-02	1.00E+00	ARFGAP2	5.87E-03	1.00E+00

RUNX3	1.36E+00	1.16E-04	BRMS1	2.01E-02	1.00E+00	PEF1	-5.74E-03	1.00E+00
GPHN	-4.58E-01	1.18E-04	GGT5	1.72E-02	1.00E+00	LOC102397874	1.44E-02	1.00E+00
STX18	-5.04E-01	1.19E-04	ADAM9	3.46E-02	1.00E+00	ZNF35	-6.41E-03	1.00E+00
SBF1	-3.72E-01	1.20E-04	LOC112583634	4.03E-02	1.00E+00	PYROXD1	-7.10E-03	1.00E+00
D2HGDH	-4.67E-01	1.22E-04	CCDC15	-1.46E-02	1.00E+00	MYLK3	1.01E-02	1.00E+00
LOC102398588	1.07E+00	1.22E-04	GLDC	-1.95E-02	1.00E+00	SYS1	-5.82E-03	1.00E+00
KIF15	5.71E-01	1.25E-04	IRAK2	1.46E-02	1.00E+00	DICER1	6.65E-03	1.00E+00
NOTCH3	-1.18E+00	1.28E-04	LOC102415369	-4.36E-02	1.00E+00	PIP5K1A	6.68E-03	1.00E+00
SLC25A21	-1.17E+00	1.28E-04	LOC112587058	9.23E-02	1.00E+00	FAM76A	6.14E-03	1.00E+00
PNPLA4	-6.29E-01	1.28E-04	ROPN1	4.64E-02	1.00E+00	CAAP1	-6.21E-03	1.00E+00
LOC102395897	-5.26E-01	1.28E-04	LOC112580778	-4.48E-02	1.00E+00	RNASE6	-2.35E-02	1.00E+00
PTCD2	-4.90E-01	1.28E-04	CUL2	-1.80E-02	1.00E+00	PROCA1	-8.96E-03	1.00E+00
C23H10orf143	-5.82E-01	1.29E-04	TBC1D12	4.31E-02	1.00E+00	ZBTB5	6.31E-03	1.00E+00
RAB4B	-4.58E-01	1.31E-04	MXRA7	-2.84E-02	1.00E+00	LRR1	-1.10E-02	1.00E+00
TM6SF2	1.36E+00	1.31E-04	LOC102392841	1.64E-02	1.00E+00	PMVK	6.16E-03	1.00E+00
HPS5	4.30E-01	1.31E-04	ACTR1B	1.56E-02	1.00E+00	KIF24	-7.14E-03	1.00E+00
CBX6	6.30E-01	1.32E-04	SEC31B	1.53E-02	1.00E+00	NFKB1	-8.14E-03	1.00E+00
PPP1R35	5.73E-01	1.32E-04	ECPAS	-1.51E-02	1.00E+00	LOC102416201	-7.88E-03	1.00E+00
LY6E	-9.96E-01	1.32E-04	S100A16	1.57E-02	1.00E+00	DHRS3	8.06E-03	1.00E+00
ZNF335	-4.85E-01	1.33E-04	GNE	2.86E-02	1.00E+00	LOC112578238	7.48E-03	1.00E+00
ZNF618	3.66E-01	1.33E-04	TLK1	1.78E-02	1.00E+00	LOC112585629	-8.33E-03	1.00E+00
CFAP100	1.52E+00	1.35E-04	PDCD7	1.67E-02	1.00E+00	PTK2	5.18E-03	1.00E+00
LOC102396111	-1.08E+00	1.35E-04	ELAVL2	1.34E-02	1.00E+00	LOC112581576	1.21E-02	1.00E+00
WBP2NL	-8.25E-01	1.35E-04	GTPBP10	1.79E-02	1.00E+00	NTN3	-1.36E-02	1.00E+00
MFN1	-3.60E-01	1.35E-04	MYPN	4.02E-02	1.00E+00	EEF1AKMT3	9.77E-03	1.00E+00
HAP1	1.32E+00	1.36E-04	C2H6orf52	2.10E-02	1.00E+00	NR0B1	1.12E-02	1.00E+00
ARHGAP27	1.35E+00	1.37E-04	ATF7	-2.15E-02	1.00E+00	PUM3	-5.59E-03	1.00E+00
MTRR	-5.43E-01	1.37E-04	SAPCD2	-3.46E-02	1.00E+00	NDUFA11	6.64E-03	1.00E+00
VPS37B	-3.73E-01	1.38E-04	FOXG1	3.48E-02	1.00E+00	RWDD1	8.18E-03	1.00E+00
EVI5L	3.55E-01	1.40E-04	LOC102403501	2.28E-02	1.00E+00	CCNJ	1.52E-02	1.00E+00
LOC112585132	-2.89E+00	1.41E-04	WDR93	1.59E-02	1.00E+00	ISOC1	5.41E-03	1.00E+00
SPHK1	-7.72E-01	1.41E-04	LOC102397684	4.42E-02	1.00E+00	CYHR1	6.20E-03	1.00E+00
ALKBH7	-6.47E-01	1.42E-04	TRAF2	-1.16E-02	1.00E+00	POLR2F	7.31E-03	1.00E+00
CDK15	1.62E+00	1.42E-04	ACOT8	-1.74E-02	1.00E+00	CENPP	7.67E-03	1.00E+00

ALDH1A2	1.24E+00	1.44E-04	KEAP1	-2.09E-02	1.00E+00	SWSAP1	-1.10E-02	1.00E+00
LOC102404027	1.13E+00	1.44E-04	LOC112585578	2.42E-02	1.00E+00	SOCS6	6.20E-03	1.00E+00
POLQ	-5.64E-01	1.44E-04	CLN3	2.90E-02	1.00E+00	IPO8	5.20E-03	1.00E+00
CYTH3	-7.03E-01	1.44E-04	TRIM37	-1.24E-02	1.00E+00	DDX49	-6.32E-03	1.00E+00
LCTL	-1.33E+00	1.45E-04	FBXW11	1.32E-02	1.00E+00	TMEM140	7.61E-03	1.00E+00
ZNF407	4.09E-01	1.45E-04	CLCC1	-2.22E-02	1.00E+00	GART	5.67E-03	1.00E+00
LOC102410458	-1.23E+00	1.45E-04	CCHCR1	1.76E-02	1.00E+00	ST14	-1.17E-02	1.00E+00
GRAP2	1.19E+00	1.46E-04	PRR12	1.92E-02	1.00E+00	NSMCE2	5.68E-03	1.00E+00
FRS3	-8.06E-01	1.47E-04	GPHN	1.94E-02	1.00E+00	EAPP	5.94E-03	1.00E+00
SUFU	4.67E-01	1.48E-04	SH3PXD2A	-2.01E-02	1.00E+00	STRN	-6.76E-03	1.00E+00
LOC112581830	2.69E+00	1.48E-04	PPP6R2	1.90E-02	1.00E+00	PQBP1	7.32E-03	1.00E+00
ARHGEF15	1.30E+00	1.48E-04	EVI5	-2.73E-02	1.00E+00	TRMT44	-5.87E-03	1.00E+00
RAB5C	3.26E-01	1.48E-04	ZMYM6	-1.82E-02	1.00E+00	PSMD13	6.42E-03	1.00E+00
MORC3	-6.07E-01	1.48E-04	SLC25A37	-5.68E-02	1.00E+00	BCAP29	-5.54E-03	1.00E+00
GAP43	2.79E+00	1.49E-04	PELI2	-1.25E-02	1.00E+00	MYO1F	2.41E-02	1.00E+00
RPUSD2	5.42E-01	1.49E-04	PRPS2	1.51E-02	1.00E+00	SNAP47	-5.47E-03	1.00E+00
SCYL1	-4.15E-01	1.49E-04	ATP1B3	-1.25E-02	1.00E+00	MINDY1	4.82E-03	1.00E+00
MAP3K12	-4.52E-01	1.50E-04	NQO1	-1.78E-02	1.00E+00	PDXK	5.12E-03	1.00E+00
MDN1	6.25E-01	1.51E-04	YKT6	-1.24E-02	1.00E+00	TMEM129	5.85E-03	1.00E+00
LOC112586804	1.51E+00	1.51E-04	RSPH3	2.84E-02	1.00E+00	TMEM63A	-5.37E-03	1.00E+00
GLUL	-4.74E-01	1.52E-04	JTB	1.44E-02	1.00E+00	HTRA2	5.89E-03	1.00E+00
DMTF1	3.46E-01	1.53E-04	AP5M1	-1.66E-02	1.00E+00	LOC102396267	-1.03E-02	1.00E+00
LOC102389062	6.96E-01	1.54E-04	DIRAS2	-1.63E-02	1.00E+00	TBC1D7	-5.93E-03	1.00E+00
LOC102408709	-4.31E-01	1.54E-04	ZPBP2	7.78E-02	1.00E+00	VPS4A	-5.16E-03	1.00E+00
TMEM219	5.69E-01	1.54E-04	LINGO1	-2.90E-02	1.00E+00	UMAD1	-1.15E-02	1.00E+00
LOC112582937	1.22E+00	1.55E-04	TRAPPC5	-2.28E-02	1.00E+00	LOXL3	5.17E-03	1.00E+00
HAUS3	-4.98E-01	1.57E-04	SLC25A28	2.59E-02	1.00E+00	NME6	8.07E-03	1.00E+00
PTRH2	5.64E-01	1.58E-04	COMP	-5.89E-02	1.00E+00	FASTKD1	-5.63E-03	1.00E+00
C4H12orf50	-1.32E+00	1.59E-04	ALKBH6	-2.49E-02	1.00E+00	LOC102407663	9.74E-03	1.00E+00
MASP2	-8.00E-01	1.60E-04	NDN	-8.21E-02	1.00E+00	ARHGEF6	5.63E-03	1.00E+00
MAPK8IP1	-5.45E-01	1.61E-04	C3H9orf40	1.72E-02	1.00E+00	ARMT1	-5.81E-03	1.00E+00
ZBTB8A	-8.67E-01	1.61E-04	MYLK3	3.97E-02	1.00E+00	DYRK1A	4.96E-03	1.00E+00
NELFCD	-4.14E-01	1.62E-04	NDUFB7	2.25E-02	1.00E+00	LOC102409319	-7.63E-03	1.00E+00

ALAD	5.71E-01	1.62E-04	RCL1	1.63E-02	1.00E+00	SELENOS	-5.65E-03	1.00E+00
REL	1.01E+00	1.62E-04	LOC102409914	2.21E-02	1.00E+00	RBM18	5.98E-03	1.00E+00
LOC112587363	1.45E+00	1.63E-04	GSTK1	5.75E-02	1.00E+00	CSDE1	-5.75E-03	1.00E+00
C16H11orf71	5.99E-01	1.66E-04	CCDC138	-2.61E-02	1.00E+00	AATF	-5.98E-03	1.00E+00
FBXO24	-1.10E+00	1.68E-04	ZNF579	1.96E-02	1.00E+00	ZNF507	-6.64E-03	1.00E+00
ZNF512B	-5.27E-01	1.68E-04	TMEM97	-3.01E-02	1.00E+00	LOC112581734	1.16E-02	1.00E+00
MCM9	3.92E-01	1.68E-04	FAM133B	2.86E-02	1.00E+00	WBP1	7.11E-03	1.00E+00
LOC112578871	1.45E+00	1.68E-04	DNAJA3	-1.15E-02	1.00E+00	TNFRSF19	1.23E-02	1.00E+00
LOC102403526	-2.10E+00	1.69E-04	ADSL	-1.48E-02	1.00E+00	ZFP3	7.68E-03	1.00E+00
LOC112581884	-3.63E+00	1.69E-04	IGFBP7	3.44E-02	1.00E+00	KDM5A	-5.63E-03	1.00E+00
CNKSR2	-6.83E-01	1.70E-04	COQ8B	1.59E-02	1.00E+00	NARS	-4.59E-03	1.00E+00
ARL6IP1	-4.29E-01	1.70E-04	TVP23A	-2.05E-02	1.00E+00	SLC35F6	4.56E-03	1.00E+00
LOC112586818	8.55E-01	1.71E-04	ZC3H15	-1.15E-02	1.00E+00	ACTG1	9.69E-03	1.00E+00
LMLN	-5.49E-01	1.72E-04	ATP10A	-3.29E-02	1.00E+00	RPL37A	-9.68E-03	1.00E+00
SNX32	1.32E+00	1.75E-04	NCAPG	-1.30E-02	1.00E+00	GLA	1.05E-02	1.00E+00
OTUD7B	3.59E-01	1.78E-04	AKAIN1	-3.27E-02	1.00E+00	CCDC126	7.05E-03	1.00E+00
LOC112583615	1.36E+00	1.81E-04	DDX39B	-1.29E-02	1.00E+00	LOC102406631	-8.12E-03	1.00E+00
MCRIP1	-5.08E-01	1.81E-04	STX6	1.16E-02	1.00E+00	C17H22orf39	6.31E-03	1.00E+00
MMP28	1.46E+00	1.82E-04	UBE2O	-1.78E-02	1.00E+00	SAMD11	-5.09E-03	1.00E+00
ND4L	-7.98E-01	1.82E-04	DCAF6	-1.37E-02	1.00E+00	MRPL54	6.54E-03	1.00E+00
ANKMY1	-1.12E+00	1.83E-04	PRR29	5.81E-02	1.00E+00	EML3	4.93E-03	1.00E+00
RBM28	4.14E-01	1.85E-04	COL4A1	-4.65E-02	1.00E+00	RPS5	-9.41E-03	1.00E+00
CNTF	1.69E+00	1.85E-04	LOC102416083	-1.78E-02	1.00E+00	MPP5	4.91E-03	1.00E+00
COQ7	5.63E-01	1.85E-04	TAF7	-1.25E-02	1.00E+00	RALBP1	-4.26E-03	1.00E+00
PNMA8A	-9.79E-01	1.86E-04	TTC8	-4.49E-02	1.00E+00	TMEM216	7.06E-03	1.00E+00
LOC112587028	-2.00E+00	1.86E-04	ST6GALNAC1	-2.84E-02	1.00E+00	DRG1	-5.18E-03	1.00E+00
ATP9B	-4.36E-01	1.88E-04	SNAPC1	-3.28E-02	1.00E+00	ATP5PF	5.70E-03	1.00E+00
UEVLD	-5.88E-01	1.89E-04	TOB2	1.83E-02	1.00E+00	ATRN	-5.08E-03	1.00E+00
LOC112583705	1.38E+00	1.89E-04	PRUNE2	1.87E-02	1.00E+00	SSPN	-6.66E-03	1.00E+00
LOC102397569	1.92E+00	1.89E-04	E4F1	3.86E-02	1.00E+00	LOC102392085	5.41E-03	1.00E+00
NUBPL	5.07E-01	1.89E-04	SERTM1	7.34E-02	1.00E+00	CCDC80	-8.34E-03	1.00E+00
SYCE1	1.15E+00	1.90E-04	TNFAIP1	2.31E-02	1.00E+00	NIT2	-5.08E-03	1.00E+00
TOMM20L	6.06E-01	1.91E-04	ELAVL1	1.11E-02	1.00E+00	ATMIN	-4.70E-03	1.00E+00

MTOR	3.55E-01	1.92E-04	MRPL1	-1.15E-02	1.00E+00	RGS3	-5.24E-03	1.00E+00
PBX1	4.15E-01	1.92E-04	HS2ST1	-3.28E-02	1.00E+00	LDHB	-7.49E-03	1.00E+00
LGR5	1.58E+00	1.93E-04	FNIP1	2.51E-02	1.00E+00	TRAPPC12	4.27E-03	1.00E+00
ASB4	1.57E+00	1.93E-04	LOC112580880	2.80E-02	1.00E+00	RGS19	-1.21E-02	1.00E+00
LOC102416484	2.58E+00	1.96E-04	GINM1	1.32E-02	1.00E+00	FLT3LG	8.80E-03	1.00E+00
LOC102390493	8.96E-01	1.97E-04	LOC102416218	-1.52E-02	1.00E+00	MAPKBP1	-5.03E-03	1.00E+00
LYST	5.86E-01	1.99E-04	CCDC102A	7.63E-02	1.00E+00	LRFN5	8.13E-03	1.00E+00
STK32C	-1.42E+00	2.00E-04	GAA	1.78E-02	1.00E+00	UHRF1BP1	4.95E-03	1.00E+00
LOC112587105	-2.10E+00	2.01E-04	DDX1	-1.58E-02	1.00E+00	BOLA1	-5.90E-03	1.00E+00
CDA	9.08E-01	2.01E-04	MAGED1	-1.16E-01	1.00E+00	XKR8	-8.42E-03	1.00E+00
DZIP1L	6.26E-01	2.01E-04	PARD6B	2.31E-02	1.00E+00	ZER1	-4.35E-03	1.00E+00
IGFLR1	6.01E-01	2.04E-04	HSPA5	1.46E-02	1.00E+00	RAC1	4.54E-03	1.00E+00
INTS2	-5.73E-01	2.04E-04	NDUFS7	-1.80E-02	1.00E+00	CD47	-5.05E-03	1.00E+00
CXHXorf38	-4.60E-01	2.05E-04	CLPS	-7.54E-02	1.00E+00	PSTK	-6.77E-03	1.00E+00
LOC102413428	1.20E+00	2.05E-04	TEX38	-6.28E-02	1.00E+00	SLC12A9	-5.03E-03	1.00E+00
LOC112587017	1.34E+00	2.06E-04	SNAP29	1.18E-02	1.00E+00	NKIRAS1	7.29E-03	1.00E+00
ZFAND5	-6.87E-01	2.07E-04	LOC102408337	5.97E-02	1.00E+00	RIC8B	4.54E-03	1.00E+00
SYNPO2	-1.28E+00	2.07E-04	FAM98B	-2.33E-02	1.00E+00	ILKAP	-4.75E-03	1.00E+00
TPMT	-6.90E-01	2.09E-04	SNRPA	-1.38E-02	1.00E+00	KLHL2	5.58E-03	1.00E+00
PDE6A	1.29E+00	2.10E-04	PDE4A	-3.39E-02	1.00E+00	TAB2	4.85E-03	1.00E+00
CNRIP1	4.26E-01	2.10E-04	CNOT9	1.67E-02	1.00E+00	UBR1	7.61E-03	1.00E+00
LOC102402202	-1.28E+00	2.10E-04	KLF16	1.36E-02	1.00E+00	AOC2	9.68E-03	1.00E+00
CRYAB	-1.03E+00	2.13E-04	LOC112585597	2.55E-02	1.00E+00	ZNF177	-5.45E-03	1.00E+00
CINP	-5.24E-01	2.13E-04	UCHL1	-1.43E-02	1.00E+00	TXLNA	4.17E-03	1.00E+00
SPATA6L	-1.11E+00	2.14E-04	JPT2	1.27E-02	1.00E+00	AIF1	1.46E-02	1.00E+00
YPEL2	4.72E-01	2.14E-04	MYOM3	7.02E-02	1.00E+00	SF3A3	-4.12E-03	1.00E+00
LOC112582074	1.17E+00	2.17E-04	LARP6	-5.46E-02	1.00E+00	MGA	-4.86E-03	1.00E+00
LOC112583870	1.52E+00	2.17E-04	HSBP1L1	-4.29E-02	1.00E+00	ARL16	-4.28E-03	1.00E+00
DDX52	-4.45E-01	2.18E-04	LOC112579935	-4.30E-02	1.00E+00	COPS4	4.15E-03	1.00E+00
LOC102406900	2.08E+00	2.19E-04	C5	2.92E-02	1.00E+00	LOC102403587	-9.84E-03	1.00E+00
LOC102396296	-7.45E-01	2.19E-04	COASY	1.56E-02	1.00E+00	RPS9	-8.17E-03	1.00E+00
POC1B	-6.47E-01	2.19E-04	LOC102412565	6.58E-02	1.00E+00	LOC112581212	-8.47E-03	1.00E+00
LMTK2	-7.37E-01	2.19E-04	CFAP46	-4.61E-02	1.00E+00	RRP9	-5.31E-03	1.00E+00
LOC102400687	2.22E+00	2.20E-04	DRD3	6.41E-02	1.00E+00	TBC1D2B	5.90E-03	1.00E+00
XRCC1	-4.97E-	2.22E-04	TPBGL	1.29E-02	1.00E+00	STRAP	3.94E-03	1.00E+00

	01							
SLC2A4	-9.16E-01	2.22E-04	ANKZF1	1.88E-02	1.00E+00	MEF2C	4.83E-03	1.00E+00
C3H17orf97	1.05E+00	2.23E-04	TTLL6	2.22E-02	1.00E+00	PROSER3	7.47E-03	1.00E+00
TPRKB	-6.31E-01	2.23E-04	RAB2A	1.02E-02	1.00E+00	TMEM33	-6.30E-03	1.00E+00
AP3B1	3.28E-01	2.24E-04	LOC112578849	5.06E-02	1.00E+00	LOC102398979	6.40E-03	1.00E+00
SLC6A14	-1.23E+00	2.24E-04	FANCE	-4.06E-02	1.00E+00	PRPF31	-4.59E-03	1.00E+00
TTC26	-5.26E-01	2.25E-04	AMN1	-1.60E-02	1.00E+00	WDR74	4.97E-03	1.00E+00
PAK2	-4.11E-01	2.28E-04	SYT5	3.20E-02	1.00E+00	SLC25A44	4.25E-03	1.00E+00
PITPNA	3.28E-01	2.29E-04	CCND2	8.53E-02	1.00E+00	ATP6V1E1	-4.44E-03	1.00E+00
NRN1	1.97E+00	2.33E-04	LOC102411450	-3.21E-02	1.00E+00	RGP1	-3.84E-03	1.00E+00
AIFM3	1.36E+00	2.33E-04	LOC112585687	3.99E-02	1.00E+00	RCC2	-4.03E-03	1.00E+00
HES1	7.48E-01	2.34E-04	ERMARD	-2.07E-02	1.00E+00	RPS23	7.46E-03	1.00E+00
SHARPIN	5.29E-01	2.34E-04	PUM3	-1.25E-02	1.00E+00	DZIP3	5.13E-03	1.00E+00
CCL24	-3.46E+00	2.36E-04	LOC102397602	4.58E-02	1.00E+00	LANCL1	-3.83E-03	1.00E+00
CC2D1B	-4.16E-01	2.37E-04	LOC112580204	4.48E-02	1.00E+00	JAK2	5.09E-03	1.00E+00
ZDHHC24	4.41E-01	2.39E-04	LOC112579908	-4.52E-02	1.00E+00	PIK3R5	-6.91E-03	1.00E+00
GOT2	-3.78E-01	2.41E-04	APBB1	1.45E-02	1.00E+00	VPS11	-3.73E-03	1.00E+00
SH3GLB2	-4.40E-01	2.41E-04	LOC102416571	-1.10E-01	1.00E+00	KIF6	6.56E-03	1.00E+00
PWWP2A	4.07E-01	2.41E-04	ALS2	1.63E-02	1.00E+00	CCNH	-4.21E-03	1.00E+00
TACR2	1.21E+00	2.42E-04	PRKAG1	-1.19E-02	1.00E+00	STX17	3.73E-03	1.00E+00
SHPRH	-7.64E-01	2.42E-04	LOC102405836	1.63E-02	1.00E+00	C3H9orf40	-4.68E-03	1.00E+00
ZSCAN30	-1.14E+00	2.43E-04	TADA2A	-1.10E-02	1.00E+00	PNP	-6.18E-03	1.00E+00
KCNIP2	-1.61E+00	2.45E-04	IL17RD	-2.85E-02	1.00E+00	ELF4	-8.58E-03	1.00E+00
DNAAF5	-4.39E-01	2.46E-04	USP21	-1.85E-02	1.00E+00	RABL6	4.19E-03	1.00E+00
LOC102398407	1.49E+00	2.48E-04	ERCC8	2.79E-02	1.00E+00	LOC102399551	-4.86E-03	1.00E+00
LOC102393855	-5.59E-01	2.48E-04	LOC102397569	8.81E-02	1.00E+00	RAB4B	-4.12E-03	1.00E+00
EXTL1	1.01E+00	2.49E-04	FAM114A2	1.23E-02	1.00E+00	ITGB2	1.79E-02	1.00E+00
VSIG10L	-8.86E-01	2.49E-04	LOC102391549	3.14E-02	1.00E+00	PSME1	5.15E-03	1.00E+00
FAM84A	1.26E+00	2.50E-04	HS6ST1	2.89E-02	1.00E+00	C5H11orf54	-6.31E-03	1.00E+00
LOC112581276	2.19E+00	2.50E-04	FAM83H	-4.08E-02	1.00E+00	TCTEX1D2	4.95E-03	1.00E+00
UXT	7.17E-01	2.53E-04	ABHD6	-1.55E-02	1.00E+00	FAM92A	-4.13E-03	1.00E+00
PTCRA	1.33E+00	2.54E-04	HYDIN	-2.14E-02	1.00E+00	ZSWIM8	4.00E-03	1.00E+00
LOC102409612	-1.01E+00	2.54E-04	PLOD3	2.24E-02	1.00E+00	LAMP2	-4.08E-03	1.00E+00
SCAF8	3.60E-01	2.54E-04	LOC102413176	-2.92E-	1.00E+00	HSDL1	3.90E-03	1.00E+00

				02				
PPP2R2A	3.58E-01	2.55E-04	HSD17B4	1.59E-02	1.00E+00	TIMM9	4.20E-03	1.00E+00
LOC112581738	-8.92E-01	2.57E-04	TMPRSS5	-5.35E-02	1.00E+00	SCN8A	6.34E-03	1.00E+00
CCNA1	8.65E-01	2.58E-04	ZNF287	3.88E-02	1.00E+00	KIAA0100	-4.78E-03	1.00E+00
LOC112579218	-1.42E+00	2.58E-04	C6H1orf52	2.15E-02	1.00E+00	LMAN2L	3.99E-03	1.00E+00
PPIL6	-1.10E+00	2.59E-04	LOC102393336	3.79E-02	1.00E+00	DENR	3.64E-03	1.00E+00
ERAL1	-4.80E-01	2.63E-04	PHETA2	-1.56E-02	1.00E+00	VAMP3	3.69E-03	1.00E+00
STX6	3.57E-01	2.64E-04	FERMT1	-1.66E-02	1.00E+00	ZNF582	-7.79E-03	1.00E+00
TMEM238	-7.23E-01	2.67E-04	OSBPL11	-2.53E-02	1.00E+00	MIDN	-4.45E-03	1.00E+00
LOC112582773	1.93E+00	2.70E-04	SLC50A1	3.33E-02	1.00E+00	IFFO2	-8.85E-03	1.00E+00
AKAP13	-6.10E-01	2.71E-04	ZFP69	1.41E-02	1.00E+00	ENY2	3.70E-03	1.00E+00
MAT1A	1.17E+00	2.72E-04	PBK	-1.12E-02	1.00E+00	FAM169B	8.48E-03	1.00E+00
LOC112578895	-1.29E+00	2.73E-04	DISP3	-1.33E-02	1.00E+00	DNHD1	7.28E-03	1.00E+00
ING5	4.23E-01	2.78E-04	LOC102413407	1.47E-02	1.00E+00	CHD9	-5.04E-03	1.00E+00
ND5	7.66E-01	2.79E-04	QTRT1	-1.92E-02	1.00E+00	UBE2L6	-5.63E-03	1.00E+00
PRPF31	-4.82E-01	2.80E-04	MRNIP	-1.54E-02	1.00E+00	MRPS31	3.71E-03	1.00E+00
CTNND2	1.98E+00	2.84E-04	CCNT2	-2.00E-02	1.00E+00	RPL13	-6.98E-03	1.00E+00
MTFMT	-4.80E-01	2.84E-04	TPH1	4.82E-02	1.00E+00	MPZL1	-3.41E-03	1.00E+00
TRAPPC9	3.95E-01	2.85E-04	LOC102412798	3.57E-02	1.00E+00	MCRIP2	-5.71E-03	1.00E+00
MAGOH	4.56E-01	2.85E-04	CCDC69	6.52E-02	1.00E+00	MAP3K11	-3.58E-03	1.00E+00
LOC102393117	-5.30E-01	2.85E-04	NCAN	1.94E-02	1.00E+00	SLC4A7	-6.06E-03	1.00E+00
CD48	-3.61E+00	2.86E-04	PLA2G4F	-2.17E-02	1.00E+00	SLC10A3	3.70E-03	1.00E+00
WDR81	-4.96E-01	2.86E-04	RHOA	1.28E-02	1.00E+00	PDK1	3.94E-03	1.00E+00
NIPAL2	4.63E-01	2.86E-04	SMYD2	-1.23E-02	1.00E+00	SAAL1	-4.24E-03	1.00E+00
SMAP1	-3.51E-01	2.87E-04	GLRA1	2.34E-02	1.00E+00	EFCAB7	-4.97E-03	1.00E+00
RPGR	4.74E-01	2.88E-04	RCC2	1.14E-02	1.00E+00	DPH5	-3.81E-03	1.00E+00
LNX2	4.35E-01	2.89E-04	JAK1	1.13E-02	1.00E+00	ZNF112	-7.01E-03	1.00E+00
TPRG1	2.05E+00	2.92E-04	TSHZ1	-3.62E-02	1.00E+00	ATP5IF1	4.99E-03	1.00E+00
LOC112581570	7.37E-01	2.93E-04	RIOX2	-1.06E-02	1.00E+00	LOC112587954	-6.57E-03	1.00E+00
ABCE1	-4.17E-01	2.94E-04	RNF215	-2.72E-02	1.00E+00	ABI2	-3.44E-03	1.00E+00
LOC102402539	-3.28E+00	2.94E-04	IK	1.09E-02	1.00E+00	ZNF165	6.85E-03	1.00E+00
CFAP70	8.70E-01	2.96E-04	HPS3	1.65E-02	1.00E+00	LOC102411666	-5.59E-03	1.00E+00
CNP	4.13E-01	2.97E-04	PRR11	2.73E-02	1.00E+00	LOC102414273	4.51E-03	1.00E+00

NADK	-3.62E-01	3.00E-04	LOC102414453	-3.45E-02	1.00E+00	C17H4orf33	6.68E-03	1.00E+00
CMC2	6.03E-01	3.01E-04	BATF2	-5.48E-02	1.00E+00	CCZ1	-3.25E-03	1.00E+00
LOC112578889	-1.01E+00	3.05E-04	PARD6A	-2.00E-02	1.00E+00	IFIT2	-8.33E-03	1.00E+00
ERI1	3.57E-01	3.05E-04	PRPF18	1.75E-02	1.00E+00	PLPPR2	6.27E-03	1.00E+00
ACBD3	-3.82E-01	3.05E-04	LOC112579189	7.83E-02	1.00E+00	NPRL2	4.24E-03	1.00E+00
LOC102397660	3.19E-01	3.06E-04	LOC102414190	1.33E-02	1.00E+00	RSRP1	-3.03E-03	1.00E+00
TRAK1	-4.26E-01	3.07E-04	TRIM35	-2.49E-02	1.00E+00	CDKN2C	4.56E-03	1.00E+00
IAH1	-5.20E-01	3.08E-04	FAM117A	-1.06E-02	1.00E+00	CWC15	-3.47E-03	1.00E+00
MAPKAPK5	1.04E+00	3.09E-04	FUT10	-8.93E-02	1.00E+00	TRANK1	-3.71E-03	1.00E+00
DHODH	-5.65E-01	3.09E-04	FOSL1	3.32E-02	1.00E+00	RSL1D1	3.35E-03	1.00E+00
SHKBP1	-4.21E-01	3.10E-04	FICD	-2.01E-02	1.00E+00	DNMT3L	1.09E-02	1.00E+00
NFATC2IP	-5.55E-01	3.11E-04	LOC102400824	2.43E-02	1.00E+00	LOC102397660	-3.18E-03	1.00E+00
MED8	-4.83E-01	3.16E-04	LOC102395807	1.93E-02	1.00E+00	PKM	5.15E-03	1.00E+00
ALG9	-4.17E-01	3.17E-04	GATM	-8.57E-02	1.00E+00	RNASEH1	3.41E-03	1.00E+00
MTFR1L	3.17E-01	3.19E-04	TGFBRAP1	1.92E-02	1.00E+00	APOO	3.86E-03	1.00E+00
FAM163B	2.08E+00	3.20E-04	CIAO1	2.60E-02	1.00E+00	RPLP0	-6.67E-03	1.00E+00
PRR22	-1.13E+00	3.20E-04	NEFL	-3.28E-02	1.00E+00	BTAF1	-4.88E-03	1.00E+00
FSD1L	7.09E-01	3.21E-04	DIAPH2	1.34E-02	1.00E+00	KLC1	3.00E-03	1.00E+00
OPRL1	1.14E+00	3.22E-04	SPAG7	2.02E-02	1.00E+00	ZFP90	-7.06E-03	1.00E+00
ESYT2	3.57E-01	3.22E-04	MAATS1	-1.67E-02	1.00E+00	SBNO2	4.47E-03	1.00E+00
CDKN1C	-7.16E-01	3.24E-04	UBE2F	-1.21E-02	1.00E+00	RUBCN	3.98E-03	1.00E+00
TEX38	1.13E+00	3.24E-04	PGBD5	4.03E-02	1.00E+00	RPL34	-5.26E-03	1.00E+00
COG3	4.48E-01	3.24E-04	DHRS1	1.39E-02	1.00E+00	LOC112579955	-6.85E-03	1.00E+00
LOC102413305	-4.62E-01	3.25E-04	NCK1	1.09E-02	1.00E+00	CHD8	-2.99E-03	1.00E+00
SLC23A2	-4.70E-01	3.26E-04	CCDC97	-2.04E-02	1.00E+00	ATP2A3	1.20E-02	1.00E+00
CRISPLD1	-1.52E+00	3.30E-04	STRAP	9.71E-03	1.00E+00	LOC112583788	6.85E-03	1.00E+00
AGAP3	-3.87E-01	3.30E-04	ARNT	-3.67E-02	1.00E+00	FKBP4	-4.70E-03	1.00E+00
CFAP57	1.29E+00	3.31E-04	LOC112583788	-4.84E-02	1.00E+00	ACAD8	3.28E-03	1.00E+00
KCNN1	9.33E-01	3.32E-04	GATA3	-6.57E-02	1.00E+00	MAPK1IP1L	8.15E-03	1.00E+00
PGAM2	8.11E-01	3.33E-04	CENPU	-1.25E-02	1.00E+00	CNOT1	3.30E-03	1.00E+00
FDPS	4.90E-01	3.33E-04	COLGALT2	8.34E-02	1.00E+00	SLC16A1	-2.79E-03	1.00E+00
LOC102410676	-4.26E-01	3.33E-04	RDH14	1.42E-02	1.00E+00	HLTF	-3.60E-03	1.00E+00
LOC112579974	-1.14E+00	3.36E-04	PCED1A	-1.64E-02	1.00E+00	PATZ1	3.06E-03	1.00E+00

LOC102398643	-1.25E+00	3.38E-04	RINT1	2.50E-02	1.00E+00	DHRS7	5.83E-03	1.00E+00
PRADC1	6.70E-01	3.38E-04	PRMT2	-2.21E-02	1.00E+00	GTF2H1	3.02E-03	1.00E+00
N4BP2L2	-3.21E-01	3.39E-04	RGS8	-1.47E-02	1.00E+00	WFDC3	-5.08E-03	1.00E+00
DHRS13	7.04E-01	3.39E-04	ACER2	4.68E-02	1.00E+00	FBXL20	3.80E-03	1.00E+00
PLEKHJ1	-5.42E-01	3.39E-04	LOC102390935	-2.80E-02	1.00E+00	DNAH12	-6.67E-03	1.00E+00
LOC112585051	-1.68E+00	3.41E-04	ZNF672	5.98E-02	1.00E+00	TCP1	-3.23E-03	1.00E+00
ASGR1	-9.87E-01	3.44E-04	ANAPC5	-2.41E-02	1.00E+00	CFP	6.26E-03	1.00E+00
CENPP	5.51E-01	3.45E-04	FBXO15	1.02E-02	1.00E+00	HAS3	3.87E-03	1.00E+00
COL19A1	1.94E+00	3.45E-04	KBTBD12	1.82E-02	1.00E+00	CRELD1	-3.17E-03	1.00E+00
TAF10	-5.62E-01	3.46E-04	DLAT	1.44E-02	1.00E+00	TRAPPC13	2.68E-03	1.00E+00
ALKBH2	5.02E-01	3.47E-04	PTS	2.66E-02	1.00E+00	LCA5L	3.68E-03	1.00E+00
PSTK	-6.78E-01	3.48E-04	GLMP	-1.71E-02	1.00E+00	NEO1	2.86E-03	1.00E+00
PPP3R1	-4.19E-01	3.49E-04	PIGK	2.81E-02	1.00E+00	EEF1AKMT1	2.84E-03	1.00E+00
LOC102414288	4.60E-01	3.51E-04	LOC112585040	-1.95E-02	1.00E+00	MRPL42	-3.16E-03	1.00E+00
CLCF1	9.20E-01	3.55E-04	VIM	-6.68E-02	1.00E+00	PDS5B	3.06E-03	1.00E+00
ZNRF1	4.29E-01	3.56E-04	FNBP4	2.23E-02	1.00E+00	IMPDH2	-3.53E-03	1.00E+00
LOC102396068	2.94E+00	3.56E-04	TJP2	1.56E-02	1.00E+00	RER1	2.77E-03	1.00E+00
NDUFAF2	-5.57E-01	3.59E-04	GPR158	1.36E-02	1.00E+00	RPSA	4.62E-03	1.00E+00
TET3	6.47E-01	3.59E-04	WDR36	-1.55E-02	1.00E+00	TTC32	5.69E-03	1.00E+00
IL18	-1.59E+00	3.62E-04	UGP2	1.05E-02	1.00E+00	ERO1A	3.24E-03	1.00E+00
MARCH8	-3.54E-01	3.62E-04	LOC102403238	3.43E-02	1.00E+00	YAE1	-3.53E-03	1.00E+00
KDELR2	-3.46E-01	3.64E-04	TLR2	-1.72E-02	1.00E+00	RETREG2	-2.54E-03	1.00E+00
TANC1	5.17E-01	3.67E-04	SLC27A2	4.57E-02	1.00E+00	LOC102402672	-5.53E-03	1.00E+00
ZNF774	-8.20E-01	3.67E-04	NOL12	1.34E-02	1.00E+00	KDM5C	2.68E-03	1.00E+00
SELENBP1	6.26E-01	3.68E-04	LOC102402768	-3.30E-02	1.00E+00	HID1	-3.19E-03	1.00E+00
SSU72	-4.16E-01	3.68E-04	CSF1R	-9.85E-03	1.00E+00	PKD2	3.72E-03	1.00E+00
NOD2	-1.84E+00	3.72E-04	GPS2	1.56E-02	1.00E+00	UBE3A	-2.54E-03	1.00E+00
SLC9A2	1.99E+00	3.76E-04	LOC102402580	1.27E-02	1.00E+00	FKBP15	2.42E-03	1.00E+00
B4GALT1	-4.15E-01	3.79E-04	LOC112586981	-5.54E-02	1.00E+00	ACP5	8.20E-03	1.00E+00
SLC35A5	4.16E-01	3.84E-04	ZUP1	1.08E-02	1.00E+00	LOC102397558	-4.39E-03	1.00E+00
DDX49	-4.59E-01	3.84E-04	SLC15A4	-9.99E-03	1.00E+00	GTF2H4	2.97E-03	1.00E+00
GPR137C	8.07E-01	3.85E-04	C9H19orf44	3.33E-02	1.00E+00	ADAM10	2.78E-03	1.00E+00
ALS2	4.35E-01	3.85E-04	RPS27L	1.65E-02	1.00E+00	TNFRSF18	-6.29E-03	1.00E+00
CDK2	4.03E-01	3.86E-04	B3GALNT1	3.20E-02	1.00E+00	PRDX5	3.41E-03	1.00E+00
COQ3	-3.59E-	3.88E-04	LRAT	-2.94E-	1.00E+00	HEXA	-2.79E-	1.00E+00

	01			02			03	
DNAJC13	4.83E-01	3.89E-04	SLC27A1	1.98E-02	1.00E+00	ZMPSTE24	2.88E-03	1.00E+00
LOC102415247	-4.12E+00	3.94E-04	UBE2A	-1.67E-02	1.00E+00	PSMB2	-2.98E-03	1.00E+00
OAF	4.92E-01	3.94E-04	FAM169A	1.13E-02	1.00E+00	ATP1B3	-2.87E-03	1.00E+00
LOC102411167	-6.36E-01	3.94E-04	ABCA4	-1.40E-02	1.00E+00	WDFY1	2.49E-03	1.00E+00
FHOD3	4.09E-01	3.99E-04	RAB1A	9.28E-03	1.00E+00	LRRC59	-2.52E-03	1.00E+00
TK2	4.49E-01	4.03E-04	IRF5	5.25E-02	1.00E+00	SHLD1	3.70E-03	1.00E+00
MAP3K4	-3.67E-01	4.04E-04	CPNE9	-2.33E-02	1.00E+00	CSK	2.45E-03	1.00E+00
GPR137	4.38E-01	4.04E-04	LOC112587785	4.12E-02	1.00E+00	CTSB	-4.50E-03	1.00E+00
GGN	1.19E+00	4.06E-04	MMP24OS	-3.23E-02	1.00E+00	PICALM	2.87E-03	1.00E+00
DCAF16	-7.79E-01	4.08E-04	CDV3	1.19E-02	1.00E+00	TMPRSS9	-5.51E-03	1.00E+00
FAM168B	-3.12E-01	4.10E-04	PRCC	-1.20E-02	1.00E+00	GRAMD4	-2.69E-03	1.00E+00
BNIP3	-4.77E-01	4.12E-04	SPRYD4	3.49E-02	1.00E+00	LOC112582946	-5.29E-03	1.00E+00
SH3TC1	-1.62E+00	4.13E-04	SPATS2	1.10E-02	1.00E+00	TRMT112	-3.49E-03	1.00E+00
CRMP1	5.25E-01	4.14E-04	MRPL9	-1.67E-02	1.00E+00	TRIM4	-2.95E-03	1.00E+00
TRIB1	-8.96E-01	4.16E-04	TMEM208	2.14E-02	1.00E+00	ATP6V0A2	-3.91E-03	1.00E+00
ARF1	-4.07E-01	4.17E-04	CCDC130	1.46E-02	1.00E+00	VTI1A	2.27E-03	1.00E+00
SYTL1	1.08E+00	4.20E-04	HPS4	-2.57E-02	1.00E+00	VWA7	-5.45E-03	1.00E+00
ITGB3BP	-9.74E-01	4.21E-04	OGA	-1.32E-02	1.00E+00	AHDC1	2.75E-03	1.00E+00
LOC102408579	8.93E-01	4.23E-04	KIAA1841	-3.10E-02	1.00E+00	ARV1	-3.08E-03	1.00E+00
LOC112586123	4.93E-01	4.26E-04	SLC25A38	9.56E-03	1.00E+00	LOC112582172	-4.80E-03	1.00E+00
RIN1	-8.69E-01	4.31E-04	LOC102392550	1.13E-02	1.00E+00	LOC102392353	3.04E-03	1.00E+00
ZMAT2	3.54E-01	4.33E-04	CCDC163	-3.94E-02	1.00E+00	LOC112584546	-4.66E-03	1.00E+00
PARK7	-5.40E-01	4.35E-04	HNRNPU	-2.32E-02	1.00E+00	SYN2	4.74E-03	1.00E+00
LOC112580306	1.27E+00	4.36E-04	LOC112579629	4.13E-02	1.00E+00	NEXMIF	3.79E-03	1.00E+00
CTBP2	3.41E-01	4.37E-04	NF1	2.00E-02	1.00E+00	SUGT1	2.51E-03	1.00E+00
HSPB2	-1.49E+00	4.37E-04	FAM24A	6.75E-02	1.00E+00	C17H5orf52	-4.78E-03	1.00E+00
MRPL38	4.48E-01	4.37E-04	PPP1R16A	-3.01E-02	1.00E+00	OTUB1	-2.53E-03	1.00E+00
LOC102402106	-1.32E+00	4.39E-04	PIGA	2.94E-02	1.00E+00	ZNF511	-3.66E-03	1.00E+00
LOC112578877	2.19E+00	4.39E-04	LOC102395459	-5.43E-02	1.00E+00	UBALD2	-3.05E-03	1.00E+00
DGUOK	3.76E-01	4.40E-04	SLC16A11	-1.88E-02	1.00E+00	KLF11	-2.54E-03	1.00E+00
LOC102409482	1.28E+00	4.41E-04	MAPK1	1.12E-02	1.00E+00	BNC2	2.58E-03	1.00E+00
WDR18	-4.04E-01	4.43E-04	SUSD4	-2.19E-02	1.00E+00	CRYZL1	2.90E-03	1.00E+00
PBRM1	6.97E-01	4.43E-04	EPHX2	2.40E-02	1.00E+00	SLC16A7	3.75E-03	1.00E+00
KCNN2	6.83E-01	4.45E-04	PSMF1	-1.26E-	1.00E+00	MAP3K1	2.87E-03	1.00E+00

				02				
ANKRD6	-5.30E-01	4.45E-04	LOC102395309	-9.28E-02	1.00E+00	LOC102396296	-3.82E-03	1.00E+00
EBNA1BP2	-5.23E-01	4.49E-04	LOC102410880	-2.97E-02	1.00E+00	TCF19	2.86E-03	1.00E+00
OTUB1	-4.08E-01	4.61E-04	MRPS26	-1.81E-02	1.00E+00	TCTA	-2.84E-03	1.00E+00
LOC112581895	4.44E-01	4.69E-04	PRKAR2B	-5.17E-02	1.00E+00	UBE2J1	2.58E-03	1.00E+00
ANKRD46	-4.26E-01	4.70E-04	SLC35A1	-1.06E-02	1.00E+00	REPS1	1.93E-03	1.00E+00
VPS16	-3.62E-01	4.75E-04	ZNF354A	-1.21E-02	1.00E+00	TRRAP	2.50E-03	1.00E+00
ANKIB1	5.16E-01	4.75E-04	POLR2J	-1.12E-02	1.00E+00	LPIN2	2.07E-03	1.00E+00
CEP350	4.82E-01	4.76E-04	CCNL1	-2.74E-02	1.00E+00	BDH2	2.05E-03	1.00E+00
SATB1	4.65E-01	4.76E-04	MPP2	-1.09E-02	1.00E+00	TARDBP	1.95E-03	1.00E+00
RAB6A	3.63E-01	4.79E-04	ELMO3	4.99E-02	1.00E+00	ATP5MC2	-2.93E-03	1.00E+00
LOC102413321	-1.39E+00	4.79E-04	C24H7orf61	-5.82E-02	1.00E+00	FCGBP	2.83E-03	1.00E+00
TMEM19	-8.59E-01	4.80E-04	DKC1	-1.03E-02	1.00E+00	ZBED4	-2.41E-03	1.00E+00
DNER	-1.79E+00	4.80E-04	EIF4A1	-1.03E-02	1.00E+00	PAQR5	-4.91E-03	1.00E+00
CD14	-4.07E+00	4.86E-04	LOC112578050	-3.74E-02	1.00E+00	SRXN1	3.73E-03	1.00E+00
LOC102394044	-1.15E+00	4.87E-04	LOC112585072	-4.06E-02	1.00E+00	STK26	4.10E-03	1.00E+00
GMDS	4.76E-01	4.95E-04	MSL2	2.29E-02	1.00E+00	COIL	2.10E-03	1.00E+00
LOC102395004	-1.59E+00	4.95E-04	MSX2	-1.59E-02	1.00E+00	LOC102393901	1.87E-03	1.00E+00
FAM49B	2.94E-01	4.99E-04	RASGRP1	1.89E-02	1.00E+00	CCDC39	4.51E-03	1.00E+00
DMBT1	-2.87E+00	5.04E-04	LOC112578444	-2.13E-02	1.00E+00	ALG2	2.59E-03	1.00E+00
SRP9	3.96E-01	5.05E-04	HDAC2	-1.31E-02	1.00E+00	TRPV2	-5.74E-03	1.00E+00
PLCB2	-2.50E+00	5.11E-04	PURB	-3.14E-02	1.00E+00	DYRK1B	1.83E-03	1.00E+00
TM2D2	-3.69E-01	5.14E-04	MCRIP2	1.30E-02	1.00E+00	HPS4	-1.84E-03	1.00E+00
APH1A	-3.32E-01	5.14E-04	ADGRA2	3.20E-02	1.00E+00	URB1	-2.30E-03	1.00E+00
LIN9	-6.54E-01	5.18E-04	KDM8	-3.30E-02	1.00E+00	CDC27	2.00E-03	1.00E+00
WDR11	3.91E-01	5.19E-04	HEMK1	3.66E-02	1.00E+00	NLGN3	-3.90E-03	1.00E+00
MORF4L2	-3.66E-01	5.21E-04	TEX14	1.64E-02	1.00E+00	LSM6	-2.51E-03	1.00E+00
DNMT3A	3.79E-01	5.22E-04	GADD45GIP1	-1.70E-02	1.00E+00	SLC16A14	4.62E-03	1.00E+00
LOC112579228	3.26E+00	5.23E-04	MALT1	-1.36E-02	1.00E+00	RPS6KA6	2.71E-03	1.00E+00
PIP4K2B	3.26E-01	5.26E-04	MPDZ	-1.77E-02	1.00E+00	MBTD1	-2.41E-03	1.00E+00
KCND2	2.03E+00	5.29E-04	TALDO1	9.90E-03	1.00E+00	CBLL1	-2.17E-03	1.00E+00
RNF25	3.96E-01	5.33E-04	LRRCC1	1.27E-02	1.00E+00	WTAP	-1.61E-03	1.00E+00
LOC112584435	-1.10E+00	5.34E-04	LOC112586944	-1.18E-02	1.00E+00	OPA3	2.12E-03	1.00E+00

GRIK3	2.06E+00	5.37E-04	SRD5A1	7.40E-02	1.00E+00	KRTCAP3	-4.33E-03	1.00E+00
CHCHD2	-7.14E-01	5.38E-04	LOC102393736	-4.10E-02	1.00E+00	MSH6	1.65E-03	1.00E+00
GEMIN2	3.84E-01	5.39E-04	LOC112578955	2.56E-02	1.00E+00	FAM168A	-1.99E-03	1.00E+00
NFX1	3.18E-01	5.41E-04	LOC102399263	2.04E-02	1.00E+00	PRR22	4.16E-03	1.00E+00
LOC102389065	1.59E+00	5.42E-04	CPA5	-1.37E-02	1.00E+00	C1H21orf2	2.33E-03	1.00E+00
MRPL52	-5.80E-01	5.42E-04	HARBI1	-1.29E-02	1.00E+00	FBXL15	3.09E-03	1.00E+00
PRDM5	-3.68E-01	5.48E-04	ERCC6L2	1.48E-02	1.00E+00	IFRD2	2.39E-03	1.00E+00
LY75	1.86E+00	5.48E-04	DNAJC21	1.18E-02	1.00E+00	USP47	1.62E-03	1.00E+00
PSMD13	-4.58E-01	5.52E-04	FAAP20	-1.85E-02	1.00E+00	TMTC3	-2.72E-03	1.00E+00
NOC4L	3.56E-01	5.62E-04	ZFP3	-1.68E-02	1.00E+00	FRYL	2.42E-03	1.00E+00
THAP1	3.85E-01	5.63E-04	LOC102410239	-6.59E-02	1.00E+00	FAM49A	-1.85E-03	1.00E+00
EIF4G2	-5.19E-01	5.63E-04	LOC112579251	4.95E-02	1.00E+00	ZNF275	2.16E-03	1.00E+00
LSM4	-5.41E-01	5.64E-04	EXOC3	9.16E-03	1.00E+00	ZNF782	-2.74E-03	1.00E+00
WRNIP1	3.92E-01	5.65E-04	SDCCAG8	-2.15E-02	1.00E+00	NBL1	2.81E-03	1.00E+00
INPP4B	-6.88E-01	5.65E-04	SLC43A2	-1.23E-02	1.00E+00	MYO18A	2.06E-03	1.00E+00
COL13A1	1.78E+00	5.76E-04	LMF2	1.64E-02	1.00E+00	GTF2F1	2.25E-03	1.00E+00
MALT1	-3.97E-01	5.76E-04	CPNE7	-4.37E-02	1.00E+00	KIF7	2.48E-03	1.00E+00
DKK2	2.12E+00	5.76E-04	LOC102394850	-1.31E-02	1.00E+00	NXT1	2.20E-03	1.00E+00
LOC102404057	-1.39E+00	5.85E-04	EPM2AIP1	-2.38E-02	1.00E+00	ERC1	1.54E-03	1.00E+00
LOC102393166	5.37E-01	5.86E-04	CDC42	-8.71E-03	1.00E+00	ASXL3	3.26E-03	1.00E+00
PRR11	-6.60E-01	5.94E-04	LUZP2	-1.95E-02	1.00E+00	HEMK1	2.23E-03	1.00E+00
INPP1	6.73E-01	5.95E-04	NUCB2	1.39E-02	1.00E+00	ELAVL3	3.32E-03	1.00E+00
DEF6	7.54E-01	5.96E-04	RNF135	-9.06E-02	1.00E+00	CNTD1	-3.45E-03	1.00E+00
LRGUK	1.52E+00	5.98E-04	DHX15	1.23E-02	1.00E+00	MGAT1	1.48E-03	1.00E+00
ZRSR2	4.04E-01	6.09E-04	RRP1B	8.54E-03	1.00E+00	LOC112578116	-2.79E-03	1.00E+00
SOWAHA	1.40E+00	6.16E-04	FKBP4	-1.00E-02	1.00E+00	GOLGA5	1.66E-03	1.00E+00
CCDC142	-7.43E-01	6.16E-04	LOC102413417	4.62E-02	1.00E+00	LOC112585052	2.98E-03	1.00E+00
WDR12	-4.35E-01	6.17E-04	FIG4	-1.12E-02	1.00E+00	MSTO1	2.11E-03	1.00E+00
LOC112578240	-1.06E+00	6.21E-04	SLC9B2	1.78E-02	1.00E+00	DGKQ	-2.38E-03	1.00E+00
AP1AR	-4.81E-01	6.22E-04	LOC112585185	3.25E-02	1.00E+00	HGH1	1.83E-03	1.00E+00
C1QC	-3.78E+00	6.23E-04	CACNA2D2	-1.05E-02	1.00E+00	GPX1	-2.10E-03	1.00E+00
KAZALD1	7.15E-01	6.26E-04	ZSWIM3	2.49E-02	1.00E+00	C1QA	-6.93E-03	1.00E+00
MFHAS1	4.01E-01	6.31E-04	NSA2	-1.42E-02	1.00E+00	PCDHB7	-3.09E-03	1.00E+00
CADPS	5.32E-01	6.41E-04	LARP7	9.88E-03	1.00E+00	ASCC3	-1.75E-03	1.00E+00

LOC112578841	1.57E+00	6.41E-04	TMEM145	1.50E-02	1.00E+00	EI24	-1.48E-03	1.00E+00
LOC102391413	1.15E+00	6.47E-04	BCL7B	1.01E-02	1.00E+00	SRGN	-3.08E-03	1.00E+00
MRPL54	-5.27E-01	6.58E-04	TRAM1	-9.08E-03	1.00E+00	ZSCAN23	1.75E-03	1.00E+00
CFAP99	-1.20E+00	6.60E-04	LOC102409912	1.11E-02	1.00E+00	TTC31	2.80E-03	1.00E+00
RTN3	-2.98E-01	6.61E-04	RAD17	9.17E-03	1.00E+00	DDX60	3.45E-03	1.00E+00
ERMP1	3.72E-01	6.63E-04	GABRB3	1.63E-02	1.00E+00	RPS11	-2.75E-03	1.00E+00
SEM1	5.50E-01	6.66E-04	SCN2A	1.87E-02	1.00E+00	ZNF263	1.83E-03	1.00E+00
ZMAT5	-5.61E-01	6.66E-04	NR1H4	1.74E-02	1.00E+00	CCDC6	-1.29E-03	1.00E+00
CPEB4	-1.12E+00	6.70E-04	RGS19	2.24E-02	1.00E+00	LOC112586503	2.01E-03	1.00E+00
MRPL2	-5.47E-01	6.74E-04	COPS2	-1.25E-02	1.00E+00	ACO1	-1.24E-03	1.00E+00
LOC112585613	2.11E+00	6.74E-04	DNAAF1	3.65E-02	1.00E+00	LOC112583745	3.19E-03	1.00E+00
SCN11A	1.23E+00	6.77E-04	TUBA8	5.14E-02	1.00E+00	APOBR	2.60E-03	1.00E+00
ZNRF3	-4.86E-01	6.86E-04	LOC102399273	7.76E-02	1.00E+00	PRMT9	1.51E-03	1.00E+00
LOC102395631	1.73E+00	6.89E-04	ASIC1	1.26E-02	1.00E+00	LOC102415264	3.31E-03	1.00E+00
TMEM107	3.70E-01	6.91E-04	LRRC28	1.49E-02	1.00E+00	RALGAPB	1.73E-03	1.00E+00
RPAP3	-3.55E-01	6.93E-04	LOC112580461	1.91E-02	1.00E+00	UBL4A	1.82E-03	1.00E+00
PTPN14	5.14E-01	6.94E-04	VPS16	9.78E-03	1.00E+00	CALCOCO2	-1.49E-03	1.00E+00
PAQR4	9.03E-01	6.98E-04	NDUFAF7	-1.41E-02	1.00E+00	ZNF19	2.27E-03	1.00E+00
PICALM	3.58E-01	7.03E-04	C6H1orf123	1.45E-02	1.00E+00	HCN3	-3.51E-03	1.00E+00
PROSER1	6.02E-01	7.14E-04	ATP6V0C	8.85E-03	1.00E+00	CSGALNACT1	1.87E-03	1.00E+00
FAM131C	-9.17E-01	7.17E-04	WBP4	1.03E-02	1.00E+00	VTI1B	-1.48E-03	1.00E+00
GPANK1	4.97E-01	7.23E-04	USP54	1.52E-02	1.00E+00	NOLC1	1.31E-03	1.00E+00
GPAT4	-4.07E-01	7.24E-04	LOC112581780	4.70E-02	1.00E+00	DCUN1D4	1.29E-03	1.00E+00
VPS13A	-5.17E-01	7.25E-04	TIMM17B	-1.29E-02	1.00E+00	CCDC124	-1.77E-03	1.00E+00
BRIX1	4.83E-01	7.26E-04	LOC112577646	5.22E-02	1.00E+00	LOC112578055	2.36E-03	1.00E+00
MTO1	3.62E-01	7.27E-04	LOC102400925	1.97E-02	1.00E+00	LOC112586818	-2.68E-03	1.00E+00
ZNF518B	4.05E-01	7.28E-04	LOC102398905	1.22E-02	1.00E+00	LRRC57	-1.43E-03	1.00E+00
LOC102402496	-1.20E+00	7.31E-04	BAG1	-1.81E-02	1.00E+00	LOC102394044	-2.66E-03	1.00E+00
RPRD2	3.89E-01	7.32E-04	KLHL25	1.01E-02	1.00E+00	PRDM6	3.05E-03	1.00E+00
MITD1	-6.82E-01	7.35E-04	LOC102403928	-4.67E-02	1.00E+00	ROM1	2.31E-03	1.00E+00
LOC102394339	1.44E+00	7.36E-04	SMTN	4.74E-02	1.00E+00	SLC6A20	2.61E-03	1.00E+00
FAM206A	-4.09E-01	7.40E-04	PSMB9	-3.00E-02	1.00E+00	COPB1	-1.17E-03	1.00E+00
MEMO1	-3.08E-01	7.44E-04	STOML2	-9.32E-03	1.00E+00	NDFIP2	1.57E-03	1.00E+00
CLIP1	4.50E-01	7.46E-04	TMEM267	-1.08E-02	1.00E+00	NPTXR	-2.69E-03	1.00E+00
LOC102403853	2.20E+00	7.49E-04	MYOM1	1.44E-02	1.00E+00	CDC73	1.32E-03	1.00E+00
ASB6	-3.70E-01	7.51E-04	AP1S2	1.32E-02	1.00E+00	PROSER1	1.78E-03	1.00E+00

ADAM23	-1.13E+00	7.52E-04	GNAO1	1.22E-02	1.00E+00	FOXJ3	-1.42E-03	1.00E+00
PREPL	7.03E-01	7.55E-04	RAE1	-8.64E-03	1.00E+00	PAK1IP1	-1.32E-03	1.00E+00
PPRC1	3.72E-01	7.56E-04	AGGF1	1.10E-02	1.00E+00	SAMHD1	-1.32E-03	1.00E+00
LOC102406855	-1.30E+00	7.57E-04	TPMT	-2.17E-02	1.00E+00	TMEM184B	-1.26E-03	1.00E+00
ELMO2	-3.54E-01	7.62E-04	TGFB1I1	6.89E-02	1.00E+00	LOC102397633	-2.16E-03	1.00E+00
QRSL1	3.49E-01	7.65E-04	EARS2	-2.13E-02	1.00E+00	POC5	1.25E-03	1.00E+00
EIF3J	4.28E-01	7.66E-04	DIS3L2	-1.85E-02	1.00E+00	FRG1	1.48E-03	1.00E+00
CSTF1	4.20E-01	7.66E-04	CA9	1.43E-02	1.00E+00	IMPA2	2.39E-03	1.00E+00
PPP1R1A	-7.52E-01	7.76E-04	LOC112583686	2.15E-02	1.00E+00	WDR33	1.02E-03	1.00E+00
LOC112586975	1.40E+00	7.79E-04	SLC43A1	-5.67E-02	1.00E+00	CD99	-1.22E-03	1.00E+00
DNAJC12	-1.06E+00	7.80E-04	BACE1	-2.30E-02	1.00E+00	SSU72	1.12E-03	1.00E+00
NFIA	-8.83E-01	7.86E-04	TMBIM4	1.26E-02	1.00E+00	TMEM42	-1.65E-03	1.00E+00
ANAPC16	1.91E+00	7.86E-04	MAN2A1	-1.29E-02	1.00E+00	SORD	1.63E-03	1.00E+00
LOC102410412	-1.17E+00	7.89E-04	MAP4K3	-2.43E-02	1.00E+00	AMBRA1	-9.54E-04	1.00E+00
TWF1	4.76E-01	8.02E-04	ARMC5	-1.76E-02	1.00E+00	RNF169	1.09E-03	1.00E+00
MCM3AP	3.39E-01	8.02E-04	RITA1	-1.18E-02	1.00E+00	COMMD9	1.29E-03	1.00E+00
LOC102410066	1.76E+00	8.07E-04	SNF8	1.23E-02	1.00E+00	THSD1	-2.42E-03	1.00E+00
C15H8orf82	-5.19E-01	8.12E-04	LEXM	2.48E-02	1.00E+00	RPS6KL1	-1.63E-03	1.00E+00
MELTF	-9.60E-01	8.16E-04	KCNN3	1.05E-02	1.00E+00	VPS16	9.89E-04	1.00E+00
GATB	3.45E-01	8.16E-04	GPAA1	1.31E-02	1.00E+00	LOC112583843	-2.27E-03	1.00E+00
LOC112582053	-6.87E-01	8.19E-04	ATF5	3.52E-02	1.00E+00	RAB2B	-9.74E-04	1.00E+00
DACH2	1.29E+00	8.20E-04	HMBS	3.01E-02	1.00E+00	VCPKMT	1.25E-03	1.00E+00
PLEKHH1	-7.44E-01	8.25E-04	SOX5	1.88E-02	1.00E+00	PLCB2	-4.29E-03	1.00E+00
DNAJC3	3.04E-01	8.25E-04	FAM135B	-2.61E-02	1.00E+00	GSK3A	8.61E-04	1.00E+00
MARF1	-4.40E-01	8.27E-04	TBL1X	-1.77E-02	1.00E+00	UBR7	8.75E-04	1.00E+00
ATP5MC1	5.65E-01	8.28E-04	C11H15orf61	-1.76E-02	1.00E+00	TRIP6	9.90E-04	1.00E+00
TTC1	3.30E-01	8.31E-04	RCOR2	-8.74E-03	1.00E+00	NAGK	-1.10E-03	1.00E+00
ALOX5AP	6.85E-01	8.38E-04	FAM131B	-5.76E-02	1.00E+00	ZKSCAN2	1.22E-03	1.00E+00
SLC9A5	1.15E+00	8.41E-04	DGKQ	-2.70E-02	1.00E+00	PJVK	1.80E-03	1.00E+00
LOC112578122	-1.10E+00	8.45E-04	ACYP1	-1.67E-02	1.00E+00	TNS2	-8.72E-04	1.00E+00
YWHAG	-3.48E-01	8.46E-04	LRP3	-4.51E-02	1.00E+00	SPG11	8.46E-04	1.00E+00
MARK4	3.30E-01	8.51E-04	LOC102415185	3.01E-02	1.00E+00	NDUFA3	-1.16E-03	1.00E+00
LOC102395556	-9.49E-01	8.54E-04	HMOX2	-8.84E-03	1.00E+00	WDR5B	1.78E-03	1.00E+00

LOC112586800	1.56E+00		8.61E-04	ALX1	9.02E-03	1.00E+00	PTPN3	-9.67E-04	1.00E+00
LOC102405195	-4.89E-01		8.62E-04	SET	9.06E-03	1.00E+00	YTHDF2	-8.26E-04	1.00E+00
LOC102406631	-6.81E-01		8.75E-04	SETD4	3.40E-02	1.00E+00	ZNF529	1.63E-03	1.00E+00
LSM5	-5.58E-01		8.82E-04	KDELR2	-8.94E-03	1.00E+00	MFGE8	-1.27E-03	1.00E+00
AARS	-3.53E-01		8.87E-04	LOC102394770	-1.88E-02	1.00E+00	WDR36	9.23E-04	1.00E+00
LOC112583873	1.53E+00		8.93E-04	FAM118A	-2.71E-02	1.00E+00	MRPS6	-9.58E-04	1.00E+00
CENPN	-5.34E-01		9.06E-04	PCDH11X	4.67E-02	1.00E+00	LIN7B	1.05E-03	1.00E+00
FRMPD1	-8.11E-01		9.08E-04	LOC112586811	4.32E-02	1.00E+00	PPP2R2C	-1.32E-03	1.00E+00
C1H3orf33	-4.21E-01		9.12E-04	MID1IP1	-3.60E-02	1.00E+00	LOC102401107	1.25E-03	1.00E+00
ZNF318	5.01E-01		9.21E-04	SH3KBP1	1.22E-02	1.00E+00	TRIR	-8.08E-04	1.00E+00
SPRY1	6.55E-01		9.29E-04	LOC102400568	-2.95E-02	1.00E+00	WWC3	6.65E-04	1.00E+00
SIRT4	-4.99E-01		9.33E-04	TCF12	1.75E-02	1.00E+00	EPB41L2	6.23E-04	1.00E+00
MRPL34	-5.53E-01		9.34E-04	ZFAND6	-9.66E-03	1.00E+00	LOC102392736	7.13E-04	1.00E+00
EFL1	3.19E-01		9.40E-04	CSE1L	8.15E-03	1.00E+00	BBX	7.39E-04	1.00E+00
LOC102394049	-7.41E-01		9.48E-04	TPH2	1.81E-02	1.00E+00	ZNF317	7.71E-04	1.00E+00
LOC102393336	-7.12E-01		9.49E-04	APEX1	8.97E-03	1.00E+00	SPNS2	1.37E-03	1.00E+00
NPNT	-4.56E-01		9.51E-04	FGR	-8.24E-02	1.00E+00	LONP1	-7.17E-04	1.00E+00
PQLC2	-4.78E-01		9.56E-04	SMARCA4	1.23E-02	1.00E+00	SOCS3	-1.39E-03	1.00E+00
TGIF2	3.62E-01		9.67E-04	TMOD1	-9.23E-03	1.00E+00	ELAC1	7.08E-04	1.00E+00
SERPINE3	-9.23E-01		9.73E-04	PGPEP1	4.29E-02	1.00E+00	TAGAP	-1.69E-03	1.00E+00
LOC102412899	-7.69E-01		9.74E-04	ARPC1B	1.10E-02	1.00E+00	HIGD2A	-7.64E-04	1.00E+00
MAST1	-1.45E+00		9.75E-04	TMCC1	2.70E-02	1.00E+00	CHMP7	-5.53E-04	1.00E+00
HNRNPF	3.79E-01		9.80E-04	UCHL3	-9.86E-03	1.00E+00	ZNHIT1	-7.92E-04	1.00E+00
RAB11FIP3	3.24E-01		9.83E-04	DCTN3	1.23E-02	1.00E+00	SRP19	-5.20E-04	1.00E+00
SHISA6	-1.63E+00		9.86E-04	LOC112579192	3.83E-02	1.00E+00	IDH3B	-5.87E-04	1.00E+00
KCNJ6	3.66E-01		9.90E-04	CRKL	9.64E-03	1.00E+00	MAST1	1.06E-03	1.00E+00
PPP1CB	-3.98E-01		9.92E-04	PSENEN	1.52E-02	1.00E+00	SLC35A3	-7.02E-04	1.00E+00
KLRG2	6.54E-01		9.97E-04	PIP4K2B	-1.13E-02	1.00E+00	SETX	4.93E-04	1.00E+00
PLEKHG3	-4.34E-01		9.99E-04	C1H21orf91	-3.63E-02	1.00E+00	PLXNA4	-1.04E-03	1.00E+00
CIP2A	-4.52E-01		1.00E-03	CRLF3	8.74E-03	1.00E+00	SPOP	4.25E-04	1.00E+00
SMG5	-3.21E-01		1.00E-03	LOC112579680	2.94E-02	1.00E+00	TTC39C	-4.66E-04	1.00E+00
NHLRC2	-4.27E-01		1.01E-03	RHOF	2.10E-02	1.00E+00	RSPH9	-5.84E-04	1.00E+00
CXCL8	-4.27E+00		1.01E-03	ATP9B	-1.45E-02	1.00E+00	LOC112584524	1.19E-03	1.00E+00

LOC112582563	1.47E+00	1.02E-03	LOC102412021	1.65E-02	1.00E+00	SEMA3B	-6.04E-04	1.00E+00
LOC102393823	-8.80E-01	1.03E-03	RNGTT	1.17E-02	1.00E+00	FKBP3	-4.87E-04	1.00E+00
LOC102412116	2.11E+00	1.03E-03	RTP1	-2.58E-02	1.00E+00	CORO2A	-1.37E-03	1.00E+00
MRPL40	-5.67E-01	1.03E-03	SPG7	1.25E-02	1.00E+00	PPP6R2	4.36E-04	1.00E+00
TRAF1	4.08E-01	1.04E-03	ATP6V0E1	-1.15E-02	1.00E+00	PHF3	4.37E-04	1.00E+00
SLC35G1	9.35E-01	1.06E-03	ARHGEF7	8.26E-03	1.00E+00	ESD	3.84E-04	1.00E+00
C16H11orf49	-4.96E-01	1.06E-03	LOC102406013	-2.64E-02	1.00E+00	VMAC	-7.32E-04	1.00E+00
PIK3R5	6.92E-01	1.07E-03	ZNF395	-1.65E-02	1.00E+00	LOC112586991	-1.50E-03	1.00E+00
FBXW2	3.08E-01	1.08E-03	IDNK	4.71E-02	1.00E+00	STK16	4.84E-04	1.00E+00
RRP9	4.28E-01	1.09E-03	GAN	-1.22E-02	1.00E+00	LARS	-3.19E-04	1.00E+00
FADD	-4.49E-01	1.09E-03	ANKDD1A	-4.49E-02	1.00E+00	VAMP5	-5.33E-04	1.00E+00
DNAJB11	-4.58E-01	1.11E-03	LOC112577729	1.13E-02	1.00E+00	DNPEP	4.51E-04	1.00E+00
LOC102408136	-1.84E+00	1.12E-03	OSBPL5	-2.05E-02	1.00E+00	CCDC138	3.80E-04	1.00E+00
RELA	-3.58E-01	1.13E-03	LOC102414236	-5.05E-02	1.00E+00	RC3H2	-5.32E-04	1.00E+00
NEIL1	-5.49E-01	1.13E-03	CHRNE	1.79E-02	1.00E+00	LOC102395384	-7.68E-04	1.00E+00
NAIP	-1.70E+00	1.13E-03	ZNF236	2.02E-02	1.00E+00	DOP1B	6.00E-04	1.00E+00
UBE2N	-3.33E-01	1.13E-03	NOBOX	8.80E-03	1.00E+00	KCTD13	3.63E-04	1.00E+00
LOC102409782	7.17E-01	1.13E-03	PXMP4	-1.76E-02	1.00E+00	HHIPL1	-4.44E-04	1.00E+00
MRPS25	5.91E-01	1.15E-03	LOC102396428	-2.91E-02	1.00E+00	AHCYL2	-4.53E-04	1.00E+00
LOC112587926	-1.45E+00	1.15E-03	NDEL1	-9.14E-03	1.00E+00	TAF15	-2.99E-04	1.00E+00
SEPT11	3.91E-01	1.15E-03	ZBTB7A	2.82E-02	1.00E+00	ATRIP	3.31E-04	1.00E+00
NECAB3	-6.42E-01	1.16E-03	DOK6	1.56E-02	1.00E+00	GALNT11	2.89E-04	1.00E+00
KANSL3	2.92E-01	1.17E-03	NTHL1	1.53E-02	1.00E+00	SDHAF3	-4.37E-04	1.00E+00
LOC112583829	1.07E+00	1.17E-03	LOC102400858	-1.11E-02	1.00E+00	ATP2B1	-2.85E-04	1.00E+00
MDGA2	-1.72E+00	1.18E-03	NFIX	-2.66E-02	1.00E+00	LOC102394862	-3.03E-04	1.00E+00
COL14A1	8.49E-01	1.18E-03	LPAR1	-5.07E-02	1.00E+00	TWNK	2.59E-04	1.00E+00
TP53BP1	-3.48E-01	1.20E-03	ALDH5A1	1.84E-02	1.00E+00	SIRT5	2.76E-04	1.00E+00
OTUD5	-3.50E-01	1.20E-03	GPC2	-2.31E-02	1.00E+00	LUZP1	-2.57E-04	1.00E+00
LRBA	3.75E-01	1.20E-03	PRICKLE4	-1.77E-02	1.00E+00	BCDIN3D	3.63E-04	1.00E+00
GTF3C3	4.40E-01	1.20E-03	ZFYVE19	-1.29E-02	1.00E+00	IPO9	1.97E-04	1.00E+00
C6H1orf52	-4.37E-01	1.20E-03	LOC102412030	-4.19E-02	1.00E+00	FIBP	-2.66E-04	1.00E+00
LOC112583852	9.63E-01	1.20E-03	NOTO	-1.02E-02	1.00E+00	PKN1	2.21E-04	1.00E+00
PLEKHO1	-4.88E-01	1.21E-03	MRPL57	-2.87E-02	1.00E+00	LOC102398059	2.75E-04	1.00E+00

FBXO4	4.68E-01	1.21E-03	ZBTB11	-1.15E-02	1.00E+00	NBR1	-2.18E-04	1.00E+00
PRKAR1A	-3.12E-01	1.21E-03	FAM234A	1.39E-02	1.00E+00	SSBP4	2.00E-04	1.00E+00
LOC112583843	8.09E-01	1.22E-03	PPIF	1.11E-02	1.00E+00	MRPL36	-2.61E-04	1.00E+00
CASC1	6.57E-01	1.22E-03	TMEM79	-1.70E-02	1.00E+00	TMEM177	-2.78E-04	1.00E+00
SMLR1	1.35E+00	1.22E-03	RALBP1	-8.82E-03	1.00E+00	INTS11	-2.06E-04	1.00E+00
NOX1	-1.30E+00	1.24E-03	DPM3	-1.65E-02	1.00E+00	SCARF2	-5.43E-04	1.00E+00
IMPACT	-4.63E-01	1.25E-03	LOC112580248	1.12E-02	1.00E+00	LOC102401999	2.60E-04	1.00E+00
DUT	-6.32E-01	1.26E-03	CAPRIN2	9.15E-03	1.00E+00	LOC102395499	2.02E-04	1.00E+00
MCM7	-4.36E-01	1.26E-03	GCNT4	2.64E-02	1.00E+00	NAA60	-9.95E-05	1.00E+00
C2H6orf132	1.67E+00	1.26E-03	GALNT9	-1.74E-02	1.00E+00	NF1	-1.12E-04	1.00E+00
RRAGA	3.13E-01	1.27E-03	CEP120	1.05E-02	1.00E+00	GUCA1A	2.44E-04	1.00E+00
SHISA9	1.58E+00	1.27E-03	DAPK1	-3.19E-02	1.00E+00			
BMP8A	-1.30E+00	1.27E-03	BSX	1.16E-02	1.00E+00			
TMEM173	-2.90E+00	1.27E-03	LIPC	-4.62E-02	1.00E+00			
SLC9A1	4.55E-01	1.27E-03	NKX2-2	5.62E-02	1.00E+00			
FAM19A4	-6.86E-01	1.27E-03	UAP1	-2.22E-02	1.00E+00			
LOC112580375	1.38E+00	1.28E-03	GLRA3	2.72E-02	1.00E+00			
ABHD3	-9.47E-01	1.28E-03	ADAM2	4.58E-02	1.00E+00			
KCNJ10	1.49E+00	1.29E-03	RIC3	1.58E-02	1.00E+00			
MAP4	-4.16E-01	1.30E-03	STK24	9.06E-03	1.00E+00			
KTN1	3.32E-01	1.31E-03	CFAP221	5.00E-02	1.00E+00			
PLEK	-2.64E+00	1.31E-03	GABBR1	-4.25E-02	1.00E+00			
LOC102411613	6.14E-01	1.31E-03	GRIP2	-1.05E-02	1.00E+00			
SIK3	-4.07E-01	1.32E-03	SBNO2	-1.41E-02	1.00E+00			
LOC102398755	5.01E-01	1.32E-03	TMEM258	-1.52E-02	1.00E+00			
SELENOF	3.34E-01	1.32E-03	ZC3H12A	2.37E-02	1.00E+00			
FITM2	-7.80E-01	1.32E-03	LOC102397977	4.61E-02	1.00E+00			
NIPSNAP2	-3.05E-01	1.33E-03	TMLHE	2.02E-02	1.00E+00			
TMC5	1.70E+00	1.33E-03	RPTOR	-9.98E-03	1.00E+00			
EIF4G3	-3.51E-01	1.33E-03	TMEM143	1.99E-02	1.00E+00			
SFXN3	-5.20E-01	1.34E-03	UVRAG	9.74E-03	1.00E+00			
ARMC12	8.44E-01	1.35E-03	PRSS22	4.82E-02	1.00E+00			
EYA4	1.73E+00	1.35E-03	SMNDC1	-1.07E-02	1.00E+00			
NAPA	-3.69E-01	1.35E-03	DAP3	1.21E-02	1.00E+00			
LOC102394519	1.21E+00	1.36E-03	LOC112585132	2.25E-02	1.00E+00			

LOC112583695	-1.02E+00	1.37E-03	HERC5	2.90E-02	1.00E+00
CYLD	-3.47E-01	1.38E-03	COL21A1	6.68E-02	1.00E+00
APBB1IP	1.26E+00	1.39E-03	SIX1	-2.23E-02	1.00E+00
EFCAB6	1.07E+00	1.40E-03	DDX17	-1.48E-02	1.00E+00
LOC112581264	6.21E-01	1.40E-03	LOC102405491	3.20E-02	1.00E+00
LOC102416404	-5.08E-01	1.40E-03	PLEKHM3	1.34E-02	1.00E+00
HRAS	4.99E-01	1.40E-03	RAI14	1.22E-02	1.00E+00
LOC102401100	1.36E+00	1.40E-03	LYPLAL1	-1.65E-02	1.00E+00
LRRC15	1.31E+00	1.41E-03	CCDC39	-2.76E-02	1.00E+00
LOC112580389	2.16E+00	1.41E-03	NDUFB8	-1.05E-02	1.00E+00
LOC102410846	-5.16E-01	1.41E-03	PSMD8	1.04E-02	1.00E+00
LOC102393546	-7.76E-01	1.42E-03	PDGFC	5.04E-02	1.00E+00
LOC102410112	2.59E+00	1.42E-03	CEBPZ	8.37E-03	1.00E+00
GRK5	8.32E-01	1.42E-03	WDR55	-1.45E-02	1.00E+00
LOC102405282	-7.16E-01	1.42E-03	CAND1	-9.09E-03	1.00E+00
RGMB	-3.84E-01	1.42E-03	TUFM	8.64E-03	1.00E+00
CD3EAP	-6.16E-01	1.43E-03	PNO1	7.58E-03	1.00E+00
RNF112	9.37E-01	1.43E-03	HS3ST5	3.53E-02	1.00E+00
ALCAM	-1.18E+00	1.43E-03	OST4	-1.27E-02	1.00E+00
FMO1	1.52E+00	1.43E-03	TGFBR2	1.34E-02	1.00E+00
LOC102406763	-4.49E-01	1.43E-03	LOC102397151	-4.13E-02	1.00E+00
TMEM94	-3.69E-01	1.44E-03	EMG1	-1.24E-02	1.00E+00
PBX3	-2.92E-01	1.45E-03	HSPB11	-1.37E-02	1.00E+00
LOC102408909	1.23E+00	1.48E-03	JAK2	2.34E-02	1.00E+00
DOCK8	4.36E-01	1.50E-03	ENTR1	-7.29E-03	1.00E+00
LOC112584686	-1.34E+00	1.52E-03	LZTS3	1.77E-02	1.00E+00
SH3KBP1	-4.82E-01	1.52E-03	DLEC1	-1.43E-02	1.00E+00
NPL	1.51E+00	1.52E-03	HOXD3	1.85E-02	1.00E+00
ZNF570	-6.52E-01	1.52E-03	NAALADL2	1.43E-02	1.00E+00
GUF1	-3.40E-01	1.53E-03	VPS25	1.12E-02	1.00E+00
CAMTA2	3.29E-01	1.53E-03	LOC112579903	3.70E-02	1.00E+00
NMRK1	-5.43E-01	1.54E-03	LZTFL1	-1.87E-02	1.00E+00
GPBP1L1	2.89E-01	1.54E-03	DEUP1	-1.79E-02	1.00E+00
INTS13	-3.86E-01	1.54E-03	PHF6	-1.38E-02	1.00E+00
ARRB1	7.16E-01	1.56E-03	CCDC91	1.18E-02	1.00E+00

TIMM17B	4.69E-01	1.57E-03	SGCZ	-4.83E-02	1.00E+00
MYCBP2	5.38E-01	1.57E-03	LOC102390113	-1.33E-02	1.00E+00
LRRIQ1	1.44E+00	1.58E-03	LOC112586866	1.94E-02	1.00E+00
AKAP12	-4.82E-01	1.58E-03	GALK1	1.45E-02	1.00E+00
CUBN	-1.33E+00	1.59E-03	FUS	-9.14E-03	1.00E+00
LOC102390935	-5.72E-01	1.59E-03	HDGFL2	-7.18E-03	1.00E+00
LOC102396011	-9.13E-01	1.60E-03	AHCTF1	-1.77E-02	1.00E+00
ZFP41	6.18E-01	1.60E-03	UBE2H	-8.78E-03	1.00E+00
IKBKG	3.37E-01	1.61E-03	IKBIP	-2.66E-02	1.00E+00
KLHL11	4.45E-01	1.61E-03	TMEM205	-2.05E-02	1.00E+00
LOC102411494	-7.75E-01	1.62E-03	CHD1	-2.38E-02	1.00E+00
USP11	2.65E-01	1.62E-03	BAALC	-1.70E-02	1.00E+00
EIF5A2	-4.84E-01	1.62E-03	PGAM2	-2.87E-02	1.00E+00
C4H12orf56	-1.23E+00	1.62E-03	BTBD1	1.01E-02	1.00E+00
LOC112587025	-1.24E+00	1.62E-03	COMMD4	1.14E-02	1.00E+00
UNC13C	-4.23E-01	1.64E-03	P2RY1	2.44E-02	1.00E+00
LOC112577644	1.41E+00	1.67E-03	LOC112581649	-2.93E-02	1.00E+00
KRT72	1.53E+00	1.67E-03	GINS1	-3.00E-02	1.00E+00
CHM	4.34E-01	1.68E-03	ABR	7.49E-03	1.00E+00
MICU3	-6.34E-01	1.68E-03	UPF2	-9.58E-03	1.00E+00
PLPP2	-1.01E+00	1.68E-03	MYH14	1.08E-02	1.00E+00
RTKN2	3.69E-01	1.69E-03	MROH5	3.91E-02	1.00E+00
ZSWIM9	-5.74E-01	1.69E-03	RAB11A	-8.75E-03	1.00E+00
C9H19orf38	5.46E-01	1.71E-03	PPM1J	4.54E-02	1.00E+00
SLMAP	-3.20E-01	1.73E-03	LOC112578654	-4.57E-02	1.00E+00
ATP6V1G2	6.93E-01	1.75E-03	PROX1	2.93E-02	1.00E+00
LOC102398191	-1.20E+00	1.75E-03	ERO1B	1.56E-02	1.00E+00
DAD1	-4.72E-01	1.76E-03	CCDC155	-3.56E-02	1.00E+00
SLC19A3	1.44E+00	1.77E-03	GEMIN4	-8.56E-03	1.00E+00
ERBB4	-6.76E-01	1.77E-03	KCND3	3.15E-02	1.00E+00
EXOSC4	-8.83E-01	1.78E-03	MBD2	-1.15E-02	1.00E+00
ABHD15	-1.16E+00	1.80E-03	A4GNT	-3.02E-02	1.00E+00
PET117	-4.96E-01	1.80E-03	SNAPC3	1.14E-02	1.00E+00
CACNA2D3	-9.85E-01	1.81E-03	PLXNC1	-9.23E-03	1.00E+00

LOC102404194	-2.79E+00	1.82E-03	EIF2A	-1.10E-02	1.00E+00		
LACTB2	4.23E-01	1.82E-03	IL1RAPL2	1.19E-02	1.00E+00		
LOC102416201	5.08E-01	1.82E-03	DERL2	8.02E-03	1.00E+00		
NECTIN4	8.68E-01	1.82E-03	PRRT1B	-1.07E-02	1.00E+00		
ZC3HC1	3.32E-01	1.82E-03	LMX1A	-2.76E-02	1.00E+00		
VIT	1.71E+00	1.83E-03	COL9A2	-5.29E-02	1.00E+00		
ZNF132	4.08E-01	1.84E-03	SLC4A3	4.55E-02	1.00E+00		
GMCL1	3.59E-01	1.85E-03	CCDC175	3.47E-02	1.00E+00		
SIT1	-1.20E+00	1.86E-03	FOXJ2	-1.36E-02	1.00E+00		
APOA4	9.93E-01	1.87E-03	LZTS1	5.13E-02	1.00E+00		
L3MBTL2	4.04E-01	1.87E-03	RUNDC1	1.23E-02	1.00E+00		
TNFAIP8L2	-1.52E+00	1.88E-03	CD36	1.74E-02	1.00E+00		
CMAS	2.58E-01	1.88E-03	NOM1	9.52E-03	1.00E+00		
TXNL4A	-4.96E-01	1.89E-03	ZNRF2	1.19E-02	1.00E+00		
LYRM7	4.59E-01	1.90E-03	DUSP12	8.95E-03	1.00E+00		
CDK19	-4.12E-01	1.90E-03	KCNMB3	3.95E-02	1.00E+00		
LOC112578157	1.60E+00	1.93E-03	KCNC4	-1.36E-02	1.00E+00		
CSNK2B	4.07E-01	1.93E-03	LOC102416276	-3.97E-02	1.00E+00		
GPS1	-5.16E-01	1.93E-03	F2R	3.11E-02	1.00E+00		
LOC102407273	1.12E+00	1.95E-03	KANSL3	-8.20E-03	1.00E+00		
RWDD2B	2.89E-01	1.97E-03	PTPN21	1.77E-02	1.00E+00		
ETV3	-5.66E-01	1.97E-03	SORCS3	8.41E-03	1.00E+00		
OPCML	1.56E+00	1.98E-03	ILK	-1.34E-02	1.00E+00		
PLCXD3	1.58E+00	2.00E-03	UBE3D	-9.51E-03	1.00E+00		
MCOLN2	2.32E+00	2.00E-03	RNF149	-2.81E-02	1.00E+00		
LIPH	7.35E-01	2.00E-03	GTSF1	7.76E-03	1.00E+00		
FAM213A	-4.23E-01	2.01E-03	LOC102393117	1.65E-02	1.00E+00		
CNTNAP4	-8.76E-01	2.02E-03	GRHL1	2.06E-02	1.00E+00		
ERN1	-4.68E-01	2.03E-03	REEP6	2.63E-02	1.00E+00		
NFATC3	4.59E-01	2.03E-03	CCNA2	-8.19E-03	1.00E+00		
TSSK3	-1.22E+00	2.04E-03	PNPT1	9.82E-03	1.00E+00		
NEU3	-7.42E-01	2.06E-03	LOC112580468	1.53E-02	1.00E+00		
LOC102408963	-3.12E+00	2.07E-03	NMUR2	-1.83E-02	1.00E+00		
LOC102397524	-7.08E-01	2.07E-03	GRPEL1	7.02E-03	1.00E+00		
ZNF385A	5.68E-01	2.08E-03	SON	1.23E-02	1.00E+00		
ATP8B1	-7.08E-01	2.08E-03	RCHY1	-1.54E-02	1.00E+00		

TOR1A	3.71E-01		2.09E-03	RMDN1	9.36E-03	1.00E+00		
LOC102409942	5.42E-01		2.09E-03	LSM12	-1.18E-02	1.00E+00		
SLC22A20P	-9.29E-01		2.09E-03	DIRC2	-2.01E-02	1.00E+00		
GGA1	2.98E-01		2.10E-03	LOC112579895	-5.15E-02	1.00E+00		
CCDC57	4.01E-01		2.10E-03	LDOC1	-3.19E-02	1.00E+00		
BMPR1B	-4.46E-01		2.11E-03	MYRIP	2.71E-02	1.00E+00		
ZPBP	5.86E-01		2.12E-03	NFATC1	1.41E-02	1.00E+00		
SHANK2	7.59E-01		2.16E-03	SSPN	6.58E-02	1.00E+00		
LOC112578487	-9.19E-01		2.17E-03	ATRIP	1.16E-02	1.00E+00		
TCF7L1	7.73E-01		2.17E-03	CCDC9B	2.30E-02	1.00E+00		
SMYD3	4.00E-01		2.18E-03	LOC112582939	5.24E-02	1.00E+00		
NCAM2	1.56E+00		2.18E-03	ECI2	-9.05E-03	1.00E+00		
SNAP91	4.15E-01		2.18E-03	LOC102400049	4.89E-02	1.00E+00		
LOC102408084	8.55E-01		2.19E-03	SNX12	8.18E-03	1.00E+00		
TMEM38A	-1.06E+00		2.19E-03	UNC119	9.63E-03	1.00E+00		
TMSB4X	-6.12E-01		2.19E-03	LOC112583727	2.00E-02	1.00E+00		
THSD7A	-8.55E-01		2.20E-03	LRRIQ3	1.71E-02	1.00E+00		
LOC112584678	1.38E+00		2.21E-03	YME1L1	6.90E-03	1.00E+00		
MYOZ1	6.16E-01		2.22E-03	PEX3	9.13E-03	1.00E+00		
RCHY1	4.11E-01		2.22E-03	PARPBP	1.47E-02	1.00E+00		
FAM199X	7.29E-01		2.23E-03	CDR2L	-1.51E-02	1.00E+00		
TRIM11	3.24E-01		2.24E-03	LOC102396011	-3.52E-02	1.00E+00		
SKAP2	4.62E-01		2.24E-03	SRSF4	7.84E-03	1.00E+00		
PIN4	5.06E-01		2.25E-03	RPS6KB1	-7.34E-03	1.00E+00		
NAGK	4.25E-01		2.25E-03	EDARADD	9.32E-03	1.00E+00		
ZC3H18	2.66E-01		2.27E-03	LOC102409906	3.87E-02	1.00E+00		
HAS1	-1.22E+00		2.28E-03	NEIL2	-7.21E-03	1.00E+00		
LOC102409438	-2.34E+00		2.29E-03	FAM161B	1.52E-02	1.00E+00		
LOC112586949	1.52E+00		2.30E-03	LOC102408196	1.32E-02	1.00E+00		
OXA1L	2.90E-01		2.31E-03	PEA15	-8.75E-03	1.00E+00		
ADAT1	5.76E-01		2.35E-03	TRPC1	4.14E-02	1.00E+00		
LOC112583788	-8.21E-01		2.35E-03	DPYSL3	-2.59E-02	1.00E+00		
LPXN	-2.31E+00		2.36E-03	DLGAP5	7.63E-03	1.00E+00		
MEIS3	1.04E+00		2.38E-03	CWC27	-8.39E-03	1.00E+00		
LOC112583961	9.78E-01		2.38E-03	HEATR3	-1.74E-02	1.00E+00		
NRCAM	-1.13E+00		2.40E-03	LOC112586819	-1.10E-02	1.00E+00		
HADH	-3.47E-01		2.42E-03	LOC112583711	-1.45E-02	1.00E+00		

EIPR1	-4.66E-01	2.43E-03	SLC49A3	-1.20E-02	1.00E+00		
EPS15L1	2.73E-01	2.44E-03	LRRN4CL	1.47E-02	1.00E+00		
LOC112581440	-9.07E-01	2.46E-03	GPR156	1.01E-02	1.00E+00		
HFM1	-6.25E-01	2.47E-03	TRAF3	6.42E-03	1.00E+00		
LOC112579694	-1.08E+00	2.48E-03	INO80E	6.46E-03	1.00E+00		
CDKL3	9.88E-01	2.49E-03	LOC102401727	1.05E-02	1.00E+00		
ZCCHC4	-3.91E-01	2.49E-03	SCML2	1.37E-02	1.00E+00		
RPS6KA6	-4.78E-01	2.49E-03	RAD51D	-1.06E-02	1.00E+00		
SEZ6L	1.03E+00	2.50E-03	BRPF1	1.08E-02	1.00E+00		
MAMDC4	-1.06E+00	2.50E-03	IRAK1	9.41E-03	1.00E+00		
LOC102399577	-7.73E-01	2.51E-03	FADS2	-3.00E-02	1.00E+00		
TAF4	-5.22E-01	2.52E-03	RALGPS1	-1.72E-02	1.00E+00		
LOC112581555	2.08E+00	2.53E-03	ETFRF1	-1.73E-02	1.00E+00		
GABPB1	3.04E-01	2.56E-03	RHOT2	9.13E-03	1.00E+00		
SSRP1	-3.64E-01	2.56E-03	UBE2D3	-8.52E-03	1.00E+00		
INAFM2	-5.05E-01	2.58E-03	LCORL	-1.37E-02	1.00E+00		
VAT1	-4.50E-01	2.60E-03	C1H3orf38	-7.37E-03	1.00E+00		
ST8SIA5	1.13E+00	2.60E-03	CARMIL2	7.59E-03	1.00E+00		
UBE2E3	-3.21E-01	2.61E-03	CTNND2	1.51E-02	1.00E+00		
LOC102415899	-3.01E+00	2.67E-03	CUTC	-1.14E-02	1.00E+00		
SMAP2	5.41E-01	2.67E-03	DSCC1	-1.17E-02	1.00E+00		
LOC112579067	1.36E+00	2.67E-03	PSMA5	-7.14E-03	1.00E+00		
HMG20A	-2.67E-01	2.70E-03	NCF2	-9.98E-03	1.00E+00		
PKP2	-4.67E-01	2.70E-03	FLYWCH2	-3.03E-02	1.00E+00		
LOC112584644	9.91E-01	2.71E-03	LOC102392023	2.32E-02	1.00E+00		
TOPORS	-4.81E-01	2.71E-03	PACSIN1	2.30E-02	1.00E+00		
LOC112587527	8.92E-01	2.74E-03	CCDC191	1.16E-02	1.00E+00		
TRMT10B	-4.18E-01	2.75E-03	GNG10	-1.10E-02	1.00E+00		
SUGCT	5.23E-01	2.76E-03	LOC102410635	3.26E-02	1.00E+00		
C17H4orf51	1.50E+00	2.78E-03	LOC112587015	4.64E-02	1.00E+00		
LOC102399587	1.36E+00	2.81E-03	HADHA	-6.61E-03	1.00E+00		
SPCS1	3.70E-01	2.81E-03	GPR153	2.48E-02	1.00E+00		
OVCA2	-4.49E-01	2.82E-03	DYDC2	1.36E-02	1.00E+00		
CAB39L	-4.56E-01	2.82E-03	ISCU	-8.46E-03	1.00E+00		
PDE8B	-3.92E-01	2.83E-03	TMEM177	2.90E-02	1.00E+00		
ABCA13	1.37E+00	2.83E-03	SETDB2	-9.18E-	1.00E+00		

				03	
HELZ	-4.45E-01	2.84E-03	ST7	1.37E-02	1.00E+00
SLC35F3	-2.06E+00	2.84E-03	RIBC2	-1.95E-02	1.00E+00
SGCZ	-1.81E+00	2.84E-03	CNOT6	1.25E-02	1.00E+00
WNT9B	6.19E-01	2.85E-03	SOWAHB	-1.79E-02	1.00E+00
UVSSA	-8.44E-01	2.85E-03	EBNA1BP2	8.53E-03	1.00E+00
MRPL13	4.35E-01	2.87E-03	PHB	-7.40E-03	1.00E+00
ATRN	-3.51E-01	2.93E-03	LOC102415442	-9.70E-03	1.00E+00
RPS6KA3	3.69E-01	2.94E-03	USP27X	9.02E-03	1.00E+00
ATP2B2	1.18E+00	2.95E-03	HLX	-1.71E-02	1.00E+00
LOC102411637	-1.38E+00	2.97E-03	SUV39H1	-9.12E-03	1.00E+00
YAF2	-4.26E-01	2.98E-03	CEP78	-1.15E-02	1.00E+00
ANKRD37	7.33E-01	2.99E-03	CTTNBP2NL	-2.67E-02	1.00E+00
SERAC1	4.67E-01	3.02E-03	ERP44	6.56E-03	1.00E+00
NEK10	-6.94E-01	3.02E-03	ZNF214	-3.02E-02	1.00E+00
LOC102409111	-1.29E+00	3.02E-03	TSPAN17	1.16E-02	1.00E+00
LOC112581414	1.34E+00	3.03E-03	CCND3	-1.07E-02	1.00E+00
SLC25A17	3.40E-01	3.03E-03	POGLUT1	3.24E-02	1.00E+00
RCCD1	-5.09E-01	3.03E-03	F11R	-1.22E-02	1.00E+00
MRPS10	2.92E-01	3.04E-03	CENPW	-8.36E-03	1.00E+00
FN3KRP	-4.85E-01	3.06E-03	IKZF2	1.95E-02	1.00E+00
MED30	3.54E-01	3.06E-03	EPN1	-7.19E-03	1.00E+00
ELP4	3.75E-01	3.06E-03	TMEM63A	1.43E-02	1.00E+00
FLNC	-7.87E-01	3.10E-03	CCDC54	2.21E-02	1.00E+00
C3H17orf75	-3.55E-01	3.11E-03	IPP	8.76E-03	1.00E+00
CAPRIN2	4.78E-01	3.12E-03	TPM1	2.24E-02	1.00E+00
TMEM186	5.10E-01	3.14E-03	E2F8	-1.42E-02	1.00E+00
C16H11orf96	7.75E-01	3.17E-03	SESN2	-7.99E-03	1.00E+00
ZNF599	-6.87E-01	3.18E-03	MRPL43	-9.21E-03	1.00E+00
ASNS	-3.29E-01	3.19E-03	PHACTR1	-7.18E-03	1.00E+00
GNL1	-3.01E-01	3.19E-03	GRK3	1.09E-02	1.00E+00
GRM5	1.57E+00	3.21E-03	CCS	1.18E-02	1.00E+00
STPG1	1.30E+00	3.21E-03	MPV17	-2.01E-02	1.00E+00
LOC112586663	-7.17E-01	3.23E-03	RAB7A	7.09E-03	1.00E+00
SYT17	3.81E-01	3.24E-03	HPRT1	-7.55E-	1.00E+00

				03	
APEX2	-3.96E-01	3.25E-03	RASGEF1C	-2.13E-02	1.00E+00
SURF2	-4.44E-01	3.26E-03	GDPD1	-1.54E-02	1.00E+00
TTC12	-5.57E-01	3.28E-03	CFAP126	-3.01E-02	1.00E+00
LOC102409978	-1.06E+00	3.30E-03	TBX4	3.00E-02	1.00E+00
LOC112586645	-1.00E+00	3.30E-03	RBFA	1.50E-02	1.00E+00
MRPL10	3.53E-01	3.32E-03	ZSCAN30	-3.19E-02	1.00E+00
CKAP2L	4.27E-01	3.33E-03	ESR1	-5.86E-02	1.00E+00
PTPN9	3.02E-01	3.33E-03	TMEM108	-1.23E-02	1.00E+00
GPATCH2L	-3.62E-01	3.33E-03	LOC102392263	-1.35E-02	1.00E+00
MBP	-2.15E+00	3.35E-03	NUP62CL	2.15E-02	1.00E+00
KIF6	-5.13E-01	3.37E-03	SLC25A14	-9.23E-03	1.00E+00
CDH18	-1.25E+00	3.38E-03	INSC	-1.86E-02	1.00E+00
OSGEPL1	-4.98E-01	3.39E-03	PRR19	-8.62E-03	1.00E+00
EXT1	3.55E-01	3.40E-03	MCM8	-1.38E-02	1.00E+00
MNAT1	3.06E-01	3.40E-03	RPGRIP1L	-8.58E-03	1.00E+00
KCNK12	1.29E+00	3.41E-03	CAT	9.98E-03	1.00E+00
SPTLC2	2.84E-01	3.46E-03	B3GAT3	1.58E-02	1.00E+00
ENG	-8.44E-01	3.47E-03	IGHMBP2	7.69E-03	1.00E+00
SYT3	-9.34E-01	3.47E-03	NAPEPLD	-8.07E-03	1.00E+00
FSIP1	1.06E+00	3.49E-03	MED13L	-9.90E-03	1.00E+00
PMAIP1	8.26E-01	3.52E-03	LOC112581847	1.16E-02	1.00E+00
LOC102395792	-3.57E-01	3.53E-03	HFM1	2.00E-02	1.00E+00
LOC112580817	9.60E-01	3.53E-03	RHOT1	-7.57E-03	1.00E+00
IRF5	-1.29E+00	3.57E-03	VAMP2	1.31E-02	1.00E+00
CAPN3	1.02E+00	3.58E-03	LOC102407046	1.10E-02	1.00E+00
STK16	4.29E-01	3.58E-03	TMEM117	-1.34E-02	1.00E+00
SPINT1	-1.16E+00	3.58E-03	LOC102413316	2.17E-02	1.00E+00
PTGFR	-1.57E+00	3.58E-03	B3GALT1	1.26E-02	1.00E+00
FAM162A	-4.95E-01	3.61E-03	COQ5	1.19E-02	1.00E+00
RTN4RL2	1.26E+00	3.61E-03	LOC102393808	-2.56E-02	1.00E+00
RELB	-6.08E-01	3.62E-03	DERA	-3.20E-02	1.00E+00
ZNF770	-5.42E-01	3.63E-03	LOC102414462	-2.19E-02	1.00E+00
RFESD	-5.93E-01	3.67E-03	ARID2	1.64E-02	1.00E+00

AGBL4	-1.44E+00	3.67E-03	LOC112578039	-3.73E-02	1.00E+00
CFAP221	-9.86E-01	3.67E-03	NIPAL2	-1.11E-02	1.00E+00
TMEM185A	2.89E-01	3.70E-03	PLCH1	-7.88E-03	1.00E+00
KCNK10	1.72E+00	3.71E-03	ADD2	-1.75E-02	1.00E+00
LOC102392650	-1.87E+00	3.74E-03	LOC112581378	2.86E-02	1.00E+00
MANBAL	3.70E-01	3.76E-03	GNAI2	-6.34E-03	1.00E+00
PIBF1	-4.58E-01	3.76E-03	COP1	-8.61E-03	1.00E+00
LOC102407787	4.68E-01	3.77E-03	ATE1	6.74E-03	1.00E+00
LOC102402292	-1.06E+00	3.77E-03	LOC102410885	2.97E-02	1.00E+00
LOC102408905	1.30E+00	3.78E-03	LOC112580392	-3.05E-02	1.00E+00
BBS10	-6.69E-01	3.79E-03	NT5M	1.55E-02	1.00E+00
LOC112582098	-1.32E+00	3.82E-03	NCKAP5	-7.81E-03	1.00E+00
LTBP4	-5.41E-01	3.82E-03	TSFM	-8.39E-03	1.00E+00
SLC6A15	8.14E-01	3.83E-03	E2F3	-2.43E-02	1.00E+00
LOC112579692	1.40E+00	3.84E-03	DNAJC11	7.32E-03	1.00E+00
ALDH1L2	-4.96E-01	3.84E-03	GSK3A	-7.66E-03	1.00E+00
NHP2	-4.77E-01	3.89E-03	TMEM170A	-2.18E-02	1.00E+00
SLC25A5	3.40E-01	3.89E-03	LOC102398414	-2.18E-02	1.00E+00
NUP205	-2.93E-01	3.92E-03	DNAJB12	7.04E-03	1.00E+00
MUSK	-5.69E-01	3.99E-03	RHOG	1.15E-02	1.00E+00
IP6K1	2.65E-01	4.01E-03	GYG1	-7.47E-03	1.00E+00
CTTN	-2.87E-01	4.04E-03	IBA57	1.08E-02	1.00E+00
SMARCA5	3.49E-01	4.11E-03	LOC102414436	3.56E-02	1.00E+00
BAZ2A	-4.17E-01	4.15E-03	HIF1A	-1.35E-02	1.00E+00
IGLON5	-1.17E+00	4.15E-03	KLHL9	-1.52E-02	1.00E+00
C3H17orf80	-4.23E-01	4.15E-03	TMEM179	-1.13E-02	1.00E+00
SLC9C2	-1.01E+00	4.17E-03	GPR1	-1.55E-02	1.00E+00
ABCC11	1.92E+00	4.19E-03	NNT	6.83E-03	1.00E+00
LOC102393901	2.68E-01	4.20E-03	LOC112583729	4.47E-02	1.00E+00
SPRTN	3.88E-01	4.22E-03	LOC112587459	-2.87E-02	1.00E+00
RGS20	9.71E-01	4.27E-03	FSIP1	-2.11E-02	1.00E+00
COPA	-3.12E-01	4.27E-03	LOC112578666	2.70E-02	1.00E+00
LOC102404511	-8.29E-01	4.31E-03	COPG2	6.28E-03	1.00E+00
MKNK2	-2.87E-01	4.32E-03	LOC102405198	-1.34E-02	1.00E+00

FAM126A	-4.88E-01	4.32E-03	PECR	1.02E-02	1.00E+00
PCDHB8	-1.28E+00	4.32E-03	ARHGAP22	9.39E-03	1.00E+00
LOC112585097	1.25E+00	4.32E-03	ZNF516	-3.13E-02	1.00E+00
OGT	-4.82E-01	4.35E-03	MDFI	3.06E-02	1.00E+00
ADAR	3.86E-01	4.36E-03	C17H22orf39	1.11E-02	1.00E+00
MYO1F	-1.79E+00	4.36E-03	RSPO1	1.13E-02	1.00E+00
FAU	-5.80E-01	4.39E-03	SKIV2L	7.81E-03	1.00E+00
LOC112582945	5.91E-01	4.39E-03	FAM83A	-1.65E-02	1.00E+00
KIF5B	-4.14E-01	4.40E-03	LOC112579660	-2.69E-02	1.00E+00
TSGA10	-5.03E-01	4.41E-03	C9H19orf38	-1.51E-02	1.00E+00
LOC112585568	7.46E-01	4.43E-03	SPNS3	-5.97E-03	1.00E+00
TBL3	-3.97E-01	4.50E-03	GOSR1	7.62E-03	1.00E+00
GNB3	6.65E-01	4.58E-03	SLC17A8	-2.32E-02	1.00E+00
UBE3C	2.88E-01	4.68E-03	ICE2	-1.19E-02	1.00E+00
CASTOR2	2.64E-01	4.68E-03	DRG2	-7.50E-03	1.00E+00
MRPL43	3.47E-01	4.74E-03	ZSWIM9	1.45E-02	1.00E+00
LOC112584706	7.46E-01	4.76E-03	KIF24	7.24E-03	1.00E+00
COG7	-2.99E-01	4.76E-03	C9H19orf67	-6.81E-03	1.00E+00
PGAP1	-4.11E-01	4.79E-03	SGO2	-1.01E-02	1.00E+00
CFAP299	1.37E+00	4.79E-03	GTF2IRD1	1.47E-02	1.00E+00
PARPBP	4.97E-01	4.80E-03	LOC112587480	-1.83E-02	1.00E+00
SLC52A2	-3.60E-01	4.81E-03	TAF10	-8.45E-03	1.00E+00
LOC102389632	-6.23E-01	4.84E-03	SUSD3	1.27E-02	1.00E+00
TATDN1	-3.85E-01	4.85E-03	NFU1	-8.24E-03	1.00E+00
CNOT10	-3.17E-01	4.88E-03	ZNF770	-1.78E-02	1.00E+00
PSEN2	-3.85E-01	4.89E-03	FAM47E	1.33E-02	1.00E+00
ENPP2	5.88E-01	4.90E-03	MED28	-1.41E-02	1.00E+00
LOC102409010	4.39E-01	4.91E-03	SMARCD3	-1.85E-02	1.00E+00
STK40	3.18E-01	4.92E-03	TRPM5	-2.53E-02	1.00E+00
SLC28A1	8.09E-01	4.93E-03	KCNQ1	-7.61E-03	1.00E+00
ZBTB22	3.50E-01	4.94E-03	IRAK1BP1	2.25E-02	1.00E+00
GNPAT	-2.44E-01	4.94E-03	SERINC4	3.36E-02	1.00E+00
HERC5	6.54E-01	4.95E-03	KIAA0586	-8.22E-03	1.00E+00
PIAS3	-3.87E-01	4.98E-03	PCMTD1	6.65E-03	1.00E+00

CNTN4	7.66E-01	5.01E-03	ASAP3	1.77E-02	1.00E+00		
SGSM1	-5.24E-01	5.02E-03	ATP8A1	-7.82E-03	1.00E+00		
LOC102397215	-6.09E-01	5.03E-03	CCDC60	-7.76E-03	1.00E+00		
DVL2	2.86E-01	5.05E-03	AUNIP	-6.07E-03	1.00E+00		
SAP30	3.73E-01	5.06E-03	KCMF1	-6.51E-03	1.00E+00		
LOC112578881	-1.51E+00	5.06E-03	LOC102412967	-1.27E-02	1.00E+00		
TBX19	3.82E-01	5.13E-03	CMAS	-6.04E-03	1.00E+00		
PRDM15	-4.77E-01	5.14E-03	DHX40	6.01E-03	1.00E+00		
CTTNBP2NL	-6.12E-01	5.21E-03	SDE2	7.36E-03	1.00E+00		
LOC102401619	-1.12E+00	5.26E-03	SPECC1L	5.90E-03	1.00E+00		
RGR	-6.66E-01	5.26E-03	PRAF2	-1.38E-02	1.00E+00		
BICD1	5.05E-01	5.34E-03	LOC112579226	4.57E-02	1.00E+00		
CLINT1	3.52E-01	5.40E-03	CDH18	-3.35E-02	1.00E+00		
SOS1	-4.65E-01	5.40E-03	LOC112585223	3.17E-02	1.00E+00		
LOC112578005	-6.28E-01	5.43E-03	TGFA	9.20E-03	1.00E+00		
ZNF584	-5.03E-01	5.46E-03	FBXL12	-1.36E-02	1.00E+00		
ZSWIM8	3.36E-01	5.47E-03	COL4A4	1.77E-02	1.00E+00		
RUVBL2	4.43E-01	5.50E-03	LOC112580375	2.84E-02	1.00E+00		
INTS3	2.90E-01	5.54E-03	PLAUR	-6.31E-03	1.00E+00		
STRN3	3.16E-01	5.54E-03	PLEKHH1	-1.54E-02	1.00E+00		
LOC102399127	1.02E+00	5.56E-03	RPL26L1	-7.50E-03	1.00E+00		
MAPK3	3.10E-01	5.56E-03	CCDC189	3.16E-02	1.00E+00		
MYNN	-3.23E-01	5.58E-03	RNFT1	2.14E-02	1.00E+00		
DICER1	-3.49E-01	5.58E-03	IMPA1	7.47E-03	1.00E+00		
LOC112577635	-6.57E-01	5.59E-03	CNDP2	-7.49E-03	1.00E+00		
RAB30	2.47E-01	5.62E-03	LOC112579701	-2.22E-02	1.00E+00		
CPOX	4.09E-01	5.70E-03	BORA	7.22E-03	1.00E+00		
LOC102403233	2.53E-01	5.75E-03	CSRNP1	2.01E-02	1.00E+00		
GFER	-4.64E-01	5.76E-03	SERTAD3	-1.17E-02	1.00E+00		
PRPF40A	2.43E-01	5.77E-03	GALT	7.72E-03	1.00E+00		
MED29	-5.28E-01	5.78E-03	ENOSF1	1.70E-02	1.00E+00		
BAP1	-2.60E-01	5.79E-03	CACNA1F	-1.16E-02	1.00E+00		
LOC102401181	-3.19E-01	5.83E-03	AMMECR1L	8.34E-03	1.00E+00		
TIGAR	3.67E-01	5.83E-03	VPS26A	-2.44E-02	1.00E+00		
SPTLC1	-2.81E-01	5.90E-03	FAM133A	-2.56E-02	1.00E+00		

CFAP77	1.26E+00	5.91E-03	LOC102391810	1.66E-02	1.00E+00
FIGN	-5.50E-01	5.94E-03	LOC102415609	-7.35E-03	1.00E+00
GAB1	5.38E-01	5.98E-03	MXD4	9.87E-03	1.00E+00
IL16	-1.07E+00	5.98E-03	FAM81A	7.33E-03	1.00E+00
PAGE4	1.14E+00	5.98E-03	ATP6V0B	6.64E-03	1.00E+00
HSP90AA1	5.02E-01	6.01E-03	SDSL	-1.15E-02	1.00E+00
LOC112586225	1.49E+00	6.01E-03	LOC102408092	1.87E-02	1.00E+00
SHPK	-9.51E-01	6.03E-03	SULT4A1	-5.58E-03	1.00E+00
EFHD2	-4.31E-01	6.04E-03	LOC112580364	-1.00E-02	1.00E+00
ENDOD1	7.33E-01	6.04E-03	LOC102408946	-2.05E-02	1.00E+00
CTNNBIP1	4.02E-01	6.06E-03	STK39	-5.02E-03	1.00E+00
TC2N	8.74E-01	6.09E-03	NEK4	8.30E-03	1.00E+00
BAZ1A	4.09E-01	6.09E-03	PTER	4.14E-02	1.00E+00
RAC2	-1.41E+00	6.13E-03	CAVIN3	3.33E-02	1.00E+00
SCIN	6.52E-01	6.13E-03	LOC102395341	8.15E-03	1.00E+00
FBXO42	3.37E-01	6.14E-03	IGSF10	-1.55E-02	1.00E+00
LRRC74B	-9.28E-01	6.14E-03	ZBTB24	1.81E-02	1.00E+00
LOC112579184	7.47E-01	6.14E-03	LOC102407762	-2.25E-02	1.00E+00
NFU1	-3.53E-01	6.15E-03	LOC102400719	-2.48E-02	1.00E+00
PDZD2	3.30E-01	6.17E-03	DLC1	3.26E-02	1.00E+00
TREX2	-6.39E-01	6.24E-03	LOC102410721	9.05E-03	1.00E+00
RBX1	2.98E-01	6.29E-03	CNOT11	7.97E-03	1.00E+00
LOC102416080	1.39E+00	6.32E-03	LOC102414288	8.67E-03	1.00E+00
EP300	4.81E-01	6.34E-03	CHAF1B	-6.83E-03	1.00E+00
STX4	-4.15E-01	6.37E-03	SKOR1	6.73E-03	1.00E+00
UIMC1	-2.53E-01	6.37E-03	RIOK1	6.58E-03	1.00E+00
LOC102410549	4.99E-01	6.38E-03	STK32A	-1.24E-02	1.00E+00
CNTN5	1.18E+00	6.45E-03	LOC112581035	1.95E-02	1.00E+00
LOC102407011	-6.21E-01	6.50E-03	CBX4	-8.08E-03	1.00E+00
LOC102402993	-1.44E+00	6.52E-03	CTR9	6.86E-03	1.00E+00
TECPR1	3.59E-01	6.53E-03	JMJD4	-9.49E-03	1.00E+00
DSCC1	-3.36E-01	6.60E-03	OVCA2	-9.41E-03	1.00E+00
SPOPL	-6.56E-01	6.64E-03	ENDOG	-8.70E-03	1.00E+00
LYPLAL1	4.24E-01	6.70E-03	LYST	-9.44E-03	1.00E+00
CDC42EP4	-3.77E-01	6.70E-03	LZTS2	7.48E-03	1.00E+00
APIP	4.18E-01	6.73E-03	LOC102411804	-6.30E-03	1.00E+00

CAMKMT	-3.73E-01	6.77E-03	RAB11FIP1	2.55E-02	1.00E+00		
XRN2	2.62E-01	6.81E-03	LOC112578483	1.87E-02	1.00E+00		
PCBP3	-8.32E-01	6.83E-03	PDE8B	-7.75E-03	1.00E+00		
TWISTNB	4.05E-01	6.87E-03	UFSP2	-1.23E-02	1.00E+00		
CORO2A	1.02E+00	6.90E-03	CIDEB	-2.23E-02	1.00E+00		
FUT8	-9.86E-01	6.91E-03	ACAD8	1.06E-02	1.00E+00		
NOL7	-3.52E-01	6.92E-03	HNRNPF	-5.12E-03	1.00E+00		
METTL22	4.06E-01	6.94E-03	ZGLP1	-6.47E-03	1.00E+00		
PAQR3	-3.44E-01	6.95E-03	FUNDC1	1.47E-02	1.00E+00		
ZNF300	4.80E-01	6.98E-03	OGFOD1	-7.20E-03	1.00E+00		
HRH2	-1.48E+00	7.02E-03	AHDC1	1.70E-02	1.00E+00		
SPTAN1	3.79E-01	7.02E-03	IARS	-6.10E-03	1.00E+00		
CHMP4C	7.28E-01	7.03E-03	CMC4	1.10E-02	1.00E+00		
PGPEP1L	1.28E+00	7.06E-03	SDHB	1.71E-02	1.00E+00		
NTHL1	-4.99E-01	7.10E-03	CACNA2D1	-5.62E-03	1.00E+00		
LOC102404909	3.98E-01	7.11E-03	APTX	-7.74E-03	1.00E+00		
ZNF684	6.52E-01	7.16E-03	LOC102401393	-6.36E-03	1.00E+00		
SNRPG	-4.46E-01	7.17E-03	U2AF1L4	-8.12E-03	1.00E+00		
IKBKB	3.09E-01	7.19E-03	MSTO1	-7.61E-03	1.00E+00		
ATF7	-3.40E-01	7.25E-03	DCAF17	-1.09E-02	1.00E+00		
CCDC191	-3.06E-01	7.26E-03	PIK3CD	6.46E-03	1.00E+00		
MRPS28	-3.57E-01	7.27E-03	DMWD	5.06E-03	1.00E+00		
PKD1L2	3.14E-01	7.28E-03	KPNA4	1.20E-02	1.00E+00		
GABRB1	-1.70E+00	7.30E-03	SBF2	-5.70E-03	1.00E+00		
ACOT6	8.99E-01	7.32E-03	MNS1	-5.90E-03	1.00E+00		
JCHAIN	8.08E-01	7.34E-03	HRAS	6.82E-03	1.00E+00		
LOC102409355	4.56E-01	7.34E-03	DNPEP	-6.83E-03	1.00E+00		
TNKS	3.34E-01	7.39E-03	PRSS45	1.71E-02	1.00E+00		
LOC102403265	-5.22E-01	7.40E-03	NUP88	-4.52E-03	1.00E+00		
ITCH	3.74E-01	7.40E-03	TPST2	7.00E-03	1.00E+00		
PECR	-3.54E-01	7.44E-03	BLOC1S4	-2.74E-02	1.00E+00		
ZBTB34	-7.61E-01	7.50E-03	CPNE4	-1.20E-02	1.00E+00		
GIF	1.31E+00	7.53E-03	SETD9	1.21E-02	1.00E+00		
TMEM230	-2.54E-01	7.55E-03	LOC102416634	8.94E-03	1.00E+00		
UBXN8	-3.42E-01	7.55E-03	ATAD1	-7.95E-03	1.00E+00		

LBH	6.29E-01	7.55E-03	CCDC172	1.02E-02	1.00E+00
VPS26C	-2.81E-01	7.55E-03	MBNL3	-5.87E-03	1.00E+00
TAF11	3.14E-01	7.56E-03	SCARB1	2.99E-02	1.00E+00
NDUFB1	-4.42E-01	7.57E-03	LOC112585146	2.03E-02	1.00E+00
ABHD17A	3.43E-01	7.64E-03	LOC102404930	-7.09E-03	1.00E+00
RAD9A	3.28E-01	7.65E-03	CHMP4C	2.13E-02	1.00E+00
GPI	4.22E-01	7.73E-03	ZZEF1	-1.04E-02	1.00E+00
SOAT1	2.98E-01	7.80E-03	LOC102397920	1.35E-02	1.00E+00
TBK1	-3.42E-01	7.81E-03	THG1L	6.04E-03	1.00E+00
SH3RF1	-4.07E-01	7.83E-03	SATB2	1.13E-02	1.00E+00
C5H1orf158	1.37E+00	7.87E-03	NKAPL	-1.04E-02	1.00E+00
PNPO	-6.26E-01	7.88E-03	IRX5	-5.86E-03	1.00E+00
LOC102416225	-3.04E+00	7.92E-03	ZNF667	-8.96E-03	1.00E+00
FBXW4	3.52E-01	7.96E-03	LOC102405660	5.92E-03	1.00E+00
LOC102396207	8.52E-01	7.99E-03	MFAP1	-4.67E-03	1.00E+00
STAMBP	2.56E-01	8.02E-03	PSME2	-6.11E-03	1.00E+00
LOC112581371	-1.15E+00	8.04E-03	LOC102389969	1.53E-02	1.00E+00
RAI1	3.01E-01	8.11E-03	LOC102398746	-4.83E-02	1.00E+00
TSTD1	4.36E-01	8.16E-03	RAB3GAP1	-5.91E-03	1.00E+00
CCP110	4.71E-01	8.17E-03	LOC112581036	-1.33E-02	1.00E+00
LOC112578141	1.29E+00	8.18E-03	EIF3L	4.29E-03	1.00E+00
MGAT4D	-1.03E+00	8.18E-03	TRIM25	2.23E-02	1.00E+00
DPY30	-3.72E-01	8.20E-03	TBC1D24	-4.88E-03	1.00E+00
SWAP70	4.02E-01	8.29E-03	BACE2	1.16E-02	1.00E+00
GALT	4.72E-01	8.29E-03	CAMKK1	1.55E-02	1.00E+00
TNFAIP2	-1.19E+00	8.36E-03	RND1	4.86E-03	1.00E+00
XRRA1	4.28E-01	8.42E-03	GAPVD1	6.51E-03	1.00E+00
ATP5PD	-3.98E-01	8.45E-03	KCNJ14	-2.48E-02	1.00E+00
RAP1GAP2	1.18E+00	8.46E-03	KLHL12	4.73E-03	1.00E+00
LOC112584596	6.04E-01	8.48E-03	C16H11orf71	9.80E-03	1.00E+00
DDB1	2.86E-01	8.48E-03	METTL14	4.78E-03	1.00E+00
SIKE1	4.43E-01	8.48E-03	TMEM25	-2.05E-02	1.00E+00
RMI2	-4.72E-01	8.49E-03	LNPEP	-6.01E-03	1.00E+00
CD81	-3.22E-01	8.51E-03	LOC112583906	1.26E-02	1.00E+00
C21H3orf20	4.51E-01	8.74E-03	FAM217A	-1.57E-02	1.00E+00
RB1CC1	2.79E-01	8.75E-03	SLC28A1	-2.00E-02	1.00E+00

SH2B1	2.64E-01	8.75E-03	LOC112585542	-1.37E-02	1.00E+00
LOC112581029	-1.12E+00	8.76E-03	ZNF576	-9.04E-03	1.00E+00
LOC102404139	-6.37E-01	8.78E-03	NAPA	-5.20E-03	1.00E+00
PRKCI	4.98E-01	8.81E-03	LOC102400025	-4.21E-03	1.00E+00
ELL3	-7.03E-01	8.94E-03	CD59	5.54E-03	1.00E+00
VAPB	-2.94E-01	8.95E-03	PPP2CB	-5.39E-03	1.00E+00
MTCH2	2.54E-01	8.99E-03	ACTR3	-6.60E-03	1.00E+00
FLVCR1	-7.01E-01	9.01E-03	CAMK2B	-1.28E-02	1.00E+00
MCRIP2	4.64E-01	9.01E-03	NDUFAF6	8.02E-03	1.00E+00
LOC102399763	-1.45E+00	9.08E-03	SMAD5	-1.19E-02	1.00E+00
MYO1H	5.00E-01	9.09E-03	LOC112584642	-1.23E-02	1.00E+00
PDCD2	2.98E-01	9.12E-03	NDUFB6	7.45E-03	1.00E+00
PSMA6	3.14E-01	9.17E-03	DDX6	7.74E-03	1.00E+00
HAUS4	-3.73E-01	9.17E-03	PDLIM1	4.34E-03	1.00E+00
PRMT8	1.40E+00	9.24E-03	C1QTNF6	-3.12E-02	1.00E+00
SPPL3	4.12E-01	9.27E-03	RIDA	-1.07E-02	1.00E+00
ANO6	-4.29E-01	9.30E-03	PTCD1	-8.00E-03	1.00E+00
RHOBTB2	2.62E-01	9.36E-03	SLC38A3	-1.39E-02	1.00E+00
FH	-2.76E-01	9.36E-03	PCNX4	6.32E-03	1.00E+00
PTPN6	-9.18E-01	9.40E-03	BUD23	-4.68E-03	1.00E+00
ROPN1L	8.57E-01	9.42E-03	EPCAM	6.13E-03	1.00E+00
LOC112586644	1.15E+00	9.61E-03	RBM6	-5.16E-03	1.00E+00
TGFBR2	4.65E-01	9.63E-03	ZMPSTE24	1.57E-02	1.00E+00
FMO5	-2.51E-01	9.64E-03	HLCS	7.27E-03	1.00E+00
LOC112587338	1.49E+00	9.66E-03	ARAP3	1.19E-02	1.00E+00
AP5S1	4.05E-01	9.66E-03	PCBD1	5.07E-03	1.00E+00
LOC102397029	3.33E-01	9.71E-03	ZFHX2	8.57E-03	1.00E+00
PLD5	-1.49E+00	9.71E-03	ESD	-5.17E-03	1.00E+00
ANPEP	1.03E+00	9.75E-03	PRKG1	-5.24E-03	1.00E+00
PSMC1	-3.36E-01	9.79E-03	GPATCH4	-1.05E-02	1.00E+00
CARMIL1	-3.16E-01	9.85E-03	DBR1	7.54E-03	1.00E+00
FBXO47	8.34E-01	9.90E-03	SLC44A3	4.48E-03	1.00E+00
NAA60	-2.76E-01	9.91E-03	LOC112581217	1.68E-02	1.00E+00
ANKDD1A	1.01E+00	9.93E-03	WEE2	-6.26E-03	1.00E+00
BTBD3	4.19E-01	9.93E-03	SFT2D3	-8.94E-03	1.00E+00

NR1H3	4.27E-01	9.93E-03	MICU3	8.68E-03	1.00E+00		
PDIA2	-7.31E-01	9.95E-03	NSFL1C	-4.07E-03	1.00E+00		
SAE1	3.19E-01	9.95E-03	MXD1	1.16E-02	1.00E+00		
FAM151B	-4.00E-01	1.00E-02	IRF9	-1.71E-02	1.00E+00		
SPAAR	-6.62E-01	1.00E-02	LOC112578055	-7.30E-03	1.00E+00		
CFD	-1.07E+00	1.01E-02	MRPL42	6.21E-03	1.00E+00		
IL34	8.09E-01	1.01E-02	PCSK7	6.15E-03	1.00E+00		
LOC102415369	-6.13E-01	1.01E-02	VPS52	7.46E-03	1.00E+00		
C9H19orf24	-5.22E-01	1.01E-02	SMC6	-7.00E-03	1.00E+00		
NEK1	-3.31E-01	1.01E-02	LOC102399902	1.39E-02	1.00E+00		
BCAS4	-5.91E-01	1.02E-02	HIBADH	-4.79E-03	1.00E+00		
TP73	1.00E+00	1.02E-02	FANCB	-9.89E-03	1.00E+00		
LOC102399857	-1.13E+00	1.02E-02	NAA25	9.33E-03	1.00E+00		
LOC112580268	-9.10E-01	1.03E-02	STOX1	-5.57E-03	1.00E+00		
USP31	3.04E-01	1.03E-02	CHML	-1.67E-02	1.00E+00		
TMEM243	-3.69E-01	1.04E-02	ZMAT5	8.64E-03	1.00E+00		
RTN4IP1	-3.18E-01	1.04E-02	NKX2-5	2.59E-02	1.00E+00		
RCOR1	-4.71E-01	1.04E-02	KLHL20	-4.57E-03	1.00E+00		
STMN3	7.16E-01	1.04E-02	DMPK	-1.70E-02	1.00E+00		
LRRC45	-4.01E-01	1.04E-02	HACE1	5.36E-03	1.00E+00		
EFCAB3	1.06E+00	1.05E-02	BTRC	-4.19E-03	1.00E+00		
TNFSF9	-1.33E+00	1.06E-02	CUL4A	-4.10E-03	1.00E+00		
TMEM203	-3.50E-01	1.07E-02	CNTN3	-1.15E-02	1.00E+00		
TECPR2	-2.81E-01	1.07E-02	LOC112581854	2.00E-02	1.00E+00		
LOC102391408	-3.37E-01	1.07E-02	TPP2	6.23E-03	1.00E+00		
HS2ST1	-5.33E-01	1.08E-02	PSEN1	3.84E-03	1.00E+00		
ALPK1	-5.98E-01	1.08E-02	TSEN34	5.61E-03	1.00E+00		
SETD9	4.33E-01	1.09E-02	FAM89A	-9.65E-03	1.00E+00		
RDH5	7.53E-01	1.09E-02	LOC112586919	-1.19E-02	1.00E+00		
IRX1	-1.03E+00	1.09E-02	C21H3orf14	-1.72E-02	1.00E+00		
MYBL1	-4.66E-01	1.10E-02	SLC22A15	-1.05E-02	1.00E+00		
OGFR	3.50E-01	1.10E-02	GPR19	4.34E-03	1.00E+00		
C17H4orf45	1.78E+00	1.10E-02	GRAMD4	-1.30E-02	1.00E+00		
MAPKAPK3	-3.89E-	1.10E-02	NTMT1	7.35E-03	1.00E+00		

	01						
SYT1	5.69E-01	1.10E-02	SMU1	4.60E-03	1.00E+00		
MRM1	-3.48E-01	1.11E-02	OSBPL8	6.48E-03	1.00E+00		
SNRPA	-3.42E-01	1.11E-02	ATN1	-5.62E-03	1.00E+00		
RNF170	-2.84E-01	1.11E-02	BCLAF3	-5.76E-03	1.00E+00		
CCL3	-1.60E+00	1.12E-02	USP33	5.35E-03	1.00E+00		
NOL8	-2.66E-01	1.12E-02	LOC102406326	-1.67E-02	1.00E+00		
NAA40	2.95E-01	1.13E-02	EEPD1	-4.59E-03	1.00E+00		
LOC102395480	-1.28E+00	1.14E-02	LMO4	-4.10E-03	1.00E+00		
LOC102406624	-7.02E-01	1.16E-02	TMEM231	3.93E-03	1.00E+00		
LOC102411164	-4.59E-01	1.16E-02	USP3	-7.03E-03	1.00E+00		
RUFY1	-2.40E-01	1.16E-02	METTL13	-6.89E-03	1.00E+00		
ATP1B3	3.10E-01	1.17E-02	KIF9	9.99E-03	1.00E+00		
NIN	4.52E-01	1.17E-02	SAYSD1	7.42E-03	1.00E+00		
CMC4	-4.70E-01	1.18E-02	EDEM3	-7.45E-03	1.00E+00		
SPAG16	-7.58E-01	1.18E-02	GRTP1	1.87E-02	1.00E+00		
B4GALT3	2.56E-01	1.19E-02	SCD	1.81E-02	1.00E+00		
ARAP3	7.25E-01	1.20E-02	TK1	-4.29E-03	1.00E+00		
CTU1	-5.68E-01	1.20E-02	FAM45A	-6.93E-03	1.00E+00		
SCAF1	2.83E-01	1.20E-02	LOC112586459	2.50E-02	1.00E+00		
TRNAU1AP	-3.06E-01	1.20E-02	DEPDC1	6.61E-03	1.00E+00		
FDXACB1	4.85E-01	1.20E-02	MASP2	-1.41E-02	1.00E+00		
INPP5D	-1.22E+00	1.20E-02	LOC102411613	1.13E-02	1.00E+00		
C24H7orf50	3.58E-01	1.21E-02	SRPK2	-3.90E-03	1.00E+00		
INO80	-3.18E-01	1.22E-02	RELT	3.63E-03	1.00E+00		
CACNB4	1.07E+00	1.22E-02	CSNK2A1	-4.49E-03	1.00E+00		
LOC102397317	-4.92E-01	1.22E-02	RECK	1.32E-02	1.00E+00		
MAPRE1	3.34E-01	1.22E-02	MECR	8.71E-03	1.00E+00		
IFFO2	-7.40E-01	1.23E-02	PCSK1	-9.60E-03	1.00E+00		
GPR88	-9.70E-01	1.23E-02	ANKRD28	7.14E-03	1.00E+00		
LOC102404545	-1.31E+00	1.24E-02	SRSF9	5.16E-03	1.00E+00		
ZFX	-3.35E-01	1.25E-02	LOC102393230	-4.40E-03	1.00E+00		
CDH12	7.00E-01	1.25E-02	EHD4	9.73E-03	1.00E+00		
KIAA1109	-4.08E-01	1.25E-02	UBXN11	-1.10E-02	1.00E+00		
USP9X	-2.81E-01	1.25E-02	TOMM40	-4.48E-03	1.00E+00		

PDZD8	3.40E-01	1.26E-02	HNRNPA1	7.02E-03	1.00E+00		
LOC112587928	4.17E-01	1.26E-02	ZFAND5	3.40E-03	1.00E+00		
LOC102399902	6.50E-01	1.26E-02	ANKRD55	-2.12E-02	1.00E+00		
ERCC6	-2.39E-01	1.26E-02	CCNA1	8.71E-03	1.00E+00		
DMD	4.53E-01	1.27E-02	LOC112578061	1.24E-02	1.00E+00		
CFAP97	3.29E-01	1.27E-02	CDKN2AIPNL	8.45E-03	1.00E+00		
LOC102404627	-1.08E+00	1.29E-02	TPK1	4.21E-03	1.00E+00		
CACNG2	1.71E+00	1.29E-02	RASL10A	-7.81E-03	1.00E+00		
IL7R	1.24E+00	1.30E-02	SNX18	1.02E-02	1.00E+00		
FOXA3	-8.07E-01	1.30E-02	AP1S3	6.89E-03	1.00E+00		
ENOSF1	-4.60E-01	1.30E-02	TENM2	3.75E-03	1.00E+00		
RETREG3	2.06E-01	1.31E-02	ZCCHC14	-8.73E-03	1.00E+00		
ZNF865	-3.49E-01	1.31E-02	GTPBP4	3.54E-03	1.00E+00		
ZNF329	2.74E-01	1.31E-02	NCKIPSD	-5.14E-03	1.00E+00		
C2H6orf89	2.21E-01	1.31E-02	MEN1	3.41E-03	1.00E+00		
HSBP1	2.98E-01	1.32E-02	FLNC	9.09E-03	1.00E+00		
TRIM68	-3.07E-01	1.32E-02	RAB28	4.00E-03	1.00E+00		
UBB	6.30E-01	1.32E-02	CAPN7	4.52E-03	1.00E+00		
LOC102397960	-1.81E+00	1.33E-02	MYADM	-1.71E-02	1.00E+00		
DNAAF2	-3.47E-01	1.34E-02	LOC102401619	2.66E-02	1.00E+00		
PFDN1	3.73E-01	1.34E-02	TTC29	-9.20E-03	1.00E+00		
ABHD10	-3.10E-01	1.34E-02	LMF1	1.47E-02	1.00E+00		
BRI3BP	2.89E-01	1.34E-02	ZNF205	-8.31E-03	1.00E+00		
ATP6V0D1	-2.86E-01	1.35E-02	VAV1	-1.54E-02	1.00E+00		
LOC102406326	6.39E-01	1.35E-02	LOC102411217	1.22E-02	1.00E+00		
DSP	9.33E-01	1.36E-02	SYCE1	-1.43E-02	1.00E+00		
ITPRID2	5.36E-01	1.36E-02	CD164L2	4.11E-03	1.00E+00		
RAB27B	-3.64E-01	1.37E-02	SERPING1	1.60E-02	1.00E+00		
DHX57	2.59E-01	1.38E-02	PAFAH1B1	-3.66E-03	1.00E+00		
TMOD3	4.29E-01	1.38E-02	SEC13	-3.93E-03	1.00E+00		
NEDD8	3.57E-01	1.38E-02	LOC112581125	4.61E-03	1.00E+00		
TSFM	3.20E-01	1.39E-02	NFKB1	7.13E-03	1.00E+00		
STARD8	5.75E-01	1.39E-02	PP2D1	-1.89E-02	1.00E+00		
LOC112580641	-8.02E-01	1.39E-02	METTL9	4.86E-03	1.00E+00		
ADAMTSL3	-1.10E+00	1.39E-02	NPC2	-3.62E-03	1.00E+00		
SLC25A23	3.30E-01	1.39E-02	RHOJ	-1.18E-02	1.00E+00		

LOC112580460	-1.04E+00	1.40E-02	CTPS1	-3.08E-03	1.00E+00
LOC102411048	1.21E+00	1.41E-02	LOC102397660	3.41E-03	1.00E+00
LOC102416336	7.70E-01	1.42E-02	EML3	-7.66E-03	1.00E+00
PDE12	3.76E-01	1.42E-02	DSN1	3.14E-03	1.00E+00
PRKD3	-4.03E-01	1.43E-02	CLASP1	4.71E-03	1.00E+00
GSPT1	-2.29E-01	1.44E-02	USP47	4.52E-03	1.00E+00
UBAP2L	2.99E-01	1.45E-02	LOC112586489	-2.44E-02	1.00E+00
DYNC2LI1	-2.80E-01	1.45E-02	SLC17A7	-6.55E-03	1.00E+00
STS	9.32E-01	1.46E-02	SLC22A23	1.06E-02	1.00E+00
RBM26	2.57E-01	1.47E-02	TCF15	9.77E-03	1.00E+00
MTHFSD	3.79E-01	1.48E-02	ND4	8.59E-03	1.00E+00
LOC102397151	-6.55E-01	1.49E-02	LOC102397183	-3.78E-03	1.00E+00
TMEM241	3.45E-01	1.49E-02	TM6SF2	1.86E-02	1.00E+00
C16H11orf74	3.72E-01	1.50E-02	LOC112579951	1.39E-02	1.00E+00
TCHP	2.63E-01	1.50E-02	LOC112587476	2.17E-02	1.00E+00
LOC112582199	-6.12E-01	1.51E-02	HPD	2.01E-02	1.00E+00
GARS	2.17E-01	1.52E-02	NXPH1	-1.14E-02	1.00E+00
UBAP1	2.32E-01	1.52E-02	MRS2	-7.62E-03	1.00E+00
MSH4	6.15E-01	1.52E-02	PDE4B	2.21E-02	1.00E+00
QSOX2	-2.89E-01	1.52E-02	GRASP	2.22E-02	1.00E+00
SRD5A2	1.59E+00	1.53E-02	HSPA4	-3.20E-03	1.00E+00
NSUN4	-2.65E-01	1.53E-02	MED11	1.65E-02	1.00E+00
ODF3L2	-1.01E+00	1.54E-02	MPLKIP	1.48E-02	1.00E+00
R3HDM1	2.45E-01	1.54E-02	DUSP6	6.32E-03	1.00E+00
LOC102409492	5.39E-01	1.56E-02	NME7	4.08E-03	1.00E+00
PCDH9	9.03E-01	1.57E-02	CLIP1	5.09E-03	1.00E+00
PGM3	-2.37E-01	1.58E-02	SYAP1	-3.45E-03	1.00E+00
LOC102406144	1.03E+00	1.58E-02	GGT7	-1.21E-02	1.00E+00
SRPX2	-5.66E-01	1.58E-02	GIPC2	-4.85E-03	1.00E+00
FECH	-3.09E-01	1.58E-02	CFAP206	-1.08E-02	1.00E+00
TEDC2	3.24E-01	1.58E-02	FMNL2	5.65E-03	1.00E+00
SLC25A4	-3.44E-01	1.59E-02	FBXO36	9.96E-03	1.00E+00
ULK2	2.78E-01	1.59E-02	HMG20A	-3.57E-03	1.00E+00
SLC22A5	-2.94E-01	1.59E-02	COL19A1	-8.95E-03	1.00E+00
GNPDA1	-5.80E-01	1.59E-02	LOC112582172	-6.50E-03	1.00E+00
INCA1	-5.15E-01	1.59E-02	SAMD7	2.23E-02	1.00E+00

DUSP27	1.25E+00	1.59E-02	LOC102390690	6.97E-03	1.00E+00
C4H12orf75	5.26E-01	1.60E-02	LOC102392353	1.10E-02	1.00E+00
LOC102396428	-5.17E-01	1.60E-02	VAT1	3.13E-03	1.00E+00
BUB1	-2.88E-01	1.62E-02	LOC112585216	-1.75E-02	1.00E+00
PKIB	-8.04E-01	1.63E-02	IGSF8	3.19E-02	1.00E+00
LOC102411003	2.76E-01	1.64E-02	YIPF5	-3.11E-03	1.00E+00
ATF6B	-3.73E-01	1.64E-02	PXT1	-7.75E-03	1.00E+00
LAMA5	-4.66E-01	1.65E-02	ELFN1	4.12E-03	1.00E+00
C2H6orf223	9.15E-01	1.65E-02	F5	-9.96E-03	1.00E+00
LCP1	-8.05E-01	1.66E-02	CLK3	4.15E-03	1.00E+00
ERH	-3.42E-01	1.66E-02	ZFYVE9	-4.05E-03	1.00E+00
ISCA1	-2.28E-01	1.67E-02	ZFAND4	-5.74E-03	1.00E+00
OARD1	-3.38E-01	1.67E-02	ANAPC2	-4.06E-03	1.00E+00
CCT3	3.47E-01	1.68E-02	RRAS	-1.52E-02	1.00E+00
SYNGAP1	-3.43E-01	1.69E-02	TUBB4B	-3.12E-03	1.00E+00
PPP1R8	-2.38E-01	1.69E-02	ANK3	-4.13E-03	1.00E+00
TRNP1	5.21E-01	1.69E-02	IL17RB	6.28E-03	1.00E+00
ATP11C	2.56E-01	1.69E-02	HIF1AN	4.03E-03	1.00E+00
CP	-8.24E-01	1.69E-02	REPS1	-3.12E-03	1.00E+00
SRRM3	6.01E-01	1.70E-02	RPL19	3.68E-03	1.00E+00
IMPG1	1.40E+00	1.71E-02	NOSIP	-3.89E-03	1.00E+00
TGFBR3	-2.80E-01	1.71E-02	MEST	-2.68E-03	1.00E+00
MRTFB	4.30E-01	1.71E-02	LOC112581613	1.73E-02	1.00E+00
SPA17	-5.36E-01	1.71E-02	HACD2	-4.44E-03	1.00E+00
FAM19A5	1.15E+00	1.73E-02	LOC102412044	-4.52E-03	1.00E+00
LOC112581897	-5.66E-01	1.73E-02	SRFBP1	-6.25E-03	1.00E+00
MLEC	-3.25E-01	1.73E-02	JHY	1.03E-02	1.00E+00
MEA1	4.34E-01	1.75E-02	BIN1	-1.65E-02	1.00E+00
TMEM131	-3.03E-01	1.77E-02	PRPF3	-2.83E-03	1.00E+00
ACER2	-5.99E-01	1.77E-02	ZNF275	-6.47E-03	1.00E+00
LOC112577660	5.34E-01	1.78E-02	SMDT1	1.04E-02	1.00E+00
SBF2	-2.70E-01	1.78E-02	LOC102413819	-3.62E-03	1.00E+00
ITGAX	-1.67E+00	1.78E-02	LOC112583592	5.38E-03	1.00E+00
C2H2orf88	-8.42E-01	1.78E-02	ADPRM	5.91E-03	1.00E+00
TM2D1	2.32E-01	1.78E-02	RIMKLB	-1.42E-	1.00E+00

				02	
SLC2A9	9.32E-01	1.78E-02	DYRK1B	-3.53E-03	1.00E+00
ZNF624	3.43E-01	1.79E-02	ENHO	4.78E-03	1.00E+00
METTL26	-4.13E-01	1.80E-02	LOC102400432	4.24E-03	1.00E+00
TIGIT	-9.37E-01	1.81E-02	MGA	-5.49E-03	1.00E+00
GLA	-5.68E-01	1.82E-02	CDH2	1.28E-02	1.00E+00
RNF40	-2.33E-01	1.82E-02	PPP2CA	-2.64E-03	1.00E+00
DPY19L2	7.32E-01	1.83E-02	LOC102394311	-1.44E-02	1.00E+00
TEF	-2.24E-01	1.84E-02	TUBD1	3.18E-03	1.00E+00
CCND3	-3.11E-01	1.84E-02	YIPF6	3.43E-03	1.00E+00
LOC112584177	1.06E+00	1.85E-02	OTOF	-1.13E-02	1.00E+00
FAXC	-4.22E-01	1.85E-02	KAZN	3.70E-03	1.00E+00
MIA2	2.26E-01	1.85E-02	METTL16	2.62E-03	1.00E+00
POMGNT2	3.03E-01	1.87E-02	TTC6	-1.44E-02	1.00E+00
CENPT	3.86E-01	1.87E-02	B4GALT1	4.41E-03	1.00E+00
TBCD	-2.79E-01	1.87E-02	BCAS1	-1.86E-02	1.00E+00
LOC102415185	-5.06E-01	1.88E-02	PSMB4	3.74E-03	1.00E+00
LIG4	-4.90E-01	1.89E-02	LGMN	2.80E-03	1.00E+00
UNC50	-3.01E-01	1.89E-02	ARHGEF10	8.56E-03	1.00E+00
GPSM2	3.00E-01	1.89E-02	SMPD2	-6.11E-03	1.00E+00
XBP1	-3.66E-01	1.89E-02	PAPLN	4.32E-03	1.00E+00
PTPN2	1.97E-01	1.89E-02	VPS11	-3.42E-03	1.00E+00
JAG1	5.84E-01	1.90E-02	ACCSL	5.59E-03	1.00E+00
LOC102390617	-4.38E-01	1.91E-02	NUP210	9.24E-03	1.00E+00
PDGFB	-8.47E-01	1.93E-02	EDRF1	3.67E-03	1.00E+00
SKIDA1	-7.95E-01	1.94E-02	DFFB	-1.26E-02	1.00E+00
VTI1B	2.81E-01	1.95E-02	RAPGEF2	5.05E-03	1.00E+00
LOC102407140	-5.12E-01	1.95E-02	MRPS27	-2.95E-03	1.00E+00
EPG5	3.75E-01	1.96E-02	CLASP2	4.31E-03	1.00E+00
PRSS35	5.00E-01	1.96E-02	TAF8	-3.74E-03	1.00E+00
ATP8A1	2.91E-01	1.97E-02	DNTTIP1	4.57E-03	1.00E+00
CA13	-8.09E-01	1.98E-02	LEMD2	3.96E-03	1.00E+00
NR2C1	-2.62E-01	1.98E-02	PTPRK	1.36E-02	1.00E+00
LOC102389969	4.69E-01	1.99E-02	AUTS2	-2.71E-03	1.00E+00
TMEM116	-3.51E-01	2.00E-02	GHDC	-3.57E-03	1.00E+00

SECISBP2	3.25E-01	2.02E-02	SRPX2	-7.85E-03	1.00E+00
NLRP12	-9.92E-01	2.02E-02	LOC112583978	-9.85E-03	1.00E+00
SMAGP	8.31E-01	2.02E-02	PPP2R5C	-3.37E-03	1.00E+00
LOC102391562	7.22E-01	2.05E-02	C2CD5	-4.52E-03	1.00E+00
CCHCR1	-3.59E-01	2.05E-02	NRDC	2.41E-03	1.00E+00
L2HGDH	-5.62E-01	2.06E-02	SPRYD3	-3.32E-03	1.00E+00
LOC102395153	3.61E-01	2.07E-02	RRM1	2.66E-03	1.00E+00
CUL9	2.28E-01	2.07E-02	CNNM3	3.31E-03	1.00E+00
TCTE3	-7.81E-01	2.08E-02	ARMC4	8.10E-03	1.00E+00
KAT6A	-3.34E-01	2.09E-02	BRI3BP	3.60E-03	1.00E+00
MINDY2	4.07E-01	2.09E-02	LPIN3	5.72E-03	1.00E+00
HPDL	-9.12E-01	2.11E-02	UBALD1	-3.26E-03	1.00E+00
PPM1J	8.65E-01	2.12E-02	AR	1.49E-02	1.00E+00
ZC3H13	-2.63E-01	2.15E-02	YES1	4.12E-03	1.00E+00
UBE2V1	2.28E-01	2.15E-02	NDUFS8	3.87E-03	1.00E+00
LOC112579984	1.32E+00	2.16E-02	LOC102391408	-4.78E-03	1.00E+00
RCVRN	7.64E-01	2.16E-02	MTIF2	-4.08E-03	1.00E+00
CEP78	2.80E-01	2.17E-02	ANKRD46	4.23E-03	1.00E+00
LOC112580648	9.51E-01	2.18E-02	PRPF31	-3.31E-03	1.00E+00
R3HCC1L	2.80E-01	2.21E-02	TAX1BP3	2.63E-03	1.00E+00
AGMO	1.05E+00	2.22E-02	CDHR3	1.00E-02	1.00E+00
DTNA	5.11E-01	2.22E-02	NSRP1	-3.11E-03	1.00E+00
MRPL19	-3.32E-01	2.24E-02	EXOSC2	-2.49E-03	1.00E+00
PTPRJ	-5.23E-01	2.24E-02	NELFA	2.12E-03	1.00E+00
SLC17A5	4.91E-01	2.25E-02	KCTD21	5.90E-03	1.00E+00
LRRC43	6.71E-01	2.26E-02	IRF3	3.15E-03	1.00E+00
PDCD4	2.36E-01	2.26E-02	PHLDA1	-5.57E-03	1.00E+00
CLDN15	-7.39E-01	2.29E-02	NAV1	6.05E-03	1.00E+00
BCO2	-1.10E+00	2.29E-02	SLC38A8	-2.94E-03	1.00E+00
SOD1	-3.02E-01	2.30E-02	SLC2A8	-2.84E-03	1.00E+00
WAPL	2.56E-01	2.32E-02	KIAA0232	-4.52E-03	1.00E+00
ARHGAP22	4.20E-01	2.32E-02	VPS35L	2.99E-03	1.00E+00
LOC102403165	1.02E+00	2.32E-02	RNF11	-4.30E-03	1.00E+00
AK8	3.72E-01	2.33E-02	GALNTL6	2.55E-03	1.00E+00
TOP3A	-2.54E-01	2.33E-02	RAB25	-3.09E-03	1.00E+00
LOC102396286	7.55E-01	2.34E-02	ARF6	-2.00E-03	1.00E+00

GEMIN5	2.32E-01		2.35E-02	FOPNL	2.98E-03	1.00E+00
MAPK6	-3.66E-01		2.35E-02	LOC102403797	-6.27E-03	1.00E+00
POP4	-2.59E-01		2.38E-02	GFRA4	8.08E-03	1.00E+00
SEC16A	2.95E-01		2.38E-02	NAA11	-5.98E-03	1.00E+00
DIAPH2	2.48E-01		2.38E-02	HDDC2	5.58E-03	1.00E+00
CBX1	-2.08E-01		2.39E-02	SERP1	-2.18E-03	1.00E+00
BROX	3.41E-01		2.39E-02	TPST1	2.80E-03	1.00E+00
LOC102405919	8.49E-01		2.40E-02	FRK	-7.43E-03	1.00E+00
LOC102413681	4.23E-01		2.40E-02	PLEKHH3	8.98E-03	1.00E+00
ANAPC13	-4.14E-01		2.40E-02	UBQLN2	3.16E-03	1.00E+00
TXNRD3	-3.63E-01		2.40E-02	ERBIN	-5.28E-03	1.00E+00
HNRNPH3	2.59E-01		2.41E-02	FHIT	-2.03E-03	1.00E+00
LOC102392508	8.23E-01		2.44E-02	MELTF	-8.68E-03	1.00E+00
RGS6	3.56E-01		2.44E-02	CCP110	3.61E-03	1.00E+00
CHRM2	2.21E-01		2.44E-02	RAB11FIP2	5.12E-03	1.00E+00
MYO1D	-4.15E-01		2.45E-02	LOC102393855	3.43E-03	1.00E+00
LOC112583979	1.33E+00		2.46E-02	SHMT1	5.51E-03	1.00E+00
MUTYH	-3.41E-01		2.47E-02	CFAP300	-3.02E-03	1.00E+00
LOC102408590	1.16E+00		2.48E-02	DIO3	-3.49E-03	1.00E+00
ALG1	-3.40E-01		2.48E-02	ZNF827	6.78E-03	1.00E+00
PPP6C	2.70E-01		2.54E-02	TIMM21	-2.35E-03	1.00E+00
FAM208B	-3.44E-01		2.56E-02	SARS2	-3.82E-03	1.00E+00
LOC112580318	7.91E-01		2.57E-02	ZNF81	7.78E-03	1.00E+00
TRIM44	2.04E-01		2.59E-02	FLT4	1.08E-02	1.00E+00
LOC112582116	7.87E-01		2.60E-02	UBE2Q1	2.10E-03	1.00E+00
EXOSC1	-2.91E-01		2.60E-02	LOC112578174	-1.13E-02	1.00E+00
ALDH18A1	-2.23E-01		2.61E-02	SNRPN	7.15E-03	1.00E+00
TSNAX	-2.51E-01		2.62E-02	ABHD15	-1.46E-02	1.00E+00
BTF3	2.69E-01		2.62E-02	LOC102396042	7.57E-03	1.00E+00
LIPC	1.30E+00		2.65E-02	INTS6	-2.46E-03	1.00E+00
SLC38A7	-2.79E-01		2.66E-02	PADI3	-9.83E-03	1.00E+00
CNOT8	2.18E-01		2.66E-02	LANCL1	2.06E-03	1.00E+00
MDH2	-2.88E-01		2.66E-02	CCDC9	-2.51E-03	1.00E+00
LOC112583810	-8.18E-01		2.67E-02	CFAP299	-4.73E-03	1.00E+00
FNDC5	-5.40E-01		2.68E-02	CCDC14	3.49E-03	1.00E+00
SLC43A3	-3.04E-01		2.69E-02	ANKRD13C	3.68E-03	1.00E+00

GDPGP1	-3.16E-01	2.69E-02	SLC25A42	-2.46E-03	1.00E+00		
DNAAF4	2.81E-01	2.70E-02	EXOC6	2.52E-03	1.00E+00		
LOC102392808	1.09E+00	2.72E-02	GNG2	-4.13E-03	1.00E+00		
CUL4A	-1.95E-01	2.72E-02	TTYH1	1.17E-02	1.00E+00		
LOC102409453	-3.80E-01	2.72E-02	LOC102406866	3.30E-03	1.00E+00		
BMP8B	-1.22E+00	2.74E-02	PIGM	-3.85E-03	1.00E+00		
DYNLT3	-2.55E-01	2.74E-02	TRMT61B	-3.07E-03	1.00E+00		
GPR62	8.91E-01	2.75E-02	IRF2BP1	-1.73E-03	1.00E+00		
TGM5	-9.25E-01	2.75E-02	WDR86	7.29E-03	1.00E+00		
COL5A3	-1.01E+00	2.79E-02	DSCAML1	-2.06E-03	1.00E+00		
CHGA	-3.67E-01	2.79E-02	IL36RN	-7.01E-03	1.00E+00		
TCTEX1D2	-3.33E-01	2.80E-02	LOC112578831	-3.87E-03	1.00E+00		
MMP25	-1.10E+00	2.80E-02	PHAX	-2.07E-03	1.00E+00		
DNAJC5	2.17E-01	2.81E-02	MTHFSD	-3.22E-03	1.00E+00		
SEMA3F	-5.45E-01	2.81E-02	ABCG2	-1.94E-03	1.00E+00		
FLT3	1.54E+00	2.82E-02	LOC112581272	5.58E-03	1.00E+00		
TRIM33	2.82E-01	2.83E-02	LOC102403045	7.62E-03	1.00E+00		
RASAL3	-7.98E-01	2.85E-02	LOC112583894	6.24E-03	1.00E+00		
MYO1B	-2.77E-01	2.86E-02	TMEM259	1.68E-03	1.00E+00		
WDR5B	5.52E-01	2.87E-02	GTF2H1	1.83E-03	1.00E+00		
TCTN3	2.39E-01	2.88E-02	GDPD5	-1.73E-03	1.00E+00		
LOC102409538	-1.66E+00	2.89E-02	GPR88	-1.06E-02	1.00E+00		
IL1RN	-1.29E+00	2.89E-02	PAIP2B	2.18E-03	1.00E+00		
LOC102408654	8.96E-01	2.89E-02	SHKBP1	2.16E-03	1.00E+00		
LOC112580228	7.64E-01	2.90E-02	ARCN1	-1.79E-03	1.00E+00		
ATP6AP1L	9.39E-01	2.91E-02	LOC102399500	6.87E-03	1.00E+00		
UNC5C	-2.51E-01	2.94E-02	UNC5B	2.17E-03	1.00E+00		
STC2	-9.88E-01	2.98E-02	TRMT6	2.61E-03	1.00E+00		
SOCS6	2.67E-01	2.98E-02	LOC102389167	1.10E-02	1.00E+00		
TSTD3	-4.58E-01	2.99E-02	ARMC9	3.81E-03	1.00E+00		
CATSPER2	4.14E-01	3.00E-02	DDX46	-1.96E-03	1.00E+00		
LOC112579935	-6.06E-01	3.00E-02	PPM1D	4.39E-03	1.00E+00		
PTBP2	-2.64E-01	3.01E-02	EXTL2	4.16E-03	1.00E+00		
YEATS4	2.71E-01	3.04E-02	SAMD5	7.41E-03	1.00E+00		
COG8	3.00E-01	3.05E-02	STK19	-4.49E-03	1.00E+00		

ATXN7L2	-3.27E-01	3.05E-02	ENOX2	-3.66E-03	1.00E+00		
SPAG9	-2.55E-01	3.06E-02	UBAC1	2.01E-03	1.00E+00		
LOC102391229	3.44E-01	3.09E-02	PPM1N	5.79E-03	1.00E+00		
MYLK3	-4.48E-01	3.09E-02	TMEM41A	-3.22E-03	1.00E+00		
C12H9orf116	5.32E-01	3.10E-02	LOC102406900	-1.15E-02	1.00E+00		
SLX4IP	-2.95E-01	3.10E-02	ARFGAP3	-2.63E-03	1.00E+00		
ST6GALNAC2	-1.16E+00	3.10E-02	CPLANE1	-3.04E-03	1.00E+00		
ZC2HC1B	5.15E-01	3.10E-02	TCHP	-2.20E-03	1.00E+00		
TMEM132B	-8.62E-01	3.10E-02	C12H2orf16	-3.82E-03	1.00E+00		
CNNM3	2.19E-01	3.11E-02	PSME1	2.62E-03	1.00E+00		
MMP12	-2.51E+00	3.11E-02	SLC25A6	1.95E-03	1.00E+00		
VPS35	2.10E-01	3.11E-02	POLM	1.74E-03	1.00E+00		
MORN1	-3.53E-01	3.12E-02	LOC102408471	4.03E-03	1.00E+00		
KRIT1	5.01E-01	3.13E-02	LOC102398834	-3.15E-03	1.00E+00		
REXO1	3.13E-01	3.13E-02	C2H6orf136	2.91E-03	1.00E+00		
NCLN	-2.40E-01	3.15E-02	RNF121	1.40E-03	1.00E+00		
C3H17orf49	-4.77E-01	3.16E-02	NDUFB2	-2.19E-03	1.00E+00		
GOPC	2.96E-01	3.17E-02	IDH3B	-2.31E-03	1.00E+00		
LOC102398019	-5.69E-01	3.17E-02	HHLA2	-2.15E-03	1.00E+00		
KCNIP1	1.33E+00	3.18E-02	HOOK2	-1.74E-03	1.00E+00		
MRPL36	-3.44E-01	3.18E-02	TAT	2.61E-03	1.00E+00		
RNF13	1.97E-01	3.18E-02	CERCAM	2.24E-03	1.00E+00		
ACRV1	-7.49E-01	3.19E-02	DRAP1	1.56E-03	1.00E+00		
COL9A2	8.75E-01	3.20E-02	ACTL6A	-2.59E-03	1.00E+00		
PLXND1	5.51E-01	3.21E-02	LOC102395582	1.96E-03	1.00E+00		
LRFN1	-3.24E-01	3.22E-02	MORC1	2.20E-03	1.00E+00		
RFTN1	-4.07E-01	3.22E-02	SLC15A5	-6.83E-03	1.00E+00		
FNBP1L	-2.97E-01	3.23E-02	C5H1orf21	6.46E-03	1.00E+00		
LSM8	-3.19E-01	3.23E-02	SIN3A	1.91E-03	1.00E+00		
ITGA2	-7.17E-01	3.25E-02	RASGRF1	6.55E-03	1.00E+00		
HECW1	-2.45E-01	3.25E-02	GDI2	-1.41E-03	1.00E+00		
INAFM1	-3.58E-01	3.32E-02	ACOT12	-7.60E-03	1.00E+00		
ESYT1	2.91E-01	3.33E-02	LOC112582937	-7.60E-03	1.00E+00		
PPP1R10	1.97E-01	3.34E-02	RLF	-2.56E-03	1.00E+00		

LOC102409347	-2.98E-01	3.35E-02	THAP3	3.30E-03	1.00E+00		
LOC112583737	4.63E-01	3.35E-02	MAP2K1	-2.25E-03	1.00E+00		
NFKBIB	3.32E-01	3.36E-02	BBS2	-1.33E-03	1.00E+00		
ATG4C	-2.67E-01	3.37E-02	LRRC59	-1.29E-03	1.00E+00		
NDUFAB1	3.19E-01	3.39E-02	LOC102393767	-5.19E-03	1.00E+00		
THOC5	2.20E-01	3.41E-02	ATG4D	-1.75E-03	1.00E+00		
THEMIS2	-8.84E-01	3.42E-02	PLEKHO2	2.90E-03	1.00E+00		
TMEM236	1.25E+00	3.43E-02	LOC102404060	6.04E-03	1.00E+00		
KPNA3	-1.99E-01	3.44E-02	EBF1	2.59E-03	1.00E+00		
DUSP5	4.28E-01	3.45E-02	DNAJC10	-1.57E-03	1.00E+00		
SGSH	2.72E-01	3.47E-02	LOC102404139	5.04E-03	1.00E+00		
AP2B1	2.01E-01	3.48E-02	COL5A3	7.93E-03	1.00E+00		
LOC102389879	-5.42E-01	3.48E-02	OXA1L	1.34E-03	1.00E+00		
BDH1	3.49E-01	3.50E-02	LOC112582356	-5.63E-03	1.00E+00		
BUD31	2.82E-01	3.54E-02	TTC13	2.59E-03	1.00E+00		
KDM2B	2.34E-01	3.54E-02	PABPC4	-1.37E-03	1.00E+00		
LOC102406414	1.20E+00	3.56E-02	PRR7	1.35E-03	1.00E+00		
ALDH7A1	2.42E-01	3.57E-02	SCGN	-4.70E-03	1.00E+00		
LVRN	-7.44E-01	3.59E-02	YWHAH	1.23E-03	1.00E+00		
STPG4	6.65E-01	3.60E-02	LOC112583539	-1.84E-03	1.00E+00		
RBM6	2.01E-01	3.60E-02	LOC102390278	4.06E-03	1.00E+00		
TUBA8	-6.31E-01	3.61E-02	PPDPF	-1.49E-03	1.00E+00		
SPSB4	-6.91E-01	3.61E-02	DELE1	2.09E-03	1.00E+00		
LOC112583669	-6.31E-01	3.62E-02	BRCC3	-1.45E-03	1.00E+00		
OIT3	-6.34E-01	3.63E-02	LOC112577760	-3.69E-03	1.00E+00		
LOC112581825	4.01E-01	3.63E-02	LOC102404020	-2.11E-03	1.00E+00		
IMMT	-1.97E-01	3.63E-02	LOC102413321	-8.73E-03	1.00E+00		
CEBPG	-2.13E-01	3.63E-02	USP1	1.43E-03	1.00E+00		
ENPP6	-8.31E-01	3.63E-02	KMT2E	1.41E-03	1.00E+00		
SMARCD1	-1.77E-01	3.64E-02	RETREG1	2.95E-03	1.00E+00		
FGR	-1.32E+00	3.66E-02	TULP2	2.48E-03	1.00E+00		
ERC2	3.58E-01	3.66E-02	SLC35F5	-1.92E-03	1.00E+00		
CCDC25	-1.93E-01	3.69E-02	LOC112582252	3.45E-03	1.00E+00		
SMIM15	2.96E-01	3.70E-02	CTU2	-3.05E-03	1.00E+00		

PHEX	3.23E-01	3.71E-02	ZNF852	3.11E-03	1.00E+00		
SLC4A5	-7.26E-01	3.76E-02	REPIN1	1.92E-03	1.00E+00		
ZDHHC3	2.26E-01	3.79E-02	FKBP10	-5.30E-03	1.00E+00		
AK9	7.96E-01	3.79E-02	REEP3	1.23E-03	1.00E+00		
NUPL2	3.22E-01	3.80E-02	ETAA1	-1.31E-03	1.00E+00		
LOC112582946	-4.99E-01	3.81E-02	SNU13	-1.10E-03	1.00E+00		
SERINC3	2.61E-01	3.82E-02	SLC17A6	2.74E-03	1.00E+00		
CDK5	3.29E-01	3.85E-02	ADM5	-2.51E-03	1.00E+00		
LOC112584602	7.14E-01	3.85E-02	ERAP1	7.81E-03	1.00E+00		
NUDT3	1.80E-01	3.86E-02	LOC102396998	2.14E-03	1.00E+00		
C4H12orf10	2.97E-01	3.87E-02	MCM3	-1.29E-03	1.00E+00		
MARK3	-1.91E-01	3.87E-02	TXLNG	1.11E-03	1.00E+00		
DAP3	2.19E-01	3.91E-02	RTN3	1.00E-03	1.00E+00		
RNF214	2.34E-01	3.93E-02	LOC112579941	-2.66E-03	1.00E+00		
RBMS1	-2.31E-01	3.93E-02	TM2D2	-1.41E-03	1.00E+00		
SPATA6	-3.52E-01	3.93E-02	EXOC2	-9.25E-04	1.00E+00		
CD36	5.19E-01	3.93E-02	MARCH11	5.38E-03	1.00E+00		
CAMKK1	5.28E-01	3.94E-02	DYNLL1	-1.16E-03	1.00E+00		
RELL1	3.54E-01	3.95E-02	TRPM1	-5.21E-03	1.00E+00		
DEGS2	8.03E-01	3.96E-02	ZFAT	1.50E-03	1.00E+00		
TNFRSF4	-5.19E-01	3.97E-02	CLCN1	3.17E-03	1.00E+00		
SUN2	2.69E-01	3.97E-02	OSBPL9	-8.83E-04	1.00E+00		
LOC102389840	-6.28E-01	3.98E-02	POLR3GL	-2.77E-03	1.00E+00		
FSTL5	-1.26E+00	3.98E-02	MANBAL	1.21E-03	1.00E+00		
GSDME	-3.88E-01	4.01E-02	LOC102397238	-1.19E-03	1.00E+00		
UBXN4	1.79E-01	4.02E-02	SLC39A10	1.61E-03	1.00E+00		
NXF1	-1.84E-01	4.02E-02	CHAF1A	1.05E-03	1.00E+00		
AFDN	-2.77E-01	4.06E-02	MLYCD	3.32E-03	1.00E+00		
PGGT1B	2.37E-01	4.06E-02	CCDC80	-6.38E-03	1.00E+00		
KIF2A	1.98E-01	4.07E-02	LOC112581361	-7.68E-03	1.00E+00		
TAPT1	-2.95E-01	4.14E-02	DDR2	-2.01E-03	1.00E+00		
CLVS2	1.55E+00	4.14E-02	LOC102411606	-1.13E-03	1.00E+00		
TWIST1	6.82E-01	4.15E-02	RARA	1.56E-03	1.00E+00		
LOC102394850	2.70E-01	4.16E-02	GNPTG	-1.27E-03	1.00E+00		
STIP1	-2.34E-01	4.16E-02	MCOLN1	-1.01E-03	1.00E+00		
FLOT2	-2.27E-	4.16E-02	ASB3	-9.59E-	1.00E+00		

	01			04			
ANXA7	2.06E-01	4.17E-02	NOL10	8.19E-04	1.00E+00		
SSSCA1	2.43E-01	4.17E-02	LOC102414919	9.81E-04	1.00E+00		
YME1L1	-1.74E-01	4.17E-02	GOLGA1	-9.85E-04	1.00E+00		
TBC1D31	5.37E-01	4.18E-02	NUP98	1.21E-03	1.00E+00		
TRMT11	-2.39E-01	4.25E-02	CACNA1S	4.37E-03	1.00E+00		
NPC1	-3.49E-01	4.27E-02	CLSTN3	-1.39E-03	1.00E+00		
LOC102405932	5.65E-01	4.28E-02	STC2	5.53E-03	1.00E+00		
LOC102414453	-5.02E-01	4.30E-02	ABCA10	4.44E-03	1.00E+00		
MOK	-3.84E-01	4.32E-02	RDH10	1.00E-03	1.00E+00		
IQCM	1.04E+00	4.32E-02	FBN3	-3.84E-03	1.00E+00		
CA10	-9.50E-01	4.33E-02	CAMK2G	-6.06E-04	1.00E+00		
RUNDC3A	-6.29E-01	4.33E-02	TRIM17	2.77E-03	1.00E+00		
CAPN7	1.98E-01	4.35E-02	DESI1	6.33E-04	1.00E+00		
RNF152	-5.98E-01	4.35E-02	ANO5	-1.31E-03	1.00E+00		
LOC112586254	3.08E-01	4.35E-02	POLR3G	8.01E-04	1.00E+00		
ZBTB11	2.51E-01	4.35E-02	DNAJB11	7.86E-04	1.00E+00		
PRR14L	-3.45E-01	4.35E-02	C24H16orf71	4.00E-03	1.00E+00		
FAM92B	-7.58E-01	4.36E-02	FABP5	8.10E-04	1.00E+00		
CLTC	-2.50E-01	4.37E-02	LOC112578057	1.69E-03	1.00E+00		
FAM181B	6.11E-01	4.37E-02	PWP1	-5.65E-04	1.00E+00		
C2H6orf10	5.75E-01	4.37E-02	TRAPPC10	1.11E-03	1.00E+00		
EXO5	3.49E-01	4.38E-02	LGALS8	-7.81E-04	1.00E+00		
PIM1	-6.53E-01	4.39E-02	TAF13	-1.44E-03	1.00E+00		
SLCO3A1	-2.12E-01	4.39E-02	TTC24	-8.77E-04	1.00E+00		
MRPL11	2.97E-01	4.40E-02	ATG3	5.89E-04	1.00E+00		
LOC112586827	9.79E-01	4.41E-02	SPAG9	-7.33E-04	1.00E+00		
ACLY	-2.43E-01	4.42E-02	TNFRSF4	1.98E-03	1.00E+00		
EXOC3L1	-4.10E-01	4.44E-02	SUPT3H	7.87E-04	1.00E+00		
LOC102408471	3.82E-01	4.44E-02	VTI1A	5.28E-04	1.00E+00		
CDK5RAP3	-2.70E-01	4.45E-02	LOC102391387	2.73E-03	1.00E+00		
PLXDC1	-6.40E-01	4.47E-02	CRISPLD2	2.32E-03	1.00E+00		
TRIM24	-2.08E-01	4.49E-02	ADPGK	9.39E-04	1.00E+00		
CLASRP	-2.30E-01	4.52E-02	PDK3	-4.76E-04	1.00E+00		
ZKSCAN5	-1.97E-01	4.52E-02	CREB5	9.45E-04	1.00E+00		
ZFP92	6.17E-01	4.52E-02	ZNF189	-1.36E-03	1.00E+00		

CPAMD8	5.93E-01	4.53E-02	LOC102396257	-1.31E-03	1.00E+00		
RHOU	-4.70E-01	4.55E-02	LOC102390265	7.02E-04	1.00E+00		
LOC112587357	7.19E-01	4.56E-02	LOC112582086	1.41E-03	1.00E+00		
TGFBRAP1	2.34E-01	4.58E-02	SDHC	5.11E-04	1.00E+00		
TIMM13	-2.74E-01	4.58E-02	LRRC9	2.49E-03	1.00E+00		
ZCCHC17	2.76E-01	4.58E-02	BCL2L11	-6.53E-04	1.00E+00		
C20H15orf39	-4.16E-01	4.59E-02	FAM131C	1.98E-03	1.00E+00		
CCDC85A	3.90E-01	4.62E-02	ZNF496	-8.07E-04	1.00E+00		
GAK	2.16E-01	4.62E-02	RAD54B	5.23E-04	1.00E+00		
ETS1	3.98E-01	4.62E-02	RARRES2	2.62E-03	1.00E+00		
MRPL51	3.03E-01	4.62E-02	C24H7orf26	4.70E-04	1.00E+00		
PCSK1	4.04E-01	4.63E-02	DAD1	-5.09E-04	1.00E+00		
ITGA9	2.87E-01	4.67E-02	EIF3G	-5.08E-04	1.00E+00		
MED24	1.78E-01	4.67E-02	LOC112586992	-3.34E-03	1.00E+00		
MRPL47	3.53E-01	4.68E-02	PHYHD1	-1.55E-03	1.00E+00		
WDR45B	1.80E-01	4.68E-02	LOC112583979	-2.96E-03	1.00E+00		
PIK3CA	-4.33E-01	4.72E-02	LOC102389985	8.44E-04	1.00E+00		
IBA57	-2.95E-01	4.74E-02	LSM14B	4.29E-04	1.00E+00		
GTF3C4	-4.99E-01	4.74E-02	LOC102404498	-1.08E-03	1.00E+00		
STAT4	-5.26E-01	4.74E-02	FECH	-6.25E-04	1.00E+00		
PRMT7	2.18E-01	4.81E-02	ARHGEF10L	-5.59E-04	1.00E+00		
KCNJ15	1.28E+00	4.81E-02	LOC102404511	-1.88E-03	1.00E+00		
TOE1	-2.77E-01	4.83E-02	RBM46	5.56E-04	1.00E+00		
LOC102412527	-3.14E-01	4.83E-02	DDIT4	-5.61E-04	1.00E+00		
LOC112581447	-3.96E-01	4.84E-02	LOC102408803	-1.21E-03	1.00E+00		
FAM53B	-1.99E-01	4.85E-02	UACA	4.00E-04	1.00E+00		
TMEM42	3.34E-01	4.85E-02	FARSA	4.15E-04	1.00E+00		
CACNB2	-3.75E-01	4.86E-02	YARS	-6.35E-04	1.00E+00		
CORO2B	-5.80E-01	4.87E-02	JAM2	-3.50E-03	1.00E+00		
LOC112579189	-7.35E-01	4.91E-02	PUSL1	-4.15E-04	1.00E+00		
CEP97	2.66E-01	4.92E-02	YARS2	-5.60E-04	1.00E+00		
ATP10A	-4.01E-01	4.92E-02	RPF1	4.17E-04	1.00E+00		
PROSER3	3.58E-01	4.94E-02	EEF2	3.26E-04	1.00E+00		
MED4	-1.65E-01	4.95E-02	CLP1	3.29E-04	1.00E+00		
APC	3.13E-01	4.95E-02	IRX1	-1.87E-	1.00E+00		

				03	
LYRM1	3.20E-01	4.97E-02	HECW1	-4.28E-04	1.00E+00
TUBB4B	-3.97E-01	4.98E-02	AJAP1	-9.19E-04	1.00E+00
CLEC11A	5.27E-01	5.00E-02	ARSB	-2.72E-04	1.00E+00
SCCPDH	-2.05E-01	5.03E-02	AJM1	-1.35E-03	1.00E+00
GNAL	-2.12E-01	5.04E-02	LOC102394580	-2.78E-04	1.00E+00
LOC112584382	4.89E-01	5.04E-02	NDUFA2	3.61E-04	1.00E+00
LOC102406239	-9.59E-01	5.10E-02	OTUD1	-5.72E-04	1.00E+00
PRPF18	-2.10E-01	5.13E-02	HOXC9	-4.75E-04	1.00E+00
LOC102414025	-8.41E-01	5.13E-02	MRM1	3.25E-04	1.00E+00
LOC102414126	-5.86E-01	5.15E-02	CCT3	-2.01E-04	1.00E+00
ANAPC11	3.21E-01	5.16E-02	UBLCP1	2.36E-04	1.00E+00
KRTCAP2	-3.13E-01	5.17E-02	LOC112580195	-7.74E-04	1.00E+00
PRKDC	3.32E-01	5.18E-02	C3H17orf53	2.29E-04	1.00E+00
LOC112577704	8.67E-01	5.21E-02	LOC112580286	3.41E-04	1.00E+00
LOC102411217	5.13E-01	5.21E-02	RPL8	1.54E-04	1.00E+00
TPD52L2	2.51E-01	5.21E-02	SYCP1	1.26E-04	1.00E+00
MAP3K6	-4.36E-01	5.21E-02	ITGA3	1.20E-04	1.00E+00
LOC102403241	-2.73E-01	5.22E-02	CADM1	1.31E-04	1.00E+00
LOC112582088	-7.65E-01	5.24E-02	SERPINC1	-5.88E-04	1.00E+00
RBM41	-2.90E-01	5.26E-02	NDUFB9	-1.05E-04	1.00E+00
ZNF385D	-8.20E-01	5.27E-02	VSTM5	2.03E-04	1.00E+00
AP2A2	-1.88E-01	5.27E-02	LOC102389807	2.85E-04	1.00E+00
HDHD5	3.31E-01	5.28E-02	PARVG	1.60E-04	1.00E+00
SLC6A9	-6.48E-01	5.29E-02	LOC102398273	-1.22E-04	1.00E+00
LYSMD1	-2.54E-01	5.29E-02	SPDYC	2.58E-05	1.00E+00
CFAP47	-5.38E-01	5.30E-02	UHRF1BP1L	-5.89E-05	1.00E+00
RAB8B	-2.24E-01	5.30E-02	PCBP4	1.53E-05	1.00E+00
MACF1	-3.36E-01	5.32E-02	KIFC3	6.99E-06	1.00E+00
NEK2	3.29E-01	5.32E-02	CHD6	6.98E-06	1.00E+00
FTCDNL1	5.21E-01	5.34E-02			
SYNCRIP	-1.94E-01	5.36E-02			
TAB2	-2.19E-01	5.37E-02			
SAP25	4.48E-01	5.38E-02			
NARS2	-2.37E-01	5.39E-02			
AKT1S1	2.21E-01	5.41E-02			

SNRPF	-3.37E-01	5.42E-02					
CFAP126	-4.90E-01	5.45E-02					
LRRC10	-7.21E-01	5.46E-02					
VWCE	4.28E-01	5.47E-02					
POLR3B	-2.29E-01	5.48E-02					
DDX55	2.12E-01	5.53E-02					
IPPK	-2.38E-01	5.53E-02					
HTRA4	-2.58E-01	5.59E-02					
MAK16	-3.61E-01	5.60E-02					
PXYLP1	-2.69E-01	5.60E-02					
ZBTB48	-2.59E-01	5.61E-02					
F3	-3.00E-01	5.62E-02					
LOC102400593	8.90E-01	5.66E-02					
RBM33	1.90E-01	5.67E-02					
CRYBB1	4.57E-01	5.70E-02					
LOC112581104	7.04E-01	5.74E-02					
LOC112585002	-5.15E-01	5.75E-02					
PLAGL1	-3.80E-01	5.75E-02					
ZNF285	-4.87E-01	5.75E-02					
SARS	-2.25E-01	5.76E-02					
CEP89	2.21E-01	5.83E-02					
ZMIZ2	2.25E-01	5.83E-02					
RPS19BP1	-3.07E-01	5.83E-02					
FKBP1B	-4.32E-01	5.84E-02					
C24H16orf71	-6.70E-01	5.84E-02					
MECP2	-1.96E-01	5.86E-02					
LOC102411097	5.39E-01	5.86E-02					
SMC5	2.46E-01	5.86E-02					
FBXO46	2.33E-01	5.87E-02					
GATAD2B	2.47E-01	5.88E-02					
LOC102393133	3.25E-01	5.88E-02					
PSMD14	-1.88E-01	5.88E-02					
EIF1AD	2.70E-01	5.90E-02					
CUTC	2.50E-01	5.90E-02					
SELENOH	3.93E-01	5.91E-02					
ME2	-1.62E-01	5.94E-02					
CCR1	-9.21E-01	5.95E-02					

MARS2	7.85E-01	6.00E-02
CDC42	1.91E-01	6.01E-02
LOC102408483	6.89E-01	6.04E-02
ALKBH8	2.42E-01	6.04E-02
C18H19orf33	-7.49E-01	6.06E-02
PFDN2	-2.68E-01	6.11E-02
DNAJA1	2.69E-01	6.11E-02
AKR1A1	-2.75E-01	6.12E-02
TXNL4B	-2.27E-01	6.14E-02
HSF1	-2.01E-01	6.16E-02
EIF4EBP3	2.89E-01	6.16E-02
LSMEM1	-8.31E-01	6.16E-02
SLC20A1	2.96E-01	6.19E-02
LOC102398822	3.59E-01	6.20E-02
COQ2	-2.50E-01	6.28E-02
VHL	-2.74E-01	6.31E-02
C4H12orf73	2.77E-01	6.32E-02
PNPT1	-1.68E-01	6.32E-02
NPRL3	-2.15E-01	6.36E-02
BSCL2	-1.88E-01	6.36E-02
RAB24	-2.46E-01	6.51E-02
PPIL4	2.38E-01	6.55E-02
LOC102416224	-2.22E-01	6.57E-02
MET	-4.80E-01	6.60E-02
LOC112581412	-1.10E+00	6.61E-02
HPSE2	-1.10E+00	6.63E-02
CEP120	1.99E-01	6.67E-02
LOC112577656	6.87E-01	6.70E-02
ATP2B1	2.11E-01	6.71E-02
HDDC3	-3.50E-01	6.71E-02
RRP7A	2.29E-01	6.72E-02
LOC112578502	-3.37E-01	6.73E-02
DAZAP2	-1.62E-01	6.73E-02
FEN1	2.41E-01	6.78E-02
MRPS2	-2.80E-01	6.79E-02
NID1	7.90E-01	6.79E-02
CENPE	-2.47E-01	6.79E-02

PPWD1	2.11E-01	6.81E-02					
LOC102409182	-7.85E-01	6.82E-02					
TMEM265	3.33E-01	6.83E-02					
HYAL3	-4.53E-01	6.85E-02					
LOC102412565	9.63E-01	6.85E-02					
SLC25A16	3.86E-01	6.88E-02					
LOC112587339	1.15E+00	6.90E-02					
ELAC2	2.14E-01	6.90E-02					
JMJD7	-4.93E-01	6.96E-02					
ENKUR	-3.28E-01	7.00E-02					
B4GALT5	-1.92E-01	7.03E-02					
C17H12orf65	-3.16E-01	7.06E-02					
SLC6A16	7.37E-01	7.07E-02					
VDR	3.81E-01	7.09E-02					
SRRD	4.27E-01	7.12E-02					
MSH6	1.78E-01	7.15E-02					
GPR161	2.31E-01	7.16E-02					
PNPLA1	-7.34E-01	7.17E-02					
LOC112580425	3.22E-01	7.18E-02					
NCOA6	2.07E-01	7.22E-02					
DKC1	-2.04E-01	7.24E-02					
OSBPL6	2.46E-01	7.24E-02					
CNTD1	4.26E-01	7.25E-02					
LAMC1	-2.40E-01	7.26E-02					
C3H9orf85	3.83E-01	7.31E-02					
FRAT2	2.86E-01	7.33E-02					
CSNK2A2	-4.08E-01	7.34E-02					
CT55	-7.03E-01	7.36E-02					
LOC112586500	-4.89E-01	7.39E-02					
JAM3	1.82E-01	7.45E-02					
ACACB	-3.43E-01	7.46E-02					
FOXO3	-2.12E-01	7.49E-02					
LOC112580384	3.96E-01	7.49E-02					
MMAB	3.16E-01	7.50E-02					
ACTR8	-1.79E-01	7.51E-02					
AKT3	-2.06E-01	7.51E-02					
ARPC2	-1.90E-01	7.52E-02					
INO80D	2.48E-01	7.53E-02					
LOC102414507	3.26E-01	7.53E-02					

LOC112585201	-6.53E-01	7.58E-02					
STKLD1	4.33E-01	7.59E-02					
TMEM127	-1.80E-01	7.61E-02					
VAMP4	-2.18E-01	7.61E-02					
ERG28	-2.54E-01	7.61E-02					
CNOT7	1.77E-01	7.61E-02					
DCDC2	1.32E+00	7.62E-02					
KAT6B	-2.80E-01	7.64E-02					
SEL1L	-2.35E-01	7.65E-02					
BCHE	-6.10E-01	7.65E-02					
NXPE3	-2.44E-01	7.69E-02					
MRPL21	2.41E-01	7.71E-02					
ZSCAN25	-2.54E-01	7.71E-02					
TOPBP1	-1.97E-01	7.71E-02					
RHEB	-1.84E-01	7.75E-02					
LOC102405068	4.77E-01	7.77E-02					
PPT2	2.77E-01	7.77E-02					
RIDA	-3.17E-01	7.78E-02					
NTN3	-7.33E-01	7.82E-02					
RNF26	-2.14E-01	7.83E-02					
ABCB4	-5.33E-01	7.86E-02					
HDGFL2	2.40E-01	7.94E-02					
TYSND1	2.66E-01	8.03E-02					
CCDC126	-2.56E-01	8.07E-02					
LOC102405806	4.77E-01	8.07E-02					
LOC102413559	1.02E+00	8.07E-02					
METTL25	-2.57E-01	8.13E-02					
RRM2	2.86E-01	8.14E-02					
TTYH1	-6.70E-01	8.15E-02					
TICAM1	-3.79E-01	8.15E-02					
TNR	9.32E-01	8.15E-02					
LOC102412752	7.49E-01	8.17E-02					
LYRM4	-2.75E-01	8.20E-02					
NEDD4	3.87E-01	8.25E-02					
ZNF839	2.26E-01	8.31E-02					
PPP1CC	-1.54E-01	8.37E-02					
LOC102407057	-1.84E-01	8.37E-02					

ISCU	-1.94E-01	8.40E-02					
IGSF10	-5.23E-01	8.41E-02					
PRPF3	1.43E-01	8.43E-02					
PANX1	1.81E-01	8.44E-02					
PPP2CA	1.71E-01	8.44E-02					
ADCY10	5.53E-01	8.46E-02					
WNK2	2.60E-01	8.46E-02					
SPOCK2	-4.98E-01	8.47E-02					
CCDC17	3.35E-01	8.50E-02					
ZMYM6	2.08E-01	8.52E-02					
MTCL1	2.86E-01	8.55E-02					
AK2	-2.03E-01	8.55E-02					
FOPNL	2.27E-01	8.57E-02					
OSBPL2	-1.50E-01	8.58E-02					
MRPL58	-2.46E-01	8.63E-02					
KCTD15	-2.94E-01	8.65E-02					
NOXO1	-5.63E-01	8.69E-02					
FER1L5	-3.38E-01	8.73E-02					
LOC102397662	-6.13E-01	8.74E-02					
PAGR1	-2.86E-01	8.75E-02					
LOC102412798	-4.05E-01	8.79E-02					
FGF2	-2.84E-01	8.79E-02					
GNAT3	7.57E-01	8.79E-02					
TUSC1	-3.03E-01	8.83E-02					
PSMD10	-1.86E-01	8.83E-02					
PAX5	1.02E+00	8.84E-02					
TM9SF3	-2.24E-01	8.85E-02					
DPM2	-2.58E-01	8.85E-02					
ATP6V0C	1.90E-01	8.89E-02					
C16H11orf1	4.26E-01	8.97E-02					
LOC102391441	-2.66E-01	8.98E-02					
WIPF1	4.05E-01	8.99E-02					
LOC102395959	7.86E-01	9.04E-02					
LTBP2	-6.04E-01	9.06E-02					
FOSL1	-5.03E-01	9.06E-02					
GNA11	-1.85E-01	9.07E-02					
METRN	-5.92E-01	9.07E-02					

PCNX4	2.13E-01	9.08E-02					
LOC102390517	-2.36E-01	9.10E-02					
DGCR8	-1.85E-01	9.13E-02					
TOP1	-2.65E-01	9.15E-02					
RHBDL3	-5.26E-01	9.16E-02					
NAGPA	-2.01E-01	9.20E-02					
ARPC4	2.09E-01	9.25E-02					
PIP5KL1	4.99E-01	9.28E-02					
GDI2	1.89E-01	9.32E-02					
CARS2	-2.37E-01	9.33E-02					
XPA	-2.00E-01	9.34E-02					
PI4K2A	-1.65E-01	9.34E-02					
NOTCH4	-3.82E-01	9.36E-02					
HGS	1.64E-01	9.39E-02					
RFLNB	4.67E-01	9.40E-02					
IGF2R	-2.79E-01	9.44E-02					
VPS4B	1.87E-01	9.44E-02					
LOC102413340	-4.86E-01	9.46E-02					
LOC102392157	-6.54E-01	9.46E-02					
LYN	-7.51E-01	9.47E-02					
VWA5B2	-6.98E-01	9.49E-02					
USP3	-2.08E-01	9.54E-02					
RTF2	-2.41E-01	9.55E-02					
LOC102408337	-4.88E-01	9.55E-02					
AKR1B1	3.70E-01	9.59E-02					
ANKAR	5.40E-01	9.62E-02					
XKR6	5.83E-01	9.63E-02					
CSNK2A1	-1.48E-01	9.75E-02					
SLC5A5	-5.22E-01	9.79E-02					
DNAL1	4.11E-01	9.82E-02					
CLGN	2.32E-01	9.86E-02					
NME9	4.80E-01	9.87E-02					
ERCC2	-1.98E-01	9.88E-02					
CLYBL	2.35E-01	9.94E-02					
LOC112585239	-5.04E-01	1.00E-01					
CPSF3	-1.54E-01	1.01E-01					
RNF126	2.05E-01	1.01E-01					

CDC42EP5	-5.22E-01	1.01E-01
CUTA	-2.18E-01	1.01E-01
TET2	-2.09E-01	1.02E-01
JMJD1C	-2.49E-01	1.02E-01
GNPNAT1	1.63E-01	1.02E-01
CACTIN	-1.57E-01	1.02E-01
ZNF23	1.93E-01	1.02E-01
AFG1L	2.26E-01	1.03E-01
LOC102415675	-1.93E-01	1.03E-01
MASP1	-9.71E-01	1.03E-01
LOC102393963	-5.23E-01	1.03E-01
KANK2	2.51E-01	1.03E-01
CLHC1	3.31E-01	1.03E-01
LOC102389123	5.71E-01	1.03E-01
PELP1	2.41E-01	1.04E-01
LOC102413479	3.20E-01	1.05E-01
ERLIN1	1.68E-01	1.05E-01
TNFSF13	-4.90E-01	1.06E-01
C2H6orf136	-2.39E-01	1.06E-01
HSPA14	1.43E-01	1.07E-01
BCAS2	1.96E-01	1.08E-01
MRC1	7.28E-01	1.09E-01
FTL	3.10E-01	1.10E-01
CATSPERB	-4.57E-01	1.10E-01
NCF1	5.17E-01	1.10E-01
CNN1	-4.12E-01	1.10E-01
SLC7A8	-4.23E-01	1.11E-01
HUS1	-1.97E-01	1.11E-01
RANBP1	-2.86E-01	1.12E-01
GOLGA1	1.71E-01	1.12E-01
SPTY2D1	3.07E-01	1.12E-01
COPS3	1.47E-01	1.12E-01
SLC10A7	-1.68E-01	1.12E-01
RYR2	-2.49E-01	1.13E-01
LOC102406570	-3.38E-01	1.13E-01
COG4	-2.01E-01	1.13E-01
JAK1	-1.89E-01	1.13E-01
NCR3LG1	2.93E-01	1.14E-01

LRRFIP2	1.39E-01	1.14E-01
STT3B	1.73E-01	1.14E-01
CHML	-5.94E-01	1.14E-01
LOC102416306	5.88E-01	1.15E-01
ELMSAN1	-2.24E-01	1.15E-01
DCUN1D3	-2.67E-01	1.15E-01
LOC102397908	-3.91E-01	1.15E-01
NUFIP2	-2.28E-01	1.15E-01
LOC112577616	5.52E-01	1.16E-01
RMND5A	1.67E-01	1.16E-01
MED26	2.01E-01	1.16E-01
ETFRF1	2.86E-01	1.16E-01
LOC112583596	3.36E-01	1.16E-01
LOC102410930	-8.02E-01	1.17E-01
LOC112586898	3.08E-01	1.17E-01
LOC112586179	5.31E-01	1.17E-01
TM9SF2	1.48E-01	1.17E-01
SMCR8	2.59E-01	1.18E-01
CLPTM1L	1.67E-01	1.18E-01
PRDX2	-2.46E-01	1.18E-01
ZEB1	-3.14E-01	1.18E-01
FAM19A3	5.61E-01	1.19E-01
SCARF2	-6.61E-01	1.20E-01
SH3D19	-1.98E-01	1.20E-01
GLIS1	7.72E-01	1.21E-01
LOC102402715	2.19E-01	1.21E-01
MIIP	-1.98E-01	1.21E-01
SMNDC1	1.74E-01	1.21E-01
PYCR2	-1.89E-01	1.21E-01
SLC31A2	-3.85E-01	1.22E-01
RBM34	2.18E-01	1.22E-01
IRF7	2.23E-01	1.22E-01
LRRC72	-3.57E-01	1.22E-01
KDM4B	-1.58E-01	1.23E-01
RAMP1	-4.41E-01	1.23E-01
SLC20A2	-3.39E-01	1.23E-01
FAM221B	-7.83E-01	1.23E-01
FAM98A	-1.63E-01	1.23E-01

SETD3	-1.57E-01	1.23E-01
PRKCB	-7.70E-01	1.23E-01
MED16	1.58E-01	1.23E-01
MFAP3L	2.23E-01	1.23E-01
TTC32	-3.71E-01	1.24E-01
ZNF518A	2.71E-01	1.24E-01
LOC102394903	-2.99E-01	1.24E-01
ANKRD13B	-1.66E-01	1.25E-01
IPMK	3.01E-01	1.25E-01
PSMD1	1.44E-01	1.26E-01
KITLG	3.74E-01	1.26E-01
WDR70	1.68E-01	1.26E-01
VAV1	8.00E-01	1.27E-01
STON1	2.29E-01	1.27E-01
SCAMP3	1.50E-01	1.27E-01
TOP3B	1.61E-01	1.27E-01
LOC102393883	6.62E-01	1.28E-01
DNASE1	3.07E-01	1.28E-01
POMP	-2.07E-01	1.28E-01
KIF14	3.20E-01	1.28E-01
ZCWPW2	-2.31E-01	1.29E-01
KLHL13	3.08E-01	1.30E-01
STARD10	-3.04E-01	1.30E-01
C4H22orf23	4.38E-01	1.30E-01
MYBL2	2.16E-01	1.30E-01
C4H12orf57	-3.00E-01	1.31E-01
IFT22	-1.70E-01	1.31E-01
CAMK2A	-6.64E-01	1.31E-01
IGSF23	-5.57E-01	1.31E-01
ANKRA2	1.65E-01	1.32E-01
GALNTL6	2.38E-01	1.32E-01
KCND3	4.24E-01	1.32E-01
AMZ2	-1.99E-01	1.33E-01
TM7SF2	-2.88E-01	1.33E-01
NBN	-1.50E-01	1.33E-01
ARHGAP29	-2.27E-01	1.34E-01
KCNK1	2.19E-01	1.34E-01
GLP2R	5.26E-01	1.35E-01
KCNJ14	4.49E-01	1.35E-01
LOC102389658	-2.77E-	1.35E-01

	01						
ACP4	3.11E-01	1.35E-01					
FILIP1	2.66E-01	1.35E-01					
POLDIP2	1.80E-01	1.35E-01					
SMIM27	-3.94E-01	1.35E-01					
VAT1L	-3.40E-01	1.36E-01					
CDC26	-2.51E-01	1.36E-01					
C3H9orf40	1.93E-01	1.36E-01					
DCLRE1B	1.82E-01	1.37E-01					
BRWD1	-1.63E-01	1.37E-01					
PTP4A2	1.75E-01	1.38E-01					
PPFIBP1	2.73E-01	1.38E-01					
SEMA3A	-3.78E-01	1.38E-01					
LAGE3	-2.26E-01	1.39E-01					
PNMA1	2.05E-01	1.40E-01					
UPK1B	-2.73E-01	1.40E-01					
PGBD5	3.52E-01	1.41E-01					
PPP2R3A	-2.26E-01	1.41E-01					
JKAMP	1.57E-01	1.42E-01					
KNOP1	1.59E-01	1.42E-01					
LOC102390113	1.86E-01	1.42E-01					
RAB18	1.40E-01	1.43E-01					
PCDH15	7.70E-01	1.43E-01					
LOC102414041	-3.41E-01	1.43E-01					
R3HDM2	2.38E-01	1.44E-01					
SNX4	-2.85E-01	1.44E-01					
LOC102413280	1.57E-01	1.45E-01					
VSIG10	1.77E-01	1.46E-01					
CADM1	-2.11E-01	1.46E-01					
ZDHHC7	1.37E-01	1.46E-01					
DNAJC7	-1.59E-01	1.47E-01					
RPL7L1	-1.98E-01	1.47E-01					
RNPS1	-1.62E-01	1.47E-01					
DLG1	1.86E-01	1.48E-01					
TKFC	-1.98E-01	1.48E-01					
STAT2	-1.95E-01	1.49E-01					
DPH6	1.88E-01	1.49E-01					
TMEM82	5.19E-01	1.49E-01					
LIMD2	2.02E-01	1.49E-01					
AMN	4.28E-01	1.49E-01					

CCT5	1.96E-01	1.49E-01					
LOC102414919	2.06E-01	1.50E-01					
PGAM5	-1.71E-01	1.50E-01					
CCDC141	6.47E-01	1.50E-01					
NR0B1	-3.72E-01	1.50E-01					
ATPAF2	2.09E-01	1.52E-01					
SHB	-2.67E-01	1.52E-01					
CCNE2	-5.52E-01	1.52E-01					
PNKP	-1.84E-01	1.52E-01					
LOC112583399	6.53E-01	1.52E-01					
TUFM	-2.03E-01	1.52E-01					
RNASEH1	-1.78E-01	1.52E-01					
VPS11	-1.35E-01	1.52E-01					
TEX11	1.65E-01	1.53E-01					
DCDC1	-3.19E-01	1.53E-01					
UMAD1	3.82E-01	1.53E-01					
C10H6orf163	-4.77E-01	1.54E-01					
FSTL1	1.72E-01	1.56E-01					
SNX13	1.91E-01	1.57E-01					
VEPH1	3.27E-01	1.57E-01					
LOC102394527	-1.93E-01	1.57E-01					
GTSE1	1.81E-01	1.57E-01					
CEP104	1.80E-01	1.58E-01					
CACFD1	-2.05E-01	1.58E-01					
MEIS2	-2.55E-01	1.58E-01					
LOC102406024	-1.72E-01	1.58E-01					
LOC102393607	-6.92E-01	1.58E-01					
SLC33A1	-2.14E-01	1.58E-01					
PTP4A3	-4.29E-01	1.58E-01					
HMGB2	-2.50E-01	1.58E-01					
CCDC86	-1.95E-01	1.58E-01					
LOC102409300	-3.96E-01	1.58E-01					
MED9	-1.96E-01	1.59E-01					
ZBTB16	-3.02E-01	1.60E-01					
LOC102411316	3.77E-01	1.60E-01					
LMBR1L	-1.66E-01	1.60E-01					

SURF6	-2.12E-01	1.61E-01					
TRRAP	-2.37E-01	1.61E-01					
BTBD1	1.41E-01	1.61E-01					
LOC102390828	7.26E-01	1.62E-01					
UNG	1.26E-01	1.62E-01					
EPHA6	5.42E-01	1.62E-01					
EFNB3	3.27E-01	1.63E-01					
LOC102403665	-8.64E-01	1.63E-01					
ADPRM	2.03E-01	1.63E-01					
SH3PXD2A	-1.85E-01	1.64E-01					
LCP2	6.94E-01	1.64E-01					
LOC102409912	1.39E-01	1.64E-01					
LOC102390987	-2.08E-01	1.64E-01					
FAM78B	4.85E-01	1.65E-01					
ITGA3	1.58E-01	1.65E-01					
LOC112579500	6.71E-01	1.66E-01					
CDK17	1.89E-01	1.66E-01					
EIF4E3	-2.25E-01	1.66E-01					
AGFG2	1.60E-01	1.66E-01					
DRC3	3.73E-01	1.66E-01					
CPLX1	6.08E-01	1.67E-01					
JPT1	1.89E-01	1.67E-01					
LDLRAD4	2.93E-01	1.68E-01					
LOC112580313	-3.02E-01	1.68E-01					
TTC6	8.92E-01	1.70E-01					
LOC102411666	-3.45E-01	1.71E-01					
NBEAL2	-2.31E-01	1.72E-01					
LOC102390513	-5.05E-01	1.73E-01					
GXYLT1	1.75E-01	1.73E-01					
LOC102392455	-2.96E-01	1.74E-01					
SLC16A2	3.50E-01	1.74E-01					
ANXA13	-6.32E-01	1.75E-01					
MAML1	-2.13E-01	1.75E-01					
SLC11A2	-1.40E-01	1.76E-01					
UTP6	-1.47E-01	1.76E-01					
ZNF45	1.78E-01	1.76E-01					
LOC102408254	1.84E-01	1.76E-01					
RFT1	1.59E-01	1.77E-01					
WDR62	-1.74E-01	1.79E-01					
TRPA1	2.60E-01	1.79E-01					

LOC112582071	-4.01E-01	1.80E-01					
LOC102411496	-3.01E-01	1.80E-01					
RTKN	-3.19E-01	1.81E-01					
LOC102390387	-5.84E-01	1.82E-01					
STOML2	-1.85E-01	1.82E-01					
PPIG	1.18E-01	1.82E-01					
CCDC103	-5.55E-01	1.83E-01					
LOC102398210	-2.69E-01	1.83E-01					
SYMPK	-1.38E-01	1.83E-01					
DNAJB1	-3.92E-01	1.83E-01					
LOC102392339	-2.06E-01	1.84E-01					
LOC112577759	5.88E-01	1.84E-01					
ZPBP2	-4.56E-01	1.86E-01					
LOC112586239	4.44E-01	1.87E-01					
DYNLT1	2.00E-01	1.87E-01					
TGFB3	-3.25E-01	1.88E-01					
LOC102415186	-1.45E-01	1.89E-01					
DNAH14	-8.39E-01	1.89E-01					
TRAPPC3	1.87E-01	1.89E-01					
ADCYAP1	-3.65E-01	1.89E-01					
GK	-2.17E-01	1.89E-01					
ESCO1	1.92E-01	1.89E-01					
ABL1	-1.51E-01	1.89E-01					
MRPS17	2.43E-01	1.91E-01					
ADGRB2	4.05E-01	1.93E-01					
ACTL6A	1.61E-01	1.93E-01					
POGZ	-1.57E-01	1.94E-01					
KIAA0513	1.60E-01	1.94E-01					
LOC102391418	-2.40E-01	1.94E-01					
LOC112580619	7.07E-01	1.94E-01					
MRPL49	1.50E-01	1.94E-01					
ZNF862	-1.55E-01	1.94E-01					
WDR66	3.86E-01	1.94E-01					
DNAH3	-4.36E-01	1.95E-01					
ZNF362	-1.35E-01	1.95E-01					
USP14	1.19E-01	1.95E-01					
ANKRD52	1.96E-01	1.96E-01					

PITPNM1	-2.87E-01	1.98E-01					
LOC102403497	-1.74E-01	1.98E-01					
AGT	4.91E-01	1.98E-01					
CLK2	1.23E-01	1.98E-01					
TMBIM6	1.35E-01	2.01E-01					
SLC39A10	2.13E-01	2.01E-01					
HMGN4	1.32E-01	2.04E-01					
RDH11	1.31E-01	2.05E-01					
LSAMP	6.42E-01	2.05E-01					
LOC112587916	-5.00E-01	2.06E-01					
FAM217B	-3.88E-01	2.07E-01					
DNAJB12	1.13E-01	2.07E-01					
SV2B	-7.71E-01	2.08E-01					
MLX	1.32E-01	2.08E-01					
LOC112577646	-5.22E-01	2.09E-01					
FSIP2	3.48E-01	2.09E-01					
MTAP	-1.36E-01	2.09E-01					
TAMM41	1.89E-01	2.09E-01					
MPHOSPH10	1.49E-01	2.09E-01					
ZBTB40	-1.58E-01	2.10E-01					
SPDEF	-3.90E-01	2.10E-01					
LOC102404010	1.53E-01	2.10E-01					
LOC102389143	4.61E-01	2.10E-01					
MCCC1	1.38E-01	2.10E-01					
NOSIP	1.88E-01	2.10E-01					
UTP11	-1.36E-01	2.10E-01					
CNTNAP1	3.34E-01	2.10E-01					
VMA21	-1.84E-01	2.12E-01					
PRR15L	4.80E-01	2.13E-01					
GOLGA5	-1.81E-01	2.13E-01					
TBC1D14	1.10E-01	2.13E-01					
RPS6KA5	-1.37E-01	2.13E-01					
CHD8	1.56E-01	2.14E-01					
AAGAB	1.12E-01	2.14E-01					
ZNF451	1.38E-01	2.14E-01					
GSTO2	-4.34E-01	2.14E-01					
LOC102399398	1.27E-01	2.15E-01					
LOC102400533	-3.60E-01	2.16E-01					
EHD3	-3.55E-01	2.16E-01					
YJU2	1.71E-01	2.16E-01					

LOC102397853	4.12E-01	2.16E-01					
ELMOD2	1.62E-01	2.16E-01					
CITED2	-1.56E-01	2.16E-01					
DNAH7	7.13E-01	2.17E-01					
DISP1	-1.43E-01	2.17E-01					
PTCHD1	5.80E-01	2.17E-01					
ERG	-6.07E-01	2.17E-01					
LIG3	-1.59E-01	2.18E-01					
LOC102408106	-2.40E-01	2.19E-01					
FOXN2	-4.27E-01	2.19E-01					
MTRF1L	1.37E-01	2.20E-01					
TRIM4	-1.74E-01	2.20E-01					
ANGPTL6	-3.06E-01	2.20E-01					
COQ10B	-2.11E-01	2.21E-01					
STK11IP	1.43E-01	2.22E-01					
C22H18orf32	1.50E-01	2.23E-01					
ESRP2	1.47E-01	2.24E-01					
USP38	-1.81E-01	2.24E-01					
PRDM1	-2.68E-01	2.24E-01					
SORL1	1.62E-01	2.24E-01					
NFYB	1.24E-01	2.25E-01					
PYGB	1.19E-01	2.25E-01					
GAREM1	-1.92E-01	2.26E-01					
GRIN2C	4.44E-01	2.26E-01					
LOC102402161	-2.44E-01	2.27E-01					
ZFAND2A	3.36E-01	2.27E-01					
MYO7B	-4.31E-01	2.27E-01					
HGH1	-1.79E-01	2.28E-01					
LOC102389727	2.45E-01	2.28E-01					
C5H1orf53	-2.50E-01	2.28E-01					
CREB3L4	3.83E-01	2.28E-01					
LARGE1	1.14E-01	2.29E-01					
ZFAND4	1.80E-01	2.29E-01					
NRG3	3.00E-01	2.29E-01					
MCRS1	-1.34E-01	2.30E-01					
PRR5L	5.99E-01	2.30E-01					
SERPINC1	-4.28E-01	2.30E-01					
ACAP2	-2.10E-01	2.31E-01					

HSCB	1.84E-01	2.32E-01
SUPT6H	-1.14E-01	2.33E-01
HPRT1	-1.34E-01	2.33E-01
TRAPPC6B	-1.36E-01	2.33E-01
ATP2A1	-3.53E-01	2.35E-01
SNRPD3	2.02E-01	2.36E-01
OGG1	1.70E-01	2.36E-01
ZNF800	1.48E-01	2.38E-01
CCDC136	-2.24E-01	2.38E-01
SFT2D3	-2.10E-01	2.39E-01
LOC102404008	-4.48E-01	2.39E-01
TSSK2	-3.75E-01	2.39E-01
LOC102400025	9.79E-02	2.39E-01
AKAP5	-4.40E-01	2.40E-01
MUL1	1.43E-01	2.40E-01
SFXN1	-2.13E-01	2.41E-01
UPP2	-3.45E-01	2.42E-01
DCAF6	1.08E-01	2.42E-01
ATP6V0A1	1.41E-01	2.43E-01
RAD9B	4.66E-01	2.43E-01
RNF44	-1.76E-01	2.44E-01
HOXD4	2.13E-01	2.44E-01
KIF9	2.21E-01	2.44E-01
FAM131B	4.74E-01	2.45E-01
RYR3	2.39E-01	2.45E-01
ZDHHC12	-1.77E-01	2.45E-01
GRIN3A	6.46E-01	2.45E-01
C4H12orf29	1.73E-01	2.46E-01
ZNF593	-2.18E-01	2.47E-01
LOC102395098	3.89E-01	2.48E-01
TBC1D1	-1.25E-01	2.48E-01
PHF1	-1.25E-01	2.49E-01
LOC102415917	1.57E-01	2.50E-01
PURG	-1.75E-01	2.50E-01
GPKOW	1.46E-01	2.50E-01
KRT19	6.38E-01	2.50E-01
SQSTM1	-1.61E-01	2.51E-01
AHR	2.81E-01	2.51E-01
CDC37	-1.49E-	2.51E-01

	01							
THYN1	1.83E-01	2.52E-01						
NEK7	-1.67E-01	2.52E-01						
OSCP1	2.06E-01	2.52E-01						
BMP4	2.91E-01	2.52E-01						
LOC112585577	3.46E-01	2.52E-01						
BECN1	-1.07E-01	2.54E-01						
HDHD2	1.24E-01	2.54E-01						
MMP24OS	2.38E-01	2.54E-01						
GPATCH4	2.09E-01	2.54E-01						
HTR1E	6.46E-01	2.58E-01						
GHDC	1.56E-01	2.58E-01						
UFM1	-1.41E-01	2.60E-01						
MRRF	-1.63E-01	2.60E-01						
ODF2	1.19E-01	2.60E-01						
LOC112587446	3.42E-01	2.61E-01						
LOC102397920	-6.32E-01	2.61E-01						
BNIP1	1.21E-01	2.62E-01						
PQBP1	1.75E-01	2.62E-01						
LCMT1	1.38E-01	2.64E-01						
RPS6KL1	-2.20E-01	2.64E-01						
RLIM	-1.51E-01	2.64E-01						
KCTD21	-1.87E-01	2.66E-01						
GRTP1	-3.86E-01	2.66E-01						
SHROOM4	2.34E-01	2.67E-01						
PTPRU	4.77E-01	2.67E-01						
MIF4GD	-1.41E-01	2.68E-01						
TMEM45B	3.94E-01	2.68E-01						
LOC102399590	3.04E-01	2.68E-01						
SCYL2	1.39E-01	2.68E-01						
GPD1L	1.40E-01	2.69E-01						
LSM7	-2.11E-01	2.70E-01						
CPLANE1	1.48E-01	2.71E-01						
TMCC1	2.15E-01	2.72E-01						
DNAJC4	-1.89E-01	2.74E-01						
LOC102407917	2.50E-01	2.74E-01						
AP1S2	1.16E-01	2.74E-01						
APLN	-3.54E-01	2.74E-01						
FAM110B	1.60E-01	2.75E-01						
ARSB	-1.34E-01	2.75E-01						
PPP5C	1.26E-01	2.75E-01						

KLHL42	-1.27E-01	2.76E-01					
LMNA	-1.55E-01	2.76E-01					
KIAA1549	-1.29E-01	2.77E-01					
NT5DC3	2.86E-01	2.77E-01					
LOC102392263	-1.58E-01	2.77E-01					
GAS8	-2.08E-01	2.78E-01					
NRG1	-5.74E-01	2.78E-01					
PARVB	3.99E-01	2.79E-01					
NAP1L4	-1.03E-01	2.80E-01					
NCOA3	-1.66E-01	2.80E-01					
ZNF76	-1.31E-01	2.80E-01					
ANO4	1.47E-01	2.81E-01					
DTD2	-2.12E-01	2.81E-01					
CIR1	1.21E-01	2.81E-01					
CCDC155	3.11E-01	2.81E-01					
PET100	-2.40E-01	2.81E-01					
SLC5A10	2.51E-01	2.82E-01					
LOC112578733	4.29E-01	2.83E-01					
ARHGAP8	2.07E-01	2.83E-01					
LOC112578855	5.76E-01	2.85E-01					
CD19	-3.73E-01	2.85E-01					
CHMP3	-1.18E-01	2.86E-01					
SLIRP	-1.97E-01	2.87E-01					
PRPSAP1	9.64E-02	2.87E-01					
LOC112578564	3.86E-01	2.87E-01					
HLCS	-1.43E-01	2.87E-01					
LOC102410239	6.66E-01	2.88E-01					
LOC102408976	2.26E-01	2.88E-01					
BOP1	-1.55E-01	2.90E-01					
OSMR	-2.69E-01	2.92E-01					
SFMBT2	2.50E-01	2.92E-01					
ITFG2	-1.27E-01	2.92E-01					
RBMS3	-1.88E-01	2.95E-01					
EIF3L	-1.19E-01	2.96E-01					
CEMIP2	2.05E-01	2.96E-01					
DNASE1L2	-3.02E-01	2.96E-01					
SLC35F1	3.54E-01	2.98E-01					

UBALD1	1.55E-01	2.98E-01					
PMM2	1.15E-01	2.99E-01					
TFPT	-1.91E-01	2.99E-01					
SDHAF1	-1.80E-01	3.01E-01					
LOC102404005	4.62E-01	3.01E-01					
MAP3K21	2.05E-01	3.02E-01					
FAM214A	-1.24E-01	3.02E-01					
NEPRO	-1.38E-01	3.03E-01					
THAP8	1.41E-01	3.03E-01					
MARCH7	-1.35E-01	3.04E-01					
HERC1	1.57E-01	3.04E-01					
GGT7	-2.18E-01	3.05E-01					
BAG4	1.74E-01	3.05E-01					
NCKAP1L	-4.84E-01	3.06E-01					
ATG2A	1.44E-01	3.07E-01					
GSK3B	-1.16E-01	3.07E-01					
NOTCH1	-3.79E-01	3.07E-01					
ANXA11	9.42E-02	3.07E-01					
INPP4A	-1.45E-01	3.07E-01					
LOC112580427	-1.30E-01	3.08E-01					
IBTK	1.04E-01	3.09E-01					
PACRG	-4.92E-01	3.10E-01					
SLC35E1	1.01E-01	3.10E-01					
LOC112586098	6.89E-01	3.11E-01					
ZNF554	2.87E-01	3.12E-01					
HOMER3	-1.49E-01	3.12E-01					
CBFA2T3	-3.95E-01	3.13E-01					
MED21	1.34E-01	3.13E-01					
MITF	-2.34E-01	3.13E-01					
CLNK	5.80E-01	3.14E-01					
ANKS6	1.49E-01	3.14E-01					
ATG2B	1.61E-01	3.15E-01					
MAST2	9.99E-02	3.15E-01					
NDST3	5.07E-01	3.16E-01					
SNRNP48	-1.34E-01	3.16E-01					
MVB12B	-1.31E-01	3.16E-01					
AP3M1	1.04E-01	3.18E-01					
DNAH1	2.11E-01	3.18E-01					
MCM8	1.63E-01	3.18E-01					

ATG101	-1.41E-01	3.20E-01					
SRP19	1.14E-01	3.21E-01					
PPP1R11	-1.14E-01	3.21E-01					
ABCA1	-3.14E-01	3.22E-01					
SMIM26	-1.89E-01	3.23E-01					
FAT2	4.59E-01	3.23E-01					
HSPBAP1	1.52E-01	3.23E-01					
NUDCD2	1.54E-01	3.24E-01					
ASCC3	-1.23E-01	3.25E-01					
ZNF579	-1.50E-01	3.25E-01					
TBL1XR1	1.25E-01	3.26E-01					
ACTR2	1.13E-01	3.27E-01					
ZCRB1	-1.31E-01	3.27E-01					
AFTPH	-1.44E-01	3.28E-01					
CCDC163	-2.65E-01	3.30E-01					
GUCY1B1	-2.37E-01	3.31E-01					
FERMT2	-2.68E-01	3.32E-01					
CASKIN2	1.10E-01	3.32E-01					
RTRAF	1.54E-01	3.32E-01					
OXTR	-2.98E-01	3.33E-01					
GNL3	-1.16E-01	3.34E-01					
EI24	1.05E-01	3.34E-01					
NDUFA6	1.58E-01	3.35E-01					
MRPL48	1.61E-01	3.36E-01					
ACOT12	3.58E-01	3.36E-01					
DDX18	-1.12E-01	3.36E-01					
PCDH1	-4.98E-01	3.37E-01					
ECD	9.29E-02	3.37E-01					
ANKRD16	-1.71E-01	3.38E-01					
SHMT1	-2.25E-01	3.40E-01					
FAM135A	-1.45E-01	3.40E-01					
WDR59	-1.19E-01	3.40E-01					
ATL2	8.88E-02	3.41E-01					
LOC112578447	7.84E-01	3.41E-01					
LPAR3	3.16E-01	3.42E-01					
TRIM46	-2.61E-01	3.42E-01					
S100A16	1.66E-01	3.44E-01					
ZPR1	-1.10E-	3.44E-01					

	01						
ASIC2	5.49E-01	3.44E-01					
XYLB	-2.70E-01	3.45E-01					
DPCD	1.52E-01	3.45E-01					
ADGRA2	-1.96E-01	3.45E-01					
AXIN1	9.88E-02	3.46E-01					
ANO2	-4.26E-01	3.48E-01					
LOC112585766	2.40E-01	3.49E-01					
FAM102A	-1.98E-01	3.49E-01					
SCFD2	1.20E-01	3.51E-01					
DNAJC11	-8.42E-02	3.52E-01					
IMP4	-1.21E-01	3.53E-01					
CA2	-2.56E-01	3.53E-01					
TMEM237	-1.58E-01	3.53E-01					
IGFBP3	2.08E-01	3.54E-01					
EGFL7	4.80E-01	3.54E-01					
LOC102406196	-3.96E-01	3.55E-01					
UBA6	-9.00E-02	3.55E-01					
SLC44A4	-4.98E-01	3.56E-01					
KCTD17	-1.80E-01	3.56E-01					
ARHGAP35	-1.18E-01	3.57E-01					
LOC112587015	-3.38E-01	3.57E-01					
TUBGCP5	1.25E-01	3.57E-01					
RPTOR	8.46E-02	3.59E-01					
REXO4	1.29E-01	3.61E-01					
BICD2	-1.19E-01	3.62E-01					
BOLA1	1.49E-01	3.62E-01					
JRK	1.15E-01	3.63E-01					
ITPA	-1.33E-01	3.64E-01					
GUCY1A2	4.28E-01	3.64E-01					
ELMOD1	1.86E-01	3.66E-01					
SNIP1	1.19E-01	3.67E-01					
LATS2	-2.03E-01	3.68E-01					
KIAA1107	1.50E-01	3.69E-01					
OSBP	9.55E-02	3.69E-01					
SDR42E1	1.30E-01	3.70E-01					
C19H5orf22	-1.19E-01	3.73E-01					
ZNF852	1.97E-01	3.75E-01					
RAD51D	1.33E-01	3.75E-01					

OXT	-2.30E-01	3.75E-01					
CCDC107	1.73E-01	3.75E-01					
PLPPR5	-3.08E-01	3.76E-01					
CACHD1	-1.15E-01	3.77E-01					
EEF1AKMT1	-9.48E-02	3.77E-01					
CCDC73	1.64E-01	3.78E-01					
CCDC151	1.47E-01	3.79E-01					
LAP3	-8.94E-02	3.82E-01					
SIRT7	1.11E-01	3.82E-01					
RECQL5	1.15E-01	3.82E-01					
HIRIP3	-1.26E-01	3.83E-01					
TASP1	-1.20E-01	3.85E-01					
LOC102403045	-2.37E-01	3.86E-01					
LOC102413604	2.64E-01	3.88E-01					
CDK5RAP2	9.59E-02	3.88E-01					
NAT14	-1.29E-01	3.89E-01					
AMMECR1L	-1.11E-01	3.89E-01					
ITIH2	-3.50E-01	3.89E-01					
LOC102416509	-3.47E-01	3.90E-01					
CALM1	-1.05E-01	3.91E-01					
HOXC4	-1.87E-01	3.92E-01					
LOC112584668	-4.54E-01	3.93E-01					
FAM173B	8.89E-02	3.93E-01					
ADCY8	4.00E-01	3.94E-01					
ARHGAP39	1.10E-01	3.95E-01					
LOC112587753	4.43E-01	3.97E-01					
CDK9	9.54E-02	3.97E-01					
HAS3	1.25E-01	3.98E-01					
POLR2G	-1.36E-01	3.99E-01					
LOC112579702	-3.83E-01	3.99E-01					
HBS1L	1.02E-01	3.99E-01					
HEATR6	8.46E-02	4.00E-01					
ABRAXAS1	1.03E-01	4.01E-01					
PARN	7.73E-02	4.03E-01					
LOC102403928	-2.65E-01	4.03E-01					
SF1	1.06E-01	4.03E-01					
NOP14	-9.88E-02	4.03E-01					
SLC10A6	2.92E-01	4.03E-01					

TUBGCP3	9.34E-02	4.05E-01					
SNPH	-2.61E-01	4.05E-01					
LOC112581723	-2.83E-01	4.05E-01					
C22H18orf54	1.36E-01	4.06E-01					
NANP	-1.21E-01	4.07E-01					
PDCD11	-8.86E-02	4.07E-01					
HSPH1	-1.61E-01	4.08E-01					
LOC112587597	-3.30E-01	4.09E-01					
TBC1D32	-1.55E-01	4.09E-01					
EXOSC2	9.25E-02	4.09E-01					
SH2D5	-1.41E-01	4.09E-01					
LOC102393658	-1.72E-01	4.09E-01					
EHD4	-1.89E-01	4.10E-01					
ODR4	-9.17E-02	4.10E-01					
IREB2	1.03E-01	4.11E-01					
CLTA	-1.12E-01	4.11E-01					
MSTO1	-1.25E-01	4.12E-01					
LOC102412377	-3.31E-01	4.13E-01					
PROX1	-2.28E-01	4.13E-01					
AP5B1	1.19E-01	4.14E-01					
FCER2	3.31E-01	4.14E-01					
FAM221A	2.97E-01	4.15E-01					
SLC16A1	-7.46E-02	4.16E-01					
ATXN1L	-1.29E-01	4.16E-01					
LOC102390690	-1.25E-01	4.18E-01					
GFAP	2.49E-01	4.18E-01					
KDM3A	8.61E-02	4.18E-01					
RPF1	-9.92E-02	4.18E-01					
GPR45	2.51E-01	4.19E-01					
ACAT2	1.17E-01	4.19E-01					
PCCB	-1.21E-01	4.20E-01					
ABCB9	1.73E-01	4.20E-01					
CDK14	-1.39E-01	4.20E-01					
DUSP23	1.96E-01	4.21E-01					
PPFIA1	9.31E-02	4.22E-01					
MRPL14	1.23E-01	4.23E-01					
LOC102407213	1.97E-01	4.23E-01					

FAM172A	1.09E-01	4.23E-01					
PIK3R2	1.49E-01	4.24E-01					
TFEB	-1.92E-01	4.24E-01					
MORC4	-1.03E-01	4.27E-01					
ERP44	7.75E-02	4.28E-01					
LOC102393778	-1.41E-01	4.29E-01					
DTWD2	2.20E-01	4.29E-01					
LOC112582123	-6.87E-01	4.30E-01					
TJP2	1.15E-01	4.31E-01					
UNC119B	8.97E-02	4.31E-01					
LOC102415868	-1.37E-01	4.32E-01					
NTAN1	-9.85E-02	4.32E-01					
POLR3A	-8.05E-02	4.32E-01					
PDGFD	2.24E-01	4.33E-01					
NTPCR	9.44E-02	4.33E-01					
REV3L	1.16E-01	4.34E-01					
LARP1	9.74E-02	4.34E-01					
NDRG3	-7.40E-02	4.34E-01					
CCDC178	-3.07E-01	4.34E-01					
SLC39A6	8.81E-02	4.35E-01					
KANK3	-1.83E-01	4.35E-01					
CCDC122	-2.67E-01	4.36E-01					
HYOU1	-1.21E-01	4.36E-01					
TRAF7	8.50E-02	4.37E-01					
UROS	1.30E-01	4.38E-01					
GCLM	-9.43E-02	4.38E-01					
CRCP	-1.26E-01	4.40E-01					
TPRN	1.12E-01	4.41E-01					
SLC6A6	3.51E-01	4.41E-01					
MIEF2	1.18E-01	4.41E-01					
ESS2	1.18E-01	4.41E-01					
LOC112581146	2.77E-01	4.43E-01					
ZGRF1	1.02E-01	4.45E-01					
LOC102414911	2.78E-01	4.46E-01					
ITGAL	-3.84E-01	4.49E-01					
SLC16A5	-1.19E-01	4.51E-01					
HAGHL	1.32E-01	4.51E-01					
COMMD4	-1.27E-01	4.51E-01					
TTC5	8.56E-02	4.52E-01					

MGA	-1.06E-01	4.52E-01					
NAALADL1	1.32E-01	4.52E-01					
C15H8orf89	1.38E-01	4.53E-01					
ZZZ3	9.10E-02	4.53E-01					
ADAM10	8.19E-02	4.53E-01					
FADS3	-1.60E-01	4.54E-01					
ADIPOR2	-9.82E-02	4.55E-01					
FGD4	-1.55E-01	4.55E-01					
TMEM192	9.13E-02	4.55E-01					
LOC112581113	3.90E-01	4.56E-01					
SCTR	3.53E-01	4.56E-01					
VPS13C	1.60E-01	4.57E-01					
LOC102412447	3.78E-01	4.58E-01					
POLD2	-1.14E-01	4.62E-01					
TBX3	2.30E-01	4.62E-01					
LNPK	1.00E-01	4.64E-01					
KLF4	2.95E-01	4.64E-01					
HES7	2.30E-01	4.68E-01					
DYNLRB2	1.47E-01	4.68E-01					
LOC102415829	-7.57E-02	4.68E-01					
BRCC3	6.55E-02	4.69E-01					
ITGB1BP1	8.04E-02	4.69E-01					
GMFG	3.03E-01	4.70E-01					
RNF113A	1.20E-01	4.71E-01					
SLC37A3	8.76E-02	4.71E-01					
STIL	1.38E-01	4.71E-01					
VANGL2	2.21E-01	4.72E-01					
MIER2	-7.78E-02	4.73E-01					
JAGN1	-9.82E-02	4.75E-01					
ZDHHC6	6.65E-02	4.77E-01					
MINK1	-7.23E-02	4.78E-01					
E2F7	-1.52E-01	4.78E-01					
RASA4B	-9.17E-02	4.78E-01					
TRIM54	-2.43E-01	4.79E-01					
LOC102399418	2.51E-01	4.80E-01					
CEP63	7.57E-02	4.81E-01					
LOC112581569	3.77E-01	4.81E-01					
CD109	1.90E-01	4.81E-01					
TONSL	-1.02E-01	4.82E-01					
GTF2H5	1.08E-01	4.83E-01					
SMIM11A	-1.33E-01	4.83E-01					

NUDT9	-7.56E-02	4.83E-01						
BASP1	1.08E-01	4.84E-01						
LOC102415962	1.60E-01	4.84E-01						
PHACTR4	8.77E-02	4.85E-01						
EZH1	7.12E-02	4.85E-01						
HMGCLL1	-3.91E-01	4.86E-01						
ATP6AP2	-8.26E-02	4.86E-01						
LOC112584008	-1.71E-01	4.87E-01						
ZNHIT3	1.18E-01	4.87E-01						
NUDT8	-1.63E-01	4.87E-01						
ARNTL2	-2.32E-01	4.88E-01						
C6H1orf50	-8.61E-02	4.88E-01						
SEC61A1	-8.32E-02	4.89E-01						
CDH20	-4.48E-01	4.89E-01						
EIF3B	-7.37E-02	4.90E-01						
SND1	-7.30E-02	4.91E-01						
CD247	-1.17E-01	4.91E-01						
MCF2L	2.12E-01	4.93E-01						
DPM1	8.37E-02	4.94E-01						
PPP2CB	6.92E-02	4.94E-01						
RPL26L1	9.45E-02	4.95E-01						
KDM5A	-8.82E-02	4.95E-01						
SHQ1	-8.06E-02	4.95E-01						
LOC102397977	2.66E-01	4.95E-01						
PAM16	1.22E-01	4.96E-01						
DPH5	8.44E-02	4.97E-01						
LOC102408653	1.61E-01	4.99E-01						
VAPA	6.21E-02	4.99E-01						
DPP10	3.37E-01	4.99E-01						
PDZD9	2.03E-01	5.00E-01						
EFHC2	1.31E-01	5.01E-01						
NIM1K	-2.15E-01	5.01E-01						
GFRA1	-2.48E-01	5.03E-01						
M1AP	-2.98E-01	5.04E-01						
LRIG3	1.13E-01	5.04E-01						
APOLD1	-2.54E-01	5.04E-01						
LOC102402857	-2.15E-01	5.06E-01						
CPNE4	1.40E-01	5.07E-01						

HIC1	1.73E-01	5.07E-01					
DGKH	-1.38E-01	5.07E-01					
SFRP1	-1.51E-01	5.08E-01					
TMEM184B	-7.96E-02	5.08E-01					
TSPAN18	-1.58E-01	5.08E-01					
CRLS1	7.48E-02	5.09E-01					
CDC20	-1.27E-01	5.09E-01					
PRDX5	-1.07E-01	5.10E-01					
LOC102413417	2.07E-01	5.10E-01					
CHIC2	8.27E-02	5.11E-01					
LOC112581369	-2.12E-01	5.15E-01					
LIN52	-7.51E-02	5.16E-01					
INSRR	2.12E-01	5.18E-01					
FCER1G	2.61E-01	5.20E-01					
RUVBL1	9.49E-02	5.20E-01					
CACNB3	-8.04E-02	5.21E-01					
MPDU1	8.25E-02	5.21E-01					
NWD1	-2.81E-01	5.23E-01					
CCDC188	-1.73E-01	5.24E-01					
SULT1C4	-2.47E-01	5.25E-01					
TRDN	3.86E-01	5.25E-01					
CLIP3	7.64E-02	5.25E-01					
EIF3H	7.83E-02	5.26E-01					
NTNG2	2.05E-01	5.27E-01					
SH3D21	-1.45E-01	5.28E-01					
LOC102397874	1.95E-01	5.28E-01					
ABCA2	-8.90E-02	5.29E-01					
BIRC5	9.05E-02	5.29E-01					
PPCDC	1.08E-01	5.29E-01					
ZNF384	6.14E-02	5.29E-01					
LMAN2L	-7.97E-02	5.29E-01					
KIF13B	8.50E-02	5.30E-01					
PCMT1	-7.82E-02	5.30E-01					
LTN1	-1.06E-01	5.30E-01					
LOC102399668	8.95E-02	5.30E-01					
SEPHS2	-6.90E-02	5.30E-01					
PRDM16	2.24E-01	5.32E-01					
MILR1	-2.73E-01	5.33E-01					

EIF2B3	-8.94E-02	5.33E-01
KHDRBS2	-2.88E-01	5.34E-01
EXOC3	5.40E-02	5.35E-01
DPYSL4	-1.76E-01	5.36E-01
PJA2	-8.21E-02	5.38E-01
PCIF1	6.66E-02	5.40E-01
LNPEP	-6.97E-02	5.41E-01
GIMAP8	1.61E-01	5.41E-01
ATAD5	-1.25E-01	5.42E-01
LRRC58	2.14E-01	5.43E-01
NTRK3	-3.24E-01	5.44E-01
EDC3	6.06E-02	5.46E-01
LOC112587459	-2.26E-01	5.48E-01
API5	1.27E-01	5.48E-01
SLC39A5	-1.64E-01	5.48E-01
LOC112586864	2.42E-01	5.48E-01
LOC112586835	-1.94E-01	5.51E-01
NKRF	1.29E-01	5.51E-01
ASB13	-1.22E-01	5.51E-01
SLC37A4	-6.65E-02	5.51E-01
HMCN2	2.17E-01	5.52E-01
SLC9A3R1	8.33E-02	5.53E-01
MARCH3	-1.55E-01	5.56E-01
ZNF408	-6.51E-02	5.56E-01
LOC102399450	-2.28E-01	5.56E-01
LOC102415348	1.06E-01	5.57E-01
TRMT5	-1.03E-01	5.59E-01
CDC34	-7.12E-02	5.59E-01
DIP2B	8.85E-02	5.59E-01
MYRIP	-1.55E-01	5.60E-01
AJAP1	2.56E-01	5.60E-01
CEP83	-6.35E-02	5.60E-01
DGKK	3.59E-01	5.60E-01
APPL1	-1.40E-01	5.60E-01
RWDD3	-8.54E-02	5.60E-01
SPRY4	1.92E-01	5.62E-01
PTDSS1	5.50E-02	5.62E-01

ITGA11	1.97E-01	5.65E-01					
RRM1	5.67E-02	5.65E-01					
DOCK6	9.27E-02	5.67E-01					
C15H8orf59	1.07E-01	5.67E-01					
LOC112587945	-1.90E-01	5.67E-01					
EFR3A	7.25E-02	5.69E-01					
PAXIP1	7.42E-02	5.70E-01					
NUDT22	1.03E-01	5.71E-01					
TIMM17A	6.63E-02	5.73E-01					
SHLD1	-9.29E-02	5.74E-01					
VPS26B	6.34E-02	5.74E-01					
LOC112583714	1.66E-01	5.76E-01					
NEK6	-1.89E-01	5.76E-01					
NSFL1C	-8.09E-02	5.77E-01					
MGAT2	-6.84E-02	5.77E-01					
LOC112580012	1.86E-01	5.77E-01					
CCDC157	1.08E-01	5.77E-01					
PLGRKT	9.40E-02	5.78E-01					
KCNQ5	-2.60E-01	5.78E-01					
ANKRD53	1.18E-01	5.79E-01					
TMEM181	-6.15E-02	5.79E-01					
LOC112584570	-1.43E-01	5.80E-01					
CD96	3.73E-01	5.81E-01					
LOC112578706	1.91E-01	5.81E-01					
LOC102397167	-2.00E-01	5.82E-01					
NOLC1	-6.07E-02	5.83E-01					
ADGRF5	-2.88E-01	5.83E-01					
TENM1	-1.22E-01	5.84E-01					
PEX19	5.95E-02	5.84E-01					
UBE2V2	-5.52E-02	5.85E-01					
ROBO1	-7.31E-02	5.86E-01					
LOC102403810	1.22E-01	5.88E-01					
NUS1	-5.52E-02	5.88E-01					
NAA38	1.06E-01	5.88E-01					
ITGB2	-3.09E-01	5.88E-01					
LOC102395906	1.87E-01	5.88E-01					
ZNF214	1.53E-01	5.89E-01					
HCLS1	-3.32E-01	5.90E-01					
LOC112577789	-8.08E-02	5.90E-01					

GPC3	-7.93E-02	5.90E-01
BRD8	5.30E-02	5.90E-01
LOC102401654	1.24E-01	5.91E-01
PKD2L2	2.26E-01	5.91E-01
ARPC5L	-5.60E-02	5.93E-01
NPTN	-5.07E-02	5.93E-01
ATG16L1	-8.04E-02	5.94E-01
DRAP1	-8.06E-02	5.95E-01
RAB8A	5.97E-02	5.96E-01
CIAPIN1	-5.71E-02	5.96E-01
LOC112585691	1.50E-01	5.96E-01
CADM2	-1.98E-01	5.97E-01
LOC102414236	1.80E-01	5.97E-01
PPARA	8.28E-02	5.97E-01
LOC102390897	1.96E-01	5.97E-01
MCM5	8.65E-02	5.98E-01
MYH6	1.40E-01	5.98E-01
USP30	7.72E-02	5.99E-01
TCF3	5.36E-02	5.99E-01
TRAK2	8.14E-02	5.99E-01
KIFC1	7.59E-02	6.01E-01
WDYHV1	5.52E-02	6.01E-01
DNAL4	6.60E-02	6.01E-01
LOC112582099	-1.48E-01	6.01E-01
LOC102406915	-2.36E-01	6.05E-01
C2H6orf226	-8.93E-02	6.06E-01
ING1	-7.54E-02	6.06E-01
SOX6	-1.74E-01	6.07E-01
MPV17L2	7.66E-02	6.07E-01
ATXN2L	7.78E-02	6.08E-01
RCC1L	6.81E-02	6.08E-01
YKT6	5.69E-02	6.09E-01
LOC112580842	1.38E-01	6.10E-01
DNM1	-9.64E-02	6.10E-01
PPP4R1	5.55E-02	6.11E-01
NRDC	4.35E-02	6.12E-01
PEX10	7.07E-02	6.12E-01
ANOS1	2.58E-01	6.12E-01
XPNPEP1	4.29E-02	6.13E-01
PAFAH1B2	-4.37E-02	6.13E-01
HMCN1	-8.94E-	6.15E-01

	02						
ABCB11	4.16E-01	6.15E-01					
CERS6	-1.70E-01	6.15E-01					
AKAP17A	6.12E-02	6.16E-01					
NRROS	2.54E-01	6.17E-01					
PSRC1	7.77E-02	6.17E-01					
MOB2	7.22E-02	6.17E-01					
ALPK2	-1.34E-01	6.18E-01					
ACKR3	-3.66E-01	6.19E-01					
SNTA1	-9.76E-02	6.20E-01					
SEC24B	-5.76E-02	6.20E-01					
SYT5	-1.07E-01	6.22E-01					
TNFSF8	2.15E-01	6.22E-01					
TCEANC	-9.66E-02	6.25E-01					
ELP6	-1.01E-01	6.26E-01					
LOC102398677	2.14E-01	6.27E-01					
TENT4B	-7.74E-02	6.27E-01					
SSBP3	-5.30E-02	6.30E-01					
TP53BP2	5.86E-02	6.31E-01					
MYO19	-6.43E-02	6.31E-01					
STARD4	-1.01E-01	6.32E-01					
LOC102400622	-1.11E-01	6.32E-01					
FAM84B	-1.30E-01	6.37E-01					
HGSNAT	-4.67E-02	6.37E-01					
PRKAA1	8.22E-02	6.38E-01					
TOMM7	7.71E-02	6.40E-01					
GSTK1	1.47E-01	6.41E-01					
LOC112585253	1.38E-01	6.41E-01					
LOC102396719	-7.89E-02	6.41E-01					
TRIM41	5.45E-02	6.43E-01					
CLDN7	-1.83E-01	6.45E-01					
PRRC2A	5.98E-02	6.45E-01					
CSKMT	9.32E-02	6.45E-01					
KRBA2	-7.03E-02	6.47E-01					
ND6	1.02E-01	6.48E-01					
HS6ST3	-1.54E-01	6.48E-01					
SRSF12	6.42E-02	6.49E-01					
VPS37C	7.88E-02	6.49E-01					

LOC102394843	2.31E-01	6.50E-01					
USP45	-1.13E-01	6.52E-01					
DMAC2	5.25E-02	6.54E-01					
SETBP1	1.99E-01	6.54E-01					
AURKB	-7.47E-02	6.55E-01					
LOC102395459	-1.58E-01	6.55E-01					
KLK15	-1.97E-01	6.55E-01					
GOLGA4	-5.64E-02	6.56E-01					
IL1RAPL1	1.19E-01	6.56E-01					
LRRC6	7.54E-02	6.57E-01					
TSGA10IP	1.34E-01	6.58E-01					
DCTN4	-4.34E-02	6.59E-01					
SIRT6	-6.98E-02	6.59E-01					
BEND7	5.26E-02	6.61E-01					
LOC102396145	-1.96E-01	6.61E-01					
DNAH10	1.56E-01	6.61E-01					
LOC102412910	-1.00E-01	6.61E-01					
DNAH2	1.49E-01	6.62E-01					
RDM1	1.26E-01	6.63E-01					
HACD3	3.83E-02	6.64E-01					
SH3GL1	-4.67E-02	6.65E-01					
KANSL1	-4.61E-02	6.65E-01					
FAM13A	-4.20E-02	6.65E-01					
HCN1	-2.67E-01	6.65E-01					
LOC102401082	1.93E-01	6.68E-01					
LOC112578039	1.48E-01	6.70E-01					
ZACN	-8.80E-02	6.70E-01					
THAP2	-6.21E-02	6.71E-01					
GLI4	-7.05E-02	6.72E-01					
LOC102411708	-7.55E-02	6.73E-01					
ZNF112	-1.07E-01	6.73E-01					
SLC30A3	-1.06E-01	6.74E-01					
TRPC6	9.39E-02	6.74E-01					
PABPC5	1.24E-01	6.74E-01					
ANKS1B	6.18E-02	6.75E-01					
FBXO6	6.24E-02	6.76E-01					
ADAM11	1.09E-01	6.76E-01					
C1GALT1C1	4.86E-02	6.76E-01					

AGTRAP	-6.65E-02	6.80E-01					
LOC102402353	9.38E-02	6.82E-01					
LOC102415556	-3.21E-01	6.83E-01					
CACNA1S	-1.37E-01	6.83E-01					
S100B	-1.34E-01	6.84E-01					
PSMB5	-6.46E-02	6.84E-01					
PPFIA3	-9.88E-02	6.85E-01					
NEXN	-7.71E-02	6.88E-01					
CPNE5	-1.29E-01	6.90E-01					
MLLT3	4.24E-02	6.90E-01					
CISD1	7.11E-02	6.90E-01					
RAB40B	-1.48E-01	6.92E-01					
LOC102392710	7.00E-02	6.92E-01					
ADAM22	1.76E-01	6.94E-01					
LOC102404534	8.74E-02	6.94E-01					
MON2	-4.86E-02	6.95E-01					
KATNAL2	-1.92E-01	6.95E-01					
KIF18B	-5.68E-02	6.96E-01					
GAL3ST3	-9.61E-02	6.96E-01					
SLC16A10	-7.30E-02	6.99E-01					
PRKAB2	-5.46E-02	6.99E-01					
KDR	9.38E-02	6.99E-01					
CUL3	3.48E-02	6.99E-01					
ATP6V1E1	4.80E-02	7.02E-01					
KLHDC4	-5.81E-02	7.03E-01					
BRWD3	5.67E-02	7.06E-01					
ACTN4	4.50E-02	7.07E-01					
TMEM43	3.45E-02	7.09E-01					
NSMCE2	-4.14E-02	7.12E-01					
SBDS	-3.69E-02	7.12E-01					
CFAP45	1.12E-01	7.14E-01					
CTSB	-6.79E-02	7.15E-01					
LOC112581644	1.56E-01	7.16E-01					
EML6	8.83E-02	7.16E-01					
PSMD12	-3.96E-02	7.17E-01					
GABPB2	-5.58E-02	7.18E-01					
MRPL39	-4.04E-02	7.19E-01					

RFX1	3.82E-02	7.19E-01					
ZMYND11	-3.68E-02	7.20E-01					
IFT46	-4.72E-02	7.20E-01					
CGN	5.97E-02	7.21E-01					
PDZRN4	-6.28E-02	7.22E-01					
FBXO48	1.13E-01	7.22E-01					
WDR13	4.68E-02	7.22E-01					
ZNF582	-8.28E-02	7.23E-01					
LOC112586485	-1.75E-01	7.23E-01					
PJVK	-9.22E-02	7.24E-01					
BABAM1	4.37E-02	7.24E-01					
CTNNA1	-3.94E-02	7.25E-01					
CRYZ	-3.72E-02	7.26E-01					
TM4SF20	5.79E-02	7.27E-01					
USP19	3.59E-02	7.28E-01					
LRRC59	3.47E-02	7.29E-01					
CTR9	-3.37E-02	7.29E-01					
BRPF1	3.91E-02	7.29E-01					
PAWR	4.53E-02	7.30E-01					
LAT2	-1.46E-01	7.30E-01					
ZNF341	5.43E-02	7.30E-01					
PSMA7	-5.40E-02	7.33E-01					
TAF8	4.52E-02	7.33E-01					
VMAC	-6.17E-02	7.33E-01					
SMAD3	5.36E-02	7.34E-01					
LOC112581760	-9.15E-02	7.35E-01					
PRKACB	4.57E-02	7.36E-01					
WARS	3.35E-02	7.37E-01					
TOM1	3.76E-02	7.37E-01					
LOC102398085	1.15E-01	7.41E-01					
ZCCHC3	-3.44E-02	7.42E-01					
COL26A1	1.39E-01	7.43E-01					
MINDY1	3.18E-02	7.44E-01					
LRRC36	1.03E-01	7.44E-01					
MPG	5.21E-02	7.44E-01					
RMI1	-4.89E-02	7.44E-01					
EPS8L2	-1.27E-01	7.45E-01					
FMNL2	-4.44E-02	7.47E-01					
SNW1	2.93E-02	7.48E-01					

FES	-8.32E-02	7.49E-01					
LOC112585202	-1.44E-01	7.49E-01					
NAXE	-4.47E-02	7.51E-01					
LOC112581828	1.06E-01	7.51E-01					
ATAD2B	-5.42E-02	7.51E-01					
KCNC2	-1.03E-01	7.51E-01					
LOC112587762	-7.23E-02	7.53E-01					
CATSPER3	-6.74E-02	7.53E-01					
CIB2	5.12E-02	7.55E-01					
NFKB1	4.81E-02	7.57E-01					
PSMA5	3.99E-02	7.57E-01					
TMEM179B	-5.01E-02	7.57E-01					
APBB2	-3.23E-02	7.59E-01					
RORC	9.97E-02	7.60E-01					
ICE2	-4.62E-02	7.61E-01					
MGAT3	9.14E-02	7.64E-01					
PDE4B	-1.75E-01	7.65E-01					
CSTF3	-2.73E-02	7.65E-01					
PRPSAP2	3.26E-02	7.65E-01					
EML4	-3.61E-02	7.67E-01					
SPX	7.44E-02	7.71E-01					
EARS2	5.50E-02	7.72E-01					
LOC112587300	9.96E-02	7.74E-01					
FAM216A	5.61E-02	7.76E-01					
SHROOM1	7.02E-02	7.76E-01					
FBN2	-1.06E-01	7.77E-01					
ZDHHC8	3.74E-02	7.77E-01					
ADHFE1	-1.06E-01	7.77E-01					
FOXO6	6.38E-02	7.78E-01					
FBXO40	8.51E-02	7.78E-01					
UCK1	3.43E-02	7.78E-01					
DOCK10	-7.08E-02	7.78E-01					
KSR1	4.32E-02	7.79E-01					
C21H3orf49	5.27E-02	7.79E-01					
LOC102401686	-5.53E-02	7.79E-01					
FAM149B1	-2.98E-02	7.79E-01					
KYAT3	4.16E-02	7.81E-01					
LOC112584376	9.15E-02	7.81E-01					
RAC3	-5.23E-	7.82E-01					

	02							
ZNF181	-3.22E-02	7.82E-01						
NKD1	-7.62E-02	7.82E-01						
KCNJ3	1.89E-01	7.84E-01						
ZNF423	-7.77E-02	7.84E-01						
ZFAND1	4.47E-02	7.87E-01						
GPC2	-5.86E-02	7.88E-01						
HECW2	1.04E-01	7.89E-01						
MN1	-1.04E-01	7.90E-01						
TAX1BP3	3.52E-02	7.91E-01						
B4GALT2	-3.07E-02	7.91E-01						
SPN	-8.39E-02	7.93E-01						
LOC102400287	3.25E-02	7.93E-01						
ADGRB3	4.74E-02	7.94E-01						
STK26	-5.10E-02	7.94E-01						
CD274	-8.54E-02	7.95E-01						
COQ6	3.67E-02	7.95E-01						
LOC102413394	-4.37E-02	7.95E-01						
LOC102407124	-2.75E-02	7.95E-01						
AHCY	3.49E-02	7.97E-01						
FANCA	4.36E-02	7.99E-01						
PCDH19	1.15E-01	8.00E-01						
GATC	-3.53E-02	8.01E-01						
CCDC43	2.79E-02	8.02E-01						
RSPH10B	-6.26E-02	8.03E-01						
EMP1	-9.97E-02	8.03E-01						
LOC102395607	4.82E-02	8.06E-01						
ZNF713	-4.12E-02	8.07E-01						
ABCA7	-6.09E-02	8.08E-01						
MAPKBP1	3.12E-02	8.10E-01						
CAVIN2	-6.83E-02	8.10E-01						
DSCAM	1.46E-01	8.11E-01						
SYK	-1.34E-01	8.11E-01						
ARHGAP18	3.17E-02	8.12E-01						
SRXN1	5.07E-02	8.12E-01						
SDHAF4	-3.55E-02	8.13E-01						
MEF2D	3.29E-02	8.14E-01						
ELOF1	-3.51E-02	8.14E-01						

TRABD2B	9.35E-02	8.16E-01					
ATP1A1	-3.24E-02	8.16E-01					
LOC112585625	1.72E-01	8.16E-01					
SMIM4	4.58E-02	8.17E-01					
MED17	-2.27E-02	8.19E-01					
RETSAT	-4.89E-02	8.19E-01					
RNF220	-2.42E-02	8.20E-01					
SMAD1	2.84E-02	8.21E-01					
XPO7	-2.36E-02	8.26E-01					
LOC112579997	8.68E-02	8.26E-01					
SMU1	-2.03E-02	8.26E-01					
LSM3	3.89E-02	8.26E-01					
SMG6	-2.09E-02	8.26E-01					
DPYSL5	6.26E-02	8.26E-01					
LOC102409480	-5.21E-02	8.27E-01					
CIDEC	4.82E-02	8.27E-01					
CHMP2A	-3.35E-02	8.28E-01					
UBL3	2.66E-02	8.28E-01					
CYHR1	-2.68E-02	8.29E-01					
FAM83A	-5.40E-02	8.30E-01					
ZNF135	-3.40E-02	8.30E-01					
PDPR	2.67E-02	8.30E-01					
TIAF1	-4.88E-02	8.30E-01					
DBNDD2	4.96E-02	8.31E-01					
RAB4A	-3.52E-02	8.31E-01					
SNAPC2	-2.99E-02	8.33E-01					
WRN	-2.85E-02	8.33E-01					
RIPK1	3.64E-02	8.36E-01					
KATNBL1	3.11E-02	8.36E-01					
LMO2	-8.77E-02	8.39E-01					
RYBP	-2.18E-02	8.40E-01					
MAP6	-1.40E-01	8.41E-01					
SHC4	-2.86E-02	8.42E-01					
SPRED3	-4.77E-02	8.43E-01					
PTAFR	1.11E-01	8.44E-01					
NARS	-1.84E-02	8.44E-01					
TAB3	2.53E-02	8.44E-01					

RECQL4	3.27E-02	8.45E-01					
LOC102392425	3.63E-02	8.45E-01					
ATIC	-1.87E-02	8.45E-01					
CHSY3	-5.35E-02	8.45E-01					
ZNF516	-8.02E-02	8.46E-01					
USP5	2.18E-02	8.47E-01					
TRIO	-2.49E-02	8.48E-01					
ADA	6.24E-02	8.49E-01					
ZNF629	-2.25E-02	8.49E-01					
N4BP1	2.19E-02	8.50E-01					
HACL1	-2.89E-02	8.50E-01					
SKP1	3.59E-02	8.51E-01					
GATA3	-7.13E-02	8.51E-01					
GOLGA2	-3.03E-02	8.52E-01					
LOC102408946	-5.09E-02	8.52E-01					
PCMTD2	2.63E-02	8.52E-01					
MCM2	2.78E-02	8.53E-01					
VBP1	2.22E-02	8.55E-01					
NDUFA9	-2.20E-02	8.55E-01					
NIT1	2.85E-02	8.55E-01					
NCAPG2	-2.39E-02	8.55E-01					
CDADC1	-2.09E-02	8.56E-01					
CSNK1G3	-2.42E-02	8.56E-01					
STRAP	1.69E-02	8.59E-01					
SMG9	-2.11E-02	8.60E-01					
SMARCC1	2.30E-02	8.60E-01					
CASP6	-2.27E-02	8.61E-01					
SGO2	-2.51E-02	8.62E-01					
ANKMY2	-3.97E-02	8.62E-01					
LOC102409123	5.11E-02	8.62E-01					
RGS3	2.39E-02	8.62E-01					
SELENOK	-2.23E-02	8.63E-01					
SUB1	-2.01E-02	8.64E-01					
LOC102398945	-4.45E-02	8.65E-01					
CAMK4	5.78E-02	8.66E-01					
LOC112581182	-4.86E-02	8.67E-01					
MSH3	-1.70E-02	8.67E-01					

ZNF703	2.75E-02	8.67E-01
INPP5B	2.20E-02	8.67E-01
TTC21A	-2.75E-02	8.67E-01
BICRAL	2.38E-02	8.67E-01
LOC102409025	3.82E-02	8.67E-01
LOC112586165	6.57E-02	8.67E-01
PLXNA4	-6.82E-02	8.70E-01
GPC4	2.14E-02	8.71E-01
TSNARE1	2.34E-02	8.71E-01
PPM1B	-1.77E-02	8.71E-01
LOC112586432	5.41E-02	8.74E-01
LOC102399273	-7.08E-02	8.75E-01
GALNT17	8.58E-02	8.75E-01
FRMPD4	6.66E-02	8.75E-01
RAD52	1.49E-02	8.75E-01
FBXO16	-4.50E-02	8.75E-01
HPCAL4	-1.19E-01	8.75E-01
GALNT6	-9.55E-02	8.76E-01
ZFR2	4.96E-02	8.78E-01
NRAS	1.91E-02	8.78E-01
TAF1C	2.27E-02	8.79E-01
PRR3	2.03E-02	8.79E-01
CC2D1A	-1.69E-02	8.79E-01
PAIP2B	1.75E-02	8.79E-01
PCYT1A	-2.02E-02	8.82E-01
PLEKHB1	-2.05E-02	8.83E-01
LOC102405926	7.97E-02	8.85E-01
KIAA0319L	-1.51E-02	8.87E-01
FZD5	-2.25E-02	8.87E-01
SSNA1	-2.40E-02	8.88E-01
ATG3	1.23E-02	8.90E-01
TANGO2	2.02E-02	8.90E-01
MYBPC2	3.43E-02	8.91E-01
ALK	5.07E-02	8.91E-01
CEP19	3.13E-02	8.92E-01
RNMT	1.31E-02	8.93E-01
PLA2G6	-1.80E-02	8.93E-01
CFAP52	4.99E-02	8.97E-01
GYS1	-1.66E-02	8.97E-01
INKA1	-4.74E-02	8.97E-01

SOWAHB	2.17E-02	9.01E-01					
OGFOD2	-2.01E-02	9.01E-01					
GORASP2	-1.14E-02	9.02E-01					
SS18L2	-1.67E-02	9.03E-01					
SLC25A33	1.81E-02	9.03E-01					
XPOT	1.17E-02	9.03E-01					
SORCS2	8.14E-02	9.04E-01					
RAB20	4.18E-02	9.05E-01					
ELAVL1	1.08E-02	9.06E-01					
WDR61	-1.53E-02	9.09E-01					
FAM111B	-3.13E-02	9.13E-01					
LOC102410314	-5.16E-02	9.15E-01					
SAAL1	1.36E-02	9.17E-01					
NCBP2-AS2	-2.51E-02	9.18E-01					
DNAJC2	-1.05E-02	9.20E-01					
APOBEC2	-2.20E-02	9.20E-01					
LGR4	2.20E-02	9.26E-01					
LOC112585811	2.96E-02	9.26E-01					
HOOK3	-1.04E-02	9.28E-01					
THEM4	-1.51E-02	9.29E-01					
IL17RE	1.95E-02	9.31E-01					
LOC102409914	1.18E-02	9.33E-01					
LOC102394770	1.34E-02	9.34E-01					
PATL1	8.28E-03	9.36E-01					
NFKB2	1.08E-02	9.38E-01					
ZNF462	-1.18E-02	9.40E-01					
COMMD9	1.10E-02	9.40E-01					
CCDC14	-1.07E-02	9.41E-01					
LOC112581780	2.04E-02	9.41E-01					
LOC102394190	-1.02E-02	9.42E-01					
ADGRL1	1.07E-02	9.44E-01					
CHD4	-1.25E-02	9.44E-01					
DEPDC5	-8.24E-03	9.44E-01					
MARCH2	1.00E-02	9.45E-01					
LOC102406115	-9.05E-03	9.45E-01					
TIPRL	-7.57E-03	9.45E-01					
COQ8B	8.21E-03	9.46E-01					
COL23A1	-3.12E-02	9.48E-01					

LOC102405334	-1.62E-02	9.48E-01
NFIL3	-1.29E-02	9.49E-01
CELSR1	1.78E-02	9.52E-01
PSME3	5.51E-03	9.54E-01
OAS2	2.45E-02	9.55E-01
CAPZA1	-5.46E-03	9.56E-01
CRADD	-7.91E-03	9.57E-01
LOC102405491	-1.11E-02	9.64E-01
BCAR1	5.28E-03	9.64E-01
HACD1	7.92E-03	9.65E-01
LRP6	5.51E-03	9.66E-01
TSPYL2	5.21E-03	9.67E-01
LOC102410310	1.40E-02	9.67E-01
C14H20orf194	-5.26E-03	9.68E-01
TAF4B	6.43E-03	9.68E-01
TPPP3	8.62E-03	9.68E-01
WDR92	4.99E-03	9.69E-01
PIGF	6.29E-03	9.69E-01
PEX14	-3.66E-03	9.71E-01
MICAL3	3.85E-03	9.73E-01
CASQ1	6.39E-03	9.75E-01
IRF2BPL	-5.20E-03	9.75E-01
SUCO	-3.54E-03	9.76E-01
SMIM12	4.40E-03	9.77E-01
PES1	3.68E-03	9.79E-01
GPR83	1.10E-02	9.79E-01
LOC102392861	-4.38E-03	9.81E-01
PITPNC1	4.87E-03	9.82E-01
SF3B6	3.32E-03	9.82E-01
NDUFC1	-3.16E-03	9.82E-01
ANKRD42	3.11E-03	9.82E-01
MEN1	-2.25E-03	9.83E-01
CMTM7	-2.68E-03	9.85E-01
PILRA	-1.05E-02	9.88E-01
RALY	1.66E-03	9.91E-01
PLIN3	1.91E-03	9.91E-01
C12H2orf92	-5.13E-03	9.91E-01
NR1D1	1.81E-03	9.91E-01
FRRS1	-2.60E-03	9.91E-01
EPHB3	-1.59E-	9.97E-01

	03						
CENPV	-1.12E-03	9.97E-01					
MARCH6	-5.73E-05	1.00E+00					
RAB3IL1	1.52E-04	1.00E+00					

Supplementary file S8. List of differentially expressed gene DEGs in oocytes (OOs) and follicular cells (FCs) between breeding (BS) and non-breeding (NBS) seasons. For each DEG the Log Fold Change (LogFC) and the False Discovery Rate (FDR) were reported.

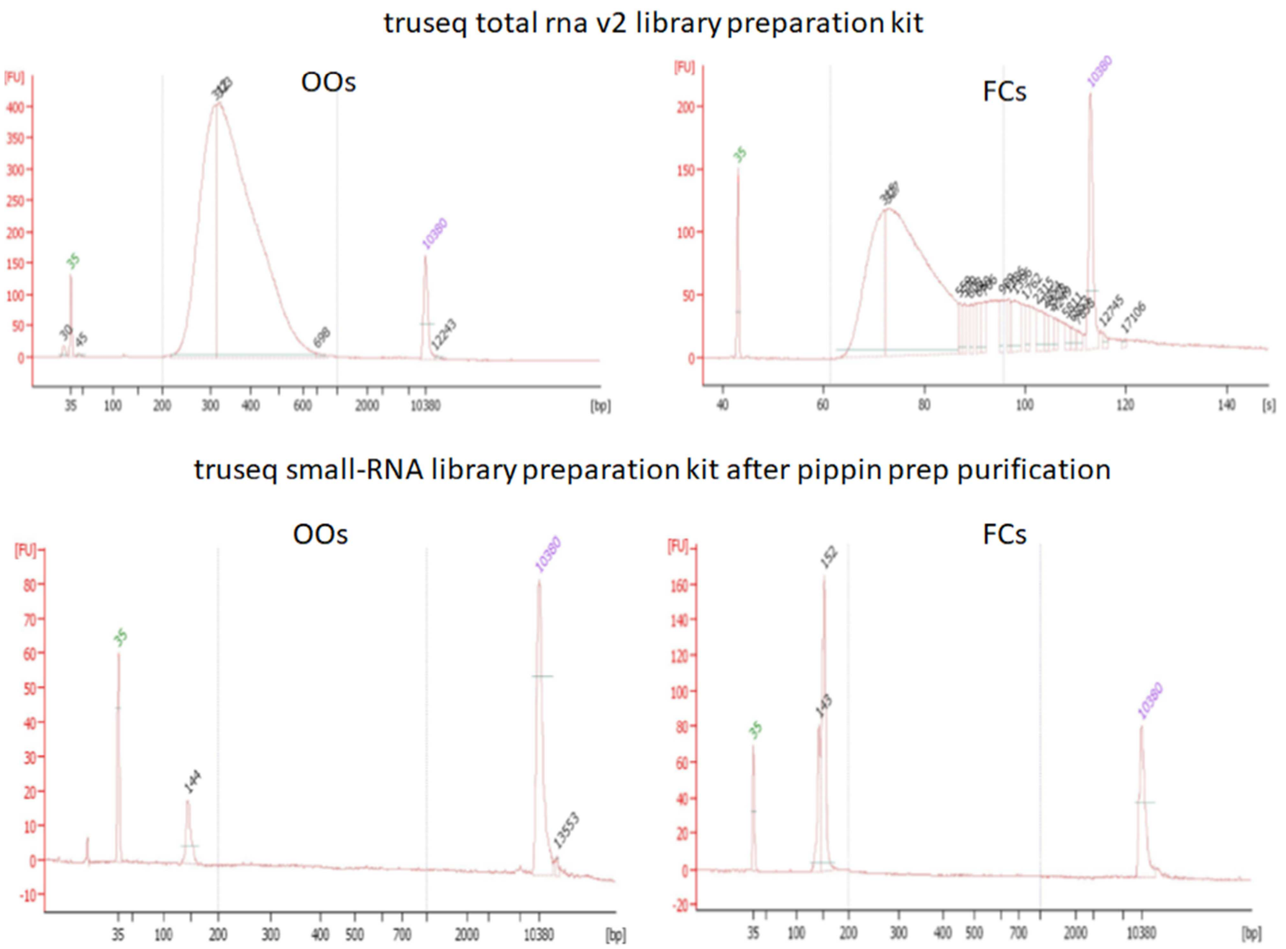

Supplementary file S9. Agilent 2100 Bioanalyzer profile of the RNA-Seq and Small RNA libraries generated by using the Illumina TruSeq RNA Sample Preparation v2 Kit and TruSeq Small RNA Library Preparation kit after pippin respectively for oocytes (OOs) and follicular cells (FCs).
